# Proteomic and Functional Characterization of Antimicrobial Peptides Derived from Fisheries Bycatch via Enzymatic Hydrolysis

**DOI:** 10.3390/md24010036

**Published:** 2026-01-10

**Authors:** Vicky Balesteros S. Blumen Galendi, Guilherme Rabelo Coelho, Letícia Murback, Wagner C. Valenti, Tavani Rocha Camargo, Marcia Regina Franzolin, Daniel Carvalho Pimenta, Rui Seabra Ferreira

**Affiliations:** 1 Botucatu Medical School (FMB), São Paulo State University (UNESP), Botucatu 18618-687, SP, Brazil; vicky.blumen@unesp.br (V.B.S.B.G.); l.murback@unesp.br (L.M.); 2 Laboratory of Toxinology, Faculty of Pharmaceutical Sciences of Ribeirao Preto, University of São Paulo (USP), São Paulo 05508-220, SP, Brazil; graco@usp.br; 3 Aquaculture Center (CAUNESP), Jaboticabal Campus, São Paulo State University (UNESP), Jaboticabal 14884-900, SP, Brazil; w.valenti@unesp.br; 4 SAMPLE—Innovation and Biotechnology in Animal Production, Registro 11900-000, SP, Brazil; tavani.rocha@unesp.br; 5 Butantan Institute, São Paulo 05503-900, SP, Brazil; marcia.franzolin@butantan.gov.br (M.R.F.); dcpimenta@butantan.gov.br (D.C.P.); 6 Center for the Study of Venoms and Venomous Animals (CEVAP), Botucatu Campus, São Paulo State University (UNESP), Botucatu 18618-687, SP, Brazil

**Keywords:** bycatch, marine peptides, antimicrobial peptides, enzymatic hydrolysis, mass spectrometry, proteomics, *Candida albicans*

## Abstract

Fisheries bycatch, while representing a major ecological concern due to the incidental capture of non-target species, also constitutes an underexplored source of marine biomass with biotechnological potential. This study aimed to generate and characterize bioactive peptides from the muscle tissue of three common bycatch species from the Brazilian coast: *Paralonchurus brasiliensis*, *Micropogonias furnieri*, and *Hepatus pudibundus*. Muscle homogenates were hydrolyzed using either Alcalase or Protamex to produce peptide-rich hydrolysates, which were analyzed through SDS-PAGE, HPLC-UV, MALDI-TOF, and LC-MS/MS. De novo sequencing and bioinformatic analyses predicted bioactivities that were subsequently validated by in vitro assays. The results demonstrated that enzyme selection strongly influenced both peptide profiles and bioactivity. The Protamex hydrolysate of *P. brasiliensis* (PBP) exhibited potent antifungal activity, inhibiting *Candida albicans* growth by 81%, whereas the Alcalase hydrolysate (PBA) showed moderate inhibition of *Staphylococcus aureus* (29%). No significant effect was observed against *Escherichia coli*. Overall, this study highlights a sustainable strategy for the valorization of fisheries bycatch through the production of bioactive marine peptides and identifies *P. brasiliensis* hydrolyzed with Protamex as a promising source of anti-*Candida* peptides for pharmaceutical and nutraceutical applications.

## 1. Introduction

The marine ecosystem, with its immense biodiversity, is a prolific source of unique bioactive compounds with therapeutic potential [1]. To date, over 41,000 bioactive molecules have been identified from marine organisms, leading to several FDA-approved drugs [2]. Concurrently, commercial fishing practices, particularly non-selective shrimp trawling, generate a significant ecological and economic problem: bycatch [3]. Globally, it is estimated that millions of tons of non-target species are discarded annually, representing a substantial loss of biomass and a threat to marine ecosystems [2,3].

Although bycatch is widely recognized as an ecological and conservation concern, the incidental capture of non-target species often results in discarded biomass [3]. When ethically and responsibly utilized, this material may contribute to reducing waste and expanding biotechnological discovery [4]. Structural proteins within the muscle of these specimens muscle can serve as a rich source of encrypted bioactive peptides, which can be released through enzymatic hydrolysis [5,6]. This approach aligns with circular bioeconomy principles that convert unavoidable waste into valuable compounds [7]. Previous work by our group and others has demonstrated that hydrolysates from bycatch species possess antioxidant and immunomodulatory properties [5,6].

The functional profile of a protein hydrolysate is critically dependent on the substrate, the specificity of the proteolytic enzyme used, and the hydrolysis conditions [8,9]. Enzymes like Alcalase and Protamex are widely used for their efficiency in hydrolyzing fish protein, but they possess different cleavage specificities, which should result in distinct peptide populations and, consequently, different bioactivities [5,6]. However, a comprehensive characterization linking the specific peptides generated by different enzymes from bycatch species to a specific antimicrobial function is still lacking.

This study aims to bridge this gap by performing a deep proteomic and functional characterization of hydrolysates from three abundant bycatch species: the fish *Paralonchurus brasiliensis* and *Micropogonias furnieri*, and the crab *Hepatus pudibundus*. We hypothesized that enzymatic hydrolysis with Alcalase and Protamex would release a diverse array of bioactive peptides from these species, and the choice of enzyme would critically determine the functional profile of the resulting hydrolysates, leading to selective and potent antimicrobial activities. Our findings reveal a powerful and specific anti-Candida activity, demonstrating the immense biotechnological potential hidden within fisheries bycatch.

## 2. Results

### 2.1. Enzymatic Hydrolysis Creates Complex and Peptide Profiles

Protein integrity and hydrolysis efficiency were evaluated by SDS-PAGE (Supplementary Materials—SM1–4).

**Figure 1:**
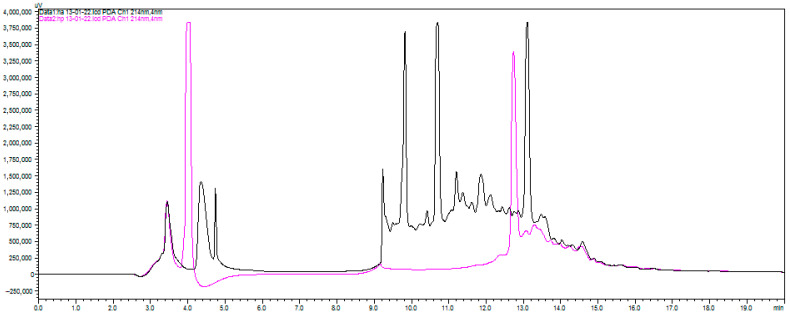
Chromatographic profile of the *Hepatus pudibundus* sample subjected to hydrolysis with Alcalase (black) and Protamex (pink).

RP-HPLC analysis revealed the complexity of the resulting peptide mixtures and highlighted the differential action of the two enzymes (Supplementary Materials—SM5–7). For all species, Alcalase generally produced a more complex chromatogram with a greater number of distinct peaks compared to Protamex, suggesting it generated a wider variety of peptides. For instance, with *H. pudibundus*, the Alcalase hydrolysate (HPA) showed seven major peaks of varying polarity, while the Protamex hydrolysate (HPP) showed only three (Figure 1). This indicates that enzyme selection is a critical parameter for controlling the final composition of the hydrolysate.

Although both enzymes produced broadly similar chromatographic patterns, closer inspection reveals subtle but consistent differences. Alcalase hydrolysates tended to show slightly broader and more heterogeneous peaks, indicating a more extensive cleavage pattern, whereas Protamex chromatograms exhibited fewer but sharper and more intense peaks. These differences, although modest in the chromatograms of (Supplementary Materials—SM5–7), were confirmed by LC-MS/MS, which showed that the two enzymes generated distinct peptide populations.

### 2.2. Mass Spectrometry Reveals Diverse Peptide Population

**Figure 2:**
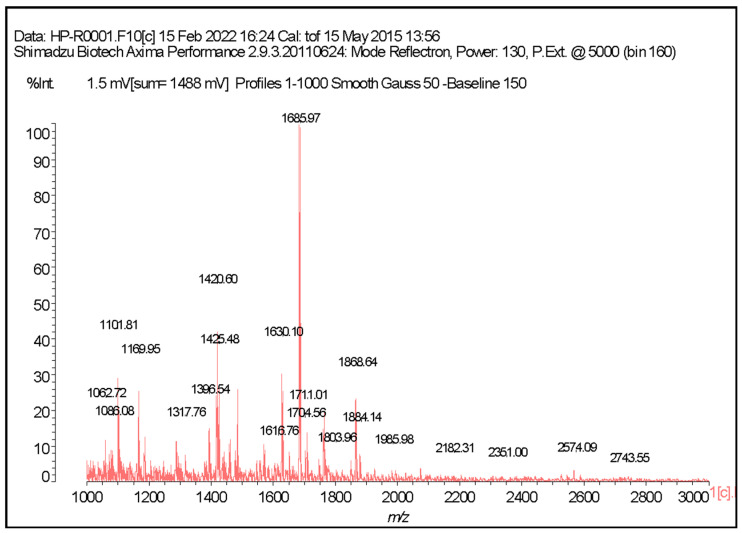
Representative MALDI-TOF mass spectrometry analysis generated from the sample of *Hepatus pudibundus* hydrolyzed with the Alcalase enzyme (HPA).

MALDI-TOF (Supplementary Materials—SM8–13) analysis revealed three major mass ranges: 1000–1500 Da, 1500–2000 Da, and 2000–3000 Da. The most abundant region corresponded to 1500–2000 Da, but substantial peptide signals were also observed in the adjacent ranges. Figure 2 shows the MALDI-TOF mass spectrum obtained from the Alcalase-hydrolyzed sample of *Hepatus pudibundus* (HPA).

LC-MS analysis further resolved this complexity, revealing hundreds of individual peptide signals that were co-eluting under the broad peaks observed in HPLC-UV (Supplementary Materials—SM14–37). This confirmed that each hydrolysate was a highly complex mixture of peptides with varying masses and polarities.

### 2.3. Proteomic Analysis Identifies Peptides with Potent Antimicrobial Activity

De novo sequencing via LC–MS/MS successfully identified thousands of individual peptide sequences across all samples. The top-scoring de novo-sequenced peptides obtained from the six experimental conditions are presented in Table 1. The complete list of peptide sequences identified by de novo LC–MS/MS analysis, as well as the corresponding raw data files, is provided in Supplementary Materials (SM38–44).

Peptide scoring was based on the occurrence frequency of bioactive motifs within each sequence. For this purpose, all possible di- and tripeptide fragments derived from each peptide were queried against the BIOPEP-UWM database https://biochemia.uwm.edu.pl (accessed on 20 February 2024) [10], and the number of matches for each bioactivity class was recorded.

OAC (Overall Activity Count) represents the total number of bioactive fragments detected within a given peptide sequence, whereas ALC (Activity Likelihood Coefficient) reflects the proportion of fragments associated with the most prevalent bioactivity category. Higher OAC and ALC values indicate a greater likelihood of functional relevance.

The identified peptides were primarily derived from highly abundant muscle proteins, such as actin, myosin, and titin, indicating effective targeting of the main structural protein sources with minimal evidence of external contamination. In addition, peptides originating from housekeeping and metabolic proteins were also annotated through BLASTp 2.16.0 analysis, suggesting their potential contribution as secondary peptide precursors (Table 2).

Sequence similarity searches were performed using BLASTp against the NCBI non-redundant protein database https://blast.ncbi.nlm.nih.gov/Blast.cgi (accessed on 20 February 2024). While Table 1 presents peptides selected based on their highest predicted bioactivity scores (OAC and ALC), BLASTp analysis was intentionally conducted on a broader set of identified peptide sequences. This approach aimed to determine the most likely parent proteins and biological origins of peptides that yielded significant sequence alignments.

Consequently, some peptide sequences reported in Table 2 do not appear in Table 1, as inclusion in the BLASTp analysis was based on alignment quality criteria (e-value ≤ 1 and sequence identity ≥ 60%), rather than bioactivity ranking alone.

In Table 2, certain peptide sequences appear more than once (e.g., LKYPLE and LEEEELKLF). This apparent redundancy reflects cases in which identical peptide sequences originate from distinct taxonomic groups. For instance, peptides detected in PBA originate from a bony fish (Teleostei), whereas those detected in HPA derive from a crustacean (Crustacea), highlighting organism-specific differences in peptide generation despite sequence similarity.

All confidently identified peptide sequences were initially screened using BLASTp; however, only those meeting the predefined significance thresholds were retained and reported in Table 2.

Through BIOPEP-UWM database analyses, the top-scored peptides were queried, and their potential bioactivities were proposed based on biological activities previously annotated in BIOPEP-UWM, as presented in Table 3.

**Figure 3:**
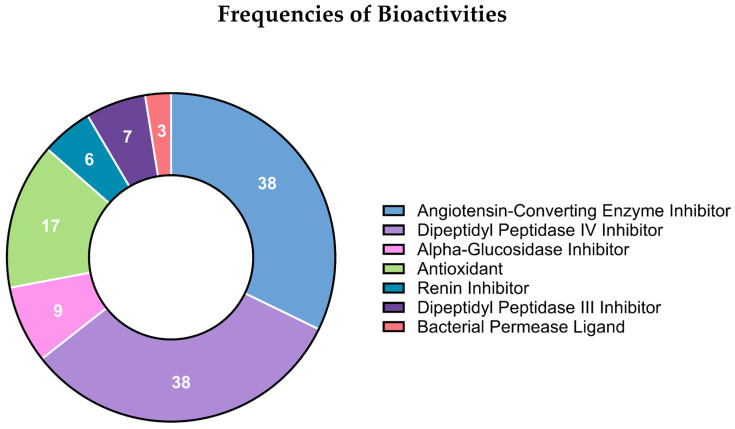
Frequency distribution of predicted biological activities associated with the sequenced peptides. Bioactivities were assigned using the BIOPEP-UWM “Profiles of Potential Biological Activity” tool, with *Teleostei* and *Crustacea* selected as reference taxonomic groups for fish- and crustacean-derived peptides, respectively. Frequencies represent the number of times each predicted activity was annotated across all peptide sequences. Only activities supported by BIOPEP-UWM database matches were included.

The global predicted biological effects of all sequenced peptides are summarized in Figure 3, which displays the frequency distribution of bioactivities assigned to each peptide. These frequencies were determined using the BIOPEP-UWM database of biologically active peptides https://biochemia.uwm.edu.pl/biopep-uwm/ (accessed on 20 February 2024). Each peptide sequence was queried individually through the “Profiles of Potential Biological Activity” tool, using *Teleostei* as the reference taxonomic group for bony fish-derived peptides and *Crustacea* for crustacean-derived peptides. Because no specific peptide databases exist for bycatch species and genetic information for these organisms remains limited, BIOPEP-UWM was used as the most comprehensive and biologically relevant reference source. For each query, the database generated a list of potential bioactivities based on sequence similarity to experimentally validated peptides stored in BIOPEP-UWM.

The frequencies shown in Figure 3 therefore represent the number of times each predicted bioactivity was annotated across all analyzed peptides. Angiotensin-converting enzyme (ACE) inhibitory peptides were identified when the sequences matched BIOPEP-UWM entries classified under the “ACE inhibitory peptides” category, following the database’s established annotation criteria. All peptide-activity assignments were extracted directly from BIOPEP-UWM outputs, and only activities supported by the database were included in the final frequency distribution.

**Figure 4:**
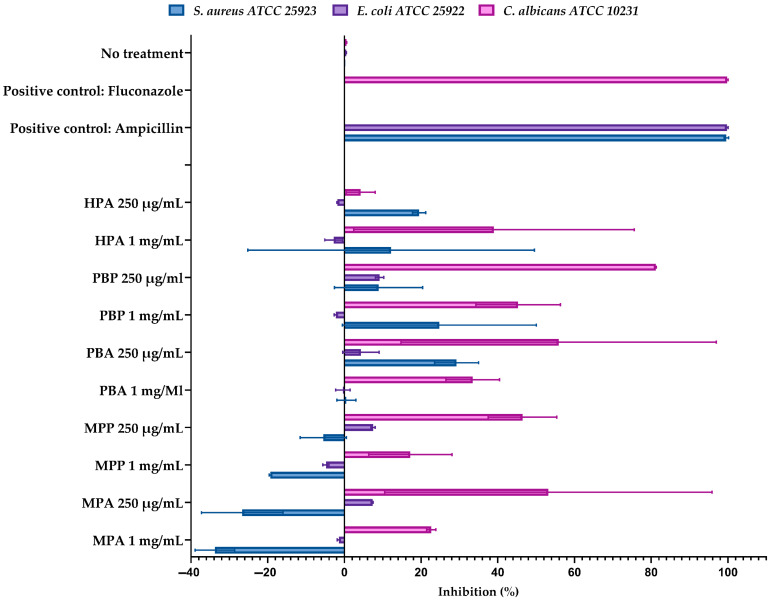
Antimicrobial activity of peptide hydrolysates (250 µg/mL and 1 mg/mL) obtained from *Micropogonias furnieri* (MPA, MPP), *Paralonchurus brasiliensis* (PBA, PBP), and *Hepatus pudibundus* (HPA) against *Staphylococcus aureus* ATCC 25953, *Escherichia coli* ATCC 25922, and *Candida albicans* ATCC 10231. Positive controls (ampicillin, 2 µg/mL, for bacteria; fluconazole, 1 µg/mL, for *C. albicans*) and a no-treatment control are included for comparison. Assays were performed in 96-well microplates following the general principles of CLSI guidelines [11,12]. Results are expressed as mean percentage inhibition ± standard deviation (SD) from duplicate experiments (*n* = 2). The corresponding raw inhibition data are provided in the Supplementary Materials (SM45–46).

The most significant finding emerged from the antimicrobial assays (Figure 4). The hydrolysates displayed highly selective activity. The Protamex hydrolysate of *P. brasiliensis* (PBP 250 µg/mL) was exceptionally active against *C. albicans*, causing 81.2% growth inhibition. The Alcalase hydrolysate of the same species (PBA 250 µg/mL) was the most effective against *S. aureus*, with 29.3% inhibition. None of the hydrolysates showed meaningful activity against the Gram-negative *E. coli* (<8% inhibition).

**Figure 5:**
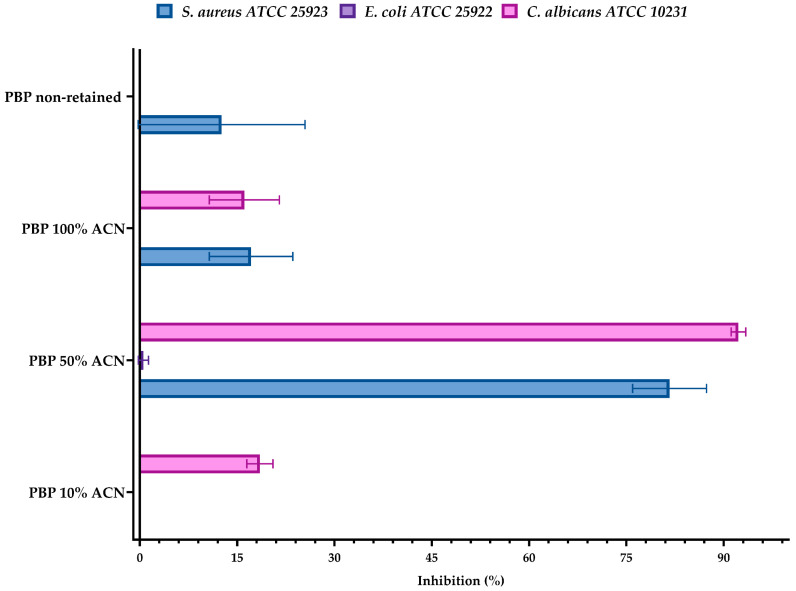
Antimicrobial activity of chromatographic fractions obtained from *Paralonchurus brasiliensis* Protamex hydrolysate (PBP) following RP–HPLC separation. Fractions included the non-retained fraction and those eluted with 10%, 50%, and 100% acetonitrile (ACN). Assays were performed against *Staphylococcus aureus* ATCC 25923, *Escherichia coli* ATCC 25922, and *Candida albicans* ATCC 10231 in duplicate (*n* = 2), following the general principles of CLSI guidelines [11,12]. Results are expressed as mean percentage inhibition ± standard deviation (SD). Raw antimicrobial inhibition data for each fraction are available in the Supplementary Materials (SM45–46).

All tested microorganisms presented 100% of inhibition to positive controls: ampicillin at 2 µg/mL for bacteria and fluconazole at 1 µg/mL for *C. albicans* (Figure 5). Given the exploratory nature of this screening and the use of duplicate measurements (*n* = 2), no inferential statistical analysis was performed. Variability among replicates is presented as mean and standard deviation (SD) to illustrate experimental dispersion. The corresponding raw inhibition values for all assays are provided in Supplementary Materials (SM45–46).

The antimicrobial activity of peptide fractions obtained from *Paralonchurus brasiliensis* hydrolyzed with Protamex was evaluated after RP–HPLC separation. Four fractions were generated: the non-retained fraction and fractions eluted with 10%, 50%, and 100% acetonitrile (ACN). Each fraction was tested against *Staphylococcus aureus* ATCC 25953, *Escherichia coli* ATCC 25922, and *Candida albicans* ATCC 10231 (Figure 5).

The fraction eluted at 10% ACN exhibited low antimicrobial activity, showing negligible inhibition against *S. aureus* and *E. coli*, while a modest inhibition was observed against *C. albicans* (≈18%). In contrast, the 50% ACN fraction displayed the highest antimicrobial activity among all chromatographic fractions, strongly inhibiting *S. aureus* (≈81%) and *C. albicans* (≈92%), while showing minimal activity against *E. coli* (≈0.5%).

The fraction eluted at 100% ACN showed moderate inhibition against *S. aureus* (≈17%) and *C. albicans* (≈16%), with no detectable activity against *E. coli*. Similarly, the non-retained fraction exhibited low but measurable inhibition against *S. aureus* (≈13%) and *C. albicans* (≈16%), while *E. coli* remained largely unaffected. The individual inhibition values underlying these observations are reported in Supplementary Materials (SM45–46).

When compared to the unfractionated *Paralonchurus brasiliensis* with Protamex hydrolysate (PBP), which exhibited strong inhibitory activity against both *S. aureus* and *C. albicans* (≈81% inhibition at 250 µg/mL), the activity profiles of the chromatographic fractions indicate that antimicrobial peptides are distributed across multiple hydrophobicity ranges. Notably, the fraction eluted at 50% ACN retained the greatest proportion of antimicrobial activity, particularly against *C. albicans*.

## 3. Discussion

This study successfully demonstrates a viable approach to mitigate the environmental impacts of fisheries bycatch, demonstrating that discarded biomass can yield potent, bioactive peptides. Rather than promoting bycatch, the work highlights a sustainable strategy to reduce waste and support marine conservation efforts through scientific innovation. Our results confirm the hypotheses that enzymatic hydrolysis effectively liberates a diverse range of peptides from bycatch muscle, and the choice of enzyme is a critical determinant of the resulting functional activity, enabling the production of hydrolysates with highly selective antimicrobial properties.

Both Alcalase and Protamex enzymes effectively hydrolyzed the muscle proteins *of Paralonchurus brasiliensis*, *Micropogonias furnieri*, and *Hepatus pudibundus*, as confirmed by SDS-PAGE (absence of high molecular weight bands) and RP-HPLC profiles. Alcalase hydrolysates presented a higher number of peaks and broader polarity range, suggesting greater peptide diversity, while Protamex produced simpler chromatograms with fewer but more intense peaks. This pattern aligns with findings from Caruso et al. (2020) [13], who reported that enzyme specificity directly influences the diversity of peptides derived from fish by-products. Enzymes with broad specificity, such as Alcalase, often generate smaller, heterogeneous peptides with multiple bioactivities, whereas mixed bacterial proteases like Protamex favor selective cleavage sites, yielding peptide populations with defined biofunctionalities.

The observed chromatographic differences are crucial because peptide diversity often correlates with multifunctionality in bioactive peptide research [13,14,15]. This enzymatic behavior highlights the need to tailor hydrolysis parameters to optimize the generation of targeted bioactive compounds from fisheries discards, reinforcing the potential of a circular bioeconomy that utilizes unavoidable waste without promoting harmful fishing practices [16,17].

LC–MS/MS analysis revealed a diverse peptide repertoire across the hydrolysates, totaling thousands of sequences, predominantly derived from canonical muscle proteins such as actin, myosin, and titin. This profile closely aligns with what has been reported for proteomic surveys of marine by-products, in which structural proteins constitute the main precursors of bioactive peptides [9]. Bioinformatic annotation (BIOPEP-UWM) confirmed the presence of peptides with predicted antioxidant, antihypertensive, and antimicrobial functions, properties widely associated with short (2–20 amino acids), amphipathic peptides enriched in hydrophobic residues, a common feature of marine-derived AMPs [13,17,18]. The abundance of multifunctional peptides reinforces the potential of ethically valorized bycatch biomass as a renewable reservoir of pharmacologically relevant molecules [4,18].

A major functional highlight of this study was the potent and selective antifungal activity observed in the Protamex hydrolysate of *Paralonchurus brasiliensis* (PBP), which achieved over 81% inhibition of *Candida albicans* in its unfractionated form. This magnitude of inhibition is highly relevant given the increasing incidence of resistant fungal infections worldwide [19]. The selectivity pattern observed (strong inhibition of yeast, moderate inhibition of *S. aureus*, and limited activity against *E. coli*) is consistent with classical AMP behavior and reflects fundamental structural differences between fungal, Gram-positive, and Gram-negative cell envelopes [19,20,21,22]. In particular, the multilayered outer membrane of Gram-negative bacteria often prevents AMP penetration, explaining the low activity against *E. coli* [10,17].

The chromatographic separation of PBP by RP–HPLC further clarified how antimicrobial activity is distributed across peptide populations of different hydrophobicities. Among the fractions, the 50% ACN eluate retained the highest antimicrobial activity, with a particularly pronounced effect against *Candida albicans* and strong activity against *Staphylococcus aureus*, while 10% ACN and non-retained fractions were only weakly active. This suggests that the most bioactive peptides fall within an intermediate hydrophobicity range, a characteristic typical of membrane-active AMPs that require balanced hydrophobic and charged residues to penetrate microbial membranes. The moderate activity observed in the 100% ACN fraction indicates that highly hydrophobic peptides also contribute, but to a lesser extent.

The integrated proteomic and functional evidence allows us to propose a plausible mechanistic explanation for these patterns. The Protamex-derived hydrolysate contained unique peptides such as LEEEELKLF (ALC 97%), an anionic–amphipathic structure characterized by a polar acidic N-terminus paired with a hydrophobic C-terminal tail (LKLF). This architecture is consistent with peptides known to disrupt fungal membranes via electrostatic attraction followed by hydrophobic insertion [17]. Other peptides in PBP, such as VDLWFK (ALC 95%), share hydrophobic and aromatic residues associated with fungal membrane destabilization. In contrast, the Alcalase hydrolysate (PBA), more active against *S. aureus*, carried distinct peptide families such as LDFDEFLMK and the proline-rich LLAPPE, consistent with cleavage specificities and substrate preferences unique to each enzyme. These results confirm that enzyme selection directly shapes both the sequence composition and biological activity of the resulting peptide pools.

Taken together, these findings demonstrate that Protamex hydrolysis of *P. brasiliensis* yields peptides with particularly strong antifungal potential, an urgently needed property amid rising resistance to conventional antifungal therapies, highlighting its potential as a lead for further development rather than a direct therapeutic substitute [22,23]. The functional differences among fractions and enzymes highlight how protease choice, peptide hydrophobicity, and physicochemical balance converge to generate distinct antimicrobial profiles [24,25]. These insights reinforce the biotechnological promise of bycatch-derived peptides while aligning with sustainable and ethical bioresource utilization practices.

By demonstrating potent and specific activity without promoting overexploitation, this study provides a model for sustainable drug discovery from unavoidable fisheries by-products. These findings build upon previous work from our group that identified antioxidant and cell-modulatory activities in hydrolysates from these same fish species and from crustacean bycatch [5,6]. The current study expands this research by identifying a potent and specific antimicrobial function, significantly increasing the potential value of these materials. The peptide sizes identified (primarily 2–20 amino acids) are consistent with the vast majority of known bioactive peptides from marine sources.

The use of fisheries bycatch as a biomaterial aligns with the principles of sustainable bioprospecting and circular bioeconomy [17]. While bycatch remains a major environmental concern, its ethical repurposing can reduce waste and generate high-value compounds. Similar initiatives have been discussed in the context of fish processing residues [13,18,19] and aquaculture by-products [4], emphasizing the dual benefits of waste minimization and resource recovery. This framework does not aim to justify bycatch, but rather to mitigate its impacts while promoting responsible innovation. Integrating biotechnological valorization with marine conservation policies could help reconcile economic and ecological goals.

### Limitations and Future Directions

This study has several limitations that open avenues for future research. First, the antimicrobial activity was determined at two screening concentrations (250 µg/mL and 1000 µg/mL). Future work must establish dose–response curves and determine the Minimum Inhibitory (MIC) and Minimum Fungicidal (MFC) concentrations for the most active hydrolysates. Second, the activity was demonstrated for a complex hydrolysate. The next logical step is to fractionate the PBP hydrolysate to isolate the specific peptide(s) responsible for the anti-Candida activity. The candidate peptides identified in Table 1, such as LEEEELKLF, should be synthesized and tested individually to confirm their function. Finally, expanding the scope to include transcriptomic or genomic data from bycatch species could enhance peptide annotation accuracy and facilitate predictive modeling of bioactivity [12]. Integrating omics approaches with ethical sourcing frameworks can set a new standard for sustainable marine drug discovery.

## 4. Materials and Methods

### 4.1. Sample Collection and Hydrolysate Preparation

Specimens of *Paralonchurus brasiliensis*, *Micropogonias furnieri*, and *Hepatus pudibundus* were collected as bycatch from shrimp trawling operations conducted in Ubatuba, São Paulo, Brazil, between 2017 and 2018 [5,6]. Muscle tissue (100 g) was homogenized in distilled water (1:2, *w*/*v*), and endogenous enzymes were inactivated by heating at 80 °C for 20 min. The homogenate was then subjected to enzymatic hydrolysis using either food-grade Alcalase^®^ 2.4 L (a serine endopeptidase from *Bacillus licheniformis*) or Protamex^®^ (a protease blend from *B. licheniformis* and *B. amyloliquefaciens*), applied at an enzyme-to-substrate ratio of 2% (*w*/*w*).

For Alcalase hydrolysis, reactions were carried out at pH 8.0 and 60 °C, whereas Protamex hydrolysis was performed at pH 7.0 and 60 °C. Reaction pH was maintained by automated titration using 1 M NaOH. Hydrolysis proceeded until the Degree of Hydrolysis (DH) reached a plateau (approximately 5 h). Enzymes were then inactivated by heating at 80 °C for 20 min. The resulting mixtures were centrifuged at 16,300× *g* for 20 min at 4 °C, freeze-dried, and stored at −20 °C until analysis. All hydrolyses were performed in sextuplicate (*n* = 6).

Short-term sample storage was carried out at 4 °C. Although long-term storage can potentially affect protein integrity, extensive hydrolysis is expected to minimize such effects by converting high-molecular-weight proteins into more stable peptide products.

### 4.2. Biochemical Characterization

SDS-PAGE: results are shown in Supplementary Materials (SM1–SM4).

Reversed-Phase High-Performance Liquid Chromatography (RP-HPLC): Lyophilized hydrolysates (0.5 mg/mL) were dissolved in 0.1% trifluoroacetic acid (TFA) and injected into a Shimadzu HPLC system (Shimadzu Co., Kyoto, Japan), model LC-20AT, equipped with a diode array ultraviolet detector (SPD-M20A). The system consisted of two LC-20AT pumps (pumps A and B), an autosampler (SIL-20AC HT), a fraction collector (FRC-10A), and a system controller (CBM-20A). Separations were performed using a C18 analytical column (4.6 × 250 mm, Wakopak®). Peptide elution was carried out using a linear gradient from 0 to 100% solvent B (90% acetonitrile, 0.1% TFA) over 20 min at a flow rate of 1 mL/min. UV detection was monitored at 214 nm.

MALDI-TOF Mass Spectrometry: Peptide mass distribution was analyzed using an Axima Performance MS/MS instrument (Shimadzu Co., Kyoto, Japan), operated in positive ion mode. Samples were mixed (1:1, *v*/*v*) with a supersaturated matrix solution, selected according to the analyte: sinapinic acid for peptides and proteins, α-cyano-4-hydroxycinnamic acid for peptides of lower molecular mass, or 2,5-dihydroxybenzoic acid (DHB), as appropriate. Aliquots (0.4–0.8 µL) of the mixtures were spotted onto the MALDI target plate and allowed to air-dry prior to analysis. Data acquisition was performed using the instrument’s automatic control mode and Launchpad software, version 2.9.3 (Shimadzu Biotech, Kyoto, Japan).

### 4.3. Proteomic Analysis (LC-MS/MS) and De Novo Sequencing

For in-depth peptide identification, samples were analyzed on an electrospray-ion trap-time of flight (ESI-IT-TOF) (Shymadzu Co., Japan) mass spectrometer equipped with binary ultra-fast liquid chromatography system (UFLC) (20A Prominence, Shimadzu Co., Japan). Lyophilized samples were reconstituted in 0.1% formic acid, and 20 µL were injected at a constant flow rate of 0.2 mL/min. Peptides were separated on a Kinetex C18 column (50 × 2.1 mm, 5 µm; Phenomenex, Torrance, CA, USA) with a 35 min gradient of 0–100% solvent B (90% ACN, 0.1% formic acid) at 0.2 mL/min. Elution was monitored using a photodiode array detector (SPD-M20A, Shimadzu Corporation, Kyoto, Japan) prior to mass spectrometric analysis. MS spectra were acquired from 350–1400 *m*/*z*, and MS/MS spectra from 50–1950 *m*/*z*. The interface voltage was set to 4.5 kV, with a capillary voltage of 1.95 kV at 200 °C. Collision-induced dissociation was performed using argon gas with a collision energy of 55%.

The resulting mgf files were processed using PEAKS Studio v7.0 (Bioinformatics Solutions Inc., Waterloo, ON, Canada). For peptide identification, de novo sequencing was performed with the following parameters: parent mass error tolerance of 0.1 Da, fragment mass error tolerance of 0.1 Da, and variable modifications of methionine oxidation and carbamidomethylation. Crucially, the cleavage parameter was set to “Non-specific” to reflect the action of Alcalase and Protamex, not a sequence-specific enzyme like trypsin. A false discovery rate (FDR) of ≤0.5% was applied, and only peptides with a high confidence score (ALC%) were considered for further analysis. Identified sequences were searched against the NCBI non-redundant database using BLASTp 2.16.0 and functionally annotated using the BIOPEP-UWM database.

### 4.4. Antimicrobial Activity Assay

The antimicrobial activity of the hydrolysates was screened against Gram-positive *Staphylococcus aureus* (ATCC 25953), Gram-negative *Escherichia coli* (ATCC 25922), and the pathogenic yeast *Candida albicans* (ATCC 10231). Assays were performed in 96-well microtiter plates following the general principles of the Clinical and Laboratory Standards Institute (CLSI) guidelines, CLSI M07 and M27 documents for antibacterial and antifungal susceptibility testing [11,12], with minor adaptations for peptide hydrolysates.

Briefly, microbial suspensions (50 µL; 1.5 × 10^6^ CFU/mL) were added to wells containing 50 µL of hydrolysate solution, yielding a final concentration of 250 µg/mL. Plates were incubated at 37 °C for 24 h, and microbial growth was assessed by measuring optical density at 595 nm. Ampicillin (for bacteria) and fluconazole (for *C. albicans*) were used as positive controls, while buffer served as the negative control.

The percentage of growth inhibition was calculated using the following equation:

% Inhibition = [1 − (OD_sample/OD_negative control)] × 100.

This single-concentration assay was employed as a preliminary screening approach. Determination of minimum inhibitory concentration (MIC) values represents a necessary next step for the most promising hydrolysates and fractions.

## 5. Conclusions

This research successfully validates the use of enzymatic hydrolysis as a powerful tool to unlock the biotechnological potential of fisheries bycatch. We have shown that the strategic selection of enzymes can produce peptide hydrolysates with potent and highly selective antimicrobial activities. Specifically, the hydrolysis of Paralonchurus brasiliensis muscle with Protamex yields a peptide mixture with strong and specific inhibitory activity against the pathogenic yeast Candida albicans. This work offers a sustainable and ethically conscious approach to repurpose a problematic waste stream, converting fisheries bycatch into biotechnological value without endorsing harmful practices. This work exemplifies the balance between biotechnology and conservation in the era of the circular bioeconomy. Additionally, it identifies a promising new source of natural antifungal compounds with potential applications in the pharmaceutical, nutraceutical, and functional food industries.

## Figures and Tables

**Table 1:** Compilation of the four peptides with the highest Overall Average Confidence (OAC/ALC) scores identified via de novo sequencing for each experimental condition.

Origin	Peptide	OAC/ALC (%)	Mass (Da)	RT (min)	ppm
PBA	LLAPPE	97	638.36	11.09	−0.8
PBA	YEKK	96	566.30	0.69	0.1
PBA	LVYPSV	94	676.37	13.97	−31.2
PBA	VKLPKL	94	696.48	11.25	−0.6
PBP	LEEEELKLF	97	1148.59	13.44	−0.4
PBP	EYKK	95	566.30	2.46	−0.3
PBP	VDLWFK	95	806.43	13.17	−0.9
PBP	LKLFL	94	632.42	13.64	−0.4
MFA	LDEVLKFF	95	1009.54	14.37	−0.4
MFA	LEHEE	95	655.28	8.51	3.2
MFA	LNNLL	93	585.34	11.73	−37.8
MFA	TKTPGLME	93	875.44	10.50	−1.2
MFP	LEEEELKLF	97	1148.59	13.44	−0.3
MFP	FLDLDQDKKFEE	94	1525.73	12.66	−0.7
MFP	LVLHL	93	593.39	12.64	−1.2
MFP	VLDQDKSGFLE	93	1249.61	11.38	−0.4
HPA	LTKLL	97	586.40	11.72	−0.5
HPA	LPTKF	95	604.35	10.77	2.1
HPA	VVAKKPQE	95	897.52	10.02	19.8
HPA	KLHWH	94	719.38	8.76	0.1
HPP	LKYPLE	95	761.43	11.29	0.0
HPP	VNVDPDGKF	94	989.48	11.26	−0.2
HPP	LPDWHPMDR	93	1165.53	10.20	1.9
HPP	VHLKLPK	93	833.54	10.11	−0.2

Origin Acronyms: PBA (*Paralonchurus brasiliensis*/Alcalase), PBP (*P. brasiliensis*/Protamex), MFA (*Micropogonias furnieri*/Alcalase), MFP (*M. furnieri*/Protamex), HPA (*Hepatus pudibundus*/Alcalase), HPP (*H. pudibundus*/Protamex).

**Table 2:** Summary of the top five peptide alignments with the lowest E-values (highest statistical significance) among the sequences yielding significant BLASTp matches for each experimental condition, identifying the most likely precursor proteins. The E-value represents the probability of a match occurring by chance.

Origin	Peptide	Mass (Da)	Query Cover (%)	E-Value	Seq. ID	Protein Description	Species
PBA	LLDQDKSGFLE	1263.63	100%	0.52	TSK14644.1	Parvalbumin	*Bagarius yarrelli*
PBA	LDFDEFLMK	1156.54	100%	1.9	XP_042563305.1	Calcium-binding protein	*Clupea harengus*
PBA	LLVYPW	789.44	100%	33	XP_028311025.1	Hemoglobin beta-A-like	*Gouania willdenowi*
PBA	LKYPLE	761.43	100%	182	RXN10808.1	Myosin	*Labeo rohita*
PBA	LVYPSV	676.37	100%	367	XP_043868191.1	Zinc finger protein	*Solea senegalensis*
PBP	LLDKNRDGLLSQ	1370.75	100%	0.89	XP_022539758.1	Calcium-binding protein	*Astyanax mexicanus*
PBP	LEEEELKLF	1148.59	100%	1.4	P86432.1	Parvalbumin	*Oncorhynchus mykiss*
PBP	LEDDLVALK	1014.55	100%	5.6	KAF6714773.1	Serine beta-lactamase-like	*Oryzias melastigma*
PBP	VDLWFKA	877.46	100%	7.3	XP_021169181.2	Nucleoside diphosphate kinase B	*Fundulus heteroclitus*
PBP	TVDDKVELE	1046.51	88%	22	XP_040927192.1	Myosin heavy chain	*Betta splendens*
MFA	LDEVLKFF	1009.54	100%	1.6	XP_034043238.1	ATPase 2-like	*Thalassophryne amazonica*
MFA	TKTPGLME	875.44	100%	2.2	TWW70116.1	Myosin heavy chain	*Takifugu rubripes*
MFA	LLAPPEVGKY	1085.61	100%	56	XP_013882412.1	Tomoregulin-1	*Austrofundulus limnaeus*
MFA	VVDGVKL	728.44	100%	116	TSK22821.1	Creatine kinase	*Bagarius yarrelli*
MFA	MKFLW	723.37	100%	141	KAA0719128.1	Zinc finger protein	*Sinocyclocheilus rhinocerous*
MFP	VLDQDKSGFLE	1249.61	100%	0.066	TSK77154.1	Parvalbumin Beta	*Bagarius yarrelli*
MFP	LEEEELKLF	1148.59	100%	2.0	TWW69872.1	Parvalbumin Beta 2	*Takifugu flavidus*
MFP	FLDLDQDKKFEE	1525.73	100%	7.2	XP_016411869.1	Ubiquitin carboxyl-hydrolase	*Sinocyclocheilus rhinocerous*
MFP	VGDEAQSK	832.39	100%	8.7	ABP97428.1	Beta actin	*Rhinichthys cataractae*
MFP	LVEELPARW	1111.60	100%	16	XP_035995563.1	Titin	*Fundulus heteroclitus*
HPA	VHLKLPK	833.54	100%	0.047	P86432.1	Parvalbumin beta 2	*Oncorhynchus mykiss*
HPA	LKYPLE	761.43	100%	1.5	P86432.1	Parvalbumin beta 2	*Oncorhynchus mykiss*
HPA	VNVDPDGKF	989.48	100%	15	XP_016411870.1	Ubiquitin carboxyl-hydrolase	*Sinocyclocheilus rhinocerous*
HPA	LVLDAGDRTH	1095.56	100%	28	XP_028972366.2	Glycogenin-2	*Esox lucius*
HPA	LDKFF	668.35	100%	366	XP_041932362.1	Nesprin-1	*Alosa sapidissima*
HPP	LLDQDKSGFLE	1263.63	100%	0.049	P86432.1	Parvalbumin beta 2	*Oncorhynchus mykiss*
HPP	LDFDEFLMK	1156.54	100%	2.0	XP_042563305.1	Calcium-binding protein	*Clupea harengus*
HPP	LKYPLE	761.43	100%	190	XP_0411101389.1	A-kinase anchoring protein	*Polyodon spathula*
HPP	LLVYPW	789.44	100%	191	XP_014836463.1	Neuronal tyrosine	*Poecilia mexicana*
HPP	LVYPSV	676.37	100%	384	XP_043868191.1	Zinc finger protein	*Solea senegalensis*

**Table 3:** Association of top-scored peptides from each experimental condition with their most frequently identified bioactive motifs and predominant predicted functions, based on analysis using the BIOPEP-UWM database.

Origin	Main Peptide (OAC/ALC %)	Frequent Bioactive Motifs Identified	Predominant Associated Bioactivities
PBA	LLAPPE (97%)	AP, LA, LP, PP, PE	DPP-IV Inhibitor, ACE Inhibitor, α-Glucosidase Inhibitor
PBA	YEKK (96%)	EK, YE, KK	ACE Inhibitor, DPP-IV Inhibitor, Bacterial Permease Ligand
PBP	LEEEELKLF (97%)	KL, LF, EL, LK	ACE Inhibitor, Antioxidant
PBP	EYKK (95%)	YK, EY, KK	ACE Inhibitor, DPP-IV Inhibitor, Bacterial Permease Ligand
MFA	LDEVLKFF (95%)	KF, FF, LK, EV	ACE Inhibitor, DPP-IV Inhibitor, Renin Inhibitor
MFA	LEHEE (95%)	EH, HE	DPP-IV Inhibitor
MFP	LEEEELKLF (97%)	KL, LF, EL, LK, EE	ACE Inhibitor, Antioxidant, Vasoactive Substance Stimulator
MFP	FLDLDQDKKFEE (94%)	KF, FL, KK, EE	ACE Inhibitor, DPP-IV Inhibitor, Bacterial Permease Ligand
HPA	LTKLL (97%)	KY, LK	ACE Inhibitor, DPP-IV Inhibitor
HPA	LPTKF (95%)	LP, KF	ACE Inhibitor, DPP-IV Inhibitor
HPP	LKYPLE (95%)	YP, PL, KY, LK	ACE Inhibitor, DPP-IV Inhibitor, Antioxidant
HPP	VNVDPDGKF (94%)	KF, DG, DP, VD	ACE Inhibitor, DPP-IV Inhibitor, Renin Inhibitor

## Data Availability

All data generated or analyzed during this study are available upon request to the corresponding author.

## Supplementary Materials

The following supporting information can be downloaded at https://www.mdpi.com/article/10.3390/md24010036/s1. SM1–4: SDS-PAGE; SM5–7: HPLC-UV; SM8–13: MALDI-TOF; SM14–37: LC/MS; SM38–44: DeNovo peptide sequencing; SM45–46: Antimicrobial analysis.
