# Supplementary material for: Proteomic and Functional Characterization of Antimicrobial Peptides Derived from Fisheries Bycatch via Enzymatic Hydrolysis
Source: Mar Drugs. 2026 Jan 10;24(1):36. doi: 10.3390/md24010036 (PMC12843163; doi:10.3390/md24010036)
Supplement: Supplementary file 1 [file marinedrugs-24-00036-s001.zip › marinedrugs-4026000-supplementary.pdf]

marine drugs

Supplementary Materials (SM1-4)

SDS-PAGE: To confirm the extent of hydrolysis, 50 μg of each hydrolysate were diluted in sample buffer (0.06 M Tris-HCl, 2% SDS, 10% glycerol, 0.025% bromophenol blue; pH 6.8), heated at 70 °C for 5 minutes, and loaded onto 12% or 20% polyacrylamide gels. Electrophoresis was performed under denaturing conditions using Tris-Glycine-SDS buffer (pH 8.3), with an initial voltage of 150 V for 12 minutes, followed by 200 V for 40 minutes. Gels were stained with 0.025% Coomassie Brilliant Blue R-250 and destained with 30% ethanol and 10% acetic acid until background was clear. Images were captured after destaining.

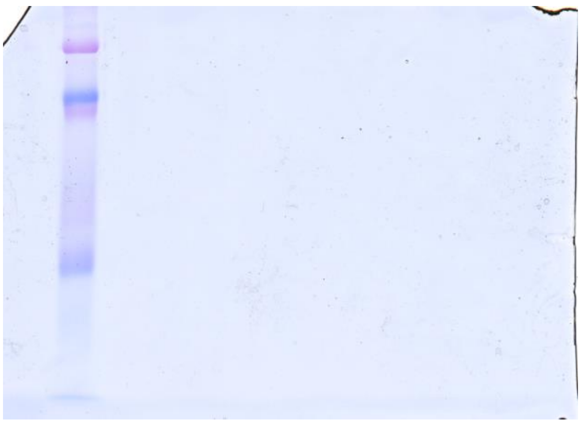

(SM1)

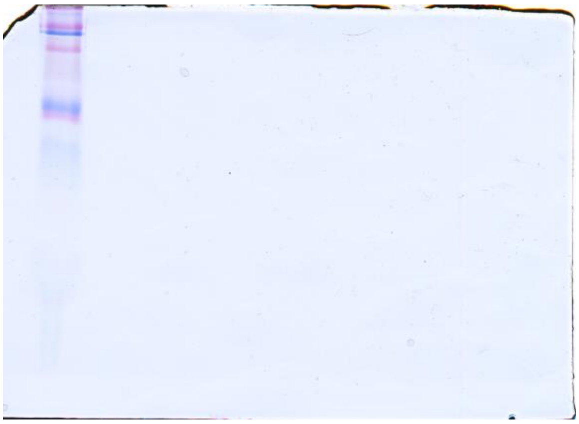

(SM2)

Figures SM1 and SM2: results of SDS-PAGE electrophoresis conducted on 12% and 20% mesh gels, respectively, of samples of *Paralonchurus brasiliensis, Micropogonias furnieri*, and *Hepatus Pudibundus*, which were hydrolyzed with Alcalase. The initial band in the gels corresponds to the standard protein band.

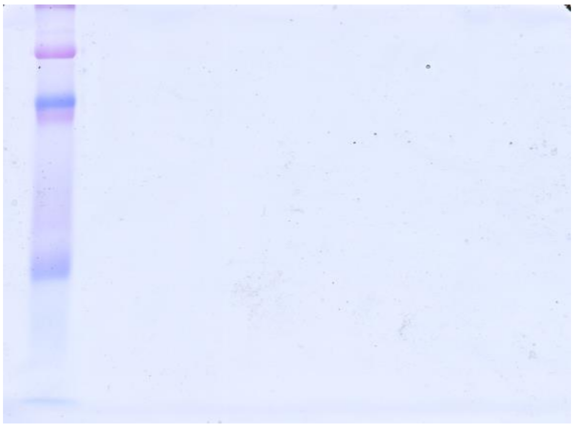

(SM3)

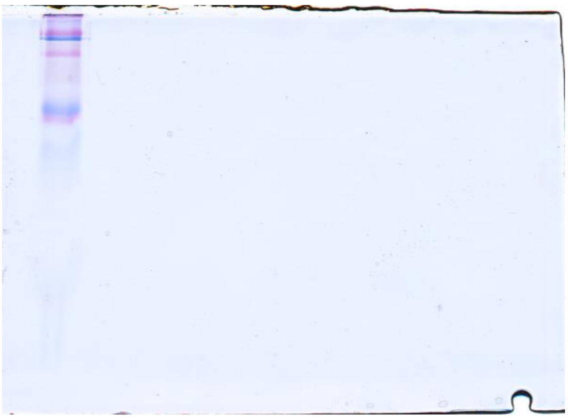

(SM4)

Figures SM3 and SM4: results of SDS-PAGE electrophoresis conducted on 12% and 20% mesh gels, respectively, of samples of *Paralonchurus brasiliensis, Micropogonias furnieri*, and *Hepatus*

 https://doi.org/10.3390/md24010036

Pudibundus, which were hydrolyzed with Protamex. The initial band in the gels corresponds to the standard protein band.

Supplementary Materials (SM5-7)

HPLC-UV:

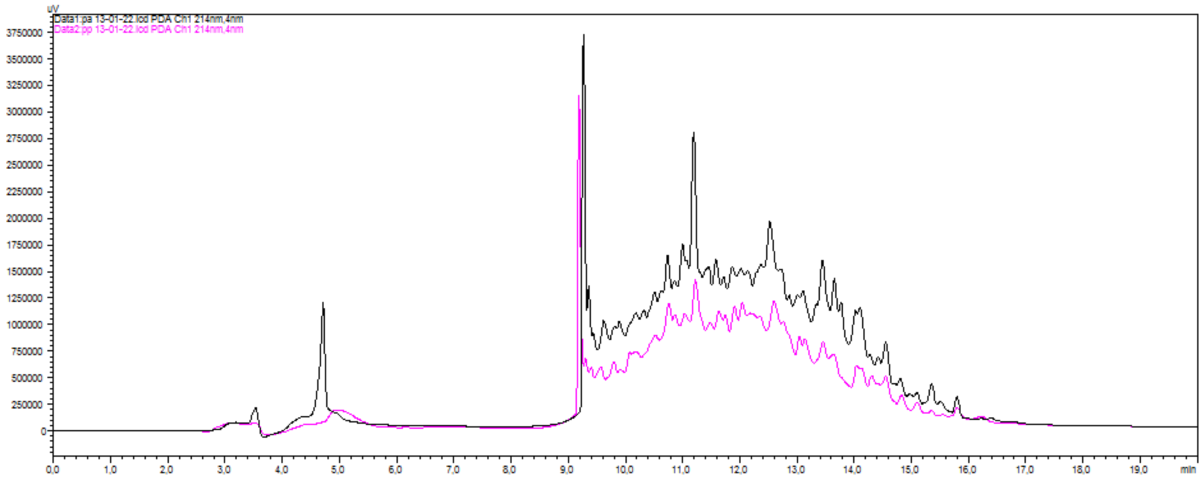

Figure SM5: Chromatographic profile of the *Paralonchurus brasiliensis* sample subjected to hydrolysis with Alcalase (black) and Protamex (pink).

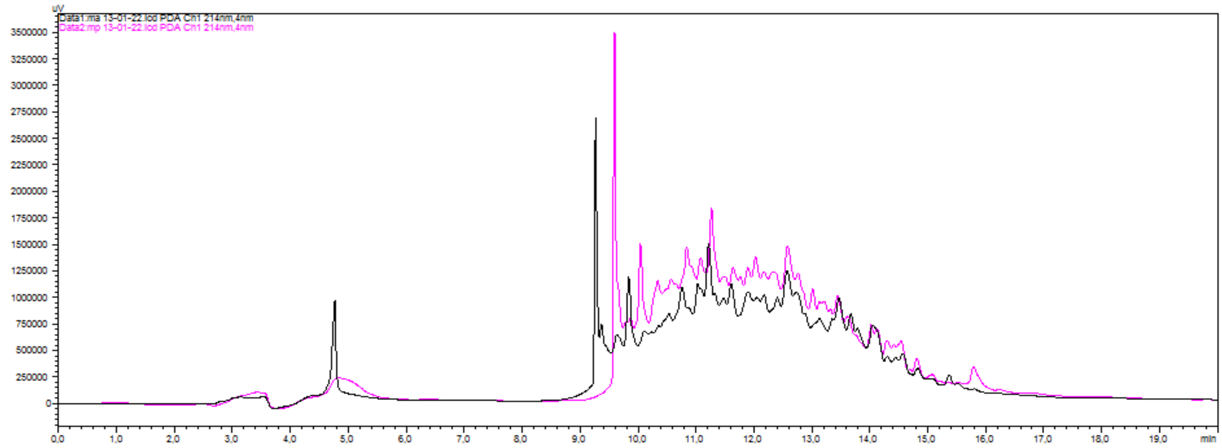

Figure SM6: Chromatographic profile of the *Micropogonias furnieri* sample subjected to hydrolysis with Alcalase (black) and Protamex (pink).

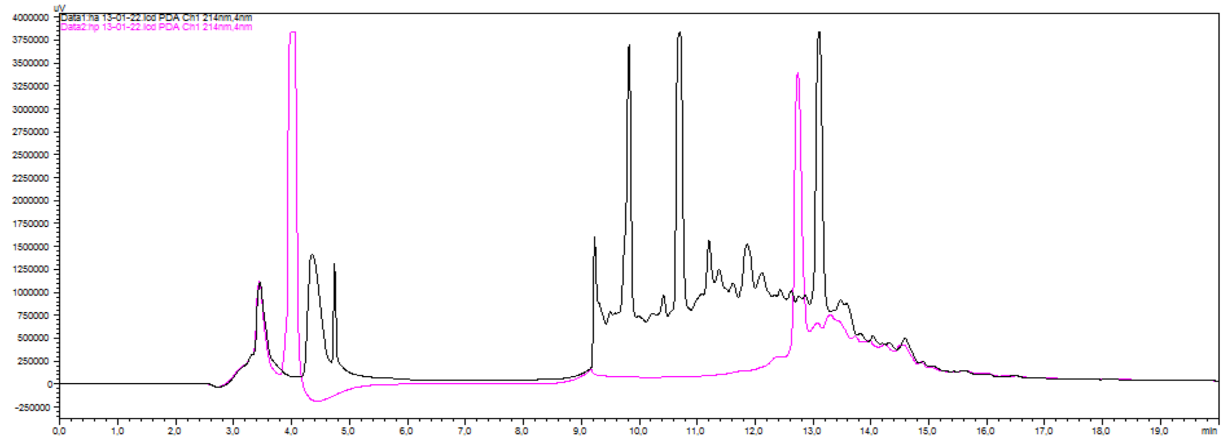

Figure SM7: Chromatographic profile of the *Hepatus pudibundus* sample subjected to hydrolysis with Alcalase (black) and Protamex (pink).

https://doi.org/10.3390/md24010036

Supplementary Materials (SM8-13)

MALDI-TOF:

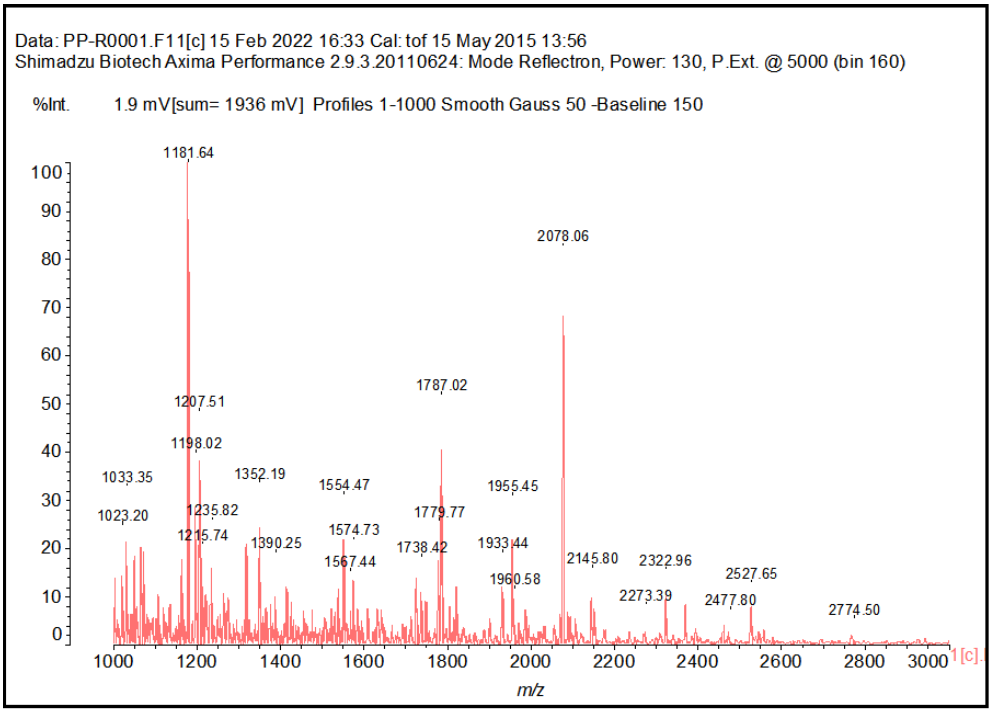

Figure SM8: Result of the MALDI-TOF mass spectrometry analysis generated from the sample of *Paralonchurus brasiliensis* hydrolyzed with the Alcalase enzyme.

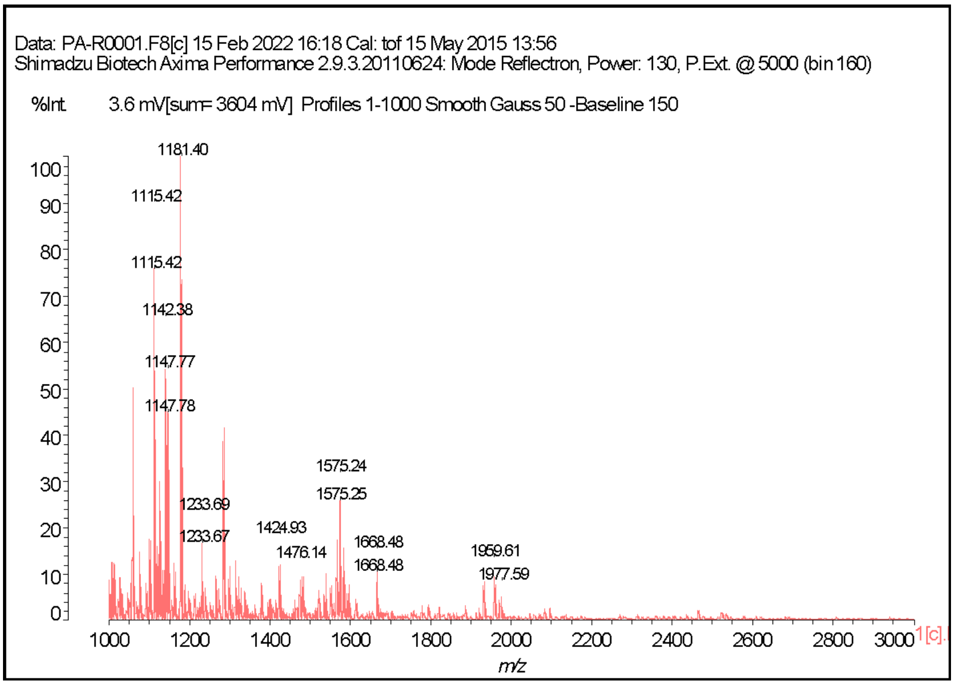

Figure SM9: Result of the MALDI-TOF mass spectrometry analysis generated from the sample of *Paralonchurus brasiliensis* hydrolyzed with the Protamex enzyme.

https://doi.org/10.3390/md24010036

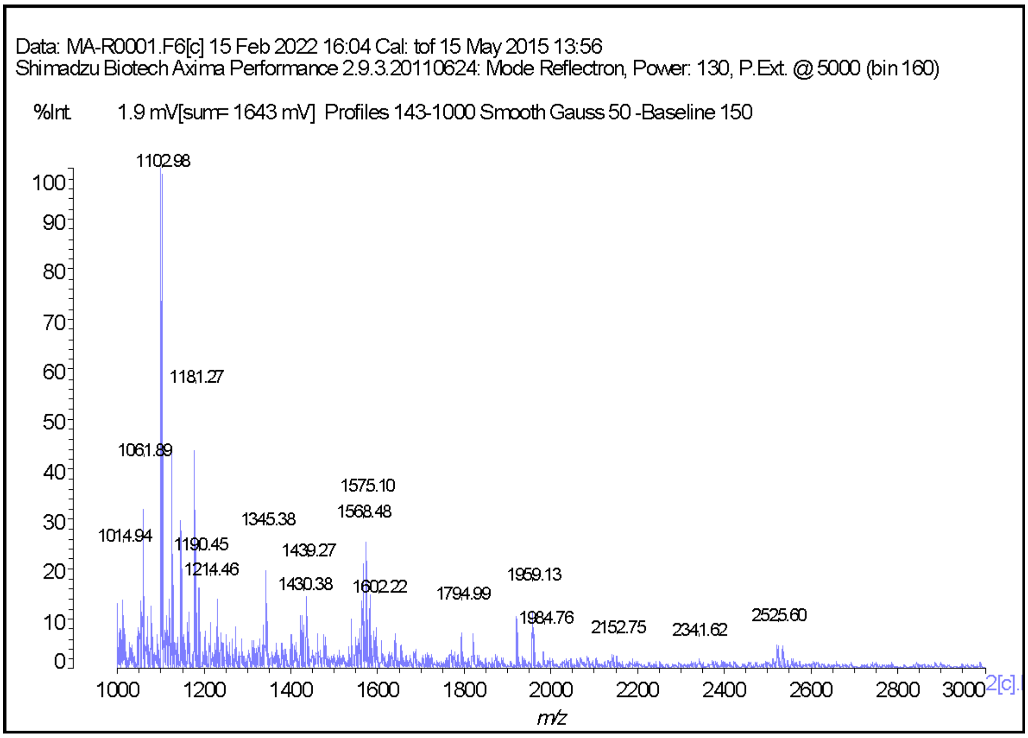

Figure SM10: Result of the MALDI-TOF mass spectrometry analysis generated from the sample of *Micropogonias furnieri* hydrolyzed with the Alcalase enzyme.

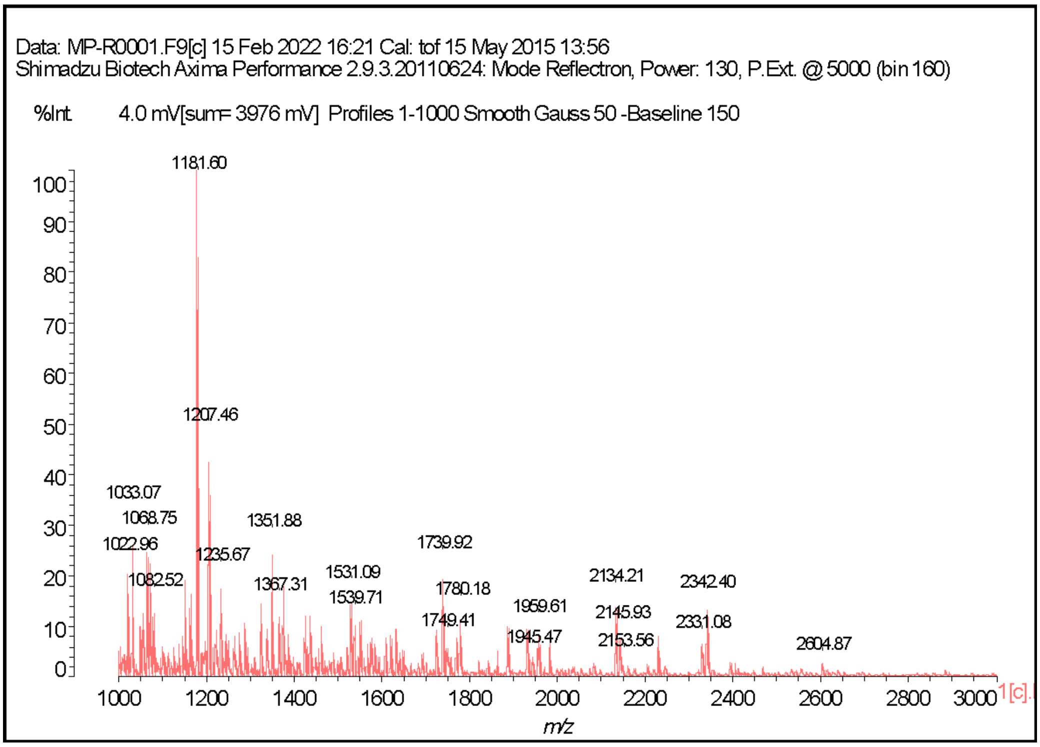

Figure SM11: Result of the MALDI-TOF mass spectrometry analysis generated from the sample of *Micropogonias furnieri* hydrolyzed with the Protamex enzyme.

https://doi.org/10.3390/md24010036

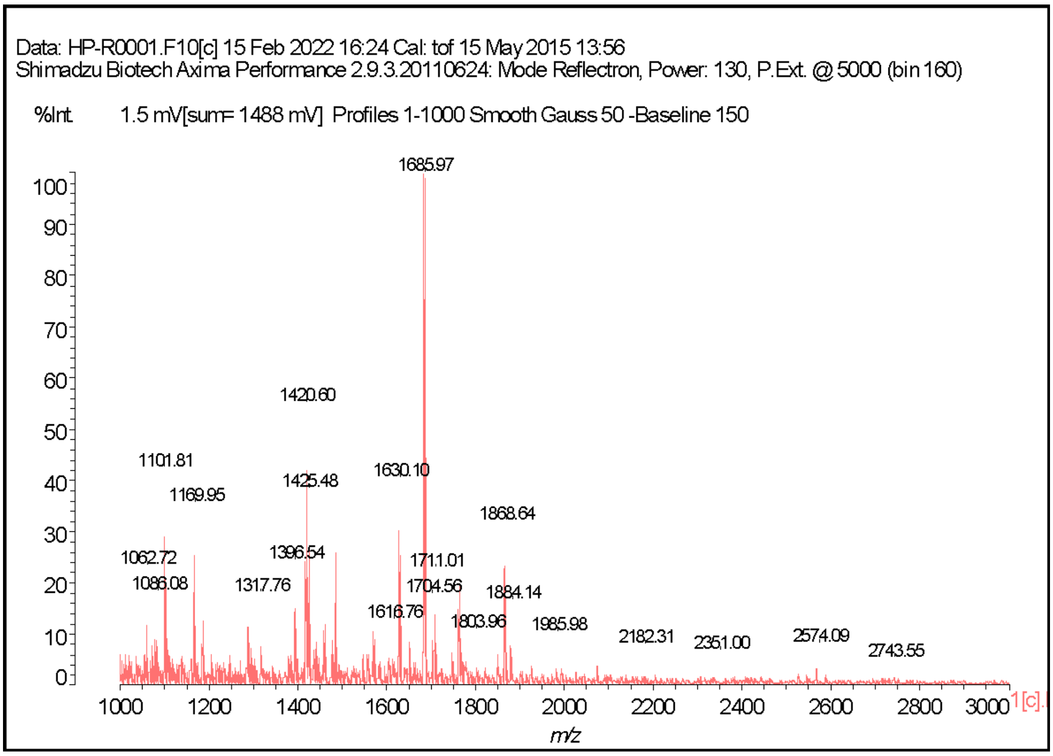

Figure SM12: Result of the MALDI-TOF mass spectrometry analysis generated from the sample of *Hepatus pudibundus* hydrolyzed with the Alcalase enzyme.

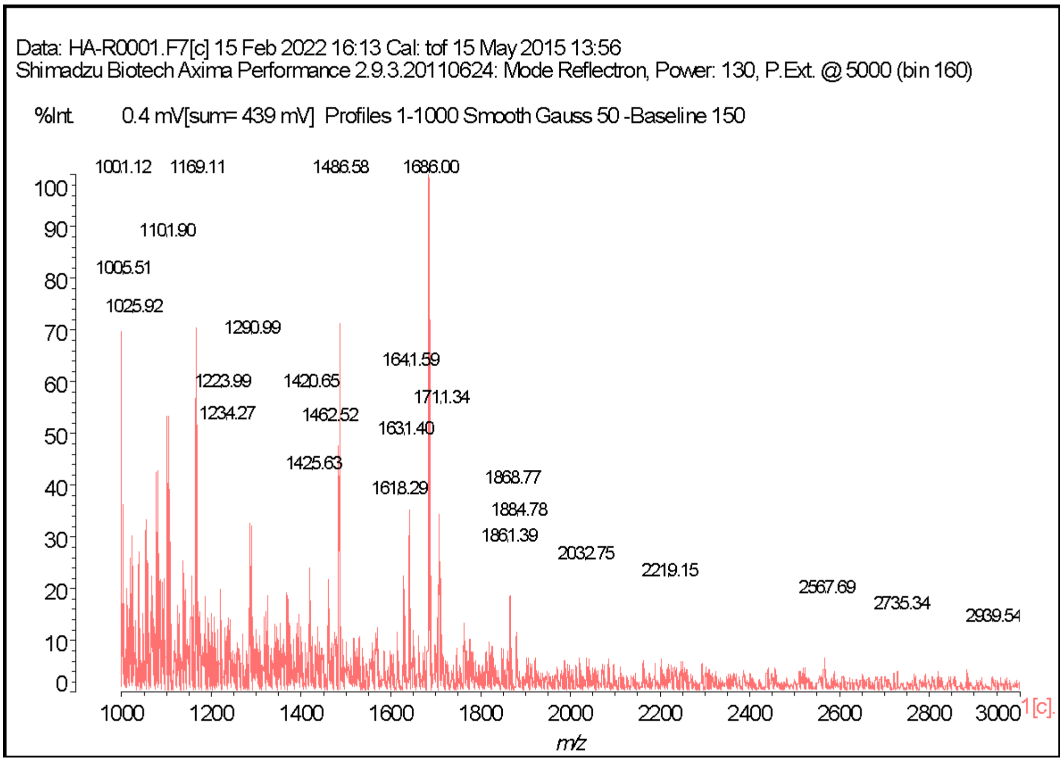

Figure SM13: Result of the MALDI-TOF mass spectrometry analysis generated from the sample of *Hepatus pudibundus* hydrolyzed with the Protamex enzyme.

Supplementary Materials (SM14-37)

LC/MS:

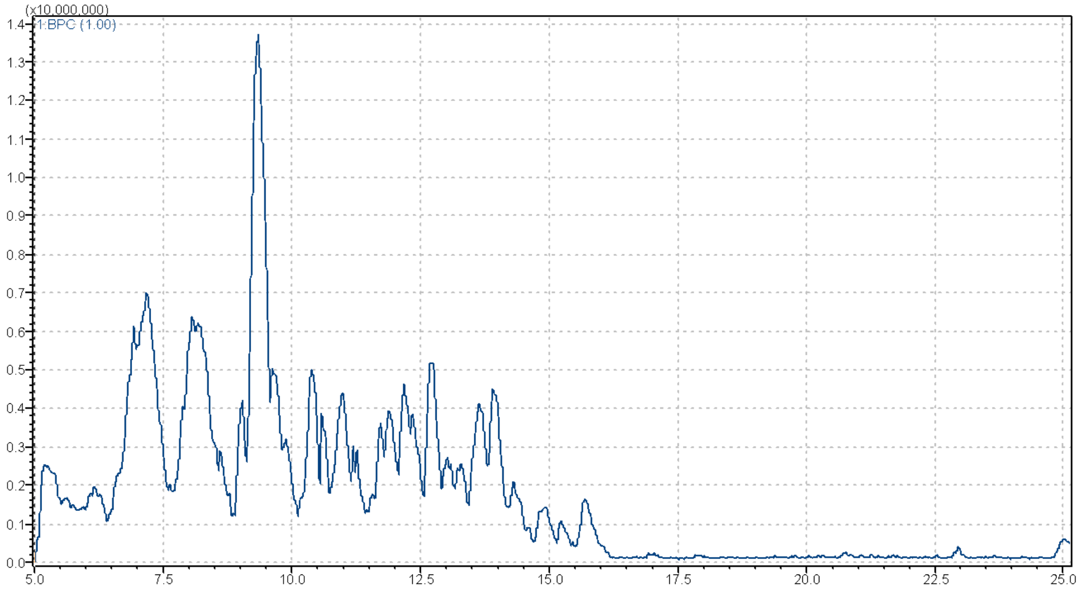

Figure SM14: The total peptide profile of the sample of *Paralonchurus brasiliensis* samples obtained through the hydrolysis with the enzyme Alcalase obtained by LC/MS analysis. This is a polar sample.

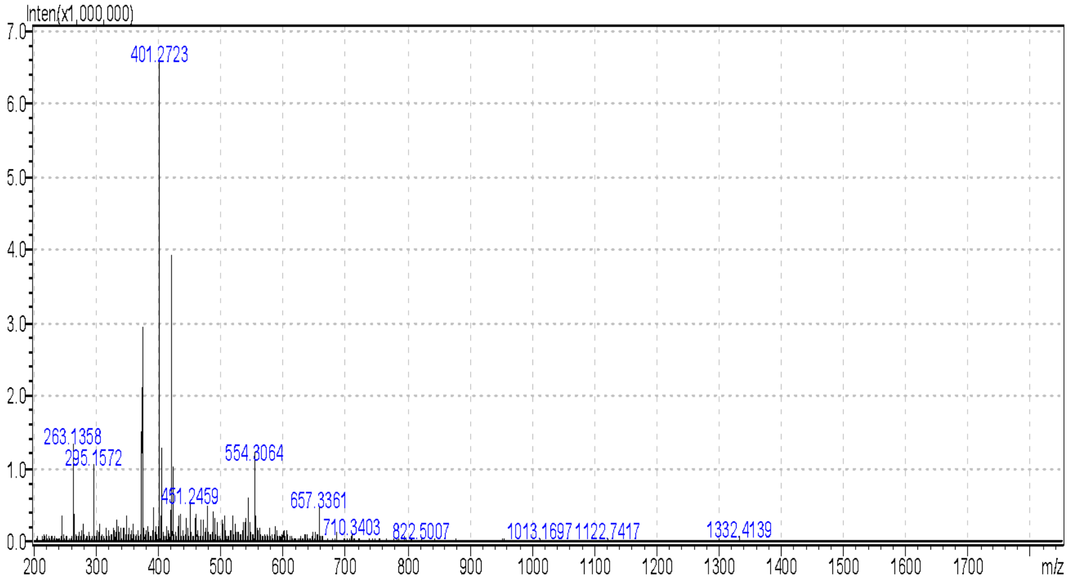

Figure SM15: Molecular masses obtained by LC/MS on the *Paralonchurus brasiliensis* hydrolyzed with Alcalase at a time of 7.14 minutes.

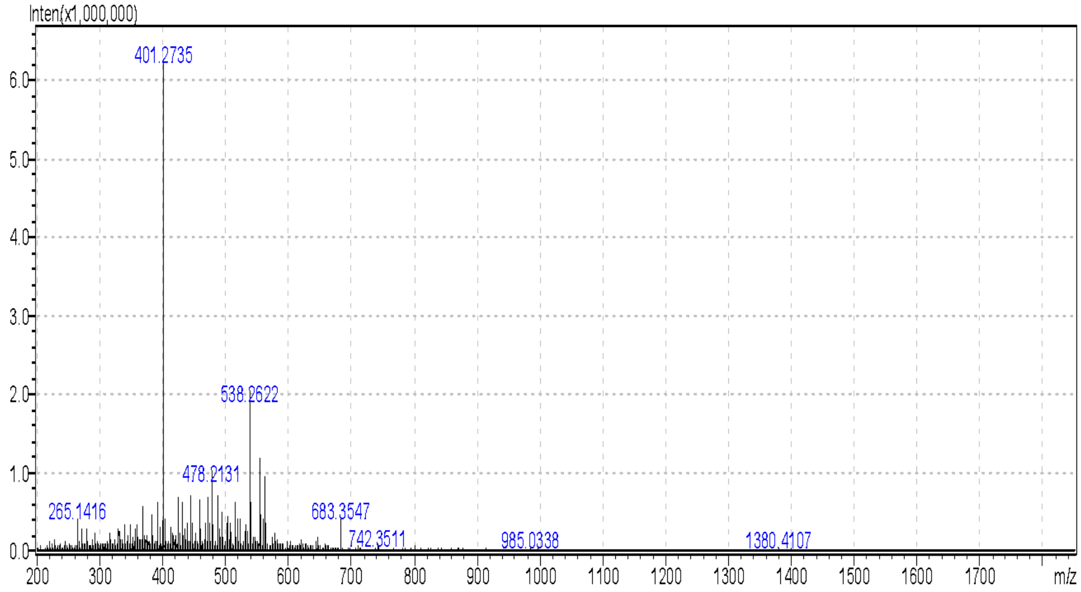

Figure SM16: Molecular masses obtained by LC/MS on the *Paralonchurus brasiliensis* hydrolyzed with Alcalase at a time of 8.17 minutes.

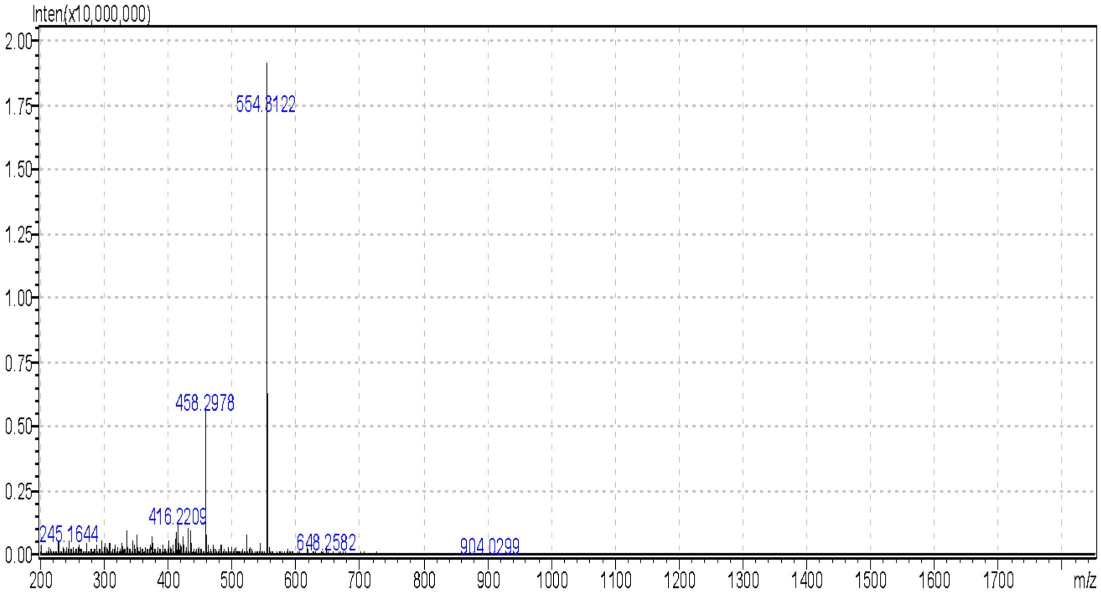

Figure SM17: Molecular masses obtained by LC/MS on the *Paralonchurus brasiliensis* hydrolyzed with Alcalase at a time of 9.35 minutes.

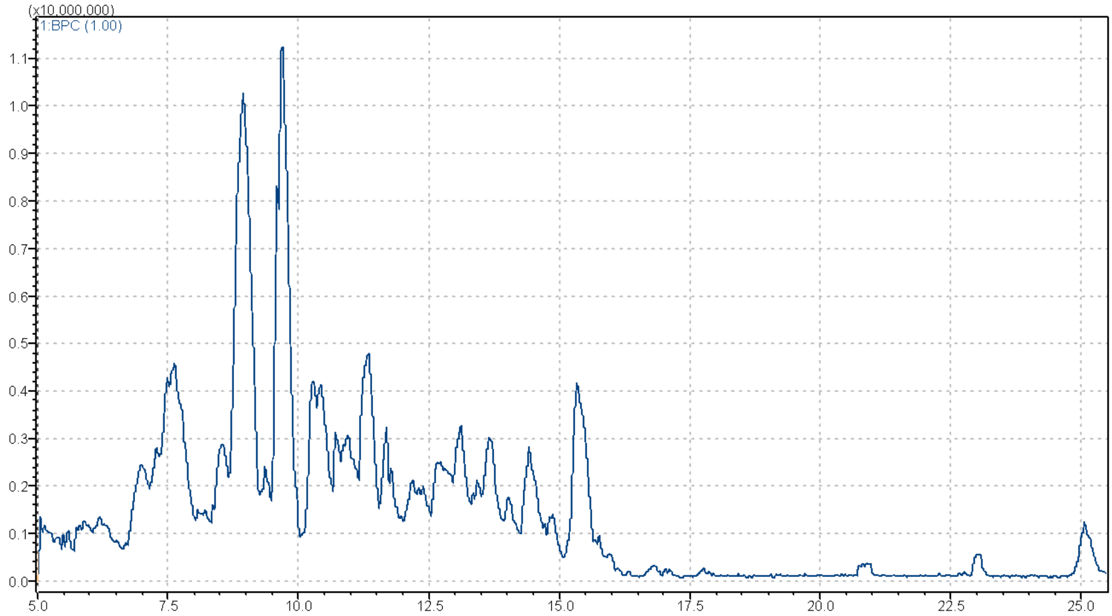

Figure SM18: The total peptide profile of the sample of *Paraloncurus brasiliensis* samples obtained through the hydrolysis with the enzyme Protamex obtained by LC/MS analysis. This is a polar sample.

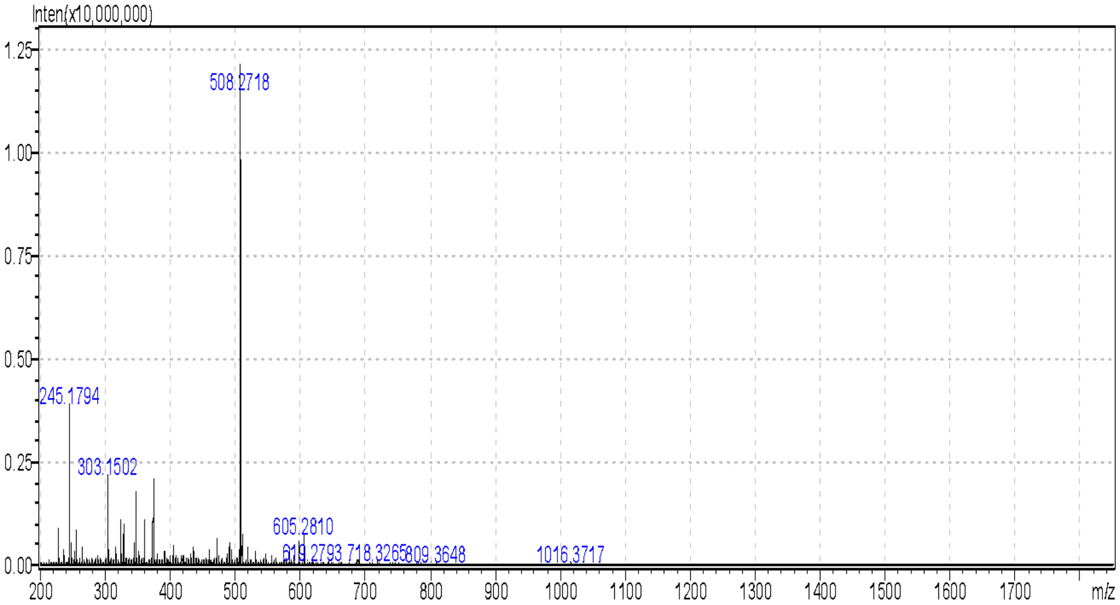

Figure SM19: Molecular masses obtained by LC/MS on the *Paraloncurus brasiliensis* hydrolyzed with Protamex at a time of 8.94 minutes.

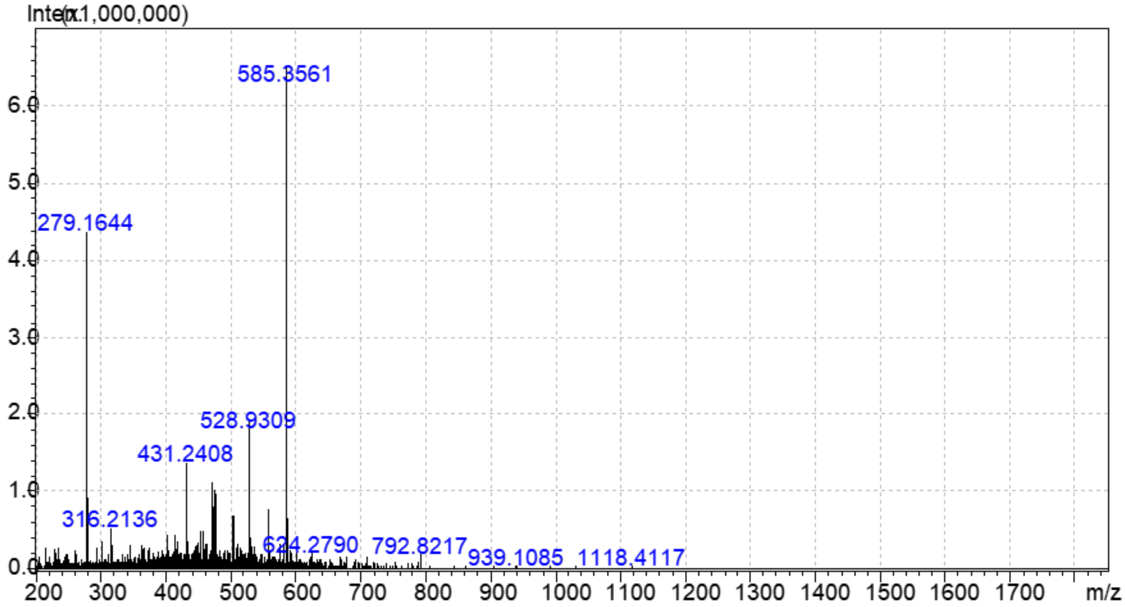

Figure SM20: Molecular masses obtained by LC/MS on the *Paralonchurus brasiliensis* hydrolyzed with Protamex at a time of 11.35 minutes.

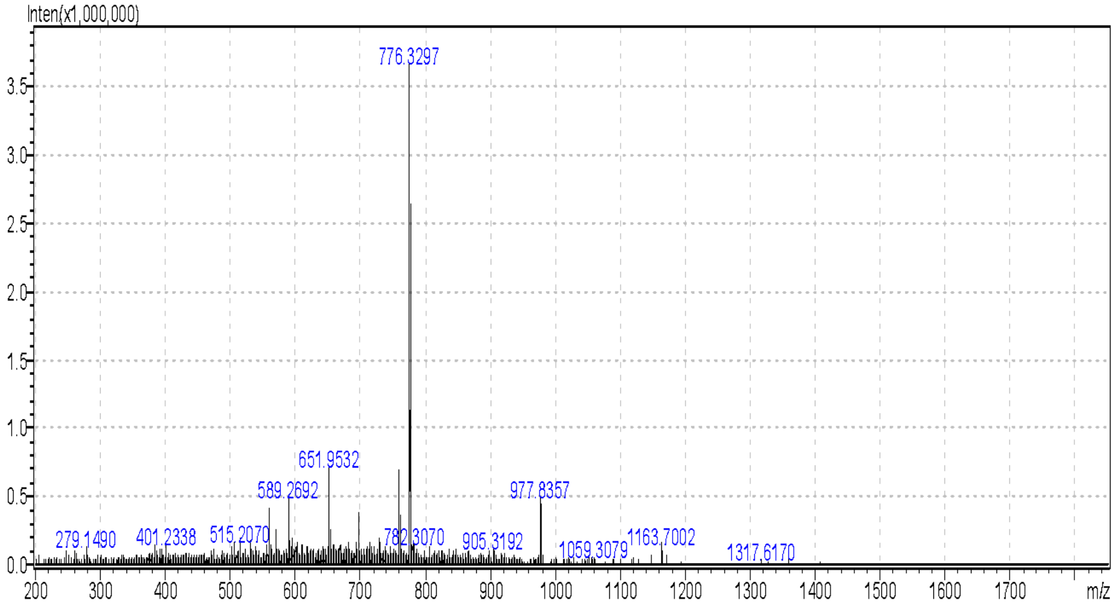

Figure SM21: Molecular masses obtained by LC/MS on the *Paralonchurus brasiliensis* hydrolyzed with Protamex at a time of 15.41.

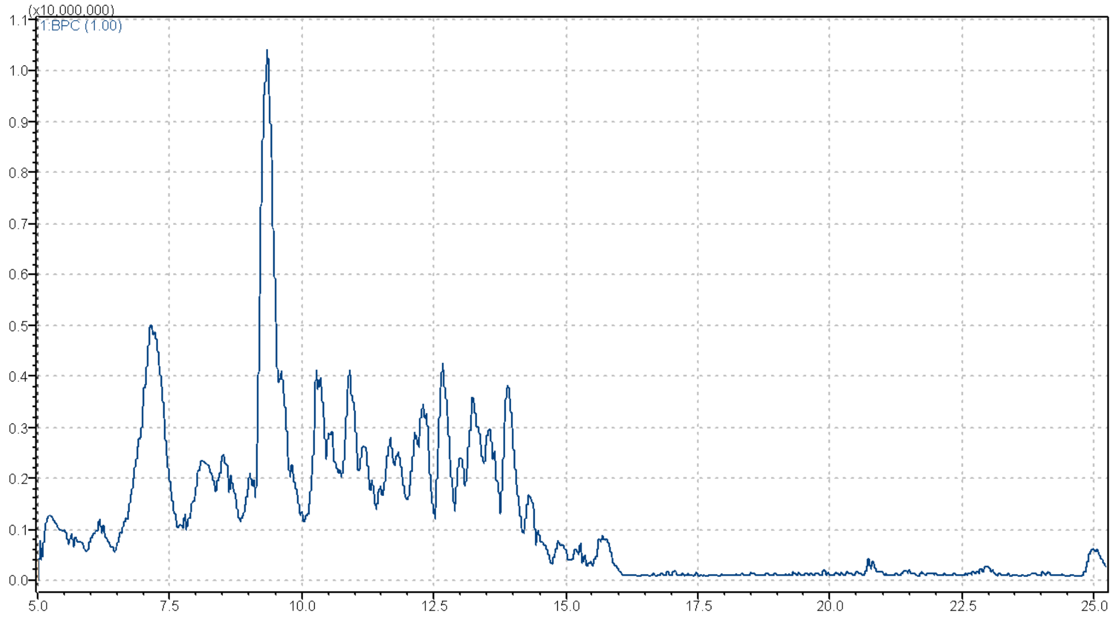

Figure SM22: The total peptide profile of the sample of *Micropogonias furnieri* samples obtained through the hydrolysis with the enzyme Protamex obtained by LC/MS analysis. This is a polar sample.

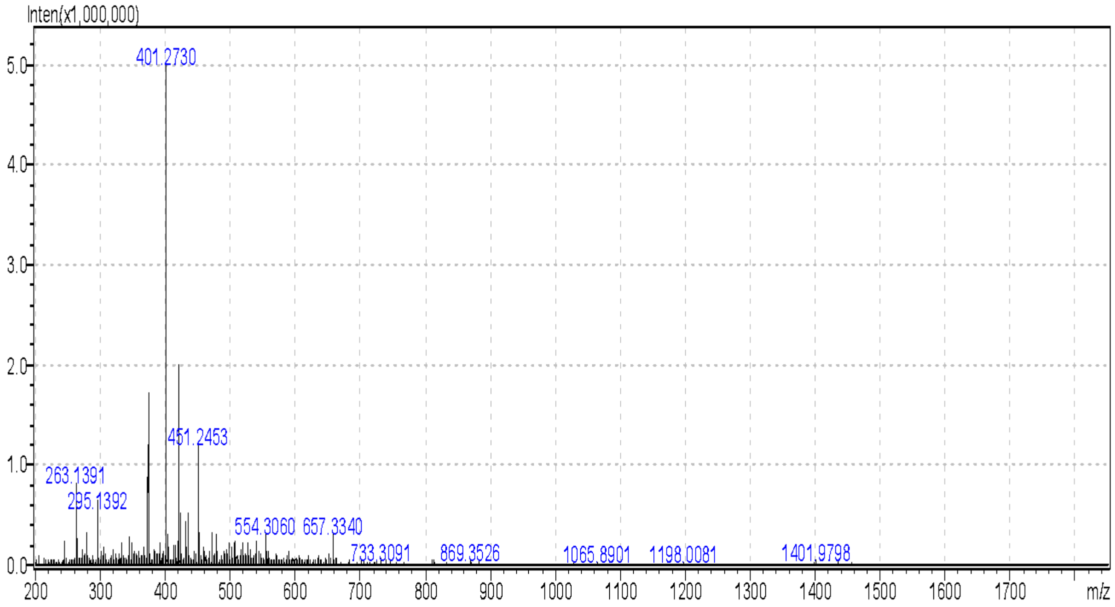

Figure SM23: Molecular masses obtained by LC/MS on the *Micropogonias furnieri* hydrolyzed with Alcalase at a time of 7.15 minutes.

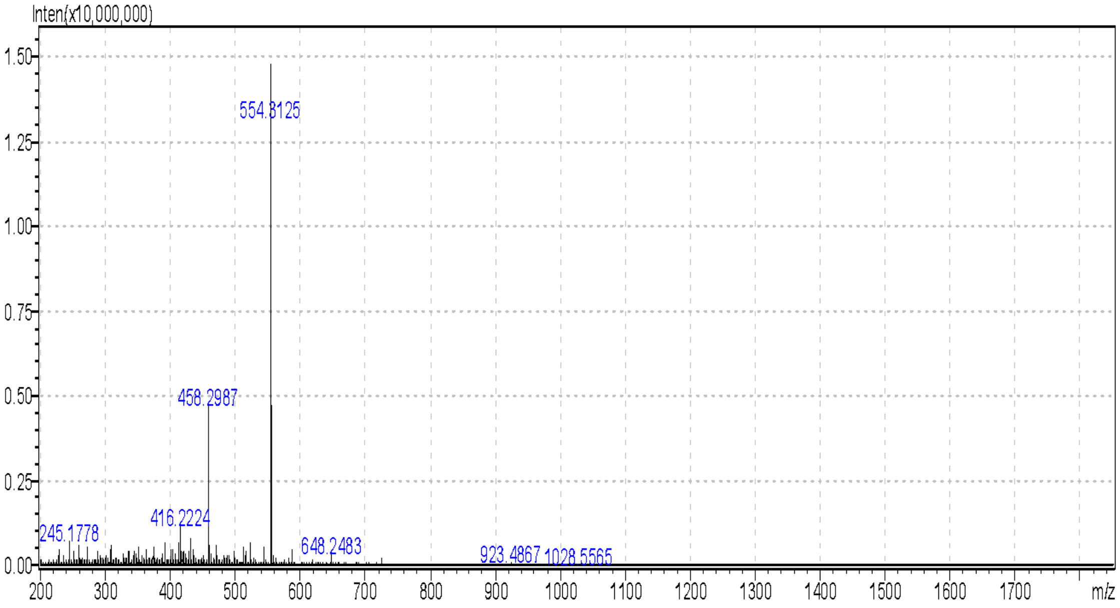

Figure SM24: Molecular masses obtained by LC/MS on the *Micropogonias furnieri* hydrolyzed with Alcalase at a time of 9.35 minutes.

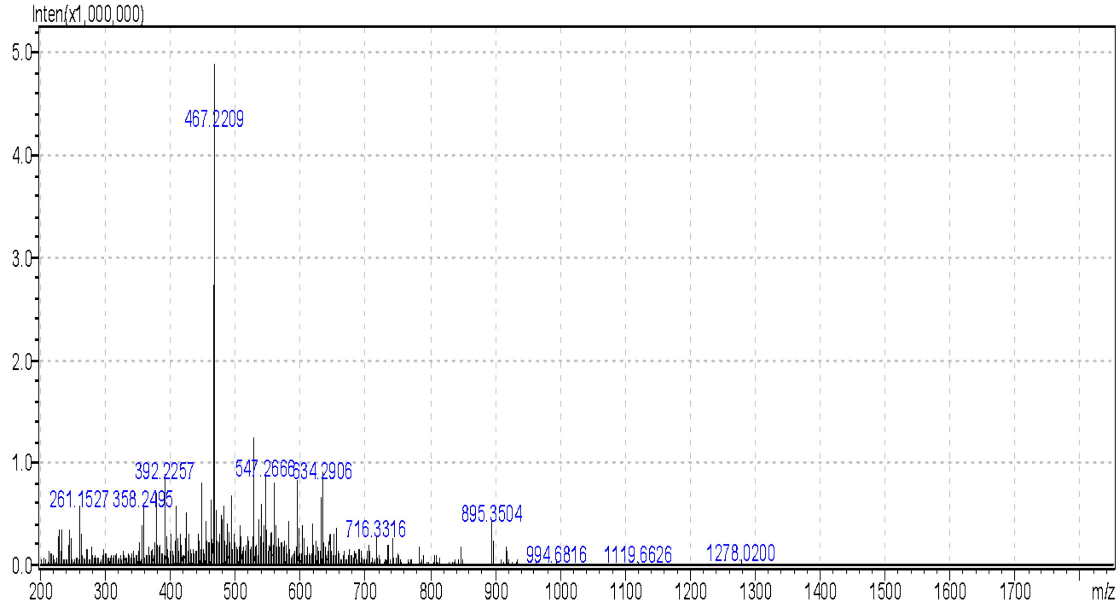

Figure SM25: Molecular masses obtained by LC/MS on the *Micropogonias furnieri* hydrolyzed with Alcalase at a time of 12.67 minutes.

https://doi.org/10.3390/md24010036

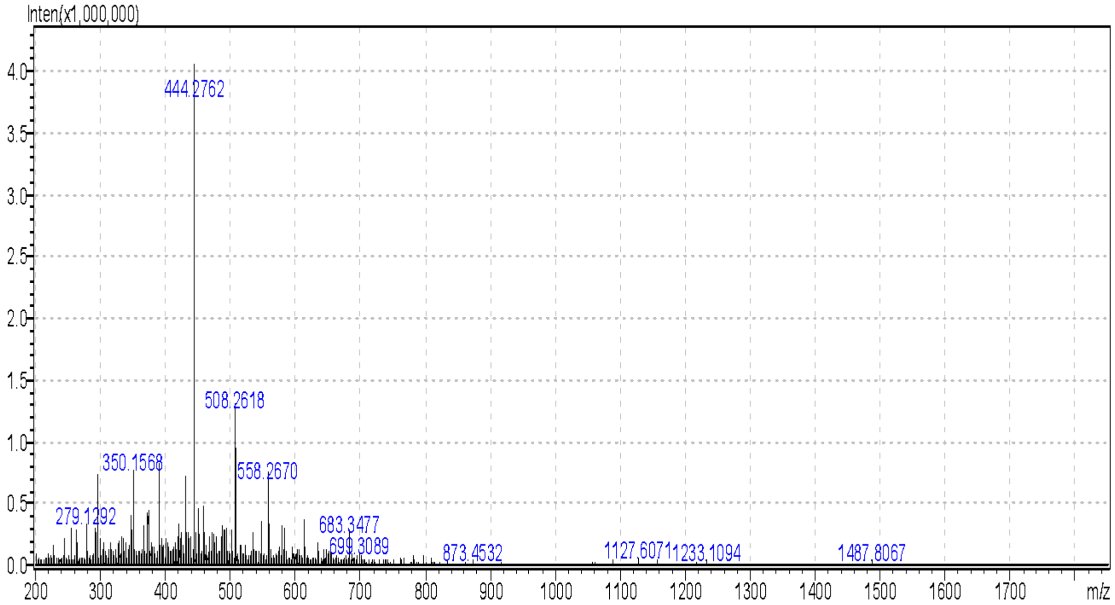

Figure SM26: The total peptide profile of the sample of *Micropogonias furnieri* samples obtained through the hydrolysis with the enzyme Protamex obtained by LC/MS analysis. This is a polar sample.

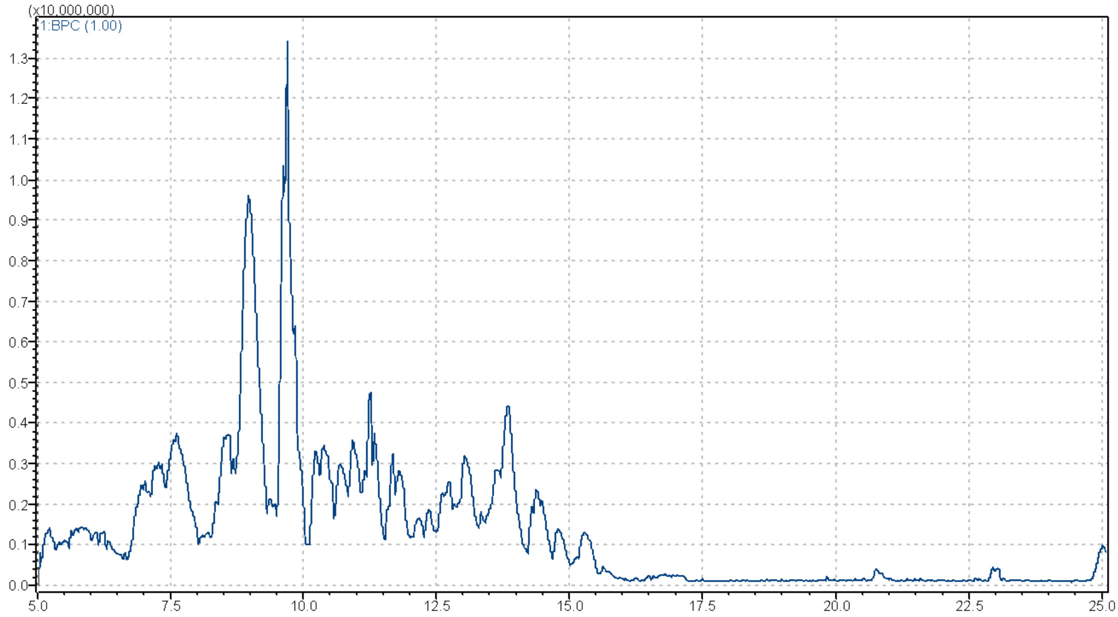

Figure SM27: Molecular masses obtained by LC/MS on the *Micropogonias furnieri* hydrolyzed with Protamex at a time of 7.5 minutes.

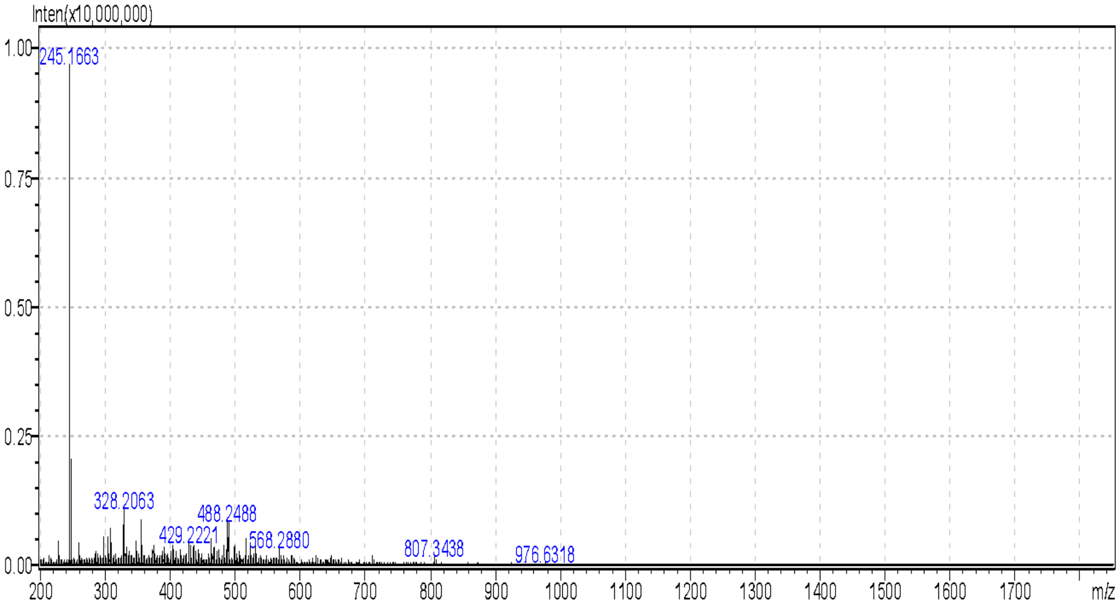

Figure SM28: Molecular masses obtained by LC/MS on the *Micropogonias furnieri* hydrolyzed with Protamex at a time of 8.9 minutes.

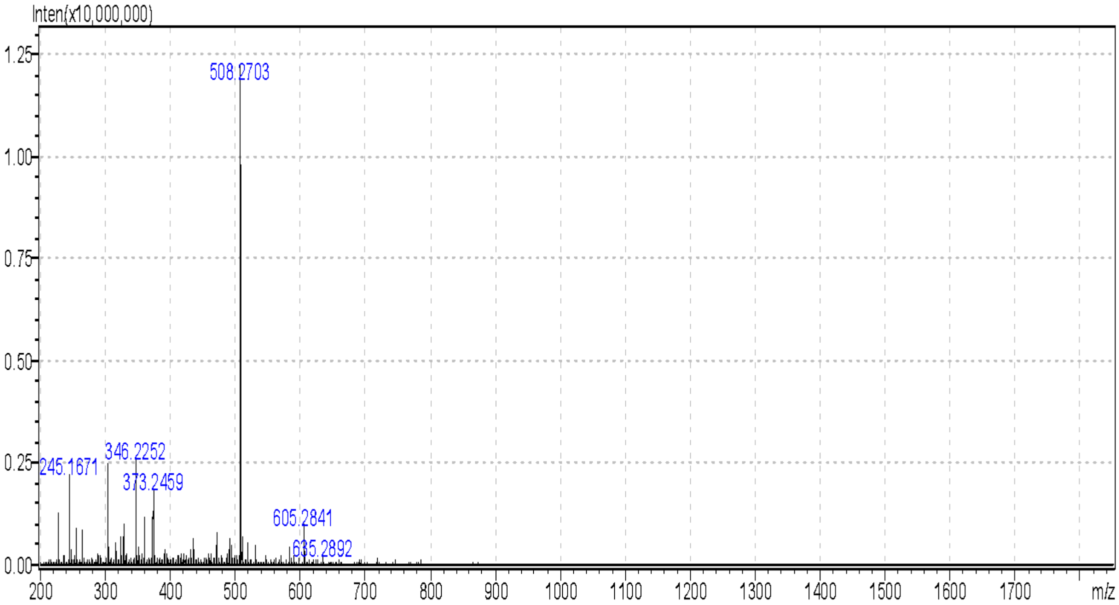

Figure SM29: Molecular masses obtained by LC/MS on the *Micropogonias furnieri* hydrolyzed with Protamex at a time of 9.65 minutes.

https://doi.org/10.3390/md24010036

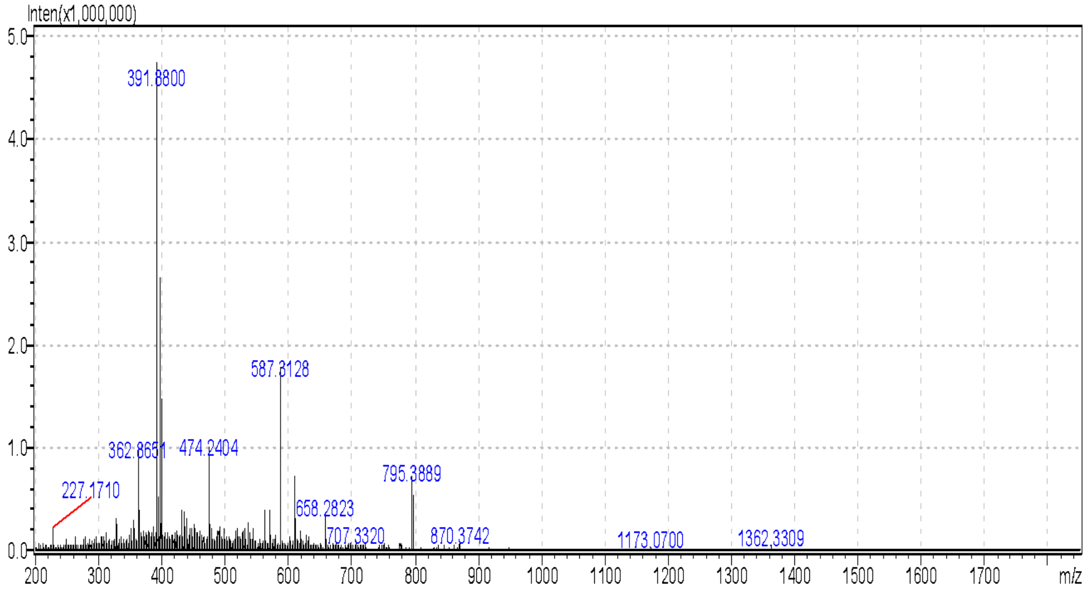

Figure SM30: The total peptide profile of the sample of *Hepatus pudibundus* samples obtained through the hydrolysis with the enzyme Alcalase obtained by LC/MS analysis. This is a polar sample.

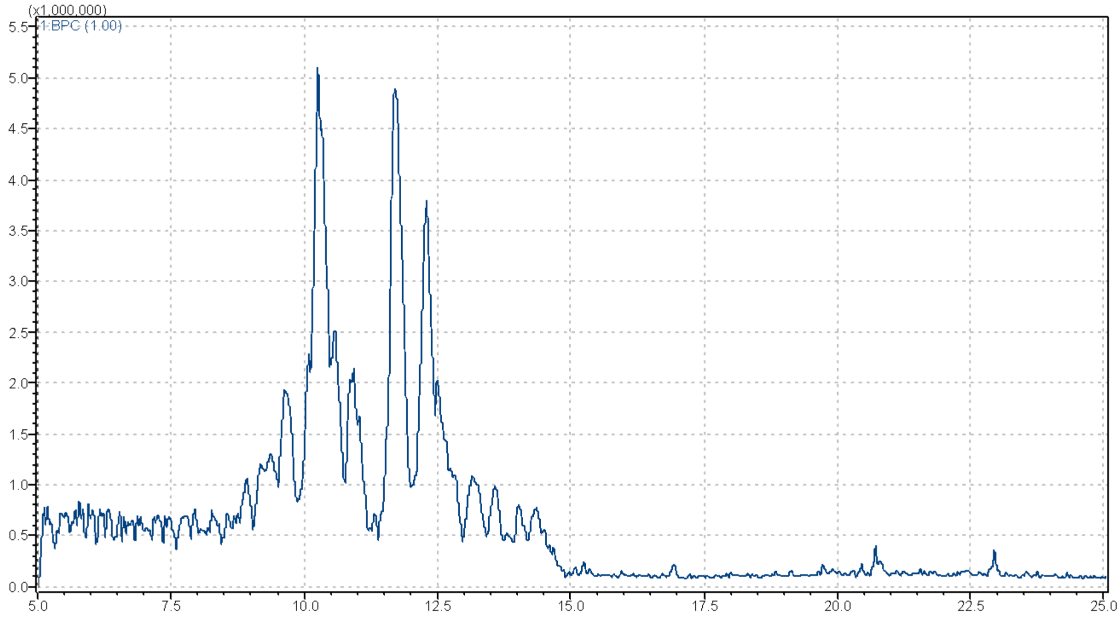

Figure SM31: Molecular masses obtained by LC/MS on the *Hepatus pudibundus* hydrolyzed with Alcalase at a time of 10.34 minutes.

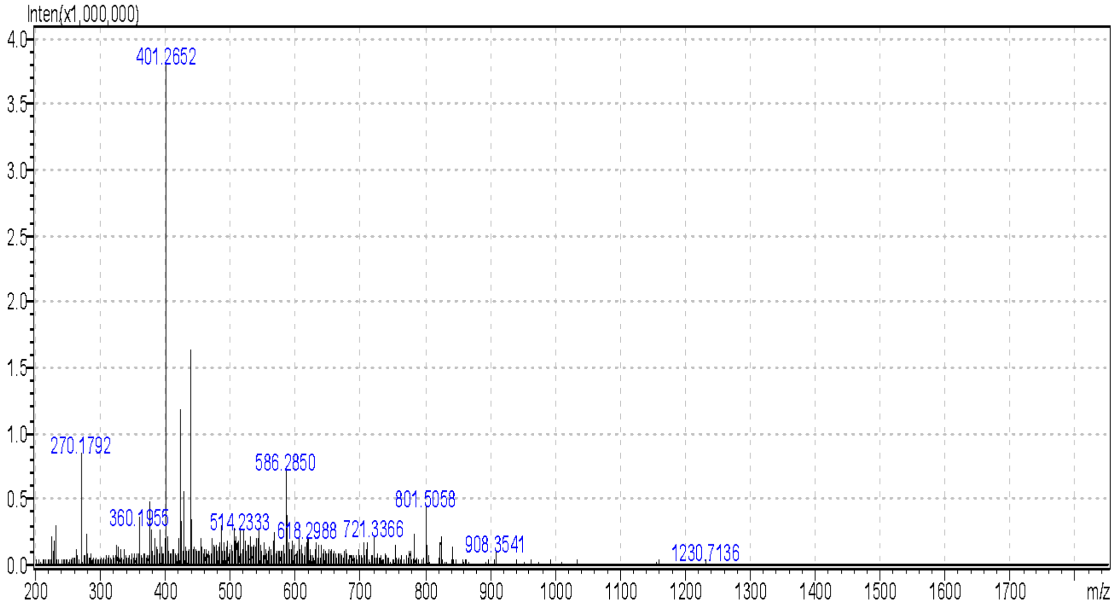

Figure SM32: Molecular masses obtained by LC/MS on the *Hepatus pudibundus* hydrolyzed with Alcalase at a time of 11.73 minutes.

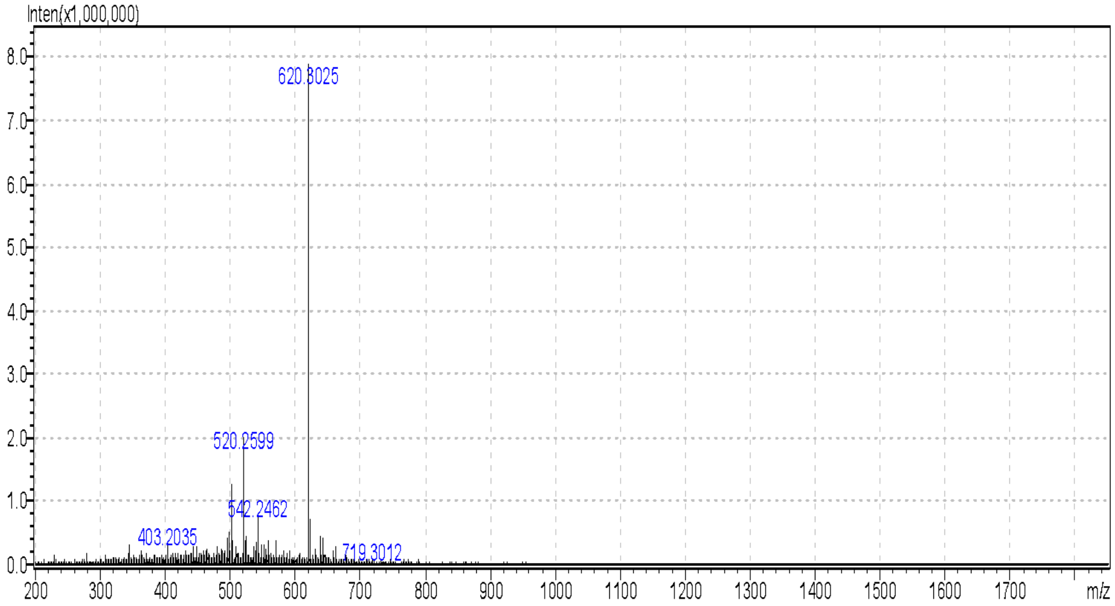

Figure SM33: Molecular masses obtained by LC/MS on the *Hepatus pudibundus* hydrolyzed with Alcalase at a time of 12.3 minutes.

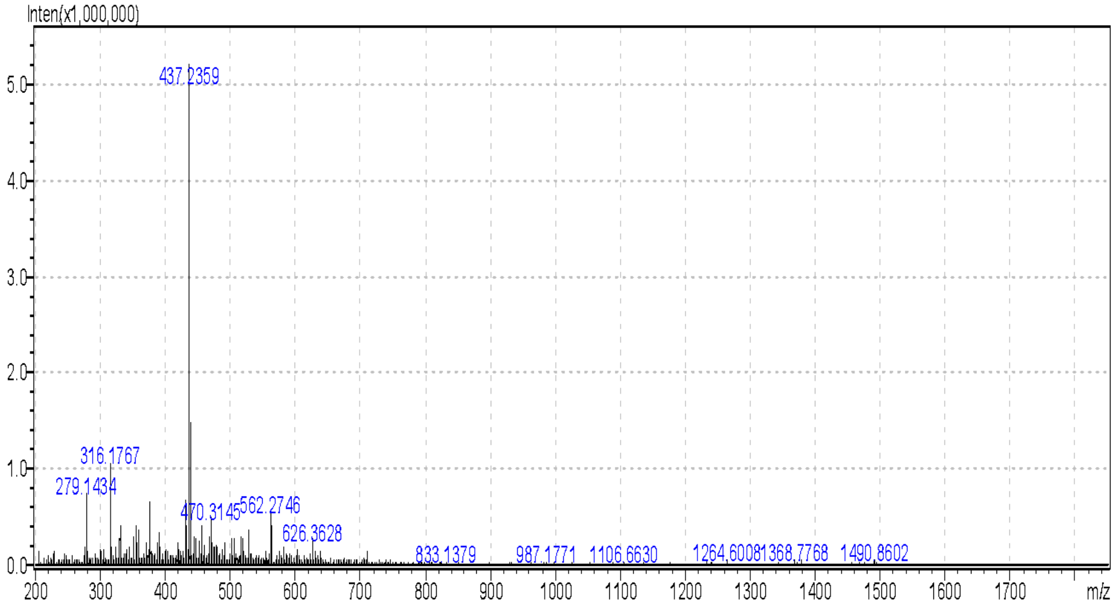

Figure SM34: The total peptide profile of the sample of *Hepatus pudibundus* samples obtained through the hydrolysis with the enzyme Protamex obtained by LC/MS analysis. This is an intermediary polar sample.

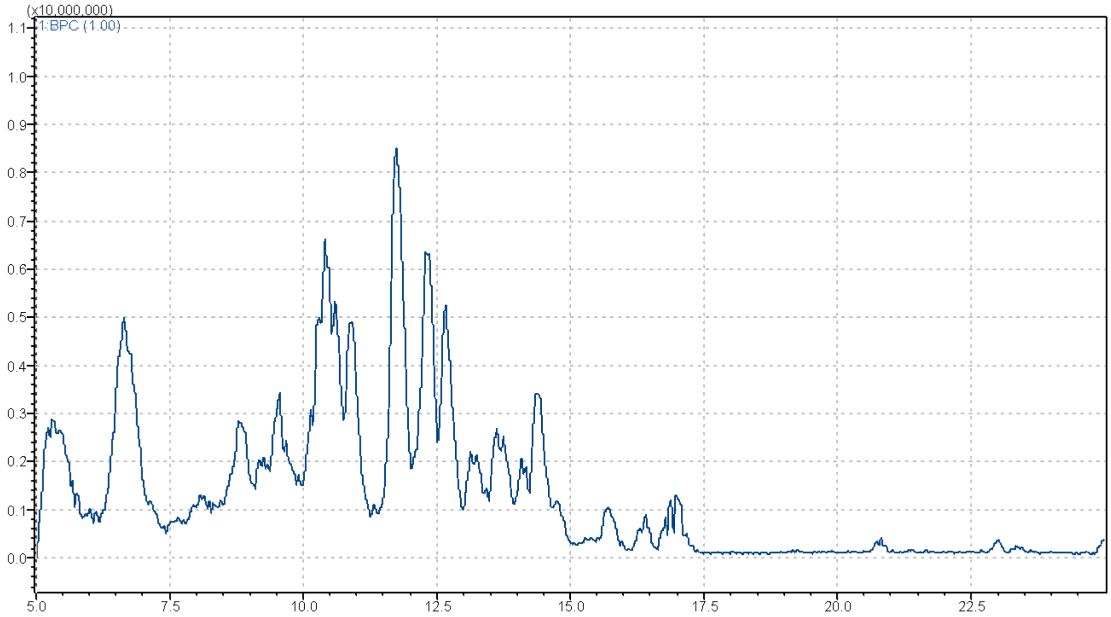

Figure SM35: Molecular masses obtained by LC/MS on the *Hepatus pudibundus* hydrolyzed with Protamex at a time of 6.63 minutes.

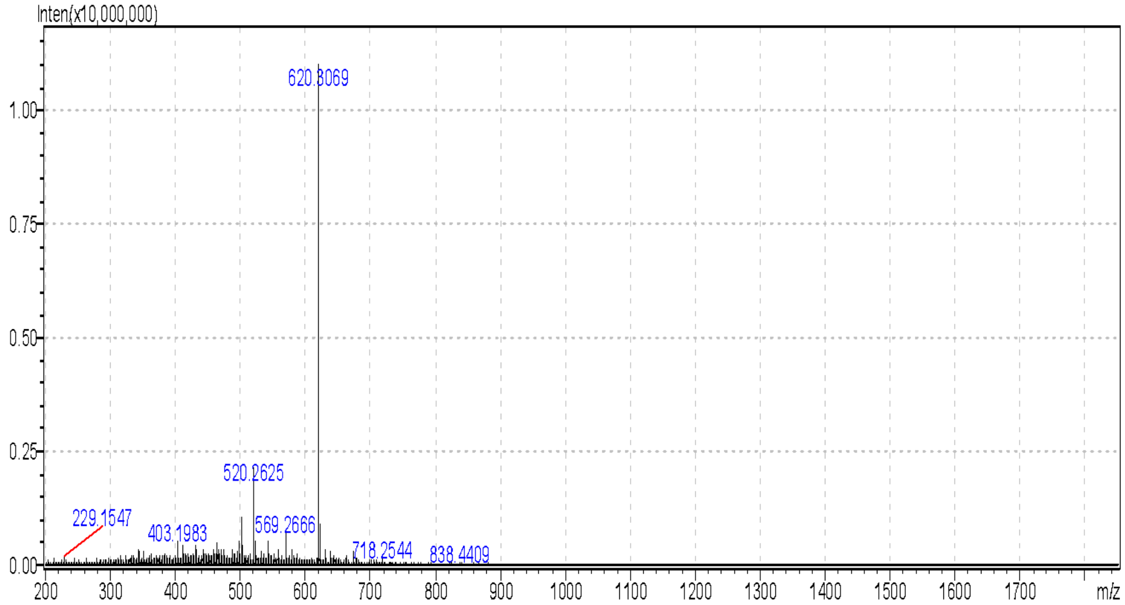

Figure SM36: Molecular masses obtained by LC/MS on the *Hepatus pudibundus* hydrolyzed with Protamex at a time of 10.44 minutes.

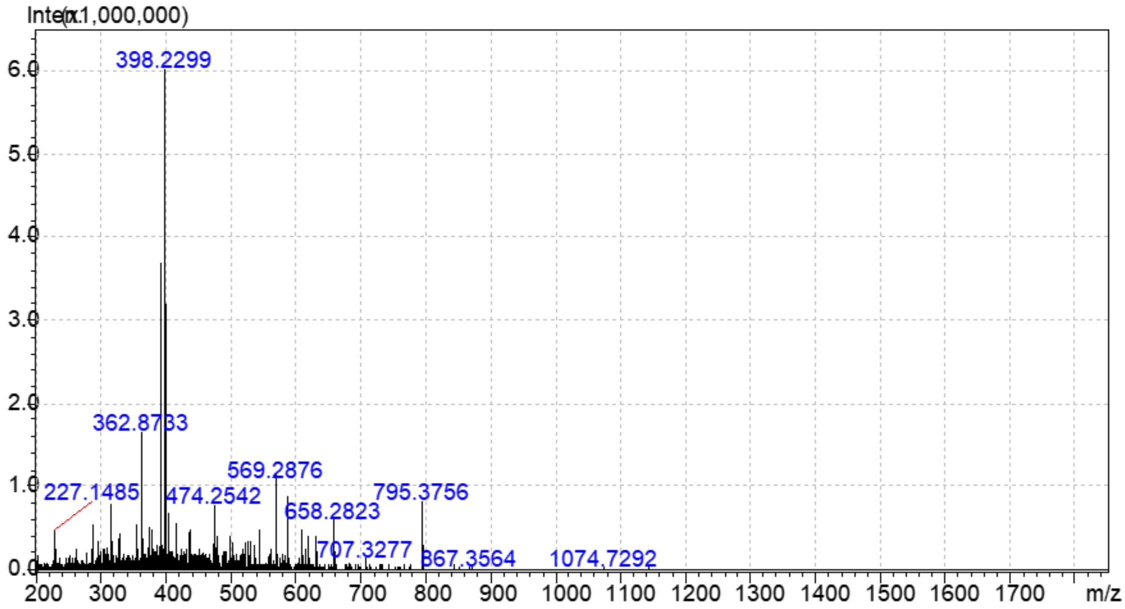

Figure SM37: Molecular masses obtained by LC/MS on the *Hepatus pudibundus* hydrolyzed with Protamex at a time of 11.79 minutes.

Supplementary Materials (SM38-44)

DeNovo peptide sequencing: performed using LC–MS/MS to support peptide identification independent of database availability. Due to file format limitations, the complete de novo sequencing tables and raw LC–MS/MS output files are provided as a compressed folder attached to the Supplementary Materials.

Supplementary Materials (SM45-46)

Antimicrobial analisys: Raw antimicrobial inhibition data (%) obtained from peptide hydrolysates and chromatographic fractions. Due to file format and size limitations, the complete raw datasets could not be embedded directly within the manuscript document. Therefore, all raw antimicrobial inhibition data supporting Figures 4 and 5 are provided as supplementary files in a compressed folder attached to the revised submission.

The datasets include raw inhibition values (%) from duplicate experiments (n = 2) for assays performed against *Staphylococcus aureus* ATCC 25953, *Escherichia coli* ATCC 25922, and *Candida albicans* ATCC 10231, as well as the corresponding calculated mean and standard deviation values.

Disclaimer/Publisher's Note: The statements, opinions and data contained in all publications are solely those of the individual author(s) and contributor(s) and not of MDPI and/or the editor(s). MDPI and/or the editor(s) disclaim responsibility for any injury to people or property resulting from any ideas, methods, instructions or products referred to in the content.

https://doi.org/10.3390/md24010036
