# Supplementary material for: Proteomic and Functional Characterization of Antimicrobial Peptides Derived from Fisheries Bycatch via Enzymatic Hydrolysis
Source: Mar Drugs. 2026 Jan 10;24(1):36. doi: 10.3390/md24010036 (PMC12843163; doi:10.3390/md24010036)
Supplement: Supplementary file 1 [file marinedrugs-24-00036-s001.zip › SM38/HA/protein.html]

proteins


Summary

  

# 1. Notes

# 2. Result Statistics

**Figure 1.**
False discovery rate (FDR) curve. X axis is the number of peptide-spectrum matches (PSM) being kept. Y axis is the corresponding FDR.

  


  

**Figure 2.**
PSM score distribution. **(a)**
Distribution of PEAKS peptide score; **(b)**
Scatterplot of PEAKS peptide score versus precursor mass error.

|  |  |  |  |
| --- | --- | --- | --- |
| **(a)**  |  | | **(b)**  |  | |

**Figure 3.**
De novo result validation. Distribution of residue local confidence: **(a)**
Residues in de novo sequences validated by confident database peptide assignment; **(b)**
Residues in "de novo only" sequences.

|  |  |  |  |
| --- | --- | --- | --- |
| **(a)**  |  | | **(b)**  |  | |

|  |  |  |  |  |  |  |  |  |  |  |  |  |  |  |  |  |  |  |  |  |  |  |  |  |  |  |  |  |  |  |  |  |  |  |  |
| --- | --- | --- | --- | --- | --- | --- | --- | --- | --- | --- | --- | --- | --- | --- | --- | --- | --- | --- | --- | --- | --- | --- | --- | --- | --- | --- | --- | --- | --- | --- | --- | --- | --- | --- | --- |
| **Table 1.** Statistics of data.  | # of MS Scans | 1020 | | # of MS/MS Scans | 2347 |    **Table 2.** Result filtration parameters.  | Peptide -10lgP | ≥15 | | Protein -10lgP | ≥20 | | Proteins unique peptides | ≥0 | | De novo ALC Score | ≥50% |    **Table 3.** Statistics of filtered result.  | Peptide-Spectrum Matches | 104 | | Peptide Sequences | 101 | | Protein Groups | 32 | | Proteins | 142 | | Proteins (#Unique Peptides) | 18 (>2); 9 (=2); 91 (=1); | | FDR (Peptide-Spectrum Matches) | 28.8% | | FDR (Peptide Sequences) | 29.7% | | FDR (Protein) | 5.6% | | De Novo Only Spectra | 619 | | **Table 4.** PTM profile.  | Name | ∆Mass | #PSM | Position | |

# 3. Experiment Control

**Figure 4.**
Precursor mass error of peptide-spectrum matches (PSM) in filtered result. **(a)**
Distribution of precursor mass error in ppm; **(b)**
Scatterplot of precursor m/z versus precursor mass error in ppm.

|  |  |  |  |
| --- | --- | --- | --- |
| **(a)**  |  | | **(b)**  |  | |

**Table 5.**
Number of identified peptides in each sample by the number of missed cleavages

|  |  |  |  |  |  |  |  |  |  |  |  |  |
| --- | --- | --- | --- | --- | --- | --- | --- | --- | --- | --- | --- | --- |
| |  |  |  |  |  |  | | --- | --- | --- | --- | --- | --- | | Missed Cleavages | 0 | 1 | 2 | 3 | 4+ | | Orbi2222 | 0 | 0 | 0 | 0 | 101 | |

# 4. Other Information

|  |  |  |  |
| --- | --- | --- | --- |
| **Table 6.** Search parameters.  | Search Engine Name: PEAKS 7.0 Parent Mass Error Tolerance: 0.03 Da Fragment Mass Error Tolerance: 0.1 Da Precursor Mass Search Type: monoisotopic Enzyme: None Max Missed Cleavages: 100 Non-specific Cleavage: both Max variable PTM per peptide: 3 Database: Arthropoda Taxon: All Searched Entry: 6735671 FDR Estimation: Enabled Merge Options: 0.1 min. 0.03 Da Precursor Options: corrected Charge Options: no correction Filter Options: no filter Process: true | | **Table 7.** Instrument parameters.  | Fractions: OB\_PIMENTA\_HA\_20220211\_01.raw Ion Source: ESI(nano-spray) Fragmentation Mode: CID/ECD MS Scan Mode: FT-ICR/Orbitrap MS/MS Scan Mode: Linear Ion Trap | |

  

Protein List

  

| Protein Group | Protein ID | Accession | -10lgP | Coverage (%) | #Peptides | #Unique | PTM | Avg. Mass | Description |
| --- | --- | --- | --- | --- | --- | --- | --- | --- | --- |
| 1 | 53234 | tr|A0A3R7M007|A0A3R7M007\_PENVA | 115.11 | 31 | 21 | 21 | N | 41323 | Actin 1 OS=Penaeus vannamei OX=6689 GN=C7M84\_012584 PE=3 SV=1 |
| 1 | 53235 | tr|A0A2H4V3D7|A0A2H4V3D7\_PENVA | 115.11 | 31 | 21 | 21 | N | 41688 | Actin 1 OS=Penaeus vannamei OX=6689 GN=C7M84\_012586 PE=2 SV=1 |
| 1 | 53236 | tr|H9B3Y9|H9B3Y9\_SCYPA | 115.11 | 31 | 21 | 21 | N | 41781 | Beta-actin OS=Scylla paramamosain OX=85552 PE=2 SV=1 |
| 1 | 53237 | tr|A0A6A0H005|A0A6A0H005\_HYAAZ | 115.11 | 31 | 21 | 21 | N | 41604 | Uncharacterized protein OS=Hyalella azteca OX=294128 GN=HAZT\_HAZT002759 PE=3 SV=1 |
| 1 | 53238 | tr|A0A3R7NX50|A0A3R7NX50\_PENVA | 115.11 | 31 | 21 | 21 | N | 41716 | Actin 1 OS=Penaeus vannamei OX=6689 GN=C7M84\_012585 PE=3 SV=1 |
| 1 | 53239 | tr|A0A482LUC2|A0A482LUC2\_CANBE | 115.11 | 31 | 21 | 21 | N | 41781 | Actin 1 OS=Cancer borealis OX=39395 GN=ACT1 PE=2 SV=1 |
| 1 | 53240 | tr|A0A0B5J9E4|A0A0B5J9E4\_ERISI | 115.11 | 31 | 21 | 21 | N | 41751 | Skeletal muscle actin 1 OS=Eriocheir sinensis OX=95602 GN=ActSK1 PE=2 SV=1 |
| 1 | 53241 | tr|A0A2H4V3D6|A0A2H4V3D6\_PENVA | 115.11 | 31 | 21 | 21 | N | 41801 | Slow-type skeletal muscle actin 10 OS=Penaeus vannamei OX=6689 PE=2 SV=1 |
| 1 | 53242 | tr|A0A2H4V3F0|A0A2H4V3F0\_PENVA | 115.11 | 31 | 21 | 21 | N | 41743 | Slow-type skeletal muscle actin 7 OS=Penaeus vannamei OX=6689 PE=2 SV=1 |
| 1 | 53243 | tr|U3M7F7|U3M7F7\_PORTR | 115.11 | 31 | 21 | 21 | N | 41793 | Skeletal muscle actin 1 OS=Portunus trituberculatus OX=210409 PE=2 SV=1 |
| 10 | 53387 | tr|A7BJ42|A7BJ42\_PROCL | 88.49 | 13 | 4 | 1 | N | 14275 | Calcification associated soluble matrix protein 2 OS=Procambarus clarkii OX=6728 GN=casp-2 PE=2 SV=1 |
| 10 | 53388 | tr|D7RVB0|D7RVB0\_CALSI | 88.49 | 12 | 4 | 1 | N | 15426 | Early cuticle protein 3 OS=Callinectes sapidus OX=6763 PE=2 SV=1 |
| 10 | 53389 | tr|D7RVB1|D7RVB1\_CALSI | 88.49 | 12 | 4 | 1 | N | 15460 | Early cuticle protein 4 OS=Callinectes sapidus OX=6763 PE=2 SV=1 |
| 10 | 53390 | tr|D7RVA8|D7RVA8\_CALSI | 88.49 | 12 | 4 | 1 | N | 15521 | Early cuticle protein 1 OS=Callinectes sapidus OX=6763 PE=2 SV=1 |
| 10 | 53391 | tr|D7RVA9|D7RVA9\_CALSI | 88.49 | 12 | 4 | 1 | N | 15462 | Early cuticle protein 2 OS=Callinectes sapidus OX=6763 PE=2 SV=1 |
| 10 | 53392 | tr|A0A5B7E6L1|A0A5B7E6L1\_PORTR | 88.49 | 11 | 4 | 1 | N | 17015 | Endocuticle structural glycoprotein SgAbd-1 OS=Portunus trituberculatus OX=210409 GN=CUD1\_1 PE=4 SV=1 |
| 10 | 53393 | tr|A0A5B7E6X8|A0A5B7E6X8\_PORTR | 88.49 | 6 | 4 | 1 | N | 52804 | Cuticle protein AM1199 OS=Portunus trituberculatus OX=210409 GN=CUPA3\_11 PE=4 SV=1 |
| 11 | 53428 | tr|A0A5B7E6A3|A0A5B7E6A3\_PORTR | 83.17 | 11 | 4 | 1 | N | 16545 | Larval cuticle protein LCP-17 OS=Portunus trituberculatus OX=210409 GN=LCP17\_3 PE=4 SV=1 |
| 4 | 53308 | tr|A0A5B7IZE3|A0A5B7IZE3\_PORTR | 81.55 | 16 | 6 | 4 | N | 26459 | Myosin heavy chain, muscle OS=Portunus trituberculatus OX=210409 GN=Mhc\_24 PE=3 SV=1 |
| 8 | 53314 | tr|F8WR03|F8WR03\_PENJP | 80.39 | 2 | 4 | 0 | N | 219503 | Myosin heavy chain type a OS=Penaeus japonicus OX=27405 GN=MYHa PE=2 SV=1 |
| 8 | 53315 | tr|K4Q111|K4Q111\_PENVA | 80.39 | 2 | 4 | 0 | N | 219712 | Myosin heavy chain type 1 OS=Penaeus vannamei OX=6689 GN=MYH1 PE=2 SV=1 |
| 8 | 53316 | tr|K4Q4N8|K4Q4N8\_PENMO | 80.39 | 2 | 4 | 0 | N | 219585 | Myosin heavy chain type 1 OS=Penaeus monodon OX=6687 GN=MYH1 PE=2 SV=1 |
| 12 | 53411 | tr|A0A2P1JJ59|A0A2P1JJ59\_PROCL | 75.46 | 3 | 4 | 4 | N | 97467 | Alpha-1,4 glucan phosphorylase (Fragment) OS=Procambarus clarkii OX=6728 GN=Glyp PE=2 SV=1 |
| 7 | 53302 | tr|A0A5B7DQZ0|A0A5B7DQZ0\_PORTR | 74.33 | 2 | 5 | 2 | N | 352200 | Myosin heavy chain, muscle OS=Portunus trituberculatus OX=210409 GN=Mhc\_1 PE=3 SV=1 |
| 6 | 53363 | tr|A0A223G1B9|A0A223G1B9\_SCYSE | 71.99 | 4 | 5 | 5 | N | 79160 | Cryptocyanin OS=Scylla serrata OX=6761 PE=2 SV=1 |
| 14 | 53398 | tr|A0A7R8CX21|A0A7R8CX21\_LEPSM | 70.54 | 1 | 3 | 0 | N | 202190 | MYH6\_7 OS=Lepeophtheirus salmonis OX=72036 GN=LSAA\_10697 PE=3 SV=1 |
| 14 | 53399 | tr|A0A7T8HL88|A0A7T8HL88\_CALRO | 70.54 | 1 | 3 | 0 | N | 215000 | Myosin heavy chain 1 OS=Caligus rogercresseyi OX=217165 GN=FKW44\_003971 PE=3 SV=1 |
| 14 | 53400 | tr|A0A7T8HL29|A0A7T8HL29\_CALRO | 70.54 | 1 | 3 | 0 | N | 214908 | Myosin heavy chain 1 OS=Caligus rogercresseyi OX=217165 GN=FKW44\_003975 PE=3 SV=1 |
| 14 | 53401 | tr|A0A0K2V7Y7|A0A0K2V7Y7\_LEPSM | 70.54 | 1 | 3 | 0 | N | 218544 | Myosin heavy chain 1 [Tribolium castaneum] OS=Lepeophtheirus salmonis OX=72036 GN=Mhc1 PE=3 SV=1 |
| 14 | 53402 | tr|A0A7R8GZZ9|A0A7R8GZZ9\_LEPSM | 70.54 | 1 | 3 | 0 | N | 220303 | MYH6\_7 OS=Lepeophtheirus salmonis OX=72036 GN=LSAA\_1562 PE=3 SV=1 |
| 14 | 53403 | tr|A0A0K2T3U6|A0A0K2T3U6\_LEPSM | 70.54 | 1 | 3 | 0 | N | 220566 | Myosin heavy chain 1 [Tribolium castaneum] OS=Lepeophtheirus salmonis OX=72036 GN=Mhc1 PE=3 SV=1 |
| 14 | 53404 | tr|A0A0K2TSJ7|A0A0K2TSJ7\_LEPSM | 70.54 | 1 | 3 | 0 | N | 222210 | Myosin heavy chain 1 [Tribolium castaneum] (Fragment) OS=Lepeophtheirus salmonis OX=72036 GN=Mhc1 PE=3 SV=1 |
| 3 | 53320 | tr|A0A4Y7MKW9|A0A4Y7MKW9\_9CRUS | 67.38 | 5 | 6 | 0 | N | 97229 | P-type Ca(2+) transporter (Fragment) OS=Daphnia magna OX=35525 GN=EOG090X00V6 PE=2 SV=1 |
| 3 | 53321 | tr|A0A0P5KSW7|A0A0P5KSW7\_9CRUS | 67.38 | 4 | 6 | 0 | N | 110371 | Calcium-transporting ATPase OS=Daphnia magna OX=35525 PE=3 SV=1 |
| 3 | 53322 | tr|A0A4Y7MXF9|A0A4Y7MXF9\_9CRUS | 67.38 | 4 | 6 | 0 | N | 110591 | Calcium-transporting ATPase OS=Daphnia pulicaria OX=35523 GN=EOG090X00V6 PE=2 SV=1 |
| 3 | 53323 | tr|A0A0P4ZGF4|A0A0P4ZGF4\_9CRUS | 67.38 | 4 | 6 | 0 | N | 110708 | Calcium-transporting ATPase OS=Daphnia magna OX=35525 GN=APZ42\_012354 PE=3 SV=1 |
| 3 | 53324 | tr|A0A4Y7MVM2|A0A4Y7MVM2\_DAPPU | 67.38 | 4 | 6 | 0 | N | 110862 | Calcium-transporting ATPase OS=Daphnia pulex OX=6669 GN=EOG090X00V6 PE=2 SV=1 |
| 3 | 53325 | tr|A0A4Y7M702|A0A4Y7M702\_9CRUS | 67.38 | 4 | 6 | 0 | N | 111071 | Calcium-transporting ATPase OS=Daphnia hispanica OX=575233 GN=EOG090X00V6 PE=2 SV=1 |
| 3 | 53326 | tr|A0A4Y7MHQ5|A0A4Y7MHQ5\_9CRUS | 67.38 | 4 | 6 | 0 | N | 111377 | Calcium-transporting ATPase OS=Daphnia magna OX=35525 GN=EOG090X00V6 PE=2 SV=1 |
| 3 | 53327 | tr|A0A4Y7ME47|A0A4Y7ME47\_9CRUS | 67.38 | 4 | 6 | 0 | N | 111392 | Calcium-transporting ATPase OS=Daphnia magna OX=35525 GN=EOG090X00V6 PE=2 SV=1 |
| 3 | 53328 | tr|A0A4Y7M4I0|A0A4Y7M4I0\_9CRUS | 67.38 | 4 | 6 | 0 | N | 111723 | Calcium-transporting ATPase OS=Daphnia dolichocephala OX=2282166 GN=EOG090X00V6 PE=2 SV=1 |
| 3 | 53329 | tr|A0A1D2MC18|A0A1D2MC18\_ORCCI | 67.38 | 4 | 6 | 0 | N | 112226 | Calcium-transporting ATPase OS=Orchesella cincta OX=48709 GN=Ocin01\_16136 PE=3 SV=1 |
| 3 | 53330 | tr|E9HR84|E9HR84\_DAPPU | 67.38 | 4 | 6 | 0 | N | 113043 | Calcium-transporting ATPase OS=Daphnia pulex OX=6669 GN=DAPPUDRAFT\_65262 PE=3 SV=1 |
| 3 | 53331 | tr|A0A0P5VJX3|A0A0P5VJX3\_9CRUS | 67.38 | 4 | 6 | 0 | N | 113098 | Calcium-transporting ATPase OS=Daphnia magna OX=35525 PE=3 SV=1 |
| 3 | 53332 | tr|A0A4Y7LYL4|A0A4Y7LYL4\_9CRUS | 67.38 | 4 | 6 | 0 | N | 113136 | Calcium-transporting ATPase OS=Daphnia atkinsoni OX=342845 GN=EOG090X00V6 PE=2 SV=1 |
| 5 | 53318 | tr|A0A0C5E1J4|A0A0C5E1J4\_CHEDE | 65.79 | 4 | 5 | 0 | N | 109565 | Calcium-transporting ATPase OS=Cherax destructor OX=6723 PE=2 SV=1 |
| 9 | 53378 | tr|A0A0N9EJJ5|A0A0N9EJJ5\_ERIVE | 61.23 | 4 | 4 | 4 | N | 77227 | Hemocyanin OS=Eriphia verrucosa OX=483417 GN=H PE=2 SV=1 |
| 2 | 53418 | tr|A0A2Z4I2U3|A0A2Z4I2U3\_CALSI | 58.61 | 4 | 7 | 1 | N | 111733 | Calcium-transporting ATPase OS=Callinectes sapidus OX=6763 GN=SERCA PE=2 SV=1 |
| 16 | 53419 | tr|A0A0P4W579|A0A0P4W579\_SCYOL | 58.49 | 4 | 2 | 2 | N | 54554 | Uncharacterized protein OS=Scylla olivacea OX=85551 PE=4 SV=1 |
| 16 | 53420 | tr|A0A0P4WKI4|A0A0P4WKI4\_SCYOL | 58.49 | 3 | 2 | 2 | N | 78230 | Uncharacterized protein OS=Scylla olivacea OX=85551 PE=4 SV=1 |
| 16 | 53421 | tr|A0A0P4WBU2|A0A0P4WBU2\_SCYOL | 58.49 | 3 | 2 | 2 | N | 94430 | Uncharacterized protein OS=Scylla olivacea OX=85551 PE=4 SV=1 |
| 17 | 53427 | tr|A0A3R7PTG0|A0A3R7PTG0\_PENVA | 49.31 | 8 | 2 | 2 | N | 18720 | Myosin light chain 2 OS=Penaeus vannamei OX=6689 GN=C7M84\_004824 PE=4 SV=1 |
| 15 | 53394 | tr|A0A195D1T6|A0A195D1T6\_9HYME | 47.57 | 0 | 2 | 2 | N | 1073055 | Titin OS=Cyphomyrmex costatus OX=456900 GN=ALC62\_02215 PE=3 SV=1 |
| 15 | 53396 | tr|A0A151I5T2|A0A151I5T2\_9HYME | 47.57 | 0 | 2 | 2 | N | 1059815 | Titin OS=Atta colombica OX=520822 GN=ALC53\_03162 PE=3 SV=1 |
| 15 | 53397 | tr|A0A151J500|A0A151J500\_9HYME | 47.57 | 0 | 2 | 2 | N | 1075189 | Titin OS=Trachymyrmex cornetzi OX=471704 GN=ALC57\_09858 PE=3 SV=1 |
| 15 | 53469 | tr|E2BUG0|E2BUG0\_HARSA | 47.57 | 0 | 2 | 2 | N | 660642 | Titin OS=Harpegnathos saltator OX=610380 GN=EAI\_13877 PE=3 SV=1 |
| 13 | 53379 | tr|A0A5N5SKQ0|A0A5N5SKQ0\_9CRUS | 45.97 | 3 | 3 | 3 | N | 101132 | Filamin-A OS=Armadillidium nasatum OX=96803 GN=cher PE=3 SV=1 |
| 13 | 53380 | tr|A0A444SHR5|A0A444SHR5\_ARMVU | 45.97 | 3 | 3 | 3 | N | 88595 | Filamin-A (Fragment) OS=Armadillidium vulgare OX=13347 GN=Avbf\_11273 PE=3 SV=1 |
| 13 | 53381 | tr|A0A3R7Q393|A0A3R7Q393\_PENVA | 45.97 | 3 | 3 | 3 | N | 99699 | Putative filamin-A isoform X4 OS=Penaeus vannamei OX=6689 GN=C7M84\_021889 PE=3 SV=1 |
| 13 | 53382 | tr|A0A2P1JJ42|A0A2P1JJ42\_PROCL | 45.97 | 2 | 3 | 3 | N | 167860 | Filamin-A isoform X5 (Fragment) OS=Procambarus clarkii OX=6728 GN=Flna PE=2 SV=1 |
| 42 | 53503 | tr|A0A834J309|A0A834J309\_RHYFE | 39.43 | 0 | 1 | 1 | N | 981760 | Uncharacterized protein OS=Rhynchophorus ferrugineus OX=354439 GN=GWI33\_002833 PE=4 SV=1 |
| 18 | 53529 | tr|A0A6M2D681|A0A6M2D681\_RHIMP | 31.83 | 11 | 1 | 1 | N | 13868 | Putative conserved secreted protein OS=Rhipicephalus microplus OX=6941 PE=4 SV=1 |
| 18 | 53530 | tr|A0A6G5A756|A0A6G5A756\_RHIMP | 31.83 | 11 | 1 | 1 | N | 13838 | Putative conserved secreted protein OS=Rhipicephalus microplus OX=6941 PE=4 SV=1 |
| 43 | 53532 | tr|A0A2J7R8E8|A0A2J7R8E8\_9NEOP | 28.61 | 1 | 1 | 1 | N | 110847 | Uncharacterized protein OS=Cryptotermes secundus OX=105785 GN=B7P43\_G07431 PE=4 SV=1 |
| 44 | 53533 | tr|A0A423SLW8|A0A423SLW8\_PENVA | 28.36 | 4 | 1 | 1 | N | 24833 | Malate dehydrogenase OS=Penaeus vannamei OX=6689 GN=C7M84\_016800 PE=3 SV=1 |
| 44 | 53534 | tr|A0A5B7CL07|A0A5B7CL07\_PORTR | 28.36 | 4 | 1 | 1 | N | 30579 | Malate dehydrogenase OS=Portunus trituberculatus OX=210409 GN=MDH1 PE=3 SV=1 |
| 44 | 53535 | tr|A0A0P4WLG3|A0A0P4WLG3\_SCYOL | 28.36 | 3 | 1 | 1 | N | 35829 | Malate dehydrogenase OS=Scylla olivacea OX=85551 PE=3 SV=1 |
| 45 | 53537 | tr|A0A1W4VNV5|A0A1W4VNV5\_DROFC | 26.84 | 1 | 1 | 1 | N | 111050 | prominin-like protein isoform X4 OS=Drosophila ficusphila OX=30025 GN=LOC108099069 PE=3 SV=1 |
| 45 | 53538 | tr|A0A1W4W0Y9|A0A1W4W0Y9\_DROFC | 26.84 | 1 | 1 | 1 | N | 113905 | prominin-like protein isoform X3 OS=Drosophila ficusphila OX=30025 GN=LOC108099069 PE=3 SV=1 |
| 45 | 53539 | tr|A0A1W4W054|A0A1W4W054\_DROFC | 26.84 | 1 | 1 | 1 | N | 116227 | prominin-like protein isoform X2 OS=Drosophila ficusphila OX=30025 GN=LOC108099069 PE=3 SV=1 |
| 45 | 53540 | tr|A0A1W4VNL1|A0A1W4VNL1\_DROFC | 26.84 | 1 | 1 | 1 | N | 116315 | prominin-like protein isoform X1 OS=Drosophila ficusphila OX=30025 GN=LOC108099069 PE=3 SV=1 |
| 46 | 53541 | tr|A0A146ME36|A0A146ME36\_LYGHE | 26.60 | 2 | 1 | 1 | N | 70072 | AP-1 complex subunit gamma-1 (Fragment) OS=Lygus hesperus OX=30085 GN=Ap1g1 PE=3 SV=1 |
| 46 | 53542 | tr|A0A0A9ZCU1|A0A0A9ZCU1\_LYGHE | 26.60 | 1 | 1 | 1 | N | 93178 | AP-1 complex subunit gamma OS=Lygus hesperus OX=30085 GN=Ap1g1\_2 PE=3 SV=1 |
| 46 | 53543 | tr|A0A0A9Z559|A0A0A9Z559\_LYGHE | 26.60 | 1 | 1 | 1 | N | 94621 | AP-1 complex subunit gamma OS=Lygus hesperus OX=30085 GN=Ap1g1\_0 PE=3 SV=1 |
| 47 | 53551 | tr|A0A553NSJ7|A0A553NSJ7\_TIGCA | 26.07 | 5 | 1 | 1 | N | 19175 | H15 domain-containing protein OS=Tigriopus californicus OX=6832 GN=TCAL\_13520 PE=3 SV=1 |
| 47 | 53552 | tr|A0A553NV74|A0A553NV74\_TIGCA | 26.07 | 5 | 1 | 1 | N | 19189 | H15 domain-containing protein OS=Tigriopus californicus OX=6832 GN=TCAL\_17452 PE=3 SV=1 |
| 48 | 53553 | tr|B4L6N9|B4L6N9\_DROMO | 25.98 | 1 | 1 | 1 | N | 99753 | Uncharacterized protein, isoform A OS=Drosophila mojavensis OX=7230 GN=Dmoj\GI16404 PE=4 SV=2 |
| 48 | 53554 | tr|A0A484AUC9|A0A484AUC9\_DRONA | 25.98 | 1 | 1 | 1 | N | 124075 | Uncharacterized protein OS=Drosophila navojoa OX=7232 GN=AWZ03\_014422 PE=4 SV=1 |
| 61 | 53716 | tr|A0A182HNK7|A0A182HNK7\_ANOAR | 24.12 | 0 | 1 | 1 | N | 239313 | RTTN\_N domain-containing protein OS=Anopheles arabiensis OX=7173 PE=4 SV=1 |
| 24 | 53595 | tr|A0A1B0CTB8|A0A1B0CTB8\_LUTLO | 23.80 | 0 | 1 | 1 | N | 466597 | Uncharacterized protein OS=Lutzomyia longipalpis OX=7200 PE=3 SV=1 |
| 24 | 53604 | tr|E9IDT0|E9IDT0\_SOLIN | 23.80 | 1 | 1 | 1 | N | 162100 | Uncharacterized protein (Fragment) OS=Solenopsis invicta OX=13686 GN=SINV\_15254 PE=4 SV=1 |
| 24 | 53606 | tr|A0A6V7J2X2|A0A6V7J2X2\_9HYME | 23.80 | 13 | 1 | 1 | N | 7026 | eRF1\_3 domain-containing protein (Fragment) OS=Bracon brevicornis OX=1563983 GN=BBRV\_LOCUS36952 PE=4 SV=1 |
| 24 | 53607 | tr|A0A7R8V5M9|A0A7R8V5M9\_HERIL | 23.80 | 3 | 1 | 1 | N | 32248 | N-acetylglucosaminylphosphatidylinositol deacetylase OS=Hermetia illucens OX=343691 GN=HERILL\_LOCUS15608 PE=3 SV=1 |
| 24 | 53608 | tr|A0A0K8TNK6|A0A0K8TNK6\_TABBR | 23.80 | 3 | 1 | 1 | N | 33165 | N-acetylglucosaminylphosphatidylinositol deacetylase (Fragment) OS=Tabanus bromius OX=304241 PE=2 SV=1 |
| 24 | 53609 | tr|B4NGC5|B4NGC5\_DROWI | 23.80 | 2 | 1 | 1 | N | 40030 | N-acetylglucosaminylphosphatidylinositol deacetylase OS=Drosophila willistoni OX=7260 GN=Dwil\GK22798 PE=3 SV=1 |
| 24 | 53610 | tr|B4M4U0|B4M4U0\_DROVI | 23.80 | 2 | 1 | 1 | N | 39814 | N-acetylglucosaminylphosphatidylinositol deacetylase OS=Drosophila virilis OX=7244 GN=Dvir\GJ10171 PE=3 SV=1 |
| 24 | 53611 | tr|A0A484BDS3|A0A484BDS3\_DRONA | 23.80 | 2 | 1 | 1 | N | 40350 | N-acetylglucosaminylphosphatidylinositol deacetylase OS=Drosophila navojoa OX=7232 GN=AWZ03\_006647 PE=3 SV=1 |
| 24 | 53612 | tr|A0A6J1M8J1|A0A6J1M8J1\_DROHY | 23.80 | 2 | 1 | 1 | N | 40571 | N-acetylglucosaminylphosphatidylinositol deacetylase OS=Drosophila hydei OX=7224 GN=LOC111602076 PE=3 SV=1 |
| 24 | 53613 | tr|B4KE34|B4KE34\_DROMO | 23.80 | 2 | 1 | 1 | N | 40618 | N-acetylglucosaminylphosphatidylinositol deacetylase OS=Drosophila mojavensis OX=7230 GN=Dmoj\GI10317 PE=3 SV=1 |
| 24 | 53614 | tr|A0A0M4EG71|A0A0M4EG71\_DROBS | 23.80 | 2 | 1 | 1 | N | 39846 | N-acetylglucosaminylphosphatidylinositol deacetylase OS=Drosophila busckii OX=30019 GN=Dbus\_chr3Rg2066 PE=3 SV=1 |
| 24 | 53615 | tr|A0A6P8XFN0|A0A6P8XFN0\_DROAB | 23.80 | 2 | 1 | 1 | N | 40056 | N-acetylglucosaminylphosphatidylinositol deacetylase OS=Drosophila albomicans OX=7291 GN=LOC117575192 PE=3 SV=1 |
| 24 | 53616 | tr|A0A6J2TAH7|A0A6J2TAH7\_DROLE | 23.80 | 2 | 1 | 1 | N | 41626 | N-acetylglucosaminylphosphatidylinositol deacetylase OS=Drosophila lebanonensis OX=7225 GN=LOC115623577 PE=3 SV=1 |
| 24 | 53617 | tr|A0A6J2TEK1|A0A6J2TEK1\_DROLE | 23.80 | 2 | 1 | 1 | N | 42257 | N-acetylglucosaminylphosphatidylinositol deacetylase OS=Drosophila lebanonensis OX=7225 GN=LOC115623577 PE=3 SV=1 |
| 24 | 53618 | tr|A0A7I0ZIP4|A0A7I0ZIP4\_DANPL | 23.80 | 1 | 1 | 1 | N | 62756 | atlastin isoform X2 OS=Danaus plexippus plexippus OX=278856 GN=LOC116772859 PE=3 SV=1 |
| 24 | 53619 | tr|A0A7I0ZK43|A0A7I0ZK43\_DANPL | 23.80 | 1 | 1 | 1 | N | 62815 | atlastin isoform X1 OS=Danaus plexippus plexippus OX=278856 GN=LOC116772859 PE=3 SV=1 |
| 24 | 53620 | tr|A0A7M6UVA6|A0A7M6UVA6\_NASVI | 23.80 | 1 | 1 | 1 | N | 82594 | Vesicle-fusing ATPase OS=Nasonia vitripennis OX=7425 PE=3 SV=1 |
| 24 | 53621 | tr|A0A7M7QVM7|A0A7M7QVM7\_NASVI | 23.80 | 1 | 1 | 1 | N | 82560 | Vesicle-fusing ATPase OS=Nasonia vitripennis OX=7425 PE=3 SV=1 |
| 24 | 53622 | tr|A0A232F624|A0A232F624\_9HYME | 23.80 | 1 | 1 | 1 | N | 83056 | Vesicle-fusing ATPase (Fragment) OS=Trichomalopsis sarcophagae OX=543379 GN=TSAR\_012849 PE=3 SV=1 |
| 51 | 53645 | tr|F4WT59|F4WT59\_ACREC | 22.27 | 1 | 1 | 1 | N | 143072 | Probable ATP-dependent RNA helicase spindle-E OS=Acromyrmex echinatior OX=103372 GN=G5I\_09034 PE=3 SV=1 |
| 51 | 53646 | tr|A0A836FB18|A0A836FB18\_9HYME | 22.27 | 1 | 1 | 1 | N | 154532 | SPNE helicase (Fragment) OS=Acromyrmex heyeri OX=230685 GN=Spne\_1 PE=4 SV=1 |
| 51 | 53647 | tr|A0A0J7L582|A0A0J7L582\_LASNI | 22.27 | 1 | 1 | 1 | N | 156393 | Probable ATP-dependent RNA helicase spindle-E OS=Lasius niger OX=67767 GN=RF55\_1956 PE=3 SV=1 |
| 51 | 53648 | tr|A0A151J597|A0A151J597\_9HYME | 22.27 | 1 | 1 | 1 | N | 160408 | Probable ATP-dependent RNA helicase spindle-E OS=Trachymyrmex cornetzi OX=471704 GN=ALC57\_09730 PE=3 SV=1 |
| 51 | 53649 | tr|A0A836JUW8|A0A836JUW8\_9HYME | 22.27 | 1 | 1 | 1 | N | 161165 | SPNE helicase (Fragment) OS=Pseudoatta argentina OX=621737 GN=Spne\_1 PE=4 SV=1 |
| 51 | 53650 | tr|A0A836JG75|A0A836JG75\_9HYME | 22.27 | 1 | 1 | 1 | N | 162126 | SPNE helicase (Fragment) OS=Acromyrmex insinuator OX=230686 GN=Spne\_0 PE=4 SV=1 |
| 25 | 53623 | tr|A0A1L3IWM3|A0A1L3IWM3\_RHOPD | 20.57 | 1 | 1 | 1 | N | 77923 | ABC transporter G family member 23 variant X1 OS=Rhopalosiphum padi OX=40932 GN=ABCG23-1 PE=2 SV=1 |
| 25 | 53656 | tr|A0A2S2QP63|A0A2S2QP63\_9HEMI | 20.57 | 3 | 1 | 1 | N | 27832 | ABC transporter G family member 20 OS=Sipha flava OX=143950 GN=abcG20\_2 PE=4 SV=1 |
| 25 | 53688 | tr|A0A2S2NYH2|A0A2S2NYH2\_SCHGA | 20.57 | 3 | 1 | 1 | N | 31666 | ABC transporter G family member 20 OS=Schizaphis graminum OX=13262 GN=abcG20\_6 PE=4 SV=1 |
| 25 | 53689 | tr|A0A6G0YXF5|A0A6G0YXF5\_APHCR | 20.57 | 1 | 1 | 1 | N | 71048 | ABC transporter G family member 23-like OS=Aphis craccivora OX=307492 GN=FWK35\_00011512 PE=4 SV=1 |
| 25 | 53690 | tr|A0A6G0UB83|A0A6G0UB83\_APHGL | 20.57 | 1 | 1 | 1 | N | 75345 | ABC transporter domain-containing protein OS=Aphis glycines OX=307491 GN=AGLY\_001125 PE=4 SV=1 |
| 53 | 53655 | tr|A0A182VZF1|A0A182VZF1\_9DIPT | 20.54 | 6 | 1 | 1 | N | 21968 | ZAD domain-containing protein OS=Anopheles minimus OX=112268 PE=4 SV=1 |
| 54 | 53657 | tr|A0A023ERZ2|A0A023ERZ2\_AEDAL | 20.51 | 3 | 1 | 1 | N | 45728 | Pyruvate kinase (Fragment) OS=Aedes albopictus OX=7160 PE=2 SV=1 |
| 54 | 53658 | tr|A0A0P8Y097|A0A0P8Y097\_DROAN | 20.51 | 2 | 1 | 1 | N | 55184 | Pyruvate kinase OS=Drosophila ananassae OX=7217 GN=Dana\GF18439 PE=3 SV=1 |
| 54 | 53659 | tr|A0A6P8K875|A0A6P8K875\_DROMA | 20.51 | 2 | 1 | 1 | N | 55073 | Pyruvate kinase OS=Drosophila mauritiana OX=7226 GN=LOC117143834 PE=3 SV=1 |
| 54 | 53660 | tr|A0A6P4F4P4|A0A6P4F4P4\_DRORH | 20.51 | 2 | 1 | 1 | N | 55028 | Pyruvate kinase OS=Drosophila rhopaloa OX=1041015 GN=LOC108048043 PE=3 SV=1 |
| 54 | 53661 | tr|A0A1Y9GJY2|A0A1Y9GJY2\_ANOAR | 20.51 | 2 | 1 | 1 | N | 55698 | Pyruvate kinase OS=Anopheles arabiensis OX=7173 PE=3 SV=1 |
| 54 | 53662 | tr|A0A0Q9X1L7|A0A0Q9X1L7\_DROMO | 20.51 | 2 | 1 | 1 | N | 55236 | Pyruvate kinase OS=Drosophila mojavensis OX=7230 GN=Dmoj\GI22374 PE=3 SV=1 |
| 54 | 53663 | tr|A0A2M3ZFB9|A0A2M3ZFB9\_9DIPT | 20.51 | 2 | 1 | 1 | N | 56104 | Pyruvate kinase (Fragment) OS=Anopheles braziliensis OX=58242 PE=3 SV=1 |
| 54 | 53664 | tr|A0A2M3ZYS6|A0A2M3ZYS6\_9DIPT | 20.51 | 2 | 1 | 1 | N | 56023 | Pyruvate kinase (Fragment) OS=Anopheles triannulatus OX=58253 PE=3 SV=1 |
| 54 | 53665 | tr|A0A2M3ZYS8|A0A2M3ZYS8\_9DIPT | 20.51 | 2 | 1 | 1 | N | 56022 | Pyruvate kinase (Fragment) OS=Anopheles triannulatus OX=58253 PE=3 SV=1 |
| 54 | 53666 | tr|A0A1Y9GJK0|A0A1Y9GJK0\_ANOAR | 20.51 | 2 | 1 | 1 | N | 56271 | Pyruvate kinase OS=Anopheles arabiensis OX=7173 PE=3 SV=1 |
| 54 | 53667 | tr|A0A6A0H7S7|A0A6A0H7S7\_HYAAZ | 20.51 | 2 | 1 | 1 | N | 56703 | Pyruvate kinase OS=Hyalella azteca OX=294128 GN=HAZT\_HAZT006469 PE=3 SV=1 |
| 54 | 53668 | tr|A0A1E1X8N4|A0A1E1X8N4\_9ACAR | 20.51 | 2 | 1 | 1 | N | 57708 | Pyruvate kinase OS=Amblyomma aureolatum OX=187763 PE=2 SV=1 |
| 54 | 53669 | tr|A0A6P4F9I4|A0A6P4F9I4\_DRORH | 20.51 | 2 | 1 | 1 | N | 57050 | Pyruvate kinase OS=Drosophila rhopaloa OX=1041015 GN=LOC108048043 PE=3 SV=1 |
| 54 | 53670 | tr|A0A182VPN6|A0A182VPN6\_9DIPT | 20.51 | 2 | 1 | 1 | N | 57977 | Pyruvate kinase OS=Anopheles minimus OX=112268 PE=3 SV=1 |
| 54 | 53671 | tr|A0A182MNQ6|A0A182MNQ6\_9DIPT | 20.51 | 2 | 1 | 1 | N | 57968 | Pyruvate kinase OS=Anopheles culicifacies OX=139723 PE=3 SV=1 |
| 54 | 53672 | tr|A0A6J2T5C5|A0A6J2T5C5\_DROLE | 20.51 | 2 | 1 | 1 | N | 57328 | Pyruvate kinase OS=Drosophila lebanonensis OX=7225 GN=LOC115620839 PE=3 SV=1 |
| 54 | 53673 | tr|A0A1W4W378|A0A1W4W378\_DROFC | 20.51 | 2 | 1 | 1 | N | 57395 | Pyruvate kinase OS=Drosophila ficusphila OX=30025 GN=LOC108099555 PE=3 SV=1 |
| 54 | 53674 | O62619|KPYK\_DROME | 20.51 | 2 | 1 | 1 | N | 57440 | Pyruvate kinase OS=Drosophila melanogaster OX=7227 GN=PyK PE=2 SV=2 |
| 54 | 53675 | tr|B4HE69|B4HE69\_DROSE | 20.51 | 2 | 1 | 1 | N | 57454 | Pyruvate kinase OS=Drosophila sechellia OX=7238 GN=Dsec\GM26420 PE=3 SV=1 |
| 54 | 53676 | tr|A0A6P4F0Q9|A0A6P4F0Q9\_DRORH | 20.51 | 2 | 1 | 1 | N | 57379 | Pyruvate kinase OS=Drosophila rhopaloa OX=1041015 GN=LOC108048043 PE=3 SV=1 |
| 54 | 53677 | tr|B3P7K4|B3P7K4\_DROER | 20.51 | 2 | 1 | 1 | N | 57539 | Pyruvate kinase OS=Drosophila erecta OX=7220 GN=Dere\GG11123 PE=3 SV=1 |
| 54 | 53678 | tr|B3M213|B3M213\_DROAN | 20.51 | 2 | 1 | 1 | N | 57565 | Pyruvate kinase OS=Drosophila ananassae OX=7217 GN=Dana\GF18439 PE=3 SV=2 |
| 54 | 53679 | tr|A0A6P8KMB4|A0A6P8KMB4\_DROMA | 20.51 | 2 | 1 | 1 | N | 57454 | Pyruvate kinase OS=Drosophila mauritiana OX=7226 GN=LOC117143834 PE=3 SV=1 |
| 54 | 53680 | tr|B4K5E3|B4K5E3\_DROMO | 20.51 | 2 | 1 | 1 | N | 57618 | Pyruvate kinase OS=Drosophila mojavensis OX=7230 GN=Dmoj\GI22374 PE=3 SV=1 |
| 54 | 53681 | tr|A0A484BWG4|A0A484BWG4\_DRONA | 20.51 | 2 | 1 | 1 | N | 57563 | Pyruvate kinase OS=Drosophila navojoa OX=7232 GN=AWZ03\_001260 PE=3 SV=1 |
| 54 | 53682 | tr|A0A131XQM7|A0A131XQM7\_9ACAR | 20.51 | 2 | 1 | 1 | N | 59290 | Pyruvate kinase OS=Hyalomma excavatum OX=257692 PE=2 SV=1 |
| 54 | 53683 | tr|A0A0P9ARZ1|A0A0P9ARZ1\_DROAN | 20.51 | 2 | 1 | 1 | N | 59439 | Pyruvate kinase OS=Drosophila ananassae OX=7217 GN=Dana\GF18439 PE=3 SV=1 |
| 54 | 53684 | tr|G3MLC1|G3MLC1\_AMBMU | 20.51 | 2 | 1 | 1 | N | 61369 | Pyruvate kinase OS=Amblyomma maculatum OX=34609 PE=2 SV=1 |
| 54 | 53685 | tr|A0A023EVC5|A0A023EVC5\_AEDAL | 20.51 | 2 | 1 | 1 | N | 63958 | Pyruvate kinase (Fragment) OS=Aedes albopictus OX=7160 PE=2 SV=1 |
| 54 | 53686 | tr|B6RHH5|B6RHH5\_PENVA | 20.51 | 2 | 1 | 1 | N | 63782 | Pyruvate kinase OS=Penaeus vannamei OX=6689 PE=2 SV=1 |
| 54 | 53687 | tr|A0A5N5T493|A0A5N5T493\_9CRUS | 20.51 | 2 | 1 | 1 | N | 68944 | Pyruvate kinase OS=Armadillidium nasatum OX=96803 GN=PyK PE=3 SV=1 |
| 21 | 53624 | tr|A0A6P4EYV9|A0A6P4EYV9\_DRORH | 20.46 | 0 | 1 | 1 | N | 2391440 | LOW QUALITY PROTEIN: uncharacterized protein LOC108047588 OS=Drosophila rhopaloa OX=1041015 GN=LOC108047588 PE=3 SV=1 |
| total 142 proteins |
| --- |

  

tr|A0A3R7M007|A0A3R7M007\_PENVA

back to list

  

| Protein Coverage
| Supporting Peptides
|

Protein Coverage:

Supporting Peptides:

| Peptide | Uniq | -10lgP | Mass | ppm | m/z | z | RT | Scan | #Spec | Start | End | PTM |
| --- | --- | --- | --- | --- | --- | --- | --- | --- | --- | --- | --- | --- |
| E.EYDESGPGIVH.R | Y | 40.93 | 1201.5251 | 1.2 | 601.7706 | 2 | 10.52 | 1080 | 1 | 358 | 368 |  |
| E.KSYELPDGQVITIG.N | Y | 38.20 | 1518.7930 | -0.7 | 760.4032 | 2 | 13.25 | 1584 | 1 | 235 | 248 |  |
| T.MYPGIADRM.Q | Y | 32.95 | 1052.4783 | 0.0 | 527.2464 | 2 | 11.68 | 1301 | 1 | 302 | 310 |  |
| L.DTGDGVTH.T | Y | 30.28 | 800.3301 | -0.3 | 401.1722 | 2 | 7.91 | 634 | 3 | 151 | 158 |  |
| K.IIAPPERKYS.V | Y | 30.21 | 1172.6553 | 2.8 | 587.3365 | 2 | 10.01 | 984 | 1 | 326 | 335 |  |
| A.GFAGDDAPR.A | Y | 28.64 | 904.4039 | 0.1 | 453.2093 | 2 | 9.83 | 953 | 1 | 17 | 25 |  |
| G.FAGDDAPRA.V | Y | 26.63 | 918.4195 | -0.5 | 460.2168 | 2 | 9.87 | 959 | 1 | 18 | 26 |  |
| K.IIAPPERKY.S | Y | 25.38 | 1085.6233 | 1.4 | 543.8197 | 2 | 10.17 | 1014 | 1 | 326 | 334 |  |
| E.KSYELPDGQVITIGNER.F | Y | 24.35 | 1917.9795 | 1.3 | 640.3346 | 3 | 12.57 | 1461 | 1 | 235 | 251 |  |
| K.SYELPDGQ.V | Y | 22.58 | 907.3923 | 0.0 | 454.7034 | 2 | 11.17 | 1205 | 1 | 236 | 243 |  |
| Y.DESGPGIVH.R | Y | 21.74 | 909.4192 | -0.6 | 455.7166 | 2 | 10.03 | 989 | 1 | 360 | 368 |  |
| T.NWDDMEKIWHH.S | Y | 20.98 | 1509.6459 | -0.3 | 755.8300 | 2 | 12.54 | 1455 | 1 | 75 | 85 |  |
| E.KSYELPDGQVITI.G | Y | 20.38 | 1461.7715 | -0.5 | 731.8926 | 2 | 13.39 | 1607 | 1 | 235 | 247 |  |
| Y.VGDEAQSK.R | Y | 20.25 | 832.3926 | 0.0 | 417.2036 | 2 | 7.67 | 599 | 2 | 51 | 58 |  |
| L.GMESVGIH.E | Y | 20.20 | 828.3800 | 2.3 | 415.1982 | 2 | 10.51 | 1077 | 1 | 265 | 272 |  |
| E.KSYELPDGQVITIGNE.R | Y | 19.11 | 1761.8784 | 10.8 | 881.9560 | 2 | 13.18 | 1572 | 1 | 235 | 250 |  |
| L.TEAPLNPK.A | Y | 17.45 | 868.4654 | 0.1 | 435.2400 | 2 | 9.61 | 912 | 1 | 103 | 110 |  |
| T.LKYPIE.H | Y | 17.39 | 761.4323 | 1.0 | 381.7238 | 2 | 11.01 | 1175 | 1 | 64 | 69 |  |
| M.YPGIADRM.Q | Y | 16.87 | 921.4378 | -0.3 | 461.7260 | 2 | 11.25 | 1221 | 1 | 303 | 310 |  |
| L.RVAPEESPVLL.T | Y | 16.76 | 1208.6764 | 0.0 | 605.3455 | 2 | 12.50 | 1451 | 1 | 92 | 102 |  |
| E.KSYELPDGQ.V | Y | 15.82 | 1035.4873 | 2.6 | 518.7523 | 2 | 10.49 | 1073 | 1 | 235 | 243 |  |
| total 21 peptides |
| --- |

tr|A0A2H4V3D7|A0A2H4V3D7\_PENVA

back to list

  

| Protein Coverage
| Supporting Peptides
|

Protein Coverage:

Supporting Peptides:

| Peptide | Uniq | -10lgP | Mass | ppm | m/z | z | RT | Scan | #Spec | Start | End | PTM |
| --- | --- | --- | --- | --- | --- | --- | --- | --- | --- | --- | --- | --- |
| E.EYDESGPGIVH.R | Y | 40.93 | 1201.5251 | 1.2 | 601.7706 | 2 | 10.52 | 1080 | 1 | 362 | 372 |  |
| E.KSYELPDGQVITIG.N | Y | 38.20 | 1518.7930 | -0.7 | 760.4032 | 2 | 13.25 | 1584 | 1 | 239 | 252 |  |
| T.MYPGIADRM.Q | Y | 32.95 | 1052.4783 | 0.0 | 527.2464 | 2 | 11.68 | 1301 | 1 | 306 | 314 |  |
| L.DTGDGVTH.T | Y | 30.28 | 800.3301 | -0.3 | 401.1722 | 2 | 7.91 | 634 | 3 | 155 | 162 |  |
| K.IIAPPERKYS.V | Y | 30.21 | 1172.6553 | 2.8 | 587.3365 | 2 | 10.01 | 984 | 1 | 330 | 339 |  |
| A.GFAGDDAPR.A | Y | 28.64 | 904.4039 | 0.1 | 453.2093 | 2 | 9.83 | 953 | 1 | 21 | 29 |  |
| G.FAGDDAPRA.V | Y | 26.63 | 918.4195 | -0.5 | 460.2168 | 2 | 9.87 | 959 | 1 | 22 | 30 |  |
| K.IIAPPERKY.S | Y | 25.38 | 1085.6233 | 1.4 | 543.8197 | 2 | 10.17 | 1014 | 1 | 330 | 338 |  |
| E.KSYELPDGQVITIGNER.F | Y | 24.35 | 1917.9795 | 1.3 | 640.3346 | 3 | 12.57 | 1461 | 1 | 239 | 255 |  |
| K.SYELPDGQ.V | Y | 22.58 | 907.3923 | 0.0 | 454.7034 | 2 | 11.17 | 1205 | 1 | 240 | 247 |  |
| Y.DESGPGIVH.R | Y | 21.74 | 909.4192 | -0.6 | 455.7166 | 2 | 10.03 | 989 | 1 | 364 | 372 |  |
| T.NWDDMEKIWHH.S | Y | 20.98 | 1509.6459 | -0.3 | 755.8300 | 2 | 12.54 | 1455 | 1 | 79 | 89 |  |
| E.KSYELPDGQVITI.G | Y | 20.38 | 1461.7715 | -0.5 | 731.8926 | 2 | 13.39 | 1607 | 1 | 239 | 251 |  |
| Y.VGDEAQSK.R | Y | 20.25 | 832.3926 | 0.0 | 417.2036 | 2 | 7.67 | 599 | 2 | 55 | 62 |  |
| L.GMESVGIH.E | Y | 20.20 | 828.3800 | 2.3 | 415.1982 | 2 | 10.51 | 1077 | 1 | 269 | 276 |  |
| E.KSYELPDGQVITIGNE.R | Y | 19.11 | 1761.8784 | 10.8 | 881.9560 | 2 | 13.18 | 1572 | 1 | 239 | 254 |  |
| L.TEAPLNPK.A | Y | 17.45 | 868.4654 | 0.1 | 435.2400 | 2 | 9.61 | 912 | 1 | 107 | 114 |  |
| T.LKYPIE.H | Y | 17.39 | 761.4323 | 1.0 | 381.7238 | 2 | 11.01 | 1175 | 1 | 68 | 73 |  |
| M.YPGIADRM.Q | Y | 16.87 | 921.4378 | -0.3 | 461.7260 | 2 | 11.25 | 1221 | 1 | 307 | 314 |  |
| L.RVAPEESPVLL.T | Y | 16.76 | 1208.6764 | 0.0 | 605.3455 | 2 | 12.50 | 1451 | 1 | 96 | 106 |  |
| E.KSYELPDGQ.V | Y | 15.82 | 1035.4873 | 2.6 | 518.7523 | 2 | 10.49 | 1073 | 1 | 239 | 247 |  |
| total 21 peptides |
| --- |

tr|H9B3Y9|H9B3Y9\_SCYPA

back to list

  

| Protein Coverage
| Supporting Peptides
|

Protein Coverage:

Supporting Peptides:

| Peptide | Uniq | -10lgP | Mass | ppm | m/z | z | RT | Scan | #Spec | Start | End | PTM |
| --- | --- | --- | --- | --- | --- | --- | --- | --- | --- | --- | --- | --- |
| E.EYDESGPGIVH.R | Y | 40.93 | 1201.5251 | 1.2 | 601.7706 | 2 | 10.52 | 1080 | 1 | 362 | 372 |  |
| E.KSYELPDGQVITIG.N | Y | 38.20 | 1518.7930 | -0.7 | 760.4032 | 2 | 13.25 | 1584 | 1 | 239 | 252 |  |
| T.MYPGIADRM.Q | Y | 32.95 | 1052.4783 | 0.0 | 527.2464 | 2 | 11.68 | 1301 | 1 | 306 | 314 |  |
| L.DTGDGVTH.T | Y | 30.28 | 800.3301 | -0.3 | 401.1722 | 2 | 7.91 | 634 | 3 | 155 | 162 |  |
| K.IIAPPERKYS.V | Y | 30.21 | 1172.6553 | 2.8 | 587.3365 | 2 | 10.01 | 984 | 1 | 330 | 339 |  |
| A.GFAGDDAPR.A | Y | 28.64 | 904.4039 | 0.1 | 453.2093 | 2 | 9.83 | 953 | 1 | 21 | 29 |  |
| G.FAGDDAPRA.V | Y | 26.63 | 918.4195 | -0.5 | 460.2168 | 2 | 9.87 | 959 | 1 | 22 | 30 |  |
| K.IIAPPERKY.S | Y | 25.38 | 1085.6233 | 1.4 | 543.8197 | 2 | 10.17 | 1014 | 1 | 330 | 338 |  |
| E.KSYELPDGQVITIGNER.F | Y | 24.35 | 1917.9795 | 1.3 | 640.3346 | 3 | 12.57 | 1461 | 1 | 239 | 255 |  |
| K.SYELPDGQ.V | Y | 22.58 | 907.3923 | 0.0 | 454.7034 | 2 | 11.17 | 1205 | 1 | 240 | 247 |  |
| Y.DESGPGIVH.R | Y | 21.74 | 909.4192 | -0.6 | 455.7166 | 2 | 10.03 | 989 | 1 | 364 | 372 |  |
| T.NWDDMEKIWHH.S | Y | 20.98 | 1509.6459 | -0.3 | 755.8300 | 2 | 12.54 | 1455 | 1 | 79 | 89 |  |
| E.KSYELPDGQVITI.G | Y | 20.38 | 1461.7715 | -0.5 | 731.8926 | 2 | 13.39 | 1607 | 1 | 239 | 251 |  |
| Y.VGDEAQSK.R | Y | 20.25 | 832.3926 | 0.0 | 417.2036 | 2 | 7.67 | 599 | 2 | 55 | 62 |  |
| L.GMESVGIH.E | Y | 20.20 | 828.3800 | 2.3 | 415.1982 | 2 | 10.51 | 1077 | 1 | 269 | 276 |  |
| E.KSYELPDGQVITIGNE.R | Y | 19.11 | 1761.8784 | 10.8 | 881.9560 | 2 | 13.18 | 1572 | 1 | 239 | 254 |  |
| L.TEAPLNPK.A | Y | 17.45 | 868.4654 | 0.1 | 435.2400 | 2 | 9.61 | 912 | 1 | 107 | 114 |  |
| T.LKYPIE.H | Y | 17.39 | 761.4323 | 1.0 | 381.7238 | 2 | 11.01 | 1175 | 1 | 68 | 73 |  |
| M.YPGIADRM.Q | Y | 16.87 | 921.4378 | -0.3 | 461.7260 | 2 | 11.25 | 1221 | 1 | 307 | 314 |  |
| L.RVAPEESPVLL.T | Y | 16.76 | 1208.6764 | 0.0 | 605.3455 | 2 | 12.50 | 1451 | 1 | 96 | 106 |  |
| E.KSYELPDGQ.V | Y | 15.82 | 1035.4873 | 2.6 | 518.7523 | 2 | 10.49 | 1073 | 1 | 239 | 247 |  |
| total 21 peptides |
| --- |

tr|A0A6A0H005|A0A6A0H005\_HYAAZ

back to list

  

| Protein Coverage
| Supporting Peptides
|

Protein Coverage:

Supporting Peptides:

| Peptide | Uniq | -10lgP | Mass | ppm | m/z | z | RT | Scan | #Spec | Start | End | PTM |
| --- | --- | --- | --- | --- | --- | --- | --- | --- | --- | --- | --- | --- |
| E.EYDESGPGIVH.R | Y | 40.93 | 1201.5251 | 1.2 | 601.7706 | 2 | 10.52 | 1080 | 1 | 362 | 372 |  |
| E.KSYELPDGQVITIG.N | Y | 38.20 | 1518.7930 | -0.7 | 760.4032 | 2 | 13.25 | 1584 | 1 | 239 | 252 |  |
| S.MYPGIADRM.Q | Y | 32.95 | 1052.4783 | 0.0 | 527.2464 | 2 | 11.68 | 1301 | 1 | 306 | 314 |  |
| L.DTGDGVTH.T | Y | 30.28 | 800.3301 | -0.3 | 401.1722 | 2 | 7.91 | 634 | 3 | 155 | 162 |  |
| K.IIAPPERKYS.V | Y | 30.21 | 1172.6553 | 2.8 | 587.3365 | 2 | 10.01 | 984 | 1 | 330 | 339 |  |
| A.GFAGDDAPR.A | Y | 28.64 | 904.4039 | 0.1 | 453.2093 | 2 | 9.83 | 953 | 1 | 21 | 29 |  |
| G.FAGDDAPRA.V | Y | 26.63 | 918.4195 | -0.5 | 460.2168 | 2 | 9.87 | 959 | 1 | 22 | 30 |  |
| K.IIAPPERKY.S | Y | 25.38 | 1085.6233 | 1.4 | 543.8197 | 2 | 10.17 | 1014 | 1 | 330 | 338 |  |
| E.KSYELPDGQVITIGNER.F | Y | 24.35 | 1917.9795 | 1.3 | 640.3346 | 3 | 12.57 | 1461 | 1 | 239 | 255 |  |
| K.SYELPDGQ.V | Y | 22.58 | 907.3923 | 0.0 | 454.7034 | 2 | 11.17 | 1205 | 1 | 240 | 247 |  |
| Y.DESGPGIVH.R | Y | 21.74 | 909.4192 | -0.6 | 455.7166 | 2 | 10.03 | 989 | 1 | 364 | 372 |  |
| T.NWDDMEKIWHH.T | Y | 20.98 | 1509.6459 | -0.3 | 755.8300 | 2 | 12.54 | 1455 | 1 | 79 | 89 |  |
| E.KSYELPDGQVITI.G | Y | 20.38 | 1461.7715 | -0.5 | 731.8926 | 2 | 13.39 | 1607 | 1 | 239 | 251 |  |
| Y.VGDEAQSK.R | Y | 20.25 | 832.3926 | 0.0 | 417.2036 | 2 | 7.67 | 599 | 2 | 55 | 62 |  |
| L.GMESVGIH.E | Y | 20.20 | 828.3800 | 2.3 | 415.1982 | 2 | 10.51 | 1077 | 1 | 269 | 276 |  |
| E.KSYELPDGQVITIGNE.R | Y | 19.11 | 1761.8784 | 10.8 | 881.9560 | 2 | 13.18 | 1572 | 1 | 239 | 254 |  |
| L.TEAPLNPK.A | Y | 17.45 | 868.4654 | 0.1 | 435.2400 | 2 | 9.61 | 912 | 1 | 107 | 114 |  |
| T.LKYPIE.H | Y | 17.39 | 761.4323 | 1.0 | 381.7238 | 2 | 11.01 | 1175 | 1 | 68 | 73 |  |
| M.YPGIADRM.Q | Y | 16.87 | 921.4378 | -0.3 | 461.7260 | 2 | 11.25 | 1221 | 1 | 307 | 314 |  |
| L.RVAPEESPVLL.T | Y | 16.76 | 1208.6764 | 0.0 | 605.3455 | 2 | 12.50 | 1451 | 1 | 96 | 106 |  |
| E.KSYELPDGQ.V | Y | 15.82 | 1035.4873 | 2.6 | 518.7523 | 2 | 10.49 | 1073 | 1 | 239 | 247 |  |
| total 21 peptides |
| --- |

tr|A0A3R7NX50|A0A3R7NX50\_PENVA

back to list

  

| Protein Coverage
| Supporting Peptides
|

Protein Coverage:

Supporting Peptides:

| Peptide | Uniq | -10lgP | Mass | ppm | m/z | z | RT | Scan | #Spec | Start | End | PTM |
| --- | --- | --- | --- | --- | --- | --- | --- | --- | --- | --- | --- | --- |
| E.EYDESGPGIVH.R | Y | 40.93 | 1201.5251 | 1.2 | 601.7706 | 2 | 10.52 | 1080 | 1 | 362 | 372 |  |
| E.KSYELPDGQVITIG.N | Y | 38.20 | 1518.7930 | -0.7 | 760.4032 | 2 | 13.25 | 1584 | 1 | 239 | 252 |  |
| T.MYPGIADRM.Q | Y | 32.95 | 1052.4783 | 0.0 | 527.2464 | 2 | 11.68 | 1301 | 1 | 306 | 314 |  |
| L.DTGDGVTH.T | Y | 30.28 | 800.3301 | -0.3 | 401.1722 | 2 | 7.91 | 634 | 3 | 155 | 162 |  |
| K.IIAPPERKYS.V | Y | 30.21 | 1172.6553 | 2.8 | 587.3365 | 2 | 10.01 | 984 | 1 | 330 | 339 |  |
| A.GFAGDDAPR.A | Y | 28.64 | 904.4039 | 0.1 | 453.2093 | 2 | 9.83 | 953 | 1 | 21 | 29 |  |
| G.FAGDDAPRA.V | Y | 26.63 | 918.4195 | -0.5 | 460.2168 | 2 | 9.87 | 959 | 1 | 22 | 30 |  |
| K.IIAPPERKY.S | Y | 25.38 | 1085.6233 | 1.4 | 543.8197 | 2 | 10.17 | 1014 | 1 | 330 | 338 |  |
| E.KSYELPDGQVITIGNER.F | Y | 24.35 | 1917.9795 | 1.3 | 640.3346 | 3 | 12.57 | 1461 | 1 | 239 | 255 |  |
| K.SYELPDGQ.V | Y | 22.58 | 907.3923 | 0.0 | 454.7034 | 2 | 11.17 | 1205 | 1 | 240 | 247 |  |
| Y.DESGPGIVH.R | Y | 21.74 | 909.4192 | -0.6 | 455.7166 | 2 | 10.03 | 989 | 1 | 364 | 372 |  |
| T.NWDDMEKIWHH.S | Y | 20.98 | 1509.6459 | -0.3 | 755.8300 | 2 | 12.54 | 1455 | 1 | 79 | 89 |  |
| E.KSYELPDGQVITI.G | Y | 20.38 | 1461.7715 | -0.5 | 731.8926 | 2 | 13.39 | 1607 | 1 | 239 | 251 |  |
| Y.VGDEAQSK.R | Y | 20.25 | 832.3926 | 0.0 | 417.2036 | 2 | 7.67 | 599 | 2 | 55 | 62 |  |
| L.GMESVGIH.E | Y | 20.20 | 828.3800 | 2.3 | 415.1982 | 2 | 10.51 | 1077 | 1 | 269 | 276 |  |
| E.KSYELPDGQVITIGNE.R | Y | 19.11 | 1761.8784 | 10.8 | 881.9560 | 2 | 13.18 | 1572 | 1 | 239 | 254 |  |
| L.TEAPLNPK.A | Y | 17.45 | 868.4654 | 0.1 | 435.2400 | 2 | 9.61 | 912 | 1 | 107 | 114 |  |
| T.LKYPIE.H | Y | 17.39 | 761.4323 | 1.0 | 381.7238 | 2 | 11.01 | 1175 | 1 | 68 | 73 |  |
| M.YPGIADRM.Q | Y | 16.87 | 921.4378 | -0.3 | 461.7260 | 2 | 11.25 | 1221 | 1 | 307 | 314 |  |
| L.RVAPEESPVLL.T | Y | 16.76 | 1208.6764 | 0.0 | 605.3455 | 2 | 12.50 | 1451 | 1 | 96 | 106 |  |
| E.KSYELPDGQ.V | Y | 15.82 | 1035.4873 | 2.6 | 518.7523 | 2 | 10.49 | 1073 | 1 | 239 | 247 |  |
| total 21 peptides |
| --- |

tr|A0A482LUC2|A0A482LUC2\_CANBE

back to list

  

| Protein Coverage
| Supporting Peptides
|

Protein Coverage:

Supporting Peptides:

| Peptide | Uniq | -10lgP | Mass | ppm | m/z | z | RT | Scan | #Spec | Start | End | PTM |
| --- | --- | --- | --- | --- | --- | --- | --- | --- | --- | --- | --- | --- |
| E.EYDESGPGIVH.R | Y | 40.93 | 1201.5251 | 1.2 | 601.7706 | 2 | 10.52 | 1080 | 1 | 362 | 372 |  |
| E.KSYELPDGQVITIG.N | Y | 38.20 | 1518.7930 | -0.7 | 760.4032 | 2 | 13.25 | 1584 | 1 | 239 | 252 |  |
| T.MYPGIADRM.Q | Y | 32.95 | 1052.4783 | 0.0 | 527.2464 | 2 | 11.68 | 1301 | 1 | 306 | 314 |  |
| L.DTGDGVTH.T | Y | 30.28 | 800.3301 | -0.3 | 401.1722 | 2 | 7.91 | 634 | 3 | 155 | 162 |  |
| K.IIAPPERKYS.V | Y | 30.21 | 1172.6553 | 2.8 | 587.3365 | 2 | 10.01 | 984 | 1 | 330 | 339 |  |
| A.GFAGDDAPR.A | Y | 28.64 | 904.4039 | 0.1 | 453.2093 | 2 | 9.83 | 953 | 1 | 21 | 29 |  |
| G.FAGDDAPRA.V | Y | 26.63 | 918.4195 | -0.5 | 460.2168 | 2 | 9.87 | 959 | 1 | 22 | 30 |  |
| K.IIAPPERKY.S | Y | 25.38 | 1085.6233 | 1.4 | 543.8197 | 2 | 10.17 | 1014 | 1 | 330 | 338 |  |
| E.KSYELPDGQVITIGNER.F | Y | 24.35 | 1917.9795 | 1.3 | 640.3346 | 3 | 12.57 | 1461 | 1 | 239 | 255 |  |
| K.SYELPDGQ.V | Y | 22.58 | 907.3923 | 0.0 | 454.7034 | 2 | 11.17 | 1205 | 1 | 240 | 247 |  |
| Y.DESGPGIVH.R | Y | 21.74 | 909.4192 | -0.6 | 455.7166 | 2 | 10.03 | 989 | 1 | 364 | 372 |  |
| T.NWDDMEKIWHH.S | Y | 20.98 | 1509.6459 | -0.3 | 755.8300 | 2 | 12.54 | 1455 | 1 | 79 | 89 |  |
| E.KSYELPDGQVITI.G | Y | 20.38 | 1461.7715 | -0.5 | 731.8926 | 2 | 13.39 | 1607 | 1 | 239 | 251 |  |
| Y.VGDEAQSK.R | Y | 20.25 | 832.3926 | 0.0 | 417.2036 | 2 | 7.67 | 599 | 2 | 55 | 62 |  |
| L.GMESVGIH.E | Y | 20.20 | 828.3800 | 2.3 | 415.1982 | 2 | 10.51 | 1077 | 1 | 269 | 276 |  |
| E.KSYELPDGQVITIGNE.R | Y | 19.11 | 1761.8784 | 10.8 | 881.9560 | 2 | 13.18 | 1572 | 1 | 239 | 254 |  |
| L.TEAPLNPK.A | Y | 17.45 | 868.4654 | 0.1 | 435.2400 | 2 | 9.61 | 912 | 1 | 107 | 114 |  |
| T.LKYPIE.H | Y | 17.39 | 761.4323 | 1.0 | 381.7238 | 2 | 11.01 | 1175 | 1 | 68 | 73 |  |
| M.YPGIADRM.Q | Y | 16.87 | 921.4378 | -0.3 | 461.7260 | 2 | 11.25 | 1221 | 1 | 307 | 314 |  |
| L.RVAPEESPVLL.T | Y | 16.76 | 1208.6764 | 0.0 | 605.3455 | 2 | 12.50 | 1451 | 1 | 96 | 106 |  |
| E.KSYELPDGQ.V | Y | 15.82 | 1035.4873 | 2.6 | 518.7523 | 2 | 10.49 | 1073 | 1 | 239 | 247 |  |
| total 21 peptides |
| --- |

tr|A0A0B5J9E4|A0A0B5J9E4\_ERISI

back to list

  

| Protein Coverage
| Supporting Peptides
|

Protein Coverage:

Supporting Peptides:

| Peptide | Uniq | -10lgP | Mass | ppm | m/z | z | RT | Scan | #Spec | Start | End | PTM |
| --- | --- | --- | --- | --- | --- | --- | --- | --- | --- | --- | --- | --- |
| E.EYDESGPGIVH.R | Y | 40.93 | 1201.5251 | 1.2 | 601.7706 | 2 | 10.52 | 1080 | 1 | 362 | 372 |  |
| E.KSYELPDGQVITIG.N | Y | 38.20 | 1518.7930 | -0.7 | 760.4032 | 2 | 13.25 | 1584 | 1 | 239 | 252 |  |
| A.MYPGIADRM.Q | Y | 32.95 | 1052.4783 | 0.0 | 527.2464 | 2 | 11.68 | 1301 | 1 | 306 | 314 |  |
| L.DTGDGVTH.T | Y | 30.28 | 800.3301 | -0.3 | 401.1722 | 2 | 7.91 | 634 | 3 | 155 | 162 |  |
| K.IIAPPERKYS.V | Y | 30.21 | 1172.6553 | 2.8 | 587.3365 | 2 | 10.01 | 984 | 1 | 330 | 339 |  |
| A.GFAGDDAPR.A | Y | 28.64 | 904.4039 | 0.1 | 453.2093 | 2 | 9.83 | 953 | 1 | 21 | 29 |  |
| G.FAGDDAPRA.V | Y | 26.63 | 918.4195 | -0.5 | 460.2168 | 2 | 9.87 | 959 | 1 | 22 | 30 |  |
| K.IIAPPERKY.S | Y | 25.38 | 1085.6233 | 1.4 | 543.8197 | 2 | 10.17 | 1014 | 1 | 330 | 338 |  |
| E.KSYELPDGQVITIGNER.F | Y | 24.35 | 1917.9795 | 1.3 | 640.3346 | 3 | 12.57 | 1461 | 1 | 239 | 255 |  |
| K.SYELPDGQ.V | Y | 22.58 | 907.3923 | 0.0 | 454.7034 | 2 | 11.17 | 1205 | 1 | 240 | 247 |  |
| Y.DESGPGIVH.R | Y | 21.74 | 909.4192 | -0.6 | 455.7166 | 2 | 10.03 | 989 | 1 | 364 | 372 |  |
| T.NWDDMEKIWHH.S | Y | 20.98 | 1509.6459 | -0.3 | 755.8300 | 2 | 12.54 | 1455 | 1 | 79 | 89 |  |
| E.KSYELPDGQVITI.G | Y | 20.38 | 1461.7715 | -0.5 | 731.8926 | 2 | 13.39 | 1607 | 1 | 239 | 251 |  |
| Y.VGDEAQSK.R | Y | 20.25 | 832.3926 | 0.0 | 417.2036 | 2 | 7.67 | 599 | 2 | 55 | 62 |  |
| L.GMESVGIH.E | Y | 20.20 | 828.3800 | 2.3 | 415.1982 | 2 | 10.51 | 1077 | 1 | 269 | 276 |  |
| E.KSYELPDGQVITIGNE.R | Y | 19.11 | 1761.8784 | 10.8 | 881.9560 | 2 | 13.18 | 1572 | 1 | 239 | 254 |  |
| L.TEAPLNPK.A | Y | 17.45 | 868.4654 | 0.1 | 435.2400 | 2 | 9.61 | 912 | 1 | 107 | 114 |  |
| T.LKYPIE.H | Y | 17.39 | 761.4323 | 1.0 | 381.7238 | 2 | 11.01 | 1175 | 1 | 68 | 73 |  |
| M.YPGIADRM.Q | Y | 16.87 | 921.4378 | -0.3 | 461.7260 | 2 | 11.25 | 1221 | 1 | 307 | 314 |  |
| L.RVAPEESPVLL.T | Y | 16.76 | 1208.6764 | 0.0 | 605.3455 | 2 | 12.50 | 1451 | 1 | 96 | 106 |  |
| E.KSYELPDGQ.V | Y | 15.82 | 1035.4873 | 2.6 | 518.7523 | 2 | 10.49 | 1073 | 1 | 239 | 247 |  |
| total 21 peptides |
| --- |

tr|A0A2H4V3D6|A0A2H4V3D6\_PENVA

back to list

  

| Protein Coverage
| Supporting Peptides
|

Protein Coverage:

Supporting Peptides:

| Peptide | Uniq | -10lgP | Mass | ppm | m/z | z | RT | Scan | #Spec | Start | End | PTM |
| --- | --- | --- | --- | --- | --- | --- | --- | --- | --- | --- | --- | --- |
| E.EYDESGPGIVH.R | Y | 40.93 | 1201.5251 | 1.2 | 601.7706 | 2 | 10.52 | 1080 | 1 | 362 | 372 |  |
| E.KSYELPDGQVITIG.N | Y | 38.20 | 1518.7930 | -0.7 | 760.4032 | 2 | 13.25 | 1584 | 1 | 239 | 252 |  |
| T.MYPGIADRM.Q | Y | 32.95 | 1052.4783 | 0.0 | 527.2464 | 2 | 11.68 | 1301 | 1 | 306 | 314 |  |
| L.DTGDGVTH.T | Y | 30.28 | 800.3301 | -0.3 | 401.1722 | 2 | 7.91 | 634 | 3 | 155 | 162 |  |
| K.IIAPPERKYS.V | Y | 30.21 | 1172.6553 | 2.8 | 587.3365 | 2 | 10.01 | 984 | 1 | 330 | 339 |  |
| A.GFAGDDAPR.A | Y | 28.64 | 904.4039 | 0.1 | 453.2093 | 2 | 9.83 | 953 | 1 | 21 | 29 |  |
| G.FAGDDAPRA.V | Y | 26.63 | 918.4195 | -0.5 | 460.2168 | 2 | 9.87 | 959 | 1 | 22 | 30 |  |
| K.IIAPPERKY.S | Y | 25.38 | 1085.6233 | 1.4 | 543.8197 | 2 | 10.17 | 1014 | 1 | 330 | 338 |  |
| E.KSYELPDGQVITIGNER.F | Y | 24.35 | 1917.9795 | 1.3 | 640.3346 | 3 | 12.57 | 1461 | 1 | 239 | 255 |  |
| K.SYELPDGQ.V | Y | 22.58 | 907.3923 | 0.0 | 454.7034 | 2 | 11.17 | 1205 | 1 | 240 | 247 |  |
| Y.DESGPGIVH.R | Y | 21.74 | 909.4192 | -0.6 | 455.7166 | 2 | 10.03 | 989 | 1 | 364 | 372 |  |
| T.NWDDMEKIWHH.S | Y | 20.98 | 1509.6459 | -0.3 | 755.8300 | 2 | 12.54 | 1455 | 1 | 79 | 89 |  |
| E.KSYELPDGQVITI.G | Y | 20.38 | 1461.7715 | -0.5 | 731.8926 | 2 | 13.39 | 1607 | 1 | 239 | 251 |  |
| Y.VGDEAQSK.R | Y | 20.25 | 832.3926 | 0.0 | 417.2036 | 2 | 7.67 | 599 | 2 | 55 | 62 |  |
| L.GMESVGIH.E | Y | 20.20 | 828.3800 | 2.3 | 415.1982 | 2 | 10.51 | 1077 | 1 | 269 | 276 |  |
| E.KSYELPDGQVITIGNE.R | Y | 19.11 | 1761.8784 | 10.8 | 881.9560 | 2 | 13.18 | 1572 | 1 | 239 | 254 |  |
| L.TEAPLNPK.A | Y | 17.45 | 868.4654 | 0.1 | 435.2400 | 2 | 9.61 | 912 | 1 | 107 | 114 |  |
| T.LKYPIE.H | Y | 17.39 | 761.4323 | 1.0 | 381.7238 | 2 | 11.01 | 1175 | 1 | 68 | 73 |  |
| M.YPGIADRM.Q | Y | 16.87 | 921.4378 | -0.3 | 461.7260 | 2 | 11.25 | 1221 | 1 | 307 | 314 |  |
| L.RVAPEESPVLL.T | Y | 16.76 | 1208.6764 | 0.0 | 605.3455 | 2 | 12.50 | 1451 | 1 | 96 | 106 |  |
| E.KSYELPDGQ.V | Y | 15.82 | 1035.4873 | 2.6 | 518.7523 | 2 | 10.49 | 1073 | 1 | 239 | 247 |  |
| total 21 peptides |
| --- |

tr|A0A2H4V3F0|A0A2H4V3F0\_PENVA

back to list

  

| Protein Coverage
| Supporting Peptides
|

Protein Coverage:

Supporting Peptides:

| Peptide | Uniq | -10lgP | Mass | ppm | m/z | z | RT | Scan | #Spec | Start | End | PTM |
| --- | --- | --- | --- | --- | --- | --- | --- | --- | --- | --- | --- | --- |
| E.EYDESGPGIVH.R | Y | 40.93 | 1201.5251 | 1.2 | 601.7706 | 2 | 10.52 | 1080 | 1 | 362 | 372 |  |
| E.KSYELPDGQVITIG.N | Y | 38.20 | 1518.7930 | -0.7 | 760.4032 | 2 | 13.25 | 1584 | 1 | 239 | 252 |  |
| T.MYPGIADRM.Q | Y | 32.95 | 1052.4783 | 0.0 | 527.2464 | 2 | 11.68 | 1301 | 1 | 306 | 314 |  |
| L.DTGDGVTH.T | Y | 30.28 | 800.3301 | -0.3 | 401.1722 | 2 | 7.91 | 634 | 3 | 155 | 162 |  |
| K.IIAPPERKYS.V | Y | 30.21 | 1172.6553 | 2.8 | 587.3365 | 2 | 10.01 | 984 | 1 | 330 | 339 |  |
| A.GFAGDDAPR.A | Y | 28.64 | 904.4039 | 0.1 | 453.2093 | 2 | 9.83 | 953 | 1 | 21 | 29 |  |
| G.FAGDDAPRA.V | Y | 26.63 | 918.4195 | -0.5 | 460.2168 | 2 | 9.87 | 959 | 1 | 22 | 30 |  |
| K.IIAPPERKY.S | Y | 25.38 | 1085.6233 | 1.4 | 543.8197 | 2 | 10.17 | 1014 | 1 | 330 | 338 |  |
| E.KSYELPDGQVITIGNER.F | Y | 24.35 | 1917.9795 | 1.3 | 640.3346 | 3 | 12.57 | 1461 | 1 | 239 | 255 |  |
| K.SYELPDGQ.V | Y | 22.58 | 907.3923 | 0.0 | 454.7034 | 2 | 11.17 | 1205 | 1 | 240 | 247 |  |
| Y.DESGPGIVH.R | Y | 21.74 | 909.4192 | -0.6 | 455.7166 | 2 | 10.03 | 989 | 1 | 364 | 372 |  |
| T.NWDDMEKIWHH.S | Y | 20.98 | 1509.6459 | -0.3 | 755.8300 | 2 | 12.54 | 1455 | 1 | 79 | 89 |  |
| E.KSYELPDGQVITI.G | Y | 20.38 | 1461.7715 | -0.5 | 731.8926 | 2 | 13.39 | 1607 | 1 | 239 | 251 |  |
| Y.VGDEAQSK.R | Y | 20.25 | 832.3926 | 0.0 | 417.2036 | 2 | 7.67 | 599 | 2 | 55 | 62 |  |
| L.GMESVGIH.E | Y | 20.20 | 828.3800 | 2.3 | 415.1982 | 2 | 10.51 | 1077 | 1 | 269 | 276 |  |
| E.KSYELPDGQVITIGNE.R | Y | 19.11 | 1761.8784 | 10.8 | 881.9560 | 2 | 13.18 | 1572 | 1 | 239 | 254 |  |
| L.TEAPLNPK.A | Y | 17.45 | 868.4654 | 0.1 | 435.2400 | 2 | 9.61 | 912 | 1 | 107 | 114 |  |
| T.LKYPIE.H | Y | 17.39 | 761.4323 | 1.0 | 381.7238 | 2 | 11.01 | 1175 | 1 | 68 | 73 |  |
| M.YPGIADRM.Q | Y | 16.87 | 921.4378 | -0.3 | 461.7260 | 2 | 11.25 | 1221 | 1 | 307 | 314 |  |
| L.RVAPEESPVLL.T | Y | 16.76 | 1208.6764 | 0.0 | 605.3455 | 2 | 12.50 | 1451 | 1 | 96 | 106 |  |
| E.KSYELPDGQ.V | Y | 15.82 | 1035.4873 | 2.6 | 518.7523 | 2 | 10.49 | 1073 | 1 | 239 | 247 |  |
| total 21 peptides |
| --- |

tr|U3M7F7|U3M7F7\_PORTR

back to list

  

| Protein Coverage
| Supporting Peptides
|

Protein Coverage:

Supporting Peptides:

| Peptide | Uniq | -10lgP | Mass | ppm | m/z | z | RT | Scan | #Spec | Start | End | PTM |
| --- | --- | --- | --- | --- | --- | --- | --- | --- | --- | --- | --- | --- |
| E.EYDESGPGIVH.R | Y | 40.93 | 1201.5251 | 1.2 | 601.7706 | 2 | 10.52 | 1080 | 1 | 362 | 372 |  |
| E.KSYELPDGQVITIG.N | Y | 38.20 | 1518.7930 | -0.7 | 760.4032 | 2 | 13.25 | 1584 | 1 | 239 | 252 |  |
| T.MYPGIADRM.Q | Y | 32.95 | 1052.4783 | 0.0 | 527.2464 | 2 | 11.68 | 1301 | 1 | 306 | 314 |  |
| L.DTGDGVTH.T | Y | 30.28 | 800.3301 | -0.3 | 401.1722 | 2 | 7.91 | 634 | 3 | 155 | 162 |  |
| K.IIAPPERKYS.V | Y | 30.21 | 1172.6553 | 2.8 | 587.3365 | 2 | 10.01 | 984 | 1 | 330 | 339 |  |
| A.GFAGDDAPR.A | Y | 28.64 | 904.4039 | 0.1 | 453.2093 | 2 | 9.83 | 953 | 1 | 21 | 29 |  |
| G.FAGDDAPRA.V | Y | 26.63 | 918.4195 | -0.5 | 460.2168 | 2 | 9.87 | 959 | 1 | 22 | 30 |  |
| K.IIAPPERKY.S | Y | 25.38 | 1085.6233 | 1.4 | 543.8197 | 2 | 10.17 | 1014 | 1 | 330 | 338 |  |
| E.KSYELPDGQVITIGNER.F | Y | 24.35 | 1917.9795 | 1.3 | 640.3346 | 3 | 12.57 | 1461 | 1 | 239 | 255 |  |
| K.SYELPDGQ.V | Y | 22.58 | 907.3923 | 0.0 | 454.7034 | 2 | 11.17 | 1205 | 1 | 240 | 247 |  |
| Y.DESGPGIVH.R | Y | 21.74 | 909.4192 | -0.6 | 455.7166 | 2 | 10.03 | 989 | 1 | 364 | 372 |  |
| T.NWDDMEKIWHH.S | Y | 20.98 | 1509.6459 | -0.3 | 755.8300 | 2 | 12.54 | 1455 | 1 | 79 | 89 |  |
| E.KSYELPDGQVITI.G | Y | 20.38 | 1461.7715 | -0.5 | 731.8926 | 2 | 13.39 | 1607 | 1 | 239 | 251 |  |
| Y.VGDEAQSK.R | Y | 20.25 | 832.3926 | 0.0 | 417.2036 | 2 | 7.67 | 599 | 2 | 55 | 62 |  |
| L.GMESVGIH.E | Y | 20.20 | 828.3800 | 2.3 | 415.1982 | 2 | 10.51 | 1077 | 1 | 269 | 276 |  |
| E.KSYELPDGQVITIGNE.R | Y | 19.11 | 1761.8784 | 10.8 | 881.9560 | 2 | 13.18 | 1572 | 1 | 239 | 254 |  |
| L.TEAPLNPK.A | Y | 17.45 | 868.4654 | 0.1 | 435.2400 | 2 | 9.61 | 912 | 1 | 107 | 114 |  |
| T.LKYPIE.H | Y | 17.39 | 761.4323 | 1.0 | 381.7238 | 2 | 11.01 | 1175 | 1 | 68 | 73 |  |
| M.YPGIADRM.Q | Y | 16.87 | 921.4378 | -0.3 | 461.7260 | 2 | 11.25 | 1221 | 1 | 307 | 314 |  |
| L.RVAPEESPVLL.T | Y | 16.76 | 1208.6764 | 0.0 | 605.3455 | 2 | 12.50 | 1451 | 1 | 96 | 106 |  |
| E.KSYELPDGQ.V | Y | 15.82 | 1035.4873 | 2.6 | 518.7523 | 2 | 10.49 | 1073 | 1 | 239 | 247 |  |
| total 21 peptides |
| --- |

tr|A7BJ42|A7BJ42\_PROCL

back to list

  

| Protein Coverage
| Supporting Peptides
|

Protein Coverage:

Supporting Peptides:

| Peptide | Uniq | -10lgP | Mass | ppm | m/z | z | RT | Scan | #Spec | Start | End | PTM |
| --- | --- | --- | --- | --- | --- | --- | --- | --- | --- | --- | --- | --- |
| Q.SDLLPVAPEFPHPIPQ.F | N | 49.35 | 1755.9196 | -0.5 | 878.9666 | 2 | 13.50 | 1627 | 1 | 96 | 111 |  |
| V.APEFPHPIPQF.V | Y | 37.99 | 1278.6396 | 0.6 | 640.3275 | 2 | 13.08 | 1553 | 1 | 102 | 112 |  |
| P.VAPEFPHPIPQ.F | N | 35.26 | 1230.6396 | -0.1 | 616.3270 | 2 | 11.74 | 1313 | 1 | 101 | 111 |  |
| V.APEFPHPIPQ.F | N | 33.54 | 1131.5713 | -0.6 | 566.7926 | 2 | 11.56 | 1278 | 1 | 102 | 111 |  |
| total 4 peptides |
| --- |

tr|D7RVB0|D7RVB0\_CALSI

back to list

  

| Protein Coverage
| Supporting Peptides
|

Protein Coverage:

Supporting Peptides:

| Peptide | Uniq | -10lgP | Mass | ppm | m/z | z | RT | Scan | #Spec | Start | End | PTM |
| --- | --- | --- | --- | --- | --- | --- | --- | --- | --- | --- | --- | --- |
| E.SDLLPVAPEFPHPIPQ.F | N | 49.35 | 1755.9196 | -0.5 | 878.9666 | 2 | 13.50 | 1627 | 1 | 103 | 118 |  |
| V.APEFPHPIPQF.V | Y | 37.99 | 1278.6396 | 0.6 | 640.3275 | 2 | 13.08 | 1553 | 1 | 109 | 119 |  |
| P.VAPEFPHPIPQ.F | N | 35.26 | 1230.6396 | -0.1 | 616.3270 | 2 | 11.74 | 1313 | 1 | 108 | 118 |  |
| V.APEFPHPIPQ.F | N | 33.54 | 1131.5713 | -0.6 | 566.7926 | 2 | 11.56 | 1278 | 1 | 109 | 118 |  |
| total 4 peptides |
| --- |

tr|D7RVB1|D7RVB1\_CALSI

back to list

  

| Protein Coverage
| Supporting Peptides
|

Protein Coverage:

Supporting Peptides:

| Peptide | Uniq | -10lgP | Mass | ppm | m/z | z | RT | Scan | #Spec | Start | End | PTM |
| --- | --- | --- | --- | --- | --- | --- | --- | --- | --- | --- | --- | --- |
| E.SDLLPVAPEFPHPIPQ.F | N | 49.35 | 1755.9196 | -0.5 | 878.9666 | 2 | 13.50 | 1627 | 1 | 103 | 118 |  |
| V.APEFPHPIPQF.V | Y | 37.99 | 1278.6396 | 0.6 | 640.3275 | 2 | 13.08 | 1553 | 1 | 109 | 119 |  |
| P.VAPEFPHPIPQ.F | N | 35.26 | 1230.6396 | -0.1 | 616.3270 | 2 | 11.74 | 1313 | 1 | 108 | 118 |  |
| V.APEFPHPIPQ.F | N | 33.54 | 1131.5713 | -0.6 | 566.7926 | 2 | 11.56 | 1278 | 1 | 109 | 118 |  |
| total 4 peptides |
| --- |

tr|D7RVA8|D7RVA8\_CALSI

back to list

  

| Protein Coverage
| Supporting Peptides
|

Protein Coverage:

Supporting Peptides:

| Peptide | Uniq | -10lgP | Mass | ppm | m/z | z | RT | Scan | #Spec | Start | End | PTM |
| --- | --- | --- | --- | --- | --- | --- | --- | --- | --- | --- | --- | --- |
| E.SDLLPVAPEFPHPIPQ.F | N | 49.35 | 1755.9196 | -0.5 | 878.9666 | 2 | 13.50 | 1627 | 1 | 103 | 118 |  |
| V.APEFPHPIPQF.V | Y | 37.99 | 1278.6396 | 0.6 | 640.3275 | 2 | 13.08 | 1553 | 1 | 109 | 119 |  |
| P.VAPEFPHPIPQ.F | N | 35.26 | 1230.6396 | -0.1 | 616.3270 | 2 | 11.74 | 1313 | 1 | 108 | 118 |  |
| V.APEFPHPIPQ.F | N | 33.54 | 1131.5713 | -0.6 | 566.7926 | 2 | 11.56 | 1278 | 1 | 109 | 118 |  |
| total 4 peptides |
| --- |

tr|D7RVA9|D7RVA9\_CALSI

back to list

  

| Protein Coverage
| Supporting Peptides
|

Protein Coverage:

Supporting Peptides:

| Peptide | Uniq | -10lgP | Mass | ppm | m/z | z | RT | Scan | #Spec | Start | End | PTM |
| --- | --- | --- | --- | --- | --- | --- | --- | --- | --- | --- | --- | --- |
| E.SDLLPVAPEFPHPIPQ.F | N | 49.35 | 1755.9196 | -0.5 | 878.9666 | 2 | 13.50 | 1627 | 1 | 103 | 118 |  |
| V.APEFPHPIPQF.V | Y | 37.99 | 1278.6396 | 0.6 | 640.3275 | 2 | 13.08 | 1553 | 1 | 109 | 119 |  |
| P.VAPEFPHPIPQ.F | N | 35.26 | 1230.6396 | -0.1 | 616.3270 | 2 | 11.74 | 1313 | 1 | 108 | 118 |  |
| V.APEFPHPIPQ.F | N | 33.54 | 1131.5713 | -0.6 | 566.7926 | 2 | 11.56 | 1278 | 1 | 109 | 118 |  |
| total 4 peptides |
| --- |

tr|A0A5B7E6L1|A0A5B7E6L1\_PORTR

back to list

  

| Protein Coverage
| Supporting Peptides
|

Protein Coverage:

Supporting Peptides:

| Peptide | Uniq | -10lgP | Mass | ppm | m/z | z | RT | Scan | #Spec | Start | End | PTM |
| --- | --- | --- | --- | --- | --- | --- | --- | --- | --- | --- | --- | --- |
| Q.SDLLPVAPEFPHPIPQ.F | N | 49.35 | 1755.9196 | -0.5 | 878.9666 | 2 | 13.50 | 1627 | 1 | 114 | 129 |  |
| V.APEFPHPIPQF.V | Y | 37.99 | 1278.6396 | 0.6 | 640.3275 | 2 | 13.08 | 1553 | 1 | 120 | 130 |  |
| P.VAPEFPHPIPQ.F | N | 35.26 | 1230.6396 | -0.1 | 616.3270 | 2 | 11.74 | 1313 | 1 | 119 | 129 |  |
| V.APEFPHPIPQ.F | N | 33.54 | 1131.5713 | -0.6 | 566.7926 | 2 | 11.56 | 1278 | 1 | 120 | 129 |  |
| total 4 peptides |
| --- |

tr|A0A5B7E6X8|A0A5B7E6X8\_PORTR

back to list

  

| Protein Coverage
| Supporting Peptides
|

Protein Coverage:

Supporting Peptides:

| Peptide | Uniq | -10lgP | Mass | ppm | m/z | z | RT | Scan | #Spec | Start | End | PTM |
| --- | --- | --- | --- | --- | --- | --- | --- | --- | --- | --- | --- | --- |
| E.SDLLPVAPEFPHPIPQ.F | N | 49.35 | 1755.9196 | -0.5 | 878.9666 | 2 | 13.50 | 1627 | 1 | 444 | 459 |  |
| V.APEFPHPIPQF.V | Y | 37.99 | 1278.6396 | 0.6 | 640.3275 | 2 | 13.08 | 1553 | 1 | 257 | 267 |  |
| P.VAPEFPHPIPQ.F | N | 35.26 | 1230.6396 | -0.1 | 616.3270 | 2 | 11.74 | 1313 | 1 | 256 | 266 |  |
| V.APEFPHPIPQ.F | N | 33.54 | 1131.5713 | -0.6 | 566.7926 | 2 | 11.56 | 1278 | 1 | 257 | 266 |  |
| total 4 peptides |
| --- |

tr|A0A5B7E6A3|A0A5B7E6A3\_PORTR

back to list

  

| Protein Coverage
| Supporting Peptides
|

Protein Coverage:

Supporting Peptides:

| Peptide | Uniq | -10lgP | Mass | ppm | m/z | z | RT | Scan | #Spec | Start | End | PTM |
| --- | --- | --- | --- | --- | --- | --- | --- | --- | --- | --- | --- | --- |
| E.SDLLPVAPEFPHPIPQ.Y | N | 49.35 | 1755.9196 | -0.5 | 878.9666 | 2 | 13.50 | 1627 | 1 | 109 | 124 |  |
| P.VAPEFPHPIPQ.Y | N | 35.26 | 1230.6396 | -0.1 | 616.3270 | 2 | 11.74 | 1313 | 1 | 114 | 124 |  |
| V.APEFPHPIPQ.Y | N | 33.54 | 1131.5713 | -0.6 | 566.7926 | 2 | 11.56 | 1278 | 1 | 115 | 124 |  |
| P.VAPEFPHPIPQY.V | Y | 20.02 | 1393.7030 | -0.5 | 697.8584 | 2 | 12.39 | 1430 | 1 | 114 | 125 |  |
| total 4 peptides |
| --- |

tr|A0A5B7IZE3|A0A5B7IZE3\_PORTR

back to list

  

| Protein Coverage
| Supporting Peptides
|

Protein Coverage:

Supporting Peptides:

| Peptide | Uniq | -10lgP | Mass | ppm | m/z | z | RT | Scan | #Spec | Start | End | PTM |
| --- | --- | --- | --- | --- | --- | --- | --- | --- | --- | --- | --- | --- |
| L.SILEEESMFPK.A | N | 38.15 | 1308.6271 | -0.2 | 655.3207 | 2 | 12.66 | 1478 | 1 | 5 | 15 |  |
| E.KNKDPLNDTLVDLL.K | Y | 33.35 | 1596.8722 | 0.6 | 799.4438 | 2 | 13.69 | 1659 | 1 | 70 | 83 |  |
| N.ETKSPGVIDAGLVM.H | Y | 31.05 | 1415.7330 | -0.3 | 708.8736 | 2 | 12.71 | 1487 | 1 | 152 | 165 |  |
| K.NKDPLNDTL.V | N | 28.03 | 1028.5138 | -0.3 | 515.2640 | 2 | 11.18 | 1207 | 1 | 71 | 79 |  |
| K.NKDPLNDTLVDLL.K | Y | 27.68 | 1468.7773 | -0.1 | 735.3959 | 2 | 14.30 | 1755 | 1 | 71 | 83 |  |
| E.KNKDPLNDTLVDL.L | Y | 22.98 | 1483.7882 | 2.7 | 742.9034 | 2 | 12.42 | 1435 | 1 | 70 | 82 |  |
| total 6 peptides |
| --- |

tr|F8WR03|F8WR03\_PENJP

back to list

  

| Protein Coverage
| Supporting Peptides
|

Protein Coverage:

Supporting Peptides:

| Peptide | Uniq | -10lgP | Mass | ppm | m/z | z | RT | Scan | #Spec | Start | End | PTM |
| --- | --- | --- | --- | --- | --- | --- | --- | --- | --- | --- | --- | --- |
| K.VTVPSIDDKEDMQF.T | N | 49.33 | 1622.7498 | -0.8 | 812.3815 | 2 | 12.56 | 1459 | 1 | 318 | 331 |  |
| L.SILEEESMFPK.A | N | 38.15 | 1308.6271 | -0.2 | 655.3207 | 2 | 12.66 | 1478 | 1 | 532 | 542 |  |
| K.VTVPSIDDKEDMQ.F | N | 21.58 | 1475.6814 | -0.3 | 738.8478 | 2 | 11.23 | 1217 | 1 | 318 | 330 |  |
| R.NEVPPHLF.A | N | 19.14 | 951.4814 | 0.3 | 476.7481 | 2 | 12.21 | 1398 | 1 | 152 | 159 |  |
| total 4 peptides |
| --- |

tr|K4Q111|K4Q111\_PENVA

back to list

  

| Protein Coverage
| Supporting Peptides
|

Protein Coverage:

Supporting Peptides:

| Peptide | Uniq | -10lgP | Mass | ppm | m/z | z | RT | Scan | #Spec | Start | End | PTM |
| --- | --- | --- | --- | --- | --- | --- | --- | --- | --- | --- | --- | --- |
| K.VTVPSIDDKEDMQF.T | N | 49.33 | 1622.7498 | -0.8 | 812.3815 | 2 | 12.56 | 1459 | 1 | 318 | 331 |  |
| L.SILEEESMFPK.A | N | 38.15 | 1308.6271 | -0.2 | 655.3207 | 2 | 12.66 | 1478 | 1 | 532 | 542 |  |
| K.VTVPSIDDKEDMQ.F | N | 21.58 | 1475.6814 | -0.3 | 738.8478 | 2 | 11.23 | 1217 | 1 | 318 | 330 |  |
| R.NEVPPHLF.A | N | 19.14 | 951.4814 | 0.3 | 476.7481 | 2 | 12.21 | 1398 | 1 | 152 | 159 |  |
| total 4 peptides |
| --- |

tr|K4Q4N8|K4Q4N8\_PENMO

back to list

  

| Protein Coverage
| Supporting Peptides
|

Protein Coverage:

Supporting Peptides:

| Peptide | Uniq | -10lgP | Mass | ppm | m/z | z | RT | Scan | #Spec | Start | End | PTM |
| --- | --- | --- | --- | --- | --- | --- | --- | --- | --- | --- | --- | --- |
| K.VTVPSIDDKEDMQF.T | N | 49.33 | 1622.7498 | -0.8 | 812.3815 | 2 | 12.56 | 1459 | 1 | 318 | 331 |  |
| L.SILEEESMFPK.A | N | 38.15 | 1308.6271 | -0.2 | 655.3207 | 2 | 12.66 | 1478 | 1 | 532 | 542 |  |
| K.VTVPSIDDKEDMQ.F | N | 21.58 | 1475.6814 | -0.3 | 738.8478 | 2 | 11.23 | 1217 | 1 | 318 | 330 |  |
| R.NEVPPHLF.A | N | 19.14 | 951.4814 | 0.3 | 476.7481 | 2 | 12.21 | 1398 | 1 | 152 | 159 |  |
| total 4 peptides |
| --- |

tr|A0A2P1JJ59|A0A2P1JJ59\_PROCL

back to list

  

| Protein Coverage
| Supporting Peptides
|

Protein Coverage:

Supporting Peptides:

| Peptide | Uniq | -10lgP | Mass | ppm | m/z | z | RT | Scan | #Spec | Start | End | PTM |
| --- | --- | --- | --- | --- | --- | --- | --- | --- | --- | --- | --- | --- |
| R.TSFDTFPDKVALQ.L | Y | 50.35 | 1467.7245 | 0.3 | 734.8698 | 2 | 12.43 | 1436 | 1 | 325 | 337 |  |
| F.FSPNNPDQFKDL.V | Y | 27.08 | 1420.6622 | 0.2 | 711.3386 | 2 | 11.99 | 1359 | 1 | 751 | 762 |  |
| R.TSFDTFPDKVA.L | Y | 20.75 | 1226.5819 | 1.2 | 614.2990 | 2 | 11.95 | 1353 | 1 | 325 | 335 |  |
| T.SFDTFPDKVALQ.L | Y | 18.60 | 1366.6769 | 0.6 | 684.3461 | 2 | 12.40 | 1431 | 1 | 326 | 337 |  |
| total 4 peptides |
| --- |

tr|A0A5B7DQZ0|A0A5B7DQZ0\_PORTR

back to list

  

| Protein Coverage
| Supporting Peptides
|

Protein Coverage:

Supporting Peptides:

| Peptide | Uniq | -10lgP | Mass | ppm | m/z | z | RT | Scan | #Spec | Start | End | PTM |
| --- | --- | --- | --- | --- | --- | --- | --- | --- | --- | --- | --- | --- |
| L.SILEEESMFPK.A | N | 38.15 | 1308.6271 | -0.2 | 655.3207 | 2 | 12.66 | 1478 | 1 | 403 | 413 |  |
| K.VTVPSIDDGEDM.Q | Y | 37.10 | 1276.5493 | -0.5 | 639.2816 | 2 | 12.32 | 1417 | 1 | 189 | 200 |  |
| K.NKDPLNDTL.V | N | 28.03 | 1028.5138 | -0.3 | 515.2640 | 2 | 11.18 | 1207 | 1 | 469 | 477 |  |
| R.NEVPPHLF.A | N | 19.14 | 951.4814 | 0.3 | 476.7481 | 2 | 12.21 | 1398 | 1 | 150 | 157 |  |
| N.LNDEIKDL.V | Y | 17.47 | 958.4971 | 0.4 | 480.2560 | 2 | 11.45 | 1259 | 1 | 1366 | 1373 |  |
| total 5 peptides |
| --- |

tr|A0A223G1B9|A0A223G1B9\_SCYSE

back to list

  

| Protein Coverage
| Supporting Peptides
|

Protein Coverage:

Supporting Peptides:

| Peptide | Uniq | -10lgP | Mass | ppm | m/z | z | RT | Scan | #Spec | Start | End | PTM |
| --- | --- | --- | --- | --- | --- | --- | --- | --- | --- | --- | --- | --- |
| H.LENPFWWKDTYGY.H | Y | 38.09 | 1717.7776 | -0.1 | 859.8960 | 2 | 14.01 | 1710 | 1 | 224 | 236 |  |
| H.LENPFWWKDTY.G | Y | 31.27 | 1497.6929 | -1.0 | 749.8530 | 2 | 13.93 | 1698 | 1 | 224 | 234 |  |
| R.GVQPDKRPF.G | Y | 26.32 | 1042.5559 | 0.0 | 522.2852 | 2 | 10.12 | 1005 | 1 | 631 | 639 |  |
| L.ENPFWWKDTY.G | Y | 23.30 | 1384.6088 | 0.4 | 693.3120 | 2 | 13.51 | 1629 | 1 | 225 | 234 |  |
| T.LPRDPAAW.K | Y | 18.29 | 924.4817 | 0.0 | 463.2481 | 2 | 11.66 | 1297 | 1 | 399 | 406 |  |
| total 5 peptides |
| --- |

tr|A0A7R8CX21|A0A7R8CX21\_LEPSM

back to list

  

| Protein Coverage
| Supporting Peptides
|

Protein Coverage:

Supporting Peptides:

| Peptide | Uniq | -10lgP | Mass | ppm | m/z | z | RT | Scan | #Spec | Start | End | PTM |
| --- | --- | --- | --- | --- | --- | --- | --- | --- | --- | --- | --- | --- |
| K.VTVPSIDDKEDMQF.A | N | 49.33 | 1622.7498 | -0.8 | 812.3815 | 2 | 12.56 | 1459 | 1 | 318 | 331 |  |
| K.NKDPLNDTI.V | N | 28.03 | 1028.5138 | -0.3 | 515.2640 | 2 | 11.18 | 1207 | 1 | 545 | 553 |  |
| K.VTVPSIDDKEDMQ.F | N | 21.58 | 1475.6814 | -0.3 | 738.8478 | 2 | 11.23 | 1217 | 1 | 318 | 330 |  |
| total 3 peptides |
| --- |

tr|A0A7T8HL88|A0A7T8HL88\_CALRO

back to list

  

| Protein Coverage
| Supporting Peptides
|

Protein Coverage:

Supporting Peptides:

| Peptide | Uniq | -10lgP | Mass | ppm | m/z | z | RT | Scan | #Spec | Start | End | PTM |
| --- | --- | --- | --- | --- | --- | --- | --- | --- | --- | --- | --- | --- |
| K.VTVPSIDDKEDMQF.A | N | 49.33 | 1622.7498 | -0.8 | 812.3815 | 2 | 12.56 | 1459 | 1 | 318 | 331 |  |
| K.NKDPLNDTI.V | N | 28.03 | 1028.5138 | -0.3 | 515.2640 | 2 | 11.18 | 1207 | 1 | 599 | 607 |  |
| K.VTVPSIDDKEDMQ.F | N | 21.58 | 1475.6814 | -0.3 | 738.8478 | 2 | 11.23 | 1217 | 1 | 318 | 330 |  |
| total 3 peptides |
| --- |

tr|A0A7T8HL29|A0A7T8HL29\_CALRO

back to list

  

| Protein Coverage
| Supporting Peptides
|

Protein Coverage:

Supporting Peptides:

| Peptide | Uniq | -10lgP | Mass | ppm | m/z | z | RT | Scan | #Spec | Start | End | PTM |
| --- | --- | --- | --- | --- | --- | --- | --- | --- | --- | --- | --- | --- |
| K.VTVPSIDDKEDMQF.A | N | 49.33 | 1622.7498 | -0.8 | 812.3815 | 2 | 12.56 | 1459 | 1 | 318 | 331 |  |
| K.NKDPLNDTI.V | N | 28.03 | 1028.5138 | -0.3 | 515.2640 | 2 | 11.18 | 1207 | 1 | 599 | 607 |  |
| K.VTVPSIDDKEDMQ.F | N | 21.58 | 1475.6814 | -0.3 | 738.8478 | 2 | 11.23 | 1217 | 1 | 318 | 330 |  |
| total 3 peptides |
| --- |

tr|A0A0K2V7Y7|A0A0K2V7Y7\_LEPSM

back to list

  

| Protein Coverage
| Supporting Peptides
|

Protein Coverage:

Supporting Peptides:

| Peptide | Uniq | -10lgP | Mass | ppm | m/z | z | RT | Scan | #Spec | Start | End | PTM |
| --- | --- | --- | --- | --- | --- | --- | --- | --- | --- | --- | --- | --- |
| K.VTVPSIDDKEDMQF.A | N | 49.33 | 1622.7498 | -0.8 | 812.3815 | 2 | 12.56 | 1459 | 1 | 318 | 331 |  |
| K.NKDPLNDTI.V | N | 28.03 | 1028.5138 | -0.3 | 515.2640 | 2 | 11.18 | 1207 | 1 | 599 | 607 |  |
| K.VTVPSIDDKEDMQ.F | N | 21.58 | 1475.6814 | -0.3 | 738.8478 | 2 | 11.23 | 1217 | 1 | 318 | 330 |  |
| total 3 peptides |
| --- |

tr|A0A7R8GZZ9|A0A7R8GZZ9\_LEPSM

back to list

  

| Protein Coverage
| Supporting Peptides
|

Protein Coverage:

Supporting Peptides:

| Peptide | Uniq | -10lgP | Mass | ppm | m/z | z | RT | Scan | #Spec | Start | End | PTM |
| --- | --- | --- | --- | --- | --- | --- | --- | --- | --- | --- | --- | --- |
| K.VTVPSIDDKEDMQF.A | N | 49.33 | 1622.7498 | -0.8 | 812.3815 | 2 | 12.56 | 1459 | 1 | 266 | 279 |  |
| K.NKDPLNDTI.V | N | 28.03 | 1028.5138 | -0.3 | 515.2640 | 2 | 11.18 | 1207 | 1 | 548 | 556 |  |
| K.VTVPSIDDKEDMQ.F | N | 21.58 | 1475.6814 | -0.3 | 738.8478 | 2 | 11.23 | 1217 | 1 | 266 | 278 |  |
| total 3 peptides |
| --- |

tr|A0A0K2T3U6|A0A0K2T3U6\_LEPSM

back to list

  

| Protein Coverage
| Supporting Peptides
|

Protein Coverage:

Supporting Peptides:

| Peptide | Uniq | -10lgP | Mass | ppm | m/z | z | RT | Scan | #Spec | Start | End | PTM |
| --- | --- | --- | --- | --- | --- | --- | --- | --- | --- | --- | --- | --- |
| K.VTVPSIDDKEDMQF.A | N | 49.33 | 1622.7498 | -0.8 | 812.3815 | 2 | 12.56 | 1459 | 1 | 318 | 331 |  |
| K.NKDPLNDTI.V | N | 28.03 | 1028.5138 | -0.3 | 515.2640 | 2 | 11.18 | 1207 | 1 | 599 | 607 |  |
| K.VTVPSIDDKEDMQ.F | N | 21.58 | 1475.6814 | -0.3 | 738.8478 | 2 | 11.23 | 1217 | 1 | 318 | 330 |  |
| total 3 peptides |
| --- |

tr|A0A0K2TSJ7|A0A0K2TSJ7\_LEPSM

back to list

  

| Protein Coverage
| Supporting Peptides
|

Protein Coverage:

Supporting Peptides:

| Peptide | Uniq | -10lgP | Mass | ppm | m/z | z | RT | Scan | #Spec | Start | End | PTM |
| --- | --- | --- | --- | --- | --- | --- | --- | --- | --- | --- | --- | --- |
| K.VTVPSIDDKEDMQF.A | N | 49.33 | 1622.7498 | -0.8 | 812.3815 | 2 | 12.56 | 1459 | 1 | 338 | 351 |  |
| K.NKDPLNDTI.V | N | 28.03 | 1028.5138 | -0.3 | 515.2640 | 2 | 11.18 | 1207 | 1 | 619 | 627 |  |
| K.VTVPSIDDKEDMQ.F | N | 21.58 | 1475.6814 | -0.3 | 738.8478 | 2 | 11.23 | 1217 | 1 | 338 | 350 |  |
| total 3 peptides |
| --- |

tr|A0A4Y7MKW9|A0A4Y7MKW9\_9CRUS

back to list

  

| Protein Coverage
| Supporting Peptides
|

Protein Coverage:

Supporting Peptides:

| Peptide | Uniq | -10lgP | Mass | ppm | m/z | z | RT | Scan | #Spec | Start | End | PTM |
| --- | --- | --- | --- | --- | --- | --- | --- | --- | --- | --- | --- | --- |
| M.TGDGVNDAPALK.K | N | 35.45 | 1156.5724 | 3.0 | 579.2952 | 2 | 10.24 | 1027 | 1 | 594 | 605 |  |
| L.GFNPPDLDIM.N | N | 24.56 | 1117.5114 | 0.1 | 559.7630 | 2 | 13.92 | 1697 | 2 | 701 | 710 |  |
| S.VGDKIPADI.R | N | 23.26 | 926.5073 | 0.7 | 464.2612 | 2 | 11.32 | 1233 | 1 | 47 | 55 |  |
| E.VSVGDKIPADI.R | N | 21.19 | 1112.6077 | 0.1 | 557.3112 | 2 | 11.82 | 1328 | 1 | 45 | 55 |  |
| V.KISLPVILL.D | N | 19.28 | 994.6790 | 0.1 | 498.3468 | 2 | 14.71 | 1817 | 1 | 865 | 873 |  |
| V.KISLPVI.L | N | 16.43 | 768.5109 | 0.2 | 385.2628 | 2 | 13.15 | 1567 | 1 | 865 | 871 |  |
| total 6 peptides |
| --- |

tr|A0A0P5KSW7|A0A0P5KSW7\_9CRUS

back to list

  

| Protein Coverage
| Supporting Peptides
|

Protein Coverage:

Supporting Peptides:

| Peptide | Uniq | -10lgP | Mass | ppm | m/z | z | RT | Scan | #Spec | Start | End | PTM |
| --- | --- | --- | --- | --- | --- | --- | --- | --- | --- | --- | --- | --- |
| M.TGDGVNDAPALK.K | N | 35.45 | 1156.5724 | 3.0 | 579.2952 | 2 | 10.24 | 1027 | 1 | 705 | 716 |  |
| L.GFNPPDLDIM.N | N | 24.56 | 1117.5114 | 0.1 | 559.7630 | 2 | 13.92 | 1697 | 2 | 812 | 821 |  |
| S.VGDKIPADI.R | N | 23.26 | 926.5073 | 0.7 | 464.2612 | 2 | 11.32 | 1233 | 1 | 158 | 166 |  |
| E.VSVGDKIPADI.R | N | 21.19 | 1112.6077 | 0.1 | 557.3112 | 2 | 11.82 | 1328 | 1 | 156 | 166 |  |
| V.KISLPVILL.D | N | 19.28 | 994.6790 | 0.1 | 498.3468 | 2 | 14.71 | 1817 | 1 | 976 | 984 |  |
| V.KISLPVI.L | N | 16.43 | 768.5109 | 0.2 | 385.2628 | 2 | 13.15 | 1567 | 1 | 976 | 982 |  |
| total 6 peptides |
| --- |

tr|A0A4Y7MXF9|A0A4Y7MXF9\_9CRUS

back to list

  

| Protein Coverage
| Supporting Peptides
|

Protein Coverage:

Supporting Peptides:

| Peptide | Uniq | -10lgP | Mass | ppm | m/z | z | RT | Scan | #Spec | Start | End | PTM |
| --- | --- | --- | --- | --- | --- | --- | --- | --- | --- | --- | --- | --- |
| M.TGDGVNDAPALK.K | N | 35.45 | 1156.5724 | 3.0 | 579.2952 | 2 | 10.24 | 1027 | 1 | 705 | 716 |  |
| L.GFNPPDLDIM.N | N | 24.56 | 1117.5114 | 0.1 | 559.7630 | 2 | 13.92 | 1697 | 2 | 812 | 821 |  |
| S.VGDKIPADI.R | N | 23.26 | 926.5073 | 0.7 | 464.2612 | 2 | 11.32 | 1233 | 1 | 158 | 166 |  |
| E.VSVGDKIPADI.R | N | 21.19 | 1112.6077 | 0.1 | 557.3112 | 2 | 11.82 | 1328 | 1 | 156 | 166 |  |
| I.KISLPVILL.D | N | 19.28 | 994.6790 | 0.1 | 498.3468 | 2 | 14.71 | 1817 | 1 | 976 | 984 |  |
| I.KISLPVI.L | N | 16.43 | 768.5109 | 0.2 | 385.2628 | 2 | 13.15 | 1567 | 1 | 976 | 982 |  |
| total 6 peptides |
| --- |

tr|A0A0P4ZGF4|A0A0P4ZGF4\_9CRUS

back to list

  

| Protein Coverage
| Supporting Peptides
|

Protein Coverage:

Supporting Peptides:

| Peptide | Uniq | -10lgP | Mass | ppm | m/z | z | RT | Scan | #Spec | Start | End | PTM |
| --- | --- | --- | --- | --- | --- | --- | --- | --- | --- | --- | --- | --- |
| M.TGDGVNDAPALK.K | N | 35.45 | 1156.5724 | 3.0 | 579.2952 | 2 | 10.24 | 1027 | 1 | 705 | 716 |  |
| L.GFNPPDLDIM.N | N | 24.56 | 1117.5114 | 0.1 | 559.7630 | 2 | 13.92 | 1697 | 2 | 812 | 821 |  |
| S.VGDKIPADI.R | N | 23.26 | 926.5073 | 0.7 | 464.2612 | 2 | 11.32 | 1233 | 1 | 158 | 166 |  |
| E.VSVGDKIPADI.R | N | 21.19 | 1112.6077 | 0.1 | 557.3112 | 2 | 11.82 | 1328 | 1 | 156 | 166 |  |
| V.KISLPVILL.D | N | 19.28 | 994.6790 | 0.1 | 498.3468 | 2 | 14.71 | 1817 | 1 | 976 | 984 |  |
| V.KISLPVI.L | N | 16.43 | 768.5109 | 0.2 | 385.2628 | 2 | 13.15 | 1567 | 1 | 976 | 982 |  |
| total 6 peptides |
| --- |

tr|A0A4Y7MVM2|A0A4Y7MVM2\_DAPPU

back to list

  

| Protein Coverage
| Supporting Peptides
|

Protein Coverage:

Supporting Peptides:

| Peptide | Uniq | -10lgP | Mass | ppm | m/z | z | RT | Scan | #Spec | Start | End | PTM |
| --- | --- | --- | --- | --- | --- | --- | --- | --- | --- | --- | --- | --- |
| M.TGDGVNDAPALK.K | N | 35.45 | 1156.5724 | 3.0 | 579.2952 | 2 | 10.24 | 1027 | 1 | 705 | 716 |  |
| L.GFNPPDLDIM.N | N | 24.56 | 1117.5114 | 0.1 | 559.7630 | 2 | 13.92 | 1697 | 2 | 812 | 821 |  |
| S.VGDKIPADI.R | N | 23.26 | 926.5073 | 0.7 | 464.2612 | 2 | 11.32 | 1233 | 1 | 158 | 166 |  |
| E.VSVGDKIPADI.R | N | 21.19 | 1112.6077 | 0.1 | 557.3112 | 2 | 11.82 | 1328 | 1 | 156 | 166 |  |
| V.KISLPVILL.D | N | 19.28 | 994.6790 | 0.1 | 498.3468 | 2 | 14.71 | 1817 | 1 | 976 | 984 |  |
| V.KISLPVI.L | N | 16.43 | 768.5109 | 0.2 | 385.2628 | 2 | 13.15 | 1567 | 1 | 976 | 982 |  |
| total 6 peptides |
| --- |

tr|A0A4Y7M702|A0A4Y7M702\_9CRUS

back to list

  

| Protein Coverage
| Supporting Peptides
|

Protein Coverage:

Supporting Peptides:

| Peptide | Uniq | -10lgP | Mass | ppm | m/z | z | RT | Scan | #Spec | Start | End | PTM |
| --- | --- | --- | --- | --- | --- | --- | --- | --- | --- | --- | --- | --- |
| M.TGDGVNDAPALK.K | N | 35.45 | 1156.5724 | 3.0 | 579.2952 | 2 | 10.24 | 1027 | 1 | 705 | 716 |  |
| L.GFNPPDLDIM.N | N | 24.56 | 1117.5114 | 0.1 | 559.7630 | 2 | 13.92 | 1697 | 2 | 812 | 821 |  |
| S.VGDKIPADI.R | N | 23.26 | 926.5073 | 0.7 | 464.2612 | 2 | 11.32 | 1233 | 1 | 158 | 166 |  |
| E.VSVGDKIPADI.R | N | 21.19 | 1112.6077 | 0.1 | 557.3112 | 2 | 11.82 | 1328 | 1 | 156 | 166 |  |
| I.KISLPVILL.D | N | 19.28 | 994.6790 | 0.1 | 498.3468 | 2 | 14.71 | 1817 | 1 | 976 | 984 |  |
| I.KISLPVI.L | N | 16.43 | 768.5109 | 0.2 | 385.2628 | 2 | 13.15 | 1567 | 1 | 976 | 982 |  |
| total 6 peptides |
| --- |

tr|A0A4Y7MHQ5|A0A4Y7MHQ5\_9CRUS

back to list

  

| Protein Coverage
| Supporting Peptides
|

Protein Coverage:

Supporting Peptides:

| Peptide | Uniq | -10lgP | Mass | ppm | m/z | z | RT | Scan | #Spec | Start | End | PTM |
| --- | --- | --- | --- | --- | --- | --- | --- | --- | --- | --- | --- | --- |
| M.TGDGVNDAPALK.K | N | 35.45 | 1156.5724 | 3.0 | 579.2952 | 2 | 10.24 | 1027 | 1 | 705 | 716 |  |
| L.GFNPPDLDIM.N | N | 24.56 | 1117.5114 | 0.1 | 559.7630 | 2 | 13.92 | 1697 | 2 | 812 | 821 |  |
| S.VGDKIPADI.R | N | 23.26 | 926.5073 | 0.7 | 464.2612 | 2 | 11.32 | 1233 | 1 | 158 | 166 |  |
| E.VSVGDKIPADI.R | N | 21.19 | 1112.6077 | 0.1 | 557.3112 | 2 | 11.82 | 1328 | 1 | 156 | 166 |  |
| V.KISLPVILL.D | N | 19.28 | 994.6790 | 0.1 | 498.3468 | 2 | 14.71 | 1817 | 1 | 976 | 984 |  |
| V.KISLPVI.L | N | 16.43 | 768.5109 | 0.2 | 385.2628 | 2 | 13.15 | 1567 | 1 | 976 | 982 |  |
| total 6 peptides |
| --- |

tr|A0A4Y7ME47|A0A4Y7ME47\_9CRUS

back to list

  

| Protein Coverage
| Supporting Peptides
|

Protein Coverage:

Supporting Peptides:

| Peptide | Uniq | -10lgP | Mass | ppm | m/z | z | RT | Scan | #Spec | Start | End | PTM |
| --- | --- | --- | --- | --- | --- | --- | --- | --- | --- | --- | --- | --- |
| M.TGDGVNDAPALK.K | N | 35.45 | 1156.5724 | 3.0 | 579.2952 | 2 | 10.24 | 1027 | 1 | 705 | 716 |  |
| L.GFNPPDLDIM.N | N | 24.56 | 1117.5114 | 0.1 | 559.7630 | 2 | 13.92 | 1697 | 2 | 812 | 821 |  |
| S.VGDKIPADI.R | N | 23.26 | 926.5073 | 0.7 | 464.2612 | 2 | 11.32 | 1233 | 1 | 158 | 166 |  |
| E.VSVGDKIPADI.R | N | 21.19 | 1112.6077 | 0.1 | 557.3112 | 2 | 11.82 | 1328 | 1 | 156 | 166 |  |
| V.KISLPVILL.D | N | 19.28 | 994.6790 | 0.1 | 498.3468 | 2 | 14.71 | 1817 | 1 | 976 | 984 |  |
| V.KISLPVI.L | N | 16.43 | 768.5109 | 0.2 | 385.2628 | 2 | 13.15 | 1567 | 1 | 976 | 982 |  |
| total 6 peptides |
| --- |

tr|A0A4Y7M4I0|A0A4Y7M4I0\_9CRUS

back to list

  

| Protein Coverage
| Supporting Peptides
|

Protein Coverage:

Supporting Peptides:

| Peptide | Uniq | -10lgP | Mass | ppm | m/z | z | RT | Scan | #Spec | Start | End | PTM |
| --- | --- | --- | --- | --- | --- | --- | --- | --- | --- | --- | --- | --- |
| M.TGDGVNDAPALK.K | N | 35.45 | 1156.5724 | 3.0 | 579.2952 | 2 | 10.24 | 1027 | 1 | 705 | 716 |  |
| L.GFNPPDLDIM.N | N | 24.56 | 1117.5114 | 0.1 | 559.7630 | 2 | 13.92 | 1697 | 2 | 812 | 821 |  |
| S.VGDKIPADI.R | N | 23.26 | 926.5073 | 0.7 | 464.2612 | 2 | 11.32 | 1233 | 1 | 158 | 166 |  |
| E.VSVGDKIPADI.R | N | 21.19 | 1112.6077 | 0.1 | 557.3112 | 2 | 11.82 | 1328 | 1 | 156 | 166 |  |
| V.KISLPVILL.D | N | 19.28 | 994.6790 | 0.1 | 498.3468 | 2 | 14.71 | 1817 | 1 | 976 | 984 |  |
| V.KISLPVI.L | N | 16.43 | 768.5109 | 0.2 | 385.2628 | 2 | 13.15 | 1567 | 1 | 976 | 982 |  |
| total 6 peptides |
| --- |

tr|A0A1D2MC18|A0A1D2MC18\_ORCCI

back to list

  

| Protein Coverage
| Supporting Peptides
|

Protein Coverage:

Supporting Peptides:

| Peptide | Uniq | -10lgP | Mass | ppm | m/z | z | RT | Scan | #Spec | Start | End | PTM |
| --- | --- | --- | --- | --- | --- | --- | --- | --- | --- | --- | --- | --- |
| M.TGDGVNDAPALK.K | N | 35.45 | 1156.5724 | 3.0 | 579.2952 | 2 | 10.24 | 1027 | 1 | 702 | 713 |  |
| L.GFNPPDLDIM.T | N | 24.56 | 1117.5114 | 0.1 | 559.7630 | 2 | 13.92 | 1697 | 2 | 809 | 818 |  |
| S.VGDKIPADI.R | N | 23.26 | 926.5073 | 0.7 | 464.2612 | 2 | 11.32 | 1233 | 1 | 159 | 167 |  |
| E.VSVGDKIPADI.R | N | 21.19 | 1112.6077 | 0.1 | 557.3112 | 2 | 11.82 | 1328 | 1 | 157 | 167 |  |
| M.KISLPVILL.D | N | 19.28 | 994.6790 | 0.1 | 498.3468 | 2 | 14.71 | 1817 | 1 | 973 | 981 |  |
| M.KISLPVI.L | N | 16.43 | 768.5109 | 0.2 | 385.2628 | 2 | 13.15 | 1567 | 1 | 973 | 979 |  |
| total 6 peptides |
| --- |

tr|E9HR84|E9HR84\_DAPPU

back to list

  

| Protein Coverage
| Supporting Peptides
|

Protein Coverage:

Supporting Peptides:

| Peptide | Uniq | -10lgP | Mass | ppm | m/z | z | RT | Scan | #Spec | Start | End | PTM |
| --- | --- | --- | --- | --- | --- | --- | --- | --- | --- | --- | --- | --- |
| M.TGDGVNDAPALK.K | N | 35.45 | 1156.5724 | 3.0 | 579.2952 | 2 | 10.24 | 1027 | 1 | 705 | 716 |  |
| L.GFNPPDLDIM.N | N | 24.56 | 1117.5114 | 0.1 | 559.7630 | 2 | 13.92 | 1697 | 2 | 812 | 821 |  |
| S.VGDKIPADI.R | N | 23.26 | 926.5073 | 0.7 | 464.2612 | 2 | 11.32 | 1233 | 1 | 158 | 166 |  |
| E.VSVGDKIPADI.R | N | 21.19 | 1112.6077 | 0.1 | 557.3112 | 2 | 11.82 | 1328 | 1 | 156 | 166 |  |
| V.KISLPVILL.D | N | 19.28 | 994.6790 | 0.1 | 498.3468 | 2 | 14.71 | 1817 | 1 | 976 | 984 |  |
| V.KISLPVI.L | N | 16.43 | 768.5109 | 0.2 | 385.2628 | 2 | 13.15 | 1567 | 1 | 976 | 982 |  |
| total 6 peptides |
| --- |

tr|A0A0P5VJX3|A0A0P5VJX3\_9CRUS

back to list

  

| Protein Coverage
| Supporting Peptides
|

Protein Coverage:

Supporting Peptides:

| Peptide | Uniq | -10lgP | Mass | ppm | m/z | z | RT | Scan | #Spec | Start | End | PTM |
| --- | --- | --- | --- | --- | --- | --- | --- | --- | --- | --- | --- | --- |
| M.TGDGVNDAPALK.K | N | 35.45 | 1156.5724 | 3.0 | 579.2952 | 2 | 10.24 | 1027 | 1 | 705 | 716 |  |
| L.GFNPPDLDIM.N | N | 24.56 | 1117.5114 | 0.1 | 559.7630 | 2 | 13.92 | 1697 | 2 | 812 | 821 |  |
| S.VGDKIPADI.R | N | 23.26 | 926.5073 | 0.7 | 464.2612 | 2 | 11.32 | 1233 | 1 | 158 | 166 |  |
| E.VSVGDKIPADI.R | N | 21.19 | 1112.6077 | 0.1 | 557.3112 | 2 | 11.82 | 1328 | 1 | 156 | 166 |  |
| V.KISLPVILL.D | N | 19.28 | 994.6790 | 0.1 | 498.3468 | 2 | 14.71 | 1817 | 1 | 976 | 984 |  |
| V.KISLPVI.L | N | 16.43 | 768.5109 | 0.2 | 385.2628 | 2 | 13.15 | 1567 | 1 | 976 | 982 |  |
| total 6 peptides |
| --- |

tr|A0A4Y7LYL4|A0A4Y7LYL4\_9CRUS

back to list

  

| Protein Coverage
| Supporting Peptides
|

Protein Coverage:

Supporting Peptides:

| Peptide | Uniq | -10lgP | Mass | ppm | m/z | z | RT | Scan | #Spec | Start | End | PTM |
| --- | --- | --- | --- | --- | --- | --- | --- | --- | --- | --- | --- | --- |
| M.TGDGVNDAPALK.K | N | 35.45 | 1156.5724 | 3.0 | 579.2952 | 2 | 10.24 | 1027 | 1 | 705 | 716 |  |
| L.GFNPPDLDIM.N | N | 24.56 | 1117.5114 | 0.1 | 559.7630 | 2 | 13.92 | 1697 | 2 | 812 | 821 |  |
| S.VGDKIPADI.R | N | 23.26 | 926.5073 | 0.7 | 464.2612 | 2 | 11.32 | 1233 | 1 | 158 | 166 |  |
| E.VSVGDKIPADI.R | N | 21.19 | 1112.6077 | 0.1 | 557.3112 | 2 | 11.82 | 1328 | 1 | 156 | 166 |  |
| V.KISLPVILL.D | N | 19.28 | 994.6790 | 0.1 | 498.3468 | 2 | 14.71 | 1817 | 1 | 976 | 984 |  |
| V.KISLPVI.L | N | 16.43 | 768.5109 | 0.2 | 385.2628 | 2 | 13.15 | 1567 | 1 | 976 | 982 |  |
| total 6 peptides |
| --- |

tr|A0A0C5E1J4|A0A0C5E1J4\_CHEDE

back to list

  

| Protein Coverage
| Supporting Peptides
|

Protein Coverage:

Supporting Peptides:

| Peptide | Uniq | -10lgP | Mass | ppm | m/z | z | RT | Scan | #Spec | Start | End | PTM |
| --- | --- | --- | --- | --- | --- | --- | --- | --- | --- | --- | --- | --- |
| M.TGDGVNDAPALK.K | N | 35.45 | 1156.5724 | 3.0 | 579.2952 | 2 | 10.24 | 1027 | 1 | 701 | 712 |  |
| L.GFNPPDLDIM.D | N | 24.56 | 1117.5114 | 0.1 | 559.7630 | 2 | 13.92 | 1697 | 2 | 808 | 817 |  |
| L.FSRVEPF.H | N | 24.00 | 880.4443 | 0.0 | 441.2294 | 2 | 11.77 | 1319 | 1 | 676 | 682 |  |
| S.VGDKIPADI.R | N | 23.26 | 926.5073 | 0.7 | 464.2612 | 2 | 11.32 | 1233 | 1 | 155 | 163 |  |
| E.VSVGDKIPADI.R | N | 21.19 | 1112.6077 | 0.1 | 557.3112 | 2 | 11.82 | 1328 | 1 | 153 | 163 |  |
| total 5 peptides |
| --- |

tr|A0A0N9EJJ5|A0A0N9EJJ5\_ERIVE

back to list

  

| Protein Coverage
| Supporting Peptides
|

Protein Coverage:

Supporting Peptides:

| Peptide | Uniq | -10lgP | Mass | ppm | m/z | z | RT | Scan | #Spec | Start | End | PTM |
| --- | --- | --- | --- | --- | --- | --- | --- | --- | --- | --- | --- | --- |
| H.VVLPPLYEVTPH.L | Y | 34.71 | 1362.7546 | 0.3 | 682.3848 | 2 | 13.33 | 1598 | 1 | 153 | 164 |  |
| N.VHFEDVDGVAR.V | Y | 27.30 | 1242.5992 | 0.8 | 622.3074 | 2 | 10.75 | 1125 | 1 | 297 | 307 |  |
| H.VVLPPLYE.V | Y | 25.66 | 928.5269 | 0.0 | 465.2707 | 2 | 13.61 | 1646 | 1 | 153 | 160 |  |
| A.TRDPAFF.R | Y | 17.22 | 852.4130 | 0.2 | 427.2138 | 2 | 12.10 | 1379 | 1 | 397 | 403 |  |
| total 4 peptides |
| --- |

tr|A0A2Z4I2U3|A0A2Z4I2U3\_CALSI

back to list

  

| Protein Coverage
| Supporting Peptides
|

Protein Coverage:

Supporting Peptides:

| Peptide | Uniq | -10lgP | Mass | ppm | m/z | z | RT | Scan | #Spec | Start | End | PTM |
| --- | --- | --- | --- | --- | --- | --- | --- | --- | --- | --- | --- | --- |
| L.GFNPPDLDIM.E | N | 24.56 | 1117.5114 | 0.1 | 559.7630 | 2 | 13.92 | 1697 | 2 | 808 | 817 |  |
| L.FSRVEPF.H | N | 24.00 | 880.4443 | 0.0 | 441.2294 | 2 | 11.77 | 1319 | 1 | 676 | 682 |  |
| S.VGDKIPADL.R | N | 23.26 | 926.5073 | 0.7 | 464.2612 | 2 | 11.32 | 1233 | 1 | 155 | 163 |  |
| E.VSVGDKIPADL.R | N | 21.19 | 1112.6077 | 0.1 | 557.3112 | 2 | 11.82 | 1328 | 1 | 153 | 163 |  |
| L.KISLPVLLL.D | N | 19.28 | 994.6790 | 0.1 | 498.3468 | 2 | 14.71 | 1817 | 1 | 972 | 980 |  |
| L.DESLKFL.A | Y | 16.75 | 850.4436 | 0.2 | 426.2292 | 2 | 12.91 | 1523 | 1 | 981 | 987 |  |
| L.KISLPVL.L | N | 16.43 | 768.5109 | 0.2 | 385.2628 | 2 | 13.15 | 1567 | 1 | 972 | 978 |  |
| total 7 peptides |
| --- |

tr|A0A0P4W579|A0A0P4W579\_SCYOL

back to list

  

| Protein Coverage
| Supporting Peptides
|

Protein Coverage:

Supporting Peptides:

| Peptide | Uniq | -10lgP | Mass | ppm | m/z | z | RT | Scan | #Spec | Start | End | PTM |
| --- | --- | --- | --- | --- | --- | --- | --- | --- | --- | --- | --- | --- |
| K.GPAPVLPKPTPLPN.L | Y | 42.82 | 1396.8077 | 0.6 | 699.4116 | 2 | 11.62 | 1290 | 1 | 294 | 307 |  |
| N.MDPIIVKPG.A | Y | 31.34 | 968.5364 | 0.2 | 485.2756 | 2 | 11.44 | 1256 | 1 | 109 | 117 |  |
| total 2 peptides |
| --- |

tr|A0A0P4WKI4|A0A0P4WKI4\_SCYOL

back to list

  

| Protein Coverage
| Supporting Peptides
|

Protein Coverage:

Supporting Peptides:

| Peptide | Uniq | -10lgP | Mass | ppm | m/z | z | RT | Scan | #Spec | Start | End | PTM |
| --- | --- | --- | --- | --- | --- | --- | --- | --- | --- | --- | --- | --- |
| K.GPAPVLPKPTPLPN.L | Y | 42.82 | 1396.8077 | 0.6 | 699.4116 | 2 | 11.62 | 1290 | 1 | 668 | 681 |  |
| N.MDPIIVKPG.A | Y | 31.34 | 968.5364 | 0.2 | 485.2756 | 2 | 11.44 | 1256 | 1 | 483 | 491 |  |
| total 2 peptides |
| --- |

tr|A0A0P4WBU2|A0A0P4WBU2\_SCYOL

back to list

  

| Protein Coverage
| Supporting Peptides
|

Protein Coverage:

Supporting Peptides:

| Peptide | Uniq | -10lgP | Mass | ppm | m/z | z | RT | Scan | #Spec | Start | End | PTM |
| --- | --- | --- | --- | --- | --- | --- | --- | --- | --- | --- | --- | --- |
| K.GPAPVLPKPTPLPN.L | Y | 42.82 | 1396.8077 | 0.6 | 699.4116 | 2 | 11.62 | 1290 | 1 | 668 | 681 |  |
| N.MDPIIVKPG.A | Y | 31.34 | 968.5364 | 0.2 | 485.2756 | 2 | 11.44 | 1256 | 1 | 483 | 491 |  |
| total 2 peptides |
| --- |

tr|A0A3R7PTG0|A0A3R7PTG0\_PENVA

back to list

  

| Protein Coverage
| Supporting Peptides
|

Protein Coverage:

Supporting Peptides:

| Peptide | Uniq | -10lgP | Mass | ppm | m/z | z | RT | Scan | #Spec | Start | End | PTM |
| --- | --- | --- | --- | --- | --- | --- | --- | --- | --- | --- | --- | --- |
| A.DAPGPINF.T | Y | 35.72 | 829.3970 | 0.2 | 415.7058 | 2 | 12.89 | 1518 | 1 | 73 | 80 |  |
| A.FGDKFT.N | Y | 27.18 | 713.3384 | -0.3 | 357.6764 | 2 | 10.67 | 1109 | 1 | 127 | 132 |  |
| total 2 peptides |
| --- |

tr|A0A195D1T6|A0A195D1T6\_9HYME

back to list

  

| Protein Coverage
| Supporting Peptides
|

Protein Coverage:

Supporting Peptides:

| Peptide | Uniq | -10lgP | Mass | ppm | m/z | z | RT | Scan | #Spec | Start | End | PTM |
| --- | --- | --- | --- | --- | --- | --- | --- | --- | --- | --- | --- | --- |
| E.VIVVDKPGPPQGPL.Q | Y | 38.18 | 1414.8184 | -0.2 | 708.4163 | 2 | 11.78 | 1320 | 1 | 6879 | 6892 |  |
| K.NWDKDFVDL.E | Y | 18.76 | 1150.5294 | 0.7 | 576.2724 | 2 | 13.28 | 1589 | 1 | 4336 | 4344 |  |
| total 2 peptides |
| --- |

tr|A0A151I5T2|A0A151I5T2\_9HYME

back to list

  

| Protein Coverage
| Supporting Peptides
|

Protein Coverage:

Supporting Peptides:

| Peptide | Uniq | -10lgP | Mass | ppm | m/z | z | RT | Scan | #Spec | Start | End | PTM |
| --- | --- | --- | --- | --- | --- | --- | --- | --- | --- | --- | --- | --- |
| E.VIVVDKPGPPQGPL.Q | Y | 38.18 | 1414.8184 | -0.2 | 708.4163 | 2 | 11.78 | 1320 | 1 | 6775 | 6788 |  |
| K.NWDKDFVDL.E | Y | 18.76 | 1150.5294 | 0.7 | 576.2724 | 2 | 13.28 | 1589 | 1 | 4232 | 4240 |  |
| total 2 peptides |
| --- |

tr|A0A151J500|A0A151J500\_9HYME

back to list

  

| Protein Coverage
| Supporting Peptides
|

Protein Coverage:

Supporting Peptides:

| Peptide | Uniq | -10lgP | Mass | ppm | m/z | z | RT | Scan | #Spec | Start | End | PTM |
| --- | --- | --- | --- | --- | --- | --- | --- | --- | --- | --- | --- | --- |
| E.VIVVDKPGPPQGPL.Q | Y | 38.18 | 1414.8184 | -0.2 | 708.4163 | 2 | 11.78 | 1320 | 1 | 6901 | 6914 |  |
| K.NWDKDFVDL.E | Y | 18.76 | 1150.5294 | 0.7 | 576.2724 | 2 | 13.28 | 1589 | 1 | 4358 | 4366 |  |
| total 2 peptides |
| --- |

tr|E2BUG0|E2BUG0\_HARSA

back to list

  

| Protein Coverage
| Supporting Peptides
|

Protein Coverage:

Supporting Peptides:

| Peptide | Uniq | -10lgP | Mass | ppm | m/z | z | RT | Scan | #Spec | Start | End | PTM |
| --- | --- | --- | --- | --- | --- | --- | --- | --- | --- | --- | --- | --- |
| E.VIVVDKPGPPQGPL.Q | Y | 38.18 | 1414.8184 | -0.2 | 708.4163 | 2 | 11.78 | 1320 | 1 | 3242 | 3255 |  |
| K.NWDKDFVDL.E | Y | 18.76 | 1150.5294 | 0.7 | 576.2724 | 2 | 13.28 | 1589 | 1 | 1187 | 1195 |  |
| total 2 peptides |
| --- |

tr|A0A5N5SKQ0|A0A5N5SKQ0\_9CRUS

back to list

  

| Protein Coverage
| Supporting Peptides
|

Protein Coverage:

Supporting Peptides:

| Peptide | Uniq | -10lgP | Mass | ppm | m/z | z | RT | Scan | #Spec | Start | End | PTM |
| --- | --- | --- | --- | --- | --- | --- | --- | --- | --- | --- | --- | --- |
| D.GIHIPGSPF.R | Y | 27.98 | 923.4865 | 0.1 | 462.7505 | 2 | 12.99 | 1537 | 1 | 696 | 704 |  |
| G.YKPTEPGYY.I | Y | 21.97 | 1116.5127 | 1.3 | 559.2643 | 2 | 10.73 | 1121 | 1 | 390 | 398 |  |
| E.MHIPGSPF.Q | Y | 21.02 | 884.4214 | 0.1 | 443.2180 | 2 | 12.65 | 1476 | 1 | 505 | 512 |  |
| total 3 peptides |
| --- |

tr|A0A444SHR5|A0A444SHR5\_ARMVU

back to list

  

| Protein Coverage
| Supporting Peptides
|

Protein Coverage:

Supporting Peptides:

| Peptide | Uniq | -10lgP | Mass | ppm | m/z | z | RT | Scan | #Spec | Start | End | PTM |
| --- | --- | --- | --- | --- | --- | --- | --- | --- | --- | --- | --- | --- |
| D.GIHIPGSPF.R | Y | 27.98 | 923.4865 | 0.1 | 462.7505 | 2 | 12.99 | 1537 | 1 | 735 | 743 |  |
| G.YKPTEPGYY.I | Y | 21.97 | 1116.5127 | 1.3 | 559.2643 | 2 | 10.73 | 1121 | 1 | 429 | 437 |  |
| E.MHIPGSPF.Q | Y | 21.02 | 884.4214 | 0.1 | 443.2180 | 2 | 12.65 | 1476 | 1 | 544 | 551 |  |
| total 3 peptides |
| --- |

tr|A0A3R7Q393|A0A3R7Q393\_PENVA

back to list

  

| Protein Coverage
| Supporting Peptides
|

Protein Coverage:

Supporting Peptides:

| Peptide | Uniq | -10lgP | Mass | ppm | m/z | z | RT | Scan | #Spec | Start | End | PTM |
| --- | --- | --- | --- | --- | --- | --- | --- | --- | --- | --- | --- | --- |
| N.GIHIPGSPF.R | Y | 27.98 | 923.4865 | 0.1 | 462.7505 | 2 | 12.99 | 1537 | 1 | 685 | 693 |  |
| G.YKPTEPGYY.I | Y | 21.97 | 1116.5127 | 1.3 | 559.2643 | 2 | 10.73 | 1121 | 1 | 377 | 385 |  |
| D.MHIPGSPF.Q | Y | 21.02 | 884.4214 | 0.1 | 443.2180 | 2 | 12.65 | 1476 | 1 | 492 | 499 |  |
| total 3 peptides |
| --- |

tr|A0A2P1JJ42|A0A2P1JJ42\_PROCL

back to list

  

| Protein Coverage
| Supporting Peptides
|

Protein Coverage:

Supporting Peptides:

| Peptide | Uniq | -10lgP | Mass | ppm | m/z | z | RT | Scan | #Spec | Start | End | PTM |
| --- | --- | --- | --- | --- | --- | --- | --- | --- | --- | --- | --- | --- |
| N.GIHIPGSPF.R | Y | 27.98 | 923.4865 | 0.1 | 462.7505 | 2 | 12.99 | 1537 | 1 | 1330 | 1338 |  |
| G.YKPTEPGYY.I | Y | 21.97 | 1116.5127 | 1.3 | 559.2643 | 2 | 10.73 | 1121 | 1 | 1026 | 1034 |  |
| E.MHIPGSPF.Q | Y | 21.02 | 884.4214 | 0.1 | 443.2180 | 2 | 12.65 | 1476 | 1 | 1141 | 1148 |  |
| total 3 peptides |
| --- |

tr|A0A834J309|A0A834J309\_RHYFE

back to list

  

| Protein Coverage
| Supporting Peptides
|

Protein Coverage:

Supporting Peptides:

| Peptide | Uniq | -10lgP | Mass | ppm | m/z | z | RT | Scan | #Spec | Start | End | PTM |
| --- | --- | --- | --- | --- | --- | --- | --- | --- | --- | --- | --- | --- |
| L.AWRPPLDDGGSPITNY.I | Y | 39.43 | 1757.8373 | 0.3 | 879.9261 | 2 | 12.74 | 1491 | 1 | 6856 | 6871 |  |
| total 1 peptides |
| --- |

tr|A0A6M2D681|A0A6M2D681\_RHIMP

back to list

  

| Protein Coverage
| Supporting Peptides
|

Protein Coverage:

Supporting Peptides:

| Peptide | Uniq | -10lgP | Mass | ppm | m/z | z | RT | Scan | #Spec | Start | End | PTM |
| --- | --- | --- | --- | --- | --- | --- | --- | --- | --- | --- | --- | --- |
| Y.LVLALVVLMAVAQ.S | Y | 31.83 | 1338.8308 | -13.7 | 670.4135 | 2 | 13.49 | 1626 | 2 | 6 | 18 |  |
| total 1 peptides |
| --- |

tr|A0A6G5A756|A0A6G5A756\_RHIMP

back to list

  

| Protein Coverage
| Supporting Peptides
|

Protein Coverage:

Supporting Peptides:

| Peptide | Uniq | -10lgP | Mass | ppm | m/z | z | RT | Scan | #Spec | Start | End | PTM |
| --- | --- | --- | --- | --- | --- | --- | --- | --- | --- | --- | --- | --- |
| Y.LVLALVVLMAVAQ.S | Y | 31.83 | 1338.8308 | -13.7 | 670.4135 | 2 | 13.49 | 1626 | 2 | 6 | 18 |  |
| total 1 peptides |
| --- |

tr|A0A2J7R8E8|A0A2J7R8E8\_9NEOP

back to list

  

| Protein Coverage
| Supporting Peptides
|

Protein Coverage:

Supporting Peptides:

| Peptide | Uniq | -10lgP | Mass | ppm | m/z | z | RT | Scan | #Spec | Start | End | PTM |
| --- | --- | --- | --- | --- | --- | --- | --- | --- | --- | --- | --- | --- |
| H.MQQLMKSGLPPEPK.R | Y | 28.61 | 1582.8210 | -7.1 | 792.4122 | 2 | 12.31 | 1415 | 1 | 85 | 98 |  |
| total 1 peptides |
| --- |

tr|A0A423SLW8|A0A423SLW8\_PENVA

back to list

  

| Protein Coverage
| Supporting Peptides
|

Protein Coverage:

Supporting Peptides:

| Peptide | Uniq | -10lgP | Mass | ppm | m/z | z | RT | Scan | #Spec | Start | End | PTM |
| --- | --- | --- | --- | --- | --- | --- | --- | --- | --- | --- | --- | --- |
| T.IVKDLPIDDF.A | Y | 28.36 | 1173.6281 | 0.3 | 587.8215 | 2 | 13.13 | 1562 | 1 | 194 | 203 |  |
| total 1 peptides |
| --- |

tr|A0A5B7CL07|A0A5B7CL07\_PORTR

back to list

  

| Protein Coverage
| Supporting Peptides
|

Protein Coverage:

Supporting Peptides:

| Peptide | Uniq | -10lgP | Mass | ppm | m/z | z | RT | Scan | #Spec | Start | End | PTM |
| --- | --- | --- | --- | --- | --- | --- | --- | --- | --- | --- | --- | --- |
| T.IVKDLPIDDF.A | Y | 28.36 | 1173.6281 | 0.3 | 587.8215 | 2 | 13.13 | 1562 | 1 | 247 | 256 |  |
| total 1 peptides |
| --- |

tr|A0A0P4WLG3|A0A0P4WLG3\_SCYOL

back to list

  

| Protein Coverage
| Supporting Peptides
|

Protein Coverage:

Supporting Peptides:

| Peptide | Uniq | -10lgP | Mass | ppm | m/z | z | RT | Scan | #Spec | Start | End | PTM |
| --- | --- | --- | --- | --- | --- | --- | --- | --- | --- | --- | --- | --- |
| S.IVKDLPIDDF.A | Y | 28.36 | 1173.6281 | 0.3 | 587.8215 | 2 | 13.13 | 1562 | 1 | 298 | 307 |  |
| total 1 peptides |
| --- |

tr|A0A1W4VNV5|A0A1W4VNV5\_DROFC

back to list

  

| Protein Coverage
| Supporting Peptides
|

Protein Coverage:

Supporting Peptides:

| Peptide | Uniq | -10lgP | Mass | ppm | m/z | z | RT | Scan | #Spec | Start | End | PTM |
| --- | --- | --- | --- | --- | --- | --- | --- | --- | --- | --- | --- | --- |
| D.EDLSGVVLL.T | Y | 26.84 | 943.5226 | 11.7 | 472.7741 | 2 | 13.85 | 1685 | 1 | 673 | 681 |  |
| total 1 peptides |
| --- |

tr|A0A1W4W0Y9|A0A1W4W0Y9\_DROFC

back to list

  

| Protein Coverage
| Supporting Peptides
|

Protein Coverage:

Supporting Peptides:

| Peptide | Uniq | -10lgP | Mass | ppm | m/z | z | RT | Scan | #Spec | Start | End | PTM |
| --- | --- | --- | --- | --- | --- | --- | --- | --- | --- | --- | --- | --- |
| D.EDLSGVVLL.T | Y | 26.84 | 943.5226 | 11.7 | 472.7741 | 2 | 13.85 | 1685 | 1 | 673 | 681 |  |
| total 1 peptides |
| --- |

tr|A0A1W4W054|A0A1W4W054\_DROFC

back to list

  

| Protein Coverage
| Supporting Peptides
|

Protein Coverage:

Supporting Peptides:

| Peptide | Uniq | -10lgP | Mass | ppm | m/z | z | RT | Scan | #Spec | Start | End | PTM |
| --- | --- | --- | --- | --- | --- | --- | --- | --- | --- | --- | --- | --- |
| D.EDLSGVVLL.T | Y | 26.84 | 943.5226 | 11.7 | 472.7741 | 2 | 13.85 | 1685 | 1 | 673 | 681 |  |
| total 1 peptides |
| --- |

tr|A0A1W4VNL1|A0A1W4VNL1\_DROFC

back to list

  

| Protein Coverage
| Supporting Peptides
|

Protein Coverage:

Supporting Peptides:

| Peptide | Uniq | -10lgP | Mass | ppm | m/z | z | RT | Scan | #Spec | Start | End | PTM |
| --- | --- | --- | --- | --- | --- | --- | --- | --- | --- | --- | --- | --- |
| D.EDLSGVVLL.T | Y | 26.84 | 943.5226 | 11.7 | 472.7741 | 2 | 13.85 | 1685 | 1 | 673 | 681 |  |
| total 1 peptides |
| --- |

tr|A0A146ME36|A0A146ME36\_LYGHE

back to list

  

| Protein Coverage
| Supporting Peptides
|

Protein Coverage:

Supporting Peptides:

| Peptide | Uniq | -10lgP | Mass | ppm | m/z | z | RT | Scan | #Spec | Start | End | PTM |
| --- | --- | --- | --- | --- | --- | --- | --- | --- | --- | --- | --- | --- |
| S.EKPIDLKLNN.E | Y | 26.60 | 1182.6608 | -24.2 | 592.3234 | 2 | 13.16 | 1568 | 1 | 479 | 488 |  |
| total 1 peptides |
| --- |

tr|A0A0A9ZCU1|A0A0A9ZCU1\_LYGHE

back to list

  

| Protein Coverage
| Supporting Peptides
|

Protein Coverage:

Supporting Peptides:

| Peptide | Uniq | -10lgP | Mass | ppm | m/z | z | RT | Scan | #Spec | Start | End | PTM |
| --- | --- | --- | --- | --- | --- | --- | --- | --- | --- | --- | --- | --- |
| S.EKPIDLKLNN.E | Y | 26.60 | 1182.6608 | -24.2 | 592.3234 | 2 | 13.16 | 1568 | 1 | 682 | 691 |  |
| total 1 peptides |
| --- |

tr|A0A0A9Z559|A0A0A9Z559\_LYGHE

back to list

  

| Protein Coverage
| Supporting Peptides
|

Protein Coverage:

Supporting Peptides:

| Peptide | Uniq | -10lgP | Mass | ppm | m/z | z | RT | Scan | #Spec | Start | End | PTM |
| --- | --- | --- | --- | --- | --- | --- | --- | --- | --- | --- | --- | --- |
| S.EKPIDLKLNN.E | Y | 26.60 | 1182.6608 | -24.2 | 592.3234 | 2 | 13.16 | 1568 | 1 | 695 | 704 |  |
| total 1 peptides |
| --- |

tr|A0A553NSJ7|A0A553NSJ7\_TIGCA

back to list

  

| Protein Coverage
| Supporting Peptides
|

Protein Coverage:

Supporting Peptides:

| Peptide | Uniq | -10lgP | Mass | ppm | m/z | z | RT | Scan | #Spec | Start | End | PTM |
| --- | --- | --- | --- | --- | --- | --- | --- | --- | --- | --- | --- | --- |
| K.VVAKKPAGE.K | Y | 26.07 | 897.5283 | 19.8 | 449.7803 | 2 | 10.02 | 986 | 1 | 117 | 125 |  |
| total 1 peptides |
| --- |

tr|A0A553NV74|A0A553NV74\_TIGCA

back to list

  

| Protein Coverage
| Supporting Peptides
|

Protein Coverage:

Supporting Peptides:

| Peptide | Uniq | -10lgP | Mass | ppm | m/z | z | RT | Scan | #Spec | Start | End | PTM |
| --- | --- | --- | --- | --- | --- | --- | --- | --- | --- | --- | --- | --- |
| K.VVAKKPAGE.K | Y | 26.07 | 897.5283 | 19.8 | 449.7803 | 2 | 10.02 | 986 | 1 | 117 | 125 |  |
| total 1 peptides |
| --- |

tr|B4L6N9|B4L6N9\_DROMO

back to list

  

| Protein Coverage
| Supporting Peptides
|

Protein Coverage:

Supporting Peptides:

| Peptide | Uniq | -10lgP | Mass | ppm | m/z | z | RT | Scan | #Spec | Start | End | PTM |
| --- | --- | --- | --- | --- | --- | --- | --- | --- | --- | --- | --- | --- |
| G.AGGAGGSPGA.G | Y | 25.98 | 700.3140 | 0.1 | 351.1643 | 2 | 8.34 | 695 | 1 | 703 | 712 |  |
| total 1 peptides |
| --- |

tr|A0A484AUC9|A0A484AUC9\_DRONA

back to list

  

| Protein Coverage
| Supporting Peptides
|

Protein Coverage:

Supporting Peptides:

| Peptide | Uniq | -10lgP | Mass | ppm | m/z | z | RT | Scan | #Spec | Start | End | PTM |
| --- | --- | --- | --- | --- | --- | --- | --- | --- | --- | --- | --- | --- |
| G.AGGAGGSPGA.G | Y | 25.98 | 700.3140 | 0.1 | 351.1643 | 2 | 8.34 | 695 | 1 | 797 | 806 |  |
| total 1 peptides |
| --- |

tr|A0A182HNK7|A0A182HNK7\_ANOAR

back to list

  

| Protein Coverage
| Supporting Peptides
|

Protein Coverage:

Supporting Peptides:

| Peptide | Uniq | -10lgP | Mass | ppm | m/z | z | RT | Scan | #Spec | Start | End | PTM |
| --- | --- | --- | --- | --- | --- | --- | --- | --- | --- | --- | --- | --- |
| C.FGYTSK.N | Y | 24.12 | 701.3384 | 8.1 | 351.6793 | 2 | 16.26 | 2033 | 1 | 634 | 639 |  |
| total 1 peptides |
| --- |

tr|A0A1B0CTB8|A0A1B0CTB8\_LUTLO

back to list

  

| Protein Coverage
| Supporting Peptides
|

Protein Coverage:

Supporting Peptides:

| Peptide | Uniq | -10lgP | Mass | ppm | m/z | z | RT | Scan | #Spec | Start | End | PTM |
| --- | --- | --- | --- | --- | --- | --- | --- | --- | --- | --- | --- | --- |
| Q.EALEHLII.I | Y | 23.80 | 936.5280 | -0.4 | 469.2711 | 2 | 13.10 | 1557 | 1 | 2360 | 2367 |  |
| total 1 peptides |
| --- |

tr|E9IDT0|E9IDT0\_SOLIN

back to list

  

| Protein Coverage
| Supporting Peptides
|

Protein Coverage:

Supporting Peptides:

| Peptide | Uniq | -10lgP | Mass | ppm | m/z | z | RT | Scan | #Spec | Start | End | PTM |
| --- | --- | --- | --- | --- | --- | --- | --- | --- | --- | --- | --- | --- |
| M.EALEHLIL.C | Y | 23.80 | 936.5280 | -0.4 | 469.2711 | 2 | 13.10 | 1557 | 1 | 530 | 537 |  |
| total 1 peptides |
| --- |

tr|A0A6V7J2X2|A0A6V7J2X2\_9HYME

back to list

  

| Protein Coverage
| Supporting Peptides
|

Protein Coverage:

Supporting Peptides:

| Peptide | Uniq | -10lgP | Mass | ppm | m/z | z | RT | Scan | #Spec | Start | End | PTM |
| --- | --- | --- | --- | --- | --- | --- | --- | --- | --- | --- | --- | --- |
| A.EAIEHLLI.S | Y | 23.80 | 936.5280 | -0.4 | 469.2711 | 2 | 13.10 | 1557 | 1 | 51 | 58 |  |
| total 1 peptides |
| --- |

tr|A0A7R8V5M9|A0A7R8V5M9\_HERIL

back to list

  

| Protein Coverage
| Supporting Peptides
|

Protein Coverage:

Supporting Peptides:

| Peptide | Uniq | -10lgP | Mass | ppm | m/z | z | RT | Scan | #Spec | Start | End | PTM |
| --- | --- | --- | --- | --- | --- | --- | --- | --- | --- | --- | --- | --- |
| T.EALEHIII.V | Y | 23.80 | 936.5280 | -0.4 | 469.2711 | 2 | 13.10 | 1557 | 1 | 30 | 37 |  |
| total 1 peptides |
| --- |

tr|A0A0K8TNK6|A0A0K8TNK6\_TABBR

back to list

  

| Protein Coverage
| Supporting Peptides
|

Protein Coverage:

Supporting Peptides:

| Peptide | Uniq | -10lgP | Mass | ppm | m/z | z | RT | Scan | #Spec | Start | End | PTM |
| --- | --- | --- | --- | --- | --- | --- | --- | --- | --- | --- | --- | --- |
| T.EALEHILI.V | Y | 23.80 | 936.5280 | -0.4 | 469.2711 | 2 | 13.10 | 1557 | 1 | 17 | 24 |  |
| total 1 peptides |
| --- |

tr|B4NGC5|B4NGC5\_DROWI

back to list

  

| Protein Coverage
| Supporting Peptides
|

Protein Coverage:

Supporting Peptides:

| Peptide | Uniq | -10lgP | Mass | ppm | m/z | z | RT | Scan | #Spec | Start | End | PTM |
| --- | --- | --- | --- | --- | --- | --- | --- | --- | --- | --- | --- | --- |
| A.EALEHILI.A | Y | 23.80 | 936.5280 | -0.4 | 469.2711 | 2 | 13.10 | 1557 | 1 | 47 | 54 |  |
| total 1 peptides |
| --- |

tr|B4M4U0|B4M4U0\_DROVI

back to list

  

| Protein Coverage
| Supporting Peptides
|

Protein Coverage:

Supporting Peptides:

| Peptide | Uniq | -10lgP | Mass | ppm | m/z | z | RT | Scan | #Spec | Start | End | PTM |
| --- | --- | --- | --- | --- | --- | --- | --- | --- | --- | --- | --- | --- |
| A.EALEHLLI.A | Y | 23.80 | 936.5280 | -0.4 | 469.2711 | 2 | 13.10 | 1557 | 1 | 50 | 57 |  |
| total 1 peptides |
| --- |

tr|A0A484BDS3|A0A484BDS3\_DRONA

back to list

  

| Protein Coverage
| Supporting Peptides
|

Protein Coverage:

Supporting Peptides:

| Peptide | Uniq | -10lgP | Mass | ppm | m/z | z | RT | Scan | #Spec | Start | End | PTM |
| --- | --- | --- | --- | --- | --- | --- | --- | --- | --- | --- | --- | --- |
| A.EALEHILI.A | Y | 23.80 | 936.5280 | -0.4 | 469.2711 | 2 | 13.10 | 1557 | 1 | 50 | 57 |  |
| total 1 peptides |
| --- |

tr|A0A6J1M8J1|A0A6J1M8J1\_DROHY

back to list

  

| Protein Coverage
| Supporting Peptides
|

Protein Coverage:

Supporting Peptides:

| Peptide | Uniq | -10lgP | Mass | ppm | m/z | z | RT | Scan | #Spec | Start | End | PTM |
| --- | --- | --- | --- | --- | --- | --- | --- | --- | --- | --- | --- | --- |
| A.EALEHILI.A | Y | 23.80 | 936.5280 | -0.4 | 469.2711 | 2 | 13.10 | 1557 | 1 | 50 | 57 |  |
| total 1 peptides |
| --- |

tr|B4KE34|B4KE34\_DROMO

back to list

  

| Protein Coverage
| Supporting Peptides
|

Protein Coverage:

Supporting Peptides:

| Peptide | Uniq | -10lgP | Mass | ppm | m/z | z | RT | Scan | #Spec | Start | End | PTM |
| --- | --- | --- | --- | --- | --- | --- | --- | --- | --- | --- | --- | --- |
| A.EALEHILI.A | Y | 23.80 | 936.5280 | -0.4 | 469.2711 | 2 | 13.10 | 1557 | 1 | 50 | 57 |  |
| total 1 peptides |
| --- |

tr|A0A0M4EG71|A0A0M4EG71\_DROBS

back to list

  

| Protein Coverage
| Supporting Peptides
|

Protein Coverage:

Supporting Peptides:

| Peptide | Uniq | -10lgP | Mass | ppm | m/z | z | RT | Scan | #Spec | Start | End | PTM |
| --- | --- | --- | --- | --- | --- | --- | --- | --- | --- | --- | --- | --- |
| A.EALEHILI.A | Y | 23.80 | 936.5280 | -0.4 | 469.2711 | 2 | 13.10 | 1557 | 1 | 49 | 56 |  |
| total 1 peptides |
| --- |

tr|A0A6P8XFN0|A0A6P8XFN0\_DROAB

back to list

  

| Protein Coverage
| Supporting Peptides
|

Protein Coverage:

Supporting Peptides:

| Peptide | Uniq | -10lgP | Mass | ppm | m/z | z | RT | Scan | #Spec | Start | End | PTM |
| --- | --- | --- | --- | --- | --- | --- | --- | --- | --- | --- | --- | --- |
| A.EALEHILI.A | Y | 23.80 | 936.5280 | -0.4 | 469.2711 | 2 | 13.10 | 1557 | 1 | 49 | 56 |  |
| total 1 peptides |
| --- |

tr|A0A6J2TAH7|A0A6J2TAH7\_DROLE

back to list

  

| Protein Coverage
| Supporting Peptides
|

Protein Coverage:

Supporting Peptides:

| Peptide | Uniq | -10lgP | Mass | ppm | m/z | z | RT | Scan | #Spec | Start | End | PTM |
| --- | --- | --- | --- | --- | --- | --- | --- | --- | --- | --- | --- | --- |
| A.EALEHILI.V | Y | 23.80 | 936.5280 | -0.4 | 469.2711 | 2 | 13.10 | 1557 | 1 | 58 | 65 |  |
| total 1 peptides |
| --- |

tr|A0A6J2TEK1|A0A6J2TEK1\_DROLE

back to list

  

| Protein Coverage
| Supporting Peptides
|

Protein Coverage:

Supporting Peptides:

| Peptide | Uniq | -10lgP | Mass | ppm | m/z | z | RT | Scan | #Spec | Start | End | PTM |
| --- | --- | --- | --- | --- | --- | --- | --- | --- | --- | --- | --- | --- |
| A.EALEHILI.V | Y | 23.80 | 936.5280 | -0.4 | 469.2711 | 2 | 13.10 | 1557 | 1 | 58 | 65 |  |
| total 1 peptides |
| --- |

tr|A0A7I0ZIP4|A0A7I0ZIP4\_DANPL

back to list

  

| Protein Coverage
| Supporting Peptides
|

Protein Coverage:

Supporting Peptides:

| Peptide | Uniq | -10lgP | Mass | ppm | m/z | z | RT | Scan | #Spec | Start | End | PTM |
| --- | --- | --- | --- | --- | --- | --- | --- | --- | --- | --- | --- | --- |
| E.EALEHLLL.Q | Y | 23.80 | 936.5280 | -0.4 | 469.2711 | 2 | 13.10 | 1557 | 1 | 50 | 57 |  |
| total 1 peptides |
| --- |

tr|A0A7I0ZK43|A0A7I0ZK43\_DANPL

back to list

  

| Protein Coverage
| Supporting Peptides
|

Protein Coverage:

Supporting Peptides:

| Peptide | Uniq | -10lgP | Mass | ppm | m/z | z | RT | Scan | #Spec | Start | End | PTM |
| --- | --- | --- | --- | --- | --- | --- | --- | --- | --- | --- | --- | --- |
| E.EALEHLLL.Q | Y | 23.80 | 936.5280 | -0.4 | 469.2711 | 2 | 13.10 | 1557 | 1 | 52 | 59 |  |
| total 1 peptides |
| --- |

tr|A0A7M6UVA6|A0A7M6UVA6\_NASVI

back to list

  

| Protein Coverage
| Supporting Peptides
|

Protein Coverage:

Supporting Peptides:

| Peptide | Uniq | -10lgP | Mass | ppm | m/z | z | RT | Scan | #Spec | Start | End | PTM |
| --- | --- | --- | --- | --- | --- | --- | --- | --- | --- | --- | --- | --- |
| A.EALEHLLI.R | Y | 23.80 | 936.5280 | -0.4 | 469.2711 | 2 | 13.10 | 1557 | 1 | 501 | 508 |  |
| total 1 peptides |
| --- |

tr|A0A7M7QVM7|A0A7M7QVM7\_NASVI

back to list

  

| Protein Coverage
| Supporting Peptides
|

Protein Coverage:

Supporting Peptides:

| Peptide | Uniq | -10lgP | Mass | ppm | m/z | z | RT | Scan | #Spec | Start | End | PTM |
| --- | --- | --- | --- | --- | --- | --- | --- | --- | --- | --- | --- | --- |
| A.EALEHLLI.R | Y | 23.80 | 936.5280 | -0.4 | 469.2711 | 2 | 13.10 | 1557 | 1 | 501 | 508 |  |
| total 1 peptides |
| --- |

tr|A0A232F624|A0A232F624\_9HYME

back to list

  

| Protein Coverage
| Supporting Peptides
|

Protein Coverage:

Supporting Peptides:

| Peptide | Uniq | -10lgP | Mass | ppm | m/z | z | RT | Scan | #Spec | Start | End | PTM |
| --- | --- | --- | --- | --- | --- | --- | --- | --- | --- | --- | --- | --- |
| A.EALEHLLI.R | Y | 23.80 | 936.5280 | -0.4 | 469.2711 | 2 | 13.10 | 1557 | 1 | 505 | 512 |  |
| total 1 peptides |
| --- |

tr|F4WT59|F4WT59\_ACREC

back to list

  

| Protein Coverage
| Supporting Peptides
|

Protein Coverage:

Supporting Peptides:

| Peptide | Uniq | -10lgP | Mass | ppm | m/z | z | RT | Scan | #Spec | Start | End | PTM |
| --- | --- | --- | --- | --- | --- | --- | --- | --- | --- | --- | --- | --- |
| I.AESSITVPDVKYVI.D | Y | 22.27 | 1519.8134 | -24.0 | 760.8957 | 2 | 12.27 | 1409 | 1 | 309 | 322 |  |
| total 1 peptides |
| --- |

tr|A0A836FB18|A0A836FB18\_9HYME

back to list

  

| Protein Coverage
| Supporting Peptides
|

Protein Coverage:

Supporting Peptides:

| Peptide | Uniq | -10lgP | Mass | ppm | m/z | z | RT | Scan | #Spec | Start | End | PTM |
| --- | --- | --- | --- | --- | --- | --- | --- | --- | --- | --- | --- | --- |
| I.AESSITVPDVKYVI.D | Y | 22.27 | 1519.8134 | -24.0 | 760.8957 | 2 | 12.27 | 1409 | 1 | 382 | 395 |  |
| total 1 peptides |
| --- |

tr|A0A0J7L582|A0A0J7L582\_LASNI

back to list

  

| Protein Coverage
| Supporting Peptides
|

Protein Coverage:

Supporting Peptides:

| Peptide | Uniq | -10lgP | Mass | ppm | m/z | z | RT | Scan | #Spec | Start | End | PTM |
| --- | --- | --- | --- | --- | --- | --- | --- | --- | --- | --- | --- | --- |
| V.AESSITVPDVKYVI.D | Y | 22.27 | 1519.8134 | -24.0 | 760.8957 | 2 | 12.27 | 1409 | 1 | 382 | 395 |  |
| total 1 peptides |
| --- |

tr|A0A151J597|A0A151J597\_9HYME

back to list

  

| Protein Coverage
| Supporting Peptides
|

Protein Coverage:

Supporting Peptides:

| Peptide | Uniq | -10lgP | Mass | ppm | m/z | z | RT | Scan | #Spec | Start | End | PTM |
| --- | --- | --- | --- | --- | --- | --- | --- | --- | --- | --- | --- | --- |
| I.AESSITVPDVKYVI.D | Y | 22.27 | 1519.8134 | -24.0 | 760.8957 | 2 | 12.27 | 1409 | 1 | 382 | 395 |  |
| total 1 peptides |
| --- |

tr|A0A836JUW8|A0A836JUW8\_9HYME

back to list

  

| Protein Coverage
| Supporting Peptides
|

Protein Coverage:

Supporting Peptides:

| Peptide | Uniq | -10lgP | Mass | ppm | m/z | z | RT | Scan | #Spec | Start | End | PTM |
| --- | --- | --- | --- | --- | --- | --- | --- | --- | --- | --- | --- | --- |
| I.AESSITVPDVKYVI.D | Y | 22.27 | 1519.8134 | -24.0 | 760.8957 | 2 | 12.27 | 1409 | 1 | 407 | 420 |  |
| total 1 peptides |
| --- |

tr|A0A836JG75|A0A836JG75\_9HYME

back to list

  

| Protein Coverage
| Supporting Peptides
|

Protein Coverage:

Supporting Peptides:

| Peptide | Uniq | -10lgP | Mass | ppm | m/z | z | RT | Scan | #Spec | Start | End | PTM |
| --- | --- | --- | --- | --- | --- | --- | --- | --- | --- | --- | --- | --- |
| I.AESSITVPDVKYVI.D | Y | 22.27 | 1519.8134 | -24.0 | 760.8957 | 2 | 12.27 | 1409 | 1 | 398 | 411 |  |
| total 1 peptides |
| --- |

tr|A0A1L3IWM3|A0A1L3IWM3\_RHOPD

back to list

  

| Protein Coverage
| Supporting Peptides
|

Protein Coverage:

Supporting Peptides:

| Peptide | Uniq | -10lgP | Mass | ppm | m/z | z | RT | Scan | #Spec | Start | End | PTM |
| --- | --- | --- | --- | --- | --- | --- | --- | --- | --- | --- | --- | --- |
| E.VLVSTVIM.Q | Y | 20.57 | 860.5042 | 11.8 | 431.2644 | 2 | 11.34 | 1236 | 1 | 537 | 544 |  |
| total 1 peptides |
| --- |

tr|A0A2S2QP63|A0A2S2QP63\_9HEMI

back to list

  

| Protein Coverage
| Supporting Peptides
|

Protein Coverage:

Supporting Peptides:

| Peptide | Uniq | -10lgP | Mass | ppm | m/z | z | RT | Scan | #Spec | Start | End | PTM |
| --- | --- | --- | --- | --- | --- | --- | --- | --- | --- | --- | --- | --- |
| E.VLVSTVIM.Q | Y | 20.57 | 860.5042 | 11.8 | 431.2644 | 2 | 11.34 | 1236 | 1 | 93 | 100 |  |
| total 1 peptides |
| --- |

tr|A0A2S2NYH2|A0A2S2NYH2\_SCHGA

back to list

  

| Protein Coverage
| Supporting Peptides
|

Protein Coverage:

Supporting Peptides:

| Peptide | Uniq | -10lgP | Mass | ppm | m/z | z | RT | Scan | #Spec | Start | End | PTM |
| --- | --- | --- | --- | --- | --- | --- | --- | --- | --- | --- | --- | --- |
| E.VLVSTVIM.Q | Y | 20.57 | 860.5042 | 11.8 | 431.2644 | 2 | 11.34 | 1236 | 1 | 127 | 134 |  |
| total 1 peptides |
| --- |

tr|A0A6G0YXF5|A0A6G0YXF5\_APHCR

back to list

  

| Protein Coverage
| Supporting Peptides
|

Protein Coverage:

Supporting Peptides:

| Peptide | Uniq | -10lgP | Mass | ppm | m/z | z | RT | Scan | #Spec | Start | End | PTM |
| --- | --- | --- | --- | --- | --- | --- | --- | --- | --- | --- | --- | --- |
| E.VLVSTVIM.Q | Y | 20.57 | 860.5042 | 11.8 | 431.2644 | 2 | 11.34 | 1236 | 1 | 489 | 496 |  |
| total 1 peptides |
| --- |

tr|A0A6G0UB83|A0A6G0UB83\_APHGL

back to list

  

| Protein Coverage
| Supporting Peptides
|

Protein Coverage:

Supporting Peptides:

| Peptide | Uniq | -10lgP | Mass | ppm | m/z | z | RT | Scan | #Spec | Start | End | PTM |
| --- | --- | --- | --- | --- | --- | --- | --- | --- | --- | --- | --- | --- |
| E.VLVSTVIM.Q | Y | 20.57 | 860.5042 | 11.8 | 431.2644 | 2 | 11.34 | 1236 | 1 | 511 | 518 |  |
| total 1 peptides |
| --- |

tr|A0A182VZF1|A0A182VZF1\_9DIPT

back to list

  

| Protein Coverage
| Supporting Peptides
|

Protein Coverage:

Supporting Peptides:

| Peptide | Uniq | -10lgP | Mass | ppm | m/z | z | RT | Scan | #Spec | Start | End | PTM |
| --- | --- | --- | --- | --- | --- | --- | --- | --- | --- | --- | --- | --- |
| K.DRTKRKPKKALT.Q | Y | 20.54 | 1440.8888 | 11.5 | 721.4600 | 2 | 22.62 | 3041 | 1 | 178 | 189 |  |
| total 1 peptides |
| --- |

tr|A0A023ERZ2|A0A023ERZ2\_AEDAL

back to list

  

| Protein Coverage
| Supporting Peptides
|

Protein Coverage:

Supporting Peptides:

| Peptide | Uniq | -10lgP | Mass | ppm | m/z | z | RT | Scan | #Spec | Start | End | PTM |
| --- | --- | --- | --- | --- | --- | --- | --- | --- | --- | --- | --- | --- |
| R.GDLGIEIPAEK.V | Y | 20.51 | 1140.6025 | 0.0 | 571.3085 | 2 | 11.94 | 1350 | 1 | 281 | 291 |  |
| total 1 peptides |
| --- |

tr|A0A0P8Y097|A0A0P8Y097\_DROAN

back to list

  

| Protein Coverage
| Supporting Peptides
|

Protein Coverage:

Supporting Peptides:

| Peptide | Uniq | -10lgP | Mass | ppm | m/z | z | RT | Scan | #Spec | Start | End | PTM |
| --- | --- | --- | --- | --- | --- | --- | --- | --- | --- | --- | --- | --- |
| R.GDLGIEIPAEK.V | Y | 20.51 | 1140.6025 | 0.0 | 571.3085 | 2 | 11.94 | 1350 | 1 | 277 | 287 |  |
| total 1 peptides |
| --- |

tr|A0A6P8K875|A0A6P8K875\_DROMA

back to list

  

| Protein Coverage
| Supporting Peptides
|

Protein Coverage:

Supporting Peptides:

| Peptide | Uniq | -10lgP | Mass | ppm | m/z | z | RT | Scan | #Spec | Start | End | PTM |
| --- | --- | --- | --- | --- | --- | --- | --- | --- | --- | --- | --- | --- |
| R.GDLGIEIPAEK.V | Y | 20.51 | 1140.6025 | 0.0 | 571.3085 | 2 | 11.94 | 1350 | 1 | 277 | 287 |  |
| total 1 peptides |
| --- |

tr|A0A6P4F4P4|A0A6P4F4P4\_DRORH

back to list

  

| Protein Coverage
| Supporting Peptides
|

Protein Coverage:

Supporting Peptides:

| Peptide | Uniq | -10lgP | Mass | ppm | m/z | z | RT | Scan | #Spec | Start | End | PTM |
| --- | --- | --- | --- | --- | --- | --- | --- | --- | --- | --- | --- | --- |
| R.GDLGIEIPAEK.V | Y | 20.51 | 1140.6025 | 0.0 | 571.3085 | 2 | 11.94 | 1350 | 1 | 277 | 287 |  |
| total 1 peptides |
| --- |

tr|A0A1Y9GJY2|A0A1Y9GJY2\_ANOAR

back to list

  

| Protein Coverage
| Supporting Peptides
|

Protein Coverage:

Supporting Peptides:

| Peptide | Uniq | -10lgP | Mass | ppm | m/z | z | RT | Scan | #Spec | Start | End | PTM |
| --- | --- | --- | --- | --- | --- | --- | --- | --- | --- | --- | --- | --- |
| R.GDLGIEIPAEK.V | Y | 20.51 | 1140.6025 | 0.0 | 571.3085 | 2 | 11.94 | 1350 | 1 | 277 | 287 |  |
| total 1 peptides |
| --- |

tr|A0A0Q9X1L7|A0A0Q9X1L7\_DROMO

back to list

  

| Protein Coverage
| Supporting Peptides
|

Protein Coverage:

Supporting Peptides:

| Peptide | Uniq | -10lgP | Mass | ppm | m/z | z | RT | Scan | #Spec | Start | End | PTM |
| --- | --- | --- | --- | --- | --- | --- | --- | --- | --- | --- | --- | --- |
| R.GDLGIEIPAEK.V | Y | 20.51 | 1140.6025 | 0.0 | 571.3085 | 2 | 11.94 | 1350 | 1 | 277 | 287 |  |
| total 1 peptides |
| --- |

tr|A0A2M3ZFB9|A0A2M3ZFB9\_9DIPT

back to list

  

| Protein Coverage
| Supporting Peptides
|

Protein Coverage:

Supporting Peptides:

| Peptide | Uniq | -10lgP | Mass | ppm | m/z | z | RT | Scan | #Spec | Start | End | PTM |
| --- | --- | --- | --- | --- | --- | --- | --- | --- | --- | --- | --- | --- |
| R.GDLGIEIPAEK.V | Y | 20.51 | 1140.6025 | 0.0 | 571.3085 | 2 | 11.94 | 1350 | 1 | 281 | 291 |  |
| total 1 peptides |
| --- |

tr|A0A2M3ZYS6|A0A2M3ZYS6\_9DIPT

back to list

  

| Protein Coverage
| Supporting Peptides
|

Protein Coverage:

Supporting Peptides:

| Peptide | Uniq | -10lgP | Mass | ppm | m/z | z | RT | Scan | #Spec | Start | End | PTM |
| --- | --- | --- | --- | --- | --- | --- | --- | --- | --- | --- | --- | --- |
| R.GDLGIEIPAEK.V | Y | 20.51 | 1140.6025 | 0.0 | 571.3085 | 2 | 11.94 | 1350 | 1 | 281 | 291 |  |
| total 1 peptides |
| --- |

tr|A0A2M3ZYS8|A0A2M3ZYS8\_9DIPT

back to list

  

| Protein Coverage
| Supporting Peptides
|

Protein Coverage:

Supporting Peptides:

| Peptide | Uniq | -10lgP | Mass | ppm | m/z | z | RT | Scan | #Spec | Start | End | PTM |
| --- | --- | --- | --- | --- | --- | --- | --- | --- | --- | --- | --- | --- |
| R.GDLGIEIPAEK.V | Y | 20.51 | 1140.6025 | 0.0 | 571.3085 | 2 | 11.94 | 1350 | 1 | 281 | 291 |  |
| total 1 peptides |
| --- |

tr|A0A1Y9GJK0|A0A1Y9GJK0\_ANOAR

back to list

  

| Protein Coverage
| Supporting Peptides
|

Protein Coverage:

Supporting Peptides:

| Peptide | Uniq | -10lgP | Mass | ppm | m/z | z | RT | Scan | #Spec | Start | End | PTM |
| --- | --- | --- | --- | --- | --- | --- | --- | --- | --- | --- | --- | --- |
| R.GDLGIEIPAEK.V | Y | 20.51 | 1140.6025 | 0.0 | 571.3085 | 2 | 11.94 | 1350 | 1 | 282 | 292 |  |
| total 1 peptides |
| --- |

tr|A0A6A0H7S7|A0A6A0H7S7\_HYAAZ

back to list

  

| Protein Coverage
| Supporting Peptides
|

Protein Coverage:

Supporting Peptides:

| Peptide | Uniq | -10lgP | Mass | ppm | m/z | z | RT | Scan | #Spec | Start | End | PTM |
| --- | --- | --- | --- | --- | --- | --- | --- | --- | --- | --- | --- | --- |
| R.GDLGIEIPAEK.V | Y | 20.51 | 1140.6025 | 0.0 | 571.3085 | 2 | 11.94 | 1350 | 1 | 286 | 296 |  |
| total 1 peptides |
| --- |

tr|A0A1E1X8N4|A0A1E1X8N4\_9ACAR

back to list

  

| Protein Coverage
| Supporting Peptides
|

Protein Coverage:

Supporting Peptides:

| Peptide | Uniq | -10lgP | Mass | ppm | m/z | z | RT | Scan | #Spec | Start | End | PTM |
| --- | --- | --- | --- | --- | --- | --- | --- | --- | --- | --- | --- | --- |
| R.GDLGIEIPAEK.V | Y | 20.51 | 1140.6025 | 0.0 | 571.3085 | 2 | 11.94 | 1350 | 1 | 290 | 300 |  |
| total 1 peptides |
| --- |

tr|A0A6P4F9I4|A0A6P4F9I4\_DRORH

back to list

  

| Protein Coverage
| Supporting Peptides
|

Protein Coverage:

Supporting Peptides:

| Peptide | Uniq | -10lgP | Mass | ppm | m/z | z | RT | Scan | #Spec | Start | End | PTM |
| --- | --- | --- | --- | --- | --- | --- | --- | --- | --- | --- | --- | --- |
| R.GDLGIEIPAEK.V | Y | 20.51 | 1140.6025 | 0.0 | 571.3085 | 2 | 11.94 | 1350 | 1 | 298 | 308 |  |
| total 1 peptides |
| --- |

tr|A0A182VPN6|A0A182VPN6\_9DIPT

back to list

  

| Protein Coverage
| Supporting Peptides
|

Protein Coverage:

Supporting Peptides:

| Peptide | Uniq | -10lgP | Mass | ppm | m/z | z | RT | Scan | #Spec | Start | End | PTM |
| --- | --- | --- | --- | --- | --- | --- | --- | --- | --- | --- | --- | --- |
| R.GDLGIEIPAEK.V | Y | 20.51 | 1140.6025 | 0.0 | 571.3085 | 2 | 11.94 | 1350 | 1 | 293 | 303 |  |
| total 1 peptides |
| --- |

tr|A0A182MNQ6|A0A182MNQ6\_9DIPT

back to list

  

| Protein Coverage
| Supporting Peptides
|

Protein Coverage:

Supporting Peptides:

| Peptide | Uniq | -10lgP | Mass | ppm | m/z | z | RT | Scan | #Spec | Start | End | PTM |
| --- | --- | --- | --- | --- | --- | --- | --- | --- | --- | --- | --- | --- |
| R.GDLGIEIPAEK.V | Y | 20.51 | 1140.6025 | 0.0 | 571.3085 | 2 | 11.94 | 1350 | 1 | 293 | 303 |  |
| total 1 peptides |
| --- |

tr|A0A6J2T5C5|A0A6J2T5C5\_DROLE

back to list

  

| Protein Coverage
| Supporting Peptides
|

Protein Coverage:

Supporting Peptides:

| Peptide | Uniq | -10lgP | Mass | ppm | m/z | z | RT | Scan | #Spec | Start | End | PTM |
| --- | --- | --- | --- | --- | --- | --- | --- | --- | --- | --- | --- | --- |
| R.GDLGIEIPAEK.V | Y | 20.51 | 1140.6025 | 0.0 | 571.3085 | 2 | 11.94 | 1350 | 1 | 298 | 308 |  |
| total 1 peptides |
| --- |

tr|A0A1W4W378|A0A1W4W378\_DROFC

back to list

  

| Protein Coverage
| Supporting Peptides
|

Protein Coverage:

Supporting Peptides:

| Peptide | Uniq | -10lgP | Mass | ppm | m/z | z | RT | Scan | #Spec | Start | End | PTM |
| --- | --- | --- | --- | --- | --- | --- | --- | --- | --- | --- | --- | --- |
| R.GDLGIEIPAEK.V | Y | 20.51 | 1140.6025 | 0.0 | 571.3085 | 2 | 11.94 | 1350 | 1 | 298 | 308 |  |
| total 1 peptides |
| --- |

O62619|KPYK\_DROME

back to list

  

| Protein Coverage
| Supporting Peptides
|

Protein Coverage:

Supporting Peptides:

| Peptide | Uniq | -10lgP | Mass | ppm | m/z | z | RT | Scan | #Spec | Start | End | PTM |
| --- | --- | --- | --- | --- | --- | --- | --- | --- | --- | --- | --- | --- |
| R.GDLGIEIPAEK.V | Y | 20.51 | 1140.6025 | 0.0 | 571.3085 | 2 | 11.94 | 1350 | 1 | 298 | 308 |  |
| total 1 peptides |
| --- |

tr|B4HE69|B4HE69\_DROSE

back to list

  

| Protein Coverage
| Supporting Peptides
|

Protein Coverage:

Supporting Peptides:

| Peptide | Uniq | -10lgP | Mass | ppm | m/z | z | RT | Scan | #Spec | Start | End | PTM |
| --- | --- | --- | --- | --- | --- | --- | --- | --- | --- | --- | --- | --- |
| R.GDLGIEIPAEK.V | Y | 20.51 | 1140.6025 | 0.0 | 571.3085 | 2 | 11.94 | 1350 | 1 | 298 | 308 |  |
| total 1 peptides |
| --- |

tr|A0A6P4F0Q9|A0A6P4F0Q9\_DRORH

back to list

  

| Protein Coverage
| Supporting Peptides
|

Protein Coverage:

Supporting Peptides:

| Peptide | Uniq | -10lgP | Mass | ppm | m/z | z | RT | Scan | #Spec | Start | End | PTM |
| --- | --- | --- | --- | --- | --- | --- | --- | --- | --- | --- | --- | --- |
| R.GDLGIEIPAEK.V | Y | 20.51 | 1140.6025 | 0.0 | 571.3085 | 2 | 11.94 | 1350 | 1 | 298 | 308 |  |
| total 1 peptides |
| --- |

tr|B3P7K4|B3P7K4\_DROER

back to list

  

| Protein Coverage
| Supporting Peptides
|

Protein Coverage:

Supporting Peptides:

| Peptide | Uniq | -10lgP | Mass | ppm | m/z | z | RT | Scan | #Spec | Start | End | PTM |
| --- | --- | --- | --- | --- | --- | --- | --- | --- | --- | --- | --- | --- |
| R.GDLGIEIPAEK.V | Y | 20.51 | 1140.6025 | 0.0 | 571.3085 | 2 | 11.94 | 1350 | 1 | 298 | 308 |  |
| total 1 peptides |
| --- |

tr|B3M213|B3M213\_DROAN

back to list

  

| Protein Coverage
| Supporting Peptides
|

Protein Coverage:

Supporting Peptides:

| Peptide | Uniq | -10lgP | Mass | ppm | m/z | z | RT | Scan | #Spec | Start | End | PTM |
| --- | --- | --- | --- | --- | --- | --- | --- | --- | --- | --- | --- | --- |
| R.GDLGIEIPAEK.V | Y | 20.51 | 1140.6025 | 0.0 | 571.3085 | 2 | 11.94 | 1350 | 1 | 298 | 308 |  |
| total 1 peptides |
| --- |

tr|A0A6P8KMB4|A0A6P8KMB4\_DROMA

back to list

  

| Protein Coverage
| Supporting Peptides
|

Protein Coverage:

Supporting Peptides:

| Peptide | Uniq | -10lgP | Mass | ppm | m/z | z | RT | Scan | #Spec | Start | End | PTM |
| --- | --- | --- | --- | --- | --- | --- | --- | --- | --- | --- | --- | --- |
| R.GDLGIEIPAEK.V | Y | 20.51 | 1140.6025 | 0.0 | 571.3085 | 2 | 11.94 | 1350 | 1 | 298 | 308 |  |
| total 1 peptides |
| --- |

tr|B4K5E3|B4K5E3\_DROMO

back to list

  

| Protein Coverage
| Supporting Peptides
|

Protein Coverage:

Supporting Peptides:

| Peptide | Uniq | -10lgP | Mass | ppm | m/z | z | RT | Scan | #Spec | Start | End | PTM |
| --- | --- | --- | --- | --- | --- | --- | --- | --- | --- | --- | --- | --- |
| R.GDLGIEIPAEK.V | Y | 20.51 | 1140.6025 | 0.0 | 571.3085 | 2 | 11.94 | 1350 | 1 | 298 | 308 |  |
| total 1 peptides |
| --- |

tr|A0A484BWG4|A0A484BWG4\_DRONA

back to list

  

| Protein Coverage
| Supporting Peptides
|

Protein Coverage:

Supporting Peptides:

| Peptide | Uniq | -10lgP | Mass | ppm | m/z | z | RT | Scan | #Spec | Start | End | PTM |
| --- | --- | --- | --- | --- | --- | --- | --- | --- | --- | --- | --- | --- |
| R.GDLGIEIPAEK.V | Y | 20.51 | 1140.6025 | 0.0 | 571.3085 | 2 | 11.94 | 1350 | 1 | 298 | 308 |  |
| total 1 peptides |
| --- |

tr|A0A131XQM7|A0A131XQM7\_9ACAR

back to list

  

| Protein Coverage
| Supporting Peptides
|

Protein Coverage:

Supporting Peptides:

| Peptide | Uniq | -10lgP | Mass | ppm | m/z | z | RT | Scan | #Spec | Start | End | PTM |
| --- | --- | --- | --- | --- | --- | --- | --- | --- | --- | --- | --- | --- |
| R.GDLGIEIPAEK.V | Y | 20.51 | 1140.6025 | 0.0 | 571.3085 | 2 | 11.94 | 1350 | 1 | 290 | 300 |  |
| total 1 peptides |
| --- |

tr|A0A0P9ARZ1|A0A0P9ARZ1\_DROAN

back to list

  

| Protein Coverage
| Supporting Peptides
|

Protein Coverage:

Supporting Peptides:

| Peptide | Uniq | -10lgP | Mass | ppm | m/z | z | RT | Scan | #Spec | Start | End | PTM |
| --- | --- | --- | --- | --- | --- | --- | --- | --- | --- | --- | --- | --- |
| R.GDLGIEIPAEK.V | Y | 20.51 | 1140.6025 | 0.0 | 571.3085 | 2 | 11.94 | 1350 | 1 | 312 | 322 |  |
| total 1 peptides |
| --- |

tr|G3MLC1|G3MLC1\_AMBMU

back to list

  

| Protein Coverage
| Supporting Peptides
|

Protein Coverage:

Supporting Peptides:

| Peptide | Uniq | -10lgP | Mass | ppm | m/z | z | RT | Scan | #Spec | Start | End | PTM |
| --- | --- | --- | --- | --- | --- | --- | --- | --- | --- | --- | --- | --- |
| R.GDLGIEIPAEK.V | Y | 20.51 | 1140.6025 | 0.0 | 571.3085 | 2 | 11.94 | 1350 | 1 | 290 | 300 |  |
| total 1 peptides |
| --- |

tr|A0A023EVC5|A0A023EVC5\_AEDAL

back to list

  

| Protein Coverage
| Supporting Peptides
|

Protein Coverage:

Supporting Peptides:

| Peptide | Uniq | -10lgP | Mass | ppm | m/z | z | RT | Scan | #Spec | Start | End | PTM |
| --- | --- | --- | --- | --- | --- | --- | --- | --- | --- | --- | --- | --- |
| R.GDLGIEIPAEK.V | Y | 20.51 | 1140.6025 | 0.0 | 571.3085 | 2 | 11.94 | 1350 | 1 | 350 | 360 |  |
| total 1 peptides |
| --- |

tr|B6RHH5|B6RHH5\_PENVA

back to list

  

| Protein Coverage
| Supporting Peptides
|

Protein Coverage:

Supporting Peptides:

| Peptide | Uniq | -10lgP | Mass | ppm | m/z | z | RT | Scan | #Spec | Start | End | PTM |
| --- | --- | --- | --- | --- | --- | --- | --- | --- | --- | --- | --- | --- |
| R.GDLGIEIPAEK.V | Y | 20.51 | 1140.6025 | 0.0 | 571.3085 | 2 | 11.94 | 1350 | 1 | 286 | 296 |  |
| total 1 peptides |
| --- |

tr|A0A5N5T493|A0A5N5T493\_9CRUS

back to list

  

| Protein Coverage
| Supporting Peptides
|

Protein Coverage:

Supporting Peptides:

| Peptide | Uniq | -10lgP | Mass | ppm | m/z | z | RT | Scan | #Spec | Start | End | PTM |
| --- | --- | --- | --- | --- | --- | --- | --- | --- | --- | --- | --- | --- |
| R.GDLGIEIPAEK.V | Y | 20.51 | 1140.6025 | 0.0 | 571.3085 | 2 | 11.94 | 1350 | 1 | 390 | 400 |  |
| total 1 peptides |
| --- |

tr|A0A6P4EYV9|A0A6P4EYV9\_DRORH

back to list

  

| Protein Coverage
| Supporting Peptides
|

Protein Coverage:

Supporting Peptides:

| Peptide | Uniq | -10lgP | Mass | ppm | m/z | z | RT | Scan | #Spec | Start | End | PTM |
| --- | --- | --- | --- | --- | --- | --- | --- | --- | --- | --- | --- | --- |
| T.IVETSIITQSVE.P | Y | 20.46 | 1317.7028 | -4.3 | 659.8558 | 2 | 14.85 | 1838 | 1 | 9452 | 9463 |  |
| total 1 peptides |
| --- |

---

Prepared with PEAKS ™ (bioinfor.com)
