# Supplementary figures and images for: Proteomic and Functional Characterization of Antimicrobial Peptides Derived from Fisheries Bycatch via Enzymatic Hydrolysis (part 2 of 2)
Source: Mar Drugs. 2026 Jan 10;24(1):36. doi: 10.3390/md24010036 (PMC12843163; doi:10.3390/md24010036)

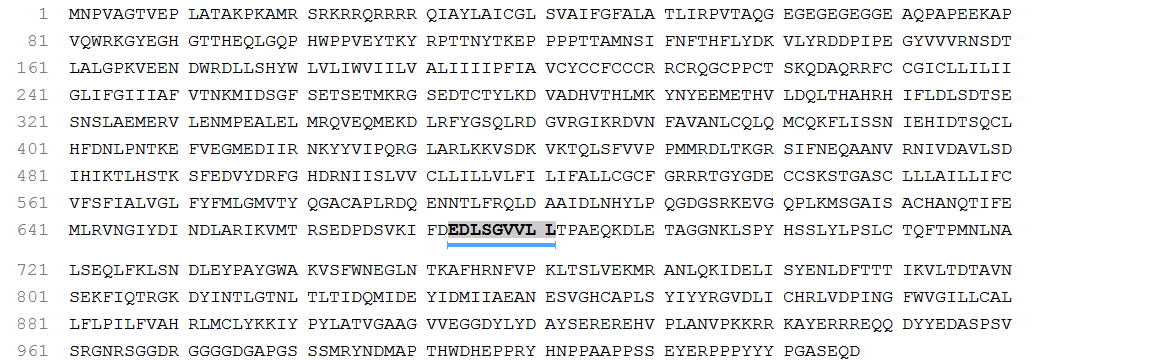

Supplement: Supplementary file 1 [file marinedrugs-24-00036-s001.zip › SM38/HA/img/cov_53539.png]

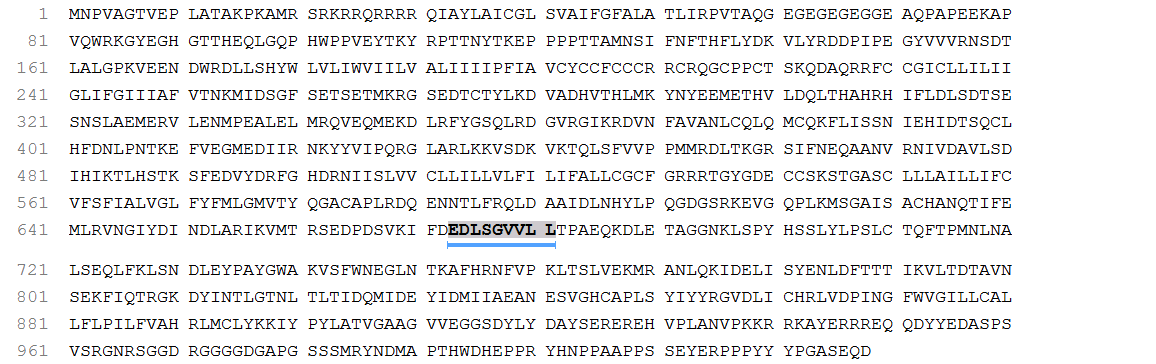

Supplement: Supplementary file 1 [file marinedrugs-24-00036-s001.zip › SM38/HA/img/cov_53540.png]

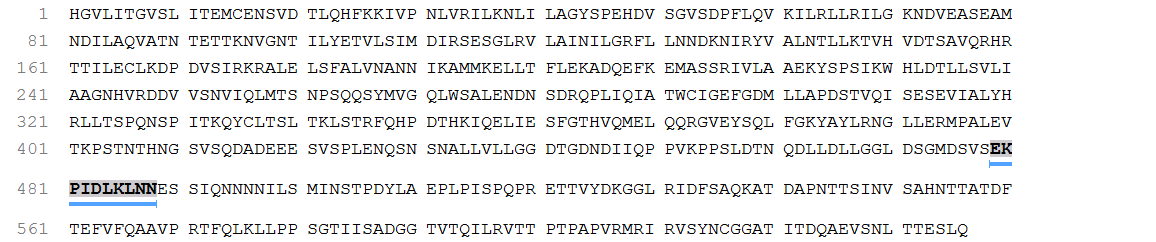

Supplement: Supplementary file 1 [file marinedrugs-24-00036-s001.zip › SM38/HA/img/cov_53541.png]

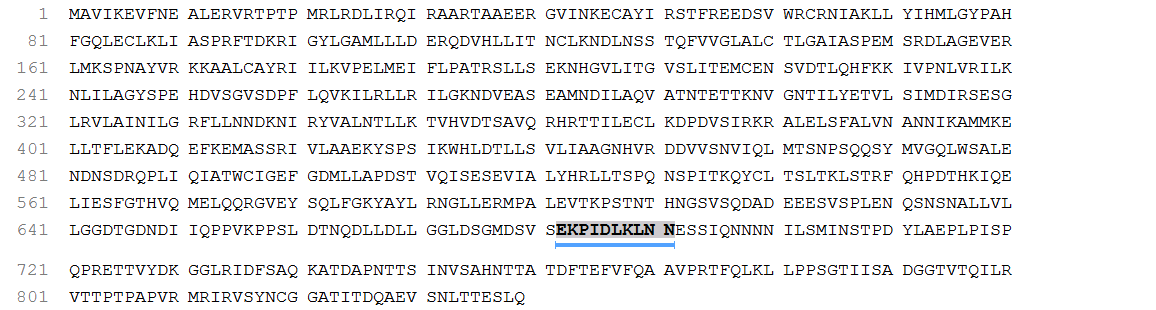

Supplement: Supplementary file 1 [file marinedrugs-24-00036-s001.zip › SM38/HA/img/cov_53542.png]

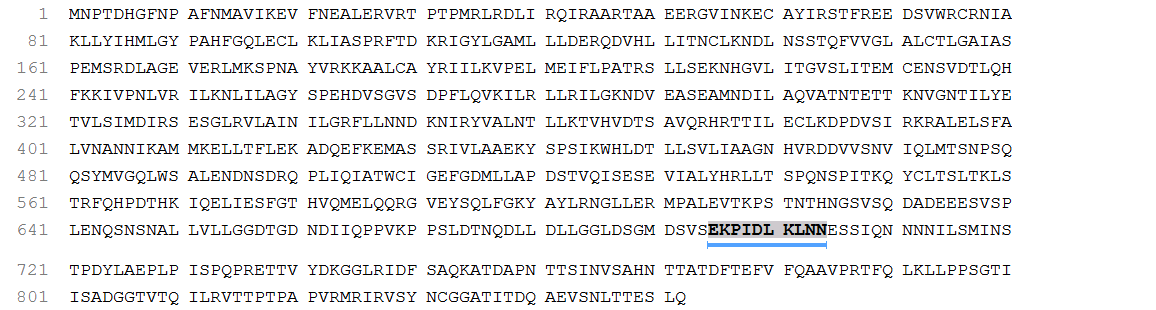

Supplement: Supplementary file 1 [file marinedrugs-24-00036-s001.zip › SM38/HA/img/cov_53543.png]

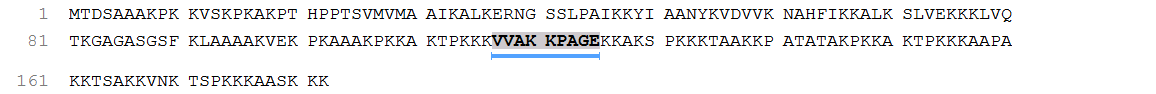

Supplement: Supplementary file 1 [file marinedrugs-24-00036-s001.zip › SM38/HA/img/cov_53551.png]

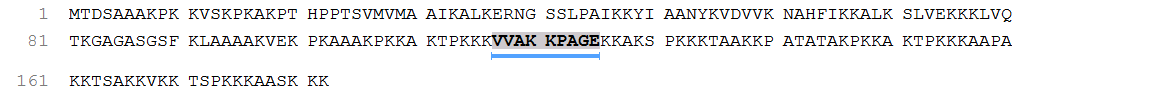

Supplement: Supplementary file 1 [file marinedrugs-24-00036-s001.zip › SM38/HA/img/cov_53552.png]

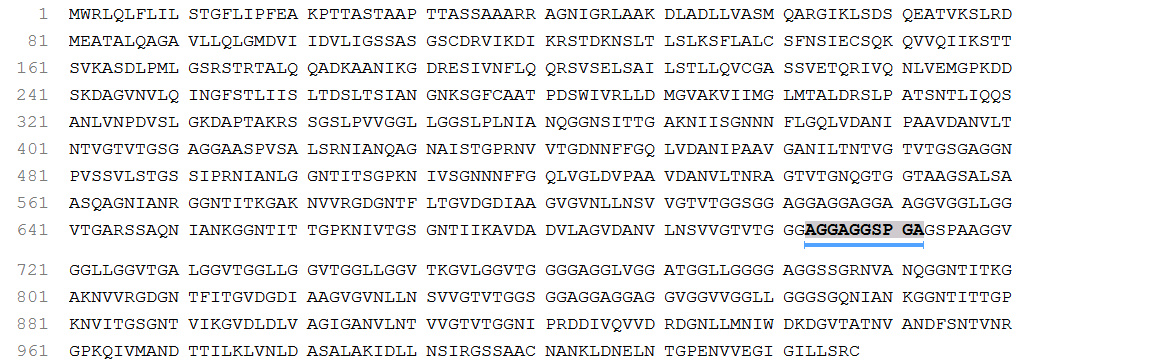

Supplement: Supplementary file 1 [file marinedrugs-24-00036-s001.zip › SM38/HA/img/cov_53553.png]

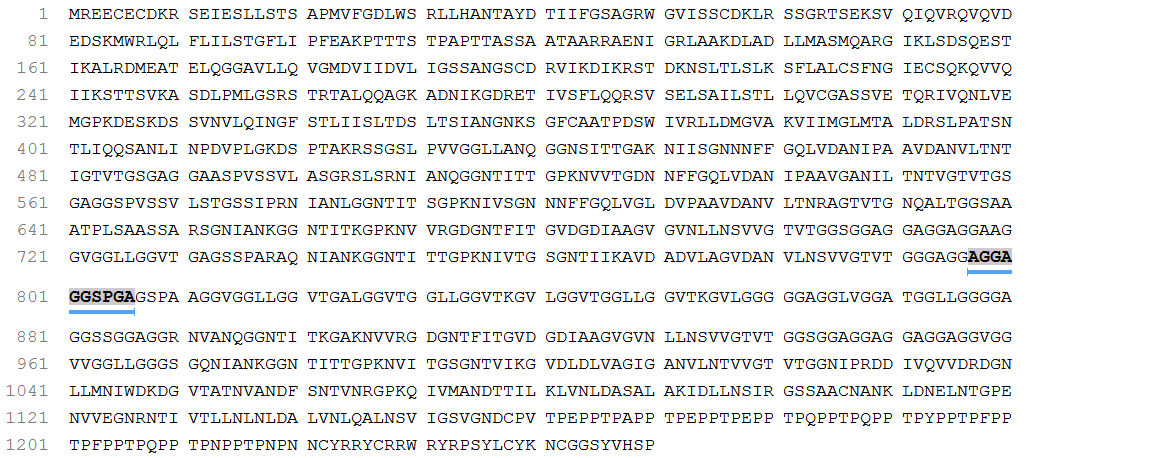

Supplement: Supplementary file 1 [file marinedrugs-24-00036-s001.zip › SM38/HA/img/cov_53554.png]

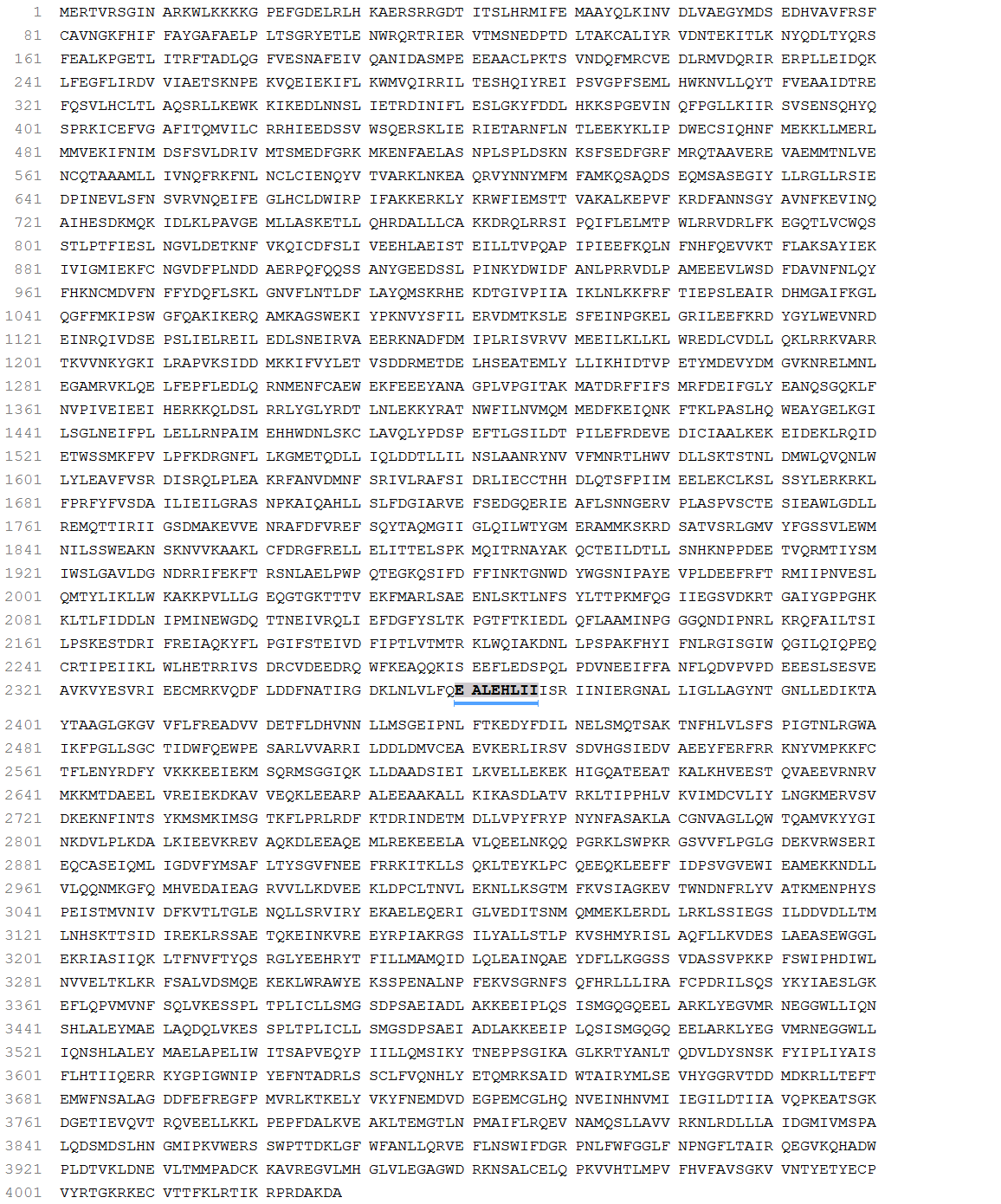

Supplement: Supplementary file 1 [file marinedrugs-24-00036-s001.zip › SM38/HA/img/cov_53595.png]

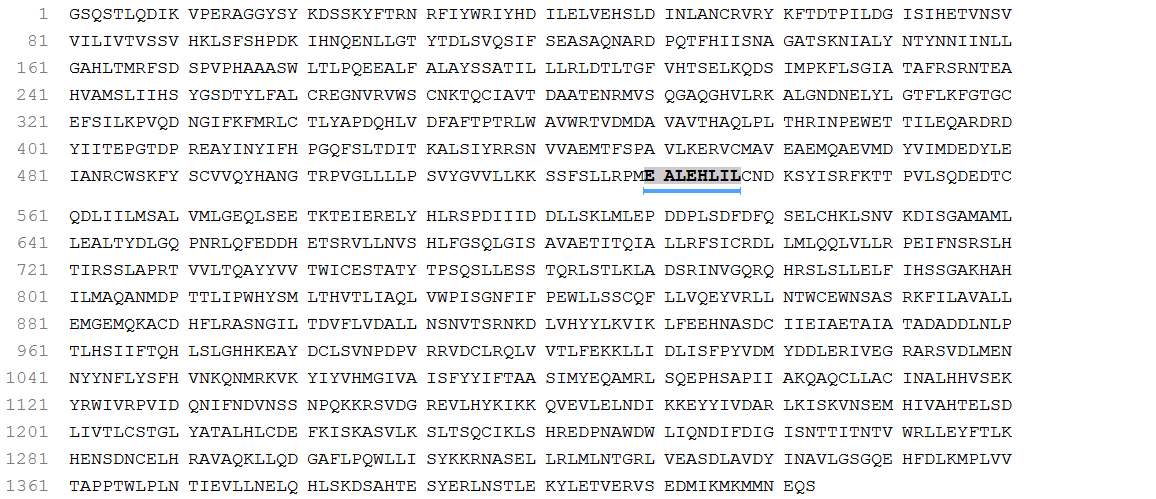

Supplement: Supplementary file 1 [file marinedrugs-24-00036-s001.zip › SM38/HA/img/cov_53604.png]

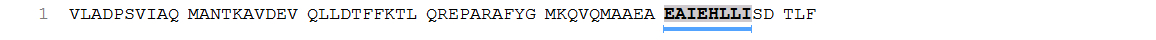

Supplement: Supplementary file 1 [file marinedrugs-24-00036-s001.zip › SM38/HA/img/cov_53606.png]

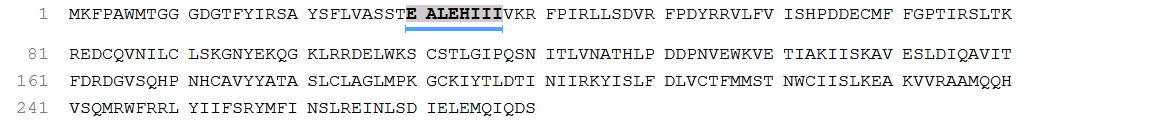

Supplement: Supplementary file 1 [file marinedrugs-24-00036-s001.zip › SM38/HA/img/cov_53607.png]

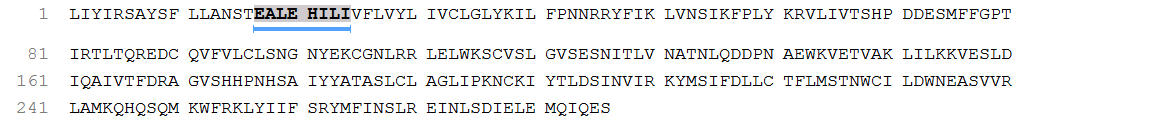

Supplement: Supplementary file 1 [file marinedrugs-24-00036-s001.zip › SM38/HA/img/cov_53608.png]

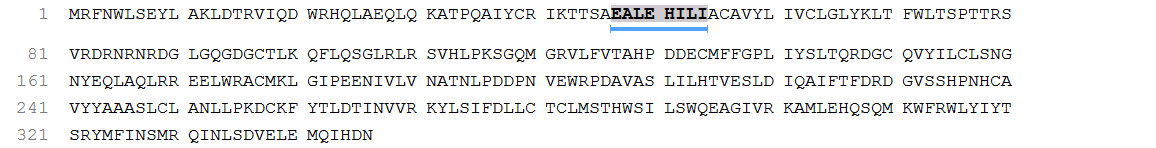

Supplement: Supplementary file 1 [file marinedrugs-24-00036-s001.zip › SM38/HA/img/cov_53609.png]

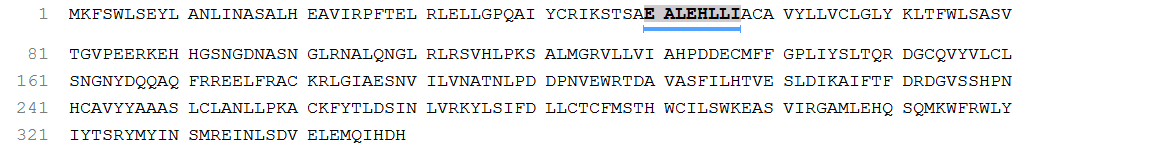

Supplement: Supplementary file 1 [file marinedrugs-24-00036-s001.zip › SM38/HA/img/cov_53610.png]

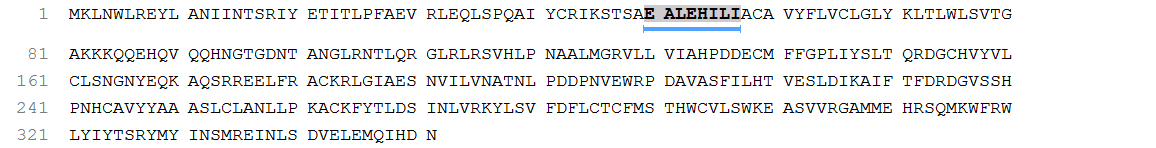

Supplement: Supplementary file 1 [file marinedrugs-24-00036-s001.zip › SM38/HA/img/cov_53611.png]

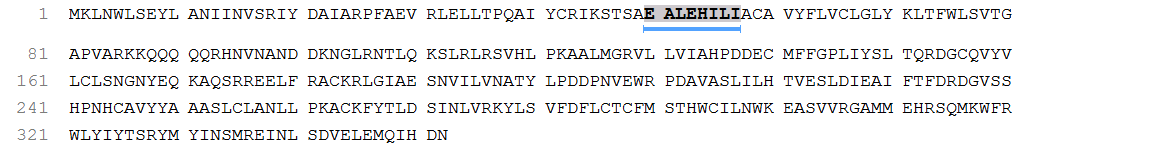

Supplement: Supplementary file 1 [file marinedrugs-24-00036-s001.zip › SM38/HA/img/cov_53612.png]

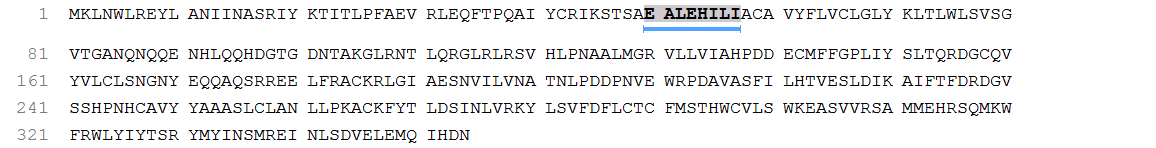

Supplement: Supplementary file 1 [file marinedrugs-24-00036-s001.zip › SM38/HA/img/cov_53613.png]

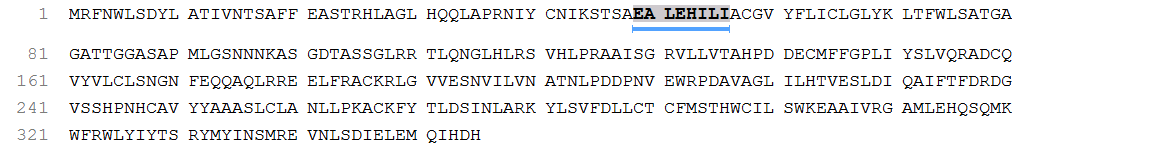

Supplement: Supplementary file 1 [file marinedrugs-24-00036-s001.zip › SM38/HA/img/cov_53614.png]

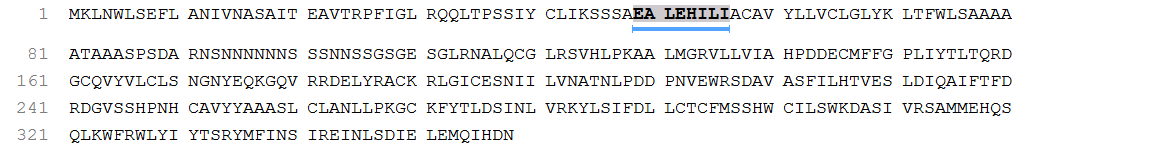

Supplement: Supplementary file 1 [file marinedrugs-24-00036-s001.zip › SM38/HA/img/cov_53615.png]

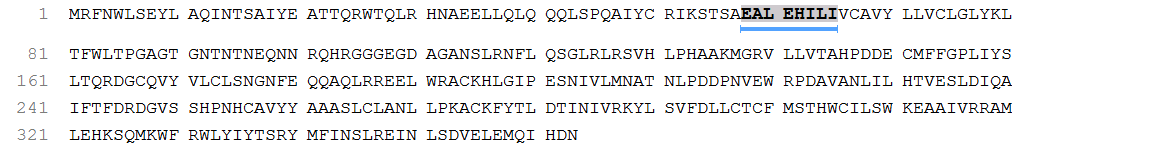

Supplement: Supplementary file 1 [file marinedrugs-24-00036-s001.zip › SM38/HA/img/cov_53616.png]

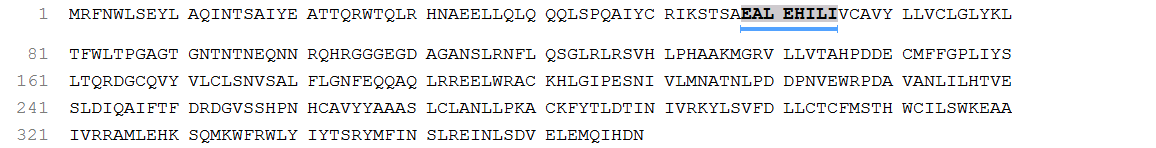

Supplement: Supplementary file 1 [file marinedrugs-24-00036-s001.zip › SM38/HA/img/cov_53617.png]

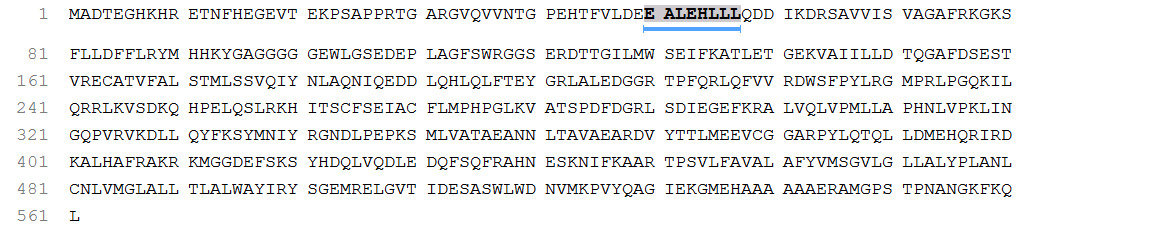

Supplement: Supplementary file 1 [file marinedrugs-24-00036-s001.zip › SM38/HA/img/cov_53618.png]

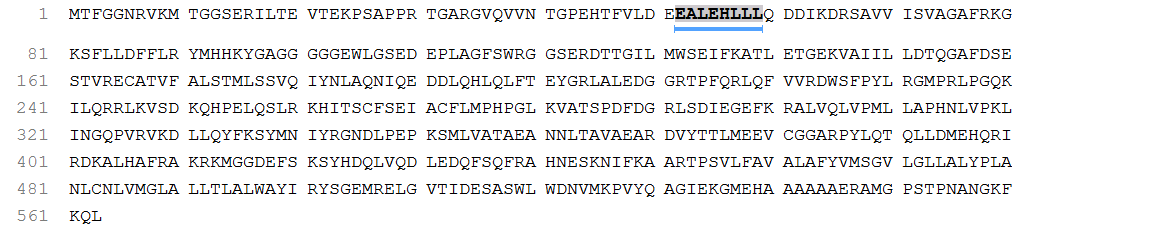

Supplement: Supplementary file 1 [file marinedrugs-24-00036-s001.zip › SM38/HA/img/cov_53619.png]

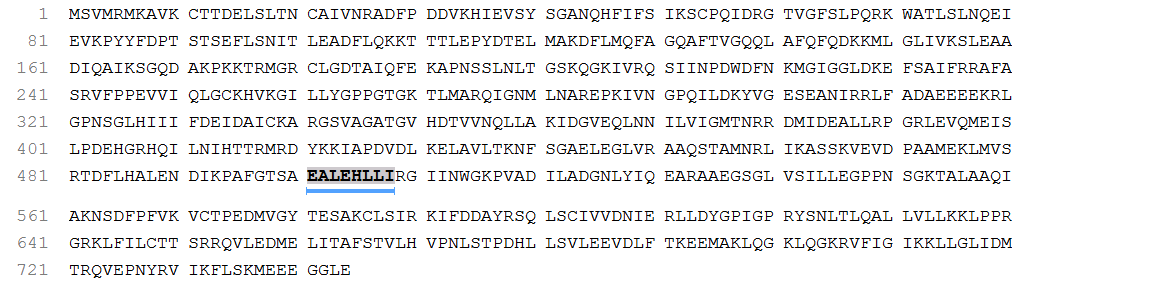

Supplement: Supplementary file 1 [file marinedrugs-24-00036-s001.zip › SM38/HA/img/cov_53620.png]

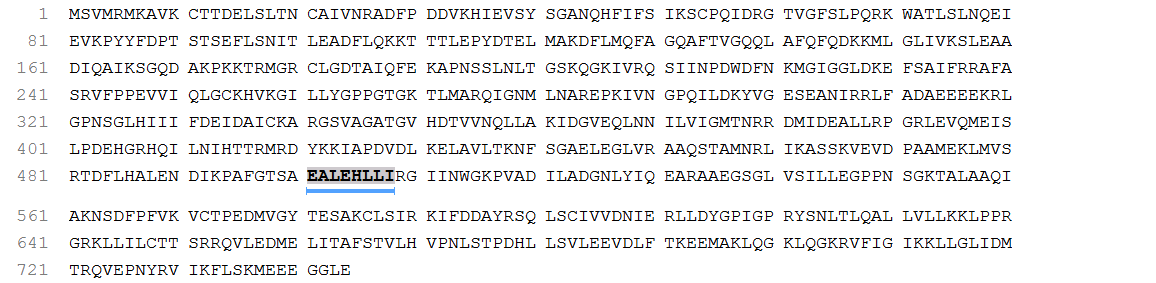

Supplement: Supplementary file 1 [file marinedrugs-24-00036-s001.zip › SM38/HA/img/cov_53621.png]

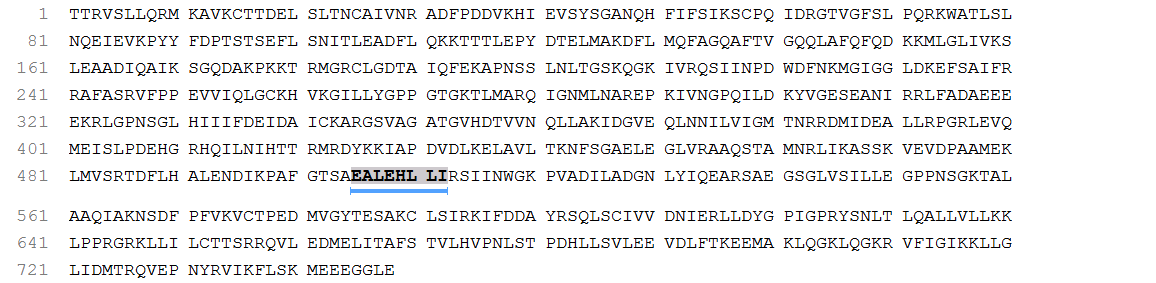

Supplement: Supplementary file 1 [file marinedrugs-24-00036-s001.zip › SM38/HA/img/cov_53622.png]

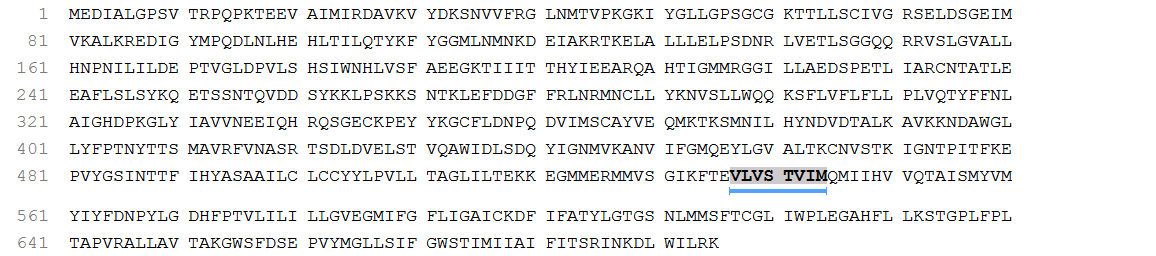

Supplement: Supplementary file 1 [file marinedrugs-24-00036-s001.zip › SM38/HA/img/cov_53623.png]

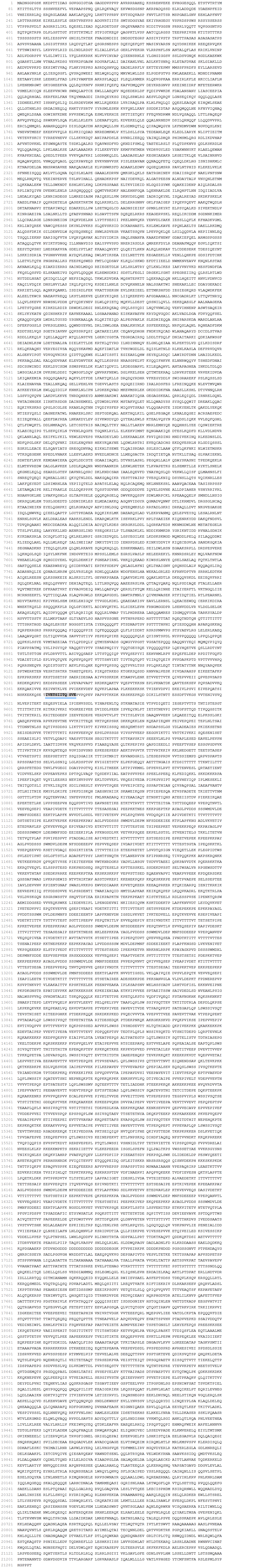

Supplement: Supplementary file 1 [file marinedrugs-24-00036-s001.zip › SM38/HA/img/cov_53624.png]

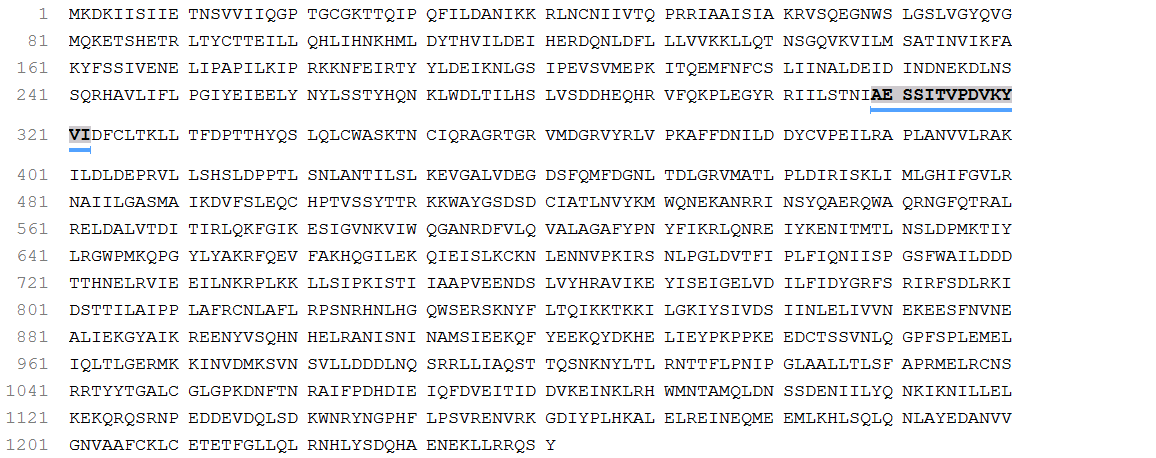

Supplement: Supplementary file 1 [file marinedrugs-24-00036-s001.zip › SM38/HA/img/cov_53645.png]

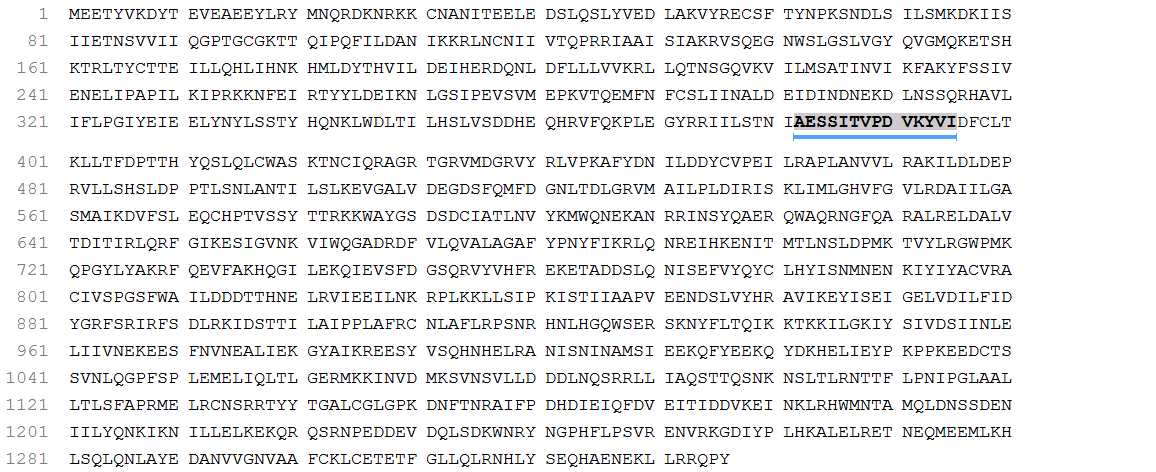

Supplement: Supplementary file 1 [file marinedrugs-24-00036-s001.zip › SM38/HA/img/cov_53646.png]

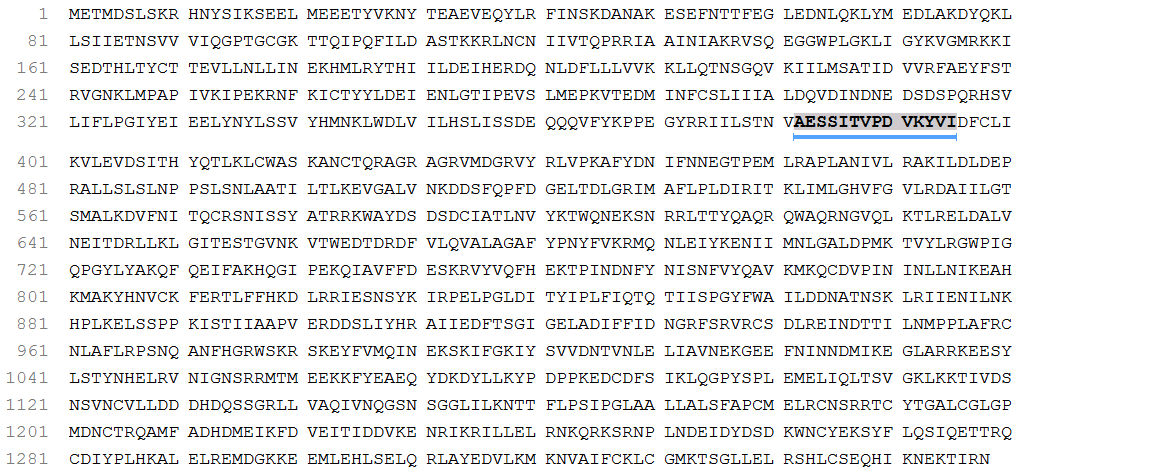

Supplement: Supplementary file 1 [file marinedrugs-24-00036-s001.zip › SM38/HA/img/cov_53647.png]

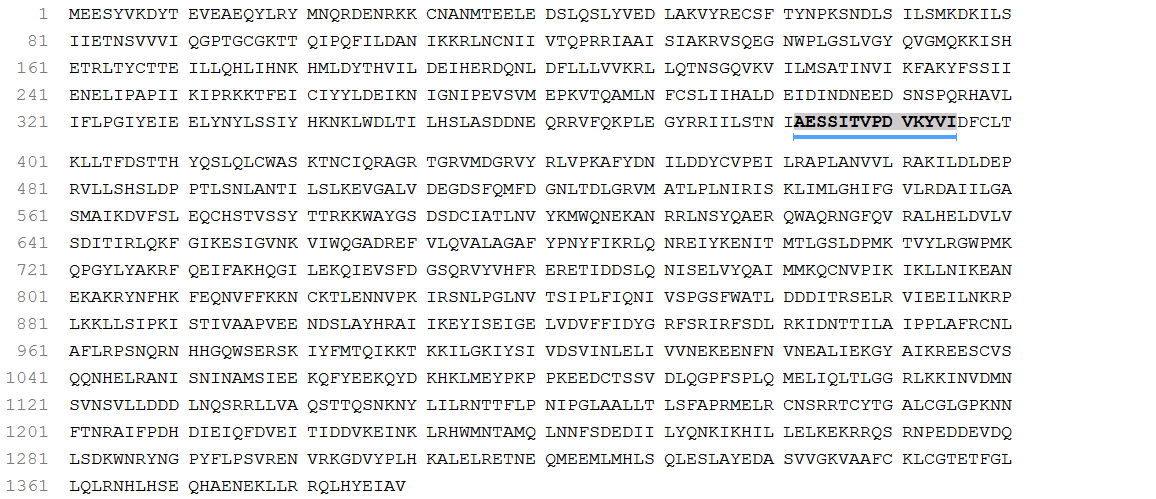

Supplement: Supplementary file 1 [file marinedrugs-24-00036-s001.zip › SM38/HA/img/cov_53648.png]

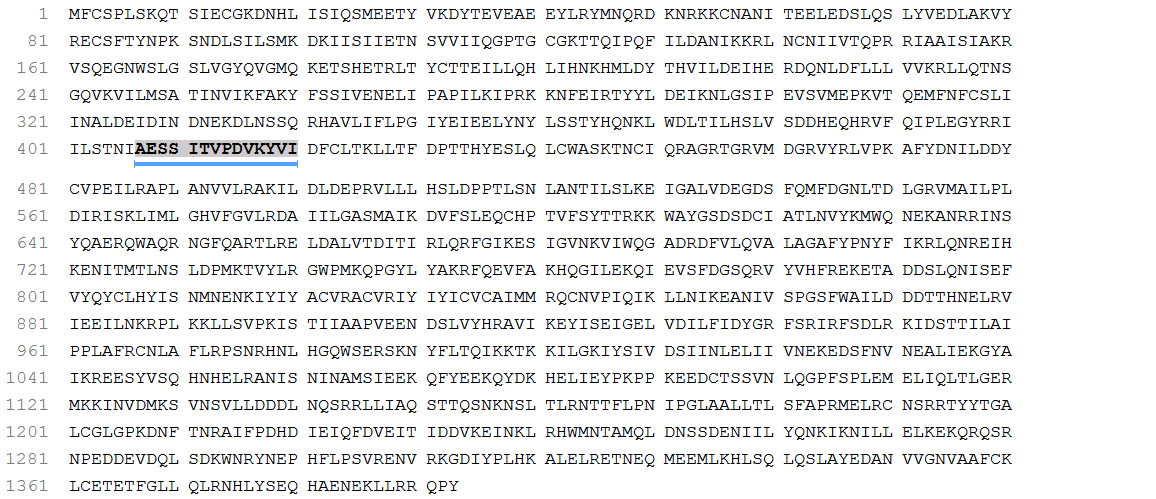

Supplement: Supplementary file 1 [file marinedrugs-24-00036-s001.zip › SM38/HA/img/cov_53649.png]

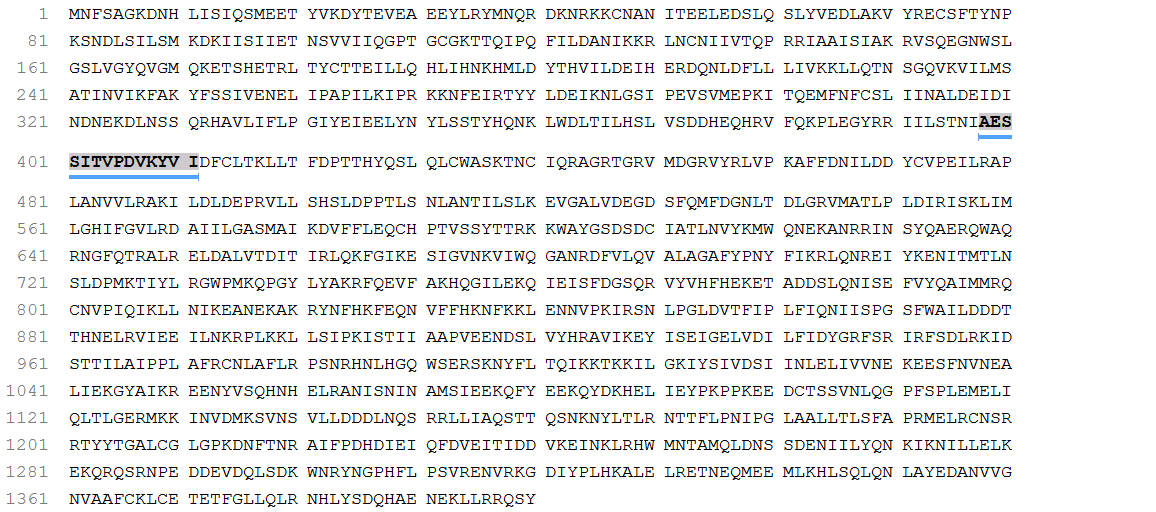

Supplement: Supplementary file 1 [file marinedrugs-24-00036-s001.zip › SM38/HA/img/cov_53650.png]

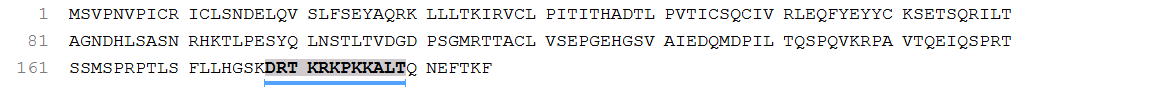

Supplement: Supplementary file 1 [file marinedrugs-24-00036-s001.zip › SM38/HA/img/cov_53655.png]

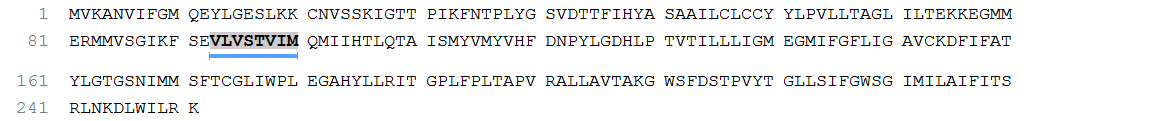

Supplement: Supplementary file 1 [file marinedrugs-24-00036-s001.zip › SM38/HA/img/cov_53656.png]

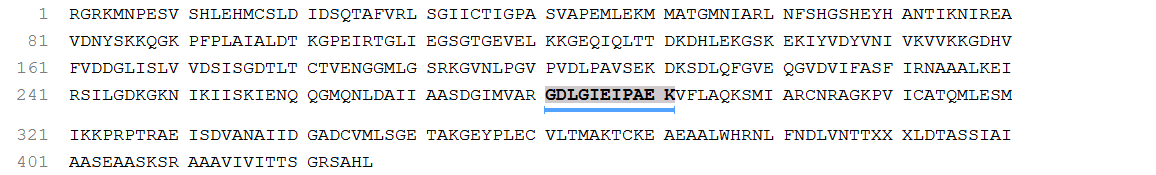

Supplement: Supplementary file 1 [file marinedrugs-24-00036-s001.zip › SM38/HA/img/cov_53657.png]

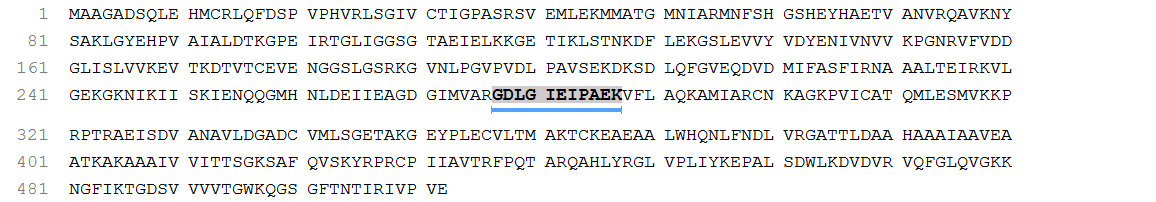

Supplement: Supplementary file 1 [file marinedrugs-24-00036-s001.zip › SM38/HA/img/cov_53658.png]

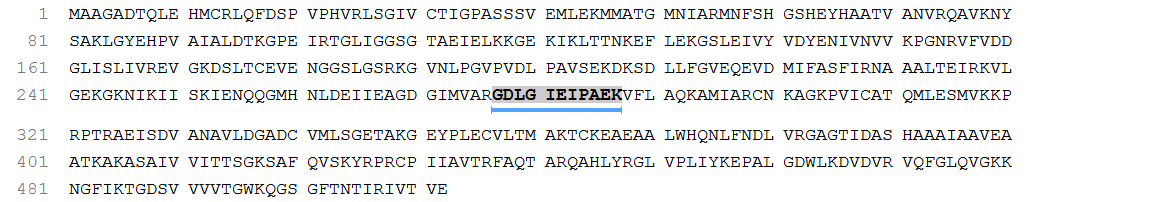

Supplement: Supplementary file 1 [file marinedrugs-24-00036-s001.zip › SM38/HA/img/cov_53659.png]

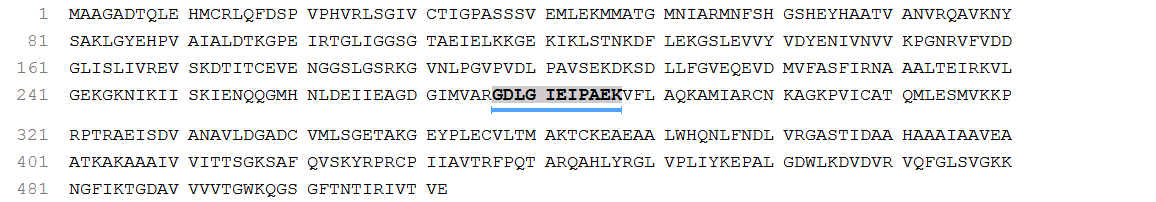

Supplement: Supplementary file 1 [file marinedrugs-24-00036-s001.zip › SM38/HA/img/cov_53660.png]

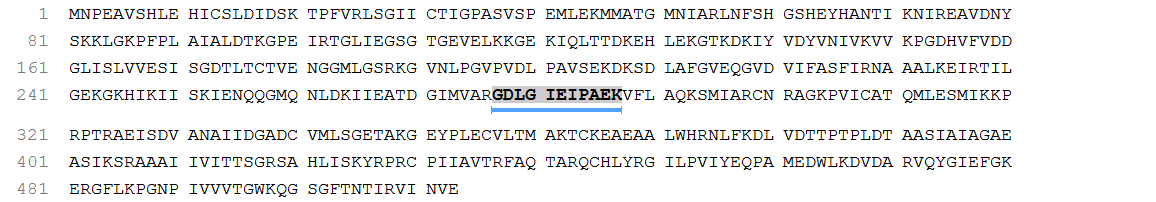

Supplement: Supplementary file 1 [file marinedrugs-24-00036-s001.zip › SM38/HA/img/cov_53661.png]

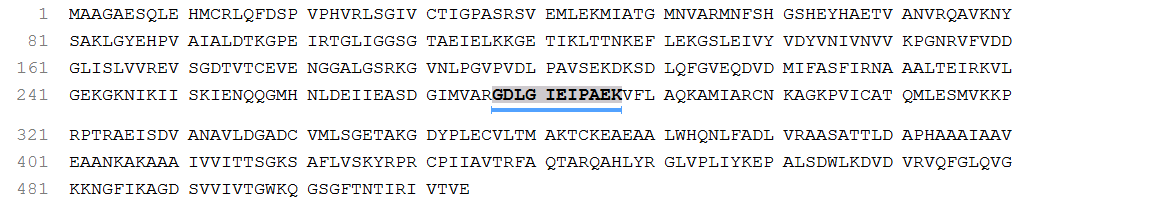

Supplement: Supplementary file 1 [file marinedrugs-24-00036-s001.zip › SM38/HA/img/cov_53662.png]

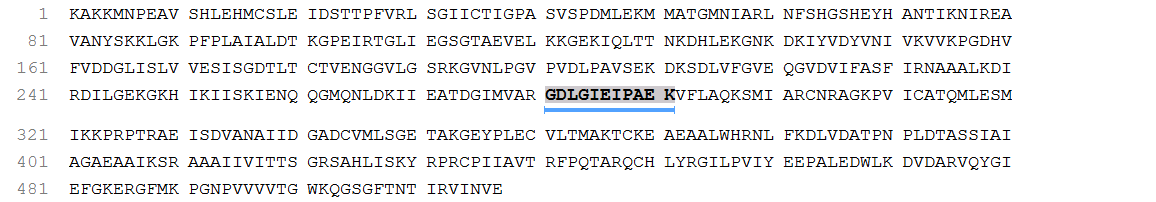

Supplement: Supplementary file 1 [file marinedrugs-24-00036-s001.zip › SM38/HA/img/cov_53663.png]

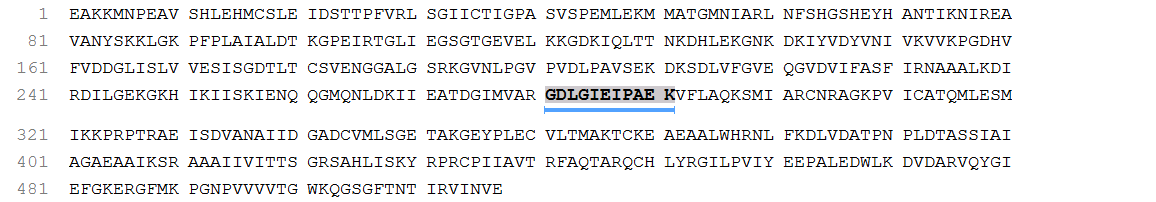

Supplement: Supplementary file 1 [file marinedrugs-24-00036-s001.zip › SM38/HA/img/cov_53664.png]

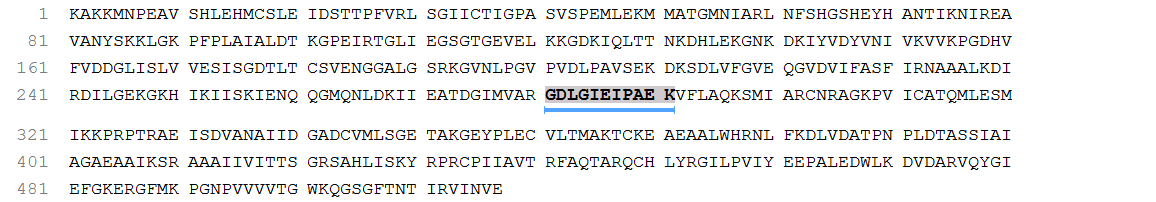

Supplement: Supplementary file 1 [file marinedrugs-24-00036-s001.zip › SM38/HA/img/cov_53665.png]

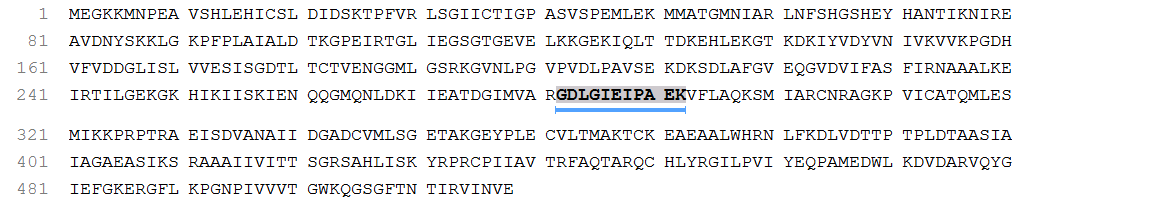

Supplement: Supplementary file 1 [file marinedrugs-24-00036-s001.zip › SM38/HA/img/cov_53666.png]

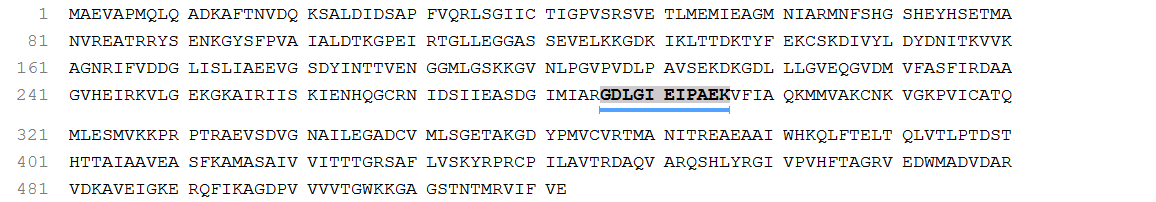

Supplement: Supplementary file 1 [file marinedrugs-24-00036-s001.zip › SM38/HA/img/cov_53667.png]

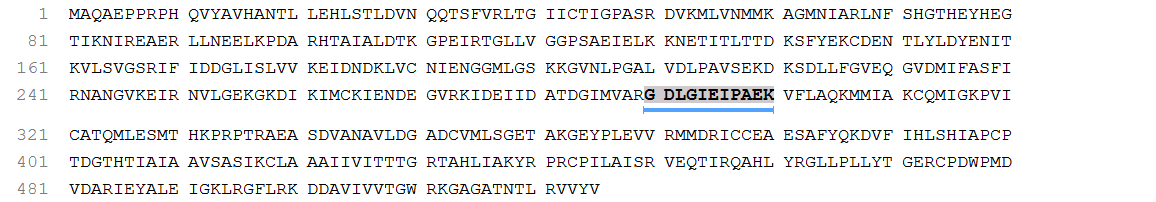

Supplement: Supplementary file 1 [file marinedrugs-24-00036-s001.zip › SM38/HA/img/cov_53668.png]

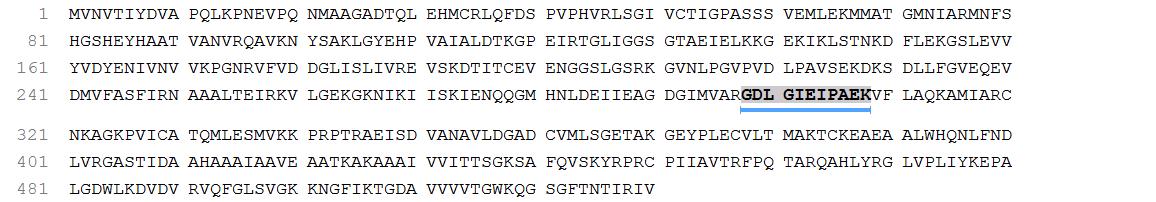

Supplement: Supplementary file 1 [file marinedrugs-24-00036-s001.zip › SM38/HA/img/cov_53669.png]

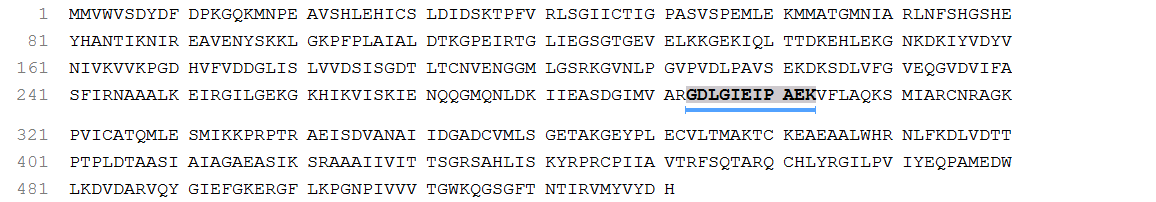

Supplement: Supplementary file 1 [file marinedrugs-24-00036-s001.zip › SM38/HA/img/cov_53670.png]

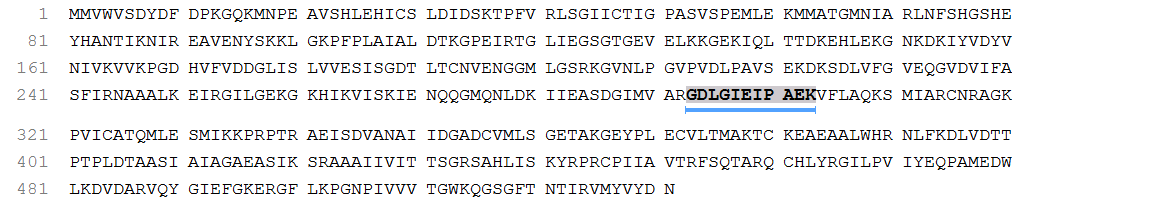

Supplement: Supplementary file 1 [file marinedrugs-24-00036-s001.zip › SM38/HA/img/cov_53671.png]

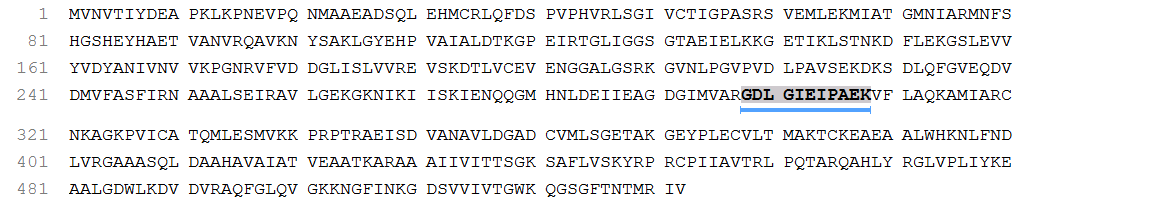

Supplement: Supplementary file 1 [file marinedrugs-24-00036-s001.zip › SM38/HA/img/cov_53672.png]

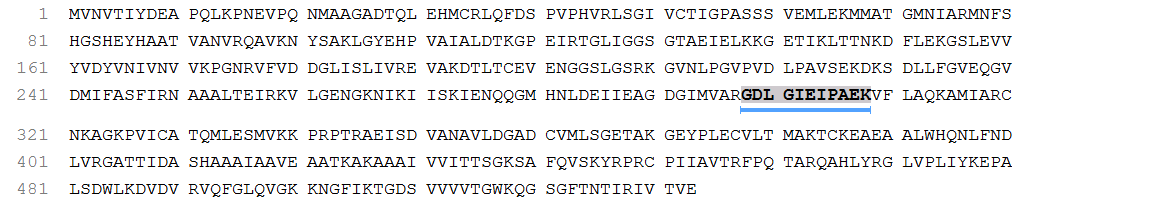

Supplement: Supplementary file 1 [file marinedrugs-24-00036-s001.zip › SM38/HA/img/cov_53673.png]

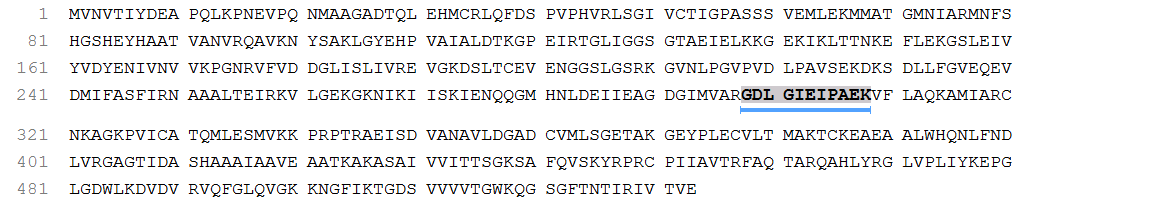

Supplement: Supplementary file 1 [file marinedrugs-24-00036-s001.zip › SM38/HA/img/cov_53674.png]

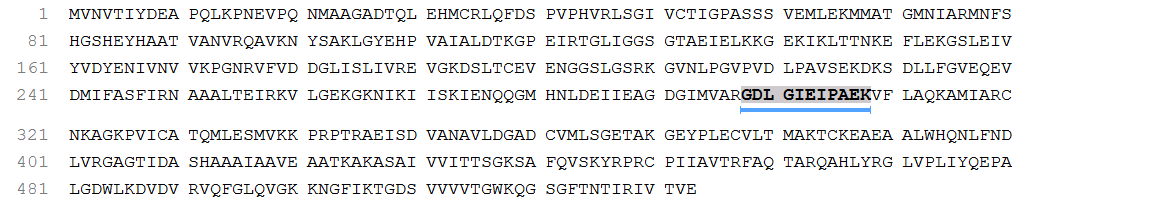

Supplement: Supplementary file 1 [file marinedrugs-24-00036-s001.zip › SM38/HA/img/cov_53675.png]

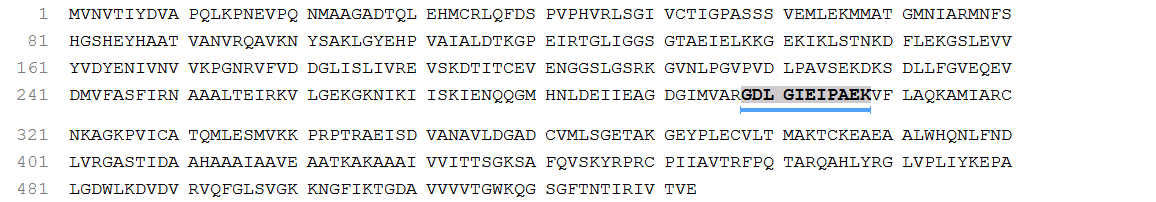

Supplement: Supplementary file 1 [file marinedrugs-24-00036-s001.zip › SM38/HA/img/cov_53676.png]

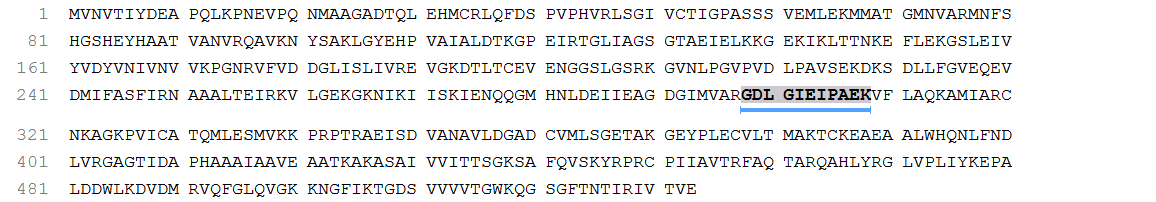

Supplement: Supplementary file 1 [file marinedrugs-24-00036-s001.zip › SM38/HA/img/cov_53677.png]

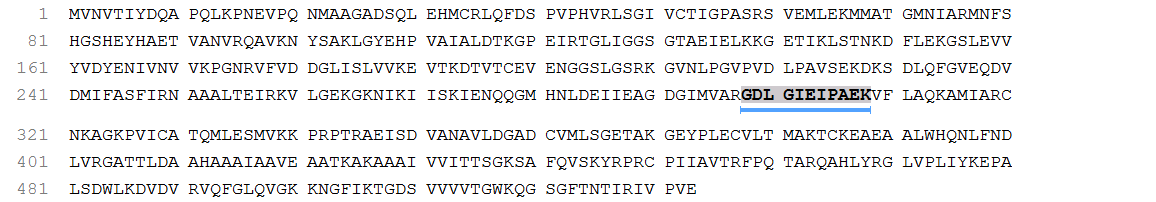

Supplement: Supplementary file 1 [file marinedrugs-24-00036-s001.zip › SM38/HA/img/cov_53678.png]

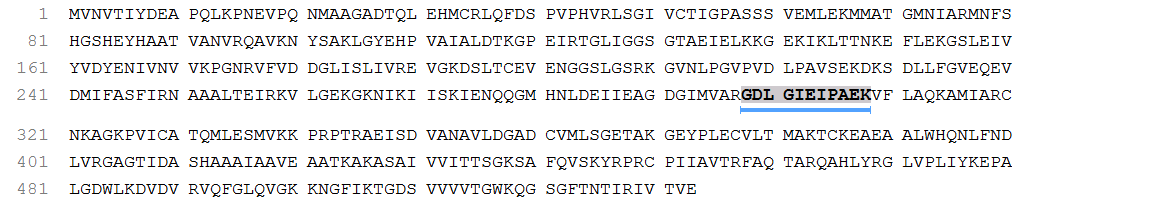

Supplement: Supplementary file 1 [file marinedrugs-24-00036-s001.zip › SM38/HA/img/cov_53679.png]

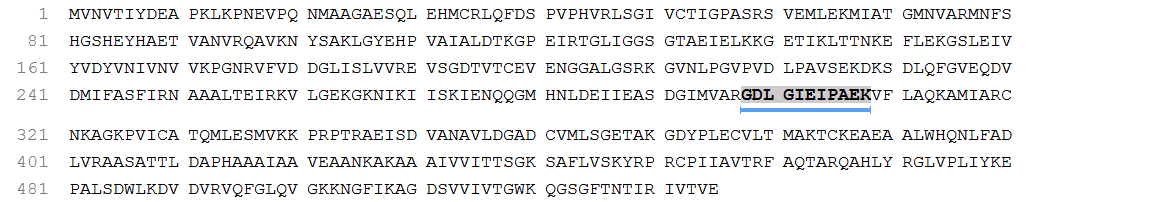

Supplement: Supplementary file 1 [file marinedrugs-24-00036-s001.zip › SM38/HA/img/cov_53680.png]

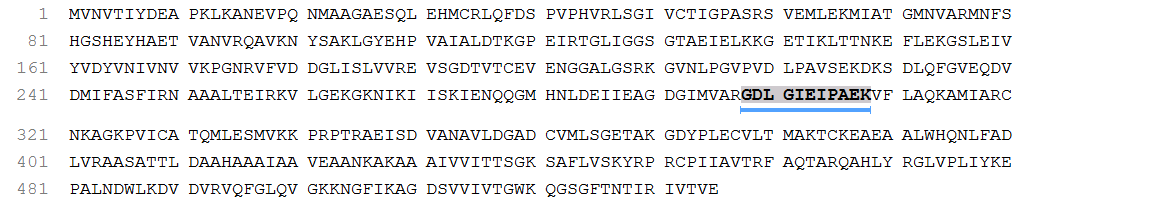

Supplement: Supplementary file 1 [file marinedrugs-24-00036-s001.zip › SM38/HA/img/cov_53681.png]

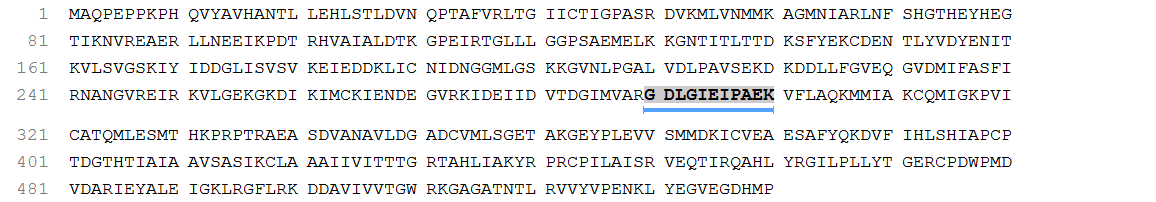

Supplement: Supplementary file 1 [file marinedrugs-24-00036-s001.zip › SM38/HA/img/cov_53682.png]

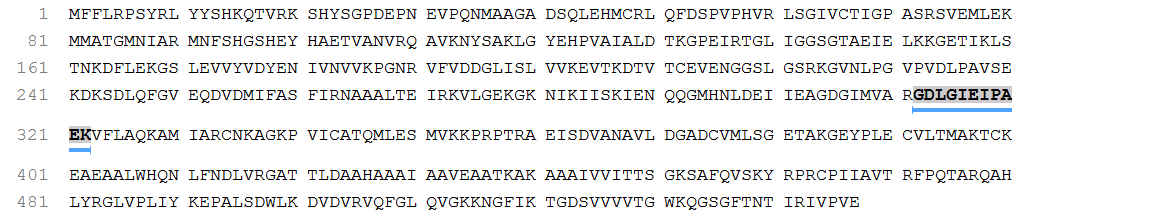

Supplement: Supplementary file 1 [file marinedrugs-24-00036-s001.zip › SM38/HA/img/cov_53683.png]

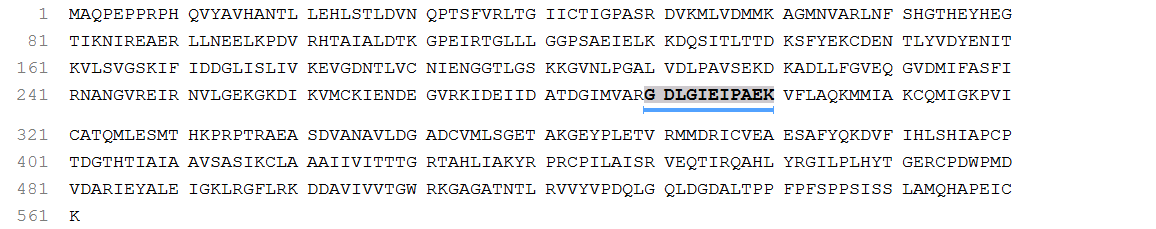

Supplement: Supplementary file 1 [file marinedrugs-24-00036-s001.zip › SM38/HA/img/cov_53684.png]

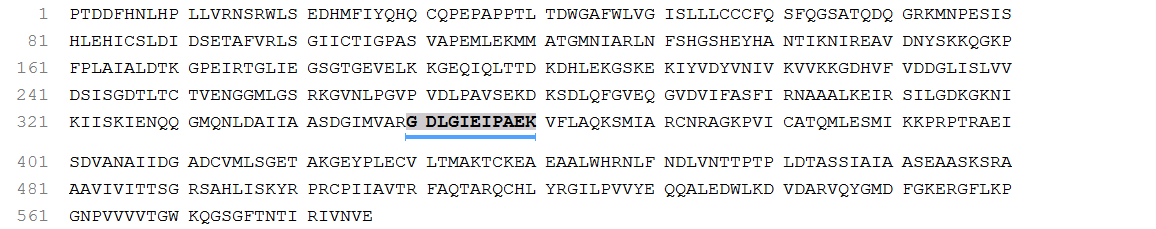

Supplement: Supplementary file 1 [file marinedrugs-24-00036-s001.zip › SM38/HA/img/cov_53685.png]

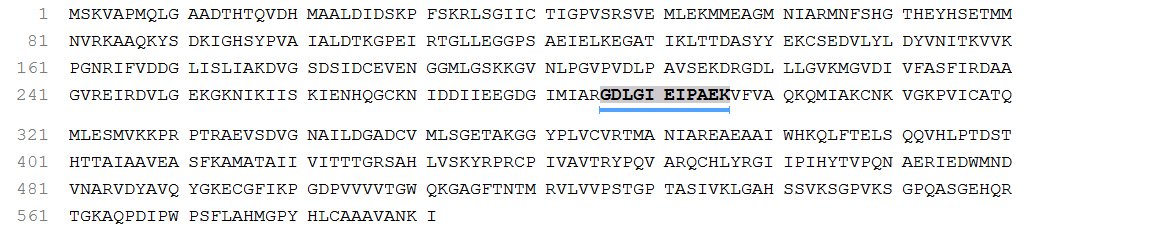

Supplement: Supplementary file 1 [file marinedrugs-24-00036-s001.zip › SM38/HA/img/cov_53686.png]

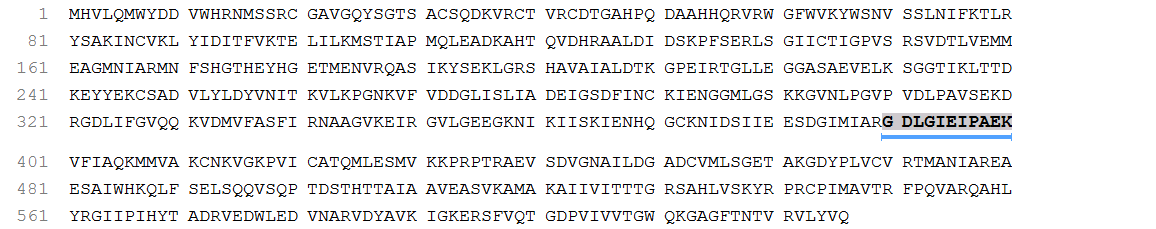

Supplement: Supplementary file 1 [file marinedrugs-24-00036-s001.zip › SM38/HA/img/cov_53687.png]

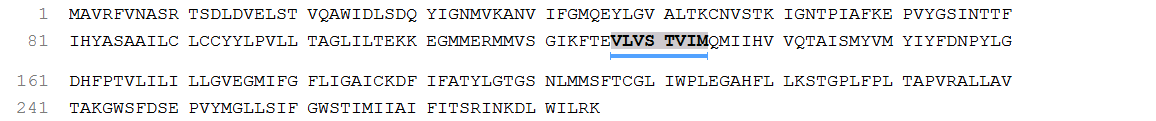

Supplement: Supplementary file 1 [file marinedrugs-24-00036-s001.zip › SM38/HA/img/cov_53688.png]

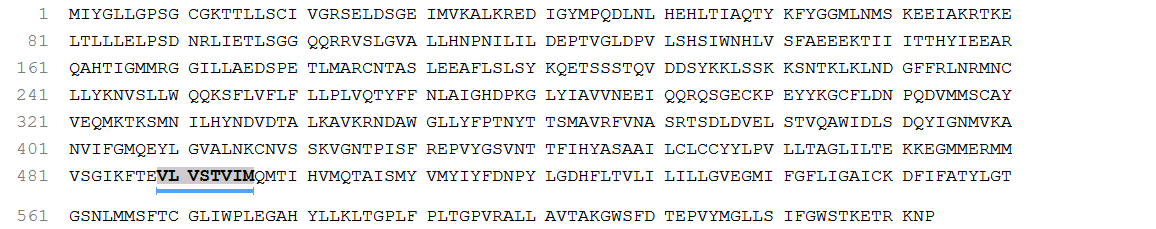

Supplement: Supplementary file 1 [file marinedrugs-24-00036-s001.zip › SM38/HA/img/cov_53689.png]

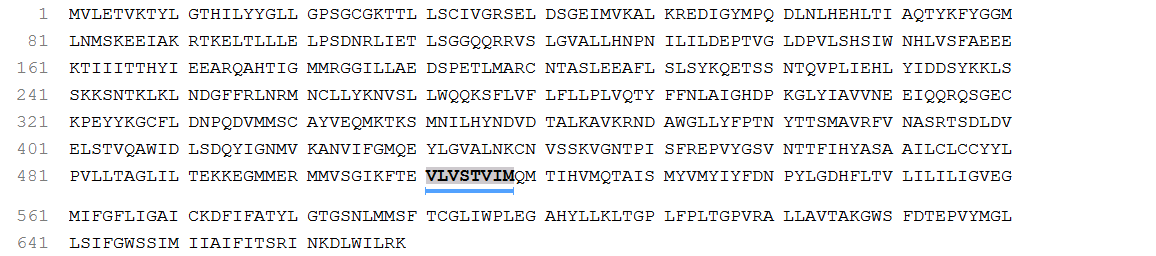

Supplement: Supplementary file 1 [file marinedrugs-24-00036-s001.zip › SM38/HA/img/cov_53690.png]

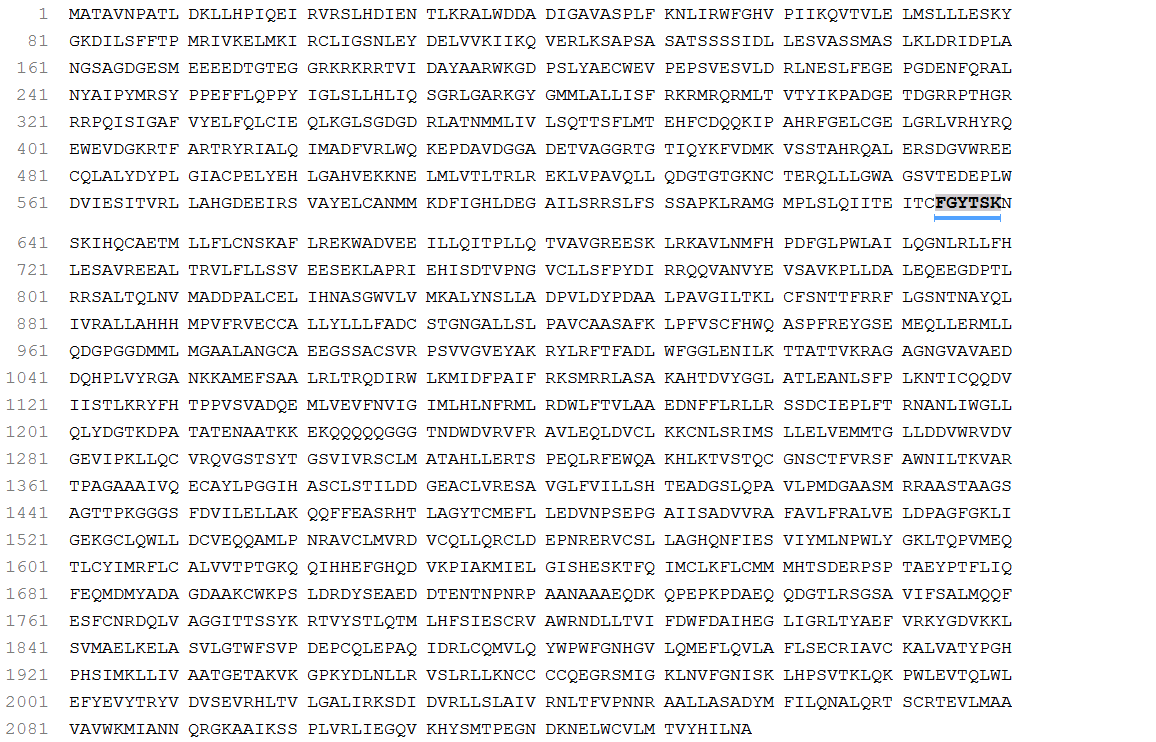

Supplement: Supplementary file 1 [file marinedrugs-24-00036-s001.zip › SM38/HA/img/cov_53716.png]

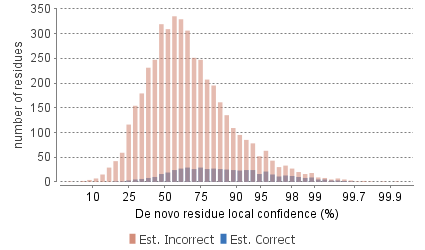

Supplement: Supplementary file 1 [file marinedrugs-24-00036-s001.zip › SM38/HA/img/DenovoOnlyLogisticHistogram4981813248977602963.png]

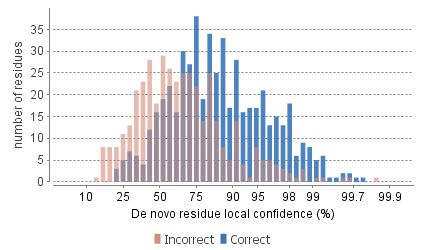

Supplement: Supplementary file 1 [file marinedrugs-24-00036-s001.zip › SM38/HA/img/DenovoVerifiedLogisticHistogram6796361207803708921.png]

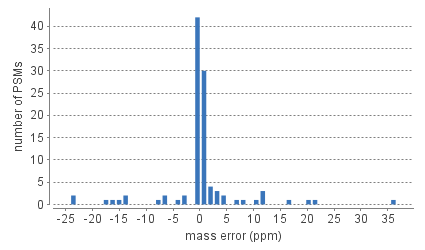

Supplement: Supplementary file 1 [file marinedrugs-24-00036-s001.zip › SM38/HA/img/ErrorHistogram7889217173053494950.png]

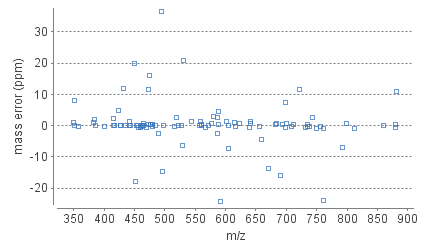

Supplement: Supplementary file 1 [file marinedrugs-24-00036-s001.zip › SM38/HA/img/ErrorPlotFigure5752970216640138921.png]

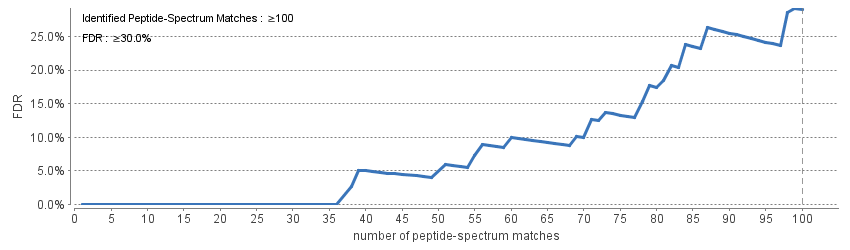

Supplement: Supplementary file 1 [file marinedrugs-24-00036-s001.zip › SM38/HA/img/FDRFigure6812499770690539697.png]

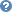

Supplement: Supplementary file 1 [file marinedrugs-24-00036-s001.zip › SM38/HA/img/q.png]

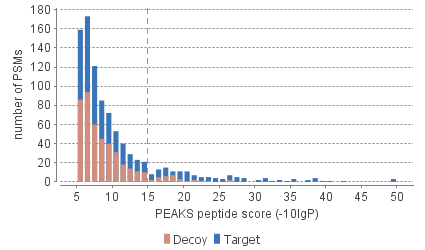

Supplement: Supplementary file 1 [file marinedrugs-24-00036-s001.zip › SM38/HA/img/ScoreHistogram1078101354087002879.png]

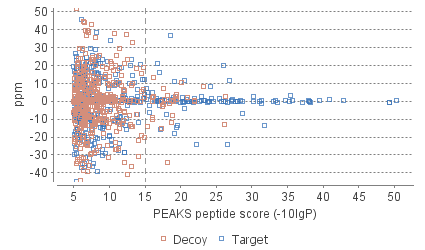

Supplement: Supplementary file 1 [file marinedrugs-24-00036-s001.zip › SM38/HA/img/ScorePlotFigure6260511389054329350.png]

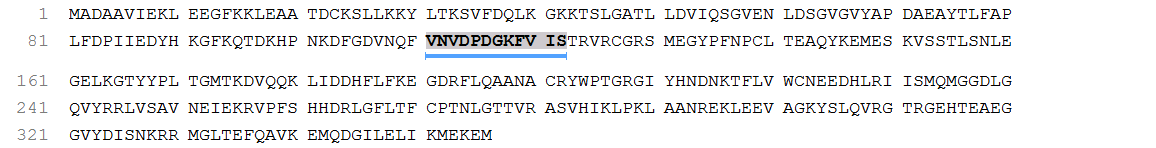

Supplement: Supplementary file 1 [file marinedrugs-24-00036-s001.zip › SM39/HP/img/cov_43542.png]

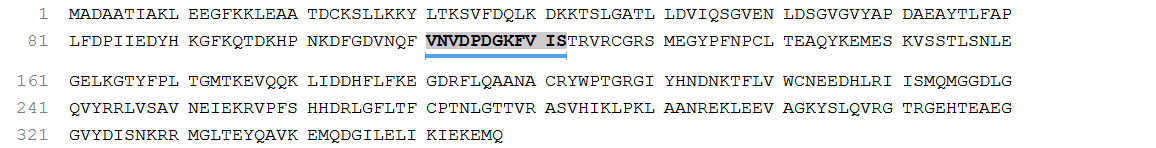

Supplement: Supplementary file 1 [file marinedrugs-24-00036-s001.zip › SM39/HP/img/cov_43543.png]

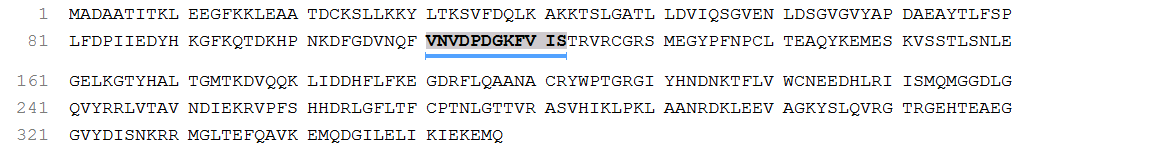

Supplement: Supplementary file 1 [file marinedrugs-24-00036-s001.zip › SM39/HP/img/cov_43544.png]

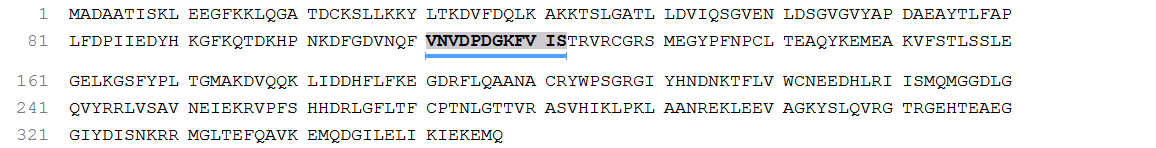

Supplement: Supplementary file 1 [file marinedrugs-24-00036-s001.zip › SM39/HP/img/cov_43545.png]

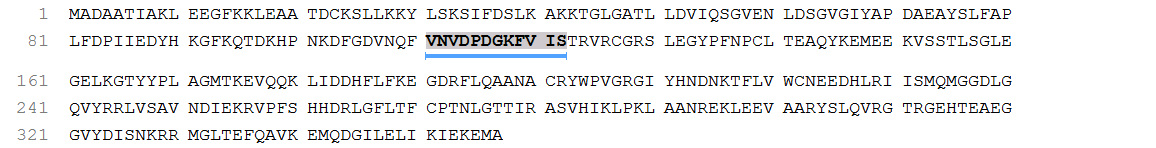

Supplement: Supplementary file 1 [file marinedrugs-24-00036-s001.zip › SM39/HP/img/cov_43546.png]

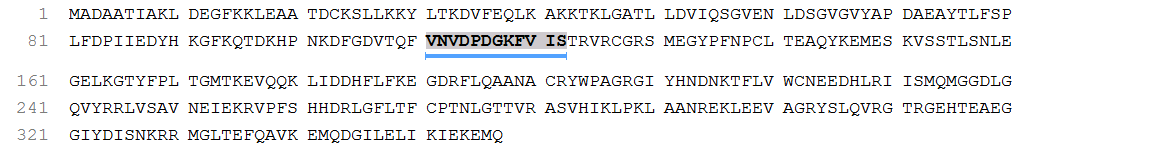

Supplement: Supplementary file 1 [file marinedrugs-24-00036-s001.zip › SM39/HP/img/cov_43548.png]

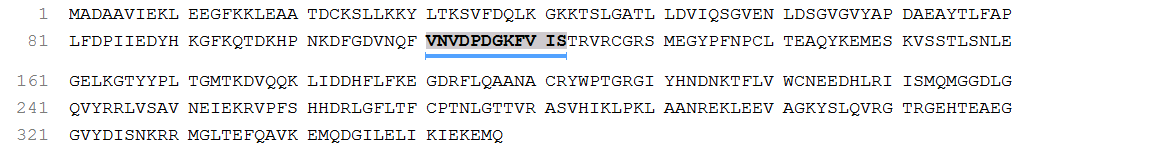

Supplement: Supplementary file 1 [file marinedrugs-24-00036-s001.zip › SM39/HP/img/cov_43549.png]

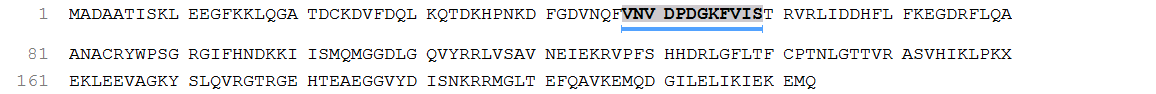

Supplement: Supplementary file 1 [file marinedrugs-24-00036-s001.zip › SM39/HP/img/cov_43554.png]

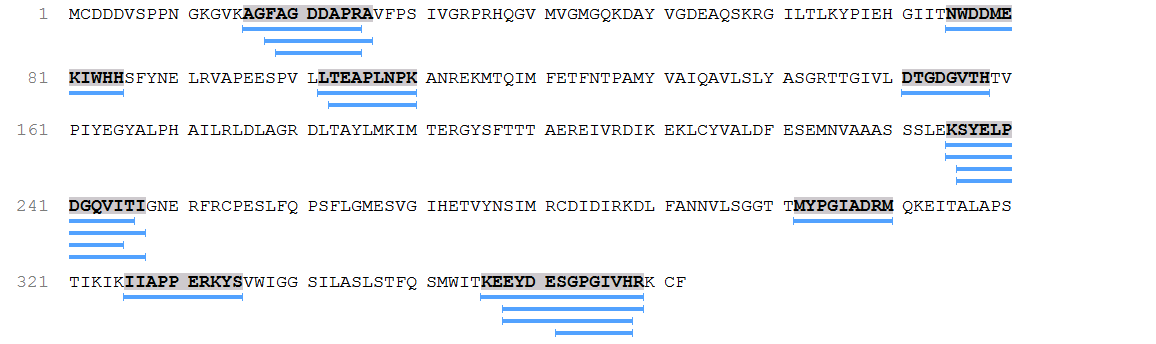

Supplement: Supplementary file 1 [file marinedrugs-24-00036-s001.zip › SM39/HP/img/cov_53234.png]

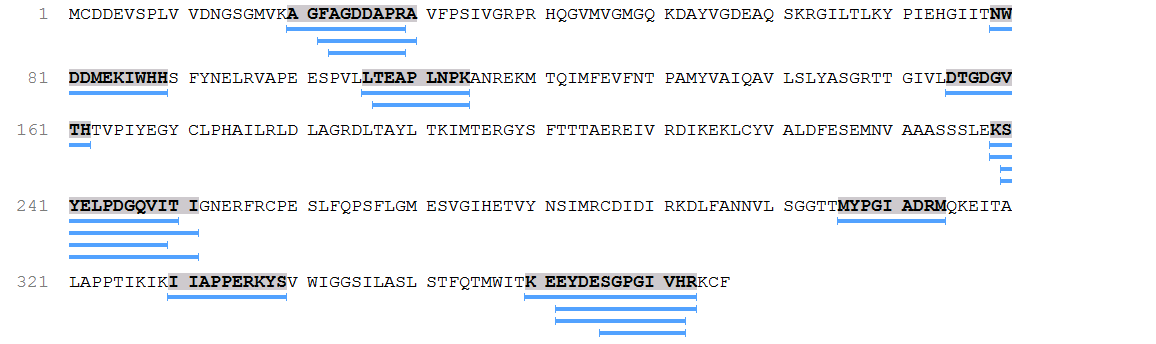

Supplement: Supplementary file 1 [file marinedrugs-24-00036-s001.zip › SM39/HP/img/cov_53236.png]

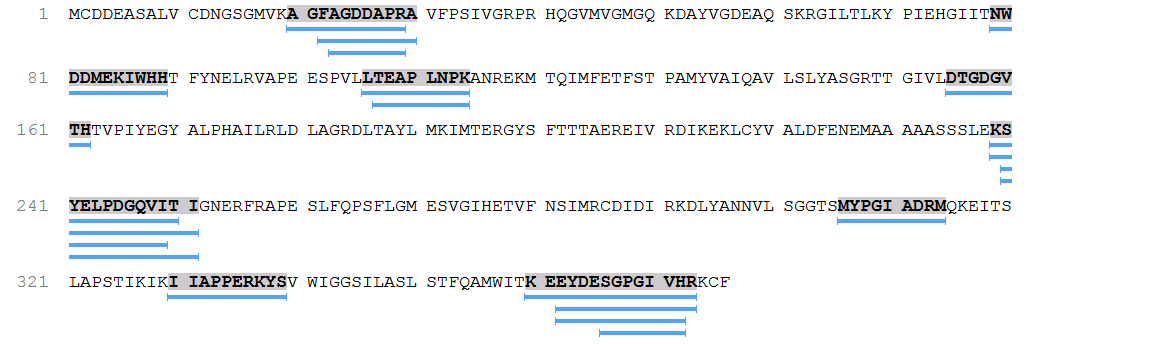

Supplement: Supplementary file 1 [file marinedrugs-24-00036-s001.zip › SM39/HP/img/cov_53237.png]

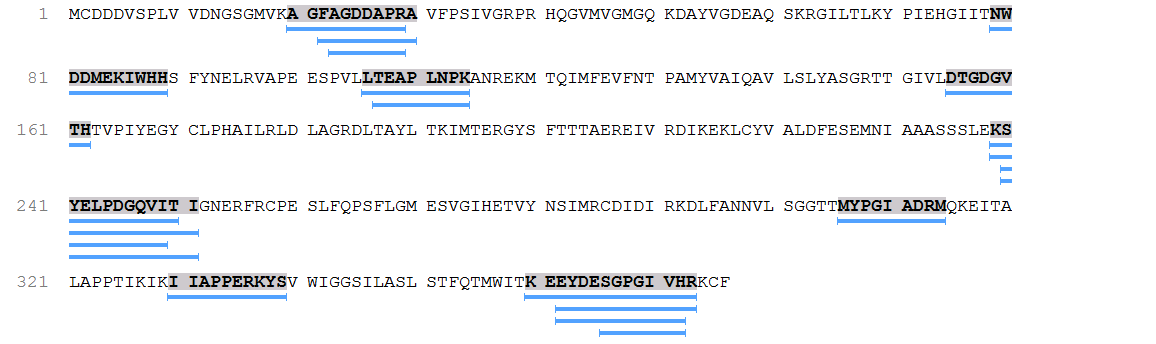

Supplement: Supplementary file 1 [file marinedrugs-24-00036-s001.zip › SM39/HP/img/cov_53239.png]

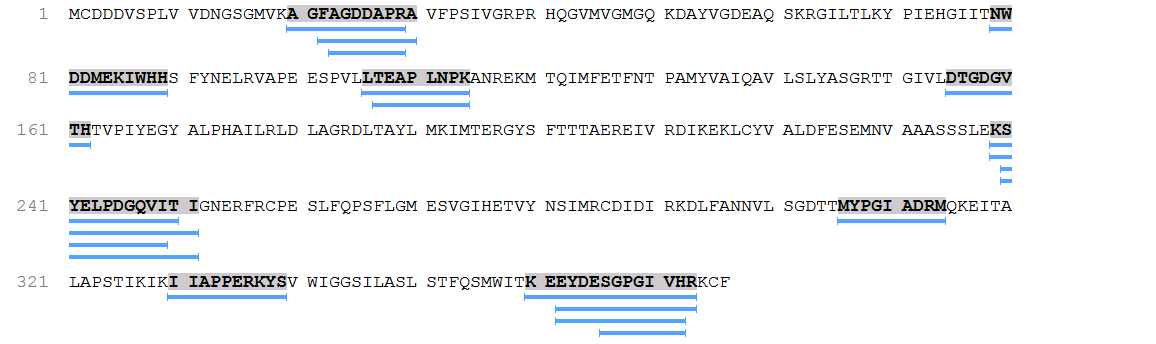

Supplement: Supplementary file 1 [file marinedrugs-24-00036-s001.zip › SM39/HP/img/cov_53241.png]

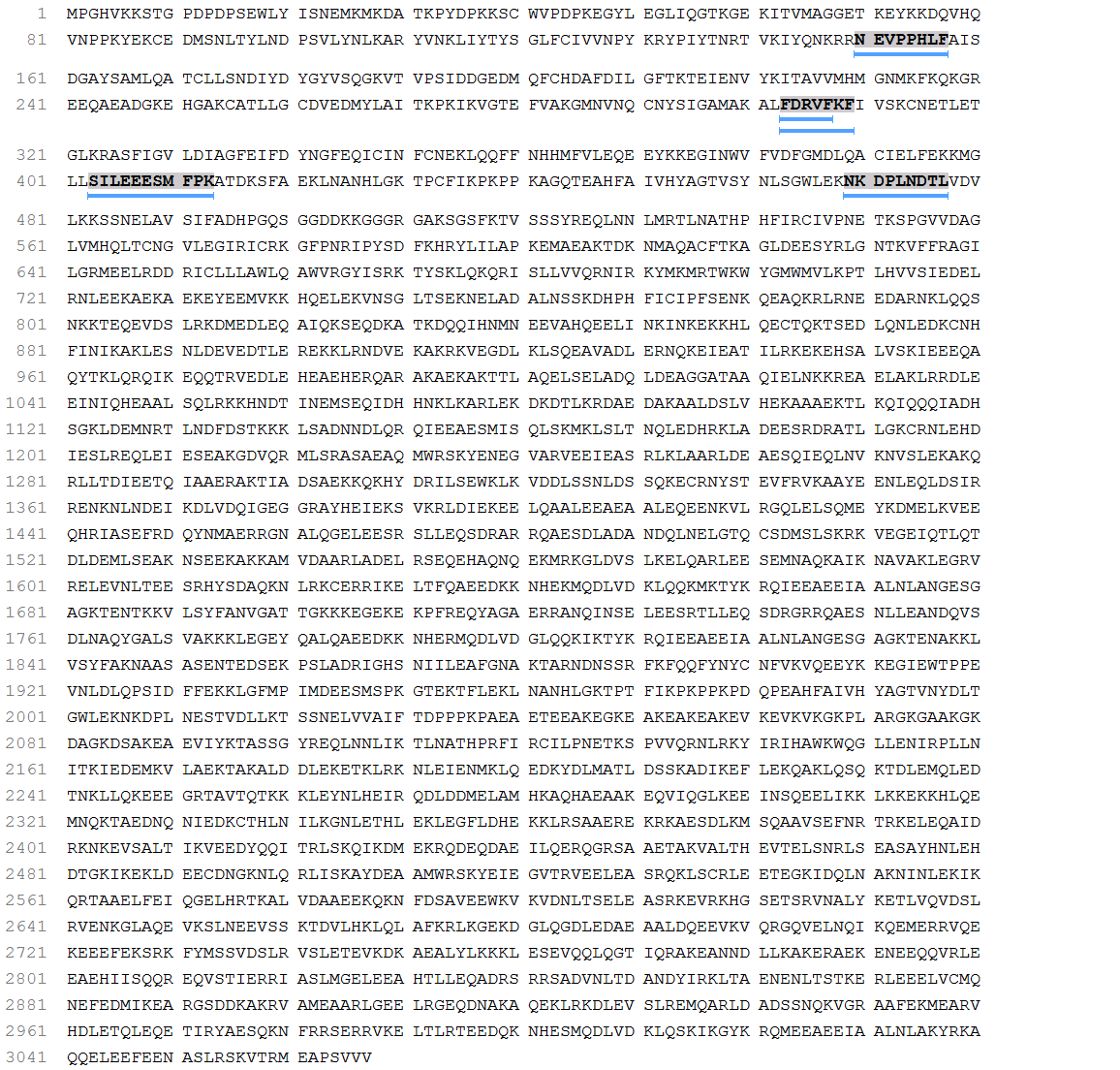

Supplement: Supplementary file 1 [file marinedrugs-24-00036-s001.zip › SM39/HP/img/cov_53302.png]

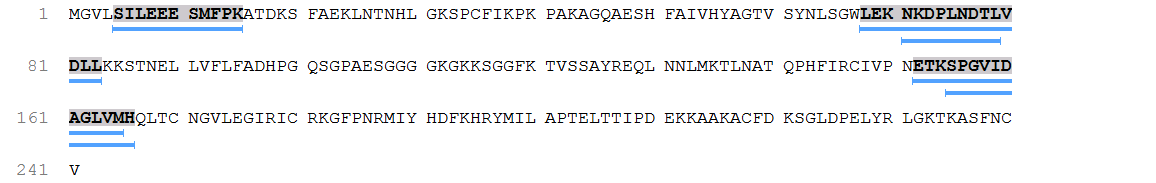

Supplement: Supplementary file 1 [file marinedrugs-24-00036-s001.zip › SM39/HP/img/cov_53308.png]
