# Supplementary figures and images for: Proteomic and Functional Characterization of Antimicrobial Peptides Derived from Fisheries Bycatch via Enzymatic Hydrolysis (part 1 of 2)
Source: Mar Drugs. 2026 Jan 10;24(1):36. doi: 10.3390/md24010036 (PMC12843163; doi:10.3390/md24010036)

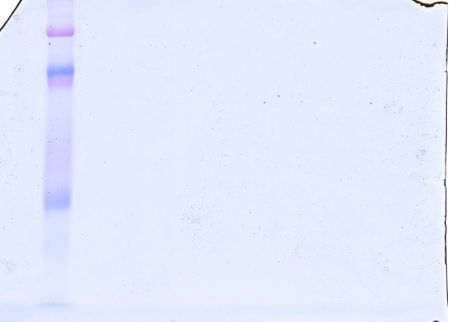

Supplement: Supplementary file 1 [file marinedrugs-24-00036-s001.zip › SM1.png]

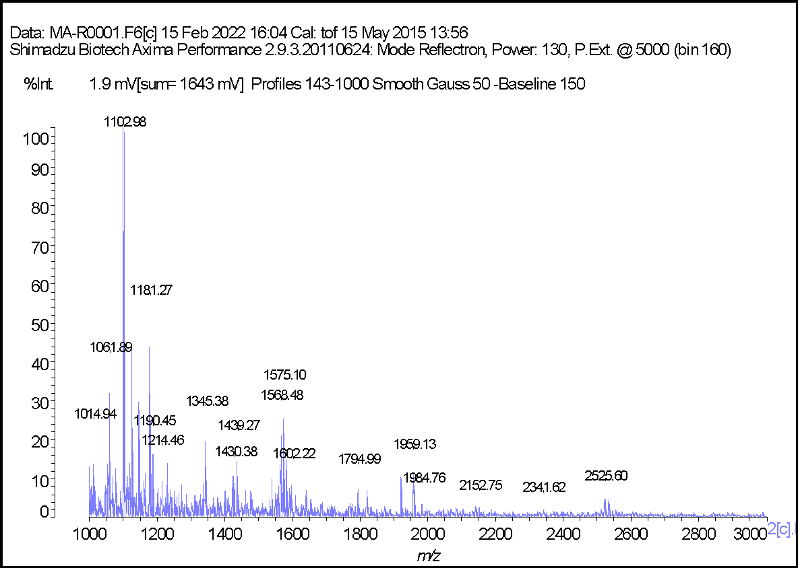

Supplement: Supplementary file 1 [file marinedrugs-24-00036-s001.zip › SM10.png]

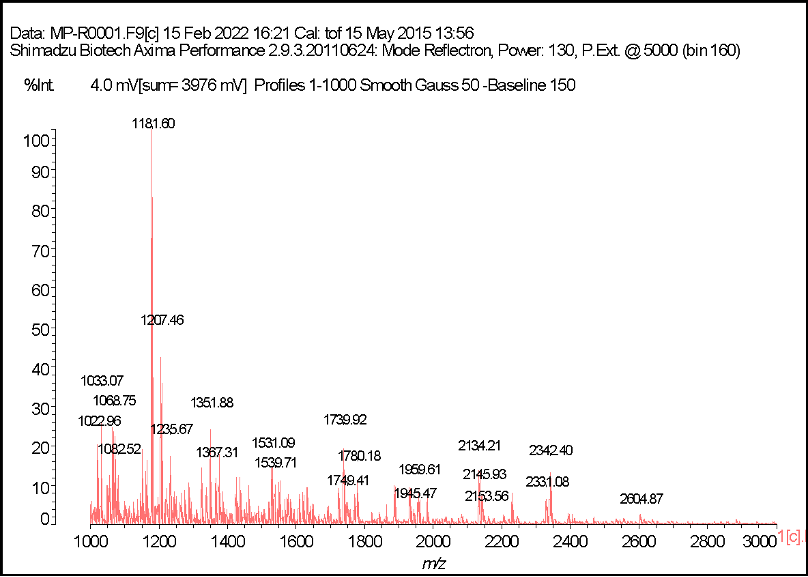

Supplement: Supplementary file 1 [file marinedrugs-24-00036-s001.zip › SM11.png]

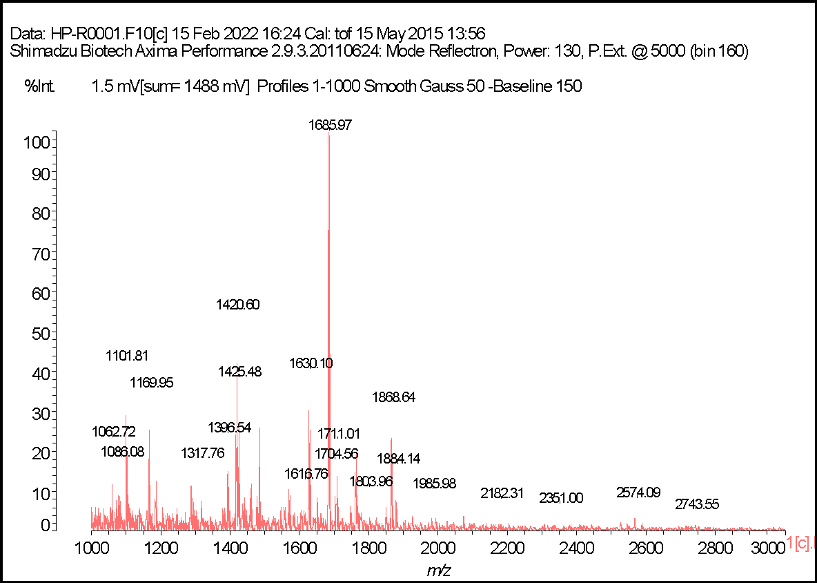

Supplement: Supplementary file 1 [file marinedrugs-24-00036-s001.zip › SM12.png]

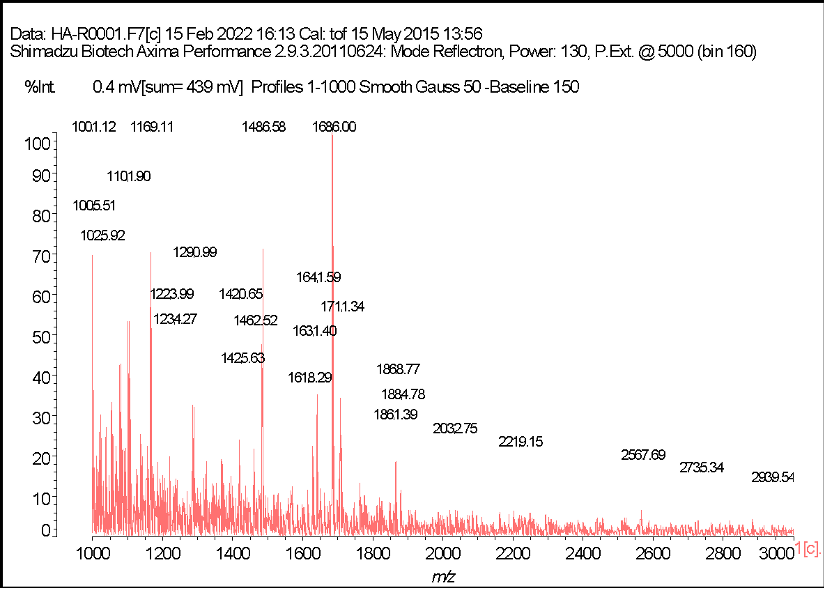

Supplement: Supplementary file 1 [file marinedrugs-24-00036-s001.zip › SM13.png]

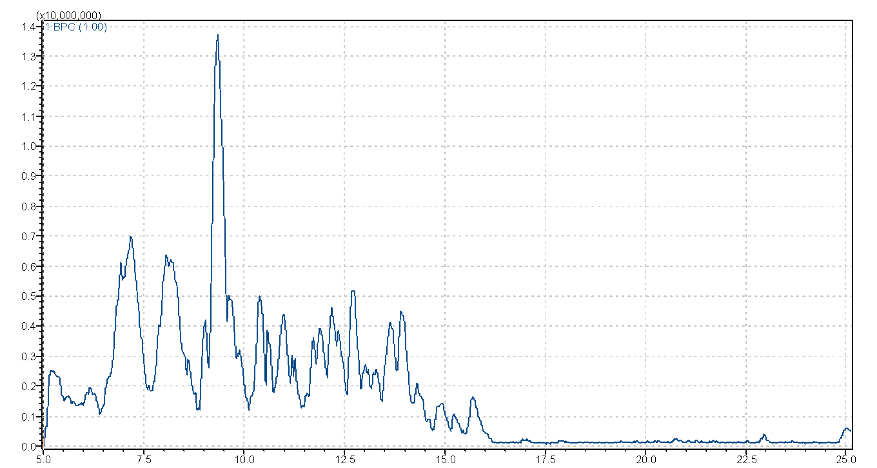

Supplement: Supplementary file 1 [file marinedrugs-24-00036-s001.zip › SM14.png]

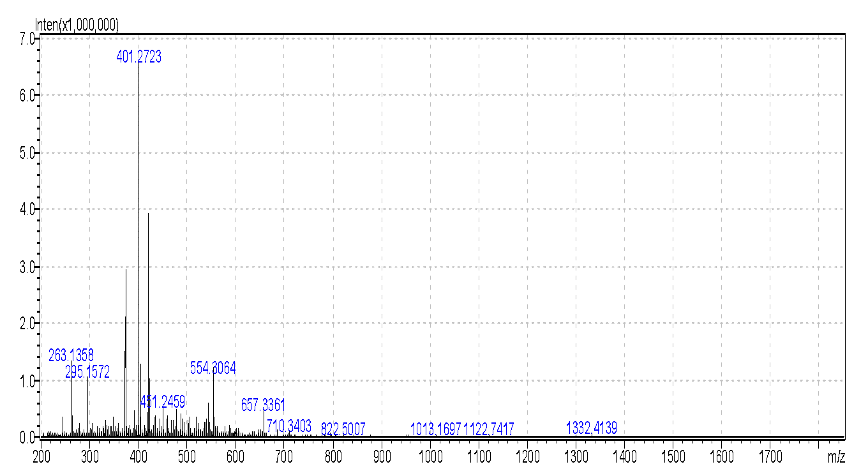

Supplement: Supplementary file 1 [file marinedrugs-24-00036-s001.zip › SM15.png]

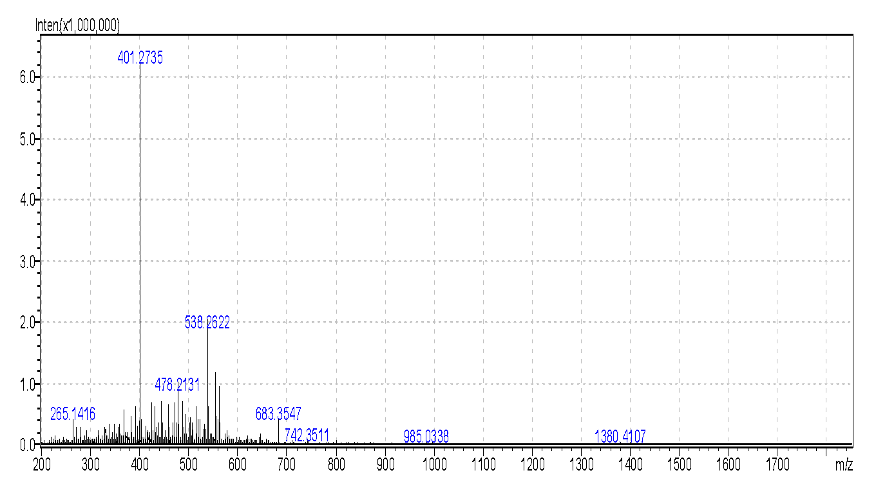

Supplement: Supplementary file 1 [file marinedrugs-24-00036-s001.zip › SM16.png]

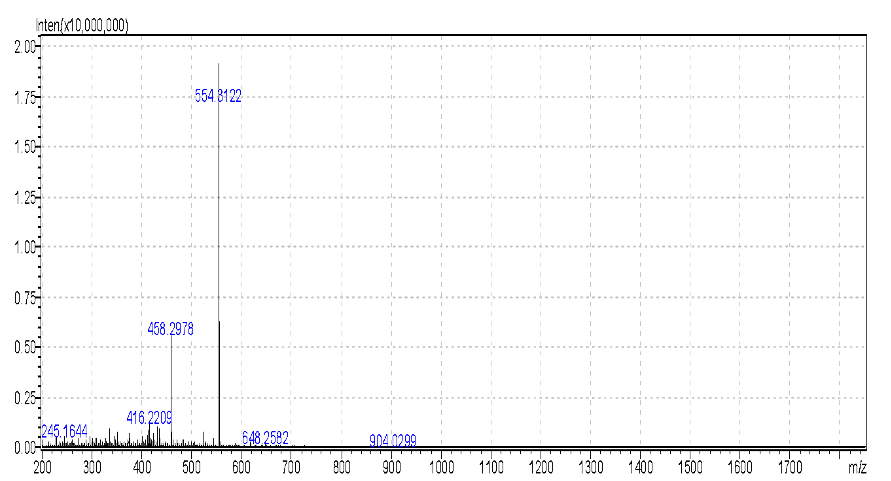

Supplement: Supplementary file 1 [file marinedrugs-24-00036-s001.zip › SM17.png]

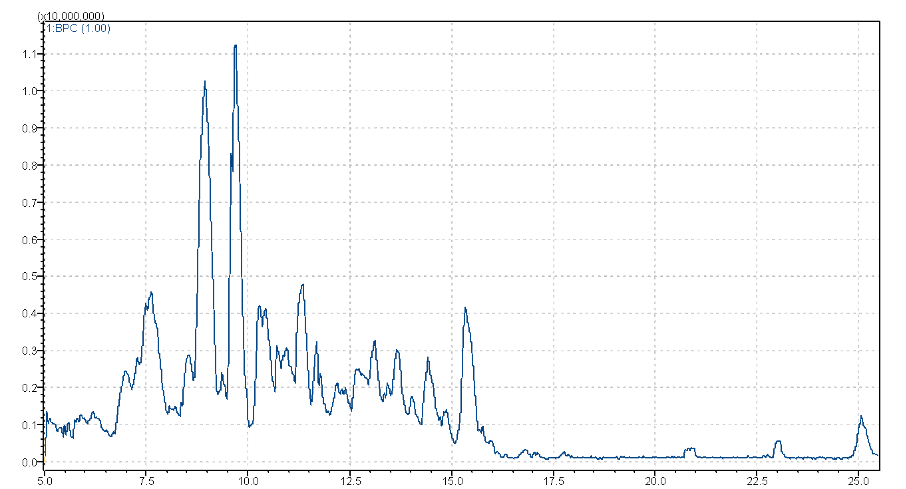

Supplement: Supplementary file 1 [file marinedrugs-24-00036-s001.zip › SM18.png]

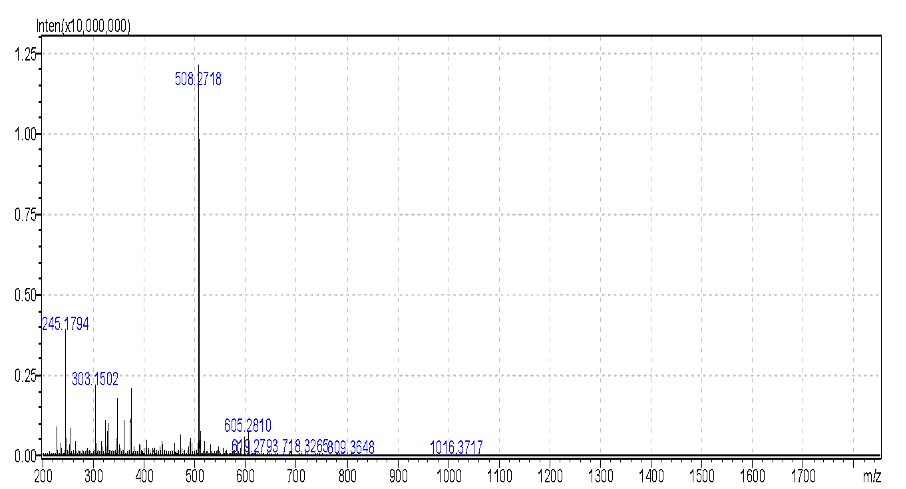

Supplement: Supplementary file 1 [file marinedrugs-24-00036-s001.zip › SM19.png]

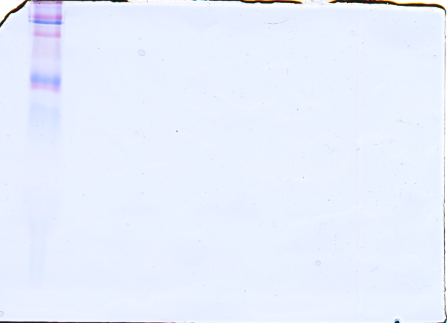

Supplement: Supplementary file 1 [file marinedrugs-24-00036-s001.zip › SM2.png]

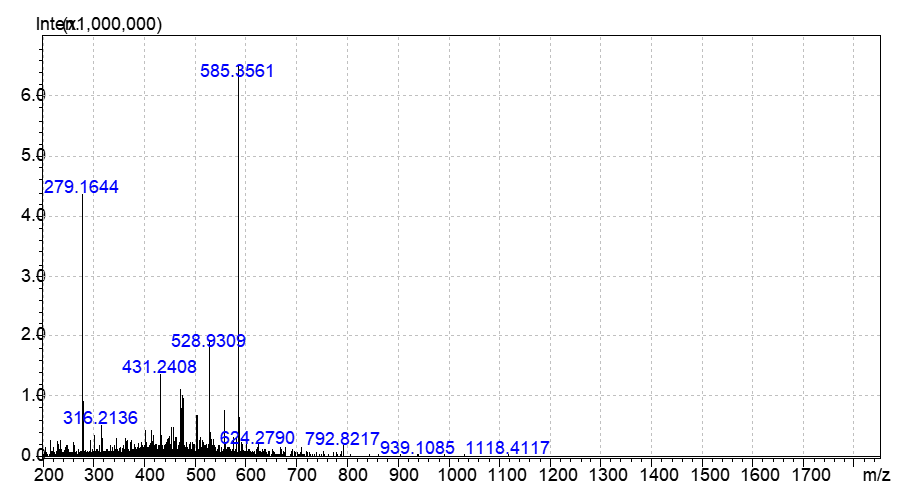

Supplement: Supplementary file 1 [file marinedrugs-24-00036-s001.zip › SM20.png]

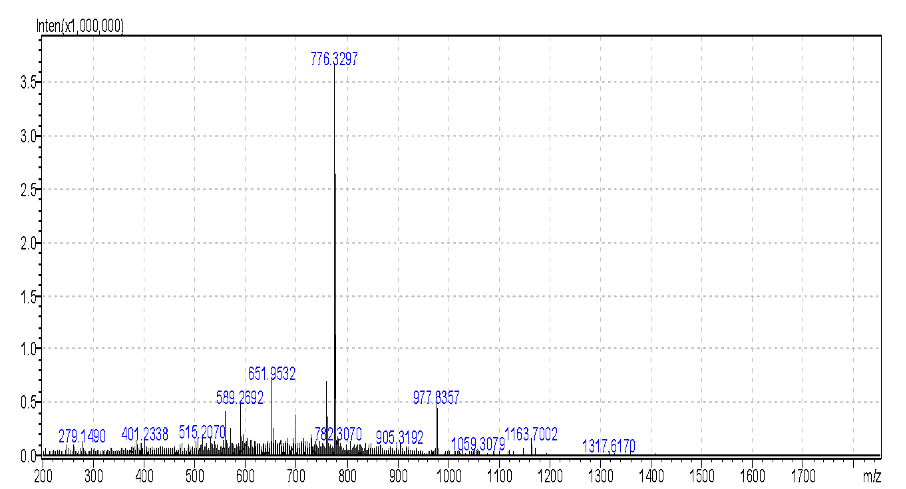

Supplement: Supplementary file 1 [file marinedrugs-24-00036-s001.zip › SM21.png]

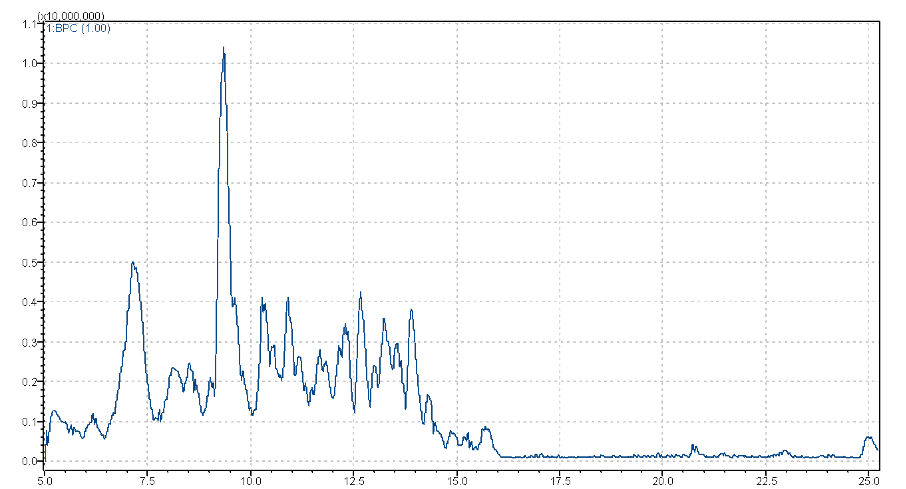

Supplement: Supplementary file 1 [file marinedrugs-24-00036-s001.zip › SM22.png]

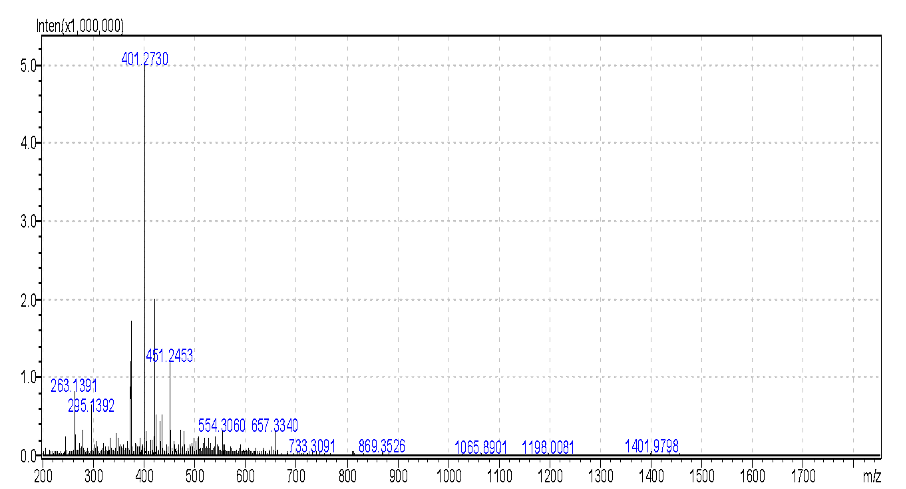

Supplement: Supplementary file 1 [file marinedrugs-24-00036-s001.zip › SM23.png]

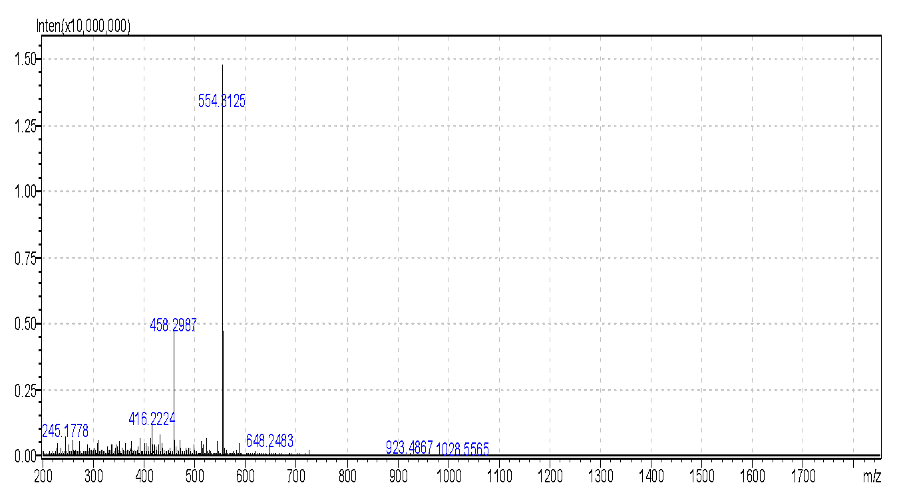

Supplement: Supplementary file 1 [file marinedrugs-24-00036-s001.zip › SM24.png]

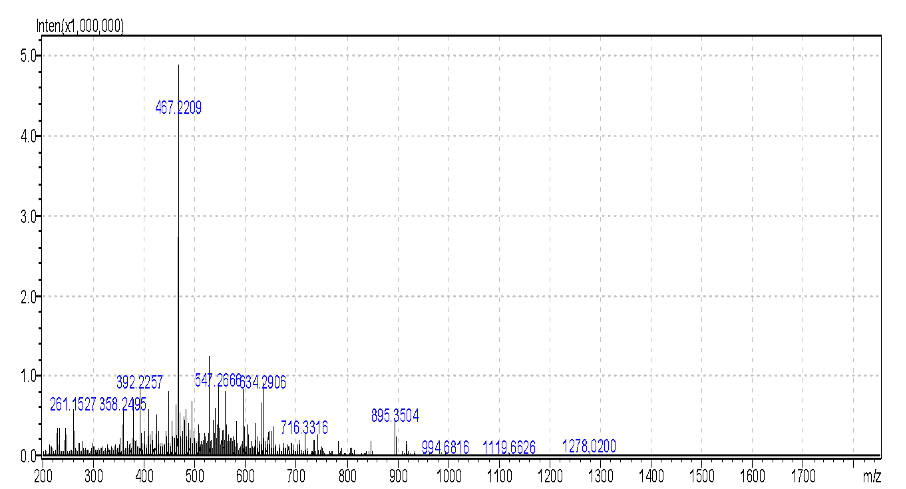

Supplement: Supplementary file 1 [file marinedrugs-24-00036-s001.zip › SM25.png]

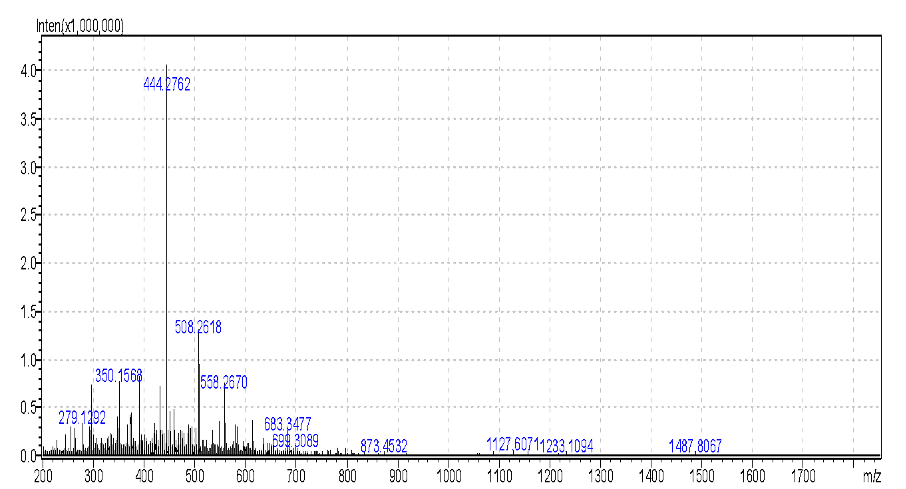

Supplement: Supplementary file 1 [file marinedrugs-24-00036-s001.zip › SM26.png]

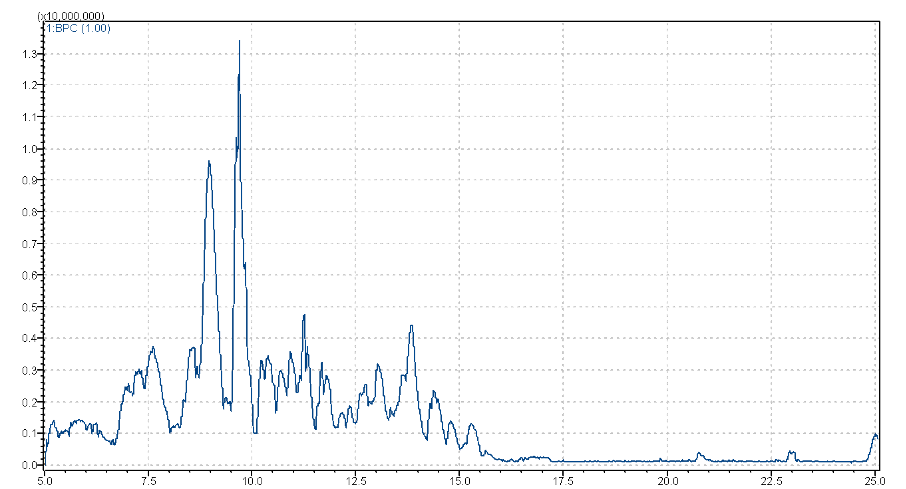

Supplement: Supplementary file 1 [file marinedrugs-24-00036-s001.zip › SM27.png]

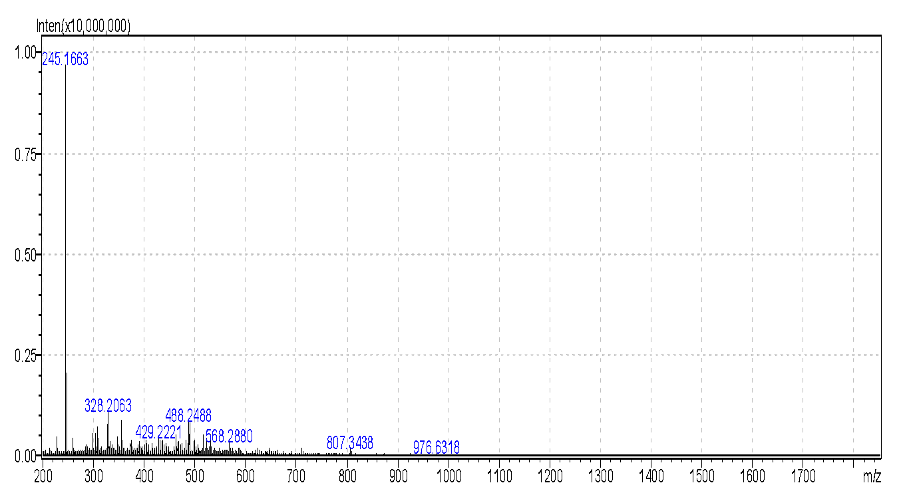

Supplement: Supplementary file 1 [file marinedrugs-24-00036-s001.zip › SM28.png]

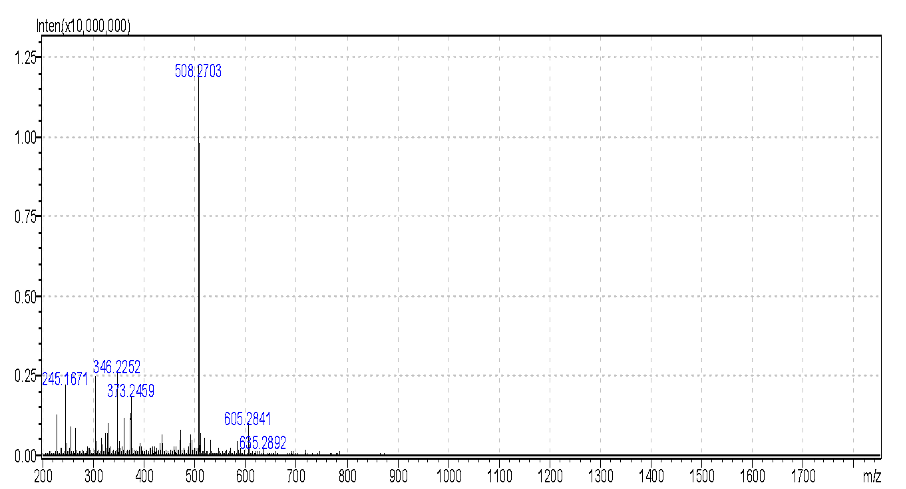

Supplement: Supplementary file 1 [file marinedrugs-24-00036-s001.zip › SM29.png]

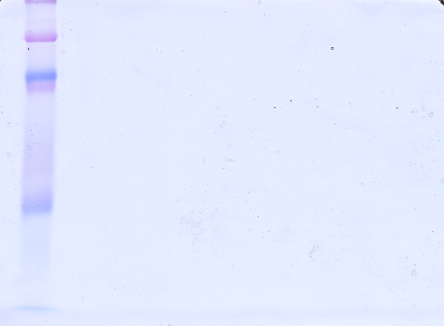

Supplement: Supplementary file 1 [file marinedrugs-24-00036-s001.zip › SM3.png]

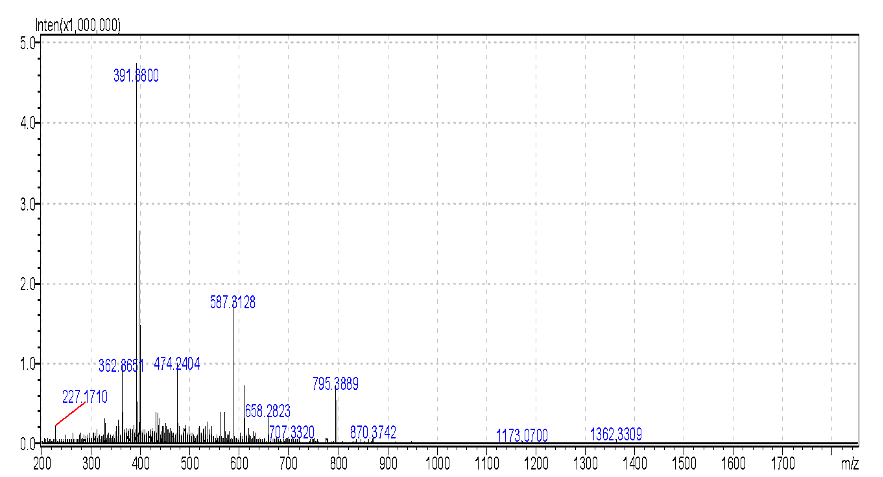

Supplement: Supplementary file 1 [file marinedrugs-24-00036-s001.zip › SM30.png]

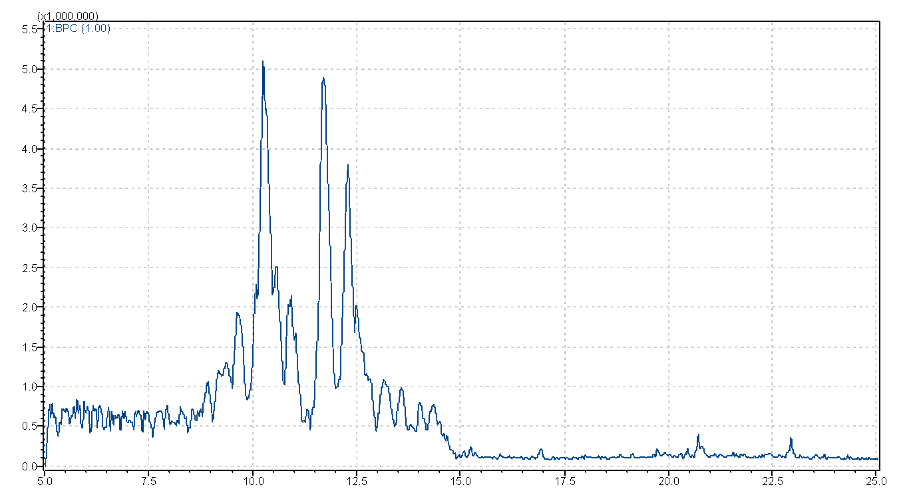

Supplement: Supplementary file 1 [file marinedrugs-24-00036-s001.zip › SM31.png]

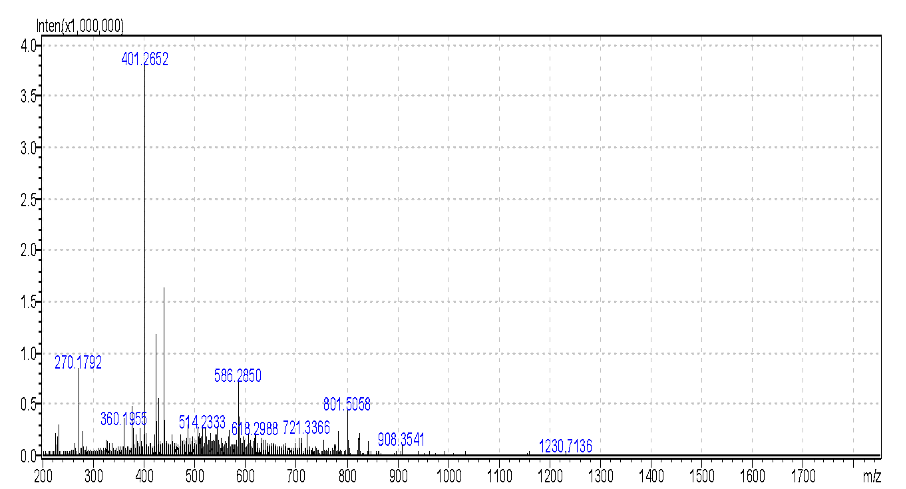

Supplement: Supplementary file 1 [file marinedrugs-24-00036-s001.zip › SM32.png]

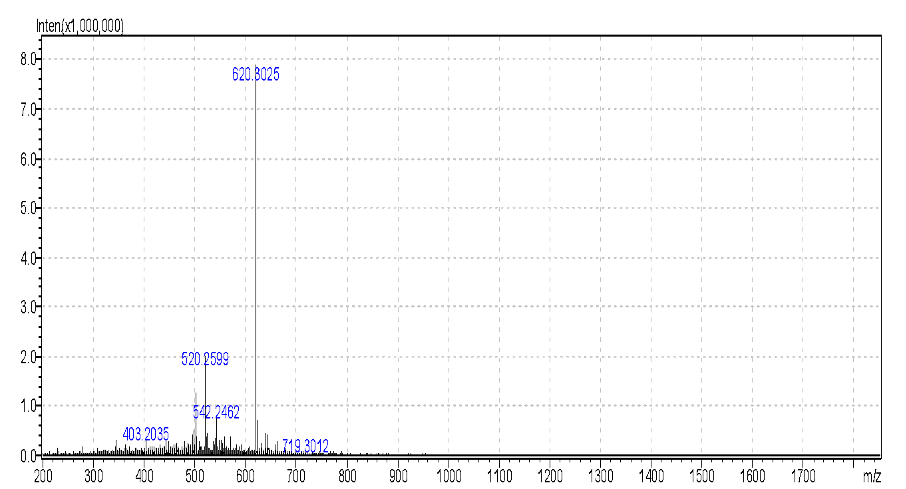

Supplement: Supplementary file 1 [file marinedrugs-24-00036-s001.zip › SM33.png]

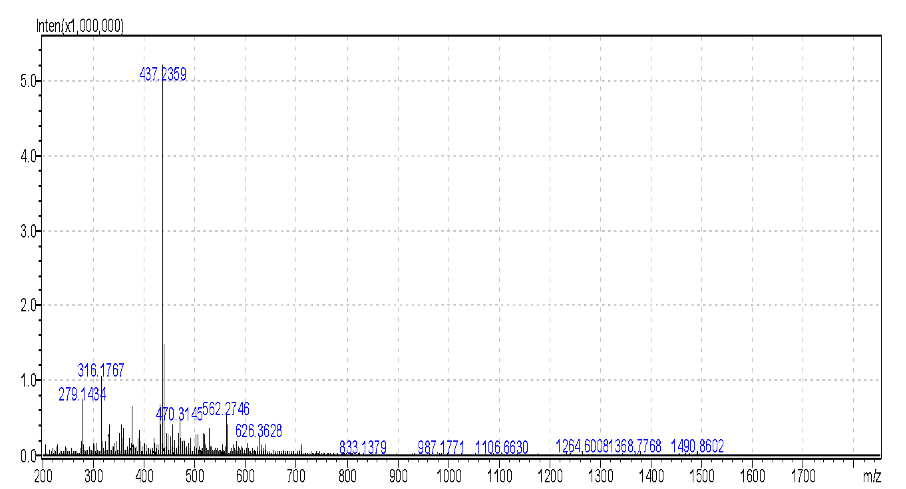

Supplement: Supplementary file 1 [file marinedrugs-24-00036-s001.zip › SM34.png]

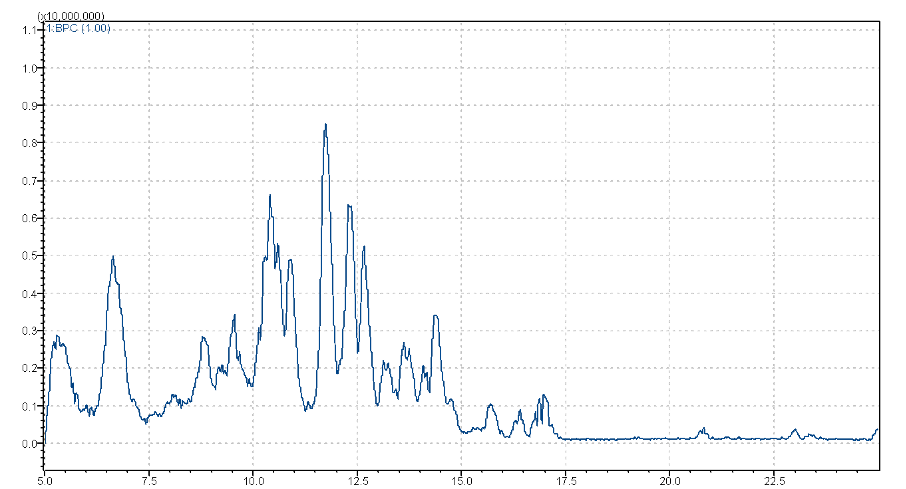

Supplement: Supplementary file 1 [file marinedrugs-24-00036-s001.zip › SM35.png]

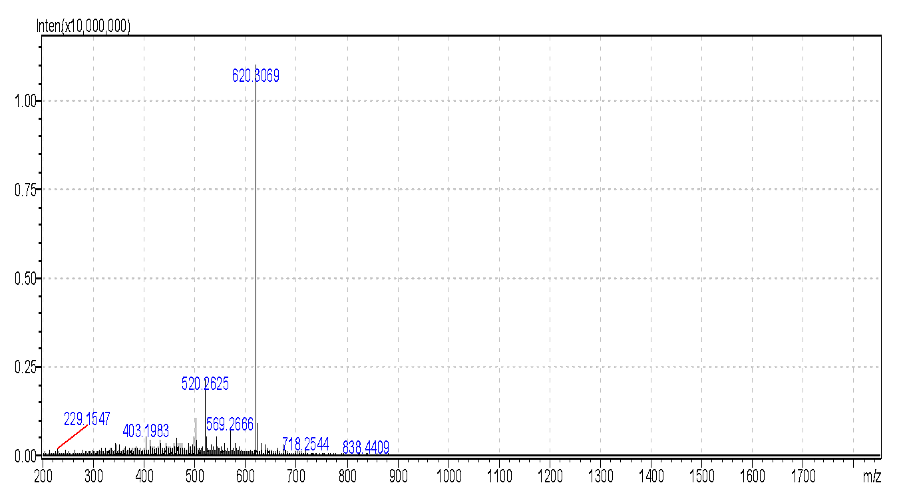

Supplement: Supplementary file 1 [file marinedrugs-24-00036-s001.zip › SM36.png]

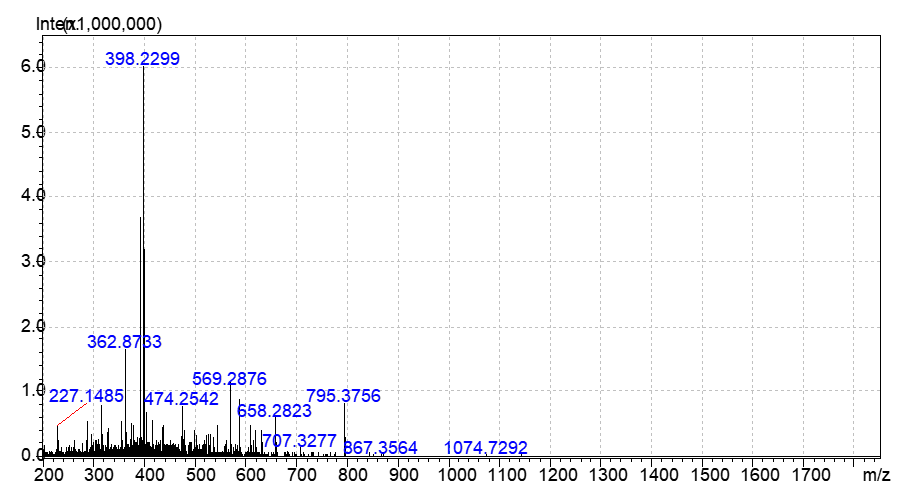

Supplement: Supplementary file 1 [file marinedrugs-24-00036-s001.zip › SM37.png]

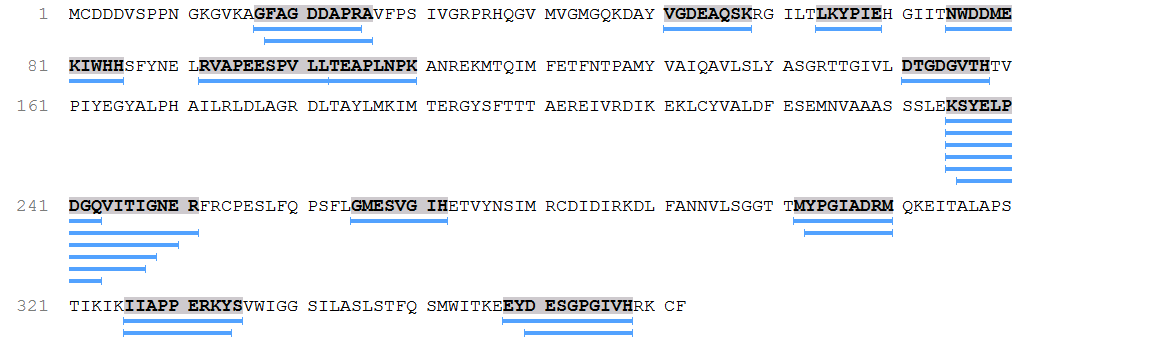

Supplement: Supplementary file 1 [file marinedrugs-24-00036-s001.zip › SM38/HA/img/cov_53234.png]

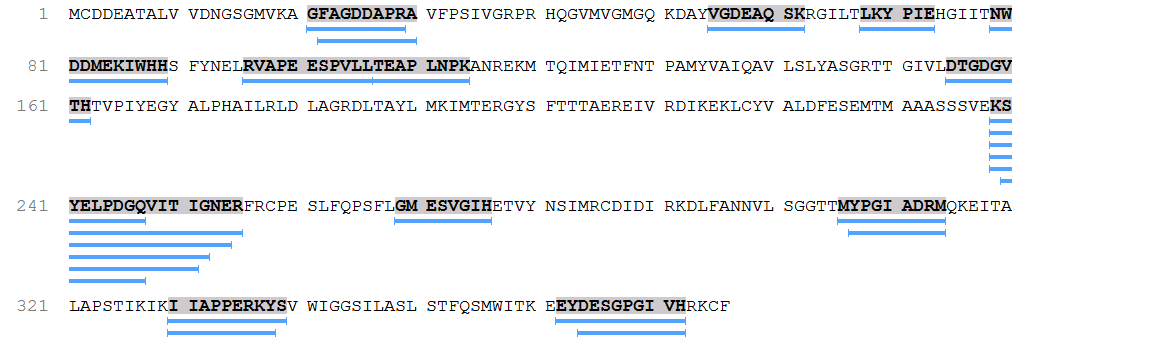

Supplement: Supplementary file 1 [file marinedrugs-24-00036-s001.zip › SM38/HA/img/cov_53235.png]

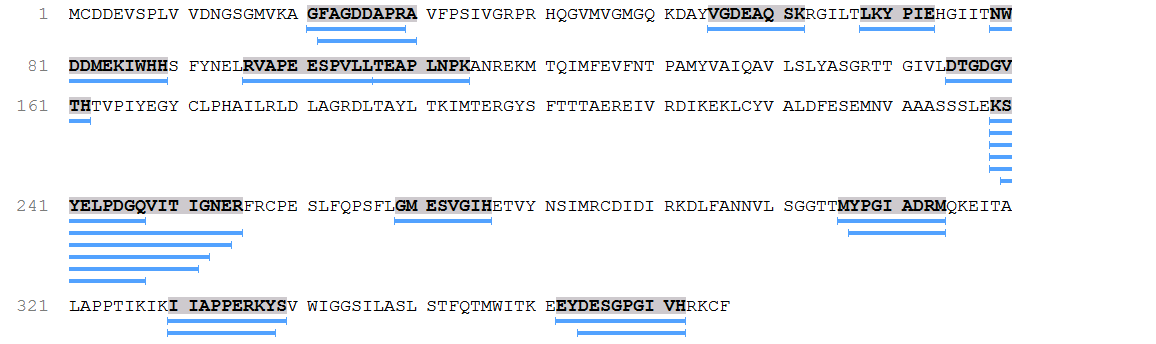

Supplement: Supplementary file 1 [file marinedrugs-24-00036-s001.zip › SM38/HA/img/cov_53236.png]

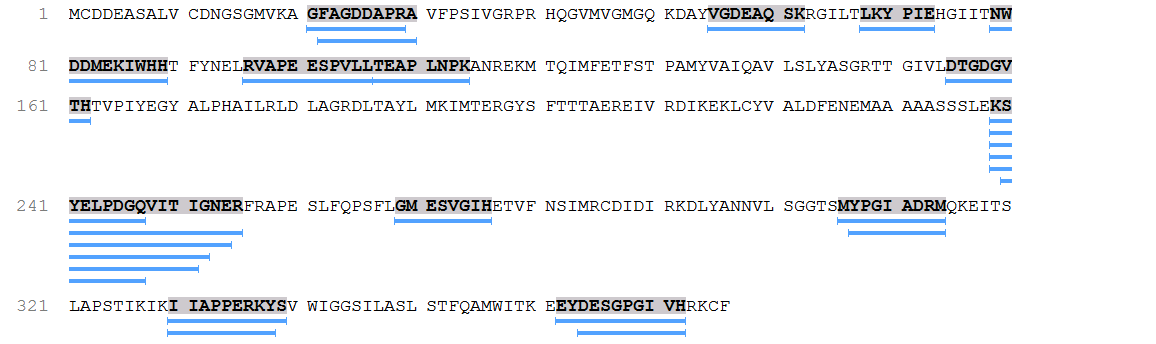

Supplement: Supplementary file 1 [file marinedrugs-24-00036-s001.zip › SM38/HA/img/cov_53237.png]

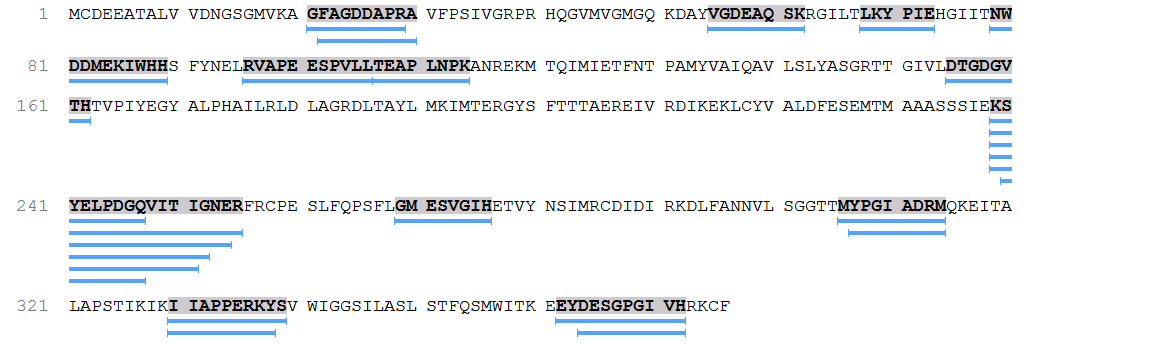

Supplement: Supplementary file 1 [file marinedrugs-24-00036-s001.zip › SM38/HA/img/cov_53238.png]

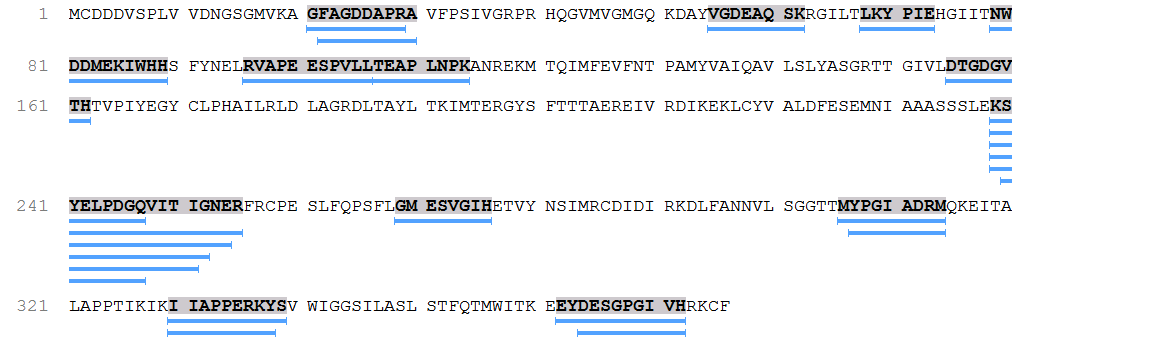

Supplement: Supplementary file 1 [file marinedrugs-24-00036-s001.zip › SM38/HA/img/cov_53239.png]

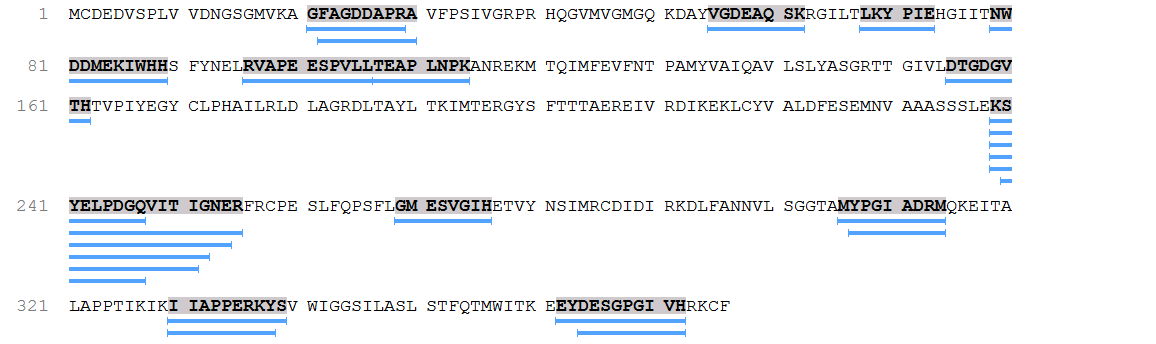

Supplement: Supplementary file 1 [file marinedrugs-24-00036-s001.zip › SM38/HA/img/cov_53240.png]

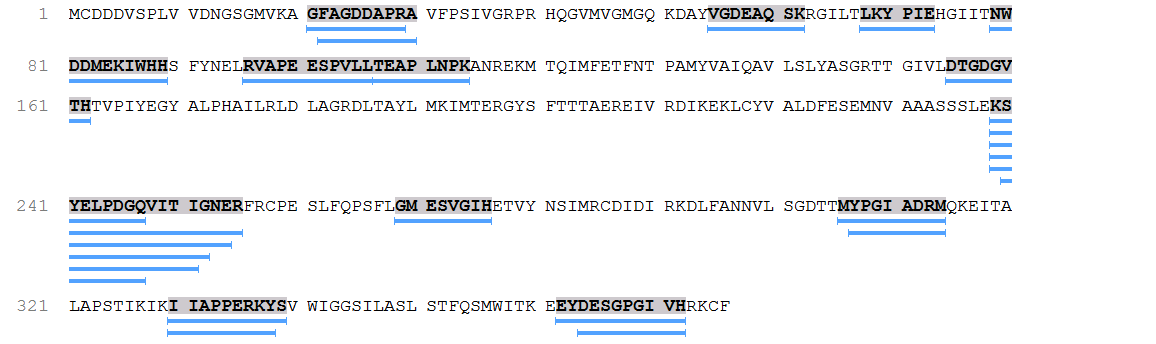

Supplement: Supplementary file 1 [file marinedrugs-24-00036-s001.zip › SM38/HA/img/cov_53241.png]

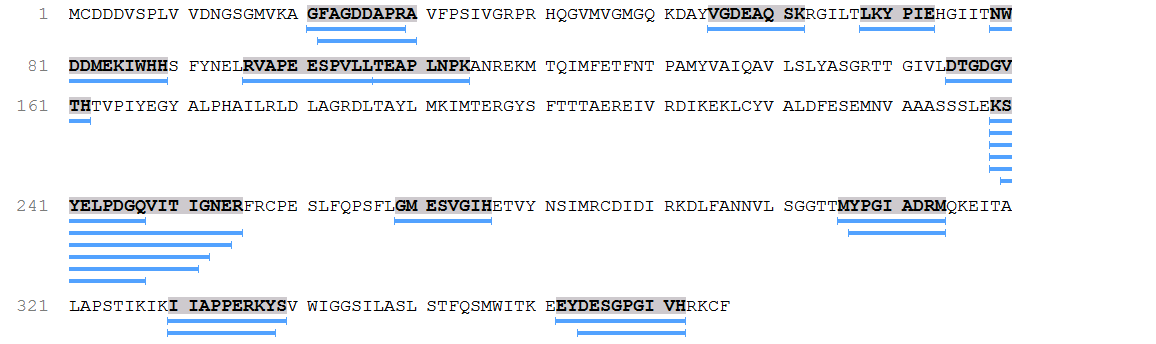

Supplement: Supplementary file 1 [file marinedrugs-24-00036-s001.zip › SM38/HA/img/cov_53242.png]

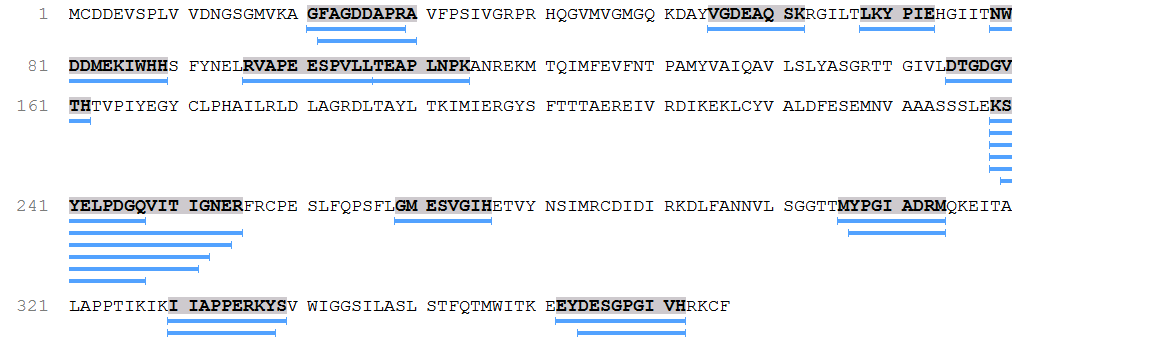

Supplement: Supplementary file 1 [file marinedrugs-24-00036-s001.zip › SM38/HA/img/cov_53243.png]

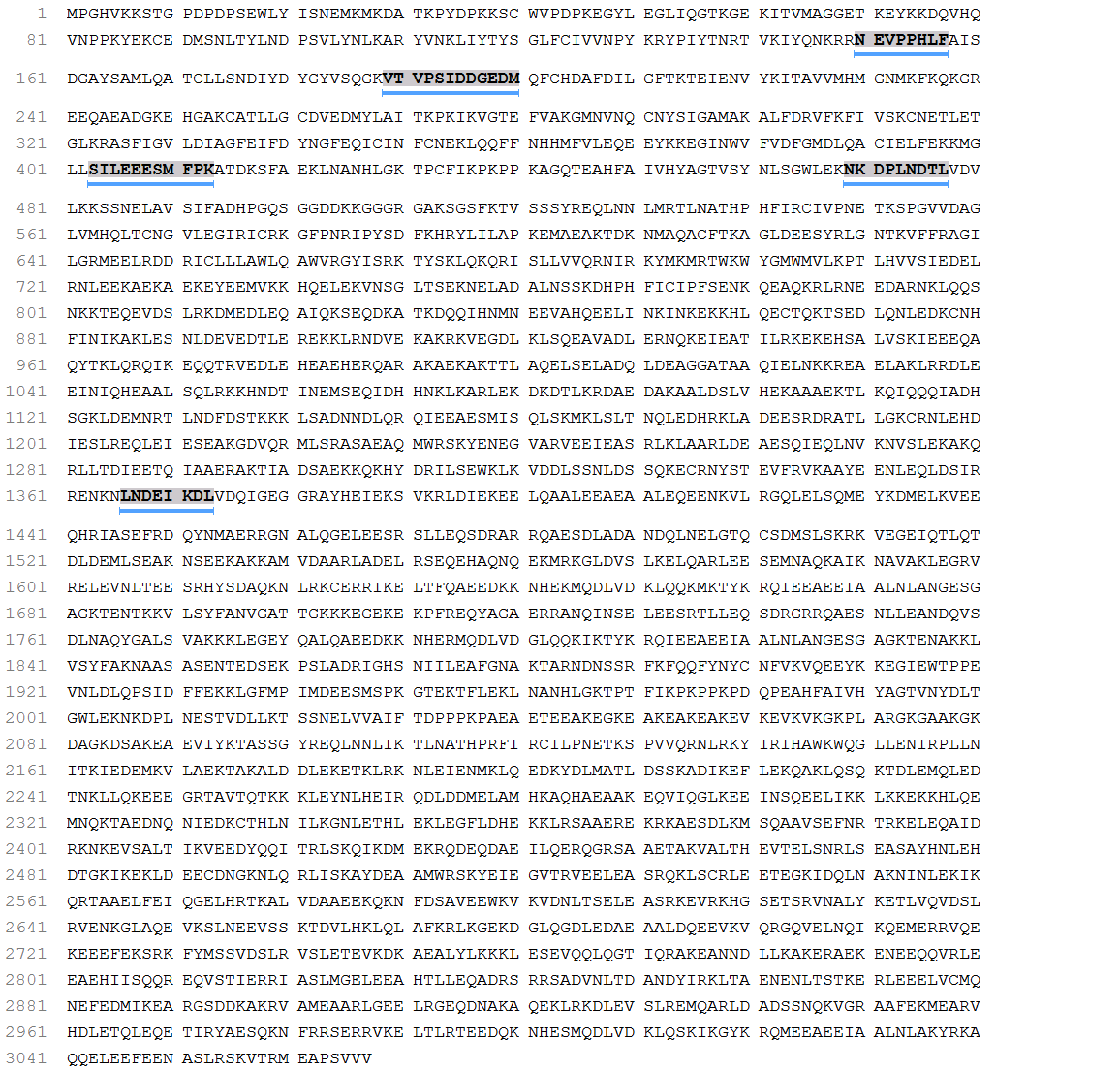

Supplement: Supplementary file 1 [file marinedrugs-24-00036-s001.zip › SM38/HA/img/cov_53302.png]

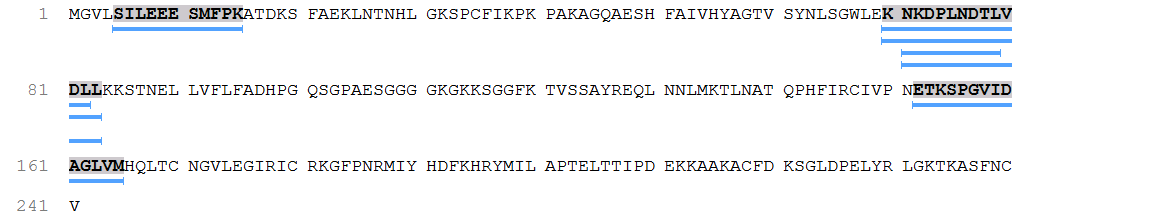

Supplement: Supplementary file 1 [file marinedrugs-24-00036-s001.zip › SM38/HA/img/cov_53308.png]

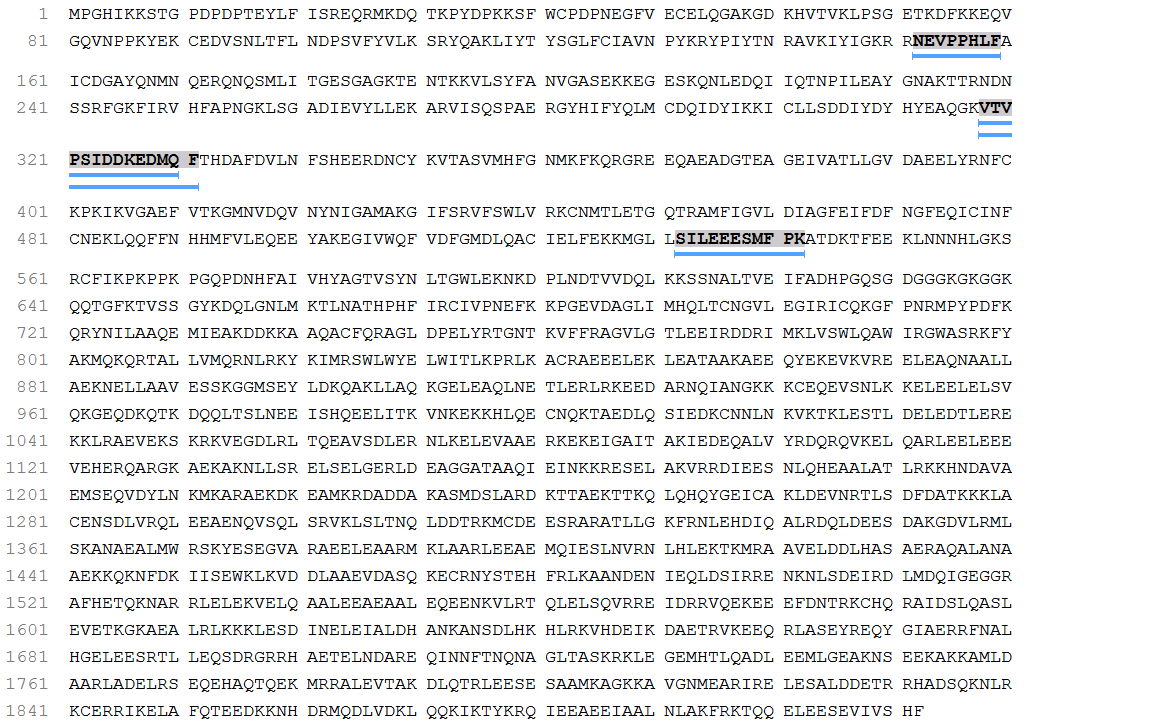

Supplement: Supplementary file 1 [file marinedrugs-24-00036-s001.zip › SM38/HA/img/cov_53314.png]

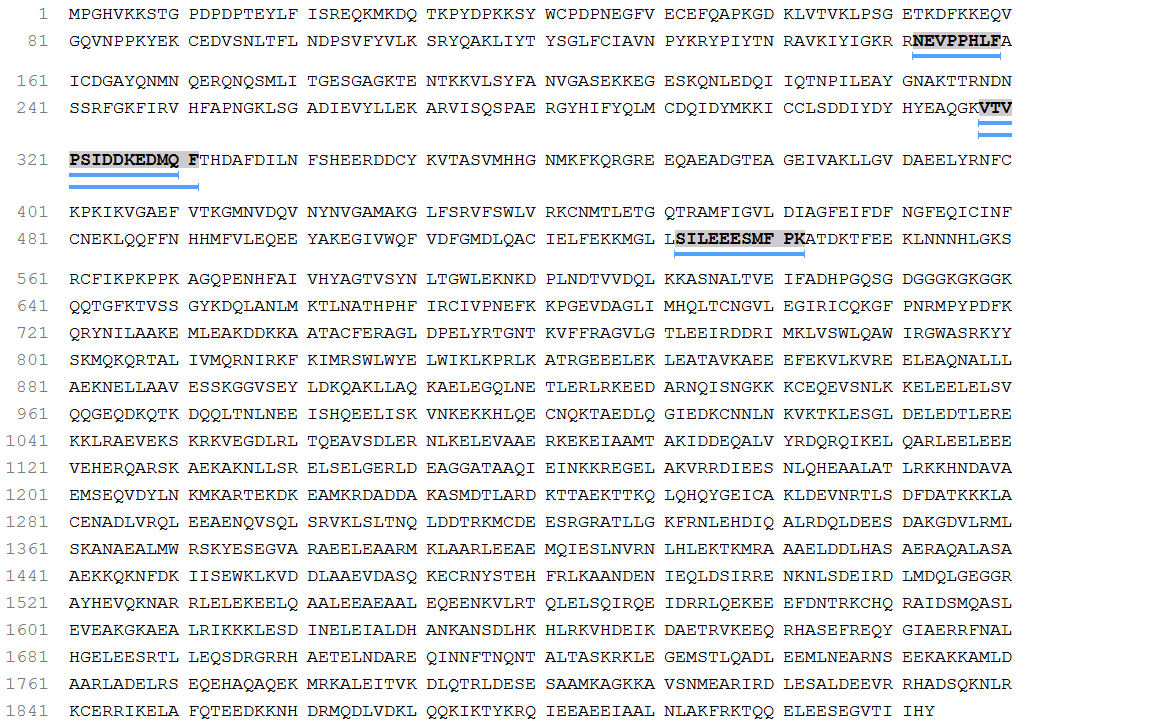

Supplement: Supplementary file 1 [file marinedrugs-24-00036-s001.zip › SM38/HA/img/cov_53315.png]

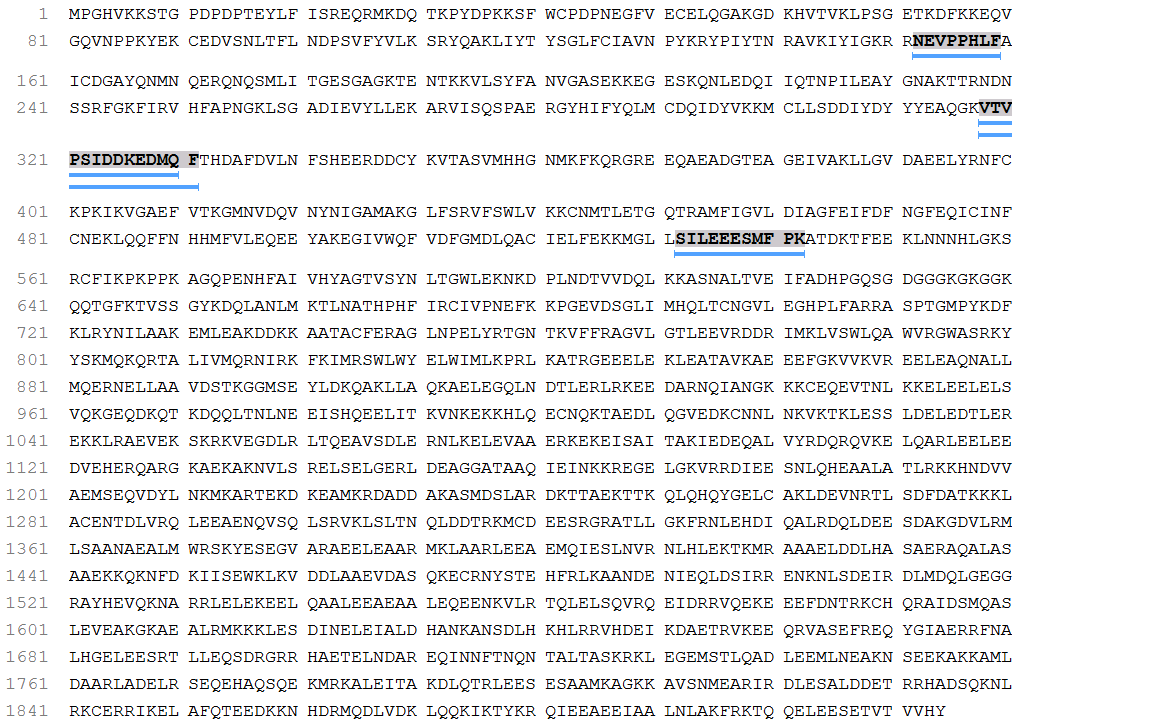

Supplement: Supplementary file 1 [file marinedrugs-24-00036-s001.zip › SM38/HA/img/cov_53316.png]

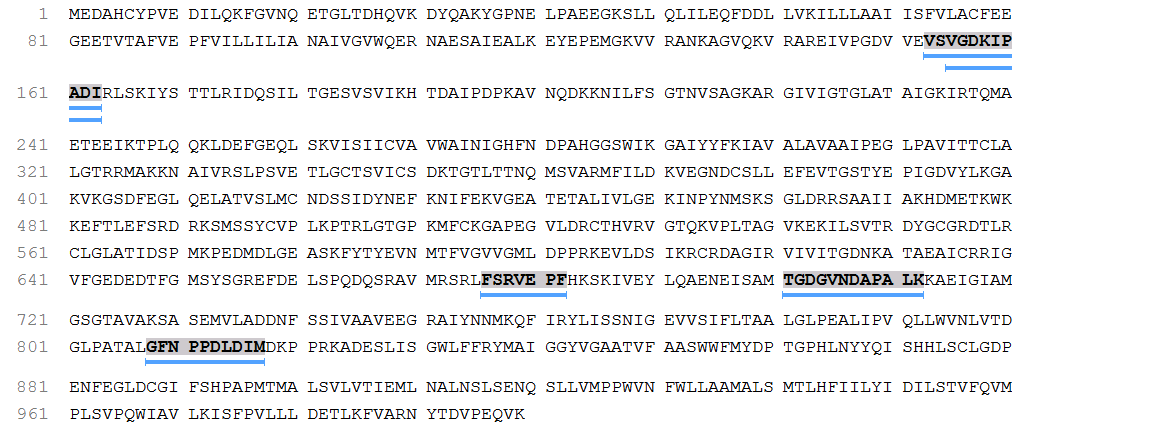

Supplement: Supplementary file 1 [file marinedrugs-24-00036-s001.zip › SM38/HA/img/cov_53318.png]

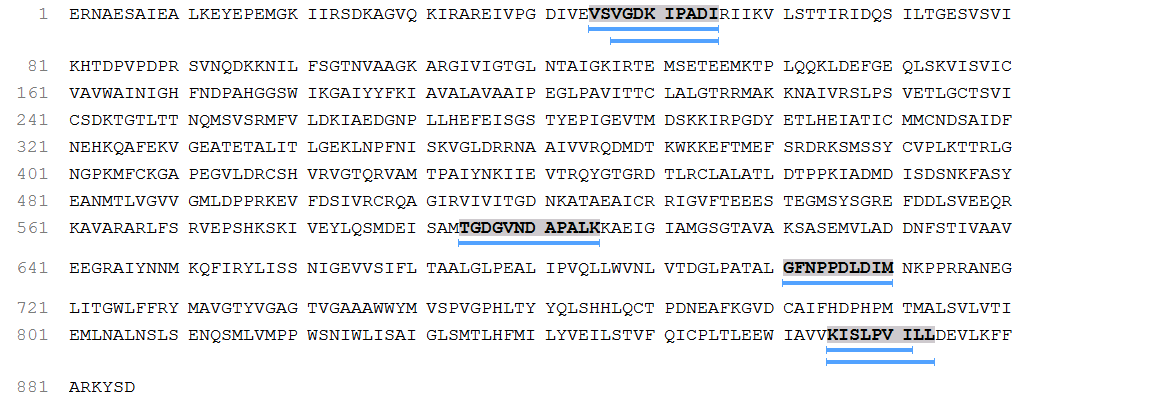

Supplement: Supplementary file 1 [file marinedrugs-24-00036-s001.zip › SM38/HA/img/cov_53320.png]

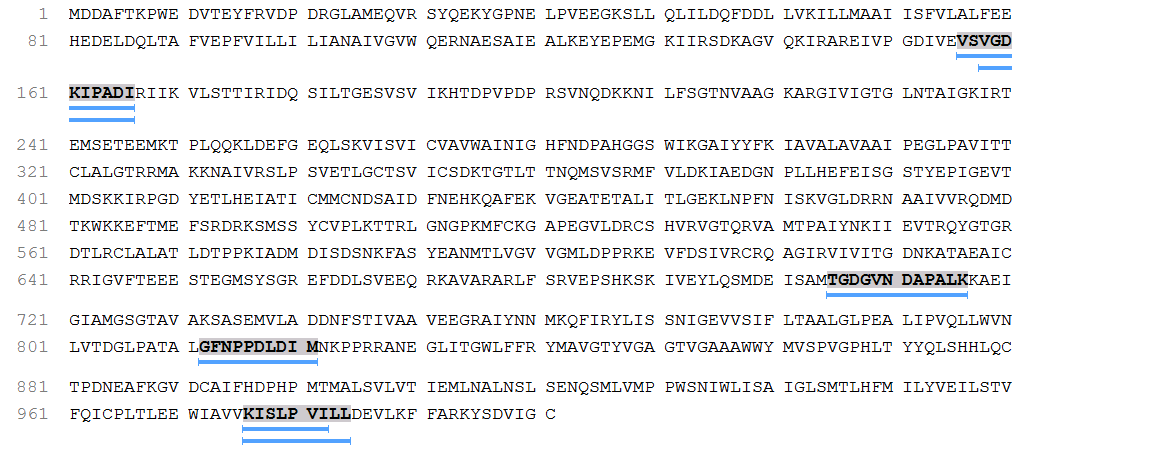

Supplement: Supplementary file 1 [file marinedrugs-24-00036-s001.zip › SM38/HA/img/cov_53321.png]

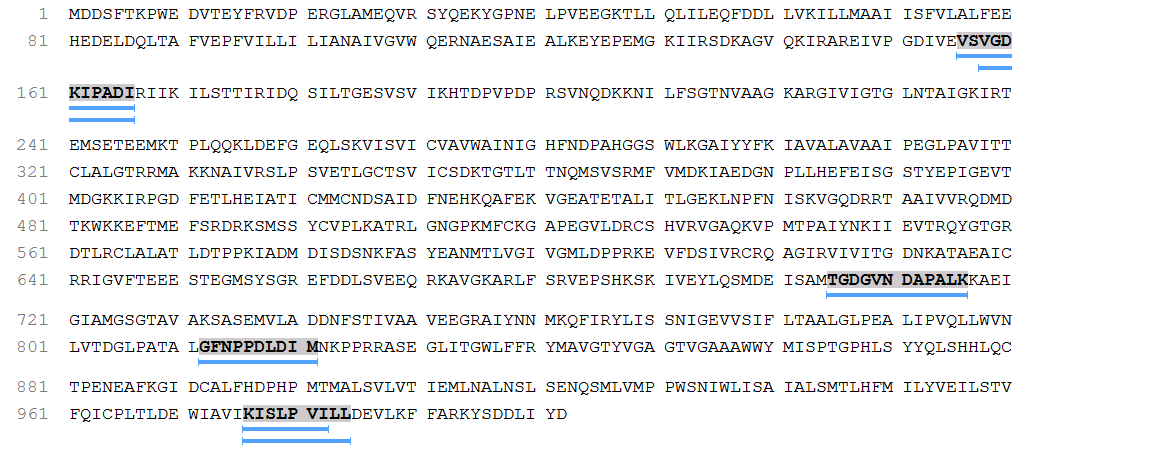

Supplement: Supplementary file 1 [file marinedrugs-24-00036-s001.zip › SM38/HA/img/cov_53322.png]

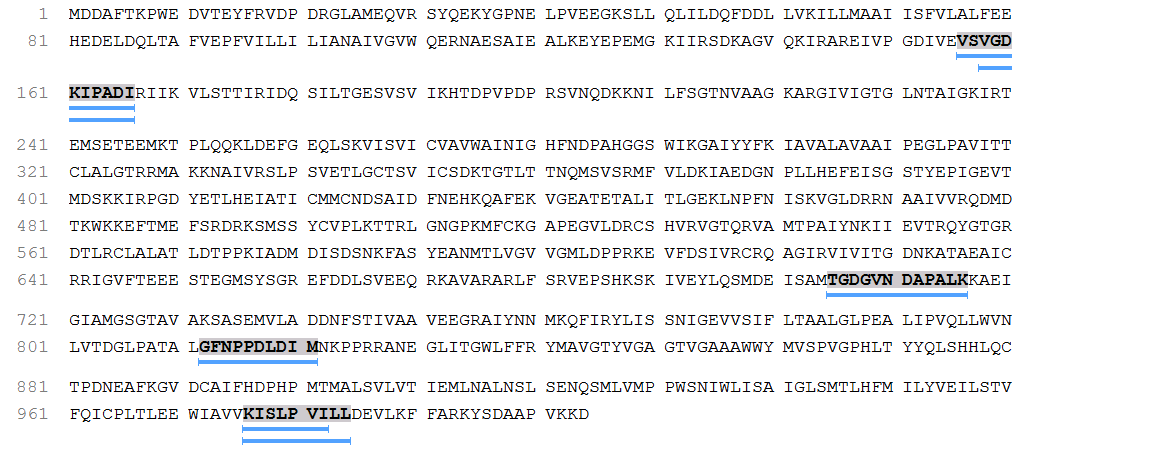

Supplement: Supplementary file 1 [file marinedrugs-24-00036-s001.zip › SM38/HA/img/cov_53323.png]

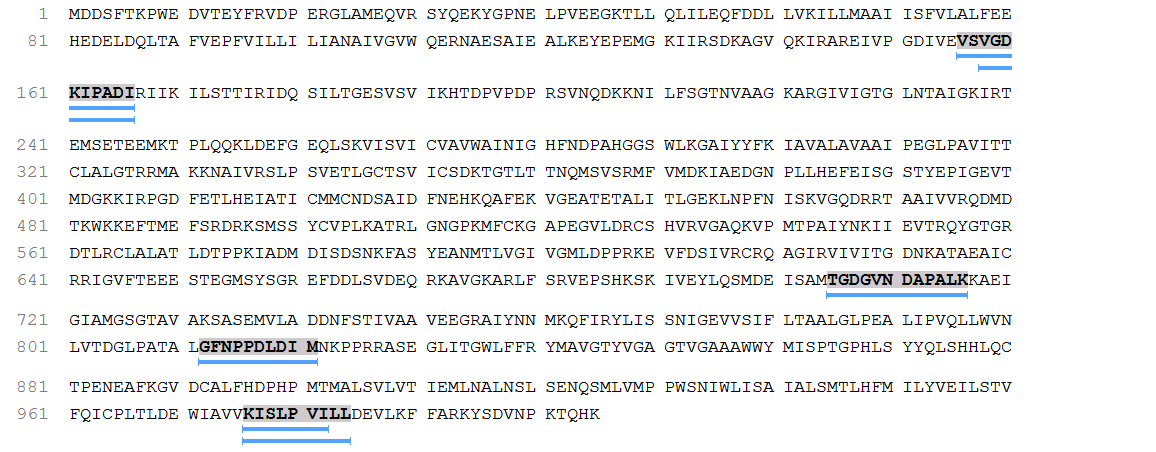

Supplement: Supplementary file 1 [file marinedrugs-24-00036-s001.zip › SM38/HA/img/cov_53324.png]

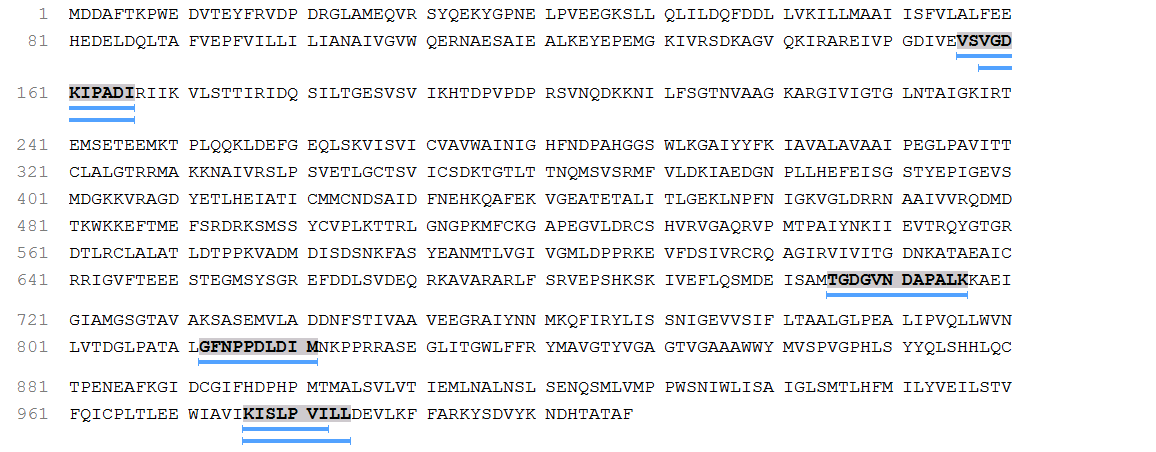

Supplement: Supplementary file 1 [file marinedrugs-24-00036-s001.zip › SM38/HA/img/cov_53325.png]

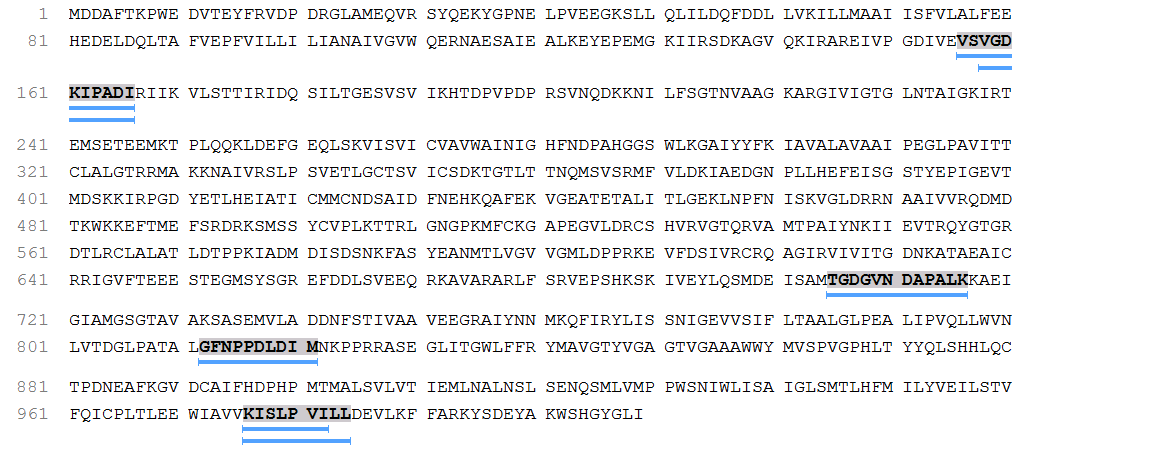

Supplement: Supplementary file 1 [file marinedrugs-24-00036-s001.zip › SM38/HA/img/cov_53326.png]

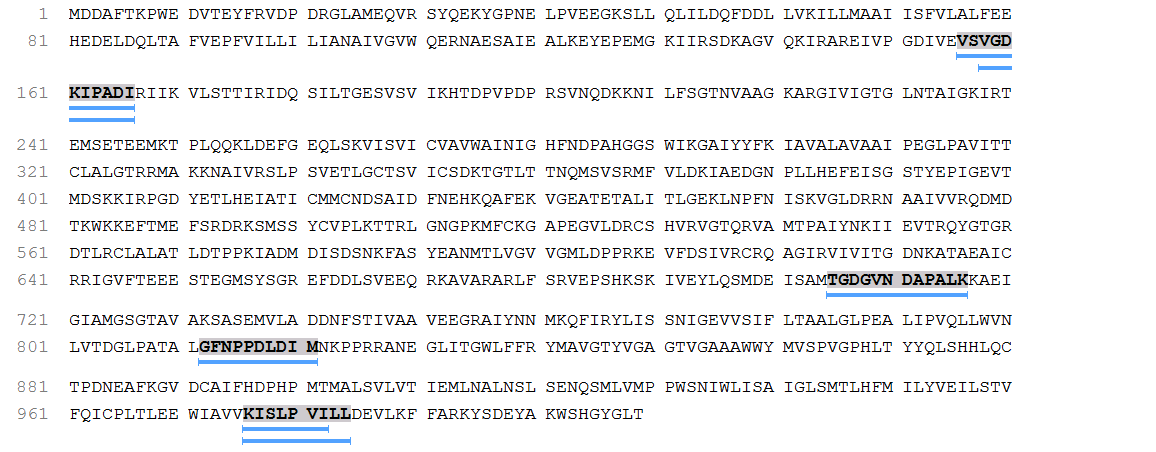

Supplement: Supplementary file 1 [file marinedrugs-24-00036-s001.zip › SM38/HA/img/cov_53327.png]

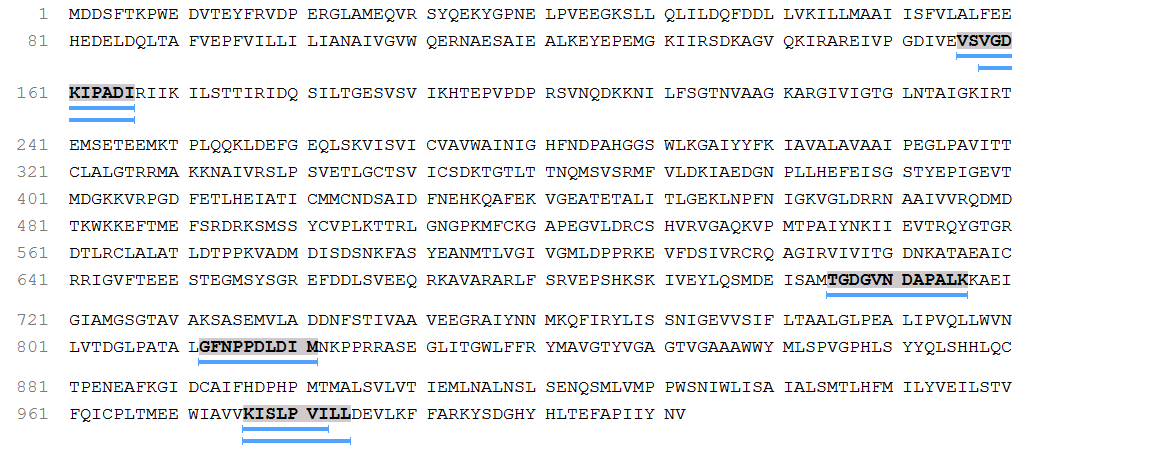

Supplement: Supplementary file 1 [file marinedrugs-24-00036-s001.zip › SM38/HA/img/cov_53328.png]

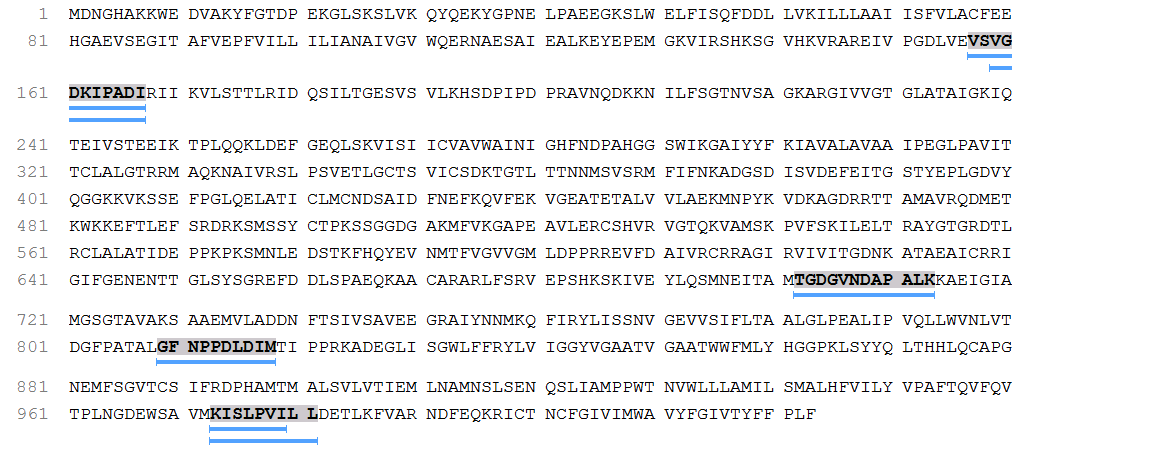

Supplement: Supplementary file 1 [file marinedrugs-24-00036-s001.zip › SM38/HA/img/cov_53329.png]

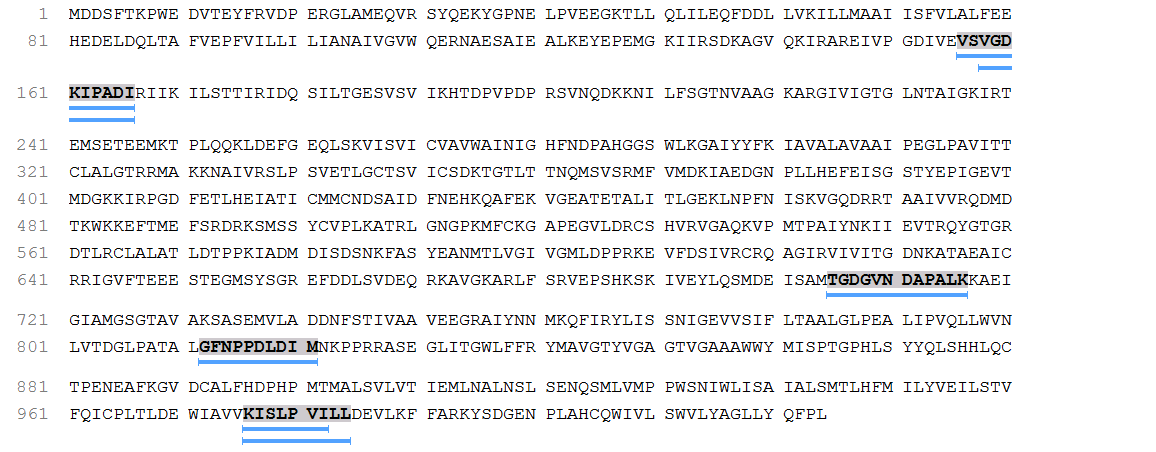

Supplement: Supplementary file 1 [file marinedrugs-24-00036-s001.zip › SM38/HA/img/cov_53330.png]

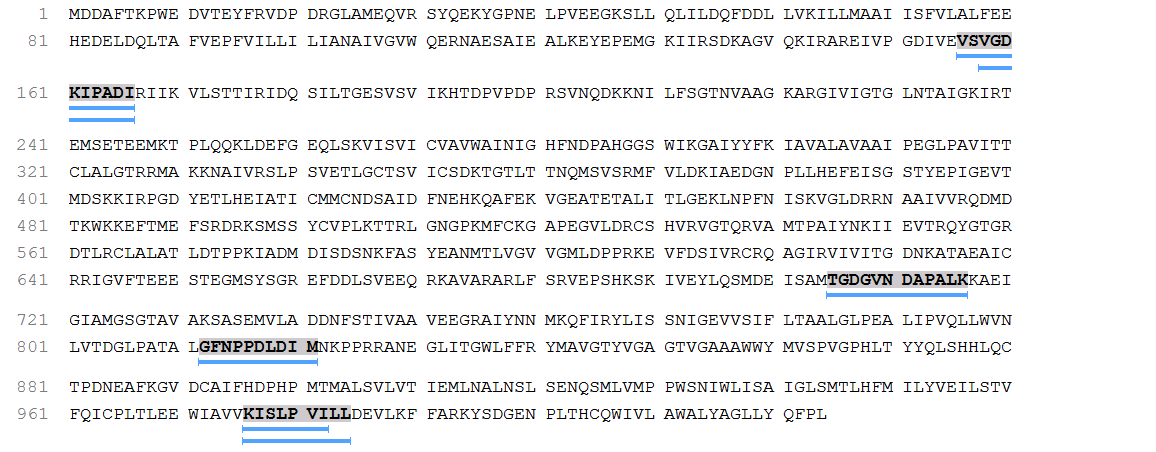

Supplement: Supplementary file 1 [file marinedrugs-24-00036-s001.zip › SM38/HA/img/cov_53331.png]

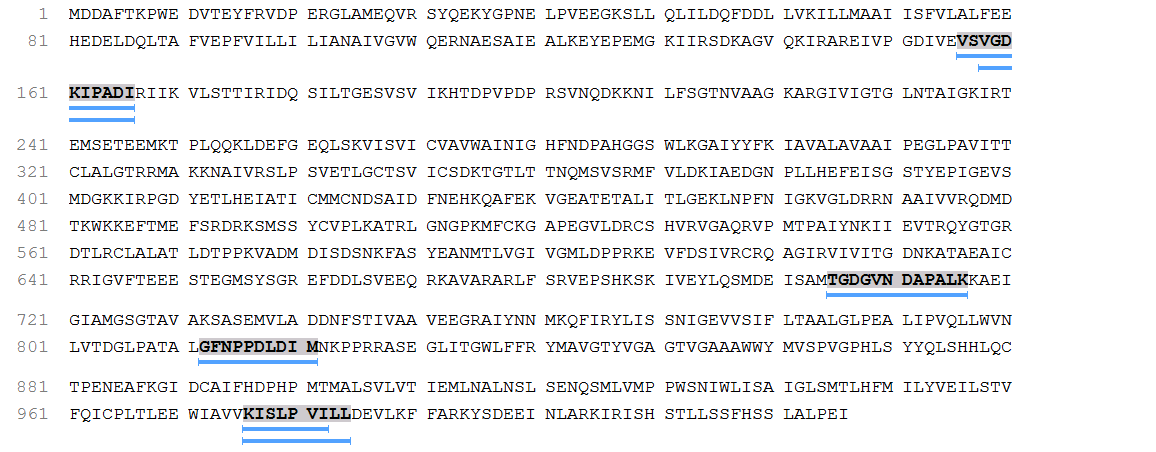

Supplement: Supplementary file 1 [file marinedrugs-24-00036-s001.zip › SM38/HA/img/cov_53332.png]

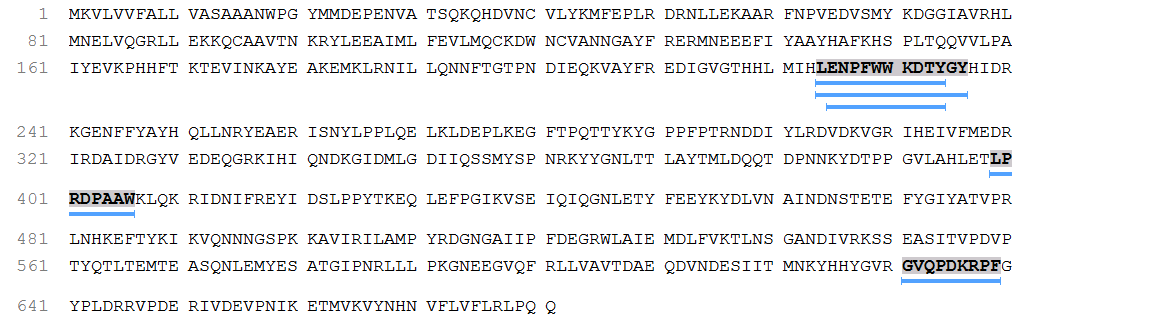

Supplement: Supplementary file 1 [file marinedrugs-24-00036-s001.zip › SM38/HA/img/cov_53363.png]

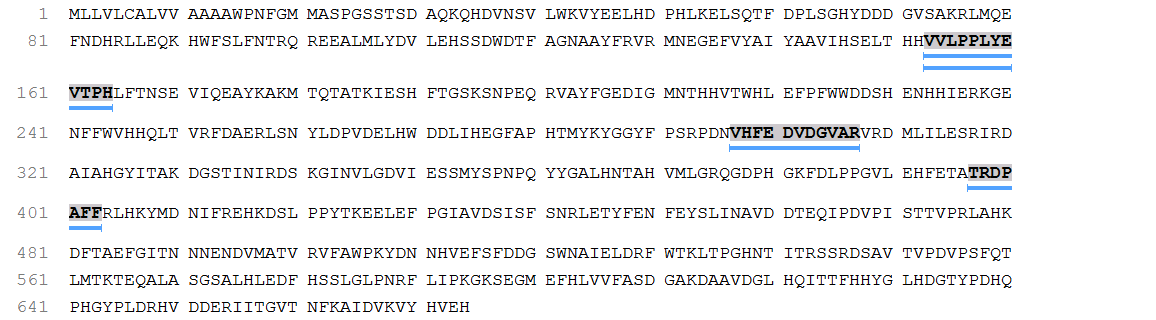

Supplement: Supplementary file 1 [file marinedrugs-24-00036-s001.zip › SM38/HA/img/cov_53378.png]

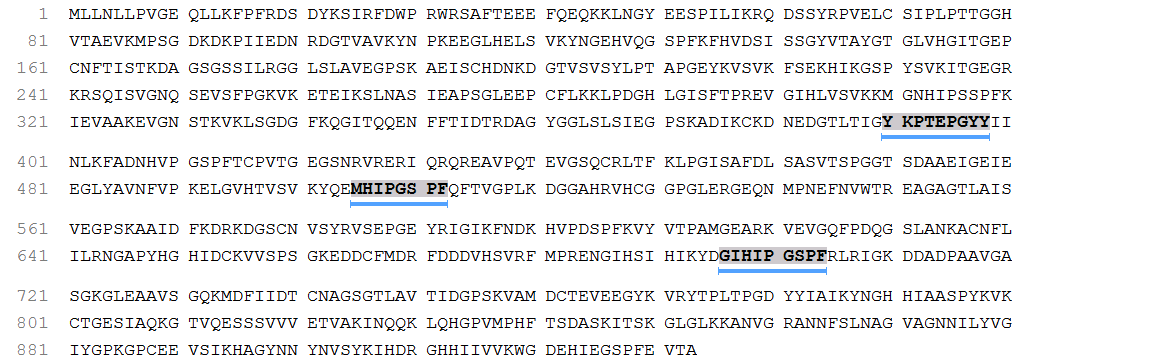

Supplement: Supplementary file 1 [file marinedrugs-24-00036-s001.zip › SM38/HA/img/cov_53379.png]

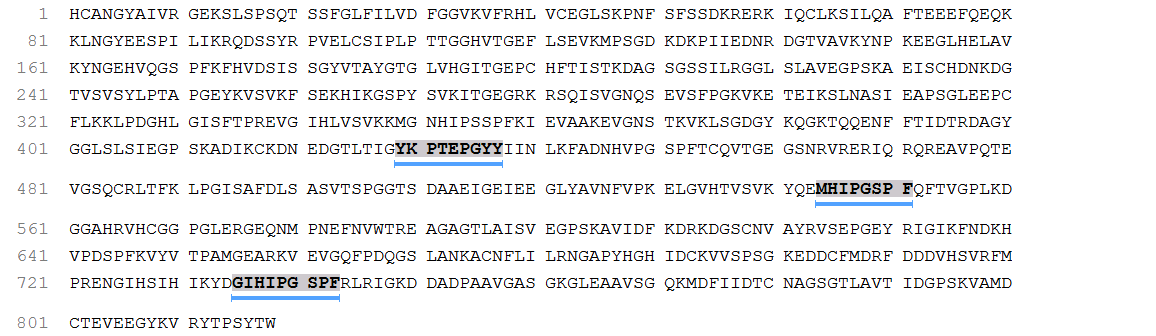

Supplement: Supplementary file 1 [file marinedrugs-24-00036-s001.zip › SM38/HA/img/cov_53380.png]

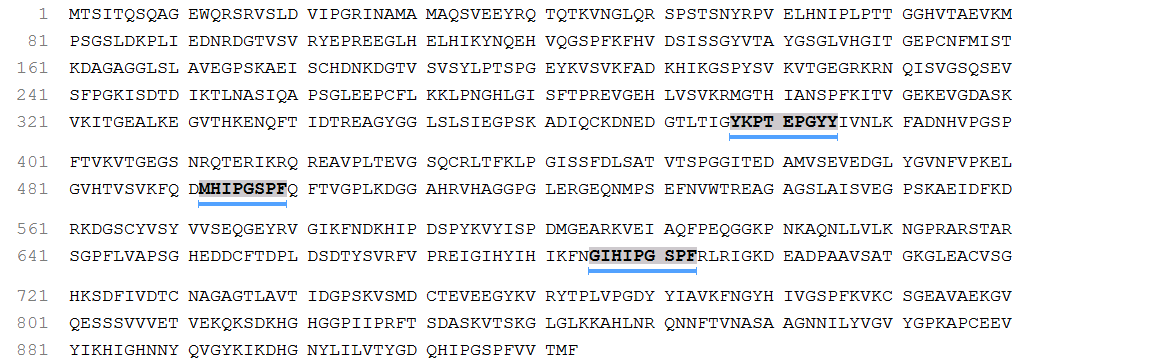

Supplement: Supplementary file 1 [file marinedrugs-24-00036-s001.zip › SM38/HA/img/cov_53381.png]

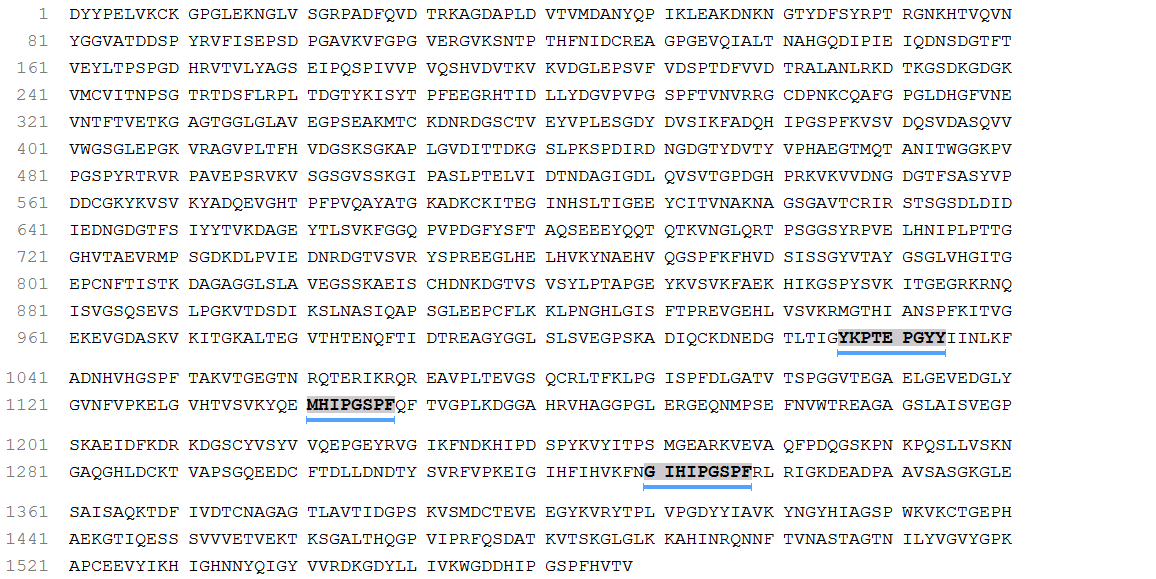

Supplement: Supplementary file 1 [file marinedrugs-24-00036-s001.zip › SM38/HA/img/cov_53382.png]

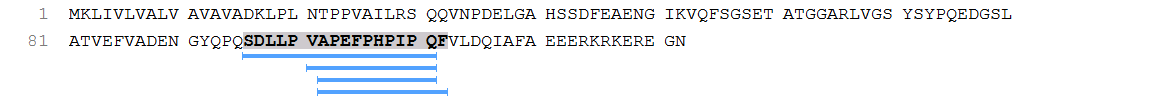

Supplement: Supplementary file 1 [file marinedrugs-24-00036-s001.zip › SM38/HA/img/cov_53387.png]

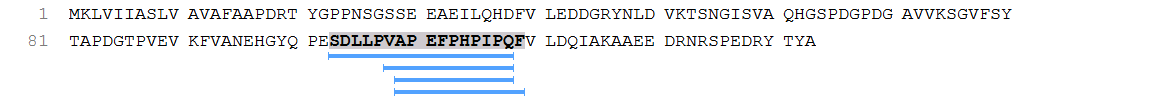

Supplement: Supplementary file 1 [file marinedrugs-24-00036-s001.zip › SM38/HA/img/cov_53388.png]

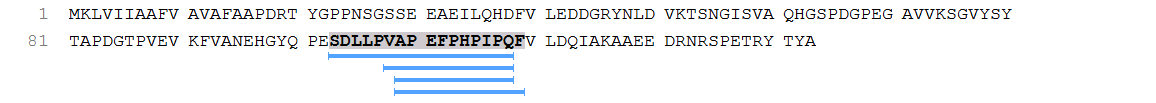

Supplement: Supplementary file 1 [file marinedrugs-24-00036-s001.zip › SM38/HA/img/cov_53389.png]

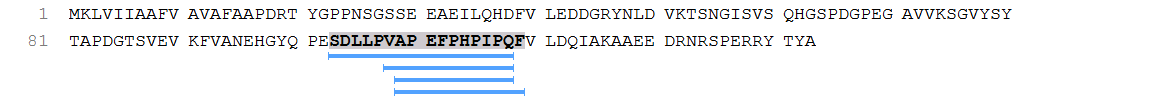

Supplement: Supplementary file 1 [file marinedrugs-24-00036-s001.zip › SM38/HA/img/cov_53390.png]

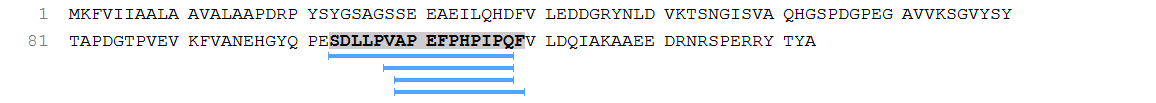

Supplement: Supplementary file 1 [file marinedrugs-24-00036-s001.zip › SM38/HA/img/cov_53391.png]

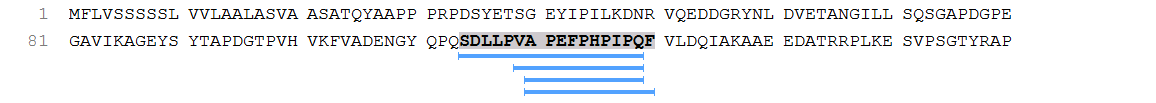

Supplement: Supplementary file 1 [file marinedrugs-24-00036-s001.zip › SM38/HA/img/cov_53392.png]

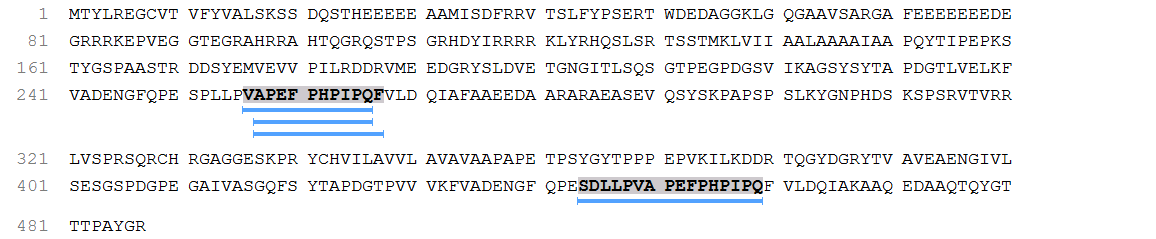

Supplement: Supplementary file 1 [file marinedrugs-24-00036-s001.zip › SM38/HA/img/cov_53393.png]

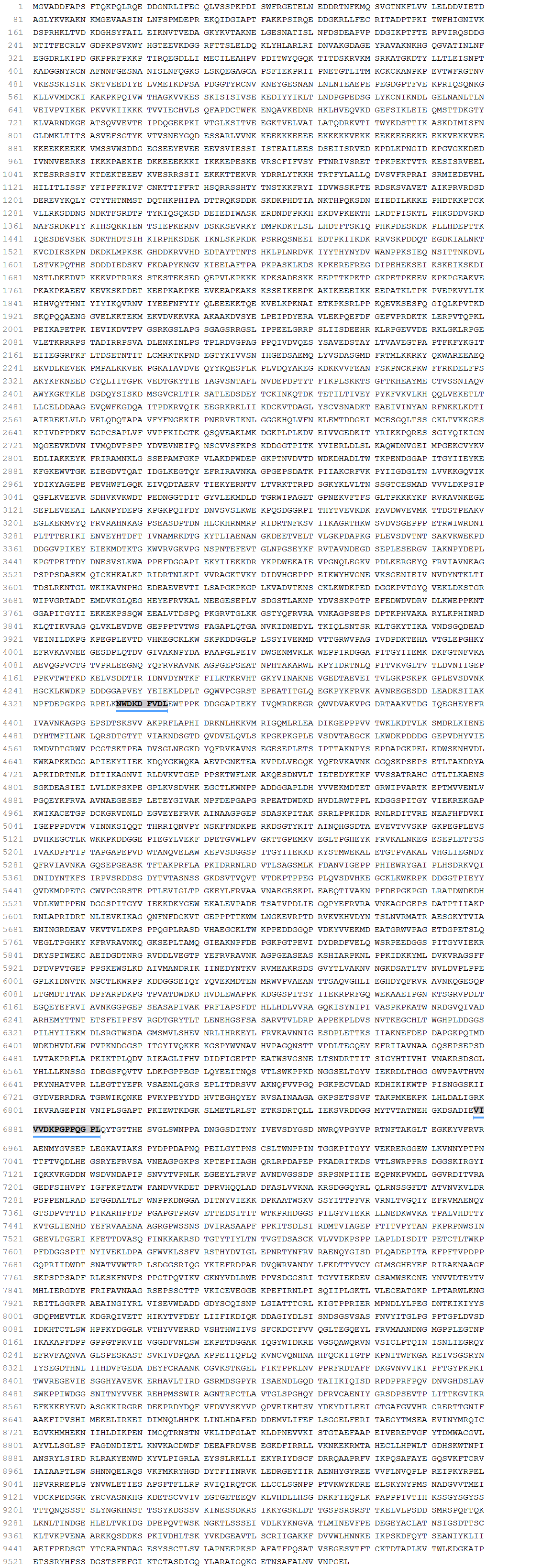

Supplement: Supplementary file 1 [file marinedrugs-24-00036-s001.zip › SM38/HA/img/cov_53394.png]

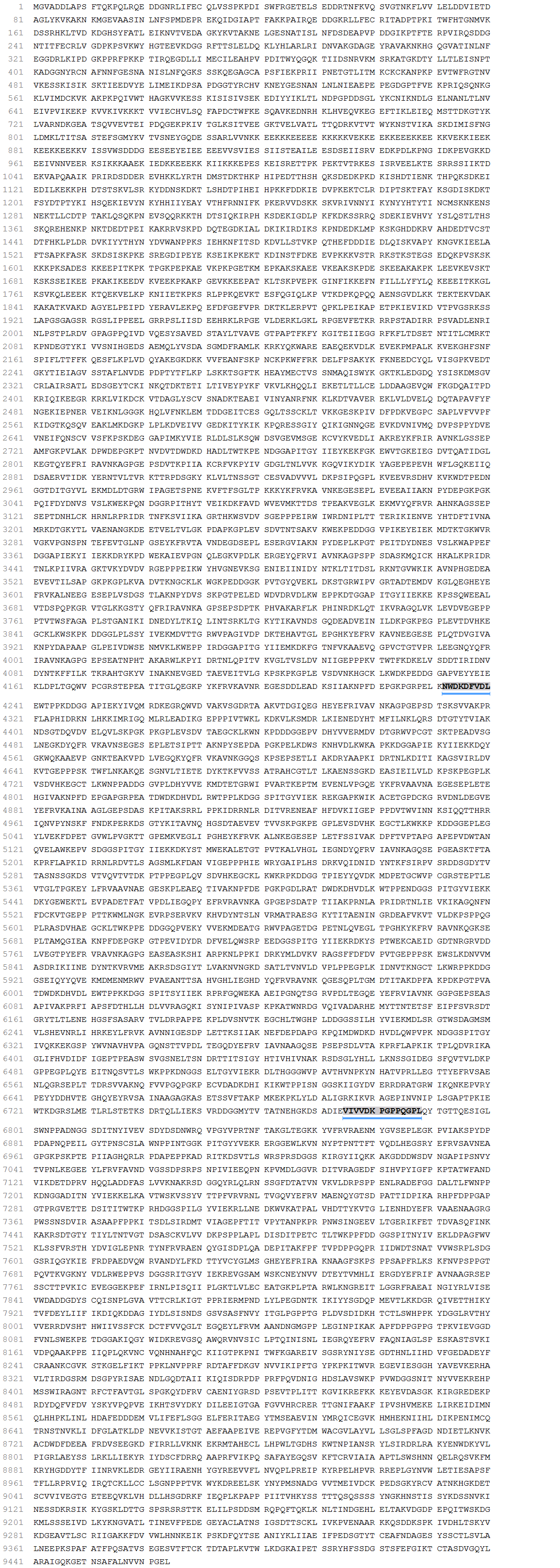

Supplement: Supplementary file 1 [file marinedrugs-24-00036-s001.zip › SM38/HA/img/cov_53396.png]

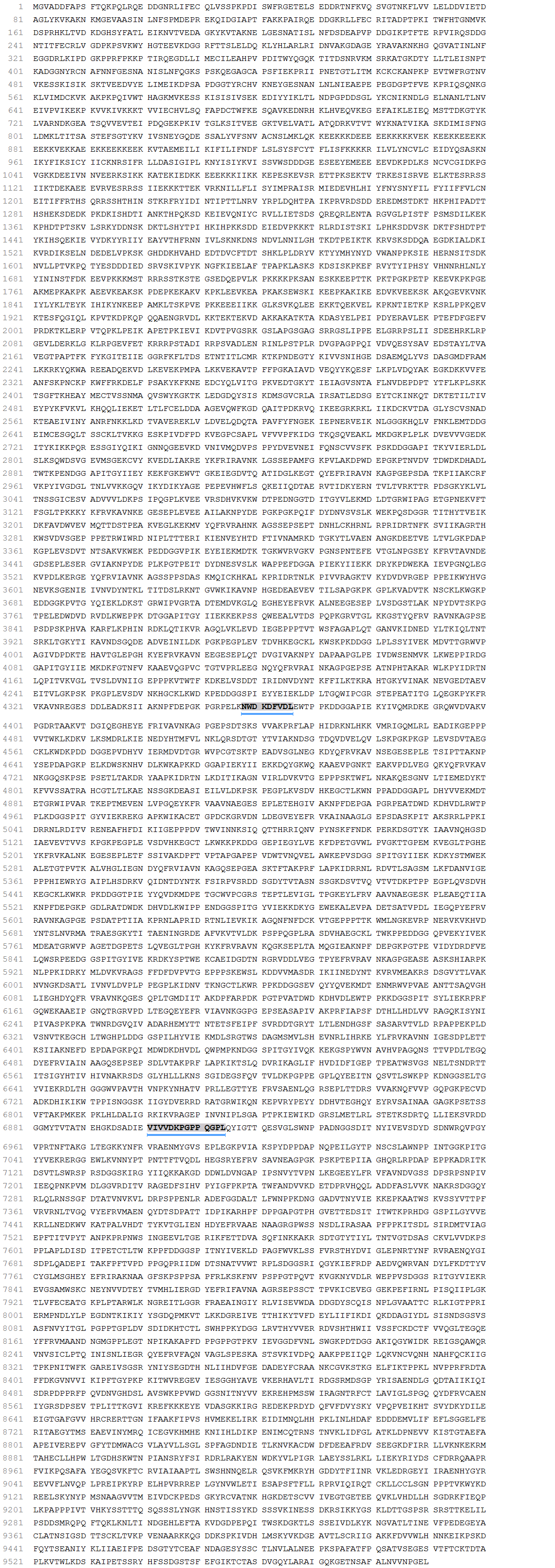

Supplement: Supplementary file 1 [file marinedrugs-24-00036-s001.zip › SM38/HA/img/cov_53397.png]

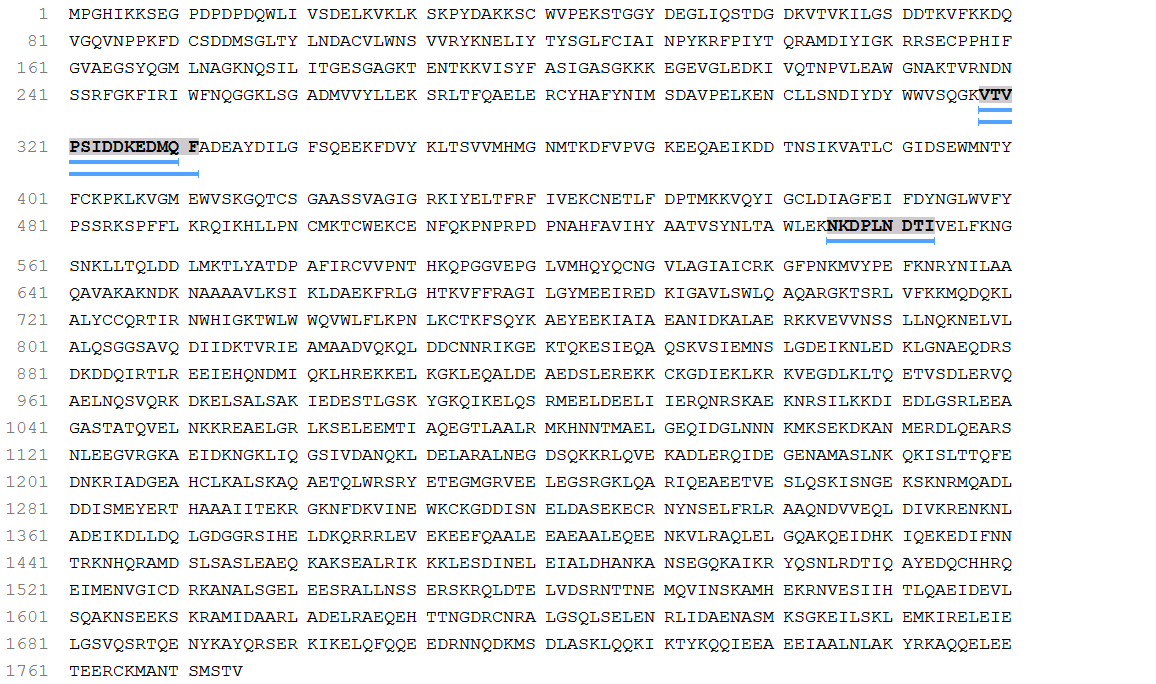

Supplement: Supplementary file 1 [file marinedrugs-24-00036-s001.zip › SM38/HA/img/cov_53398.png]

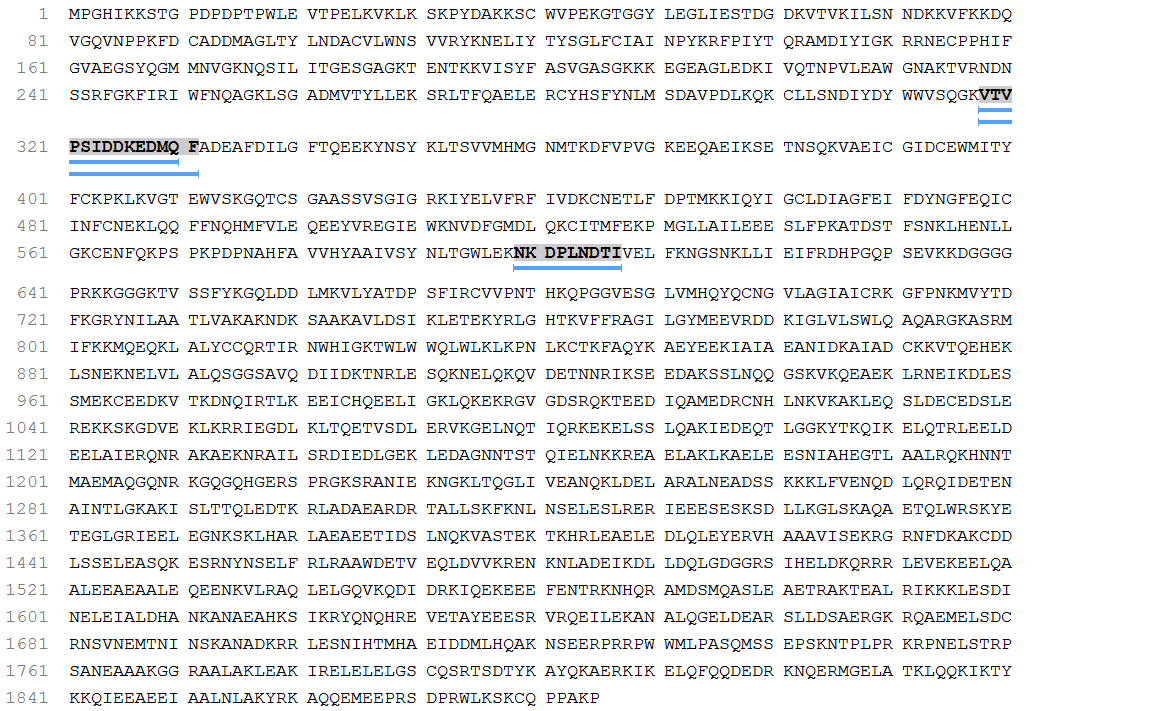

Supplement: Supplementary file 1 [file marinedrugs-24-00036-s001.zip › SM38/HA/img/cov_53399.png]

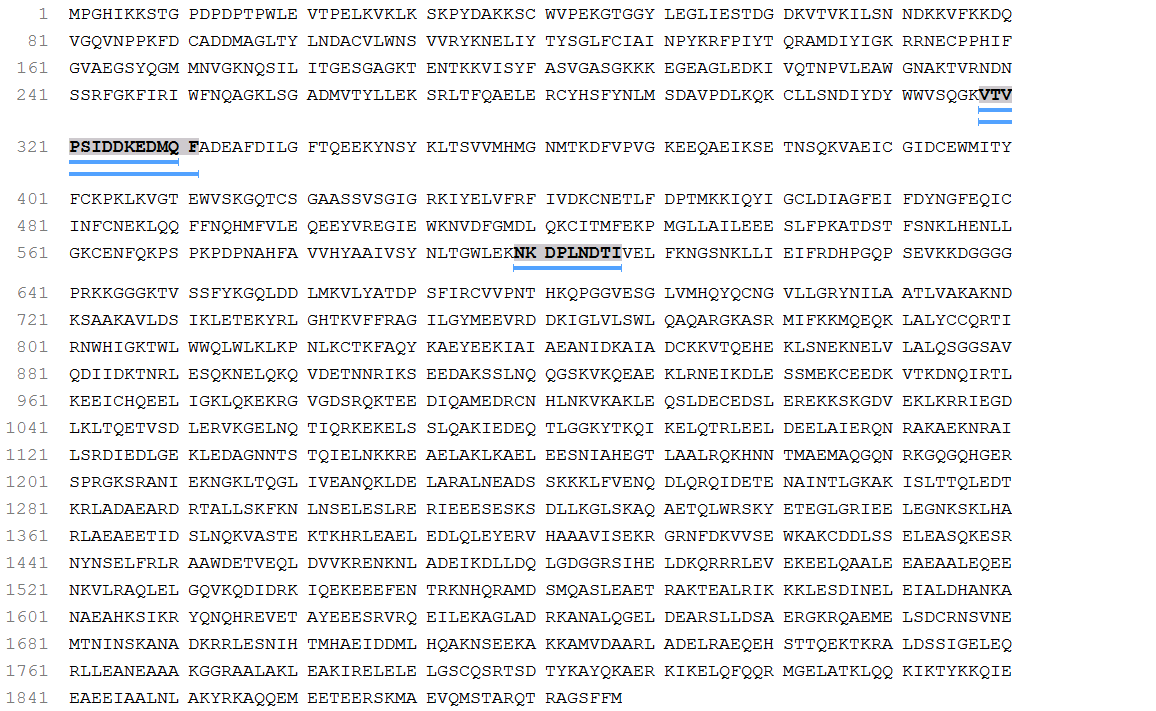

Supplement: Supplementary file 1 [file marinedrugs-24-00036-s001.zip › SM38/HA/img/cov_53400.png]

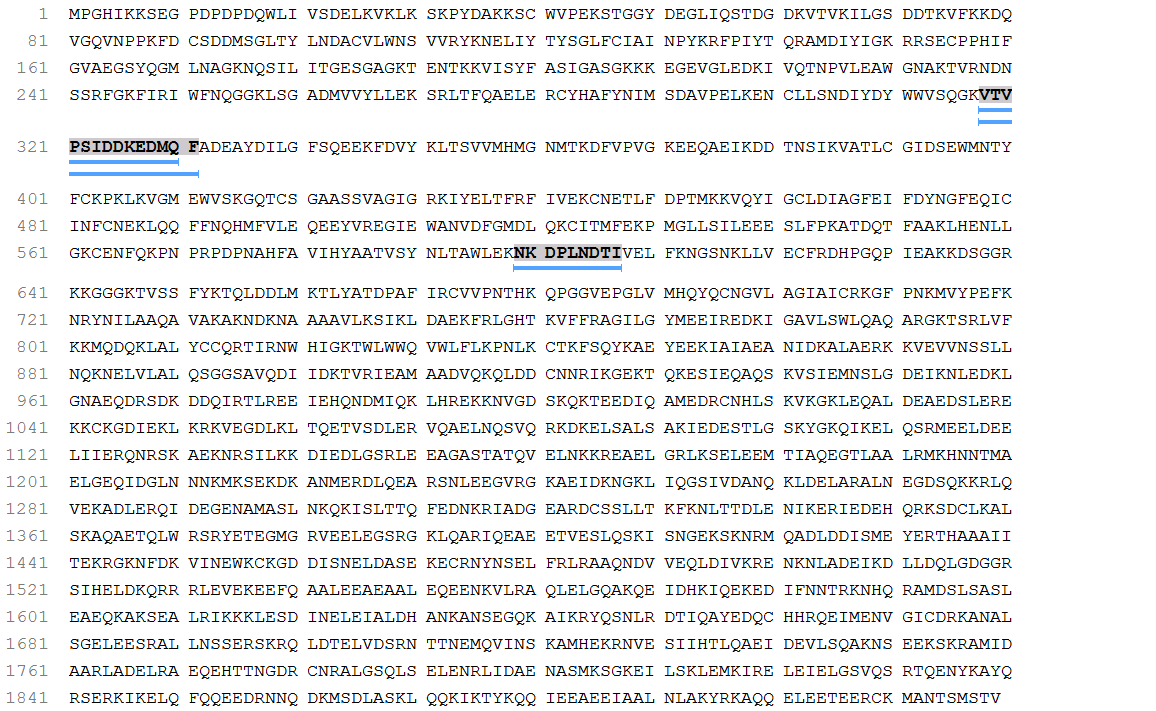

Supplement: Supplementary file 1 [file marinedrugs-24-00036-s001.zip › SM38/HA/img/cov_53401.png]

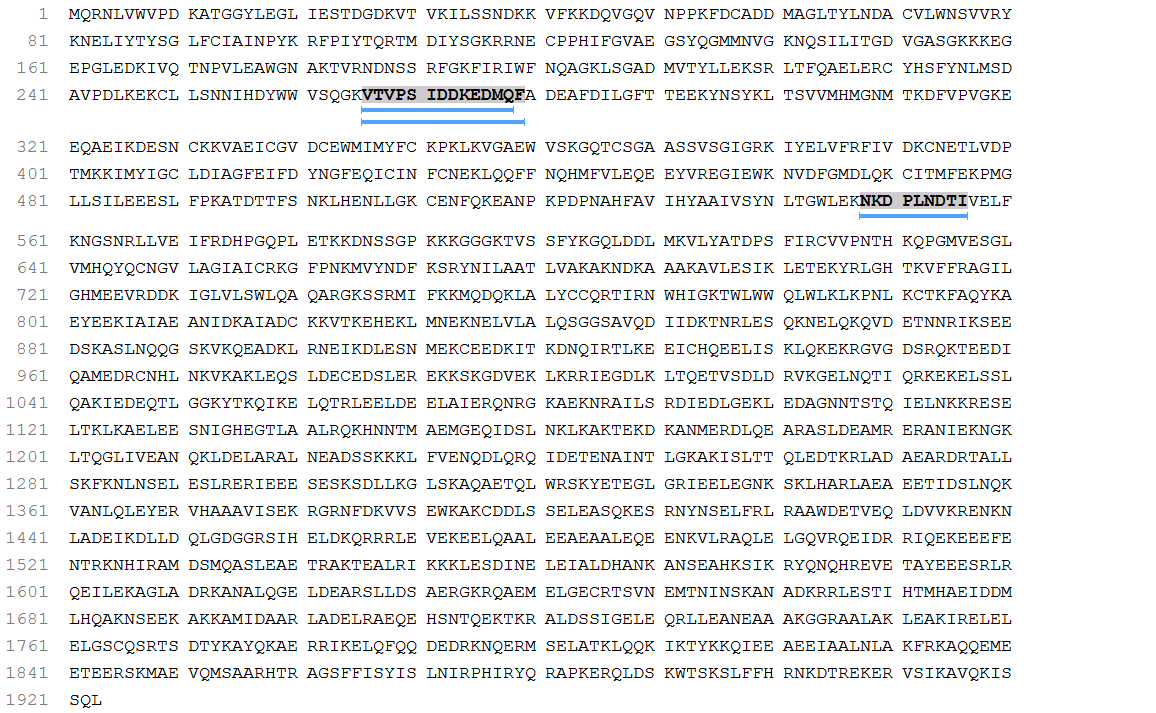

Supplement: Supplementary file 1 [file marinedrugs-24-00036-s001.zip › SM38/HA/img/cov_53402.png]

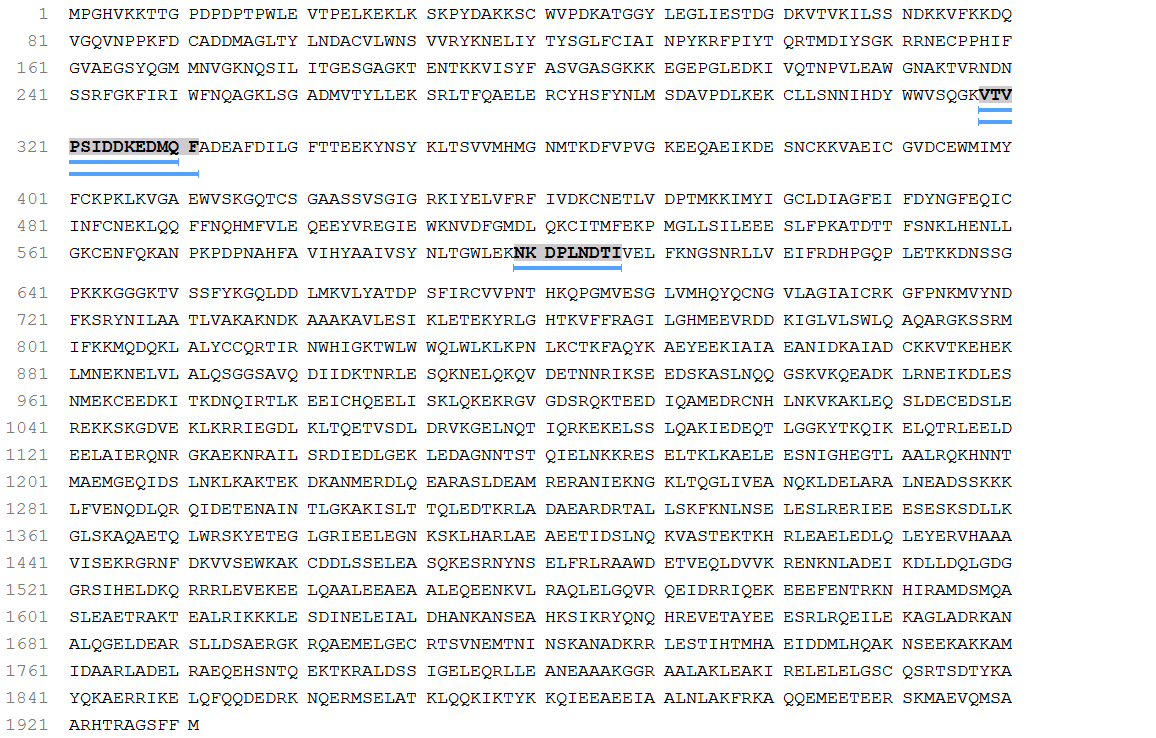

Supplement: Supplementary file 1 [file marinedrugs-24-00036-s001.zip › SM38/HA/img/cov_53403.png]

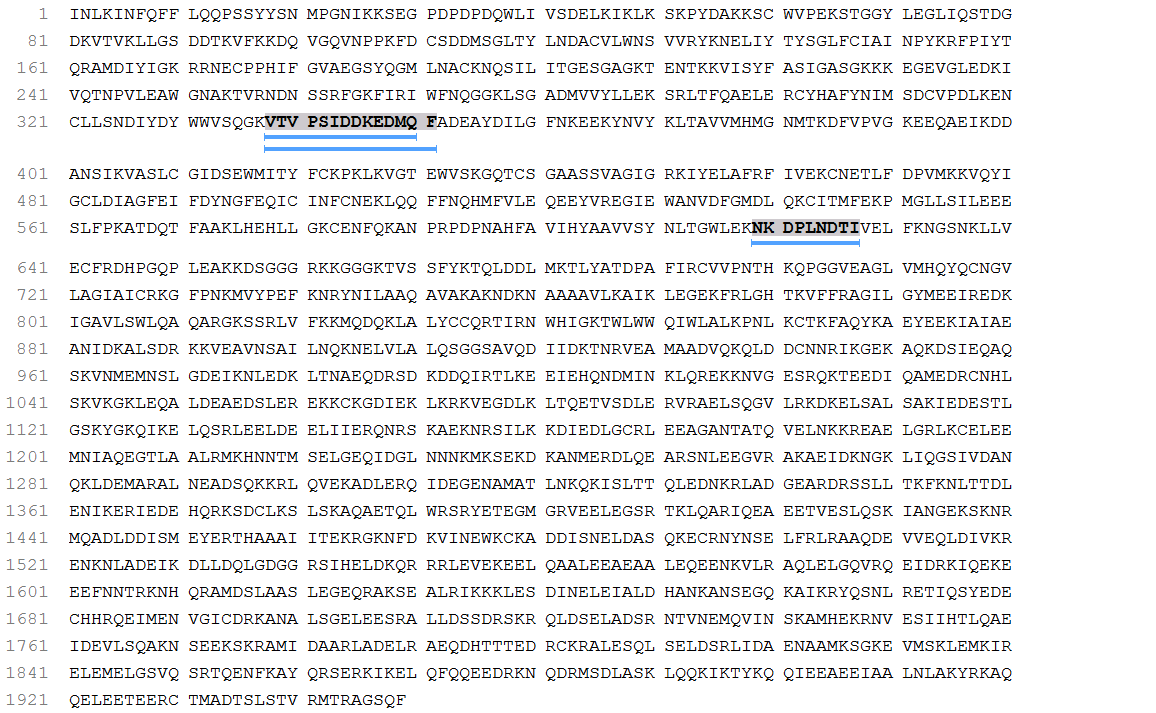

Supplement: Supplementary file 1 [file marinedrugs-24-00036-s001.zip › SM38/HA/img/cov_53404.png]

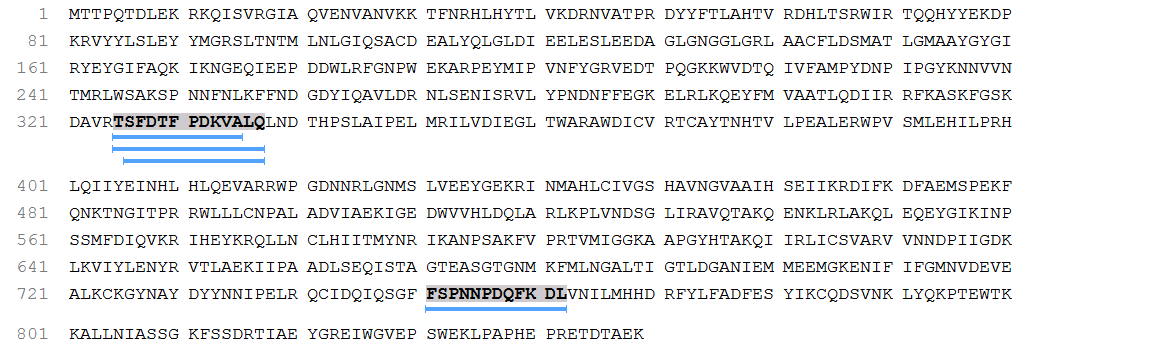

Supplement: Supplementary file 1 [file marinedrugs-24-00036-s001.zip › SM38/HA/img/cov_53411.png]

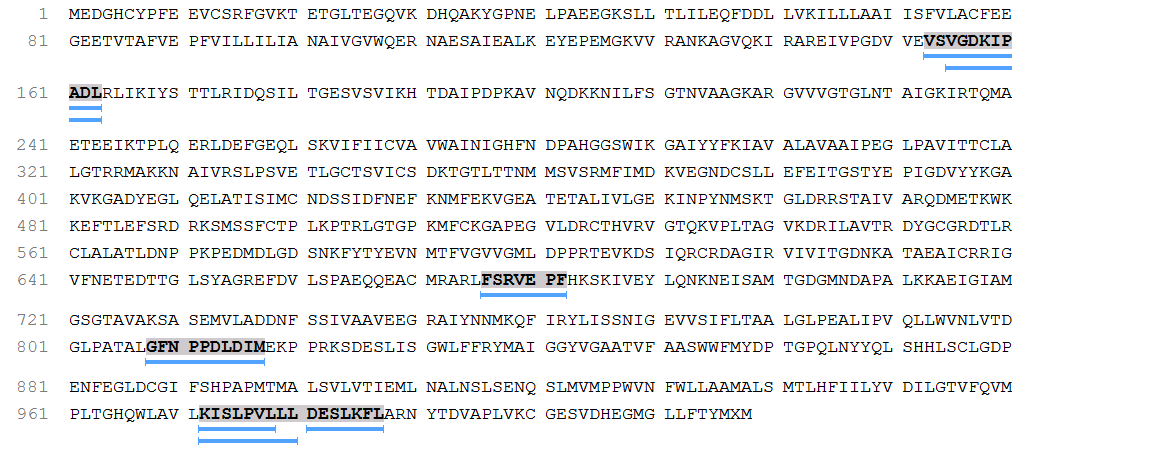

Supplement: Supplementary file 1 [file marinedrugs-24-00036-s001.zip › SM38/HA/img/cov_53418.png]

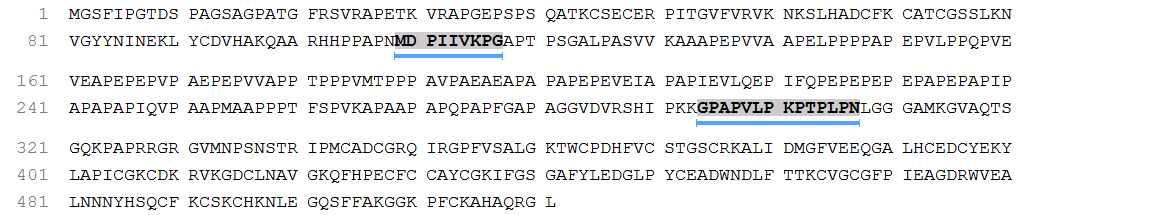

Supplement: Supplementary file 1 [file marinedrugs-24-00036-s001.zip › SM38/HA/img/cov_53419.png]

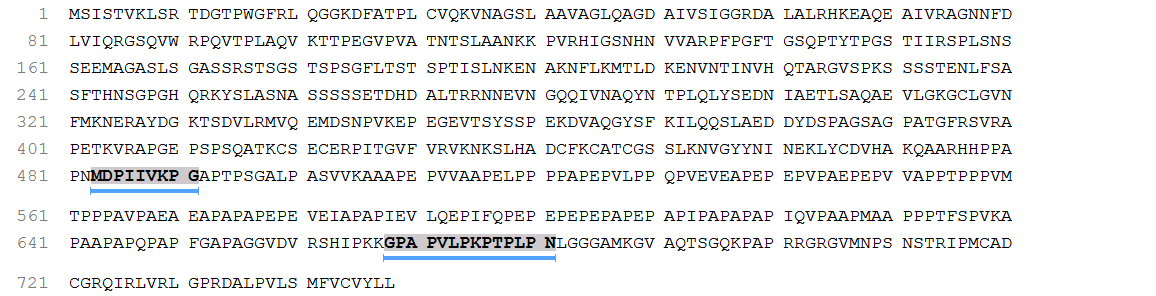

Supplement: Supplementary file 1 [file marinedrugs-24-00036-s001.zip › SM38/HA/img/cov_53420.png]

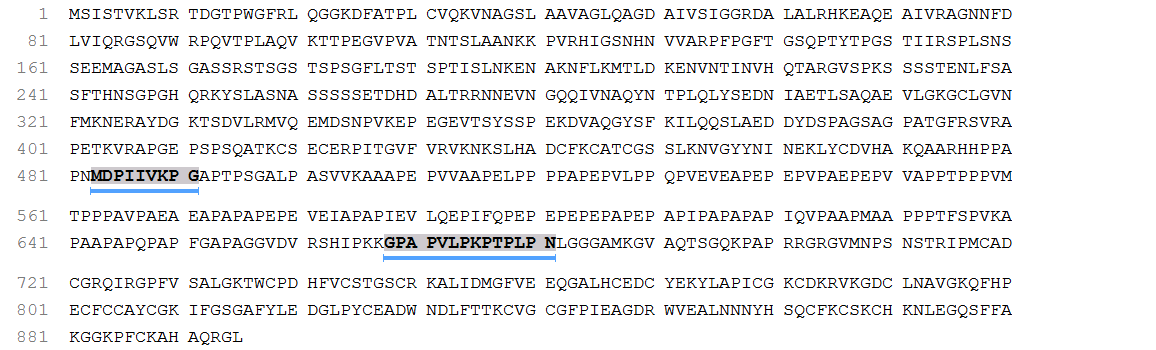

Supplement: Supplementary file 1 [file marinedrugs-24-00036-s001.zip › SM38/HA/img/cov_53421.png]

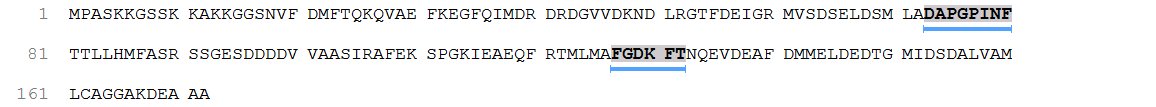

Supplement: Supplementary file 1 [file marinedrugs-24-00036-s001.zip › SM38/HA/img/cov_53427.png]

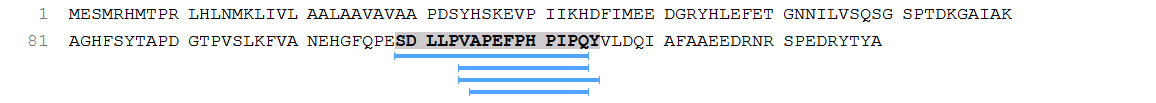

Supplement: Supplementary file 1 [file marinedrugs-24-00036-s001.zip › SM38/HA/img/cov_53428.png]

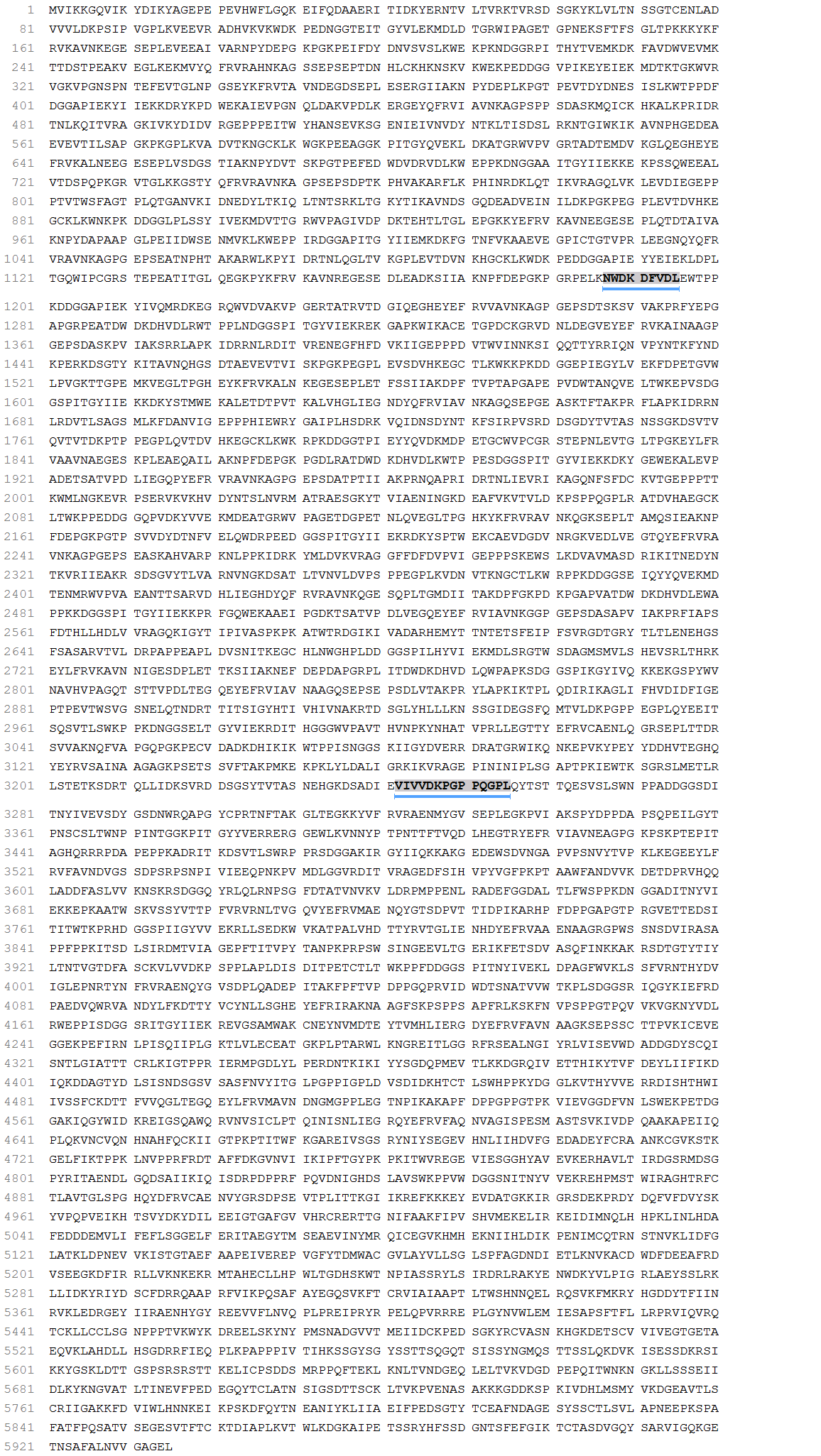

Supplement: Supplementary file 1 [file marinedrugs-24-00036-s001.zip › SM38/HA/img/cov_53469.png]

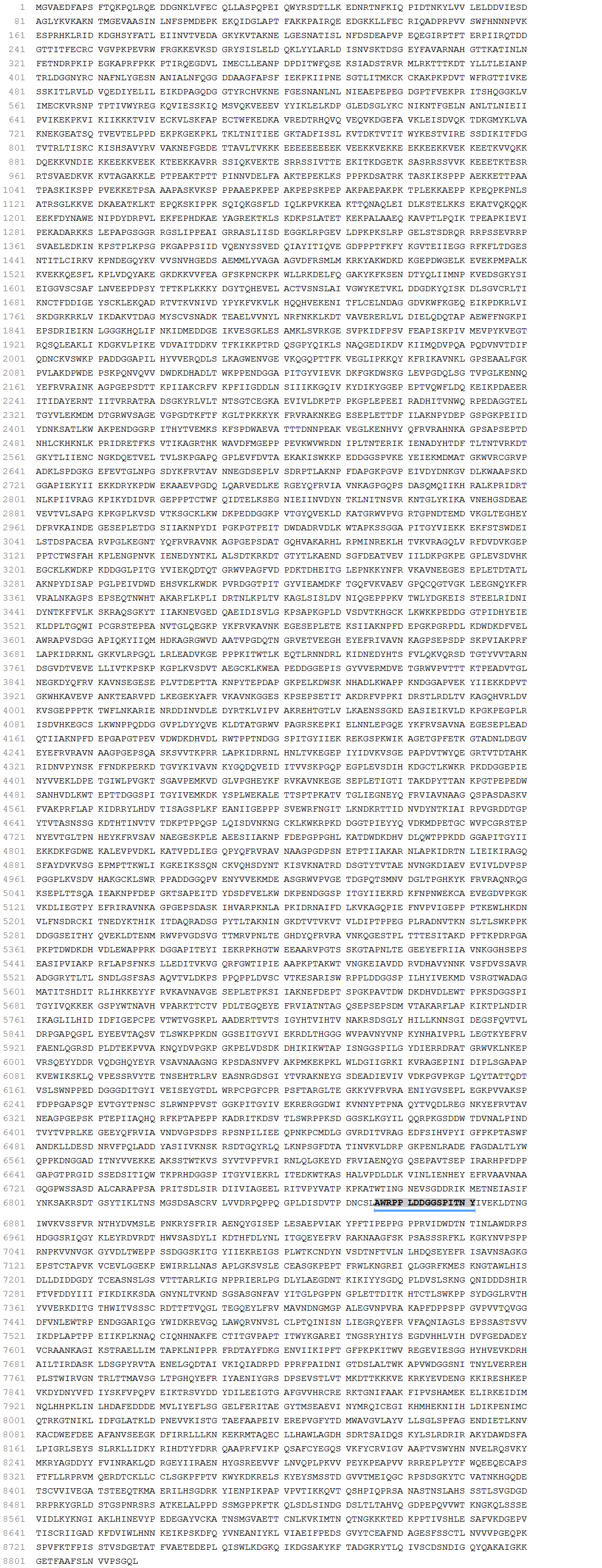

Supplement: Supplementary file 1 [file marinedrugs-24-00036-s001.zip › SM38/HA/img/cov_53503.png]

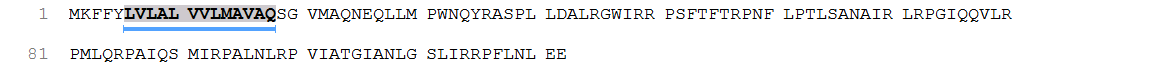

Supplement: Supplementary file 1 [file marinedrugs-24-00036-s001.zip › SM38/HA/img/cov_53529.png]

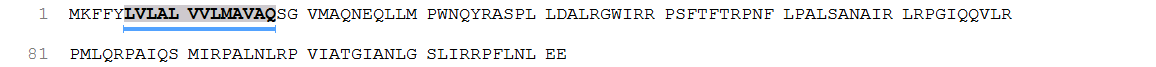

Supplement: Supplementary file 1 [file marinedrugs-24-00036-s001.zip › SM38/HA/img/cov_53530.png]

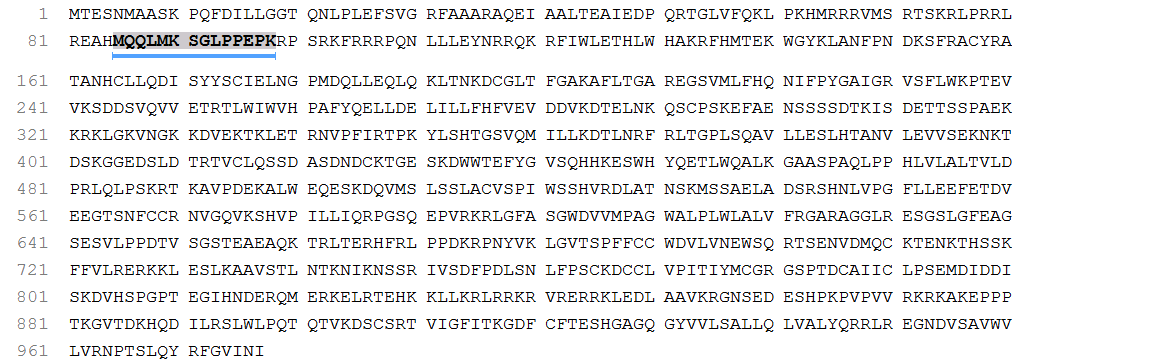

Supplement: Supplementary file 1 [file marinedrugs-24-00036-s001.zip › SM38/HA/img/cov_53532.png]

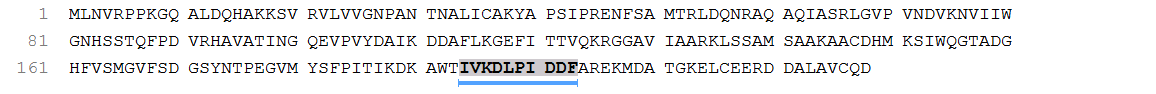

Supplement: Supplementary file 1 [file marinedrugs-24-00036-s001.zip › SM38/HA/img/cov_53533.png]

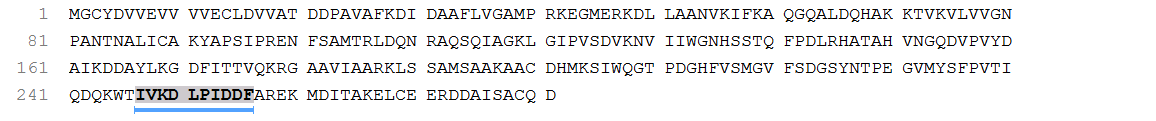

Supplement: Supplementary file 1 [file marinedrugs-24-00036-s001.zip › SM38/HA/img/cov_53534.png]

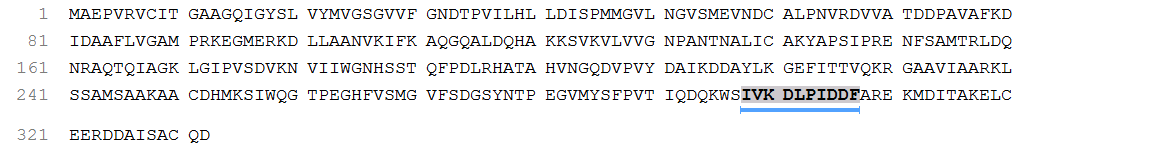

Supplement: Supplementary file 1 [file marinedrugs-24-00036-s001.zip › SM38/HA/img/cov_53535.png]

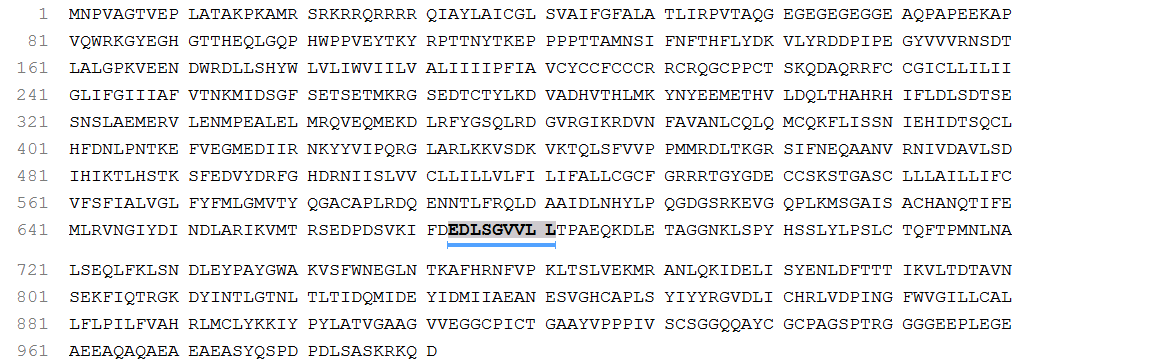

Supplement: Supplementary file 1 [file marinedrugs-24-00036-s001.zip › SM38/HA/img/cov_53537.png]

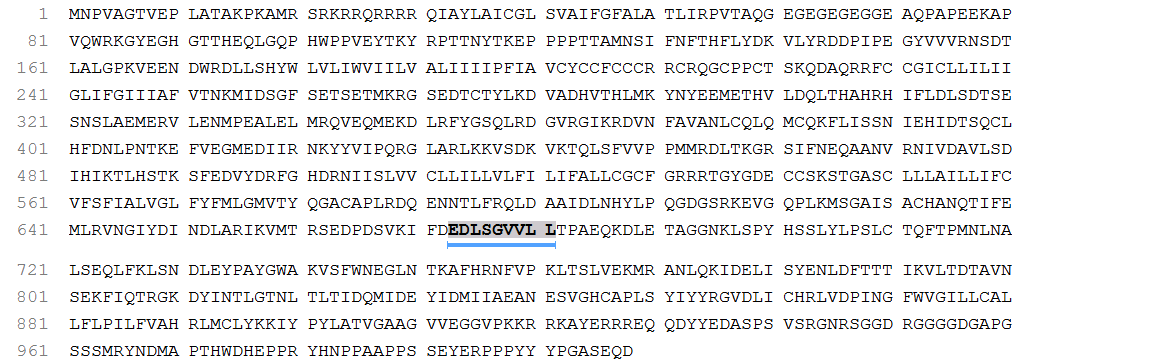

Supplement: Supplementary file 1 [file marinedrugs-24-00036-s001.zip › SM38/HA/img/cov_53538.png]
