# Supplementary material for: Proteomic and Functional Characterization of Antimicrobial Peptides Derived from Fisheries Bycatch via Enzymatic Hydrolysis
Source: Mar Drugs. 2026 Jan 10;24(1):36. doi: 10.3390/md24010036 (PMC12843163; doi:10.3390/md24010036)
Supplement: Supplementary file 1 [file marinedrugs-24-00036-s001.zip › SM39/HP/protein.html]

proteins


Summary

  

# 1. Notes

# 2. Result Statistics

**Figure 1.**
False discovery rate (FDR) curve. X axis is the number of peptide-spectrum matches (PSM) being kept. Y axis is the corresponding FDR.

  


  

**Figure 2.**
PSM score distribution.  **(a)**
Distribution of PEAKS peptide score;  **(b)**
Scatterplot of PEAKS peptide score versus precursor mass error.

**(a)**

**(b)**

**Figure 3.**
De novo result validation. Distribution of residue local confidence:  **(a)**
Residues in de novo sequences validated by confident database peptide assignment;  **(b)**
Residues in "de novo only" sequences.

**(a)**

**(b)**

**Table 1.**
Statistics of data.

|  |  |
| --- | --- |
| # of MS Scans | 1027 |
| # of MS/MS Scans | 2900 |


**Table 2.**
Result filtration parameters.

|  |  |
| --- | --- |
| Peptide -10lgP | ≥15 |
| Protein -10lgP | ≥20 |
| Proteins unique peptides | ≥0 |
| De novo ALC Score | ≥50% |


**Table 3.**
Statistics of filtered result.

|  |  |
| --- | --- |
| Peptide-Spectrum Matches | 96 |
| Peptide Sequences | 96 |
| Protein Groups | 29 |
| Proteins | 144 |
| Proteins (#Unique Peptides) | 39 (>2); 4 (=2); 74 (=1); |
| FDR (Peptide-Spectrum Matches) | 20.8% |
| FDR (Peptide Sequences) | 20.8% |
| FDR (Protein) | 2.8% |
| De Novo Only Spectra | 1110 |

**Table 4.**
PTM profile.

| Name | ∆Mass | #PSM | Position |
| --- | --- | --- | --- |

# 3. Experiment Control

**Figure 4.**
Precursor mass error of peptide-spectrum matches (PSM) in filtered result.  **(a)**
Distribution of precursor mass error in ppm;  **(b)**
Scatterplot of precursor m/z versus precursor mass error in ppm.

**(a)**

**(b)**

**Table 5.**
Number of identified peptides in each sample by the number of missed cleavages

|  |  |  |  |  |  |
| --- | --- | --- | --- | --- | --- |
| Missed Cleavages | 0 | 1 | 2 | 3 | 4+ |
| Orbi2222 | 0 | 0 | 0 | 0 | 96 |

# 4. Other Information

**Table 6.**
Search parameters.

Search Engine Name: PEAKS 7.0  
Parent Mass Error Tolerance: 0.03 Da  
Fragment Mass Error Tolerance: 0.1 Da  
Precursor Mass Search Type: monoisotopic  
Enzyme: None  
Max Missed Cleavages: 100  
Non-specific Cleavage: both  
Max variable PTM per peptide: 3  
Database: Arthropoda  
Taxon: All  
Searched Entry: 6735671  
FDR Estimation: Enabled  
Merge Options: 0.1 min. 0.03 Da  
Precursor Options: corrected  
Charge Options: no correction  
Filter Options: no filter  
Process: true

**Table 7.**
Instrument parameters.

Fractions: OB\_PIMENTA\_HP\_20220211\_04.raw  
Ion Source: ESI(nano-spray)  
Fragmentation Mode: CID/ECD  
MS Scan Mode: FT-ICR/Orbitrap  
MS/MS Scan Mode: Linear Ion Trap

  

Protein List

  

| Protein Group | Protein ID | Accession | -10lgP | Coverage (%) | #Peptides | #Unique | PTM | Avg. Mass | Description |
| --- | --- | --- | --- | --- | --- | --- | --- | --- | --- |
| 1 | 53234 | tr\|A0A3R7M007\|A0A3R7M007\_PENVA | 121.02 | 23 | 17 | 17 | N | 41323 | Actin 1 OS=Penaeus vannamei OX=6689 GN=C7M84\_012584 PE=3 SV=1 |
| 1 | 53236 | tr\|H9B3Y9\|H9B3Y9\_SCYPA | 121.02 | 23 | 17 | 17 | N | 41781 | Beta-actin OS=Scylla paramamosain OX=85552 PE=2 SV=1 |
| 1 | 53237 | tr\|A0A6A0H005\|A0A6A0H005\_HYAAZ | 121.02 | 23 | 17 | 17 | N | 41604 | Uncharacterized protein OS=Hyalella azteca OX=294128 GN=HAZT\_HAZT002759 PE=3 SV=1 |
| 1 | 53239 | tr\|A0A482LUC2\|A0A482LUC2\_CANBE | 121.02 | 23 | 17 | 17 | N | 41781 | Actin 1 OS=Cancer borealis OX=39395 GN=ACT1 PE=2 SV=1 |
| 1 | 53241 | tr\|A0A2H4V3D6\|A0A2H4V3D6\_PENVA | 121.02 | 23 | 17 | 17 | N | 41801 | Slow-type skeletal muscle actin 10 OS=Penaeus vannamei OX=6689 PE=2 SV=1 |
| 5 | 53308 | tr\|A0A5B7IZE3\|A0A5B7IZE3\_PORTR | 78.60 | 17 | 5 | 1 | N | 26459 | Myosin heavy chain, muscle OS=Portunus trituberculatus OX=210409 GN=Mhc\_24 PE=3 SV=1 |
| 6 | 53314 | tr\|F8WR03\|F8WR03\_PENJP | 74.27 | 2 | 5 | 0 | N | 219503 | Myosin heavy chain type a OS=Penaeus japonicus OX=27405 GN=MYHa PE=2 SV=1 |
| 6 | 53315 | tr\|K4Q111\|K4Q111\_PENVA | 74.27 | 2 | 5 | 0 | N | 219712 | Myosin heavy chain type 1 OS=Penaeus vannamei OX=6689 GN=MYH1 PE=2 SV=1 |
| 6 | 53316 | tr\|K4Q4N8\|K4Q4N8\_PENMO | 74.27 | 2 | 5 | 0 | N | 219585 | Myosin heavy chain type 1 OS=Penaeus monodon OX=6687 GN=MYH1 PE=2 SV=1 |
| 4 | 53320 | tr\|A0A4Y7MKW9\|A0A4Y7MKW9\_9CRUS | 74.24 | 5 | 6 | 0 | N | 97229 | P-type Ca(2+) transporter (Fragment) OS=Daphnia magna OX=35525 GN=EOG090X00V6 PE=2 SV=1 |
| 4 | 53321 | tr\|A0A0P5KSW7\|A0A0P5KSW7\_9CRUS | 74.24 | 4 | 6 | 0 | N | 110371 | Calcium-transporting ATPase OS=Daphnia magna OX=35525 PE=3 SV=1 |
| 4 | 53322 | tr\|A0A4Y7MXF9\|A0A4Y7MXF9\_9CRUS | 74.24 | 4 | 6 | 0 | N | 110591 | Calcium-transporting ATPase OS=Daphnia pulicaria OX=35523 GN=EOG090X00V6 PE=2 SV=1 |
| 4 | 53323 | tr\|A0A0P4ZGF4\|A0A0P4ZGF4\_9CRUS | 74.24 | 4 | 6 | 0 | N | 110708 | Calcium-transporting ATPase OS=Daphnia magna OX=35525 GN=APZ42\_012354 PE=3 SV=1 |
| 4 | 53324 | tr\|A0A4Y7MVM2\|A0A4Y7MVM2\_DAPPU | 74.24 | 4 | 6 | 0 | N | 110862 | Calcium-transporting ATPase OS=Daphnia pulex OX=6669 GN=EOG090X00V6 PE=2 SV=1 |
| 4 | 53325 | tr\|A0A4Y7M702\|A0A4Y7M702\_9CRUS | 74.24 | 4 | 6 | 0 | N | 111071 | Calcium-transporting ATPase OS=Daphnia hispanica OX=575233 GN=EOG090X00V6 PE=2 SV=1 |
| 4 | 53326 | tr\|A0A4Y7MHQ5\|A0A4Y7MHQ5\_9CRUS | 74.24 | 4 | 6 | 0 | N | 111377 | Calcium-transporting ATPase OS=Daphnia magna OX=35525 GN=EOG090X00V6 PE=2 SV=1 |
| 4 | 53327 | tr\|A0A4Y7ME47\|A0A4Y7ME47\_9CRUS | 74.24 | 4 | 6 | 0 | N | 111392 | Calcium-transporting ATPase OS=Daphnia magna OX=35525 GN=EOG090X00V6 PE=2 SV=1 |
| 4 | 53328 | tr\|A0A4Y7M4I0\|A0A4Y7M4I0\_9CRUS | 74.24 | 4 | 6 | 0 | N | 111723 | Calcium-transporting ATPase OS=Daphnia dolichocephala OX=2282166 GN=EOG090X00V6 PE=2 SV=1 |
| 4 | 53329 | tr\|A0A1D2MC18\|A0A1D2MC18\_ORCCI | 74.24 | 4 | 6 | 0 | N | 112226 | Calcium-transporting ATPase OS=Orchesella cincta OX=48709 GN=Ocin01\_16136 PE=3 SV=1 |
| 4 | 53330 | tr\|E9HR84\|E9HR84\_DAPPU | 74.24 | 4 | 6 | 0 | N | 113043 | Calcium-transporting ATPase OS=Daphnia pulex OX=6669 GN=DAPPUDRAFT\_65262 PE=3 SV=1 |
| 4 | 53331 | tr\|A0A0P5VJX3\|A0A0P5VJX3\_9CRUS | 74.24 | 4 | 6 | 0 | N | 113098 | Calcium-transporting ATPase OS=Daphnia magna OX=35525 PE=3 SV=1 |
| 4 | 53332 | tr\|A0A4Y7LYL4\|A0A4Y7LYL4\_9CRUS | 74.24 | 4 | 6 | 0 | N | 113136 | Calcium-transporting ATPase OS=Daphnia atkinsoni OX=342845 GN=EOG090X00V6 PE=2 SV=1 |
| 2 | 53318 | tr\|A0A0C5E1J4\|A0A0C5E1J4\_CHEDE | 74.06 | 4 | 6 | 0 | N | 109565 | Calcium-transporting ATPase OS=Cherax destructor OX=6723 PE=2 SV=1 |
| 11 | 55331 | tr\|Q86GD6\|Q86GD6\_PROCL | 73.88 | 0 | 3 | 1 | N | 962665 | Projectin OS=Procambarus clarkii OX=6728 GN=proj PE=2 SV=1 |
| 10 | 53406 | tr\|A0A444TVR3\|A0A444TVR3\_ARMVU | 71.61 | 12 | 4 | 4 | N | 14352 | Cuticle protein AMP1A (Fragment) OS=Armadillidium vulgare OX=13347 GN=Avbf\_10288 PE=4 SV=1 |
| 9 | 53398 | tr\|A0A7R8CX21\|A0A7R8CX21\_LEPSM | 69.38 | 2 | 4 | 0 | N | 202190 | MYH6\_7 OS=Lepeophtheirus salmonis OX=72036 GN=LSAA\_10697 PE=3 SV=1 |
| 9 | 53401 | tr\|A0A0K2V7Y7\|A0A0K2V7Y7\_LEPSM | 69.38 | 2 | 4 | 0 | N | 218544 | Myosin heavy chain 1 [Tribolium castaneum] OS=Lepeophtheirus salmonis OX=72036 GN=Mhc1 PE=3 SV=1 |
| 9 | 53399 | tr\|A0A7T8HL88\|A0A7T8HL88\_CALRO | 69.38 | 2 | 4 | 0 | N | 215000 | Myosin heavy chain 1 OS=Caligus rogercresseyi OX=217165 GN=FKW44\_003971 PE=3 SV=1 |
| 9 | 53400 | tr\|A0A7T8HL29\|A0A7T8HL29\_CALRO | 69.38 | 2 | 4 | 0 | N | 214908 | Myosin heavy chain 1 OS=Caligus rogercresseyi OX=217165 GN=FKW44\_003975 PE=3 SV=1 |
| 9 | 53403 | tr\|A0A0K2T3U6\|A0A0K2T3U6\_LEPSM | 69.38 | 2 | 4 | 0 | N | 220566 | Myosin heavy chain 1 [Tribolium castaneum] OS=Lepeophtheirus salmonis OX=72036 GN=Mhc1 PE=3 SV=1 |
| 9 | 53404 | tr\|A0A0K2TSJ7\|A0A0K2TSJ7\_LEPSM | 69.38 | 2 | 4 | 0 | N | 222210 | Myosin heavy chain 1 [Tribolium castaneum] (Fragment) OS=Lepeophtheirus salmonis OX=72036 GN=Mhc1 PE=3 SV=1 |
| 14 | 55341 | tr\|A0A0P4WFG0\|A0A0P4WFG0\_SCYOL | 62.37 | 4 | 3 | 3 | N | 64765 | Uncharacterized protein OS=Scylla olivacea OX=85551 PE=3 SV=1 |
| 14 | 55342 | tr\|A0A5B7GFK1\|A0A5B7GFK1\_PORTR | 62.37 | 4 | 3 | 3 | N | 64777 | Sodium/calcium exchanger 3 OS=Portunus trituberculatus OX=210409 GN=Slc8a3 PE=3 SV=1 |
| 14 | 55343 | tr\|A0A110A0P3\|A0A110A0P3\_ASTLP | 62.37 | 2 | 3 | 3 | N | 100282 | Sodium-calcium exchanger OS=Astacus leptodactylus OX=6717 PE=2 SV=1 |
| 12 | 55333 | tr\|A0A5B7EE36\|A0A5B7EE36\_PORTR | 58.38 | 2 | 3 | 0 | N | 161842 | Myosin heavy chain, muscle OS=Portunus trituberculatus OX=210409 GN=Mhc\_15 PE=3 SV=1 |
| 3 | 53418 | tr\|A0A2Z4I2U3\|A0A2Z4I2U3\_CALSI | 58.27 | 4 | 6 | 0 | N | 111733 | Calcium-transporting ATPase OS=Callinectes sapidus OX=6763 GN=SERCA PE=2 SV=1 |
| 16 | 55332 | tr\|Q95YM2\|Q95YM2\_PROCL | 52.84 | 0 | 2 | 2 | N | 1962327 | I-connectin OS=Procambarus clarkii OX=6728 GN=I-con PE=2 SV=1 |
| 8 | 55348 | tr\|A0A6P7F415\|A0A6P7F415\_DIAVI | 50.86 | 2 | 5 | 0 | N | 224155 | myosin heavy chain, muscle isoform X15 OS=Diabrotica virgifera virgifera OX=50390 GN=LOC114326036 PE=3 SV=1 |
| 13 | 53782 | tr\|A0A5E4QJ96\|A0A5E4QJ96\_9NEOP | 50.28 | 9 | 3 | 3 | N | 14403 | Fructose-bisphosphate aldolase OS=Leptidea sinapis OX=189913 GN=LSINAPIS\_LOCUS8982 PE=3 SV=1 |
| 13 | 53783 | tr\|A0A0A7BYS9\|A0A0A7BYS9\_OSTFU | 50.28 | 6 | 3 | 3 | N | 22770 | Fructose-bisphosphate aldolase (Fragment) OS=Ostrinia furnacalis OX=93504 PE=2 SV=1 |
| 13 | 53784 | tr\|A0A2H1WAZ2\|A0A2H1WAZ2\_SPOFR | 50.28 | 5 | 3 | 3 | N | 25625 | Fructose-bisphosphate aldolase (Fragment) OS=Spodoptera frugiperda OX=7108 GN=SFRICE\_035466 PE=3 SV=1 |
| 13 | 53785 | tr\|A0A6J3BVA9\|A0A6J3BVA9\_GALME | 50.28 | 5 | 3 | 3 | N | 26581 | Fructose-bisphosphate aldolase OS=Galleria mellonella OX=7137 GN=LOC113515179 PE=3 SV=1 |
| 13 | 53786 | tr\|A0A6J1WK99\|A0A6J1WK99\_GALME | 50.28 | 5 | 3 | 3 | N | 26425 | Fructose-bisphosphate aldolase OS=Galleria mellonella OX=7137 GN=LOC113515179 PE=3 SV=1 |
| 13 | 54142 | tr\|A0A444T6K9\|A0A444T6K9\_ARMVU | 50.28 | 4 | 3 | 3 | N | 34837 | Fructose-bisphosphate aldolase OS=Armadillidium vulgare OX=13347 GN=Avbf\_05003 PE=3 SV=1 |
| 13 | 53787 | tr\|A0A222AJC5\|A0A222AJC5\_SPOLI | 50.28 | 4 | 3 | 3 | N | 36607 | Fructose-bisphosphate aldolase (Fragment) OS=Spodoptera littoralis OX=7109 PE=3 SV=1 |
| 13 | 53788 | tr\|A0A0Q9WRY5\|A0A0Q9WRY5\_DROVI | 50.28 | 3 | 3 | 3 | N | 38982 | Fructose-bisphosphate aldolase OS=Drosophila virilis OX=7244 GN=Dvir\GJ23350 PE=3 SV=1 |
| 13 | 53789 | tr\|B4NKQ7\|B4NKQ7\_DROWI | 50.28 | 3 | 3 | 3 | N | 38856 | Fructose-bisphosphate aldolase OS=Drosophila willistoni OX=7260 GN=Dwil\GK13316 PE=3 SV=2 |
| 13 | 53790 | tr\|B4LYD3\|B4LYD3\_DROVI | 50.28 | 3 | 3 | 3 | N | 39331 | Fructose-bisphosphate aldolase OS=Drosophila virilis OX=7244 GN=Dvir\GJ23350 PE=3 SV=1 |
| 13 | 53791 | tr\|A0A0Q9WWZ4\|A0A0Q9WWZ4\_DROWI | 50.28 | 3 | 3 | 3 | N | 39342 | Fructose-bisphosphate aldolase OS=Drosophila willistoni OX=7260 GN=Dwil\GK13316 PE=3 SV=1 |
| 13 | 53792 | tr\|A0A0Q9WQB4\|A0A0Q9WQB4\_DROVI | 50.28 | 3 | 3 | 3 | N | 39454 | Fructose-bisphosphate aldolase OS=Drosophila virilis OX=7244 GN=Dvir\GJ23350 PE=3 SV=1 |
| 13 | 53793 | tr\|A0A2A4K0C6\|A0A2A4K0C6\_HELVI | 50.28 | 3 | 3 | 3 | N | 39578 | Fructose-bisphosphate aldolase OS=Heliothis virescens OX=7102 GN=B5V51\_7176 PE=3 SV=1 |
| 13 | 53794 | tr\|A0A6J1NBL3\|A0A6J1NBL3\_BICAN | 50.28 | 3 | 3 | 3 | N | 39565 | Fructose-bisphosphate aldolase OS=Bicyclus anynana OX=110368 GN=LOC112048916 PE=3 SV=1 |
| 13 | 53795 | tr\|A0A6J2KNU6\|A0A6J2KNU6\_BOMMA | 50.28 | 3 | 3 | 3 | N | 39653 | Fructose-bisphosphate aldolase OS=Bombyx mandarina OX=7092 GN=LOC114252770 PE=3 SV=1 |
| 13 | 53796 | tr\|A0A6J2KQG2\|A0A6J2KQG2\_BOMMA | 50.28 | 3 | 3 | 3 | N | 39554 | Fructose-bisphosphate aldolase OS=Bombyx mandarina OX=7092 GN=LOC114252770 PE=3 SV=1 |
| 13 | 53797 | tr\|Q1HPN7\|Q1HPN7\_BOMMO | 50.28 | 3 | 3 | 3 | N | 39653 | Fructose-bisphosphate aldolase OS=Bombyx mori OX=7091 GN=ALD PE=2 SV=1 |
| 13 | 53798 | tr\|S4PKR2\|S4PKR2\_9NEOP | 50.28 | 3 | 3 | 3 | N | 39565 | Fructose-bisphosphate aldolase OS=Pararge aegeria OX=116150 PE=3 SV=1 |
| 13 | 53799 | tr\|A0A7S5LJX7\|A0A7S5LJX7\_BEMTA | 50.28 | 3 | 3 | 3 | N | 39474 | Fructose-bisphosphate aldolase OS=Bemisia tabaci OX=7038 PE=2 SV=1 |
| 13 | 53800 | tr\|A0A7R9X220\|A0A7R9X220\_9CRUS | 50.28 | 3 | 3 | 3 | N | 39795 | Fructose-bisphosphate aldolase (Fragment) OS=Argulus foliaceus OX=509924 GN=AFOL1\_LOCUS42511 PE=3 SV=1 |
| 13 | 53801 | tr\|A0A7R9A8N4\|A0A7R9A8N4\_9CRUS | 50.28 | 3 | 3 | 3 | N | 40451 | Fructose-bisphosphate aldolase OS=Darwinula stevensoni OX=69355 GN=DSTB1V02\_LOCUS9328 PE=3 SV=1 |
| 13 | 53802 | tr\|A0A7R9A8M7\|A0A7R9A8M7\_9CRUS | 50.28 | 3 | 3 | 3 | N | 40361 | Fructose-bisphosphate aldolase OS=Darwinula stevensoni OX=69355 GN=DSTB1V02\_LOCUS9328 PE=3 SV=1 |
| 13 | 53803 | tr\|A0A4C1UD71\|A0A4C1UD71\_EUMVA | 50.28 | 3 | 3 | 3 | N | 41921 | Fructose-bisphosphate aldolase OS=Eumeta variegata OX=151549 GN=Ald PE=3 SV=1 |
| 13 | 53804 | tr\|A0A6J2KM35\|A0A6J2KM35\_BOMMA | 50.28 | 3 | 3 | 3 | N | 41930 | Fructose-bisphosphate aldolase OS=Bombyx mandarina OX=7092 GN=LOC114252770 PE=3 SV=1 |
| 13 | 53805 | tr\|A0A821PCJ9\|A0A821PCJ9\_9NEOP | 50.28 | 3 | 3 | 3 | N | 42283 | Hypothetical protein OS=Pieris macdunnoughi OX=345717 GN=PMACD\_LOCUS3545 PE=4 SV=1 |
| 13 | 53806 | tr\|A0A7E5VF38\|A0A7E5VF38\_TRINI | 50.28 | 3 | 3 | 3 | N | 42482 | Fructose-bisphosphate aldolase OS=Trichoplusia ni OX=7111 GN=LOC113493218 PE=3 SV=1 |
| 13 | 53807 | tr\|A0A7I1A027\|A0A7I1A027\_DANPL | 50.28 | 3 | 3 | 3 | N | 44674 | Fructose-bisphosphate aldolase OS=Danaus plexippus plexippus OX=278856 GN=LOC116777233 PE=3 SV=1 |
| 13 | 53808 | tr\|A0A821PCK8\|A0A821PCK8\_9NEOP | 50.28 | 3 | 3 | 3 | N | 44744 | Hypothetical protein OS=Pieris macdunnoughi OX=345717 GN=PMACD\_LOCUS3545 PE=4 SV=1 |
| 13 | 53809 | tr\|A0A811X398\|A0A811X398\_PLUXY | 50.28 | 3 | 3 | 3 | N | 45068 | (diamondback moth) hypothetical protein OS=Plutella xylostella OX=51655 GN=PLXY1\_LOCUS14297 PE=4 SV=1 |
| 13 | 53810 | tr\|A0A2P8YG29\|A0A2P8YG29\_BLAGE | 50.28 | 2 | 3 | 3 | N | 66209 | Fructose-bisphosphate aldolase (Fragment) OS=Blattella germanica OX=6973 GN=C0J52\_17262 PE=3 SV=1 |
| 7 | 53302 | tr\|A0A5B7DQZ0\|A0A5B7DQZ0\_PORTR | 49.27 | 1 | 5 | 0 | N | 352200 | Myosin heavy chain, muscle OS=Portunus trituberculatus OX=210409 GN=Mhc\_1 PE=3 SV=1 |
| 19 | 53698 | tr\|A0A821V8C5\|A0A821V8C5\_9NEOP | 47.29 | 0 | 2 | 1 | N | 829790 | Hypothetical protein OS=Pieris macdunnoughi OX=345717 GN=PMACD\_LOCUS11411 PE=4 SV=1 |
| 20 | 55356 | tr\|A0A7J7AR02\|A0A7J7AR02\_9COLE | 42.81 | 2 | 2 | 2 | N | 56047 | DNA topoisomerase 2 OS=Lamprigera yunnana OX=370605 GN=FQA39\_LY13024 PE=3 SV=1 |
| 17 | 55347 | tr\|A0A444T9K0\|A0A444T9K0\_ARMVU | 38.42 | 0 | 2 | 1 | N | 677780 | Twitchin OS=Armadillidium vulgare OX=13347 GN=Avbf\_03670 PE=3 SV=1 |
| 18 | 53382 | tr\|A0A2P1JJ42\|A0A2P1JJ42\_PROCL | 33.62 | 1 | 2 | 2 | N | 167860 | Filamin-A isoform X5 (Fragment) OS=Procambarus clarkii OX=6728 GN=Flna PE=2 SV=1 |
| 15 | 53378 | tr\|A0A0N9EJJ5\|A0A0N9EJJ5\_ERIVE | 30.04 | 2 | 2 | 2 | N | 77227 | Hemocyanin OS=Eriphia verrucosa OX=483417 GN=H PE=2 SV=1 |
| 29 | 55367 | tr\|A0A1J1IN48\|A0A1J1IN48\_9DIPT | 25.85 | 0 | 1 | 1 | N | 464835 | HECT-type E3 ubiquitin transferase OS=Clunio marinus OX=568069 GN=CLUMA\_CG014451 PE=4 SV=1 |
| 29 | 55370 | tr\|A0A1E1XMV5\|A0A1E1XMV5\_9ACAR | 25.85 | 2 | 1 | 1 | N | 48988 | F-box domain-containing protein (Fragment) OS=Amblyomma sculptum OX=1581419 PE=2 SV=1 |
| 39 | 55371 | tr\|B4J4N0\|B4J4N0\_DROGR | 25.31 | 6 | 1 | 1 | N | 14634 | GH22151 OS=Drosophila grimshawi OX=7222 GN=Dgri\GH22151 PE=4 SV=1 |
| 23 | 55369 | tr\|A0A4S2KBN7\|A0A4S2KBN7\_9HYME | 25.31 | 0 | 1 | 1 | N | 276373 | Uncharacterized protein (Fragment) OS=Temnothorax longispinosus OX=300112 GN=DBV15\_11441 PE=4 SV=1 |
| 40 | 55404 | tr\|A0A2P8XAH3\|A0A2P8XAH3\_BLAGE | 23.47 | 9 | 1 | 1 | N | 9271 | Uncharacterized protein (Fragment) OS=Blattella germanica OX=6973 GN=C0J52\_27736 PE=4 SV=1 |
| 22 | 55372 | tr\|R9YY56\|R9YY56\_9EUCA | 21.53 | 6 | 1 | 1 | N | 22569 | Arginine kinase (Fragment) OS=Kiwa sp. n. East Scotia Ridge OX=1335429 GN=AK PE=3 SV=1 |
| 22 | 55373 | tr\|R9YY47\|R9YY47\_9EUCA | 21.53 | 6 | 1 | 1 | N | 22701 | Arginine kinase (Fragment) OS=Eumunida picta OX=497012 GN=AK PE=3 SV=1 |
| 22 | 55374 | tr\|G3C6J8\|G3C6J8\_9EUCA | 21.53 | 5 | 1 | 1 | N | 25125 | Arginine kinase (Fragment) OS=Eumunida funambulus OX=497008 GN=AK PE=3 SV=1 |
| 22 | 55375 | tr\|G3C6I8\|G3C6I8\_AEGAL | 21.53 | 5 | 1 | 1 | N | 25111 | Arginine kinase (Fragment) OS=Aegla alacalufi OX=172761 GN=AK PE=3 SV=1 |
| 22 | 55376 | tr\|G3C6N2\|G3C6N2\_9EUCA | 21.53 | 5 | 1 | 1 | N | 25296 | Arginine kinase (Fragment) OS=Conchoecetes artificiosus OX=516906 GN=AK PE=3 SV=1 |
| 22 | 43554 | P86699\|KARG\_CHIOP | 21.53 | 5 | 1 | 1 | N | 25324 | Arginine kinase (Fragments) OS=Chionoecetes opilio OX=41210 PE=1 SV=1 |
| 22 | 55377 | tr\|G3D680\|G3D680\_PORTR | 21.53 | 4 | 1 | 1 | N | 35589 | Arginine kinase OS=Portunus trituberculatus OX=210409 PE=3 SV=1 |
| 22 | 55378 | tr\|I1TMM2\|I1TMM2\_SCYPA | 21.53 | 3 | 1 | 1 | N | 39899 | Arginine kinase (Fragment) OS=Scylla paramamosain OX=85552 PE=2 SV=1 |
| 22 | 53723 | tr\|K7PD48\|K7PD48\_SCYPA | 21.53 | 3 | 1 | 1 | N | 40187 | Arginine kinase OS=Scylla paramamosain OX=85552 PE=2 SV=1 |
| 22 | 43542 | C9EIP1\|KARG\_SCYSE | 21.53 | 3 | 1 | 1 | N | 40189 | Arginine kinase Scy s 2 OS=Scylla serrata OX=6761 PE=1 SV=1 |
| 22 | 55379 | tr\|G3D692\|G3D692\_PORTR | 21.53 | 3 | 1 | 1 | N | 40315 | Arginine kinase OS=Portunus trituberculatus OX=210409 GN=KARG PE=3 SV=1 |
| 22 | 55380 | tr\|H6AC60\|H6AC60\_PORTR | 21.53 | 3 | 1 | 1 | N | 40291 | Arginine kinase OS=Portunus trituberculatus OX=210409 PE=3 SV=1 |
| 22 | 55381 | tr\|A0A8A8ZWC2\|A0A8A8ZWC2\_CALBE | 21.53 | 3 | 1 | 1 | N | 40243 | Arginine kinase OS=Callinectes bellicosus OX=257890 GN=AK PE=4 SV=1 |
| 22 | 55382 | tr\|G3D676\|G3D676\_PORTR | 21.53 | 3 | 1 | 1 | N | 40299 | Arginine kinase OS=Portunus trituberculatus OX=210409 PE=3 SV=1 |
| 22 | 43545 | Q9GYX1\|KARG\_PACMR | 21.53 | 3 | 1 | 1 | N | 40243 | Arginine kinase OS=Pachygrapsus marmoratus OX=135190 PE=2 SV=1 |
| 22 | 43546 | H6VGI2\|KARG\_PROCL | 21.53 | 3 | 1 | 1 | N | 40120 | Arginine kinase Pro c 2.0101 OS=Procambarus clarkii OX=6728 PE=1 SV=1 |
| 22 | 55383 | tr\|G3D677\|G3D677\_PORTR | 21.53 | 3 | 1 | 1 | N | 40287 | Arginine kinase OS=Portunus trituberculatus OX=210409 PE=3 SV=1 |
| 22 | 55384 | tr\|H6AC58\|H6AC58\_PORTR | 21.53 | 3 | 1 | 1 | N | 40373 | Arginine kinase OS=Portunus trituberculatus OX=210409 PE=3 SV=1 |
| 22 | 55385 | tr\|A0A068EFU4\|A0A068EFU4\_9EUCA | 21.53 | 3 | 1 | 1 | N | 40328 | Arginine kinase OS=Macrophthalmus japonicus OX=138195 PE=2 SV=1 |
| 22 | 55386 | tr\|G3D691\|G3D691\_PORTR | 21.53 | 3 | 1 | 1 | N | 40287 | Arginine kinase OS=Portunus trituberculatus OX=210409 PE=3 SV=1 |
| 22 | 43543 | Q9NH49\|KARG\_CALSI | 21.53 | 3 | 1 | 1 | N | 40315 | Arginine kinase OS=Callinectes sapidus OX=6763 PE=2 SV=1 |
| 22 | 43544 | Q9U9J4\|KARG\_CARMA | 21.53 | 3 | 1 | 1 | N | 40237 | Arginine kinase OS=Carcinus maenas OX=6759 PE=2 SV=1 |
| 22 | 55387 | tr\|H6UKS0\|H6UKS0\_SCYPA | 21.53 | 3 | 1 | 1 | N | 40244 | Arginine kinase OS=Scylla paramamosain OX=85552 PE=3 SV=1 |
| 22 | 43548 | Q9NH48\|KARG\_ERISI | 21.53 | 3 | 1 | 1 | N | 40339 | Arginine kinase OS=Eriocheir sinensis OX=95602 PE=2 SV=1 |
| 22 | 55388 | tr\|G3D675\|G3D675\_PORTR | 21.53 | 3 | 1 | 1 | N | 40244 | Arginine kinase OS=Portunus trituberculatus OX=210409 PE=3 SV=1 |
| 22 | 43549 | H6VGI3\|KARG0\_SCYPA | 21.53 | 3 | 1 | 1 | N | 40299 | Arginine kinase Scy p 2.0101 OS=Scylla paramamosain OX=85552 PE=1 SV=1 |
| 22 | 55405 | tr\|A0A1C6ZZL8\|A0A1C6ZZL8\_PROCL | 21.53 | 4 | 1 | 1 | N | 31849 | Arginine kinase (Fragment) OS=Procambarus clarkii OX=6728 PE=2 SV=1 |
| 22 | 55406 | tr\|A0A0C5DEY3\|A0A0C5DEY3\_CHEDE | 21.53 | 3 | 1 | 1 | N | 40205 | Arginine kinase OS=Cherax destructor OX=6723 PE=2 SV=1 |
| 22 | 55464 | tr\|D2DSQ8\|D2DSQ8\_SCYPA | 21.53 | 12 | 1 | 1 | N | 10580 | Arginine kinase (AK) (Fragment) OS=Scylla paramamosain OX=85552 PE=2 SV=1 |
| 22 | 55465 | tr\|F5A6C6\|F5A6C6\_PROCL | 21.53 | 5 | 1 | 1 | N | 27566 | Arginine kinase OS=Procambarus clarkii OX=6728 PE=2 SV=1 |
| 42 | 55470 | tr\|J9K049\|J9K049\_ACYPI | 21.41 | 0 | 1 | 1 | N | 224673 | Uncharacterized protein OS=Acyrthosiphon pisum OX=7029 PE=4 SV=2 |
| 43 | 55473 | tr\|A0A0R3NUC8\|A0A0R3NUC8\_DROPS | 20.28 | 9 | 1 | 1 | N | 12839 | Uncharacterized protein, isoform B OS=Drosophila pseudoobscura pseudoobscura OX=46245 GN=Dpse\GA28972 PE=4 SV=1 |
| 43 | 55474 | tr\|B4GX09\|B4GX09\_DROPE | 20.28 | 3 | 1 | 1 | N | 37162 | GL21085 OS=Drosophila persimilis OX=7234 GN=Dper\GL21085 PE=4 SV=1 |
| 27 | 55477 | tr\|A0A821UPU7\|A0A821UPU7\_9NEOP | 20.24 | 0 | 1 | 1 | N | 1690556 | Hypothetical protein OS=Pieris macdunnoughi OX=345717 GN=PMACD\_LOCUS10683 PE=4 SV=1 |
| 44 | 53657 | tr\|A0A023ERZ2\|A0A023ERZ2\_AEDAL | 20.21 | 3 | 1 | 1 | N | 45728 | Pyruvate kinase (Fragment) OS=Aedes albopictus OX=7160 PE=2 SV=1 |
| 44 | 53658 | tr\|A0A0P8Y097\|A0A0P8Y097\_DROAN | 20.21 | 2 | 1 | 1 | N | 55184 | Pyruvate kinase OS=Drosophila ananassae OX=7217 GN=Dana\GF18439 PE=3 SV=1 |
| 44 | 53659 | tr\|A0A6P8K875\|A0A6P8K875\_DROMA | 20.21 | 2 | 1 | 1 | N | 55073 | Pyruvate kinase OS=Drosophila mauritiana OX=7226 GN=LOC117143834 PE=3 SV=1 |
| 44 | 53660 | tr\|A0A6P4F4P4\|A0A6P4F4P4\_DRORH | 20.21 | 2 | 1 | 1 | N | 55028 | Pyruvate kinase OS=Drosophila rhopaloa OX=1041015 GN=LOC108048043 PE=3 SV=1 |
| 44 | 53661 | tr\|A0A1Y9GJY2\|A0A1Y9GJY2\_ANOAR | 20.21 | 2 | 1 | 1 | N | 55698 | Pyruvate kinase OS=Anopheles arabiensis OX=7173 PE=3 SV=1 |
| 44 | 53662 | tr\|A0A0Q9X1L7\|A0A0Q9X1L7\_DROMO | 20.21 | 2 | 1 | 1 | N | 55236 | Pyruvate kinase OS=Drosophila mojavensis OX=7230 GN=Dmoj\GI22374 PE=3 SV=1 |
| 44 | 55475 | tr\|A0A182UE55\|A0A182UE55\_9DIPT | 20.21 | 2 | 1 | 1 | N | 56051 | Pyruvate kinase OS=Anopheles melas OX=34690 PE=3 SV=2 |
| 44 | 53663 | tr\|A0A2M3ZFB9\|A0A2M3ZFB9\_9DIPT | 20.21 | 2 | 1 | 1 | N | 56104 | Pyruvate kinase (Fragment) OS=Anopheles braziliensis OX=58242 PE=3 SV=1 |
| 44 | 53664 | tr\|A0A2M3ZYS6\|A0A2M3ZYS6\_9DIPT | 20.21 | 2 | 1 | 1 | N | 56023 | Pyruvate kinase (Fragment) OS=Anopheles triannulatus OX=58253 PE=3 SV=1 |
| 44 | 53665 | tr\|A0A2M3ZYS8\|A0A2M3ZYS8\_9DIPT | 20.21 | 2 | 1 | 1 | N | 56022 | Pyruvate kinase (Fragment) OS=Anopheles triannulatus OX=58253 PE=3 SV=1 |
| 44 | 53666 | tr\|A0A1Y9GJK0\|A0A1Y9GJK0\_ANOAR | 20.21 | 2 | 1 | 1 | N | 56271 | Pyruvate kinase OS=Anopheles arabiensis OX=7173 PE=3 SV=1 |
| 44 | 53667 | tr\|A0A6A0H7S7\|A0A6A0H7S7\_HYAAZ | 20.21 | 2 | 1 | 1 | N | 56703 | Pyruvate kinase OS=Hyalella azteca OX=294128 GN=HAZT\_HAZT006469 PE=3 SV=1 |
| 44 | 53668 | tr\|A0A1E1X8N4\|A0A1E1X8N4\_9ACAR | 20.21 | 2 | 1 | 1 | N | 57708 | Pyruvate kinase OS=Amblyomma aureolatum OX=187763 PE=2 SV=1 |
| 44 | 53669 | tr\|A0A6P4F9I4\|A0A6P4F9I4\_DRORH | 20.21 | 2 | 1 | 1 | N | 57050 | Pyruvate kinase OS=Drosophila rhopaloa OX=1041015 GN=LOC108048043 PE=3 SV=1 |
| 44 | 53670 | tr\|A0A182VPN6\|A0A182VPN6\_9DIPT | 20.21 | 2 | 1 | 1 | N | 57977 | Pyruvate kinase OS=Anopheles minimus OX=112268 PE=3 SV=1 |
| 44 | 53671 | tr\|A0A182MNQ6\|A0A182MNQ6\_9DIPT | 20.21 | 2 | 1 | 1 | N | 57968 | Pyruvate kinase OS=Anopheles culicifacies OX=139723 PE=3 SV=1 |
| 44 | 53672 | tr\|A0A6J2T5C5\|A0A6J2T5C5\_DROLE | 20.21 | 2 | 1 | 1 | N | 57328 | Pyruvate kinase OS=Drosophila lebanonensis OX=7225 GN=LOC115620839 PE=3 SV=1 |
| 44 | 53673 | tr\|A0A1W4W378\|A0A1W4W378\_DROFC | 20.21 | 2 | 1 | 1 | N | 57395 | Pyruvate kinase OS=Drosophila ficusphila OX=30025 GN=LOC108099555 PE=3 SV=1 |
| 44 | 53674 | O62619\|KPYK\_DROME | 20.21 | 2 | 1 | 1 | N | 57440 | Pyruvate kinase OS=Drosophila melanogaster OX=7227 GN=PyK PE=2 SV=2 |
| 44 | 53675 | tr\|B4HE69\|B4HE69\_DROSE | 20.21 | 2 | 1 | 1 | N | 57454 | Pyruvate kinase OS=Drosophila sechellia OX=7238 GN=Dsec\GM26420 PE=3 SV=1 |
| 44 | 53676 | tr\|A0A6P4F0Q9\|A0A6P4F0Q9\_DRORH | 20.21 | 2 | 1 | 1 | N | 57379 | Pyruvate kinase OS=Drosophila rhopaloa OX=1041015 GN=LOC108048043 PE=3 SV=1 |
| 44 | 53677 | tr\|B3P7K4\|B3P7K4\_DROER | 20.21 | 2 | 1 | 1 | N | 57539 | Pyruvate kinase OS=Drosophila erecta OX=7220 GN=Dere\GG11123 PE=3 SV=1 |
| 44 | 53678 | tr\|B3M213\|B3M213\_DROAN | 20.21 | 2 | 1 | 1 | N | 57565 | Pyruvate kinase OS=Drosophila ananassae OX=7217 GN=Dana\GF18439 PE=3 SV=2 |
| 44 | 53679 | tr\|A0A6P8KMB4\|A0A6P8KMB4\_DROMA | 20.21 | 2 | 1 | 1 | N | 57454 | Pyruvate kinase OS=Drosophila mauritiana OX=7226 GN=LOC117143834 PE=3 SV=1 |
| 44 | 53680 | tr\|B4K5E3\|B4K5E3\_DROMO | 20.21 | 2 | 1 | 1 | N | 57618 | Pyruvate kinase OS=Drosophila mojavensis OX=7230 GN=Dmoj\GI22374 PE=3 SV=1 |
| 44 | 53681 | tr\|A0A484BWG4\|A0A484BWG4\_DRONA | 20.21 | 2 | 1 | 1 | N | 57563 | Pyruvate kinase OS=Drosophila navojoa OX=7232 GN=AWZ03\_001260 PE=3 SV=1 |
| 44 | 53682 | tr\|A0A131XQM7\|A0A131XQM7\_9ACAR | 20.21 | 2 | 1 | 1 | N | 59290 | Pyruvate kinase OS=Hyalomma excavatum OX=257692 PE=2 SV=1 |
| 44 | 53683 | tr\|A0A0P9ARZ1\|A0A0P9ARZ1\_DROAN | 20.21 | 2 | 1 | 1 | N | 59439 | Pyruvate kinase OS=Drosophila ananassae OX=7217 GN=Dana\GF18439 PE=3 SV=1 |
| 44 | 53684 | tr\|G3MLC1\|G3MLC1\_AMBMU | 20.21 | 2 | 1 | 1 | N | 61369 | Pyruvate kinase OS=Amblyomma maculatum OX=34609 PE=2 SV=1 |
| 44 | 53685 | tr\|A0A023EVC5\|A0A023EVC5\_AEDAL | 20.21 | 2 | 1 | 1 | N | 63958 | Pyruvate kinase (Fragment) OS=Aedes albopictus OX=7160 PE=2 SV=1 |
| 44 | 53687 | tr\|A0A5N5T493\|A0A5N5T493\_9CRUS | 20.21 | 2 | 1 | 1 | N | 68944 | Pyruvate kinase OS=Armadillidium nasatum OX=96803 GN=PyK PE=3 SV=1 |
| total 144 proteins |  |  |  |  |  |  |  |  |  |
| --- | --- | --- | --- | --- | --- | --- | --- | --- | --- |

  

tr|A0A3R7M007|A0A3R7M007\_PENVA

back to list

  

| Protein Coverage
| Supporting Peptides
|

Protein Coverage:

Supporting Peptides:

| Peptide | Uniq | -10lgP | Mass | ppm | m/z | z | RT | Scan | #Spec | Start | End | PTM |
| --- | --- | --- | --- | --- | --- | --- | --- | --- | --- | --- | --- | --- |
| T.KEEYDESGPGIVHR.K | Y | 49.90 | 1614.7638 | -1.3 | 539.2612 | 3 | 10.08 | 1349 | 2 | 356 | 369 |  |
| K.SYELPDGQVITI.G | Y | 39.95 | 1333.6765 | -0.3 | 667.8453 | 2 | 14.58 | 2163 | 1 | 236 | 247 |  |
| T.NWDDMEKIWHH.S | Y | 36.38 | 1509.6459 | 0.3 | 755.8304 | 2 | 12.78 | 1846 | 1 | 75 | 85 |  |
| G.FAGDDAPRA.V | Y | 34.08 | 918.4195 | -1.0 | 460.2166 | 2 | 10.01 | 1336 | 1 | 18 | 26 |  |
| E.EYDESGPGIVH.R | Y | 32.14 | 1201.5251 | -0.1 | 601.7698 | 2 | 10.85 | 1492 | 1 | 358 | 368 |  |
| K.IIAPPERKYS.V | Y | 26.54 | 1172.6553 | -0.6 | 587.3345 | 2 | 10.19 | 1367 | 1 | 326 | 335 |  |
| E.KSYELPDGQVITI.G | Y | 23.84 | 1461.7715 | -0.3 | 731.8928 | 2 | 13.76 | 2020 | 1 | 235 | 247 |  |
| L.DTGDGVTH.T | Y | 22.68 | 800.3301 | 0.0 | 401.1723 | 2 | 8.83 | 1133 | 2 | 151 | 158 |  |
| E.EYDESGPGIVHR.K | Y | 22.68 | 1357.6262 | -0.8 | 679.8198 | 2 | 10.41 | 1408 | 1 | 358 | 369 |  |
| K.AGFAGDDAPR.A | Y | 19.76 | 975.4410 | -1.8 | 488.7269 | 2 | 10.25 | 1378 | 1 | 16 | 25 |  |
| T.MYPGIADRM.Q | Y | 19.04 | 1052.4783 | -0.3 | 527.2463 | 2 | 11.98 | 1702 | 1 | 302 | 310 |  |
| L.LTEAPLNPK.A | Y | 18.48 | 981.5494 | -1.0 | 491.7815 | 2 | 10.51 | 1426 | 1 | 102 | 110 |  |
| E.KSYELPDGQVIT.I | Y | 17.94 | 1348.6874 | -0.7 | 675.3505 | 2 | 12.15 | 1733 | 1 | 235 | 246 |  |
| L.TEAPLNPK.A | Y | 17.21 | 868.4654 | -0.5 | 435.2397 | 2 | 9.70 | 1282 | 2 | 103 | 110 |  |
| E.SGPGIVH.R | Y | 16.57 | 665.3497 | -0.5 | 333.6819 | 2 | 9.75 | 1291 | 1 | 362 | 368 |  |
| K.SYELPDGQVI.T | Y | 16.19 | 1119.5448 | -0.3 | 560.7795 | 2 | 13.65 | 2001 | 1 | 236 | 245 |  |
| F.AGDDAPR.A | Y | 15.34 | 700.3140 | -5.0 | 351.1625 | 2 | 15.31 | 2292 | 2 | 19 | 25 |  |
| total 17 peptides |  |  |  |  |  |  |  |  |  |  |  |  |
| --- | --- | --- | --- | --- | --- | --- | --- | --- | --- | --- | --- | --- |

tr|H9B3Y9|H9B3Y9\_SCYPA

back to list

  

| Protein Coverage
| Supporting Peptides
|

Protein Coverage:

Supporting Peptides:

| Peptide | Uniq | -10lgP | Mass | ppm | m/z | z | RT | Scan | #Spec | Start | End | PTM |
| --- | --- | --- | --- | --- | --- | --- | --- | --- | --- | --- | --- | --- |
| T.KEEYDESGPGIVHR.K | Y | 49.90 | 1614.7638 | -1.3 | 539.2612 | 3 | 10.08 | 1349 | 2 | 360 | 373 |  |
| K.SYELPDGQVITI.G | Y | 39.95 | 1333.6765 | -0.3 | 667.8453 | 2 | 14.58 | 2163 | 1 | 240 | 251 |  |
| T.NWDDMEKIWHH.S | Y | 36.38 | 1509.6459 | 0.3 | 755.8304 | 2 | 12.78 | 1846 | 1 | 79 | 89 |  |
| G.FAGDDAPRA.V | Y | 34.08 | 918.4195 | -1.0 | 460.2166 | 2 | 10.01 | 1336 | 1 | 22 | 30 |  |
| E.EYDESGPGIVH.R | Y | 32.14 | 1201.5251 | -0.1 | 601.7698 | 2 | 10.85 | 1492 | 1 | 362 | 372 |  |
| K.IIAPPERKYS.V | Y | 26.54 | 1172.6553 | -0.6 | 587.3345 | 2 | 10.19 | 1367 | 1 | 330 | 339 |  |
| E.KSYELPDGQVITI.G | Y | 23.84 | 1461.7715 | -0.3 | 731.8928 | 2 | 13.76 | 2020 | 1 | 239 | 251 |  |
| L.DTGDGVTH.T | Y | 22.68 | 800.3301 | 0.0 | 401.1723 | 2 | 8.83 | 1133 | 2 | 155 | 162 |  |
| E.EYDESGPGIVHR.K | Y | 22.68 | 1357.6262 | -0.8 | 679.8198 | 2 | 10.41 | 1408 | 1 | 362 | 373 |  |
| K.AGFAGDDAPR.A | Y | 19.76 | 975.4410 | -1.8 | 488.7269 | 2 | 10.25 | 1378 | 1 | 20 | 29 |  |
| T.MYPGIADRM.Q | Y | 19.04 | 1052.4783 | -0.3 | 527.2463 | 2 | 11.98 | 1702 | 1 | 306 | 314 |  |
| L.LTEAPLNPK.A | Y | 18.48 | 981.5494 | -1.0 | 491.7815 | 2 | 10.51 | 1426 | 1 | 106 | 114 |  |
| E.KSYELPDGQVIT.I | Y | 17.94 | 1348.6874 | -0.7 | 675.3505 | 2 | 12.15 | 1733 | 1 | 239 | 250 |  |
| L.TEAPLNPK.A | Y | 17.21 | 868.4654 | -0.5 | 435.2397 | 2 | 9.70 | 1282 | 2 | 107 | 114 |  |
| E.SGPGIVH.R | Y | 16.57 | 665.3497 | -0.5 | 333.6819 | 2 | 9.75 | 1291 | 1 | 366 | 372 |  |
| K.SYELPDGQVI.T | Y | 16.19 | 1119.5448 | -0.3 | 560.7795 | 2 | 13.65 | 2001 | 1 | 240 | 249 |  |
| F.AGDDAPR.A | Y | 15.34 | 700.3140 | -5.0 | 351.1625 | 2 | 15.31 | 2292 | 2 | 23 | 29 |  |
| total 17 peptides |  |  |  |  |  |  |  |  |  |  |  |  |
| --- | --- | --- | --- | --- | --- | --- | --- | --- | --- | --- | --- | --- |

tr|A0A6A0H005|A0A6A0H005\_HYAAZ

back to list

  

| Protein Coverage
| Supporting Peptides
|

Protein Coverage:

Supporting Peptides:

| Peptide | Uniq | -10lgP | Mass | ppm | m/z | z | RT | Scan | #Spec | Start | End | PTM |
| --- | --- | --- | --- | --- | --- | --- | --- | --- | --- | --- | --- | --- |
| T.KEEYDESGPGIVHR.K | Y | 49.90 | 1614.7638 | -1.3 | 539.2612 | 3 | 10.08 | 1349 | 2 | 360 | 373 |  |
| K.SYELPDGQVITI.G | Y | 39.95 | 1333.6765 | -0.3 | 667.8453 | 2 | 14.58 | 2163 | 1 | 240 | 251 |  |
| T.NWDDMEKIWHH.T | Y | 36.38 | 1509.6459 | 0.3 | 755.8304 | 2 | 12.78 | 1846 | 1 | 79 | 89 |  |
| G.FAGDDAPRA.V | Y | 34.08 | 918.4195 | -1.0 | 460.2166 | 2 | 10.01 | 1336 | 1 | 22 | 30 |  |
| E.EYDESGPGIVH.R | Y | 32.14 | 1201.5251 | -0.1 | 601.7698 | 2 | 10.85 | 1492 | 1 | 362 | 372 |  |
| K.IIAPPERKYS.V | Y | 26.54 | 1172.6553 | -0.6 | 587.3345 | 2 | 10.19 | 1367 | 1 | 330 | 339 |  |
| E.KSYELPDGQVITI.G | Y | 23.84 | 1461.7715 | -0.3 | 731.8928 | 2 | 13.76 | 2020 | 1 | 239 | 251 |  |
| L.DTGDGVTH.T | Y | 22.68 | 800.3301 | 0.0 | 401.1723 | 2 | 8.83 | 1133 | 2 | 155 | 162 |  |
| E.EYDESGPGIVHR.K | Y | 22.68 | 1357.6262 | -0.8 | 679.8198 | 2 | 10.41 | 1408 | 1 | 362 | 373 |  |
| K.AGFAGDDAPR.A | Y | 19.76 | 975.4410 | -1.8 | 488.7269 | 2 | 10.25 | 1378 | 1 | 20 | 29 |  |
| S.MYPGIADRM.Q | Y | 19.04 | 1052.4783 | -0.3 | 527.2463 | 2 | 11.98 | 1702 | 1 | 306 | 314 |  |
| L.LTEAPLNPK.A | Y | 18.48 | 981.5494 | -1.0 | 491.7815 | 2 | 10.51 | 1426 | 1 | 106 | 114 |  |
| E.KSYELPDGQVIT.I | Y | 17.94 | 1348.6874 | -0.7 | 675.3505 | 2 | 12.15 | 1733 | 1 | 239 | 250 |  |
| L.TEAPLNPK.A | Y | 17.21 | 868.4654 | -0.5 | 435.2397 | 2 | 9.70 | 1282 | 2 | 107 | 114 |  |
| E.SGPGIVH.R | Y | 16.57 | 665.3497 | -0.5 | 333.6819 | 2 | 9.75 | 1291 | 1 | 366 | 372 |  |
| K.SYELPDGQVI.T | Y | 16.19 | 1119.5448 | -0.3 | 560.7795 | 2 | 13.65 | 2001 | 1 | 240 | 249 |  |
| F.AGDDAPR.A | Y | 15.34 | 700.3140 | -5.0 | 351.1625 | 2 | 15.31 | 2292 | 2 | 23 | 29 |  |
| total 17 peptides |  |  |  |  |  |  |  |  |  |  |  |  |
| --- | --- | --- | --- | --- | --- | --- | --- | --- | --- | --- | --- | --- |

tr|A0A482LUC2|A0A482LUC2\_CANBE

back to list

  

| Protein Coverage
| Supporting Peptides
|

Protein Coverage:

Supporting Peptides:

| Peptide | Uniq | -10lgP | Mass | ppm | m/z | z | RT | Scan | #Spec | Start | End | PTM |
| --- | --- | --- | --- | --- | --- | --- | --- | --- | --- | --- | --- | --- |
| T.KEEYDESGPGIVHR.K | Y | 49.90 | 1614.7638 | -1.3 | 539.2612 | 3 | 10.08 | 1349 | 2 | 360 | 373 |  |
| K.SYELPDGQVITI.G | Y | 39.95 | 1333.6765 | -0.3 | 667.8453 | 2 | 14.58 | 2163 | 1 | 240 | 251 |  |
| T.NWDDMEKIWHH.S | Y | 36.38 | 1509.6459 | 0.3 | 755.8304 | 2 | 12.78 | 1846 | 1 | 79 | 89 |  |
| G.FAGDDAPRA.V | Y | 34.08 | 918.4195 | -1.0 | 460.2166 | 2 | 10.01 | 1336 | 1 | 22 | 30 |  |
| E.EYDESGPGIVH.R | Y | 32.14 | 1201.5251 | -0.1 | 601.7698 | 2 | 10.85 | 1492 | 1 | 362 | 372 |  |
| K.IIAPPERKYS.V | Y | 26.54 | 1172.6553 | -0.6 | 587.3345 | 2 | 10.19 | 1367 | 1 | 330 | 339 |  |
| E.KSYELPDGQVITI.G | Y | 23.84 | 1461.7715 | -0.3 | 731.8928 | 2 | 13.76 | 2020 | 1 | 239 | 251 |  |
| L.DTGDGVTH.T | Y | 22.68 | 800.3301 | 0.0 | 401.1723 | 2 | 8.83 | 1133 | 2 | 155 | 162 |  |
| E.EYDESGPGIVHR.K | Y | 22.68 | 1357.6262 | -0.8 | 679.8198 | 2 | 10.41 | 1408 | 1 | 362 | 373 |  |
| K.AGFAGDDAPR.A | Y | 19.76 | 975.4410 | -1.8 | 488.7269 | 2 | 10.25 | 1378 | 1 | 20 | 29 |  |
| T.MYPGIADRM.Q | Y | 19.04 | 1052.4783 | -0.3 | 527.2463 | 2 | 11.98 | 1702 | 1 | 306 | 314 |  |
| L.LTEAPLNPK.A | Y | 18.48 | 981.5494 | -1.0 | 491.7815 | 2 | 10.51 | 1426 | 1 | 106 | 114 |  |
| E.KSYELPDGQVIT.I | Y | 17.94 | 1348.6874 | -0.7 | 675.3505 | 2 | 12.15 | 1733 | 1 | 239 | 250 |  |
| L.TEAPLNPK.A | Y | 17.21 | 868.4654 | -0.5 | 435.2397 | 2 | 9.70 | 1282 | 2 | 107 | 114 |  |
| E.SGPGIVH.R | Y | 16.57 | 665.3497 | -0.5 | 333.6819 | 2 | 9.75 | 1291 | 1 | 366 | 372 |  |
| K.SYELPDGQVI.T | Y | 16.19 | 1119.5448 | -0.3 | 560.7795 | 2 | 13.65 | 2001 | 1 | 240 | 249 |  |
| F.AGDDAPR.A | Y | 15.34 | 700.3140 | -5.0 | 351.1625 | 2 | 15.31 | 2292 | 2 | 23 | 29 |  |
| total 17 peptides |  |  |  |  |  |  |  |  |  |  |  |  |
| --- | --- | --- | --- | --- | --- | --- | --- | --- | --- | --- | --- | --- |

tr|A0A2H4V3D6|A0A2H4V3D6\_PENVA

back to list

  

| Protein Coverage
| Supporting Peptides
|

Protein Coverage:

Supporting Peptides:

| Peptide | Uniq | -10lgP | Mass | ppm | m/z | z | RT | Scan | #Spec | Start | End | PTM |
| --- | --- | --- | --- | --- | --- | --- | --- | --- | --- | --- | --- | --- |
| T.KEEYDESGPGIVHR.K | Y | 49.90 | 1614.7638 | -1.3 | 539.2612 | 3 | 10.08 | 1349 | 2 | 360 | 373 |  |
| K.SYELPDGQVITI.G | Y | 39.95 | 1333.6765 | -0.3 | 667.8453 | 2 | 14.58 | 2163 | 1 | 240 | 251 |  |
| T.NWDDMEKIWHH.S | Y | 36.38 | 1509.6459 | 0.3 | 755.8304 | 2 | 12.78 | 1846 | 1 | 79 | 89 |  |
| G.FAGDDAPRA.V | Y | 34.08 | 918.4195 | -1.0 | 460.2166 | 2 | 10.01 | 1336 | 1 | 22 | 30 |  |
| E.EYDESGPGIVH.R | Y | 32.14 | 1201.5251 | -0.1 | 601.7698 | 2 | 10.85 | 1492 | 1 | 362 | 372 |  |
| K.IIAPPERKYS.V | Y | 26.54 | 1172.6553 | -0.6 | 587.3345 | 2 | 10.19 | 1367 | 1 | 330 | 339 |  |
| E.KSYELPDGQVITI.G | Y | 23.84 | 1461.7715 | -0.3 | 731.8928 | 2 | 13.76 | 2020 | 1 | 239 | 251 |  |
| L.DTGDGVTH.T | Y | 22.68 | 800.3301 | 0.0 | 401.1723 | 2 | 8.83 | 1133 | 2 | 155 | 162 |  |
| E.EYDESGPGIVHR.K | Y | 22.68 | 1357.6262 | -0.8 | 679.8198 | 2 | 10.41 | 1408 | 1 | 362 | 373 |  |
| K.AGFAGDDAPR.A | Y | 19.76 | 975.4410 | -1.8 | 488.7269 | 2 | 10.25 | 1378 | 1 | 20 | 29 |  |
| T.MYPGIADRM.Q | Y | 19.04 | 1052.4783 | -0.3 | 527.2463 | 2 | 11.98 | 1702 | 1 | 306 | 314 |  |
| L.LTEAPLNPK.A | Y | 18.48 | 981.5494 | -1.0 | 491.7815 | 2 | 10.51 | 1426 | 1 | 106 | 114 |  |
| E.KSYELPDGQVIT.I | Y | 17.94 | 1348.6874 | -0.7 | 675.3505 | 2 | 12.15 | 1733 | 1 | 239 | 250 |  |
| L.TEAPLNPK.A | Y | 17.21 | 868.4654 | -0.5 | 435.2397 | 2 | 9.70 | 1282 | 2 | 107 | 114 |  |
| E.SGPGIVH.R | Y | 16.57 | 665.3497 | -0.5 | 333.6819 | 2 | 9.75 | 1291 | 1 | 366 | 372 |  |
| K.SYELPDGQVI.T | Y | 16.19 | 1119.5448 | -0.3 | 560.7795 | 2 | 13.65 | 2001 | 1 | 240 | 249 |  |
| F.AGDDAPR.A | Y | 15.34 | 700.3140 | -5.0 | 351.1625 | 2 | 15.31 | 2292 | 2 | 23 | 29 |  |
| total 17 peptides |  |  |  |  |  |  |  |  |  |  |  |  |
| --- | --- | --- | --- | --- | --- | --- | --- | --- | --- | --- | --- | --- |

tr|A0A5B7IZE3|A0A5B7IZE3\_PORTR

back to list

  

| Protein Coverage
| Supporting Peptides
|

Protein Coverage:

Supporting Peptides:

| Peptide | Uniq | -10lgP | Mass | ppm | m/z | z | RT | Scan | #Spec | Start | End | PTM |
| --- | --- | --- | --- | --- | --- | --- | --- | --- | --- | --- | --- | --- |
| K.SPGVIDAGLVMH.Q | N | 44.78 | 1194.6067 | -0.9 | 598.3101 | 2 | 12.56 | 1806 | 1 | 155 | 166 |  |
| W.LEKNKDPLNDTLVDLL.K | Y | 33.09 | 1838.9989 | 0.3 | 920.5070 | 2 | 14.28 | 2111 | 1 | 68 | 83 |  |
| L.SILEEESMFPK.A | N | 26.45 | 1308.6271 | -0.7 | 655.3204 | 2 | 12.91 | 1869 | 1 | 5 | 15 |  |
| K.NKDPLNDTL.V | N | 20.74 | 1028.5138 | -0.9 | 515.2637 | 2 | 11.46 | 1603 | 1 | 71 | 79 |  |
| N.ETKSPGVIDAGLVM.H | N | 16.36 | 1415.7330 | -0.4 | 708.8735 | 2 | 12.98 | 1882 | 1 | 152 | 165 |  |
| total 5 peptides |  |  |  |  |  |  |  |  |  |  |  |  |
| --- | --- | --- | --- | --- | --- | --- | --- | --- | --- | --- | --- | --- |

tr|F8WR03|F8WR03\_PENJP

back to list

  

| Protein Coverage
| Supporting Peptides
|

Protein Coverage:

Supporting Peptides:

| Peptide | Uniq | -10lgP | Mass | ppm | m/z | z | RT | Scan | #Spec | Start | End | PTM |
| --- | --- | --- | --- | --- | --- | --- | --- | --- | --- | --- | --- | --- |
| K.VTVPSIDDKEDMQF.T | N | 40.27 | 1622.7498 | -0.4 | 812.3818 | 2 | 12.86 | 1860 | 1 | 318 | 331 |  |
| K.VTVPSIDDKEDMQ.F | N | 31.89 | 1475.6814 | -0.1 | 738.8479 | 2 | 11.58 | 1627 | 1 | 318 | 330 |  |
| L.SILEEESMFPK.A | N | 26.45 | 1308.6271 | -0.7 | 655.3204 | 2 | 12.91 | 1869 | 1 | 532 | 542 |  |
| L.ITGESGAGK.T | N | 23.91 | 818.4134 | -0.2 | 410.2139 | 2 | 8.67 | 1104 | 1 | 180 | 188 |  |
| R.NEVPPHLF.A | N | 16.25 | 951.4814 | -0.3 | 476.7478 | 2 | 13.07 | 1899 | 1 | 152 | 159 |  |
| total 5 peptides |  |  |  |  |  |  |  |  |  |  |  |  |
| --- | --- | --- | --- | --- | --- | --- | --- | --- | --- | --- | --- | --- |

tr|K4Q111|K4Q111\_PENVA

back to list

  

| Protein Coverage
| Supporting Peptides
|

Protein Coverage:

Supporting Peptides:

| Peptide | Uniq | -10lgP | Mass | ppm | m/z | z | RT | Scan | #Spec | Start | End | PTM |
| --- | --- | --- | --- | --- | --- | --- | --- | --- | --- | --- | --- | --- |
| K.VTVPSIDDKEDMQF.T | N | 40.27 | 1622.7498 | -0.4 | 812.3818 | 2 | 12.86 | 1860 | 1 | 318 | 331 |  |
| K.VTVPSIDDKEDMQ.F | N | 31.89 | 1475.6814 | -0.1 | 738.8479 | 2 | 11.58 | 1627 | 1 | 318 | 330 |  |
| L.SILEEESMFPK.A | N | 26.45 | 1308.6271 | -0.7 | 655.3204 | 2 | 12.91 | 1869 | 1 | 532 | 542 |  |
| L.ITGESGAGK.T | N | 23.91 | 818.4134 | -0.2 | 410.2139 | 2 | 8.67 | 1104 | 1 | 180 | 188 |  |
| R.NEVPPHLF.A | N | 16.25 | 951.4814 | -0.3 | 476.7478 | 2 | 13.07 | 1899 | 1 | 152 | 159 |  |
| total 5 peptides |  |  |  |  |  |  |  |  |  |  |  |  |
| --- | --- | --- | --- | --- | --- | --- | --- | --- | --- | --- | --- | --- |

tr|K4Q4N8|K4Q4N8\_PENMO

back to list

  

| Protein Coverage
| Supporting Peptides
|

Protein Coverage:

Supporting Peptides:

| Peptide | Uniq | -10lgP | Mass | ppm | m/z | z | RT | Scan | #Spec | Start | End | PTM |
| --- | --- | --- | --- | --- | --- | --- | --- | --- | --- | --- | --- | --- |
| K.VTVPSIDDKEDMQF.T | N | 40.27 | 1622.7498 | -0.4 | 812.3818 | 2 | 12.86 | 1860 | 1 | 318 | 331 |  |
| K.VTVPSIDDKEDMQ.F | N | 31.89 | 1475.6814 | -0.1 | 738.8479 | 2 | 11.58 | 1627 | 1 | 318 | 330 |  |
| L.SILEEESMFPK.A | N | 26.45 | 1308.6271 | -0.7 | 655.3204 | 2 | 12.91 | 1869 | 1 | 532 | 542 |  |
| L.ITGESGAGK.T | N | 23.91 | 818.4134 | -0.2 | 410.2139 | 2 | 8.67 | 1104 | 1 | 180 | 188 |  |
| R.NEVPPHLF.A | N | 16.25 | 951.4814 | -0.3 | 476.7478 | 2 | 13.07 | 1899 | 1 | 152 | 159 |  |
| total 5 peptides |  |  |  |  |  |  |  |  |  |  |  |  |
| --- | --- | --- | --- | --- | --- | --- | --- | --- | --- | --- | --- | --- |

tr|A0A4Y7MKW9|A0A4Y7MKW9\_9CRUS

back to list

  

| Protein Coverage
| Supporting Peptides
|

Protein Coverage:

Supporting Peptides:

| Peptide | Uniq | -10lgP | Mass | ppm | m/z | z | RT | Scan | #Spec | Start | End | PTM |
| --- | --- | --- | --- | --- | --- | --- | --- | --- | --- | --- | --- | --- |
| M.TGDGVNDAPALK.K | N | 40.51 | 1156.5724 | -0.4 | 579.2932 | 2 | 10.50 | 1423 | 1 | 594 | 605 |  |
| V.SVGDKIPAD.I | N | 30.16 | 900.4552 | -0.6 | 451.2346 | 2 | 10.20 | 1370 | 1 | 46 | 54 |  |
| L.GFNPPDLDIM.N | N | 23.83 | 1117.5114 | -0.4 | 559.7628 | 2 | 14.97 | 2231 | 1 | 701 | 710 |  |
| E.VSVGDKIPAD.I | N | 18.74 | 999.5236 | -0.1 | 500.7690 | 2 | 10.76 | 1472 | 1 | 45 | 54 |  |
| V.KISLPVILL.D | N | 16.43 | 994.6790 | -0.3 | 498.3466 | 2 | 15.13 | 2259 | 1 | 865 | 873 |  |
| S.VGDKIPADI.R | N | 16.36 | 926.5073 | -0.4 | 464.2607 | 2 | 11.57 | 1624 | 1 | 47 | 55 |  |
| total 6 peptides |  |  |  |  |  |  |  |  |  |  |  |  |
| --- | --- | --- | --- | --- | --- | --- | --- | --- | --- | --- | --- | --- |

tr|A0A0P5KSW7|A0A0P5KSW7\_9CRUS

back to list

  

| Protein Coverage
| Supporting Peptides
|

Protein Coverage:

Supporting Peptides:

| Peptide | Uniq | -10lgP | Mass | ppm | m/z | z | RT | Scan | #Spec | Start | End | PTM |
| --- | --- | --- | --- | --- | --- | --- | --- | --- | --- | --- | --- | --- |
| M.TGDGVNDAPALK.K | N | 40.51 | 1156.5724 | -0.4 | 579.2932 | 2 | 10.50 | 1423 | 1 | 705 | 716 |  |
| V.SVGDKIPAD.I | N | 30.16 | 900.4552 | -0.6 | 451.2346 | 2 | 10.20 | 1370 | 1 | 157 | 165 |  |
| L.GFNPPDLDIM.N | N | 23.83 | 1117.5114 | -0.4 | 559.7628 | 2 | 14.97 | 2231 | 1 | 812 | 821 |  |
| E.VSVGDKIPAD.I | N | 18.74 | 999.5236 | -0.1 | 500.7690 | 2 | 10.76 | 1472 | 1 | 156 | 165 |  |
| V.KISLPVILL.D | N | 16.43 | 994.6790 | -0.3 | 498.3466 | 2 | 15.13 | 2259 | 1 | 976 | 984 |  |
| S.VGDKIPADI.R | N | 16.36 | 926.5073 | -0.4 | 464.2607 | 2 | 11.57 | 1624 | 1 | 158 | 166 |  |
| total 6 peptides |  |  |  |  |  |  |  |  |  |  |  |  |
| --- | --- | --- | --- | --- | --- | --- | --- | --- | --- | --- | --- | --- |

tr|A0A4Y7MXF9|A0A4Y7MXF9\_9CRUS

back to list

  

| Protein Coverage
| Supporting Peptides
|

Protein Coverage:

Supporting Peptides:

| Peptide | Uniq | -10lgP | Mass | ppm | m/z | z | RT | Scan | #Spec | Start | End | PTM |
| --- | --- | --- | --- | --- | --- | --- | --- | --- | --- | --- | --- | --- |
| M.TGDGVNDAPALK.K | N | 40.51 | 1156.5724 | -0.4 | 579.2932 | 2 | 10.50 | 1423 | 1 | 705 | 716 |  |
| V.SVGDKIPAD.I | N | 30.16 | 900.4552 | -0.6 | 451.2346 | 2 | 10.20 | 1370 | 1 | 157 | 165 |  |
| L.GFNPPDLDIM.N | N | 23.83 | 1117.5114 | -0.4 | 559.7628 | 2 | 14.97 | 2231 | 1 | 812 | 821 |  |
| E.VSVGDKIPAD.I | N | 18.74 | 999.5236 | -0.1 | 500.7690 | 2 | 10.76 | 1472 | 1 | 156 | 165 |  |
| I.KISLPVILL.D | N | 16.43 | 994.6790 | -0.3 | 498.3466 | 2 | 15.13 | 2259 | 1 | 976 | 984 |  |
| S.VGDKIPADI.R | N | 16.36 | 926.5073 | -0.4 | 464.2607 | 2 | 11.57 | 1624 | 1 | 158 | 166 |  |
| total 6 peptides |  |  |  |  |  |  |  |  |  |  |  |  |
| --- | --- | --- | --- | --- | --- | --- | --- | --- | --- | --- | --- | --- |

tr|A0A0P4ZGF4|A0A0P4ZGF4\_9CRUS

back to list

  

| Protein Coverage
| Supporting Peptides
|

Protein Coverage:

Supporting Peptides:

| Peptide | Uniq | -10lgP | Mass | ppm | m/z | z | RT | Scan | #Spec | Start | End | PTM |
| --- | --- | --- | --- | --- | --- | --- | --- | --- | --- | --- | --- | --- |
| M.TGDGVNDAPALK.K | N | 40.51 | 1156.5724 | -0.4 | 579.2932 | 2 | 10.50 | 1423 | 1 | 705 | 716 |  |
| V.SVGDKIPAD.I | N | 30.16 | 900.4552 | -0.6 | 451.2346 | 2 | 10.20 | 1370 | 1 | 157 | 165 |  |
| L.GFNPPDLDIM.N | N | 23.83 | 1117.5114 | -0.4 | 559.7628 | 2 | 14.97 | 2231 | 1 | 812 | 821 |  |
| E.VSVGDKIPAD.I | N | 18.74 | 999.5236 | -0.1 | 500.7690 | 2 | 10.76 | 1472 | 1 | 156 | 165 |  |
| V.KISLPVILL.D | N | 16.43 | 994.6790 | -0.3 | 498.3466 | 2 | 15.13 | 2259 | 1 | 976 | 984 |  |
| S.VGDKIPADI.R | N | 16.36 | 926.5073 | -0.4 | 464.2607 | 2 | 11.57 | 1624 | 1 | 158 | 166 |  |
| total 6 peptides |  |  |  |  |  |  |  |  |  |  |  |  |
| --- | --- | --- | --- | --- | --- | --- | --- | --- | --- | --- | --- | --- |

tr|A0A4Y7MVM2|A0A4Y7MVM2\_DAPPU

back to list

  

| Protein Coverage
| Supporting Peptides
|

Protein Coverage:

Supporting Peptides:

| Peptide | Uniq | -10lgP | Mass | ppm | m/z | z | RT | Scan | #Spec | Start | End | PTM |
| --- | --- | --- | --- | --- | --- | --- | --- | --- | --- | --- | --- | --- |
| M.TGDGVNDAPALK.K | N | 40.51 | 1156.5724 | -0.4 | 579.2932 | 2 | 10.50 | 1423 | 1 | 705 | 716 |  |
| V.SVGDKIPAD.I | N | 30.16 | 900.4552 | -0.6 | 451.2346 | 2 | 10.20 | 1370 | 1 | 157 | 165 |  |
| L.GFNPPDLDIM.N | N | 23.83 | 1117.5114 | -0.4 | 559.7628 | 2 | 14.97 | 2231 | 1 | 812 | 821 |  |
| E.VSVGDKIPAD.I | N | 18.74 | 999.5236 | -0.1 | 500.7690 | 2 | 10.76 | 1472 | 1 | 156 | 165 |  |
| V.KISLPVILL.D | N | 16.43 | 994.6790 | -0.3 | 498.3466 | 2 | 15.13 | 2259 | 1 | 976 | 984 |  |
| S.VGDKIPADI.R | N | 16.36 | 926.5073 | -0.4 | 464.2607 | 2 | 11.57 | 1624 | 1 | 158 | 166 |  |
| total 6 peptides |  |  |  |  |  |  |  |  |  |  |  |  |
| --- | --- | --- | --- | --- | --- | --- | --- | --- | --- | --- | --- | --- |

tr|A0A4Y7M702|A0A4Y7M702\_9CRUS

back to list

  

| Protein Coverage
| Supporting Peptides
|

Protein Coverage:

Supporting Peptides:

| Peptide | Uniq | -10lgP | Mass | ppm | m/z | z | RT | Scan | #Spec | Start | End | PTM |
| --- | --- | --- | --- | --- | --- | --- | --- | --- | --- | --- | --- | --- |
| M.TGDGVNDAPALK.K | N | 40.51 | 1156.5724 | -0.4 | 579.2932 | 2 | 10.50 | 1423 | 1 | 705 | 716 |  |
| V.SVGDKIPAD.I | N | 30.16 | 900.4552 | -0.6 | 451.2346 | 2 | 10.20 | 1370 | 1 | 157 | 165 |  |
| L.GFNPPDLDIM.N | N | 23.83 | 1117.5114 | -0.4 | 559.7628 | 2 | 14.97 | 2231 | 1 | 812 | 821 |  |
| E.VSVGDKIPAD.I | N | 18.74 | 999.5236 | -0.1 | 500.7690 | 2 | 10.76 | 1472 | 1 | 156 | 165 |  |
| I.KISLPVILL.D | N | 16.43 | 994.6790 | -0.3 | 498.3466 | 2 | 15.13 | 2259 | 1 | 976 | 984 |  |
| S.VGDKIPADI.R | N | 16.36 | 926.5073 | -0.4 | 464.2607 | 2 | 11.57 | 1624 | 1 | 158 | 166 |  |
| total 6 peptides |  |  |  |  |  |  |  |  |  |  |  |  |
| --- | --- | --- | --- | --- | --- | --- | --- | --- | --- | --- | --- | --- |

tr|A0A4Y7MHQ5|A0A4Y7MHQ5\_9CRUS

back to list

  

| Protein Coverage
| Supporting Peptides
|

Protein Coverage:

Supporting Peptides:

| Peptide | Uniq | -10lgP | Mass | ppm | m/z | z | RT | Scan | #Spec | Start | End | PTM |
| --- | --- | --- | --- | --- | --- | --- | --- | --- | --- | --- | --- | --- |
| M.TGDGVNDAPALK.K | N | 40.51 | 1156.5724 | -0.4 | 579.2932 | 2 | 10.50 | 1423 | 1 | 705 | 716 |  |
| V.SVGDKIPAD.I | N | 30.16 | 900.4552 | -0.6 | 451.2346 | 2 | 10.20 | 1370 | 1 | 157 | 165 |  |
| L.GFNPPDLDIM.N | N | 23.83 | 1117.5114 | -0.4 | 559.7628 | 2 | 14.97 | 2231 | 1 | 812 | 821 |  |
| E.VSVGDKIPAD.I | N | 18.74 | 999.5236 | -0.1 | 500.7690 | 2 | 10.76 | 1472 | 1 | 156 | 165 |  |
| V.KISLPVILL.D | N | 16.43 | 994.6790 | -0.3 | 498.3466 | 2 | 15.13 | 2259 | 1 | 976 | 984 |  |
| S.VGDKIPADI.R | N | 16.36 | 926.5073 | -0.4 | 464.2607 | 2 | 11.57 | 1624 | 1 | 158 | 166 |  |
| total 6 peptides |  |  |  |  |  |  |  |  |  |  |  |  |
| --- | --- | --- | --- | --- | --- | --- | --- | --- | --- | --- | --- | --- |

tr|A0A4Y7ME47|A0A4Y7ME47\_9CRUS

back to list

  

| Protein Coverage
| Supporting Peptides
|

Protein Coverage:

Supporting Peptides:

| Peptide | Uniq | -10lgP | Mass | ppm | m/z | z | RT | Scan | #Spec | Start | End | PTM |
| --- | --- | --- | --- | --- | --- | --- | --- | --- | --- | --- | --- | --- |
| M.TGDGVNDAPALK.K | N | 40.51 | 1156.5724 | -0.4 | 579.2932 | 2 | 10.50 | 1423 | 1 | 705 | 716 |  |
| V.SVGDKIPAD.I | N | 30.16 | 900.4552 | -0.6 | 451.2346 | 2 | 10.20 | 1370 | 1 | 157 | 165 |  |
| L.GFNPPDLDIM.N | N | 23.83 | 1117.5114 | -0.4 | 559.7628 | 2 | 14.97 | 2231 | 1 | 812 | 821 |  |
| E.VSVGDKIPAD.I | N | 18.74 | 999.5236 | -0.1 | 500.7690 | 2 | 10.76 | 1472 | 1 | 156 | 165 |  |
| V.KISLPVILL.D | N | 16.43 | 994.6790 | -0.3 | 498.3466 | 2 | 15.13 | 2259 | 1 | 976 | 984 |  |
| S.VGDKIPADI.R | N | 16.36 | 926.5073 | -0.4 | 464.2607 | 2 | 11.57 | 1624 | 1 | 158 | 166 |  |
| total 6 peptides |  |  |  |  |  |  |  |  |  |  |  |  |
| --- | --- | --- | --- | --- | --- | --- | --- | --- | --- | --- | --- | --- |

tr|A0A4Y7M4I0|A0A4Y7M4I0\_9CRUS

back to list

  

| Protein Coverage
| Supporting Peptides
|

Protein Coverage:

Supporting Peptides:

| Peptide | Uniq | -10lgP | Mass | ppm | m/z | z | RT | Scan | #Spec | Start | End | PTM |
| --- | --- | --- | --- | --- | --- | --- | --- | --- | --- | --- | --- | --- |
| M.TGDGVNDAPALK.K | N | 40.51 | 1156.5724 | -0.4 | 579.2932 | 2 | 10.50 | 1423 | 1 | 705 | 716 |  |
| V.SVGDKIPAD.I | N | 30.16 | 900.4552 | -0.6 | 451.2346 | 2 | 10.20 | 1370 | 1 | 157 | 165 |  |
| L.GFNPPDLDIM.N | N | 23.83 | 1117.5114 | -0.4 | 559.7628 | 2 | 14.97 | 2231 | 1 | 812 | 821 |  |
| E.VSVGDKIPAD.I | N | 18.74 | 999.5236 | -0.1 | 500.7690 | 2 | 10.76 | 1472 | 1 | 156 | 165 |  |
| V.KISLPVILL.D | N | 16.43 | 994.6790 | -0.3 | 498.3466 | 2 | 15.13 | 2259 | 1 | 976 | 984 |  |
| S.VGDKIPADI.R | N | 16.36 | 926.5073 | -0.4 | 464.2607 | 2 | 11.57 | 1624 | 1 | 158 | 166 |  |
| total 6 peptides |  |  |  |  |  |  |  |  |  |  |  |  |
| --- | --- | --- | --- | --- | --- | --- | --- | --- | --- | --- | --- | --- |

tr|A0A1D2MC18|A0A1D2MC18\_ORCCI

back to list

  

| Protein Coverage
| Supporting Peptides
|

Protein Coverage:

Supporting Peptides:

| Peptide | Uniq | -10lgP | Mass | ppm | m/z | z | RT | Scan | #Spec | Start | End | PTM |
| --- | --- | --- | --- | --- | --- | --- | --- | --- | --- | --- | --- | --- |
| M.TGDGVNDAPALK.K | N | 40.51 | 1156.5724 | -0.4 | 579.2932 | 2 | 10.50 | 1423 | 1 | 702 | 713 |  |
| V.SVGDKIPAD.I | N | 30.16 | 900.4552 | -0.6 | 451.2346 | 2 | 10.20 | 1370 | 1 | 158 | 166 |  |
| L.GFNPPDLDIM.T | N | 23.83 | 1117.5114 | -0.4 | 559.7628 | 2 | 14.97 | 2231 | 1 | 809 | 818 |  |
| E.VSVGDKIPAD.I | N | 18.74 | 999.5236 | -0.1 | 500.7690 | 2 | 10.76 | 1472 | 1 | 157 | 166 |  |
| M.KISLPVILL.D | N | 16.43 | 994.6790 | -0.3 | 498.3466 | 2 | 15.13 | 2259 | 1 | 973 | 981 |  |
| S.VGDKIPADI.R | N | 16.36 | 926.5073 | -0.4 | 464.2607 | 2 | 11.57 | 1624 | 1 | 159 | 167 |  |
| total 6 peptides |  |  |  |  |  |  |  |  |  |  |  |  |
| --- | --- | --- | --- | --- | --- | --- | --- | --- | --- | --- | --- | --- |

tr|E9HR84|E9HR84\_DAPPU

back to list

  

| Protein Coverage
| Supporting Peptides
|

Protein Coverage:

Supporting Peptides:

| Peptide | Uniq | -10lgP | Mass | ppm | m/z | z | RT | Scan | #Spec | Start | End | PTM |
| --- | --- | --- | --- | --- | --- | --- | --- | --- | --- | --- | --- | --- |
| M.TGDGVNDAPALK.K | N | 40.51 | 1156.5724 | -0.4 | 579.2932 | 2 | 10.50 | 1423 | 1 | 705 | 716 |  |
| V.SVGDKIPAD.I | N | 30.16 | 900.4552 | -0.6 | 451.2346 | 2 | 10.20 | 1370 | 1 | 157 | 165 |  |
| L.GFNPPDLDIM.N | N | 23.83 | 1117.5114 | -0.4 | 559.7628 | 2 | 14.97 | 2231 | 1 | 812 | 821 |  |
| E.VSVGDKIPAD.I | N | 18.74 | 999.5236 | -0.1 | 500.7690 | 2 | 10.76 | 1472 | 1 | 156 | 165 |  |
| V.KISLPVILL.D | N | 16.43 | 994.6790 | -0.3 | 498.3466 | 2 | 15.13 | 2259 | 1 | 976 | 984 |  |
| S.VGDKIPADI.R | N | 16.36 | 926.5073 | -0.4 | 464.2607 | 2 | 11.57 | 1624 | 1 | 158 | 166 |  |
| total 6 peptides |  |  |  |  |  |  |  |  |  |  |  |  |
| --- | --- | --- | --- | --- | --- | --- | --- | --- | --- | --- | --- | --- |

tr|A0A0P5VJX3|A0A0P5VJX3\_9CRUS

back to list

  

| Protein Coverage
| Supporting Peptides
|

Protein Coverage:

Supporting Peptides:

| Peptide | Uniq | -10lgP | Mass | ppm | m/z | z | RT | Scan | #Spec | Start | End | PTM |
| --- | --- | --- | --- | --- | --- | --- | --- | --- | --- | --- | --- | --- |
| M.TGDGVNDAPALK.K | N | 40.51 | 1156.5724 | -0.4 | 579.2932 | 2 | 10.50 | 1423 | 1 | 705 | 716 |  |
| V.SVGDKIPAD.I | N | 30.16 | 900.4552 | -0.6 | 451.2346 | 2 | 10.20 | 1370 | 1 | 157 | 165 |  |
| L.GFNPPDLDIM.N | N | 23.83 | 1117.5114 | -0.4 | 559.7628 | 2 | 14.97 | 2231 | 1 | 812 | 821 |  |
| E.VSVGDKIPAD.I | N | 18.74 | 999.5236 | -0.1 | 500.7690 | 2 | 10.76 | 1472 | 1 | 156 | 165 |  |
| V.KISLPVILL.D | N | 16.43 | 994.6790 | -0.3 | 498.3466 | 2 | 15.13 | 2259 | 1 | 976 | 984 |  |
| S.VGDKIPADI.R | N | 16.36 | 926.5073 | -0.4 | 464.2607 | 2 | 11.57 | 1624 | 1 | 158 | 166 |  |
| total 6 peptides |  |  |  |  |  |  |  |  |  |  |  |  |
| --- | --- | --- | --- | --- | --- | --- | --- | --- | --- | --- | --- | --- |

tr|A0A4Y7LYL4|A0A4Y7LYL4\_9CRUS

back to list

  

| Protein Coverage
| Supporting Peptides
|

Protein Coverage:

Supporting Peptides:

| Peptide | Uniq | -10lgP | Mass | ppm | m/z | z | RT | Scan | #Spec | Start | End | PTM |
| --- | --- | --- | --- | --- | --- | --- | --- | --- | --- | --- | --- | --- |
| M.TGDGVNDAPALK.K | N | 40.51 | 1156.5724 | -0.4 | 579.2932 | 2 | 10.50 | 1423 | 1 | 705 | 716 |  |
| V.SVGDKIPAD.I | N | 30.16 | 900.4552 | -0.6 | 451.2346 | 2 | 10.20 | 1370 | 1 | 157 | 165 |  |
| L.GFNPPDLDIM.N | N | 23.83 | 1117.5114 | -0.4 | 559.7628 | 2 | 14.97 | 2231 | 1 | 812 | 821 |  |
| E.VSVGDKIPAD.I | N | 18.74 | 999.5236 | -0.1 | 500.7690 | 2 | 10.76 | 1472 | 1 | 156 | 165 |  |
| V.KISLPVILL.D | N | 16.43 | 994.6790 | -0.3 | 498.3466 | 2 | 15.13 | 2259 | 1 | 976 | 984 |  |
| S.VGDKIPADI.R | N | 16.36 | 926.5073 | -0.4 | 464.2607 | 2 | 11.57 | 1624 | 1 | 158 | 166 |  |
| total 6 peptides |  |  |  |  |  |  |  |  |  |  |  |  |
| --- | --- | --- | --- | --- | --- | --- | --- | --- | --- | --- | --- | --- |

tr|A0A0C5E1J4|A0A0C5E1J4\_CHEDE

back to list

  

| Protein Coverage
| Supporting Peptides
|

Protein Coverage:

Supporting Peptides:

| Peptide | Uniq | -10lgP | Mass | ppm | m/z | z | RT | Scan | #Spec | Start | End | PTM |
| --- | --- | --- | --- | --- | --- | --- | --- | --- | --- | --- | --- | --- |
| M.TGDGVNDAPALK.K | N | 40.51 | 1156.5724 | -0.4 | 579.2932 | 2 | 10.50 | 1423 | 1 | 701 | 712 |  |
| V.SVGDKIPAD.I | N | 30.16 | 900.4552 | -0.6 | 451.2346 | 2 | 10.20 | 1370 | 1 | 154 | 162 |  |
| L.GFNPPDLDIM.D | N | 23.83 | 1117.5114 | -0.4 | 559.7628 | 2 | 14.97 | 2231 | 1 | 808 | 817 |  |
| E.VSVGDKIPAD.I | N | 18.74 | 999.5236 | -0.1 | 500.7690 | 2 | 10.76 | 1472 | 1 | 153 | 162 |  |
| S.VGDKIPADI.R | N | 16.36 | 926.5073 | -0.4 | 464.2607 | 2 | 11.57 | 1624 | 1 | 155 | 163 |  |
| K.HTDAIPDPKA.V | N | 15.37 | 1063.5298 | -0.3 | 532.7720 | 2 | 10.00 | 1334 | 1 | 190 | 199 |  |
| total 6 peptides |  |  |  |  |  |  |  |  |  |  |  |  |
| --- | --- | --- | --- | --- | --- | --- | --- | --- | --- | --- | --- | --- |

tr|Q86GD6|Q86GD6\_PROCL

back to list

  

| Protein Coverage
| Supporting Peptides
|

Protein Coverage:

Supporting Peptides:

| Peptide | Uniq | -10lgP | Mass | ppm | m/z | z | RT | Scan | #Spec | Start | End | PTM |
| --- | --- | --- | --- | --- | --- | --- | --- | --- | --- | --- | --- | --- |
| N.LNLPVPVVGEPPATK.Q | Y | 59.77 | 1529.8817 | -1.6 | 765.9469 | 2 | 13.13 | 1909 | 1 | 4993 | 5007 |  |
| A.VIKIPF.I | N | 17.57 | 715.4632 | 0.5 | 358.7390 | 2 | 13.33 | 1944 | 1 | 7458 | 7463 |  |
| R.IKDPNAATPPEII.T | N | 15.97 | 1377.7504 | -1.0 | 689.8818 | 2 | 12.19 | 1742 | 1 | 7335 | 7347 |  |
| total 3 peptides |  |  |  |  |  |  |  |  |  |  |  |  |
| --- | --- | --- | --- | --- | --- | --- | --- | --- | --- | --- | --- | --- |

tr|A0A444TVR3|A0A444TVR3\_ARMVU

back to list

  

| Protein Coverage
| Supporting Peptides
|

Protein Coverage:

Supporting Peptides:

| Peptide | Uniq | -10lgP | Mass | ppm | m/z | z | RT | Scan | #Spec | Start | End | PTM |
| --- | --- | --- | --- | --- | --- | --- | --- | --- | --- | --- | --- | --- |
| S.KWLPVAPEFPHPIPQ.F | Y | 44.03 | 1754.9507 | -8.3 | 878.4753 | 2 | 13.73 | 2015 | 1 | 94 | 108 |  |
| V.APEFPHPIPQ.F | Y | 29.02 | 1131.5713 | -0.9 | 566.7924 | 2 | 11.82 | 1672 | 1 | 99 | 108 |  |
| W.LPVAPEFPHPIPQ.F | Y | 25.73 | 1440.7765 | -0.8 | 721.3950 | 2 | 12.95 | 1877 | 1 | 96 | 108 |  |
| P.VAPEFPHPIPQ.F | Y | 17.95 | 1230.6396 | -0.4 | 616.3268 | 2 | 12.02 | 1709 | 1 | 98 | 108 |  |
| total 4 peptides |  |  |  |  |  |  |  |  |  |  |  |  |
| --- | --- | --- | --- | --- | --- | --- | --- | --- | --- | --- | --- | --- |

tr|A0A7R8CX21|A0A7R8CX21\_LEPSM

back to list

  

| Protein Coverage
| Supporting Peptides
|

Protein Coverage:

Supporting Peptides:

| Peptide | Uniq | -10lgP | Mass | ppm | m/z | z | RT | Scan | #Spec | Start | End | PTM |
| --- | --- | --- | --- | --- | --- | --- | --- | --- | --- | --- | --- | --- |
| K.VTVPSIDDKEDMQF.A | N | 40.27 | 1622.7498 | -0.4 | 812.3818 | 2 | 12.86 | 1860 | 1 | 318 | 331 |  |
| K.VTVPSIDDKEDMQ.F | N | 31.89 | 1475.6814 | -0.1 | 738.8479 | 2 | 11.58 | 1627 | 1 | 318 | 330 |  |
| L.ITGESGAGK.T | N | 23.91 | 818.4134 | -0.2 | 410.2139 | 2 | 8.67 | 1104 | 1 | 181 | 189 |  |
| K.NKDPLNDTI.V | N | 20.74 | 1028.5138 | -0.9 | 515.2637 | 2 | 11.46 | 1603 | 1 | 545 | 553 |  |
| total 4 peptides |  |  |  |  |  |  |  |  |  |  |  |  |
| --- | --- | --- | --- | --- | --- | --- | --- | --- | --- | --- | --- | --- |

tr|A0A0K2V7Y7|A0A0K2V7Y7\_LEPSM

back to list

  

| Protein Coverage
| Supporting Peptides
|

Protein Coverage:

Supporting Peptides:

| Peptide | Uniq | -10lgP | Mass | ppm | m/z | z | RT | Scan | #Spec | Start | End | PTM |
| --- | --- | --- | --- | --- | --- | --- | --- | --- | --- | --- | --- | --- |
| K.VTVPSIDDKEDMQF.A | N | 40.27 | 1622.7498 | -0.4 | 812.3818 | 2 | 12.86 | 1860 | 1 | 318 | 331 |  |
| K.VTVPSIDDKEDMQ.F | N | 31.89 | 1475.6814 | -0.1 | 738.8479 | 2 | 11.58 | 1627 | 1 | 318 | 330 |  |
| L.ITGESGAGK.T | N | 23.91 | 818.4134 | -0.2 | 410.2139 | 2 | 8.67 | 1104 | 1 | 181 | 189 |  |
| K.NKDPLNDTI.V | N | 20.74 | 1028.5138 | -0.9 | 515.2637 | 2 | 11.46 | 1603 | 1 | 599 | 607 |  |
| total 4 peptides |  |  |  |  |  |  |  |  |  |  |  |  |
| --- | --- | --- | --- | --- | --- | --- | --- | --- | --- | --- | --- | --- |

tr|A0A7T8HL88|A0A7T8HL88\_CALRO

back to list

  

| Protein Coverage
| Supporting Peptides
|

Protein Coverage:

Supporting Peptides:

| Peptide | Uniq | -10lgP | Mass | ppm | m/z | z | RT | Scan | #Spec | Start | End | PTM |
| --- | --- | --- | --- | --- | --- | --- | --- | --- | --- | --- | --- | --- |
| K.VTVPSIDDKEDMQF.A | N | 40.27 | 1622.7498 | -0.4 | 812.3818 | 2 | 12.86 | 1860 | 1 | 318 | 331 |  |
| K.VTVPSIDDKEDMQ.F | N | 31.89 | 1475.6814 | -0.1 | 738.8479 | 2 | 11.58 | 1627 | 1 | 318 | 330 |  |
| L.ITGESGAGK.T | N | 23.91 | 818.4134 | -0.2 | 410.2139 | 2 | 8.67 | 1104 | 1 | 181 | 189 |  |
| K.NKDPLNDTI.V | N | 20.74 | 1028.5138 | -0.9 | 515.2637 | 2 | 11.46 | 1603 | 1 | 599 | 607 |  |
| total 4 peptides |  |  |  |  |  |  |  |  |  |  |  |  |
| --- | --- | --- | --- | --- | --- | --- | --- | --- | --- | --- | --- | --- |

tr|A0A7T8HL29|A0A7T8HL29\_CALRO

back to list

  

| Protein Coverage
| Supporting Peptides
|

Protein Coverage:

Supporting Peptides:

| Peptide | Uniq | -10lgP | Mass | ppm | m/z | z | RT | Scan | #Spec | Start | End | PTM |
| --- | --- | --- | --- | --- | --- | --- | --- | --- | --- | --- | --- | --- |
| K.VTVPSIDDKEDMQF.A | N | 40.27 | 1622.7498 | -0.4 | 812.3818 | 2 | 12.86 | 1860 | 1 | 318 | 331 |  |
| K.VTVPSIDDKEDMQ.F | N | 31.89 | 1475.6814 | -0.1 | 738.8479 | 2 | 11.58 | 1627 | 1 | 318 | 330 |  |
| L.ITGESGAGK.T | N | 23.91 | 818.4134 | -0.2 | 410.2139 | 2 | 8.67 | 1104 | 1 | 181 | 189 |  |
| K.NKDPLNDTI.V | N | 20.74 | 1028.5138 | -0.9 | 515.2637 | 2 | 11.46 | 1603 | 1 | 599 | 607 |  |
| total 4 peptides |  |  |  |  |  |  |  |  |  |  |  |  |
| --- | --- | --- | --- | --- | --- | --- | --- | --- | --- | --- | --- | --- |

tr|A0A0K2T3U6|A0A0K2T3U6\_LEPSM

back to list

  

| Protein Coverage
| Supporting Peptides
|

Protein Coverage:

Supporting Peptides:

| Peptide | Uniq | -10lgP | Mass | ppm | m/z | z | RT | Scan | #Spec | Start | End | PTM |
| --- | --- | --- | --- | --- | --- | --- | --- | --- | --- | --- | --- | --- |
| K.VTVPSIDDKEDMQF.A | N | 40.27 | 1622.7498 | -0.4 | 812.3818 | 2 | 12.86 | 1860 | 1 | 318 | 331 |  |
| K.VTVPSIDDKEDMQ.F | N | 31.89 | 1475.6814 | -0.1 | 738.8479 | 2 | 11.58 | 1627 | 1 | 318 | 330 |  |
| L.ITGESGAGK.T | N | 23.91 | 818.4134 | -0.2 | 410.2139 | 2 | 8.67 | 1104 | 1 | 181 | 189 |  |
| K.NKDPLNDTI.V | N | 20.74 | 1028.5138 | -0.9 | 515.2637 | 2 | 11.46 | 1603 | 1 | 599 | 607 |  |
| total 4 peptides |  |  |  |  |  |  |  |  |  |  |  |  |
| --- | --- | --- | --- | --- | --- | --- | --- | --- | --- | --- | --- | --- |

tr|A0A0K2TSJ7|A0A0K2TSJ7\_LEPSM

back to list

  

| Protein Coverage
| Supporting Peptides
|

Protein Coverage:

Supporting Peptides:

| Peptide | Uniq | -10lgP | Mass | ppm | m/z | z | RT | Scan | #Spec | Start | End | PTM |
| --- | --- | --- | --- | --- | --- | --- | --- | --- | --- | --- | --- | --- |
| K.VTVPSIDDKEDMQF.A | N | 40.27 | 1622.7498 | -0.4 | 812.3818 | 2 | 12.86 | 1860 | 1 | 338 | 351 |  |
| K.VTVPSIDDKEDMQ.F | N | 31.89 | 1475.6814 | -0.1 | 738.8479 | 2 | 11.58 | 1627 | 1 | 338 | 350 |  |
| L.ITGESGAGK.T | N | 23.91 | 818.4134 | -0.2 | 410.2139 | 2 | 8.67 | 1104 | 1 | 201 | 209 |  |
| K.NKDPLNDTI.V | N | 20.74 | 1028.5138 | -0.9 | 515.2637 | 2 | 11.46 | 1603 | 1 | 619 | 627 |  |
| total 4 peptides |  |  |  |  |  |  |  |  |  |  |  |  |
| --- | --- | --- | --- | --- | --- | --- | --- | --- | --- | --- | --- | --- |

tr|A0A0P4WFG0|A0A0P4WFG0\_SCYOL

back to list

  

| Protein Coverage
| Supporting Peptides
|

Protein Coverage:

Supporting Peptides:

| Peptide | Uniq | -10lgP | Mass | ppm | m/z | z | RT | Scan | #Spec | Start | End | PTM |
| --- | --- | --- | --- | --- | --- | --- | --- | --- | --- | --- | --- | --- |
| M.ELAAPYVATIMILDDDHGGIF.N | Y | 39.77 | 2260.1084 | -1.0 | 1131.0603 | 2 | 15.95 | 2403 | 1 | 497 | 517 |  |
| E.LAAPYVATIMILDDDHGGIF.N | Y | 28.96 | 2131.0659 | -0.4 | 1066.5398 | 2 | 15.73 | 2367 | 1 | 498 | 517 |  |
| L.AAPYVATIMILDDDHGGIF.N | Y | 24.35 | 2017.9818 | -0.3 | 1009.9979 | 2 | 15.31 | 2291 | 1 | 499 | 517 |  |
| total 3 peptides |  |  |  |  |  |  |  |  |  |  |  |  |
| --- | --- | --- | --- | --- | --- | --- | --- | --- | --- | --- | --- | --- |

tr|A0A5B7GFK1|A0A5B7GFK1\_PORTR

back to list

  

| Protein Coverage
| Supporting Peptides
|

Protein Coverage:

Supporting Peptides:

| Peptide | Uniq | -10lgP | Mass | ppm | m/z | z | RT | Scan | #Spec | Start | End | PTM |
| --- | --- | --- | --- | --- | --- | --- | --- | --- | --- | --- | --- | --- |
| M.ELAAPYVATIMILDDDHGGIF.N | Y | 39.77 | 2260.1084 | -1.0 | 1131.0603 | 2 | 15.95 | 2403 | 1 | 497 | 517 |  |
| E.LAAPYVATIMILDDDHGGIF.N | Y | 28.96 | 2131.0659 | -0.4 | 1066.5398 | 2 | 15.73 | 2367 | 1 | 498 | 517 |  |
| L.AAPYVATIMILDDDHGGIF.N | Y | 24.35 | 2017.9818 | -0.3 | 1009.9979 | 2 | 15.31 | 2291 | 1 | 499 | 517 |  |
| total 3 peptides |  |  |  |  |  |  |  |  |  |  |  |  |
| --- | --- | --- | --- | --- | --- | --- | --- | --- | --- | --- | --- | --- |

tr|A0A110A0P3|A0A110A0P3\_ASTLP

back to list

  

| Protein Coverage
| Supporting Peptides
|

Protein Coverage:

Supporting Peptides:

| Peptide | Uniq | -10lgP | Mass | ppm | m/z | z | RT | Scan | #Spec | Start | End | PTM |
| --- | --- | --- | --- | --- | --- | --- | --- | --- | --- | --- | --- | --- |
| M.ELAAPYVATIMILDDDHGGIF.N | Y | 39.77 | 2260.1084 | -1.0 | 1131.0603 | 2 | 15.95 | 2403 | 1 | 498 | 518 |  |
| E.LAAPYVATIMILDDDHGGIF.N | Y | 28.96 | 2131.0659 | -0.4 | 1066.5398 | 2 | 15.73 | 2367 | 1 | 499 | 518 |  |
| L.AAPYVATIMILDDDHGGIF.N | Y | 24.35 | 2017.9818 | -0.3 | 1009.9979 | 2 | 15.31 | 2291 | 1 | 500 | 518 |  |
| total 3 peptides |  |  |  |  |  |  |  |  |  |  |  |  |
| --- | --- | --- | --- | --- | --- | --- | --- | --- | --- | --- | --- | --- |

tr|A0A5B7EE36|A0A5B7EE36\_PORTR

back to list

  

| Protein Coverage
| Supporting Peptides
|

Protein Coverage:

Supporting Peptides:

| Peptide | Uniq | -10lgP | Mass | ppm | m/z | z | RT | Scan | #Spec | Start | End | PTM |
| --- | --- | --- | --- | --- | --- | --- | --- | --- | --- | --- | --- | --- |
| K.SPGVIDAGLVMH.Q | N | 44.78 | 1194.6067 | -0.9 | 598.3101 | 2 | 12.56 | 1806 | 1 | 205 | 216 |  |
| N.ETKSPGVIDAGLVM.H | N | 16.36 | 1415.7330 | -0.4 | 708.8735 | 2 | 12.98 | 1882 | 1 | 202 | 215 |  |
| R.NEVPPHLF.A | N | 16.25 | 951.4814 | -0.3 | 476.7478 | 2 | 13.07 | 1899 | 1 | 150 | 157 |  |
| total 3 peptides |  |  |  |  |  |  |  |  |  |  |  |  |
| --- | --- | --- | --- | --- | --- | --- | --- | --- | --- | --- | --- | --- |

tr|A0A2Z4I2U3|A0A2Z4I2U3\_CALSI

back to list

  

| Protein Coverage
| Supporting Peptides
|

Protein Coverage:

Supporting Peptides:

| Peptide | Uniq | -10lgP | Mass | ppm | m/z | z | RT | Scan | #Spec | Start | End | PTM |
| --- | --- | --- | --- | --- | --- | --- | --- | --- | --- | --- | --- | --- |
| V.SVGDKIPAD.L | N | 30.16 | 900.4552 | -0.6 | 451.2346 | 2 | 10.20 | 1370 | 1 | 154 | 162 |  |
| L.GFNPPDLDIM.E | N | 23.83 | 1117.5114 | -0.4 | 559.7628 | 2 | 14.97 | 2231 | 1 | 808 | 817 |  |
| E.VSVGDKIPAD.L | N | 18.74 | 999.5236 | -0.1 | 500.7690 | 2 | 10.76 | 1472 | 1 | 153 | 162 |  |
| L.KISLPVLLL.D | N | 16.43 | 994.6790 | -0.3 | 498.3466 | 2 | 15.13 | 2259 | 1 | 972 | 980 |  |
| S.VGDKIPADL.R | N | 16.36 | 926.5073 | -0.4 | 464.2607 | 2 | 11.57 | 1624 | 1 | 155 | 163 |  |
| K.HTDAIPDPKA.V | N | 15.37 | 1063.5298 | -0.3 | 532.7720 | 2 | 10.00 | 1334 | 1 | 190 | 199 |  |
| total 6 peptides |  |  |  |  |  |  |  |  |  |  |  |  |
| --- | --- | --- | --- | --- | --- | --- | --- | --- | --- | --- | --- | --- |

tr|Q95YM2|Q95YM2\_PROCL

back to list

  

| Protein Coverage
| Supporting Peptides
|

Protein Coverage:

Supporting Peptides:

| Peptide | Uniq | -10lgP | Mass | ppm | m/z | z | RT | Scan | #Spec | Start | End | PTM |
| --- | --- | --- | --- | --- | --- | --- | --- | --- | --- | --- | --- | --- |
| K.SPFDKPGPPIGPF.D | Y | 41.08 | 1354.6920 | 1.2 | 678.3541 | 2 | 13.50 | 1974 | 1 | 16981 | 16993 |  |
| L.IPVGDPDLK.V | Y | 23.50 | 952.5229 | -0.4 | 477.2685 | 2 | 11.07 | 1530 | 1 | 1731 | 1739 |  |
| total 2 peptides |  |  |  |  |  |  |  |  |  |  |  |  |
| --- | --- | --- | --- | --- | --- | --- | --- | --- | --- | --- | --- | --- |

tr|A0A6P7F415|A0A6P7F415\_DIAVI

back to list

  

| Protein Coverage
| Supporting Peptides
|

Protein Coverage:

Supporting Peptides:

| Peptide | Uniq | -10lgP | Mass | ppm | m/z | z | RT | Scan | #Spec | Start | End | PTM |
| --- | --- | --- | --- | --- | --- | --- | --- | --- | --- | --- | --- | --- |
| L.SILEEESMFPK.A | N | 26.45 | 1308.6271 | -0.7 | 655.3204 | 2 | 12.91 | 1869 | 1 | 533 | 543 |  |
| L.ITGESGAGK.T | N | 23.91 | 818.4134 | -0.2 | 410.2139 | 2 | 8.67 | 1104 | 1 | 178 | 186 |  |
| R.NEVPPHIF.A | N | 16.25 | 951.4814 | -0.3 | 476.7478 | 2 | 13.07 | 1899 | 1 | 150 | 157 |  |
| M.FDRVF.K | N | 15.90 | 682.3438 | -0.9 | 342.1789 | 2 | 11.93 | 1692 | 1 | 433 | 437 |  |
| M.FDRVFKF.L | N | 15.27 | 957.5072 | -1.0 | 479.7604 | 2 | 12.36 | 1771 | 1 | 433 | 439 |  |
| total 5 peptides |  |  |  |  |  |  |  |  |  |  |  |  |
| --- | --- | --- | --- | --- | --- | --- | --- | --- | --- | --- | --- | --- |

tr|A0A5E4QJ96|A0A5E4QJ96\_9NEOP

back to list

  

| Protein Coverage
| Supporting Peptides
|

Protein Coverage:

Supporting Peptides:

| Peptide | Uniq | -10lgP | Mass | ppm | m/z | z | RT | Scan | #Spec | Start | End | PTM |
| --- | --- | --- | --- | --- | --- | --- | --- | --- | --- | --- | --- | --- |
| V.LPDGEHDLDR.A | Y | 33.64 | 1165.5364 | -0.2 | 583.7753 | 2 | 10.20 | 1369 | 1 | 97 | 106 |  |
| V.LPDGEHDLDRA.Q | Y | 22.49 | 1236.5735 | -0.7 | 619.2936 | 2 | 10.49 | 1422 | 2 | 97 | 107 |  |
| E.VLPDGEHDLDRA.Q | Y | 16.15 | 1335.6418 | -0.7 | 668.8277 | 2 | 10.73 | 1466 | 1 | 96 | 107 |  |
| total 3 peptides |  |  |  |  |  |  |  |  |  |  |  |  |
| --- | --- | --- | --- | --- | --- | --- | --- | --- | --- | --- | --- | --- |

tr|A0A0A7BYS9|A0A0A7BYS9\_OSTFU

back to list

  

| Protein Coverage
| Supporting Peptides
|

Protein Coverage:

Supporting Peptides:

| Peptide | Uniq | -10lgP | Mass | ppm | m/z | z | RT | Scan | #Spec | Start | End | PTM |
| --- | --- | --- | --- | --- | --- | --- | --- | --- | --- | --- | --- | --- |
| V.LPDGEHDLDR.A | Y | 33.64 | 1165.5364 | -0.2 | 583.7753 | 2 | 10.20 | 1369 | 1 | 192 | 201 |  |
| V.LPDGEHDLDRA.Q | Y | 22.49 | 1236.5735 | -0.7 | 619.2936 | 2 | 10.49 | 1422 | 2 | 192 | 202 |  |
| E.VLPDGEHDLDRA.Q | Y | 16.15 | 1335.6418 | -0.7 | 668.8277 | 2 | 10.73 | 1466 | 1 | 191 | 202 |  |
| total 3 peptides |  |  |  |  |  |  |  |  |  |  |  |  |
| --- | --- | --- | --- | --- | --- | --- | --- | --- | --- | --- | --- | --- |

tr|A0A2H1WAZ2|A0A2H1WAZ2\_SPOFR

back to list

  

| Protein Coverage
| Supporting Peptides
|

Protein Coverage:

Supporting Peptides:

| Peptide | Uniq | -10lgP | Mass | ppm | m/z | z | RT | Scan | #Spec | Start | End | PTM |
| --- | --- | --- | --- | --- | --- | --- | --- | --- | --- | --- | --- | --- |
| V.LPDGEHDLDR.A | Y | 33.64 | 1165.5364 | -0.2 | 583.7753 | 2 | 10.20 | 1369 | 1 | 65 | 74 |  |
| V.LPDGEHDLDRA.Q | Y | 22.49 | 1236.5735 | -0.7 | 619.2936 | 2 | 10.49 | 1422 | 2 | 65 | 75 |  |
| E.VLPDGEHDLDRA.Q | Y | 16.15 | 1335.6418 | -0.7 | 668.8277 | 2 | 10.73 | 1466 | 1 | 64 | 75 |  |
| total 3 peptides |  |  |  |  |  |  |  |  |  |  |  |  |
| --- | --- | --- | --- | --- | --- | --- | --- | --- | --- | --- | --- | --- |

tr|A0A6J3BVA9|A0A6J3BVA9\_GALME

back to list

  

| Protein Coverage
| Supporting Peptides
|

Protein Coverage:

Supporting Peptides:

| Peptide | Uniq | -10lgP | Mass | ppm | m/z | z | RT | Scan | #Spec | Start | End | PTM |
| --- | --- | --- | --- | --- | --- | --- | --- | --- | --- | --- | --- | --- |
| V.LPDGEHDLDR.A | Y | 33.64 | 1165.5364 | -0.2 | 583.7753 | 2 | 10.20 | 1369 | 1 | 73 | 82 |  |
| V.LPDGEHDLDRA.Q | Y | 22.49 | 1236.5735 | -0.7 | 619.2936 | 2 | 10.49 | 1422 | 2 | 73 | 83 |  |
| E.VLPDGEHDLDRA.Q | Y | 16.15 | 1335.6418 | -0.7 | 668.8277 | 2 | 10.73 | 1466 | 1 | 72 | 83 |  |
| total 3 peptides |  |  |  |  |  |  |  |  |  |  |  |  |
| --- | --- | --- | --- | --- | --- | --- | --- | --- | --- | --- | --- | --- |

tr|A0A6J1WK99|A0A6J1WK99\_GALME

back to list

  

| Protein Coverage
| Supporting Peptides
|

Protein Coverage:

Supporting Peptides:

| Peptide | Uniq | -10lgP | Mass | ppm | m/z | z | RT | Scan | #Spec | Start | End | PTM |
| --- | --- | --- | --- | --- | --- | --- | --- | --- | --- | --- | --- | --- |
| V.LPDGEHDLDR.A | Y | 33.64 | 1165.5364 | -0.2 | 583.7753 | 2 | 10.20 | 1369 | 1 | 73 | 82 |  |
| V.LPDGEHDLDRA.Q | Y | 22.49 | 1236.5735 | -0.7 | 619.2936 | 2 | 10.49 | 1422 | 2 | 73 | 83 |  |
| E.VLPDGEHDLDRA.Q | Y | 16.15 | 1335.6418 | -0.7 | 668.8277 | 2 | 10.73 | 1466 | 1 | 72 | 83 |  |
| total 3 peptides |  |  |  |  |  |  |  |  |  |  |  |  |
| --- | --- | --- | --- | --- | --- | --- | --- | --- | --- | --- | --- | --- |

tr|A0A444T6K9|A0A444T6K9\_ARMVU

back to list

  

| Protein Coverage
| Supporting Peptides
|

Protein Coverage:

Supporting Peptides:

| Peptide | Uniq | -10lgP | Mass | ppm | m/z | z | RT | Scan | #Spec | Start | End | PTM |
| --- | --- | --- | --- | --- | --- | --- | --- | --- | --- | --- | --- | --- |
| V.LPDGEHDLDR.A | Y | 33.64 | 1165.5364 | -0.2 | 583.7753 | 2 | 10.20 | 1369 | 1 | 148 | 157 |  |
| V.LPDGEHDLDRA.Q | Y | 22.49 | 1236.5735 | -0.7 | 619.2936 | 2 | 10.49 | 1422 | 2 | 148 | 158 |  |
| I.VLPDGEHDLDRA.Q | Y | 16.15 | 1335.6418 | -0.7 | 668.8277 | 2 | 10.73 | 1466 | 1 | 147 | 158 |  |
| total 3 peptides |  |  |  |  |  |  |  |  |  |  |  |  |
| --- | --- | --- | --- | --- | --- | --- | --- | --- | --- | --- | --- | --- |

tr|A0A222AJC5|A0A222AJC5\_SPOLI

back to list

  

| Protein Coverage
| Supporting Peptides
|

Protein Coverage:

Supporting Peptides:

| Peptide | Uniq | -10lgP | Mass | ppm | m/z | z | RT | Scan | #Spec | Start | End | PTM |
| --- | --- | --- | --- | --- | --- | --- | --- | --- | --- | --- | --- | --- |
| V.LPDGEHDLDR.A | Y | 33.64 | 1165.5364 | -0.2 | 583.7753 | 2 | 10.20 | 1369 | 1 | 192 | 201 |  |
| V.LPDGEHDLDRA.Q | Y | 22.49 | 1236.5735 | -0.7 | 619.2936 | 2 | 10.49 | 1422 | 2 | 192 | 202 |  |
| E.VLPDGEHDLDRA.Q | Y | 16.15 | 1335.6418 | -0.7 | 668.8277 | 2 | 10.73 | 1466 | 1 | 191 | 202 |  |
| total 3 peptides |  |  |  |  |  |  |  |  |  |  |  |  |
| --- | --- | --- | --- | --- | --- | --- | --- | --- | --- | --- | --- | --- |

tr|A0A0Q9WRY5|A0A0Q9WRY5\_DROVI

back to list

  

| Protein Coverage
| Supporting Peptides
|

Protein Coverage:

Supporting Peptides:

| Peptide | Uniq | -10lgP | Mass | ppm | m/z | z | RT | Scan | #Spec | Start | End | PTM |
| --- | --- | --- | --- | --- | --- | --- | --- | --- | --- | --- | --- | --- |
| V.LPDGEHDLDR.A | Y | 33.64 | 1165.5364 | -0.2 | 583.7753 | 2 | 10.20 | 1369 | 1 | 192 | 201 |  |
| V.LPDGEHDLDRA.Q | Y | 22.49 | 1236.5735 | -0.7 | 619.2936 | 2 | 10.49 | 1422 | 2 | 192 | 202 |  |
| E.VLPDGEHDLDRA.Q | Y | 16.15 | 1335.6418 | -0.7 | 668.8277 | 2 | 10.73 | 1466 | 1 | 191 | 202 |  |
| total 3 peptides |  |  |  |  |  |  |  |  |  |  |  |  |
| --- | --- | --- | --- | --- | --- | --- | --- | --- | --- | --- | --- | --- |

tr|B4NKQ7|B4NKQ7\_DROWI

back to list

  

| Protein Coverage
| Supporting Peptides
|

Protein Coverage:

Supporting Peptides:

| Peptide | Uniq | -10lgP | Mass | ppm | m/z | z | RT | Scan | #Spec | Start | End | PTM |
| --- | --- | --- | --- | --- | --- | --- | --- | --- | --- | --- | --- | --- |
| V.LPDGEHDLDR.A | Y | 33.64 | 1165.5364 | -0.2 | 583.7753 | 2 | 10.20 | 1369 | 1 | 192 | 201 |  |
| V.LPDGEHDLDRA.Q | Y | 22.49 | 1236.5735 | -0.7 | 619.2936 | 2 | 10.49 | 1422 | 2 | 192 | 202 |  |
| E.VLPDGEHDLDRA.Q | Y | 16.15 | 1335.6418 | -0.7 | 668.8277 | 2 | 10.73 | 1466 | 1 | 191 | 202 |  |
| total 3 peptides |  |  |  |  |  |  |  |  |  |  |  |  |
| --- | --- | --- | --- | --- | --- | --- | --- | --- | --- | --- | --- | --- |

tr|B4LYD3|B4LYD3\_DROVI

back to list

  

| Protein Coverage
| Supporting Peptides
|

Protein Coverage:

Supporting Peptides:

| Peptide | Uniq | -10lgP | Mass | ppm | m/z | z | RT | Scan | #Spec | Start | End | PTM |
| --- | --- | --- | --- | --- | --- | --- | --- | --- | --- | --- | --- | --- |
| V.LPDGEHDLDR.A | Y | 33.64 | 1165.5364 | -0.2 | 583.7753 | 2 | 10.20 | 1369 | 1 | 192 | 201 |  |
| V.LPDGEHDLDRA.Q | Y | 22.49 | 1236.5735 | -0.7 | 619.2936 | 2 | 10.49 | 1422 | 2 | 192 | 202 |  |
| E.VLPDGEHDLDRA.Q | Y | 16.15 | 1335.6418 | -0.7 | 668.8277 | 2 | 10.73 | 1466 | 1 | 191 | 202 |  |
| total 3 peptides |  |  |  |  |  |  |  |  |  |  |  |  |
| --- | --- | --- | --- | --- | --- | --- | --- | --- | --- | --- | --- | --- |

tr|A0A0Q9WWZ4|A0A0Q9WWZ4\_DROWI

back to list

  

| Protein Coverage
| Supporting Peptides
|

Protein Coverage:

Supporting Peptides:

| Peptide | Uniq | -10lgP | Mass | ppm | m/z | z | RT | Scan | #Spec | Start | End | PTM |
| --- | --- | --- | --- | --- | --- | --- | --- | --- | --- | --- | --- | --- |
| V.LPDGEHDLDR.A | Y | 33.64 | 1165.5364 | -0.2 | 583.7753 | 2 | 10.20 | 1369 | 1 | 192 | 201 |  |
| V.LPDGEHDLDRA.Q | Y | 22.49 | 1236.5735 | -0.7 | 619.2936 | 2 | 10.49 | 1422 | 2 | 192 | 202 |  |
| E.VLPDGEHDLDRA.Q | Y | 16.15 | 1335.6418 | -0.7 | 668.8277 | 2 | 10.73 | 1466 | 1 | 191 | 202 |  |
| total 3 peptides |  |  |  |  |  |  |  |  |  |  |  |  |
| --- | --- | --- | --- | --- | --- | --- | --- | --- | --- | --- | --- | --- |

tr|A0A0Q9WQB4|A0A0Q9WQB4\_DROVI

back to list

  

| Protein Coverage
| Supporting Peptides
|

Protein Coverage:

Supporting Peptides:

| Peptide | Uniq | -10lgP | Mass | ppm | m/z | z | RT | Scan | #Spec | Start | End | PTM |
| --- | --- | --- | --- | --- | --- | --- | --- | --- | --- | --- | --- | --- |
| V.LPDGEHDLDR.A | Y | 33.64 | 1165.5364 | -0.2 | 583.7753 | 2 | 10.20 | 1369 | 1 | 192 | 201 |  |
| V.LPDGEHDLDRA.Q | Y | 22.49 | 1236.5735 | -0.7 | 619.2936 | 2 | 10.49 | 1422 | 2 | 192 | 202 |  |
| E.VLPDGEHDLDRA.Q | Y | 16.15 | 1335.6418 | -0.7 | 668.8277 | 2 | 10.73 | 1466 | 1 | 191 | 202 |  |
| total 3 peptides |  |  |  |  |  |  |  |  |  |  |  |  |
| --- | --- | --- | --- | --- | --- | --- | --- | --- | --- | --- | --- | --- |

tr|A0A2A4K0C6|A0A2A4K0C6\_HELVI

back to list

  

| Protein Coverage
| Supporting Peptides
|

Protein Coverage:

Supporting Peptides:

| Peptide | Uniq | -10lgP | Mass | ppm | m/z | z | RT | Scan | #Spec | Start | End | PTM |
| --- | --- | --- | --- | --- | --- | --- | --- | --- | --- | --- | --- | --- |
| V.LPDGEHDLDR.A | Y | 33.64 | 1165.5364 | -0.2 | 583.7753 | 2 | 10.20 | 1369 | 1 | 192 | 201 |  |
| V.LPDGEHDLDRA.Q | Y | 22.49 | 1236.5735 | -0.7 | 619.2936 | 2 | 10.49 | 1422 | 2 | 192 | 202 |  |
| E.VLPDGEHDLDRA.Q | Y | 16.15 | 1335.6418 | -0.7 | 668.8277 | 2 | 10.73 | 1466 | 1 | 191 | 202 |  |
| total 3 peptides |  |  |  |  |  |  |  |  |  |  |  |  |
| --- | --- | --- | --- | --- | --- | --- | --- | --- | --- | --- | --- | --- |

tr|A0A6J1NBL3|A0A6J1NBL3\_BICAN

back to list

  

| Protein Coverage
| Supporting Peptides
|

Protein Coverage:

Supporting Peptides:

| Peptide | Uniq | -10lgP | Mass | ppm | m/z | z | RT | Scan | #Spec | Start | End | PTM |
| --- | --- | --- | --- | --- | --- | --- | --- | --- | --- | --- | --- | --- |
| V.LPDGEHDLDR.A | Y | 33.64 | 1165.5364 | -0.2 | 583.7753 | 2 | 10.20 | 1369 | 1 | 192 | 201 |  |
| V.LPDGEHDLDRA.Q | Y | 22.49 | 1236.5735 | -0.7 | 619.2936 | 2 | 10.49 | 1422 | 2 | 192 | 202 |  |
| E.VLPDGEHDLDRA.Q | Y | 16.15 | 1335.6418 | -0.7 | 668.8277 | 2 | 10.73 | 1466 | 1 | 191 | 202 |  |
| total 3 peptides |  |  |  |  |  |  |  |  |  |  |  |  |
| --- | --- | --- | --- | --- | --- | --- | --- | --- | --- | --- | --- | --- |

tr|A0A6J2KNU6|A0A6J2KNU6\_BOMMA

back to list

  

| Protein Coverage
| Supporting Peptides
|

Protein Coverage:

Supporting Peptides:

| Peptide | Uniq | -10lgP | Mass | ppm | m/z | z | RT | Scan | #Spec | Start | End | PTM |
| --- | --- | --- | --- | --- | --- | --- | --- | --- | --- | --- | --- | --- |
| V.LPDGEHDLDR.A | Y | 33.64 | 1165.5364 | -0.2 | 583.7753 | 2 | 10.20 | 1369 | 1 | 192 | 201 |  |
| V.LPDGEHDLDRA.Q | Y | 22.49 | 1236.5735 | -0.7 | 619.2936 | 2 | 10.49 | 1422 | 2 | 192 | 202 |  |
| E.VLPDGEHDLDRA.Q | Y | 16.15 | 1335.6418 | -0.7 | 668.8277 | 2 | 10.73 | 1466 | 1 | 191 | 202 |  |
| total 3 peptides |  |  |  |  |  |  |  |  |  |  |  |  |
| --- | --- | --- | --- | --- | --- | --- | --- | --- | --- | --- | --- | --- |

tr|A0A6J2KQG2|A0A6J2KQG2\_BOMMA

back to list

  

| Protein Coverage
| Supporting Peptides
|

Protein Coverage:

Supporting Peptides:

| Peptide | Uniq | -10lgP | Mass | ppm | m/z | z | RT | Scan | #Spec | Start | End | PTM |
| --- | --- | --- | --- | --- | --- | --- | --- | --- | --- | --- | --- | --- |
| V.LPDGEHDLDR.A | Y | 33.64 | 1165.5364 | -0.2 | 583.7753 | 2 | 10.20 | 1369 | 1 | 192 | 201 |  |
| V.LPDGEHDLDRA.Q | Y | 22.49 | 1236.5735 | -0.7 | 619.2936 | 2 | 10.49 | 1422 | 2 | 192 | 202 |  |
| E.VLPDGEHDLDRA.Q | Y | 16.15 | 1335.6418 | -0.7 | 668.8277 | 2 | 10.73 | 1466 | 1 | 191 | 202 |  |
| total 3 peptides |  |  |  |  |  |  |  |  |  |  |  |  |
| --- | --- | --- | --- | --- | --- | --- | --- | --- | --- | --- | --- | --- |

tr|Q1HPN7|Q1HPN7\_BOMMO

back to list

  

| Protein Coverage
| Supporting Peptides
|

Protein Coverage:

Supporting Peptides:

| Peptide | Uniq | -10lgP | Mass | ppm | m/z | z | RT | Scan | #Spec | Start | End | PTM |
| --- | --- | --- | --- | --- | --- | --- | --- | --- | --- | --- | --- | --- |
| V.LPDGEHDLDR.A | Y | 33.64 | 1165.5364 | -0.2 | 583.7753 | 2 | 10.20 | 1369 | 1 | 192 | 201 |  |
| V.LPDGEHDLDRA.Q | Y | 22.49 | 1236.5735 | -0.7 | 619.2936 | 2 | 10.49 | 1422 | 2 | 192 | 202 |  |
| E.VLPDGEHDLDRA.Q | Y | 16.15 | 1335.6418 | -0.7 | 668.8277 | 2 | 10.73 | 1466 | 1 | 191 | 202 |  |
| total 3 peptides |  |  |  |  |  |  |  |  |  |  |  |  |
| --- | --- | --- | --- | --- | --- | --- | --- | --- | --- | --- | --- | --- |

tr|S4PKR2|S4PKR2\_9NEOP

back to list

  

| Protein Coverage
| Supporting Peptides
|

Protein Coverage:

Supporting Peptides:

| Peptide | Uniq | -10lgP | Mass | ppm | m/z | z | RT | Scan | #Spec | Start | End | PTM |
| --- | --- | --- | --- | --- | --- | --- | --- | --- | --- | --- | --- | --- |
| V.LPDGEHDLDR.A | Y | 33.64 | 1165.5364 | -0.2 | 583.7753 | 2 | 10.20 | 1369 | 1 | 192 | 201 |  |
| V.LPDGEHDLDRA.Q | Y | 22.49 | 1236.5735 | -0.7 | 619.2936 | 2 | 10.49 | 1422 | 2 | 192 | 202 |  |
| E.VLPDGEHDLDRA.Q | Y | 16.15 | 1335.6418 | -0.7 | 668.8277 | 2 | 10.73 | 1466 | 1 | 191 | 202 |  |
| total 3 peptides |  |  |  |  |  |  |  |  |  |  |  |  |
| --- | --- | --- | --- | --- | --- | --- | --- | --- | --- | --- | --- | --- |

tr|A0A7S5LJX7|A0A7S5LJX7\_BEMTA

back to list

  

| Protein Coverage
| Supporting Peptides
|

Protein Coverage:

Supporting Peptides:

| Peptide | Uniq | -10lgP | Mass | ppm | m/z | z | RT | Scan | #Spec | Start | End | PTM |
| --- | --- | --- | --- | --- | --- | --- | --- | --- | --- | --- | --- | --- |
| V.LPDGEHDLDR.A | Y | 33.64 | 1165.5364 | -0.2 | 583.7753 | 2 | 10.20 | 1369 | 1 | 192 | 201 |  |
| V.LPDGEHDLDRA.Q | Y | 22.49 | 1236.5735 | -0.7 | 619.2936 | 2 | 10.49 | 1422 | 2 | 192 | 202 |  |
| E.VLPDGEHDLDRA.Q | Y | 16.15 | 1335.6418 | -0.7 | 668.8277 | 2 | 10.73 | 1466 | 1 | 191 | 202 |  |
| total 3 peptides |  |  |  |  |  |  |  |  |  |  |  |  |
| --- | --- | --- | --- | --- | --- | --- | --- | --- | --- | --- | --- | --- |

tr|A0A7R9X220|A0A7R9X220\_9CRUS

back to list

  

| Protein Coverage
| Supporting Peptides
|

Protein Coverage:

Supporting Peptides:

| Peptide | Uniq | -10lgP | Mass | ppm | m/z | z | RT | Scan | #Spec | Start | End | PTM |
| --- | --- | --- | --- | --- | --- | --- | --- | --- | --- | --- | --- | --- |
| V.LPDGEHDLDR.A | Y | 33.64 | 1165.5364 | -0.2 | 583.7753 | 2 | 10.20 | 1369 | 1 | 195 | 204 |  |
| V.LPDGEHDLDRA.Q | Y | 22.49 | 1236.5735 | -0.7 | 619.2936 | 2 | 10.49 | 1422 | 2 | 195 | 205 |  |
| E.VLPDGEHDLDRA.Q | Y | 16.15 | 1335.6418 | -0.7 | 668.8277 | 2 | 10.73 | 1466 | 1 | 194 | 205 |  |
| total 3 peptides |  |  |  |  |  |  |  |  |  |  |  |  |
| --- | --- | --- | --- | --- | --- | --- | --- | --- | --- | --- | --- | --- |

tr|A0A7R9A8N4|A0A7R9A8N4\_9CRUS

back to list

  

| Protein Coverage
| Supporting Peptides
|

Protein Coverage:

Supporting Peptides:

| Peptide | Uniq | -10lgP | Mass | ppm | m/z | z | RT | Scan | #Spec | Start | End | PTM |
| --- | --- | --- | --- | --- | --- | --- | --- | --- | --- | --- | --- | --- |
| V.LPDGEHDLDR.A | Y | 33.64 | 1165.5364 | -0.2 | 583.7753 | 2 | 10.20 | 1369 | 1 | 199 | 208 |  |
| V.LPDGEHDLDRA.Q | Y | 22.49 | 1236.5735 | -0.7 | 619.2936 | 2 | 10.49 | 1422 | 2 | 199 | 209 |  |
| E.VLPDGEHDLDRA.Q | Y | 16.15 | 1335.6418 | -0.7 | 668.8277 | 2 | 10.73 | 1466 | 1 | 198 | 209 |  |
| total 3 peptides |  |  |  |  |  |  |  |  |  |  |  |  |
| --- | --- | --- | --- | --- | --- | --- | --- | --- | --- | --- | --- | --- |

tr|A0A7R9A8M7|A0A7R9A8M7\_9CRUS

back to list

  

| Protein Coverage
| Supporting Peptides
|

Protein Coverage:

Supporting Peptides:

| Peptide | Uniq | -10lgP | Mass | ppm | m/z | z | RT | Scan | #Spec | Start | End | PTM |
| --- | --- | --- | --- | --- | --- | --- | --- | --- | --- | --- | --- | --- |
| V.LPDGEHDLDR.A | Y | 33.64 | 1165.5364 | -0.2 | 583.7753 | 2 | 10.20 | 1369 | 1 | 199 | 208 |  |
| V.LPDGEHDLDRA.Q | Y | 22.49 | 1236.5735 | -0.7 | 619.2936 | 2 | 10.49 | 1422 | 2 | 199 | 209 |  |
| E.VLPDGEHDLDRA.Q | Y | 16.15 | 1335.6418 | -0.7 | 668.8277 | 2 | 10.73 | 1466 | 1 | 198 | 209 |  |
| total 3 peptides |  |  |  |  |  |  |  |  |  |  |  |  |
| --- | --- | --- | --- | --- | --- | --- | --- | --- | --- | --- | --- | --- |

tr|A0A4C1UD71|A0A4C1UD71\_EUMVA

back to list

  

| Protein Coverage
| Supporting Peptides
|

Protein Coverage:

Supporting Peptides:

| Peptide | Uniq | -10lgP | Mass | ppm | m/z | z | RT | Scan | #Spec | Start | End | PTM |
| --- | --- | --- | --- | --- | --- | --- | --- | --- | --- | --- | --- | --- |
| V.LPDGEHDLDR.A | Y | 33.64 | 1165.5364 | -0.2 | 583.7753 | 2 | 10.20 | 1369 | 1 | 212 | 221 |  |
| V.LPDGEHDLDRA.Q | Y | 22.49 | 1236.5735 | -0.7 | 619.2936 | 2 | 10.49 | 1422 | 2 | 212 | 222 |  |
| E.VLPDGEHDLDRA.Q | Y | 16.15 | 1335.6418 | -0.7 | 668.8277 | 2 | 10.73 | 1466 | 1 | 211 | 222 |  |
| total 3 peptides |  |  |  |  |  |  |  |  |  |  |  |  |
| --- | --- | --- | --- | --- | --- | --- | --- | --- | --- | --- | --- | --- |

tr|A0A6J2KM35|A0A6J2KM35\_BOMMA

back to list

  

| Protein Coverage
| Supporting Peptides
|

Protein Coverage:

Supporting Peptides:

| Peptide | Uniq | -10lgP | Mass | ppm | m/z | z | RT | Scan | #Spec | Start | End | PTM |
| --- | --- | --- | --- | --- | --- | --- | --- | --- | --- | --- | --- | --- |
| V.LPDGEHDLDR.A | Y | 33.64 | 1165.5364 | -0.2 | 583.7753 | 2 | 10.20 | 1369 | 1 | 192 | 201 |  |
| V.LPDGEHDLDRA.Q | Y | 22.49 | 1236.5735 | -0.7 | 619.2936 | 2 | 10.49 | 1422 | 2 | 192 | 202 |  |
| E.VLPDGEHDLDRA.Q | Y | 16.15 | 1335.6418 | -0.7 | 668.8277 | 2 | 10.73 | 1466 | 1 | 191 | 202 |  |
| total 3 peptides |  |  |  |  |  |  |  |  |  |  |  |  |
| --- | --- | --- | --- | --- | --- | --- | --- | --- | --- | --- | --- | --- |

tr|A0A821PCJ9|A0A821PCJ9\_9NEOP

back to list

  

| Protein Coverage
| Supporting Peptides
|

Protein Coverage:

Supporting Peptides:

| Peptide | Uniq | -10lgP | Mass | ppm | m/z | z | RT | Scan | #Spec | Start | End | PTM |
| --- | --- | --- | --- | --- | --- | --- | --- | --- | --- | --- | --- | --- |
| V.LPDGEHDLDR.A | Y | 33.64 | 1165.5364 | -0.2 | 583.7753 | 2 | 10.20 | 1369 | 1 | 218 | 227 |  |
| V.LPDGEHDLDRA.Q | Y | 22.49 | 1236.5735 | -0.7 | 619.2936 | 2 | 10.49 | 1422 | 2 | 218 | 228 |  |
| E.VLPDGEHDLDRA.Q | Y | 16.15 | 1335.6418 | -0.7 | 668.8277 | 2 | 10.73 | 1466 | 1 | 217 | 228 |  |
| total 3 peptides |  |  |  |  |  |  |  |  |  |  |  |  |
| --- | --- | --- | --- | --- | --- | --- | --- | --- | --- | --- | --- | --- |

tr|A0A7E5VF38|A0A7E5VF38\_TRINI

back to list

  

| Protein Coverage
| Supporting Peptides
|

Protein Coverage:

Supporting Peptides:

| Peptide | Uniq | -10lgP | Mass | ppm | m/z | z | RT | Scan | #Spec | Start | End | PTM |
| --- | --- | --- | --- | --- | --- | --- | --- | --- | --- | --- | --- | --- |
| V.LPDGEHDLDR.A | Y | 33.64 | 1165.5364 | -0.2 | 583.7753 | 2 | 10.20 | 1369 | 1 | 220 | 229 |  |
| V.LPDGEHDLDRA.Q | Y | 22.49 | 1236.5735 | -0.7 | 619.2936 | 2 | 10.49 | 1422 | 2 | 220 | 230 |  |
| E.VLPDGEHDLDRA.Q | Y | 16.15 | 1335.6418 | -0.7 | 668.8277 | 2 | 10.73 | 1466 | 1 | 219 | 230 |  |
| total 3 peptides |  |  |  |  |  |  |  |  |  |  |  |  |
| --- | --- | --- | --- | --- | --- | --- | --- | --- | --- | --- | --- | --- |

tr|A0A7I1A027|A0A7I1A027\_DANPL

back to list

  

| Protein Coverage
| Supporting Peptides
|

Protein Coverage:

Supporting Peptides:

| Peptide | Uniq | -10lgP | Mass | ppm | m/z | z | RT | Scan | #Spec | Start | End | PTM |
| --- | --- | --- | --- | --- | --- | --- | --- | --- | --- | --- | --- | --- |
| V.LPDGEHDLDR.A | Y | 33.64 | 1165.5364 | -0.2 | 583.7753 | 2 | 10.20 | 1369 | 1 | 218 | 227 |  |
| V.LPDGEHDLDRA.Q | Y | 22.49 | 1236.5735 | -0.7 | 619.2936 | 2 | 10.49 | 1422 | 2 | 218 | 228 |  |
| E.VLPDGEHDLDRA.Q | Y | 16.15 | 1335.6418 | -0.7 | 668.8277 | 2 | 10.73 | 1466 | 1 | 217 | 228 |  |
| total 3 peptides |  |  |  |  |  |  |  |  |  |  |  |  |
| --- | --- | --- | --- | --- | --- | --- | --- | --- | --- | --- | --- | --- |

tr|A0A821PCK8|A0A821PCK8\_9NEOP

back to list

  

| Protein Coverage
| Supporting Peptides
|

Protein Coverage:

Supporting Peptides:

| Peptide | Uniq | -10lgP | Mass | ppm | m/z | z | RT | Scan | #Spec | Start | End | PTM |
| --- | --- | --- | --- | --- | --- | --- | --- | --- | --- | --- | --- | --- |
| V.LPDGEHDLDR.A | Y | 33.64 | 1165.5364 | -0.2 | 583.7753 | 2 | 10.20 | 1369 | 1 | 218 | 227 |  |
| V.LPDGEHDLDRA.Q | Y | 22.49 | 1236.5735 | -0.7 | 619.2936 | 2 | 10.49 | 1422 | 2 | 218 | 228 |  |
| E.VLPDGEHDLDRA.Q | Y | 16.15 | 1335.6418 | -0.7 | 668.8277 | 2 | 10.73 | 1466 | 1 | 217 | 228 |  |
| total 3 peptides |  |  |  |  |  |  |  |  |  |  |  |  |
| --- | --- | --- | --- | --- | --- | --- | --- | --- | --- | --- | --- | --- |

tr|A0A811X398|A0A811X398\_PLUXY

back to list

  

| Protein Coverage
| Supporting Peptides
|

Protein Coverage:

Supporting Peptides:

| Peptide | Uniq | -10lgP | Mass | ppm | m/z | z | RT | Scan | #Spec | Start | End | PTM |
| --- | --- | --- | --- | --- | --- | --- | --- | --- | --- | --- | --- | --- |
| V.LPDGEHDLDR.A | Y | 33.64 | 1165.5364 | -0.2 | 583.7753 | 2 | 10.20 | 1369 | 1 | 221 | 230 |  |
| V.LPDGEHDLDRA.Q | Y | 22.49 | 1236.5735 | -0.7 | 619.2936 | 2 | 10.49 | 1422 | 2 | 221 | 231 |  |
| E.VLPDGEHDLDRA.Q | Y | 16.15 | 1335.6418 | -0.7 | 668.8277 | 2 | 10.73 | 1466 | 1 | 220 | 231 |  |
| total 3 peptides |  |  |  |  |  |  |  |  |  |  |  |  |
| --- | --- | --- | --- | --- | --- | --- | --- | --- | --- | --- | --- | --- |

tr|A0A2P8YG29|A0A2P8YG29\_BLAGE

back to list

  

| Protein Coverage
| Supporting Peptides
|

Protein Coverage:

Supporting Peptides:

| Peptide | Uniq | -10lgP | Mass | ppm | m/z | z | RT | Scan | #Spec | Start | End | PTM |
| --- | --- | --- | --- | --- | --- | --- | --- | --- | --- | --- | --- | --- |
| V.LPDGEHDLDR.A | Y | 33.64 | 1165.5364 | -0.2 | 583.7753 | 2 | 10.20 | 1369 | 1 | 65 | 74 |  |
| V.LPDGEHDLDRA.Q | Y | 22.49 | 1236.5735 | -0.7 | 619.2936 | 2 | 10.49 | 1422 | 2 | 65 | 75 |  |
| E.VLPDGEHDLDRA.Q | Y | 16.15 | 1335.6418 | -0.7 | 668.8277 | 2 | 10.73 | 1466 | 1 | 64 | 75 |  |
| total 3 peptides |  |  |  |  |  |  |  |  |  |  |  |  |
| --- | --- | --- | --- | --- | --- | --- | --- | --- | --- | --- | --- | --- |

tr|A0A5B7DQZ0|A0A5B7DQZ0\_PORTR

back to list

  

| Protein Coverage
| Supporting Peptides
|

Protein Coverage:

Supporting Peptides:

| Peptide | Uniq | -10lgP | Mass | ppm | m/z | z | RT | Scan | #Spec | Start | End | PTM |
| --- | --- | --- | --- | --- | --- | --- | --- | --- | --- | --- | --- | --- |
| L.SILEEESMFPK.A | N | 26.45 | 1308.6271 | -0.7 | 655.3204 | 2 | 12.91 | 1869 | 1 | 403 | 413 |  |
| K.NKDPLNDTL.V | N | 20.74 | 1028.5138 | -0.9 | 515.2637 | 2 | 11.46 | 1603 | 1 | 469 | 477 |  |
| R.NEVPPHLF.A | N | 16.25 | 951.4814 | -0.3 | 476.7478 | 2 | 13.07 | 1899 | 1 | 150 | 157 |  |
| L.FDRVF.K | N | 15.90 | 682.3438 | -0.9 | 342.1789 | 2 | 11.93 | 1692 | 1 | 303 | 307 |  |
| L.FDRVFKF.I | N | 15.27 | 957.5072 | -1.0 | 479.7604 | 2 | 12.36 | 1771 | 1 | 303 | 309 |  |
| total 5 peptides |  |  |  |  |  |  |  |  |  |  |  |  |
| --- | --- | --- | --- | --- | --- | --- | --- | --- | --- | --- | --- | --- |

tr|A0A821V8C5|A0A821V8C5\_9NEOP

back to list

  

| Protein Coverage
| Supporting Peptides
|

Protein Coverage:

Supporting Peptides:

| Peptide | Uniq | -10lgP | Mass | ppm | m/z | z | RT | Scan | #Spec | Start | End | PTM |
| --- | --- | --- | --- | --- | --- | --- | --- | --- | --- | --- | --- | --- |
| E.VIVVDKPGPPKGPL.S | Y | 38.50 | 1414.8547 | -26.0 | 708.4163 | 2 | 12.03 | 1712 | 1 | 4803 | 4816 |  |
| V.VIKIPF.T | N | 17.57 | 715.4632 | 0.5 | 358.7390 | 2 | 13.33 | 1944 | 1 | 6310 | 6315 |  |
| total 2 peptides |  |  |  |  |  |  |  |  |  |  |  |  |
| --- | --- | --- | --- | --- | --- | --- | --- | --- | --- | --- | --- | --- |

tr|A0A7J7AR02|A0A7J7AR02\_9COLE

back to list

  

| Protein Coverage
| Supporting Peptides
|

Protein Coverage:

Supporting Peptides:

| Peptide | Uniq | -10lgP | Mass | ppm | m/z | z | RT | Scan | #Spec | Start | End | PTM |
| --- | --- | --- | --- | --- | --- | --- | --- | --- | --- | --- | --- | --- |
| L.DTFSLPGK.L | Y | 29.05 | 863.4388 | -0.6 | 432.7264 | 2 | 11.48 | 1607 | 1 | 290 | 297 |  |
| L.DTFSLPGKLS.D | Y | 27.50 | 1063.5549 | -0.1 | 532.7847 | 2 | 11.99 | 1704 | 1 | 290 | 299 |  |
| total 2 peptides |  |  |  |  |  |  |  |  |  |  |  |  |
| --- | --- | --- | --- | --- | --- | --- | --- | --- | --- | --- | --- | --- |

tr|A0A444T9K0|A0A444T9K0\_ARMVU

back to list

  

| Protein Coverage
| Supporting Peptides
|

Protein Coverage:

Supporting Peptides:

| Peptide | Uniq | -10lgP | Mass | ppm | m/z | z | RT | Scan | #Spec | Start | End | PTM |
| --- | --- | --- | --- | --- | --- | --- | --- | --- | --- | --- | --- | --- |
| K.APYDPPSPPGIPK.V | Y | 30.43 | 1334.6870 | -0.5 | 668.3505 | 2 | 11.62 | 1633 | 1 | 4704 | 4716 |  |
| K.IKDPNAATPPEII.S | N | 15.97 | 1377.7504 | -1.0 | 689.8818 | 2 | 12.19 | 1742 | 1 | 4803 | 4815 |  |
| total 2 peptides |  |  |  |  |  |  |  |  |  |  |  |  |
| --- | --- | --- | --- | --- | --- | --- | --- | --- | --- | --- | --- | --- |

tr|A0A2P1JJ42|A0A2P1JJ42\_PROCL

back to list

  

| Protein Coverage
| Supporting Peptides
|

Protein Coverage:

Supporting Peptides:

| Peptide | Uniq | -10lgP | Mass | ppm | m/z | z | RT | Scan | #Spec | Start | End | PTM |
| --- | --- | --- | --- | --- | --- | --- | --- | --- | --- | --- | --- | --- |
| G.IHIPGSPF.R | Y | 25.89 | 866.4650 | -1.2 | 434.2393 | 2 | 13.11 | 1905 | 1 | 1331 | 1338 |  |
| F.KLPGISPF.D | Y | 15.46 | 857.5010 | -0.2 | 429.7577 | 2 | 13.01 | 1888 | 1 | 1087 | 1094 |  |
| total 2 peptides |  |  |  |  |  |  |  |  |  |  |  |  |
| --- | --- | --- | --- | --- | --- | --- | --- | --- | --- | --- | --- | --- |

tr|A0A0N9EJJ5|A0A0N9EJJ5\_ERIVE

back to list

  

| Protein Coverage
| Supporting Peptides
|

Protein Coverage:

Supporting Peptides:

| Peptide | Uniq | -10lgP | Mass | ppm | m/z | z | RT | Scan | #Spec | Start | End | PTM |
| --- | --- | --- | --- | --- | --- | --- | --- | --- | --- | --- | --- | --- |
| R.VFAWPK.Y | Y | 21.14 | 746.4115 | -0.8 | 374.2127 | 2 | 12.32 | 1763 | 1 | 502 | 507 |  |
| H.VVLPPLYE.V | Y | 17.78 | 928.5269 | 0.0 | 465.2708 | 2 | 13.82 | 2031 | 1 | 153 | 160 |  |
| total 2 peptides |  |  |  |  |  |  |  |  |  |  |  |  |
| --- | --- | --- | --- | --- | --- | --- | --- | --- | --- | --- | --- | --- |

tr|A0A1J1IN48|A0A1J1IN48\_9DIPT

back to list

  

| Protein Coverage
| Supporting Peptides
|

Protein Coverage:

Supporting Peptides:

| Peptide | Uniq | -10lgP | Mass | ppm | m/z | z | RT | Scan | #Spec | Start | End | PTM |
| --- | --- | --- | --- | --- | --- | --- | --- | --- | --- | --- | --- | --- |
| M.KVPCANAL.K | Y | 25.85 | 814.4371 | -3.7 | 408.2243 | 2 | 11.33 | 1580 | 1 | 1614 | 1621 |  |
| total 1 peptides |  |  |  |  |  |  |  |  |  |  |  |  |
| --- | --- | --- | --- | --- | --- | --- | --- | --- | --- | --- | --- | --- |

tr|A0A1E1XMV5|A0A1E1XMV5\_9ACAR

back to list

  

| Protein Coverage
| Supporting Peptides
|

Protein Coverage:

Supporting Peptides:

| Peptide | Uniq | -10lgP | Mass | ppm | m/z | z | RT | Scan | #Spec | Start | End | PTM |
| --- | --- | --- | --- | --- | --- | --- | --- | --- | --- | --- | --- | --- |
| V.KVPCANAI.N | Y | 25.85 | 814.4371 | -3.7 | 408.2243 | 2 | 11.33 | 1580 | 1 | 2 | 9 |  |
| total 1 peptides |  |  |  |  |  |  |  |  |  |  |  |  |
| --- | --- | --- | --- | --- | --- | --- | --- | --- | --- | --- | --- | --- |

tr|B4J4N0|B4J4N0\_DROGR

back to list

  

| Protein Coverage
| Supporting Peptides
|

Protein Coverage:

Supporting Peptides:

| Peptide | Uniq | -10lgP | Mass | ppm | m/z | z | RT | Scan | #Spec | Start | End | PTM |
| --- | --- | --- | --- | --- | --- | --- | --- | --- | --- | --- | --- | --- |
| G.RVKVPVVP.H | Y | 25.31 | 892.5858 | 1.2 | 447.3007 | 2 | 15.88 | 2392 | 1 | 70 | 77 |  |
| total 1 peptides |  |  |  |  |  |  |  |  |  |  |  |  |
| --- | --- | --- | --- | --- | --- | --- | --- | --- | --- | --- | --- | --- |

tr|A0A4S2KBN7|A0A4S2KBN7\_9HYME

back to list

  

| Protein Coverage
| Supporting Peptides
|

Protein Coverage:

Supporting Peptides:

| Peptide | Uniq | -10lgP | Mass | ppm | m/z | z | RT | Scan | #Spec | Start | End | PTM |
| --- | --- | --- | --- | --- | --- | --- | --- | --- | --- | --- | --- | --- |
| D.EDSIKFI.V | Y | 25.31 | 850.4436 | 0.0 | 426.2291 | 2 | 13.21 | 1923 | 1 | 1873 | 1879 |  |
| total 1 peptides |  |  |  |  |  |  |  |  |  |  |  |  |
| --- | --- | --- | --- | --- | --- | --- | --- | --- | --- | --- | --- | --- |

tr|A0A2P8XAH3|A0A2P8XAH3\_BLAGE

back to list

  

| Protein Coverage
| Supporting Peptides
|

Protein Coverage:

Supporting Peptides:

| Peptide | Uniq | -10lgP | Mass | ppm | m/z | z | RT | Scan | #Spec | Start | End | PTM |
| --- | --- | --- | --- | --- | --- | --- | --- | --- | --- | --- | --- | --- |
| I.AELHKNIL.V | Y | 23.47 | 936.5392 | -12.2 | 469.2712 | 2 | 13.35 | 1948 | 1 | 39 | 46 |  |
| total 1 peptides |  |  |  |  |  |  |  |  |  |  |  |  |
| --- | --- | --- | --- | --- | --- | --- | --- | --- | --- | --- | --- | --- |

tr|R9YY56|R9YY56\_9EUCA

back to list

  

| Protein Coverage
| Supporting Peptides
|

Protein Coverage:

Supporting Peptides:

| Peptide | Uniq | -10lgP | Mass | ppm | m/z | z | RT | Scan | #Spec | Start | End | PTM |
| --- | --- | --- | --- | --- | --- | --- | --- | --- | --- | --- | --- | --- |
| F.VNVDPDGKFVIS.T | Y | 21.53 | 1288.6663 | -0.6 | 645.3400 | 2 | 12.28 | 1756 | 1 | 7 | 18 |  |
| total 1 peptides |  |  |  |  |  |  |  |  |  |  |  |  |
| --- | --- | --- | --- | --- | --- | --- | --- | --- | --- | --- | --- | --- |

tr|R9YY47|R9YY47\_9EUCA

back to list

  

| Protein Coverage
| Supporting Peptides
|

Protein Coverage:

Supporting Peptides:

| Peptide | Uniq | -10lgP | Mass | ppm | m/z | z | RT | Scan | #Spec | Start | End | PTM |
| --- | --- | --- | --- | --- | --- | --- | --- | --- | --- | --- | --- | --- |
| F.VNVDPDGKFVIS.T | Y | 21.53 | 1288.6663 | -0.6 | 645.3400 | 2 | 12.28 | 1756 | 1 | 8 | 19 |  |
| total 1 peptides |  |  |  |  |  |  |  |  |  |  |  |  |
| --- | --- | --- | --- | --- | --- | --- | --- | --- | --- | --- | --- | --- |

tr|G3C6J8|G3C6J8\_9EUCA

back to list

  

| Protein Coverage
| Supporting Peptides
|

Protein Coverage:

Supporting Peptides:

| Peptide | Uniq | -10lgP | Mass | ppm | m/z | z | RT | Scan | #Spec | Start | End | PTM |
| --- | --- | --- | --- | --- | --- | --- | --- | --- | --- | --- | --- | --- |
| F.VNVDPDGKFVIS.T | Y | 21.53 | 1288.6663 | -0.6 | 645.3400 | 2 | 12.28 | 1756 | 1 | 19 | 30 |  |
| total 1 peptides |  |  |  |  |  |  |  |  |  |  |  |  |
| --- | --- | --- | --- | --- | --- | --- | --- | --- | --- | --- | --- | --- |

tr|G3C6I8|G3C6I8\_AEGAL

back to list

  

| Protein Coverage
| Supporting Peptides
|

Protein Coverage:

Supporting Peptides:

| Peptide | Uniq | -10lgP | Mass | ppm | m/z | z | RT | Scan | #Spec | Start | End | PTM |
| --- | --- | --- | --- | --- | --- | --- | --- | --- | --- | --- | --- | --- |
| F.VNVDPDGKFVIS.T | Y | 21.53 | 1288.6663 | -0.6 | 645.3400 | 2 | 12.28 | 1756 | 1 | 19 | 30 |  |
| total 1 peptides |  |  |  |  |  |  |  |  |  |  |  |  |
| --- | --- | --- | --- | --- | --- | --- | --- | --- | --- | --- | --- | --- |

tr|G3C6N2|G3C6N2\_9EUCA

back to list

  

| Protein Coverage
| Supporting Peptides
|

Protein Coverage:

Supporting Peptides:

| Peptide | Uniq | -10lgP | Mass | ppm | m/z | z | RT | Scan | #Spec | Start | End | PTM |
| --- | --- | --- | --- | --- | --- | --- | --- | --- | --- | --- | --- | --- |
| F.VNVDPDGKFVIS.T | Y | 21.53 | 1288.6663 | -0.6 | 645.3400 | 2 | 12.28 | 1756 | 1 | 19 | 30 |  |
| total 1 peptides |  |  |  |  |  |  |  |  |  |  |  |  |
| --- | --- | --- | --- | --- | --- | --- | --- | --- | --- | --- | --- | --- |

P86699|KARG\_CHIOP

back to list

  

| Protein Coverage
| Supporting Peptides
|

Protein Coverage:

Supporting Peptides:

| Peptide | Uniq | -10lgP | Mass | ppm | m/z | z | RT | Scan | #Spec | Start | End | PTM |
| --- | --- | --- | --- | --- | --- | --- | --- | --- | --- | --- | --- | --- |
| F.VNVDPDGKFVIS.T | Y | 21.53 | 1288.6663 | -0.6 | 645.3400 | 2 | 12.28 | 1756 | 1 | 48 | 59 |  |
| total 1 peptides |  |  |  |  |  |  |  |  |  |  |  |  |
| --- | --- | --- | --- | --- | --- | --- | --- | --- | --- | --- | --- | --- |

tr|G3D680|G3D680\_PORTR

back to list

  

| Protein Coverage
| Supporting Peptides
|

Protein Coverage:

Supporting Peptides:

| Peptide | Uniq | -10lgP | Mass | ppm | m/z | z | RT | Scan | #Spec | Start | End | PTM |
| --- | --- | --- | --- | --- | --- | --- | --- | --- | --- | --- | --- | --- |
| F.VNVDPDGKFVIS.T | Y | 21.53 | 1288.6663 | -0.6 | 645.3400 | 2 | 12.28 | 1756 | 1 | 111 | 122 |  |
| total 1 peptides |  |  |  |  |  |  |  |  |  |  |  |  |
| --- | --- | --- | --- | --- | --- | --- | --- | --- | --- | --- | --- | --- |

tr|I1TMM2|I1TMM2\_SCYPA

back to list

  

| Protein Coverage
| Supporting Peptides
|

Protein Coverage:

Supporting Peptides:

| Peptide | Uniq | -10lgP | Mass | ppm | m/z | z | RT | Scan | #Spec | Start | End | PTM |
| --- | --- | --- | --- | --- | --- | --- | --- | --- | --- | --- | --- | --- |
| F.VNVDPDGKFVIS.T | Y | 21.53 | 1288.6663 | -0.6 | 645.3400 | 2 | 12.28 | 1756 | 1 | 107 | 118 |  |
| total 1 peptides |  |  |  |  |  |  |  |  |  |  |  |  |
| --- | --- | --- | --- | --- | --- | --- | --- | --- | --- | --- | --- | --- |

tr|K7PD48|K7PD48\_SCYPA

back to list

  

| Protein Coverage
| Supporting Peptides
|

Protein Coverage:

Supporting Peptides:

| Peptide | Uniq | -10lgP | Mass | ppm | m/z | z | RT | Scan | #Spec | Start | End | PTM |
| --- | --- | --- | --- | --- | --- | --- | --- | --- | --- | --- | --- | --- |
| F.VNVDPDGKFVIS.T | Y | 21.53 | 1288.6663 | -0.6 | 645.3400 | 2 | 12.28 | 1756 | 1 | 110 | 121 |  |
| total 1 peptides |  |  |  |  |  |  |  |  |  |  |  |  |
| --- | --- | --- | --- | --- | --- | --- | --- | --- | --- | --- | --- | --- |

C9EIP1|KARG\_SCYSE

back to list

  

| Protein Coverage
| Supporting Peptides
|

Protein Coverage:

Supporting Peptides:

| Peptide | Uniq | -10lgP | Mass | ppm | m/z | z | RT | Scan | #Spec | Start | End | PTM |
| --- | --- | --- | --- | --- | --- | --- | --- | --- | --- | --- | --- | --- |
| F.VNVDPDGKFVIS.T | Y | 21.53 | 1288.6663 | -0.6 | 645.3400 | 2 | 12.28 | 1756 | 1 | 111 | 122 |  |
| total 1 peptides |  |  |  |  |  |  |  |  |  |  |  |  |
| --- | --- | --- | --- | --- | --- | --- | --- | --- | --- | --- | --- | --- |

tr|G3D692|G3D692\_PORTR

back to list

  

| Protein Coverage
| Supporting Peptides
|

Protein Coverage:

Supporting Peptides:

| Peptide | Uniq | -10lgP | Mass | ppm | m/z | z | RT | Scan | #Spec | Start | End | PTM |
| --- | --- | --- | --- | --- | --- | --- | --- | --- | --- | --- | --- | --- |
| F.VNVDPDGKFVIS.T | Y | 21.53 | 1288.6663 | -0.6 | 645.3400 | 2 | 12.28 | 1756 | 1 | 111 | 122 |  |
| total 1 peptides |  |  |  |  |  |  |  |  |  |  |  |  |
| --- | --- | --- | --- | --- | --- | --- | --- | --- | --- | --- | --- | --- |

tr|H6AC60|H6AC60\_PORTR

back to list

  

| Protein Coverage
| Supporting Peptides
|

Protein Coverage:

Supporting Peptides:

| Peptide | Uniq | -10lgP | Mass | ppm | m/z | z | RT | Scan | #Spec | Start | End | PTM |
| --- | --- | --- | --- | --- | --- | --- | --- | --- | --- | --- | --- | --- |
| F.VNVDPDGKFVIS.T | Y | 21.53 | 1288.6663 | -0.6 | 645.3400 | 2 | 12.28 | 1756 | 1 | 111 | 122 |  |
| total 1 peptides |  |  |  |  |  |  |  |  |  |  |  |  |
| --- | --- | --- | --- | --- | --- | --- | --- | --- | --- | --- | --- | --- |

tr|A0A8A8ZWC2|A0A8A8ZWC2\_CALBE

back to list

  

| Protein Coverage
| Supporting Peptides
|

Protein Coverage:

Supporting Peptides:

| Peptide | Uniq | -10lgP | Mass | ppm | m/z | z | RT | Scan | #Spec | Start | End | PTM |
| --- | --- | --- | --- | --- | --- | --- | --- | --- | --- | --- | --- | --- |
| F.VNVDPDGKFVIS.T | Y | 21.53 | 1288.6663 | -0.6 | 645.3400 | 2 | 12.28 | 1756 | 1 | 111 | 122 |  |
| total 1 peptides |  |  |  |  |  |  |  |  |  |  |  |  |
| --- | --- | --- | --- | --- | --- | --- | --- | --- | --- | --- | --- | --- |

tr|G3D676|G3D676\_PORTR

back to list

  

| Protein Coverage
| Supporting Peptides
|

Protein Coverage:

Supporting Peptides:

| Peptide | Uniq | -10lgP | Mass | ppm | m/z | z | RT | Scan | #Spec | Start | End | PTM |
| --- | --- | --- | --- | --- | --- | --- | --- | --- | --- | --- | --- | --- |
| F.VNVDPDGKFVIS.T | Y | 21.53 | 1288.6663 | -0.6 | 645.3400 | 2 | 12.28 | 1756 | 1 | 111 | 122 |  |
| total 1 peptides |  |  |  |  |  |  |  |  |  |  |  |  |
| --- | --- | --- | --- | --- | --- | --- | --- | --- | --- | --- | --- | --- |

Q9GYX1|KARG\_PACMR

back to list

  

| Protein Coverage
| Supporting Peptides
|

Protein Coverage:

Supporting Peptides:

| Peptide | Uniq | -10lgP | Mass | ppm | m/z | z | RT | Scan | #Spec | Start | End | PTM |
| --- | --- | --- | --- | --- | --- | --- | --- | --- | --- | --- | --- | --- |
| F.VNVDPDGKFVIS.T | Y | 21.53 | 1288.6663 | -0.6 | 645.3400 | 2 | 12.28 | 1756 | 1 | 111 | 122 |  |
| total 1 peptides |  |  |  |  |  |  |  |  |  |  |  |  |
| --- | --- | --- | --- | --- | --- | --- | --- | --- | --- | --- | --- | --- |

H6VGI2|KARG\_PROCL

back to list

  

| Protein Coverage
| Supporting Peptides
|

Protein Coverage:

Supporting Peptides:

| Peptide | Uniq | -10lgP | Mass | ppm | m/z | z | RT | Scan | #Spec | Start | End | PTM |
| --- | --- | --- | --- | --- | --- | --- | --- | --- | --- | --- | --- | --- |
| F.VNVDPDGKFVIS.T | Y | 21.53 | 1288.6663 | -0.6 | 645.3400 | 2 | 12.28 | 1756 | 1 | 111 | 122 |  |
| total 1 peptides |  |  |  |  |  |  |  |  |  |  |  |  |
| --- | --- | --- | --- | --- | --- | --- | --- | --- | --- | --- | --- | --- |

tr|G3D677|G3D677\_PORTR

back to list

  

| Protein Coverage
| Supporting Peptides
|

Protein Coverage:

Supporting Peptides:

| Peptide | Uniq | -10lgP | Mass | ppm | m/z | z | RT | Scan | #Spec | Start | End | PTM |
| --- | --- | --- | --- | --- | --- | --- | --- | --- | --- | --- | --- | --- |
| F.VNVDPDGKFVIS.T | Y | 21.53 | 1288.6663 | -0.6 | 645.3400 | 2 | 12.28 | 1756 | 1 | 111 | 122 |  |
| total 1 peptides |  |  |  |  |  |  |  |  |  |  |  |  |
| --- | --- | --- | --- | --- | --- | --- | --- | --- | --- | --- | --- | --- |

tr|H6AC58|H6AC58\_PORTR

back to list

  

| Protein Coverage
| Supporting Peptides
|

Protein Coverage:

Supporting Peptides:

| Peptide | Uniq | -10lgP | Mass | ppm | m/z | z | RT | Scan | #Spec | Start | End | PTM |
| --- | --- | --- | --- | --- | --- | --- | --- | --- | --- | --- | --- | --- |
| F.VNVDPDGKFVIS.T | Y | 21.53 | 1288.6663 | -0.6 | 645.3400 | 2 | 12.28 | 1756 | 1 | 111 | 122 |  |
| total 1 peptides |  |  |  |  |  |  |  |  |  |  |  |  |
| --- | --- | --- | --- | --- | --- | --- | --- | --- | --- | --- | --- | --- |

tr|A0A068EFU4|A0A068EFU4\_9EUCA

back to list

  

| Protein Coverage
| Supporting Peptides
|

Protein Coverage:

Supporting Peptides:

| Peptide | Uniq | -10lgP | Mass | ppm | m/z | z | RT | Scan | #Spec | Start | End | PTM |
| --- | --- | --- | --- | --- | --- | --- | --- | --- | --- | --- | --- | --- |
| F.VNVDPDGKFVIS.T | Y | 21.53 | 1288.6663 | -0.6 | 645.3400 | 2 | 12.28 | 1756 | 1 | 111 | 122 |  |
| total 1 peptides |  |  |  |  |  |  |  |  |  |  |  |  |
| --- | --- | --- | --- | --- | --- | --- | --- | --- | --- | --- | --- | --- |

tr|G3D691|G3D691\_PORTR

back to list

  

| Protein Coverage
| Supporting Peptides
|

Protein Coverage:

Supporting Peptides:

| Peptide | Uniq | -10lgP | Mass | ppm | m/z | z | RT | Scan | #Spec | Start | End | PTM |
| --- | --- | --- | --- | --- | --- | --- | --- | --- | --- | --- | --- | --- |
| F.VNVDPDGKFVIS.T | Y | 21.53 | 1288.6663 | -0.6 | 645.3400 | 2 | 12.28 | 1756 | 1 | 111 | 122 |  |
| total 1 peptides |  |  |  |  |  |  |  |  |  |  |  |  |
| --- | --- | --- | --- | --- | --- | --- | --- | --- | --- | --- | --- | --- |

Q9NH49|KARG\_CALSI

back to list

  

| Protein Coverage
| Supporting Peptides
|

Protein Coverage:

Supporting Peptides:

| Peptide | Uniq | -10lgP | Mass | ppm | m/z | z | RT | Scan | #Spec | Start | End | PTM |
| --- | --- | --- | --- | --- | --- | --- | --- | --- | --- | --- | --- | --- |
| F.VNVDPDGKFVIS.T | Y | 21.53 | 1288.6663 | -0.6 | 645.3400 | 2 | 12.28 | 1756 | 1 | 111 | 122 |  |
| total 1 peptides |  |  |  |  |  |  |  |  |  |  |  |  |
| --- | --- | --- | --- | --- | --- | --- | --- | --- | --- | --- | --- | --- |

Q9U9J4|KARG\_CARMA

back to list

  

| Protein Coverage
| Supporting Peptides
|

Protein Coverage:

Supporting Peptides:

| Peptide | Uniq | -10lgP | Mass | ppm | m/z | z | RT | Scan | #Spec | Start | End | PTM |
| --- | --- | --- | --- | --- | --- | --- | --- | --- | --- | --- | --- | --- |
| F.VNVDPDGKFVIS.T | Y | 21.53 | 1288.6663 | -0.6 | 645.3400 | 2 | 12.28 | 1756 | 1 | 111 | 122 |  |
| total 1 peptides |  |  |  |  |  |  |  |  |  |  |  |  |
| --- | --- | --- | --- | --- | --- | --- | --- | --- | --- | --- | --- | --- |

tr|H6UKS0|H6UKS0\_SCYPA

back to list

  

| Protein Coverage
| Supporting Peptides
|

Protein Coverage:

Supporting Peptides:

| Peptide | Uniq | -10lgP | Mass | ppm | m/z | z | RT | Scan | #Spec | Start | End | PTM |
| --- | --- | --- | --- | --- | --- | --- | --- | --- | --- | --- | --- | --- |
| F.VNVDPDGKFVIS.T | Y | 21.53 | 1288.6663 | -0.6 | 645.3400 | 2 | 12.28 | 1756 | 1 | 111 | 122 |  |
| total 1 peptides |  |  |  |  |  |  |  |  |  |  |  |  |
| --- | --- | --- | --- | --- | --- | --- | --- | --- | --- | --- | --- | --- |

Q9NH48|KARG\_ERISI

back to list

  

| Protein Coverage
| Supporting Peptides
|

Protein Coverage:

Supporting Peptides:

| Peptide | Uniq | -10lgP | Mass | ppm | m/z | z | RT | Scan | #Spec | Start | End | PTM |
| --- | --- | --- | --- | --- | --- | --- | --- | --- | --- | --- | --- | --- |
| F.VNVDPDGKFVIS.T | Y | 21.53 | 1288.6663 | -0.6 | 645.3400 | 2 | 12.28 | 1756 | 1 | 111 | 122 |  |
| total 1 peptides |  |  |  |  |  |  |  |  |  |  |  |  |
| --- | --- | --- | --- | --- | --- | --- | --- | --- | --- | --- | --- | --- |

tr|G3D675|G3D675\_PORTR

back to list

  

| Protein Coverage
| Supporting Peptides
|

Protein Coverage:

Supporting Peptides:

| Peptide | Uniq | -10lgP | Mass | ppm | m/z | z | RT | Scan | #Spec | Start | End | PTM |
| --- | --- | --- | --- | --- | --- | --- | --- | --- | --- | --- | --- | --- |
| F.VNVDPDGKFVIS.T | Y | 21.53 | 1288.6663 | -0.6 | 645.3400 | 2 | 12.28 | 1756 | 1 | 111 | 122 |  |
| total 1 peptides |  |  |  |  |  |  |  |  |  |  |  |  |
| --- | --- | --- | --- | --- | --- | --- | --- | --- | --- | --- | --- | --- |

H6VGI3|KARG0\_SCYPA

back to list

  

| Protein Coverage
| Supporting Peptides
|

Protein Coverage:

Supporting Peptides:

| Peptide | Uniq | -10lgP | Mass | ppm | m/z | z | RT | Scan | #Spec | Start | End | PTM |
| --- | --- | --- | --- | --- | --- | --- | --- | --- | --- | --- | --- | --- |
| F.VNVDPDGKFVIS.T | Y | 21.53 | 1288.6663 | -0.6 | 645.3400 | 2 | 12.28 | 1756 | 1 | 111 | 122 |  |
| total 1 peptides |  |  |  |  |  |  |  |  |  |  |  |  |
| --- | --- | --- | --- | --- | --- | --- | --- | --- | --- | --- | --- | --- |

tr|A0A1C6ZZL8|A0A1C6ZZL8\_PROCL

back to list

  

| Protein Coverage
| Supporting Peptides
|

Protein Coverage:

Supporting Peptides:

| Peptide | Uniq | -10lgP | Mass | ppm | m/z | z | RT | Scan | #Spec | Start | End | PTM |
| --- | --- | --- | --- | --- | --- | --- | --- | --- | --- | --- | --- | --- |
| F.VNVDPDGKFVIS.T | Y | 21.53 | 1288.6663 | -0.6 | 645.3400 | 2 | 12.28 | 1756 | 1 | 111 | 122 |  |
| total 1 peptides |  |  |  |  |  |  |  |  |  |  |  |  |
| --- | --- | --- | --- | --- | --- | --- | --- | --- | --- | --- | --- | --- |

tr|A0A0C5DEY3|A0A0C5DEY3\_CHEDE

back to list

  

| Protein Coverage
| Supporting Peptides
|

Protein Coverage:

Supporting Peptides:

| Peptide | Uniq | -10lgP | Mass | ppm | m/z | z | RT | Scan | #Spec | Start | End | PTM |
| --- | --- | --- | --- | --- | --- | --- | --- | --- | --- | --- | --- | --- |
| F.VNVDPDGKFVIS.T | Y | 21.53 | 1288.6663 | -0.6 | 645.3400 | 2 | 12.28 | 1756 | 1 | 111 | 122 |  |
| total 1 peptides |  |  |  |  |  |  |  |  |  |  |  |  |
| --- | --- | --- | --- | --- | --- | --- | --- | --- | --- | --- | --- | --- |

tr|D2DSQ8|D2DSQ8\_SCYPA

back to list

  

| Protein Coverage
| Supporting Peptides
|

Protein Coverage:

Supporting Peptides:

| Peptide | Uniq | -10lgP | Mass | ppm | m/z | z | RT | Scan | #Spec | Start | End | PTM |
| --- | --- | --- | --- | --- | --- | --- | --- | --- | --- | --- | --- | --- |
| F.VNVDPDGKFVIS.T | Y | 21.53 | 1288.6663 | -0.6 | 645.3400 | 2 | 12.28 | 1756 | 1 | 82 | 93 |  |
| total 1 peptides |  |  |  |  |  |  |  |  |  |  |  |  |
| --- | --- | --- | --- | --- | --- | --- | --- | --- | --- | --- | --- | --- |

tr|F5A6C6|F5A6C6\_PROCL

back to list

  

| Protein Coverage
| Supporting Peptides
|

Protein Coverage:

Supporting Peptides:

| Peptide | Uniq | -10lgP | Mass | ppm | m/z | z | RT | Scan | #Spec | Start | End | PTM |
| --- | --- | --- | --- | --- | --- | --- | --- | --- | --- | --- | --- | --- |
| F.VNVDPDGKFVIS.T | Y | 21.53 | 1288.6663 | -0.6 | 645.3400 | 2 | 12.28 | 1756 | 1 | 111 | 122 |  |
| total 1 peptides |  |  |  |  |  |  |  |  |  |  |  |  |
| --- | --- | --- | --- | --- | --- | --- | --- | --- | --- | --- | --- | --- |

tr|J9K049|J9K049\_ACYPI

back to list

  

| Protein Coverage
| Supporting Peptides
|

Protein Coverage:

Supporting Peptides:

| Peptide | Uniq | -10lgP | Mass | ppm | m/z | z | RT | Scan | #Spec | Start | End | PTM |
| --- | --- | --- | --- | --- | --- | --- | --- | --- | --- | --- | --- | --- |
| Q.ETQVINAV.G | Y | 21.41 | 872.4603 | 0.1 | 437.2375 | 2 | 11.26 | 1566 | 1 | 559 | 566 |  |
| total 1 peptides |  |  |  |  |  |  |  |  |  |  |  |  |
| --- | --- | --- | --- | --- | --- | --- | --- | --- | --- | --- | --- | --- |

tr|A0A0R3NUC8|A0A0R3NUC8\_DROPS

back to list

  

| Protein Coverage
| Supporting Peptides
|

Protein Coverage:

Supporting Peptides:

| Peptide | Uniq | -10lgP | Mass | ppm | m/z | z | RT | Scan | #Spec | Start | End | PTM |
| --- | --- | --- | --- | --- | --- | --- | --- | --- | --- | --- | --- | --- |
| A.EDADKGVLVAL.V | Y | 20.28 | 1128.6027 | -22.2 | 565.2961 | 2 | 12.43 | 1783 | 1 | 70 | 80 |  |
| total 1 peptides |  |  |  |  |  |  |  |  |  |  |  |  |
| --- | --- | --- | --- | --- | --- | --- | --- | --- | --- | --- | --- | --- |

tr|B4GX09|B4GX09\_DROPE

back to list

  

| Protein Coverage
| Supporting Peptides
|

Protein Coverage:

Supporting Peptides:

| Peptide | Uniq | -10lgP | Mass | ppm | m/z | z | RT | Scan | #Spec | Start | End | PTM |
| --- | --- | --- | --- | --- | --- | --- | --- | --- | --- | --- | --- | --- |
| A.EDADKGVLVAL.V | Y | 20.28 | 1128.6027 | -22.2 | 565.2961 | 2 | 12.43 | 1783 | 1 | 48 | 58 |  |
| total 1 peptides |  |  |  |  |  |  |  |  |  |  |  |  |
| --- | --- | --- | --- | --- | --- | --- | --- | --- | --- | --- | --- | --- |

tr|A0A821UPU7|A0A821UPU7\_9NEOP

back to list

  

| Protein Coverage
| Supporting Peptides
|

Protein Coverage:

Supporting Peptides:

| Peptide | Uniq | -10lgP | Mass | ppm | m/z | z | RT | Scan | #Spec | Start | End | PTM |
| --- | --- | --- | --- | --- | --- | --- | --- | --- | --- | --- | --- | --- |
| I.LKNKTLLQEI.S | Y | 20.24 | 1198.7285 | 0.3 | 600.3717 | 2 | 14.24 | 2104 | 1 | 3604 | 3613 |  |
| total 1 peptides |  |  |  |  |  |  |  |  |  |  |  |  |
| --- | --- | --- | --- | --- | --- | --- | --- | --- | --- | --- | --- | --- |

tr|A0A023ERZ2|A0A023ERZ2\_AEDAL

back to list

  

| Protein Coverage
| Supporting Peptides
|

Protein Coverage:

Supporting Peptides:

| Peptide | Uniq | -10lgP | Mass | ppm | m/z | z | RT | Scan | #Spec | Start | End | PTM |
| --- | --- | --- | --- | --- | --- | --- | --- | --- | --- | --- | --- | --- |
| R.GDLGIEIPAEK.V | Y | 20.21 | 1140.6025 | -0.7 | 571.3082 | 2 | 12.21 | 1745 | 1 | 281 | 291 |  |
| total 1 peptides |  |  |  |  |  |  |  |  |  |  |  |  |
| --- | --- | --- | --- | --- | --- | --- | --- | --- | --- | --- | --- | --- |

tr|A0A0P8Y097|A0A0P8Y097\_DROAN

back to list

  

| Protein Coverage
| Supporting Peptides
|

Protein Coverage:

Supporting Peptides:

| Peptide | Uniq | -10lgP | Mass | ppm | m/z | z | RT | Scan | #Spec | Start | End | PTM |
| --- | --- | --- | --- | --- | --- | --- | --- | --- | --- | --- | --- | --- |
| R.GDLGIEIPAEK.V | Y | 20.21 | 1140.6025 | -0.7 | 571.3082 | 2 | 12.21 | 1745 | 1 | 277 | 287 |  |
| total 1 peptides |  |  |  |  |  |  |  |  |  |  |  |  |
| --- | --- | --- | --- | --- | --- | --- | --- | --- | --- | --- | --- | --- |

tr|A0A6P8K875|A0A6P8K875\_DROMA

back to list

  

| Protein Coverage
| Supporting Peptides
|

Protein Coverage:

Supporting Peptides:

| Peptide | Uniq | -10lgP | Mass | ppm | m/z | z | RT | Scan | #Spec | Start | End | PTM |
| --- | --- | --- | --- | --- | --- | --- | --- | --- | --- | --- | --- | --- |
| R.GDLGIEIPAEK.V | Y | 20.21 | 1140.6025 | -0.7 | 571.3082 | 2 | 12.21 | 1745 | 1 | 277 | 287 |  |
| total 1 peptides |  |  |  |  |  |  |  |  |  |  |  |  |
| --- | --- | --- | --- | --- | --- | --- | --- | --- | --- | --- | --- | --- |

tr|A0A6P4F4P4|A0A6P4F4P4\_DRORH

back to list

  

| Protein Coverage
| Supporting Peptides
|

Protein Coverage:

Supporting Peptides:

| Peptide | Uniq | -10lgP | Mass | ppm | m/z | z | RT | Scan | #Spec | Start | End | PTM |
| --- | --- | --- | --- | --- | --- | --- | --- | --- | --- | --- | --- | --- |
| R.GDLGIEIPAEK.V | Y | 20.21 | 1140.6025 | -0.7 | 571.3082 | 2 | 12.21 | 1745 | 1 | 277 | 287 |  |
| total 1 peptides |  |  |  |  |  |  |  |  |  |  |  |  |
| --- | --- | --- | --- | --- | --- | --- | --- | --- | --- | --- | --- | --- |

tr|A0A1Y9GJY2|A0A1Y9GJY2\_ANOAR

back to list

  

| Protein Coverage
| Supporting Peptides
|

Protein Coverage:

Supporting Peptides:

| Peptide | Uniq | -10lgP | Mass | ppm | m/z | z | RT | Scan | #Spec | Start | End | PTM |
| --- | --- | --- | --- | --- | --- | --- | --- | --- | --- | --- | --- | --- |
| R.GDLGIEIPAEK.V | Y | 20.21 | 1140.6025 | -0.7 | 571.3082 | 2 | 12.21 | 1745 | 1 | 277 | 287 |  |
| total 1 peptides |  |  |  |  |  |  |  |  |  |  |  |  |
| --- | --- | --- | --- | --- | --- | --- | --- | --- | --- | --- | --- | --- |

tr|A0A0Q9X1L7|A0A0Q9X1L7\_DROMO

back to list

  

| Protein Coverage
| Supporting Peptides
|

Protein Coverage:

Supporting Peptides:

| Peptide | Uniq | -10lgP | Mass | ppm | m/z | z | RT | Scan | #Spec | Start | End | PTM |
| --- | --- | --- | --- | --- | --- | --- | --- | --- | --- | --- | --- | --- |
| R.GDLGIEIPAEK.V | Y | 20.21 | 1140.6025 | -0.7 | 571.3082 | 2 | 12.21 | 1745 | 1 | 277 | 287 |  |
| total 1 peptides |  |  |  |  |  |  |  |  |  |  |  |  |
| --- | --- | --- | --- | --- | --- | --- | --- | --- | --- | --- | --- | --- |

tr|A0A182UE55|A0A182UE55\_9DIPT

back to list

  

| Protein Coverage
| Supporting Peptides
|

Protein Coverage:

Supporting Peptides:

| Peptide | Uniq | -10lgP | Mass | ppm | m/z | z | RT | Scan | #Spec | Start | End | PTM |
| --- | --- | --- | --- | --- | --- | --- | --- | --- | --- | --- | --- | --- |
| R.GDLGIEIPAEK.V | Y | 20.21 | 1140.6025 | -0.7 | 571.3082 | 2 | 12.21 | 1745 | 1 | 277 | 287 |  |
| total 1 peptides |  |  |  |  |  |  |  |  |  |  |  |  |
| --- | --- | --- | --- | --- | --- | --- | --- | --- | --- | --- | --- | --- |

tr|A0A2M3ZFB9|A0A2M3ZFB9\_9DIPT

back to list

  

| Protein Coverage
| Supporting Peptides
|

Protein Coverage:

Supporting Peptides:

| Peptide | Uniq | -10lgP | Mass | ppm | m/z | z | RT | Scan | #Spec | Start | End | PTM |
| --- | --- | --- | --- | --- | --- | --- | --- | --- | --- | --- | --- | --- |
| R.GDLGIEIPAEK.V | Y | 20.21 | 1140.6025 | -0.7 | 571.3082 | 2 | 12.21 | 1745 | 1 | 281 | 291 |  |
| total 1 peptides |  |  |  |  |  |  |  |  |  |  |  |  |
| --- | --- | --- | --- | --- | --- | --- | --- | --- | --- | --- | --- | --- |

tr|A0A2M3ZYS6|A0A2M3ZYS6\_9DIPT

back to list

  

| Protein Coverage
| Supporting Peptides
|

Protein Coverage:

Supporting Peptides:

| Peptide | Uniq | -10lgP | Mass | ppm | m/z | z | RT | Scan | #Spec | Start | End | PTM |
| --- | --- | --- | --- | --- | --- | --- | --- | --- | --- | --- | --- | --- |
| R.GDLGIEIPAEK.V | Y | 20.21 | 1140.6025 | -0.7 | 571.3082 | 2 | 12.21 | 1745 | 1 | 281 | 291 |  |
| total 1 peptides |  |  |  |  |  |  |  |  |  |  |  |  |
| --- | --- | --- | --- | --- | --- | --- | --- | --- | --- | --- | --- | --- |

tr|A0A2M3ZYS8|A0A2M3ZYS8\_9DIPT

back to list

  

| Protein Coverage
| Supporting Peptides
|

Protein Coverage:

Supporting Peptides:

| Peptide | Uniq | -10lgP | Mass | ppm | m/z | z | RT | Scan | #Spec | Start | End | PTM |
| --- | --- | --- | --- | --- | --- | --- | --- | --- | --- | --- | --- | --- |
| R.GDLGIEIPAEK.V | Y | 20.21 | 1140.6025 | -0.7 | 571.3082 | 2 | 12.21 | 1745 | 1 | 281 | 291 |  |
| total 1 peptides |  |  |  |  |  |  |  |  |  |  |  |  |
| --- | --- | --- | --- | --- | --- | --- | --- | --- | --- | --- | --- | --- |

tr|A0A1Y9GJK0|A0A1Y9GJK0\_ANOAR

back to list

  

| Protein Coverage
| Supporting Peptides
|

Protein Coverage:

Supporting Peptides:

| Peptide | Uniq | -10lgP | Mass | ppm | m/z | z | RT | Scan | #Spec | Start | End | PTM |
| --- | --- | --- | --- | --- | --- | --- | --- | --- | --- | --- | --- | --- |
| R.GDLGIEIPAEK.V | Y | 20.21 | 1140.6025 | -0.7 | 571.3082 | 2 | 12.21 | 1745 | 1 | 282 | 292 |  |
| total 1 peptides |  |  |  |  |  |  |  |  |  |  |  |  |
| --- | --- | --- | --- | --- | --- | --- | --- | --- | --- | --- | --- | --- |

tr|A0A6A0H7S7|A0A6A0H7S7\_HYAAZ

back to list

  

| Protein Coverage
| Supporting Peptides
|

Protein Coverage:

Supporting Peptides:

| Peptide | Uniq | -10lgP | Mass | ppm | m/z | z | RT | Scan | #Spec | Start | End | PTM |
| --- | --- | --- | --- | --- | --- | --- | --- | --- | --- | --- | --- | --- |
| R.GDLGIEIPAEK.V | Y | 20.21 | 1140.6025 | -0.7 | 571.3082 | 2 | 12.21 | 1745 | 1 | 286 | 296 |  |
| total 1 peptides |  |  |  |  |  |  |  |  |  |  |  |  |
| --- | --- | --- | --- | --- | --- | --- | --- | --- | --- | --- | --- | --- |

tr|A0A1E1X8N4|A0A1E1X8N4\_9ACAR

back to list

  

| Protein Coverage
| Supporting Peptides
|

Protein Coverage:

Supporting Peptides:

| Peptide | Uniq | -10lgP | Mass | ppm | m/z | z | RT | Scan | #Spec | Start | End | PTM |
| --- | --- | --- | --- | --- | --- | --- | --- | --- | --- | --- | --- | --- |
| R.GDLGIEIPAEK.V | Y | 20.21 | 1140.6025 | -0.7 | 571.3082 | 2 | 12.21 | 1745 | 1 | 290 | 300 |  |
| total 1 peptides |  |  |  |  |  |  |  |  |  |  |  |  |
| --- | --- | --- | --- | --- | --- | --- | --- | --- | --- | --- | --- | --- |

tr|A0A6P4F9I4|A0A6P4F9I4\_DRORH

back to list

  

| Protein Coverage
| Supporting Peptides
|

Protein Coverage:

Supporting Peptides:

| Peptide | Uniq | -10lgP | Mass | ppm | m/z | z | RT | Scan | #Spec | Start | End | PTM |
| --- | --- | --- | --- | --- | --- | --- | --- | --- | --- | --- | --- | --- |
| R.GDLGIEIPAEK.V | Y | 20.21 | 1140.6025 | -0.7 | 571.3082 | 2 | 12.21 | 1745 | 1 | 298 | 308 |  |
| total 1 peptides |  |  |  |  |  |  |  |  |  |  |  |  |
| --- | --- | --- | --- | --- | --- | --- | --- | --- | --- | --- | --- | --- |

tr|A0A182VPN6|A0A182VPN6\_9DIPT

back to list

  

| Protein Coverage
| Supporting Peptides
|

Protein Coverage:

Supporting Peptides:

| Peptide | Uniq | -10lgP | Mass | ppm | m/z | z | RT | Scan | #Spec | Start | End | PTM |
| --- | --- | --- | --- | --- | --- | --- | --- | --- | --- | --- | --- | --- |
| R.GDLGIEIPAEK.V | Y | 20.21 | 1140.6025 | -0.7 | 571.3082 | 2 | 12.21 | 1745 | 1 | 293 | 303 |  |
| total 1 peptides |  |  |  |  |  |  |  |  |  |  |  |  |
| --- | --- | --- | --- | --- | --- | --- | --- | --- | --- | --- | --- | --- |

tr|A0A182MNQ6|A0A182MNQ6\_9DIPT

back to list

  

| Protein Coverage
| Supporting Peptides
|

Protein Coverage:

Supporting Peptides:

| Peptide | Uniq | -10lgP | Mass | ppm | m/z | z | RT | Scan | #Spec | Start | End | PTM |
| --- | --- | --- | --- | --- | --- | --- | --- | --- | --- | --- | --- | --- |
| R.GDLGIEIPAEK.V | Y | 20.21 | 1140.6025 | -0.7 | 571.3082 | 2 | 12.21 | 1745 | 1 | 293 | 303 |  |
| total 1 peptides |  |  |  |  |  |  |  |  |  |  |  |  |
| --- | --- | --- | --- | --- | --- | --- | --- | --- | --- | --- | --- | --- |

tr|A0A6J2T5C5|A0A6J2T5C5\_DROLE

back to list

  

| Protein Coverage
| Supporting Peptides
|

Protein Coverage:

Supporting Peptides:

| Peptide | Uniq | -10lgP | Mass | ppm | m/z | z | RT | Scan | #Spec | Start | End | PTM |
| --- | --- | --- | --- | --- | --- | --- | --- | --- | --- | --- | --- | --- |
| R.GDLGIEIPAEK.V | Y | 20.21 | 1140.6025 | -0.7 | 571.3082 | 2 | 12.21 | 1745 | 1 | 298 | 308 |  |
| total 1 peptides |  |  |  |  |  |  |  |  |  |  |  |  |
| --- | --- | --- | --- | --- | --- | --- | --- | --- | --- | --- | --- | --- |

tr|A0A1W4W378|A0A1W4W378\_DROFC

back to list

  

| Protein Coverage
| Supporting Peptides
|

Protein Coverage:

Supporting Peptides:

| Peptide | Uniq | -10lgP | Mass | ppm | m/z | z | RT | Scan | #Spec | Start | End | PTM |
| --- | --- | --- | --- | --- | --- | --- | --- | --- | --- | --- | --- | --- |
| R.GDLGIEIPAEK.V | Y | 20.21 | 1140.6025 | -0.7 | 571.3082 | 2 | 12.21 | 1745 | 1 | 298 | 308 |  |
| total 1 peptides |  |  |  |  |  |  |  |  |  |  |  |  |
| --- | --- | --- | --- | --- | --- | --- | --- | --- | --- | --- | --- | --- |

O62619|KPYK\_DROME

back to list

  

| Protein Coverage
| Supporting Peptides
|

Protein Coverage:

Supporting Peptides:

| Peptide | Uniq | -10lgP | Mass | ppm | m/z | z | RT | Scan | #Spec | Start | End | PTM |
| --- | --- | --- | --- | --- | --- | --- | --- | --- | --- | --- | --- | --- |
| R.GDLGIEIPAEK.V | Y | 20.21 | 1140.6025 | -0.7 | 571.3082 | 2 | 12.21 | 1745 | 1 | 298 | 308 |  |
| total 1 peptides |  |  |  |  |  |  |  |  |  |  |  |  |
| --- | --- | --- | --- | --- | --- | --- | --- | --- | --- | --- | --- | --- |

tr|B4HE69|B4HE69\_DROSE

back to list

  

| Protein Coverage
| Supporting Peptides
|

Protein Coverage:

Supporting Peptides:

| Peptide | Uniq | -10lgP | Mass | ppm | m/z | z | RT | Scan | #Spec | Start | End | PTM |
| --- | --- | --- | --- | --- | --- | --- | --- | --- | --- | --- | --- | --- |
| R.GDLGIEIPAEK.V | Y | 20.21 | 1140.6025 | -0.7 | 571.3082 | 2 | 12.21 | 1745 | 1 | 298 | 308 |  |
| total 1 peptides |  |  |  |  |  |  |  |  |  |  |  |  |
| --- | --- | --- | --- | --- | --- | --- | --- | --- | --- | --- | --- | --- |

tr|A0A6P4F0Q9|A0A6P4F0Q9\_DRORH

back to list

  

| Protein Coverage
| Supporting Peptides
|

Protein Coverage:

Supporting Peptides:

| Peptide | Uniq | -10lgP | Mass | ppm | m/z | z | RT | Scan | #Spec | Start | End | PTM |
| --- | --- | --- | --- | --- | --- | --- | --- | --- | --- | --- | --- | --- |
| R.GDLGIEIPAEK.V | Y | 20.21 | 1140.6025 | -0.7 | 571.3082 | 2 | 12.21 | 1745 | 1 | 298 | 308 |  |
| total 1 peptides |  |  |  |  |  |  |  |  |  |  |  |  |
| --- | --- | --- | --- | --- | --- | --- | --- | --- | --- | --- | --- | --- |

tr|B3P7K4|B3P7K4\_DROER

back to list

  

| Protein Coverage
| Supporting Peptides
|

Protein Coverage:

Supporting Peptides:

| Peptide | Uniq | -10lgP | Mass | ppm | m/z | z | RT | Scan | #Spec | Start | End | PTM |
| --- | --- | --- | --- | --- | --- | --- | --- | --- | --- | --- | --- | --- |
| R.GDLGIEIPAEK.V | Y | 20.21 | 1140.6025 | -0.7 | 571.3082 | 2 | 12.21 | 1745 | 1 | 298 | 308 |  |
| total 1 peptides |  |  |  |  |  |  |  |  |  |  |  |  |
| --- | --- | --- | --- | --- | --- | --- | --- | --- | --- | --- | --- | --- |

tr|B3M213|B3M213\_DROAN

back to list

  

| Protein Coverage
| Supporting Peptides
|

Protein Coverage:

Supporting Peptides:

| Peptide | Uniq | -10lgP | Mass | ppm | m/z | z | RT | Scan | #Spec | Start | End | PTM |
| --- | --- | --- | --- | --- | --- | --- | --- | --- | --- | --- | --- | --- |
| R.GDLGIEIPAEK.V | Y | 20.21 | 1140.6025 | -0.7 | 571.3082 | 2 | 12.21 | 1745 | 1 | 298 | 308 |  |
| total 1 peptides |  |  |  |  |  |  |  |  |  |  |  |  |
| --- | --- | --- | --- | --- | --- | --- | --- | --- | --- | --- | --- | --- |

tr|A0A6P8KMB4|A0A6P8KMB4\_DROMA

back to list

  

| Protein Coverage
| Supporting Peptides
|

Protein Coverage:

Supporting Peptides:

| Peptide | Uniq | -10lgP | Mass | ppm | m/z | z | RT | Scan | #Spec | Start | End | PTM |
| --- | --- | --- | --- | --- | --- | --- | --- | --- | --- | --- | --- | --- |
| R.GDLGIEIPAEK.V | Y | 20.21 | 1140.6025 | -0.7 | 571.3082 | 2 | 12.21 | 1745 | 1 | 298 | 308 |  |
| total 1 peptides |  |  |  |  |  |  |  |  |  |  |  |  |
| --- | --- | --- | --- | --- | --- | --- | --- | --- | --- | --- | --- | --- |

tr|B4K5E3|B4K5E3\_DROMO

back to list

  

| Protein Coverage
| Supporting Peptides
|

Protein Coverage:

Supporting Peptides:

| Peptide | Uniq | -10lgP | Mass | ppm | m/z | z | RT | Scan | #Spec | Start | End | PTM |
| --- | --- | --- | --- | --- | --- | --- | --- | --- | --- | --- | --- | --- |
| R.GDLGIEIPAEK.V | Y | 20.21 | 1140.6025 | -0.7 | 571.3082 | 2 | 12.21 | 1745 | 1 | 298 | 308 |  |
| total 1 peptides |  |  |  |  |  |  |  |  |  |  |  |  |
| --- | --- | --- | --- | --- | --- | --- | --- | --- | --- | --- | --- | --- |

tr|A0A484BWG4|A0A484BWG4\_DRONA

back to list

  

| Protein Coverage
| Supporting Peptides
|

Protein Coverage:

Supporting Peptides:

| Peptide | Uniq | -10lgP | Mass | ppm | m/z | z | RT | Scan | #Spec | Start | End | PTM |
| --- | --- | --- | --- | --- | --- | --- | --- | --- | --- | --- | --- | --- |
| R.GDLGIEIPAEK.V | Y | 20.21 | 1140.6025 | -0.7 | 571.3082 | 2 | 12.21 | 1745 | 1 | 298 | 308 |  |
| total 1 peptides |  |  |  |  |  |  |  |  |  |  |  |  |
| --- | --- | --- | --- | --- | --- | --- | --- | --- | --- | --- | --- | --- |

tr|A0A131XQM7|A0A131XQM7\_9ACAR

back to list

  

| Protein Coverage
| Supporting Peptides
|

Protein Coverage:

Supporting Peptides:

| Peptide | Uniq | -10lgP | Mass | ppm | m/z | z | RT | Scan | #Spec | Start | End | PTM |
| --- | --- | --- | --- | --- | --- | --- | --- | --- | --- | --- | --- | --- |
| R.GDLGIEIPAEK.V | Y | 20.21 | 1140.6025 | -0.7 | 571.3082 | 2 | 12.21 | 1745 | 1 | 290 | 300 |  |
| total 1 peptides |  |  |  |  |  |  |  |  |  |  |  |  |
| --- | --- | --- | --- | --- | --- | --- | --- | --- | --- | --- | --- | --- |

tr|A0A0P9ARZ1|A0A0P9ARZ1\_DROAN

back to list

  

| Protein Coverage
| Supporting Peptides
|

Protein Coverage:

Supporting Peptides:

| Peptide | Uniq | -10lgP | Mass | ppm | m/z | z | RT | Scan | #Spec | Start | End | PTM |
| --- | --- | --- | --- | --- | --- | --- | --- | --- | --- | --- | --- | --- |
| R.GDLGIEIPAEK.V | Y | 20.21 | 1140.6025 | -0.7 | 571.3082 | 2 | 12.21 | 1745 | 1 | 312 | 322 |  |
| total 1 peptides |  |  |  |  |  |  |  |  |  |  |  |  |
| --- | --- | --- | --- | --- | --- | --- | --- | --- | --- | --- | --- | --- |

tr|G3MLC1|G3MLC1\_AMBMU

back to list

  

| Protein Coverage
| Supporting Peptides
|

Protein Coverage:

Supporting Peptides:

| Peptide | Uniq | -10lgP | Mass | ppm | m/z | z | RT | Scan | #Spec | Start | End | PTM |
| --- | --- | --- | --- | --- | --- | --- | --- | --- | --- | --- | --- | --- |
| R.GDLGIEIPAEK.V | Y | 20.21 | 1140.6025 | -0.7 | 571.3082 | 2 | 12.21 | 1745 | 1 | 290 | 300 |  |
| total 1 peptides |  |  |  |  |  |  |  |  |  |  |  |  |
| --- | --- | --- | --- | --- | --- | --- | --- | --- | --- | --- | --- | --- |

tr|A0A023EVC5|A0A023EVC5\_AEDAL

back to list

  

| Protein Coverage
| Supporting Peptides
|

Protein Coverage:

Supporting Peptides:

| Peptide | Uniq | -10lgP | Mass | ppm | m/z | z | RT | Scan | #Spec | Start | End | PTM |
| --- | --- | --- | --- | --- | --- | --- | --- | --- | --- | --- | --- | --- |
| R.GDLGIEIPAEK.V | Y | 20.21 | 1140.6025 | -0.7 | 571.3082 | 2 | 12.21 | 1745 | 1 | 350 | 360 |  |
| total 1 peptides |  |  |  |  |  |  |  |  |  |  |  |  |
| --- | --- | --- | --- | --- | --- | --- | --- | --- | --- | --- | --- | --- |

tr|A0A5N5T493|A0A5N5T493\_9CRUS

back to list

  

| Protein Coverage
| Supporting Peptides
|

Protein Coverage:

Supporting Peptides:

| Peptide | Uniq | -10lgP | Mass | ppm | m/z | z | RT | Scan | #Spec | Start | End | PTM |
| --- | --- | --- | --- | --- | --- | --- | --- | --- | --- | --- | --- | --- |
| R.GDLGIEIPAEK.V | Y | 20.21 | 1140.6025 | -0.7 | 571.3082 | 2 | 12.21 | 1745 | 1 | 390 | 400 |  |
| total 1 peptides |  |  |  |  |  |  |  |  |  |  |  |  |
| --- | --- | --- | --- | --- | --- | --- | --- | --- | --- | --- | --- | --- |

---

Prepared with PEAKS ™ (bioinfor.com)
