# Supplementary material for: Proteomic and Functional Characterization of Antimicrobial Peptides Derived from Fisheries Bycatch via Enzymatic Hydrolysis
Source: Mar Drugs. 2026 Jan 10;24(1):36. doi: 10.3390/md24010036 (PMC12843163; doi:10.3390/md24010036)
Supplement: Supplementary file 1 [file marinedrugs-24-00036-s001.zip › SM40/MA/protein.html]

proteins


Summary

  

# 1. Notes

# 2. Result Statistics

**Figure 1.**
False discovery rate (FDR) curve. X axis is the number of peptide-spectrum matches (PSM) being kept. Y axis is the corresponding FDR.

  


  

**Figure 2.**
PSM score distribution.  **(a)**
Distribution of PEAKS peptide score;  **(b)**
Scatterplot of PEAKS peptide score versus precursor mass error.

**(a)**

**(b)**

**Figure 3.**
De novo result validation. Distribution of residue local confidence:  **(a)**
Residues in de novo sequences validated by confident database peptide assignment;  **(b)**
Residues in "de novo only" sequences.

**(a)**

**(b)**

**Table 1.**
Statistics of data.

|  |  |
| --- | --- |
| # of MS Scans | 1037 |
| # of MS/MS Scans | 2802 |


**Table 2.**
Result filtration parameters.

|  |  |
| --- | --- |
| Peptide -10lgP | ≥15 |
| Protein -10lgP | ≥20 |
| Proteins unique peptides | ≥0 |
| De novo ALC Score | ≥50% |


**Table 3.**
Statistics of filtered result.

|  |  |
| --- | --- |
| Peptide-Spectrum Matches | 92 |
| Peptide Sequences | 88 |
| Protein Groups | 34 |
| Proteins | 221 |
| Proteins (#Unique Peptides) | 31 (>2); 57 (=2); 130 (=1); |
| FDR (Peptide-Spectrum Matches) | 12.0% |
| FDR (Peptide Sequences) | 12.5% |
| FDR (Protein) | 0.5% |
| De Novo Only Spectra | 1033 |

**Table 4.**
PTM profile.

| Name | ∆Mass | #PSM | Position |
| --- | --- | --- | --- |

# 3. Experiment Control

**Figure 4.**
Precursor mass error of peptide-spectrum matches (PSM) in filtered result.  **(a)**
Distribution of precursor mass error in ppm;  **(b)**
Scatterplot of precursor m/z versus precursor mass error in ppm.

**(a)**

**(b)**

**Table 5.**
Number of identified peptides in each sample by the number of missed cleavages

|  |  |  |  |  |  |
| --- | --- | --- | --- | --- | --- |
| Missed Cleavages | 0 | 1 | 2 | 3 | 4+ |
| Orbi2222 | 0 | 0 | 0 | 0 | 88 |

# 4. Other Information

**Table 6.**
Search parameters.

Search Engine Name: PEAKS 7.0  
Parent Mass Error Tolerance: 0.03 Da  
Fragment Mass Error Tolerance: 0.1 Da  
Precursor Mass Search Type: monoisotopic  
Enzyme: None  
Max Missed Cleavages: 100  
Non-specific Cleavage: both  
Max variable PTM per peptide: 3  
Database: Teleosteo  
Taxon: All  
Searched Entry: 4529691  
FDR Estimation: Enabled  
Merge Options: 0.1 min. 0.03 Da  
Precursor Options: corrected  
Charge Options: no correction  
Filter Options: no filter  
Process: true

**Table 7.**
Instrument parameters.

Fractions: OB\_PIMENTA\_MA\_20220211\_02.raw  
Ion Source: ESI(nano-spray)  
Fragmentation Mode: CID/ECD  
MS Scan Mode: FT-ICR/Orbitrap  
MS/MS Scan Mode: Linear Ion Trap

  

Protein List

  

| Protein Group | Protein ID | Accession | -10lgP | Coverage (%) | #Peptides | #Unique | PTM | Avg. Mass | Description |
| --- | --- | --- | --- | --- | --- | --- | --- | --- | --- |
| 1 | 66811 | tr\|A0A3P9CRE7\|A0A3P9CRE7\_9CICH | 97.15 | 4 | 11 | 1 | N | 219343 | Myosin heavy chain, fast skeletal muscle OS=Maylandia zebra OX=106582 PE=3 SV=1 |
| 1 | 69465 | tr\|A0A669BHT6\|A0A669BHT6\_ORENI | 97.15 | 4 | 11 | 1 | N | 221312 | Uncharacterized protein OS=Oreochromis niloticus OX=8128 GN=LOC100712344 PE=3 SV=1 |
| 1 | 66813 | tr\|A0A3P8Q1P8\|A0A3P8Q1P8\_ASTCA | 97.15 | 4 | 11 | 1 | N | 221800 | Myosin heavy chain, fast skeletal muscle-like OS=Astatotilapia calliptera OX=8154 PE=3 SV=1 |
| 1 | 66814 | tr\|A0A3P8QMC2\|A0A3P8QMC2\_ASTCA | 97.15 | 4 | 11 | 1 | N | 222125 | Myosin heavy chain, fast skeletal muscle-like OS=Astatotilapia calliptera OX=8154 PE=3 SV=1 |
| 1 | 66815 | tr\|A0A3P8Q1G5\|A0A3P8Q1G5\_ASTCA | 97.15 | 4 | 11 | 1 | N | 222421 | Myosin heavy chain, fast skeletal muscle-like OS=Astatotilapia calliptera OX=8154 PE=3 SV=1 |
| 3 | 69467 | tr\|I3IZY8\|I3IZY8\_ORENI | 93.85 | 4 | 10 | 0 | N | 218461 | Uncharacterized protein OS=Oreochromis niloticus OX=8128 GN=LOC100707599 PE=3 SV=2 |
| 3 | 69468 | tr\|I3IZU1\|I3IZU1\_ORENI | 93.85 | 4 | 10 | 0 | N | 218648 | Uncharacterized protein OS=Oreochromis niloticus OX=8128 GN=LOC100707599 PE=3 SV=2 |
| 2 | 12040 | tr\|A0A3S2MUF9\|A0A3S2MUF9\_ORYJA | 87.01 | 15 | 10 | 10 | N | 41945 | Uncharacterized protein OS=Oryzias javanicus OX=123683 GN=OJAV\_G00024300 PE=3 SV=1 |
| 2 | 66846 | tr\|A0A7J6C441\|A0A7J6C441\_9TELE | 87.01 | 15 | 10 | 10 | N | 41959 | Uncharacterized protein OS=Onychostoma macrolepis OX=369639 GN=G5714\_016621 PE=3 SV=1 |
| 4 | 69474 | tr\|A0A3B4YLK9\|A0A3B4YLK9\_SERLL | 77.19 | 3 | 9 | 0 | N | 222756 | Myosin heavy chain, fast skeletal muscle-like OS=Seriola lalandi dorsalis OX=1841481 PE=3 SV=1 |
| 11 | 67380 | tr\|A0A671XZM8\|A0A671XZM8\_SPAAU | 64.75 | 9 | 3 | 3 | N | 45256 | Calsequestrin OS=Sparus aurata OX=8175 GN=LOC115572796 PE=3 SV=1 |
| 11 | 67381 | tr\|A0A671Y6A5\|A0A671Y6A5\_SPAAU | 64.75 | 8 | 3 | 3 | N | 50365 | Calsequestrin OS=Sparus aurata OX=8175 GN=LOC115572796 PE=3 SV=1 |
| 11 | 67384 | tr\|A0A671XZM4\|A0A671XZM4\_SPAAU | 64.75 | 8 | 3 | 3 | N | 51763 | Calsequestrin OS=Sparus aurata OX=8175 GN=LOC115572796 PE=3 SV=1 |
| 11 | 67389 | tr\|A0A671XZK3\|A0A671XZK3\_SPAAU | 64.75 | 8 | 3 | 3 | N | 52734 | Calsequestrin OS=Sparus aurata OX=8175 GN=LOC115572796 PE=3 SV=1 |
| 11 | 67395 | tr\|A0A671XZP8\|A0A671XZP8\_SPAAU | 64.75 | 7 | 3 | 3 | N | 55189 | Calsequestrin OS=Sparus aurata OX=8175 GN=LOC115572796 PE=3 SV=1 |
| 8 | 69471 | tr\|A0A834CTG4\|A0A834CTG4\_ORYME | 63.38 | 1 | 3 | 3 | N | 850198 | Titin OS=Oryzias melastigma OX=30732 GN=FQA47\_016182 PE=4 SV=1 |
| 6 | 69487 | tr\|A0A315VR11\|A0A315VR11\_GAMAF | 58.47 | 4 | 5 | 5 | N | 93743 | Creatine kinase (Fragment) OS=Gambusia affinis OX=33528 GN=CCH79\_00019883 PE=3 SV=1 |
| 9 | 66962 | tr\|A0A6G0IP12\|A0A6G0IP12\_LARCR | 57.75 | 12 | 3 | 3 | N | 18230 | Troponin C, skeletal muscle OS=Larimichthys crocea OX=215358 GN=D5F01\_LYC08192 PE=4 SV=1 |
| 9 | 67020 | tr\|A0A1A8D9W7\|A0A1A8D9W7\_9TELE | 57.75 | 15 | 3 | 3 | N | 15353 | Troponin C type 2 (Fast) OS=Nothobranchius kadleci OX=1051664 GN=TNNC2 PE=4 SV=1 |
| 9 | 67021 | tr\|A0A1A8CTF6\|A0A1A8CTF6\_9TELE | 57.75 | 12 | 3 | 3 | N | 18199 | Troponin C type 2 (Fast) OS=Nothobranchius kadleci OX=1051664 GN=TNNC2 PE=4 SV=1 |
| 9 | 67022 | tr\|A0A1A8QW41\|A0A1A8QW41\_9TELE | 57.75 | 12 | 3 | 3 | N | 18199 | Troponin C type 2 (Fast) OS=Nothobranchius rachovii OX=451742 GN=TNNC2 PE=4 SV=1 |
| 9 | 67023 | tr\|A0A1A8B5H7\|A0A1A8B5H7\_NOTFU | 57.75 | 12 | 3 | 3 | N | 18199 | Troponin C type 2 (Fast) OS=Nothobranchius furzeri OX=105023 GN=TNNC2 PE=4 SV=1 |
| 9 | 67397 | tr\|A0A1A8IG78\|A0A1A8IG78\_NOTKU | 57.75 | 22 | 3 | 3 | N | 10482 | Troponin C type 2 (Fast) (Fragment) OS=Nothobranchius kuhntae OX=321403 GN=TNNC2 PE=4 SV=1 |
| 9 | 67398 | tr\|A0A1A8ML32\|A0A1A8ML32\_9TELE | 57.75 | 22 | 3 | 3 | N | 10482 | Troponin C type 2 (Fast) (Fragment) OS=Nothobranchius pienaari OX=704102 GN=TNNC2 PE=4 SV=1 |
| 9 | 67210 | tr\|A0A1A8SNN3\|A0A1A8SNN3\_9TELE | 57.75 | 21 | 3 | 3 | N | 11028 | Troponin C type 2 (Fast) (Fragment) OS=Nothobranchius rachovii OX=451742 GN=TNNC2 PE=4 SV=1 |
| 9 | 66963 | tr\|A0A1A8Q0J1\|A0A1A8Q0J1\_9TELE | 57.75 | 12 | 3 | 3 | N | 18215 | Troponin C type 2 (Fast) OS=Nothobranchius pienaari OX=704102 GN=TNNC2 PE=4 SV=1 |
| 5 | 66958 | tr\|A0A6J2PW36\|A0A6J2PW36\_COTGO | 56.57 | 3 | 7 | 1 | N | 221235 | myosin heavy chain, fast skeletal muscle-like OS=Cottoperca gobio OX=56716 GN=LOC115009632 PE=3 SV=1 |
| 21 | 68363 | tr\|A0A6A5EZU1\|A0A6A5EZU1\_PERFL | 50.73 | 1 | 1 | 1 | N | 136636 | Uncharacterized protein OS=Perca fluviatilis OX=8168 GN=PFLUV\_G00182780 PE=4 SV=1 |
| 21 | 69488 | tr\|A0A4Z2EZH8\|A0A4Z2EZH8\_9TELE | 50.73 | 2 | 1 | 1 | N | 72089 | Collagen alpha-2(I) chain OS=Liparis tanakae OX=230148 GN=Col1a2\_1 PE=4 SV=1 |
| 21 | 69489 | tr\|A0A6A4S346\|A0A6A4S346\_SCOMX | 50.73 | 1 | 1 | 1 | N | 152271 | Uncharacterized protein OS=Scophthalmus maximus OX=52904 GN=F2P81\_022633 PE=4 SV=1 |
| 15 | 69486 | tr\|C6GKU3\|C6GKU3\_THUAL | 48.28 | 21 | 2 | 2 | N | 11548 | Parvalbumin OS=Thunnus albacares OX=8236 GN=pvalb1 PE=2 SV=1 |
| 15 | 67518 | tr\|D2KQG3\|D2KQG3\_SINCH | 48.28 | 21 | 2 | 2 | N | 11617 | Parvalbumin OS=Siniperca chuatsi OX=119488 PE=2 SV=1 |
| 15 | 67527 | tr\|A0A7J6CHT3\|A0A7J6CHT3\_9TELE | 48.28 | 21 | 2 | 2 | N | 11577 | Parvalbumin OS=Onychostoma macrolepis OX=369639 GN=G5714\_012686 PE=3 SV=1 |
| 15 | 21897 | P05941\|PRVB\_OPSTA | 48.28 | 21 | 2 | 2 | N | 11757 | Parvalbumin beta OS=Opsanus tau OX=8068 PE=1 SV=2 |
| 15 | 67519 | tr\|B9VJM3\|B9VJM3\_SINCH | 48.28 | 21 | 2 | 2 | N | 11589 | Parvalbumin OS=Siniperca chuatsi OX=119488 PE=2 SV=1 |
| 12 | 69544 | tr\|A0A668SJD1\|A0A668SJD1\_OREAU | 46.28 | 2 | 3 | 1 | N | 123810 | Myosin binding protein C, fast type b OS=Oreochromis aureus OX=47969 PE=4 SV=1 |
| 12 | 69545 | tr\|A0A6P7L5W1\|A0A6P7L5W1\_BETSP | 46.28 | 2 | 3 | 1 | N | 124685 | myosin-binding protein C, fast-type-like isoform X8 OS=Betta splendens OX=158456 GN=LOC114845484 PE=4 SV=1 |
| 12 | 69546 | tr\|A0A3P9C2F1\|A0A3P9C2F1\_9CICH | 46.28 | 2 | 3 | 1 | N | 124875 | Myosin-binding protein C, fast-type OS=Maylandia zebra OX=106582 PE=4 SV=1 |
| 12 | 69547 | tr\|A0A6P7L5V6\|A0A6P7L5V6\_BETSP | 46.28 | 2 | 3 | 1 | N | 125396 | myosin-binding protein C, fast-type-like isoform X6 OS=Betta splendens OX=158456 GN=LOC114845484 PE=4 SV=1 |
| 12 | 69548 | tr\|A0A668SIT2\|A0A668SIT2\_OREAU | 46.28 | 2 | 3 | 1 | N | 126234 | Myosin binding protein C, fast type b OS=Oreochromis aureus OX=47969 PE=4 SV=1 |
| 12 | 69549 | tr\|A0A6P7L5V2\|A0A6P7L5V2\_BETSP | 46.28 | 2 | 3 | 1 | N | 126921 | myosin-binding protein C, fast-type-like isoform X2 OS=Betta splendens OX=158456 GN=LOC114845484 PE=4 SV=1 |
| 20 | 67265 | tr\|A0A6P7INY3\|A0A6P7INY3\_9TELE | 46.23 | 1 | 1 | 1 | N | 123690 | collagen alpha-1(I) chain-like isoform X2 OS=Parambassis ranga OX=210632 GN=LOC114439468 PE=4 SV=1 |
| 20 | 67266 | tr\|A0A6P7IU28\|A0A6P7IU28\_9TELE | 46.23 | 1 | 1 | 1 | N | 136458 | collagen alpha-1(I) chain-like isoform X1 OS=Parambassis ranga OX=210632 GN=LOC114439468 PE=4 SV=1 |
| 20 | 67009 | tr\|A0A2I4D9D9\|A0A2I4D9D9\_9TELE | 46.23 | 1 | 1 | 1 | N | 133831 | collagen alpha-1(I) chain-like isoform X2 OS=Austrofundulus limnaeus OX=52670 GN=LOC106536191 PE=4 SV=1 |
| 20 | 67267 | tr\|A0A7J6DBF9\|A0A7J6DBF9\_9TELE | 46.23 | 1 | 1 | 1 | N | 136989 | Uncharacterized protein OS=Onychostoma macrolepis OX=369639 GN=G5714\_003834 PE=4 SV=1 |
| 20 | 67010 | tr\|A0A2I4D9D6\|A0A2I4D9D6\_9TELE | 46.23 | 1 | 1 | 1 | N | 136808 | collagen alpha-1(I) chain-like isoform X1 OS=Austrofundulus limnaeus OX=52670 GN=LOC106536191 PE=4 SV=1 |
| 20 | 67268 | tr\|A0A6P6JU61\|A0A6P6JU61\_CARAU | 46.23 | 1 | 1 | 1 | N | 137216 | collagen alpha-1(I) chain-like OS=Carassius auratus OX=7957 GN=LOC113046705 PE=4 SV=1 |
| 20 | 67012 | tr\|A0A3B4UIY5\|A0A3B4UIY5\_SERDU | 46.23 | 1 | 1 | 1 | N | 132549 | Collagen alpha-1(I) chain-like OS=Seriola dumerili OX=41447 PE=4 SV=1 |
| 20 | 67281 | tr\|A0A2I4D9D4\|A0A2I4D9D4\_9TELE | 46.23 | 1 | 1 | 1 | N | 114299 | collagen alpha-1(I) chain-like isoform X3 OS=Austrofundulus limnaeus OX=52670 GN=LOC106536191 PE=4 SV=1 |
| 20 | 67571 | tr\|A0A060WU51\|A0A060WU51\_ONCMY | 46.23 | 7 | 1 | 1 | N | 22546 | VWFC domain-containing protein OS=Oncorhynchus mykiss OX=8022 GN=GSONMT00040585001 PE=4 SV=1 |
| 20 | 67572 | tr\|A0A6F9C010\|A0A6F9C010\_9TELE | 46.23 | 5 | 1 | 1 | N | 27312 | VWFC domain-containing protein OS=Coregonus sp. 'balchen' OX=861768 GN=CSTEINMANNI\_LOCUS3426487 PE=4 SV=1 |
| 20 | 67573 | tr\|A0A060X5H1\|A0A060X5H1\_ONCMY | 46.23 | 4 | 1 | 1 | N | 41631 | VWFC domain-containing protein OS=Oncorhynchus mykiss OX=8022 GN=GSONMT00019804001 PE=4 SV=1 |
| 20 | 67574 | tr\|A0A4W5KL14\|A0A4W5KL14\_9TELE | 46.23 | 3 | 1 | 1 | N | 59489 | Collagen, type I, alpha 1a OS=Hucho hucho OX=62062 PE=4 SV=1 |
| 20 | 67284 | tr\|A0A673A8U8\|A0A673A8U8\_9TELE | 46.23 | 1 | 1 | 1 | N | 111385 | Collagen, type I, alpha 1b OS=Sphaeramia orbicularis OX=375764 PE=4 SV=1 |
| 20 | 67018 | tr\|I3JGN1\|I3JGN1\_ORENI | 46.23 | 1 | 1 | 1 | N | 121918 | Uncharacterized protein OS=Oreochromis niloticus OX=8128 GN=col1a1 PE=4 SV=2 |
| 20 | 67417 | tr\|A0A671YED8\|A0A671YED8\_SPAAU | 46.23 | 1 | 1 | 1 | N | 131412 | Collagen, type I, alpha 1b OS=Sparus aurata OX=8175 PE=4 SV=1 |
| 20 | 67418 | tr\|A0A671YFV1\|A0A671YFV1\_SPAAU | 46.23 | 1 | 1 | 1 | N | 132045 | Collagen, type I, alpha 1b OS=Sparus aurata OX=8175 PE=4 SV=1 |
| 20 | 67275 | tr\|A0A3Q2W9Y5\|A0A3Q2W9Y5\_HAPBU | 46.23 | 1 | 1 | 1 | N | 135591 | Collagen type I alpha 1 chain OS=Haplochromis burtoni OX=8153 PE=4 SV=1 |
| 20 | 67575 | tr\|A0A674DLV9\|A0A674DLV9\_SALTR | 46.23 | 1 | 1 | 1 | N | 135355 | Collagen alpha-1(I) chain-like OS=Salmo trutta OX=8032 GN=LOC115170991 PE=4 SV=1 |
| 20 | 67276 | tr\|A0A3Q3CY74\|A0A3Q3CY74\_HAPBU | 46.23 | 1 | 1 | 1 | N | 137155 | Collagen type I alpha 1 chain OS=Haplochromis burtoni OX=8153 PE=4 SV=1 |
| 20 | 67019 | tr\|G9M6I5\|G9M6I5\_ORENI | 46.23 | 1 | 1 | 1 | N | 137283 | Collagen type I alpha 1 OS=Oreochromis niloticus OX=8128 GN=COL1A1 PE=2 SV=1 |
| 20 | 67576 | tr\|A0A674DL09\|A0A674DL09\_SALTR | 46.23 | 1 | 1 | 1 | N | 136924 | Collagen alpha-1(I) chain-like OS=Salmo trutta OX=8032 GN=LOC115170991 PE=4 SV=1 |
| 20 | 67577 | tr\|Q910C0\|Q910C0\_ONCMY | 46.23 | 1 | 1 | 1 | N | 137117 | Collagen a1(I) OS=Oncorhynchus mykiss OX=8022 GN=COL1A1 PE=2 SV=1 |
| 20 | 67578 | tr\|A0A1S3S6G4\|A0A1S3S6G4\_SALSA | 46.23 | 1 | 1 | 1 | N | 136936 | collagen alpha-1(I) chain OS=Salmo salar OX=8030 GN=LOC100286406 PE=4 SV=1 |
| 20 | 67579 | tr\|A0A6J2RRG5\|A0A6J2RRG5\_COTGO | 46.23 | 1 | 1 | 1 | N | 136726 | collagen alpha-1(I) chain-like OS=Cottoperca gobio OX=56716 GN=LOC115024512 PE=4 SV=1 |
| 20 | 67580 | tr\|A0A1S3R8F9\|A0A1S3R8F9\_SALSA | 46.23 | 1 | 1 | 1 | N | 136917 | collagen alpha-1(I) chain-like OS=Salmo salar OX=8030 GN=LOC106600852 PE=4 SV=1 |
| 10 | 69550 | tr\|A0A1S3NGD5\|A0A1S3NGD5\_SALSA | 45.17 | 2 | 3 | 1 | N | 127246 | myosin-binding protein C, fast-type-like OS=Salmo salar OX=8030 GN=LOC106579178 PE=4 SV=1 |
| 10 | 69563 | tr\|A0A1A7W9Y4\|A0A1A7W9Y4\_9TELE | 45.17 | 2 | 3 | 1 | N | 124706 | Myosin binding protein C, fast type b OS=Iconisemion striatum OX=60296 GN=MYBPC2B PE=4 SV=1 |
| 7 | 69552 | tr\|A0A0F8B178\|A0A0F8B178\_LARCR | 44.42 | 4 | 4 | 4 | N | 108080 | Calcium-transporting ATPase OS=Larimichthys crocea OX=215358 GN=EH28\_02549 PE=3 SV=1 |
| 7 | 69553 | tr\|A0A3P8PD71\|A0A3P8PD71\_ASTCA | 44.42 | 4 | 4 | 4 | N | 108538 | Calcium-transporting ATPase OS=Astatotilapia calliptera OX=8154 PE=3 SV=1 |
| 7 | 67215 | tr\|A0A3Q2WQG2\|A0A3Q2WQG2\_HAPBU | 44.42 | 4 | 4 | 4 | N | 108554 | Calcium-transporting ATPase OS=Haplochromis burtoni OX=8153 PE=3 SV=1 |
| 7 | 69554 | tr\|A0A6G0HZ09\|A0A6G0HZ09\_LARCR | 44.42 | 4 | 4 | 4 | N | 108647 | Calcium-transporting ATPase OS=Larimichthys crocea OX=215358 GN=D5F01\_LYC17582 PE=3 SV=1 |
| 7 | 69555 | tr\|A0A3B4GKY8\|A0A3B4GKY8\_9CICH | 44.42 | 4 | 4 | 4 | N | 108592 | Calcium-transporting ATPase OS=Pundamilia nyererei OX=303518 PE=3 SV=1 |
| 7 | 69556 | tr\|A0A3P9C8S8\|A0A3P9C8S8\_9CICH | 44.42 | 4 | 4 | 4 | N | 108556 | Calcium-transporting ATPase OS=Maylandia zebra OX=106582 PE=3 SV=1 |
| 7 | 69557 | tr\|A0A3P8PD17\|A0A3P8PD17\_ASTCA | 44.42 | 4 | 4 | 4 | N | 109053 | Calcium-transporting ATPase OS=Astatotilapia calliptera OX=8154 PE=3 SV=1 |
| 7 | 69558 | tr\|A0A3B4GL15\|A0A3B4GL15\_9CICH | 44.42 | 4 | 4 | 4 | N | 109107 | Calcium-transporting ATPase OS=Pundamilia nyererei OX=303518 PE=3 SV=1 |
| 7 | 67219 | tr\|A0A3P9C9M1\|A0A3P9C9M1\_9CICH | 44.42 | 4 | 4 | 4 | N | 109087 | Calcium-transporting ATPase OS=Maylandia zebra OX=106582 PE=3 SV=1 |
| 7 | 69559 | tr\|A0A3Q2WG66\|A0A3Q2WG66\_HAPBU | 44.42 | 4 | 4 | 4 | N | 109069 | Calcium-transporting ATPase OS=Haplochromis burtoni OX=8153 PE=3 SV=1 |
| 7 | 69560 | tr\|A0A3P8PDJ9\|A0A3P8PDJ9\_ASTCA | 44.42 | 4 | 4 | 4 | N | 110865 | Calcium-transporting ATPase OS=Astatotilapia calliptera OX=8154 PE=3 SV=1 |
| 7 | 69561 | tr\|A0A3P8PDC7\|A0A3P8PDC7\_ASTCA | 44.42 | 4 | 4 | 4 | N | 111173 | Calcium-transporting ATPase OS=Astatotilapia calliptera OX=8154 PE=3 SV=1 |
| 7 | 69562 | tr\|A0A3Q2WG59\|A0A3Q2WG59\_HAPBU | 44.42 | 4 | 4 | 4 | N | 111189 | Calcium-transporting ATPase OS=Haplochromis burtoni OX=8153 PE=3 SV=1 |
| 27 | 69493 | tr\|A0A3P8P4C2\|A0A3P8P4C2\_ASTCA | 35.05 | 1 | 1 | 1 | N | 157122 | Myomesin-2-like OS=Astatotilapia calliptera OX=8154 GN=MYOM2 PE=4 SV=1 |
| 27 | 69494 | tr\|A0A3Q1D109\|A0A3Q1D109\_AMPOC | 35.05 | 1 | 1 | 1 | N | 156426 | Myomesin-2-like OS=Amphiprion ocellaris OX=80972 GN=MYOM2 PE=4 SV=1 |
| 27 | 69495 | tr\|A0A3Q2WWP1\|A0A3Q2WWP1\_HAPBU | 35.05 | 1 | 1 | 1 | N | 156996 | Myomesin-2-like OS=Haplochromis burtoni OX=8153 PE=4 SV=1 |
| 27 | 69519 | tr\|A0A3Q4GHK3\|A0A3Q4GHK3\_NEOBR | 35.05 | 2 | 1 | 1 | N | 99617 | Myomesin-2-like OS=Neolamprologus brichardi OX=32507 GN=MYOM2 PE=4 SV=1 |
| 27 | 69520 | tr\|A0A672ZI78\|A0A672ZI78\_9TELE | 35.05 | 1 | 1 | 1 | N | 126360 | Uncharacterized protein OS=Sphaeramia orbicularis OX=375764 GN=LOC115437523 PE=4 SV=1 |
| 27 | 69521 | tr\|A0A669ELF6\|A0A669ELF6\_ORENI | 35.05 | 1 | 1 | 1 | N | 132152 | Uncharacterized protein OS=Oreochromis niloticus OX=8128 GN=LOC100698303 PE=4 SV=1 |
| 27 | 69522 | tr\|A0A4U5U6X9\|A0A4U5U6X9\_COLLU | 35.05 | 1 | 1 | 1 | N | 136304 | Myomesin-2 165 kDa connectin-associated protein 165 kDa titin-associated protein M-protein OS=Collichthys lucidus OX=240159 GN=D9C73\_004091 PE=4 SV=1 |
| 27 | 69523 | tr\|A0A3Q4GXU7\|A0A3Q4GXU7\_NEOBR | 35.05 | 1 | 1 | 1 | N | 139979 | Myomesin-2-like OS=Neolamprologus brichardi OX=32507 GN=MYOM2 PE=4 SV=1 |
| 27 | 69524 | tr\|A0A669CKL2\|A0A669CKL2\_ORENI | 35.05 | 1 | 1 | 1 | N | 141158 | Uncharacterized protein OS=Oreochromis niloticus OX=8128 GN=LOC100698303 PE=4 SV=1 |
| 27 | 69525 | tr\|A0A671Y401\|A0A671Y401\_SPAAU | 35.05 | 1 | 1 | 1 | N | 145648 | Myomesin-2-like OS=Sparus aurata OX=8175 GN=MYOM2 PE=4 SV=1 |
| 27 | 69526 | tr\|A0A672FNF5\|A0A672FNF5\_SALFA | 35.05 | 1 | 1 | 1 | N | 148203 | Uncharacterized protein OS=Salarias fasciatus OX=181472 GN=LOC115388674 PE=4 SV=1 |
| 27 | 69527 | tr\|A0A6G0J0W8\|A0A6G0J0W8\_LARCR | 35.05 | 1 | 1 | 1 | N | 152797 | Myomesin-2 165 kDa connectin-associated protein OS=Larimichthys crocea OX=215358 GN=D5F01\_LYC03862 PE=4 SV=1 |
| 27 | 69528 | tr\|A0A671Y575\|A0A671Y575\_SPAAU | 35.05 | 1 | 1 | 1 | N | 154320 | Myomesin-2-like OS=Sparus aurata OX=8175 GN=MYOM2 PE=4 SV=1 |
| 27 | 69529 | tr\|A0A3B3IJA0\|A0A3B3IJA0\_ORYLA | 35.05 | 1 | 1 | 1 | N | 155226 | Uncharacterized protein OS=Oryzias latipes OX=8090 GN=LOC101163890 PE=4 SV=1 |
| 27 | 69530 | tr\|A0A6G1P7U8\|A0A6G1P7U8\_9TELE | 35.05 | 1 | 1 | 1 | N | 156806 | Myomesin-2 165 kDa connectin-associated protein OS=Channa argus OX=215402 GN=EXN66\_Car002007 PE=4 SV=1 |
| 27 | 69531 | tr\|A0A087YFH0\|A0A087YFH0\_POEFO | 35.05 | 1 | 1 | 1 | N | 156966 | Myomesin-2-like OS=Poecilia formosa OX=48698 GN=MYOM2 PE=4 SV=2 |
| 27 | 69532 | tr\|A0A3P9PGW1\|A0A3P9PGW1\_POERE | 35.05 | 1 | 1 | 1 | N | 156917 | Myomesin-2-like OS=Poecilia reticulata OX=8081 PE=4 SV=1 |
| 27 | 69533 | tr\|A0A672ZHV0\|A0A672ZHV0\_9TELE | 35.05 | 1 | 1 | 1 | N | 156079 | Uncharacterized protein OS=Sphaeramia orbicularis OX=375764 GN=LOC115437523 PE=4 SV=1 |
| 27 | 69534 | tr\|I3JDJ1\|I3JDJ1\_ORENI | 35.05 | 1 | 1 | 1 | N | 157148 | Uncharacterized protein OS=Oreochromis niloticus OX=8128 GN=LOC100698303 PE=4 SV=2 |
| 27 | 69535 | tr\|A0A2I4AP94\|A0A2I4AP94\_9TELE | 35.05 | 1 | 1 | 1 | N | 156788 | M-protein, striated muscle-like OS=Austrofundulus limnaeus OX=52670 GN=LOC106513170 PE=4 SV=1 |
| 27 | 69536 | tr\|A0A834CMG6\|A0A834CMG6\_ORYME | 35.05 | 1 | 1 | 1 | N | 157078 | Myomesin-2 OS=Oryzias melastigma OX=30732 GN=FQA47\_006815 PE=4 SV=1 |
| 27 | 69537 | tr\|A0A3B3BCL7\|A0A3B3BCL7\_ORYME | 35.05 | 1 | 1 | 1 | N | 157034 | Myomesin-2-like OS=Oryzias melastigma OX=30732 PE=4 SV=1 |
| 27 | 69538 | tr\|A0A3P9IT90\|A0A3P9IT90\_ORYLA | 35.05 | 1 | 1 | 1 | N | 156872 | Uncharacterized protein OS=Oryzias latipes OX=8090 PE=4 SV=1 |
| 27 | 69539 | tr\|H2M2B3\|H2M2B3\_ORYLA | 35.05 | 1 | 1 | 1 | N | 156953 | Uncharacterized protein OS=Oryzias latipes OX=8090 GN=LOC101163890 PE=4 SV=2 |
| 27 | 69540 | tr\|A0A3P9LTE0\|A0A3P9LTE0\_ORYLA | 35.05 | 1 | 1 | 1 | N | 157011 | Uncharacterized protein OS=Oryzias latipes OX=8090 PE=4 SV=1 |
| 27 | 69541 | tr\|A0A3B3D3C5\|A0A3B3D3C5\_ORYME | 35.05 | 1 | 1 | 1 | N | 157566 | Myomesin-2-like OS=Oryzias melastigma OX=30732 PE=4 SV=1 |
| 27 | 69542 | tr\|A0A6P7LYN0\|A0A6P7LYN0\_BETSP | 35.05 | 1 | 1 | 1 | N | 158082 | myomesin-2-like isoform X1 OS=Betta splendens OX=158456 GN=LOC114852036 PE=4 SV=1 |
| 27 | 69543 | tr\|A0A3B3HWS2\|A0A3B3HWS2\_ORYLA | 35.05 | 1 | 1 | 1 | N | 158285 | Uncharacterized protein OS=Oryzias latipes OX=8090 GN=LOC101163890 PE=4 SV=1 |
| 16 | 69651 | tr\|A0A665WT54\|A0A665WT54\_ECHNA | 34.94 | 7 | 2 | 2 | N | 12976 | LIM domain-binding protein 3-like OS=Echeneis naucrates OX=173247 GN=LOC115060026 PE=4 SV=1 |
| 16 | 69652 | tr\|A0A671N228\|A0A671N228\_9TELE | 34.94 | 3 | 2 | 2 | N | 28684 | LIM domain-binding protein 3-like OS=Sinocyclocheilus anshuiensis OX=1608454 GN=LOC107698793 PE=4 SV=1 |
| 16 | 69653 | tr\|A0A3Q3Q7K2\|A0A3Q3Q7K2\_MONAL | 34.94 | 3 | 2 | 2 | N | 29692 | Synuclein-like OS=Monopterus albus OX=43700 PE=4 SV=1 |
| 16 | 69654 | tr\|A0A671MU99\|A0A671MU99\_9TELE | 34.94 | 3 | 2 | 2 | N | 29538 | LIM domain-binding protein 3-like OS=Sinocyclocheilus anshuiensis OX=1608454 GN=LOC107698793 PE=4 SV=1 |
| 16 | 69655 | tr\|A0A672KK19\|A0A672KK19\_SINGR | 34.94 | 3 | 2 | 2 | N | 30329 | LIM domain-binding protein 3-like OS=Sinocyclocheilus grahami OX=75366 GN=LOC107552745 PE=4 SV=1 |
| 16 | 69656 | tr\|A0A673LYE6\|A0A673LYE6\_9TELE | 34.94 | 3 | 2 | 2 | N | 30316 | LIM domain-binding protein 3-like OS=Sinocyclocheilus rhinocerous OX=307959 GN=LOC107749342 PE=4 SV=1 |
| 16 | 69657 | tr\|A0A3P8VEA1\|A0A3P8VEA1\_CYNSE | 34.94 | 3 | 2 | 2 | N | 30334 | LIM domain-binding protein 3-like OS=Cynoglossus semilaevis OX=244447 PE=4 SV=1 |
| 16 | 69658 | tr\|A0A3Q1II99\|A0A3Q1II99\_ANATE | 34.94 | 3 | 2 | 2 | N | 30310 | LIM domain binding 3b OS=Anabas testudineus OX=64144 PE=4 SV=1 |
| 16 | 69659 | tr\|A0A6P7L9I7\|A0A6P7L9I7\_BETSP | 34.94 | 3 | 2 | 2 | N | 30536 | LIM domain-binding protein 3-like isoform X2 OS=Betta splendens OX=158456 GN=LOC114846412 PE=4 SV=1 |
| 16 | 69660 | tr\|A0A3P8X8T4\|A0A3P8X8T4\_ESOLU | 34.94 | 3 | 2 | 2 | N | 30572 | LIM domain-binding protein 3 OS=Esox lucius OX=8010 PE=4 SV=1 |
| 16 | 69661 | tr\|A0A3Q0R4X6\|A0A3Q0R4X6\_AMPCI | 34.94 | 3 | 2 | 2 | N | 30403 | LIM domain binding 3b OS=Amphilophus citrinellus OX=61819 PE=4 SV=1 |
| 16 | 69662 | tr\|A0A3P8VK01\|A0A3P8VK01\_CYNSE | 34.94 | 3 | 2 | 2 | N | 30591 | LIM domain-binding protein 3-like OS=Cynoglossus semilaevis OX=244447 PE=4 SV=1 |
| 16 | 69663 | tr\|A0A3P8XBI5\|A0A3P8XBI5\_ESOLU | 34.94 | 3 | 2 | 2 | N | 30957 | LIM domain-binding protein 3 OS=Esox lucius OX=8010 PE=4 SV=2 |
| 16 | 69664 | tr\|A0A3Q1H919\|A0A3Q1H919\_ANATE | 34.94 | 3 | 2 | 2 | N | 31065 | LIM domain binding 3b OS=Anabas testudineus OX=64144 PE=4 SV=1 |
| 16 | 69665 | tr\|A0A3Q3KN21\|A0A3Q3KN21\_9TELE | 34.94 | 3 | 2 | 2 | N | 31352 | LIM domain binding 3b OS=Mastacembelus armatus OX=205130 PE=4 SV=2 |
| 16 | 69666 | tr\|A0A6P7L9I9\|A0A6P7L9I9\_BETSP | 34.94 | 3 | 2 | 2 | N | 31290 | LIM domain-binding protein 3-like isoform X1 OS=Betta splendens OX=158456 GN=LOC114846412 PE=4 SV=1 |
| 16 | 69667 | tr\|A0A3P8VE68\|A0A3P8VE68\_CYNSE | 34.94 | 3 | 2 | 2 | N | 31336 | LIM domain-binding protein 3-like OS=Cynoglossus semilaevis OX=244447 PE=4 SV=1 |
| 16 | 69668 | tr\|A0A6G0HYN5\|A0A6G0HYN5\_LARCR | 34.94 | 3 | 2 | 2 | N | 31123 | LIM domain-binding protein 3 Protein cypher Z-band alternatively spliced PDZ-motif protein OS=Larimichthys crocea OX=215358 GN=D5F01\_LYC17526 PE=4 SV=1 |
| 16 | 69669 | tr\|A0A3P8ZCR8\|A0A3P8ZCR8\_ESOLU | 34.94 | 3 | 2 | 2 | N | 31398 | LIM domain-binding protein 3 OS=Esox lucius OX=8010 PE=4 SV=1 |
| 16 | 69670 | tr\|A0A3Q3IQV7\|A0A3Q3IQV7\_MONAL | 34.94 | 3 | 2 | 2 | N | 31456 | Synuclein-like OS=Monopterus albus OX=43700 PE=4 SV=1 |
| 16 | 69671 | tr\|A0A665WTS5\|A0A665WTS5\_ECHNA | 34.94 | 3 | 2 | 2 | N | 31290 | LIM domain-binding protein 3-like OS=Echeneis naucrates OX=173247 GN=LOC115060026 PE=4 SV=1 |
| 16 | 69672 | tr\|A0A7N8Y585\|A0A7N8Y585\_9TELE | 34.94 | 3 | 2 | 2 | N | 31804 | LIM domain binding 3b OS=Mastacembelus armatus OX=205130 PE=4 SV=1 |
| 16 | 69673 | tr\|A0A6P6QFT2\|A0A6P6QFT2\_CARAU | 34.94 | 3 | 2 | 2 | N | 31663 | LIM domain-binding protein 3-like isoform X2 OS=Carassius auratus OX=7957 GN=LOC113111546 PE=4 SV=1 |
| 16 | 69674 | tr\|A0A672KNU9\|A0A672KNU9\_SINGR | 34.94 | 3 | 2 | 2 | N | 31638 | LIM domain-binding protein 3-like OS=Sinocyclocheilus grahami OX=75366 GN=LOC107552745 PE=4 SV=1 |
| 16 | 69675 | tr\|A0A673M1Q3\|A0A673M1Q3\_9TELE | 34.94 | 3 | 2 | 2 | N | 31625 | LIM domain-binding protein 3-like OS=Sinocyclocheilus rhinocerous OX=307959 GN=LOC107749342 PE=4 SV=1 |
| 16 | 69676 | tr\|A0A7J6CGV6\|A0A7J6CGV6\_9TELE | 34.94 | 3 | 2 | 2 | N | 31765 | PDZ domain-containing protein OS=Onychostoma macrolepis OX=369639 GN=G5714\_012537 PE=4 SV=1 |
| 16 | 69677 | tr\|A0A6P6QIW1\|A0A6P6QIW1\_CARAU | 34.94 | 3 | 2 | 2 | N | 34204 | LIM domain-binding protein 3-like isoform X1 OS=Carassius auratus OX=7957 GN=LOC113111546 PE=4 SV=1 |
| 16 | 69678 | tr\|A0A6Q2XWF7\|A0A6Q2XWF7\_ESOLU | 34.94 | 3 | 2 | 2 | N | 35095 | LIM domain-binding protein 3 OS=Esox lucius OX=8010 PE=4 SV=1 |
| 16 | 69679 | tr\|A0A0F8AMH6\|A0A0F8AMH6\_LARCR | 34.94 | 2 | 2 | 2 | N | 43721 | LIM domain-binding protein 3 OS=Larimichthys crocea OX=215358 GN=EH28\_03737 PE=4 SV=1 |
| 16 | 69680 | tr\|A0A6G0HYU6\|A0A6G0HYU6\_LARCR | 34.94 | 1 | 2 | 2 | N | 80212 | Melanopsin-A Mammalian-like melanopsin Melanopsin-M Opsin-4-A Opsin-4M OS=Larimichthys crocea OX=215358 GN=D5F01\_LYC17520 PE=4 SV=1 |
| 23 | 69577 | tr\|A0A0P7UGN8\|A0A0P7UGN8\_SCLFO | 30.17 | 2 | 1 | 1 | N | 52152 | Arrestin domain-containing protein 1-like OS=Scleropages formosus OX=113540 GN=Z043\_114076 PE=3 SV=1 |
| 23 | 69587 | tr\|A0A3P8UKD4\|A0A3P8UKD4\_CYNSE | 30.17 | 6 | 1 | 1 | N | 15153 | Ig-like domain-containing protein OS=Cynoglossus semilaevis OX=244447 PE=4 SV=1 |
| 23 | 69588 | tr\|A0A668SIM0\|A0A668SIM0\_OREAU | 30.17 | 2 | 1 | 1 | N | 39658 | Ig-like domain-containing protein OS=Oreochromis aureus OX=47969 PE=4 SV=1 |
| 23 | 69589 | tr\|A0A6P7NE86\|A0A6P7NE86\_BETSP | 30.17 | 2 | 1 | 1 | N | 40039 | arrestin domain-containing protein 1 isoform X2 OS=Betta splendens OX=158456 GN=arrdc1 PE=3 SV=1 |
| 23 | 69590 | tr\|A0A5J5CFY0\|A0A5J5CFY0\_9PERO | 30.17 | 2 | 1 | 1 | N | 43851 | Uncharacterized protein (Fragment) OS=Etheostoma spectabile OX=54343 GN=FQN60\_006042 PE=4 SV=1 |
| 23 | 69591 | tr\|A0A315V5I0\|A0A315V5I0\_GAMAF | 30.17 | 2 | 1 | 1 | N | 48027 | Arrestin\_C domain-containing protein (Fragment) OS=Gambusia affinis OX=33528 GN=CCH79\_00009980 PE=3 SV=1 |
| 23 | 69592 | tr\|A0A6P7NIN0\|A0A6P7NIN0\_BETSP | 30.17 | 2 | 1 | 1 | N | 48523 | arrestin domain-containing protein 1 isoform X1 OS=Betta splendens OX=158456 GN=arrdc1 PE=3 SV=1 |
| 23 | 69593 | tr\|A0A6J2QDT9\|A0A6J2QDT9\_COTGO | 30.17 | 2 | 1 | 1 | N | 48759 | arrestin domain-containing protein 1-like OS=Cottoperca gobio OX=56716 GN=LOC115013564 PE=3 SV=1 |
| 23 | 69594 | tr\|M3ZP84\|M3ZP84\_XIPMA | 30.17 | 2 | 1 | 1 | N | 49386 | Arrestin\_C domain-containing protein OS=Xiphophorus maculatus OX=8083 PE=3 SV=2 |
| 23 | 69595 | tr\|A0A3P9PCX4\|A0A3P9PCX4\_POERE | 30.17 | 2 | 1 | 1 | N | 49543 | Arrestin domain-containing protein 1-like OS=Poecilia reticulata OX=8081 PE=3 SV=1 |
| 23 | 69596 | tr\|A0A3B3WUC6\|A0A3B3WUC6\_9TELE | 30.17 | 2 | 1 | 1 | N | 49610 | Arrestin domain-containing protein 1-like OS=Poecilia mexicana OX=48701 PE=3 SV=1 |
| 23 | 69612 | tr\|A0A6G0J5Q9\|A0A6G0J5Q9\_LARCR | 30.17 | 0 | 1 | 1 | N | 928406 | Titin OS=Larimichthys crocea OX=215358 GN=D5F01\_LYC01467 PE=4 SV=1 |
| 13 | 69619 | tr\|A0A6J2RVQ6\|A0A6J2RVQ6\_COTGO | 28.13 | 0 | 2 | 2 | N | 676713 | nebulin isoform X14 OS=Cottoperca gobio OX=56716 GN=neb PE=4 SV=1 |
| 13 | 69620 | tr\|A0A6J2RYJ1\|A0A6J2RYJ1\_COTGO | 28.13 | 0 | 2 | 2 | N | 678455 | nebulin isoform X13 OS=Cottoperca gobio OX=56716 GN=neb PE=4 SV=1 |
| 13 | 69621 | tr\|A0A6J2RTY3\|A0A6J2RTY3\_COTGO | 28.13 | 0 | 2 | 2 | N | 691692 | nebulin isoform X12 OS=Cottoperca gobio OX=56716 GN=neb PE=4 SV=1 |
| 13 | 69622 | tr\|A0A6J2RY66\|A0A6J2RY66\_COTGO | 28.13 | 0 | 2 | 2 | N | 698051 | nebulin isoform X11 OS=Cottoperca gobio OX=56716 GN=neb PE=4 SV=1 |
| 13 | 69623 | tr\|A0A6J2RVQ1\|A0A6J2RVQ1\_COTGO | 28.13 | 0 | 2 | 2 | N | 701558 | nebulin isoform X10 OS=Cottoperca gobio OX=56716 GN=neb PE=4 SV=1 |
| 13 | 69624 | tr\|A0A3Q1HC41\|A0A3Q1HC41\_ANATE | 28.13 | 0 | 2 | 2 | N | 704841 | Nebulin OS=Anabas testudineus OX=64144 GN=NEB PE=4 SV=2 |
| 13 | 69625 | tr\|A0A6J2RYI7\|A0A6J2RYI7\_COTGO | 28.13 | 0 | 2 | 2 | N | 705639 | nebulin isoform X9 OS=Cottoperca gobio OX=56716 GN=neb PE=4 SV=1 |
| 13 | 69626 | tr\|A0A6J2RWT1\|A0A6J2RWT1\_COTGO | 28.13 | 0 | 2 | 2 | N | 712677 | nebulin isoform X8 OS=Cottoperca gobio OX=56716 GN=neb PE=4 SV=1 |
| 13 | 69627 | tr\|A0A6J2RY61\|A0A6J2RY61\_COTGO | 28.13 | 0 | 2 | 2 | N | 715609 | nebulin isoform X6 OS=Cottoperca gobio OX=56716 GN=neb PE=4 SV=1 |
| 13 | 69628 | tr\|A0A6J2RYI1\|A0A6J2RYI1\_COTGO | 28.13 | 0 | 2 | 2 | N | 716207 | nebulin isoform X4 OS=Cottoperca gobio OX=56716 GN=neb PE=4 SV=1 |
| 13 | 69629 | tr\|A0A6J2RVP6\|A0A6J2RVP6\_COTGO | 28.13 | 0 | 2 | 2 | N | 716181 | nebulin isoform X5 OS=Cottoperca gobio OX=56716 GN=neb PE=4 SV=1 |
| 13 | 69630 | tr\|A0A6J2RY55\|A0A6J2RY55\_COTGO | 28.13 | 0 | 2 | 2 | N | 716421 | nebulin isoform X3 OS=Cottoperca gobio OX=56716 GN=neb PE=4 SV=1 |
| 13 | 69631 | tr\|A0A6J2RVP0\|A0A6J2RVP0\_COTGO | 28.13 | 0 | 2 | 2 | N | 717944 | nebulin isoform X2 OS=Cottoperca gobio OX=56716 GN=neb PE=4 SV=1 |
| 13 | 69632 | tr\|A0A6J2RWS0\|A0A6J2RWS0\_COTGO | 28.13 | 0 | 2 | 2 | N | 719690 | nebulin isoform X1 OS=Cottoperca gobio OX=56716 GN=neb PE=4 SV=1 |
| 13 | 69633 | tr\|A0A6G0J8D3\|A0A6G0J8D3\_LARCR | 28.13 | 0 | 2 | 2 | N | 592244 | Nebulin OS=Larimichthys crocea OX=215358 GN=D5F01\_LYC02135 PE=4 SV=1 |
| 13 | 69634 | tr\|A0A5C6PNB9\|A0A5C6PNB9\_9TELE | 28.13 | 0 | 2 | 2 | N | 690778 | Nebulin OS=Takifugu flavidus OX=433684 GN=D4764\_01G0002010 PE=4 SV=1 |
| 13 | 69635 | tr\|A0A669CKX5\|A0A669CKX5\_ORENI | 28.13 | 0 | 2 | 2 | N | 700756 | Nebulin OS=Oreochromis niloticus OX=8128 PE=4 SV=1 |
| 13 | 69636 | tr\|I3KK23\|I3KK23\_ORENI | 28.13 | 0 | 2 | 2 | N | 705912 | Nebulin OS=Oreochromis niloticus OX=8128 PE=4 SV=2 |
| 13 | 18868 | tr\|A0A3P8QJ88\|A0A3P8QJ88\_ASTCA | 28.13 | 0 | 2 | 2 | N | 709333 | Nebulin OS=Astatotilapia calliptera OX=8154 PE=4 SV=1 |
| 13 | 69637 | tr\|A0A3P9CU79\|A0A3P9CU79\_9CICH | 28.13 | 0 | 2 | 2 | N | 709930 | Nebulin OS=Maylandia zebra OX=106582 PE=4 SV=1 |
| 13 | 69638 | tr\|A0A6J2RTX8\|A0A6J2RTX8\_COTGO | 28.13 | 0 | 2 | 2 | N | 715423 | nebulin isoform X7 OS=Cottoperca gobio OX=56716 GN=neb PE=4 SV=1 |
| 13 | 69639 | tr\|A0A4U5U1Y5\|A0A4U5U1Y5\_COLLU | 28.13 | 0 | 2 | 2 | N | 731042 | Nebulin OS=Collichthys lucidus OX=240159 GN=D9C73\_000886 PE=4 SV=1 |
| 24 | 69583 | tr\|A0A4W4F305\|A0A4W4F305\_ELEEL | 28.12 | 0 | 1 | 1 | N | 373023 | Uncharacterized protein OS=Electrophorus electricus OX=8005 GN=LOC113571677 PE=4 SV=1 |
| 24 | 69611 | tr\|W5KZP4\|W5KZP4\_ASTMX | 28.12 | 1 | 1 | 1 | N | 116741 | Collagen, type XXVIII, alpha 2b OS=Astyanax mexicanus OX=7994 PE=4 SV=2 |
| 22 | 67639 | tr\|A0A6P3VUU5\|A0A6P3VUU5\_CLUHA | 26.91 | 1 | 1 | 1 | N | 141605 | collagen alpha-1(II) chain-like isoform X1 OS=Clupea harengus OX=7950 GN=LOC105899289 PE=4 SV=1 |
| 28 | 68490 | tr\|A0A6J2PWT2\|A0A6J2PWT2\_COTGO | 26.90 | 4 | 1 | 1 | N | 27686 | troponin T, fast skeletal muscle isoforms-like isoform X4 OS=Cottoperca gobio OX=56716 GN=LOC115009585 PE=3 SV=1 |
| 28 | 68487 | tr\|A0A6J2PTP5\|A0A6J2PTP5\_COTGO | 26.90 | 4 | 1 | 1 | N | 27279 | troponin T, fast skeletal muscle isoforms-like isoform X5 OS=Cottoperca gobio OX=56716 GN=LOC115009585 PE=3 SV=1 |
| 28 | 68543 | tr\|A0A6J2PW38\|A0A6J2PW38\_COTGO | 26.90 | 4 | 1 | 1 | N | 33340 | troponin T, fast skeletal muscle isoforms-like isoform X3 OS=Cottoperca gobio OX=56716 GN=LOC115009585 PE=3 SV=1 |
| 28 | 68546 | tr\|A0A6J2PTY9\|A0A6J2PTY9\_COTGO | 26.90 | 3 | 1 | 1 | N | 33991 | LOW QUALITY PROTEIN: troponin T, fast skeletal muscle isoforms-like OS=Cottoperca gobio OX=56716 GN=LOC115009631 PE=3 SV=1 |
| 28 | 68547 | tr\|A0A6J2PVR7\|A0A6J2PVR7\_COTGO | 26.90 | 3 | 1 | 1 | N | 33971 | troponin T, fast skeletal muscle isoforms-like isoform X2 OS=Cottoperca gobio OX=56716 GN=LOC115009585 PE=3 SV=1 |
| 28 | 68549 | tr\|A0A6J2PTN9\|A0A6J2PTN9\_COTGO | 26.90 | 3 | 1 | 1 | N | 34378 | troponin T, fast skeletal muscle isoforms-like isoform X1 OS=Cottoperca gobio OX=56716 GN=LOC115009585 PE=3 SV=1 |
| 28 | 69618 | tr\|A0A5A9PG84\|A0A5A9PG84\_9TELE | 26.90 | 3 | 1 | 1 | N | 39345 | Troponin T, cardiac muscle OS=Triplophysa tibetana OX=1572043 GN=E1301\_Tti002088 PE=3 SV=1 |
| 29 | 67404 | tr\|A0A6F9CEX9\|A0A6F9CEX9\_9TELE | 26.71 | 0 | 1 | 1 | N | 1022296 | Uncharacterized protein OS=Coregonus sp. 'balchen' OX=861768 GN=CSTEINMANNI\_LOCUS3957823 PE=4 SV=1 |
| 19 | 69734 | tr\|A0A3B3ICW0\|A0A3B3ICW0\_ORYLA | 24.32 | 4 | 2 | 1 | N | 47229 | 2-phospho-D-glycerate hydro-lyase OS=Oryzias latipes OX=8090 GN=eno1 PE=3 SV=1 |
| 30 | 67800 | tr\|A0A3Q4GK85\|A0A3Q4GK85\_NEOBR | 24.20 | 6 | 1 | 1 | N | 21230 | Adenylate kinase isoenzyme 1 OS=Neolamprologus brichardi OX=32507 GN=AK1 PE=3 SV=1 |
| 30 | 67801 | tr\|A0A3Q4GS43\|A0A3Q4GS43\_NEOBR | 24.20 | 6 | 1 | 1 | N | 21240 | Adenylate kinase isoenzyme 1 OS=Neolamprologus brichardi OX=32507 GN=AK1 PE=3 SV=1 |
| 30 | 67802 | tr\|A0A3P8P8S3\|A0A3P8P8S3\_ASTCA | 24.20 | 6 | 1 | 1 | N | 21228 | Adenylate kinase isoenzyme 1 OS=Astatotilapia calliptera OX=8154 GN=AK1 PE=3 SV=1 |
| 30 | 67795 | tr\|A0A3Q1HW42\|A0A3Q1HW42\_9TELE | 24.20 | 6 | 1 | 1 | N | 21205 | Adenylate kinase isoenzyme 1 OS=Acanthochromis polyacanthus OX=80966 GN=AK1 PE=3 SV=1 |
| 30 | 67803 | tr\|A0A3P9CWL7\|A0A3P9CWL7\_9CICH | 24.20 | 6 | 1 | 1 | N | 21258 | Adenylate kinase isoenzyme 1 OS=Maylandia zebra OX=106582 GN=AK1 PE=3 SV=1 |
| 30 | 67796 | tr\|A0A3B5BIY5\|A0A3B5BIY5\_9TELE | 24.20 | 6 | 1 | 1 | N | 21206 | Adenylate kinase isoenzyme 1 OS=Stegastes partitus OX=144197 GN=AK1 PE=3 SV=1 |
| 30 | 67804 | tr\|A0A3Q2VY92\|A0A3Q2VY92\_HAPBU | 24.20 | 6 | 1 | 1 | N | 21228 | Adenylate kinase isoenzyme 1 OS=Haplochromis burtoni OX=8153 GN=AK1 PE=3 SV=1 |
| 30 | 67805 | tr\|A0A3B4FMK3\|A0A3B4FMK3\_9CICH | 24.20 | 6 | 1 | 1 | N | 21228 | Adenylate kinase isoenzyme 1 OS=Pundamilia nyererei OX=303518 GN=AK1 PE=3 SV=1 |
| 30 | 67806 | tr\|A0A3Q3CWR9\|A0A3Q3CWR9\_HAPBU | 24.20 | 5 | 1 | 1 | N | 23163 | Adenylate kinase isoenzyme 1 OS=Haplochromis burtoni OX=8153 GN=AK1 PE=3 SV=1 |
| 30 | 67807 | tr\|A0A3P8P8V3\|A0A3P8P8V3\_ASTCA | 24.20 | 5 | 1 | 1 | N | 23216 | Adenylate kinase isoenzyme 1 OS=Astatotilapia calliptera OX=8154 GN=AK1 PE=3 SV=1 |
| 14 | 69881 | tr\|A0A3Q1FHQ1\|A0A3Q1FHQ1\_9TELE | 23.71 | 0 | 2 | 1 | N | 545809 | Ryanodine receptor 3-like OS=Acanthochromis polyacanthus OX=80966 GN=RYR3 PE=4 SV=1 |
| 14 | 69882 | tr\|G3NYZ7\|G3NYZ7\_GASAC | 23.71 | 0 | 2 | 1 | N | 545639 | Ryanodine receptor 3 OS=Gasterosteus aculeatus OX=69293 PE=4 SV=1 |
| 14 | 69883 | tr\|A0A674BRX0\|A0A674BRX0\_SALTR | 23.71 | 0 | 2 | 1 | N | 549015 | Ryanodine receptor 3-like OS=Salmo trutta OX=8032 GN=RYR3 PE=4 SV=1 |
| 31 | 69681 | tr\|A0A0F8B8T7\|A0A0F8B8T7\_LARCR | 23.41 | 1 | 1 | 1 | N | 158653 | Collagen alpha-1(IV) chain OS=Larimichthys crocea OX=215358 GN=EH28\_07982 PE=4 SV=1 |
| 39 | 69682 | tr\|A0A665TL93\|A0A665TL93\_ECHNA | 22.82 | 2 | 1 | 1 | N | 107933 | Uncharacterized protein OS=Echeneis naucrates OX=173247 PE=4 SV=1 |
| 39 | 69683 | tr\|A0A7J5Y3X5\|A0A7J5Y3X5\_DISMA | 22.82 | 1 | 1 | 1 | N | 148241 | Uncharacterized protein OS=Dissostichus mawsoni OX=36200 GN=F7725\_002632 PE=4 SV=1 |
| 40 | 69686 | tr\|A0A3Q1G4M0\|A0A3Q1G4M0\_9TELE | 22.56 | 2 | 1 | 1 | N | 62757 | Collagen alpha chain-like OS=Acanthochromis polyacanthus OX=80966 PE=4 SV=1 |
| 41 | 69687 | tr\|A0A834FMH6\|A0A834FMH6\_ORYME | 21.61 | 3 | 1 | 1 | N | 29285 | DNA polymerase eta OS=Oryzias melastigma OX=30732 GN=FQA47\_004450 PE=4 SV=1 |
| 42 | 69694 | tr\|A0A673HU84\|A0A673HU84\_9TELE | 21.18 | 5 | 1 | 1 | N | 26537 | Uncharacterized protein OS=Sinocyclocheilus rhinocerous OX=307959 PE=3 SV=1 |
| 42 | 69696 | tr\|A0A2Z4XFW9\|A0A2Z4XFW9\_CTEID | 21.18 | 4 | 1 | 1 | N | 33893 | G-protein coupled estrogen receptor 1b (Fragment) OS=Ctenopharyngodon idella OX=7959 PE=2 SV=1 |
| 42 | 69697 | tr\|A0A4W4E5X7\|A0A4W4E5X7\_ELEEL | 21.18 | 3 | 1 | 1 | N | 37717 | G\_PROTEIN\_RECEP\_F1\_2 domain-containing protein OS=Electrophorus electricus OX=8005 PE=3 SV=1 |
| 42 | 69698 | tr\|A0A672KLI0\|A0A672KLI0\_SINGR | 21.18 | 3 | 1 | 1 | N | 38253 | G\_PROTEIN\_RECEP\_F1\_2 domain-containing protein OS=Sinocyclocheilus grahami OX=75366 PE=3 SV=1 |
| 42 | 69699 | tr\|A0A671RDB8\|A0A671RDB8\_9TELE | 21.18 | 3 | 1 | 1 | N | 39470 | G\_PROTEIN\_RECEP\_F1\_2 domain-containing protein OS=Sinocyclocheilus anshuiensis OX=1608454 PE=3 SV=1 |
| 42 | 69700 | tr\|W5LSJ6\|W5LSJ6\_ASTMX | 21.18 | 3 | 1 | 1 | N | 40447 | G\_PROTEIN\_RECEP\_F1\_2 domain-containing protein OS=Astyanax mexicanus OX=7994 PE=3 SV=2 |
| 42 | 69701 | tr\|A0A3B4C497\|A0A3B4C497\_PYGNA | 21.18 | 3 | 1 | 1 | N | 41083 | G-protein coupled estrogen receptor 1-like OS=Pygocentrus nattereri OX=42514 PE=3 SV=1 |
| 42 | 69702 | tr\|A0A6P3WBU3\|A0A6P3WBU3\_CLUHA | 21.18 | 3 | 1 | 1 | N | 41861 | G-protein coupled estrogen receptor 1-like OS=Clupea harengus OX=7950 GN=LOC105910498 PE=3 SV=1 |
| 42 | 69703 | tr\|A0A6P6J7W1\|A0A6P6J7W1\_CARAU | 21.18 | 3 | 1 | 1 | N | 43164 | G-protein coupled estrogen receptor 1-like isoform X2 OS=Carassius auratus OX=7957 GN=LOC113042077 PE=3 SV=1 |
| 42 | 69704 | tr\|A0A5A9N4U0\|A0A5A9N4U0\_9TELE | 21.18 | 3 | 1 | 1 | N | 43365 | G-protein coupled estrogen receptor 1 G protein-coupled estrogen receptor 1 OS=Triplophysa tibetana OX=1572043 GN=E1301\_Tti017821 PE=3 SV=1 |
| 42 | 69705 | tr\|A0A3N0XI69\|A0A3N0XI69\_ANAGA | 21.18 | 3 | 1 | 1 | N | 43107 | G-protein coupled estrogen receptor 1 OS=Anabarilius grahami OX=495550 GN=DPX16\_4475 PE=3 SV=1 |
| 42 | 69706 | tr\|A0A6J2UVU6\|A0A6J2UVU6\_CHACN | 21.18 | 3 | 1 | 1 | N | 44149 | G-protein coupled estrogen receptor 1-like OS=Chanos chanos OX=29144 GN=LOC115807694 PE=3 SV=1 |
| 42 | 69707 | tr\|A0A6P6JAH3\|A0A6P6JAH3\_CARAU | 21.18 | 3 | 1 | 1 | N | 47039 | G-protein coupled estrogen receptor 1-like isoform X1 OS=Carassius auratus OX=7957 GN=LOC113042077 PE=3 SV=1 |
| 42 | 69708 | tr\|A0A6P6JAN8\|A0A6P6JAN8\_CARAU | 21.18 | 3 | 1 | 1 | N | 47299 | G-protein coupled estrogen receptor 1-like OS=Carassius auratus OX=7957 GN=LOC113042127 PE=3 SV=1 |
| 42 | 69709 | tr\|A0A498LK34\|A0A498LK34\_LABRO | 21.18 | 2 | 1 | 1 | N | 54338 | G-coupled estrogen receptor 1-like protein OS=Labeo rohita OX=84645 GN=ROHU\_006898 PE=3 SV=1 |
| 43 | 69695 | tr\|A0A4W6ESI4\|A0A4W6ESI4\_LATCA | 20.60 | 1 | 1 | 1 | N | 116901 | Collagen, type I, alpha 2 OS=Lates calcarifer OX=8187 PE=4 SV=1 |
| 44 | 69710 | tr\|A0A7J6A7M3\|A0A7J6A7M3\_AMEME | 20.39 | 0 | 1 | 1 | N | 242148 | Separase OS=Ameiurus melas OX=219545 GN=AMELA\_G00185210 PE=4 SV=1 |
| 17 | 69711 | tr\|A0A6G1P920\|A0A6G1P920\_9TELE | 20.06 | 1 | 1 | 1 | N | 161430 | WD repeat and HMG-box DNA-binding protein 1 Acidic nucleoplasmic DNA-binding protein 1 OS=Channa argus OX=215402 GN=EXN66\_Car002427 PE=4 SV=1 |
| total 221 proteins |  |  |  |  |  |  |  |  |  |
| --- | --- | --- | --- | --- | --- | --- | --- | --- | --- |

  

tr|A0A3P9CRE7|A0A3P9CRE7\_9CICH

back to list

  

| Protein Coverage
| Supporting Peptides
|

Protein Coverage:

Supporting Peptides:

| Peptide | Uniq | -10lgP | Mass | ppm | m/z | z | RT | Scan | #Spec | Start | End | PTM |
| --- | --- | --- | --- | --- | --- | --- | --- | --- | --- | --- | --- | --- |
| E.TKTPGLME.N | N | 44.96 | 875.4423 | -1.2 | 438.7279 | 2 | 10.50 | 1395 | 1 | 650 | 657 |  |
| W.LDKNKDPLNDSVVQ.L | N | 37.14 | 1583.8154 | -1.9 | 792.9135 | 2 | 10.88 | 1463 | 1 | 571 | 584 |  |
| E.VADKIAY.L | N | 25.34 | 778.4225 | -1.1 | 390.2181 | 2 | 10.49 | 1394 | 1 | 357 | 363 |  |
| L.SKIEDEQSL.G | N | 24.72 | 1047.5084 | -0.8 | 524.7610 | 2 | 10.24 | 1352 | 1 | 1064 | 1072 |  |
| E.APPHIF.S | N | 22.93 | 680.3646 | -1.5 | 341.1891 | 2 | 11.68 | 1610 | 1 | 144 | 149 |  |
| Q.TEEDKKNLVRL.Q | N | 22.80 | 1343.7408 | -0.9 | 448.9205 | 3 | 10.25 | 1353 | 1 | 1826 | 1836 |  |
| K.RIEAPPHIF.S | Y | 22.23 | 1078.5923 | -1.5 | 540.3026 | 2 | 11.79 | 1629 | 3 | 141 | 149 |  |
| K.QRNFDKVL.A | N | 20.14 | 1018.5560 | -1.1 | 340.5255 | 3 | 10.65 | 1421 | 1 | 1418 | 1425 |  |
| L.KKDIDDLE.L | N | 16.50 | 974.4920 | -0.8 | 488.2529 | 2 | 9.80 | 1275 | 1 | 923 | 930 |  |
| T.LEHEE.A | N | 16.09 | 655.2813 | 3.2 | 328.6490 | 2 | 8.51 | 1054 | 1 | 1523 | 1527 |  |
| T.GWLDKNKDPLNDS.V | N | 15.99 | 1500.7208 | 4.7 | 751.3712 | 2 | 11.80 | 1632 | 1 | 569 | 581 |  |
| total 11 peptides |  |  |  |  |  |  |  |  |  |  |  |  |
| --- | --- | --- | --- | --- | --- | --- | --- | --- | --- | --- | --- | --- |

tr|A0A669BHT6|A0A669BHT6\_ORENI

back to list

  

| Protein Coverage
| Supporting Peptides
|

Protein Coverage:

Supporting Peptides:

| Peptide | Uniq | -10lgP | Mass | ppm | m/z | z | RT | Scan | #Spec | Start | End | PTM |
| --- | --- | --- | --- | --- | --- | --- | --- | --- | --- | --- | --- | --- |
| E.TKTPGLME.N | N | 44.96 | 875.4423 | -1.2 | 438.7279 | 2 | 10.50 | 1395 | 1 | 671 | 678 |  |
| W.LDKNKDPLNDSVVQ.L | N | 37.14 | 1583.8154 | -1.9 | 792.9135 | 2 | 10.88 | 1463 | 1 | 589 | 602 |  |
| E.VADKIAY.L | N | 25.34 | 778.4225 | -1.1 | 390.2181 | 2 | 10.49 | 1394 | 1 | 375 | 381 |  |
| L.SKIEDEQSL.G | N | 24.72 | 1047.5084 | -0.8 | 524.7610 | 2 | 10.24 | 1352 | 1 | 1085 | 1093 |  |
| E.APPHIF.S | N | 22.93 | 680.3646 | -1.5 | 341.1891 | 2 | 11.68 | 1610 | 1 | 144 | 149 |  |
| Q.TEEDKKNLVRL.Q | N | 22.80 | 1343.7408 | -0.9 | 448.9205 | 3 | 10.25 | 1353 | 1 | 1847 | 1857 |  |
| K.RIEAPPHIF.S | Y | 22.23 | 1078.5923 | -1.5 | 540.3026 | 2 | 11.79 | 1629 | 3 | 141 | 149 |  |
| K.QRNFDKVL.A | N | 20.14 | 1018.5560 | -1.1 | 340.5255 | 3 | 10.65 | 1421 | 1 | 1439 | 1446 |  |
| L.KKDIDDLE.L | N | 16.50 | 974.4920 | -0.8 | 488.2529 | 2 | 9.80 | 1275 | 1 | 944 | 951 |  |
| T.LEHEE.A | N | 16.09 | 655.2813 | 3.2 | 328.6490 | 2 | 8.51 | 1054 | 1 | 1544 | 1548 |  |
| S.GWLDKNKDPLNDS.V | N | 15.99 | 1500.7208 | 4.7 | 751.3712 | 2 | 11.80 | 1632 | 1 | 587 | 599 |  |
| total 11 peptides |  |  |  |  |  |  |  |  |  |  |  |  |
| --- | --- | --- | --- | --- | --- | --- | --- | --- | --- | --- | --- | --- |

tr|A0A3P8Q1P8|A0A3P8Q1P8\_ASTCA

back to list

  

| Protein Coverage
| Supporting Peptides
|

Protein Coverage:

Supporting Peptides:

| Peptide | Uniq | -10lgP | Mass | ppm | m/z | z | RT | Scan | #Spec | Start | End | PTM |
| --- | --- | --- | --- | --- | --- | --- | --- | --- | --- | --- | --- | --- |
| E.TKTPGLME.N | N | 44.96 | 875.4423 | -1.2 | 438.7279 | 2 | 10.50 | 1395 | 1 | 679 | 686 |  |
| W.LDKNKDPLNDSVVQ.L | N | 37.14 | 1583.8154 | -1.9 | 792.9135 | 2 | 10.88 | 1463 | 1 | 596 | 609 |  |
| E.VADKIAY.L | N | 25.34 | 778.4225 | -1.1 | 390.2181 | 2 | 10.49 | 1394 | 1 | 382 | 388 |  |
| L.SKIEDEQSL.G | N | 24.72 | 1047.5084 | -0.8 | 524.7610 | 2 | 10.24 | 1352 | 1 | 1093 | 1101 |  |
| E.APPHIF.S | N | 22.93 | 680.3646 | -1.5 | 341.1891 | 2 | 11.68 | 1610 | 1 | 151 | 156 |  |
| Q.TEEDKKNLVRL.Q | N | 22.80 | 1343.7408 | -0.9 | 448.9205 | 3 | 10.25 | 1353 | 1 | 1855 | 1865 |  |
| K.RIEAPPHIF.S | Y | 22.23 | 1078.5923 | -1.5 | 540.3026 | 2 | 11.79 | 1629 | 3 | 148 | 156 |  |
| K.QRNFDKVL.A | N | 20.14 | 1018.5560 | -1.1 | 340.5255 | 3 | 10.65 | 1421 | 1 | 1447 | 1454 |  |
| L.KKDIDDLE.L | N | 16.50 | 974.4920 | -0.8 | 488.2529 | 2 | 9.80 | 1275 | 1 | 952 | 959 |  |
| T.LEHEE.A | N | 16.09 | 655.2813 | 3.2 | 328.6490 | 2 | 8.51 | 1054 | 1 | 1552 | 1556 |  |
| T.GWLDKNKDPLNDS.V | N | 15.99 | 1500.7208 | 4.7 | 751.3712 | 2 | 11.80 | 1632 | 1 | 594 | 606 |  |
| total 11 peptides |  |  |  |  |  |  |  |  |  |  |  |  |
| --- | --- | --- | --- | --- | --- | --- | --- | --- | --- | --- | --- | --- |

tr|A0A3P8QMC2|A0A3P8QMC2\_ASTCA

back to list

  

| Protein Coverage
| Supporting Peptides
|

Protein Coverage:

Supporting Peptides:

| Peptide | Uniq | -10lgP | Mass | ppm | m/z | z | RT | Scan | #Spec | Start | End | PTM |
| --- | --- | --- | --- | --- | --- | --- | --- | --- | --- | --- | --- | --- |
| E.TKTPGLME.N | N | 44.96 | 875.4423 | -1.2 | 438.7279 | 2 | 10.50 | 1395 | 1 | 676 | 683 |  |
| W.LDKNKDPLNDSVVQ.L | N | 37.14 | 1583.8154 | -1.9 | 792.9135 | 2 | 10.88 | 1463 | 1 | 596 | 609 |  |
| E.VADKIAY.L | N | 25.34 | 778.4225 | -1.1 | 390.2181 | 2 | 10.49 | 1394 | 1 | 382 | 388 |  |
| L.SKIEDEQSL.G | N | 24.72 | 1047.5084 | -0.8 | 524.7610 | 2 | 10.24 | 1352 | 1 | 1090 | 1098 |  |
| E.APPHIF.S | N | 22.93 | 680.3646 | -1.5 | 341.1891 | 2 | 11.68 | 1610 | 1 | 151 | 156 |  |
| Q.TEEDKKNLVRL.Q | N | 22.80 | 1343.7408 | -0.9 | 448.9205 | 3 | 10.25 | 1353 | 1 | 1852 | 1862 |  |
| K.RIEAPPHIF.S | Y | 22.23 | 1078.5923 | -1.5 | 540.3026 | 2 | 11.79 | 1629 | 3 | 148 | 156 |  |
| K.QRNFDKVL.A | N | 20.14 | 1018.5560 | -1.1 | 340.5255 | 3 | 10.65 | 1421 | 1 | 1444 | 1451 |  |
| L.KKDIDDLE.L | N | 16.50 | 974.4920 | -0.8 | 488.2529 | 2 | 9.80 | 1275 | 1 | 949 | 956 |  |
| T.LEHEE.A | N | 16.09 | 655.2813 | 3.2 | 328.6490 | 2 | 8.51 | 1054 | 1 | 1549 | 1553 |  |
| T.GWLDKNKDPLNDS.V | N | 15.99 | 1500.7208 | 4.7 | 751.3712 | 2 | 11.80 | 1632 | 1 | 594 | 606 |  |
| total 11 peptides |  |  |  |  |  |  |  |  |  |  |  |  |
| --- | --- | --- | --- | --- | --- | --- | --- | --- | --- | --- | --- | --- |

tr|A0A3P8Q1G5|A0A3P8Q1G5\_ASTCA

back to list

  

| Protein Coverage
| Supporting Peptides
|

Protein Coverage:

Supporting Peptides:

| Peptide | Uniq | -10lgP | Mass | ppm | m/z | z | RT | Scan | #Spec | Start | End | PTM |
| --- | --- | --- | --- | --- | --- | --- | --- | --- | --- | --- | --- | --- |
| E.TKTPGLME.N | N | 44.96 | 875.4423 | -1.2 | 438.7279 | 2 | 10.50 | 1395 | 1 | 677 | 684 |  |
| W.LDKNKDPLNDSVVQ.L | N | 37.14 | 1583.8154 | -1.9 | 792.9135 | 2 | 10.88 | 1463 | 1 | 596 | 609 |  |
| E.VADKIAY.L | N | 25.34 | 778.4225 | -1.1 | 390.2181 | 2 | 10.49 | 1394 | 1 | 382 | 388 |  |
| L.SKIEDEQSL.G | N | 24.72 | 1047.5084 | -0.8 | 524.7610 | 2 | 10.24 | 1352 | 1 | 1091 | 1099 |  |
| E.APPHIF.S | N | 22.93 | 680.3646 | -1.5 | 341.1891 | 2 | 11.68 | 1610 | 1 | 151 | 156 |  |
| Q.TEEDKKNLVRL.Q | N | 22.80 | 1343.7408 | -0.9 | 448.9205 | 3 | 10.25 | 1353 | 1 | 1853 | 1863 |  |
| K.RIEAPPHIF.S | Y | 22.23 | 1078.5923 | -1.5 | 540.3026 | 2 | 11.79 | 1629 | 3 | 148 | 156 |  |
| K.QRNFDKVL.A | N | 20.14 | 1018.5560 | -1.1 | 340.5255 | 3 | 10.65 | 1421 | 1 | 1445 | 1452 |  |
| L.KKDIDDLE.L | N | 16.50 | 974.4920 | -0.8 | 488.2529 | 2 | 9.80 | 1275 | 1 | 950 | 957 |  |
| T.LEHEE.A | N | 16.09 | 655.2813 | 3.2 | 328.6490 | 2 | 8.51 | 1054 | 1 | 1550 | 1554 |  |
| T.GWLDKNKDPLNDS.V | N | 15.99 | 1500.7208 | 4.7 | 751.3712 | 2 | 11.80 | 1632 | 1 | 594 | 606 |  |
| total 11 peptides |  |  |  |  |  |  |  |  |  |  |  |  |
| --- | --- | --- | --- | --- | --- | --- | --- | --- | --- | --- | --- | --- |

tr|I3IZY8|I3IZY8\_ORENI

back to list

  

| Protein Coverage
| Supporting Peptides
|

Protein Coverage:

Supporting Peptides:

| Peptide | Uniq | -10lgP | Mass | ppm | m/z | z | RT | Scan | #Spec | Start | End | PTM |
| --- | --- | --- | --- | --- | --- | --- | --- | --- | --- | --- | --- | --- |
| E.TKTPGLME.N | N | 44.96 | 875.4423 | -1.2 | 438.7279 | 2 | 10.50 | 1395 | 1 | 680 | 687 |  |
| W.LDKNKDPLNDSVVQ.L | N | 37.14 | 1583.8154 | -1.9 | 792.9135 | 2 | 10.88 | 1463 | 1 | 596 | 609 |  |
| E.VADKIAY.L | N | 25.34 | 778.4225 | -1.1 | 390.2181 | 2 | 10.49 | 1394 | 1 | 382 | 388 |  |
| E.APPHIF.S | N | 22.93 | 680.3646 | -1.5 | 341.1891 | 2 | 11.68 | 1610 | 1 | 151 | 156 |  |
| Q.TEEDKKNLVRL.Q | N | 22.80 | 1343.7408 | -0.9 | 448.9205 | 3 | 10.25 | 1353 | 1 | 1831 | 1841 |  |
| K.QRNFDKVL.A | N | 20.14 | 1018.5560 | -1.1 | 340.5255 | 3 | 10.65 | 1421 | 1 | 1423 | 1430 |  |
| K.RVEAPPHIF.S | N | 19.35 | 1064.5767 | -1.6 | 533.2947 | 2 | 11.56 | 1588 | 1 | 148 | 156 |  |
| L.KKDIDDLE.L | N | 16.50 | 974.4920 | -0.8 | 488.2529 | 2 | 9.80 | 1275 | 1 | 953 | 960 |  |
| T.LEHEE.A | N | 16.09 | 655.2813 | 3.2 | 328.6490 | 2 | 8.51 | 1054 | 1 | 1528 | 1532 |  |
| T.GWLDKNKDPLNDS.V | N | 15.99 | 1500.7208 | 4.7 | 751.3712 | 2 | 11.80 | 1632 | 1 | 594 | 606 |  |
| total 10 peptides |  |  |  |  |  |  |  |  |  |  |  |  |
| --- | --- | --- | --- | --- | --- | --- | --- | --- | --- | --- | --- | --- |

tr|I3IZU1|I3IZU1\_ORENI

back to list

  

| Protein Coverage
| Supporting Peptides
|

Protein Coverage:

Supporting Peptides:

| Peptide | Uniq | -10lgP | Mass | ppm | m/z | z | RT | Scan | #Spec | Start | End | PTM |
| --- | --- | --- | --- | --- | --- | --- | --- | --- | --- | --- | --- | --- |
| E.TKTPGLME.N | N | 44.96 | 875.4423 | -1.2 | 438.7279 | 2 | 10.50 | 1395 | 1 | 678 | 685 |  |
| W.LDKNKDPLNDSVVQ.L | N | 37.14 | 1583.8154 | -1.9 | 792.9135 | 2 | 10.88 | 1463 | 1 | 596 | 609 |  |
| E.VADKIAY.L | N | 25.34 | 778.4225 | -1.1 | 390.2181 | 2 | 10.49 | 1394 | 1 | 382 | 388 |  |
| E.APPHIF.S | N | 22.93 | 680.3646 | -1.5 | 341.1891 | 2 | 11.68 | 1610 | 1 | 151 | 156 |  |
| Q.TEEDKKNLVRL.Q | N | 22.80 | 1343.7408 | -0.9 | 448.9205 | 3 | 10.25 | 1353 | 1 | 1829 | 1839 |  |
| K.QRNFDKVL.A | N | 20.14 | 1018.5560 | -1.1 | 340.5255 | 3 | 10.65 | 1421 | 1 | 1421 | 1428 |  |
| K.RVEAPPHIF.S | N | 19.35 | 1064.5767 | -1.6 | 533.2947 | 2 | 11.56 | 1588 | 1 | 148 | 156 |  |
| L.KKDIDDLE.L | N | 16.50 | 974.4920 | -0.8 | 488.2529 | 2 | 9.80 | 1275 | 1 | 951 | 958 |  |
| T.LEHEE.A | N | 16.09 | 655.2813 | 3.2 | 328.6490 | 2 | 8.51 | 1054 | 1 | 1526 | 1530 |  |
| T.GWLDKNKDPLNDS.V | N | 15.99 | 1500.7208 | 4.7 | 751.3712 | 2 | 11.80 | 1632 | 1 | 594 | 606 |  |
| total 10 peptides |  |  |  |  |  |  |  |  |  |  |  |  |
| --- | --- | --- | --- | --- | --- | --- | --- | --- | --- | --- | --- | --- |

tr|A0A3S2MUF9|A0A3S2MUF9\_ORYJA

back to list

  

| Protein Coverage
| Supporting Peptides
|

Protein Coverage:

Supporting Peptides:

| Peptide | Uniq | -10lgP | Mass | ppm | m/z | z | RT | Scan | #Spec | Start | End | PTM |
| --- | --- | --- | --- | --- | --- | --- | --- | --- | --- | --- | --- | --- |
| A.GFAGDDAPRA.V | Y | 37.63 | 975.4410 | -1.4 | 488.7271 | 2 | 10.18 | 1340 | 1 | 22 | 31 |  |
| T.MYPGIADRM.Q | Y | 36.60 | 1052.4783 | -1.8 | 527.2455 | 2 | 11.84 | 1640 | 1 | 307 | 315 |  |
| G.FAGDDAPRA.V | Y | 28.57 | 918.4195 | -1.0 | 460.2166 | 2 | 9.82 | 1278 | 1 | 23 | 31 |  |
| L.DAGDGVTH.N | Y | 22.86 | 770.3195 | 0.3 | 386.1671 | 2 | 8.70 | 1091 | 2 | 156 | 163 |  |
| K.IIAPPERKY.S | Y | 22.73 | 1085.6233 | -2.0 | 543.8178 | 2 | 10.01 | 1311 | 2 | 331 | 339 |  |
| K.IIAPPERK.Y | Y | 18.76 | 922.5599 | -1.4 | 308.5268 | 3 | 9.49 | 1222 | 1 | 331 | 338 |  |
| Y.ELPDGQVI.T | Y | 18.22 | 869.4494 | -1.6 | 435.7313 | 2 | 12.46 | 1756 | 1 | 243 | 250 |  |
| M.KIKIIAPPERKY.S | Y | 18.16 | 1454.8972 | -1.2 | 485.9724 | 3 | 9.98 | 1305 | 1 | 328 | 339 |  |
| E.KSYELPDGQVITI.G | Y | 15.76 | 1461.7715 | -0.4 | 731.8927 | 2 | 13.67 | 1973 | 1 | 240 | 252 |  |
| E.EHPTLL.T | Y | 15.28 | 708.3806 | -1.3 | 355.1971 | 2 | 11.27 | 1532 | 1 | 102 | 107 |  |
| total 10 peptides |  |  |  |  |  |  |  |  |  |  |  |  |
| --- | --- | --- | --- | --- | --- | --- | --- | --- | --- | --- | --- | --- |

tr|A0A7J6C441|A0A7J6C441\_9TELE

back to list

  

| Protein Coverage
| Supporting Peptides
|

Protein Coverage:

Supporting Peptides:

| Peptide | Uniq | -10lgP | Mass | ppm | m/z | z | RT | Scan | #Spec | Start | End | PTM |
| --- | --- | --- | --- | --- | --- | --- | --- | --- | --- | --- | --- | --- |
| A.GFAGDDAPRA.V | Y | 37.63 | 975.4410 | -1.4 | 488.7271 | 2 | 10.18 | 1340 | 1 | 22 | 31 |  |
| T.MYPGIADRM.Q | Y | 36.60 | 1052.4783 | -1.8 | 527.2455 | 2 | 11.84 | 1640 | 1 | 307 | 315 |  |
| G.FAGDDAPRA.V | Y | 28.57 | 918.4195 | -1.0 | 460.2166 | 2 | 9.82 | 1278 | 1 | 23 | 31 |  |
| L.DAGDGVTH.N | Y | 22.86 | 770.3195 | 0.3 | 386.1671 | 2 | 8.70 | 1091 | 2 | 156 | 163 |  |
| K.IIAPPERKY.S | Y | 22.73 | 1085.6233 | -2.0 | 543.8178 | 2 | 10.01 | 1311 | 2 | 331 | 339 |  |
| K.IIAPPERK.Y | Y | 18.76 | 922.5599 | -1.4 | 308.5268 | 3 | 9.49 | 1222 | 1 | 331 | 338 |  |
| Y.ELPDGQVI.T | Y | 18.22 | 869.4494 | -1.6 | 435.7313 | 2 | 12.46 | 1756 | 1 | 243 | 250 |  |
| M.KIKIIAPPERKY.S | Y | 18.16 | 1454.8972 | -1.2 | 485.9724 | 3 | 9.98 | 1305 | 1 | 328 | 339 |  |
| E.KSYELPDGQVITI.G | Y | 15.76 | 1461.7715 | -0.4 | 731.8927 | 2 | 13.67 | 1973 | 1 | 240 | 252 |  |
| E.EHPTLL.T | Y | 15.28 | 708.3806 | -1.3 | 355.1971 | 2 | 11.27 | 1532 | 1 | 102 | 107 |  |
| total 10 peptides |  |  |  |  |  |  |  |  |  |  |  |  |
| --- | --- | --- | --- | --- | --- | --- | --- | --- | --- | --- | --- | --- |

tr|A0A3B4YLK9|A0A3B4YLK9\_SERLL

back to list

  

| Protein Coverage
| Supporting Peptides
|

Protein Coverage:

Supporting Peptides:

| Peptide | Uniq | -10lgP | Mass | ppm | m/z | z | RT | Scan | #Spec | Start | End | PTM |
| --- | --- | --- | --- | --- | --- | --- | --- | --- | --- | --- | --- | --- |
| W.LDKNKDPLNDSVVQ.L | N | 37.14 | 1583.8154 | -1.9 | 792.9135 | 2 | 10.88 | 1463 | 1 | 595 | 608 |  |
| E.VADKIAY.L | N | 25.34 | 778.4225 | -1.1 | 390.2181 | 2 | 10.49 | 1394 | 1 | 381 | 387 |  |
| L.SKIEDEQSL.S | N | 24.72 | 1047.5084 | -0.8 | 524.7610 | 2 | 10.24 | 1352 | 1 | 1093 | 1101 |  |
| E.APPHIF.S | N | 22.93 | 680.3646 | -1.5 | 341.1891 | 2 | 11.68 | 1610 | 1 | 150 | 155 |  |
| K.QRNFDKVL.A | N | 20.14 | 1018.5560 | -1.1 | 340.5255 | 3 | 10.65 | 1421 | 1 | 1447 | 1454 |  |
| K.RVEAPPHIF.S | N | 19.35 | 1064.5767 | -1.6 | 533.2947 | 2 | 11.56 | 1588 | 1 | 147 | 155 |  |
| L.KKDIDDLE.L | N | 16.50 | 974.4920 | -0.8 | 488.2529 | 2 | 9.80 | 1275 | 1 | 952 | 959 |  |
| T.LEHEE.S | N | 16.09 | 655.2813 | 3.2 | 328.6490 | 2 | 8.51 | 1054 | 1 | 1552 | 1556 |  |
| L.GWLDKNKDPLNDS.V | N | 15.99 | 1500.7208 | 4.7 | 751.3712 | 2 | 11.80 | 1632 | 1 | 593 | 605 |  |
| total 9 peptides |  |  |  |  |  |  |  |  |  |  |  |  |
| --- | --- | --- | --- | --- | --- | --- | --- | --- | --- | --- | --- | --- |

tr|A0A671XZM8|A0A671XZM8\_SPAAU

back to list

  

| Protein Coverage
| Supporting Peptides
|

Protein Coverage:

Supporting Peptides:

| Peptide | Uniq | -10lgP | Mass | ppm | m/z | z | RT | Scan | #Spec | Start | End | PTM |
| --- | --- | --- | --- | --- | --- | --- | --- | --- | --- | --- | --- | --- |
| V.WIDPDDFPLLLPH.W | Y | 40.97 | 1576.7925 | -1.3 | 789.4025 | 2 | 15.34 | 2265 | 1 | 309 | 321 |  |
| F.MDDPVVIPGKPY.T | Y | 30.08 | 1329.6638 | -1.8 | 665.8380 | 2 | 12.26 | 1718 | 1 | 225 | 236 |  |
| E.LEDWIEDVLS.V | Y | 26.20 | 1217.5815 | -0.3 | 609.7979 | 2 | 15.35 | 2267 | 1 | 362 | 371 |  |
| total 3 peptides |  |  |  |  |  |  |  |  |  |  |  |  |
| --- | --- | --- | --- | --- | --- | --- | --- | --- | --- | --- | --- | --- |

tr|A0A671Y6A5|A0A671Y6A5\_SPAAU

back to list

  

| Protein Coverage
| Supporting Peptides
|

Protein Coverage:

Supporting Peptides:

| Peptide | Uniq | -10lgP | Mass | ppm | m/z | z | RT | Scan | #Spec | Start | End | PTM |
| --- | --- | --- | --- | --- | --- | --- | --- | --- | --- | --- | --- | --- |
| V.WIDPDDFPLLLPH.W | Y | 40.97 | 1576.7925 | -1.3 | 789.4025 | 2 | 15.34 | 2265 | 1 | 286 | 298 |  |
| F.MDDPVVIPGKPY.T | Y | 30.08 | 1329.6638 | -1.8 | 665.8380 | 2 | 12.26 | 1718 | 1 | 211 | 222 |  |
| E.LEDWIEDVLS.G | Y | 26.20 | 1217.5815 | -0.3 | 609.7979 | 2 | 15.35 | 2267 | 1 | 339 | 348 |  |
| total 3 peptides |  |  |  |  |  |  |  |  |  |  |  |  |
| --- | --- | --- | --- | --- | --- | --- | --- | --- | --- | --- | --- | --- |

tr|A0A671XZM4|A0A671XZM4\_SPAAU

back to list

  

| Protein Coverage
| Supporting Peptides
|

Protein Coverage:

Supporting Peptides:

| Peptide | Uniq | -10lgP | Mass | ppm | m/z | z | RT | Scan | #Spec | Start | End | PTM |
| --- | --- | --- | --- | --- | --- | --- | --- | --- | --- | --- | --- | --- |
| V.WIDPDDFPLLLPH.W | Y | 40.97 | 1576.7925 | -1.3 | 789.4025 | 2 | 15.34 | 2265 | 1 | 299 | 311 |  |
| F.MDDPVVIPGKPY.T | Y | 30.08 | 1329.6638 | -1.8 | 665.8380 | 2 | 12.26 | 1718 | 1 | 224 | 235 |  |
| E.LEDWIEDVLS.G | Y | 26.20 | 1217.5815 | -0.3 | 609.7979 | 2 | 15.35 | 2267 | 1 | 352 | 361 |  |
| total 3 peptides |  |  |  |  |  |  |  |  |  |  |  |  |
| --- | --- | --- | --- | --- | --- | --- | --- | --- | --- | --- | --- | --- |

tr|A0A671XZK3|A0A671XZK3\_SPAAU

back to list

  

| Protein Coverage
| Supporting Peptides
|

Protein Coverage:

Supporting Peptides:

| Peptide | Uniq | -10lgP | Mass | ppm | m/z | z | RT | Scan | #Spec | Start | End | PTM |
| --- | --- | --- | --- | --- | --- | --- | --- | --- | --- | --- | --- | --- |
| V.WIDPDDFPLLLPH.W | Y | 40.97 | 1576.7925 | -1.3 | 789.4025 | 2 | 15.34 | 2265 | 1 | 309 | 321 |  |
| F.MDDPVVIPGKPY.T | Y | 30.08 | 1329.6638 | -1.8 | 665.8380 | 2 | 12.26 | 1718 | 1 | 225 | 236 |  |
| E.LEDWIEDVLS.G | Y | 26.20 | 1217.5815 | -0.3 | 609.7979 | 2 | 15.35 | 2267 | 1 | 362 | 371 |  |
| total 3 peptides |  |  |  |  |  |  |  |  |  |  |  |  |
| --- | --- | --- | --- | --- | --- | --- | --- | --- | --- | --- | --- | --- |

tr|A0A671XZP8|A0A671XZP8\_SPAAU

back to list

  

| Protein Coverage
| Supporting Peptides
|

Protein Coverage:

Supporting Peptides:

| Peptide | Uniq | -10lgP | Mass | ppm | m/z | z | RT | Scan | #Spec | Start | End | PTM |
| --- | --- | --- | --- | --- | --- | --- | --- | --- | --- | --- | --- | --- |
| V.WIDPDDFPLLLPH.W | Y | 40.97 | 1576.7925 | -1.3 | 789.4025 | 2 | 15.34 | 2265 | 1 | 328 | 340 |  |
| F.MDDPVVIPGKPY.T | Y | 30.08 | 1329.6638 | -1.8 | 665.8380 | 2 | 12.26 | 1718 | 1 | 253 | 264 |  |
| E.LEDWIEDVLS.G | Y | 26.20 | 1217.5815 | -0.3 | 609.7979 | 2 | 15.35 | 2267 | 1 | 381 | 390 |  |
| total 3 peptides |  |  |  |  |  |  |  |  |  |  |  |  |
| --- | --- | --- | --- | --- | --- | --- | --- | --- | --- | --- | --- | --- |

tr|A0A834CTG4|A0A834CTG4\_ORYME

back to list

  

| Protein Coverage
| Supporting Peptides
|

Protein Coverage:

Supporting Peptides:

| Peptide | Uniq | -10lgP | Mass | ppm | m/z | z | RT | Scan | #Spec | Start | End | PTM |
| --- | --- | --- | --- | --- | --- | --- | --- | --- | --- | --- | --- | --- |
| A.MNPLFPPGPPKDL.H | Y | 36.23 | 1421.7377 | -3.1 | 711.8739 | 2 | 13.54 | 1950 | 2 | 1811 | 1823 |  |
| E.VGAGDPSPPSKPVFA.K | Y | 35.36 | 1424.7299 | -2.2 | 713.3707 | 2 | 11.38 | 1554 | 1 | 1499 | 1513 |  |
| L.GPPGPPVGPIKF.T | Y | 28.36 | 1161.6545 | -1.2 | 581.8339 | 2 | 12.78 | 1814 | 1 | 595 | 606 |  |
| total 3 peptides |  |  |  |  |  |  |  |  |  |  |  |  |
| --- | --- | --- | --- | --- | --- | --- | --- | --- | --- | --- | --- | --- |

tr|A0A315VR11|A0A315VR11\_GAMAF

back to list

  

| Protein Coverage
| Supporting Peptides
|

Protein Coverage:

Supporting Peptides:

| Peptide | Uniq | -10lgP | Mass | ppm | m/z | z | RT | Scan | #Spec | Start | End | PTM |
| --- | --- | --- | --- | --- | --- | --- | --- | --- | --- | --- | --- | --- |
| L.FDKPVSPL.L | Y | 29.51 | 901.4908 | -1.7 | 451.7520 | 2 | 11.53 | 1581 | 2 | 628 | 635 |  |
| R.HGGYKPT.D | Y | 24.75 | 758.3711 | -0.1 | 380.1928 | 2 | 8.43 | 1038 | 1 | 531 | 537 |  |
| L.VVDGVKLM.V | Y | 21.72 | 859.4837 | -1.9 | 430.7483 | 2 | 11.82 | 1636 | 1 | 787 | 794 |  |
| L.VVDGVKL.M | Y | 21.20 | 728.4432 | -1.5 | 365.2283 | 2 | 11.24 | 1526 | 1 | 787 | 793 |  |
| T.GVDNPGHPF.I | Y | 20.20 | 938.4246 | -0.2 | 470.2195 | 2 | 16.17 | 2408 | 3 | 494 | 502 |  |
| total 5 peptides |  |  |  |  |  |  |  |  |  |  |  |  |
| --- | --- | --- | --- | --- | --- | --- | --- | --- | --- | --- | --- | --- |

tr|A0A6G0IP12|A0A6G0IP12\_LARCR

back to list

  

| Protein Coverage
| Supporting Peptides
|

Protein Coverage:

Supporting Peptides:

| Peptide | Uniq | -10lgP | Mass | ppm | m/z | z | RT | Scan | #Spec | Start | End | PTM |
| --- | --- | --- | --- | --- | --- | --- | --- | --- | --- | --- | --- | --- |
| N.SDGMLDFDEFLKM.M | Y | 35.71 | 1546.6683 | -0.5 | 774.3411 | 2 | 15.25 | 2249 | 1 | 143 | 155 |  |
| N.SDGMLDFDEFLK.M | Y | 33.64 | 1415.6278 | -0.2 | 708.8210 | 2 | 14.54 | 2125 | 1 | 143 | 154 |  |
| L.MKDGDKNSDGMLDFDEFLK.M | Y | 15.63 | 2203.9766 | -2.0 | 735.6647 | 3 | 13.43 | 1932 | 1 | 136 | 154 |  |
| total 3 peptides |  |  |  |  |  |  |  |  |  |  |  |  |
| --- | --- | --- | --- | --- | --- | --- | --- | --- | --- | --- | --- | --- |

tr|A0A1A8D9W7|A0A1A8D9W7\_9TELE

back to list

  

| Protein Coverage
| Supporting Peptides
|

Protein Coverage:

Supporting Peptides:

| Peptide | Uniq | -10lgP | Mass | ppm | m/z | z | RT | Scan | #Spec | Start | End | PTM |
| --- | --- | --- | --- | --- | --- | --- | --- | --- | --- | --- | --- | --- |
| N.SDGMLDFDEFLKM.M | Y | 35.71 | 1546.6683 | -0.5 | 774.3411 | 2 | 15.25 | 2249 | 1 | 118 | 130 |  |
| N.SDGMLDFDEFLK.M | Y | 33.64 | 1415.6278 | -0.2 | 708.8210 | 2 | 14.54 | 2125 | 1 | 118 | 129 |  |
| L.MKDGDKNSDGMLDFDEFLK.M | Y | 15.63 | 2203.9766 | -2.0 | 735.6647 | 3 | 13.43 | 1932 | 1 | 111 | 129 |  |
| total 3 peptides |  |  |  |  |  |  |  |  |  |  |  |  |
| --- | --- | --- | --- | --- | --- | --- | --- | --- | --- | --- | --- | --- |

tr|A0A1A8CTF6|A0A1A8CTF6\_9TELE

back to list

  

| Protein Coverage
| Supporting Peptides
|

Protein Coverage:

Supporting Peptides:

| Peptide | Uniq | -10lgP | Mass | ppm | m/z | z | RT | Scan | #Spec | Start | End | PTM |
| --- | --- | --- | --- | --- | --- | --- | --- | --- | --- | --- | --- | --- |
| N.SDGMLDFDEFLKM.M | Y | 35.71 | 1546.6683 | -0.5 | 774.3411 | 2 | 15.25 | 2249 | 1 | 143 | 155 |  |
| N.SDGMLDFDEFLK.M | Y | 33.64 | 1415.6278 | -0.2 | 708.8210 | 2 | 14.54 | 2125 | 1 | 143 | 154 |  |
| L.MKDGDKNSDGMLDFDEFLK.M | Y | 15.63 | 2203.9766 | -2.0 | 735.6647 | 3 | 13.43 | 1932 | 1 | 136 | 154 |  |
| total 3 peptides |  |  |  |  |  |  |  |  |  |  |  |  |
| --- | --- | --- | --- | --- | --- | --- | --- | --- | --- | --- | --- | --- |

tr|A0A1A8QW41|A0A1A8QW41\_9TELE

back to list

  

| Protein Coverage
| Supporting Peptides
|

Protein Coverage:

Supporting Peptides:

| Peptide | Uniq | -10lgP | Mass | ppm | m/z | z | RT | Scan | #Spec | Start | End | PTM |
| --- | --- | --- | --- | --- | --- | --- | --- | --- | --- | --- | --- | --- |
| N.SDGMLDFDEFLKM.M | Y | 35.71 | 1546.6683 | -0.5 | 774.3411 | 2 | 15.25 | 2249 | 1 | 143 | 155 |  |
| N.SDGMLDFDEFLK.M | Y | 33.64 | 1415.6278 | -0.2 | 708.8210 | 2 | 14.54 | 2125 | 1 | 143 | 154 |  |
| L.MKDGDKNSDGMLDFDEFLK.M | Y | 15.63 | 2203.9766 | -2.0 | 735.6647 | 3 | 13.43 | 1932 | 1 | 136 | 154 |  |
| total 3 peptides |  |  |  |  |  |  |  |  |  |  |  |  |
| --- | --- | --- | --- | --- | --- | --- | --- | --- | --- | --- | --- | --- |

tr|A0A1A8B5H7|A0A1A8B5H7\_NOTFU

back to list

  

| Protein Coverage
| Supporting Peptides
|

Protein Coverage:

Supporting Peptides:

| Peptide | Uniq | -10lgP | Mass | ppm | m/z | z | RT | Scan | #Spec | Start | End | PTM |
| --- | --- | --- | --- | --- | --- | --- | --- | --- | --- | --- | --- | --- |
| N.SDGMLDFDEFLKM.M | Y | 35.71 | 1546.6683 | -0.5 | 774.3411 | 2 | 15.25 | 2249 | 1 | 143 | 155 |  |
| N.SDGMLDFDEFLK.M | Y | 33.64 | 1415.6278 | -0.2 | 708.8210 | 2 | 14.54 | 2125 | 1 | 143 | 154 |  |
| L.MKDGDKNSDGMLDFDEFLK.M | Y | 15.63 | 2203.9766 | -2.0 | 735.6647 | 3 | 13.43 | 1932 | 1 | 136 | 154 |  |
| total 3 peptides |  |  |  |  |  |  |  |  |  |  |  |  |
| --- | --- | --- | --- | --- | --- | --- | --- | --- | --- | --- | --- | --- |

tr|A0A1A8IG78|A0A1A8IG78\_NOTKU

back to list

  

| Protein Coverage
| Supporting Peptides
|

Protein Coverage:

Supporting Peptides:

| Peptide | Uniq | -10lgP | Mass | ppm | m/z | z | RT | Scan | #Spec | Start | End | PTM |
| --- | --- | --- | --- | --- | --- | --- | --- | --- | --- | --- | --- | --- |
| N.SDGMLDFDEFLKM.M | Y | 35.71 | 1546.6683 | -0.5 | 774.3411 | 2 | 15.25 | 2249 | 1 | 73 | 85 |  |
| N.SDGMLDFDEFLK.M | Y | 33.64 | 1415.6278 | -0.2 | 708.8210 | 2 | 14.54 | 2125 | 1 | 73 | 84 |  |
| L.MKDGDKNSDGMLDFDEFLK.M | Y | 15.63 | 2203.9766 | -2.0 | 735.6647 | 3 | 13.43 | 1932 | 1 | 66 | 84 |  |
| total 3 peptides |  |  |  |  |  |  |  |  |  |  |  |  |
| --- | --- | --- | --- | --- | --- | --- | --- | --- | --- | --- | --- | --- |

tr|A0A1A8ML32|A0A1A8ML32\_9TELE

back to list

  

| Protein Coverage
| Supporting Peptides
|

Protein Coverage:

Supporting Peptides:

| Peptide | Uniq | -10lgP | Mass | ppm | m/z | z | RT | Scan | #Spec | Start | End | PTM |
| --- | --- | --- | --- | --- | --- | --- | --- | --- | --- | --- | --- | --- |
| N.SDGMLDFDEFLKM.M | Y | 35.71 | 1546.6683 | -0.5 | 774.3411 | 2 | 15.25 | 2249 | 1 | 73 | 85 |  |
| N.SDGMLDFDEFLK.M | Y | 33.64 | 1415.6278 | -0.2 | 708.8210 | 2 | 14.54 | 2125 | 1 | 73 | 84 |  |
| L.MKDGDKNSDGMLDFDEFLK.M | Y | 15.63 | 2203.9766 | -2.0 | 735.6647 | 3 | 13.43 | 1932 | 1 | 66 | 84 |  |
| total 3 peptides |  |  |  |  |  |  |  |  |  |  |  |  |
| --- | --- | --- | --- | --- | --- | --- | --- | --- | --- | --- | --- | --- |

tr|A0A1A8SNN3|A0A1A8SNN3\_9TELE

back to list

  

| Protein Coverage
| Supporting Peptides
|

Protein Coverage:

Supporting Peptides:

| Peptide | Uniq | -10lgP | Mass | ppm | m/z | z | RT | Scan | #Spec | Start | End | PTM |
| --- | --- | --- | --- | --- | --- | --- | --- | --- | --- | --- | --- | --- |
| N.SDGMLDFDEFLKM.M | Y | 35.71 | 1546.6683 | -0.5 | 774.3411 | 2 | 15.25 | 2249 | 1 | 79 | 91 |  |
| N.SDGMLDFDEFLK.M | Y | 33.64 | 1415.6278 | -0.2 | 708.8210 | 2 | 14.54 | 2125 | 1 | 79 | 90 |  |
| L.MKDGDKNSDGMLDFDEFLK.M | Y | 15.63 | 2203.9766 | -2.0 | 735.6647 | 3 | 13.43 | 1932 | 1 | 72 | 90 |  |
| total 3 peptides |  |  |  |  |  |  |  |  |  |  |  |  |
| --- | --- | --- | --- | --- | --- | --- | --- | --- | --- | --- | --- | --- |

tr|A0A1A8Q0J1|A0A1A8Q0J1\_9TELE

back to list

  

| Protein Coverage
| Supporting Peptides
|

Protein Coverage:

Supporting Peptides:

| Peptide | Uniq | -10lgP | Mass | ppm | m/z | z | RT | Scan | #Spec | Start | End | PTM |
| --- | --- | --- | --- | --- | --- | --- | --- | --- | --- | --- | --- | --- |
| N.SDGMLDFDEFLKM.M | Y | 35.71 | 1546.6683 | -0.5 | 774.3411 | 2 | 15.25 | 2249 | 1 | 144 | 156 |  |
| N.SDGMLDFDEFLK.M | Y | 33.64 | 1415.6278 | -0.2 | 708.8210 | 2 | 14.54 | 2125 | 1 | 144 | 155 |  |
| L.MKDGDKNSDGMLDFDEFLK.M | Y | 15.63 | 2203.9766 | -2.0 | 735.6647 | 3 | 13.43 | 1932 | 1 | 137 | 155 |  |
| total 3 peptides |  |  |  |  |  |  |  |  |  |  |  |  |
| --- | --- | --- | --- | --- | --- | --- | --- | --- | --- | --- | --- | --- |

tr|A0A6J2PW36|A0A6J2PW36\_COTGO

back to list

  

| Protein Coverage
| Supporting Peptides
|

Protein Coverage:

Supporting Peptides:

| Peptide | Uniq | -10lgP | Mass | ppm | m/z | z | RT | Scan | #Spec | Start | End | PTM |
| --- | --- | --- | --- | --- | --- | --- | --- | --- | --- | --- | --- | --- |
| L.SKIEDEQSL.G | N | 24.72 | 1047.5084 | -0.8 | 524.7610 | 2 | 10.24 | 1352 | 1 | 1088 | 1096 |  |
| E.APPHIF.S | N | 22.93 | 680.3646 | -1.5 | 341.1891 | 2 | 11.68 | 1610 | 1 | 152 | 157 |  |
| N.MENVAK.A | Y | 21.24 | 690.3370 | -0.7 | 346.1756 | 2 | 9.03 | 1148 | 1 | 1231 | 1236 |  |
| K.QRNFDKVL.A | N | 20.14 | 1018.5560 | -1.1 | 340.5255 | 3 | 10.65 | 1421 | 1 | 1442 | 1449 |  |
| L.KKDIDDLE.L | N | 16.50 | 974.4920 | -0.8 | 488.2529 | 2 | 9.80 | 1275 | 1 | 947 | 954 |  |
| T.LEHEE.S | N | 16.09 | 655.2813 | 3.2 | 328.6490 | 2 | 8.51 | 1054 | 1 | 1547 | 1551 |  |
| N.GWLDKNKDPLNDS.V | N | 15.99 | 1500.7208 | 4.7 | 751.3712 | 2 | 11.80 | 1632 | 1 | 590 | 602 |  |
| total 7 peptides |  |  |  |  |  |  |  |  |  |  |  |  |
| --- | --- | --- | --- | --- | --- | --- | --- | --- | --- | --- | --- | --- |

tr|A0A6A5EZU1|A0A6A5EZU1\_PERFL

back to list

  

| Protein Coverage
| Supporting Peptides
|

Protein Coverage:

Supporting Peptides:

| Peptide | Uniq | -10lgP | Mass | ppm | m/z | z | RT | Scan | #Spec | Start | End | PTM |
| --- | --- | --- | --- | --- | --- | --- | --- | --- | --- | --- | --- | --- |
| M.GPRGPPGSPGSSGPQ.G | Y | 50.73 | 1333.6375 | -1.1 | 667.8253 | 2 | 9.41 | 1210 | 1 | 169 | 183 |  |
| total 1 peptides |  |  |  |  |  |  |  |  |  |  |  |  |
| --- | --- | --- | --- | --- | --- | --- | --- | --- | --- | --- | --- | --- |

tr|A0A4Z2EZH8|A0A4Z2EZH8\_9TELE

back to list

  

| Protein Coverage
| Supporting Peptides
|

Protein Coverage:

Supporting Peptides:

| Peptide | Uniq | -10lgP | Mass | ppm | m/z | z | RT | Scan | #Spec | Start | End | PTM |
| --- | --- | --- | --- | --- | --- | --- | --- | --- | --- | --- | --- | --- |
| M.GPRGPPGSPGSSGPQ.G | Y | 50.73 | 1333.6375 | -1.1 | 667.8253 | 2 | 9.41 | 1210 | 1 | 71 | 85 |  |
| total 1 peptides |  |  |  |  |  |  |  |  |  |  |  |  |
| --- | --- | --- | --- | --- | --- | --- | --- | --- | --- | --- | --- | --- |

tr|A0A6A4S346|A0A6A4S346\_SCOMX

back to list

  

| Protein Coverage
| Supporting Peptides
|

Protein Coverage:

Supporting Peptides:

| Peptide | Uniq | -10lgP | Mass | ppm | m/z | z | RT | Scan | #Spec | Start | End | PTM |
| --- | --- | --- | --- | --- | --- | --- | --- | --- | --- | --- | --- | --- |
| M.GPRGPPGSPGSSGPQ.G | Y | 50.73 | 1333.6375 | -1.1 | 667.8253 | 2 | 9.41 | 1210 | 1 | 200 | 214 |  |
| total 1 peptides |  |  |  |  |  |  |  |  |  |  |  |  |
| --- | --- | --- | --- | --- | --- | --- | --- | --- | --- | --- | --- | --- |

tr|C6GKU3|C6GKU3\_THUAL

back to list

  

| Protein Coverage
| Supporting Peptides
|

Protein Coverage:

Supporting Peptides:

| Peptide | Uniq | -10lgP | Mass | ppm | m/z | z | RT | Scan | #Spec | Start | End | PTM |
| --- | --- | --- | --- | --- | --- | --- | --- | --- | --- | --- | --- | --- |
| A.GDSDGDGKIGID.E | Y | 36.13 | 1147.4993 | -1.2 | 574.7562 | 2 | 10.59 | 1412 | 1 | 90 | 101 |  |
| A.VIDQDKSGFIE.E | Y | 24.31 | 1249.6190 | -2.1 | 625.8155 | 2 | 11.36 | 1550 | 1 | 50 | 60 |  |
| total 2 peptides |  |  |  |  |  |  |  |  |  |  |  |  |
| --- | --- | --- | --- | --- | --- | --- | --- | --- | --- | --- | --- | --- |

tr|D2KQG3|D2KQG3\_SINCH

back to list

  

| Protein Coverage
| Supporting Peptides
|

Protein Coverage:

Supporting Peptides:

| Peptide | Uniq | -10lgP | Mass | ppm | m/z | z | RT | Scan | #Spec | Start | End | PTM |
| --- | --- | --- | --- | --- | --- | --- | --- | --- | --- | --- | --- | --- |
| A.GDSDGDGKIGID.E | Y | 36.13 | 1147.4993 | -1.2 | 574.7562 | 2 | 10.59 | 1412 | 1 | 90 | 101 |  |
| E.VIDQDKSGFIE.E | Y | 24.31 | 1249.6190 | -2.1 | 625.8155 | 2 | 11.36 | 1550 | 1 | 50 | 60 |  |
| total 2 peptides |  |  |  |  |  |  |  |  |  |  |  |  |
| --- | --- | --- | --- | --- | --- | --- | --- | --- | --- | --- | --- | --- |

tr|A0A7J6CHT3|A0A7J6CHT3\_9TELE

back to list

  

| Protein Coverage
| Supporting Peptides
|

Protein Coverage:

Supporting Peptides:

| Peptide | Uniq | -10lgP | Mass | ppm | m/z | z | RT | Scan | #Spec | Start | End | PTM |
| --- | --- | --- | --- | --- | --- | --- | --- | --- | --- | --- | --- | --- |
| A.GDSDGDGKIGID.E | Y | 36.13 | 1147.4993 | -1.2 | 574.7562 | 2 | 10.59 | 1412 | 1 | 90 | 101 |  |
| A.VIDQDKSGFIE.E | Y | 24.31 | 1249.6190 | -2.1 | 625.8155 | 2 | 11.36 | 1550 | 1 | 50 | 60 |  |
| total 2 peptides |  |  |  |  |  |  |  |  |  |  |  |  |
| --- | --- | --- | --- | --- | --- | --- | --- | --- | --- | --- | --- | --- |

P05941|PRVB\_OPSTA

back to list

  

| Protein Coverage
| Supporting Peptides
|

Protein Coverage:

Supporting Peptides:

| Peptide | Uniq | -10lgP | Mass | ppm | m/z | z | RT | Scan | #Spec | Start | End | PTM |
| --- | --- | --- | --- | --- | --- | --- | --- | --- | --- | --- | --- | --- |
| A.GDSDGDGKIGID.E | Y | 36.13 | 1147.4993 | -1.2 | 574.7562 | 2 | 10.59 | 1412 | 1 | 89 | 100 |  |
| Y.VIDQDKSGFIE.E | Y | 24.31 | 1249.6190 | -2.1 | 625.8155 | 2 | 11.36 | 1550 | 1 | 49 | 59 |  |
| total 2 peptides |  |  |  |  |  |  |  |  |  |  |  |  |
| --- | --- | --- | --- | --- | --- | --- | --- | --- | --- | --- | --- | --- |

tr|B9VJM3|B9VJM3\_SINCH

back to list

  

| Protein Coverage
| Supporting Peptides
|

Protein Coverage:

Supporting Peptides:

| Peptide | Uniq | -10lgP | Mass | ppm | m/z | z | RT | Scan | #Spec | Start | End | PTM |
| --- | --- | --- | --- | --- | --- | --- | --- | --- | --- | --- | --- | --- |
| A.GDSDGDGKIGID.E | Y | 36.13 | 1147.4993 | -1.2 | 574.7562 | 2 | 10.59 | 1412 | 1 | 90 | 101 |  |
| E.VIDQDKSGFIE.E | Y | 24.31 | 1249.6190 | -2.1 | 625.8155 | 2 | 11.36 | 1550 | 1 | 50 | 60 |  |
| total 2 peptides |  |  |  |  |  |  |  |  |  |  |  |  |
| --- | --- | --- | --- | --- | --- | --- | --- | --- | --- | --- | --- | --- |

tr|A0A668SJD1|A0A668SJD1\_OREAU

back to list

  

| Protein Coverage
| Supporting Peptides
|

Protein Coverage:

Supporting Peptides:

| Peptide | Uniq | -10lgP | Mass | ppm | m/z | z | RT | Scan | #Spec | Start | End | PTM |
| --- | --- | --- | --- | --- | --- | --- | --- | --- | --- | --- | --- | --- |
| K.IDYVPR.Q | N | 27.60 | 761.4072 | -1.1 | 381.7104 | 2 | 10.67 | 1426 | 1 | 472 | 477 |  |
| K.MIIGEDPKYL.M | Y | 23.06 | 1177.6052 | -1.8 | 589.8088 | 2 | 12.67 | 1793 | 1 | 1025 | 1034 |  |
| F.VKEPPVTIT.K | N | 21.46 | 982.5699 | -1.7 | 492.2914 | 2 | 10.81 | 1449 | 1 | 285 | 293 |  |
| total 3 peptides |  |  |  |  |  |  |  |  |  |  |  |  |
| --- | --- | --- | --- | --- | --- | --- | --- | --- | --- | --- | --- | --- |

tr|A0A6P7L5W1|A0A6P7L5W1\_BETSP

back to list

  

| Protein Coverage
| Supporting Peptides
|

Protein Coverage:

Supporting Peptides:

| Peptide | Uniq | -10lgP | Mass | ppm | m/z | z | RT | Scan | #Spec | Start | End | PTM |
| --- | --- | --- | --- | --- | --- | --- | --- | --- | --- | --- | --- | --- |
| K.IDYVPR.Q | N | 27.60 | 761.4072 | -1.1 | 381.7104 | 2 | 10.67 | 1426 | 1 | 490 | 495 |  |
| K.MIIGEDPKYL.M | Y | 23.06 | 1177.6052 | -1.8 | 589.8088 | 2 | 12.67 | 1793 | 1 | 1047 | 1056 |  |
| F.VKEPPVTIT.K | N | 21.46 | 982.5699 | -1.7 | 492.2914 | 2 | 10.81 | 1449 | 1 | 303 | 311 |  |
| total 3 peptides |  |  |  |  |  |  |  |  |  |  |  |  |
| --- | --- | --- | --- | --- | --- | --- | --- | --- | --- | --- | --- | --- |

tr|A0A3P9C2F1|A0A3P9C2F1\_9CICH

back to list

  

| Protein Coverage
| Supporting Peptides
|

Protein Coverage:

Supporting Peptides:

| Peptide | Uniq | -10lgP | Mass | ppm | m/z | z | RT | Scan | #Spec | Start | End | PTM |
| --- | --- | --- | --- | --- | --- | --- | --- | --- | --- | --- | --- | --- |
| K.IDYVPR.Q | N | 27.60 | 761.4072 | -1.1 | 381.7104 | 2 | 10.67 | 1426 | 1 | 495 | 500 |  |
| K.MIIGEDPKYL.M | Y | 23.06 | 1177.6052 | -1.8 | 589.8088 | 2 | 12.67 | 1793 | 1 | 1052 | 1061 |  |
| F.VKEPPVTIT.K | N | 21.46 | 982.5699 | -1.7 | 492.2914 | 2 | 10.81 | 1449 | 1 | 308 | 316 |  |
| total 3 peptides |  |  |  |  |  |  |  |  |  |  |  |  |
| --- | --- | --- | --- | --- | --- | --- | --- | --- | --- | --- | --- | --- |

tr|A0A6P7L5V6|A0A6P7L5V6\_BETSP

back to list

  

| Protein Coverage
| Supporting Peptides
|

Protein Coverage:

Supporting Peptides:

| Peptide | Uniq | -10lgP | Mass | ppm | m/z | z | RT | Scan | #Spec | Start | End | PTM |
| --- | --- | --- | --- | --- | --- | --- | --- | --- | --- | --- | --- | --- |
| K.IDYVPR.Q | N | 27.60 | 761.4072 | -1.1 | 381.7104 | 2 | 10.67 | 1426 | 1 | 497 | 502 |  |
| K.MIIGEDPKYL.M | Y | 23.06 | 1177.6052 | -1.8 | 589.8088 | 2 | 12.67 | 1793 | 1 | 1054 | 1063 |  |
| F.VKEPPVTIT.K | N | 21.46 | 982.5699 | -1.7 | 492.2914 | 2 | 10.81 | 1449 | 1 | 310 | 318 |  |
| total 3 peptides |  |  |  |  |  |  |  |  |  |  |  |  |
| --- | --- | --- | --- | --- | --- | --- | --- | --- | --- | --- | --- | --- |

tr|A0A668SIT2|A0A668SIT2\_OREAU

back to list

  

| Protein Coverage
| Supporting Peptides
|

Protein Coverage:

Supporting Peptides:

| Peptide | Uniq | -10lgP | Mass | ppm | m/z | z | RT | Scan | #Spec | Start | End | PTM |
| --- | --- | --- | --- | --- | --- | --- | --- | --- | --- | --- | --- | --- |
| K.IDYVPR.Q | N | 27.60 | 761.4072 | -1.1 | 381.7104 | 2 | 10.67 | 1426 | 1 | 482 | 487 |  |
| K.MIIGEDPKYL.M | Y | 23.06 | 1177.6052 | -1.8 | 589.8088 | 2 | 12.67 | 1793 | 1 | 1052 | 1061 |  |
| F.VKEPPVTIT.K | N | 21.46 | 982.5699 | -1.7 | 492.2914 | 2 | 10.81 | 1449 | 1 | 295 | 303 |  |
| total 3 peptides |  |  |  |  |  |  |  |  |  |  |  |  |
| --- | --- | --- | --- | --- | --- | --- | --- | --- | --- | --- | --- | --- |

tr|A0A6P7L5V2|A0A6P7L5V2\_BETSP

back to list

  

| Protein Coverage
| Supporting Peptides
|

Protein Coverage:

Supporting Peptides:

| Peptide | Uniq | -10lgP | Mass | ppm | m/z | z | RT | Scan | #Spec | Start | End | PTM |
| --- | --- | --- | --- | --- | --- | --- | --- | --- | --- | --- | --- | --- |
| K.IDYVPR.Q | N | 27.60 | 761.4072 | -1.1 | 381.7104 | 2 | 10.67 | 1426 | 1 | 510 | 515 |  |
| K.MIIGEDPKYL.M | Y | 23.06 | 1177.6052 | -1.8 | 589.8088 | 2 | 12.67 | 1793 | 1 | 1067 | 1076 |  |
| F.VKEPPVTIT.K | N | 21.46 | 982.5699 | -1.7 | 492.2914 | 2 | 10.81 | 1449 | 1 | 323 | 331 |  |
| total 3 peptides |  |  |  |  |  |  |  |  |  |  |  |  |
| --- | --- | --- | --- | --- | --- | --- | --- | --- | --- | --- | --- | --- |

tr|A0A6P7INY3|A0A6P7INY3\_9TELE

back to list

  

| Protein Coverage
| Supporting Peptides
|

Protein Coverage:

Supporting Peptides:

| Peptide | Uniq | -10lgP | Mass | ppm | m/z | z | RT | Scan | #Spec | Start | End | PTM |
| --- | --- | --- | --- | --- | --- | --- | --- | --- | --- | --- | --- | --- |
| M.GPRGPPGPPGSSGPQ.G | Y | 46.23 | 1343.6582 | -1.5 | 672.8354 | 2 | 9.65 | 1250 | 1 | 169 | 183 |  |
| total 1 peptides |  |  |  |  |  |  |  |  |  |  |  |  |
| --- | --- | --- | --- | --- | --- | --- | --- | --- | --- | --- | --- | --- |

tr|A0A6P7IU28|A0A6P7IU28\_9TELE

back to list

  

| Protein Coverage
| Supporting Peptides
|

Protein Coverage:

Supporting Peptides:

| Peptide | Uniq | -10lgP | Mass | ppm | m/z | z | RT | Scan | #Spec | Start | End | PTM |
| --- | --- | --- | --- | --- | --- | --- | --- | --- | --- | --- | --- | --- |
| M.GPRGPPGPPGSSGPQ.G | Y | 46.23 | 1343.6582 | -1.5 | 672.8354 | 2 | 9.65 | 1250 | 1 | 169 | 183 |  |
| total 1 peptides |  |  |  |  |  |  |  |  |  |  |  |  |
| --- | --- | --- | --- | --- | --- | --- | --- | --- | --- | --- | --- | --- |

tr|A0A2I4D9D9|A0A2I4D9D9\_9TELE

back to list

  

| Protein Coverage
| Supporting Peptides
|

Protein Coverage:

Supporting Peptides:

| Peptide | Uniq | -10lgP | Mass | ppm | m/z | z | RT | Scan | #Spec | Start | End | PTM |
| --- | --- | --- | --- | --- | --- | --- | --- | --- | --- | --- | --- | --- |
| M.GPRGPPGPPGSSGPQ.G | Y | 46.23 | 1343.6582 | -1.5 | 672.8354 | 2 | 9.65 | 1250 | 1 | 169 | 183 |  |
| total 1 peptides |  |  |  |  |  |  |  |  |  |  |  |  |
| --- | --- | --- | --- | --- | --- | --- | --- | --- | --- | --- | --- | --- |

tr|A0A7J6DBF9|A0A7J6DBF9\_9TELE

back to list

  

| Protein Coverage
| Supporting Peptides
|

Protein Coverage:

Supporting Peptides:

| Peptide | Uniq | -10lgP | Mass | ppm | m/z | z | RT | Scan | #Spec | Start | End | PTM |
| --- | --- | --- | --- | --- | --- | --- | --- | --- | --- | --- | --- | --- |
| M.GPRGPPGPPGSSGPQ.G | Y | 46.23 | 1343.6582 | -1.5 | 672.8354 | 2 | 9.65 | 1250 | 1 | 169 | 183 |  |
| total 1 peptides |  |  |  |  |  |  |  |  |  |  |  |  |
| --- | --- | --- | --- | --- | --- | --- | --- | --- | --- | --- | --- | --- |

tr|A0A2I4D9D6|A0A2I4D9D6\_9TELE

back to list

  

| Protein Coverage
| Supporting Peptides
|

Protein Coverage:

Supporting Peptides:

| Peptide | Uniq | -10lgP | Mass | ppm | m/z | z | RT | Scan | #Spec | Start | End | PTM |
| --- | --- | --- | --- | --- | --- | --- | --- | --- | --- | --- | --- | --- |
| M.GPRGPPGPPGSSGPQ.G | Y | 46.23 | 1343.6582 | -1.5 | 672.8354 | 2 | 9.65 | 1250 | 1 | 169 | 183 |  |
| total 1 peptides |  |  |  |  |  |  |  |  |  |  |  |  |
| --- | --- | --- | --- | --- | --- | --- | --- | --- | --- | --- | --- | --- |

tr|A0A6P6JU61|A0A6P6JU61\_CARAU

back to list

  

| Protein Coverage
| Supporting Peptides
|

Protein Coverage:

Supporting Peptides:

| Peptide | Uniq | -10lgP | Mass | ppm | m/z | z | RT | Scan | #Spec | Start | End | PTM |
| --- | --- | --- | --- | --- | --- | --- | --- | --- | --- | --- | --- | --- |
| M.GPRGPPGPPGSSGPQ.G | Y | 46.23 | 1343.6582 | -1.5 | 672.8354 | 2 | 9.65 | 1250 | 1 | 170 | 184 |  |
| total 1 peptides |  |  |  |  |  |  |  |  |  |  |  |  |
| --- | --- | --- | --- | --- | --- | --- | --- | --- | --- | --- | --- | --- |

tr|A0A3B4UIY5|A0A3B4UIY5\_SERDU

back to list

  

| Protein Coverage
| Supporting Peptides
|

Protein Coverage:

Supporting Peptides:

| Peptide | Uniq | -10lgP | Mass | ppm | m/z | z | RT | Scan | #Spec | Start | End | PTM |
| --- | --- | --- | --- | --- | --- | --- | --- | --- | --- | --- | --- | --- |
| M.GPRGPPGPPGSSGPQ.G | Y | 46.23 | 1343.6582 | -1.5 | 672.8354 | 2 | 9.65 | 1250 | 1 | 149 | 163 |  |
| total 1 peptides |  |  |  |  |  |  |  |  |  |  |  |  |
| --- | --- | --- | --- | --- | --- | --- | --- | --- | --- | --- | --- | --- |

tr|A0A2I4D9D4|A0A2I4D9D4\_9TELE

back to list

  

| Protein Coverage
| Supporting Peptides
|

Protein Coverage:

Supporting Peptides:

| Peptide | Uniq | -10lgP | Mass | ppm | m/z | z | RT | Scan | #Spec | Start | End | PTM |
| --- | --- | --- | --- | --- | --- | --- | --- | --- | --- | --- | --- | --- |
| M.GPRGPPGPPGSSGPQ.G | Y | 46.23 | 1343.6582 | -1.5 | 672.8354 | 2 | 9.65 | 1250 | 1 | 169 | 183 |  |
| total 1 peptides |  |  |  |  |  |  |  |  |  |  |  |  |
| --- | --- | --- | --- | --- | --- | --- | --- | --- | --- | --- | --- | --- |

tr|A0A060WU51|A0A060WU51\_ONCMY

back to list

  

| Protein Coverage
| Supporting Peptides
|

Protein Coverage:

Supporting Peptides:

| Peptide | Uniq | -10lgP | Mass | ppm | m/z | z | RT | Scan | #Spec | Start | End | PTM |
| --- | --- | --- | --- | --- | --- | --- | --- | --- | --- | --- | --- | --- |
| M.GPRGPPGPPGSSGPQ.G | Y | 46.23 | 1343.6582 | -1.5 | 672.8354 | 2 | 9.65 | 1250 | 1 | 170 | 184 |  |
| total 1 peptides |  |  |  |  |  |  |  |  |  |  |  |  |
| --- | --- | --- | --- | --- | --- | --- | --- | --- | --- | --- | --- | --- |

tr|A0A6F9C010|A0A6F9C010\_9TELE

back to list

  

| Protein Coverage
| Supporting Peptides
|

Protein Coverage:

Supporting Peptides:

| Peptide | Uniq | -10lgP | Mass | ppm | m/z | z | RT | Scan | #Spec | Start | End | PTM |
| --- | --- | --- | --- | --- | --- | --- | --- | --- | --- | --- | --- | --- |
| M.GPRGPPGPPGSSGPQ.G | Y | 46.23 | 1343.6582 | -1.5 | 672.8354 | 2 | 9.65 | 1250 | 1 | 170 | 184 |  |
| total 1 peptides |  |  |  |  |  |  |  |  |  |  |  |  |
| --- | --- | --- | --- | --- | --- | --- | --- | --- | --- | --- | --- | --- |

tr|A0A060X5H1|A0A060X5H1\_ONCMY

back to list

  

| Protein Coverage
| Supporting Peptides
|

Protein Coverage:

Supporting Peptides:

| Peptide | Uniq | -10lgP | Mass | ppm | m/z | z | RT | Scan | #Spec | Start | End | PTM |
| --- | --- | --- | --- | --- | --- | --- | --- | --- | --- | --- | --- | --- |
| M.GPRGPPGPPGSSGPQ.G | Y | 46.23 | 1343.6582 | -1.5 | 672.8354 | 2 | 9.65 | 1250 | 1 | 170 | 184 |  |
| total 1 peptides |  |  |  |  |  |  |  |  |  |  |  |  |
| --- | --- | --- | --- | --- | --- | --- | --- | --- | --- | --- | --- | --- |

tr|A0A4W5KL14|A0A4W5KL14\_9TELE

back to list

  

| Protein Coverage
| Supporting Peptides
|

Protein Coverage:

Supporting Peptides:

| Peptide | Uniq | -10lgP | Mass | ppm | m/z | z | RT | Scan | #Spec | Start | End | PTM |
| --- | --- | --- | --- | --- | --- | --- | --- | --- | --- | --- | --- | --- |
| M.GPRGPPGPPGSSGPQ.G | Y | 46.23 | 1343.6582 | -1.5 | 672.8354 | 2 | 9.65 | 1250 | 1 | 170 | 184 |  |
| total 1 peptides |  |  |  |  |  |  |  |  |  |  |  |  |
| --- | --- | --- | --- | --- | --- | --- | --- | --- | --- | --- | --- | --- |

tr|A0A673A8U8|A0A673A8U8\_9TELE

back to list

  

| Protein Coverage
| Supporting Peptides
|

Protein Coverage:

Supporting Peptides:

| Peptide | Uniq | -10lgP | Mass | ppm | m/z | z | RT | Scan | #Spec | Start | End | PTM |
| --- | --- | --- | --- | --- | --- | --- | --- | --- | --- | --- | --- | --- |
| M.GPRGPPGPPGSSGPQ.G | Y | 46.23 | 1343.6582 | -1.5 | 672.8354 | 2 | 9.65 | 1250 | 1 | 78 | 92 |  |
| total 1 peptides |  |  |  |  |  |  |  |  |  |  |  |  |
| --- | --- | --- | --- | --- | --- | --- | --- | --- | --- | --- | --- | --- |

tr|I3JGN1|I3JGN1\_ORENI

back to list

  

| Protein Coverage
| Supporting Peptides
|

Protein Coverage:

Supporting Peptides:

| Peptide | Uniq | -10lgP | Mass | ppm | m/z | z | RT | Scan | #Spec | Start | End | PTM |
| --- | --- | --- | --- | --- | --- | --- | --- | --- | --- | --- | --- | --- |
| M.GPRGPPGPPGSSGPQ.G | Y | 46.23 | 1343.6582 | -1.5 | 672.8354 | 2 | 9.65 | 1250 | 1 | 163 | 177 |  |
| total 1 peptides |  |  |  |  |  |  |  |  |  |  |  |  |
| --- | --- | --- | --- | --- | --- | --- | --- | --- | --- | --- | --- | --- |

tr|A0A671YED8|A0A671YED8\_SPAAU

back to list

  

| Protein Coverage
| Supporting Peptides
|

Protein Coverage:

Supporting Peptides:

| Peptide | Uniq | -10lgP | Mass | ppm | m/z | z | RT | Scan | #Spec | Start | End | PTM |
| --- | --- | --- | --- | --- | --- | --- | --- | --- | --- | --- | --- | --- |
| M.GPRGPPGPPGSSGPQ.G | Y | 46.23 | 1343.6582 | -1.5 | 672.8354 | 2 | 9.65 | 1250 | 1 | 108 | 122 |  |
| total 1 peptides |  |  |  |  |  |  |  |  |  |  |  |  |
| --- | --- | --- | --- | --- | --- | --- | --- | --- | --- | --- | --- | --- |

tr|A0A671YFV1|A0A671YFV1\_SPAAU

back to list

  

| Protein Coverage
| Supporting Peptides
|

Protein Coverage:

Supporting Peptides:

| Peptide | Uniq | -10lgP | Mass | ppm | m/z | z | RT | Scan | #Spec | Start | End | PTM |
| --- | --- | --- | --- | --- | --- | --- | --- | --- | --- | --- | --- | --- |
| M.GPRGPPGPPGSSGPQ.G | Y | 46.23 | 1343.6582 | -1.5 | 672.8354 | 2 | 9.65 | 1250 | 1 | 169 | 183 |  |
| total 1 peptides |  |  |  |  |  |  |  |  |  |  |  |  |
| --- | --- | --- | --- | --- | --- | --- | --- | --- | --- | --- | --- | --- |

tr|A0A3Q2W9Y5|A0A3Q2W9Y5\_HAPBU

back to list

  

| Protein Coverage
| Supporting Peptides
|

Protein Coverage:

Supporting Peptides:

| Peptide | Uniq | -10lgP | Mass | ppm | m/z | z | RT | Scan | #Spec | Start | End | PTM |
| --- | --- | --- | --- | --- | --- | --- | --- | --- | --- | --- | --- | --- |
| M.GPRGPPGPPGSSGPQ.G | Y | 46.23 | 1343.6582 | -1.5 | 672.8354 | 2 | 9.65 | 1250 | 1 | 169 | 183 |  |
| total 1 peptides |  |  |  |  |  |  |  |  |  |  |  |  |
| --- | --- | --- | --- | --- | --- | --- | --- | --- | --- | --- | --- | --- |

tr|A0A674DLV9|A0A674DLV9\_SALTR

back to list

  

| Protein Coverage
| Supporting Peptides
|

Protein Coverage:

Supporting Peptides:

| Peptide | Uniq | -10lgP | Mass | ppm | m/z | z | RT | Scan | #Spec | Start | End | PTM |
| --- | --- | --- | --- | --- | --- | --- | --- | --- | --- | --- | --- | --- |
| M.GPRGPPGPPGSSGPQ.G | Y | 46.23 | 1343.6582 | -1.5 | 672.8354 | 2 | 9.65 | 1250 | 1 | 170 | 184 |  |
| total 1 peptides |  |  |  |  |  |  |  |  |  |  |  |  |
| --- | --- | --- | --- | --- | --- | --- | --- | --- | --- | --- | --- | --- |

tr|A0A3Q3CY74|A0A3Q3CY74\_HAPBU

back to list

  

| Protein Coverage
| Supporting Peptides
|

Protein Coverage:

Supporting Peptides:

| Peptide | Uniq | -10lgP | Mass | ppm | m/z | z | RT | Scan | #Spec | Start | End | PTM |
| --- | --- | --- | --- | --- | --- | --- | --- | --- | --- | --- | --- | --- |
| M.GPRGPPGPPGSSGPQ.G | Y | 46.23 | 1343.6582 | -1.5 | 672.8354 | 2 | 9.65 | 1250 | 1 | 169 | 183 |  |
| total 1 peptides |  |  |  |  |  |  |  |  |  |  |  |  |
| --- | --- | --- | --- | --- | --- | --- | --- | --- | --- | --- | --- | --- |

tr|G9M6I5|G9M6I5\_ORENI

back to list

  

| Protein Coverage
| Supporting Peptides
|

Protein Coverage:

Supporting Peptides:

| Peptide | Uniq | -10lgP | Mass | ppm | m/z | z | RT | Scan | #Spec | Start | End | PTM |
| --- | --- | --- | --- | --- | --- | --- | --- | --- | --- | --- | --- | --- |
| M.GPRGPPGPPGSSGPQ.G | Y | 46.23 | 1343.6582 | -1.5 | 672.8354 | 2 | 9.65 | 1250 | 1 | 169 | 183 |  |
| total 1 peptides |  |  |  |  |  |  |  |  |  |  |  |  |
| --- | --- | --- | --- | --- | --- | --- | --- | --- | --- | --- | --- | --- |

tr|A0A674DL09|A0A674DL09\_SALTR

back to list

  

| Protein Coverage
| Supporting Peptides
|

Protein Coverage:

Supporting Peptides:

| Peptide | Uniq | -10lgP | Mass | ppm | m/z | z | RT | Scan | #Spec | Start | End | PTM |
| --- | --- | --- | --- | --- | --- | --- | --- | --- | --- | --- | --- | --- |
| M.GPRGPPGPPGSSGPQ.G | Y | 46.23 | 1343.6582 | -1.5 | 672.8354 | 2 | 9.65 | 1250 | 1 | 170 | 184 |  |
| total 1 peptides |  |  |  |  |  |  |  |  |  |  |  |  |
| --- | --- | --- | --- | --- | --- | --- | --- | --- | --- | --- | --- | --- |

tr|Q910C0|Q910C0\_ONCMY

back to list

  

| Protein Coverage
| Supporting Peptides
|

Protein Coverage:

Supporting Peptides:

| Peptide | Uniq | -10lgP | Mass | ppm | m/z | z | RT | Scan | #Spec | Start | End | PTM |
| --- | --- | --- | --- | --- | --- | --- | --- | --- | --- | --- | --- | --- |
| M.GPRGPPGPPGSSGPQ.G | Y | 46.23 | 1343.6582 | -1.5 | 672.8354 | 2 | 9.65 | 1250 | 1 | 170 | 184 |  |
| total 1 peptides |  |  |  |  |  |  |  |  |  |  |  |  |
| --- | --- | --- | --- | --- | --- | --- | --- | --- | --- | --- | --- | --- |

tr|A0A1S3S6G4|A0A1S3S6G4\_SALSA

back to list

  

| Protein Coverage
| Supporting Peptides
|

Protein Coverage:

Supporting Peptides:

| Peptide | Uniq | -10lgP | Mass | ppm | m/z | z | RT | Scan | #Spec | Start | End | PTM |
| --- | --- | --- | --- | --- | --- | --- | --- | --- | --- | --- | --- | --- |
| M.GPRGPPGPPGSSGPQ.G | Y | 46.23 | 1343.6582 | -1.5 | 672.8354 | 2 | 9.65 | 1250 | 1 | 170 | 184 |  |
| total 1 peptides |  |  |  |  |  |  |  |  |  |  |  |  |
| --- | --- | --- | --- | --- | --- | --- | --- | --- | --- | --- | --- | --- |

tr|A0A6J2RRG5|A0A6J2RRG5\_COTGO

back to list

  

| Protein Coverage
| Supporting Peptides
|

Protein Coverage:

Supporting Peptides:

| Peptide | Uniq | -10lgP | Mass | ppm | m/z | z | RT | Scan | #Spec | Start | End | PTM |
| --- | --- | --- | --- | --- | --- | --- | --- | --- | --- | --- | --- | --- |
| M.GPRGPPGPPGSSGPQ.G | Y | 46.23 | 1343.6582 | -1.5 | 672.8354 | 2 | 9.65 | 1250 | 1 | 170 | 184 |  |
| total 1 peptides |  |  |  |  |  |  |  |  |  |  |  |  |
| --- | --- | --- | --- | --- | --- | --- | --- | --- | --- | --- | --- | --- |

tr|A0A1S3R8F9|A0A1S3R8F9\_SALSA

back to list

  

| Protein Coverage
| Supporting Peptides
|

Protein Coverage:

Supporting Peptides:

| Peptide | Uniq | -10lgP | Mass | ppm | m/z | z | RT | Scan | #Spec | Start | End | PTM |
| --- | --- | --- | --- | --- | --- | --- | --- | --- | --- | --- | --- | --- |
| M.GPRGPPGPPGSSGPQ.G | Y | 46.23 | 1343.6582 | -1.5 | 672.8354 | 2 | 9.65 | 1250 | 1 | 170 | 184 |  |
| total 1 peptides |  |  |  |  |  |  |  |  |  |  |  |  |
| --- | --- | --- | --- | --- | --- | --- | --- | --- | --- | --- | --- | --- |

tr|A0A1S3NGD5|A0A1S3NGD5\_SALSA

back to list

  

| Protein Coverage
| Supporting Peptides
|

Protein Coverage:

Supporting Peptides:

| Peptide | Uniq | -10lgP | Mass | ppm | m/z | z | RT | Scan | #Spec | Start | End | PTM |
| --- | --- | --- | --- | --- | --- | --- | --- | --- | --- | --- | --- | --- |
| K.IDYVPR.Q | N | 27.60 | 761.4072 | -1.1 | 381.7104 | 2 | 10.67 | 1426 | 1 | 513 | 518 |  |
| F.VKEPPVTIT.K | N | 21.46 | 982.5699 | -1.7 | 492.2914 | 2 | 10.81 | 1449 | 1 | 327 | 335 |  |
| R.VGDKINL.T | Y | 20.52 | 757.4333 | -1.1 | 379.7235 | 2 | 11.14 | 1509 | 1 | 838 | 844 |  |
| total 3 peptides |  |  |  |  |  |  |  |  |  |  |  |  |
| --- | --- | --- | --- | --- | --- | --- | --- | --- | --- | --- | --- | --- |

tr|A0A1A7W9Y4|A0A1A7W9Y4\_9TELE

back to list

  

| Protein Coverage
| Supporting Peptides
|

Protein Coverage:

Supporting Peptides:

| Peptide | Uniq | -10lgP | Mass | ppm | m/z | z | RT | Scan | #Spec | Start | End | PTM |
| --- | --- | --- | --- | --- | --- | --- | --- | --- | --- | --- | --- | --- |
| K.IDYVPR.Q | N | 27.60 | 761.4072 | -1.1 | 381.7104 | 2 | 10.67 | 1426 | 1 | 497 | 502 |  |
| Y.VKEPPVTIT.K | N | 21.46 | 982.5699 | -1.7 | 492.2914 | 2 | 10.81 | 1449 | 1 | 309 | 317 |  |
| K.VGDKINL.T | Y | 20.52 | 757.4333 | -1.1 | 379.7235 | 2 | 11.14 | 1509 | 1 | 822 | 828 |  |
| total 3 peptides |  |  |  |  |  |  |  |  |  |  |  |  |
| --- | --- | --- | --- | --- | --- | --- | --- | --- | --- | --- | --- | --- |

tr|A0A0F8B178|A0A0F8B178\_LARCR

back to list

  

| Protein Coverage
| Supporting Peptides
|

Protein Coverage:

Supporting Peptides:

| Peptide | Uniq | -10lgP | Mass | ppm | m/z | z | RT | Scan | #Spec | Start | End | PTM |
| --- | --- | --- | --- | --- | --- | --- | --- | --- | --- | --- | --- | --- |
| K.TVDGDHVDLD.A | Y | 22.82 | 1084.4673 | -1.3 | 543.2402 | 2 | 10.79 | 1446 | 1 | 365 | 374 |  |
| Q.SLVRMPPWSNF.W | Y | 21.63 | 1332.6648 | -1.9 | 667.3384 | 2 | 14.19 | 2063 | 1 | 912 | 922 |  |
| L.IDEVLKFF.A | Y | 20.20 | 1009.5484 | -0.4 | 505.7813 | 2 | 14.37 | 2094 | 1 | 971 | 978 |  |
| E.VSVGDKVPADIR.I | Y | 16.19 | 1254.6931 | -1.0 | 419.2379 | 3 | 10.68 | 1427 | 2 | 147 | 158 |  |
| total 4 peptides |  |  |  |  |  |  |  |  |  |  |  |  |
| --- | --- | --- | --- | --- | --- | --- | --- | --- | --- | --- | --- | --- |

tr|A0A3P8PD71|A0A3P8PD71\_ASTCA

back to list

  

| Protein Coverage
| Supporting Peptides
|

Protein Coverage:

Supporting Peptides:

| Peptide | Uniq | -10lgP | Mass | ppm | m/z | z | RT | Scan | #Spec | Start | End | PTM |
| --- | --- | --- | --- | --- | --- | --- | --- | --- | --- | --- | --- | --- |
| K.TVDGDHVDLD.A | Y | 22.82 | 1084.4673 | -1.3 | 543.2402 | 2 | 10.79 | 1446 | 1 | 371 | 380 |  |
| Q.SLVRMPPWSNF.W | Y | 21.63 | 1332.6648 | -1.9 | 667.3384 | 2 | 14.19 | 2063 | 1 | 918 | 928 |  |
| A.IDEVLKFF.A | Y | 20.20 | 1009.5484 | -0.4 | 505.7813 | 2 | 14.37 | 2094 | 1 | 977 | 984 |  |
| E.VSVGDKVPADIR.I | Y | 16.19 | 1254.6931 | -1.0 | 419.2379 | 3 | 10.68 | 1427 | 2 | 153 | 164 |  |
| total 4 peptides |  |  |  |  |  |  |  |  |  |  |  |  |
| --- | --- | --- | --- | --- | --- | --- | --- | --- | --- | --- | --- | --- |

tr|A0A3Q2WQG2|A0A3Q2WQG2\_HAPBU

back to list

  

| Protein Coverage
| Supporting Peptides
|

Protein Coverage:

Supporting Peptides:

| Peptide | Uniq | -10lgP | Mass | ppm | m/z | z | RT | Scan | #Spec | Start | End | PTM |
| --- | --- | --- | --- | --- | --- | --- | --- | --- | --- | --- | --- | --- |
| K.TVDGDHVDLD.A | Y | 22.82 | 1084.4673 | -1.3 | 543.2402 | 2 | 10.79 | 1446 | 1 | 371 | 380 |  |
| Q.SLVRMPPWSNF.W | Y | 21.63 | 1332.6648 | -1.9 | 667.3384 | 2 | 14.19 | 2063 | 1 | 918 | 928 |  |
| A.IDEVLKFF.A | Y | 20.20 | 1009.5484 | -0.4 | 505.7813 | 2 | 14.37 | 2094 | 1 | 977 | 984 |  |
| E.VSVGDKVPADIR.I | Y | 16.19 | 1254.6931 | -1.0 | 419.2379 | 3 | 10.68 | 1427 | 2 | 153 | 164 |  |
| total 4 peptides |  |  |  |  |  |  |  |  |  |  |  |  |
| --- | --- | --- | --- | --- | --- | --- | --- | --- | --- | --- | --- | --- |

tr|A0A6G0HZ09|A0A6G0HZ09\_LARCR

back to list

  

| Protein Coverage
| Supporting Peptides
|

Protein Coverage:

Supporting Peptides:

| Peptide | Uniq | -10lgP | Mass | ppm | m/z | z | RT | Scan | #Spec | Start | End | PTM |
| --- | --- | --- | --- | --- | --- | --- | --- | --- | --- | --- | --- | --- |
| K.TVDGDHVDLD.A | Y | 22.82 | 1084.4673 | -1.3 | 543.2402 | 2 | 10.79 | 1446 | 1 | 371 | 380 |  |
| Q.SLVRMPPWSNF.W | Y | 21.63 | 1332.6648 | -1.9 | 667.3384 | 2 | 14.19 | 2063 | 1 | 918 | 928 |  |
| L.IDEVLKFF.A | Y | 20.20 | 1009.5484 | -0.4 | 505.7813 | 2 | 14.37 | 2094 | 1 | 977 | 984 |  |
| E.VSVGDKVPADIR.I | Y | 16.19 | 1254.6931 | -1.0 | 419.2379 | 3 | 10.68 | 1427 | 2 | 153 | 164 |  |
| total 4 peptides |  |  |  |  |  |  |  |  |  |  |  |  |
| --- | --- | --- | --- | --- | --- | --- | --- | --- | --- | --- | --- | --- |

tr|A0A3B4GKY8|A0A3B4GKY8\_9CICH

back to list

  

| Protein Coverage
| Supporting Peptides
|

Protein Coverage:

Supporting Peptides:

| Peptide | Uniq | -10lgP | Mass | ppm | m/z | z | RT | Scan | #Spec | Start | End | PTM |
| --- | --- | --- | --- | --- | --- | --- | --- | --- | --- | --- | --- | --- |
| K.TVDGDHVDLD.A | Y | 22.82 | 1084.4673 | -1.3 | 543.2402 | 2 | 10.79 | 1446 | 1 | 371 | 380 |  |
| Q.SLVRMPPWSNF.W | Y | 21.63 | 1332.6648 | -1.9 | 667.3384 | 2 | 14.19 | 2063 | 1 | 918 | 928 |  |
| A.IDEVLKFF.A | Y | 20.20 | 1009.5484 | -0.4 | 505.7813 | 2 | 14.37 | 2094 | 1 | 977 | 984 |  |
| E.VSVGDKVPADIR.I | Y | 16.19 | 1254.6931 | -1.0 | 419.2379 | 3 | 10.68 | 1427 | 2 | 153 | 164 |  |
| total 4 peptides |  |  |  |  |  |  |  |  |  |  |  |  |
| --- | --- | --- | --- | --- | --- | --- | --- | --- | --- | --- | --- | --- |

tr|A0A3P9C8S8|A0A3P9C8S8\_9CICH

back to list

  

| Protein Coverage
| Supporting Peptides
|

Protein Coverage:

Supporting Peptides:

| Peptide | Uniq | -10lgP | Mass | ppm | m/z | z | RT | Scan | #Spec | Start | End | PTM |
| --- | --- | --- | --- | --- | --- | --- | --- | --- | --- | --- | --- | --- |
| K.TVDGDHVDLD.A | Y | 22.82 | 1084.4673 | -1.3 | 543.2402 | 2 | 10.79 | 1446 | 1 | 371 | 380 |  |
| Q.SLVRMPPWSNF.W | Y | 21.63 | 1332.6648 | -1.9 | 667.3384 | 2 | 14.19 | 2063 | 1 | 918 | 928 |  |
| A.IDEVLKFF.A | Y | 20.20 | 1009.5484 | -0.4 | 505.7813 | 2 | 14.37 | 2094 | 1 | 977 | 984 |  |
| E.VSVGDKVPADIR.I | Y | 16.19 | 1254.6931 | -1.0 | 419.2379 | 3 | 10.68 | 1427 | 2 | 153 | 164 |  |
| total 4 peptides |  |  |  |  |  |  |  |  |  |  |  |  |
| --- | --- | --- | --- | --- | --- | --- | --- | --- | --- | --- | --- | --- |

tr|A0A3P8PD17|A0A3P8PD17\_ASTCA

back to list

  

| Protein Coverage
| Supporting Peptides
|

Protein Coverage:

Supporting Peptides:

| Peptide | Uniq | -10lgP | Mass | ppm | m/z | z | RT | Scan | #Spec | Start | End | PTM |
| --- | --- | --- | --- | --- | --- | --- | --- | --- | --- | --- | --- | --- |
| K.TVDGDHVDLD.A | Y | 22.82 | 1084.4673 | -1.3 | 543.2402 | 2 | 10.79 | 1446 | 1 | 371 | 380 |  |
| Q.SLVRMPPWSNF.W | Y | 21.63 | 1332.6648 | -1.9 | 667.3384 | 2 | 14.19 | 2063 | 1 | 918 | 928 |  |
| A.IDEVLKFF.A | Y | 20.20 | 1009.5484 | -0.4 | 505.7813 | 2 | 14.37 | 2094 | 1 | 977 | 984 |  |
| E.VSVGDKVPADIR.I | Y | 16.19 | 1254.6931 | -1.0 | 419.2379 | 3 | 10.68 | 1427 | 2 | 153 | 164 |  |
| total 4 peptides |  |  |  |  |  |  |  |  |  |  |  |  |
| --- | --- | --- | --- | --- | --- | --- | --- | --- | --- | --- | --- | --- |

tr|A0A3B4GL15|A0A3B4GL15\_9CICH

back to list

  

| Protein Coverage
| Supporting Peptides
|

Protein Coverage:

Supporting Peptides:

| Peptide | Uniq | -10lgP | Mass | ppm | m/z | z | RT | Scan | #Spec | Start | End | PTM |
| --- | --- | --- | --- | --- | --- | --- | --- | --- | --- | --- | --- | --- |
| K.TVDGDHVDLD.A | Y | 22.82 | 1084.4673 | -1.3 | 543.2402 | 2 | 10.79 | 1446 | 1 | 371 | 380 |  |
| Q.SLVRMPPWSNF.W | Y | 21.63 | 1332.6648 | -1.9 | 667.3384 | 2 | 14.19 | 2063 | 1 | 918 | 928 |  |
| A.IDEVLKFF.A | Y | 20.20 | 1009.5484 | -0.4 | 505.7813 | 2 | 14.37 | 2094 | 1 | 977 | 984 |  |
| E.VSVGDKVPADIR.I | Y | 16.19 | 1254.6931 | -1.0 | 419.2379 | 3 | 10.68 | 1427 | 2 | 153 | 164 |  |
| total 4 peptides |  |  |  |  |  |  |  |  |  |  |  |  |
| --- | --- | --- | --- | --- | --- | --- | --- | --- | --- | --- | --- | --- |

tr|A0A3P9C9M1|A0A3P9C9M1\_9CICH

back to list

  

| Protein Coverage
| Supporting Peptides
|

Protein Coverage:

Supporting Peptides:

| Peptide | Uniq | -10lgP | Mass | ppm | m/z | z | RT | Scan | #Spec | Start | End | PTM |
| --- | --- | --- | --- | --- | --- | --- | --- | --- | --- | --- | --- | --- |
| K.TVDGDHVDLD.A | Y | 22.82 | 1084.4673 | -1.3 | 543.2402 | 2 | 10.79 | 1446 | 1 | 371 | 380 |  |
| Q.SLVRMPPWSNF.W | Y | 21.63 | 1332.6648 | -1.9 | 667.3384 | 2 | 14.19 | 2063 | 1 | 918 | 928 |  |
| A.IDEVLKFF.A | Y | 20.20 | 1009.5484 | -0.4 | 505.7813 | 2 | 14.37 | 2094 | 1 | 977 | 984 |  |
| E.VSVGDKVPADIR.I | Y | 16.19 | 1254.6931 | -1.0 | 419.2379 | 3 | 10.68 | 1427 | 2 | 153 | 164 |  |
| total 4 peptides |  |  |  |  |  |  |  |  |  |  |  |  |
| --- | --- | --- | --- | --- | --- | --- | --- | --- | --- | --- | --- | --- |

tr|A0A3Q2WG66|A0A3Q2WG66\_HAPBU

back to list

  

| Protein Coverage
| Supporting Peptides
|

Protein Coverage:

Supporting Peptides:

| Peptide | Uniq | -10lgP | Mass | ppm | m/z | z | RT | Scan | #Spec | Start | End | PTM |
| --- | --- | --- | --- | --- | --- | --- | --- | --- | --- | --- | --- | --- |
| K.TVDGDHVDLD.A | Y | 22.82 | 1084.4673 | -1.3 | 543.2402 | 2 | 10.79 | 1446 | 1 | 371 | 380 |  |
| Q.SLVRMPPWSNF.W | Y | 21.63 | 1332.6648 | -1.9 | 667.3384 | 2 | 14.19 | 2063 | 1 | 918 | 928 |  |
| A.IDEVLKFF.A | Y | 20.20 | 1009.5484 | -0.4 | 505.7813 | 2 | 14.37 | 2094 | 1 | 977 | 984 |  |
| E.VSVGDKVPADIR.I | Y | 16.19 | 1254.6931 | -1.0 | 419.2379 | 3 | 10.68 | 1427 | 2 | 153 | 164 |  |
| total 4 peptides |  |  |  |  |  |  |  |  |  |  |  |  |
| --- | --- | --- | --- | --- | --- | --- | --- | --- | --- | --- | --- | --- |

tr|A0A3P8PDJ9|A0A3P8PDJ9\_ASTCA

back to list

  

| Protein Coverage
| Supporting Peptides
|

Protein Coverage:

Supporting Peptides:

| Peptide | Uniq | -10lgP | Mass | ppm | m/z | z | RT | Scan | #Spec | Start | End | PTM |
| --- | --- | --- | --- | --- | --- | --- | --- | --- | --- | --- | --- | --- |
| K.TVDGDHVDLD.A | Y | 22.82 | 1084.4673 | -1.3 | 543.2402 | 2 | 10.79 | 1446 | 1 | 369 | 378 |  |
| Q.SLVRMPPWSNF.W | Y | 21.63 | 1332.6648 | -1.9 | 667.3384 | 2 | 14.19 | 2063 | 1 | 916 | 926 |  |
| A.IDEVLKFF.A | Y | 20.20 | 1009.5484 | -0.4 | 505.7813 | 2 | 14.37 | 2094 | 1 | 975 | 982 |  |
| E.VSVGDKVPADIR.I | Y | 16.19 | 1254.6931 | -1.0 | 419.2379 | 3 | 10.68 | 1427 | 2 | 151 | 162 |  |
| total 4 peptides |  |  |  |  |  |  |  |  |  |  |  |  |
| --- | --- | --- | --- | --- | --- | --- | --- | --- | --- | --- | --- | --- |

tr|A0A3P8PDC7|A0A3P8PDC7\_ASTCA

back to list

  

| Protein Coverage
| Supporting Peptides
|

Protein Coverage:

Supporting Peptides:

| Peptide | Uniq | -10lgP | Mass | ppm | m/z | z | RT | Scan | #Spec | Start | End | PTM |
| --- | --- | --- | --- | --- | --- | --- | --- | --- | --- | --- | --- | --- |
| K.TVDGDHVDLD.A | Y | 22.82 | 1084.4673 | -1.3 | 543.2402 | 2 | 10.79 | 1446 | 1 | 371 | 380 |  |
| Q.SLVRMPPWSNF.W | Y | 21.63 | 1332.6648 | -1.9 | 667.3384 | 2 | 14.19 | 2063 | 1 | 918 | 928 |  |
| A.IDEVLKFF.A | Y | 20.20 | 1009.5484 | -0.4 | 505.7813 | 2 | 14.37 | 2094 | 1 | 977 | 984 |  |
| E.VSVGDKVPADIR.I | Y | 16.19 | 1254.6931 | -1.0 | 419.2379 | 3 | 10.68 | 1427 | 2 | 153 | 164 |  |
| total 4 peptides |  |  |  |  |  |  |  |  |  |  |  |  |
| --- | --- | --- | --- | --- | --- | --- | --- | --- | --- | --- | --- | --- |

tr|A0A3Q2WG59|A0A3Q2WG59\_HAPBU

back to list

  

| Protein Coverage
| Supporting Peptides
|

Protein Coverage:

Supporting Peptides:

| Peptide | Uniq | -10lgP | Mass | ppm | m/z | z | RT | Scan | #Spec | Start | End | PTM |
| --- | --- | --- | --- | --- | --- | --- | --- | --- | --- | --- | --- | --- |
| K.TVDGDHVDLD.A | Y | 22.82 | 1084.4673 | -1.3 | 543.2402 | 2 | 10.79 | 1446 | 1 | 371 | 380 |  |
| Q.SLVRMPPWSNF.W | Y | 21.63 | 1332.6648 | -1.9 | 667.3384 | 2 | 14.19 | 2063 | 1 | 918 | 928 |  |
| A.IDEVLKFF.A | Y | 20.20 | 1009.5484 | -0.4 | 505.7813 | 2 | 14.37 | 2094 | 1 | 977 | 984 |  |
| E.VSVGDKVPADIR.I | Y | 16.19 | 1254.6931 | -1.0 | 419.2379 | 3 | 10.68 | 1427 | 2 | 153 | 164 |  |
| total 4 peptides |  |  |  |  |  |  |  |  |  |  |  |  |
| --- | --- | --- | --- | --- | --- | --- | --- | --- | --- | --- | --- | --- |

tr|A0A3P8P4C2|A0A3P8P4C2\_ASTCA

back to list

  

| Protein Coverage
| Supporting Peptides
|

Protein Coverage:

Supporting Peptides:

| Peptide | Uniq | -10lgP | Mass | ppm | m/z | z | RT | Scan | #Spec | Start | End | PTM |
| --- | --- | --- | --- | --- | --- | --- | --- | --- | --- | --- | --- | --- |
| K.TIIPPKLPIEIPQPK.T | Y | 35.05 | 1683.0334 | 0.1 | 842.5240 | 2 | 13.77 | 1991 | 1 | 1366 | 1380 |  |
| total 1 peptides |  |  |  |  |  |  |  |  |  |  |  |  |
| --- | --- | --- | --- | --- | --- | --- | --- | --- | --- | --- | --- | --- |

tr|A0A3Q1D109|A0A3Q1D109\_AMPOC

back to list

  

| Protein Coverage
| Supporting Peptides
|

Protein Coverage:

Supporting Peptides:

| Peptide | Uniq | -10lgP | Mass | ppm | m/z | z | RT | Scan | #Spec | Start | End | PTM |
| --- | --- | --- | --- | --- | --- | --- | --- | --- | --- | --- | --- | --- |
| K.TIIPPKLPIEIPQPK.T | Y | 35.05 | 1683.0334 | 0.1 | 842.5240 | 2 | 13.77 | 1991 | 1 | 1366 | 1380 |  |
| total 1 peptides |  |  |  |  |  |  |  |  |  |  |  |  |
| --- | --- | --- | --- | --- | --- | --- | --- | --- | --- | --- | --- | --- |

tr|A0A3Q2WWP1|A0A3Q2WWP1\_HAPBU

back to list

  

| Protein Coverage
| Supporting Peptides
|

Protein Coverage:

Supporting Peptides:

| Peptide | Uniq | -10lgP | Mass | ppm | m/z | z | RT | Scan | #Spec | Start | End | PTM |
| --- | --- | --- | --- | --- | --- | --- | --- | --- | --- | --- | --- | --- |
| K.TIIPPKLPIEIPQPK.T | Y | 35.05 | 1683.0334 | 0.1 | 842.5240 | 2 | 13.77 | 1991 | 1 | 1366 | 1380 |  |
| total 1 peptides |  |  |  |  |  |  |  |  |  |  |  |  |
| --- | --- | --- | --- | --- | --- | --- | --- | --- | --- | --- | --- | --- |

tr|A0A3Q4GHK3|A0A3Q4GHK3\_NEOBR

back to list

  

| Protein Coverage
| Supporting Peptides
|

Protein Coverage:

Supporting Peptides:

| Peptide | Uniq | -10lgP | Mass | ppm | m/z | z | RT | Scan | #Spec | Start | End | PTM |
| --- | --- | --- | --- | --- | --- | --- | --- | --- | --- | --- | --- | --- |
| K.TIIPPKLPIEIPQPK.P | Y | 35.05 | 1683.0334 | 0.1 | 842.5240 | 2 | 13.77 | 1991 | 1 | 858 | 872 |  |
| total 1 peptides |  |  |  |  |  |  |  |  |  |  |  |  |
| --- | --- | --- | --- | --- | --- | --- | --- | --- | --- | --- | --- | --- |

tr|A0A672ZI78|A0A672ZI78\_9TELE

back to list

  

| Protein Coverage
| Supporting Peptides
|

Protein Coverage:

Supporting Peptides:

| Peptide | Uniq | -10lgP | Mass | ppm | m/z | z | RT | Scan | #Spec | Start | End | PTM |
| --- | --- | --- | --- | --- | --- | --- | --- | --- | --- | --- | --- | --- |
| K.TIIPPKLPIEIPQPK.T | Y | 35.05 | 1683.0334 | 0.1 | 842.5240 | 2 | 13.77 | 1991 | 1 | 1101 | 1115 |  |
| total 1 peptides |  |  |  |  |  |  |  |  |  |  |  |  |
| --- | --- | --- | --- | --- | --- | --- | --- | --- | --- | --- | --- | --- |

tr|A0A669ELF6|A0A669ELF6\_ORENI

back to list

  

| Protein Coverage
| Supporting Peptides
|

Protein Coverage:

Supporting Peptides:

| Peptide | Uniq | -10lgP | Mass | ppm | m/z | z | RT | Scan | #Spec | Start | End | PTM |
| --- | --- | --- | --- | --- | --- | --- | --- | --- | --- | --- | --- | --- |
| K.TIIPPKLPIEIPQPK.T | Y | 35.05 | 1683.0334 | 0.1 | 842.5240 | 2 | 13.77 | 1991 | 1 | 1146 | 1160 |  |
| total 1 peptides |  |  |  |  |  |  |  |  |  |  |  |  |
| --- | --- | --- | --- | --- | --- | --- | --- | --- | --- | --- | --- | --- |

tr|A0A4U5U6X9|A0A4U5U6X9\_COLLU

back to list

  

| Protein Coverage
| Supporting Peptides
|

Protein Coverage:

Supporting Peptides:

| Peptide | Uniq | -10lgP | Mass | ppm | m/z | z | RT | Scan | #Spec | Start | End | PTM |
| --- | --- | --- | --- | --- | --- | --- | --- | --- | --- | --- | --- | --- |
| K.TIIPPKLPIEIPQPK.T | Y | 35.05 | 1683.0334 | 0.1 | 842.5240 | 2 | 13.77 | 1991 | 1 | 1190 | 1204 |  |
| total 1 peptides |  |  |  |  |  |  |  |  |  |  |  |  |
| --- | --- | --- | --- | --- | --- | --- | --- | --- | --- | --- | --- | --- |

tr|A0A3Q4GXU7|A0A3Q4GXU7\_NEOBR

back to list

  

| Protein Coverage
| Supporting Peptides
|

Protein Coverage:

Supporting Peptides:

| Peptide | Uniq | -10lgP | Mass | ppm | m/z | z | RT | Scan | #Spec | Start | End | PTM |
| --- | --- | --- | --- | --- | --- | --- | --- | --- | --- | --- | --- | --- |
| K.TIIPPKLPIEIPQPK.P | Y | 35.05 | 1683.0334 | 0.1 | 842.5240 | 2 | 13.77 | 1991 | 1 | 1184 | 1198 |  |
| total 1 peptides |  |  |  |  |  |  |  |  |  |  |  |  |
| --- | --- | --- | --- | --- | --- | --- | --- | --- | --- | --- | --- | --- |

tr|A0A669CKL2|A0A669CKL2\_ORENI

back to list

  

| Protein Coverage
| Supporting Peptides
|

Protein Coverage:

Supporting Peptides:

| Peptide | Uniq | -10lgP | Mass | ppm | m/z | z | RT | Scan | #Spec | Start | End | PTM |
| --- | --- | --- | --- | --- | --- | --- | --- | --- | --- | --- | --- | --- |
| K.TIIPPKLPIEIPQPK.T | Y | 35.05 | 1683.0334 | 0.1 | 842.5240 | 2 | 13.77 | 1991 | 1 | 1224 | 1238 |  |
| total 1 peptides |  |  |  |  |  |  |  |  |  |  |  |  |
| --- | --- | --- | --- | --- | --- | --- | --- | --- | --- | --- | --- | --- |

tr|A0A671Y401|A0A671Y401\_SPAAU

back to list

  

| Protein Coverage
| Supporting Peptides
|

Protein Coverage:

Supporting Peptides:

| Peptide | Uniq | -10lgP | Mass | ppm | m/z | z | RT | Scan | #Spec | Start | End | PTM |
| --- | --- | --- | --- | --- | --- | --- | --- | --- | --- | --- | --- | --- |
| K.TIIPPKLPIEIPQPK.T | Y | 35.05 | 1683.0334 | 0.1 | 842.5240 | 2 | 13.77 | 1991 | 1 | 1268 | 1282 |  |
| total 1 peptides |  |  |  |  |  |  |  |  |  |  |  |  |
| --- | --- | --- | --- | --- | --- | --- | --- | --- | --- | --- | --- | --- |

tr|A0A672FNF5|A0A672FNF5\_SALFA

back to list

  

| Protein Coverage
| Supporting Peptides
|

Protein Coverage:

Supporting Peptides:

| Peptide | Uniq | -10lgP | Mass | ppm | m/z | z | RT | Scan | #Spec | Start | End | PTM |
| --- | --- | --- | --- | --- | --- | --- | --- | --- | --- | --- | --- | --- |
| K.TIIPPKLPIEIPQPK.T | Y | 35.05 | 1683.0334 | 0.1 | 842.5240 | 2 | 13.77 | 1991 | 1 | 1294 | 1308 |  |
| total 1 peptides |  |  |  |  |  |  |  |  |  |  |  |  |
| --- | --- | --- | --- | --- | --- | --- | --- | --- | --- | --- | --- | --- |

tr|A0A6G0J0W8|A0A6G0J0W8\_LARCR

back to list

  

| Protein Coverage
| Supporting Peptides
|

Protein Coverage:

Supporting Peptides:

| Peptide | Uniq | -10lgP | Mass | ppm | m/z | z | RT | Scan | #Spec | Start | End | PTM |
| --- | --- | --- | --- | --- | --- | --- | --- | --- | --- | --- | --- | --- |
| K.TIIPPKLPIEIPQPK.T | Y | 35.05 | 1683.0334 | 0.1 | 842.5240 | 2 | 13.77 | 1991 | 1 | 1335 | 1349 |  |
| total 1 peptides |  |  |  |  |  |  |  |  |  |  |  |  |
| --- | --- | --- | --- | --- | --- | --- | --- | --- | --- | --- | --- | --- |

tr|A0A671Y575|A0A671Y575\_SPAAU

back to list

  

| Protein Coverage
| Supporting Peptides
|

Protein Coverage:

Supporting Peptides:

| Peptide | Uniq | -10lgP | Mass | ppm | m/z | z | RT | Scan | #Spec | Start | End | PTM |
| --- | --- | --- | --- | --- | --- | --- | --- | --- | --- | --- | --- | --- |
| K.TIIPPKLPIEIPQPK.T | Y | 35.05 | 1683.0334 | 0.1 | 842.5240 | 2 | 13.77 | 1991 | 1 | 1349 | 1363 |  |
| total 1 peptides |  |  |  |  |  |  |  |  |  |  |  |  |
| --- | --- | --- | --- | --- | --- | --- | --- | --- | --- | --- | --- | --- |

tr|A0A3B3IJA0|A0A3B3IJA0\_ORYLA

back to list

  

| Protein Coverage
| Supporting Peptides
|

Protein Coverage:

Supporting Peptides:

| Peptide | Uniq | -10lgP | Mass | ppm | m/z | z | RT | Scan | #Spec | Start | End | PTM |
| --- | --- | --- | --- | --- | --- | --- | --- | --- | --- | --- | --- | --- |
| K.TIIPPKLPIEIPQPK.T | Y | 35.05 | 1683.0334 | 0.1 | 842.5240 | 2 | 13.77 | 1991 | 1 | 1351 | 1365 |  |
| total 1 peptides |  |  |  |  |  |  |  |  |  |  |  |  |
| --- | --- | --- | --- | --- | --- | --- | --- | --- | --- | --- | --- | --- |

tr|A0A6G1P7U8|A0A6G1P7U8\_9TELE

back to list

  

| Protein Coverage
| Supporting Peptides
|

Protein Coverage:

Supporting Peptides:

| Peptide | Uniq | -10lgP | Mass | ppm | m/z | z | RT | Scan | #Spec | Start | End | PTM |
| --- | --- | --- | --- | --- | --- | --- | --- | --- | --- | --- | --- | --- |
| K.TIIPPKLPIEIPQPK.T | Y | 35.05 | 1683.0334 | 0.1 | 842.5240 | 2 | 13.77 | 1991 | 1 | 1366 | 1380 |  |
| total 1 peptides |  |  |  |  |  |  |  |  |  |  |  |  |
| --- | --- | --- | --- | --- | --- | --- | --- | --- | --- | --- | --- | --- |

tr|A0A087YFH0|A0A087YFH0\_POEFO

back to list

  

| Protein Coverage
| Supporting Peptides
|

Protein Coverage:

Supporting Peptides:

| Peptide | Uniq | -10lgP | Mass | ppm | m/z | z | RT | Scan | #Spec | Start | End | PTM |
| --- | --- | --- | --- | --- | --- | --- | --- | --- | --- | --- | --- | --- |
| K.TIIPPKLPIEIPQPK.T | Y | 35.05 | 1683.0334 | 0.1 | 842.5240 | 2 | 13.77 | 1991 | 1 | 1365 | 1379 |  |
| total 1 peptides |  |  |  |  |  |  |  |  |  |  |  |  |
| --- | --- | --- | --- | --- | --- | --- | --- | --- | --- | --- | --- | --- |

tr|A0A3P9PGW1|A0A3P9PGW1\_POERE

back to list

  

| Protein Coverage
| Supporting Peptides
|

Protein Coverage:

Supporting Peptides:

| Peptide | Uniq | -10lgP | Mass | ppm | m/z | z | RT | Scan | #Spec | Start | End | PTM |
| --- | --- | --- | --- | --- | --- | --- | --- | --- | --- | --- | --- | --- |
| K.TIIPPKLPIEIPQPK.T | Y | 35.05 | 1683.0334 | 0.1 | 842.5240 | 2 | 13.77 | 1991 | 1 | 1365 | 1379 |  |
| total 1 peptides |  |  |  |  |  |  |  |  |  |  |  |  |
| --- | --- | --- | --- | --- | --- | --- | --- | --- | --- | --- | --- | --- |

tr|A0A672ZHV0|A0A672ZHV0\_9TELE

back to list

  

| Protein Coverage
| Supporting Peptides
|

Protein Coverage:

Supporting Peptides:

| Peptide | Uniq | -10lgP | Mass | ppm | m/z | z | RT | Scan | #Spec | Start | End | PTM |
| --- | --- | --- | --- | --- | --- | --- | --- | --- | --- | --- | --- | --- |
| K.TIIPPKLPIEIPQPK.T | Y | 35.05 | 1683.0334 | 0.1 | 842.5240 | 2 | 13.77 | 1991 | 1 | 1366 | 1380 |  |
| total 1 peptides |  |  |  |  |  |  |  |  |  |  |  |  |
| --- | --- | --- | --- | --- | --- | --- | --- | --- | --- | --- | --- | --- |

tr|I3JDJ1|I3JDJ1\_ORENI

back to list

  

| Protein Coverage
| Supporting Peptides
|

Protein Coverage:

Supporting Peptides:

| Peptide | Uniq | -10lgP | Mass | ppm | m/z | z | RT | Scan | #Spec | Start | End | PTM |
| --- | --- | --- | --- | --- | --- | --- | --- | --- | --- | --- | --- | --- |
| K.TIIPPKLPIEIPQPK.T | Y | 35.05 | 1683.0334 | 0.1 | 842.5240 | 2 | 13.77 | 1991 | 1 | 1366 | 1380 |  |
| total 1 peptides |  |  |  |  |  |  |  |  |  |  |  |  |
| --- | --- | --- | --- | --- | --- | --- | --- | --- | --- | --- | --- | --- |

tr|A0A2I4AP94|A0A2I4AP94\_9TELE

back to list

  

| Protein Coverage
| Supporting Peptides
|

Protein Coverage:

Supporting Peptides:

| Peptide | Uniq | -10lgP | Mass | ppm | m/z | z | RT | Scan | #Spec | Start | End | PTM |
| --- | --- | --- | --- | --- | --- | --- | --- | --- | --- | --- | --- | --- |
| K.TIIPPKLPIEIPQPK.T | Y | 35.05 | 1683.0334 | 0.1 | 842.5240 | 2 | 13.77 | 1991 | 1 | 1366 | 1380 |  |
| total 1 peptides |  |  |  |  |  |  |  |  |  |  |  |  |
| --- | --- | --- | --- | --- | --- | --- | --- | --- | --- | --- | --- | --- |

tr|A0A834CMG6|A0A834CMG6\_ORYME

back to list

  

| Protein Coverage
| Supporting Peptides
|

Protein Coverage:

Supporting Peptides:

| Peptide | Uniq | -10lgP | Mass | ppm | m/z | z | RT | Scan | #Spec | Start | End | PTM |
| --- | --- | --- | --- | --- | --- | --- | --- | --- | --- | --- | --- | --- |
| K.TIIPPKLPIEIPQPK.T | Y | 35.05 | 1683.0334 | 0.1 | 842.5240 | 2 | 13.77 | 1991 | 1 | 1366 | 1380 |  |
| total 1 peptides |  |  |  |  |  |  |  |  |  |  |  |  |
| --- | --- | --- | --- | --- | --- | --- | --- | --- | --- | --- | --- | --- |

tr|A0A3B3BCL7|A0A3B3BCL7\_ORYME

back to list

  

| Protein Coverage
| Supporting Peptides
|

Protein Coverage:

Supporting Peptides:

| Peptide | Uniq | -10lgP | Mass | ppm | m/z | z | RT | Scan | #Spec | Start | End | PTM |
| --- | --- | --- | --- | --- | --- | --- | --- | --- | --- | --- | --- | --- |
| K.TIIPPKLPIEIPQPK.T | Y | 35.05 | 1683.0334 | 0.1 | 842.5240 | 2 | 13.77 | 1991 | 1 | 1366 | 1380 |  |
| total 1 peptides |  |  |  |  |  |  |  |  |  |  |  |  |
| --- | --- | --- | --- | --- | --- | --- | --- | --- | --- | --- | --- | --- |

tr|A0A3P9IT90|A0A3P9IT90\_ORYLA

back to list

  

| Protein Coverage
| Supporting Peptides
|

Protein Coverage:

Supporting Peptides:

| Peptide | Uniq | -10lgP | Mass | ppm | m/z | z | RT | Scan | #Spec | Start | End | PTM |
| --- | --- | --- | --- | --- | --- | --- | --- | --- | --- | --- | --- | --- |
| K.TIIPPKLPIEIPQPK.T | Y | 35.05 | 1683.0334 | 0.1 | 842.5240 | 2 | 13.77 | 1991 | 1 | 1366 | 1380 |  |
| total 1 peptides |  |  |  |  |  |  |  |  |  |  |  |  |
| --- | --- | --- | --- | --- | --- | --- | --- | --- | --- | --- | --- | --- |

tr|H2M2B3|H2M2B3\_ORYLA

back to list

  

| Protein Coverage
| Supporting Peptides
|

Protein Coverage:

Supporting Peptides:

| Peptide | Uniq | -10lgP | Mass | ppm | m/z | z | RT | Scan | #Spec | Start | End | PTM |
| --- | --- | --- | --- | --- | --- | --- | --- | --- | --- | --- | --- | --- |
| K.TIIPPKLPIEIPQPK.T | Y | 35.05 | 1683.0334 | 0.1 | 842.5240 | 2 | 13.77 | 1991 | 1 | 1366 | 1380 |  |
| total 1 peptides |  |  |  |  |  |  |  |  |  |  |  |  |
| --- | --- | --- | --- | --- | --- | --- | --- | --- | --- | --- | --- | --- |

tr|A0A3P9LTE0|A0A3P9LTE0\_ORYLA

back to list

  

| Protein Coverage
| Supporting Peptides
|

Protein Coverage:

Supporting Peptides:

| Peptide | Uniq | -10lgP | Mass | ppm | m/z | z | RT | Scan | #Spec | Start | End | PTM |
| --- | --- | --- | --- | --- | --- | --- | --- | --- | --- | --- | --- | --- |
| K.TIIPPKLPIEIPQPK.T | Y | 35.05 | 1683.0334 | 0.1 | 842.5240 | 2 | 13.77 | 1991 | 1 | 1366 | 1380 |  |
| total 1 peptides |  |  |  |  |  |  |  |  |  |  |  |  |
| --- | --- | --- | --- | --- | --- | --- | --- | --- | --- | --- | --- | --- |

tr|A0A3B3D3C5|A0A3B3D3C5\_ORYME

back to list

  

| Protein Coverage
| Supporting Peptides
|

Protein Coverage:

Supporting Peptides:

| Peptide | Uniq | -10lgP | Mass | ppm | m/z | z | RT | Scan | #Spec | Start | End | PTM |
| --- | --- | --- | --- | --- | --- | --- | --- | --- | --- | --- | --- | --- |
| K.TIIPPKLPIEIPQPK.T | Y | 35.05 | 1683.0334 | 0.1 | 842.5240 | 2 | 13.77 | 1991 | 1 | 1371 | 1385 |  |
| total 1 peptides |  |  |  |  |  |  |  |  |  |  |  |  |
| --- | --- | --- | --- | --- | --- | --- | --- | --- | --- | --- | --- | --- |

tr|A0A6P7LYN0|A0A6P7LYN0\_BETSP

back to list

  

| Protein Coverage
| Supporting Peptides
|

Protein Coverage:

Supporting Peptides:

| Peptide | Uniq | -10lgP | Mass | ppm | m/z | z | RT | Scan | #Spec | Start | End | PTM |
| --- | --- | --- | --- | --- | --- | --- | --- | --- | --- | --- | --- | --- |
| K.TIIPPKLPIELPQPK.T | Y | 35.05 | 1683.0334 | 0.1 | 842.5240 | 2 | 13.77 | 1991 | 1 | 1382 | 1396 |  |
| total 1 peptides |  |  |  |  |  |  |  |  |  |  |  |  |
| --- | --- | --- | --- | --- | --- | --- | --- | --- | --- | --- | --- | --- |

tr|A0A3B3HWS2|A0A3B3HWS2\_ORYLA

back to list

  

| Protein Coverage
| Supporting Peptides
|

Protein Coverage:

Supporting Peptides:

| Peptide | Uniq | -10lgP | Mass | ppm | m/z | z | RT | Scan | #Spec | Start | End | PTM |
| --- | --- | --- | --- | --- | --- | --- | --- | --- | --- | --- | --- | --- |
| K.TIIPPKLPIEIPQPK.T | Y | 35.05 | 1683.0334 | 0.1 | 842.5240 | 2 | 13.77 | 1991 | 1 | 1378 | 1392 |  |
| total 1 peptides |  |  |  |  |  |  |  |  |  |  |  |  |
| --- | --- | --- | --- | --- | --- | --- | --- | --- | --- | --- | --- | --- |

tr|A0A665WT54|A0A665WT54\_ECHNA

back to list

  

| Protein Coverage
| Supporting Peptides
|

Protein Coverage:

Supporting Peptides:

| Peptide | Uniq | -10lgP | Mass | ppm | m/z | z | RT | Scan | #Spec | Start | End | PTM |
| --- | --- | --- | --- | --- | --- | --- | --- | --- | --- | --- | --- | --- |
| M.DSPMPIIPH.Q | Y | 23.87 | 1005.4954 | -1.3 | 503.7543 | 2 | 12.27 | 1720 | 1 | 98 | 106 |  |
| D.SPMPIIPH.Q | Y | 22.14 | 890.4684 | -2.1 | 446.2405 | 2 | 11.82 | 1635 | 1 | 99 | 106 |  |
| total 2 peptides |  |  |  |  |  |  |  |  |  |  |  |  |
| --- | --- | --- | --- | --- | --- | --- | --- | --- | --- | --- | --- | --- |

tr|A0A671N228|A0A671N228\_9TELE

back to list

  

| Protein Coverage
| Supporting Peptides
|

Protein Coverage:

Supporting Peptides:

| Peptide | Uniq | -10lgP | Mass | ppm | m/z | z | RT | Scan | #Spec | Start | End | PTM |
| --- | --- | --- | --- | --- | --- | --- | --- | --- | --- | --- | --- | --- |
| M.DSPMPIIPH.Q | Y | 23.87 | 1005.4954 | -1.3 | 503.7543 | 2 | 12.27 | 1720 | 1 | 98 | 106 |  |
| D.SPMPIIPH.Q | Y | 22.14 | 890.4684 | -2.1 | 446.2405 | 2 | 11.82 | 1635 | 1 | 99 | 106 |  |
| total 2 peptides |  |  |  |  |  |  |  |  |  |  |  |  |
| --- | --- | --- | --- | --- | --- | --- | --- | --- | --- | --- | --- | --- |

tr|A0A3Q3Q7K2|A0A3Q3Q7K2\_MONAL

back to list

  

| Protein Coverage
| Supporting Peptides
|

Protein Coverage:

Supporting Peptides:

| Peptide | Uniq | -10lgP | Mass | ppm | m/z | z | RT | Scan | #Spec | Start | End | PTM |
| --- | --- | --- | --- | --- | --- | --- | --- | --- | --- | --- | --- | --- |
| M.DSPMPIIPH.Q | Y | 23.87 | 1005.4954 | -1.3 | 503.7543 | 2 | 12.27 | 1720 | 1 | 98 | 106 |  |
| D.SPMPIIPH.Q | Y | 22.14 | 890.4684 | -2.1 | 446.2405 | 2 | 11.82 | 1635 | 1 | 99 | 106 |  |
| total 2 peptides |  |  |  |  |  |  |  |  |  |  |  |  |
| --- | --- | --- | --- | --- | --- | --- | --- | --- | --- | --- | --- | --- |

tr|A0A671MU99|A0A671MU99\_9TELE

back to list

  

| Protein Coverage
| Supporting Peptides
|

Protein Coverage:

Supporting Peptides:

| Peptide | Uniq | -10lgP | Mass | ppm | m/z | z | RT | Scan | #Spec | Start | End | PTM |
| --- | --- | --- | --- | --- | --- | --- | --- | --- | --- | --- | --- | --- |
| M.DSPMPIIPH.Q | Y | 23.87 | 1005.4954 | -1.3 | 503.7543 | 2 | 12.27 | 1720 | 1 | 98 | 106 |  |
| D.SPMPIIPH.Q | Y | 22.14 | 890.4684 | -2.1 | 446.2405 | 2 | 11.82 | 1635 | 1 | 99 | 106 |  |
| total 2 peptides |  |  |  |  |  |  |  |  |  |  |  |  |
| --- | --- | --- | --- | --- | --- | --- | --- | --- | --- | --- | --- | --- |

tr|A0A672KK19|A0A672KK19\_SINGR

back to list

  

| Protein Coverage
| Supporting Peptides
|

Protein Coverage:

Supporting Peptides:

| Peptide | Uniq | -10lgP | Mass | ppm | m/z | z | RT | Scan | #Spec | Start | End | PTM |
| --- | --- | --- | --- | --- | --- | --- | --- | --- | --- | --- | --- | --- |
| M.DSPMPIIPH.Q | Y | 23.87 | 1005.4954 | -1.3 | 503.7543 | 2 | 12.27 | 1720 | 1 | 98 | 106 |  |
| D.SPMPIIPH.Q | Y | 22.14 | 890.4684 | -2.1 | 446.2405 | 2 | 11.82 | 1635 | 1 | 99 | 106 |  |
| total 2 peptides |  |  |  |  |  |  |  |  |  |  |  |  |
| --- | --- | --- | --- | --- | --- | --- | --- | --- | --- | --- | --- | --- |

tr|A0A673LYE6|A0A673LYE6\_9TELE

back to list

  

| Protein Coverage
| Supporting Peptides
|

Protein Coverage:

Supporting Peptides:

| Peptide | Uniq | -10lgP | Mass | ppm | m/z | z | RT | Scan | #Spec | Start | End | PTM |
| --- | --- | --- | --- | --- | --- | --- | --- | --- | --- | --- | --- | --- |
| M.DSPMPIIPH.Q | Y | 23.87 | 1005.4954 | -1.3 | 503.7543 | 2 | 12.27 | 1720 | 1 | 98 | 106 |  |
| D.SPMPIIPH.Q | Y | 22.14 | 890.4684 | -2.1 | 446.2405 | 2 | 11.82 | 1635 | 1 | 99 | 106 |  |
| total 2 peptides |  |  |  |  |  |  |  |  |  |  |  |  |
| --- | --- | --- | --- | --- | --- | --- | --- | --- | --- | --- | --- | --- |

tr|A0A3P8VEA1|A0A3P8VEA1\_CYNSE

back to list

  

| Protein Coverage
| Supporting Peptides
|

Protein Coverage:

Supporting Peptides:

| Peptide | Uniq | -10lgP | Mass | ppm | m/z | z | RT | Scan | #Spec | Start | End | PTM |
| --- | --- | --- | --- | --- | --- | --- | --- | --- | --- | --- | --- | --- |
| M.DSPMPIIPH.Q | Y | 23.87 | 1005.4954 | -1.3 | 503.7543 | 2 | 12.27 | 1720 | 1 | 98 | 106 |  |
| D.SPMPIIPH.Q | Y | 22.14 | 890.4684 | -2.1 | 446.2405 | 2 | 11.82 | 1635 | 1 | 99 | 106 |  |
| total 2 peptides |  |  |  |  |  |  |  |  |  |  |  |  |
| --- | --- | --- | --- | --- | --- | --- | --- | --- | --- | --- | --- | --- |

tr|A0A3Q1II99|A0A3Q1II99\_ANATE

back to list

  

| Protein Coverage
| Supporting Peptides
|

Protein Coverage:

Supporting Peptides:

| Peptide | Uniq | -10lgP | Mass | ppm | m/z | z | RT | Scan | #Spec | Start | End | PTM |
| --- | --- | --- | --- | --- | --- | --- | --- | --- | --- | --- | --- | --- |
| M.DSPMPIIPH.Q | Y | 23.87 | 1005.4954 | -1.3 | 503.7543 | 2 | 12.27 | 1720 | 1 | 98 | 106 |  |
| D.SPMPIIPH.Q | Y | 22.14 | 890.4684 | -2.1 | 446.2405 | 2 | 11.82 | 1635 | 1 | 99 | 106 |  |
| total 2 peptides |  |  |  |  |  |  |  |  |  |  |  |  |
| --- | --- | --- | --- | --- | --- | --- | --- | --- | --- | --- | --- | --- |

tr|A0A6P7L9I7|A0A6P7L9I7\_BETSP

back to list

  

| Protein Coverage
| Supporting Peptides
|

Protein Coverage:

Supporting Peptides:

| Peptide | Uniq | -10lgP | Mass | ppm | m/z | z | RT | Scan | #Spec | Start | End | PTM |
| --- | --- | --- | --- | --- | --- | --- | --- | --- | --- | --- | --- | --- |
| M.DSPMPIIPH.Q | Y | 23.87 | 1005.4954 | -1.3 | 503.7543 | 2 | 12.27 | 1720 | 1 | 98 | 106 |  |
| D.SPMPIIPH.Q | Y | 22.14 | 890.4684 | -2.1 | 446.2405 | 2 | 11.82 | 1635 | 1 | 99 | 106 |  |
| total 2 peptides |  |  |  |  |  |  |  |  |  |  |  |  |
| --- | --- | --- | --- | --- | --- | --- | --- | --- | --- | --- | --- | --- |

tr|A0A3P8X8T4|A0A3P8X8T4\_ESOLU

back to list

  

| Protein Coverage
| Supporting Peptides
|

Protein Coverage:

Supporting Peptides:

| Peptide | Uniq | -10lgP | Mass | ppm | m/z | z | RT | Scan | #Spec | Start | End | PTM |
| --- | --- | --- | --- | --- | --- | --- | --- | --- | --- | --- | --- | --- |
| M.DSPMPIIPH.Q | Y | 23.87 | 1005.4954 | -1.3 | 503.7543 | 2 | 12.27 | 1720 | 1 | 97 | 105 |  |
| D.SPMPIIPH.Q | Y | 22.14 | 890.4684 | -2.1 | 446.2405 | 2 | 11.82 | 1635 | 1 | 98 | 105 |  |
| total 2 peptides |  |  |  |  |  |  |  |  |  |  |  |  |
| --- | --- | --- | --- | --- | --- | --- | --- | --- | --- | --- | --- | --- |

tr|A0A3Q0R4X6|A0A3Q0R4X6\_AMPCI

back to list

  

| Protein Coverage
| Supporting Peptides
|

Protein Coverage:

Supporting Peptides:

| Peptide | Uniq | -10lgP | Mass | ppm | m/z | z | RT | Scan | #Spec | Start | End | PTM |
| --- | --- | --- | --- | --- | --- | --- | --- | --- | --- | --- | --- | --- |
| M.DSPMPIIPH.Q | Y | 23.87 | 1005.4954 | -1.3 | 503.7543 | 2 | 12.27 | 1720 | 1 | 98 | 106 |  |
| D.SPMPIIPH.Q | Y | 22.14 | 890.4684 | -2.1 | 446.2405 | 2 | 11.82 | 1635 | 1 | 99 | 106 |  |
| total 2 peptides |  |  |  |  |  |  |  |  |  |  |  |  |
| --- | --- | --- | --- | --- | --- | --- | --- | --- | --- | --- | --- | --- |

tr|A0A3P8VK01|A0A3P8VK01\_CYNSE

back to list

  

| Protein Coverage
| Supporting Peptides
|

Protein Coverage:

Supporting Peptides:

| Peptide | Uniq | -10lgP | Mass | ppm | m/z | z | RT | Scan | #Spec | Start | End | PTM |
| --- | --- | --- | --- | --- | --- | --- | --- | --- | --- | --- | --- | --- |
| M.DSPMPIIPH.Q | Y | 23.87 | 1005.4954 | -1.3 | 503.7543 | 2 | 12.27 | 1720 | 1 | 98 | 106 |  |
| D.SPMPIIPH.Q | Y | 22.14 | 890.4684 | -2.1 | 446.2405 | 2 | 11.82 | 1635 | 1 | 99 | 106 |  |
| total 2 peptides |  |  |  |  |  |  |  |  |  |  |  |  |
| --- | --- | --- | --- | --- | --- | --- | --- | --- | --- | --- | --- | --- |

tr|A0A3P8XBI5|A0A3P8XBI5\_ESOLU

back to list

  

| Protein Coverage
| Supporting Peptides
|

Protein Coverage:

Supporting Peptides:

| Peptide | Uniq | -10lgP | Mass | ppm | m/z | z | RT | Scan | #Spec | Start | End | PTM |
| --- | --- | --- | --- | --- | --- | --- | --- | --- | --- | --- | --- | --- |
| M.DSPMPIIPH.Q | Y | 23.87 | 1005.4954 | -1.3 | 503.7543 | 2 | 12.27 | 1720 | 1 | 97 | 105 |  |
| D.SPMPIIPH.Q | Y | 22.14 | 890.4684 | -2.1 | 446.2405 | 2 | 11.82 | 1635 | 1 | 98 | 105 |  |
| total 2 peptides |  |  |  |  |  |  |  |  |  |  |  |  |
| --- | --- | --- | --- | --- | --- | --- | --- | --- | --- | --- | --- | --- |

tr|A0A3Q1H919|A0A3Q1H919\_ANATE

back to list

  

| Protein Coverage
| Supporting Peptides
|

Protein Coverage:

Supporting Peptides:

| Peptide | Uniq | -10lgP | Mass | ppm | m/z | z | RT | Scan | #Spec | Start | End | PTM |
| --- | --- | --- | --- | --- | --- | --- | --- | --- | --- | --- | --- | --- |
| M.DSPMPIIPH.Q | Y | 23.87 | 1005.4954 | -1.3 | 503.7543 | 2 | 12.27 | 1720 | 1 | 98 | 106 |  |
| D.SPMPIIPH.Q | Y | 22.14 | 890.4684 | -2.1 | 446.2405 | 2 | 11.82 | 1635 | 1 | 99 | 106 |  |
| total 2 peptides |  |  |  |  |  |  |  |  |  |  |  |  |
| --- | --- | --- | --- | --- | --- | --- | --- | --- | --- | --- | --- | --- |

tr|A0A3Q3KN21|A0A3Q3KN21\_9TELE

back to list

  

| Protein Coverage
| Supporting Peptides
|

Protein Coverage:

Supporting Peptides:

| Peptide | Uniq | -10lgP | Mass | ppm | m/z | z | RT | Scan | #Spec | Start | End | PTM |
| --- | --- | --- | --- | --- | --- | --- | --- | --- | --- | --- | --- | --- |
| M.DSPMPIIPH.Q | Y | 23.87 | 1005.4954 | -1.3 | 503.7543 | 2 | 12.27 | 1720 | 1 | 98 | 106 |  |
| D.SPMPIIPH.Q | Y | 22.14 | 890.4684 | -2.1 | 446.2405 | 2 | 11.82 | 1635 | 1 | 99 | 106 |  |
| total 2 peptides |  |  |  |  |  |  |  |  |  |  |  |  |
| --- | --- | --- | --- | --- | --- | --- | --- | --- | --- | --- | --- | --- |

tr|A0A6P7L9I9|A0A6P7L9I9\_BETSP

back to list

  

| Protein Coverage
| Supporting Peptides
|

Protein Coverage:

Supporting Peptides:

| Peptide | Uniq | -10lgP | Mass | ppm | m/z | z | RT | Scan | #Spec | Start | End | PTM |
| --- | --- | --- | --- | --- | --- | --- | --- | --- | --- | --- | --- | --- |
| M.DSPMPIIPH.Q | Y | 23.87 | 1005.4954 | -1.3 | 503.7543 | 2 | 12.27 | 1720 | 1 | 98 | 106 |  |
| D.SPMPIIPH.Q | Y | 22.14 | 890.4684 | -2.1 | 446.2405 | 2 | 11.82 | 1635 | 1 | 99 | 106 |  |
| total 2 peptides |  |  |  |  |  |  |  |  |  |  |  |  |
| --- | --- | --- | --- | --- | --- | --- | --- | --- | --- | --- | --- | --- |

tr|A0A3P8VE68|A0A3P8VE68\_CYNSE

back to list

  

| Protein Coverage
| Supporting Peptides
|

Protein Coverage:

Supporting Peptides:

| Peptide | Uniq | -10lgP | Mass | ppm | m/z | z | RT | Scan | #Spec | Start | End | PTM |
| --- | --- | --- | --- | --- | --- | --- | --- | --- | --- | --- | --- | --- |
| M.DSPMPIIPH.Q | Y | 23.87 | 1005.4954 | -1.3 | 503.7543 | 2 | 12.27 | 1720 | 1 | 98 | 106 |  |
| D.SPMPIIPH.Q | Y | 22.14 | 890.4684 | -2.1 | 446.2405 | 2 | 11.82 | 1635 | 1 | 99 | 106 |  |
| total 2 peptides |  |  |  |  |  |  |  |  |  |  |  |  |
| --- | --- | --- | --- | --- | --- | --- | --- | --- | --- | --- | --- | --- |

tr|A0A6G0HYN5|A0A6G0HYN5\_LARCR

back to list

  

| Protein Coverage
| Supporting Peptides
|

Protein Coverage:

Supporting Peptides:

| Peptide | Uniq | -10lgP | Mass | ppm | m/z | z | RT | Scan | #Spec | Start | End | PTM |
| --- | --- | --- | --- | --- | --- | --- | --- | --- | --- | --- | --- | --- |
| M.DSPMPIIPH.Q | Y | 23.87 | 1005.4954 | -1.3 | 503.7543 | 2 | 12.27 | 1720 | 1 | 98 | 106 |  |
| D.SPMPIIPH.Q | Y | 22.14 | 890.4684 | -2.1 | 446.2405 | 2 | 11.82 | 1635 | 1 | 99 | 106 |  |
| total 2 peptides |  |  |  |  |  |  |  |  |  |  |  |  |
| --- | --- | --- | --- | --- | --- | --- | --- | --- | --- | --- | --- | --- |

tr|A0A3P8ZCR8|A0A3P8ZCR8\_ESOLU

back to list

  

| Protein Coverage
| Supporting Peptides
|

Protein Coverage:

Supporting Peptides:

| Peptide | Uniq | -10lgP | Mass | ppm | m/z | z | RT | Scan | #Spec | Start | End | PTM |
| --- | --- | --- | --- | --- | --- | --- | --- | --- | --- | --- | --- | --- |
| M.DSPMPIIPH.Q | Y | 23.87 | 1005.4954 | -1.3 | 503.7543 | 2 | 12.27 | 1720 | 1 | 97 | 105 |  |
| D.SPMPIIPH.Q | Y | 22.14 | 890.4684 | -2.1 | 446.2405 | 2 | 11.82 | 1635 | 1 | 98 | 105 |  |
| total 2 peptides |  |  |  |  |  |  |  |  |  |  |  |  |
| --- | --- | --- | --- | --- | --- | --- | --- | --- | --- | --- | --- | --- |

tr|A0A3Q3IQV7|A0A3Q3IQV7\_MONAL

back to list

  

| Protein Coverage
| Supporting Peptides
|

Protein Coverage:

Supporting Peptides:

| Peptide | Uniq | -10lgP | Mass | ppm | m/z | z | RT | Scan | #Spec | Start | End | PTM |
| --- | --- | --- | --- | --- | --- | --- | --- | --- | --- | --- | --- | --- |
| M.DSPMPIIPH.Q | Y | 23.87 | 1005.4954 | -1.3 | 503.7543 | 2 | 12.27 | 1720 | 1 | 98 | 106 |  |
| D.SPMPIIPH.Q | Y | 22.14 | 890.4684 | -2.1 | 446.2405 | 2 | 11.82 | 1635 | 1 | 99 | 106 |  |
| total 2 peptides |  |  |  |  |  |  |  |  |  |  |  |  |
| --- | --- | --- | --- | --- | --- | --- | --- | --- | --- | --- | --- | --- |

tr|A0A665WTS5|A0A665WTS5\_ECHNA

back to list

  

| Protein Coverage
| Supporting Peptides
|

Protein Coverage:

Supporting Peptides:

| Peptide | Uniq | -10lgP | Mass | ppm | m/z | z | RT | Scan | #Spec | Start | End | PTM |
| --- | --- | --- | --- | --- | --- | --- | --- | --- | --- | --- | --- | --- |
| M.DSPMPIIPH.Q | Y | 23.87 | 1005.4954 | -1.3 | 503.7543 | 2 | 12.27 | 1720 | 1 | 98 | 106 |  |
| D.SPMPIIPH.Q | Y | 22.14 | 890.4684 | -2.1 | 446.2405 | 2 | 11.82 | 1635 | 1 | 99 | 106 |  |
| total 2 peptides |  |  |  |  |  |  |  |  |  |  |  |  |
| --- | --- | --- | --- | --- | --- | --- | --- | --- | --- | --- | --- | --- |

tr|A0A7N8Y585|A0A7N8Y585\_9TELE

back to list

  

| Protein Coverage
| Supporting Peptides
|

Protein Coverage:

Supporting Peptides:

| Peptide | Uniq | -10lgP | Mass | ppm | m/z | z | RT | Scan | #Spec | Start | End | PTM |
| --- | --- | --- | --- | --- | --- | --- | --- | --- | --- | --- | --- | --- |
| M.DSPMPIIPH.Q | Y | 23.87 | 1005.4954 | -1.3 | 503.7543 | 2 | 12.27 | 1720 | 1 | 101 | 109 |  |
| D.SPMPIIPH.Q | Y | 22.14 | 890.4684 | -2.1 | 446.2405 | 2 | 11.82 | 1635 | 1 | 102 | 109 |  |
| total 2 peptides |  |  |  |  |  |  |  |  |  |  |  |  |
| --- | --- | --- | --- | --- | --- | --- | --- | --- | --- | --- | --- | --- |

tr|A0A6P6QFT2|A0A6P6QFT2\_CARAU

back to list

  

| Protein Coverage
| Supporting Peptides
|

Protein Coverage:

Supporting Peptides:

| Peptide | Uniq | -10lgP | Mass | ppm | m/z | z | RT | Scan | #Spec | Start | End | PTM |
| --- | --- | --- | --- | --- | --- | --- | --- | --- | --- | --- | --- | --- |
| M.DSPMPIIPH.Q | Y | 23.87 | 1005.4954 | -1.3 | 503.7543 | 2 | 12.27 | 1720 | 1 | 98 | 106 |  |
| D.SPMPIIPH.Q | Y | 22.14 | 890.4684 | -2.1 | 446.2405 | 2 | 11.82 | 1635 | 1 | 99 | 106 |  |
| total 2 peptides |  |  |  |  |  |  |  |  |  |  |  |  |
| --- | --- | --- | --- | --- | --- | --- | --- | --- | --- | --- | --- | --- |

tr|A0A672KNU9|A0A672KNU9\_SINGR

back to list

  

| Protein Coverage
| Supporting Peptides
|

Protein Coverage:

Supporting Peptides:

| Peptide | Uniq | -10lgP | Mass | ppm | m/z | z | RT | Scan | #Spec | Start | End | PTM |
| --- | --- | --- | --- | --- | --- | --- | --- | --- | --- | --- | --- | --- |
| M.DSPMPIIPH.Q | Y | 23.87 | 1005.4954 | -1.3 | 503.7543 | 2 | 12.27 | 1720 | 1 | 98 | 106 |  |
| D.SPMPIIPH.Q | Y | 22.14 | 890.4684 | -2.1 | 446.2405 | 2 | 11.82 | 1635 | 1 | 99 | 106 |  |
| total 2 peptides |  |  |  |  |  |  |  |  |  |  |  |  |
| --- | --- | --- | --- | --- | --- | --- | --- | --- | --- | --- | --- | --- |

tr|A0A673M1Q3|A0A673M1Q3\_9TELE

back to list

  

| Protein Coverage
| Supporting Peptides
|

Protein Coverage:

Supporting Peptides:

| Peptide | Uniq | -10lgP | Mass | ppm | m/z | z | RT | Scan | #Spec | Start | End | PTM |
| --- | --- | --- | --- | --- | --- | --- | --- | --- | --- | --- | --- | --- |
| M.DSPMPIIPH.Q | Y | 23.87 | 1005.4954 | -1.3 | 503.7543 | 2 | 12.27 | 1720 | 1 | 98 | 106 |  |
| D.SPMPIIPH.Q | Y | 22.14 | 890.4684 | -2.1 | 446.2405 | 2 | 11.82 | 1635 | 1 | 99 | 106 |  |
| total 2 peptides |  |  |  |  |  |  |  |  |  |  |  |  |
| --- | --- | --- | --- | --- | --- | --- | --- | --- | --- | --- | --- | --- |

tr|A0A7J6CGV6|A0A7J6CGV6\_9TELE

back to list

  

| Protein Coverage
| Supporting Peptides
|

Protein Coverage:

Supporting Peptides:

| Peptide | Uniq | -10lgP | Mass | ppm | m/z | z | RT | Scan | #Spec | Start | End | PTM |
| --- | --- | --- | --- | --- | --- | --- | --- | --- | --- | --- | --- | --- |
| M.DSPMPIIPH.Q | Y | 23.87 | 1005.4954 | -1.3 | 503.7543 | 2 | 12.27 | 1720 | 1 | 98 | 106 |  |
| D.SPMPIIPH.Q | Y | 22.14 | 890.4684 | -2.1 | 446.2405 | 2 | 11.82 | 1635 | 1 | 99 | 106 |  |
| total 2 peptides |  |  |  |  |  |  |  |  |  |  |  |  |
| --- | --- | --- | --- | --- | --- | --- | --- | --- | --- | --- | --- | --- |

tr|A0A6P6QIW1|A0A6P6QIW1\_CARAU

back to list

  

| Protein Coverage
| Supporting Peptides
|

Protein Coverage:

Supporting Peptides:

| Peptide | Uniq | -10lgP | Mass | ppm | m/z | z | RT | Scan | #Spec | Start | End | PTM |
| --- | --- | --- | --- | --- | --- | --- | --- | --- | --- | --- | --- | --- |
| M.DSPMPIIPH.Q | Y | 23.87 | 1005.4954 | -1.3 | 503.7543 | 2 | 12.27 | 1720 | 1 | 98 | 106 |  |
| D.SPMPIIPH.Q | Y | 22.14 | 890.4684 | -2.1 | 446.2405 | 2 | 11.82 | 1635 | 1 | 99 | 106 |  |
| total 2 peptides |  |  |  |  |  |  |  |  |  |  |  |  |
| --- | --- | --- | --- | --- | --- | --- | --- | --- | --- | --- | --- | --- |

tr|A0A6Q2XWF7|A0A6Q2XWF7\_ESOLU

back to list

  

| Protein Coverage
| Supporting Peptides
|

Protein Coverage:

Supporting Peptides:

| Peptide | Uniq | -10lgP | Mass | ppm | m/z | z | RT | Scan | #Spec | Start | End | PTM |
| --- | --- | --- | --- | --- | --- | --- | --- | --- | --- | --- | --- | --- |
| M.DSPMPIIPH.Q | Y | 23.87 | 1005.4954 | -1.3 | 503.7543 | 2 | 12.27 | 1720 | 1 | 97 | 105 |  |
| D.SPMPIIPH.Q | Y | 22.14 | 890.4684 | -2.1 | 446.2405 | 2 | 11.82 | 1635 | 1 | 98 | 105 |  |
| total 2 peptides |  |  |  |  |  |  |  |  |  |  |  |  |
| --- | --- | --- | --- | --- | --- | --- | --- | --- | --- | --- | --- | --- |

tr|A0A0F8AMH6|A0A0F8AMH6\_LARCR

back to list

  

| Protein Coverage
| Supporting Peptides
|

Protein Coverage:

Supporting Peptides:

| Peptide | Uniq | -10lgP | Mass | ppm | m/z | z | RT | Scan | #Spec | Start | End | PTM |
| --- | --- | --- | --- | --- | --- | --- | --- | --- | --- | --- | --- | --- |
| M.DSPMPIIPH.Q | Y | 23.87 | 1005.4954 | -1.3 | 503.7543 | 2 | 12.27 | 1720 | 1 | 98 | 106 |  |
| D.SPMPIIPH.Q | Y | 22.14 | 890.4684 | -2.1 | 446.2405 | 2 | 11.82 | 1635 | 1 | 99 | 106 |  |
| total 2 peptides |  |  |  |  |  |  |  |  |  |  |  |  |
| --- | --- | --- | --- | --- | --- | --- | --- | --- | --- | --- | --- | --- |

tr|A0A6G0HYU6|A0A6G0HYU6\_LARCR

back to list

  

| Protein Coverage
| Supporting Peptides
|

Protein Coverage:

Supporting Peptides:

| Peptide | Uniq | -10lgP | Mass | ppm | m/z | z | RT | Scan | #Spec | Start | End | PTM |
| --- | --- | --- | --- | --- | --- | --- | --- | --- | --- | --- | --- | --- |
| M.DSPMPIIPH.Q | Y | 23.87 | 1005.4954 | -1.3 | 503.7543 | 2 | 12.27 | 1720 | 1 | 545 | 553 |  |
| D.SPMPIIPH.Q | Y | 22.14 | 890.4684 | -2.1 | 446.2405 | 2 | 11.82 | 1635 | 1 | 546 | 553 |  |
| total 2 peptides |  |  |  |  |  |  |  |  |  |  |  |  |
| --- | --- | --- | --- | --- | --- | --- | --- | --- | --- | --- | --- | --- |

tr|A0A0P7UGN8|A0A0P7UGN8\_SCLFO

back to list

  

| Protein Coverage
| Supporting Peptides
|

Protein Coverage:

Supporting Peptides:

| Peptide | Uniq | -10lgP | Mass | ppm | m/z | z | RT | Scan | #Spec | Start | End | PTM |
| --- | --- | --- | --- | --- | --- | --- | --- | --- | --- | --- | --- | --- |
| Q.IIVPALPQ.S | Y | 30.17 | 849.5323 | -1.3 | 425.7729 | 2 | 13.25 | 1898 | 1 | 263 | 270 |  |
| total 1 peptides |  |  |  |  |  |  |  |  |  |  |  |  |
| --- | --- | --- | --- | --- | --- | --- | --- | --- | --- | --- | --- | --- |

tr|A0A3P8UKD4|A0A3P8UKD4\_CYNSE

back to list

  

| Protein Coverage
| Supporting Peptides
|

Protein Coverage:

Supporting Peptides:

| Peptide | Uniq | -10lgP | Mass | ppm | m/z | z | RT | Scan | #Spec | Start | End | PTM |
| --- | --- | --- | --- | --- | --- | --- | --- | --- | --- | --- | --- | --- |
| L.IIVPALPQ.D | Y | 30.17 | 849.5323 | -1.3 | 425.7729 | 2 | 13.25 | 1898 | 1 | 102 | 109 |  |
| total 1 peptides |  |  |  |  |  |  |  |  |  |  |  |  |
| --- | --- | --- | --- | --- | --- | --- | --- | --- | --- | --- | --- | --- |

tr|A0A668SIM0|A0A668SIM0\_OREAU

back to list

  

| Protein Coverage
| Supporting Peptides
|

Protein Coverage:

Supporting Peptides:

| Peptide | Uniq | -10lgP | Mass | ppm | m/z | z | RT | Scan | #Spec | Start | End | PTM |
| --- | --- | --- | --- | --- | --- | --- | --- | --- | --- | --- | --- | --- |
| L.IIVPALPQ.D | Y | 30.17 | 849.5323 | -1.3 | 425.7729 | 2 | 13.25 | 1898 | 1 | 262 | 269 |  |
| total 1 peptides |  |  |  |  |  |  |  |  |  |  |  |  |
| --- | --- | --- | --- | --- | --- | --- | --- | --- | --- | --- | --- | --- |

tr|A0A6P7NE86|A0A6P7NE86\_BETSP

back to list

  

| Protein Coverage
| Supporting Peptides
|

Protein Coverage:

Supporting Peptides:

| Peptide | Uniq | -10lgP | Mass | ppm | m/z | z | RT | Scan | #Spec | Start | End | PTM |
| --- | --- | --- | --- | --- | --- | --- | --- | --- | --- | --- | --- | --- |
| Q.IIVPALPQ.S | Y | 30.17 | 849.5323 | -1.3 | 425.7729 | 2 | 13.25 | 1898 | 1 | 164 | 171 |  |
| total 1 peptides |  |  |  |  |  |  |  |  |  |  |  |  |
| --- | --- | --- | --- | --- | --- | --- | --- | --- | --- | --- | --- | --- |

tr|A0A5J5CFY0|A0A5J5CFY0\_9PERO

back to list

  

| Protein Coverage
| Supporting Peptides
|

Protein Coverage:

Supporting Peptides:

| Peptide | Uniq | -10lgP | Mass | ppm | m/z | z | RT | Scan | #Spec | Start | End | PTM |
| --- | --- | --- | --- | --- | --- | --- | --- | --- | --- | --- | --- | --- |
| L.IIVPALPQ.D | Y | 30.17 | 849.5323 | -1.3 | 425.7729 | 2 | 13.25 | 1898 | 1 | 164 | 171 |  |
| total 1 peptides |  |  |  |  |  |  |  |  |  |  |  |  |
| --- | --- | --- | --- | --- | --- | --- | --- | --- | --- | --- | --- | --- |

tr|A0A315V5I0|A0A315V5I0\_GAMAF

back to list

  

| Protein Coverage
| Supporting Peptides
|

Protein Coverage:

Supporting Peptides:

| Peptide | Uniq | -10lgP | Mass | ppm | m/z | z | RT | Scan | #Spec | Start | End | PTM |
| --- | --- | --- | --- | --- | --- | --- | --- | --- | --- | --- | --- | --- |
| Q.IIVPALPQ.S | Y | 30.17 | 849.5323 | -1.3 | 425.7729 | 2 | 13.25 | 1898 | 1 | 242 | 249 |  |
| total 1 peptides |  |  |  |  |  |  |  |  |  |  |  |  |
| --- | --- | --- | --- | --- | --- | --- | --- | --- | --- | --- | --- | --- |

tr|A0A6P7NIN0|A0A6P7NIN0\_BETSP

back to list

  

| Protein Coverage
| Supporting Peptides
|

Protein Coverage:

Supporting Peptides:

| Peptide | Uniq | -10lgP | Mass | ppm | m/z | z | RT | Scan | #Spec | Start | End | PTM |
| --- | --- | --- | --- | --- | --- | --- | --- | --- | --- | --- | --- | --- |
| Q.IIVPALPQ.S | Y | 30.17 | 849.5323 | -1.3 | 425.7729 | 2 | 13.25 | 1898 | 1 | 242 | 249 |  |
| total 1 peptides |  |  |  |  |  |  |  |  |  |  |  |  |
| --- | --- | --- | --- | --- | --- | --- | --- | --- | --- | --- | --- | --- |

tr|A0A6J2QDT9|A0A6J2QDT9\_COTGO

back to list

  

| Protein Coverage
| Supporting Peptides
|

Protein Coverage:

Supporting Peptides:

| Peptide | Uniq | -10lgP | Mass | ppm | m/z | z | RT | Scan | #Spec | Start | End | PTM |
| --- | --- | --- | --- | --- | --- | --- | --- | --- | --- | --- | --- | --- |
| Q.IIVPALPQ.S | Y | 30.17 | 849.5323 | -1.3 | 425.7729 | 2 | 13.25 | 1898 | 1 | 242 | 249 |  |
| total 1 peptides |  |  |  |  |  |  |  |  |  |  |  |  |
| --- | --- | --- | --- | --- | --- | --- | --- | --- | --- | --- | --- | --- |

tr|M3ZP84|M3ZP84\_XIPMA

back to list

  

| Protein Coverage
| Supporting Peptides
|

Protein Coverage:

Supporting Peptides:

| Peptide | Uniq | -10lgP | Mass | ppm | m/z | z | RT | Scan | #Spec | Start | End | PTM |
| --- | --- | --- | --- | --- | --- | --- | --- | --- | --- | --- | --- | --- |
| Q.IIVPALPQ.S | Y | 30.17 | 849.5323 | -1.3 | 425.7729 | 2 | 13.25 | 1898 | 1 | 242 | 249 |  |
| total 1 peptides |  |  |  |  |  |  |  |  |  |  |  |  |
| --- | --- | --- | --- | --- | --- | --- | --- | --- | --- | --- | --- | --- |

tr|A0A3P9PCX4|A0A3P9PCX4\_POERE

back to list

  

| Protein Coverage
| Supporting Peptides
|

Protein Coverage:

Supporting Peptides:

| Peptide | Uniq | -10lgP | Mass | ppm | m/z | z | RT | Scan | #Spec | Start | End | PTM |
| --- | --- | --- | --- | --- | --- | --- | --- | --- | --- | --- | --- | --- |
| Q.IIVPALPQ.S | Y | 30.17 | 849.5323 | -1.3 | 425.7729 | 2 | 13.25 | 1898 | 1 | 242 | 249 |  |
| total 1 peptides |  |  |  |  |  |  |  |  |  |  |  |  |
| --- | --- | --- | --- | --- | --- | --- | --- | --- | --- | --- | --- | --- |

tr|A0A3B3WUC6|A0A3B3WUC6\_9TELE

back to list

  

| Protein Coverage
| Supporting Peptides
|

Protein Coverage:

Supporting Peptides:

| Peptide | Uniq | -10lgP | Mass | ppm | m/z | z | RT | Scan | #Spec | Start | End | PTM |
| --- | --- | --- | --- | --- | --- | --- | --- | --- | --- | --- | --- | --- |
| Q.IIVPALPQ.S | Y | 30.17 | 849.5323 | -1.3 | 425.7729 | 2 | 13.25 | 1898 | 1 | 242 | 249 |  |
| total 1 peptides |  |  |  |  |  |  |  |  |  |  |  |  |
| --- | --- | --- | --- | --- | --- | --- | --- | --- | --- | --- | --- | --- |

tr|A0A6G0J5Q9|A0A6G0J5Q9\_LARCR

back to list

  

| Protein Coverage
| Supporting Peptides
|

Protein Coverage:

Supporting Peptides:

| Peptide | Uniq | -10lgP | Mass | ppm | m/z | z | RT | Scan | #Spec | Start | End | PTM |
| --- | --- | --- | --- | --- | --- | --- | --- | --- | --- | --- | --- | --- |
| L.IIVPALPQ.D | Y | 30.17 | 849.5323 | -1.3 | 425.7729 | 2 | 13.25 | 1898 | 1 | 1289 | 1296 |  |
| total 1 peptides |  |  |  |  |  |  |  |  |  |  |  |  |
| --- | --- | --- | --- | --- | --- | --- | --- | --- | --- | --- | --- | --- |

tr|A0A6J2RVQ6|A0A6J2RVQ6\_COTGO

back to list

  

| Protein Coverage
| Supporting Peptides
|

Protein Coverage:

Supporting Peptides:

| Peptide | Uniq | -10lgP | Mass | ppm | m/z | z | RT | Scan | #Spec | Start | End | PTM |
| --- | --- | --- | --- | --- | --- | --- | --- | --- | --- | --- | --- | --- |
| R.SIQDDPLLVH.Y | Y | 20.27 | 1135.5873 | -1.5 | 568.8000 | 2 | 12.24 | 1715 | 1 | 835 | 844 |  |
| C.VIPVVDDKLTL.L | Y | 15.71 | 1210.7173 | -1.2 | 606.3652 | 2 | 13.28 | 1904 | 1 | 5321 | 5331 |  |
| total 2 peptides |  |  |  |  |  |  |  |  |  |  |  |  |
| --- | --- | --- | --- | --- | --- | --- | --- | --- | --- | --- | --- | --- |

tr|A0A6J2RYJ1|A0A6J2RYJ1\_COTGO

back to list

  

| Protein Coverage
| Supporting Peptides
|

Protein Coverage:

Supporting Peptides:

| Peptide | Uniq | -10lgP | Mass | ppm | m/z | z | RT | Scan | #Spec | Start | End | PTM |
| --- | --- | --- | --- | --- | --- | --- | --- | --- | --- | --- | --- | --- |
| R.SIQDDPLLVH.Y | Y | 20.27 | 1135.5873 | -1.5 | 568.8000 | 2 | 12.24 | 1715 | 1 | 835 | 844 |  |
| C.VIPVVDDKLTL.L | Y | 15.71 | 1210.7173 | -1.2 | 606.3652 | 2 | 13.28 | 1904 | 1 | 5321 | 5331 |  |
| total 2 peptides |  |  |  |  |  |  |  |  |  |  |  |  |
| --- | --- | --- | --- | --- | --- | --- | --- | --- | --- | --- | --- | --- |

tr|A0A6J2RTY3|A0A6J2RTY3\_COTGO

back to list

  

| Protein Coverage
| Supporting Peptides
|

Protein Coverage:

Supporting Peptides:

| Peptide | Uniq | -10lgP | Mass | ppm | m/z | z | RT | Scan | #Spec | Start | End | PTM |
| --- | --- | --- | --- | --- | --- | --- | --- | --- | --- | --- | --- | --- |
| R.SIQDDPLLVH.Y | Y | 20.27 | 1135.5873 | -1.5 | 568.8000 | 2 | 12.24 | 1715 | 1 | 835 | 844 |  |
| C.VIPVVDDKLTL.L | Y | 15.71 | 1210.7173 | -1.2 | 606.3652 | 2 | 13.28 | 1904 | 1 | 5077 | 5087 |  |
| total 2 peptides |  |  |  |  |  |  |  |  |  |  |  |  |
| --- | --- | --- | --- | --- | --- | --- | --- | --- | --- | --- | --- | --- |

tr|A0A6J2RY66|A0A6J2RY66\_COTGO

back to list

  

| Protein Coverage
| Supporting Peptides
|

Protein Coverage:

Supporting Peptides:

| Peptide | Uniq | -10lgP | Mass | ppm | m/z | z | RT | Scan | #Spec | Start | End | PTM |
| --- | --- | --- | --- | --- | --- | --- | --- | --- | --- | --- | --- | --- |
| R.SIQDDPLLVH.Y | Y | 20.27 | 1135.5873 | -1.5 | 568.8000 | 2 | 12.24 | 1715 | 1 | 835 | 844 |  |
| C.VIPVVDDKLTL.L | Y | 15.71 | 1210.7173 | -1.2 | 606.3652 | 2 | 13.28 | 1904 | 1 | 5321 | 5331 |  |
| total 2 peptides |  |  |  |  |  |  |  |  |  |  |  |  |
| --- | --- | --- | --- | --- | --- | --- | --- | --- | --- | --- | --- | --- |

tr|A0A6J2RVQ1|A0A6J2RVQ1\_COTGO

back to list

  

| Protein Coverage
| Supporting Peptides
|

Protein Coverage:

Supporting Peptides:

| Peptide | Uniq | -10lgP | Mass | ppm | m/z | z | RT | Scan | #Spec | Start | End | PTM |
| --- | --- | --- | --- | --- | --- | --- | --- | --- | --- | --- | --- | --- |
| R.SIQDDPLLVH.Y | Y | 20.27 | 1135.5873 | -1.5 | 568.8000 | 2 | 12.24 | 1715 | 1 | 835 | 844 |  |
| C.VIPVVDDKLTL.L | Y | 15.71 | 1210.7173 | -1.2 | 606.3652 | 2 | 13.28 | 1904 | 1 | 5321 | 5331 |  |
| total 2 peptides |  |  |  |  |  |  |  |  |  |  |  |  |
| --- | --- | --- | --- | --- | --- | --- | --- | --- | --- | --- | --- | --- |

tr|A0A3Q1HC41|A0A3Q1HC41\_ANATE

back to list

  

| Protein Coverage
| Supporting Peptides
|

Protein Coverage:

Supporting Peptides:

| Peptide | Uniq | -10lgP | Mass | ppm | m/z | z | RT | Scan | #Spec | Start | End | PTM |
| --- | --- | --- | --- | --- | --- | --- | --- | --- | --- | --- | --- | --- |
| R.SLQDDPLLVH.Y | Y | 20.27 | 1135.5873 | -1.5 | 568.8000 | 2 | 12.24 | 1715 | 1 | 777 | 786 |  |
| C.VIPVVDDKLTL.L | Y | 15.71 | 1210.7173 | -1.2 | 606.3652 | 2 | 13.28 | 1904 | 1 | 5240 | 5250 |  |
| total 2 peptides |  |  |  |  |  |  |  |  |  |  |  |  |
| --- | --- | --- | --- | --- | --- | --- | --- | --- | --- | --- | --- | --- |

tr|A0A6J2RYI7|A0A6J2RYI7\_COTGO

back to list

  

| Protein Coverage
| Supporting Peptides
|

Protein Coverage:

Supporting Peptides:

| Peptide | Uniq | -10lgP | Mass | ppm | m/z | z | RT | Scan | #Spec | Start | End | PTM |
| --- | --- | --- | --- | --- | --- | --- | --- | --- | --- | --- | --- | --- |
| R.SIQDDPLLVH.Y | Y | 20.27 | 1135.5873 | -1.5 | 568.8000 | 2 | 12.24 | 1715 | 1 | 835 | 844 |  |
| C.VIPVVDDKLTL.L | Y | 15.71 | 1210.7173 | -1.2 | 606.3652 | 2 | 13.28 | 1904 | 1 | 5321 | 5331 |  |
| total 2 peptides |  |  |  |  |  |  |  |  |  |  |  |  |
| --- | --- | --- | --- | --- | --- | --- | --- | --- | --- | --- | --- | --- |

tr|A0A6J2RWT1|A0A6J2RWT1\_COTGO

back to list

  

| Protein Coverage
| Supporting Peptides
|

Protein Coverage:

Supporting Peptides:

| Peptide | Uniq | -10lgP | Mass | ppm | m/z | z | RT | Scan | #Spec | Start | End | PTM |
| --- | --- | --- | --- | --- | --- | --- | --- | --- | --- | --- | --- | --- |
| R.SIQDDPLLVH.Y | Y | 20.27 | 1135.5873 | -1.5 | 568.8000 | 2 | 12.24 | 1715 | 1 | 835 | 844 |  |
| C.VIPVVDDKLTL.L | Y | 15.71 | 1210.7173 | -1.2 | 606.3652 | 2 | 13.28 | 1904 | 1 | 5321 | 5331 |  |
| total 2 peptides |  |  |  |  |  |  |  |  |  |  |  |  |
| --- | --- | --- | --- | --- | --- | --- | --- | --- | --- | --- | --- | --- |

tr|A0A6J2RY61|A0A6J2RY61\_COTGO

back to list

  

| Protein Coverage
| Supporting Peptides
|

Protein Coverage:

Supporting Peptides:

| Peptide | Uniq | -10lgP | Mass | ppm | m/z | z | RT | Scan | #Spec | Start | End | PTM |
| --- | --- | --- | --- | --- | --- | --- | --- | --- | --- | --- | --- | --- |
| R.SIQDDPLLVH.Y | Y | 20.27 | 1135.5873 | -1.5 | 568.8000 | 2 | 12.24 | 1715 | 1 | 835 | 844 |  |
| C.VIPVVDDKLTL.L | Y | 15.71 | 1210.7173 | -1.2 | 606.3652 | 2 | 13.28 | 1904 | 1 | 5321 | 5331 |  |
| total 2 peptides |  |  |  |  |  |  |  |  |  |  |  |  |
| --- | --- | --- | --- | --- | --- | --- | --- | --- | --- | --- | --- | --- |

tr|A0A6J2RYI1|A0A6J2RYI1\_COTGO

back to list

  

| Protein Coverage
| Supporting Peptides
|

Protein Coverage:

Supporting Peptides:

| Peptide | Uniq | -10lgP | Mass | ppm | m/z | z | RT | Scan | #Spec | Start | End | PTM |
| --- | --- | --- | --- | --- | --- | --- | --- | --- | --- | --- | --- | --- |
| R.SIQDDPLLVH.Y | Y | 20.27 | 1135.5873 | -1.5 | 568.8000 | 2 | 12.24 | 1715 | 1 | 835 | 844 |  |
| C.VIPVVDDKLTL.L | Y | 15.71 | 1210.7173 | -1.2 | 606.3652 | 2 | 13.28 | 1904 | 1 | 5321 | 5331 |  |
| total 2 peptides |  |  |  |  |  |  |  |  |  |  |  |  |
| --- | --- | --- | --- | --- | --- | --- | --- | --- | --- | --- | --- | --- |

tr|A0A6J2RVP6|A0A6J2RVP6\_COTGO

back to list

  

| Protein Coverage
| Supporting Peptides
|

Protein Coverage:

Supporting Peptides:

| Peptide | Uniq | -10lgP | Mass | ppm | m/z | z | RT | Scan | #Spec | Start | End | PTM |
| --- | --- | --- | --- | --- | --- | --- | --- | --- | --- | --- | --- | --- |
| R.SIQDDPLLVH.Y | Y | 20.27 | 1135.5873 | -1.5 | 568.8000 | 2 | 12.24 | 1715 | 1 | 835 | 844 |  |
| C.VIPVVDDKLTL.L | Y | 15.71 | 1210.7173 | -1.2 | 606.3652 | 2 | 13.28 | 1904 | 1 | 5321 | 5331 |  |
| total 2 peptides |  |  |  |  |  |  |  |  |  |  |  |  |
| --- | --- | --- | --- | --- | --- | --- | --- | --- | --- | --- | --- | --- |

tr|A0A6J2RY55|A0A6J2RY55\_COTGO

back to list

  

| Protein Coverage
| Supporting Peptides
|

Protein Coverage:

Supporting Peptides:

| Peptide | Uniq | -10lgP | Mass | ppm | m/z | z | RT | Scan | #Spec | Start | End | PTM |
| --- | --- | --- | --- | --- | --- | --- | --- | --- | --- | --- | --- | --- |
| R.SIQDDPLLVH.Y | Y | 20.27 | 1135.5873 | -1.5 | 568.8000 | 2 | 12.24 | 1715 | 1 | 835 | 844 |  |
| C.VIPVVDDKLTL.L | Y | 15.71 | 1210.7173 | -1.2 | 606.3652 | 2 | 13.28 | 1904 | 1 | 5321 | 5331 |  |
| total 2 peptides |  |  |  |  |  |  |  |  |  |  |  |  |
| --- | --- | --- | --- | --- | --- | --- | --- | --- | --- | --- | --- | --- |

tr|A0A6J2RVP0|A0A6J2RVP0\_COTGO

back to list

  

| Protein Coverage
| Supporting Peptides
|

Protein Coverage:

Supporting Peptides:

| Peptide | Uniq | -10lgP | Mass | ppm | m/z | z | RT | Scan | #Spec | Start | End | PTM |
| --- | --- | --- | --- | --- | --- | --- | --- | --- | --- | --- | --- | --- |
| R.SIQDDPLLVH.Y | Y | 20.27 | 1135.5873 | -1.5 | 568.8000 | 2 | 12.24 | 1715 | 1 | 819 | 828 |  |
| C.VIPVVDDKLTL.L | Y | 15.71 | 1210.7173 | -1.2 | 606.3652 | 2 | 13.28 | 1904 | 1 | 5305 | 5315 |  |
| total 2 peptides |  |  |  |  |  |  |  |  |  |  |  |  |
| --- | --- | --- | --- | --- | --- | --- | --- | --- | --- | --- | --- | --- |

tr|A0A6J2RWS0|A0A6J2RWS0\_COTGO

back to list

  

| Protein Coverage
| Supporting Peptides
|

Protein Coverage:

Supporting Peptides:

| Peptide | Uniq | -10lgP | Mass | ppm | m/z | z | RT | Scan | #Spec | Start | End | PTM |
| --- | --- | --- | --- | --- | --- | --- | --- | --- | --- | --- | --- | --- |
| R.SIQDDPLLVH.Y | Y | 20.27 | 1135.5873 | -1.5 | 568.8000 | 2 | 12.24 | 1715 | 1 | 835 | 844 |  |
| C.VIPVVDDKLTL.L | Y | 15.71 | 1210.7173 | -1.2 | 606.3652 | 2 | 13.28 | 1904 | 1 | 5321 | 5331 |  |
| total 2 peptides |  |  |  |  |  |  |  |  |  |  |  |  |
| --- | --- | --- | --- | --- | --- | --- | --- | --- | --- | --- | --- | --- |

tr|A0A6G0J8D3|A0A6G0J8D3\_LARCR

back to list

  

| Protein Coverage
| Supporting Peptides
|

Protein Coverage:

Supporting Peptides:

| Peptide | Uniq | -10lgP | Mass | ppm | m/z | z | RT | Scan | #Spec | Start | End | PTM |
| --- | --- | --- | --- | --- | --- | --- | --- | --- | --- | --- | --- | --- |
| R.SLQDDPLLVH.Y | Y | 20.27 | 1135.5873 | -1.5 | 568.8000 | 2 | 12.24 | 1715 | 1 | 830 | 839 |  |
| C.VIPVVDDKLTL.L | Y | 15.71 | 1210.7173 | -1.2 | 606.3652 | 2 | 13.28 | 1904 | 1 | 4101 | 4111 |  |
| total 2 peptides |  |  |  |  |  |  |  |  |  |  |  |  |
| --- | --- | --- | --- | --- | --- | --- | --- | --- | --- | --- | --- | --- |

tr|A0A5C6PNB9|A0A5C6PNB9\_9TELE

back to list

  

| Protein Coverage
| Supporting Peptides
|

Protein Coverage:

Supporting Peptides:

| Peptide | Uniq | -10lgP | Mass | ppm | m/z | z | RT | Scan | #Spec | Start | End | PTM |
| --- | --- | --- | --- | --- | --- | --- | --- | --- | --- | --- | --- | --- |
| R.SLQDDPLLVH.Y | Y | 20.27 | 1135.5873 | -1.5 | 568.8000 | 2 | 12.24 | 1715 | 1 | 788 | 797 |  |
| C.VIPVVDDKLTL.L | Y | 15.71 | 1210.7173 | -1.2 | 606.3652 | 2 | 13.28 | 1904 | 1 | 4995 | 5005 |  |
| total 2 peptides |  |  |  |  |  |  |  |  |  |  |  |  |
| --- | --- | --- | --- | --- | --- | --- | --- | --- | --- | --- | --- | --- |

tr|A0A669CKX5|A0A669CKX5\_ORENI

back to list

  

| Protein Coverage
| Supporting Peptides
|

Protein Coverage:

Supporting Peptides:

| Peptide | Uniq | -10lgP | Mass | ppm | m/z | z | RT | Scan | #Spec | Start | End | PTM |
| --- | --- | --- | --- | --- | --- | --- | --- | --- | --- | --- | --- | --- |
| R.SLQDDPLLVH.Y | Y | 20.27 | 1135.5873 | -1.5 | 568.8000 | 2 | 12.24 | 1715 | 1 | 733 | 742 |  |
| C.VIPVVDDKLTL.L | Y | 15.71 | 1210.7173 | -1.2 | 606.3652 | 2 | 13.28 | 1904 | 1 | 5207 | 5217 |  |
| total 2 peptides |  |  |  |  |  |  |  |  |  |  |  |  |
| --- | --- | --- | --- | --- | --- | --- | --- | --- | --- | --- | --- | --- |

tr|I3KK23|I3KK23\_ORENI

back to list

  

| Protein Coverage
| Supporting Peptides
|

Protein Coverage:

Supporting Peptides:

| Peptide | Uniq | -10lgP | Mass | ppm | m/z | z | RT | Scan | #Spec | Start | End | PTM |
| --- | --- | --- | --- | --- | --- | --- | --- | --- | --- | --- | --- | --- |
| R.SLQDDPLLVH.Y | Y | 20.27 | 1135.5873 | -1.5 | 568.8000 | 2 | 12.24 | 1715 | 1 | 777 | 786 |  |
| C.VIPVVDDKLTL.L | Y | 15.71 | 1210.7173 | -1.2 | 606.3652 | 2 | 13.28 | 1904 | 1 | 5251 | 5261 |  |
| total 2 peptides |  |  |  |  |  |  |  |  |  |  |  |  |
| --- | --- | --- | --- | --- | --- | --- | --- | --- | --- | --- | --- | --- |

tr|A0A3P8QJ88|A0A3P8QJ88\_ASTCA

back to list

  

| Protein Coverage
| Supporting Peptides
|

Protein Coverage:

Supporting Peptides:

| Peptide | Uniq | -10lgP | Mass | ppm | m/z | z | RT | Scan | #Spec | Start | End | PTM |
| --- | --- | --- | --- | --- | --- | --- | --- | --- | --- | --- | --- | --- |
| R.SLQDDPLLVH.Y | Y | 20.27 | 1135.5873 | -1.5 | 568.8000 | 2 | 12.24 | 1715 | 1 | 799 | 808 |  |
| C.VIPVVDDKLTL.L | Y | 15.71 | 1210.7173 | -1.2 | 606.3652 | 2 | 13.28 | 1904 | 1 | 5273 | 5283 |  |
| total 2 peptides |  |  |  |  |  |  |  |  |  |  |  |  |
| --- | --- | --- | --- | --- | --- | --- | --- | --- | --- | --- | --- | --- |

tr|A0A3P9CU79|A0A3P9CU79\_9CICH

back to list

  

| Protein Coverage
| Supporting Peptides
|

Protein Coverage:

Supporting Peptides:

| Peptide | Uniq | -10lgP | Mass | ppm | m/z | z | RT | Scan | #Spec | Start | End | PTM |
| --- | --- | --- | --- | --- | --- | --- | --- | --- | --- | --- | --- | --- |
| R.SLQDDPLLVH.Y | Y | 20.27 | 1135.5873 | -1.5 | 568.8000 | 2 | 12.24 | 1715 | 1 | 818 | 827 |  |
| C.VIPVVDDKLTL.L | Y | 15.71 | 1210.7173 | -1.2 | 606.3652 | 2 | 13.28 | 1904 | 1 | 5292 | 5302 |  |
| total 2 peptides |  |  |  |  |  |  |  |  |  |  |  |  |
| --- | --- | --- | --- | --- | --- | --- | --- | --- | --- | --- | --- | --- |

tr|A0A6J2RTX8|A0A6J2RTX8\_COTGO

back to list

  

| Protein Coverage
| Supporting Peptides
|

Protein Coverage:

Supporting Peptides:

| Peptide | Uniq | -10lgP | Mass | ppm | m/z | z | RT | Scan | #Spec | Start | End | PTM |
| --- | --- | --- | --- | --- | --- | --- | --- | --- | --- | --- | --- | --- |
| R.SIQDDPLLVH.Y | Y | 20.27 | 1135.5873 | -1.5 | 568.8000 | 2 | 12.24 | 1715 | 1 | 835 | 844 |  |
| C.VIPVVDDKLTL.L | Y | 15.71 | 1210.7173 | -1.2 | 606.3652 | 2 | 13.28 | 1904 | 1 | 5321 | 5331 |  |
| total 2 peptides |  |  |  |  |  |  |  |  |  |  |  |  |
| --- | --- | --- | --- | --- | --- | --- | --- | --- | --- | --- | --- | --- |

tr|A0A4U5U1Y5|A0A4U5U1Y5\_COLLU

back to list

  

| Protein Coverage
| Supporting Peptides
|

Protein Coverage:

Supporting Peptides:

| Peptide | Uniq | -10lgP | Mass | ppm | m/z | z | RT | Scan | #Spec | Start | End | PTM |
| --- | --- | --- | --- | --- | --- | --- | --- | --- | --- | --- | --- | --- |
| R.SLQDDPLLVH.Y | Y | 20.27 | 1135.5873 | -1.5 | 568.8000 | 2 | 12.24 | 1715 | 1 | 912 | 921 |  |
| C.VIPVVDDKLTL.L | Y | 15.71 | 1210.7173 | -1.2 | 606.3652 | 2 | 13.28 | 1904 | 1 | 5403 | 5413 |  |
| total 2 peptides |  |  |  |  |  |  |  |  |  |  |  |  |
| --- | --- | --- | --- | --- | --- | --- | --- | --- | --- | --- | --- | --- |

tr|A0A4W4F305|A0A4W4F305\_ELEEL

back to list

  

| Protein Coverage
| Supporting Peptides
|

Protein Coverage:

Supporting Peptides:

| Peptide | Uniq | -10lgP | Mass | ppm | m/z | z | RT | Scan | #Spec | Start | End | PTM |
| --- | --- | --- | --- | --- | --- | --- | --- | --- | --- | --- | --- | --- |
| Q.RGLPGPAGPPGE.S | Y | 28.12 | 1103.5723 | -1.5 | 552.7926 | 2 | 10.58 | 1409 | 1 | 3336 | 3347 |  |
| total 1 peptides |  |  |  |  |  |  |  |  |  |  |  |  |
| --- | --- | --- | --- | --- | --- | --- | --- | --- | --- | --- | --- | --- |

tr|W5KZP4|W5KZP4\_ASTMX

back to list

  

| Protein Coverage
| Supporting Peptides
|

Protein Coverage:

Supporting Peptides:

| Peptide | Uniq | -10lgP | Mass | ppm | m/z | z | RT | Scan | #Spec | Start | End | PTM |
| --- | --- | --- | --- | --- | --- | --- | --- | --- | --- | --- | --- | --- |
| F.RGLPGPAGPPGE.G | Y | 28.12 | 1103.5723 | -1.5 | 552.7926 | 2 | 10.58 | 1409 | 1 | 590 | 601 |  |
| total 1 peptides |  |  |  |  |  |  |  |  |  |  |  |  |
| --- | --- | --- | --- | --- | --- | --- | --- | --- | --- | --- | --- | --- |

tr|A0A6P3VUU5|A0A6P3VUU5\_CLUHA

back to list

  

| Protein Coverage
| Supporting Peptides
|

Protein Coverage:

Supporting Peptides:

| Peptide | Uniq | -10lgP | Mass | ppm | m/z | z | RT | Scan | #Spec | Start | End | PTM |
| --- | --- | --- | --- | --- | --- | --- | --- | --- | --- | --- | --- | --- |
| F.AGPPGSDGQPGAK.G | Y | 26.91 | 1137.5414 | -1.0 | 569.7774 | 2 | 9.07 | 1156 | 1 | 837 | 849 |  |
| total 1 peptides |  |  |  |  |  |  |  |  |  |  |  |  |
| --- | --- | --- | --- | --- | --- | --- | --- | --- | --- | --- | --- | --- |

tr|A0A6J2PWT2|A0A6J2PWT2\_COTGO

back to list

  

| Protein Coverage
| Supporting Peptides
|

Protein Coverage:

Supporting Peptides:

| Peptide | Uniq | -10lgP | Mass | ppm | m/z | z | RT | Scan | #Spec | Start | End | PTM |
| --- | --- | --- | --- | --- | --- | --- | --- | --- | --- | --- | --- | --- |
| S.APKLPDGEKV.D | Y | 26.90 | 1052.5865 | -1.2 | 527.2999 | 2 | 10.12 | 1331 | 1 | 24 | 33 |  |
| total 1 peptides |  |  |  |  |  |  |  |  |  |  |  |  |
| --- | --- | --- | --- | --- | --- | --- | --- | --- | --- | --- | --- | --- |

tr|A0A6J2PTP5|A0A6J2PTP5\_COTGO

back to list

  

| Protein Coverage
| Supporting Peptides
|

Protein Coverage:

Supporting Peptides:

| Peptide | Uniq | -10lgP | Mass | ppm | m/z | z | RT | Scan | #Spec | Start | End | PTM |
| --- | --- | --- | --- | --- | --- | --- | --- | --- | --- | --- | --- | --- |
| S.APKLPDGEKV.D | Y | 26.90 | 1052.5865 | -1.2 | 527.2999 | 2 | 10.12 | 1331 | 1 | 21 | 30 |  |
| total 1 peptides |  |  |  |  |  |  |  |  |  |  |  |  |
| --- | --- | --- | --- | --- | --- | --- | --- | --- | --- | --- | --- | --- |

tr|A0A6J2PW38|A0A6J2PW38\_COTGO

back to list

  

| Protein Coverage
| Supporting Peptides
|

Protein Coverage:

Supporting Peptides:

| Peptide | Uniq | -10lgP | Mass | ppm | m/z | z | RT | Scan | #Spec | Start | End | PTM |
| --- | --- | --- | --- | --- | --- | --- | --- | --- | --- | --- | --- | --- |
| S.APKLPDGEKV.D | Y | 26.90 | 1052.5865 | -1.2 | 527.2999 | 2 | 10.12 | 1331 | 1 | 78 | 87 |  |
| total 1 peptides |  |  |  |  |  |  |  |  |  |  |  |  |
| --- | --- | --- | --- | --- | --- | --- | --- | --- | --- | --- | --- | --- |

tr|A0A6J2PTY9|A0A6J2PTY9\_COTGO

back to list

  

| Protein Coverage
| Supporting Peptides
|

Protein Coverage:

Supporting Peptides:

| Peptide | Uniq | -10lgP | Mass | ppm | m/z | z | RT | Scan | #Spec | Start | End | PTM |
| --- | --- | --- | --- | --- | --- | --- | --- | --- | --- | --- | --- | --- |
| S.APKLPDGEKV.D | Y | 26.90 | 1052.5865 | -1.2 | 527.2999 | 2 | 10.12 | 1331 | 1 | 84 | 93 |  |
| total 1 peptides |  |  |  |  |  |  |  |  |  |  |  |  |
| --- | --- | --- | --- | --- | --- | --- | --- | --- | --- | --- | --- | --- |

tr|A0A6J2PVR7|A0A6J2PVR7\_COTGO

back to list

  

| Protein Coverage
| Supporting Peptides
|

Protein Coverage:

Supporting Peptides:

| Peptide | Uniq | -10lgP | Mass | ppm | m/z | z | RT | Scan | #Spec | Start | End | PTM |
| --- | --- | --- | --- | --- | --- | --- | --- | --- | --- | --- | --- | --- |
| S.APKLPDGEKV.D | Y | 26.90 | 1052.5865 | -1.2 | 527.2999 | 2 | 10.12 | 1331 | 1 | 84 | 93 |  |
| total 1 peptides |  |  |  |  |  |  |  |  |  |  |  |  |
| --- | --- | --- | --- | --- | --- | --- | --- | --- | --- | --- | --- | --- |

tr|A0A6J2PTN9|A0A6J2PTN9\_COTGO

back to list

  

| Protein Coverage
| Supporting Peptides
|

Protein Coverage:

Supporting Peptides:

| Peptide | Uniq | -10lgP | Mass | ppm | m/z | z | RT | Scan | #Spec | Start | End | PTM |
| --- | --- | --- | --- | --- | --- | --- | --- | --- | --- | --- | --- | --- |
| S.APKLPDGEKV.D | Y | 26.90 | 1052.5865 | -1.2 | 527.2999 | 2 | 10.12 | 1331 | 1 | 87 | 96 |  |
| total 1 peptides |  |  |  |  |  |  |  |  |  |  |  |  |
| --- | --- | --- | --- | --- | --- | --- | --- | --- | --- | --- | --- | --- |

tr|A0A5A9PG84|A0A5A9PG84\_9TELE

back to list

  

| Protein Coverage
| Supporting Peptides
|

Protein Coverage:

Supporting Peptides:

| Peptide | Uniq | -10lgP | Mass | ppm | m/z | z | RT | Scan | #Spec | Start | End | PTM |
| --- | --- | --- | --- | --- | --- | --- | --- | --- | --- | --- | --- | --- |
| I.APKLPDGEKV.D | Y | 26.90 | 1052.5865 | -1.2 | 527.2999 | 2 | 10.12 | 1331 | 1 | 127 | 136 |  |
| total 1 peptides |  |  |  |  |  |  |  |  |  |  |  |  |
| --- | --- | --- | --- | --- | --- | --- | --- | --- | --- | --- | --- | --- |

tr|A0A6F9CEX9|A0A6F9CEX9\_9TELE

back to list

  

| Protein Coverage
| Supporting Peptides
|

Protein Coverage:

Supporting Peptides:

| Peptide | Uniq | -10lgP | Mass | ppm | m/z | z | RT | Scan | #Spec | Start | End | PTM |
| --- | --- | --- | --- | --- | --- | --- | --- | --- | --- | --- | --- | --- |
| A.LDPIDAPGKPDVISIT.R | Y | 26.71 | 1649.8876 | -1.8 | 825.9496 | 2 | 12.83 | 1822 | 1 | 8748 | 8763 |  |
| total 1 peptides |  |  |  |  |  |  |  |  |  |  |  |  |
| --- | --- | --- | --- | --- | --- | --- | --- | --- | --- | --- | --- | --- |

tr|A0A3B3ICW0|A0A3B3ICW0\_ORYLA

back to list

  

| Protein Coverage
| Supporting Peptides
|

Protein Coverage:

Supporting Peptides:

| Peptide | Uniq | -10lgP | Mass | ppm | m/z | z | RT | Scan | #Spec | Start | End | PTM |
| --- | --- | --- | --- | --- | --- | --- | --- | --- | --- | --- | --- | --- |
| E.VILPVPAF.N | Y | 16.42 | 854.5266 | -0.2 | 428.2704 | 2 | 14.94 | 2196 | 1 | 143 | 150 |  |
| V.GDDLTVTNPK.R | N | 15.81 | 1058.5244 | -0.4 | 530.2693 | 2 | 10.15 | 1336 | 1 | 317 | 326 |  |
| total 2 peptides |  |  |  |  |  |  |  |  |  |  |  |  |
| --- | --- | --- | --- | --- | --- | --- | --- | --- | --- | --- | --- | --- |

tr|A0A3Q4GK85|A0A3Q4GK85\_NEOBR

back to list

  

| Protein Coverage
| Supporting Peptides
|

Protein Coverage:

Supporting Peptides:

| Peptide | Uniq | -10lgP | Mass | ppm | m/z | z | RT | Scan | #Spec | Start | End | PTM |
| --- | --- | --- | --- | --- | --- | --- | --- | --- | --- | --- | --- | --- |
| D.SELPVDDVFGQ.V | Y | 24.20 | 1204.5612 | 0.4 | 603.2881 | 2 | 13.63 | 1966 | 1 | 174 | 184 |  |
| total 1 peptides |  |  |  |  |  |  |  |  |  |  |  |  |
| --- | --- | --- | --- | --- | --- | --- | --- | --- | --- | --- | --- | --- |

tr|A0A3Q4GS43|A0A3Q4GS43\_NEOBR

back to list

  

| Protein Coverage
| Supporting Peptides
|

Protein Coverage:

Supporting Peptides:

| Peptide | Uniq | -10lgP | Mass | ppm | m/z | z | RT | Scan | #Spec | Start | End | PTM |
| --- | --- | --- | --- | --- | --- | --- | --- | --- | --- | --- | --- | --- |
| D.SELPVDDVFGQ.V | Y | 24.20 | 1204.5612 | 0.4 | 603.2881 | 2 | 13.63 | 1966 | 1 | 175 | 185 |  |
| total 1 peptides |  |  |  |  |  |  |  |  |  |  |  |  |
| --- | --- | --- | --- | --- | --- | --- | --- | --- | --- | --- | --- | --- |

tr|A0A3P8P8S3|A0A3P8P8S3\_ASTCA

back to list

  

| Protein Coverage
| Supporting Peptides
|

Protein Coverage:

Supporting Peptides:

| Peptide | Uniq | -10lgP | Mass | ppm | m/z | z | RT | Scan | #Spec | Start | End | PTM |
| --- | --- | --- | --- | --- | --- | --- | --- | --- | --- | --- | --- | --- |
| D.SELPVDDVFGQ.V | Y | 24.20 | 1204.5612 | 0.4 | 603.2881 | 2 | 13.63 | 1966 | 1 | 175 | 185 |  |
| total 1 peptides |  |  |  |  |  |  |  |  |  |  |  |  |
| --- | --- | --- | --- | --- | --- | --- | --- | --- | --- | --- | --- | --- |

tr|A0A3Q1HW42|A0A3Q1HW42\_9TELE

back to list

  

| Protein Coverage
| Supporting Peptides
|

Protein Coverage:

Supporting Peptides:

| Peptide | Uniq | -10lgP | Mass | ppm | m/z | z | RT | Scan | #Spec | Start | End | PTM |
| --- | --- | --- | --- | --- | --- | --- | --- | --- | --- | --- | --- | --- |
| D.SELPVDDVFGQ.V | Y | 24.20 | 1204.5612 | 0.4 | 603.2881 | 2 | 13.63 | 1966 | 1 | 175 | 185 |  |
| total 1 peptides |  |  |  |  |  |  |  |  |  |  |  |  |
| --- | --- | --- | --- | --- | --- | --- | --- | --- | --- | --- | --- | --- |

tr|A0A3P9CWL7|A0A3P9CWL7\_9CICH

back to list

  

| Protein Coverage
| Supporting Peptides
|

Protein Coverage:

Supporting Peptides:

| Peptide | Uniq | -10lgP | Mass | ppm | m/z | z | RT | Scan | #Spec | Start | End | PTM |
| --- | --- | --- | --- | --- | --- | --- | --- | --- | --- | --- | --- | --- |
| D.SELPVDDVFGQ.V | Y | 24.20 | 1204.5612 | 0.4 | 603.2881 | 2 | 13.63 | 1966 | 1 | 175 | 185 |  |
| total 1 peptides |  |  |  |  |  |  |  |  |  |  |  |  |
| --- | --- | --- | --- | --- | --- | --- | --- | --- | --- | --- | --- | --- |

tr|A0A3B5BIY5|A0A3B5BIY5\_9TELE

back to list

  

| Protein Coverage
| Supporting Peptides
|

Protein Coverage:

Supporting Peptides:

| Peptide | Uniq | -10lgP | Mass | ppm | m/z | z | RT | Scan | #Spec | Start | End | PTM |
| --- | --- | --- | --- | --- | --- | --- | --- | --- | --- | --- | --- | --- |
| D.SELPVDDVFGQ.V | Y | 24.20 | 1204.5612 | 0.4 | 603.2881 | 2 | 13.63 | 1966 | 1 | 175 | 185 |  |
| total 1 peptides |  |  |  |  |  |  |  |  |  |  |  |  |
| --- | --- | --- | --- | --- | --- | --- | --- | --- | --- | --- | --- | --- |

tr|A0A3Q2VY92|A0A3Q2VY92\_HAPBU

back to list

  

| Protein Coverage
| Supporting Peptides
|

Protein Coverage:

Supporting Peptides:

| Peptide | Uniq | -10lgP | Mass | ppm | m/z | z | RT | Scan | #Spec | Start | End | PTM |
| --- | --- | --- | --- | --- | --- | --- | --- | --- | --- | --- | --- | --- |
| D.SELPVDDVFGQ.V | Y | 24.20 | 1204.5612 | 0.4 | 603.2881 | 2 | 13.63 | 1966 | 1 | 175 | 185 |  |
| total 1 peptides |  |  |  |  |  |  |  |  |  |  |  |  |
| --- | --- | --- | --- | --- | --- | --- | --- | --- | --- | --- | --- | --- |

tr|A0A3B4FMK3|A0A3B4FMK3\_9CICH

back to list

  

| Protein Coverage
| Supporting Peptides
|

Protein Coverage:

Supporting Peptides:

| Peptide | Uniq | -10lgP | Mass | ppm | m/z | z | RT | Scan | #Spec | Start | End | PTM |
| --- | --- | --- | --- | --- | --- | --- | --- | --- | --- | --- | --- | --- |
| D.SELPVDDVFGQ.V | Y | 24.20 | 1204.5612 | 0.4 | 603.2881 | 2 | 13.63 | 1966 | 1 | 175 | 185 |  |
| total 1 peptides |  |  |  |  |  |  |  |  |  |  |  |  |
| --- | --- | --- | --- | --- | --- | --- | --- | --- | --- | --- | --- | --- |

tr|A0A3Q3CWR9|A0A3Q3CWR9\_HAPBU

back to list

  

| Protein Coverage
| Supporting Peptides
|

Protein Coverage:

Supporting Peptides:

| Peptide | Uniq | -10lgP | Mass | ppm | m/z | z | RT | Scan | #Spec | Start | End | PTM |
| --- | --- | --- | --- | --- | --- | --- | --- | --- | --- | --- | --- | --- |
| D.SELPVDDVFGQ.V | Y | 24.20 | 1204.5612 | 0.4 | 603.2881 | 2 | 13.63 | 1966 | 1 | 193 | 203 |  |
| total 1 peptides |  |  |  |  |  |  |  |  |  |  |  |  |
| --- | --- | --- | --- | --- | --- | --- | --- | --- | --- | --- | --- | --- |

tr|A0A3P8P8V3|A0A3P8P8V3\_ASTCA

back to list

  

| Protein Coverage
| Supporting Peptides
|

Protein Coverage:

Supporting Peptides:

| Peptide | Uniq | -10lgP | Mass | ppm | m/z | z | RT | Scan | #Spec | Start | End | PTM |
| --- | --- | --- | --- | --- | --- | --- | --- | --- | --- | --- | --- | --- |
| D.SELPVDDVFGQ.V | Y | 24.20 | 1204.5612 | 0.4 | 603.2881 | 2 | 13.63 | 1966 | 1 | 193 | 203 |  |
| total 1 peptides |  |  |  |  |  |  |  |  |  |  |  |  |
| --- | --- | --- | --- | --- | --- | --- | --- | --- | --- | --- | --- | --- |

tr|A0A3Q1FHQ1|A0A3Q1FHQ1\_9TELE

back to list

  

| Protein Coverage
| Supporting Peptides
|

Protein Coverage:

Supporting Peptides:

| Peptide | Uniq | -10lgP | Mass | ppm | m/z | z | RT | Scan | #Spec | Start | End | PTM |
| --- | --- | --- | --- | --- | --- | --- | --- | --- | --- | --- | --- | --- |
| E.LEHEE.R | N | 16.09 | 655.2813 | 3.2 | 328.6490 | 2 | 8.51 | 1054 | 1 | 446 | 450 |  |
| L.MIDGLGDI.M | Y | 15.23 | 832.4000 | -3.9 | 417.2057 | 2 | 14.21 | 2067 | 1 | 2023 | 2030 |  |
| total 2 peptides |  |  |  |  |  |  |  |  |  |  |  |  |
| --- | --- | --- | --- | --- | --- | --- | --- | --- | --- | --- | --- | --- |

tr|G3NYZ7|G3NYZ7\_GASAC

back to list

  

| Protein Coverage
| Supporting Peptides
|

Protein Coverage:

Supporting Peptides:

| Peptide | Uniq | -10lgP | Mass | ppm | m/z | z | RT | Scan | #Spec | Start | End | PTM |
| --- | --- | --- | --- | --- | --- | --- | --- | --- | --- | --- | --- | --- |
| E.LEHEE.K | N | 16.09 | 655.2813 | 3.2 | 328.6490 | 2 | 8.51 | 1054 | 1 | 440 | 444 |  |
| L.MIDGLGDI.M | Y | 15.23 | 832.4000 | -3.9 | 417.2057 | 2 | 14.21 | 2067 | 1 | 1963 | 1970 |  |
| total 2 peptides |  |  |  |  |  |  |  |  |  |  |  |  |
| --- | --- | --- | --- | --- | --- | --- | --- | --- | --- | --- | --- | --- |

tr|A0A674BRX0|A0A674BRX0\_SALTR

back to list

  

| Protein Coverage
| Supporting Peptides
|

Protein Coverage:

Supporting Peptides:

| Peptide | Uniq | -10lgP | Mass | ppm | m/z | z | RT | Scan | #Spec | Start | End | PTM |
| --- | --- | --- | --- | --- | --- | --- | --- | --- | --- | --- | --- | --- |
| E.LEHEE.K | N | 16.09 | 655.2813 | 3.2 | 328.6490 | 2 | 8.51 | 1054 | 1 | 446 | 450 |  |
| L.MIDGLGDI.M | Y | 15.23 | 832.4000 | -3.9 | 417.2057 | 2 | 14.21 | 2067 | 1 | 2017 | 2024 |  |
| total 2 peptides |  |  |  |  |  |  |  |  |  |  |  |  |
| --- | --- | --- | --- | --- | --- | --- | --- | --- | --- | --- | --- | --- |

tr|A0A0F8B8T7|A0A0F8B8T7\_LARCR

back to list

  

| Protein Coverage
| Supporting Peptides
|

Protein Coverage:

Supporting Peptides:

| Peptide | Uniq | -10lgP | Mass | ppm | m/z | z | RT | Scan | #Spec | Start | End | PTM |
| --- | --- | --- | --- | --- | --- | --- | --- | --- | --- | --- | --- | --- |
| I.KGESGLPGPPG.R | Y | 23.41 | 994.5083 | -2.0 | 498.2604 | 2 | 11.00 | 1484 | 1 | 338 | 348 |  |
| total 1 peptides |  |  |  |  |  |  |  |  |  |  |  |  |
| --- | --- | --- | --- | --- | --- | --- | --- | --- | --- | --- | --- | --- |

tr|A0A665TL93|A0A665TL93\_ECHNA

back to list

  

| Protein Coverage
| Supporting Peptides
|

Protein Coverage:

Supporting Peptides:

| Peptide | Uniq | -10lgP | Mass | ppm | m/z | z | RT | Scan | #Spec | Start | End | PTM |
| --- | --- | --- | --- | --- | --- | --- | --- | --- | --- | --- | --- | --- |
| A.EDTIFQPGPPGNPRVL.D | Y | 22.82 | 1735.8893 | -2.1 | 868.9501 | 2 | 12.93 | 1840 | 1 | 272 | 287 |  |
| total 1 peptides |  |  |  |  |  |  |  |  |  |  |  |  |
| --- | --- | --- | --- | --- | --- | --- | --- | --- | --- | --- | --- | --- |

tr|A0A7J5Y3X5|A0A7J5Y3X5\_DISMA

back to list

  

| Protein Coverage
| Supporting Peptides
|

Protein Coverage:

Supporting Peptides:

| Peptide | Uniq | -10lgP | Mass | ppm | m/z | z | RT | Scan | #Spec | Start | End | PTM |
| --- | --- | --- | --- | --- | --- | --- | --- | --- | --- | --- | --- | --- |
| A.EDTIFQPGPPGNPRVL.D | Y | 22.82 | 1735.8893 | -2.1 | 868.9501 | 2 | 12.93 | 1840 | 1 | 543 | 558 |  |
| total 1 peptides |  |  |  |  |  |  |  |  |  |  |  |  |
| --- | --- | --- | --- | --- | --- | --- | --- | --- | --- | --- | --- | --- |

tr|A0A3Q1G4M0|A0A3Q1G4M0\_9TELE

back to list

  

| Protein Coverage
| Supporting Peptides
|

Protein Coverage:

Supporting Peptides:

| Peptide | Uniq | -10lgP | Mass | ppm | m/z | z | RT | Scan | #Spec | Start | End | PTM |
| --- | --- | --- | --- | --- | --- | --- | --- | --- | --- | --- | --- | --- |
| V.AGDPGKGLPGLDGPQ.G | Y | 22.56 | 1377.6887 | 6.6 | 689.8562 | 2 | 10.47 | 1391 | 1 | 551 | 565 |  |
| total 1 peptides |  |  |  |  |  |  |  |  |  |  |  |  |
| --- | --- | --- | --- | --- | --- | --- | --- | --- | --- | --- | --- | --- |

tr|A0A834FMH6|A0A834FMH6\_ORYME

back to list

  

| Protein Coverage
| Supporting Peptides
|

Protein Coverage:

Supporting Peptides:

| Peptide | Uniq | -10lgP | Mass | ppm | m/z | z | RT | Scan | #Spec | Start | End | PTM |
| --- | --- | --- | --- | --- | --- | --- | --- | --- | --- | --- | --- | --- |
| Q.FGPAAKRQ.R | Y | 21.61 | 873.4821 | -4.9 | 437.7462 | 2 | 12.19 | 1706 | 1 | 250 | 257 |  |
| total 1 peptides |  |  |  |  |  |  |  |  |  |  |  |  |
| --- | --- | --- | --- | --- | --- | --- | --- | --- | --- | --- | --- | --- |

tr|A0A673HU84|A0A673HU84\_9TELE

back to list

  

| Protein Coverage
| Supporting Peptides
|

Protein Coverage:

Supporting Peptides:

| Peptide | Uniq | -10lgP | Mass | ppm | m/z | z | RT | Scan | #Spec | Start | End | PTM |
| --- | --- | --- | --- | --- | --- | --- | --- | --- | --- | --- | --- | --- |
| L.ALADLVLVLDS.L | Y | 21.18 | 1127.6438 | 22.2 | 564.8417 | 2 | 17.39 | 2610 | 1 | 90 | 100 |  |
| total 1 peptides |  |  |  |  |  |  |  |  |  |  |  |  |
| --- | --- | --- | --- | --- | --- | --- | --- | --- | --- | --- | --- | --- |

tr|A0A2Z4XFW9|A0A2Z4XFW9\_CTEID

back to list

  

| Protein Coverage
| Supporting Peptides
|

Protein Coverage:

Supporting Peptides:

| Peptide | Uniq | -10lgP | Mass | ppm | m/z | z | RT | Scan | #Spec | Start | End | PTM |
| --- | --- | --- | --- | --- | --- | --- | --- | --- | --- | --- | --- | --- |
| L.ALADLVLVLDS.L | Y | 21.18 | 1127.6438 | 22.2 | 564.8417 | 2 | 17.39 | 2610 | 1 | 5 | 15 |  |
| total 1 peptides |  |  |  |  |  |  |  |  |  |  |  |  |
| --- | --- | --- | --- | --- | --- | --- | --- | --- | --- | --- | --- | --- |

tr|A0A4W4E5X7|A0A4W4E5X7\_ELEEL

back to list

  

| Protein Coverage
| Supporting Peptides
|

Protein Coverage:

Supporting Peptides:

| Peptide | Uniq | -10lgP | Mass | ppm | m/z | z | RT | Scan | #Spec | Start | End | PTM |
| --- | --- | --- | --- | --- | --- | --- | --- | --- | --- | --- | --- | --- |
| L.ALADLVLVLDS.L | Y | 21.18 | 1127.6438 | 22.2 | 564.8417 | 2 | 17.39 | 2610 | 1 | 81 | 91 |  |
| total 1 peptides |  |  |  |  |  |  |  |  |  |  |  |  |
| --- | --- | --- | --- | --- | --- | --- | --- | --- | --- | --- | --- | --- |

tr|A0A672KLI0|A0A672KLI0\_SINGR

back to list

  

| Protein Coverage
| Supporting Peptides
|

Protein Coverage:

Supporting Peptides:

| Peptide | Uniq | -10lgP | Mass | ppm | m/z | z | RT | Scan | #Spec | Start | End | PTM |
| --- | --- | --- | --- | --- | --- | --- | --- | --- | --- | --- | --- | --- |
| L.ALADLVLVLDS.L | Y | 21.18 | 1127.6438 | 22.2 | 564.8417 | 2 | 17.39 | 2610 | 1 | 79 | 89 |  |
| total 1 peptides |  |  |  |  |  |  |  |  |  |  |  |  |
| --- | --- | --- | --- | --- | --- | --- | --- | --- | --- | --- | --- | --- |

tr|A0A671RDB8|A0A671RDB8\_9TELE

back to list

  

| Protein Coverage
| Supporting Peptides
|

Protein Coverage:

Supporting Peptides:

| Peptide | Uniq | -10lgP | Mass | ppm | m/z | z | RT | Scan | #Spec | Start | End | PTM |
| --- | --- | --- | --- | --- | --- | --- | --- | --- | --- | --- | --- | --- |
| L.ALADLVLVLDS.L | Y | 21.18 | 1127.6438 | 22.2 | 564.8417 | 2 | 17.39 | 2610 | 1 | 84 | 94 |  |
| total 1 peptides |  |  |  |  |  |  |  |  |  |  |  |  |
| --- | --- | --- | --- | --- | --- | --- | --- | --- | --- | --- | --- | --- |

tr|W5LSJ6|W5LSJ6\_ASTMX

back to list

  

| Protein Coverage
| Supporting Peptides
|

Protein Coverage:

Supporting Peptides:

| Peptide | Uniq | -10lgP | Mass | ppm | m/z | z | RT | Scan | #Spec | Start | End | PTM |
| --- | --- | --- | --- | --- | --- | --- | --- | --- | --- | --- | --- | --- |
| L.ALADLVLVLDS.L | Y | 21.18 | 1127.6438 | 22.2 | 564.8417 | 2 | 17.39 | 2610 | 1 | 76 | 86 |  |
| total 1 peptides |  |  |  |  |  |  |  |  |  |  |  |  |
| --- | --- | --- | --- | --- | --- | --- | --- | --- | --- | --- | --- | --- |

tr|A0A3B4C497|A0A3B4C497\_PYGNA

back to list

  

| Protein Coverage
| Supporting Peptides
|

Protein Coverage:

Supporting Peptides:

| Peptide | Uniq | -10lgP | Mass | ppm | m/z | z | RT | Scan | #Spec | Start | End | PTM |
| --- | --- | --- | --- | --- | --- | --- | --- | --- | --- | --- | --- | --- |
| L.ALADLVLVLDS.L | Y | 21.18 | 1127.6438 | 22.2 | 564.8417 | 2 | 17.39 | 2610 | 1 | 78 | 88 |  |
| total 1 peptides |  |  |  |  |  |  |  |  |  |  |  |  |
| --- | --- | --- | --- | --- | --- | --- | --- | --- | --- | --- | --- | --- |

tr|A0A6P3WBU3|A0A6P3WBU3\_CLUHA

back to list

  

| Protein Coverage
| Supporting Peptides
|

Protein Coverage:

Supporting Peptides:

| Peptide | Uniq | -10lgP | Mass | ppm | m/z | z | RT | Scan | #Spec | Start | End | PTM |
| --- | --- | --- | --- | --- | --- | --- | --- | --- | --- | --- | --- | --- |
| L.ALADLVLVLDS.L | Y | 21.18 | 1127.6438 | 22.2 | 564.8417 | 2 | 17.39 | 2610 | 1 | 80 | 90 |  |
| total 1 peptides |  |  |  |  |  |  |  |  |  |  |  |  |
| --- | --- | --- | --- | --- | --- | --- | --- | --- | --- | --- | --- | --- |

tr|A0A6P6J7W1|A0A6P6J7W1\_CARAU

back to list

  

| Protein Coverage
| Supporting Peptides
|

Protein Coverage:

Supporting Peptides:

| Peptide | Uniq | -10lgP | Mass | ppm | m/z | z | RT | Scan | #Spec | Start | End | PTM |
| --- | --- | --- | --- | --- | --- | --- | --- | --- | --- | --- | --- | --- |
| L.ALADLVLVLDS.L | Y | 21.18 | 1127.6438 | 22.2 | 564.8417 | 2 | 17.39 | 2610 | 1 | 87 | 97 |  |
| total 1 peptides |  |  |  |  |  |  |  |  |  |  |  |  |
| --- | --- | --- | --- | --- | --- | --- | --- | --- | --- | --- | --- | --- |

tr|A0A5A9N4U0|A0A5A9N4U0\_9TELE

back to list

  

| Protein Coverage
| Supporting Peptides
|

Protein Coverage:

Supporting Peptides:

| Peptide | Uniq | -10lgP | Mass | ppm | m/z | z | RT | Scan | #Spec | Start | End | PTM |
| --- | --- | --- | --- | --- | --- | --- | --- | --- | --- | --- | --- | --- |
| L.ALADLVLVLDS.L | Y | 21.18 | 1127.6438 | 22.2 | 564.8417 | 2 | 17.39 | 2610 | 1 | 85 | 95 |  |
| total 1 peptides |  |  |  |  |  |  |  |  |  |  |  |  |
| --- | --- | --- | --- | --- | --- | --- | --- | --- | --- | --- | --- | --- |

tr|A0A3N0XI69|A0A3N0XI69\_ANAGA

back to list

  

| Protein Coverage
| Supporting Peptides
|

Protein Coverage:

Supporting Peptides:

| Peptide | Uniq | -10lgP | Mass | ppm | m/z | z | RT | Scan | #Spec | Start | End | PTM |
| --- | --- | --- | --- | --- | --- | --- | --- | --- | --- | --- | --- | --- |
| L.ALADLVLVLDS.L | Y | 21.18 | 1127.6438 | 22.2 | 564.8417 | 2 | 17.39 | 2610 | 1 | 87 | 97 |  |
| total 1 peptides |  |  |  |  |  |  |  |  |  |  |  |  |
| --- | --- | --- | --- | --- | --- | --- | --- | --- | --- | --- | --- | --- |

tr|A0A6J2UVU6|A0A6J2UVU6\_CHACN

back to list

  

| Protein Coverage
| Supporting Peptides
|

Protein Coverage:

Supporting Peptides:

| Peptide | Uniq | -10lgP | Mass | ppm | m/z | z | RT | Scan | #Spec | Start | End | PTM |
| --- | --- | --- | --- | --- | --- | --- | --- | --- | --- | --- | --- | --- |
| L.ALADLVLVLDS.L | Y | 21.18 | 1127.6438 | 22.2 | 564.8417 | 2 | 17.39 | 2610 | 1 | 80 | 90 |  |
| total 1 peptides |  |  |  |  |  |  |  |  |  |  |  |  |
| --- | --- | --- | --- | --- | --- | --- | --- | --- | --- | --- | --- | --- |

tr|A0A6P6JAH3|A0A6P6JAH3\_CARAU

back to list

  

| Protein Coverage
| Supporting Peptides
|

Protein Coverage:

Supporting Peptides:

| Peptide | Uniq | -10lgP | Mass | ppm | m/z | z | RT | Scan | #Spec | Start | End | PTM |
| --- | --- | --- | --- | --- | --- | --- | --- | --- | --- | --- | --- | --- |
| L.ALADLVLVLDS.L | Y | 21.18 | 1127.6438 | 22.2 | 564.8417 | 2 | 17.39 | 2610 | 1 | 122 | 132 |  |
| total 1 peptides |  |  |  |  |  |  |  |  |  |  |  |  |
| --- | --- | --- | --- | --- | --- | --- | --- | --- | --- | --- | --- | --- |

tr|A0A6P6JAN8|A0A6P6JAN8\_CARAU

back to list

  

| Protein Coverage
| Supporting Peptides
|

Protein Coverage:

Supporting Peptides:

| Peptide | Uniq | -10lgP | Mass | ppm | m/z | z | RT | Scan | #Spec | Start | End | PTM |
| --- | --- | --- | --- | --- | --- | --- | --- | --- | --- | --- | --- | --- |
| L.ALADLVLVLDS.L | Y | 21.18 | 1127.6438 | 22.2 | 564.8417 | 2 | 17.39 | 2610 | 1 | 125 | 135 |  |
| total 1 peptides |  |  |  |  |  |  |  |  |  |  |  |  |
| --- | --- | --- | --- | --- | --- | --- | --- | --- | --- | --- | --- | --- |

tr|A0A498LK34|A0A498LK34\_LABRO

back to list

  

| Protein Coverage
| Supporting Peptides
|

Protein Coverage:

Supporting Peptides:

| Peptide | Uniq | -10lgP | Mass | ppm | m/z | z | RT | Scan | #Spec | Start | End | PTM |
| --- | --- | --- | --- | --- | --- | --- | --- | --- | --- | --- | --- | --- |
| L.ALADLVLVLDS.L | Y | 21.18 | 1127.6438 | 22.2 | 564.8417 | 2 | 17.39 | 2610 | 1 | 80 | 90 |  |
| total 1 peptides |  |  |  |  |  |  |  |  |  |  |  |  |
| --- | --- | --- | --- | --- | --- | --- | --- | --- | --- | --- | --- | --- |

tr|A0A4W6ESI4|A0A4W6ESI4\_LATCA

back to list

  

| Protein Coverage
| Supporting Peptides
|

Protein Coverage:

Supporting Peptides:

| Peptide | Uniq | -10lgP | Mass | ppm | m/z | z | RT | Scan | #Spec | Start | End | PTM |
| --- | --- | --- | --- | --- | --- | --- | --- | --- | --- | --- | --- | --- |
| E.SGPGGPAGPVGA.A | Y | 20.60 | 922.4508 | -1.2 | 462.2321 | 2 | 10.46 | 1389 | 1 | 840 | 851 |  |
| total 1 peptides |  |  |  |  |  |  |  |  |  |  |  |  |
| --- | --- | --- | --- | --- | --- | --- | --- | --- | --- | --- | --- | --- |

tr|A0A7J6A7M3|A0A7J6A7M3\_AMEME

back to list

  

| Protein Coverage
| Supporting Peptides
|

Protein Coverage:

Supporting Peptides:

| Peptide | Uniq | -10lgP | Mass | ppm | m/z | z | RT | Scan | #Spec | Start | End | PTM |
| --- | --- | --- | --- | --- | --- | --- | --- | --- | --- | --- | --- | --- |
| V.DEEVCLRLL.E | Y | 20.39 | 1088.5536 | 29.5 | 545.3001 | 2 | 12.18 | 1703 | 1 | 590 | 598 |  |
| total 1 peptides |  |  |  |  |  |  |  |  |  |  |  |  |
| --- | --- | --- | --- | --- | --- | --- | --- | --- | --- | --- | --- | --- |

tr|A0A6G1P920|A0A6G1P920\_9TELE

back to list

  

| Protein Coverage
| Supporting Peptides
|

Protein Coverage:

Supporting Peptides:

| Peptide | Uniq | -10lgP | Mass | ppm | m/z | z | RT | Scan | #Spec | Start | End | PTM |
| --- | --- | --- | --- | --- | --- | --- | --- | --- | --- | --- | --- | --- |
| D.TEAPLKPN.V | Y | 20.06 | 868.4654 | -0.2 | 435.2399 | 2 | 22.56 | 3493 | 2 | 186 | 193 |  |
| total 1 peptides |  |  |  |  |  |  |  |  |  |  |  |  |
| --- | --- | --- | --- | --- | --- | --- | --- | --- | --- | --- | --- | --- |

---

Prepared with PEAKS ™ (bioinfor.com)
