# Supplementary material for: Proteomic and Functional Characterization of Antimicrobial Peptides Derived from Fisheries Bycatch via Enzymatic Hydrolysis
Source: Mar Drugs. 2026 Jan 10;24(1):36. doi: 10.3390/md24010036 (PMC12843163; doi:10.3390/md24010036)
Supplement: Supplementary file 1 [file marinedrugs-24-00036-s001.zip › SM41/MP/protein.html]

proteins


Summary

  

# 1. Notes

# 2. Result Statistics

**Figure 1.**
False discovery rate (FDR) curve. X axis is the number of peptide-spectrum matches (PSM) being kept. Y axis is the corresponding FDR.

  


  

**Figure 2.**
PSM score distribution.  **(a)**
Distribution of PEAKS peptide score;  **(b)**
Scatterplot of PEAKS peptide score versus precursor mass error.

**(a)**

**(b)**

**Figure 3.**
De novo result validation. Distribution of residue local confidence:  **(a)**
Residues in de novo sequences validated by confident database peptide assignment;  **(b)**
Residues in "de novo only" sequences.

**(a)**

**(b)**

**Table 1.**
Statistics of data.

|  |  |
| --- | --- |
| # of MS Scans | 1013 |
| # of MS/MS Scans | 2952 |


**Table 2.**
Result filtration parameters.

|  |  |
| --- | --- |
| Peptide -10lgP | ≥15 |
| Protein -10lgP | ≥20 |
| Proteins unique peptides | ≥0 |
| De novo ALC Score | ≥50% |


**Table 3.**
Statistics of filtered result.

|  |  |
| --- | --- |
| Peptide-Spectrum Matches | 159 |
| Peptide Sequences | 144 |
| Protein Groups | 64 |
| Proteins | 327 |
| Proteins (#Unique Peptides) | 141 (>2); 46 (=2); 42 (=1); |
| FDR (Peptide-Spectrum Matches) | 10.1% |
| FDR (Peptide Sequences) | 11.1% |
| FDR (Protein) | 0.0% |
| De Novo Only Spectra | 1323 |

**Table 4.**
PTM profile.

| Name | ∆Mass | #PSM | Position |
| --- | --- | --- | --- |

# 3. Experiment Control

**Figure 4.**
Precursor mass error of peptide-spectrum matches (PSM) in filtered result.  **(a)**
Distribution of precursor mass error in ppm;  **(b)**
Scatterplot of precursor m/z versus precursor mass error in ppm.

**(a)**

**(b)**

**Table 5.**
Number of identified peptides in each sample by the number of missed cleavages

|  |  |  |  |  |  |
| --- | --- | --- | --- | --- | --- |
| Missed Cleavages | 0 | 1 | 2 | 3 | 4+ |
| Orbi2222 | 0 | 0 | 0 | 0 | 144 |

# 4. Other Information

**Table 6.**
Search parameters.

Search Engine Name: PEAKS 7.0  
Parent Mass Error Tolerance: 0.03 Da  
Fragment Mass Error Tolerance: 0.1 Da  
Precursor Mass Search Type: monoisotopic  
Enzyme: None  
Max Missed Cleavages: 100  
Non-specific Cleavage: both  
Max variable PTM per peptide: 3  
Database: Teleosteo  
Taxon: All  
Searched Entry: 4529691  
FDR Estimation: Enabled  
Merge Options: 0.1 min. 0.03 Da  
Precursor Options: corrected  
Charge Options: no correction  
Filter Options: no filter  
Process: true

**Table 7.**
Instrument parameters.

Fractions: OB\_PIMENTA\_MP\_20220211\_05.raw  
Ion Source: ESI(nano-spray)  
Fragmentation Mode: CID/ECD  
MS Scan Mode: FT-ICR/Orbitrap  
MS/MS Scan Mode: Linear Ion Trap

  

Protein List

  

| Protein Group | Protein ID | Accession | -10lgP | Coverage (%) | #Peptides | #Unique | PTM | Avg. Mass | Description |
| --- | --- | --- | --- | --- | --- | --- | --- | --- | --- |
| 1 | 66837 | tr\|A0A3B4T4B1\|A0A3B4T4B1\_SERDU | 158.76 | 24 | 23 | 20 | N | 41596 | Actin, alpha skeletal muscle A OS=Seriola dumerili OX=41447 PE=3 SV=1 |
| 1 | 66838 | tr\|A0A3Q3BBJ2\|A0A3Q3BBJ2\_KRYMA | 158.76 | 23 | 23 | 20 | N | 41945 | Actin, alpha skeletal muscle A OS=Kryptolebias marmoratus OX=37003 GN=ACTA1 PE=3 SV=1 |
| 1 | 66839 | tr\|Q76MZ4\|Q76MZ4\_9TELE | 158.76 | 23 | 23 | 20 | N | 41959 | Skeletal alpha-actin type-1 OS=Coryphaenoides cinereus OX=83391 PE=2 SV=1 |
| 1 | 12040 | tr\|A0A3S2MUF9\|A0A3S2MUF9\_ORYJA | 158.76 | 23 | 23 | 20 | N | 41945 | Uncharacterized protein OS=Oryzias javanicus OX=123683 GN=OJAV\_G00024300 PE=3 SV=1 |
| 1 | 66840 | tr\|A0A3Q0RI14\|A0A3Q0RI14\_AMPCI | 158.76 | 23 | 23 | 20 | N | 41945 | Actin alpha 1, skeletal muscle OS=Amphilophus citrinellus OX=61819 GN=ACTA1 PE=3 SV=1 |
| 1 | 66841 | tr\|M4AKR3\|M4AKR3\_XIPMA | 158.76 | 23 | 23 | 20 | N | 41945 | Uncharacterized protein OS=Xiphophorus maculatus OX=8083 GN=ACTA1 PE=3 SV=2 |
| 1 | 66842 | tr\|C0LLE8\|C0LLE8\_DICLA | 158.76 | 23 | 23 | 20 | N | 41929 | Alpha actin OS=Dicentrarchus labrax OX=13489 PE=2 SV=1 |
| 1 | 66843 | tr\|A0A3Q3N432\|A0A3Q3N432\_9TELE | 158.76 | 23 | 23 | 20 | N | 41945 | Actin alpha 1, skeletal muscle b OS=Mastacembelus armatus OX=205130 GN=ACTA1 PE=3 SV=1 |
| 1 | 12049 | tr\|Q76LU1\|Q76LU1\_GADCH | 158.76 | 23 | 23 | 20 | N | 41959 | Alpha skeletal actin-1 OS=Gadus chalcogrammus OX=1042646 PE=2 SV=1 |
| 1 | 12038 | tr\|F5BZL8\|F5BZL8\_EPIBR | 158.76 | 23 | 23 | 20 | N | 41945 | Alpha actin (Fragment) OS=Epinephelus bruneus OX=323802 PE=2 SV=1 |
| 1 | 66844 | tr\|A0A3Q4BGG8\|A0A3Q4BGG8\_MOLML | 158.76 | 23 | 23 | 20 | N | 41945 | Actin alpha 1, skeletal muscle OS=Mola mola OX=94237 PE=3 SV=1 |
| 1 | 66845 | tr\|A0A3Q1DE47\|A0A3Q1DE47\_AMPOC | 158.76 | 23 | 23 | 20 | N | 41945 | Actin, alpha skeletal muscle A OS=Amphiprion ocellaris OX=80972 GN=ACTA1 PE=3 SV=1 |
| 1 | 66846 | tr\|A0A7J6C441\|A0A7J6C441\_9TELE | 158.76 | 23 | 23 | 20 | N | 41959 | Uncharacterized protein OS=Onychostoma macrolepis OX=369639 GN=G5714\_016621 PE=3 SV=1 |
| 1 | 66847 | tr\|A0A6P7M219\|A0A6P7M219\_BETSP | 158.76 | 23 | 23 | 20 | N | 41959 | actin, alpha skeletal muscle OS=Betta splendens OX=158456 GN=acta1 PE=3 SV=1 |
| 1 | 66848 | tr\|A0A3Q2P9J5\|A0A3Q2P9J5\_FUNHE | 158.76 | 23 | 23 | 20 | N | 41945 | Actin, alpha skeletal muscle A OS=Fundulus heteroclitus OX=8078 GN=ACTA1 PE=3 SV=1 |
| 1 | 66849 | tr\|D4P4P1\|D4P4P1\_COBCH | 158.76 | 23 | 23 | 20 | N | 41920 | Skeletal alpha-actin 1.2 OS=Cobitis choii OX=457511 GN=acta1.2 PE=2 SV=1 |
| 1 | 66850 | tr\|A3F5V3\|A3F5V3\_ORENI | 158.76 | 25 | 23 | 20 | N | 39189 | Alpha actin (Fragment) OS=Oreochromis niloticus OX=8128 PE=2 SV=1 |
| 3 | 66792 | tr\|A0A3P8QM38\|A0A3P8QM38\_ASTCA | 116.80 | 8 | 16 | 0 | N | 221264 | Uncharacterized protein OS=Astatotilapia calliptera OX=8154 PE=3 SV=1 |
| 2 | 66795 | tr\|A0A6G0HED4\|A0A6G0HED4\_LARCR | 115.75 | 9 | 17 | 0 | N | 221587 | Myosin heavy chain, fast skeletal muscle OS=Larimichthys crocea OX=215358 GN=D5F01\_LYC24499 PE=3 SV=1 |
| 2 | 66797 | tr\|A0A665X5K8\|A0A665X5K8\_ECHNA | 115.75 | 9 | 17 | 0 | N | 223744 | Myosin heavy chain, fast skeletal muscle-like OS=Echeneis naucrates OX=173247 GN=LOC115047598 PE=3 SV=1 |
| 10 | 66791 | tr\|A0A6J2PN08\|A0A6J2PN08\_COTGO | 115.41 | 7 | 12 | 0 | N | 223516 | myosin heavy chain, fast skeletal muscle-like isoform X1 OS=Cottoperca gobio OX=56716 GN=LOC115007825 PE=3 SV=1 |
| 8 | 66793 | tr\|A0A6P7NC66\|A0A6P7NC66\_BETSP | 114.09 | 8 | 15 | 0 | N | 221411 | myosin heavy chain, fast skeletal muscle-like OS=Betta splendens OX=158456 GN=LOC114860972 PE=3 SV=1 |
| 5 | 66801 | tr\|Q90YF6\|Q90YF6\_PARFO | 113.36 | 8 | 16 | 0 | N | 221374 | Myosin heavy chain OS=Paracirrhites forsteri OX=100206 GN=MyoHC-T54 PE=2 SV=1 |
| 6 | 66798 | tr\|A0A665WXF0\|A0A665WXF0\_ECHNA | 113.26 | 7 | 15 | 0 | N | 221777 | Myosin heavy chain, fast skeletal muscle-like OS=Echeneis naucrates OX=173247 GN=LOC115047599 PE=3 SV=1 |
| 4 | 66903 | tr\|A0A7N8X0U0\|A0A7N8X0U0\_9TELE | 112.54 | 9 | 17 | 0 | N | 220309 | Myosin heavy chain, fast skeletal muscle-like OS=Mastacembelus armatus OX=205130 PE=3 SV=1 |
| 4 | 66904 | tr\|A0A7N8WLS2\|A0A7N8WLS2\_9TELE | 112.54 | 8 | 17 | 0 | N | 221458 | Myosin heavy chain, fast skeletal muscle-like OS=Mastacembelus armatus OX=205130 PE=3 SV=1 |
| 27 | 66962 | tr\|A0A6G0IP12\|A0A6G0IP12\_LARCR | 110.62 | 31 | 6 | 6 | N | 18230 | Troponin C, skeletal muscle OS=Larimichthys crocea OX=215358 GN=D5F01\_LYC08192 PE=4 SV=1 |
| 27 | 66963 | tr\|A0A1A8Q0J1\|A0A1A8Q0J1\_9TELE | 110.62 | 31 | 6 | 6 | N | 18215 | Troponin C type 2 (Fast) OS=Nothobranchius pienaari OX=704102 GN=TNNC2 PE=4 SV=1 |
| 7 | 66912 | tr\|A0A3P8SK70\|A0A3P8SK70\_AMPPE | 110.05 | 8 | 16 | 0 | N | 221849 | Uncharacterized protein OS=Amphiprion percula OX=161767 PE=3 SV=1 |
| 7 | 66913 | tr\|A0A3P8SLP0\|A0A3P8SLP0\_AMPPE | 110.05 | 8 | 16 | 0 | N | 221886 | Uncharacterized protein OS=Amphiprion percula OX=161767 PE=3 SV=1 |
| 9 | 66910 | tr\|A0A3Q3WZY2\|A0A3Q3WZY2\_MOLML | 108.37 | 8 | 15 | 0 | N | 205901 | Uncharacterized protein OS=Mola mola OX=94237 PE=3 SV=1 |
| 12 | 66820 | tr\|A0A6P6KAZ0\|A0A6P6KAZ0\_CARAU | 106.22 | 6 | 11 | 0 | N | 222556 | myosin heavy chain, fast skeletal muscle-like OS=Carassius auratus OX=7957 GN=LOC113050383 PE=3 SV=1 |
| 15 | 66863 | tr\|A0A3Q3EJE1\|A0A3Q3EJE1\_9LABR | 104.70 | 5 | 11 | 0 | N | 220889 | Myosin heavy chain, fast skeletal muscle-like OS=Labrus bergylta OX=56723 PE=3 SV=1 |
| 15 | 66864 | tr\|A0A6P8GH29\|A0A6P8GH29\_CLUHA | 104.70 | 5 | 11 | 0 | N | 220733 | myosin heavy chain, fast skeletal muscle-like OS=Clupea harengus OX=7950 GN=LOC105894483 PE=3 SV=1 |
| 11 | 66961 | tr\|A0A7N8XS20\|A0A7N8XS20\_9TELE | 103.67 | 8 | 16 | 0 | N | 222388 | Myosin heavy chain, fast skeletal muscle OS=Mastacembelus armatus OX=205130 PE=3 SV=1 |
| 13 | 66862 | tr\|A0A6P8FM41\|A0A6P8FM41\_CLUHA | 102.05 | 4 | 9 | 0 | N | 222593 | myosin heavy chain, fast skeletal muscle OS=Clupea harengus OX=7950 GN=LOC105889197 PE=3 SV=1 |
| 14 | 66867 | tr\|A0A6P8G2F8\|A0A6P8G2F8\_CLUHA | 101.68 | 4 | 9 | 0 | N | 212617 | myosin heavy chain, fast skeletal muscle-like OS=Clupea harengus OX=7950 GN=LOC105902796 PE=3 SV=1 |
| 14 | 66868 | tr\|A0A6P8G1S8\|A0A6P8G1S8\_CLUHA | 101.68 | 4 | 9 | 0 | N | 212837 | myosin heavy chain, fast skeletal muscle-like OS=Clupea harengus OX=7950 GN=LOC105902797 PE=3 SV=1 |
| 20 | 67069 | tr\|A0A2U9CMP6\|A0A2U9CMP6\_SCOMX | 98.55 | 17 | 5 | 3 | N | 11876 | Parvalbumin OS=Scophthalmus maximus OX=52904 GN=SMAX5B\_020129 PE=3 SV=1 |
| 20 | 67070 | tr\|A0A6A4RT91\|A0A6A4RT91\_SCOMX | 98.55 | 1 | 5 | 3 | N | 177174 | Parvalbumin OS=Scophthalmus maximus OX=52904 GN=F2P81\_022114 PE=3 SV=1 |
| 20 | 67087 | tr\|A0A3B3XMC4\|A0A3B3XMC4\_9TELE | 98.55 | 17 | 5 | 3 | N | 11357 | Parvalbumin OS=Poecilia mexicana OX=48701 PE=3 SV=1 |
| 20 | 67088 | tr\|A0A3B5LQ20\|A0A3B5LQ20\_9TELE | 98.55 | 17 | 5 | 3 | N | 11419 | Parvalbumin OS=Xiphophorus couchianus OX=32473 PE=3 SV=1 |
| 20 | 67089 | tr\|A0A3B3Z292\|A0A3B3Z292\_9TELE | 98.55 | 17 | 5 | 3 | N | 11593 | Parvalbumin OS=Poecilia mexicana OX=48701 PE=3 SV=1 |
| 20 | 67090 | tr\|A0A671XKN2\|A0A671XKN2\_SPAAU | 98.55 | 17 | 5 | 3 | N | 11732 | Parvalbumin OS=Sparus aurata OX=8175 GN=LOC115574776 PE=3 SV=1 |
| 20 | 67091 | tr\|A0A672Z6Y0\|A0A672Z6Y0\_9TELE | 98.55 | 17 | 5 | 3 | N | 11480 | Parvalbumin OS=Sphaeramia orbicularis OX=375764 GN=LOC115424708 PE=3 SV=1 |
| 20 | 67092 | tr\|D0VB96\|D0VB96\_SPAAU | 98.55 | 17 | 5 | 3 | N | 11500 | Parvalbumin OS=Sparus aurata OX=8175 GN=LOC115574776 PE=2 SV=1 |
| 20 | 67093 | tr\|A0A087Y091\|A0A087Y091\_POEFO | 98.55 | 17 | 5 | 3 | N | 11775 | Parvalbumin OS=Poecilia formosa OX=48698 PE=3 SV=1 |
| 20 | 67094 | tr\|A0A5N5MFD7\|A0A5N5MFD7\_PANHP | 98.55 | 17 | 5 | 3 | N | 11727 | Parvalbumin OS=Pangasianodon hypophthalmus OX=310915 GN=PHYPO\_G00043940 PE=3 SV=1 |
| 20 | 67095 | tr\|A0A087Y0W0\|A0A087Y0W0\_POEFO | 98.55 | 17 | 5 | 3 | N | 11793 | Parvalbumin OS=Poecilia formosa OX=48698 PE=3 SV=1 |
| 20 | 67096 | tr\|A0A668AEA1\|A0A668AEA1\_9TELE | 98.55 | 17 | 5 | 3 | N | 11577 | Parvalbumin OS=Myripristis murdjan OX=586833 GN=LOC115377764 PE=3 SV=1 |
| 20 | 67097 | tr\|C6GKU4\|C6GKU4\_9TELE | 98.55 | 17 | 5 | 3 | N | 11446 | Parvalbumin OS=Sebastes marinus OX=34821 GN=pvalb1 PE=2 SV=1 |
| 20 | 67098 | tr\|A0A087Y5E9\|A0A087Y5E9\_POEFO | 98.55 | 17 | 5 | 3 | N | 11413 | Parvalbumin OS=Poecilia formosa OX=48698 PE=3 SV=2 |
| 20 | 67099 | tr\|A0A1A8GBU0\|A0A1A8GBU0\_9TELE | 98.55 | 17 | 5 | 3 | N | 11460 | Parvalbumin OS=Nothobranchius korthausae OX=1143690 GN=PVALB4 PE=3 SV=1 |
| 20 | 67100 | tr\|E0WD96\|E0WD96\_9TELE | 98.55 | 17 | 5 | 3 | N | 11402 | Parvalbumin OS=Sebastes marinus OX=34821 GN=pvalb1 PE=3 SV=1 |
| 20 | 67101 | tr\|A0A1A7WES6\|A0A1A7WES6\_9TELE | 98.55 | 17 | 5 | 3 | N | 11426 | Parvalbumin OS=Iconisemion striatum OX=60296 GN=PVALB4 PE=3 SV=1 |
| 20 | 67102 | tr\|A0A1A8EAV3\|A0A1A8EAV3\_9TELE | 98.55 | 17 | 5 | 3 | N | 11414 | Parvalbumin OS=Nothobranchius kadleci OX=1051664 GN=PVALB4 PE=3 SV=1 |
| 20 | 67103 | tr\|A0A1A8PM62\|A0A1A8PM62\_9TELE | 98.55 | 17 | 5 | 3 | N | 11414 | Parvalbumin OS=Nothobranchius rachovii OX=451742 GN=PVALB4 PE=3 SV=1 |
| 20 | 67104 | tr\|A0A3B3TQ55\|A0A3B3TQ55\_9TELE | 98.55 | 17 | 5 | 3 | N | 11413 | Parvalbumin OS=Poecilia latipinna OX=48699 PE=3 SV=1 |
| 20 | 67105 | tr\|A0A1A8JGR3\|A0A1A8JGR3\_NOTKU | 98.55 | 17 | 5 | 3 | N | 11414 | Parvalbumin OS=Nothobranchius kuhntae OX=321403 GN=PVALB4 PE=3 SV=1 |
| 20 | 67106 | tr\|A0A3B3XME4\|A0A3B3XME4\_9TELE | 98.55 | 16 | 5 | 3 | N | 11973 | Parvalbumin OS=Poecilia mexicana OX=48701 PE=3 SV=1 |
| 20 | 67107 | tr\|A0A668AEA6\|A0A668AEA6\_9TELE | 98.55 | 15 | 5 | 3 | N | 12474 | Parvalbumin OS=Myripristis murdjan OX=586833 GN=LOC115377764 PE=3 SV=1 |
| 20 | 67108 | tr\|A0A5N5MKE2\|A0A5N5MKE2\_PANHP | 98.55 | 15 | 5 | 3 | N | 12804 | Parvalbumin OS=Pangasianodon hypophthalmus OX=310915 GN=PHYPO\_G00034490 PE=3 SV=1 |
| 20 | 67109 | tr\|A0A668ANA4\|A0A668ANA4\_9TELE | 98.55 | 14 | 5 | 3 | N | 13836 | Parvalbumin OS=Myripristis murdjan OX=586833 GN=LOC115377764 PE=3 SV=1 |
| 20 | 67110 | tr\|A0A3B3UHS0\|A0A3B3UHS0\_9TELE | 98.55 | 13 | 5 | 3 | N | 14779 | Parvalbumin OS=Poecilia latipinna OX=48699 PE=3 SV=1 |
| 20 | 67111 | tr\|A0A556TIT9\|A0A556TIT9\_BAGYA | 98.55 | 9 | 5 | 3 | N | 21698 | Parvalbumin OS=Bagarius yarrelli OX=175774 GN=Baya\_0626 PE=3 SV=1 |
| 20 | 67112 | tr\|A0A7J6BCR9\|A0A7J6BCR9\_AMEME | 98.55 | 7 | 5 | 3 | N | 26250 | Parvalbumin OS=Ameiurus melas OX=219545 GN=AMELA\_G00025130 PE=3 SV=1 |
| 20 | 67113 | tr\|A0A315V786\|A0A315V786\_GAMAF | 98.55 | 6 | 5 | 3 | N | 35443 | Parvalbumin OS=Gambusia affinis OX=33528 GN=CCH79\_00014604 PE=3 SV=1 |
| 16 | 66956 | tr\|A0A1S3QJI3\|A0A1S3QJI3\_SALSA | 96.89 | 5 | 10 | 0 | N | 222009 | myosin heavy chain, fast skeletal muscle-like OS=Salmo salar OX=8030 GN=LOC106593382 PE=3 SV=1 |
| 17 | 66957 | tr\|A0A6J2PWE3\|A0A6J2PWE3\_COTGO | 95.55 | 3 | 9 | 0 | N | 221042 | myosin heavy chain, fast skeletal muscle-like OS=Cottoperca gobio OX=56716 GN=LOC115009637 PE=3 SV=1 |
| 21 | 67118 | tr\|A0A3Q3WCX3\|A0A3Q3WCX3\_MOLML | 95.06 | 16 | 8 | 2 | N | 42571 | Creatine kinase OS=Mola mola OX=94237 PE=3 SV=1 |
| 21 | 67119 | tr\|A0A0S7H3J1\|A0A0S7H3J1\_9TELE | 95.06 | 15 | 8 | 2 | N | 42849 | Creatine kinase OS=Poeciliopsis prolifica OX=188132 GN=KCRM PE=3 SV=1 |
| 21 | 67120 | tr\|A0A3B3XY74\|A0A3B3XY74\_9TELE | 95.06 | 15 | 8 | 2 | N | 42904 | Creatine kinase OS=Poecilia mexicana OX=48701 PE=3 SV=1 |
| 21 | 67121 | tr\|A0A3B3W291\|A0A3B3W291\_9TELE | 95.06 | 15 | 8 | 2 | N | 42860 | Creatine kinase OS=Poecilia latipinna OX=48699 PE=3 SV=1 |
| 21 | 67122 | tr\|A0A087YG46\|A0A087YG46\_POEFO | 95.06 | 15 | 8 | 2 | N | 43062 | Creatine kinase OS=Poecilia formosa OX=48698 GN=CKM PE=3 SV=2 |
| 24 | 67007 | tr\|A0A3Q2D194\|A0A3Q2D194\_CYPVA | 91.50 | 16 | 7 | 1 | N | 42846 | Creatine kinase OS=Cyprinodon variegatus OX=28743 GN=CKM PE=3 SV=1 |
| 24 | 67008 | tr\|A0A3Q2D1K7\|A0A3Q2D1K7\_CYPVA | 91.50 | 17 | 7 | 1 | N | 39261 | Creatine kinase OS=Cyprinodon variegatus OX=28743 GN=CKM PE=3 SV=1 |
| 19 | 67115 | tr\|C7ASM1\|C7ASM1\_SINCH | 91.47 | 18 | 8 | 1 | N | 42922 | Creatine kinase OS=Siniperca chuatsi OX=119488 PE=2 SV=1 |
| 19 | 67116 | tr\|M4AU26\|M4AU26\_XIPMA | 91.47 | 18 | 8 | 1 | N | 42994 | Creatine kinase OS=Xiphophorus maculatus OX=8083 PE=3 SV=1 |
| 19 | 67117 | tr\|A0A3B5M9I5\|A0A3B5M9I5\_9TELE | 91.47 | 18 | 8 | 1 | N | 42994 | Creatine kinase OS=Xiphophorus couchianus OX=32473 PE=3 SV=1 |
| 18 | 66970 | tr\|A0A6J2Q2L7\|A0A6J2Q2L7\_COTGO | 90.05 | 4 | 9 | 0 | N | 224430 | myosin heavy chain, fast skeletal muscle-like OS=Cottoperca gobio OX=56716 GN=LOC115011664 PE=3 SV=1 |
| 22 | 66980 | tr\|A0A6P3WCZ7\|A0A6P3WCZ7\_CLUHA | 86.02 | 4 | 7 | 0 | N | 224559 | myosin heavy chain, fast skeletal muscle-like OS=Clupea harengus OX=7950 GN=LOC105910910 PE=3 SV=1 |
| 23 | 66971 | tr\|A0A4W4E3Q5\|A0A4W4E3Q5\_ELEEL | 85.58 | 7 | 6 | 0 | N | 116476 | Myosin\_tail\_1 domain-containing protein OS=Electrophorus electricus OX=8005 PE=4 SV=1 |
| 25 | 66978 | tr\|A0A3B4FY50\|A0A3B4FY50\_9CICH | 83.82 | 3 | 6 | 0 | N | 222437 | Myosin heavy chain, fast skeletal muscle-like OS=Pundamilia nyererei OX=303518 PE=3 SV=1 |
| 25 | 66979 | tr\|A0A3P9ART7\|A0A3P9ART7\_9CICH | 83.82 | 3 | 6 | 0 | N | 222529 | Myosin heavy chain, fast skeletal muscle-like OS=Maylandia zebra OX=106582 PE=3 SV=1 |
| 32 | 67518 | tr\|D2KQG3\|D2KQG3\_SINCH | 75.08 | 12 | 2 | 1 | N | 11617 | Parvalbumin OS=Siniperca chuatsi OX=119488 PE=2 SV=1 |
| 32 | 67519 | tr\|B9VJM3\|B9VJM3\_SINCH | 75.08 | 12 | 2 | 1 | N | 11589 | Parvalbumin OS=Siniperca chuatsi OX=119488 PE=2 SV=1 |
| 34 | 67240 | tr\|A0A3Q0QYR9\|A0A3Q0QYR9\_AMPCI | 73.47 | 11 | 4 | 4 | N | 13728 | Nucleoside diphosphate kinase B OS=Amphilophus citrinellus OX=61819 PE=3 SV=1 |
| 34 | 30118 | P85280\|NDKB\_MERME | 73.47 | 10 | 4 | 4 | N | 14207 | Nucleoside diphosphate kinase B (Fragments) OS=Merluccius merluccius OX=8063 GN=nme2 PE=1 SV=1 |
| 34 | 30119 | P85284\|NDKB\_MERPA | 73.47 | 10 | 4 | 4 | N | 14207 | Nucleoside diphosphate kinase B (Fragments) OS=Merluccius paradoxus OX=89950 GN=nme2 PE=1 SV=1 |
| 34 | 30114 | P85292\|NDKB\_MACMG | 73.47 | 10 | 4 | 4 | N | 14118 | Nucleoside diphosphate kinase B (Fragments) OS=Macruronus magellanicus OX=92050 GN=nme2 PE=1 SV=1 |
| 34 | 30115 | P85282\|NDKB\_MERSE | 73.47 | 10 | 4 | 4 | N | 14207 | Nucleoside diphosphate kinase B (Fragments) OS=Merluccius senegalensis OX=89953 GN=nme2 PE=1 SV=1 |
| 34 | 30116 | P85283\|NDKB\_MERPO | 73.47 | 10 | 4 | 4 | N | 14207 | Nucleoside diphosphate kinase B (Fragments) OS=Merluccius polli OX=89951 GN=nme2 PE=1 SV=1 |
| 34 | 30123 | P85287\|NDKB\_MERAP | 73.47 | 10 | 4 | 4 | N | 14393 | Nucleoside diphosphate kinase B (Fragments) OS=Merluccius australis polylepis OX=307685 GN=nme2 PE=1 SV=1 |
| 34 | 30124 | P85289\|NDKB\_MERPR | 73.47 | 10 | 4 | 4 | N | 14393 | Nucleoside diphosphate kinase B (Fragments) OS=Merluccius productus OX=89952 GN=nme2 PE=1 SV=1 |
| 34 | 30121 | P85290\|NDKB\_MERBI | 73.47 | 10 | 4 | 4 | N | 14407 | Nucleoside diphosphate kinase B (Fragments) OS=Merluccius bilinearis OX=79698 GN=nme2 PE=1 SV=1 |
| 34 | 67241 | tr\|A0A672Y4P6\|A0A672Y4P6\_9TELE | 73.47 | 9 | 4 | 4 | N | 17050 | Nucleoside diphosphate kinase B OS=Sphaeramia orbicularis OX=375764 GN=LOC115423821 PE=3 SV=1 |
| 34 | 67242 | tr\|A0A2I4CF45\|A0A2I4CF45\_9TELE | 73.47 | 9 | 4 | 4 | N | 16890 | Nucleoside diphosphate kinase B OS=Austrofundulus limnaeus OX=52670 GN=LOC106528082 PE=3 SV=1 |
| 34 | 67243 | tr\|A0A3Q1K2U7\|A0A3Q1K2U7\_ANATE | 73.47 | 9 | 4 | 4 | N | 17020 | Nucleoside diphosphate kinase B OS=Anabas testudineus OX=64144 PE=3 SV=1 |
| 34 | 67244 | tr\|A0A1A8C566\|A0A1A8C566\_9TELE | 73.47 | 9 | 4 | 4 | N | 16875 | Nucleoside diphosphate kinase B OS=Nothobranchius kadleci OX=1051664 GN=NME2B.2 PE=3 SV=1 |
| 34 | 67245 | tr\|A0A6P7ISB4\|A0A6P7ISB4\_9TELE | 73.47 | 9 | 4 | 4 | N | 16935 | Nucleoside diphosphate kinase B OS=Parambassis ranga OX=210632 GN=LOC114440125 PE=3 SV=1 |
| 34 | 67246 | tr\|A0A3Q4I698\|A0A3Q4I698\_NEOBR | 73.47 | 9 | 4 | 4 | N | 16950 | Nucleoside diphosphate kinase B OS=Neolamprologus brichardi OX=32507 PE=3 SV=1 |
| 34 | 67247 | tr\|A0A3Q3L2T8\|A0A3Q3L2T8\_9TELE | 73.47 | 9 | 4 | 4 | N | 17100 | Nucleoside diphosphate kinase B OS=Mastacembelus armatus OX=205130 PE=3 SV=2 |
| 34 | 67248 | tr\|A0A6P7NDJ7\|A0A6P7NDJ7\_BETSP | 73.47 | 9 | 4 | 4 | N | 17113 | Nucleoside diphosphate kinase B OS=Betta splendens OX=158456 GN=LOC114861025 PE=3 SV=1 |
| 34 | 67249 | tr\|A0A3Q3A341\|A0A3Q3A341\_KRYMA | 73.47 | 9 | 4 | 4 | N | 16887 | Nucleoside diphosphate kinase B OS=Kryptolebias marmoratus OX=37003 PE=3 SV=1 |
| 34 | 67250 | tr\|A0A3Q4HQ85\|A0A3Q4HQ85\_NEOBR | 73.47 | 9 | 4 | 4 | N | 16851 | Nucleoside diphosphate kinase B OS=Neolamprologus brichardi OX=32507 PE=3 SV=1 |
| 34 | 67251 | tr\|A0A3P8Q104\|A0A3P8Q104\_ASTCA | 73.47 | 9 | 4 | 4 | N | 17195 | Nucleoside diphosphate kinase B OS=Astatotilapia calliptera OX=8154 PE=3 SV=1 |
| 34 | 67252 | tr\|A0A3Q3CUD8\|A0A3Q3CUD8\_HAPBU | 73.47 | 9 | 4 | 4 | N | 17195 | Nucleoside diphosphate kinase B OS=Haplochromis burtoni OX=8153 PE=3 SV=1 |
| 34 | 67253 | tr\|A0A3Q4I6B8\|A0A3Q4I6B8\_NEOBR | 73.47 | 9 | 4 | 4 | N | 17195 | Nucleoside diphosphate kinase B OS=Neolamprologus brichardi OX=32507 PE=3 SV=1 |
| 34 | 67254 | tr\|A0A3Q0QSW6\|A0A3Q0QSW6\_AMPCI | 73.47 | 9 | 4 | 4 | N | 17238 | Nucleoside diphosphate kinase B OS=Amphilophus citrinellus OX=61819 PE=3 SV=1 |
| 34 | 67255 | tr\|A0A3Q0QYR1\|A0A3Q0QYR1\_AMPCI | 73.47 | 9 | 4 | 4 | N | 17278 | Nucleoside diphosphate kinase B OS=Amphilophus citrinellus OX=61819 PE=3 SV=1 |
| 34 | 67256 | tr\|A0A3Q3R7S1\|A0A3Q3R7S1\_MONAL | 73.47 | 9 | 4 | 4 | N | 17252 | Nucleoside diphosphate kinase B OS=Monopterus albus OX=43700 PE=3 SV=1 |
| 34 | 67257 | tr\|A0A3Q0QXA4\|A0A3Q0QXA4\_AMPCI | 73.47 | 8 | 4 | 4 | N | 17296 | Nucleoside diphosphate kinase B OS=Amphilophus citrinellus OX=61819 PE=3 SV=1 |
| 34 | 67258 | tr\|M4ADR4\|M4ADR4\_XIPMA | 73.47 | 8 | 4 | 4 | N | 18277 | Nucleoside diphosphate kinase B OS=Xiphophorus maculatus OX=8083 PE=3 SV=2 |
| 34 | 67259 | tr\|A0A1A8J8K8\|A0A1A8J8K8\_NOTKU | 73.47 | 8 | 4 | 4 | N | 18247 | Nucleoside diphosphate kinase B (Fragment) OS=Nothobranchius kuhntae OX=321403 GN=NME2B.2 PE=3 SV=1 |
| 34 | 67260 | tr\|A0A1A7XFD3\|A0A1A7XFD3\_9TELE | 73.47 | 7 | 4 | 4 | N | 20309 | Nucleoside diphosphate kinase B (Fragment) OS=Iconisemion striatum OX=60296 GN=NME2B.2 PE=3 SV=1 |
| 34 | 67261 | tr\|A0A0S7LYJ8\|A0A0S7LYJ8\_9TELE | 73.47 | 7 | 4 | 4 | N | 20062 | Nucleoside diphosphate kinase B (Fragment) OS=Poeciliopsis prolifica OX=188132 GN=NDKB PE=3 SV=1 |
| 28 | 67031 | tr\|H3CAK5\|H3CAK5\_TETNG | 71.68 | 14 | 4 | 4 | N | 37106 | 2-phospho-D-glycerate hydro-lyase OS=Tetraodon nigroviridis OX=99883 PE=3 SV=1 |
| 28 | 67032 | tr\|A0A3B3D6F1\|A0A3B3D6F1\_ORYME | 71.68 | 12 | 4 | 4 | N | 42602 | 2-phospho-D-glycerate hydro-lyase OS=Oryzias melastigma OX=30732 PE=3 SV=1 |
| 28 | 67033 | tr\|A0A3B3BLA1\|A0A3B3BLA1\_ORYME | 71.68 | 11 | 4 | 4 | N | 47528 | 2-phospho-D-glycerate hydro-lyase OS=Oryzias melastigma OX=30732 GN=FQA47\_002919 PE=3 SV=1 |
| 28 | 67034 | tr\|A0A3B4CPC9\|A0A3B4CPC9\_PYGNA | 71.68 | 11 | 4 | 4 | N | 47482 | 2-phospho-D-glycerate hydro-lyase OS=Pygocentrus nattereri OX=42514 PE=3 SV=1 |
| 28 | 67035 | tr\|A0A6M4C6H3\|A0A6M4C6H3\_DIOHY | 71.68 | 11 | 4 | 4 | N | 47513 | 2-phospho-D-glycerate hydro-lyase OS=Diodon hystrix OX=223145 GN=ENO3 PE=2 SV=1 |
| 28 | 67036 | tr\|A0A0F8AN77\|A0A0F8AN77\_LARCR | 71.68 | 11 | 4 | 4 | N | 47431 | 2-phospho-D-glycerate hydro-lyase OS=Larimichthys crocea OX=215358 GN=EH28\_01590 PE=3 SV=1 |
| 28 | 67037 | tr\|A0A437CFA6\|A0A437CFA6\_ORYJA | 71.68 | 9 | 4 | 4 | N | 58417 | 2-phospho-D-glycerate hydro-lyase OS=Oryzias javanicus OX=123683 GN=OJAV\_G00185520 PE=3 SV=1 |
| 28 | 67040 | tr\|A0A671TWQ7\|A0A671TWQ7\_SPAAU | 71.68 | 13 | 4 | 4 | N | 38363 | 2-phospho-D-glycerate hydro-lyase OS=Sparus aurata OX=8175 PE=3 SV=1 |
| 28 | 67041 | tr\|A0A671TWN1\|A0A671TWN1\_SPAAU | 71.68 | 13 | 4 | 4 | N | 39726 | 2-phospho-D-glycerate hydro-lyase OS=Sparus aurata OX=8175 PE=3 SV=1 |
| 28 | 67042 | tr\|A0A671TZ14\|A0A671TZ14\_SPAAU | 71.68 | 12 | 4 | 4 | N | 41134 | 2-phospho-D-glycerate hydro-lyase OS=Sparus aurata OX=8175 PE=3 SV=1 |
| 28 | 67043 | tr\|F5BZS7\|F5BZS7\_EPIBR | 71.68 | 12 | 4 | 4 | N | 43226 | 2-phospho-D-glycerate hydro-lyase (Fragment) OS=Epinephelus bruneus OX=323802 PE=2 SV=1 |
| 28 | 67044 | tr\|Q4TBD1\|Q4TBD1\_TETNG | 71.68 | 11 | 4 | 4 | N | 47111 | 2-phospho-D-glycerate hydro-lyase (Fragment) OS=Tetraodon nigroviridis OX=99883 GN=GSTENG00003809001 PE=3 SV=1 |
| 28 | 67045 | tr\|A0A2U9B0J8\|A0A2U9B0J8\_SCOMX | 71.68 | 11 | 4 | 4 | N | 47494 | 2-phospho-D-glycerate hydro-lyase OS=Scophthalmus maximus OX=52904 GN=SMAX5B\_008009 PE=3 SV=1 |
| 28 | 67046 | tr\|A0A6J2P9G6\|A0A6J2P9G6\_COTGO | 71.68 | 11 | 4 | 4 | N | 47397 | 2-phospho-D-glycerate hydro-lyase OS=Cottoperca gobio OX=56716 GN=LOC115004495 PE=3 SV=1 |
| 28 | 67047 | tr\|D6PVP1\|D6PVP1\_EPICO | 71.68 | 11 | 4 | 4 | N | 47516 | 2-phospho-D-glycerate hydro-lyase OS=Epinephelus coioides OX=94232 PE=2 SV=1 |
| 28 | 67048 | tr\|A0A6A4S9A7\|A0A6A4S9A7\_SCOMX | 71.68 | 7 | 4 | 4 | N | 70779 | 2-phospho-D-glycerate hydro-lyase OS=Scophthalmus maximus OX=52904 GN=F2P81\_017933 PE=3 SV=1 |
| 31 | 67269 | tr\|A0A6P7KDL5\|A0A6P7KDL5\_9TELE | 71.63 | 8 | 4 | 4 | N | 32714 | tropomyosin alpha-1 chain isoform X1 OS=Parambassis ranga OX=210632 GN=LOC114452528 PE=3 SV=1 |
| 31 | 67270 | tr\|A0A6P7MR93\|A0A6P7MR93\_BETSP | 71.63 | 8 | 4 | 4 | N | 32714 | tropomyosin alpha-1 chain isoform X1 OS=Betta splendens OX=158456 GN=tpm1 PE=3 SV=1 |
| 31 | 67271 | tr\|A0A3P9D7I0\|A0A3P9D7I0\_9CICH | 71.63 | 8 | 4 | 4 | N | 32822 | Tropomyosin 1 OS=Maylandia zebra OX=106582 GN=TPM1 PE=3 SV=1 |
| 31 | 67272 | tr\|A0A665VR95\|A0A665VR95\_ECHNA | 71.63 | 8 | 4 | 4 | N | 32714 | Tropomyosin 1 OS=Echeneis naucrates OX=173247 GN=tpm1 PE=3 SV=1 |
| 31 | 67273 | tr\|A0A3P8SLC6\|A0A3P8SLC6\_AMPPE | 71.63 | 8 | 4 | 4 | N | 32754 | Uncharacterized protein OS=Amphiprion percula OX=161767 PE=3 SV=1 |
| 31 | 67274 | tr\|A0A668UDB6\|A0A668UDB6\_OREAU | 71.63 | 7 | 4 | 4 | N | 38586 | Tropomyosin 1 OS=Oreochromis aureus OX=47969 PE=3 SV=1 |
| 31 | 67282 | tr\|X2J4M2\|X2J4M2\_PAROL | 71.63 | 8 | 4 | 4 | N | 32670 | Tropomyosin alpha-1 OS=Paralichthys olivaceus OX=8255 PE=2 SV=1 |
| 31 | 67283 | tr\|H2SQV9\|H2SQV9\_TAKRU | 71.63 | 8 | 4 | 4 | N | 31672 | Uncharacterized protein OS=Takifugu rubripes OX=31033 PE=3 SV=3 |
| 31 | 67311 | tr\|A0A4U5UG20\|A0A4U5UG20\_COLLU | 71.63 | 6 | 4 | 4 | N | 39015 | Tropomyosin alpha-1 chain OS=Collichthys lucidus OX=240159 GN=D9C73\_007538 PE=3 SV=1 |
| 31 | 67337 | tr\|A0A6P3VQ87\|A0A6P3VQ87\_CLUHA | 71.63 | 8 | 4 | 4 | N | 32706 | tropomyosin alpha-3 chain isoform X5 OS=Clupea harengus OX=7950 GN=LOC105896158 PE=3 SV=1 |
| 31 | 67340 | tr\|A0A4W4GLX2\|A0A4W4GLX2\_ELEEL | 71.63 | 8 | 4 | 4 | N | 32017 | Uncharacterized protein OS=Electrophorus electricus OX=8005 GN=LOC113589122 PE=3 SV=1 |
| 33 | 67009 | tr\|A0A2I4D9D9\|A0A2I4D9D9\_9TELE | 63.49 | 4 | 4 | 1 | N | 133831 | collagen alpha-1(I) chain-like isoform X2 OS=Austrofundulus limnaeus OX=52670 GN=LOC106536191 PE=4 SV=1 |
| 33 | 67010 | tr\|A0A2I4D9D6\|A0A2I4D9D6\_9TELE | 63.49 | 4 | 4 | 1 | N | 136808 | collagen alpha-1(I) chain-like isoform X1 OS=Austrofundulus limnaeus OX=52670 GN=LOC106536191 PE=4 SV=1 |
| 26 | 67071 | tr\|A0A3P8WWA2\|A0A3P8WWA2\_CYNSE | 63.15 | 7 | 4 | 1 | N | 28542 | Actin-85C-like OS=Cynoglossus semilaevis OX=244447 PE=3 SV=1 |
| 26 | 67072 | tr\|A0A3P8X278\|A0A3P8X278\_CYNSE | 63.15 | 5 | 4 | 1 | N | 41692 | Actin-85C-like OS=Cynoglossus semilaevis OX=244447 PE=3 SV=1 |
| 26 | 67073 | tr\|A0A3P8X031\|A0A3P8X031\_CYNSE | 63.15 | 5 | 4 | 1 | N | 42049 | Actin-85C-like OS=Cynoglossus semilaevis OX=244447 PE=3 SV=1 |
| 26 | 67074 | tr\|A0A3P8WTX4\|A0A3P8WTX4\_CYNSE | 63.15 | 17 | 4 | 1 | N | 12782 | Actin-85C-like OS=Cynoglossus semilaevis OX=244447 PE=4 SV=1 |
| 26 | 67075 | tr\|A0A3P8WU36\|A0A3P8WU36\_CYNSE | 63.15 | 14 | 4 | 1 | N | 15094 | Actin-85C-like OS=Cynoglossus semilaevis OX=244447 PE=4 SV=1 |
| 26 | 67076 | tr\|A0A3P8WUR0\|A0A3P8WUR0\_CYNSE | 63.15 | 13 | 4 | 1 | N | 16136 | Actin-85C-like OS=Cynoglossus semilaevis OX=244447 PE=4 SV=1 |
| 29 | 67212 | tr\|A0A7N8WP58\|A0A7N8WP58\_9TELE | 63.04 | 4 | 5 | 5 | N | 104947 | Calcium-transporting ATPase OS=Mastacembelus armatus OX=205130 PE=3 SV=1 |
| 29 | 67213 | tr\|A0A668SKK1\|A0A668SKK1\_OREAU | 63.04 | 4 | 5 | 5 | N | 108090 | Calcium-transporting ATPase OS=Oreochromis aureus OX=47969 PE=3 SV=1 |
| 29 | 67214 | tr\|A0A3Q3BHF7\|A0A3Q3BHF7\_KRYMA | 63.04 | 4 | 5 | 5 | N | 108762 | Calcium-transporting ATPase OS=Kryptolebias marmoratus OX=37003 PE=3 SV=1 |
| 29 | 67215 | tr\|A0A3Q2WQG2\|A0A3Q2WQG2\_HAPBU | 63.04 | 4 | 5 | 5 | N | 108554 | Calcium-transporting ATPase OS=Haplochromis burtoni OX=8153 PE=3 SV=1 |
| 29 | 67216 | tr\|A0A3B3DXK1\|A0A3B3DXK1\_ORYME | 63.04 | 4 | 5 | 5 | N | 108606 | Calcium-transporting ATPase OS=Oryzias melastigma OX=30732 PE=3 SV=1 |
| 29 | 67217 | tr\|A0A3Q3BR63\|A0A3Q3BR63\_HAPBU | 63.04 | 4 | 5 | 5 | N | 108978 | Calcium-transporting ATPase OS=Haplochromis burtoni OX=8153 PE=3 SV=1 |
| 29 | 67218 | tr\|A0A3Q3H0F6\|A0A3Q3H0F6\_KRYMA | 63.04 | 4 | 5 | 5 | N | 109277 | Calcium-transporting ATPase OS=Kryptolebias marmoratus OX=37003 PE=3 SV=1 |
| 29 | 67219 | tr\|A0A3P9C9M1\|A0A3P9C9M1\_9CICH | 63.04 | 4 | 5 | 5 | N | 109087 | Calcium-transporting ATPase OS=Maylandia zebra OX=106582 PE=3 SV=1 |
| 29 | 67220 | tr\|A0A668SKH5\|A0A668SKH5\_OREAU | 63.04 | 4 | 5 | 5 | N | 109261 | Calcium-transporting ATPase OS=Oreochromis aureus OX=47969 PE=3 SV=1 |
| 29 | 67221 | tr\|A0A669D951\|A0A669D951\_ORENI | 63.04 | 4 | 5 | 5 | N | 109951 | Calcium-transporting ATPase OS=Oreochromis niloticus OX=8128 GN=LOC100706607 PE=3 SV=1 |
| 29 | 67222 | tr\|A0A674PDV8\|A0A674PDV8\_TAKRU | 63.04 | 4 | 5 | 5 | N | 109836 | Calcium-transporting ATPase OS=Takifugu rubripes OX=31033 GN=LOC101069346 PE=3 SV=1 |
| 29 | 67223 | tr\|A0A674P4B1\|A0A674P4B1\_TAKRU | 63.04 | 4 | 5 | 5 | N | 111179 | Calcium-transporting ATPase OS=Takifugu rubripes OX=31033 GN=LOC101069346 PE=3 SV=1 |
| 30 | 67156 | tr\|A0A3Q1CVI2\|A0A3Q1CVI2\_AMPOC | 62.38 | 23 | 4 | 4 | N | 16402 | Myosin light chain, phosphorylatable, fast skeletal muscle OS=Amphiprion ocellaris OX=80972 PE=4 SV=1 |
| 30 | 67157 | tr\|A0A4U5VU95\|A0A4U5VU95\_COLLU | 62.38 | 23 | 4 | 4 | N | 16791 | Myosin regulatory light chain 2, skeletal muscle isoform DTNB OS=Collichthys lucidus OX=240159 GN=D9C73\_025504 PE=4 SV=1 |
| 30 | 67158 | tr\|A0A6P7II88\|A0A6P7II88\_9TELE | 62.38 | 20 | 4 | 4 | N | 18948 | myosin regulatory light chain 2, skeletal muscle isoform OS=Parambassis ranga OX=210632 GN=mylpf PE=4 SV=1 |
| 30 | 67159 | tr\|A0A3Q4GCJ7\|A0A3Q4GCJ7\_NEOBR | 62.38 | 20 | 4 | 4 | N | 19081 | Myosin regulatory light chain 2, skeletal muscle isoform-like OS=Neolamprologus brichardi OX=32507 PE=4 SV=1 |
| 30 | 67160 | tr\|A0A3P9IWY3\|A0A3P9IWY3\_ORYLA | 62.38 | 20 | 4 | 4 | N | 18979 | Uncharacterized protein OS=Oryzias latipes OX=8090 PE=4 SV=1 |
| 30 | 67161 | tr\|H3C4G7\|H3C4G7\_TETNG | 62.38 | 20 | 4 | 4 | N | 19008 | Myosin light chain, phosphorylatable, fast skeletal muscle a OS=Tetraodon nigroviridis OX=99883 PE=4 SV=1 |
| 30 | 67162 | tr\|A0A3B4VIC8\|A0A3B4VIC8\_SERDU | 62.38 | 20 | 4 | 4 | N | 19032 | Myosin light chain, phosphorylatable, fast skeletal muscle OS=Seriola dumerili OX=41447 PE=4 SV=1 |
| 30 | 67163 | tr\|I6U9W3\|I6U9W3\_ORYJA | 62.38 | 20 | 4 | 4 | N | 19039 | Myosin light chain 2 polypeptide OS=Oryzias javanicus OX=123683 GN=OJAV\_G00082200 PE=2 SV=1 |
| 30 | 67164 | tr\|A0A3P9KRI4\|A0A3P9KRI4\_ORYLA | 62.38 | 18 | 4 | 4 | N | 21414 | Myosin light chain, phosphorylatable, fast skeletal muscle a OS=Oryzias latipes OX=8090 PE=4 SV=1 |
| 30 | 67165 | tr\|A0A3B3HA26\|A0A3B3HA26\_ORYLA | 62.38 | 13 | 4 | 4 | N | 28219 | Uncharacterized protein OS=Oryzias latipes OX=8090 GN=mylpf PE=4 SV=1 |
| 54 | 67200 | tr\|A0A7N8YD85\|A0A7N8YD85\_9TELE | 62.16 | 7 | 2 | 0 | N | 50163 | Calsequestrin OS=Mastacembelus armatus OX=205130 PE=3 SV=1 |
| 54 | 67201 | tr\|A0A7N8XIB6\|A0A7N8XIB6\_9TELE | 62.16 | 7 | 2 | 0 | N | 50507 | Calsequestrin OS=Mastacembelus armatus OX=205130 PE=3 SV=1 |
| 54 | 67202 | tr\|A0A7N8Y4P6\|A0A7N8Y4P6\_9TELE | 62.16 | 7 | 2 | 0 | N | 50544 | Calsequestrin OS=Mastacembelus armatus OX=205130 PE=3 SV=1 |
| 54 | 67203 | tr\|A0A7N8WMA4\|A0A7N8WMA4\_9TELE | 62.16 | 7 | 2 | 0 | N | 50567 | Calsequestrin OS=Mastacembelus armatus OX=205130 PE=3 SV=1 |
| 54 | 67204 | tr\|A0A7N8XJX7\|A0A7N8XJX7\_9TELE | 62.16 | 6 | 2 | 0 | N | 52431 | Calsequestrin OS=Mastacembelus armatus OX=205130 PE=3 SV=1 |
| 54 | 67205 | tr\|A0A3Q3SHA7\|A0A3Q3SHA7\_9TELE | 62.16 | 6 | 2 | 0 | N | 52425 | Calsequestrin OS=Mastacembelus armatus OX=205130 PE=3 SV=2 |
| 54 | 67206 | tr\|A0A7N8XSZ9\|A0A7N8XSZ9\_9TELE | 62.16 | 6 | 2 | 0 | N | 52256 | Calsequestrin OS=Mastacembelus armatus OX=205130 PE=3 SV=1 |
| 54 | 67207 | tr\|A0A7N8XRW1\|A0A7N8XRW1\_9TELE | 62.16 | 6 | 2 | 0 | N | 52191 | Calsequestrin OS=Mastacembelus armatus OX=205130 PE=3 SV=1 |
| 54 | 67208 | tr\|A0A7N8Y2R6\|A0A7N8Y2R6\_9TELE | 62.16 | 6 | 2 | 0 | N | 52373 | Calsequestrin OS=Mastacembelus armatus OX=205130 PE=3 SV=1 |
| 54 | 67209 | tr\|A0A7N8Y336\|A0A7N8Y336\_9TELE | 62.16 | 6 | 2 | 0 | N | 52793 | Calsequestrin OS=Mastacembelus armatus OX=205130 PE=3 SV=1 |
| 41 | 67018 | tr\|I3JGN1\|I3JGN1\_ORENI | 56.43 | 4 | 3 | 0 | N | 121918 | Uncharacterized protein OS=Oreochromis niloticus OX=8128 GN=col1a1 PE=4 SV=2 |
| 41 | 67019 | tr\|G9M6I5\|G9M6I5\_ORENI | 56.43 | 3 | 3 | 0 | N | 137283 | Collagen type I alpha 1 OS=Oreochromis niloticus OX=8128 GN=COL1A1 PE=2 SV=1 |
| 37 | 67005 | tr\|Q4RE90\|Q4RE90\_TETNG | 55.57 | 1 | 3 | 3 | N | 863297 | Chromosome undetermined SCAF15134, whole genome shotgun sequence (Fragment) OS=Tetraodon nigroviridis OX=99883 GN=GSTENG00035871001 PE=4 SV=1 |
| 48 | 67341 | tr\|A0A484CUS5\|A0A484CUS5\_PERFV | 55.47 | 0 | 2 | 1 | N | 867139 | Uncharacterized protein OS=Perca flavescens OX=8167 GN=EPR50\_G00113850 PE=4 SV=1 |
| 38 | 67211 | tr\|A0A6G0I8D2\|A0A6G0I8D2\_LARCR | 53.80 | 2 | 3 | 3 | N | 97204 | Alpha-1,4 glucan phosphorylase OS=Larimichthys crocea OX=215358 GN=D5F01\_LYC13586 PE=3 SV=1 |
| 38 | 67322 | tr\|A0A669CG83\|A0A669CG83\_ORENI | 53.80 | 2 | 3 | 3 | N | 91665 | Alpha-1,4 glucan phosphorylase OS=Oreochromis niloticus OX=8128 PE=3 SV=1 |
| 38 | 67323 | tr\|A0A3P8QJ74\|A0A3P8QJ74\_ASTCA | 53.80 | 2 | 3 | 3 | N | 91586 | Alpha-1,4 glucan phosphorylase OS=Astatotilapia calliptera OX=8154 PE=3 SV=1 |
| 38 | 67324 | tr\|A0A669E543\|A0A669E543\_ORENI | 53.80 | 2 | 3 | 3 | N | 93767 | Alpha-1,4 glucan phosphorylase OS=Oreochromis niloticus OX=8128 PE=3 SV=1 |
| 38 | 67325 | tr\|A0A3B4C8L5\|A0A3B4C8L5\_PYGNA | 53.80 | 2 | 3 | 3 | N | 93768 | Alpha-1,4 glucan phosphorylase OS=Pygocentrus nattereri OX=42514 PE=3 SV=1 |
| 38 | 67326 | tr\|A0A669F5E7\|A0A669F5E7\_ORENI | 53.80 | 2 | 3 | 3 | N | 94615 | Alpha-1,4 glucan phosphorylase OS=Oreochromis niloticus OX=8128 PE=3 SV=1 |
| 38 | 67327 | tr\|A0A672GIA2\|A0A672GIA2\_SALFA | 53.80 | 2 | 3 | 3 | N | 96233 | Alpha-1,4 glucan phosphorylase OS=Salarias fasciatus OX=181472 GN=LOC115395314 PE=3 SV=1 |
| 38 | 67328 | tr\|A0A3B4B6P0\|A0A3B4B6P0\_9GOBI | 53.80 | 2 | 3 | 3 | N | 97017 | Alpha-1,4 glucan phosphorylase OS=Periophthalmus magnuspinnatus OX=409849 PE=3 SV=1 |
| 38 | 67329 | tr\|A0A6P7PB25\|A0A6P7PB25\_BETSP | 53.80 | 2 | 3 | 3 | N | 97104 | Alpha-1,4 glucan phosphorylase OS=Betta splendens OX=158456 GN=LOC114869705 PE=3 SV=1 |
| 38 | 67330 | tr\|M4A9F5\|M4A9F5\_XIPMA | 53.80 | 2 | 3 | 3 | N | 97256 | Alpha-1,4 glucan phosphorylase OS=Xiphophorus maculatus OX=8083 PE=3 SV=1 |
| 38 | 67331 | tr\|A0A498LG69\|A0A498LG69\_LABRO | 53.80 | 2 | 3 | 3 | N | 97018 | Alpha-1,4 glucan phosphorylase OS=Labeo rohita OX=84645 GN=ROHU\_012033 PE=3 SV=1 |
| 38 | 67332 | tr\|A0A0S7H0F8\|A0A0S7H0F8\_9TELE | 53.80 | 2 | 3 | 3 | N | 97085 | Alpha-1,4 glucan phosphorylase OS=Poeciliopsis prolifica OX=188132 GN=PYGM PE=3 SV=1 |
| 38 | 67335 | tr\|A0A5J5D262\|A0A5J5D262\_9PERO | 53.80 | 2 | 3 | 3 | N | 98712 | Alpha-1,4 glucan phosphorylase (Fragment) OS=Etheostoma spectabile OX=54343 GN=FQN60\_000806 PE=3 SV=1 |
| 49 | 67343 | tr\|A0A6I9P2J6\|A0A6I9P2J6\_9TELE | 52.39 | 6 | 2 | 0 | N | 51099 | Calsequestrin OS=Notothenia coriiceps OX=8208 GN=LOC104956576 PE=3 SV=1 |
| 49 | 67344 | tr\|A0A2U9BKK8\|A0A2U9BKK8\_SCOMX | 52.39 | 6 | 2 | 0 | N | 52892 | Calsequestrin OS=Scophthalmus maximus OX=52904 GN=SMAX5B\_006105 PE=3 SV=1 |
| 49 | 67345 | tr\|A0A6J2RKV3\|A0A6J2RKV3\_COTGO | 52.39 | 6 | 2 | 0 | N | 53535 | Calsequestrin OS=Cottoperca gobio OX=56716 GN=LOC115023113 PE=3 SV=1 |
| 49 | 67346 | tr\|A0A6P8WKA1\|A0A6P8WKA1\_GYMAC | 52.39 | 6 | 2 | 0 | N | 55298 | Calsequestrin OS=Gymnodraco acuticeps OX=8218 GN=casq1a PE=3 SV=1 |
| 49 | 67347 | tr\|A0A6A4TIJ9\|A0A6A4TIJ9\_SCOMX | 52.39 | 4 | 2 | 0 | N | 89028 | Calsequestrin OS=Scophthalmus maximus OX=52904 GN=F2P81\_000969 PE=3 SV=1 |
| 49 | 67436 | tr\|A0A3P8VNQ7\|A0A3P8VNQ7\_CYNSE | 52.39 | 6 | 2 | 0 | N | 49877 | Calsequestrin OS=Cynoglossus semilaevis OX=244447 PE=3 SV=1 |
| 49 | 67437 | tr\|A0A3P8VQY9\|A0A3P8VQY9\_CYNSE | 52.39 | 6 | 2 | 0 | N | 52455 | Calsequestrin OS=Cynoglossus semilaevis OX=244447 PE=3 SV=1 |
| 49 | 67438 | tr\|A0A2I4B9K0\|A0A2I4B9K0\_9TELE | 52.39 | 5 | 2 | 0 | N | 59100 | Calsequestrin OS=Austrofundulus limnaeus OX=52670 GN=LOC106517901 PE=3 SV=1 |
| 55 | 67284 | tr\|A0A673A8U8\|A0A673A8U8\_9TELE | 51.18 | 3 | 2 | 1 | N | 111385 | Collagen, type I, alpha 1b OS=Sphaeramia orbicularis OX=375764 PE=4 SV=1 |
| 51 | 67439 | tr\|A0A671U7M6\|A0A671U7M6\_SPAAU | 49.51 | 4 | 2 | 2 | N | 39670 | Troponin T, fast skeletal muscle isoforms-like OS=Sparus aurata OX=8175 GN=LOC115587311 PE=4 SV=1 |
| 51 | 67449 | tr\|A0A3B3ZS88\|A0A3B3ZS88\_9GOBI | 49.51 | 7 | 2 | 2 | N | 25528 | Uncharacterized protein OS=Periophthalmus magnuspinnatus OX=409849 PE=3 SV=1 |
| 51 | 67450 | tr\|A0A3Q1CJW7\|A0A3Q1CJW7\_AMPOC | 49.51 | 7 | 2 | 2 | N | 26282 | Troponin T, fast skeletal muscle isoforms-like OS=Amphiprion ocellaris OX=80972 PE=3 SV=1 |
| 51 | 67451 | tr\|A0A3Q3EFK1\|A0A3Q3EFK1\_9LABR | 49.51 | 7 | 2 | 2 | N | 26666 | Troponin T, fast skeletal muscle isoforms-like OS=Labrus bergylta OX=56723 PE=3 SV=1 |
| 51 | 67452 | tr\|A0A3Q3AF19\|A0A3Q3AF19\_KRYMA | 49.51 | 7 | 2 | 2 | N | 26771 | Troponin T, fast skeletal muscle isoforms-like OS=Kryptolebias marmoratus OX=37003 PE=3 SV=1 |
| 51 | 67453 | tr\|A0A3Q3AF32\|A0A3Q3AF32\_KRYMA | 49.51 | 7 | 2 | 2 | N | 26884 | Troponin T, fast skeletal muscle isoforms-like OS=Kryptolebias marmoratus OX=37003 PE=3 SV=1 |
| 51 | 67454 | tr\|A0A3P8UNE3\|A0A3P8UNE3\_CYNSE | 49.51 | 7 | 2 | 2 | N | 27351 | Troponin T, fast skeletal muscle isoforms-like OS=Cynoglossus semilaevis OX=244447 PE=3 SV=1 |
| 51 | 67455 | tr\|A0SJB3\|A0SJB3\_SPAAU | 49.51 | 7 | 2 | 2 | N | 27348 | Fast/white muscle troponin T larval isoform OS=Sparus aurata OX=8175 PE=2 SV=1 |
| 51 | 67456 | tr\|A0A672Y3T4\|A0A672Y3T4\_9TELE | 49.51 | 7 | 2 | 2 | N | 27423 | Uncharacterized protein OS=Sphaeramia orbicularis OX=375764 GN=LOC115421585 PE=3 SV=1 |
| 51 | 67457 | tr\|A0SJB2\|A0SJB2\_SPAAU | 49.51 | 6 | 2 | 2 | N | 27755 | Fast/white muscle troponin T adult isoform OS=Sparus aurata OX=8175 PE=2 SV=1 |
| 51 | 67458 | tr\|A0A3P8UNF6\|A0A3P8UNF6\_CYNSE | 49.51 | 6 | 2 | 2 | N | 28353 | Troponin T, fast skeletal muscle isoforms-like OS=Cynoglossus semilaevis OX=244447 PE=3 SV=1 |
| 51 | 67459 | tr\|A0A3B4UYC0\|A0A3B4UYC0\_SERDU | 49.51 | 6 | 2 | 2 | N | 29388 | Troponin T type 3a (skeletal, fast) OS=Seriola dumerili OX=41447 PE=3 SV=1 |
| 51 | 67460 | tr\|A0A3P8URR7\|A0A3P8URR7\_CYNSE | 49.51 | 6 | 2 | 2 | N | 29425 | Troponin T, fast skeletal muscle isoforms-like OS=Cynoglossus semilaevis OX=244447 PE=3 SV=1 |
| 51 | 67461 | tr\|A0A3P8URM3\|A0A3P8URM3\_CYNSE | 49.51 | 6 | 2 | 2 | N | 30206 | Troponin T, fast skeletal muscle isoforms-like OS=Cynoglossus semilaevis OX=244447 PE=3 SV=1 |
| 51 | 67462 | tr\|A0A3Q3XL97\|A0A3Q3XL97\_MOLML | 49.51 | 6 | 2 | 2 | N | 30493 | Troponin T type 3a (skeletal, fast) OS=Mola mola OX=94237 PE=3 SV=1 |
| 51 | 67463 | tr\|A0A3P8ULW6\|A0A3P8ULW6\_CYNSE | 49.51 | 6 | 2 | 2 | N | 31797 | Troponin T, fast skeletal muscle isoforms-like OS=Cynoglossus semilaevis OX=244447 PE=3 SV=1 |
| 51 | 67464 | tr\|A0A5C6P7G2\|A0A5C6P7G2\_9TELE | 49.51 | 6 | 2 | 2 | N | 32128 | Troponin T, fast skeletal muscle isoforms OS=Takifugu flavidus OX=433684 GN=D4764\_13G0000270 PE=3 SV=1 |
| 51 | 67465 | tr\|A0A3Q1CRB3\|A0A3Q1CRB3\_AMPOC | 49.51 | 5 | 2 | 2 | N | 32379 | Troponin T, fast skeletal muscle isoforms-like OS=Amphiprion ocellaris OX=80972 PE=3 SV=1 |
| 51 | 67466 | tr\|A0A665T7Y2\|A0A665T7Y2\_ECHNA | 49.51 | 5 | 2 | 2 | N | 33067 | Troponin T type 3a (skeletal, fast) OS=Echeneis naucrates OX=173247 PE=3 SV=1 |
| 51 | 67467 | tr\|A0A3Q1BEW8\|A0A3Q1BEW8\_AMPOC | 49.51 | 5 | 2 | 2 | N | 33080 | Troponin T, fast skeletal muscle isoforms-like OS=Amphiprion ocellaris OX=80972 PE=3 SV=1 |
| 51 | 67468 | tr\|A0A3P8THB6\|A0A3P8THB6\_AMPPE | 49.51 | 5 | 2 | 2 | N | 33365 | Troponin T type 3a (skeletal, fast) OS=Amphiprion percula OX=161767 PE=3 SV=1 |
| 51 | 67469 | tr\|A0A671U7Q2\|A0A671U7Q2\_SPAAU | 49.51 | 5 | 2 | 2 | N | 33456 | Troponin T, fast skeletal muscle isoforms-like OS=Sparus aurata OX=8175 GN=LOC115587311 PE=3 SV=1 |
| 51 | 67470 | tr\|A0A672YCL7\|A0A672YCL7\_9TELE | 49.51 | 5 | 2 | 2 | N | 33478 | Uncharacterized protein OS=Sphaeramia orbicularis OX=375764 GN=LOC115421585 PE=3 SV=1 |
| 51 | 67471 | tr\|A0A672YCL2\|A0A672YCL2\_9TELE | 49.51 | 5 | 2 | 2 | N | 34165 | Uncharacterized protein OS=Sphaeramia orbicularis OX=375764 GN=LOC115421585 PE=3 SV=1 |
| 51 | 67472 | tr\|A0A6P7KCL6\|A0A6P7KCL6\_9TELE | 49.51 | 5 | 2 | 2 | N | 34619 | troponin T, fast skeletal muscle isoforms-like isoform X3 OS=Parambassis ranga OX=210632 GN=LOC114452235 PE=3 SV=1 |
| 51 | 67473 | tr\|A0A6P7KCQ1\|A0A6P7KCQ1\_9TELE | 49.51 | 5 | 2 | 2 | N | 35105 | troponin T, fast skeletal muscle isoforms-like isoform X2 OS=Parambassis ranga OX=210632 GN=LOC114452235 PE=3 SV=1 |
| 51 | 67474 | tr\|A0A671UB90\|A0A671UB90\_SPAAU | 49.51 | 4 | 2 | 2 | N | 38604 | Troponin T, fast skeletal muscle isoforms-like OS=Sparus aurata OX=8175 GN=LOC115587311 PE=3 SV=1 |
| 51 | 67475 | tr\|A0A671U8G8\|A0A671U8G8\_SPAAU | 49.51 | 4 | 2 | 2 | N | 39011 | Troponin T, fast skeletal muscle isoforms-like OS=Sparus aurata OX=8175 GN=LOC115587311 PE=3 SV=1 |
| 51 | 67545 | tr\|A0A671U7M0\|A0A671U7M0\_SPAAU | 49.51 | 5 | 2 | 2 | N | 36576 | Troponin T, fast skeletal muscle isoforms-like OS=Sparus aurata OX=8175 GN=LOC115587311 PE=4 SV=1 |
| 51 | 67546 | tr\|A0A671UB76\|A0A671UB76\_SPAAU | 49.51 | 4 | 2 | 2 | N | 38011 | Troponin T, fast skeletal muscle isoforms-like OS=Sparus aurata OX=8175 GN=LOC115587311 PE=4 SV=1 |
| 36 | 67357 | tr\|A0A6A5F2V4\|A0A6A5F2V4\_PERFL | 48.73 | 0 | 3 | 1 | N | 1083091 | Uncharacterized protein OS=Perca fluviatilis OX=8168 GN=PFLUV\_G00150840 PE=4 SV=1 |
| 45 | 67277 | tr\|A0A0S7EPU5\|A0A0S7EPU5\_9TELE | 48.44 | 0 | 2 | 0 | N | 534030 | TITIN OS=Poeciliopsis prolifica OX=188132 GN=TITIN PE=4 SV=1 |
| 45 | 67278 | tr\|A0A0S7ETZ8\|A0A0S7ETZ8\_9TELE | 48.44 | 0 | 2 | 0 | N | 536335 | TITIN (Fragment) OS=Poeciliopsis prolifica OX=188132 GN=TITIN PE=4 SV=1 |
| 45 | 67279 | tr\|A0A0S7EQ56\|A0A0S7EQ56\_9TELE | 48.44 | 0 | 2 | 0 | N | 555084 | TITIN OS=Poeciliopsis prolifica OX=188132 GN=TITIN PE=4 SV=1 |
| 45 | 67280 | tr\|A0A0S7F0D9\|A0A0S7F0D9\_9TELE | 48.44 | 0 | 2 | 0 | N | 557390 | TITIN (Fragment) OS=Poeciliopsis prolifica OX=188132 GN=TITIN PE=4 SV=1 |
| 57 | 67596 | tr\|A0A146UF80\|A0A146UF80\_FUNHE | 47.30 | 5 | 2 | 2 | N | 29240 | Phosphoglycerate mutase (Fragment) OS=Fundulus heteroclitus OX=8078 PE=3 SV=1 |
| 39 | 67077 | tr\|A0A671YMU3\|A0A671YMU3\_SPAAU | 47.29 | 3 | 3 | 0 | N | 118161 | Collagen alpha-1(I) chain-like OS=Sparus aurata OX=8175 GN=LOC115575148 PE=4 SV=1 |
| 39 | 67078 | tr\|A0A671YMZ1\|A0A671YMZ1\_SPAAU | 47.29 | 3 | 3 | 0 | N | 120762 | Collagen alpha-1(I) chain-like OS=Sparus aurata OX=8175 GN=LOC115575148 PE=4 SV=1 |
| 39 | 67079 | tr\|A0A671YQ85\|A0A671YQ85\_SPAAU | 47.29 | 3 | 3 | 0 | N | 120531 | Collagen alpha-1(I) chain-like OS=Sparus aurata OX=8175 GN=LOC115575148 PE=4 SV=1 |
| 39 | 67080 | tr\|A0A671YMZ6\|A0A671YMZ6\_SPAAU | 47.29 | 3 | 3 | 0 | N | 122692 | Collagen alpha-1(I) chain-like OS=Sparus aurata OX=8175 GN=LOC115575148 PE=4 SV=1 |
| 39 | 67081 | tr\|A0A671YNQ4\|A0A671YNQ4\_SPAAU | 47.29 | 3 | 3 | 0 | N | 122121 | Collagen alpha-1(I) chain-like OS=Sparus aurata OX=8175 GN=LOC115575148 PE=4 SV=1 |
| 39 | 67082 | tr\|A0A671YN33\|A0A671YN33\_SPAAU | 47.29 | 3 | 3 | 0 | N | 129109 | Collagen alpha-1(I) chain-like OS=Sparus aurata OX=8175 GN=LOC115575148 PE=4 SV=1 |
| 39 | 67083 | tr\|A0A671YQA1\|A0A671YQA1\_SPAAU | 47.29 | 3 | 3 | 0 | N | 128328 | Collagen alpha-1(I) chain-like OS=Sparus aurata OX=8175 GN=LOC115575148 PE=4 SV=1 |
| 39 | 67084 | tr\|A0A671YNR4\|A0A671YNR4\_SPAAU | 47.29 | 3 | 3 | 0 | N | 130557 | Collagen alpha-1(I) chain-like OS=Sparus aurata OX=8175 GN=LOC115575148 PE=4 SV=1 |
| 39 | 67085 | tr\|A0A671YPG6\|A0A671YPG6\_SPAAU | 47.29 | 3 | 3 | 0 | N | 134738 | Collagen alpha-1(I) chain-like OS=Sparus aurata OX=8175 GN=LOC115575148 PE=4 SV=1 |
| 39 | 67086 | tr\|A0A671YMN4\|A0A671YMN4\_SPAAU | 47.29 | 3 | 3 | 0 | N | 136688 | Collagen alpha-1(I) chain-like OS=Sparus aurata OX=8175 GN=LOC115575148 PE=4 SV=1 |
| 39 | 67114 | tr\|A0A3Q3RR05\|A0A3Q3RR05\_9TELE | 47.29 | 3 | 3 | 0 | N | 117109 | Collagen, type I, alpha 1a OS=Mastacembelus armatus OX=205130 PE=4 SV=2 |
| 39 | 67147 | tr\|A0A0F8B7E5\|A0A0F8B7E5\_LARCR | 47.29 | 3 | 3 | 0 | N | 133708 | Collagen alpha-1(I) chain OS=Larimichthys crocea OX=215358 GN=EH28\_11446 PE=4 SV=1 |
| 39 | 67148 | tr\|A0A6G0HX74\|A0A6G0HX74\_LARCR | 47.29 | 3 | 3 | 0 | N | 136864 | Collagen alpha-1(I) chain Alpha-1 type I collagen OS=Larimichthys crocea OX=215358 GN=D5F01\_LYC19014 PE=4 SV=1 |
| 39 | 67149 | tr\|A0A5J6XWD3\|A0A5J6XWD3\_NIBCO | 47.29 | 3 | 3 | 0 | N | 136596 | Collagen type I alpha 1 chain OS=Nibea coibor OX=396775 GN=COL1A1 PE=2 SV=1 |
| 39 | 67150 | tr\|A0A7N8XDE6\|A0A7N8XDE6\_9TELE | 47.29 | 3 | 3 | 0 | N | 116695 | Collagen, type I, alpha 1a OS=Mastacembelus armatus OX=205130 PE=4 SV=1 |
| 39 | 67151 | tr\|A0A7N8WW01\|A0A7N8WW01\_9TELE | 47.29 | 3 | 3 | 0 | N | 123048 | Collagen, type I, alpha 1a OS=Mastacembelus armatus OX=205130 PE=4 SV=1 |
| 39 | 67152 | tr\|A0A7N8XAN7\|A0A7N8XAN7\_9TELE | 47.29 | 3 | 3 | 0 | N | 126162 | Collagen, type I, alpha 1a OS=Mastacembelus armatus OX=205130 PE=4 SV=1 |
| 35 | 67400 | tr\|A0A4W4EA72\|A0A4W4EA72\_ELEEL | 44.91 | 17 | 2 | 1 | N | 11756 | Parvalbumin OS=Electrophorus electricus OX=8005 GN=LOC113569881 PE=3 SV=1 |
| 47 | 67404 | tr\|A0A6F9CEX9\|A0A6F9CEX9\_9TELE | 44.31 | 0 | 2 | 1 | N | 1022296 | Uncharacterized protein OS=Coregonus sp. 'balchen' OX=861768 GN=CSTEINMANNI\_LOCUS3957823 PE=4 SV=1 |
| 42 | 67348 | tr\|A0A668V0Q0\|A0A668V0Q0\_OREAU | 44.26 | 6 | 2 | 1 | N | 37062 | Glyceraldehyde-3-phosphate dehydrogenase OS=Oreochromis aureus OX=47969 PE=3 SV=1 |
| 42 | 67355 | tr\|Q8AWX8\|Q8AWX8\_GADMO | 44.26 | 6 | 2 | 1 | N | 36092 | Glyceraldehyde-3-phosphate dehydrogenase OS=Gadus morhua OX=8049 PE=2 SV=1 |
| 42 | 67356 | tr\|A0A6P7KWB3\|A0A6P7KWB3\_BETSP | 44.26 | 6 | 2 | 1 | N | 35917 | Glyceraldehyde-3-phosphate dehydrogenase OS=Betta splendens OX=158456 GN=gapdh PE=3 SV=1 |
| 42 | 67507 | tr\|Q4VKA7\|Q4VKA7\_PARLE | 44.26 | 18 | 2 | 1 | N | 12254 | Glyceraldehyde-3-phosphate dehydrogenase (Fragment) OS=Paralichthys lethostigma OX=141886 GN=GAPDH PE=2 SV=1 |
| 50 | 67379 | tr\|A0A4W6CL45\|A0A4W6CL45\_LATCA | 43.11 | 6 | 2 | 0 | N | 43959 | Calsequestrin OS=Lates calcarifer OX=8187 GN=LOC108883924 PE=3 SV=1 |
| 50 | 67380 | tr\|A0A671XZM8\|A0A671XZM8\_SPAAU | 43.11 | 6 | 2 | 0 | N | 45256 | Calsequestrin OS=Sparus aurata OX=8175 GN=LOC115572796 PE=3 SV=1 |
| 50 | 67381 | tr\|A0A671Y6A5\|A0A671Y6A5\_SPAAU | 43.11 | 5 | 2 | 0 | N | 50365 | Calsequestrin OS=Sparus aurata OX=8175 GN=LOC115572796 PE=3 SV=1 |
| 50 | 67382 | tr\|A0A672HWD2\|A0A672HWD2\_SALFA | 43.11 | 5 | 2 | 0 | N | 50618 | Calsequestrin OS=Salarias fasciatus OX=181472 PE=3 SV=1 |
| 50 | 67383 | tr\|A0A672HWL2\|A0A672HWL2\_SALFA | 43.11 | 5 | 2 | 0 | N | 51610 | Calsequestrin OS=Salarias fasciatus OX=181472 PE=3 SV=1 |
| 50 | 67384 | tr\|A0A671XZM4\|A0A671XZM4\_SPAAU | 43.11 | 5 | 2 | 0 | N | 51763 | Calsequestrin OS=Sparus aurata OX=8175 GN=LOC115572796 PE=3 SV=1 |
| 50 | 67385 | tr\|A0A672HWC2\|A0A672HWC2\_SALFA | 43.11 | 5 | 2 | 0 | N | 52548 | Calsequestrin OS=Salarias fasciatus OX=181472 PE=3 SV=1 |
| 50 | 67386 | tr\|A0A672HXH8\|A0A672HXH8\_SALFA | 43.11 | 5 | 2 | 0 | N | 52344 | Calsequestrin OS=Salarias fasciatus OX=181472 PE=3 SV=1 |
| 50 | 67387 | tr\|A0A672HWL0\|A0A672HWL0\_SALFA | 43.11 | 5 | 2 | 0 | N | 52538 | Calsequestrin OS=Salarias fasciatus OX=181472 PE=3 SV=1 |
| 50 | 67388 | tr\|A0A672HXF4\|A0A672HXF4\_SALFA | 43.11 | 5 | 2 | 0 | N | 52636 | Calsequestrin OS=Salarias fasciatus OX=181472 PE=3 SV=1 |
| 50 | 67389 | tr\|A0A671XZK3\|A0A671XZK3\_SPAAU | 43.11 | 5 | 2 | 0 | N | 52734 | Calsequestrin OS=Sparus aurata OX=8175 GN=LOC115572796 PE=3 SV=1 |
| 50 | 67390 | tr\|A0A672HWP0\|A0A672HWP0\_SALFA | 43.11 | 5 | 2 | 0 | N | 52933 | Calsequestrin OS=Salarias fasciatus OX=181472 PE=3 SV=1 |
| 50 | 67391 | tr\|A0A4W6CL16\|A0A4W6CL16\_LATCA | 43.11 | 5 | 2 | 0 | N | 52990 | Calsequestrin OS=Lates calcarifer OX=8187 GN=LOC108883924 PE=3 SV=1 |
| 50 | 67392 | tr\|A0A4W6CL41\|A0A4W6CL41\_LATCA | 43.11 | 5 | 2 | 0 | N | 53391 | Calsequestrin OS=Lates calcarifer OX=8187 GN=LOC108883924 PE=3 SV=1 |
| 50 | 67393 | tr\|A0A672HXA9\|A0A672HXA9\_SALFA | 43.11 | 5 | 2 | 0 | N | 53688 | Calsequestrin OS=Salarias fasciatus OX=181472 PE=3 SV=1 |
| 50 | 67394 | tr\|A0A672HW84\|A0A672HW84\_SALFA | 43.11 | 5 | 2 | 0 | N | 53918 | Calsequestrin OS=Salarias fasciatus OX=181472 PE=3 SV=1 |
| 50 | 67395 | tr\|A0A671XZP8\|A0A671XZP8\_SPAAU | 43.11 | 5 | 2 | 0 | N | 55189 | Calsequestrin OS=Sparus aurata OX=8175 GN=LOC115572796 PE=3 SV=1 |
| 50 | 67396 | tr\|A0A4U5UZN8\|A0A4U5UZN8\_COLLU | 43.11 | 3 | 2 | 0 | N | 98899 | Calsequestrin OS=Collichthys lucidus OX=240159 GN=D9C73\_013496 PE=3 SV=1 |
| 50 | 67508 | tr\|A0A3Q3JCJ6\|A0A3Q3JCJ6\_MONAL | 43.11 | 5 | 2 | 0 | N | 49224 | Calsequestrin OS=Monopterus albus OX=43700 PE=3 SV=1 |
| 50 | 67509 | tr\|A0A3B4WCW4\|A0A3B4WCW4\_SERLL | 43.11 | 5 | 2 | 0 | N | 50048 | Calsequestrin OS=Seriola lalandi dorsalis OX=1841481 PE=3 SV=1 |
| 50 | 67510 | tr\|A0A3B4UFZ1\|A0A3B4UFZ1\_SERDU | 43.11 | 5 | 2 | 0 | N | 50935 | Calsequestrin OS=Seriola dumerili OX=41447 PE=3 SV=1 |
| 50 | 67511 | tr\|A0A3B4WCV3\|A0A3B4WCV3\_SERLL | 43.11 | 5 | 2 | 0 | N | 51404 | Calsequestrin OS=Seriola lalandi dorsalis OX=1841481 PE=3 SV=1 |
| 40 | 67440 | tr\|A0A484CIB2\|A0A484CIB2\_PERFV | 41.48 | 3 | 3 | 0 | N | 136760 | Uncharacterized protein OS=Perca flavescens OX=8167 GN=EPR50\_G00163440 PE=4 SV=1 |
| 52 | 67489 | tr\|A0A6A4SNF9\|A0A6A4SNF9\_SCOMX | 41.47 | 3 | 2 | 1 | N | 77468 | Parvalbumin OS=Scophthalmus maximus OX=52904 GN=F2P81\_014924 PE=3 SV=1 |
| 56 | 67550 | tr\|A0A3B3CIU5\|A0A3B3CIU5\_ORYME | 39.53 | 2 | 2 | 0 | N | 111992 | Collagen type II alpha 1 chain OS=Oryzias melastigma OX=30732 GN=COL2A1 PE=4 SV=1 |
| 56 | 67551 | tr\|A0A834FQR3\|A0A834FQR3\_ORYME | 39.53 | 2 | 2 | 0 | N | 140416 | Collagen alpha-1(II) chain OS=Oryzias melastigma OX=30732 GN=FQA47\_017639 PE=4 SV=1 |
| 56 | 67552 | tr\|A0A437DEJ9\|A0A437DEJ9\_ORYJA | 39.53 | 2 | 2 | 0 | N | 142714 | Uncharacterized protein OS=Oryzias javanicus OX=123683 GN=OJAV\_G00046120 PE=4 SV=1 |
| 46 | 67490 | tr\|G3N515\|G3N515\_GASAC | 38.85 | 0 | 2 | 0 | N | 451257 | Uncharacterized protein OS=Gasterosteus aculeatus OX=69293 PE=4 SV=1 |
| 46 | 67497 | tr\|Q4T444\|Q4T444\_TETNG | 38.85 | 0 | 2 | 0 | N | 1041680 | Chromosome undetermined SCAF9829, whole genome shotgun sequence (Fragment) OS=Tetraodon nigroviridis OX=99883 GN=GSTENG00007498001 PE=4 SV=1 |
| 75 | 67581 | tr\|A0A6P7IXE2\|A0A6P7IXE2\_9TELE | 37.39 | 4 | 1 | 1 | N | 48050 | keratin, type I cytoskeletal 13-like OS=Parambassis ranga OX=210632 GN=LOC114440068 PE=4 SV=1 |
| 53 | 67795 | tr\|A0A3Q1HW42\|A0A3Q1HW42\_9TELE | 36.31 | 7 | 2 | 2 | N | 21205 | Adenylate kinase isoenzyme 1 OS=Acanthochromis polyacanthus OX=80966 GN=AK1 PE=3 SV=1 |
| 53 | 67796 | tr\|A0A3B5BIY5\|A0A3B5BIY5\_9TELE | 36.31 | 7 | 2 | 2 | N | 21206 | Adenylate kinase isoenzyme 1 OS=Stegastes partitus OX=144197 GN=AK1 PE=3 SV=1 |
| 53 | 67800 | tr\|A0A3Q4GK85\|A0A3Q4GK85\_NEOBR | 36.31 | 7 | 2 | 2 | N | 21230 | Adenylate kinase isoenzyme 1 OS=Neolamprologus brichardi OX=32507 GN=AK1 PE=3 SV=1 |
| 53 | 67801 | tr\|A0A3Q4GS43\|A0A3Q4GS43\_NEOBR | 36.31 | 7 | 2 | 2 | N | 21240 | Adenylate kinase isoenzyme 1 OS=Neolamprologus brichardi OX=32507 GN=AK1 PE=3 SV=1 |
| 53 | 67802 | tr\|A0A3P8P8S3\|A0A3P8P8S3\_ASTCA | 36.31 | 7 | 2 | 2 | N | 21228 | Adenylate kinase isoenzyme 1 OS=Astatotilapia calliptera OX=8154 GN=AK1 PE=3 SV=1 |
| 53 | 67803 | tr\|A0A3P9CWL7\|A0A3P9CWL7\_9CICH | 36.31 | 7 | 2 | 2 | N | 21258 | Adenylate kinase isoenzyme 1 OS=Maylandia zebra OX=106582 GN=AK1 PE=3 SV=1 |
| 53 | 67804 | tr\|A0A3Q2VY92\|A0A3Q2VY92\_HAPBU | 36.31 | 7 | 2 | 2 | N | 21228 | Adenylate kinase isoenzyme 1 OS=Haplochromis burtoni OX=8153 GN=AK1 PE=3 SV=1 |
| 53 | 67805 | tr\|A0A3B4FMK3\|A0A3B4FMK3\_9CICH | 36.31 | 7 | 2 | 2 | N | 21228 | Adenylate kinase isoenzyme 1 OS=Pundamilia nyererei OX=303518 GN=AK1 PE=3 SV=1 |
| 53 | 67806 | tr\|A0A3Q3CWR9\|A0A3Q3CWR9\_HAPBU | 36.31 | 6 | 2 | 2 | N | 23163 | Adenylate kinase isoenzyme 1 OS=Haplochromis burtoni OX=8153 GN=AK1 PE=3 SV=1 |
| 53 | 67807 | tr\|A0A3P8P8V3\|A0A3P8P8V3\_ASTCA | 36.31 | 6 | 2 | 2 | N | 23216 | Adenylate kinase isoenzyme 1 OS=Astatotilapia calliptera OX=8154 GN=AK1 PE=3 SV=1 |
| 44 | 67549 | tr\|A0A3Q3WSG6\|A0A3Q3WSG6\_MOLML | 33.08 | 7 | 2 | 1 | N | 35991 | Glyceraldehyde-3-phosphate dehydrogenase OS=Mola mola OX=94237 PE=3 SV=1 |
| 43 | 67850 | tr\|A0A6P7K206\|A0A6P7K206\_9TELE | 23.54 | 6 | 2 | 0 | N | 35440 | Glyceraldehyde-3-phosphate dehydrogenase OS=Parambassis ranga OX=210632 GN=LOC114449667 PE=3 SV=1 |
| 43 | 67852 | tr\|F5BZT7\|F5BZT7\_EPIBR | 23.54 | 10 | 2 | 0 | N | 21481 | Glyceraldehyde 3-phosphate dehydrogenase (Fragment) OS=Epinephelus bruneus OX=323802 PE=2 SV=1 |
| 43 | 67876 | tr\|A0A076U5J2\|A0A076U5J2\_SALFO | 23.54 | 12 | 2 | 0 | N | 17964 | Glyceraldehyde 3-phosphate dehydrogenase (Fragment) OS=Salvelinus fontinalis OX=8038 GN=GAPDH PE=2 SV=1 |
| 76 | 67808 | tr\|A0A1A8SH28\|A0A1A8SH28\_9TELE | 23.48 | 1 | 1 | 1 | N | 120560 | Collagen, type XI, alpha 1a (Fragment) OS=Nothobranchius rachovii OX=451742 GN=COL11A1A PE=4 SV=1 |
| 76 | 67809 | tr\|A0A1A8AFF3\|A0A1A8AFF3\_NOTFU | 23.48 | 1 | 1 | 1 | N | 141734 | Collagen, type XI, alpha 1a (Fragment) OS=Nothobranchius furzeri OX=105023 GN=COL11A1A PE=4 SV=1 |
| 76 | 67810 | tr\|A0A6P6JF49\|A0A6P6JF49\_CARAU | 23.48 | 1 | 1 | 1 | N | 166733 | collagen alpha-1(XI) chain-like isoform X5 OS=Carassius auratus OX=7957 GN=LOC113042513 PE=4 SV=1 |
| 76 | 67811 | tr\|A0A1A7ZX47\|A0A1A7ZX47\_NOTFU | 23.48 | 1 | 1 | 1 | N | 169593 | Collagen, type XI, alpha 1a OS=Nothobranchius furzeri OX=105023 GN=COL11A1A PE=4 SV=1 |
| 76 | 67812 | tr\|A0A6P6JAI8\|A0A6P6JAI8\_CARAU | 23.48 | 1 | 1 | 1 | N | 169942 | collagen alpha-1(XI) chain-like isoform X4 OS=Carassius auratus OX=7957 GN=LOC113042513 PE=4 SV=1 |
| 76 | 67813 | tr\|A0A1A8VA48\|A0A1A8VA48\_NOTFU | 23.48 | 1 | 1 | 1 | N | 167925 | Collagen, type XI, alpha 1a OS=Nothobranchius furzeri OX=105023 GN=COL11A1A PE=4 SV=1 |
| 76 | 67814 | tr\|A0A6P6JA06\|A0A6P6JA06\_CARAU | 23.48 | 1 | 1 | 1 | N | 172657 | collagen alpha-1(XI) chain-like isoform X3 OS=Carassius auratus OX=7957 GN=LOC113042513 PE=4 SV=1 |
| 76 | 67815 | tr\|A0A6P6J9V5\|A0A6P6J9V5\_CARAU | 23.48 | 1 | 1 | 1 | N | 177532 | collagen alpha-1(XI) chain-like isoform X2 OS=Carassius auratus OX=7957 GN=LOC113042513 PE=4 SV=1 |
| 76 | 67816 | tr\|A0A1A8S1T5\|A0A1A8S1T5\_9TELE | 23.48 | 1 | 1 | 1 | N | 179570 | Collagen, type XI, alpha 1a OS=Nothobranchius rachovii OX=451742 GN=COL11A1A PE=4 SV=1 |
| 76 | 67817 | tr\|A0A6P6JCL5\|A0A6P6JCL5\_CARAU | 23.48 | 1 | 1 | 1 | N | 180741 | collagen alpha-1(XI) chain-like isoform X1 OS=Carassius auratus OX=7957 GN=LOC113042513 PE=4 SV=1 |
| 77 | 67825 | tr\|A0A3P8TNL0\|A0A3P8TNL0\_AMPPE | 22.74 | 1 | 1 | 1 | N | 151454 | ATP-binding cassette, sub-family C (CFTR/MRP), member 5 OS=Amphiprion percula OX=161767 PE=4 SV=1 |
| 59 | 67827 | tr\|A0A6P6M1S2\|A0A6P6M1S2\_CARAU | 22.02 | 1 | 1 | 1 | N | 109031 | ankycorbin-like isoform X2 OS=Carassius auratus OX=7957 GN=LOC113064207 PE=4 SV=1 |
| 58 | 67890 | tr\|A0A315V5V2\|A0A315V5V2\_GAMAF | 21.66 | 1 | 1 | 1 | N | 130242 | Uncharacterized protein OS=Gambusia affinis OX=33528 GN=CCH79\_00005593 PE=4 SV=1 |
| 78 | 67958 | tr\|A0A3P8PXL3\|A0A3P8PXL3\_ASTCA | 21.04 | 1 | 1 | 1 | N | 126408 | SEC24 homolog A, COPII coat complex component OS=Astatotilapia calliptera OX=8154 PE=3 SV=1 |
| 61 | 67894 | tr\|A0A674A9W5\|A0A674A9W5\_SALTR | 20.23 | 3 | 1 | 1 | N | 39767 | Fructose-bisphosphate aldolase OS=Salmo trutta OX=8032 GN=ALDOA PE=3 SV=1 |
| total 327 proteins |  |  |  |  |  |  |  |  |  |
| --- | --- | --- | --- | --- | --- | --- | --- | --- | --- |

  

tr|A0A3B4T4B1|A0A3B4T4B1\_SERDU

back to list

  

| Protein Coverage
| Supporting Peptides
|

Protein Coverage:

Supporting Peptides:

| Peptide | Uniq | -10lgP | Mass | ppm | m/z | z | RT | Scan | #Spec | Start | End | PTM |
| --- | --- | --- | --- | --- | --- | --- | --- | --- | --- | --- | --- | --- |
| S.LEKSYELPDGQVITIGNE.R | Y | 62.40 | 2004.0051 | -0.7 | 1003.0091 | 2 | 13.51 | 2043 | 1 | 235 | 252 |  |
| S.LEKSYELPDGQVITIG.N | Y | 47.00 | 1760.9196 | -0.2 | 881.4669 | 2 | 13.63 | 2063 | 2 | 235 | 250 |  |
| E.KSYELPDGQVITIGNE.R | Y | 42.86 | 1761.8784 | -1.3 | 881.9453 | 2 | 13.39 | 2022 | 2 | 237 | 252 |  |
| L.EKSYELPDGQVITIG.N | Y | 40.62 | 1647.8356 | -0.1 | 824.9250 | 2 | 13.49 | 2040 | 1 | 236 | 250 |  |
| G.IVLDAGDGVTH.N | Y | 40.00 | 1095.5560 | 0.0 | 548.7853 | 2 | 11.28 | 1650 | 1 | 150 | 160 |  |
| A.GFAGDDAPRA.V | N | 39.52 | 975.4410 | -0.2 | 488.7277 | 2 | 10.22 | 1469 | 4 | 19 | 28 |  |
| D.LAGRDLTDYLMK.I | Y | 35.48 | 1394.7228 | -0.4 | 698.3684 | 2 | 12.53 | 1874 | 1 | 179 | 190 |  |
| L.EKSYELPDGQVIT.I | Y | 34.60 | 1477.7300 | -1.0 | 739.8715 | 2 | 12.16 | 1807 | 1 | 236 | 248 |  |
| E.KSYELPDGQ.V | Y | 29.74 | 1035.4873 | 4.6 | 518.7533 | 2 | 10.72 | 1551 | 1 | 237 | 245 |  |
| L.DAGDGVTH.N | Y | 29.15 | 770.3195 | 0.0 | 386.1670 | 2 | 8.66 | 1223 | 3 | 153 | 160 |  |
| E.KSYELPDGQVITIG.N | Y | 29.00 | 1518.7930 | -0.3 | 760.4036 | 2 | 13.49 | 2039 | 2 | 237 | 250 |  |
| E.KSYELPDGQVIT.I | Y | 28.67 | 1348.6874 | -0.9 | 675.3503 | 2 | 11.98 | 1776 | 1 | 237 | 248 |  |
| S.LEKSYELPDGQVI.T | Y | 28.05 | 1489.7664 | -1.6 | 745.8893 | 2 | 12.81 | 1923 | 1 | 235 | 247 |  |
| Y.VGDEAQSK.R | N | 26.03 | 832.3926 | 0.0 | 417.2036 | 2 | 8.09 | 1115 | 1 | 53 | 60 |  |
| S.LEKSYELPDGQVITI.G | Y | 22.10 | 1703.8981 | 1.1 | 852.9572 | 2 | 13.95 | 2115 | 1 | 235 | 249 |  |
| L.DLAGRDLTDYLM.K | Y | 21.76 | 1381.6548 | 0.3 | 691.8349 | 2 | 14.01 | 2125 | 1 | 178 | 189 |  |
| T.MYPGIADRM.Q | Y | 19.69 | 1052.4783 | -1.2 | 527.2458 | 2 | 11.85 | 1751 | 1 | 304 | 312 |  |
| R.VAPEEHPTL.L | Y | 19.35 | 991.4974 | 0.6 | 496.7563 | 2 | 10.69 | 1547 | 1 | 95 | 103 |  |
| G.IITNWDDMEK.I | Y | 19.16 | 1263.5806 | -1.2 | 632.7968 | 2 | 12.15 | 1805 | 1 | 74 | 83 |  |
| G.FAGDDAPRA.V | N | 18.34 | 918.4195 | 0.1 | 460.2171 | 2 | 17.21 | 2654 | 7 | 20 | 28 |  |
| S.LEKSYELPDGQ.V | Y | 16.48 | 1277.6139 | -0.9 | 639.8137 | 2 | 11.17 | 1631 | 1 | 235 | 245 |  |
| L.DLAGRDLTDYLMK.I | Y | 15.77 | 1509.7498 | -1.3 | 755.8812 | 2 | 13.04 | 1962 | 1 | 178 | 190 |  |
| L.EKSYELPDGQVITI.G | Y | 15.45 | 1590.8141 | -0.6 | 796.4139 | 2 | 13.72 | 2078 | 1 | 236 | 249 |  |
| total 23 peptides |  |  |  |  |  |  |  |  |  |  |  |  |
| --- | --- | --- | --- | --- | --- | --- | --- | --- | --- | --- | --- | --- |

tr|A0A3Q3BBJ2|A0A3Q3BBJ2\_KRYMA

back to list

  

| Protein Coverage
| Supporting Peptides
|

Protein Coverage:

Supporting Peptides:

| Peptide | Uniq | -10lgP | Mass | ppm | m/z | z | RT | Scan | #Spec | Start | End | PTM |
| --- | --- | --- | --- | --- | --- | --- | --- | --- | --- | --- | --- | --- |
| S.LEKSYELPDGQVITIGNE.R | Y | 62.40 | 2004.0051 | -0.7 | 1003.0091 | 2 | 13.51 | 2043 | 1 | 238 | 255 |  |
| S.LEKSYELPDGQVITIG.N | Y | 47.00 | 1760.9196 | -0.2 | 881.4669 | 2 | 13.63 | 2063 | 2 | 238 | 253 |  |
| E.KSYELPDGQVITIGNE.R | Y | 42.86 | 1761.8784 | -1.3 | 881.9453 | 2 | 13.39 | 2022 | 2 | 240 | 255 |  |
| L.EKSYELPDGQVITIG.N | Y | 40.62 | 1647.8356 | -0.1 | 824.9250 | 2 | 13.49 | 2040 | 1 | 239 | 253 |  |
| G.IVLDAGDGVTH.N | Y | 40.00 | 1095.5560 | 0.0 | 548.7853 | 2 | 11.28 | 1650 | 1 | 153 | 163 |  |
| A.GFAGDDAPRA.V | N | 39.52 | 975.4410 | -0.2 | 488.7277 | 2 | 10.22 | 1469 | 4 | 22 | 31 |  |
| D.LAGRDLTDYLMK.I | Y | 35.48 | 1394.7228 | -0.4 | 698.3684 | 2 | 12.53 | 1874 | 1 | 182 | 193 |  |
| L.EKSYELPDGQVIT.I | Y | 34.60 | 1477.7300 | -1.0 | 739.8715 | 2 | 12.16 | 1807 | 1 | 239 | 251 |  |
| E.KSYELPDGQ.V | Y | 29.74 | 1035.4873 | 4.6 | 518.7533 | 2 | 10.72 | 1551 | 1 | 240 | 248 |  |
| L.DAGDGVTH.N | Y | 29.15 | 770.3195 | 0.0 | 386.1670 | 2 | 8.66 | 1223 | 3 | 156 | 163 |  |
| E.KSYELPDGQVITIG.N | Y | 29.00 | 1518.7930 | -0.3 | 760.4036 | 2 | 13.49 | 2039 | 2 | 240 | 253 |  |
| E.KSYELPDGQVIT.I | Y | 28.67 | 1348.6874 | -0.9 | 675.3503 | 2 | 11.98 | 1776 | 1 | 240 | 251 |  |
| S.LEKSYELPDGQVI.T | Y | 28.05 | 1489.7664 | -1.6 | 745.8893 | 2 | 12.81 | 1923 | 1 | 238 | 250 |  |
| Y.VGDEAQSK.R | N | 26.03 | 832.3926 | 0.0 | 417.2036 | 2 | 8.09 | 1115 | 1 | 56 | 63 |  |
| S.LEKSYELPDGQVITI.G | Y | 22.10 | 1703.8981 | 1.1 | 852.9572 | 2 | 13.95 | 2115 | 1 | 238 | 252 |  |
| L.DLAGRDLTDYLM.K | Y | 21.76 | 1381.6548 | 0.3 | 691.8349 | 2 | 14.01 | 2125 | 1 | 181 | 192 |  |
| T.MYPGIADRM.Q | Y | 19.69 | 1052.4783 | -1.2 | 527.2458 | 2 | 11.85 | 1751 | 1 | 307 | 315 |  |
| R.VAPEEHPTL.L | Y | 19.35 | 991.4974 | 0.6 | 496.7563 | 2 | 10.69 | 1547 | 1 | 98 | 106 |  |
| G.IITNWDDMEK.I | Y | 19.16 | 1263.5806 | -1.2 | 632.7968 | 2 | 12.15 | 1805 | 1 | 77 | 86 |  |
| G.FAGDDAPRA.V | N | 18.34 | 918.4195 | 0.1 | 460.2171 | 2 | 17.21 | 2654 | 7 | 23 | 31 |  |
| S.LEKSYELPDGQ.V | Y | 16.48 | 1277.6139 | -0.9 | 639.8137 | 2 | 11.17 | 1631 | 1 | 238 | 248 |  |
| L.DLAGRDLTDYLMK.I | Y | 15.77 | 1509.7498 | -1.3 | 755.8812 | 2 | 13.04 | 1962 | 1 | 181 | 193 |  |
| L.EKSYELPDGQVITI.G | Y | 15.45 | 1590.8141 | -0.6 | 796.4139 | 2 | 13.72 | 2078 | 1 | 239 | 252 |  |
| total 23 peptides |  |  |  |  |  |  |  |  |  |  |  |  |
| --- | --- | --- | --- | --- | --- | --- | --- | --- | --- | --- | --- | --- |

tr|Q76MZ4|Q76MZ4\_9TELE

back to list

  

| Protein Coverage
| Supporting Peptides
|

Protein Coverage:

Supporting Peptides:

| Peptide | Uniq | -10lgP | Mass | ppm | m/z | z | RT | Scan | #Spec | Start | End | PTM |
| --- | --- | --- | --- | --- | --- | --- | --- | --- | --- | --- | --- | --- |
| S.LEKSYELPDGQVITIGNE.R | Y | 62.40 | 2004.0051 | -0.7 | 1003.0091 | 2 | 13.51 | 2043 | 1 | 238 | 255 |  |
| S.LEKSYELPDGQVITIG.N | Y | 47.00 | 1760.9196 | -0.2 | 881.4669 | 2 | 13.63 | 2063 | 2 | 238 | 253 |  |
| E.KSYELPDGQVITIGNE.R | Y | 42.86 | 1761.8784 | -1.3 | 881.9453 | 2 | 13.39 | 2022 | 2 | 240 | 255 |  |
| L.EKSYELPDGQVITIG.N | Y | 40.62 | 1647.8356 | -0.1 | 824.9250 | 2 | 13.49 | 2040 | 1 | 239 | 253 |  |
| G.IVLDAGDGVTH.N | Y | 40.00 | 1095.5560 | 0.0 | 548.7853 | 2 | 11.28 | 1650 | 1 | 153 | 163 |  |
| A.GFAGDDAPRA.V | N | 39.52 | 975.4410 | -0.2 | 488.7277 | 2 | 10.22 | 1469 | 4 | 22 | 31 |  |
| D.LAGRDLTDYLMK.I | Y | 35.48 | 1394.7228 | -0.4 | 698.3684 | 2 | 12.53 | 1874 | 1 | 182 | 193 |  |
| L.EKSYELPDGQVIT.I | Y | 34.60 | 1477.7300 | -1.0 | 739.8715 | 2 | 12.16 | 1807 | 1 | 239 | 251 |  |
| E.KSYELPDGQ.V | Y | 29.74 | 1035.4873 | 4.6 | 518.7533 | 2 | 10.72 | 1551 | 1 | 240 | 248 |  |
| L.DAGDGVTH.N | Y | 29.15 | 770.3195 | 0.0 | 386.1670 | 2 | 8.66 | 1223 | 3 | 156 | 163 |  |
| E.KSYELPDGQVITIG.N | Y | 29.00 | 1518.7930 | -0.3 | 760.4036 | 2 | 13.49 | 2039 | 2 | 240 | 253 |  |
| E.KSYELPDGQVIT.I | Y | 28.67 | 1348.6874 | -0.9 | 675.3503 | 2 | 11.98 | 1776 | 1 | 240 | 251 |  |
| S.LEKSYELPDGQVI.T | Y | 28.05 | 1489.7664 | -1.6 | 745.8893 | 2 | 12.81 | 1923 | 1 | 238 | 250 |  |
| Y.VGDEAQSK.R | N | 26.03 | 832.3926 | 0.0 | 417.2036 | 2 | 8.09 | 1115 | 1 | 56 | 63 |  |
| S.LEKSYELPDGQVITI.G | Y | 22.10 | 1703.8981 | 1.1 | 852.9572 | 2 | 13.95 | 2115 | 1 | 238 | 252 |  |
| L.DLAGRDLTDYLM.K | Y | 21.76 | 1381.6548 | 0.3 | 691.8349 | 2 | 14.01 | 2125 | 1 | 181 | 192 |  |
| T.MYPGIADRM.Q | Y | 19.69 | 1052.4783 | -1.2 | 527.2458 | 2 | 11.85 | 1751 | 1 | 307 | 315 |  |
| R.VAPEEHPTL.L | Y | 19.35 | 991.4974 | 0.6 | 496.7563 | 2 | 10.69 | 1547 | 1 | 98 | 106 |  |
| G.IITNWDDMEK.I | Y | 19.16 | 1263.5806 | -1.2 | 632.7968 | 2 | 12.15 | 1805 | 1 | 77 | 86 |  |
| G.FAGDDAPRA.V | N | 18.34 | 918.4195 | 0.1 | 460.2171 | 2 | 17.21 | 2654 | 7 | 23 | 31 |  |
| S.LEKSYELPDGQ.V | Y | 16.48 | 1277.6139 | -0.9 | 639.8137 | 2 | 11.17 | 1631 | 1 | 238 | 248 |  |
| L.DLAGRDLTDYLMK.I | Y | 15.77 | 1509.7498 | -1.3 | 755.8812 | 2 | 13.04 | 1962 | 1 | 181 | 193 |  |
| L.EKSYELPDGQVITI.G | Y | 15.45 | 1590.8141 | -0.6 | 796.4139 | 2 | 13.72 | 2078 | 1 | 239 | 252 |  |
| total 23 peptides |  |  |  |  |  |  |  |  |  |  |  |  |
| --- | --- | --- | --- | --- | --- | --- | --- | --- | --- | --- | --- | --- |

tr|A0A3S2MUF9|A0A3S2MUF9\_ORYJA

back to list

  

| Protein Coverage
| Supporting Peptides
|

Protein Coverage:

Supporting Peptides:

| Peptide | Uniq | -10lgP | Mass | ppm | m/z | z | RT | Scan | #Spec | Start | End | PTM |
| --- | --- | --- | --- | --- | --- | --- | --- | --- | --- | --- | --- | --- |
| S.LEKSYELPDGQVITIGNE.R | Y | 62.40 | 2004.0051 | -0.7 | 1003.0091 | 2 | 13.51 | 2043 | 1 | 238 | 255 |  |
| S.LEKSYELPDGQVITIG.N | Y | 47.00 | 1760.9196 | -0.2 | 881.4669 | 2 | 13.63 | 2063 | 2 | 238 | 253 |  |
| E.KSYELPDGQVITIGNE.R | Y | 42.86 | 1761.8784 | -1.3 | 881.9453 | 2 | 13.39 | 2022 | 2 | 240 | 255 |  |
| L.EKSYELPDGQVITIG.N | Y | 40.62 | 1647.8356 | -0.1 | 824.9250 | 2 | 13.49 | 2040 | 1 | 239 | 253 |  |
| G.IVLDAGDGVTH.N | Y | 40.00 | 1095.5560 | 0.0 | 548.7853 | 2 | 11.28 | 1650 | 1 | 153 | 163 |  |
| A.GFAGDDAPRA.V | N | 39.52 | 975.4410 | -0.2 | 488.7277 | 2 | 10.22 | 1469 | 4 | 22 | 31 |  |
| D.LAGRDLTDYLMK.I | Y | 35.48 | 1394.7228 | -0.4 | 698.3684 | 2 | 12.53 | 1874 | 1 | 182 | 193 |  |
| L.EKSYELPDGQVIT.I | Y | 34.60 | 1477.7300 | -1.0 | 739.8715 | 2 | 12.16 | 1807 | 1 | 239 | 251 |  |
| E.KSYELPDGQ.V | Y | 29.74 | 1035.4873 | 4.6 | 518.7533 | 2 | 10.72 | 1551 | 1 | 240 | 248 |  |
| L.DAGDGVTH.N | Y | 29.15 | 770.3195 | 0.0 | 386.1670 | 2 | 8.66 | 1223 | 3 | 156 | 163 |  |
| E.KSYELPDGQVITIG.N | Y | 29.00 | 1518.7930 | -0.3 | 760.4036 | 2 | 13.49 | 2039 | 2 | 240 | 253 |  |
| E.KSYELPDGQVIT.I | Y | 28.67 | 1348.6874 | -0.9 | 675.3503 | 2 | 11.98 | 1776 | 1 | 240 | 251 |  |
| S.LEKSYELPDGQVI.T | Y | 28.05 | 1489.7664 | -1.6 | 745.8893 | 2 | 12.81 | 1923 | 1 | 238 | 250 |  |
| Y.VGDEAQSK.R | N | 26.03 | 832.3926 | 0.0 | 417.2036 | 2 | 8.09 | 1115 | 1 | 56 | 63 |  |
| S.LEKSYELPDGQVITI.G | Y | 22.10 | 1703.8981 | 1.1 | 852.9572 | 2 | 13.95 | 2115 | 1 | 238 | 252 |  |
| L.DLAGRDLTDYLM.K | Y | 21.76 | 1381.6548 | 0.3 | 691.8349 | 2 | 14.01 | 2125 | 1 | 181 | 192 |  |
| T.MYPGIADRM.Q | Y | 19.69 | 1052.4783 | -1.2 | 527.2458 | 2 | 11.85 | 1751 | 1 | 307 | 315 |  |
| R.VAPEEHPTL.L | Y | 19.35 | 991.4974 | 0.6 | 496.7563 | 2 | 10.69 | 1547 | 1 | 98 | 106 |  |
| G.IITNWDDMEK.I | Y | 19.16 | 1263.5806 | -1.2 | 632.7968 | 2 | 12.15 | 1805 | 1 | 77 | 86 |  |
| G.FAGDDAPRA.V | N | 18.34 | 918.4195 | 0.1 | 460.2171 | 2 | 17.21 | 2654 | 7 | 23 | 31 |  |
| S.LEKSYELPDGQ.V | Y | 16.48 | 1277.6139 | -0.9 | 639.8137 | 2 | 11.17 | 1631 | 1 | 238 | 248 |  |
| L.DLAGRDLTDYLMK.I | Y | 15.77 | 1509.7498 | -1.3 | 755.8812 | 2 | 13.04 | 1962 | 1 | 181 | 193 |  |
| L.EKSYELPDGQVITI.G | Y | 15.45 | 1590.8141 | -0.6 | 796.4139 | 2 | 13.72 | 2078 | 1 | 239 | 252 |  |
| total 23 peptides |  |  |  |  |  |  |  |  |  |  |  |  |
| --- | --- | --- | --- | --- | --- | --- | --- | --- | --- | --- | --- | --- |

tr|A0A3Q0RI14|A0A3Q0RI14\_AMPCI

back to list

  

| Protein Coverage
| Supporting Peptides
|

Protein Coverage:

Supporting Peptides:

| Peptide | Uniq | -10lgP | Mass | ppm | m/z | z | RT | Scan | #Spec | Start | End | PTM |
| --- | --- | --- | --- | --- | --- | --- | --- | --- | --- | --- | --- | --- |
| S.LEKSYELPDGQVITIGNE.R | Y | 62.40 | 2004.0051 | -0.7 | 1003.0091 | 2 | 13.51 | 2043 | 1 | 238 | 255 |  |
| S.LEKSYELPDGQVITIG.N | Y | 47.00 | 1760.9196 | -0.2 | 881.4669 | 2 | 13.63 | 2063 | 2 | 238 | 253 |  |
| E.KSYELPDGQVITIGNE.R | Y | 42.86 | 1761.8784 | -1.3 | 881.9453 | 2 | 13.39 | 2022 | 2 | 240 | 255 |  |
| L.EKSYELPDGQVITIG.N | Y | 40.62 | 1647.8356 | -0.1 | 824.9250 | 2 | 13.49 | 2040 | 1 | 239 | 253 |  |
| G.IVLDAGDGVTH.N | Y | 40.00 | 1095.5560 | 0.0 | 548.7853 | 2 | 11.28 | 1650 | 1 | 153 | 163 |  |
| A.GFAGDDAPRA.V | N | 39.52 | 975.4410 | -0.2 | 488.7277 | 2 | 10.22 | 1469 | 4 | 22 | 31 |  |
| D.LAGRDLTDYLMK.I | Y | 35.48 | 1394.7228 | -0.4 | 698.3684 | 2 | 12.53 | 1874 | 1 | 182 | 193 |  |
| L.EKSYELPDGQVIT.I | Y | 34.60 | 1477.7300 | -1.0 | 739.8715 | 2 | 12.16 | 1807 | 1 | 239 | 251 |  |
| E.KSYELPDGQ.V | Y | 29.74 | 1035.4873 | 4.6 | 518.7533 | 2 | 10.72 | 1551 | 1 | 240 | 248 |  |
| L.DAGDGVTH.N | Y | 29.15 | 770.3195 | 0.0 | 386.1670 | 2 | 8.66 | 1223 | 3 | 156 | 163 |  |
| E.KSYELPDGQVITIG.N | Y | 29.00 | 1518.7930 | -0.3 | 760.4036 | 2 | 13.49 | 2039 | 2 | 240 | 253 |  |
| E.KSYELPDGQVIT.I | Y | 28.67 | 1348.6874 | -0.9 | 675.3503 | 2 | 11.98 | 1776 | 1 | 240 | 251 |  |
| S.LEKSYELPDGQVI.T | Y | 28.05 | 1489.7664 | -1.6 | 745.8893 | 2 | 12.81 | 1923 | 1 | 238 | 250 |  |
| Y.VGDEAQSK.R | N | 26.03 | 832.3926 | 0.0 | 417.2036 | 2 | 8.09 | 1115 | 1 | 56 | 63 |  |
| S.LEKSYELPDGQVITI.G | Y | 22.10 | 1703.8981 | 1.1 | 852.9572 | 2 | 13.95 | 2115 | 1 | 238 | 252 |  |
| L.DLAGRDLTDYLM.K | Y | 21.76 | 1381.6548 | 0.3 | 691.8349 | 2 | 14.01 | 2125 | 1 | 181 | 192 |  |
| T.MYPGIADRM.Q | Y | 19.69 | 1052.4783 | -1.2 | 527.2458 | 2 | 11.85 | 1751 | 1 | 307 | 315 |  |
| R.VAPEEHPTL.L | Y | 19.35 | 991.4974 | 0.6 | 496.7563 | 2 | 10.69 | 1547 | 1 | 98 | 106 |  |
| G.IITNWDDMEK.I | Y | 19.16 | 1263.5806 | -1.2 | 632.7968 | 2 | 12.15 | 1805 | 1 | 77 | 86 |  |
| G.FAGDDAPRA.V | N | 18.34 | 918.4195 | 0.1 | 460.2171 | 2 | 17.21 | 2654 | 7 | 23 | 31 |  |
| S.LEKSYELPDGQ.V | Y | 16.48 | 1277.6139 | -0.9 | 639.8137 | 2 | 11.17 | 1631 | 1 | 238 | 248 |  |
| L.DLAGRDLTDYLMK.I | Y | 15.77 | 1509.7498 | -1.3 | 755.8812 | 2 | 13.04 | 1962 | 1 | 181 | 193 |  |
| L.EKSYELPDGQVITI.G | Y | 15.45 | 1590.8141 | -0.6 | 796.4139 | 2 | 13.72 | 2078 | 1 | 239 | 252 |  |
| total 23 peptides |  |  |  |  |  |  |  |  |  |  |  |  |
| --- | --- | --- | --- | --- | --- | --- | --- | --- | --- | --- | --- | --- |

tr|M4AKR3|M4AKR3\_XIPMA

back to list

  

| Protein Coverage
| Supporting Peptides
|

Protein Coverage:

Supporting Peptides:

| Peptide | Uniq | -10lgP | Mass | ppm | m/z | z | RT | Scan | #Spec | Start | End | PTM |
| --- | --- | --- | --- | --- | --- | --- | --- | --- | --- | --- | --- | --- |
| S.LEKSYELPDGQVITIGNE.R | Y | 62.40 | 2004.0051 | -0.7 | 1003.0091 | 2 | 13.51 | 2043 | 1 | 238 | 255 |  |
| S.LEKSYELPDGQVITIG.N | Y | 47.00 | 1760.9196 | -0.2 | 881.4669 | 2 | 13.63 | 2063 | 2 | 238 | 253 |  |
| E.KSYELPDGQVITIGNE.R | Y | 42.86 | 1761.8784 | -1.3 | 881.9453 | 2 | 13.39 | 2022 | 2 | 240 | 255 |  |
| L.EKSYELPDGQVITIG.N | Y | 40.62 | 1647.8356 | -0.1 | 824.9250 | 2 | 13.49 | 2040 | 1 | 239 | 253 |  |
| G.IVLDAGDGVTH.N | Y | 40.00 | 1095.5560 | 0.0 | 548.7853 | 2 | 11.28 | 1650 | 1 | 153 | 163 |  |
| A.GFAGDDAPRA.V | N | 39.52 | 975.4410 | -0.2 | 488.7277 | 2 | 10.22 | 1469 | 4 | 22 | 31 |  |
| D.LAGRDLTDYLMK.I | Y | 35.48 | 1394.7228 | -0.4 | 698.3684 | 2 | 12.53 | 1874 | 1 | 182 | 193 |  |
| L.EKSYELPDGQVIT.I | Y | 34.60 | 1477.7300 | -1.0 | 739.8715 | 2 | 12.16 | 1807 | 1 | 239 | 251 |  |
| E.KSYELPDGQ.V | Y | 29.74 | 1035.4873 | 4.6 | 518.7533 | 2 | 10.72 | 1551 | 1 | 240 | 248 |  |
| L.DAGDGVTH.N | Y | 29.15 | 770.3195 | 0.0 | 386.1670 | 2 | 8.66 | 1223 | 3 | 156 | 163 |  |
| E.KSYELPDGQVITIG.N | Y | 29.00 | 1518.7930 | -0.3 | 760.4036 | 2 | 13.49 | 2039 | 2 | 240 | 253 |  |
| E.KSYELPDGQVIT.I | Y | 28.67 | 1348.6874 | -0.9 | 675.3503 | 2 | 11.98 | 1776 | 1 | 240 | 251 |  |
| S.LEKSYELPDGQVI.T | Y | 28.05 | 1489.7664 | -1.6 | 745.8893 | 2 | 12.81 | 1923 | 1 | 238 | 250 |  |
| Y.VGDEAQSK.R | N | 26.03 | 832.3926 | 0.0 | 417.2036 | 2 | 8.09 | 1115 | 1 | 56 | 63 |  |
| S.LEKSYELPDGQVITI.G | Y | 22.10 | 1703.8981 | 1.1 | 852.9572 | 2 | 13.95 | 2115 | 1 | 238 | 252 |  |
| L.DLAGRDLTDYLM.K | Y | 21.76 | 1381.6548 | 0.3 | 691.8349 | 2 | 14.01 | 2125 | 1 | 181 | 192 |  |
| T.MYPGIADRM.Q | Y | 19.69 | 1052.4783 | -1.2 | 527.2458 | 2 | 11.85 | 1751 | 1 | 307 | 315 |  |
| R.VAPEEHPTL.L | Y | 19.35 | 991.4974 | 0.6 | 496.7563 | 2 | 10.69 | 1547 | 1 | 98 | 106 |  |
| G.IITNWDDMEK.I | Y | 19.16 | 1263.5806 | -1.2 | 632.7968 | 2 | 12.15 | 1805 | 1 | 77 | 86 |  |
| G.FAGDDAPRA.V | N | 18.34 | 918.4195 | 0.1 | 460.2171 | 2 | 17.21 | 2654 | 7 | 23 | 31 |  |
| S.LEKSYELPDGQ.V | Y | 16.48 | 1277.6139 | -0.9 | 639.8137 | 2 | 11.17 | 1631 | 1 | 238 | 248 |  |
| L.DLAGRDLTDYLMK.I | Y | 15.77 | 1509.7498 | -1.3 | 755.8812 | 2 | 13.04 | 1962 | 1 | 181 | 193 |  |
| L.EKSYELPDGQVITI.G | Y | 15.45 | 1590.8141 | -0.6 | 796.4139 | 2 | 13.72 | 2078 | 1 | 239 | 252 |  |
| total 23 peptides |  |  |  |  |  |  |  |  |  |  |  |  |
| --- | --- | --- | --- | --- | --- | --- | --- | --- | --- | --- | --- | --- |

tr|C0LLE8|C0LLE8\_DICLA

back to list

  

| Protein Coverage
| Supporting Peptides
|

Protein Coverage:

Supporting Peptides:

| Peptide | Uniq | -10lgP | Mass | ppm | m/z | z | RT | Scan | #Spec | Start | End | PTM |
| --- | --- | --- | --- | --- | --- | --- | --- | --- | --- | --- | --- | --- |
| S.LEKSYELPDGQVITIGNE.R | Y | 62.40 | 2004.0051 | -0.7 | 1003.0091 | 2 | 13.51 | 2043 | 1 | 238 | 255 |  |
| S.LEKSYELPDGQVITIG.N | Y | 47.00 | 1760.9196 | -0.2 | 881.4669 | 2 | 13.63 | 2063 | 2 | 238 | 253 |  |
| E.KSYELPDGQVITIGNE.R | Y | 42.86 | 1761.8784 | -1.3 | 881.9453 | 2 | 13.39 | 2022 | 2 | 240 | 255 |  |
| L.EKSYELPDGQVITIG.N | Y | 40.62 | 1647.8356 | -0.1 | 824.9250 | 2 | 13.49 | 2040 | 1 | 239 | 253 |  |
| G.IVLDAGDGVTH.N | Y | 40.00 | 1095.5560 | 0.0 | 548.7853 | 2 | 11.28 | 1650 | 1 | 153 | 163 |  |
| A.GFAGDDAPRA.V | N | 39.52 | 975.4410 | -0.2 | 488.7277 | 2 | 10.22 | 1469 | 4 | 22 | 31 |  |
| D.LAGRDLTDYLMK.I | Y | 35.48 | 1394.7228 | -0.4 | 698.3684 | 2 | 12.53 | 1874 | 1 | 182 | 193 |  |
| L.EKSYELPDGQVIT.I | Y | 34.60 | 1477.7300 | -1.0 | 739.8715 | 2 | 12.16 | 1807 | 1 | 239 | 251 |  |
| E.KSYELPDGQ.V | Y | 29.74 | 1035.4873 | 4.6 | 518.7533 | 2 | 10.72 | 1551 | 1 | 240 | 248 |  |
| L.DAGDGVTH.N | Y | 29.15 | 770.3195 | 0.0 | 386.1670 | 2 | 8.66 | 1223 | 3 | 156 | 163 |  |
| E.KSYELPDGQVITIG.N | Y | 29.00 | 1518.7930 | -0.3 | 760.4036 | 2 | 13.49 | 2039 | 2 | 240 | 253 |  |
| E.KSYELPDGQVIT.I | Y | 28.67 | 1348.6874 | -0.9 | 675.3503 | 2 | 11.98 | 1776 | 1 | 240 | 251 |  |
| S.LEKSYELPDGQVI.T | Y | 28.05 | 1489.7664 | -1.6 | 745.8893 | 2 | 12.81 | 1923 | 1 | 238 | 250 |  |
| Y.VGDEAQSK.R | N | 26.03 | 832.3926 | 0.0 | 417.2036 | 2 | 8.09 | 1115 | 1 | 56 | 63 |  |
| S.LEKSYELPDGQVITI.G | Y | 22.10 | 1703.8981 | 1.1 | 852.9572 | 2 | 13.95 | 2115 | 1 | 238 | 252 |  |
| L.DLAGRDLTDYLM.K | Y | 21.76 | 1381.6548 | 0.3 | 691.8349 | 2 | 14.01 | 2125 | 1 | 181 | 192 |  |
| T.MYPGIADRM.Q | Y | 19.69 | 1052.4783 | -1.2 | 527.2458 | 2 | 11.85 | 1751 | 1 | 307 | 315 |  |
| R.VAPEEHPTL.L | Y | 19.35 | 991.4974 | 0.6 | 496.7563 | 2 | 10.69 | 1547 | 1 | 98 | 106 |  |
| G.IITNWDDMEK.I | Y | 19.16 | 1263.5806 | -1.2 | 632.7968 | 2 | 12.15 | 1805 | 1 | 77 | 86 |  |
| G.FAGDDAPRA.V | N | 18.34 | 918.4195 | 0.1 | 460.2171 | 2 | 17.21 | 2654 | 7 | 23 | 31 |  |
| S.LEKSYELPDGQ.V | Y | 16.48 | 1277.6139 | -0.9 | 639.8137 | 2 | 11.17 | 1631 | 1 | 238 | 248 |  |
| L.DLAGRDLTDYLMK.I | Y | 15.77 | 1509.7498 | -1.3 | 755.8812 | 2 | 13.04 | 1962 | 1 | 181 | 193 |  |
| L.EKSYELPDGQVITI.G | Y | 15.45 | 1590.8141 | -0.6 | 796.4139 | 2 | 13.72 | 2078 | 1 | 239 | 252 |  |
| total 23 peptides |  |  |  |  |  |  |  |  |  |  |  |  |
| --- | --- | --- | --- | --- | --- | --- | --- | --- | --- | --- | --- | --- |

tr|A0A3Q3N432|A0A3Q3N432\_9TELE

back to list

  

| Protein Coverage
| Supporting Peptides
|

Protein Coverage:

Supporting Peptides:

| Peptide | Uniq | -10lgP | Mass | ppm | m/z | z | RT | Scan | #Spec | Start | End | PTM |
| --- | --- | --- | --- | --- | --- | --- | --- | --- | --- | --- | --- | --- |
| S.LEKSYELPDGQVITIGNE.R | Y | 62.40 | 2004.0051 | -0.7 | 1003.0091 | 2 | 13.51 | 2043 | 1 | 238 | 255 |  |
| S.LEKSYELPDGQVITIG.N | Y | 47.00 | 1760.9196 | -0.2 | 881.4669 | 2 | 13.63 | 2063 | 2 | 238 | 253 |  |
| E.KSYELPDGQVITIGNE.R | Y | 42.86 | 1761.8784 | -1.3 | 881.9453 | 2 | 13.39 | 2022 | 2 | 240 | 255 |  |
| L.EKSYELPDGQVITIG.N | Y | 40.62 | 1647.8356 | -0.1 | 824.9250 | 2 | 13.49 | 2040 | 1 | 239 | 253 |  |
| G.IVLDAGDGVTH.N | Y | 40.00 | 1095.5560 | 0.0 | 548.7853 | 2 | 11.28 | 1650 | 1 | 153 | 163 |  |
| A.GFAGDDAPRA.V | N | 39.52 | 975.4410 | -0.2 | 488.7277 | 2 | 10.22 | 1469 | 4 | 22 | 31 |  |
| D.LAGRDLTDYLMK.I | Y | 35.48 | 1394.7228 | -0.4 | 698.3684 | 2 | 12.53 | 1874 | 1 | 182 | 193 |  |
| L.EKSYELPDGQVIT.I | Y | 34.60 | 1477.7300 | -1.0 | 739.8715 | 2 | 12.16 | 1807 | 1 | 239 | 251 |  |
| E.KSYELPDGQ.V | Y | 29.74 | 1035.4873 | 4.6 | 518.7533 | 2 | 10.72 | 1551 | 1 | 240 | 248 |  |
| L.DAGDGVTH.N | Y | 29.15 | 770.3195 | 0.0 | 386.1670 | 2 | 8.66 | 1223 | 3 | 156 | 163 |  |
| E.KSYELPDGQVITIG.N | Y | 29.00 | 1518.7930 | -0.3 | 760.4036 | 2 | 13.49 | 2039 | 2 | 240 | 253 |  |
| E.KSYELPDGQVIT.I | Y | 28.67 | 1348.6874 | -0.9 | 675.3503 | 2 | 11.98 | 1776 | 1 | 240 | 251 |  |
| S.LEKSYELPDGQVI.T | Y | 28.05 | 1489.7664 | -1.6 | 745.8893 | 2 | 12.81 | 1923 | 1 | 238 | 250 |  |
| Y.VGDEAQSK.R | N | 26.03 | 832.3926 | 0.0 | 417.2036 | 2 | 8.09 | 1115 | 1 | 56 | 63 |  |
| S.LEKSYELPDGQVITI.G | Y | 22.10 | 1703.8981 | 1.1 | 852.9572 | 2 | 13.95 | 2115 | 1 | 238 | 252 |  |
| L.DLAGRDLTDYLM.K | Y | 21.76 | 1381.6548 | 0.3 | 691.8349 | 2 | 14.01 | 2125 | 1 | 181 | 192 |  |
| T.MYPGIADRM.Q | Y | 19.69 | 1052.4783 | -1.2 | 527.2458 | 2 | 11.85 | 1751 | 1 | 307 | 315 |  |
| R.VAPEEHPTL.L | Y | 19.35 | 991.4974 | 0.6 | 496.7563 | 2 | 10.69 | 1547 | 1 | 98 | 106 |  |
| G.IITNWDDMEK.I | Y | 19.16 | 1263.5806 | -1.2 | 632.7968 | 2 | 12.15 | 1805 | 1 | 77 | 86 |  |
| G.FAGDDAPRA.V | N | 18.34 | 918.4195 | 0.1 | 460.2171 | 2 | 17.21 | 2654 | 7 | 23 | 31 |  |
| S.LEKSYELPDGQ.V | Y | 16.48 | 1277.6139 | -0.9 | 639.8137 | 2 | 11.17 | 1631 | 1 | 238 | 248 |  |
| L.DLAGRDLTDYLMK.I | Y | 15.77 | 1509.7498 | -1.3 | 755.8812 | 2 | 13.04 | 1962 | 1 | 181 | 193 |  |
| L.EKSYELPDGQVITI.G | Y | 15.45 | 1590.8141 | -0.6 | 796.4139 | 2 | 13.72 | 2078 | 1 | 239 | 252 |  |
| total 23 peptides |  |  |  |  |  |  |  |  |  |  |  |  |
| --- | --- | --- | --- | --- | --- | --- | --- | --- | --- | --- | --- | --- |

tr|Q76LU1|Q76LU1\_GADCH

back to list

  

| Protein Coverage
| Supporting Peptides
|

Protein Coverage:

Supporting Peptides:

| Peptide | Uniq | -10lgP | Mass | ppm | m/z | z | RT | Scan | #Spec | Start | End | PTM |
| --- | --- | --- | --- | --- | --- | --- | --- | --- | --- | --- | --- | --- |
| S.LEKSYELPDGQVITIGNE.R | Y | 62.40 | 2004.0051 | -0.7 | 1003.0091 | 2 | 13.51 | 2043 | 1 | 238 | 255 |  |
| S.LEKSYELPDGQVITIG.N | Y | 47.00 | 1760.9196 | -0.2 | 881.4669 | 2 | 13.63 | 2063 | 2 | 238 | 253 |  |
| E.KSYELPDGQVITIGNE.R | Y | 42.86 | 1761.8784 | -1.3 | 881.9453 | 2 | 13.39 | 2022 | 2 | 240 | 255 |  |
| L.EKSYELPDGQVITIG.N | Y | 40.62 | 1647.8356 | -0.1 | 824.9250 | 2 | 13.49 | 2040 | 1 | 239 | 253 |  |
| G.IVLDAGDGVTH.N | Y | 40.00 | 1095.5560 | 0.0 | 548.7853 | 2 | 11.28 | 1650 | 1 | 153 | 163 |  |
| A.GFAGDDAPRA.V | N | 39.52 | 975.4410 | -0.2 | 488.7277 | 2 | 10.22 | 1469 | 4 | 22 | 31 |  |
| D.LAGRDLTDYLMK.I | Y | 35.48 | 1394.7228 | -0.4 | 698.3684 | 2 | 12.53 | 1874 | 1 | 182 | 193 |  |
| L.EKSYELPDGQVIT.I | Y | 34.60 | 1477.7300 | -1.0 | 739.8715 | 2 | 12.16 | 1807 | 1 | 239 | 251 |  |
| E.KSYELPDGQ.V | Y | 29.74 | 1035.4873 | 4.6 | 518.7533 | 2 | 10.72 | 1551 | 1 | 240 | 248 |  |
| L.DAGDGVTH.N | Y | 29.15 | 770.3195 | 0.0 | 386.1670 | 2 | 8.66 | 1223 | 3 | 156 | 163 |  |
| E.KSYELPDGQVITIG.N | Y | 29.00 | 1518.7930 | -0.3 | 760.4036 | 2 | 13.49 | 2039 | 2 | 240 | 253 |  |
| E.KSYELPDGQVIT.I | Y | 28.67 | 1348.6874 | -0.9 | 675.3503 | 2 | 11.98 | 1776 | 1 | 240 | 251 |  |
| S.LEKSYELPDGQVI.T | Y | 28.05 | 1489.7664 | -1.6 | 745.8893 | 2 | 12.81 | 1923 | 1 | 238 | 250 |  |
| Y.VGDEAQSK.R | N | 26.03 | 832.3926 | 0.0 | 417.2036 | 2 | 8.09 | 1115 | 1 | 56 | 63 |  |
| S.LEKSYELPDGQVITI.G | Y | 22.10 | 1703.8981 | 1.1 | 852.9572 | 2 | 13.95 | 2115 | 1 | 238 | 252 |  |
| L.DLAGRDLTDYLM.K | Y | 21.76 | 1381.6548 | 0.3 | 691.8349 | 2 | 14.01 | 2125 | 1 | 181 | 192 |  |
| T.MYPGIADRM.Q | Y | 19.69 | 1052.4783 | -1.2 | 527.2458 | 2 | 11.85 | 1751 | 1 | 307 | 315 |  |
| R.VAPEEHPTL.L | Y | 19.35 | 991.4974 | 0.6 | 496.7563 | 2 | 10.69 | 1547 | 1 | 98 | 106 |  |
| G.IITNWDDMEK.I | Y | 19.16 | 1263.5806 | -1.2 | 632.7968 | 2 | 12.15 | 1805 | 1 | 77 | 86 |  |
| G.FAGDDAPRA.V | N | 18.34 | 918.4195 | 0.1 | 460.2171 | 2 | 17.21 | 2654 | 7 | 23 | 31 |  |
| S.LEKSYELPDGQ.V | Y | 16.48 | 1277.6139 | -0.9 | 639.8137 | 2 | 11.17 | 1631 | 1 | 238 | 248 |  |
| L.DLAGRDLTDYLMK.I | Y | 15.77 | 1509.7498 | -1.3 | 755.8812 | 2 | 13.04 | 1962 | 1 | 181 | 193 |  |
| L.EKSYELPDGQVITI.G | Y | 15.45 | 1590.8141 | -0.6 | 796.4139 | 2 | 13.72 | 2078 | 1 | 239 | 252 |  |
| total 23 peptides |  |  |  |  |  |  |  |  |  |  |  |  |
| --- | --- | --- | --- | --- | --- | --- | --- | --- | --- | --- | --- | --- |

tr|F5BZL8|F5BZL8\_EPIBR

back to list

  

| Protein Coverage
| Supporting Peptides
|

Protein Coverage:

Supporting Peptides:

| Peptide | Uniq | -10lgP | Mass | ppm | m/z | z | RT | Scan | #Spec | Start | End | PTM |
| --- | --- | --- | --- | --- | --- | --- | --- | --- | --- | --- | --- | --- |
| S.LEKSYELPDGQVITIGNE.R | Y | 62.40 | 2004.0051 | -0.7 | 1003.0091 | 2 | 13.51 | 2043 | 1 | 238 | 255 |  |
| S.LEKSYELPDGQVITIG.N | Y | 47.00 | 1760.9196 | -0.2 | 881.4669 | 2 | 13.63 | 2063 | 2 | 238 | 253 |  |
| E.KSYELPDGQVITIGNE.R | Y | 42.86 | 1761.8784 | -1.3 | 881.9453 | 2 | 13.39 | 2022 | 2 | 240 | 255 |  |
| L.EKSYELPDGQVITIG.N | Y | 40.62 | 1647.8356 | -0.1 | 824.9250 | 2 | 13.49 | 2040 | 1 | 239 | 253 |  |
| G.IVLDAGDGVTH.N | Y | 40.00 | 1095.5560 | 0.0 | 548.7853 | 2 | 11.28 | 1650 | 1 | 153 | 163 |  |
| A.GFAGDDAPRA.V | N | 39.52 | 975.4410 | -0.2 | 488.7277 | 2 | 10.22 | 1469 | 4 | 22 | 31 |  |
| D.LAGRDLTDYLMK.I | Y | 35.48 | 1394.7228 | -0.4 | 698.3684 | 2 | 12.53 | 1874 | 1 | 182 | 193 |  |
| L.EKSYELPDGQVIT.I | Y | 34.60 | 1477.7300 | -1.0 | 739.8715 | 2 | 12.16 | 1807 | 1 | 239 | 251 |  |
| E.KSYELPDGQ.V | Y | 29.74 | 1035.4873 | 4.6 | 518.7533 | 2 | 10.72 | 1551 | 1 | 240 | 248 |  |
| L.DAGDGVTH.N | Y | 29.15 | 770.3195 | 0.0 | 386.1670 | 2 | 8.66 | 1223 | 3 | 156 | 163 |  |
| E.KSYELPDGQVITIG.N | Y | 29.00 | 1518.7930 | -0.3 | 760.4036 | 2 | 13.49 | 2039 | 2 | 240 | 253 |  |
| E.KSYELPDGQVIT.I | Y | 28.67 | 1348.6874 | -0.9 | 675.3503 | 2 | 11.98 | 1776 | 1 | 240 | 251 |  |
| S.LEKSYELPDGQVI.T | Y | 28.05 | 1489.7664 | -1.6 | 745.8893 | 2 | 12.81 | 1923 | 1 | 238 | 250 |  |
| Y.VGDEAQSK.R | N | 26.03 | 832.3926 | 0.0 | 417.2036 | 2 | 8.09 | 1115 | 1 | 56 | 63 |  |
| S.LEKSYELPDGQVITI.G | Y | 22.10 | 1703.8981 | 1.1 | 852.9572 | 2 | 13.95 | 2115 | 1 | 238 | 252 |  |
| L.DLAGRDLTDYLM.K | Y | 21.76 | 1381.6548 | 0.3 | 691.8349 | 2 | 14.01 | 2125 | 1 | 181 | 192 |  |
| T.MYPGIADRM.Q | Y | 19.69 | 1052.4783 | -1.2 | 527.2458 | 2 | 11.85 | 1751 | 1 | 307 | 315 |  |
| R.VAPEEHPTL.L | Y | 19.35 | 991.4974 | 0.6 | 496.7563 | 2 | 10.69 | 1547 | 1 | 98 | 106 |  |
| G.IITNWDDMEK.I | Y | 19.16 | 1263.5806 | -1.2 | 632.7968 | 2 | 12.15 | 1805 | 1 | 77 | 86 |  |
| G.FAGDDAPRA.V | N | 18.34 | 918.4195 | 0.1 | 460.2171 | 2 | 17.21 | 2654 | 7 | 23 | 31 |  |
| S.LEKSYELPDGQ.V | Y | 16.48 | 1277.6139 | -0.9 | 639.8137 | 2 | 11.17 | 1631 | 1 | 238 | 248 |  |
| L.DLAGRDLTDYLMK.I | Y | 15.77 | 1509.7498 | -1.3 | 755.8812 | 2 | 13.04 | 1962 | 1 | 181 | 193 |  |
| L.EKSYELPDGQVITI.G | Y | 15.45 | 1590.8141 | -0.6 | 796.4139 | 2 | 13.72 | 2078 | 1 | 239 | 252 |  |
| total 23 peptides |  |  |  |  |  |  |  |  |  |  |  |  |
| --- | --- | --- | --- | --- | --- | --- | --- | --- | --- | --- | --- | --- |

tr|A0A3Q4BGG8|A0A3Q4BGG8\_MOLML

back to list

  

| Protein Coverage
| Supporting Peptides
|

Protein Coverage:

Supporting Peptides:

| Peptide | Uniq | -10lgP | Mass | ppm | m/z | z | RT | Scan | #Spec | Start | End | PTM |
| --- | --- | --- | --- | --- | --- | --- | --- | --- | --- | --- | --- | --- |
| S.LEKSYELPDGQVITIGNE.R | Y | 62.40 | 2004.0051 | -0.7 | 1003.0091 | 2 | 13.51 | 2043 | 1 | 238 | 255 |  |
| S.LEKSYELPDGQVITIG.N | Y | 47.00 | 1760.9196 | -0.2 | 881.4669 | 2 | 13.63 | 2063 | 2 | 238 | 253 |  |
| E.KSYELPDGQVITIGNE.R | Y | 42.86 | 1761.8784 | -1.3 | 881.9453 | 2 | 13.39 | 2022 | 2 | 240 | 255 |  |
| L.EKSYELPDGQVITIG.N | Y | 40.62 | 1647.8356 | -0.1 | 824.9250 | 2 | 13.49 | 2040 | 1 | 239 | 253 |  |
| G.IVLDAGDGVTH.N | Y | 40.00 | 1095.5560 | 0.0 | 548.7853 | 2 | 11.28 | 1650 | 1 | 153 | 163 |  |
| A.GFAGDDAPRA.V | N | 39.52 | 975.4410 | -0.2 | 488.7277 | 2 | 10.22 | 1469 | 4 | 22 | 31 |  |
| D.LAGRDLTDYLMK.I | Y | 35.48 | 1394.7228 | -0.4 | 698.3684 | 2 | 12.53 | 1874 | 1 | 182 | 193 |  |
| L.EKSYELPDGQVIT.I | Y | 34.60 | 1477.7300 | -1.0 | 739.8715 | 2 | 12.16 | 1807 | 1 | 239 | 251 |  |
| E.KSYELPDGQ.V | Y | 29.74 | 1035.4873 | 4.6 | 518.7533 | 2 | 10.72 | 1551 | 1 | 240 | 248 |  |
| L.DAGDGVTH.N | Y | 29.15 | 770.3195 | 0.0 | 386.1670 | 2 | 8.66 | 1223 | 3 | 156 | 163 |  |
| E.KSYELPDGQVITIG.N | Y | 29.00 | 1518.7930 | -0.3 | 760.4036 | 2 | 13.49 | 2039 | 2 | 240 | 253 |  |
| E.KSYELPDGQVIT.I | Y | 28.67 | 1348.6874 | -0.9 | 675.3503 | 2 | 11.98 | 1776 | 1 | 240 | 251 |  |
| S.LEKSYELPDGQVI.T | Y | 28.05 | 1489.7664 | -1.6 | 745.8893 | 2 | 12.81 | 1923 | 1 | 238 | 250 |  |
| Y.VGDEAQSK.R | N | 26.03 | 832.3926 | 0.0 | 417.2036 | 2 | 8.09 | 1115 | 1 | 56 | 63 |  |
| S.LEKSYELPDGQVITI.G | Y | 22.10 | 1703.8981 | 1.1 | 852.9572 | 2 | 13.95 | 2115 | 1 | 238 | 252 |  |
| L.DLAGRDLTDYLM.K | Y | 21.76 | 1381.6548 | 0.3 | 691.8349 | 2 | 14.01 | 2125 | 1 | 181 | 192 |  |
| T.MYPGIADRM.Q | Y | 19.69 | 1052.4783 | -1.2 | 527.2458 | 2 | 11.85 | 1751 | 1 | 307 | 315 |  |
| R.VAPEEHPTL.L | Y | 19.35 | 991.4974 | 0.6 | 496.7563 | 2 | 10.69 | 1547 | 1 | 98 | 106 |  |
| G.IITNWDDMEK.I | Y | 19.16 | 1263.5806 | -1.2 | 632.7968 | 2 | 12.15 | 1805 | 1 | 77 | 86 |  |
| G.FAGDDAPRA.V | N | 18.34 | 918.4195 | 0.1 | 460.2171 | 2 | 17.21 | 2654 | 7 | 23 | 31 |  |
| S.LEKSYELPDGQ.V | Y | 16.48 | 1277.6139 | -0.9 | 639.8137 | 2 | 11.17 | 1631 | 1 | 238 | 248 |  |
| L.DLAGRDLTDYLMK.I | Y | 15.77 | 1509.7498 | -1.3 | 755.8812 | 2 | 13.04 | 1962 | 1 | 181 | 193 |  |
| L.EKSYELPDGQVITI.G | Y | 15.45 | 1590.8141 | -0.6 | 796.4139 | 2 | 13.72 | 2078 | 1 | 239 | 252 |  |
| total 23 peptides |  |  |  |  |  |  |  |  |  |  |  |  |
| --- | --- | --- | --- | --- | --- | --- | --- | --- | --- | --- | --- | --- |

tr|A0A3Q1DE47|A0A3Q1DE47\_AMPOC

back to list

  

| Protein Coverage
| Supporting Peptides
|

Protein Coverage:

Supporting Peptides:

| Peptide | Uniq | -10lgP | Mass | ppm | m/z | z | RT | Scan | #Spec | Start | End | PTM |
| --- | --- | --- | --- | --- | --- | --- | --- | --- | --- | --- | --- | --- |
| S.LEKSYELPDGQVITIGNE.R | Y | 62.40 | 2004.0051 | -0.7 | 1003.0091 | 2 | 13.51 | 2043 | 1 | 238 | 255 |  |
| S.LEKSYELPDGQVITIG.N | Y | 47.00 | 1760.9196 | -0.2 | 881.4669 | 2 | 13.63 | 2063 | 2 | 238 | 253 |  |
| E.KSYELPDGQVITIGNE.R | Y | 42.86 | 1761.8784 | -1.3 | 881.9453 | 2 | 13.39 | 2022 | 2 | 240 | 255 |  |
| L.EKSYELPDGQVITIG.N | Y | 40.62 | 1647.8356 | -0.1 | 824.9250 | 2 | 13.49 | 2040 | 1 | 239 | 253 |  |
| G.IVLDAGDGVTH.N | Y | 40.00 | 1095.5560 | 0.0 | 548.7853 | 2 | 11.28 | 1650 | 1 | 153 | 163 |  |
| A.GFAGDDAPRA.V | N | 39.52 | 975.4410 | -0.2 | 488.7277 | 2 | 10.22 | 1469 | 4 | 22 | 31 |  |
| D.LAGRDLTDYLMK.I | Y | 35.48 | 1394.7228 | -0.4 | 698.3684 | 2 | 12.53 | 1874 | 1 | 182 | 193 |  |
| L.EKSYELPDGQVIT.I | Y | 34.60 | 1477.7300 | -1.0 | 739.8715 | 2 | 12.16 | 1807 | 1 | 239 | 251 |  |
| E.KSYELPDGQ.V | Y | 29.74 | 1035.4873 | 4.6 | 518.7533 | 2 | 10.72 | 1551 | 1 | 240 | 248 |  |
| L.DAGDGVTH.N | Y | 29.15 | 770.3195 | 0.0 | 386.1670 | 2 | 8.66 | 1223 | 3 | 156 | 163 |  |
| E.KSYELPDGQVITIG.N | Y | 29.00 | 1518.7930 | -0.3 | 760.4036 | 2 | 13.49 | 2039 | 2 | 240 | 253 |  |
| E.KSYELPDGQVIT.I | Y | 28.67 | 1348.6874 | -0.9 | 675.3503 | 2 | 11.98 | 1776 | 1 | 240 | 251 |  |
| S.LEKSYELPDGQVI.T | Y | 28.05 | 1489.7664 | -1.6 | 745.8893 | 2 | 12.81 | 1923 | 1 | 238 | 250 |  |
| Y.VGDEAQSK.R | N | 26.03 | 832.3926 | 0.0 | 417.2036 | 2 | 8.09 | 1115 | 1 | 56 | 63 |  |
| S.LEKSYELPDGQVITI.G | Y | 22.10 | 1703.8981 | 1.1 | 852.9572 | 2 | 13.95 | 2115 | 1 | 238 | 252 |  |
| L.DLAGRDLTDYLM.K | Y | 21.76 | 1381.6548 | 0.3 | 691.8349 | 2 | 14.01 | 2125 | 1 | 181 | 192 |  |
| T.MYPGIADRM.Q | Y | 19.69 | 1052.4783 | -1.2 | 527.2458 | 2 | 11.85 | 1751 | 1 | 307 | 315 |  |
| R.VAPEEHPTL.L | Y | 19.35 | 991.4974 | 0.6 | 496.7563 | 2 | 10.69 | 1547 | 1 | 98 | 106 |  |
| G.IITNWDDMEK.I | Y | 19.16 | 1263.5806 | -1.2 | 632.7968 | 2 | 12.15 | 1805 | 1 | 77 | 86 |  |
| G.FAGDDAPRA.V | N | 18.34 | 918.4195 | 0.1 | 460.2171 | 2 | 17.21 | 2654 | 7 | 23 | 31 |  |
| S.LEKSYELPDGQ.V | Y | 16.48 | 1277.6139 | -0.9 | 639.8137 | 2 | 11.17 | 1631 | 1 | 238 | 248 |  |
| L.DLAGRDLTDYLMK.I | Y | 15.77 | 1509.7498 | -1.3 | 755.8812 | 2 | 13.04 | 1962 | 1 | 181 | 193 |  |
| L.EKSYELPDGQVITI.G | Y | 15.45 | 1590.8141 | -0.6 | 796.4139 | 2 | 13.72 | 2078 | 1 | 239 | 252 |  |
| total 23 peptides |  |  |  |  |  |  |  |  |  |  |  |  |
| --- | --- | --- | --- | --- | --- | --- | --- | --- | --- | --- | --- | --- |

tr|A0A7J6C441|A0A7J6C441\_9TELE

back to list

  

| Protein Coverage
| Supporting Peptides
|

Protein Coverage:

Supporting Peptides:

| Peptide | Uniq | -10lgP | Mass | ppm | m/z | z | RT | Scan | #Spec | Start | End | PTM |
| --- | --- | --- | --- | --- | --- | --- | --- | --- | --- | --- | --- | --- |
| S.LEKSYELPDGQVITIGNE.R | Y | 62.40 | 2004.0051 | -0.7 | 1003.0091 | 2 | 13.51 | 2043 | 1 | 238 | 255 |  |
| S.LEKSYELPDGQVITIG.N | Y | 47.00 | 1760.9196 | -0.2 | 881.4669 | 2 | 13.63 | 2063 | 2 | 238 | 253 |  |
| E.KSYELPDGQVITIGNE.R | Y | 42.86 | 1761.8784 | -1.3 | 881.9453 | 2 | 13.39 | 2022 | 2 | 240 | 255 |  |
| L.EKSYELPDGQVITIG.N | Y | 40.62 | 1647.8356 | -0.1 | 824.9250 | 2 | 13.49 | 2040 | 1 | 239 | 253 |  |
| G.IVLDAGDGVTH.N | Y | 40.00 | 1095.5560 | 0.0 | 548.7853 | 2 | 11.28 | 1650 | 1 | 153 | 163 |  |
| A.GFAGDDAPRA.V | N | 39.52 | 975.4410 | -0.2 | 488.7277 | 2 | 10.22 | 1469 | 4 | 22 | 31 |  |
| D.LAGRDLTDYLMK.I | Y | 35.48 | 1394.7228 | -0.4 | 698.3684 | 2 | 12.53 | 1874 | 1 | 182 | 193 |  |
| L.EKSYELPDGQVIT.I | Y | 34.60 | 1477.7300 | -1.0 | 739.8715 | 2 | 12.16 | 1807 | 1 | 239 | 251 |  |
| E.KSYELPDGQ.V | Y | 29.74 | 1035.4873 | 4.6 | 518.7533 | 2 | 10.72 | 1551 | 1 | 240 | 248 |  |
| L.DAGDGVTH.N | Y | 29.15 | 770.3195 | 0.0 | 386.1670 | 2 | 8.66 | 1223 | 3 | 156 | 163 |  |
| E.KSYELPDGQVITIG.N | Y | 29.00 | 1518.7930 | -0.3 | 760.4036 | 2 | 13.49 | 2039 | 2 | 240 | 253 |  |
| E.KSYELPDGQVIT.I | Y | 28.67 | 1348.6874 | -0.9 | 675.3503 | 2 | 11.98 | 1776 | 1 | 240 | 251 |  |
| S.LEKSYELPDGQVI.T | Y | 28.05 | 1489.7664 | -1.6 | 745.8893 | 2 | 12.81 | 1923 | 1 | 238 | 250 |  |
| Y.VGDEAQSK.R | N | 26.03 | 832.3926 | 0.0 | 417.2036 | 2 | 8.09 | 1115 | 1 | 56 | 63 |  |
| S.LEKSYELPDGQVITI.G | Y | 22.10 | 1703.8981 | 1.1 | 852.9572 | 2 | 13.95 | 2115 | 1 | 238 | 252 |  |
| L.DLAGRDLTDYLM.K | Y | 21.76 | 1381.6548 | 0.3 | 691.8349 | 2 | 14.01 | 2125 | 1 | 181 | 192 |  |
| T.MYPGIADRM.Q | Y | 19.69 | 1052.4783 | -1.2 | 527.2458 | 2 | 11.85 | 1751 | 1 | 307 | 315 |  |
| R.VAPEEHPTL.L | Y | 19.35 | 991.4974 | 0.6 | 496.7563 | 2 | 10.69 | 1547 | 1 | 98 | 106 |  |
| G.IITNWDDMEK.I | Y | 19.16 | 1263.5806 | -1.2 | 632.7968 | 2 | 12.15 | 1805 | 1 | 77 | 86 |  |
| G.FAGDDAPRA.V | N | 18.34 | 918.4195 | 0.1 | 460.2171 | 2 | 17.21 | 2654 | 7 | 23 | 31 |  |
| S.LEKSYELPDGQ.V | Y | 16.48 | 1277.6139 | -0.9 | 639.8137 | 2 | 11.17 | 1631 | 1 | 238 | 248 |  |
| L.DLAGRDLTDYLMK.I | Y | 15.77 | 1509.7498 | -1.3 | 755.8812 | 2 | 13.04 | 1962 | 1 | 181 | 193 |  |
| L.EKSYELPDGQVITI.G | Y | 15.45 | 1590.8141 | -0.6 | 796.4139 | 2 | 13.72 | 2078 | 1 | 239 | 252 |  |
| total 23 peptides |  |  |  |  |  |  |  |  |  |  |  |  |
| --- | --- | --- | --- | --- | --- | --- | --- | --- | --- | --- | --- | --- |

tr|A0A6P7M219|A0A6P7M219\_BETSP

back to list

  

| Protein Coverage
| Supporting Peptides
|

Protein Coverage:

Supporting Peptides:

| Peptide | Uniq | -10lgP | Mass | ppm | m/z | z | RT | Scan | #Spec | Start | End | PTM |
| --- | --- | --- | --- | --- | --- | --- | --- | --- | --- | --- | --- | --- |
| S.LEKSYELPDGQVITIGNE.R | Y | 62.40 | 2004.0051 | -0.7 | 1003.0091 | 2 | 13.51 | 2043 | 1 | 238 | 255 |  |
| S.LEKSYELPDGQVITIG.N | Y | 47.00 | 1760.9196 | -0.2 | 881.4669 | 2 | 13.63 | 2063 | 2 | 238 | 253 |  |
| E.KSYELPDGQVITIGNE.R | Y | 42.86 | 1761.8784 | -1.3 | 881.9453 | 2 | 13.39 | 2022 | 2 | 240 | 255 |  |
| L.EKSYELPDGQVITIG.N | Y | 40.62 | 1647.8356 | -0.1 | 824.9250 | 2 | 13.49 | 2040 | 1 | 239 | 253 |  |
| G.IVLDAGDGVTH.N | Y | 40.00 | 1095.5560 | 0.0 | 548.7853 | 2 | 11.28 | 1650 | 1 | 153 | 163 |  |
| A.GFAGDDAPRA.V | N | 39.52 | 975.4410 | -0.2 | 488.7277 | 2 | 10.22 | 1469 | 4 | 22 | 31 |  |
| D.LAGRDLTDYLMK.I | Y | 35.48 | 1394.7228 | -0.4 | 698.3684 | 2 | 12.53 | 1874 | 1 | 182 | 193 |  |
| L.EKSYELPDGQVIT.I | Y | 34.60 | 1477.7300 | -1.0 | 739.8715 | 2 | 12.16 | 1807 | 1 | 239 | 251 |  |
| E.KSYELPDGQ.V | Y | 29.74 | 1035.4873 | 4.6 | 518.7533 | 2 | 10.72 | 1551 | 1 | 240 | 248 |  |
| L.DAGDGVTH.N | Y | 29.15 | 770.3195 | 0.0 | 386.1670 | 2 | 8.66 | 1223 | 3 | 156 | 163 |  |
| E.KSYELPDGQVITIG.N | Y | 29.00 | 1518.7930 | -0.3 | 760.4036 | 2 | 13.49 | 2039 | 2 | 240 | 253 |  |
| E.KSYELPDGQVIT.I | Y | 28.67 | 1348.6874 | -0.9 | 675.3503 | 2 | 11.98 | 1776 | 1 | 240 | 251 |  |
| S.LEKSYELPDGQVI.T | Y | 28.05 | 1489.7664 | -1.6 | 745.8893 | 2 | 12.81 | 1923 | 1 | 238 | 250 |  |
| Y.VGDEAQSK.R | N | 26.03 | 832.3926 | 0.0 | 417.2036 | 2 | 8.09 | 1115 | 1 | 56 | 63 |  |
| S.LEKSYELPDGQVITI.G | Y | 22.10 | 1703.8981 | 1.1 | 852.9572 | 2 | 13.95 | 2115 | 1 | 238 | 252 |  |
| L.DLAGRDLTDYLM.K | Y | 21.76 | 1381.6548 | 0.3 | 691.8349 | 2 | 14.01 | 2125 | 1 | 181 | 192 |  |
| T.MYPGIADRM.Q | Y | 19.69 | 1052.4783 | -1.2 | 527.2458 | 2 | 11.85 | 1751 | 1 | 307 | 315 |  |
| R.VAPEEHPTL.L | Y | 19.35 | 991.4974 | 0.6 | 496.7563 | 2 | 10.69 | 1547 | 1 | 98 | 106 |  |
| G.IITNWDDMEK.I | Y | 19.16 | 1263.5806 | -1.2 | 632.7968 | 2 | 12.15 | 1805 | 1 | 77 | 86 |  |
| G.FAGDDAPRA.V | N | 18.34 | 918.4195 | 0.1 | 460.2171 | 2 | 17.21 | 2654 | 7 | 23 | 31 |  |
| S.LEKSYELPDGQ.V | Y | 16.48 | 1277.6139 | -0.9 | 639.8137 | 2 | 11.17 | 1631 | 1 | 238 | 248 |  |
| L.DLAGRDLTDYLMK.I | Y | 15.77 | 1509.7498 | -1.3 | 755.8812 | 2 | 13.04 | 1962 | 1 | 181 | 193 |  |
| L.EKSYELPDGQVITI.G | Y | 15.45 | 1590.8141 | -0.6 | 796.4139 | 2 | 13.72 | 2078 | 1 | 239 | 252 |  |
| total 23 peptides |  |  |  |  |  |  |  |  |  |  |  |  |
| --- | --- | --- | --- | --- | --- | --- | --- | --- | --- | --- | --- | --- |

tr|A0A3Q2P9J5|A0A3Q2P9J5\_FUNHE

back to list

  

| Protein Coverage
| Supporting Peptides
|

Protein Coverage:

Supporting Peptides:

| Peptide | Uniq | -10lgP | Mass | ppm | m/z | z | RT | Scan | #Spec | Start | End | PTM |
| --- | --- | --- | --- | --- | --- | --- | --- | --- | --- | --- | --- | --- |
| S.LEKSYELPDGQVITIGNE.R | Y | 62.40 | 2004.0051 | -0.7 | 1003.0091 | 2 | 13.51 | 2043 | 1 | 238 | 255 |  |
| S.LEKSYELPDGQVITIG.N | Y | 47.00 | 1760.9196 | -0.2 | 881.4669 | 2 | 13.63 | 2063 | 2 | 238 | 253 |  |
| E.KSYELPDGQVITIGNE.R | Y | 42.86 | 1761.8784 | -1.3 | 881.9453 | 2 | 13.39 | 2022 | 2 | 240 | 255 |  |
| L.EKSYELPDGQVITIG.N | Y | 40.62 | 1647.8356 | -0.1 | 824.9250 | 2 | 13.49 | 2040 | 1 | 239 | 253 |  |
| G.IVLDAGDGVTH.N | Y | 40.00 | 1095.5560 | 0.0 | 548.7853 | 2 | 11.28 | 1650 | 1 | 153 | 163 |  |
| A.GFAGDDAPRA.V | N | 39.52 | 975.4410 | -0.2 | 488.7277 | 2 | 10.22 | 1469 | 4 | 22 | 31 |  |
| D.LAGRDLTDYLMK.I | Y | 35.48 | 1394.7228 | -0.4 | 698.3684 | 2 | 12.53 | 1874 | 1 | 182 | 193 |  |
| L.EKSYELPDGQVIT.I | Y | 34.60 | 1477.7300 | -1.0 | 739.8715 | 2 | 12.16 | 1807 | 1 | 239 | 251 |  |
| E.KSYELPDGQ.V | Y | 29.74 | 1035.4873 | 4.6 | 518.7533 | 2 | 10.72 | 1551 | 1 | 240 | 248 |  |
| L.DAGDGVTH.N | Y | 29.15 | 770.3195 | 0.0 | 386.1670 | 2 | 8.66 | 1223 | 3 | 156 | 163 |  |
| E.KSYELPDGQVITIG.N | Y | 29.00 | 1518.7930 | -0.3 | 760.4036 | 2 | 13.49 | 2039 | 2 | 240 | 253 |  |
| E.KSYELPDGQVIT.I | Y | 28.67 | 1348.6874 | -0.9 | 675.3503 | 2 | 11.98 | 1776 | 1 | 240 | 251 |  |
| S.LEKSYELPDGQVI.T | Y | 28.05 | 1489.7664 | -1.6 | 745.8893 | 2 | 12.81 | 1923 | 1 | 238 | 250 |  |
| Y.VGDEAQSK.R | N | 26.03 | 832.3926 | 0.0 | 417.2036 | 2 | 8.09 | 1115 | 1 | 56 | 63 |  |
| S.LEKSYELPDGQVITI.G | Y | 22.10 | 1703.8981 | 1.1 | 852.9572 | 2 | 13.95 | 2115 | 1 | 238 | 252 |  |
| L.DLAGRDLTDYLM.K | Y | 21.76 | 1381.6548 | 0.3 | 691.8349 | 2 | 14.01 | 2125 | 1 | 181 | 192 |  |
| T.MYPGIADRM.Q | Y | 19.69 | 1052.4783 | -1.2 | 527.2458 | 2 | 11.85 | 1751 | 1 | 307 | 315 |  |
| R.VAPEEHPTL.L | Y | 19.35 | 991.4974 | 0.6 | 496.7563 | 2 | 10.69 | 1547 | 1 | 98 | 106 |  |
| G.IITNWDDMEK.I | Y | 19.16 | 1263.5806 | -1.2 | 632.7968 | 2 | 12.15 | 1805 | 1 | 77 | 86 |  |
| G.FAGDDAPRA.V | N | 18.34 | 918.4195 | 0.1 | 460.2171 | 2 | 17.21 | 2654 | 7 | 23 | 31 |  |
| S.LEKSYELPDGQ.V | Y | 16.48 | 1277.6139 | -0.9 | 639.8137 | 2 | 11.17 | 1631 | 1 | 238 | 248 |  |
| L.DLAGRDLTDYLMK.I | Y | 15.77 | 1509.7498 | -1.3 | 755.8812 | 2 | 13.04 | 1962 | 1 | 181 | 193 |  |
| L.EKSYELPDGQVITI.G | Y | 15.45 | 1590.8141 | -0.6 | 796.4139 | 2 | 13.72 | 2078 | 1 | 239 | 252 |  |
| total 23 peptides |  |  |  |  |  |  |  |  |  |  |  |  |
| --- | --- | --- | --- | --- | --- | --- | --- | --- | --- | --- | --- | --- |

tr|D4P4P1|D4P4P1\_COBCH

back to list

  

| Protein Coverage
| Supporting Peptides
|

Protein Coverage:

Supporting Peptides:

| Peptide | Uniq | -10lgP | Mass | ppm | m/z | z | RT | Scan | #Spec | Start | End | PTM |
| --- | --- | --- | --- | --- | --- | --- | --- | --- | --- | --- | --- | --- |
| S.LEKSYELPDGQVITIGNE.R | Y | 62.40 | 2004.0051 | -0.7 | 1003.0091 | 2 | 13.51 | 2043 | 1 | 238 | 255 |  |
| S.LEKSYELPDGQVITIG.N | Y | 47.00 | 1760.9196 | -0.2 | 881.4669 | 2 | 13.63 | 2063 | 2 | 238 | 253 |  |
| E.KSYELPDGQVITIGNE.R | Y | 42.86 | 1761.8784 | -1.3 | 881.9453 | 2 | 13.39 | 2022 | 2 | 240 | 255 |  |
| L.EKSYELPDGQVITIG.N | Y | 40.62 | 1647.8356 | -0.1 | 824.9250 | 2 | 13.49 | 2040 | 1 | 239 | 253 |  |
| G.IVLDAGDGVTH.N | Y | 40.00 | 1095.5560 | 0.0 | 548.7853 | 2 | 11.28 | 1650 | 1 | 153 | 163 |  |
| A.GFAGDDAPRA.V | N | 39.52 | 975.4410 | -0.2 | 488.7277 | 2 | 10.22 | 1469 | 4 | 22 | 31 |  |
| D.LAGRDLTDYLMK.I | Y | 35.48 | 1394.7228 | -0.4 | 698.3684 | 2 | 12.53 | 1874 | 1 | 182 | 193 |  |
| L.EKSYELPDGQVIT.I | Y | 34.60 | 1477.7300 | -1.0 | 739.8715 | 2 | 12.16 | 1807 | 1 | 239 | 251 |  |
| E.KSYELPDGQ.V | Y | 29.74 | 1035.4873 | 4.6 | 518.7533 | 2 | 10.72 | 1551 | 1 | 240 | 248 |  |
| L.DAGDGVTH.N | Y | 29.15 | 770.3195 | 0.0 | 386.1670 | 2 | 8.66 | 1223 | 3 | 156 | 163 |  |
| E.KSYELPDGQVITIG.N | Y | 29.00 | 1518.7930 | -0.3 | 760.4036 | 2 | 13.49 | 2039 | 2 | 240 | 253 |  |
| E.KSYELPDGQVIT.I | Y | 28.67 | 1348.6874 | -0.9 | 675.3503 | 2 | 11.98 | 1776 | 1 | 240 | 251 |  |
| S.LEKSYELPDGQVI.T | Y | 28.05 | 1489.7664 | -1.6 | 745.8893 | 2 | 12.81 | 1923 | 1 | 238 | 250 |  |
| Y.VGDEAQSK.R | N | 26.03 | 832.3926 | 0.0 | 417.2036 | 2 | 8.09 | 1115 | 1 | 56 | 63 |  |
| S.LEKSYELPDGQVITI.G | Y | 22.10 | 1703.8981 | 1.1 | 852.9572 | 2 | 13.95 | 2115 | 1 | 238 | 252 |  |
| L.DLAGRDLTDYLM.K | Y | 21.76 | 1381.6548 | 0.3 | 691.8349 | 2 | 14.01 | 2125 | 1 | 181 | 192 |  |
| T.MYPGIADRM.Q | Y | 19.69 | 1052.4783 | -1.2 | 527.2458 | 2 | 11.85 | 1751 | 1 | 307 | 315 |  |
| R.VAPEEHPTL.L | Y | 19.35 | 991.4974 | 0.6 | 496.7563 | 2 | 10.69 | 1547 | 1 | 98 | 106 |  |
| G.IITNWDDMEK.I | Y | 19.16 | 1263.5806 | -1.2 | 632.7968 | 2 | 12.15 | 1805 | 1 | 77 | 86 |  |
| G.FAGDDAPRA.V | N | 18.34 | 918.4195 | 0.1 | 460.2171 | 2 | 17.21 | 2654 | 7 | 23 | 31 |  |
| S.LEKSYELPDGQ.V | Y | 16.48 | 1277.6139 | -0.9 | 639.8137 | 2 | 11.17 | 1631 | 1 | 238 | 248 |  |
| L.DLAGRDLTDYLMK.I | Y | 15.77 | 1509.7498 | -1.3 | 755.8812 | 2 | 13.04 | 1962 | 1 | 181 | 193 |  |
| L.EKSYELPDGQVITI.G | Y | 15.45 | 1590.8141 | -0.6 | 796.4139 | 2 | 13.72 | 2078 | 1 | 239 | 252 |  |
| total 23 peptides |  |  |  |  |  |  |  |  |  |  |  |  |
| --- | --- | --- | --- | --- | --- | --- | --- | --- | --- | --- | --- | --- |

tr|A3F5V3|A3F5V3\_ORENI

back to list

  

| Protein Coverage
| Supporting Peptides
|

Protein Coverage:

Supporting Peptides:

| Peptide | Uniq | -10lgP | Mass | ppm | m/z | z | RT | Scan | #Spec | Start | End | PTM |
| --- | --- | --- | --- | --- | --- | --- | --- | --- | --- | --- | --- | --- |
| S.LEKSYELPDGQVITIGNE.R | Y | 62.40 | 2004.0051 | -0.7 | 1003.0091 | 2 | 13.51 | 2043 | 1 | 233 | 250 |  |
| S.LEKSYELPDGQVITIG.N | Y | 47.00 | 1760.9196 | -0.2 | 881.4669 | 2 | 13.63 | 2063 | 2 | 233 | 248 |  |
| E.KSYELPDGQVITIGNE.R | Y | 42.86 | 1761.8784 | -1.3 | 881.9453 | 2 | 13.39 | 2022 | 2 | 235 | 250 |  |
| L.EKSYELPDGQVITIG.N | Y | 40.62 | 1647.8356 | -0.1 | 824.9250 | 2 | 13.49 | 2040 | 1 | 234 | 248 |  |
| G.IVLDAGDGVTH.N | Y | 40.00 | 1095.5560 | 0.0 | 548.7853 | 2 | 11.28 | 1650 | 1 | 148 | 158 |  |
| A.GFAGDDAPRA.V | N | 39.52 | 975.4410 | -0.2 | 488.7277 | 2 | 10.22 | 1469 | 4 | 17 | 26 |  |
| D.LAGRDLTDYLMK.I | Y | 35.48 | 1394.7228 | -0.4 | 698.3684 | 2 | 12.53 | 1874 | 1 | 177 | 188 |  |
| L.EKSYELPDGQVIT.I | Y | 34.60 | 1477.7300 | -1.0 | 739.8715 | 2 | 12.16 | 1807 | 1 | 234 | 246 |  |
| E.KSYELPDGQ.V | Y | 29.74 | 1035.4873 | 4.6 | 518.7533 | 2 | 10.72 | 1551 | 1 | 235 | 243 |  |
| L.DAGDGVTH.N | Y | 29.15 | 770.3195 | 0.0 | 386.1670 | 2 | 8.66 | 1223 | 3 | 151 | 158 |  |
| E.KSYELPDGQVITIG.N | Y | 29.00 | 1518.7930 | -0.3 | 760.4036 | 2 | 13.49 | 2039 | 2 | 235 | 248 |  |
| E.KSYELPDGQVIT.I | Y | 28.67 | 1348.6874 | -0.9 | 675.3503 | 2 | 11.98 | 1776 | 1 | 235 | 246 |  |
| S.LEKSYELPDGQVI.T | Y | 28.05 | 1489.7664 | -1.6 | 745.8893 | 2 | 12.81 | 1923 | 1 | 233 | 245 |  |
| Y.VGDEAQSK.R | N | 26.03 | 832.3926 | 0.0 | 417.2036 | 2 | 8.09 | 1115 | 1 | 51 | 58 |  |
| S.LEKSYELPDGQVITI.G | Y | 22.10 | 1703.8981 | 1.1 | 852.9572 | 2 | 13.95 | 2115 | 1 | 233 | 247 |  |
| L.DLAGRDLTDYLM.K | Y | 21.76 | 1381.6548 | 0.3 | 691.8349 | 2 | 14.01 | 2125 | 1 | 176 | 187 |  |
| T.MYPGIADRM.H | Y | 19.69 | 1052.4783 | -1.2 | 527.2458 | 2 | 11.85 | 1751 | 1 | 302 | 310 |  |
| R.VAPEEHPTL.L | Y | 19.35 | 991.4974 | 0.6 | 496.7563 | 2 | 10.69 | 1547 | 1 | 93 | 101 |  |
| G.IITNWDDMEK.I | Y | 19.16 | 1263.5806 | -1.2 | 632.7968 | 2 | 12.15 | 1805 | 1 | 72 | 81 |  |
| G.FAGDDAPRA.V | N | 18.34 | 918.4195 | 0.1 | 460.2171 | 2 | 17.21 | 2654 | 7 | 18 | 26 |  |
| S.LEKSYELPDGQ.V | Y | 16.48 | 1277.6139 | -0.9 | 639.8137 | 2 | 11.17 | 1631 | 1 | 233 | 243 |  |
| L.DLAGRDLTDYLMK.I | Y | 15.77 | 1509.7498 | -1.3 | 755.8812 | 2 | 13.04 | 1962 | 1 | 176 | 188 |  |
| L.EKSYELPDGQVITI.G | Y | 15.45 | 1590.8141 | -0.6 | 796.4139 | 2 | 13.72 | 2078 | 1 | 234 | 247 |  |
| total 23 peptides |  |  |  |  |  |  |  |  |  |  |  |  |
| --- | --- | --- | --- | --- | --- | --- | --- | --- | --- | --- | --- | --- |

tr|A0A3P8QM38|A0A3P8QM38\_ASTCA

back to list

  

| Protein Coverage
| Supporting Peptides
|

Protein Coverage:

Supporting Peptides:

| Peptide | Uniq | -10lgP | Mass | ppm | m/z | z | RT | Scan | #Spec | Start | End | PTM |
| --- | --- | --- | --- | --- | --- | --- | --- | --- | --- | --- | --- | --- |
| K.LEQQVDDLEGSLE.Q | N | 41.80 | 1473.6835 | -0.6 | 737.8486 | 2 | 12.66 | 1896 | 1 | 1026 | 1038 |  |
| W.LDKNKDPLNDSVVQ.L | N | 41.51 | 1583.8154 | -4.2 | 792.9117 | 2 | 15.56 | 2391 | 8 | 596 | 609 |  |
| E.NDKQQSDEKIKKK.D | N | 41.47 | 1587.8580 | -0.2 | 794.9362 | 2 | 7.54 | 1010 | 2 | 1070 | 1082 |  |
| T.VKEDDIHPM.N | N | 31.40 | 1082.5066 | -0.8 | 542.2601 | 2 | 10.15 | 1457 | 1 | 72 | 80 |  |
| S.VIPEGQF.I | N | 30.48 | 788.4068 | -0.7 | 395.2104 | 2 | 12.26 | 1825 | 2 | 729 | 735 |  |
| Q.IGETGKT.I | N | 30.06 | 704.3705 | -0.2 | 353.1924 | 2 | 8.67 | 1226 | 1 | 1515 | 1521 |  |
| E.YKKEGIEWEFIDF.G | N | 29.33 | 1702.8242 | -0.4 | 852.4191 | 2 | 14.27 | 2169 | 1 | 503 | 515 |  |
| K.KMEGDLNEM.E | N | 28.74 | 1065.4470 | -0.5 | 533.7305 | 2 | 10.99 | 1598 | 1 | 1616 | 1624 |  |
| L.KAKNDENVRQ.L | N | 26.18 | 1200.6211 | -0.6 | 601.3174 | 2 | 7.67 | 1035 | 1 | 1259 | 1268 |  |
| Q.GEVDDAVQEAR.N | N | 26.15 | 1187.5417 | 0.3 | 594.7783 | 2 | 10.44 | 1506 | 1 | 1738 | 1748 |  |
| K.SINDVEEF.I | N | 23.98 | 951.4185 | -0.5 | 476.7163 | 2 | 12.53 | 1873 | 1 | 324 | 331 |  |
| K.LHDQH.L | N | 17.45 | 648.2980 | 0.3 | 325.1564 | 2 | 3.70 | 484 | 3 | 554 | 558 |  |
| E.LKKEQDTSAH.L | N | 16.64 | 1155.5884 | 0.0 | 578.8015 | 2 | 7.73 | 1044 | 1 | 1768 | 1777 |  |
| N.VKNWPW.L | N | 16.17 | 828.4282 | -1.1 | 415.2209 | 2 | 12.79 | 1919 | 1 | 824 | 829 |  |
| K.FKQKQREEQAEPDGTEVADK.I | N | 16.13 | 2332.1294 | -0.3 | 778.3835 | 3 | 9.20 | 1310 | 1 | 366 | 385 |  |
| N.FTNEKLQQF.F | N | 16.00 | 1153.5768 | 0.0 | 577.7957 | 2 | 11.46 | 1682 | 1 | 482 | 490 |  |
| total 16 peptides |  |  |  |  |  |  |  |  |  |  |  |  |
| --- | --- | --- | --- | --- | --- | --- | --- | --- | --- | --- | --- | --- |

tr|A0A6G0HED4|A0A6G0HED4\_LARCR

back to list

  

| Protein Coverage
| Supporting Peptides
|

Protein Coverage:

Supporting Peptides:

| Peptide | Uniq | -10lgP | Mass | ppm | m/z | z | RT | Scan | #Spec | Start | End | PTM |
| --- | --- | --- | --- | --- | --- | --- | --- | --- | --- | --- | --- | --- |
| K.LEQQVDDLEGSLE.Q | N | 41.80 | 1473.6835 | -0.6 | 737.8486 | 2 | 12.66 | 1896 | 1 | 1029 | 1041 |  |
| W.LDKNKDPLNDSVVQ.L | N | 41.51 | 1583.8154 | -4.2 | 792.9117 | 2 | 15.56 | 2391 | 8 | 596 | 609 |  |
| E.NDKQQSDEKIKKK.D | N | 41.47 | 1587.8580 | -0.2 | 794.9362 | 2 | 7.54 | 1010 | 2 | 1073 | 1085 |  |
| S.VIPEGQF.I | N | 30.48 | 788.4068 | -0.7 | 395.2104 | 2 | 12.26 | 1825 | 2 | 731 | 737 |  |
| E.YKKEGIEWEFIDF.G | N | 29.33 | 1702.8242 | -0.4 | 852.4191 | 2 | 14.27 | 2169 | 1 | 503 | 515 |  |
| K.KMEGDLNEM.E | N | 28.74 | 1065.4470 | -0.5 | 533.7305 | 2 | 10.99 | 1598 | 1 | 1619 | 1627 |  |
| L.SKIEDEQSL.G | N | 26.50 | 1047.5084 | -0.3 | 524.7614 | 2 | 10.37 | 1494 | 1 | 1094 | 1102 |  |
| L.KAKNDENVRQ.L | N | 26.18 | 1200.6211 | -0.6 | 601.3174 | 2 | 7.67 | 1035 | 1 | 1262 | 1271 |  |
| Q.GEVDDAVQEAR.N | N | 26.15 | 1187.5417 | 0.3 | 594.7783 | 2 | 10.44 | 1506 | 1 | 1741 | 1751 |  |
| K.SINDVEEF.D | N | 23.98 | 951.4185 | -0.5 | 476.7163 | 2 | 12.53 | 1873 | 1 | 324 | 331 |  |
| L.SKIEDEQSLGAQ.L | N | 20.57 | 1303.6255 | -0.3 | 652.8198 | 2 | 10.23 | 1470 | 1 | 1094 | 1105 |  |
| K.LHDQH.L | N | 17.45 | 648.2980 | 0.3 | 325.1564 | 2 | 3.70 | 484 | 3 | 554 | 558 |  |
| E.LKKEQDTSAH.L | N | 16.64 | 1155.5884 | 0.0 | 578.8015 | 2 | 7.73 | 1044 | 1 | 1771 | 1780 |  |
| N.VKNWPW.L | N | 16.17 | 828.4282 | -1.1 | 415.2209 | 2 | 12.79 | 1919 | 1 | 826 | 831 |  |
| K.FKQKQREEQAEPDGTEVADK.I | N | 16.13 | 2332.1294 | -0.3 | 778.3835 | 3 | 9.20 | 1310 | 1 | 366 | 385 |  |
| N.FTNEKLQQF.F | N | 16.00 | 1153.5768 | 0.0 | 577.7957 | 2 | 11.46 | 1682 | 1 | 482 | 490 |  |
| A.EWKQKYEEGQAELE.G | N | 15.02 | 1765.8158 | -1.1 | 883.9142 | 2 | 10.99 | 1599 | 1 | 1457 | 1470 |  |
| total 17 peptides |  |  |  |  |  |  |  |  |  |  |  |  |
| --- | --- | --- | --- | --- | --- | --- | --- | --- | --- | --- | --- | --- |

tr|A0A665X5K8|A0A665X5K8\_ECHNA

back to list

  

| Protein Coverage
| Supporting Peptides
|

Protein Coverage:

Supporting Peptides:

| Peptide | Uniq | -10lgP | Mass | ppm | m/z | z | RT | Scan | #Spec | Start | End | PTM |
| --- | --- | --- | --- | --- | --- | --- | --- | --- | --- | --- | --- | --- |
| K.LEQQVDDLEGSLE.Q | N | 41.80 | 1473.6835 | -0.6 | 737.8486 | 2 | 12.66 | 1896 | 1 | 1024 | 1036 |  |
| W.LDKNKDPLNDSVVQ.L | N | 41.51 | 1583.8154 | -4.2 | 792.9117 | 2 | 15.56 | 2391 | 8 | 596 | 609 |  |
| E.NDKQQSDEKIKKK.D | N | 41.47 | 1587.8580 | -0.2 | 794.9362 | 2 | 7.54 | 1010 | 2 | 1068 | 1080 |  |
| S.VIPEGQF.I | N | 30.48 | 788.4068 | -0.7 | 395.2104 | 2 | 12.26 | 1825 | 2 | 728 | 734 |  |
| E.YKKEGIEWEFIDF.G | N | 29.33 | 1702.8242 | -0.4 | 852.4191 | 2 | 14.27 | 2169 | 1 | 503 | 515 |  |
| K.KMEGDLNEM.E | N | 28.74 | 1065.4470 | -0.5 | 533.7305 | 2 | 10.99 | 1598 | 1 | 1614 | 1622 |  |
| L.SKIEDEQSL.G | N | 26.50 | 1047.5084 | -0.3 | 524.7614 | 2 | 10.37 | 1494 | 1 | 1089 | 1097 |  |
| L.KAKNDENVRQ.L | N | 26.18 | 1200.6211 | -0.6 | 601.3174 | 2 | 7.67 | 1035 | 1 | 1257 | 1266 |  |
| Q.GEVDDAVQEAR.N | N | 26.15 | 1187.5417 | 0.3 | 594.7783 | 2 | 10.44 | 1506 | 1 | 1736 | 1746 |  |
| K.SINDVEEF.I | N | 23.98 | 951.4185 | -0.5 | 476.7163 | 2 | 12.53 | 1873 | 1 | 324 | 331 |  |
| L.SKIEDEQSLGAQ.L | N | 20.57 | 1303.6255 | -0.3 | 652.8198 | 2 | 10.23 | 1470 | 1 | 1089 | 1100 |  |
| K.LHDQH.L | N | 17.45 | 648.2980 | 0.3 | 325.1564 | 2 | 3.70 | 484 | 3 | 554 | 558 |  |
| E.LKKEQDTSAH.L | N | 16.64 | 1155.5884 | 0.0 | 578.8015 | 2 | 7.73 | 1044 | 1 | 1766 | 1775 |  |
| N.VKNWPW.L | N | 16.17 | 828.4282 | -1.1 | 415.2209 | 2 | 12.79 | 1919 | 1 | 823 | 828 |  |
| K.FKQKQREEQAEPDGTEVADK.I | N | 16.13 | 2332.1294 | -0.3 | 778.3835 | 3 | 9.20 | 1310 | 1 | 366 | 385 |  |
| N.FTNEKLQQF.F | N | 16.00 | 1153.5768 | 0.0 | 577.7957 | 2 | 11.46 | 1682 | 1 | 482 | 490 |  |
| A.EWKQKYEEGQAELE.G | N | 15.02 | 1765.8158 | -1.1 | 883.9142 | 2 | 10.99 | 1599 | 1 | 1452 | 1465 |  |
| total 17 peptides |  |  |  |  |  |  |  |  |  |  |  |  |
| --- | --- | --- | --- | --- | --- | --- | --- | --- | --- | --- | --- | --- |

tr|A0A6J2PN08|A0A6J2PN08\_COTGO

back to list

  

| Protein Coverage
| Supporting Peptides
|

Protein Coverage:

Supporting Peptides:

| Peptide | Uniq | -10lgP | Mass | ppm | m/z | z | RT | Scan | #Spec | Start | End | PTM |
| --- | --- | --- | --- | --- | --- | --- | --- | --- | --- | --- | --- | --- |
| K.LEQQVDDLEGSLE.Q | N | 41.80 | 1473.6835 | -0.6 | 737.8486 | 2 | 12.66 | 1896 | 1 | 1041 | 1053 |  |
| W.LDKNKDPLNDSVVQ.L | N | 41.51 | 1583.8154 | -4.2 | 792.9117 | 2 | 15.56 | 2391 | 8 | 595 | 608 |  |
| E.NDKQQSDEKIKKK.D | N | 41.47 | 1587.8580 | -0.2 | 794.9362 | 2 | 7.54 | 1010 | 2 | 1085 | 1097 |  |
| P.MNPPKYDKIEDM.A | N | 39.50 | 1479.6737 | -0.8 | 740.8436 | 2 | 11.36 | 1664 | 1 | 79 | 90 |  |
| L.GFTADEKIGIY.K | N | 39.36 | 1212.6027 | -0.6 | 607.3082 | 2 | 12.49 | 1867 | 1 | 341 | 351 |  |
| S.VIPEGQF.I | N | 30.48 | 788.4068 | -0.7 | 395.2104 | 2 | 12.26 | 1825 | 2 | 743 | 749 |  |
| E.YKKEGIEWEFIDF.G | N | 29.33 | 1702.8242 | -0.4 | 852.4191 | 2 | 14.27 | 2169 | 1 | 502 | 514 |  |
| K.KMEGDLNEM.E | N | 28.74 | 1065.4470 | -0.5 | 533.7305 | 2 | 10.99 | 1598 | 1 | 1631 | 1639 |  |
| M.NPPKYDKIEDM.A | N | 26.69 | 1348.6333 | -0.3 | 675.3237 | 2 | 10.87 | 1577 | 1 | 80 | 90 |  |
| L.SKIEDEQSL.G | N | 26.50 | 1047.5084 | -0.3 | 524.7614 | 2 | 10.37 | 1494 | 1 | 1106 | 1114 |  |
| K.FKQKQREEQAEPDGTEVADK.I | N | 16.13 | 2332.1294 | -0.3 | 778.3835 | 3 | 9.20 | 1310 | 1 | 365 | 384 |  |
| N.FTNEKLQQF.F | N | 16.00 | 1153.5768 | 0.0 | 577.7957 | 2 | 11.46 | 1682 | 1 | 481 | 489 |  |
| total 12 peptides |  |  |  |  |  |  |  |  |  |  |  |  |
| --- | --- | --- | --- | --- | --- | --- | --- | --- | --- | --- | --- | --- |

tr|A0A6P7NC66|A0A6P7NC66\_BETSP

back to list

  

| Protein Coverage
| Supporting Peptides
|

Protein Coverage:

Supporting Peptides:

| Peptide | Uniq | -10lgP | Mass | ppm | m/z | z | RT | Scan | #Spec | Start | End | PTM |
| --- | --- | --- | --- | --- | --- | --- | --- | --- | --- | --- | --- | --- |
| K.LEQQVDDLEGSLE.Q | N | 41.80 | 1473.6835 | -0.6 | 737.8486 | 2 | 12.66 | 1896 | 1 | 1029 | 1041 |  |
| W.LDKNKDPLNDSVVQ.L | N | 41.51 | 1583.8154 | -4.2 | 792.9117 | 2 | 15.56 | 2391 | 8 | 595 | 608 |  |
| E.NDKQQSDEKIKKK.D | N | 41.47 | 1587.8580 | -0.2 | 794.9362 | 2 | 7.54 | 1010 | 2 | 1073 | 1085 |  |
| S.VIPEGQF.I | N | 30.48 | 788.4068 | -0.7 | 395.2104 | 2 | 12.26 | 1825 | 2 | 731 | 737 |  |
| Q.IGETGKT.I | N | 30.06 | 704.3705 | -0.2 | 353.1924 | 2 | 8.67 | 1226 | 1 | 1518 | 1524 |  |
| E.YKKEGIEWEFIDF.G | N | 29.33 | 1702.8242 | -0.4 | 852.4191 | 2 | 14.27 | 2169 | 1 | 502 | 514 |  |
| K.KMEGDLNEM.E | N | 28.74 | 1065.4470 | -0.5 | 533.7305 | 2 | 10.99 | 1598 | 1 | 1619 | 1627 |  |
| R.NPPKYDKIEDM.A | N | 26.69 | 1348.6333 | -0.3 | 675.3237 | 2 | 10.87 | 1577 | 1 | 80 | 90 |  |
| L.KAKNDENVRQ.L | N | 26.18 | 1200.6211 | -0.6 | 601.3174 | 2 | 7.67 | 1035 | 1 | 1262 | 1271 |  |
| K.SINDVEEF.I | N | 23.98 | 951.4185 | -0.5 | 476.7163 | 2 | 12.53 | 1873 | 1 | 323 | 330 |  |
| K.LHDQH.L | N | 17.45 | 648.2980 | 0.3 | 325.1564 | 2 | 3.70 | 484 | 3 | 553 | 557 |  |
| E.LKKEQDTSAH.L | N | 16.64 | 1155.5884 | 0.0 | 578.8015 | 2 | 7.73 | 1044 | 1 | 1771 | 1780 |  |
| N.VKNWPW.L | N | 16.17 | 828.4282 | -1.1 | 415.2209 | 2 | 12.79 | 1919 | 1 | 826 | 831 |  |
| K.FKQKQREEQAEPDGTEVADK.I | N | 16.13 | 2332.1294 | -0.3 | 778.3835 | 3 | 9.20 | 1310 | 1 | 365 | 384 |  |
| N.FTNEKLQQF.F | N | 16.00 | 1153.5768 | 0.0 | 577.7957 | 2 | 11.46 | 1682 | 1 | 481 | 489 |  |
| total 15 peptides |  |  |  |  |  |  |  |  |  |  |  |  |
| --- | --- | --- | --- | --- | --- | --- | --- | --- | --- | --- | --- | --- |

tr|Q90YF6|Q90YF6\_PARFO

back to list

  

| Protein Coverage
| Supporting Peptides
|

Protein Coverage:

Supporting Peptides:

| Peptide | Uniq | -10lgP | Mass | ppm | m/z | z | RT | Scan | #Spec | Start | End | PTM |
| --- | --- | --- | --- | --- | --- | --- | --- | --- | --- | --- | --- | --- |
| K.LEQQVDDLEGSLE.Q | N | 41.80 | 1473.6835 | -0.6 | 737.8486 | 2 | 12.66 | 1896 | 1 | 1029 | 1041 |  |
| W.LDKNKDPLNDSVVQ.L | N | 41.51 | 1583.8154 | -4.2 | 792.9117 | 2 | 15.56 | 2391 | 8 | 596 | 609 |  |
| E.NDKQQSDEKIKKK.D | N | 41.47 | 1587.8580 | -0.2 | 794.9362 | 2 | 7.54 | 1010 | 2 | 1073 | 1085 |  |
| S.VIPEGQF.I | N | 30.48 | 788.4068 | -0.7 | 395.2104 | 2 | 12.26 | 1825 | 2 | 731 | 737 |  |
| K.KMEGDLNEM.E | N | 28.74 | 1065.4470 | -0.5 | 533.7305 | 2 | 10.99 | 1598 | 1 | 1619 | 1627 |  |
| R.NPPKYDKIEDM.A | N | 26.69 | 1348.6333 | -0.3 | 675.3237 | 2 | 10.87 | 1577 | 1 | 81 | 91 |  |
| L.SKIEDEQSL.G | N | 26.50 | 1047.5084 | -0.3 | 524.7614 | 2 | 10.37 | 1494 | 1 | 1094 | 1102 |  |
| L.KAKNDENVRQ.L | N | 26.18 | 1200.6211 | -0.6 | 601.3174 | 2 | 7.67 | 1035 | 1 | 1262 | 1271 |  |
| K.SINDVEEF.I | N | 23.98 | 951.4185 | -0.5 | 476.7163 | 2 | 12.53 | 1873 | 1 | 324 | 331 |  |
| L.SKIEDEQSLGAQ.L | N | 20.57 | 1303.6255 | -0.3 | 652.8198 | 2 | 10.23 | 1470 | 1 | 1094 | 1105 |  |
| K.LHDQH.L | N | 17.45 | 648.2980 | 0.3 | 325.1564 | 2 | 3.70 | 484 | 3 | 554 | 558 |  |
| E.LKKEQDTSAH.L | N | 16.64 | 1155.5884 | 0.0 | 578.8015 | 2 | 7.73 | 1044 | 1 | 1771 | 1780 |  |
| N.VKNWPW.L | N | 16.17 | 828.4282 | -1.1 | 415.2209 | 2 | 12.79 | 1919 | 1 | 826 | 831 |  |
| K.FKQKQREEQAEPDGTEVADK.I | N | 16.13 | 2332.1294 | -0.3 | 778.3835 | 3 | 9.20 | 1310 | 1 | 366 | 385 |  |
| N.FTNEKLQQF.F | N | 16.00 | 1153.5768 | 0.0 | 577.7957 | 2 | 11.46 | 1682 | 1 | 482 | 490 |  |
| A.EWKQKYEEGQAELE.G | N | 15.02 | 1765.8158 | -1.1 | 883.9142 | 2 | 10.99 | 1599 | 1 | 1457 | 1470 |  |
| total 16 peptides |  |  |  |  |  |  |  |  |  |  |  |  |
| --- | --- | --- | --- | --- | --- | --- | --- | --- | --- | --- | --- | --- |

tr|A0A665WXF0|A0A665WXF0\_ECHNA

back to list

  

| Protein Coverage
| Supporting Peptides
|

Protein Coverage:

Supporting Peptides:

| Peptide | Uniq | -10lgP | Mass | ppm | m/z | z | RT | Scan | #Spec | Start | End | PTM |
| --- | --- | --- | --- | --- | --- | --- | --- | --- | --- | --- | --- | --- |
| K.LEQQVDDLEGSLE.Q | N | 41.80 | 1473.6835 | -0.6 | 737.8486 | 2 | 12.66 | 1896 | 1 | 1029 | 1041 |  |
| W.LDKNKDPLNDSVVQ.L | N | 41.51 | 1583.8154 | -4.2 | 792.9117 | 2 | 15.56 | 2391 | 8 | 594 | 607 |  |
| E.NDKQQSDEKIKKK.D | N | 41.47 | 1587.8580 | -0.2 | 794.9362 | 2 | 7.54 | 1010 | 2 | 1073 | 1085 |  |
| T.VKEDDIHPM.N | N | 31.40 | 1082.5066 | -0.8 | 542.2601 | 2 | 10.15 | 1457 | 1 | 72 | 80 |  |
| S.VIPEGQF.I | N | 30.48 | 788.4068 | -0.7 | 395.2104 | 2 | 12.26 | 1825 | 2 | 731 | 737 |  |
| K.KMEGDLNEM.E | N | 28.74 | 1065.4470 | -0.5 | 533.7305 | 2 | 10.99 | 1598 | 1 | 1619 | 1627 |  |
| L.SKIEDEQSL.G | N | 26.50 | 1047.5084 | -0.3 | 524.7614 | 2 | 10.37 | 1494 | 1 | 1094 | 1102 |  |
| Q.GEVDDAVQEAR.N | N | 26.15 | 1187.5417 | 0.3 | 594.7783 | 2 | 10.44 | 1506 | 1 | 1741 | 1751 |  |
| K.SINDVEEF.I | N | 23.98 | 951.4185 | -0.5 | 476.7163 | 2 | 12.53 | 1873 | 1 | 322 | 329 |  |
| L.SKIEDEQSLGAQ.L | N | 20.57 | 1303.6255 | -0.3 | 652.8198 | 2 | 10.23 | 1470 | 1 | 1094 | 1105 |  |
| K.LHDQH.L | N | 17.45 | 648.2980 | 0.3 | 325.1564 | 2 | 3.70 | 484 | 3 | 552 | 556 |  |
| E.LKKEQDTSAH.L | N | 16.64 | 1155.5884 | 0.0 | 578.8015 | 2 | 7.73 | 1044 | 1 | 1771 | 1780 |  |
| N.VKNWPW.M | N | 16.17 | 828.4282 | -1.1 | 415.2209 | 2 | 12.79 | 1919 | 1 | 826 | 831 |  |
| N.FTNEKLQQF.F | N | 16.00 | 1153.5768 | 0.0 | 577.7957 | 2 | 11.46 | 1682 | 1 | 480 | 488 |  |
| A.EWKQKYEEGQAELE.G | N | 15.02 | 1765.8158 | -1.1 | 883.9142 | 2 | 10.99 | 1599 | 1 | 1457 | 1470 |  |
| total 15 peptides |  |  |  |  |  |  |  |  |  |  |  |  |
| --- | --- | --- | --- | --- | --- | --- | --- | --- | --- | --- | --- | --- |

tr|A0A7N8X0U0|A0A7N8X0U0\_9TELE

back to list

  

| Protein Coverage
| Supporting Peptides
|

Protein Coverage:

Supporting Peptides:

| Peptide | Uniq | -10lgP | Mass | ppm | m/z | z | RT | Scan | #Spec | Start | End | PTM |
| --- | --- | --- | --- | --- | --- | --- | --- | --- | --- | --- | --- | --- |
| K.LEQQVDDLEGSLE.Q | N | 41.80 | 1473.6835 | -0.6 | 737.8486 | 2 | 12.66 | 1896 | 1 | 1018 | 1030 |  |
| W.LDKNKDPLNDSVVQ.L | N | 41.51 | 1583.8154 | -4.2 | 792.9117 | 2 | 15.56 | 2391 | 8 | 596 | 609 |  |
| T.VKEDDIHPM.N | N | 31.40 | 1082.5066 | -0.8 | 542.2601 | 2 | 10.15 | 1457 | 1 | 72 | 80 |  |
| S.VIPEGQF.I | N | 30.48 | 788.4068 | -0.7 | 395.2104 | 2 | 12.26 | 1825 | 2 | 729 | 735 |  |
| Q.IGETGKT.I | N | 30.06 | 704.3705 | -0.2 | 353.1924 | 2 | 8.67 | 1226 | 1 | 1507 | 1513 |  |
| K.KMEGDLNEM.E | N | 28.74 | 1065.4470 | -0.5 | 533.7305 | 2 | 10.99 | 1598 | 1 | 1608 | 1616 |  |
| L.SKIEDEQSL.G | N | 26.50 | 1047.5084 | -0.3 | 524.7614 | 2 | 10.37 | 1494 | 1 | 1083 | 1091 |  |
| L.KAKNDENVRQ.L | N | 26.18 | 1200.6211 | -0.6 | 601.3174 | 2 | 7.67 | 1035 | 1 | 1251 | 1260 |  |
| Q.GEVDDAVQEAR.N | N | 26.15 | 1187.5417 | 0.3 | 594.7783 | 2 | 10.44 | 1506 | 1 | 1730 | 1740 |  |
| K.SINDVEEF.I | N | 23.98 | 951.4185 | -0.5 | 476.7163 | 2 | 12.53 | 1873 | 1 | 324 | 331 |  |
| L.SKIEDEQSLGAQ.L | N | 20.57 | 1303.6255 | -0.3 | 652.8198 | 2 | 10.23 | 1470 | 1 | 1083 | 1094 |  |
| K.LHDQH.L | N | 17.45 | 648.2980 | 0.3 | 325.1564 | 2 | 3.70 | 484 | 3 | 554 | 558 |  |
| E.LKKEQDTSAH.L | N | 16.64 | 1155.5884 | 0.0 | 578.8015 | 2 | 7.73 | 1044 | 1 | 1760 | 1769 |  |
| N.VKNWPW.L | N | 16.17 | 828.4282 | -1.1 | 415.2209 | 2 | 12.79 | 1919 | 1 | 824 | 829 |  |
| K.FKQKQREEQAEPDGTEVADK.I | N | 16.13 | 2332.1294 | -0.3 | 778.3835 | 3 | 9.20 | 1310 | 1 | 366 | 385 |  |
| N.FTNEKLQQF.F | N | 16.00 | 1153.5768 | 0.0 | 577.7957 | 2 | 11.46 | 1682 | 1 | 482 | 490 |  |
| A.EWKQKYEEGQAELE.G | N | 15.02 | 1765.8158 | -1.1 | 883.9142 | 2 | 10.99 | 1599 | 1 | 1446 | 1459 |  |
| total 17 peptides |  |  |  |  |  |  |  |  |  |  |  |  |
| --- | --- | --- | --- | --- | --- | --- | --- | --- | --- | --- | --- | --- |

tr|A0A7N8WLS2|A0A7N8WLS2\_9TELE

back to list

  

| Protein Coverage
| Supporting Peptides
|

Protein Coverage:

Supporting Peptides:

| Peptide | Uniq | -10lgP | Mass | ppm | m/z | z | RT | Scan | #Spec | Start | End | PTM |
| --- | --- | --- | --- | --- | --- | --- | --- | --- | --- | --- | --- | --- |
| K.LEQQVDDLEGSLE.Q | N | 41.80 | 1473.6835 | -0.6 | 737.8486 | 2 | 12.66 | 1896 | 1 | 1026 | 1038 |  |
| W.LDKNKDPLNDSVVQ.L | N | 41.51 | 1583.8154 | -4.2 | 792.9117 | 2 | 15.56 | 2391 | 8 | 596 | 609 |  |
| T.VKEDDIHPM.N | N | 31.40 | 1082.5066 | -0.8 | 542.2601 | 2 | 10.15 | 1457 | 1 | 72 | 80 |  |
| S.VIPEGQF.I | N | 30.48 | 788.4068 | -0.7 | 395.2104 | 2 | 12.26 | 1825 | 2 | 728 | 734 |  |
| Q.IGETGKT.I | N | 30.06 | 704.3705 | -0.2 | 353.1924 | 2 | 8.67 | 1226 | 1 | 1515 | 1521 |  |
| K.KMEGDLNEM.E | N | 28.74 | 1065.4470 | -0.5 | 533.7305 | 2 | 10.99 | 1598 | 1 | 1616 | 1624 |  |
| L.SKIEDEQSL.G | N | 26.50 | 1047.5084 | -0.3 | 524.7614 | 2 | 10.37 | 1494 | 1 | 1091 | 1099 |  |
| L.KAKNDENVRQ.L | N | 26.18 | 1200.6211 | -0.6 | 601.3174 | 2 | 7.67 | 1035 | 1 | 1259 | 1268 |  |
| Q.GEVDDAVQEAR.N | N | 26.15 | 1187.5417 | 0.3 | 594.7783 | 2 | 10.44 | 1506 | 1 | 1738 | 1748 |  |
| K.SINDVEEF.I | N | 23.98 | 951.4185 | -0.5 | 476.7163 | 2 | 12.53 | 1873 | 1 | 324 | 331 |  |
| L.SKIEDEQSLGAQ.L | N | 20.57 | 1303.6255 | -0.3 | 652.8198 | 2 | 10.23 | 1470 | 1 | 1091 | 1102 |  |
| K.LHDQH.L | N | 17.45 | 648.2980 | 0.3 | 325.1564 | 2 | 3.70 | 484 | 3 | 554 | 558 |  |
| E.LKKEQDTSAH.L | N | 16.64 | 1155.5884 | 0.0 | 578.8015 | 2 | 7.73 | 1044 | 1 | 1768 | 1777 |  |
| N.VKNWPW.L | N | 16.17 | 828.4282 | -1.1 | 415.2209 | 2 | 12.79 | 1919 | 1 | 823 | 828 |  |
| K.FKQKQREEQAEPDGTEVADK.I | N | 16.13 | 2332.1294 | -0.3 | 778.3835 | 3 | 9.20 | 1310 | 1 | 366 | 385 |  |
| N.FTNEKLQQF.F | N | 16.00 | 1153.5768 | 0.0 | 577.7957 | 2 | 11.46 | 1682 | 1 | 482 | 490 |  |
| A.EWKQKYEEGQAELE.G | N | 15.02 | 1765.8158 | -1.1 | 883.9142 | 2 | 10.99 | 1599 | 1 | 1454 | 1467 |  |
| total 17 peptides |  |  |  |  |  |  |  |  |  |  |  |  |
| --- | --- | --- | --- | --- | --- | --- | --- | --- | --- | --- | --- | --- |

tr|A0A6G0IP12|A0A6G0IP12\_LARCR

back to list

  

| Protein Coverage
| Supporting Peptides
|

Protein Coverage:

Supporting Peptides:

| Peptide | Uniq | -10lgP | Mass | ppm | m/z | z | RT | Scan | #Spec | Start | End | PTM |
| --- | --- | --- | --- | --- | --- | --- | --- | --- | --- | --- | --- | --- |
| A.FDMFDTDGGGDISTK.E | Y | 62.48 | 1604.6664 | -1.2 | 803.3395 | 2 | 12.80 | 1921 | 1 | 24 | 38 |  |
| E.VDEDGSGTIDFEEF.L | Y | 46.93 | 1558.6311 | 0.5 | 780.3232 | 2 | 14.02 | 2127 | 1 | 63 | 76 |  |
| N.SDGMLDFDEFLKM.M | Y | 35.92 | 1546.6683 | -1.4 | 774.3404 | 2 | 15.29 | 2343 | 1 | 143 | 155 |  |
| D.EDGSGTIDFEEF.L | Y | 24.63 | 1344.5356 | 0.3 | 673.2753 | 2 | 13.97 | 2119 | 1 | 65 | 76 |  |
| M.KDGDKNSDGMLDFDEFLK.M | Y | 18.77 | 2072.9360 | -0.2 | 691.9858 | 3 | 13.50 | 2041 | 1 | 137 | 154 |  |
| E.LMKDGDKNSDGMLDFDEFLK.M | Y | 16.70 | 2317.0605 | -0.1 | 773.3607 | 3 | 13.52 | 2045 | 1 | 135 | 154 |  |
| total 6 peptides |  |  |  |  |  |  |  |  |  |  |  |  |
| --- | --- | --- | --- | --- | --- | --- | --- | --- | --- | --- | --- | --- |

tr|A0A1A8Q0J1|A0A1A8Q0J1\_9TELE

back to list

  

| Protein Coverage
| Supporting Peptides
|

Protein Coverage:

Supporting Peptides:

| Peptide | Uniq | -10lgP | Mass | ppm | m/z | z | RT | Scan | #Spec | Start | End | PTM |
| --- | --- | --- | --- | --- | --- | --- | --- | --- | --- | --- | --- | --- |
| A.FDMFDTDGGGDISTK.E | Y | 62.48 | 1604.6664 | -1.2 | 803.3395 | 2 | 12.80 | 1921 | 1 | 25 | 39 |  |
| E.VDEDGSGTIDFEEF.L | Y | 46.93 | 1558.6311 | 0.5 | 780.3232 | 2 | 14.02 | 2127 | 1 | 64 | 77 |  |
| N.SDGMLDFDEFLKM.M | Y | 35.92 | 1546.6683 | -1.4 | 774.3404 | 2 | 15.29 | 2343 | 1 | 144 | 156 |  |
| D.EDGSGTIDFEEF.L | Y | 24.63 | 1344.5356 | 0.3 | 673.2753 | 2 | 13.97 | 2119 | 1 | 66 | 77 |  |
| M.KDGDKNSDGMLDFDEFLK.M | Y | 18.77 | 2072.9360 | -0.2 | 691.9858 | 3 | 13.50 | 2041 | 1 | 138 | 155 |  |
| E.LMKDGDKNSDGMLDFDEFLK.M | Y | 16.70 | 2317.0605 | -0.1 | 773.3607 | 3 | 13.52 | 2045 | 1 | 136 | 155 |  |
| total 6 peptides |  |  |  |  |  |  |  |  |  |  |  |  |
| --- | --- | --- | --- | --- | --- | --- | --- | --- | --- | --- | --- | --- |

tr|A0A3P8SK70|A0A3P8SK70\_AMPPE

back to list

  

| Protein Coverage
| Supporting Peptides
|

Protein Coverage:

Supporting Peptides:

| Peptide | Uniq | -10lgP | Mass | ppm | m/z | z | RT | Scan | #Spec | Start | End | PTM |
| --- | --- | --- | --- | --- | --- | --- | --- | --- | --- | --- | --- | --- |
| K.LEQQVDDLEGSLE.Q | N | 41.80 | 1473.6835 | -0.6 | 737.8486 | 2 | 12.66 | 1896 | 1 | 1027 | 1039 |  |
| W.LDKNKDPLNDSVVQ.L | N | 41.51 | 1583.8154 | -4.2 | 792.9117 | 2 | 15.56 | 2391 | 8 | 596 | 609 |  |
| S.VIPEGQF.I | N | 30.48 | 788.4068 | -0.7 | 395.2104 | 2 | 12.26 | 1825 | 2 | 729 | 735 |  |
| Q.IGETGKT.I | N | 30.06 | 704.3705 | -0.2 | 353.1924 | 2 | 8.67 | 1226 | 1 | 1516 | 1522 |  |
| E.YKKEGIEWEFIDF.G | N | 29.33 | 1702.8242 | -0.4 | 852.4191 | 2 | 14.27 | 2169 | 1 | 503 | 515 |  |
| K.KMEGDLNEM.E | N | 28.74 | 1065.4470 | -0.5 | 533.7305 | 2 | 10.99 | 1598 | 1 | 1617 | 1625 |  |
| L.SKIEDEQSL.G | N | 26.50 | 1047.5084 | -0.3 | 524.7614 | 2 | 10.37 | 1494 | 1 | 1092 | 1100 |  |
| Q.GEVDDAVQEAR.N | N | 26.15 | 1187.5417 | 0.3 | 594.7783 | 2 | 10.44 | 1506 | 1 | 1739 | 1749 |  |
| K.SINDVEEF.I | N | 23.98 | 951.4185 | -0.5 | 476.7163 | 2 | 12.53 | 1873 | 1 | 324 | 331 |  |
| L.SKIEDEQSLGAQ.L | N | 20.57 | 1303.6255 | -0.3 | 652.8198 | 2 | 10.23 | 1470 | 1 | 1092 | 1103 |  |
| K.LHDQH.L | N | 17.45 | 648.2980 | 0.3 | 325.1564 | 2 | 3.70 | 484 | 3 | 554 | 558 |  |
| E.LKKEQDTSAH.L | N | 16.64 | 1155.5884 | 0.0 | 578.8015 | 2 | 7.73 | 1044 | 1 | 1769 | 1778 |  |
| N.VKNWPW.L | N | 16.17 | 828.4282 | -1.1 | 415.2209 | 2 | 12.79 | 1919 | 1 | 824 | 829 |  |
| K.FKQKQREEQAEPDGTEVADK.I | N | 16.13 | 2332.1294 | -0.3 | 778.3835 | 3 | 9.20 | 1310 | 1 | 366 | 385 |  |
| N.FTNEKLQQF.F | N | 16.00 | 1153.5768 | 0.0 | 577.7957 | 2 | 11.46 | 1682 | 1 | 482 | 490 |  |
| A.EWKQKYEEGQAELE.G | N | 15.02 | 1765.8158 | -1.1 | 883.9142 | 2 | 10.99 | 1599 | 1 | 1455 | 1468 |  |
| total 16 peptides |  |  |  |  |  |  |  |  |  |  |  |  |
| --- | --- | --- | --- | --- | --- | --- | --- | --- | --- | --- | --- | --- |

tr|A0A3P8SLP0|A0A3P8SLP0\_AMPPE

back to list

  

| Protein Coverage
| Supporting Peptides
|

Protein Coverage:

Supporting Peptides:

| Peptide | Uniq | -10lgP | Mass | ppm | m/z | z | RT | Scan | #Spec | Start | End | PTM |
| --- | --- | --- | --- | --- | --- | --- | --- | --- | --- | --- | --- | --- |
| K.LEQQVDDLEGSLE.Q | N | 41.80 | 1473.6835 | -0.6 | 737.8486 | 2 | 12.66 | 1896 | 1 | 1031 | 1043 |  |
| W.LDKNKDPLNDSVVQ.L | N | 41.51 | 1583.8154 | -4.2 | 792.9117 | 2 | 15.56 | 2391 | 8 | 596 | 609 |  |
| S.VIPEGQF.I | N | 30.48 | 788.4068 | -0.7 | 395.2104 | 2 | 12.26 | 1825 | 2 | 733 | 739 |  |
| Q.IGETGKT.I | N | 30.06 | 704.3705 | -0.2 | 353.1924 | 2 | 8.67 | 1226 | 1 | 1520 | 1526 |  |
| E.YKKEGIEWEFIDF.G | N | 29.33 | 1702.8242 | -0.4 | 852.4191 | 2 | 14.27 | 2169 | 1 | 503 | 515 |  |
| K.KMEGDLNEM.E | N | 28.74 | 1065.4470 | -0.5 | 533.7305 | 2 | 10.99 | 1598 | 1 | 1621 | 1629 |  |
| L.SKIEDEQSL.G | N | 26.50 | 1047.5084 | -0.3 | 524.7614 | 2 | 10.37 | 1494 | 1 | 1096 | 1104 |  |
| Q.GEVDDAVQEAR.N | N | 26.15 | 1187.5417 | 0.3 | 594.7783 | 2 | 10.44 | 1506 | 1 | 1743 | 1753 |  |
| K.SINDVEEF.I | N | 23.98 | 951.4185 | -0.5 | 476.7163 | 2 | 12.53 | 1873 | 1 | 324 | 331 |  |
| L.SKIEDEQSLGAQ.L | N | 20.57 | 1303.6255 | -0.3 | 652.8198 | 2 | 10.23 | 1470 | 1 | 1096 | 1107 |  |
| K.LHDQH.L | N | 17.45 | 648.2980 | 0.3 | 325.1564 | 2 | 3.70 | 484 | 3 | 554 | 558 |  |
| E.LKKEQDTSAH.L | N | 16.64 | 1155.5884 | 0.0 | 578.8015 | 2 | 7.73 | 1044 | 1 | 1773 | 1782 |  |
| N.VKNWPW.L | N | 16.17 | 828.4282 | -1.1 | 415.2209 | 2 | 12.79 | 1919 | 1 | 828 | 833 |  |
| K.FKQKQREEQAEPDGTEVADK.I | N | 16.13 | 2332.1294 | -0.3 | 778.3835 | 3 | 9.20 | 1310 | 1 | 366 | 385 |  |
| N.FTNEKLQQF.F | N | 16.00 | 1153.5768 | 0.0 | 577.7957 | 2 | 11.46 | 1682 | 1 | 482 | 490 |  |
| A.EWKQKYEEGQAELE.G | N | 15.02 | 1765.8158 | -1.1 | 883.9142 | 2 | 10.99 | 1599 | 1 | 1459 | 1472 |  |
| total 16 peptides |  |  |  |  |  |  |  |  |  |  |  |  |
| --- | --- | --- | --- | --- | --- | --- | --- | --- | --- | --- | --- | --- |

tr|A0A3Q3WZY2|A0A3Q3WZY2\_MOLML

back to list

  

| Protein Coverage
| Supporting Peptides
|

Protein Coverage:

Supporting Peptides:

| Peptide | Uniq | -10lgP | Mass | ppm | m/z | z | RT | Scan | #Spec | Start | End | PTM |
| --- | --- | --- | --- | --- | --- | --- | --- | --- | --- | --- | --- | --- |
| K.LEQQVDDLEGSLE.Q | N | 41.80 | 1473.6835 | -0.6 | 737.8486 | 2 | 12.66 | 1896 | 1 | 1001 | 1013 |  |
| W.LDKNKDPLNDSVVQ.L | N | 41.51 | 1583.8154 | -4.2 | 792.9117 | 2 | 15.56 | 2391 | 8 | 596 | 609 |  |
| T.VKEDDIHPM.N | N | 31.40 | 1082.5066 | -0.8 | 542.2601 | 2 | 10.15 | 1457 | 1 | 72 | 80 |  |
| S.VIPEGQF.I | N | 30.48 | 788.4068 | -0.7 | 395.2104 | 2 | 12.26 | 1825 | 2 | 730 | 736 |  |
| E.YKKEGIEWEFIDF.G | N | 29.33 | 1702.8242 | -0.4 | 852.4191 | 2 | 14.27 | 2169 | 1 | 503 | 515 |  |
| K.KMEGDLNEM.E | N | 28.74 | 1065.4470 | -0.5 | 533.7305 | 2 | 10.99 | 1598 | 1 | 1473 | 1481 |  |
| L.SKIEDEQSL.G | N | 26.50 | 1047.5084 | -0.3 | 524.7614 | 2 | 10.37 | 1494 | 1 | 1066 | 1074 |  |
| K.SINDVEEF.D | N | 23.98 | 951.4185 | -0.5 | 476.7163 | 2 | 12.53 | 1873 | 1 | 324 | 331 |  |
| L.SKIEDEQSLGAQ.L | N | 20.57 | 1303.6255 | -0.3 | 652.8198 | 2 | 10.23 | 1470 | 1 | 1066 | 1077 |  |
| K.LHDQH.L | N | 17.45 | 648.2980 | 0.3 | 325.1564 | 2 | 3.70 | 484 | 3 | 554 | 558 |  |
| E.LKKEQDTSAH.L | N | 16.64 | 1155.5884 | 0.0 | 578.8015 | 2 | 7.73 | 1044 | 1 | 1625 | 1634 |  |
| N.VKNWPW.M | N | 16.17 | 828.4282 | -1.1 | 415.2209 | 2 | 12.79 | 1919 | 1 | 825 | 830 |  |
| K.FKQKQREEQAEPDGTEVADK.I | N | 16.13 | 2332.1294 | -0.3 | 778.3835 | 3 | 9.20 | 1310 | 1 | 366 | 385 |  |
| N.FTNEKLQQF.F | N | 16.00 | 1153.5768 | 0.0 | 577.7957 | 2 | 11.46 | 1682 | 1 | 482 | 490 |  |
| A.EWKQKYEEGQAELE.G | N | 15.02 | 1765.8158 | -1.1 | 883.9142 | 2 | 10.99 | 1599 | 1 | 1311 | 1324 |  |
| total 15 peptides |  |  |  |  |  |  |  |  |  |  |  |  |
| --- | --- | --- | --- | --- | --- | --- | --- | --- | --- | --- | --- | --- |

tr|A0A6P6KAZ0|A0A6P6KAZ0\_CARAU

back to list

  

| Protein Coverage
| Supporting Peptides
|

Protein Coverage:

Supporting Peptides:

| Peptide | Uniq | -10lgP | Mass | ppm | m/z | z | RT | Scan | #Spec | Start | End | PTM |
| --- | --- | --- | --- | --- | --- | --- | --- | --- | --- | --- | --- | --- |
| K.LEQQVDDLEGSLE.Q | N | 41.80 | 1473.6835 | -0.6 | 737.8486 | 2 | 12.66 | 1896 | 1 | 1027 | 1039 |  |
| W.LDKNKDPLNDSVVQ.L | N | 41.51 | 1583.8154 | -4.2 | 792.9117 | 2 | 15.56 | 2391 | 8 | 594 | 607 |  |
| L.GFTADEKIGIY.K | N | 39.36 | 1212.6027 | -0.6 | 607.3082 | 2 | 12.49 | 1867 | 1 | 340 | 350 |  |
| S.VIPEGQF.I | N | 30.48 | 788.4068 | -0.7 | 395.2104 | 2 | 12.26 | 1825 | 2 | 729 | 735 |  |
| Q.LGETGKT.I | N | 30.06 | 704.3705 | -0.2 | 353.1924 | 2 | 8.67 | 1226 | 1 | 1516 | 1522 |  |
| E.YKKEGIEWEFIDF.G | N | 29.33 | 1702.8242 | -0.4 | 852.4191 | 2 | 14.27 | 2169 | 1 | 501 | 513 |  |
| K.KMEGDLNEM.E | N | 28.74 | 1065.4470 | -0.5 | 533.7305 | 2 | 10.99 | 1598 | 1 | 1617 | 1625 |  |
| K.SINDVEEF.V | N | 23.98 | 951.4185 | -0.5 | 476.7163 | 2 | 12.53 | 1873 | 1 | 322 | 329 |  |
| E.LKKEQDTSAH.L | N | 16.64 | 1155.5884 | 0.0 | 578.8015 | 2 | 7.73 | 1044 | 1 | 1769 | 1778 |  |
| K.FKQKQREEQAEPDGTEVADK.I | N | 16.13 | 2332.1294 | -0.3 | 778.3835 | 3 | 9.20 | 1310 | 1 | 364 | 383 |  |
| N.FTNEKLQQF.F | N | 16.00 | 1153.5768 | 0.0 | 577.7957 | 2 | 11.46 | 1682 | 1 | 480 | 488 |  |
| total 11 peptides |  |  |  |  |  |  |  |  |  |  |  |  |
| --- | --- | --- | --- | --- | --- | --- | --- | --- | --- | --- | --- | --- |

tr|A0A3Q3EJE1|A0A3Q3EJE1\_9LABR

back to list

  

| Protein Coverage
| Supporting Peptides
|

Protein Coverage:

Supporting Peptides:

| Peptide | Uniq | -10lgP | Mass | ppm | m/z | z | RT | Scan | #Spec | Start | End | PTM |
| --- | --- | --- | --- | --- | --- | --- | --- | --- | --- | --- | --- | --- |
| K.LEQQVDDLEGSLE.Q | N | 41.80 | 1473.6835 | -0.6 | 737.8486 | 2 | 12.66 | 1896 | 1 | 1019 | 1031 |  |
| E.NDKQQSDEKIKKK.D | N | 41.47 | 1587.8580 | -0.2 | 794.9362 | 2 | 7.54 | 1010 | 2 | 1063 | 1075 |  |
| P.MNPPKYDKIEDM.A | N | 39.50 | 1479.6737 | -0.8 | 740.8436 | 2 | 11.36 | 1664 | 1 | 79 | 90 |  |
| S.VIPEGQF.I | N | 30.48 | 788.4068 | -0.7 | 395.2104 | 2 | 12.26 | 1825 | 2 | 721 | 727 |  |
| K.KMEGDLNEM.E | N | 28.74 | 1065.4470 | -0.5 | 533.7305 | 2 | 10.99 | 1598 | 1 | 1609 | 1617 |  |
| M.NPPKYDKIEDM.A | N | 26.69 | 1348.6333 | -0.3 | 675.3237 | 2 | 10.87 | 1577 | 1 | 80 | 90 |  |
| L.SKIEDEQSL.G | N | 26.50 | 1047.5084 | -0.3 | 524.7614 | 2 | 10.37 | 1494 | 1 | 1084 | 1092 |  |
| L.SKIEDEQSLGAQ.L | N | 20.57 | 1303.6255 | -0.3 | 652.8198 | 2 | 10.23 | 1470 | 1 | 1084 | 1095 |  |
| E.LKKEQDTSAH.L | N | 16.64 | 1155.5884 | 0.0 | 578.8015 | 2 | 7.73 | 1044 | 1 | 1761 | 1770 |  |
| N.FTNEKLQQF.F | N | 16.00 | 1153.5768 | 0.0 | 577.7957 | 2 | 11.46 | 1682 | 1 | 476 | 484 |  |
| A.EWKQKYEEGQAELE.G | N | 15.02 | 1765.8158 | -1.1 | 883.9142 | 2 | 10.99 | 1599 | 1 | 1447 | 1460 |  |
| total 11 peptides |  |  |  |  |  |  |  |  |  |  |  |  |
| --- | --- | --- | --- | --- | --- | --- | --- | --- | --- | --- | --- | --- |

tr|A0A6P8GH29|A0A6P8GH29\_CLUHA

back to list

  

| Protein Coverage
| Supporting Peptides
|

Protein Coverage:

Supporting Peptides:

| Peptide | Uniq | -10lgP | Mass | ppm | m/z | z | RT | Scan | #Spec | Start | End | PTM |
| --- | --- | --- | --- | --- | --- | --- | --- | --- | --- | --- | --- | --- |
| K.LEQQVDDLEGSLE.Q | N | 41.80 | 1473.6835 | -0.6 | 737.8486 | 2 | 12.66 | 1896 | 1 | 1026 | 1038 |  |
| E.NDKQQSDEKLKKK.D | N | 41.47 | 1587.8580 | -0.2 | 794.9362 | 2 | 7.54 | 1010 | 2 | 1070 | 1082 |  |
| P.MNPPKYDKIEDM.A | N | 39.50 | 1479.6737 | -0.8 | 740.8436 | 2 | 11.36 | 1664 | 1 | 80 | 91 |  |
| S.VIPEGQF.I | N | 30.48 | 788.4068 | -0.7 | 395.2104 | 2 | 12.26 | 1825 | 2 | 728 | 734 |  |
| K.KMEGDLNEM.E | N | 28.74 | 1065.4470 | -0.5 | 533.7305 | 2 | 10.99 | 1598 | 1 | 1616 | 1624 |  |
| M.NPPKYDKIEDM.A | N | 26.69 | 1348.6333 | -0.3 | 675.3237 | 2 | 10.87 | 1577 | 1 | 81 | 91 |  |
| L.SKIEDEQSL.G | N | 26.50 | 1047.5084 | -0.3 | 524.7614 | 2 | 10.37 | 1494 | 1 | 1091 | 1099 |  |
| L.SKIEDEQSLGAQ.L | N | 20.57 | 1303.6255 | -0.3 | 652.8198 | 2 | 10.23 | 1470 | 1 | 1091 | 1102 |  |
| E.LKKEQDTSAH.L | N | 16.64 | 1155.5884 | 0.0 | 578.8015 | 2 | 7.73 | 1044 | 1 | 1768 | 1777 |  |
| N.FTNEKLQQF.F | N | 16.00 | 1153.5768 | 0.0 | 577.7957 | 2 | 11.46 | 1682 | 1 | 481 | 489 |  |
| A.EWKQKYEEGQAELE.G | N | 15.02 | 1765.8158 | -1.1 | 883.9142 | 2 | 10.99 | 1599 | 1 | 1454 | 1467 |  |
| total 11 peptides |  |  |  |  |  |  |  |  |  |  |  |  |
| --- | --- | --- | --- | --- | --- | --- | --- | --- | --- | --- | --- | --- |

tr|A0A7N8XS20|A0A7N8XS20\_9TELE

back to list

  

| Protein Coverage
| Supporting Peptides
|

Protein Coverage:

Supporting Peptides:

| Peptide | Uniq | -10lgP | Mass | ppm | m/z | z | RT | Scan | #Spec | Start | End | PTM |
| --- | --- | --- | --- | --- | --- | --- | --- | --- | --- | --- | --- | --- |
| K.LEQQVDDLEGSLE.Q | N | 41.80 | 1473.6835 | -0.6 | 737.8486 | 2 | 12.66 | 1896 | 1 | 1030 | 1042 |  |
| S.VIPEGQF.I | N | 30.48 | 788.4068 | -0.7 | 395.2104 | 2 | 12.26 | 1825 | 2 | 732 | 738 |  |
| Q.IGETGKT.I | N | 30.06 | 704.3705 | -0.2 | 353.1924 | 2 | 8.67 | 1226 | 1 | 1519 | 1525 |  |
| E.YKKEGIEWEFIDF.G | N | 29.33 | 1702.8242 | -0.4 | 852.4191 | 2 | 14.27 | 2169 | 1 | 503 | 515 |  |
| K.KMEGDLNEM.E | N | 28.74 | 1065.4470 | -0.5 | 533.7305 | 2 | 10.99 | 1598 | 1 | 1620 | 1628 |  |
| L.SKIEDEQSL.G | N | 26.50 | 1047.5084 | -0.3 | 524.7614 | 2 | 10.37 | 1494 | 1 | 1095 | 1103 |  |
| L.KAKNDENVRQ.V | N | 26.18 | 1200.6211 | -0.6 | 601.3174 | 2 | 7.67 | 1035 | 1 | 1263 | 1272 |  |
| Q.GEVDDAVQEAR.N | N | 26.15 | 1187.5417 | 0.3 | 594.7783 | 2 | 10.44 | 1506 | 1 | 1742 | 1752 |  |
| K.SINDVEEF.I | N | 23.98 | 951.4185 | -0.5 | 476.7163 | 2 | 12.53 | 1873 | 1 | 324 | 331 |  |
| L.SKIEDEQSLGAQ.L | N | 20.57 | 1303.6255 | -0.3 | 652.8198 | 2 | 10.23 | 1470 | 1 | 1095 | 1106 |  |
| K.LHDQH.L | N | 17.45 | 648.2980 | 0.3 | 325.1564 | 2 | 3.70 | 484 | 3 | 554 | 558 |  |
| E.LKKEQDTSAH.L | N | 16.64 | 1155.5884 | 0.0 | 578.8015 | 2 | 7.73 | 1044 | 1 | 1772 | 1781 |  |
| N.VKNWPW.L | N | 16.17 | 828.4282 | -1.1 | 415.2209 | 2 | 12.79 | 1919 | 1 | 827 | 832 |  |
| K.FKQKQREEQAEPDGTEVADK.I | N | 16.13 | 2332.1294 | -0.3 | 778.3835 | 3 | 9.20 | 1310 | 1 | 366 | 385 |  |
| N.FTNEKLQQF.F | N | 16.00 | 1153.5768 | 0.0 | 577.7957 | 2 | 11.46 | 1682 | 1 | 482 | 490 |  |
| A.EWKQKYEEGQAELE.G | N | 15.02 | 1765.8158 | -1.1 | 883.9142 | 2 | 10.99 | 1599 | 1 | 1458 | 1471 |  |
| total 16 peptides |  |  |  |  |  |  |  |  |  |  |  |  |
| --- | --- | --- | --- | --- | --- | --- | --- | --- | --- | --- | --- | --- |

tr|A0A6P8FM41|A0A6P8FM41\_CLUHA

back to list

  

| Protein Coverage
| Supporting Peptides
|

Protein Coverage:

Supporting Peptides:

| Peptide | Uniq | -10lgP | Mass | ppm | m/z | z | RT | Scan | #Spec | Start | End | PTM |
| --- | --- | --- | --- | --- | --- | --- | --- | --- | --- | --- | --- | --- |
| K.LEQQVDDLEGSLE.Q | N | 41.80 | 1473.6835 | -0.6 | 737.8486 | 2 | 12.66 | 1896 | 1 | 1030 | 1042 |  |
| W.LDKNKDPLNDSVVQ.L | N | 41.51 | 1583.8154 | -4.2 | 792.9117 | 2 | 15.56 | 2391 | 8 | 598 | 611 |  |
| P.MNPPKYDKIEDM.A | N | 39.50 | 1479.6737 | -0.8 | 740.8436 | 2 | 11.36 | 1664 | 1 | 82 | 93 |  |
| T.VKEDDIHPM.N | N | 31.40 | 1082.5066 | -0.8 | 542.2601 | 2 | 10.15 | 1457 | 1 | 74 | 82 |  |
| Q.LGETGKT.I | N | 30.06 | 704.3705 | -0.2 | 353.1924 | 2 | 8.67 | 1226 | 1 | 1519 | 1525 |  |
| K.KMEGDLNEM.E | N | 28.74 | 1065.4470 | -0.5 | 533.7305 | 2 | 10.99 | 1598 | 1 | 1620 | 1628 |  |
| M.NPPKYDKIEDM.A | N | 26.69 | 1348.6333 | -0.3 | 675.3237 | 2 | 10.87 | 1577 | 1 | 83 | 93 |  |
| E.LKKEQDTSAH.L | N | 16.64 | 1155.5884 | 0.0 | 578.8015 | 2 | 7.73 | 1044 | 1 | 1772 | 1781 |  |
| N.FTNEKLQQF.F | N | 16.00 | 1153.5768 | 0.0 | 577.7957 | 2 | 11.46 | 1682 | 1 | 484 | 492 |  |
| total 9 peptides |  |  |  |  |  |  |  |  |  |  |  |  |
| --- | --- | --- | --- | --- | --- | --- | --- | --- | --- | --- | --- | --- |

tr|A0A6P8G2F8|A0A6P8G2F8\_CLUHA

back to list

  

| Protein Coverage
| Supporting Peptides
|

Protein Coverage:

Supporting Peptides:

| Peptide | Uniq | -10lgP | Mass | ppm | m/z | z | RT | Scan | #Spec | Start | End | PTM |
| --- | --- | --- | --- | --- | --- | --- | --- | --- | --- | --- | --- | --- |
| K.LEQQVDDLEGSLE.Q | N | 41.80 | 1473.6835 | -0.6 | 737.8486 | 2 | 12.66 | 1896 | 1 | 948 | 960 |  |
| W.LDKNKDPLNDSVVQ.L | N | 41.51 | 1583.8154 | -4.2 | 792.9117 | 2 | 15.56 | 2391 | 8 | 519 | 532 |  |
| MNPPKYDKIEDM.A | N | 39.50 | 1479.6737 | -0.8 | 740.8436 | 2 | 11.36 | 1664 | 1 | 1 | 12 |  |
| S.VIPEGQF.I | N | 30.48 | 788.4068 | -0.7 | 395.2104 | 2 | 12.26 | 1825 | 2 | 650 | 656 |  |
| K.KMEGDLNEM.E | N | 28.74 | 1065.4470 | -0.5 | 533.7305 | 2 | 10.99 | 1598 | 1 | 1538 | 1546 |  |
| M.NPPKYDKIEDM.A | N | 26.69 | 1348.6333 | -0.3 | 675.3237 | 2 | 10.87 | 1577 | 1 | 2 | 12 |  |
| L.SKIEDEQSL.G | N | 26.50 | 1047.5084 | -0.3 | 524.7614 | 2 | 10.37 | 1494 | 1 | 1013 | 1021 |  |
| L.SKIEDEQSLGAQ.L | N | 20.57 | 1303.6255 | -0.3 | 652.8198 | 2 | 10.23 | 1470 | 1 | 1013 | 1024 |  |
| N.FTNEKLQQF.F | N | 16.00 | 1153.5768 | 0.0 | 577.7957 | 2 | 11.46 | 1682 | 1 | 405 | 413 |  |
| total 9 peptides |  |  |  |  |  |  |  |  |  |  |  |  |
| --- | --- | --- | --- | --- | --- | --- | --- | --- | --- | --- | --- | --- |

tr|A0A6P8G1S8|A0A6P8G1S8\_CLUHA

back to list

  

| Protein Coverage
| Supporting Peptides
|

Protein Coverage:

Supporting Peptides:

| Peptide | Uniq | -10lgP | Mass | ppm | m/z | z | RT | Scan | #Spec | Start | End | PTM |
| --- | --- | --- | --- | --- | --- | --- | --- | --- | --- | --- | --- | --- |
| K.LEQQVDDLEGSLE.Q | N | 41.80 | 1473.6835 | -0.6 | 737.8486 | 2 | 12.66 | 1896 | 1 | 949 | 961 |  |
| W.LDKNKDPLNDSVVQ.L | N | 41.51 | 1583.8154 | -4.2 | 792.9117 | 2 | 15.56 | 2391 | 8 | 520 | 533 |  |
| MNPPKYDKIEDM.A | N | 39.50 | 1479.6737 | -0.8 | 740.8436 | 2 | 11.36 | 1664 | 1 | 1 | 12 |  |
| S.VIPEGQF.I | N | 30.48 | 788.4068 | -0.7 | 395.2104 | 2 | 12.26 | 1825 | 2 | 651 | 657 |  |
| K.KMEGDLNEM.E | N | 28.74 | 1065.4470 | -0.5 | 533.7305 | 2 | 10.99 | 1598 | 1 | 1539 | 1547 |  |
| M.NPPKYDKIEDM.A | N | 26.69 | 1348.6333 | -0.3 | 675.3237 | 2 | 10.87 | 1577 | 1 | 2 | 12 |  |
| L.SKIEDEQSL.G | N | 26.50 | 1047.5084 | -0.3 | 524.7614 | 2 | 10.37 | 1494 | 1 | 1014 | 1022 |  |
| L.SKIEDEQSLGAQ.L | N | 20.57 | 1303.6255 | -0.3 | 652.8198 | 2 | 10.23 | 1470 | 1 | 1014 | 1025 |  |
| N.FTNEKLQQF.F | N | 16.00 | 1153.5768 | 0.0 | 577.7957 | 2 | 11.46 | 1682 | 1 | 406 | 414 |  |
| total 9 peptides |  |  |  |  |  |  |  |  |  |  |  |  |
| --- | --- | --- | --- | --- | --- | --- | --- | --- | --- | --- | --- | --- |

tr|A0A2U9CMP6|A0A2U9CMP6\_SCOMX

back to list

  

| Protein Coverage
| Supporting Peptides
|

Protein Coverage:

Supporting Peptides:

| Peptide | Uniq | -10lgP | Mass | ppm | m/z | z | RT | Scan | #Spec | Start | End | PTM |
| --- | --- | --- | --- | --- | --- | --- | --- | --- | --- | --- | --- | --- |
| Y.VIDQDKSGFIE.E | N | 52.45 | 1249.6190 | -0.5 | 625.8165 | 2 | 11.38 | 1669 | 4 | 50 | 60 |  |
| Y.VIDQDKSGFIEEEE.L | Y | 43.10 | 1636.7467 | -0.4 | 819.3803 | 2 | 11.51 | 1691 | 1 | 50 | 63 |  |
| Y.VIDQDKSGFIEEEELK.L | Y | 33.74 | 1877.9258 | -1.1 | 939.9692 | 2 | 11.62 | 1710 | 2 | 50 | 65 |  |
| F.IEEEELKLF.L | N | 30.75 | 1148.5964 | -0.3 | 575.3053 | 2 | 13.44 | 2031 | 1 | 59 | 67 |  |
| Y.VIDQDKSGFIEEEELKLF.L | Y | 28.04 | 2138.0781 | -1.0 | 1070.0453 | 2 | 13.82 | 2095 | 1 | 50 | 67 |  |
| total 5 peptides |  |  |  |  |  |  |  |  |  |  |  |  |
| --- | --- | --- | --- | --- | --- | --- | --- | --- | --- | --- | --- | --- |

tr|A0A6A4RT91|A0A6A4RT91\_SCOMX

back to list

  

| Protein Coverage
| Supporting Peptides
|

Protein Coverage:

Supporting Peptides:

| Peptide | Uniq | -10lgP | Mass | ppm | m/z | z | RT | Scan | #Spec | Start | End | PTM |
| --- | --- | --- | --- | --- | --- | --- | --- | --- | --- | --- | --- | --- |
| Y.VIDQDKSGFIE.E | N | 52.45 | 1249.6190 | -0.5 | 625.8165 | 2 | 11.38 | 1669 | 4 | 214 | 224 |  |
| Y.VIDQDKSGFIEEEE.L | Y | 43.10 | 1636.7467 | -0.4 | 819.3803 | 2 | 11.51 | 1691 | 1 | 214 | 227 |  |
| Y.VIDQDKSGFIEEEELK.L | Y | 33.74 | 1877.9258 | -1.1 | 939.9692 | 2 | 11.62 | 1710 | 2 | 214 | 229 |  |
| F.IEEEELKLF.L | N | 30.75 | 1148.5964 | -0.3 | 575.3053 | 2 | 13.44 | 2031 | 1 | 223 | 231 |  |
| Y.VIDQDKSGFIEEEELKLF.L | Y | 28.04 | 2138.0781 | -1.0 | 1070.0453 | 2 | 13.82 | 2095 | 1 | 214 | 231 |  |
| total 5 peptides |  |  |  |  |  |  |  |  |  |  |  |  |
| --- | --- | --- | --- | --- | --- | --- | --- | --- | --- | --- | --- | --- |

tr|A0A3B3XMC4|A0A3B3XMC4\_9TELE

back to list

  

| Protein Coverage
| Supporting Peptides
|

Protein Coverage:

Supporting Peptides:

| Peptide | Uniq | -10lgP | Mass | ppm | m/z | z | RT | Scan | #Spec | Start | End | PTM |
| --- | --- | --- | --- | --- | --- | --- | --- | --- | --- | --- | --- | --- |
| S.VLDQDKSGFIE.E | N | 52.45 | 1249.6190 | -0.5 | 625.8165 | 2 | 11.38 | 1669 | 4 | 50 | 60 |  |
| S.VLDQDKSGFIEEEE.L | Y | 43.10 | 1636.7467 | -0.4 | 819.3803 | 2 | 11.51 | 1691 | 1 | 50 | 63 |  |
| S.VLDQDKSGFIEEEELK.L | Y | 33.74 | 1877.9258 | -1.1 | 939.9692 | 2 | 11.62 | 1710 | 2 | 50 | 65 |  |
| F.IEEEELKLF.L | N | 30.75 | 1148.5964 | -0.3 | 575.3053 | 2 | 13.44 | 2031 | 1 | 59 | 67 |  |
| S.VLDQDKSGFIEEEELKLF.L | Y | 28.04 | 2138.0781 | -1.0 | 1070.0453 | 2 | 13.82 | 2095 | 1 | 50 | 67 |  |
| total 5 peptides |  |  |  |  |  |  |  |  |  |  |  |  |
| --- | --- | --- | --- | --- | --- | --- | --- | --- | --- | --- | --- | --- |

tr|A0A3B5LQ20|A0A3B5LQ20\_9TELE

back to list

  

| Protein Coverage
| Supporting Peptides
|

Protein Coverage:

Supporting Peptides:

| Peptide | Uniq | -10lgP | Mass | ppm | m/z | z | RT | Scan | #Spec | Start | End | PTM |
| --- | --- | --- | --- | --- | --- | --- | --- | --- | --- | --- | --- | --- |
| T.VLDQDKSGFIE.E | N | 52.45 | 1249.6190 | -0.5 | 625.8165 | 2 | 11.38 | 1669 | 4 | 50 | 60 |  |
| T.VLDQDKSGFIEEEE.L | Y | 43.10 | 1636.7467 | -0.4 | 819.3803 | 2 | 11.51 | 1691 | 1 | 50 | 63 |  |
| T.VLDQDKSGFIEEEELK.L | Y | 33.74 | 1877.9258 | -1.1 | 939.9692 | 2 | 11.62 | 1710 | 2 | 50 | 65 |  |
| F.IEEEELKLF.L | N | 30.75 | 1148.5964 | -0.3 | 575.3053 | 2 | 13.44 | 2031 | 1 | 59 | 67 |  |
| T.VLDQDKSGFIEEEELKLF.L | Y | 28.04 | 2138.0781 | -1.0 | 1070.0453 | 2 | 13.82 | 2095 | 1 | 50 | 67 |  |
| total 5 peptides |  |  |  |  |  |  |  |  |  |  |  |  |
| --- | --- | --- | --- | --- | --- | --- | --- | --- | --- | --- | --- | --- |

tr|A0A3B3Z292|A0A3B3Z292\_9TELE

back to list

  

| Protein Coverage
| Supporting Peptides
|

Protein Coverage:

Supporting Peptides:

| Peptide | Uniq | -10lgP | Mass | ppm | m/z | z | RT | Scan | #Spec | Start | End | PTM |
| --- | --- | --- | --- | --- | --- | --- | --- | --- | --- | --- | --- | --- |
| S.VLDQDKSGFIE.E | N | 52.45 | 1249.6190 | -0.5 | 625.8165 | 2 | 11.38 | 1669 | 4 | 48 | 58 |  |
| S.VLDQDKSGFIEEEE.L | Y | 43.10 | 1636.7467 | -0.4 | 819.3803 | 2 | 11.51 | 1691 | 1 | 48 | 61 |  |
| S.VLDQDKSGFIEEEELK.L | Y | 33.74 | 1877.9258 | -1.1 | 939.9692 | 2 | 11.62 | 1710 | 2 | 48 | 63 |  |
| F.IEEEELKLF.L | N | 30.75 | 1148.5964 | -0.3 | 575.3053 | 2 | 13.44 | 2031 | 1 | 57 | 65 |  |
| S.VLDQDKSGFIEEEELKLF.L | Y | 28.04 | 2138.0781 | -1.0 | 1070.0453 | 2 | 13.82 | 2095 | 1 | 48 | 65 |  |
| total 5 peptides |  |  |  |  |  |  |  |  |  |  |  |  |
| --- | --- | --- | --- | --- | --- | --- | --- | --- | --- | --- | --- | --- |

tr|A0A671XKN2|A0A671XKN2\_SPAAU

back to list

  

| Protein Coverage
| Supporting Peptides
|

Protein Coverage:

Supporting Peptides:

| Peptide | Uniq | -10lgP | Mass | ppm | m/z | z | RT | Scan | #Spec | Start | End | PTM |
| --- | --- | --- | --- | --- | --- | --- | --- | --- | --- | --- | --- | --- |
| A.VIDQDKSGFIE.E | N | 52.45 | 1249.6190 | -0.5 | 625.8165 | 2 | 11.38 | 1669 | 4 | 48 | 58 |  |
| A.VIDQDKSGFIEEEE.L | Y | 43.10 | 1636.7467 | -0.4 | 819.3803 | 2 | 11.51 | 1691 | 1 | 48 | 61 |  |
| A.VIDQDKSGFIEEEELK.L | Y | 33.74 | 1877.9258 | -1.1 | 939.9692 | 2 | 11.62 | 1710 | 2 | 48 | 63 |  |
| F.IEEEELKLF.L | N | 30.75 | 1148.5964 | -0.3 | 575.3053 | 2 | 13.44 | 2031 | 1 | 57 | 65 |  |
| A.VIDQDKSGFIEEEELKLF.L | Y | 28.04 | 2138.0781 | -1.0 | 1070.0453 | 2 | 13.82 | 2095 | 1 | 48 | 65 |  |
| total 5 peptides |  |  |  |  |  |  |  |  |  |  |  |  |
| --- | --- | --- | --- | --- | --- | --- | --- | --- | --- | --- | --- | --- |

tr|A0A672Z6Y0|A0A672Z6Y0\_9TELE

back to list

  

| Protein Coverage
| Supporting Peptides
|

Protein Coverage:

Supporting Peptides:

| Peptide | Uniq | -10lgP | Mass | ppm | m/z | z | RT | Scan | #Spec | Start | End | PTM |
| --- | --- | --- | --- | --- | --- | --- | --- | --- | --- | --- | --- | --- |
| A.VIDQDKSGFIE.E | N | 52.45 | 1249.6190 | -0.5 | 625.8165 | 2 | 11.38 | 1669 | 4 | 50 | 60 |  |
| A.VIDQDKSGFIEEEE.L | Y | 43.10 | 1636.7467 | -0.4 | 819.3803 | 2 | 11.51 | 1691 | 1 | 50 | 63 |  |
| A.VIDQDKSGFIEEEELK.L | Y | 33.74 | 1877.9258 | -1.1 | 939.9692 | 2 | 11.62 | 1710 | 2 | 50 | 65 |  |
| F.IEEEELKLF.L | N | 30.75 | 1148.5964 | -0.3 | 575.3053 | 2 | 13.44 | 2031 | 1 | 59 | 67 |  |
| A.VIDQDKSGFIEEEELKLF.L | Y | 28.04 | 2138.0781 | -1.0 | 1070.0453 | 2 | 13.82 | 2095 | 1 | 50 | 67 |  |
| total 5 peptides |  |  |  |  |  |  |  |  |  |  |  |  |
| --- | --- | --- | --- | --- | --- | --- | --- | --- | --- | --- | --- | --- |

tr|D0VB96|D0VB96\_SPAAU

back to list

  

| Protein Coverage
| Supporting Peptides
|

Protein Coverage:

Supporting Peptides:

| Peptide | Uniq | -10lgP | Mass | ppm | m/z | z | RT | Scan | #Spec | Start | End | PTM |
| --- | --- | --- | --- | --- | --- | --- | --- | --- | --- | --- | --- | --- |
| A.VIDQDKSGFIE.E | N | 52.45 | 1249.6190 | -0.5 | 625.8165 | 2 | 11.38 | 1669 | 4 | 49 | 59 |  |
| A.VIDQDKSGFIEEEE.L | Y | 43.10 | 1636.7467 | -0.4 | 819.3803 | 2 | 11.51 | 1691 | 1 | 49 | 62 |  |
| A.VIDQDKSGFIEEEELK.L | Y | 33.74 | 1877.9258 | -1.1 | 939.9692 | 2 | 11.62 | 1710 | 2 | 49 | 64 |  |
| F.IEEEELKLF.L | N | 30.75 | 1148.5964 | -0.3 | 575.3053 | 2 | 13.44 | 2031 | 1 | 58 | 66 |  |
| A.VIDQDKSGFIEEEELKLF.L | Y | 28.04 | 2138.0781 | -1.0 | 1070.0453 | 2 | 13.82 | 2095 | 1 | 49 | 66 |  |
| total 5 peptides |  |  |  |  |  |  |  |  |  |  |  |  |
| --- | --- | --- | --- | --- | --- | --- | --- | --- | --- | --- | --- | --- |

tr|A0A087Y091|A0A087Y091\_POEFO

back to list

  

| Protein Coverage
| Supporting Peptides
|

Protein Coverage:

Supporting Peptides:

| Peptide | Uniq | -10lgP | Mass | ppm | m/z | z | RT | Scan | #Spec | Start | End | PTM |
| --- | --- | --- | --- | --- | --- | --- | --- | --- | --- | --- | --- | --- |
| T.VLDQDKSGFIE.E | N | 52.45 | 1249.6190 | -0.5 | 625.8165 | 2 | 11.38 | 1669 | 4 | 50 | 60 |  |
| T.VLDQDKSGFIEEEE.L | Y | 43.10 | 1636.7467 | -0.4 | 819.3803 | 2 | 11.51 | 1691 | 1 | 50 | 63 |  |
| T.VLDQDKSGFIEEEELK.L | Y | 33.74 | 1877.9258 | -1.1 | 939.9692 | 2 | 11.62 | 1710 | 2 | 50 | 65 |  |
| F.IEEEELKLF.L | N | 30.75 | 1148.5964 | -0.3 | 575.3053 | 2 | 13.44 | 2031 | 1 | 59 | 67 |  |
| T.VLDQDKSGFIEEEELKLF.L | Y | 28.04 | 2138.0781 | -1.0 | 1070.0453 | 2 | 13.82 | 2095 | 1 | 50 | 67 |  |
| total 5 peptides |  |  |  |  |  |  |  |  |  |  |  |  |
| --- | --- | --- | --- | --- | --- | --- | --- | --- | --- | --- | --- | --- |

tr|A0A5N5MFD7|A0A5N5MFD7\_PANHP

back to list

  

| Protein Coverage
| Supporting Peptides
|

Protein Coverage:

Supporting Peptides:

| Peptide | Uniq | -10lgP | Mass | ppm | m/z | z | RT | Scan | #Spec | Start | End | PTM |
| --- | --- | --- | --- | --- | --- | --- | --- | --- | --- | --- | --- | --- |
| K.VLDQDKSGFIE.E | N | 52.45 | 1249.6190 | -0.5 | 625.8165 | 2 | 11.38 | 1669 | 4 | 50 | 60 |  |
| K.VLDQDKSGFIEEEE.L | Y | 43.10 | 1636.7467 | -0.4 | 819.3803 | 2 | 11.51 | 1691 | 1 | 50 | 63 |  |
| K.VLDQDKSGFIEEEELK.L | Y | 33.74 | 1877.9258 | -1.1 | 939.9692 | 2 | 11.62 | 1710 | 2 | 50 | 65 |  |
| F.IEEEELKLF.L | N | 30.75 | 1148.5964 | -0.3 | 575.3053 | 2 | 13.44 | 2031 | 1 | 59 | 67 |  |
| K.VLDQDKSGFIEEEELKLF.L | Y | 28.04 | 2138.0781 | -1.0 | 1070.0453 | 2 | 13.82 | 2095 | 1 | 50 | 67 |  |
| total 5 peptides |  |  |  |  |  |  |  |  |  |  |  |  |
| --- | --- | --- | --- | --- | --- | --- | --- | --- | --- | --- | --- | --- |

tr|A0A087Y0W0|A0A087Y0W0\_POEFO

back to list

  

| Protein Coverage
| Supporting Peptides
|

Protein Coverage:

Supporting Peptides:

| Peptide | Uniq | -10lgP | Mass | ppm | m/z | z | RT | Scan | #Spec | Start | End | PTM |
| --- | --- | --- | --- | --- | --- | --- | --- | --- | --- | --- | --- | --- |
| T.VLDQDKSGFIE.E | N | 52.45 | 1249.6190 | -0.5 | 625.8165 | 2 | 11.38 | 1669 | 4 | 50 | 60 |  |
| T.VLDQDKSGFIEEEE.L | Y | 43.10 | 1636.7467 | -0.4 | 819.3803 | 2 | 11.51 | 1691 | 1 | 50 | 63 |  |
| T.VLDQDKSGFIEEEELK.L | Y | 33.74 | 1877.9258 | -1.1 | 939.9692 | 2 | 11.62 | 1710 | 2 | 50 | 65 |  |
| F.IEEEELKLF.L | N | 30.75 | 1148.5964 | -0.3 | 575.3053 | 2 | 13.44 | 2031 | 1 | 59 | 67 |  |
| T.VLDQDKSGFIEEEELKLF.L | Y | 28.04 | 2138.0781 | -1.0 | 1070.0453 | 2 | 13.82 | 2095 | 1 | 50 | 67 |  |
| total 5 peptides |  |  |  |  |  |  |  |  |  |  |  |  |
| --- | --- | --- | --- | --- | --- | --- | --- | --- | --- | --- | --- | --- |

tr|A0A668AEA1|A0A668AEA1\_9TELE

back to list

  

| Protein Coverage
| Supporting Peptides
|

Protein Coverage:

Supporting Peptides:

| Peptide | Uniq | -10lgP | Mass | ppm | m/z | z | RT | Scan | #Spec | Start | End | PTM |
| --- | --- | --- | --- | --- | --- | --- | --- | --- | --- | --- | --- | --- |
| Y.VIDQDKSGFIE.E | N | 52.45 | 1249.6190 | -0.5 | 625.8165 | 2 | 11.38 | 1669 | 4 | 50 | 60 |  |
| Y.VIDQDKSGFIEEEE.L | Y | 43.10 | 1636.7467 | -0.4 | 819.3803 | 2 | 11.51 | 1691 | 1 | 50 | 63 |  |
| Y.VIDQDKSGFIEEEELK.L | Y | 33.74 | 1877.9258 | -1.1 | 939.9692 | 2 | 11.62 | 1710 | 2 | 50 | 65 |  |
| F.IEEEELKLF.L | N | 30.75 | 1148.5964 | -0.3 | 575.3053 | 2 | 13.44 | 2031 | 1 | 59 | 67 |  |
| Y.VIDQDKSGFIEEEELKLF.L | Y | 28.04 | 2138.0781 | -1.0 | 1070.0453 | 2 | 13.82 | 2095 | 1 | 50 | 67 |  |
| total 5 peptides |  |  |  |  |  |  |  |  |  |  |  |  |
| --- | --- | --- | --- | --- | --- | --- | --- | --- | --- | --- | --- | --- |

tr|C6GKU4|C6GKU4\_9TELE

back to list

  

| Protein Coverage
| Supporting Peptides
|

Protein Coverage:

Supporting Peptides:

| Peptide | Uniq | -10lgP | Mass | ppm | m/z | z | RT | Scan | #Spec | Start | End | PTM |
| --- | --- | --- | --- | --- | --- | --- | --- | --- | --- | --- | --- | --- |
| K.VIDQDKSGFIE.E | N | 52.45 | 1249.6190 | -0.5 | 625.8165 | 2 | 11.38 | 1669 | 4 | 50 | 60 |  |
| K.VIDQDKSGFIEEEE.L | Y | 43.10 | 1636.7467 | -0.4 | 819.3803 | 2 | 11.51 | 1691 | 1 | 50 | 63 |  |
| K.VIDQDKSGFIEEEELK.L | Y | 33.74 | 1877.9258 | -1.1 | 939.9692 | 2 | 11.62 | 1710 | 2 | 50 | 65 |  |
| F.IEEEELKLF.L | N | 30.75 | 1148.5964 | -0.3 | 575.3053 | 2 | 13.44 | 2031 | 1 | 59 | 67 |  |
| K.VIDQDKSGFIEEEELKLF.L | Y | 28.04 | 2138.0781 | -1.0 | 1070.0453 | 2 | 13.82 | 2095 | 1 | 50 | 67 |  |
| total 5 peptides |  |  |  |  |  |  |  |  |  |  |  |  |
| --- | --- | --- | --- | --- | --- | --- | --- | --- | --- | --- | --- | --- |

tr|A0A087Y5E9|A0A087Y5E9\_POEFO

back to list

  

| Protein Coverage
| Supporting Peptides
|

Protein Coverage:

Supporting Peptides:

| Peptide | Uniq | -10lgP | Mass | ppm | m/z | z | RT | Scan | #Spec | Start | End | PTM |
| --- | --- | --- | --- | --- | --- | --- | --- | --- | --- | --- | --- | --- |
| A.VIDQDKSGFIE.E | N | 52.45 | 1249.6190 | -0.5 | 625.8165 | 2 | 11.38 | 1669 | 4 | 50 | 60 |  |
| A.VIDQDKSGFIEEEE.L | Y | 43.10 | 1636.7467 | -0.4 | 819.3803 | 2 | 11.51 | 1691 | 1 | 50 | 63 |  |
| A.VIDQDKSGFIEEEELK.L | Y | 33.74 | 1877.9258 | -1.1 | 939.9692 | 2 | 11.62 | 1710 | 2 | 50 | 65 |  |
| F.IEEEELKLF.L | N | 30.75 | 1148.5964 | -0.3 | 575.3053 | 2 | 13.44 | 2031 | 1 | 59 | 67 |  |
| A.VIDQDKSGFIEEEELKLF.L | Y | 28.04 | 2138.0781 | -1.0 | 1070.0453 | 2 | 13.82 | 2095 | 1 | 50 | 67 |  |
| total 5 peptides |  |  |  |  |  |  |  |  |  |  |  |  |
| --- | --- | --- | --- | --- | --- | --- | --- | --- | --- | --- | --- | --- |

tr|A0A1A8GBU0|A0A1A8GBU0\_9TELE

back to list

  

| Protein Coverage
| Supporting Peptides
|

Protein Coverage:

Supporting Peptides:

| Peptide | Uniq | -10lgP | Mass | ppm | m/z | z | RT | Scan | #Spec | Start | End | PTM |
| --- | --- | --- | --- | --- | --- | --- | --- | --- | --- | --- | --- | --- |
| A.VIDQDKSGFIE.E | N | 52.45 | 1249.6190 | -0.5 | 625.8165 | 2 | 11.38 | 1669 | 4 | 50 | 60 |  |
| A.VIDQDKSGFIEEEE.L | Y | 43.10 | 1636.7467 | -0.4 | 819.3803 | 2 | 11.51 | 1691 | 1 | 50 | 63 |  |
| A.VIDQDKSGFIEEEELK.L | Y | 33.74 | 1877.9258 | -1.1 | 939.9692 | 2 | 11.62 | 1710 | 2 | 50 | 65 |  |
| F.IEEEELKLF.L | N | 30.75 | 1148.5964 | -0.3 | 575.3053 | 2 | 13.44 | 2031 | 1 | 59 | 67 |  |
| A.VIDQDKSGFIEEEELKLF.L | Y | 28.04 | 2138.0781 | -1.0 | 1070.0453 | 2 | 13.82 | 2095 | 1 | 50 | 67 |  |
| total 5 peptides |  |  |  |  |  |  |  |  |  |  |  |  |
| --- | --- | --- | --- | --- | --- | --- | --- | --- | --- | --- | --- | --- |

tr|E0WD96|E0WD96\_9TELE

back to list

  

| Protein Coverage
| Supporting Peptides
|

Protein Coverage:

Supporting Peptides:

| Peptide | Uniq | -10lgP | Mass | ppm | m/z | z | RT | Scan | #Spec | Start | End | PTM |
| --- | --- | --- | --- | --- | --- | --- | --- | --- | --- | --- | --- | --- |
| K.VIDQDKSGFIE.E | N | 52.45 | 1249.6190 | -0.5 | 625.8165 | 2 | 11.38 | 1669 | 4 | 50 | 60 |  |
| K.VIDQDKSGFIEEEE.L | Y | 43.10 | 1636.7467 | -0.4 | 819.3803 | 2 | 11.51 | 1691 | 1 | 50 | 63 |  |
| K.VIDQDKSGFIEEEELK.L | Y | 33.74 | 1877.9258 | -1.1 | 939.9692 | 2 | 11.62 | 1710 | 2 | 50 | 65 |  |
| F.IEEEELKLF.L | N | 30.75 | 1148.5964 | -0.3 | 575.3053 | 2 | 13.44 | 2031 | 1 | 59 | 67 |  |
| K.VIDQDKSGFIEEEELKLF.L | Y | 28.04 | 2138.0781 | -1.0 | 1070.0453 | 2 | 13.82 | 2095 | 1 | 50 | 67 |  |
| total 5 peptides |  |  |  |  |  |  |  |  |  |  |  |  |
| --- | --- | --- | --- | --- | --- | --- | --- | --- | --- | --- | --- | --- |

tr|A0A1A7WES6|A0A1A7WES6\_9TELE

back to list

  

| Protein Coverage
| Supporting Peptides
|

Protein Coverage:

Supporting Peptides:

| Peptide | Uniq | -10lgP | Mass | ppm | m/z | z | RT | Scan | #Spec | Start | End | PTM |
| --- | --- | --- | --- | --- | --- | --- | --- | --- | --- | --- | --- | --- |
| A.VIDQDKSGFIE.E | N | 52.45 | 1249.6190 | -0.5 | 625.8165 | 2 | 11.38 | 1669 | 4 | 50 | 60 |  |
| A.VIDQDKSGFIEEEE.L | Y | 43.10 | 1636.7467 | -0.4 | 819.3803 | 2 | 11.51 | 1691 | 1 | 50 | 63 |  |
| A.VIDQDKSGFIEEEELK.L | Y | 33.74 | 1877.9258 | -1.1 | 939.9692 | 2 | 11.62 | 1710 | 2 | 50 | 65 |  |
| F.IEEEELKLF.L | N | 30.75 | 1148.5964 | -0.3 | 575.3053 | 2 | 13.44 | 2031 | 1 | 59 | 67 |  |
| A.VIDQDKSGFIEEEELKLF.L | Y | 28.04 | 2138.0781 | -1.0 | 1070.0453 | 2 | 13.82 | 2095 | 1 | 50 | 67 |  |
| total 5 peptides |  |  |  |  |  |  |  |  |  |  |  |  |
| --- | --- | --- | --- | --- | --- | --- | --- | --- | --- | --- | --- | --- |

tr|A0A1A8EAV3|A0A1A8EAV3\_9TELE

back to list

  

| Protein Coverage
| Supporting Peptides
|

Protein Coverage:

Supporting Peptides:

| Peptide | Uniq | -10lgP | Mass | ppm | m/z | z | RT | Scan | #Spec | Start | End | PTM |
| --- | --- | --- | --- | --- | --- | --- | --- | --- | --- | --- | --- | --- |
| A.VIDQDKSGFIE.E | N | 52.45 | 1249.6190 | -0.5 | 625.8165 | 2 | 11.38 | 1669 | 4 | 50 | 60 |  |
| A.VIDQDKSGFIEEEE.L | Y | 43.10 | 1636.7467 | -0.4 | 819.3803 | 2 | 11.51 | 1691 | 1 | 50 | 63 |  |
| A.VIDQDKSGFIEEEELK.L | Y | 33.74 | 1877.9258 | -1.1 | 939.9692 | 2 | 11.62 | 1710 | 2 | 50 | 65 |  |
| F.IEEEELKLF.L | N | 30.75 | 1148.5964 | -0.3 | 575.3053 | 2 | 13.44 | 2031 | 1 | 59 | 67 |  |
| A.VIDQDKSGFIEEEELKLF.L | Y | 28.04 | 2138.0781 | -1.0 | 1070.0453 | 2 | 13.82 | 2095 | 1 | 50 | 67 |  |
| total 5 peptides |  |  |  |  |  |  |  |  |  |  |  |  |
| --- | --- | --- | --- | --- | --- | --- | --- | --- | --- | --- | --- | --- |

tr|A0A1A8PM62|A0A1A8PM62\_9TELE

back to list

  

| Protein Coverage
| Supporting Peptides
|

Protein Coverage:

Supporting Peptides:

| Peptide | Uniq | -10lgP | Mass | ppm | m/z | z | RT | Scan | #Spec | Start | End | PTM |
| --- | --- | --- | --- | --- | --- | --- | --- | --- | --- | --- | --- | --- |
| A.VIDQDKSGFIE.E | N | 52.45 | 1249.6190 | -0.5 | 625.8165 | 2 | 11.38 | 1669 | 4 | 50 | 60 |  |
| A.VIDQDKSGFIEEEE.L | Y | 43.10 | 1636.7467 | -0.4 | 819.3803 | 2 | 11.51 | 1691 | 1 | 50 | 63 |  |
| A.VIDQDKSGFIEEEELK.L | Y | 33.74 | 1877.9258 | -1.1 | 939.9692 | 2 | 11.62 | 1710 | 2 | 50 | 65 |  |
| F.IEEEELKLF.L | N | 30.75 | 1148.5964 | -0.3 | 575.3053 | 2 | 13.44 | 2031 | 1 | 59 | 67 |  |
| A.VIDQDKSGFIEEEELKLF.L | Y | 28.04 | 2138.0781 | -1.0 | 1070.0453 | 2 | 13.82 | 2095 | 1 | 50 | 67 |  |
| total 5 peptides |  |  |  |  |  |  |  |  |  |  |  |  |
| --- | --- | --- | --- | --- | --- | --- | --- | --- | --- | --- | --- | --- |

tr|A0A3B3TQ55|A0A3B3TQ55\_9TELE

back to list

  

| Protein Coverage
| Supporting Peptides
|

Protein Coverage:

Supporting Peptides:

| Peptide | Uniq | -10lgP | Mass | ppm | m/z | z | RT | Scan | #Spec | Start | End | PTM |
| --- | --- | --- | --- | --- | --- | --- | --- | --- | --- | --- | --- | --- |
| A.VIDQDKSGFIE.E | N | 52.45 | 1249.6190 | -0.5 | 625.8165 | 2 | 11.38 | 1669 | 4 | 50 | 60 |  |
| A.VIDQDKSGFIEEEE.L | Y | 43.10 | 1636.7467 | -0.4 | 819.3803 | 2 | 11.51 | 1691 | 1 | 50 | 63 |  |
| A.VIDQDKSGFIEEEELK.L | Y | 33.74 | 1877.9258 | -1.1 | 939.9692 | 2 | 11.62 | 1710 | 2 | 50 | 65 |  |
| F.IEEEELKLF.L | N | 30.75 | 1148.5964 | -0.3 | 575.3053 | 2 | 13.44 | 2031 | 1 | 59 | 67 |  |
| A.VIDQDKSGFIEEEELKLF.L | Y | 28.04 | 2138.0781 | -1.0 | 1070.0453 | 2 | 13.82 | 2095 | 1 | 50 | 67 |  |
| total 5 peptides |  |  |  |  |  |  |  |  |  |  |  |  |
| --- | --- | --- | --- | --- | --- | --- | --- | --- | --- | --- | --- | --- |

tr|A0A1A8JGR3|A0A1A8JGR3\_NOTKU

back to list

  

| Protein Coverage
| Supporting Peptides
|

Protein Coverage:

Supporting Peptides:

| Peptide | Uniq | -10lgP | Mass | ppm | m/z | z | RT | Scan | #Spec | Start | End | PTM |
| --- | --- | --- | --- | --- | --- | --- | --- | --- | --- | --- | --- | --- |
| A.VIDQDKSGFIE.E | N | 52.45 | 1249.6190 | -0.5 | 625.8165 | 2 | 11.38 | 1669 | 4 | 50 | 60 |  |
| A.VIDQDKSGFIEEEE.L | Y | 43.10 | 1636.7467 | -0.4 | 819.3803 | 2 | 11.51 | 1691 | 1 | 50 | 63 |  |
| A.VIDQDKSGFIEEEELK.L | Y | 33.74 | 1877.9258 | -1.1 | 939.9692 | 2 | 11.62 | 1710 | 2 | 50 | 65 |  |
| F.IEEEELKLF.L | N | 30.75 | 1148.5964 | -0.3 | 575.3053 | 2 | 13.44 | 2031 | 1 | 59 | 67 |  |
| A.VIDQDKSGFIEEEELKLF.L | Y | 28.04 | 2138.0781 | -1.0 | 1070.0453 | 2 | 13.82 | 2095 | 1 | 50 | 67 |  |
| total 5 peptides |  |  |  |  |  |  |  |  |  |  |  |  |
| --- | --- | --- | --- | --- | --- | --- | --- | --- | --- | --- | --- | --- |

tr|A0A3B3XME4|A0A3B3XME4\_9TELE

back to list

  

| Protein Coverage
| Supporting Peptides
|

Protein Coverage:

Supporting Peptides:

| Peptide | Uniq | -10lgP | Mass | ppm | m/z | z | RT | Scan | #Spec | Start | End | PTM |
| --- | --- | --- | --- | --- | --- | --- | --- | --- | --- | --- | --- | --- |
| S.VLDQDKSGFIE.E | N | 52.45 | 1249.6190 | -0.5 | 625.8165 | 2 | 11.38 | 1669 | 4 | 50 | 60 |  |
| S.VLDQDKSGFIEEEE.L | Y | 43.10 | 1636.7467 | -0.4 | 819.3803 | 2 | 11.51 | 1691 | 1 | 50 | 63 |  |
| S.VLDQDKSGFIEEEELK.L | Y | 33.74 | 1877.9258 | -1.1 | 939.9692 | 2 | 11.62 | 1710 | 2 | 50 | 65 |  |
| F.IEEEELKLF.L | N | 30.75 | 1148.5964 | -0.3 | 575.3053 | 2 | 13.44 | 2031 | 1 | 59 | 67 |  |
| S.VLDQDKSGFIEEEELKLF.L | Y | 28.04 | 2138.0781 | -1.0 | 1070.0453 | 2 | 13.82 | 2095 | 1 | 50 | 67 |  |
| total 5 peptides |  |  |  |  |  |  |  |  |  |  |  |  |
| --- | --- | --- | --- | --- | --- | --- | --- | --- | --- | --- | --- | --- |

tr|A0A668AEA6|A0A668AEA6\_9TELE

back to list

  

| Protein Coverage
| Supporting Peptides
|

Protein Coverage:

Supporting Peptides:

| Peptide | Uniq | -10lgP | Mass | ppm | m/z | z | RT | Scan | #Spec | Start | End | PTM |
| --- | --- | --- | --- | --- | --- | --- | --- | --- | --- | --- | --- | --- |
| Y.VIDQDKSGFIE.E | N | 52.45 | 1249.6190 | -0.5 | 625.8165 | 2 | 11.38 | 1669 | 4 | 50 | 60 |  |
| Y.VIDQDKSGFIEEEE.L | Y | 43.10 | 1636.7467 | -0.4 | 819.3803 | 2 | 11.51 | 1691 | 1 | 50 | 63 |  |
| Y.VIDQDKSGFIEEEELK.L | Y | 33.74 | 1877.9258 | -1.1 | 939.9692 | 2 | 11.62 | 1710 | 2 | 50 | 65 |  |
| F.IEEEELKLF.L | N | 30.75 | 1148.5964 | -0.3 | 575.3053 | 2 | 13.44 | 2031 | 1 | 59 | 67 |  |
| Y.VIDQDKSGFIEEEELKLF.L | Y | 28.04 | 2138.0781 | -1.0 | 1070.0453 | 2 | 13.82 | 2095 | 1 | 50 | 67 |  |
| total 5 peptides |  |  |  |  |  |  |  |  |  |  |  |  |
| --- | --- | --- | --- | --- | --- | --- | --- | --- | --- | --- | --- | --- |

tr|A0A5N5MKE2|A0A5N5MKE2\_PANHP

back to list

  

| Protein Coverage
| Supporting Peptides
|

Protein Coverage:

Supporting Peptides:

| Peptide | Uniq | -10lgP | Mass | ppm | m/z | z | RT | Scan | #Spec | Start | End | PTM |
| --- | --- | --- | --- | --- | --- | --- | --- | --- | --- | --- | --- | --- |
| Y.VIDQDKSGFIE.E | N | 52.45 | 1249.6190 | -0.5 | 625.8165 | 2 | 11.38 | 1669 | 4 | 60 | 70 |  |
| Y.VIDQDKSGFIEEEE.L | Y | 43.10 | 1636.7467 | -0.4 | 819.3803 | 2 | 11.51 | 1691 | 1 | 60 | 73 |  |
| Y.VIDQDKSGFIEEEELK.L | Y | 33.74 | 1877.9258 | -1.1 | 939.9692 | 2 | 11.62 | 1710 | 2 | 60 | 75 |  |
| F.IEEEELKLF.L | N | 30.75 | 1148.5964 | -0.3 | 575.3053 | 2 | 13.44 | 2031 | 1 | 69 | 77 |  |
| Y.VIDQDKSGFIEEEELKLF.L | Y | 28.04 | 2138.0781 | -1.0 | 1070.0453 | 2 | 13.82 | 2095 | 1 | 60 | 77 |  |
| total 5 peptides |  |  |  |  |  |  |  |  |  |  |  |  |
| --- | --- | --- | --- | --- | --- | --- | --- | --- | --- | --- | --- | --- |

tr|A0A668ANA4|A0A668ANA4\_9TELE

back to list

  

| Protein Coverage
| Supporting Peptides
|

Protein Coverage:

Supporting Peptides:

| Peptide | Uniq | -10lgP | Mass | ppm | m/z | z | RT | Scan | #Spec | Start | End | PTM |
| --- | --- | --- | --- | --- | --- | --- | --- | --- | --- | --- | --- | --- |
| Y.VIDQDKSGFIE.E | N | 52.45 | 1249.6190 | -0.5 | 625.8165 | 2 | 11.38 | 1669 | 4 | 50 | 60 |  |
| Y.VIDQDKSGFIEEEE.L | Y | 43.10 | 1636.7467 | -0.4 | 819.3803 | 2 | 11.51 | 1691 | 1 | 50 | 63 |  |
| Y.VIDQDKSGFIEEEELK.L | Y | 33.74 | 1877.9258 | -1.1 | 939.9692 | 2 | 11.62 | 1710 | 2 | 50 | 65 |  |
| F.IEEEELKLF.L | N | 30.75 | 1148.5964 | -0.3 | 575.3053 | 2 | 13.44 | 2031 | 1 | 59 | 67 |  |
| Y.VIDQDKSGFIEEEELKLF.L | Y | 28.04 | 2138.0781 | -1.0 | 1070.0453 | 2 | 13.82 | 2095 | 1 | 50 | 67 |  |
| total 5 peptides |  |  |  |  |  |  |  |  |  |  |  |  |
| --- | --- | --- | --- | --- | --- | --- | --- | --- | --- | --- | --- | --- |

tr|A0A3B3UHS0|A0A3B3UHS0\_9TELE

back to list

  

| Protein Coverage
| Supporting Peptides
|

Protein Coverage:

Supporting Peptides:

| Peptide | Uniq | -10lgP | Mass | ppm | m/z | z | RT | Scan | #Spec | Start | End | PTM |
| --- | --- | --- | --- | --- | --- | --- | --- | --- | --- | --- | --- | --- |
| T.VLDQDKSGFIE.E | N | 52.45 | 1249.6190 | -0.5 | 625.8165 | 2 | 11.38 | 1669 | 4 | 50 | 60 |  |
| T.VLDQDKSGFIEEEE.L | Y | 43.10 | 1636.7467 | -0.4 | 819.3803 | 2 | 11.51 | 1691 | 1 | 50 | 63 |  |
| T.VLDQDKSGFIEEEELK.L | Y | 33.74 | 1877.9258 | -1.1 | 939.9692 | 2 | 11.62 | 1710 | 2 | 50 | 65 |  |
| F.IEEEELKLF.L | N | 30.75 | 1148.5964 | -0.3 | 575.3053 | 2 | 13.44 | 2031 | 1 | 59 | 67 |  |
| T.VLDQDKSGFIEEEELKLF.L | Y | 28.04 | 2138.0781 | -1.0 | 1070.0453 | 2 | 13.82 | 2095 | 1 | 50 | 67 |  |
| total 5 peptides |  |  |  |  |  |  |  |  |  |  |  |  |
| --- | --- | --- | --- | --- | --- | --- | --- | --- | --- | --- | --- | --- |

tr|A0A556TIT9|A0A556TIT9\_BAGYA

back to list

  

| Protein Coverage
| Supporting Peptides
|

Protein Coverage:

Supporting Peptides:

| Peptide | Uniq | -10lgP | Mass | ppm | m/z | z | RT | Scan | #Spec | Start | End | PTM |
| --- | --- | --- | --- | --- | --- | --- | --- | --- | --- | --- | --- | --- |
| F.VIDQDKSGFIE.E | N | 52.45 | 1249.6190 | -0.5 | 625.8165 | 2 | 11.38 | 1669 | 4 | 50 | 60 |  |
| F.VIDQDKSGFIEEEE.L | Y | 43.10 | 1636.7467 | -0.4 | 819.3803 | 2 | 11.51 | 1691 | 1 | 50 | 63 |  |
| F.VIDQDKSGFIEEEELK.L | Y | 33.74 | 1877.9258 | -1.1 | 939.9692 | 2 | 11.62 | 1710 | 2 | 50 | 65 |  |
| F.IEEEELKLF.L | N | 30.75 | 1148.5964 | -0.3 | 575.3053 | 2 | 13.44 | 2031 | 1 | 59 | 67 |  |
| F.VIDQDKSGFIEEEELKLF.L | Y | 28.04 | 2138.0781 | -1.0 | 1070.0453 | 2 | 13.82 | 2095 | 1 | 50 | 67 |  |
| total 5 peptides |  |  |  |  |  |  |  |  |  |  |  |  |
| --- | --- | --- | --- | --- | --- | --- | --- | --- | --- | --- | --- | --- |

tr|A0A7J6BCR9|A0A7J6BCR9\_AMEME

back to list

  

| Protein Coverage
| Supporting Peptides
|

Protein Coverage:

Supporting Peptides:

| Peptide | Uniq | -10lgP | Mass | ppm | m/z | z | RT | Scan | #Spec | Start | End | PTM |
| --- | --- | --- | --- | --- | --- | --- | --- | --- | --- | --- | --- | --- |
| K.VLDQDKSGFIE.E | N | 52.45 | 1249.6190 | -0.5 | 625.8165 | 2 | 11.38 | 1669 | 4 | 50 | 60 |  |
| K.VLDQDKSGFIEEEE.L | Y | 43.10 | 1636.7467 | -0.4 | 819.3803 | 2 | 11.51 | 1691 | 1 | 50 | 63 |  |
| K.VLDQDKSGFIEEEELK.L | Y | 33.74 | 1877.9258 | -1.1 | 939.9692 | 2 | 11.62 | 1710 | 2 | 50 | 65 |  |
| F.IEEEELKLF.L | N | 30.75 | 1148.5964 | -0.3 | 575.3053 | 2 | 13.44 | 2031 | 1 | 59 | 67 |  |
| K.VLDQDKSGFIEEEELKLF.L | Y | 28.04 | 2138.0781 | -1.0 | 1070.0453 | 2 | 13.82 | 2095 | 1 | 50 | 67 |  |
| total 5 peptides |  |  |  |  |  |  |  |  |  |  |  |  |
| --- | --- | --- | --- | --- | --- | --- | --- | --- | --- | --- | --- | --- |

tr|A0A315V786|A0A315V786\_GAMAF

back to list

  

| Protein Coverage
| Supporting Peptides
|

Protein Coverage:

Supporting Peptides:

| Peptide | Uniq | -10lgP | Mass | ppm | m/z | z | RT | Scan | #Spec | Start | End | PTM |
| --- | --- | --- | --- | --- | --- | --- | --- | --- | --- | --- | --- | --- |
| T.VLDQDKSGFIE.E | N | 52.45 | 1249.6190 | -0.5 | 625.8165 | 2 | 11.38 | 1669 | 4 | 245 | 255 |  |
| T.VLDQDKSGFIEEEE.L | Y | 43.10 | 1636.7467 | -0.4 | 819.3803 | 2 | 11.51 | 1691 | 1 | 245 | 258 |  |
| T.VLDQDKSGFIEEEELK.L | Y | 33.74 | 1877.9258 | -1.1 | 939.9692 | 2 | 11.62 | 1710 | 2 | 245 | 260 |  |
| F.IEEEELKLF.L | N | 30.75 | 1148.5964 | -0.3 | 575.3053 | 2 | 13.44 | 2031 | 1 | 254 | 262 |  |
| T.VLDQDKSGFIEEEELKLF.L | Y | 28.04 | 2138.0781 | -1.0 | 1070.0453 | 2 | 13.82 | 2095 | 1 | 245 | 262 |  |
| total 5 peptides |  |  |  |  |  |  |  |  |  |  |  |  |
| --- | --- | --- | --- | --- | --- | --- | --- | --- | --- | --- | --- | --- |

tr|A0A1S3QJI3|A0A1S3QJI3\_SALSA

back to list

  

| Protein Coverage
| Supporting Peptides
|

Protein Coverage:

Supporting Peptides:

| Peptide | Uniq | -10lgP | Mass | ppm | m/z | z | RT | Scan | #Spec | Start | End | PTM |
| --- | --- | --- | --- | --- | --- | --- | --- | --- | --- | --- | --- | --- |
| K.LEQQVDDLEGSLE.Q | N | 41.80 | 1473.6835 | -0.6 | 737.8486 | 2 | 12.66 | 1896 | 1 | 1031 | 1043 |  |
| E.MNPPKYDKIEDM.A | N | 39.50 | 1479.6737 | -0.8 | 740.8436 | 2 | 11.36 | 1664 | 1 | 85 | 96 |  |
| S.VIPEGQF.M | N | 30.48 | 788.4068 | -0.7 | 395.2104 | 2 | 12.26 | 1825 | 2 | 733 | 739 |  |
| K.KMEGDLNEM.E | N | 28.74 | 1065.4470 | -0.5 | 533.7305 | 2 | 10.99 | 1598 | 1 | 1621 | 1629 |  |
| M.NPPKYDKIEDM.A | N | 26.69 | 1348.6333 | -0.3 | 675.3237 | 2 | 10.87 | 1577 | 1 | 86 | 96 |  |
| L.SKIEDEQSL.G | N | 26.50 | 1047.5084 | -0.3 | 524.7614 | 2 | 10.37 | 1494 | 1 | 1096 | 1104 |  |
| L.SKIEDEQSLGAQ.L | N | 20.57 | 1303.6255 | -0.3 | 652.8198 | 2 | 10.23 | 1470 | 1 | 1096 | 1107 |  |
| K.FKQKQREEQAEPDGTEVADK.I | N | 16.13 | 2332.1294 | -0.3 | 778.3835 | 3 | 9.20 | 1310 | 1 | 371 | 390 |  |
| N.FTNEKLQQF.F | N | 16.00 | 1153.5768 | 0.0 | 577.7957 | 2 | 11.46 | 1682 | 1 | 487 | 495 |  |
| A.EWKQKYEEGQAELE.G | N | 15.02 | 1765.8158 | -1.1 | 883.9142 | 2 | 10.99 | 1599 | 1 | 1459 | 1472 |  |
| total 10 peptides |  |  |  |  |  |  |  |  |  |  |  |  |
| --- | --- | --- | --- | --- | --- | --- | --- | --- | --- | --- | --- | --- |

tr|A0A6J2PWE3|A0A6J2PWE3\_COTGO

back to list

  

| Protein Coverage
| Supporting Peptides
|

Protein Coverage:

Supporting Peptides:

| Peptide | Uniq | -10lgP | Mass | ppm | m/z | z | RT | Scan | #Spec | Start | End | PTM |
| --- | --- | --- | --- | --- | --- | --- | --- | --- | --- | --- | --- | --- |
| K.LEQQVDDLEGSLE.Q | N | 41.80 | 1473.6835 | -0.6 | 737.8486 | 2 | 12.66 | 1896 | 1 | 1023 | 1035 |  |
| P.MNPPKYDKIEDM.A | N | 39.50 | 1479.6737 | -0.8 | 740.8436 | 2 | 11.36 | 1664 | 1 | 81 | 92 |  |
| G.VIPEGQF.I | N | 30.48 | 788.4068 | -0.7 | 395.2104 | 2 | 12.26 | 1825 | 2 | 725 | 731 |  |
| K.KMEGDLNEM.E | N | 28.74 | 1065.4470 | -0.5 | 533.7305 | 2 | 10.99 | 1598 | 1 | 1613 | 1621 |  |
| M.NPPKYDKIEDM.A | N | 26.69 | 1348.6333 | -0.3 | 675.3237 | 2 | 10.87 | 1577 | 1 | 82 | 92 |  |
| L.SKIEDEQSL.G | N | 26.50 | 1047.5084 | -0.3 | 524.7614 | 2 | 10.37 | 1494 | 1 | 1088 | 1096 |  |
| L.SKIEDEQSLGAQ.L | N | 20.57 | 1303.6255 | -0.3 | 652.8198 | 2 | 10.23 | 1470 | 1 | 1088 | 1099 |  |
| K.LHDQH.L | N | 17.45 | 648.2980 | 0.3 | 325.1564 | 2 | 3.70 | 484 | 3 | 550 | 554 |  |
| N.FTNEKLQQF.F | N | 16.00 | 1153.5768 | 0.0 | 577.7957 | 2 | 11.46 | 1682 | 1 | 478 | 486 |  |
| total 9 peptides |  |  |  |  |  |  |  |  |  |  |  |  |
| --- | --- | --- | --- | --- | --- | --- | --- | --- | --- | --- | --- | --- |

tr|A0A3Q3WCX3|A0A3Q3WCX3\_MOLML

back to list

  

| Protein Coverage
| Supporting Peptides
|

Protein Coverage:

Supporting Peptides:

| Peptide | Uniq | -10lgP | Mass | ppm | m/z | z | RT | Scan | #Spec | Start | End | PTM |
| --- | --- | --- | --- | --- | --- | --- | --- | --- | --- | --- | --- | --- |
| K.FSVDEEFPDLSQH.N | Y | 58.89 | 1548.6732 | 0.9 | 775.3446 | 2 | 13.11 | 1976 | 1 | 13 | 25 |  |
| S.VDEEFPDLSQH.N | Y | 25.80 | 1314.5728 | -0.7 | 658.2932 | 2 | 12.03 | 1784 | 1 | 15 | 25 |  |
| T.GVDNPGHPFIM.T | N | 23.48 | 1182.5492 | -0.7 | 592.2814 | 2 | 12.43 | 1855 | 1 | 59 | 69 |  |
| D.NLKGGDDLDPNY.V | N | 18.44 | 1319.5994 | -0.9 | 660.8063 | 2 | 11.14 | 1626 | 1 | 113 | 124 |  |
| L.LDPIISDR.H | N | 18.39 | 927.5025 | -0.8 | 464.7581 | 2 | 11.37 | 1667 | 1 | 88 | 95 |  |
| T.GVDNPGHPF.I | N | 17.65 | 938.4246 | -0.1 | 470.2195 | 2 | 10.83 | 1571 | 1 | 59 | 67 |  |
| L.VVDGVKL.M | N | 15.93 | 728.4432 | 0.0 | 365.2289 | 2 | 11.27 | 1649 | 1 | 352 | 358 |  |
| F.DISNADRL.G | N | 15.49 | 902.4457 | -0.7 | 452.2298 | 2 | 10.97 | 1595 | 1 | 334 | 341 |  |
| total 8 peptides |  |  |  |  |  |  |  |  |  |  |  |  |
| --- | --- | --- | --- | --- | --- | --- | --- | --- | --- | --- | --- | --- |

tr|A0A0S7H3J1|A0A0S7H3J1\_9TELE

back to list

  

| Protein Coverage
| Supporting Peptides
|

Protein Coverage:

Supporting Peptides:

| Peptide | Uniq | -10lgP | Mass | ppm | m/z | z | RT | Scan | #Spec | Start | End | PTM |
| --- | --- | --- | --- | --- | --- | --- | --- | --- | --- | --- | --- | --- |
| K.FSVDEEFPDLSQH.N | Y | 58.89 | 1548.6732 | 0.9 | 775.3446 | 2 | 13.11 | 1976 | 1 | 15 | 27 |  |
| S.VDEEFPDLSQH.N | Y | 25.80 | 1314.5728 | -0.7 | 658.2932 | 2 | 12.03 | 1784 | 1 | 17 | 27 |  |
| T.GVDNPGHPFIM.T | N | 23.48 | 1182.5492 | -0.7 | 592.2814 | 2 | 12.43 | 1855 | 1 | 61 | 71 |  |
| E.NLKGGDDLDPNY.V | N | 18.44 | 1319.5994 | -0.9 | 660.8063 | 2 | 11.14 | 1626 | 1 | 115 | 126 |  |
| L.LDPIISDR.H | N | 18.39 | 927.5025 | -0.8 | 464.7581 | 2 | 11.37 | 1667 | 1 | 90 | 97 |  |
| T.GVDNPGHPF.I | N | 17.65 | 938.4246 | -0.1 | 470.2195 | 2 | 10.83 | 1571 | 1 | 61 | 69 |  |
| L.VVDGVKL.M | N | 15.93 | 728.4432 | 0.0 | 365.2289 | 2 | 11.27 | 1649 | 1 | 354 | 360 |  |
| F.DISNADRL.G | N | 15.49 | 902.4457 | -0.7 | 452.2298 | 2 | 10.97 | 1595 | 1 | 336 | 343 |  |
| total 8 peptides |  |  |  |  |  |  |  |  |  |  |  |  |
| --- | --- | --- | --- | --- | --- | --- | --- | --- | --- | --- | --- | --- |

tr|A0A3B3XY74|A0A3B3XY74\_9TELE

back to list

  

| Protein Coverage
| Supporting Peptides
|

Protein Coverage:

Supporting Peptides:

| Peptide | Uniq | -10lgP | Mass | ppm | m/z | z | RT | Scan | #Spec | Start | End | PTM |
| --- | --- | --- | --- | --- | --- | --- | --- | --- | --- | --- | --- | --- |
| K.FSVDEEFPDLSQH.N | Y | 58.89 | 1548.6732 | 0.9 | 775.3446 | 2 | 13.11 | 1976 | 1 | 15 | 27 |  |
| S.VDEEFPDLSQH.N | Y | 25.80 | 1314.5728 | -0.7 | 658.2932 | 2 | 12.03 | 1784 | 1 | 17 | 27 |  |
| T.GVDNPGHPFIM.T | N | 23.48 | 1182.5492 | -0.7 | 592.2814 | 2 | 12.43 | 1855 | 1 | 61 | 71 |  |
| E.NLKGGDDLDPNY.V | N | 18.44 | 1319.5994 | -0.9 | 660.8063 | 2 | 11.14 | 1626 | 1 | 115 | 126 |  |
| L.LDPIISDR.H | N | 18.39 | 927.5025 | -0.8 | 464.7581 | 2 | 11.37 | 1667 | 1 | 90 | 97 |  |
| T.GVDNPGHPF.I | N | 17.65 | 938.4246 | -0.1 | 470.2195 | 2 | 10.83 | 1571 | 1 | 61 | 69 |  |
| L.VVDGVKL.M | N | 15.93 | 728.4432 | 0.0 | 365.2289 | 2 | 11.27 | 1649 | 1 | 354 | 360 |  |
| F.DISNADRL.G | N | 15.49 | 902.4457 | -0.7 | 452.2298 | 2 | 10.97 | 1595 | 1 | 336 | 343 |  |
| total 8 peptides |  |  |  |  |  |  |  |  |  |  |  |  |
| --- | --- | --- | --- | --- | --- | --- | --- | --- | --- | --- | --- | --- |

tr|A0A3B3W291|A0A3B3W291\_9TELE

back to list

  

| Protein Coverage
| Supporting Peptides
|

Protein Coverage:

Supporting Peptides:

| Peptide | Uniq | -10lgP | Mass | ppm | m/z | z | RT | Scan | #Spec | Start | End | PTM |
| --- | --- | --- | --- | --- | --- | --- | --- | --- | --- | --- | --- | --- |
| K.FSVDEEFPDLSQH.N | Y | 58.89 | 1548.6732 | 0.9 | 775.3446 | 2 | 13.11 | 1976 | 1 | 15 | 27 |  |
| S.VDEEFPDLSQH.N | Y | 25.80 | 1314.5728 | -0.7 | 658.2932 | 2 | 12.03 | 1784 | 1 | 17 | 27 |  |
| T.GVDNPGHPFIM.T | N | 23.48 | 1182.5492 | -0.7 | 592.2814 | 2 | 12.43 | 1855 | 1 | 61 | 71 |  |
| E.NLKGGDDLDPNY.V | N | 18.44 | 1319.5994 | -0.9 | 660.8063 | 2 | 11.14 | 1626 | 1 | 115 | 126 |  |
| L.LDPIISDR.H | N | 18.39 | 927.5025 | -0.8 | 464.7581 | 2 | 11.37 | 1667 | 1 | 90 | 97 |  |
| T.GVDNPGHPF.I | N | 17.65 | 938.4246 | -0.1 | 470.2195 | 2 | 10.83 | 1571 | 1 | 61 | 69 |  |
| L.VVDGVKL.M | N | 15.93 | 728.4432 | 0.0 | 365.2289 | 2 | 11.27 | 1649 | 1 | 354 | 360 |  |
| F.DISNADRL.G | N | 15.49 | 902.4457 | -0.7 | 452.2298 | 2 | 10.97 | 1595 | 1 | 336 | 343 |  |
| total 8 peptides |  |  |  |  |  |  |  |  |  |  |  |  |
| --- | --- | --- | --- | --- | --- | --- | --- | --- | --- | --- | --- | --- |

tr|A0A087YG46|A0A087YG46\_POEFO

back to list

  

| Protein Coverage
| Supporting Peptides
|

Protein Coverage:

Supporting Peptides:

| Peptide | Uniq | -10lgP | Mass | ppm | m/z | z | RT | Scan | #Spec | Start | End | PTM |
| --- | --- | --- | --- | --- | --- | --- | --- | --- | --- | --- | --- | --- |
| K.FSVDEEFPDLSQH.N | Y | 58.89 | 1548.6732 | 0.9 | 775.3446 | 2 | 13.11 | 1976 | 1 | 15 | 27 |  |
| S.VDEEFPDLSQH.N | Y | 25.80 | 1314.5728 | -0.7 | 658.2932 | 2 | 12.03 | 1784 | 1 | 17 | 27 |  |
| T.GVDNPGHPFIM.T | N | 23.48 | 1182.5492 | -0.7 | 592.2814 | 2 | 12.43 | 1855 | 1 | 61 | 71 |  |
| E.NLKGGDDLDPNY.V | N | 18.44 | 1319.5994 | -0.9 | 660.8063 | 2 | 11.14 | 1626 | 1 | 115 | 126 |  |
| L.LDPIISDR.H | N | 18.39 | 927.5025 | -0.8 | 464.7581 | 2 | 11.37 | 1667 | 1 | 90 | 97 |  |
| T.GVDNPGHPF.I | N | 17.65 | 938.4246 | -0.1 | 470.2195 | 2 | 10.83 | 1571 | 1 | 61 | 69 |  |
| L.VVDGVKL.M | N | 15.93 | 728.4432 | 0.0 | 365.2289 | 2 | 11.27 | 1649 | 1 | 355 | 361 |  |
| F.DISNADRL.G | N | 15.49 | 902.4457 | -0.7 | 452.2298 | 2 | 10.97 | 1595 | 1 | 337 | 344 |  |
| total 8 peptides |  |  |  |  |  |  |  |  |  |  |  |  |
| --- | --- | --- | --- | --- | --- | --- | --- | --- | --- | --- | --- | --- |

tr|A0A3Q2D194|A0A3Q2D194\_CYPVA

back to list

  

| Protein Coverage
| Supporting Peptides
|

Protein Coverage:

Supporting Peptides:

| Peptide | Uniq | -10lgP | Mass | ppm | m/z | z | RT | Scan | #Spec | Start | End | PTM |
| --- | --- | --- | --- | --- | --- | --- | --- | --- | --- | --- | --- | --- |
| L.GSSEVDQVQLVVDGVKLM.V | N | 58.08 | 1901.9768 | -0.1 | 951.9955 | 2 | 14.47 | 2202 | 1 | 344 | 361 |  |
| F.SVDEEFPDLSKH.N | Y | 25.18 | 1401.6411 | -26.7 | 701.8091 | 2 | 12.20 | 1814 | 1 | 16 | 27 |  |
| T.GVDNPGHPFIM.T | N | 23.48 | 1182.5492 | -0.7 | 592.2814 | 2 | 12.43 | 1855 | 1 | 61 | 71 |  |
| E.NLKGGDDLDPNY.V | N | 18.44 | 1319.5994 | -0.9 | 660.8063 | 2 | 11.14 | 1626 | 1 | 115 | 126 |  |
| T.GVDNPGHPF.I | N | 17.65 | 938.4246 | -0.1 | 470.2195 | 2 | 10.83 | 1571 | 1 | 61 | 69 |  |
| L.VVDGVKL.M | N | 15.93 | 728.4432 | 0.0 | 365.2289 | 2 | 11.27 | 1649 | 1 | 354 | 360 |  |
| F.DISNADRL.G | N | 15.49 | 902.4457 | -0.7 | 452.2298 | 2 | 10.97 | 1595 | 1 | 336 | 343 |  |
| total 7 peptides |  |  |  |  |  |  |  |  |  |  |  |  |
| --- | --- | --- | --- | --- | --- | --- | --- | --- | --- | --- | --- | --- |

tr|A0A3Q2D1K7|A0A3Q2D1K7\_CYPVA

back to list

  

| Protein Coverage
| Supporting Peptides
|

Protein Coverage:

Supporting Peptides:

| Peptide | Uniq | -10lgP | Mass | ppm | m/z | z | RT | Scan | #Spec | Start | End | PTM |
| --- | --- | --- | --- | --- | --- | --- | --- | --- | --- | --- | --- | --- |
| L.GSSEVDQVQLVVDGVKLM.V | N | 58.08 | 1901.9768 | -0.1 | 951.9955 | 2 | 14.47 | 2202 | 1 | 313 | 330 |  |
| F.SVDEEFPDLSKH.N | Y | 25.18 | 1401.6411 | -26.7 | 701.8091 | 2 | 12.20 | 1814 | 1 | 16 | 27 |  |
| T.GVDNPGHPFIM.T | N | 23.48 | 1182.5492 | -0.7 | 592.2814 | 2 | 12.43 | 1855 | 1 | 61 | 71 |  |
| E.NLKGGDDLDPNY.V | N | 18.44 | 1319.5994 | -0.9 | 660.8063 | 2 | 11.14 | 1626 | 1 | 115 | 126 |  |
| T.GVDNPGHPF.I | N | 17.65 | 938.4246 | -0.1 | 470.2195 | 2 | 10.83 | 1571 | 1 | 61 | 69 |  |
| L.VVDGVKL.M | N | 15.93 | 728.4432 | 0.0 | 365.2289 | 2 | 11.27 | 1649 | 1 | 323 | 329 |  |
| F.DISNADRL.G | N | 15.49 | 902.4457 | -0.7 | 452.2298 | 2 | 10.97 | 1595 | 1 | 305 | 312 |  |
| total 7 peptides |  |  |  |  |  |  |  |  |  |  |  |  |
| --- | --- | --- | --- | --- | --- | --- | --- | --- | --- | --- | --- | --- |

tr|C7ASM1|C7ASM1\_SINCH

back to list

  

| Protein Coverage
| Supporting Peptides
|

Protein Coverage:

Supporting Peptides:

| Peptide | Uniq | -10lgP | Mass | ppm | m/z | z | RT | Scan | #Spec | Start | End | PTM |
| --- | --- | --- | --- | --- | --- | --- | --- | --- | --- | --- | --- | --- |
| L.GSSEVDQVQLVVDGVKLM.V | N | 58.08 | 1901.9768 | -0.1 | 951.9955 | 2 | 14.47 | 2202 | 1 | 343 | 360 |  |
| T.GVDNPGHPFIM.T | N | 23.48 | 1182.5492 | -0.7 | 592.2814 | 2 | 12.43 | 1855 | 1 | 60 | 70 |  |
| K.VEEEFPDLSKH.N | Y | 18.63 | 1328.6248 | -0.4 | 665.3194 | 2 | 11.41 | 1673 | 1 | 16 | 26 |  |
| E.NLKGGDDLDPNY.V | N | 18.44 | 1319.5994 | -0.9 | 660.8063 | 2 | 11.14 | 1626 | 1 | 114 | 125 |  |
| L.LDPIISDR.H | N | 18.39 | 927.5025 | -0.8 | 464.7581 | 2 | 11.37 | 1667 | 1 | 89 | 96 |  |
| T.GVDNPGHPF.I | N | 17.65 | 938.4246 | -0.1 | 470.2195 | 2 | 10.83 | 1571 | 1 | 60 | 68 |  |
| L.VVDGVKL.M | N | 15.93 | 728.4432 | 0.0 | 365.2289 | 2 | 11.27 | 1649 | 1 | 353 | 359 |  |
| L.DISNADRL.G | N | 15.49 | 902.4457 | -0.7 | 452.2298 | 2 | 10.97 | 1595 | 1 | 335 | 342 |  |
| total 8 peptides |  |  |  |  |  |  |  |  |  |  |  |  |
| --- | --- | --- | --- | --- | --- | --- | --- | --- | --- | --- | --- | --- |

tr|M4AU26|M4AU26\_XIPMA

back to list

  

| Protein Coverage
| Supporting Peptides
|

Protein Coverage:

Supporting Peptides:

| Peptide | Uniq | -10lgP | Mass | ppm | m/z | z | RT | Scan | #Spec | Start | End | PTM |
| --- | --- | --- | --- | --- | --- | --- | --- | --- | --- | --- | --- | --- |
| L.GSSEVDQVQLVVDGVKLM.V | N | 58.08 | 1901.9768 | -0.1 | 951.9955 | 2 | 14.47 | 2202 | 1 | 343 | 360 |  |
| T.GVDNPGHPFIM.T | N | 23.48 | 1182.5492 | -0.7 | 592.2814 | 2 | 12.43 | 1855 | 1 | 60 | 70 |  |
| K.VEEEFPDLSKH.N | Y | 18.63 | 1328.6248 | -0.4 | 665.3194 | 2 | 11.41 | 1673 | 1 | 16 | 26 |  |
| E.NLKGGDDLDPNY.V | N | 18.44 | 1319.5994 | -0.9 | 660.8063 | 2 | 11.14 | 1626 | 1 | 114 | 125 |  |
| L.LDPIISDR.H | N | 18.39 | 927.5025 | -0.8 | 464.7581 | 2 | 11.37 | 1667 | 1 | 89 | 96 |  |
| T.GVDNPGHPF.I | N | 17.65 | 938.4246 | -0.1 | 470.2195 | 2 | 10.83 | 1571 | 1 | 60 | 68 |  |
| L.VVDGVKL.M | N | 15.93 | 728.4432 | 0.0 | 365.2289 | 2 | 11.27 | 1649 | 1 | 353 | 359 |  |
| F.DISNADRL.G | N | 15.49 | 902.4457 | -0.7 | 452.2298 | 2 | 10.97 | 1595 | 1 | 335 | 342 |  |
| total 8 peptides |  |  |  |  |  |  |  |  |  |  |  |  |
| --- | --- | --- | --- | --- | --- | --- | --- | --- | --- | --- | --- | --- |

tr|A0A3B5M9I5|A0A3B5M9I5\_9TELE

back to list

  

| Protein Coverage
| Supporting Peptides
|

Protein Coverage:

Supporting Peptides:

| Peptide | Uniq | -10lgP | Mass | ppm | m/z | z | RT | Scan | #Spec | Start | End | PTM |
| --- | --- | --- | --- | --- | --- | --- | --- | --- | --- | --- | --- | --- |
| L.GSSEVDQVQLVVDGVKLM.V | N | 58.08 | 1901.9768 | -0.1 | 951.9955 | 2 | 14.47 | 2202 | 1 | 343 | 360 |  |
| T.GVDNPGHPFIM.T | N | 23.48 | 1182.5492 | -0.7 | 592.2814 | 2 | 12.43 | 1855 | 1 | 60 | 70 |  |
| K.VEEEFPDLSKH.N | Y | 18.63 | 1328.6248 | -0.4 | 665.3194 | 2 | 11.41 | 1673 | 1 | 16 | 26 |  |
| E.NLKGGDDLDPNY.V | N | 18.44 | 1319.5994 | -0.9 | 660.8063 | 2 | 11.14 | 1626 | 1 | 114 | 125 |  |
| L.LDPIISDR.H | N | 18.39 | 927.5025 | -0.8 | 464.7581 | 2 | 11.37 | 1667 | 1 | 89 | 96 |  |
| T.GVDNPGHPF.I | N | 17.65 | 938.4246 | -0.1 | 470.2195 | 2 | 10.83 | 1571 | 1 | 60 | 68 |  |
| L.VVDGVKL.M | N | 15.93 | 728.4432 | 0.0 | 365.2289 | 2 | 11.27 | 1649 | 1 | 353 | 359 |  |
| F.DISNADRL.G | N | 15.49 | 902.4457 | -0.7 | 452.2298 | 2 | 10.97 | 1595 | 1 | 335 | 342 |  |
| total 8 peptides |  |  |  |  |  |  |  |  |  |  |  |  |
| --- | --- | --- | --- | --- | --- | --- | --- | --- | --- | --- | --- | --- |

tr|A0A6J2Q2L7|A0A6J2Q2L7\_COTGO

back to list

  

| Protein Coverage
| Supporting Peptides
|

Protein Coverage:

Supporting Peptides:

| Peptide | Uniq | -10lgP | Mass | ppm | m/z | z | RT | Scan | #Spec | Start | End | PTM |
| --- | --- | --- | --- | --- | --- | --- | --- | --- | --- | --- | --- | --- |
| K.LEQQVDDLEGSLE.Q | N | 41.80 | 1473.6835 | -0.6 | 737.8486 | 2 | 12.66 | 1896 | 1 | 1030 | 1042 |  |
| S.VIPEGQF.I | N | 30.48 | 788.4068 | -0.7 | 395.2104 | 2 | 12.26 | 1825 | 2 | 732 | 738 |  |
| E.YKKEGIEWEFIDF.G | N | 29.33 | 1702.8242 | -0.4 | 852.4191 | 2 | 14.27 | 2169 | 1 | 503 | 515 |  |
| K.KMEGDLNEM.E | N | 28.74 | 1065.4470 | -0.5 | 533.7305 | 2 | 10.99 | 1598 | 1 | 1620 | 1628 |  |
| R.NPPKYDKIEDM.A | N | 26.69 | 1348.6333 | -0.3 | 675.3237 | 2 | 10.87 | 1577 | 1 | 81 | 91 |  |
| L.SKIEDEQSL.S | N | 26.50 | 1047.5084 | -0.3 | 524.7614 | 2 | 10.37 | 1494 | 1 | 1095 | 1103 |  |
| K.LHDQH.L | N | 17.45 | 648.2980 | 0.3 | 325.1564 | 2 | 3.70 | 484 | 3 | 554 | 558 |  |
| N.VKNWPW.L | N | 16.17 | 828.4282 | -1.1 | 415.2209 | 2 | 12.79 | 1919 | 1 | 827 | 832 |  |
| N.FTNEKLQQF.F | N | 16.00 | 1153.5768 | 0.0 | 577.7957 | 2 | 11.46 | 1682 | 1 | 482 | 490 |  |
| total 9 peptides |  |  |  |  |  |  |  |  |  |  |  |  |
| --- | --- | --- | --- | --- | --- | --- | --- | --- | --- | --- | --- | --- |

tr|A0A6P3WCZ7|A0A6P3WCZ7\_CLUHA

back to list

  

| Protein Coverage
| Supporting Peptides
|

Protein Coverage:

Supporting Peptides:

| Peptide | Uniq | -10lgP | Mass | ppm | m/z | z | RT | Scan | #Spec | Start | End | PTM |
| --- | --- | --- | --- | --- | --- | --- | --- | --- | --- | --- | --- | --- |
| K.LEQQVDDLEGSLE.Q | N | 41.80 | 1473.6835 | -0.6 | 737.8486 | 2 | 12.66 | 1896 | 1 | 1034 | 1046 |  |
| P.MNPPKYDKIEDM.V | N | 39.50 | 1479.6737 | -0.8 | 740.8436 | 2 | 11.36 | 1664 | 1 | 80 | 91 |  |
| K.KMEGDLNEM.E | N | 28.74 | 1065.4470 | -0.5 | 533.7305 | 2 | 10.99 | 1598 | 1 | 1624 | 1632 |  |
| M.NPPKYDKIEDM.V | N | 26.69 | 1348.6333 | -0.3 | 675.3237 | 2 | 10.87 | 1577 | 1 | 81 | 91 |  |
| N.VKNWPW.M | N | 16.17 | 828.4282 | -1.1 | 415.2209 | 2 | 12.79 | 1919 | 1 | 831 | 836 |  |
| K.FKQKQREEQAEPDGTEVADK.V | N | 16.13 | 2332.1294 | -0.3 | 778.3835 | 3 | 9.20 | 1310 | 1 | 366 | 385 |  |
| N.FTNEKLQQF.F | N | 16.00 | 1153.5768 | 0.0 | 577.7957 | 2 | 11.46 | 1682 | 1 | 482 | 490 |  |
| total 7 peptides |  |  |  |  |  |  |  |  |  |  |  |  |
| --- | --- | --- | --- | --- | --- | --- | --- | --- | --- | --- | --- | --- |

tr|A0A4W4E3Q5|A0A4W4E3Q5\_ELEEL

back to list

  

| Protein Coverage
| Supporting Peptides
|

Protein Coverage:

Supporting Peptides:

| Peptide | Uniq | -10lgP | Mass | ppm | m/z | z | RT | Scan | #Spec | Start | End | PTM |
| --- | --- | --- | --- | --- | --- | --- | --- | --- | --- | --- | --- | --- |
| K.LEQQVDDLEGSLE.Q | N | 41.80 | 1473.6835 | -0.6 | 737.8486 | 2 | 12.66 | 1896 | 1 | 71 | 83 |  |
| E.NDKQQSDEKIKKK.D | N | 41.47 | 1587.8580 | -0.2 | 794.9362 | 2 | 7.54 | 1010 | 2 | 115 | 127 |  |
| Q.IGETGKT.I | N | 30.06 | 704.3705 | -0.2 | 353.1924 | 2 | 8.67 | 1226 | 1 | 560 | 566 |  |
| K.KMEGDLNEM.E | N | 28.74 | 1065.4470 | -0.5 | 533.7305 | 2 | 10.99 | 1598 | 1 | 661 | 669 |  |
| E.LKKEQDTSAH.L | N | 16.64 | 1155.5884 | 0.0 | 578.8015 | 2 | 7.73 | 1044 | 1 | 807 | 816 |  |
| A.EWKQKYEEGQAELE.G | N | 15.02 | 1765.8158 | -1.1 | 883.9142 | 2 | 10.99 | 1599 | 1 | 499 | 512 |  |
| total 6 peptides |  |  |  |  |  |  |  |  |  |  |  |  |
| --- | --- | --- | --- | --- | --- | --- | --- | --- | --- | --- | --- | --- |

tr|A0A3B4FY50|A0A3B4FY50\_9CICH

back to list

  

| Protein Coverage
| Supporting Peptides
|

Protein Coverage:

Supporting Peptides:

| Peptide | Uniq | -10lgP | Mass | ppm | m/z | z | RT | Scan | #Spec | Start | End | PTM |
| --- | --- | --- | --- | --- | --- | --- | --- | --- | --- | --- | --- | --- |
| K.LEQQVDDLEGSLE.Q | N | 41.80 | 1473.6835 | -0.6 | 737.8486 | 2 | 12.66 | 1896 | 1 | 1027 | 1039 |  |
| P.MNPPKYDKIEDM.A | N | 39.50 | 1479.6737 | -0.8 | 740.8436 | 2 | 11.36 | 1664 | 1 | 80 | 91 |  |
| K.KMEGDLNEM.E | N | 28.74 | 1065.4470 | -0.5 | 533.7305 | 2 | 10.99 | 1598 | 1 | 1617 | 1625 |  |
| M.NPPKYDKIEDM.A | N | 26.69 | 1348.6333 | -0.3 | 675.3237 | 2 | 10.87 | 1577 | 1 | 81 | 91 |  |
| E.LKKEQDTSAH.L | N | 16.64 | 1155.5884 | 0.0 | 578.8015 | 2 | 7.73 | 1044 | 1 | 1769 | 1778 |  |
| K.FKQKQREEQAEPDGTEVADK.V | N | 16.13 | 2332.1294 | -0.3 | 778.3835 | 3 | 9.20 | 1310 | 1 | 366 | 385 |  |
| total 6 peptides |  |  |  |  |  |  |  |  |  |  |  |  |
| --- | --- | --- | --- | --- | --- | --- | --- | --- | --- | --- | --- | --- |

tr|A0A3P9ART7|A0A3P9ART7\_9CICH

back to list

  

| Protein Coverage
| Supporting Peptides
|

Protein Coverage:

Supporting Peptides:

| Peptide | Uniq | -10lgP | Mass | ppm | m/z | z | RT | Scan | #Spec | Start | End | PTM |
| --- | --- | --- | --- | --- | --- | --- | --- | --- | --- | --- | --- | --- |
| K.LEQQVDDLEGSLE.Q | N | 41.80 | 1473.6835 | -0.6 | 737.8486 | 2 | 12.66 | 1896 | 1 | 1027 | 1039 |  |
| P.MNPPKYDKIEDM.A | N | 39.50 | 1479.6737 | -0.8 | 740.8436 | 2 | 11.36 | 1664 | 1 | 80 | 91 |  |
| K.KMEGDLNEM.E | N | 28.74 | 1065.4470 | -0.5 | 533.7305 | 2 | 10.99 | 1598 | 1 | 1617 | 1625 |  |
| M.NPPKYDKIEDM.A | N | 26.69 | 1348.6333 | -0.3 | 675.3237 | 2 | 10.87 | 1577 | 1 | 81 | 91 |  |
| E.LKKEQDTSAH.L | N | 16.64 | 1155.5884 | 0.0 | 578.8015 | 2 | 7.73 | 1044 | 1 | 1769 | 1778 |  |
| K.FKQKQREEQAEPDGTEVADK.V | N | 16.13 | 2332.1294 | -0.3 | 778.3835 | 3 | 9.20 | 1310 | 1 | 366 | 385 |  |
| total 6 peptides |  |  |  |  |  |  |  |  |  |  |  |  |
| --- | --- | --- | --- | --- | --- | --- | --- | --- | --- | --- | --- | --- |

tr|D2KQG3|D2KQG3\_SINCH

back to list

  

| Protein Coverage
| Supporting Peptides
|

Protein Coverage:

Supporting Peptides:

| Peptide | Uniq | -10lgP | Mass | ppm | m/z | z | RT | Scan | #Spec | Start | End | PTM |
| --- | --- | --- | --- | --- | --- | --- | --- | --- | --- | --- | --- | --- |
| E.VIDQDKSGFIE.E | N | 52.45 | 1249.6190 | -0.5 | 625.8165 | 2 | 11.38 | 1669 | 4 | 50 | 60 |  |
| A.FEVIDQDKSGFIE.E | Y | 45.26 | 1525.7300 | -0.7 | 763.8717 | 2 | 12.66 | 1897 | 2 | 48 | 60 |  |
| total 2 peptides |  |  |  |  |  |  |  |  |  |  |  |  |
| --- | --- | --- | --- | --- | --- | --- | --- | --- | --- | --- | --- | --- |

tr|B9VJM3|B9VJM3\_SINCH

back to list

  

| Protein Coverage
| Supporting Peptides
|

Protein Coverage:

Supporting Peptides:

| Peptide | Uniq | -10lgP | Mass | ppm | m/z | z | RT | Scan | #Spec | Start | End | PTM |
| --- | --- | --- | --- | --- | --- | --- | --- | --- | --- | --- | --- | --- |
| E.VIDQDKSGFIE.E | N | 52.45 | 1249.6190 | -0.5 | 625.8165 | 2 | 11.38 | 1669 | 4 | 50 | 60 |  |
| A.FEVIDQDKSGFIE.E | Y | 45.26 | 1525.7300 | -0.7 | 763.8717 | 2 | 12.66 | 1897 | 2 | 48 | 60 |  |
| total 2 peptides |  |  |  |  |  |  |  |  |  |  |  |  |
| --- | --- | --- | --- | --- | --- | --- | --- | --- | --- | --- | --- | --- |

tr|A0A3Q0QYR9|A0A3Q0QYR9\_AMPCI

back to list

  

| Protein Coverage
| Supporting Peptides
|

Protein Coverage:

Supporting Peptides:

| Peptide | Uniq | -10lgP | Mass | ppm | m/z | z | RT | Scan | #Spec | Start | End | PTM |
| --- | --- | --- | --- | --- | --- | --- | --- | --- | --- | --- | --- | --- |
| H.YLDLKDMPFYA.G | Y | 45.04 | 1374.6530 | -1.3 | 688.3329 | 2 | 14.23 | 2163 | 1 | 20 | 30 |  |
| Y.LDLKDMPFY.A | Y | 30.13 | 1140.5525 | 0.0 | 571.2835 | 2 | 14.03 | 2129 | 1 | 21 | 29 |  |
| Y.LDLKDMPFYAGL.C | Y | 25.91 | 1381.6951 | 0.1 | 691.8549 | 2 | 14.80 | 2258 | 1 | 21 | 32 |  |
| H.YLDLKDMPFYAG.L | Y | 18.91 | 1431.6744 | -0.4 | 716.8442 | 2 | 14.10 | 2141 | 1 | 20 | 31 |  |
| total 4 peptides |  |  |  |  |  |  |  |  |  |  |  |  |
| --- | --- | --- | --- | --- | --- | --- | --- | --- | --- | --- | --- | --- |

P85280|NDKB\_MERME

back to list

  

| Protein Coverage
| Supporting Peptides
|

Protein Coverage:

Supporting Peptides:

| Peptide | Uniq | -10lgP | Mass | ppm | m/z | z | RT | Scan | #Spec | Start | End | PTM |
| --- | --- | --- | --- | --- | --- | --- | --- | --- | --- | --- | --- | --- |
| H.YLDLKDMPFYA.G | Y | 45.04 | 1374.6530 | -1.3 | 688.3329 | 2 | 14.23 | 2163 | 1 | 29 | 39 |  |
| Y.LDLKDMPFY.A | Y | 30.13 | 1140.5525 | 0.0 | 571.2835 | 2 | 14.03 | 2129 | 1 | 30 | 38 |  |
| Y.LDLKDMPFYAGL.C | Y | 25.91 | 1381.6951 | 0.1 | 691.8549 | 2 | 14.80 | 2258 | 1 | 30 | 41 |  |
| H.YLDLKDMPFYAG.L | Y | 18.91 | 1431.6744 | -0.4 | 716.8442 | 2 | 14.10 | 2141 | 1 | 29 | 40 |  |
| total 4 peptides |  |  |  |  |  |  |  |  |  |  |  |  |
| --- | --- | --- | --- | --- | --- | --- | --- | --- | --- | --- | --- | --- |

P85284|NDKB\_MERPA

back to list

  

| Protein Coverage
| Supporting Peptides
|

Protein Coverage:

Supporting Peptides:

| Peptide | Uniq | -10lgP | Mass | ppm | m/z | z | RT | Scan | #Spec | Start | End | PTM |
| --- | --- | --- | --- | --- | --- | --- | --- | --- | --- | --- | --- | --- |
| H.YLDLKDMPFYA.G | Y | 45.04 | 1374.6530 | -1.3 | 688.3329 | 2 | 14.23 | 2163 | 1 | 29 | 39 |  |
| Y.LDLKDMPFY.A | Y | 30.13 | 1140.5525 | 0.0 | 571.2835 | 2 | 14.03 | 2129 | 1 | 30 | 38 |  |
| Y.LDLKDMPFYAGL.C | Y | 25.91 | 1381.6951 | 0.1 | 691.8549 | 2 | 14.80 | 2258 | 1 | 30 | 41 |  |
| H.YLDLKDMPFYAG.L | Y | 18.91 | 1431.6744 | -0.4 | 716.8442 | 2 | 14.10 | 2141 | 1 | 29 | 40 |  |
| total 4 peptides |  |  |  |  |  |  |  |  |  |  |  |  |
| --- | --- | --- | --- | --- | --- | --- | --- | --- | --- | --- | --- | --- |

P85292|NDKB\_MACMG

back to list

  

| Protein Coverage
| Supporting Peptides
|

Protein Coverage:

Supporting Peptides:

| Peptide | Uniq | -10lgP | Mass | ppm | m/z | z | RT | Scan | #Spec | Start | End | PTM |
| --- | --- | --- | --- | --- | --- | --- | --- | --- | --- | --- | --- | --- |
| H.YLDLKDMPFYA.G | Y | 45.04 | 1374.6530 | -1.3 | 688.3329 | 2 | 14.23 | 2163 | 1 | 29 | 39 |  |
| Y.LDLKDMPFY.A | Y | 30.13 | 1140.5525 | 0.0 | 571.2835 | 2 | 14.03 | 2129 | 1 | 30 | 38 |  |
| Y.LDLKDMPFYAGL.C | Y | 25.91 | 1381.6951 | 0.1 | 691.8549 | 2 | 14.80 | 2258 | 1 | 30 | 41 |  |
| H.YLDLKDMPFYAG.L | Y | 18.91 | 1431.6744 | -0.4 | 716.8442 | 2 | 14.10 | 2141 | 1 | 29 | 40 |  |
| total 4 peptides |  |  |  |  |  |  |  |  |  |  |  |  |
| --- | --- | --- | --- | --- | --- | --- | --- | --- | --- | --- | --- | --- |

P85282|NDKB\_MERSE

back to list

  

| Protein Coverage
| Supporting Peptides
|

Protein Coverage:

Supporting Peptides:

| Peptide | Uniq | -10lgP | Mass | ppm | m/z | z | RT | Scan | #Spec | Start | End | PTM |
| --- | --- | --- | --- | --- | --- | --- | --- | --- | --- | --- | --- | --- |
| H.YLDLKDMPFYA.G | Y | 45.04 | 1374.6530 | -1.3 | 688.3329 | 2 | 14.23 | 2163 | 1 | 29 | 39 |  |
| Y.LDLKDMPFY.A | Y | 30.13 | 1140.5525 | 0.0 | 571.2835 | 2 | 14.03 | 2129 | 1 | 30 | 38 |  |
| Y.LDLKDMPFYAGL.C | Y | 25.91 | 1381.6951 | 0.1 | 691.8549 | 2 | 14.80 | 2258 | 1 | 30 | 41 |  |
| H.YLDLKDMPFYAG.L | Y | 18.91 | 1431.6744 | -0.4 | 716.8442 | 2 | 14.10 | 2141 | 1 | 29 | 40 |  |
| total 4 peptides |  |  |  |  |  |  |  |  |  |  |  |  |
| --- | --- | --- | --- | --- | --- | --- | --- | --- | --- | --- | --- | --- |

P85283|NDKB\_MERPO

back to list

  

| Protein Coverage
| Supporting Peptides
|

Protein Coverage:

Supporting Peptides:

| Peptide | Uniq | -10lgP | Mass | ppm | m/z | z | RT | Scan | #Spec | Start | End | PTM |
| --- | --- | --- | --- | --- | --- | --- | --- | --- | --- | --- | --- | --- |
| H.YLDLKDMPFYA.G | Y | 45.04 | 1374.6530 | -1.3 | 688.3329 | 2 | 14.23 | 2163 | 1 | 29 | 39 |  |
| Y.LDLKDMPFY.A | Y | 30.13 | 1140.5525 | 0.0 | 571.2835 | 2 | 14.03 | 2129 | 1 | 30 | 38 |  |
| Y.LDLKDMPFYAGL.C | Y | 25.91 | 1381.6951 | 0.1 | 691.8549 | 2 | 14.80 | 2258 | 1 | 30 | 41 |  |
| H.YLDLKDMPFYAG.L | Y | 18.91 | 1431.6744 | -0.4 | 716.8442 | 2 | 14.10 | 2141 | 1 | 29 | 40 |  |
| total 4 peptides |  |  |  |  |  |  |  |  |  |  |  |  |
| --- | --- | --- | --- | --- | --- | --- | --- | --- | --- | --- | --- | --- |

P85287|NDKB\_MERAP

back to list

  

| Protein Coverage
| Supporting Peptides
|

Protein Coverage:

Supporting Peptides:

| Peptide | Uniq | -10lgP | Mass | ppm | m/z | z | RT | Scan | #Spec | Start | End | PTM |
| --- | --- | --- | --- | --- | --- | --- | --- | --- | --- | --- | --- | --- |
| H.YLDLKDMPFYA.G | Y | 45.04 | 1374.6530 | -1.3 | 688.3329 | 2 | 14.23 | 2163 | 1 | 31 | 41 |  |
| Y.LDLKDMPFY.A | Y | 30.13 | 1140.5525 | 0.0 | 571.2835 | 2 | 14.03 | 2129 | 1 | 32 | 40 |  |
| Y.LDLKDMPFYAGL.C | Y | 25.91 | 1381.6951 | 0.1 | 691.8549 | 2 | 14.80 | 2258 | 1 | 32 | 43 |  |
| H.YLDLKDMPFYAG.L | Y | 18.91 | 1431.6744 | -0.4 | 716.8442 | 2 | 14.10 | 2141 | 1 | 31 | 42 |  |
| total 4 peptides |  |  |  |  |  |  |  |  |  |  |  |  |
| --- | --- | --- | --- | --- | --- | --- | --- | --- | --- | --- | --- | --- |

P85289|NDKB\_MERPR

back to list

  

| Protein Coverage
| Supporting Peptides
|

Protein Coverage:

Supporting Peptides:

| Peptide | Uniq | -10lgP | Mass | ppm | m/z | z | RT | Scan | #Spec | Start | End | PTM |
| --- | --- | --- | --- | --- | --- | --- | --- | --- | --- | --- | --- | --- |
| H.YLDLKDMPFYA.G | Y | 45.04 | 1374.6530 | -1.3 | 688.3329 | 2 | 14.23 | 2163 | 1 | 31 | 41 |  |
| Y.LDLKDMPFY.A | Y | 30.13 | 1140.5525 | 0.0 | 571.2835 | 2 | 14.03 | 2129 | 1 | 32 | 40 |  |
| Y.LDLKDMPFYAGL.C | Y | 25.91 | 1381.6951 | 0.1 | 691.8549 | 2 | 14.80 | 2258 | 1 | 32 | 43 |  |
| H.YLDLKDMPFYAG.L | Y | 18.91 | 1431.6744 | -0.4 | 716.8442 | 2 | 14.10 | 2141 | 1 | 31 | 42 |  |
| total 4 peptides |  |  |  |  |  |  |  |  |  |  |  |  |
| --- | --- | --- | --- | --- | --- | --- | --- | --- | --- | --- | --- | --- |

P85290|NDKB\_MERBI

back to list

  

| Protein Coverage
| Supporting Peptides
|

Protein Coverage:

Supporting Peptides:

| Peptide | Uniq | -10lgP | Mass | ppm | m/z | z | RT | Scan | #Spec | Start | End | PTM |
| --- | --- | --- | --- | --- | --- | --- | --- | --- | --- | --- | --- | --- |
| H.YLDLKDMPFYA.G | Y | 45.04 | 1374.6530 | -1.3 | 688.3329 | 2 | 14.23 | 2163 | 1 | 31 | 41 |  |
| Y.LDLKDMPFY.A | Y | 30.13 | 1140.5525 | 0.0 | 571.2835 | 2 | 14.03 | 2129 | 1 | 32 | 40 |  |
| Y.LDLKDMPFYAGL.C | Y | 25.91 | 1381.6951 | 0.1 | 691.8549 | 2 | 14.80 | 2258 | 1 | 32 | 43 |  |
| H.YLDLKDMPFYAG.L | Y | 18.91 | 1431.6744 | -0.4 | 716.8442 | 2 | 14.10 | 2141 | 1 | 31 | 42 |  |
| total 4 peptides |  |  |  |  |  |  |  |  |  |  |  |  |
| --- | --- | --- | --- | --- | --- | --- | --- | --- | --- | --- | --- | --- |

tr|A0A672Y4P6|A0A672Y4P6\_9TELE

back to list

  

| Protein Coverage
| Supporting Peptides
|

Protein Coverage:

Supporting Peptides:

| Peptide | Uniq | -10lgP | Mass | ppm | m/z | z | RT | Scan | #Spec | Start | End | PTM |
| --- | --- | --- | --- | --- | --- | --- | --- | --- | --- | --- | --- | --- |
| H.YLDLKDMPFYA.G | Y | 45.04 | 1374.6530 | -1.3 | 688.3329 | 2 | 14.23 | 2163 | 1 | 49 | 59 |  |
| Y.LDLKDMPFY.A | Y | 30.13 | 1140.5525 | 0.0 | 571.2835 | 2 | 14.03 | 2129 | 1 | 50 | 58 |  |
| Y.LDLKDMPFYAGL.C | Y | 25.91 | 1381.6951 | 0.1 | 691.8549 | 2 | 14.80 | 2258 | 1 | 50 | 61 |  |
| H.YLDLKDMPFYAG.L | Y | 18.91 | 1431.6744 | -0.4 | 716.8442 | 2 | 14.10 | 2141 | 1 | 49 | 60 |  |
| total 4 peptides |  |  |  |  |  |  |  |  |  |  |  |  |
| --- | --- | --- | --- | --- | --- | --- | --- | --- | --- | --- | --- | --- |

tr|A0A2I4CF45|A0A2I4CF45\_9TELE

back to list

  

| Protein Coverage
| Supporting Peptides
|

Protein Coverage:

Supporting Peptides:

| Peptide | Uniq | -10lgP | Mass | ppm | m/z | z | RT | Scan | #Spec | Start | End | PTM |
| --- | --- | --- | --- | --- | --- | --- | --- | --- | --- | --- | --- | --- |
| H.YLDLKDMPFYA.G | Y | 45.04 | 1374.6530 | -1.3 | 688.3329 | 2 | 14.23 | 2163 | 1 | 49 | 59 |  |
| Y.LDLKDMPFY.A | Y | 30.13 | 1140.5525 | 0.0 | 571.2835 | 2 | 14.03 | 2129 | 1 | 50 | 58 |  |
| Y.LDLKDMPFYAGL.C | Y | 25.91 | 1381.6951 | 0.1 | 691.8549 | 2 | 14.80 | 2258 | 1 | 50 | 61 |  |
| H.YLDLKDMPFYAG.L | Y | 18.91 | 1431.6744 | -0.4 | 716.8442 | 2 | 14.10 | 2141 | 1 | 49 | 60 |  |
| total 4 peptides |  |  |  |  |  |  |  |  |  |  |  |  |
| --- | --- | --- | --- | --- | --- | --- | --- | --- | --- | --- | --- | --- |

tr|A0A3Q1K2U7|A0A3Q1K2U7\_ANATE

back to list

  

| Protein Coverage
| Supporting Peptides
|

Protein Coverage:

Supporting Peptides:

| Peptide | Uniq | -10lgP | Mass | ppm | m/z | z | RT | Scan | #Spec | Start | End | PTM |
| --- | --- | --- | --- | --- | --- | --- | --- | --- | --- | --- | --- | --- |
| H.YLDLKDMPFYA.G | Y | 45.04 | 1374.6530 | -1.3 | 688.3329 | 2 | 14.23 | 2163 | 1 | 49 | 59 |  |
| Y.LDLKDMPFY.A | Y | 30.13 | 1140.5525 | 0.0 | 571.2835 | 2 | 14.03 | 2129 | 1 | 50 | 58 |  |
| Y.LDLKDMPFYAGL.C | Y | 25.91 | 1381.6951 | 0.1 | 691.8549 | 2 | 14.80 | 2258 | 1 | 50 | 61 |  |
| H.YLDLKDMPFYAG.L | Y | 18.91 | 1431.6744 | -0.4 | 716.8442 | 2 | 14.10 | 2141 | 1 | 49 | 60 |  |
| total 4 peptides |  |  |  |  |  |  |  |  |  |  |  |  |
| --- | --- | --- | --- | --- | --- | --- | --- | --- | --- | --- | --- | --- |

tr|A0A1A8C566|A0A1A8C566\_9TELE

back to list

  

| Protein Coverage
| Supporting Peptides
|

Protein Coverage:

Supporting Peptides:

| Peptide | Uniq | -10lgP | Mass | ppm | m/z | z | RT | Scan | #Spec | Start | End | PTM |
| --- | --- | --- | --- | --- | --- | --- | --- | --- | --- | --- | --- | --- |
| H.YLDLKDMPFYA.G | Y | 45.04 | 1374.6530 | -1.3 | 688.3329 | 2 | 14.23 | 2163 | 1 | 49 | 59 |  |
| Y.LDLKDMPFY.A | Y | 30.13 | 1140.5525 | 0.0 | 571.2835 | 2 | 14.03 | 2129 | 1 | 50 | 58 |  |
| Y.LDLKDMPFYAGL.C | Y | 25.91 | 1381.6951 | 0.1 | 691.8549 | 2 | 14.80 | 2258 | 1 | 50 | 61 |  |
| H.YLDLKDMPFYAG.L | Y | 18.91 | 1431.6744 | -0.4 | 716.8442 | 2 | 14.10 | 2141 | 1 | 49 | 60 |  |
| total 4 peptides |  |  |  |  |  |  |  |  |  |  |  |  |
| --- | --- | --- | --- | --- | --- | --- | --- | --- | --- | --- | --- | --- |

tr|A0A6P7ISB4|A0A6P7ISB4\_9TELE

back to list

  

| Protein Coverage
| Supporting Peptides
|

Protein Coverage:

Supporting Peptides:

| Peptide | Uniq | -10lgP | Mass | ppm | m/z | z | RT | Scan | #Spec | Start | End | PTM |
| --- | --- | --- | --- | --- | --- | --- | --- | --- | --- | --- | --- | --- |
| H.YLDLKDMPFYA.G | Y | 45.04 | 1374.6530 | -1.3 | 688.3329 | 2 | 14.23 | 2163 | 1 | 49 | 59 |  |
| Y.LDLKDMPFY.A | Y | 30.13 | 1140.5525 | 0.0 | 571.2835 | 2 | 14.03 | 2129 | 1 | 50 | 58 |  |
| Y.LDLKDMPFYAGL.C | Y | 25.91 | 1381.6951 | 0.1 | 691.8549 | 2 | 14.80 | 2258 | 1 | 50 | 61 |  |
| H.YLDLKDMPFYAG.L | Y | 18.91 | 1431.6744 | -0.4 | 716.8442 | 2 | 14.10 | 2141 | 1 | 49 | 60 |  |
| total 4 peptides |  |  |  |  |  |  |  |  |  |  |  |  |
| --- | --- | --- | --- | --- | --- | --- | --- | --- | --- | --- | --- | --- |

tr|A0A3Q4I698|A0A3Q4I698\_NEOBR

back to list

  

| Protein Coverage
| Supporting Peptides
|

Protein Coverage:

Supporting Peptides:

| Peptide | Uniq | -10lgP | Mass | ppm | m/z | z | RT | Scan | #Spec | Start | End | PTM |
| --- | --- | --- | --- | --- | --- | --- | --- | --- | --- | --- | --- | --- |
| H.YLDLKDMPFYA.G | Y | 45.04 | 1374.6530 | -1.3 | 688.3329 | 2 | 14.23 | 2163 | 1 | 49 | 59 |  |
| Y.LDLKDMPFY.A | Y | 30.13 | 1140.5525 | 0.0 | 571.2835 | 2 | 14.03 | 2129 | 1 | 50 | 58 |  |
| Y.LDLKDMPFYAGL.C | Y | 25.91 | 1381.6951 | 0.1 | 691.8549 | 2 | 14.80 | 2258 | 1 | 50 | 61 |  |
| H.YLDLKDMPFYAG.L | Y | 18.91 | 1431.6744 | -0.4 | 716.8442 | 2 | 14.10 | 2141 | 1 | 49 | 60 |  |
| total 4 peptides |  |  |  |  |  |  |  |  |  |  |  |  |
| --- | --- | --- | --- | --- | --- | --- | --- | --- | --- | --- | --- | --- |

tr|A0A3Q3L2T8|A0A3Q3L2T8\_9TELE

back to list

  

| Protein Coverage
| Supporting Peptides
|

Protein Coverage:

Supporting Peptides:

| Peptide | Uniq | -10lgP | Mass | ppm | m/z | z | RT | Scan | #Spec | Start | End | PTM |
| --- | --- | --- | --- | --- | --- | --- | --- | --- | --- | --- | --- | --- |
| H.YLDLKDMPFYA.G | Y | 45.04 | 1374.6530 | -1.3 | 688.3329 | 2 | 14.23 | 2163 | 1 | 49 | 59 |  |
| Y.LDLKDMPFY.A | Y | 30.13 | 1140.5525 | 0.0 | 571.2835 | 2 | 14.03 | 2129 | 1 | 50 | 58 |  |
| Y.LDLKDMPFYAGL.C | Y | 25.91 | 1381.6951 | 0.1 | 691.8549 | 2 | 14.80 | 2258 | 1 | 50 | 61 |  |
| H.YLDLKDMPFYAG.L | Y | 18.91 | 1431.6744 | -0.4 | 716.8442 | 2 | 14.10 | 2141 | 1 | 49 | 60 |  |
| total 4 peptides |  |  |  |  |  |  |  |  |  |  |  |  |
| --- | --- | --- | --- | --- | --- | --- | --- | --- | --- | --- | --- | --- |

tr|A0A6P7NDJ7|A0A6P7NDJ7\_BETSP

back to list

  

| Protein Coverage
| Supporting Peptides
|

Protein Coverage:

Supporting Peptides:

| Peptide | Uniq | -10lgP | Mass | ppm | m/z | z | RT | Scan | #Spec | Start | End | PTM |
| --- | --- | --- | --- | --- | --- | --- | --- | --- | --- | --- | --- | --- |
| H.YLDLKDMPFYA.G | Y | 45.04 | 1374.6530 | -1.3 | 688.3329 | 2 | 14.23 | 2163 | 1 | 49 | 59 |  |
| Y.LDLKDMPFY.A | Y | 30.13 | 1140.5525 | 0.0 | 571.2835 | 2 | 14.03 | 2129 | 1 | 50 | 58 |  |
| Y.LDLKDMPFYAGL.C | Y | 25.91 | 1381.6951 | 0.1 | 691.8549 | 2 | 14.80 | 2258 | 1 | 50 | 61 |  |
| H.YLDLKDMPFYAG.L | Y | 18.91 | 1431.6744 | -0.4 | 716.8442 | 2 | 14.10 | 2141 | 1 | 49 | 60 |  |
| total 4 peptides |  |  |  |  |  |  |  |  |  |  |  |  |
| --- | --- | --- | --- | --- | --- | --- | --- | --- | --- | --- | --- | --- |

tr|A0A3Q3A341|A0A3Q3A341\_KRYMA

back to list

  

| Protein Coverage
| Supporting Peptides
|

Protein Coverage:

Supporting Peptides:

| Peptide | Uniq | -10lgP | Mass | ppm | m/z | z | RT | Scan | #Spec | Start | End | PTM |
| --- | --- | --- | --- | --- | --- | --- | --- | --- | --- | --- | --- | --- |
| H.YLDLKDMPFYA.G | Y | 45.04 | 1374.6530 | -1.3 | 688.3329 | 2 | 14.23 | 2163 | 1 | 49 | 59 |  |
| Y.LDLKDMPFY.A | Y | 30.13 | 1140.5525 | 0.0 | 571.2835 | 2 | 14.03 | 2129 | 1 | 50 | 58 |  |
| Y.LDLKDMPFYAGL.C | Y | 25.91 | 1381.6951 | 0.1 | 691.8549 | 2 | 14.80 | 2258 | 1 | 50 | 61 |  |
| H.YLDLKDMPFYAG.L | Y | 18.91 | 1431.6744 | -0.4 | 716.8442 | 2 | 14.10 | 2141 | 1 | 49 | 60 |  |
| total 4 peptides |  |  |  |  |  |  |  |  |  |  |  |  |
| --- | --- | --- | --- | --- | --- | --- | --- | --- | --- | --- | --- | --- |

tr|A0A3Q4HQ85|A0A3Q4HQ85\_NEOBR

back to list

  

| Protein Coverage
| Supporting Peptides
|

Protein Coverage:

Supporting Peptides:

| Peptide | Uniq | -10lgP | Mass | ppm | m/z | z | RT | Scan | #Spec | Start | End | PTM |
| --- | --- | --- | --- | --- | --- | --- | --- | --- | --- | --- | --- | --- |
| H.YLDLKDMPFYA.G | Y | 45.04 | 1374.6530 | -1.3 | 688.3329 | 2 | 14.23 | 2163 | 1 | 49 | 59 |  |
| Y.LDLKDMPFY.A | Y | 30.13 | 1140.5525 | 0.0 | 571.2835 | 2 | 14.03 | 2129 | 1 | 50 | 58 |  |
| Y.LDLKDMPFYAGL.C | Y | 25.91 | 1381.6951 | 0.1 | 691.8549 | 2 | 14.80 | 2258 | 1 | 50 | 61 |  |
| H.YLDLKDMPFYAG.L | Y | 18.91 | 1431.6744 | -0.4 | 716.8442 | 2 | 14.10 | 2141 | 1 | 49 | 60 |  |
| total 4 peptides |  |  |  |  |  |  |  |  |  |  |  |  |
| --- | --- | --- | --- | --- | --- | --- | --- | --- | --- | --- | --- | --- |

tr|A0A3P8Q104|A0A3P8Q104\_ASTCA

back to list

  

| Protein Coverage
| Supporting Peptides
|

Protein Coverage:

Supporting Peptides:

| Peptide | Uniq | -10lgP | Mass | ppm | m/z | z | RT | Scan | #Spec | Start | End | PTM |
| --- | --- | --- | --- | --- | --- | --- | --- | --- | --- | --- | --- | --- |
| H.YLDLKDMPFYA.G | Y | 45.04 | 1374.6530 | -1.3 | 688.3329 | 2 | 14.23 | 2163 | 1 | 51 | 61 |  |
| Y.LDLKDMPFY.A | Y | 30.13 | 1140.5525 | 0.0 | 571.2835 | 2 | 14.03 | 2129 | 1 | 52 | 60 |  |
| Y.LDLKDMPFYAGL.C | Y | 25.91 | 1381.6951 | 0.1 | 691.8549 | 2 | 14.80 | 2258 | 1 | 52 | 63 |  |
| H.YLDLKDMPFYAG.L | Y | 18.91 | 1431.6744 | -0.4 | 716.8442 | 2 | 14.10 | 2141 | 1 | 51 | 62 |  |
| total 4 peptides |  |  |  |  |  |  |  |  |  |  |  |  |
| --- | --- | --- | --- | --- | --- | --- | --- | --- | --- | --- | --- | --- |

tr|A0A3Q3CUD8|A0A3Q3CUD8\_HAPBU

back to list

  

| Protein Coverage
| Supporting Peptides
|

Protein Coverage:

Supporting Peptides:

| Peptide | Uniq | -10lgP | Mass | ppm | m/z | z | RT | Scan | #Spec | Start | End | PTM |
| --- | --- | --- | --- | --- | --- | --- | --- | --- | --- | --- | --- | --- |
| H.YLDLKDMPFYA.G | Y | 45.04 | 1374.6530 | -1.3 | 688.3329 | 2 | 14.23 | 2163 | 1 | 51 | 61 |  |
| Y.LDLKDMPFY.A | Y | 30.13 | 1140.5525 | 0.0 | 571.2835 | 2 | 14.03 | 2129 | 1 | 52 | 60 |  |
| Y.LDLKDMPFYAGL.C | Y | 25.91 | 1381.6951 | 0.1 | 691.8549 | 2 | 14.80 | 2258 | 1 | 52 | 63 |  |
| H.YLDLKDMPFYAG.L | Y | 18.91 | 1431.6744 | -0.4 | 716.8442 | 2 | 14.10 | 2141 | 1 | 51 | 62 |  |
| total 4 peptides |  |  |  |  |  |  |  |  |  |  |  |  |
| --- | --- | --- | --- | --- | --- | --- | --- | --- | --- | --- | --- | --- |

tr|A0A3Q4I6B8|A0A3Q4I6B8\_NEOBR

back to list

  

| Protein Coverage
| Supporting Peptides
|

Protein Coverage:

Supporting Peptides:

| Peptide | Uniq | -10lgP | Mass | ppm | m/z | z | RT | Scan | #Spec | Start | End | PTM |
| --- | --- | --- | --- | --- | --- | --- | --- | --- | --- | --- | --- | --- |
| H.YLDLKDMPFYA.G | Y | 45.04 | 1374.6530 | -1.3 | 688.3329 | 2 | 14.23 | 2163 | 1 | 51 | 61 |  |
| Y.LDLKDMPFY.A | Y | 30.13 | 1140.5525 | 0.0 | 571.2835 | 2 | 14.03 | 2129 | 1 | 52 | 60 |  |
| Y.LDLKDMPFYAGL.C | Y | 25.91 | 1381.6951 | 0.1 | 691.8549 | 2 | 14.80 | 2258 | 1 | 52 | 63 |  |
| H.YLDLKDMPFYAG.L | Y | 18.91 | 1431.6744 | -0.4 | 716.8442 | 2 | 14.10 | 2141 | 1 | 51 | 62 |  |
| total 4 peptides |  |  |  |  |  |  |  |  |  |  |  |  |
| --- | --- | --- | --- | --- | --- | --- | --- | --- | --- | --- | --- | --- |

tr|A0A3Q0QSW6|A0A3Q0QSW6\_AMPCI

back to list

  

| Protein Coverage
| Supporting Peptides
|

Protein Coverage:

Supporting Peptides:

| Peptide | Uniq | -10lgP | Mass | ppm | m/z | z | RT | Scan | #Spec | Start | End | PTM |
| --- | --- | --- | --- | --- | --- | --- | --- | --- | --- | --- | --- | --- |
| H.YLDLKDMPFYA.G | Y | 45.04 | 1374.6530 | -1.3 | 688.3329 | 2 | 14.23 | 2163 | 1 | 49 | 59 |  |
| Y.LDLKDMPFY.A | Y | 30.13 | 1140.5525 | 0.0 | 571.2835 | 2 | 14.03 | 2129 | 1 | 50 | 58 |  |
| Y.LDLKDMPFYAGL.C | Y | 25.91 | 1381.6951 | 0.1 | 691.8549 | 2 | 14.80 | 2258 | 1 | 50 | 61 |  |
| H.YLDLKDMPFYAG.L | Y | 18.91 | 1431.6744 | -0.4 | 716.8442 | 2 | 14.10 | 2141 | 1 | 49 | 60 |  |
| total 4 peptides |  |  |  |  |  |  |  |  |  |  |  |  |
| --- | --- | --- | --- | --- | --- | --- | --- | --- | --- | --- | --- | --- |

tr|A0A3Q0QYR1|A0A3Q0QYR1\_AMPCI

back to list

  

| Protein Coverage
| Supporting Peptides
|

Protein Coverage:

Supporting Peptides:

| Peptide | Uniq | -10lgP | Mass | ppm | m/z | z | RT | Scan | #Spec | Start | End | PTM |
| --- | --- | --- | --- | --- | --- | --- | --- | --- | --- | --- | --- | --- |
| H.YLDLKDMPFYA.G | Y | 45.04 | 1374.6530 | -1.3 | 688.3329 | 2 | 14.23 | 2163 | 1 | 52 | 62 |  |
| Y.LDLKDMPFY.A | Y | 30.13 | 1140.5525 | 0.0 | 571.2835 | 2 | 14.03 | 2129 | 1 | 53 | 61 |  |
| Y.LDLKDMPFYAGL.C | Y | 25.91 | 1381.6951 | 0.1 | 691.8549 | 2 | 14.80 | 2258 | 1 | 53 | 64 |  |
| H.YLDLKDMPFYAG.L | Y | 18.91 | 1431.6744 | -0.4 | 716.8442 | 2 | 14.10 | 2141 | 1 | 52 | 63 |  |
| total 4 peptides |  |  |  |  |  |  |  |  |  |  |  |  |
| --- | --- | --- | --- | --- | --- | --- | --- | --- | --- | --- | --- | --- |

tr|A0A3Q3R7S1|A0A3Q3R7S1\_MONAL

back to list

  

| Protein Coverage
| Supporting Peptides
|

Protein Coverage:

Supporting Peptides:

| Peptide | Uniq | -10lgP | Mass | ppm | m/z | z | RT | Scan | #Spec | Start | End | PTM |
| --- | --- | --- | --- | --- | --- | --- | --- | --- | --- | --- | --- | --- |
| H.YLDLKDMPFYA.G | Y | 45.04 | 1374.6530 | -1.3 | 688.3329 | 2 | 14.23 | 2163 | 1 | 52 | 62 |  |
| Y.LDLKDMPFY.A | Y | 30.13 | 1140.5525 | 0.0 | 571.2835 | 2 | 14.03 | 2129 | 1 | 53 | 61 |  |
| Y.LDLKDMPFYAGL.C | Y | 25.91 | 1381.6951 | 0.1 | 691.8549 | 2 | 14.80 | 2258 | 1 | 53 | 64 |  |
| H.YLDLKDMPFYAG.L | Y | 18.91 | 1431.6744 | -0.4 | 716.8442 | 2 | 14.10 | 2141 | 1 | 52 | 63 |  |
| total 4 peptides |  |  |  |  |  |  |  |  |  |  |  |  |
| --- | --- | --- | --- | --- | --- | --- | --- | --- | --- | --- | --- | --- |

tr|A0A3Q0QXA4|A0A3Q0QXA4\_AMPCI

back to list

  

| Protein Coverage
| Supporting Peptides
|

Protein Coverage:

Supporting Peptides:

| Peptide | Uniq | -10lgP | Mass | ppm | m/z | z | RT | Scan | #Spec | Start | End | PTM |
| --- | --- | --- | --- | --- | --- | --- | --- | --- | --- | --- | --- | --- |
| H.YLDLKDMPFYA.G | Y | 45.04 | 1374.6530 | -1.3 | 688.3329 | 2 | 14.23 | 2163 | 1 | 53 | 63 |  |
| Y.LDLKDMPFY.A | Y | 30.13 | 1140.5525 | 0.0 | 571.2835 | 2 | 14.03 | 2129 | 1 | 54 | 62 |  |
| Y.LDLKDMPFYAGL.C | Y | 25.91 | 1381.6951 | 0.1 | 691.8549 | 2 | 14.80 | 2258 | 1 | 54 | 65 |  |
| H.YLDLKDMPFYAG.L | Y | 18.91 | 1431.6744 | -0.4 | 716.8442 | 2 | 14.10 | 2141 | 1 | 53 | 64 |  |
| total 4 peptides |  |  |  |  |  |  |  |  |  |  |  |  |
| --- | --- | --- | --- | --- | --- | --- | --- | --- | --- | --- | --- | --- |

tr|M4ADR4|M4ADR4\_XIPMA

back to list

  

| Protein Coverage
| Supporting Peptides
|

Protein Coverage:

Supporting Peptides:

| Peptide | Uniq | -10lgP | Mass | ppm | m/z | z | RT | Scan | #Spec | Start | End | PTM |
| --- | --- | --- | --- | --- | --- | --- | --- | --- | --- | --- | --- | --- |
| H.YLDLKDMPFYA.G | Y | 45.04 | 1374.6530 | -1.3 | 688.3329 | 2 | 14.23 | 2163 | 1 | 62 | 72 |  |
| Y.LDLKDMPFY.A | Y | 30.13 | 1140.5525 | 0.0 | 571.2835 | 2 | 14.03 | 2129 | 1 | 63 | 71 |  |
| Y.LDLKDMPFYAGL.C | Y | 25.91 | 1381.6951 | 0.1 | 691.8549 | 2 | 14.80 | 2258 | 1 | 63 | 74 |  |
| H.YLDLKDMPFYAG.L | Y | 18.91 | 1431.6744 | -0.4 | 716.8442 | 2 | 14.10 | 2141 | 1 | 62 | 73 |  |
| total 4 peptides |  |  |  |  |  |  |  |  |  |  |  |  |
| --- | --- | --- | --- | --- | --- | --- | --- | --- | --- | --- | --- | --- |

tr|A0A1A8J8K8|A0A1A8J8K8\_NOTKU

back to list

  

| Protein Coverage
| Supporting Peptides
|

Protein Coverage:

Supporting Peptides:

| Peptide | Uniq | -10lgP | Mass | ppm | m/z | z | RT | Scan | #Spec | Start | End | PTM |
| --- | --- | --- | --- | --- | --- | --- | --- | --- | --- | --- | --- | --- |
| H.YLDLKDMPFYA.G | Y | 45.04 | 1374.6530 | -1.3 | 688.3329 | 2 | 14.23 | 2163 | 1 | 62 | 72 |  |
| Y.LDLKDMPFY.A | Y | 30.13 | 1140.5525 | 0.0 | 571.2835 | 2 | 14.03 | 2129 | 1 | 63 | 71 |  |
| Y.LDLKDMPFYAGL.C | Y | 25.91 | 1381.6951 | 0.1 | 691.8549 | 2 | 14.80 | 2258 | 1 | 63 | 74 |  |
| H.YLDLKDMPFYAG.L | Y | 18.91 | 1431.6744 | -0.4 | 716.8442 | 2 | 14.10 | 2141 | 1 | 62 | 73 |  |
| total 4 peptides |  |  |  |  |  |  |  |  |  |  |  |  |
| --- | --- | --- | --- | --- | --- | --- | --- | --- | --- | --- | --- | --- |

tr|A0A1A7XFD3|A0A1A7XFD3\_9TELE

back to list

  

| Protein Coverage
| Supporting Peptides
|

Protein Coverage:

Supporting Peptides:

| Peptide | Uniq | -10lgP | Mass | ppm | m/z | z | RT | Scan | #Spec | Start | End | PTM |
| --- | --- | --- | --- | --- | --- | --- | --- | --- | --- | --- | --- | --- |
| H.YLDLKDMPFYA.G | Y | 45.04 | 1374.6530 | -1.3 | 688.3329 | 2 | 14.23 | 2163 | 1 | 82 | 92 |  |
| Y.LDLKDMPFY.A | Y | 30.13 | 1140.5525 | 0.0 | 571.2835 | 2 | 14.03 | 2129 | 1 | 83 | 91 |  |
| Y.LDLKDMPFYAGL.C | Y | 25.91 | 1381.6951 | 0.1 | 691.8549 | 2 | 14.80 | 2258 | 1 | 83 | 94 |  |
| H.YLDLKDMPFYAG.L | Y | 18.91 | 1431.6744 | -0.4 | 716.8442 | 2 | 14.10 | 2141 | 1 | 82 | 93 |  |
| total 4 peptides |  |  |  |  |  |  |  |  |  |  |  |  |
| --- | --- | --- | --- | --- | --- | --- | --- | --- | --- | --- | --- | --- |

tr|A0A0S7LYJ8|A0A0S7LYJ8\_9TELE

back to list

  

| Protein Coverage
| Supporting Peptides
|

Protein Coverage:

Supporting Peptides:

| Peptide | Uniq | -10lgP | Mass | ppm | m/z | z | RT | Scan | #Spec | Start | End | PTM |
| --- | --- | --- | --- | --- | --- | --- | --- | --- | --- | --- | --- | --- |
| H.YLDLKDMPFYA.G | Y | 45.04 | 1374.6530 | -1.3 | 688.3329 | 2 | 14.23 | 2163 | 1 | 74 | 84 |  |
| Y.LDLKDMPFY.A | Y | 30.13 | 1140.5525 | 0.0 | 571.2835 | 2 | 14.03 | 2129 | 1 | 75 | 83 |  |
| Y.LDLKDMPFYAGL.C | Y | 25.91 | 1381.6951 | 0.1 | 691.8549 | 2 | 14.80 | 2258 | 1 | 75 | 86 |  |
| H.YLDLKDMPFYAG.L | Y | 18.91 | 1431.6744 | -0.4 | 716.8442 | 2 | 14.10 | 2141 | 1 | 74 | 85 |  |
| total 4 peptides |  |  |  |  |  |  |  |  |  |  |  |  |
| --- | --- | --- | --- | --- | --- | --- | --- | --- | --- | --- | --- | --- |

tr|H3CAK5|H3CAK5\_TETNG

back to list

  

| Protein Coverage
| Supporting Peptides
|

Protein Coverage:

Supporting Peptides:

| Peptide | Uniq | -10lgP | Mass | ppm | m/z | z | RT | Scan | #Spec | Start | End | PTM |
| --- | --- | --- | --- | --- | --- | --- | --- | --- | --- | --- | --- | --- |
| Q.SIEDPFDQDDWENWAK.F | Y | 43.87 | 1993.8330 | 2.3 | 997.9260 | 2 | 14.25 | 2166 | 1 | 198 | 213 |  |
| K.YGKDATNVGDEGGFAPN.I | Y | 32.36 | 1710.7485 | -0.8 | 856.3809 | 2 | 11.09 | 1617 | 1 | 107 | 123 |  |
| Q.IVGDDLTVTNPKR.I | Y | 20.07 | 1426.7780 | 0.0 | 476.5999 | 3 | 10.77 | 1559 | 2 | 222 | 234 |  |
| Q.SIEDPFDQDDWE.N | Y | 19.74 | 1494.5786 | -0.3 | 748.2963 | 2 | 14.19 | 2156 | 1 | 198 | 209 |  |
| total 4 peptides |  |  |  |  |  |  |  |  |  |  |  |  |
| --- | --- | --- | --- | --- | --- | --- | --- | --- | --- | --- | --- | --- |

tr|A0A3B3D6F1|A0A3B3D6F1\_ORYME

back to list

  

| Protein Coverage
| Supporting Peptides
|

Protein Coverage:

Supporting Peptides:

| Peptide | Uniq | -10lgP | Mass | ppm | m/z | z | RT | Scan | #Spec | Start | End | PTM |
| --- | --- | --- | --- | --- | --- | --- | --- | --- | --- | --- | --- | --- |
| Q.SIEDPFDQDDWENWAK.F | Y | 43.87 | 1993.8330 | 2.3 | 997.9260 | 2 | 14.25 | 2166 | 1 | 248 | 263 |  |
| K.YGKDATNVGDEGGFAPN.I | Y | 32.36 | 1710.7485 | -0.8 | 856.3809 | 2 | 11.09 | 1617 | 1 | 157 | 173 |  |
| Q.IVGDDLTVTNPKR.I | Y | 20.07 | 1426.7780 | 0.0 | 476.5999 | 3 | 10.77 | 1559 | 2 | 272 | 284 |  |
| Q.SIEDPFDQDDWE.N | Y | 19.74 | 1494.5786 | -0.3 | 748.2963 | 2 | 14.19 | 2156 | 1 | 248 | 259 |  |
| total 4 peptides |  |  |  |  |  |  |  |  |  |  |  |  |
| --- | --- | --- | --- | --- | --- | --- | --- | --- | --- | --- | --- | --- |

tr|A0A3B3BLA1|A0A3B3BLA1\_ORYME

back to list

  

| Protein Coverage
| Supporting Peptides
|

Protein Coverage:

Supporting Peptides:

| Peptide | Uniq | -10lgP | Mass | ppm | m/z | z | RT | Scan | #Spec | Start | End | PTM |
| --- | --- | --- | --- | --- | --- | --- | --- | --- | --- | --- | --- | --- |
| Q.SIEDPFDQDDWENWAK.F | Y | 43.87 | 1993.8330 | 2.3 | 997.9260 | 2 | 14.25 | 2166 | 1 | 291 | 306 |  |
| K.YGKDATNVGDEGGFAPN.I | Y | 32.36 | 1710.7485 | -0.8 | 856.3809 | 2 | 11.09 | 1617 | 1 | 200 | 216 |  |
| Q.IVGDDLTVTNPKR.I | Y | 20.07 | 1426.7780 | 0.0 | 476.5999 | 3 | 10.77 | 1559 | 2 | 315 | 327 |  |
| Q.SIEDPFDQDDWE.N | Y | 19.74 | 1494.5786 | -0.3 | 748.2963 | 2 | 14.19 | 2156 | 1 | 291 | 302 |  |
| total 4 peptides |  |  |  |  |  |  |  |  |  |  |  |  |
| --- | --- | --- | --- | --- | --- | --- | --- | --- | --- | --- | --- | --- |

tr|A0A3B4CPC9|A0A3B4CPC9\_PYGNA

back to list

  

| Protein Coverage
| Supporting Peptides
|

Protein Coverage:

Supporting Peptides:

| Peptide | Uniq | -10lgP | Mass | ppm | m/z | z | RT | Scan | #Spec | Start | End | PTM |
| --- | --- | --- | --- | --- | --- | --- | --- | --- | --- | --- | --- | --- |
| M.SIEDPFDQDDWENWAK.F | Y | 43.87 | 1993.8330 | 2.3 | 997.9260 | 2 | 14.25 | 2166 | 1 | 291 | 306 |  |
| K.YGKDATNVGDEGGFAPN.I | Y | 32.36 | 1710.7485 | -0.8 | 856.3809 | 2 | 11.09 | 1617 | 1 | 200 | 216 |  |
| Q.IVGDDLTVTNPKR.I | Y | 20.07 | 1426.7780 | 0.0 | 476.5999 | 3 | 10.77 | 1559 | 2 | 315 | 327 |  |
| M.SIEDPFDQDDWE.N | Y | 19.74 | 1494.5786 | -0.3 | 748.2963 | 2 | 14.19 | 2156 | 1 | 291 | 302 |  |
| total 4 peptides |  |  |  |  |  |  |  |  |  |  |  |  |
| --- | --- | --- | --- | --- | --- | --- | --- | --- | --- | --- | --- | --- |

tr|A0A6M4C6H3|A0A6M4C6H3\_DIOHY

back to list

  

| Protein Coverage
| Supporting Peptides
|

Protein Coverage:

Supporting Peptides:

| Peptide | Uniq | -10lgP | Mass | ppm | m/z | z | RT | Scan | #Spec | Start | End | PTM |
| --- | --- | --- | --- | --- | --- | --- | --- | --- | --- | --- | --- | --- |
| Q.SIEDPFDQDDWENWAK.F | Y | 43.87 | 1993.8330 | 2.3 | 997.9260 | 2 | 14.25 | 2166 | 1 | 291 | 306 |  |
| K.YGKDATNVGDEGGFAPN.I | Y | 32.36 | 1710.7485 | -0.8 | 856.3809 | 2 | 11.09 | 1617 | 1 | 200 | 216 |  |
| Q.IVGDDLTVTNPKR.I | Y | 20.07 | 1426.7780 | 0.0 | 476.5999 | 3 | 10.77 | 1559 | 2 | 315 | 327 |  |
| Q.SIEDPFDQDDWE.N | Y | 19.74 | 1494.5786 | -0.3 | 748.2963 | 2 | 14.19 | 2156 | 1 | 291 | 302 |  |
| total 4 peptides |  |  |  |  |  |  |  |  |  |  |  |  |
| --- | --- | --- | --- | --- | --- | --- | --- | --- | --- | --- | --- | --- |

tr|A0A0F8AN77|A0A0F8AN77\_LARCR

back to list

  

| Protein Coverage
| Supporting Peptides
|

Protein Coverage:

Supporting Peptides:

| Peptide | Uniq | -10lgP | Mass | ppm | m/z | z | RT | Scan | #Spec | Start | End | PTM |
| --- | --- | --- | --- | --- | --- | --- | --- | --- | --- | --- | --- | --- |
| Q.SIEDPFDQDDWENWAK.F | Y | 43.87 | 1993.8330 | 2.3 | 997.9260 | 2 | 14.25 | 2166 | 1 | 291 | 306 |  |
| K.YGKDATNVGDEGGFAPN.I | Y | 32.36 | 1710.7485 | -0.8 | 856.3809 | 2 | 11.09 | 1617 | 1 | 200 | 216 |  |
| Q.IVGDDLTVTNPKR.I | Y | 20.07 | 1426.7780 | 0.0 | 476.5999 | 3 | 10.77 | 1559 | 2 | 315 | 327 |  |
| Q.SIEDPFDQDDWE.N | Y | 19.74 | 1494.5786 | -0.3 | 748.2963 | 2 | 14.19 | 2156 | 1 | 291 | 302 |  |
| total 4 peptides |  |  |  |  |  |  |  |  |  |  |  |  |
| --- | --- | --- | --- | --- | --- | --- | --- | --- | --- | --- | --- | --- |

tr|A0A437CFA6|A0A437CFA6\_ORYJA

back to list

  

| Protein Coverage
| Supporting Peptides
|

Protein Coverage:

Supporting Peptides:

| Peptide | Uniq | -10lgP | Mass | ppm | m/z | z | RT | Scan | #Spec | Start | End | PTM |
| --- | --- | --- | --- | --- | --- | --- | --- | --- | --- | --- | --- | --- |
| Q.SIEDPFDQDDWENWAK.F | Y | 43.87 | 1993.8330 | 2.3 | 997.9260 | 2 | 14.25 | 2166 | 1 | 390 | 405 |  |
| K.YGKDATNVGDEGGFAPN.I | Y | 32.36 | 1710.7485 | -0.8 | 856.3809 | 2 | 11.09 | 1617 | 1 | 299 | 315 |  |
| Q.IVGDDLTVTNPKR.I | Y | 20.07 | 1426.7780 | 0.0 | 476.5999 | 3 | 10.77 | 1559 | 2 | 414 | 426 |  |
| Q.SIEDPFDQDDWE.N | Y | 19.74 | 1494.5786 | -0.3 | 748.2963 | 2 | 14.19 | 2156 | 1 | 390 | 401 |  |
| total 4 peptides |  |  |  |  |  |  |  |  |  |  |  |  |
| --- | --- | --- | --- | --- | --- | --- | --- | --- | --- | --- | --- | --- |

tr|A0A671TWQ7|A0A671TWQ7\_SPAAU

back to list

  

| Protein Coverage
| Supporting Peptides
|

Protein Coverage:

Supporting Peptides:

| Peptide | Uniq | -10lgP | Mass | ppm | m/z | z | RT | Scan | #Spec | Start | End | PTM |
| --- | --- | --- | --- | --- | --- | --- | --- | --- | --- | --- | --- | --- |
| Q.SIEDPFDQDDWENWAK.F | Y | 43.87 | 1993.8330 | 2.3 | 997.9260 | 2 | 14.25 | 2166 | 1 | 208 | 223 |  |
| K.YGKDATNVGDEGGFAPN.I | Y | 32.36 | 1710.7485 | -0.8 | 856.3809 | 2 | 11.09 | 1617 | 1 | 117 | 133 |  |
| Q.IVGDDLTVTNPKR.I | Y | 20.07 | 1426.7780 | 0.0 | 476.5999 | 3 | 10.77 | 1559 | 2 | 232 | 244 |  |
| Q.SIEDPFDQDDWE.N | Y | 19.74 | 1494.5786 | -0.3 | 748.2963 | 2 | 14.19 | 2156 | 1 | 208 | 219 |  |
| total 4 peptides |  |  |  |  |  |  |  |  |  |  |  |  |
| --- | --- | --- | --- | --- | --- | --- | --- | --- | --- | --- | --- | --- |

tr|A0A671TWN1|A0A671TWN1\_SPAAU

back to list

  

| Protein Coverage
| Supporting Peptides
|

Protein Coverage:

Supporting Peptides:

| Peptide | Uniq | -10lgP | Mass | ppm | m/z | z | RT | Scan | #Spec | Start | End | PTM |
| --- | --- | --- | --- | --- | --- | --- | --- | --- | --- | --- | --- | --- |
| Q.SIEDPFDQDDWENWAK.F | Y | 43.87 | 1993.8330 | 2.3 | 997.9260 | 2 | 14.25 | 2166 | 1 | 219 | 234 |  |
| K.YGKDATNVGDEGGFAPN.I | Y | 32.36 | 1710.7485 | -0.8 | 856.3809 | 2 | 11.09 | 1617 | 1 | 128 | 144 |  |
| Q.IVGDDLTVTNPKR.I | Y | 20.07 | 1426.7780 | 0.0 | 476.5999 | 3 | 10.77 | 1559 | 2 | 243 | 255 |  |
| Q.SIEDPFDQDDWE.N | Y | 19.74 | 1494.5786 | -0.3 | 748.2963 | 2 | 14.19 | 2156 | 1 | 219 | 230 |  |
| total 4 peptides |  |  |  |  |  |  |  |  |  |  |  |  |
| --- | --- | --- | --- | --- | --- | --- | --- | --- | --- | --- | --- | --- |

tr|A0A671TZ14|A0A671TZ14\_SPAAU

back to list

  

| Protein Coverage
| Supporting Peptides
|

Protein Coverage:

Supporting Peptides:

| Peptide | Uniq | -10lgP | Mass | ppm | m/z | z | RT | Scan | #Spec | Start | End | PTM |
| --- | --- | --- | --- | --- | --- | --- | --- | --- | --- | --- | --- | --- |
| Q.SIEDPFDQDDWENWAK.F | Y | 43.87 | 1993.8330 | 2.3 | 997.9260 | 2 | 14.25 | 2166 | 1 | 198 | 213 |  |
| K.YGKDATNVGDEGGFAPN.I | Y | 32.36 | 1710.7485 | -0.8 | 856.3809 | 2 | 11.09 | 1617 | 1 | 107 | 123 |  |
| Q.IVGDDLTVTNPKR.I | Y | 20.07 | 1426.7780 | 0.0 | 476.5999 | 3 | 10.77 | 1559 | 2 | 222 | 234 |  |
| Q.SIEDPFDQDDWE.N | Y | 19.74 | 1494.5786 | -0.3 | 748.2963 | 2 | 14.19 | 2156 | 1 | 198 | 209 |  |
| total 4 peptides |  |  |  |  |  |  |  |  |  |  |  |  |
| --- | --- | --- | --- | --- | --- | --- | --- | --- | --- | --- | --- | --- |

tr|F5BZS7|F5BZS7\_EPIBR

back to list

  

| Protein Coverage
| Supporting Peptides
|

Protein Coverage:

Supporting Peptides:

| Peptide | Uniq | -10lgP | Mass | ppm | m/z | z | RT | Scan | #Spec | Start | End | PTM |
| --- | --- | --- | --- | --- | --- | --- | --- | --- | --- | --- | --- | --- |
| Q.SIEDPFDQDDWENWAK.F | Y | 43.87 | 1993.8330 | 2.3 | 997.9260 | 2 | 14.25 | 2166 | 1 | 291 | 306 |  |
| K.YGKDATNVGDEGGFAPN.I | Y | 32.36 | 1710.7485 | -0.8 | 856.3809 | 2 | 11.09 | 1617 | 1 | 200 | 216 |  |
| Q.IVGDDLTVTNPKR.I | Y | 20.07 | 1426.7780 | 0.0 | 476.5999 | 3 | 10.77 | 1559 | 2 | 315 | 327 |  |
| Q.SIEDPFDQDDWE.N | Y | 19.74 | 1494.5786 | -0.3 | 748.2963 | 2 | 14.19 | 2156 | 1 | 291 | 302 |  |
| total 4 peptides |  |  |  |  |  |  |  |  |  |  |  |  |
| --- | --- | --- | --- | --- | --- | --- | --- | --- | --- | --- | --- | --- |

tr|Q4TBD1|Q4TBD1\_TETNG

back to list

  

| Protein Coverage
| Supporting Peptides
|

Protein Coverage:

Supporting Peptides:

| Peptide | Uniq | -10lgP | Mass | ppm | m/z | z | RT | Scan | #Spec | Start | End | PTM |
| --- | --- | --- | --- | --- | --- | --- | --- | --- | --- | --- | --- | --- |
| Q.SIEDPFDQDDWENWAK.F | Y | 43.87 | 1993.8330 | 2.3 | 997.9260 | 2 | 14.25 | 2166 | 1 | 291 | 306 |  |
| K.YGKDATNVGDEGGFAPN.I | Y | 32.36 | 1710.7485 | -0.8 | 856.3809 | 2 | 11.09 | 1617 | 1 | 200 | 216 |  |
| Q.IVGDDLTVTNPKR.I | Y | 20.07 | 1426.7780 | 0.0 | 476.5999 | 3 | 10.77 | 1559 | 2 | 315 | 327 |  |
| Q.SIEDPFDQDDWE.N | Y | 19.74 | 1494.5786 | -0.3 | 748.2963 | 2 | 14.19 | 2156 | 1 | 291 | 302 |  |
| total 4 peptides |  |  |  |  |  |  |  |  |  |  |  |  |
| --- | --- | --- | --- | --- | --- | --- | --- | --- | --- | --- | --- | --- |

tr|A0A2U9B0J8|A0A2U9B0J8\_SCOMX

back to list

  

| Protein Coverage
| Supporting Peptides
|

Protein Coverage:

Supporting Peptides:

| Peptide | Uniq | -10lgP | Mass | ppm | m/z | z | RT | Scan | #Spec | Start | End | PTM |
| --- | --- | --- | --- | --- | --- | --- | --- | --- | --- | --- | --- | --- |
| Q.SIEDPFDQDDWENWAK.F | Y | 43.87 | 1993.8330 | 2.3 | 997.9260 | 2 | 14.25 | 2166 | 1 | 291 | 306 |  |
| K.YGKDATNVGDEGGFAPN.I | Y | 32.36 | 1710.7485 | -0.8 | 856.3809 | 2 | 11.09 | 1617 | 1 | 200 | 216 |  |
| Q.IVGDDLTVTNPKR.I | Y | 20.07 | 1426.7780 | 0.0 | 476.5999 | 3 | 10.77 | 1559 | 2 | 315 | 327 |  |
| Q.SIEDPFDQDDWE.N | Y | 19.74 | 1494.5786 | -0.3 | 748.2963 | 2 | 14.19 | 2156 | 1 | 291 | 302 |  |
| total 4 peptides |  |  |  |  |  |  |  |  |  |  |  |  |
| --- | --- | --- | --- | --- | --- | --- | --- | --- | --- | --- | --- | --- |

tr|A0A6J2P9G6|A0A6J2P9G6\_COTGO

back to list

  

| Protein Coverage
| Supporting Peptides
|

Protein Coverage:

Supporting Peptides:

| Peptide | Uniq | -10lgP | Mass | ppm | m/z | z | RT | Scan | #Spec | Start | End | PTM |
| --- | --- | --- | --- | --- | --- | --- | --- | --- | --- | --- | --- | --- |
| Q.SIEDPFDQDDWENWAK.F | Y | 43.87 | 1993.8330 | 2.3 | 997.9260 | 2 | 14.25 | 2166 | 1 | 291 | 306 |  |
| K.YGKDATNVGDEGGFAPN.I | Y | 32.36 | 1710.7485 | -0.8 | 856.3809 | 2 | 11.09 | 1617 | 1 | 200 | 216 |  |
| Q.IVGDDLTVTNPKR.I | Y | 20.07 | 1426.7780 | 0.0 | 476.5999 | 3 | 10.77 | 1559 | 2 | 315 | 327 |  |
| Q.SIEDPFDQDDWE.N | Y | 19.74 | 1494.5786 | -0.3 | 748.2963 | 2 | 14.19 | 2156 | 1 | 291 | 302 |  |
| total 4 peptides |  |  |  |  |  |  |  |  |  |  |  |  |
| --- | --- | --- | --- | --- | --- | --- | --- | --- | --- | --- | --- | --- |

tr|D6PVP1|D6PVP1\_EPICO

back to list

  

| Protein Coverage
| Supporting Peptides
|

Protein Coverage:

Supporting Peptides:

| Peptide | Uniq | -10lgP | Mass | ppm | m/z | z | RT | Scan | #Spec | Start | End | PTM |
| --- | --- | --- | --- | --- | --- | --- | --- | --- | --- | --- | --- | --- |
| Q.SIEDPFDQDDWENWAK.F | Y | 43.87 | 1993.8330 | 2.3 | 997.9260 | 2 | 14.25 | 2166 | 1 | 291 | 306 |  |
| K.YGKDATNVGDEGGFAPN.I | Y | 32.36 | 1710.7485 | -0.8 | 856.3809 | 2 | 11.09 | 1617 | 1 | 200 | 216 |  |
| Q.IVGDDLTVTNPKR.I | Y | 20.07 | 1426.7780 | 0.0 | 476.5999 | 3 | 10.77 | 1559 | 2 | 315 | 327 |  |
| Q.SIEDPFDQDDWE.N | Y | 19.74 | 1494.5786 | -0.3 | 748.2963 | 2 | 14.19 | 2156 | 1 | 291 | 302 |  |
| total 4 peptides |  |  |  |  |  |  |  |  |  |  |  |  |
| --- | --- | --- | --- | --- | --- | --- | --- | --- | --- | --- | --- | --- |

tr|A0A6A4S9A7|A0A6A4S9A7\_SCOMX

back to list

  

| Protein Coverage
| Supporting Peptides
|

Protein Coverage:

Supporting Peptides:

| Peptide | Uniq | -10lgP | Mass | ppm | m/z | z | RT | Scan | #Spec | Start | End | PTM |
| --- | --- | --- | --- | --- | --- | --- | --- | --- | --- | --- | --- | --- |
| Q.SIEDPFDQDDWENWAK.F | Y | 43.87 | 1993.8330 | 2.3 | 997.9260 | 2 | 14.25 | 2166 | 1 | 568 | 583 |  |
| K.YGKDATNVGDEGGFAPN.I | Y | 32.36 | 1710.7485 | -0.8 | 856.3809 | 2 | 11.09 | 1617 | 1 | 477 | 493 |  |
| Q.IVGDDLTVTNPKR.I | Y | 20.07 | 1426.7780 | 0.0 | 476.5999 | 3 | 10.77 | 1559 | 2 | 592 | 604 |  |
| Q.SIEDPFDQDDWE.N | Y | 19.74 | 1494.5786 | -0.3 | 748.2963 | 2 | 14.19 | 2156 | 1 | 568 | 579 |  |
| total 4 peptides |  |  |  |  |  |  |  |  |  |  |  |  |
| --- | --- | --- | --- | --- | --- | --- | --- | --- | --- | --- | --- | --- |

tr|A0A6P7KDL5|A0A6P7KDL5\_9TELE

back to list

  

| Protein Coverage
| Supporting Peptides
|

Protein Coverage:

Supporting Peptides:

| Peptide | Uniq | -10lgP | Mass | ppm | m/z | z | RT | Scan | #Spec | Start | End | PTM |
| --- | --- | --- | --- | --- | --- | --- | --- | --- | --- | --- | --- | --- |
| K.LEKTIDDLEDELY.A | Y | 42.92 | 1594.7614 | -1.6 | 798.3867 | 2 | 13.33 | 2012 | 1 | 249 | 261 |  |
| K.LEKTIDDLEDELYAQ.K | Y | 35.49 | 1793.8571 | -6.3 | 897.9302 | 2 | 13.28 | 2004 | 1 | 249 | 263 |  |
| K.LEKTIDDLEDE.L | Y | 19.62 | 1318.6140 | -0.6 | 660.3139 | 2 | 11.52 | 1693 | 1 | 249 | 259 |  |
| K.LKGTEDE.L | Y | 17.70 | 790.3708 | -0.3 | 396.1926 | 2 | 8.61 | 1214 | 1 | 50 | 56 |  |
| total 4 peptides |  |  |  |  |  |  |  |  |  |  |  |  |
| --- | --- | --- | --- | --- | --- | --- | --- | --- | --- | --- | --- | --- |

tr|A0A6P7MR93|A0A6P7MR93\_BETSP

back to list

  

| Protein Coverage
| Supporting Peptides
|

Protein Coverage:

Supporting Peptides:

| Peptide | Uniq | -10lgP | Mass | ppm | m/z | z | RT | Scan | #Spec | Start | End | PTM |
| --- | --- | --- | --- | --- | --- | --- | --- | --- | --- | --- | --- | --- |
| K.LEKTIDDLEDELY.A | Y | 42.92 | 1594.7614 | -1.6 | 798.3867 | 2 | 13.33 | 2012 | 1 | 249 | 261 |  |
| K.LEKTIDDLEDELYAQ.K | Y | 35.49 | 1793.8571 | -6.3 | 897.9302 | 2 | 13.28 | 2004 | 1 | 249 | 263 |  |
| K.LEKTIDDLEDE.L | Y | 19.62 | 1318.6140 | -0.6 | 660.3139 | 2 | 11.52 | 1693 | 1 | 249 | 259 |  |
| K.LKGTEDE.L | Y | 17.70 | 790.3708 | -0.3 | 396.1926 | 2 | 8.61 | 1214 | 1 | 50 | 56 |  |
| total 4 peptides |  |  |  |  |  |  |  |  |  |  |  |  |
| --- | --- | --- | --- | --- | --- | --- | --- | --- | --- | --- | --- | --- |

tr|A0A3P9D7I0|A0A3P9D7I0\_9CICH

back to list

  

| Protein Coverage
| Supporting Peptides
|

Protein Coverage:

Supporting Peptides:

| Peptide | Uniq | -10lgP | Mass | ppm | m/z | z | RT | Scan | #Spec | Start | End | PTM |
| --- | --- | --- | --- | --- | --- | --- | --- | --- | --- | --- | --- | --- |
| K.LEKTIDDLEDELY.A | Y | 42.92 | 1594.7614 | -1.6 | 798.3867 | 2 | 13.33 | 2012 | 1 | 249 | 261 |  |
| K.LEKTIDDLEDELYAQ.K | Y | 35.49 | 1793.8571 | -6.3 | 897.9302 | 2 | 13.28 | 2004 | 1 | 249 | 263 |  |
| K.LEKTIDDLEDE.L | Y | 19.62 | 1318.6140 | -0.6 | 660.3139 | 2 | 11.52 | 1693 | 1 | 249 | 259 |  |
| K.LKGTEDE.L | Y | 17.70 | 790.3708 | -0.3 | 396.1926 | 2 | 8.61 | 1214 | 1 | 50 | 56 |  |
| total 4 peptides |  |  |  |  |  |  |  |  |  |  |  |  |
| --- | --- | --- | --- | --- | --- | --- | --- | --- | --- | --- | --- | --- |

tr|A0A665VR95|A0A665VR95\_ECHNA

back to list

  

| Protein Coverage
| Supporting Peptides
|

Protein Coverage:

Supporting Peptides:

| Peptide | Uniq | -10lgP | Mass | ppm | m/z | z | RT | Scan | #Spec | Start | End | PTM |
| --- | --- | --- | --- | --- | --- | --- | --- | --- | --- | --- | --- | --- |
| K.LEKTIDDLEDELY.A | Y | 42.92 | 1594.7614 | -1.6 | 798.3867 | 2 | 13.33 | 2012 | 1 | 249 | 261 |  |
| K.LEKTIDDLEDELYAQ.K | Y | 35.49 | 1793.8571 | -6.3 | 897.9302 | 2 | 13.28 | 2004 | 1 | 249 | 263 |  |
| K.LEKTIDDLEDE.L | Y | 19.62 | 1318.6140 | -0.6 | 660.3139 | 2 | 11.52 | 1693 | 1 | 249 | 259 |  |
| K.LKGTEDE.L | Y | 17.70 | 790.3708 | -0.3 | 396.1926 | 2 | 8.61 | 1214 | 1 | 50 | 56 |  |
| total 4 peptides |  |  |  |  |  |  |  |  |  |  |  |  |
| --- | --- | --- | --- | --- | --- | --- | --- | --- | --- | --- | --- | --- |

tr|A0A3P8SLC6|A0A3P8SLC6\_AMPPE

back to list

  

| Protein Coverage
| Supporting Peptides
|

Protein Coverage:

Supporting Peptides:

| Peptide | Uniq | -10lgP | Mass | ppm | m/z | z | RT | Scan | #Spec | Start | End | PTM |
| --- | --- | --- | --- | --- | --- | --- | --- | --- | --- | --- | --- | --- |
| K.LEKTIDDLEDELY.A | Y | 42.92 | 1594.7614 | -1.6 | 798.3867 | 2 | 13.33 | 2012 | 1 | 249 | 261 |  |
| K.LEKTIDDLEDELYAQ.K | Y | 35.49 | 1793.8571 | -6.3 | 897.9302 | 2 | 13.28 | 2004 | 1 | 249 | 263 |  |
| K.LEKTIDDLEDE.L | Y | 19.62 | 1318.6140 | -0.6 | 660.3139 | 2 | 11.52 | 1693 | 1 | 249 | 259 |  |
| K.LKGTEDE.L | Y | 17.70 | 790.3708 | -0.3 | 396.1926 | 2 | 8.61 | 1214 | 1 | 50 | 56 |  |
| total 4 peptides |  |  |  |  |  |  |  |  |  |  |  |  |
| --- | --- | --- | --- | --- | --- | --- | --- | --- | --- | --- | --- | --- |

tr|A0A668UDB6|A0A668UDB6\_OREAU

back to list

  

| Protein Coverage
| Supporting Peptides
|

Protein Coverage:

Supporting Peptides:

| Peptide | Uniq | -10lgP | Mass | ppm | m/z | z | RT | Scan | #Spec | Start | End | PTM |
| --- | --- | --- | --- | --- | --- | --- | --- | --- | --- | --- | --- | --- |
| K.LEKTIDDLEDELY.A | Y | 42.92 | 1594.7614 | -1.6 | 798.3867 | 2 | 13.33 | 2012 | 1 | 300 | 312 |  |
| K.LEKTIDDLEDELYAQ.K | Y | 35.49 | 1793.8571 | -6.3 | 897.9302 | 2 | 13.28 | 2004 | 1 | 300 | 314 |  |
| K.LEKTIDDLEDE.L | Y | 19.62 | 1318.6140 | -0.6 | 660.3139 | 2 | 11.52 | 1693 | 1 | 300 | 310 |  |
| K.LKGTEDE.L | Y | 17.70 | 790.3708 | -0.3 | 396.1926 | 2 | 8.61 | 1214 | 1 | 101 | 107 |  |
| total 4 peptides |  |  |  |  |  |  |  |  |  |  |  |  |
| --- | --- | --- | --- | --- | --- | --- | --- | --- | --- | --- | --- | --- |

tr|X2J4M2|X2J4M2\_PAROL

back to list

  

| Protein Coverage
| Supporting Peptides
|

Protein Coverage:

Supporting Peptides:

| Peptide | Uniq | -10lgP | Mass | ppm | m/z | z | RT | Scan | #Spec | Start | End | PTM |
| --- | --- | --- | --- | --- | --- | --- | --- | --- | --- | --- | --- | --- |
| K.LEKTIDDLEDELY.A | Y | 42.92 | 1594.7614 | -1.6 | 798.3867 | 2 | 13.33 | 2012 | 1 | 249 | 261 |  |
| K.LEKTIDDLEDELYAQ.K | Y | 35.49 | 1793.8571 | -6.3 | 897.9302 | 2 | 13.28 | 2004 | 1 | 249 | 263 |  |
| K.LEKTIDDLEDE.L | Y | 19.62 | 1318.6140 | -0.6 | 660.3139 | 2 | 11.52 | 1693 | 1 | 249 | 259 |  |
| K.LKGTEDE.L | Y | 17.70 | 790.3708 | -0.3 | 396.1926 | 2 | 8.61 | 1214 | 1 | 50 | 56 |  |
| total 4 peptides |  |  |  |  |  |  |  |  |  |  |  |  |
| --- | --- | --- | --- | --- | --- | --- | --- | --- | --- | --- | --- | --- |

tr|H2SQV9|H2SQV9\_TAKRU

back to list

  

| Protein Coverage
| Supporting Peptides
|

Protein Coverage:

Supporting Peptides:

| Peptide | Uniq | -10lgP | Mass | ppm | m/z | z | RT | Scan | #Spec | Start | End | PTM |
| --- | --- | --- | --- | --- | --- | --- | --- | --- | --- | --- | --- | --- |
| K.LEKTIDDLEDELY.A | Y | 42.92 | 1594.7614 | -1.6 | 798.3867 | 2 | 13.33 | 2012 | 1 | 236 | 248 |  |
| K.LEKTIDDLEDELYAQ.K | Y | 35.49 | 1793.8571 | -6.3 | 897.9302 | 2 | 13.28 | 2004 | 1 | 236 | 250 |  |
| K.LEKTIDDLEDE.L | Y | 19.62 | 1318.6140 | -0.6 | 660.3139 | 2 | 11.52 | 1693 | 1 | 236 | 246 |  |
| K.LKGTEDE.L | Y | 17.70 | 790.3708 | -0.3 | 396.1926 | 2 | 8.61 | 1214 | 1 | 50 | 56 |  |
| total 4 peptides |  |  |  |  |  |  |  |  |  |  |  |  |
| --- | --- | --- | --- | --- | --- | --- | --- | --- | --- | --- | --- | --- |

tr|A0A4U5UG20|A0A4U5UG20\_COLLU

back to list

  

| Protein Coverage
| Supporting Peptides
|

Protein Coverage:

Supporting Peptides:

| Peptide | Uniq | -10lgP | Mass | ppm | m/z | z | RT | Scan | #Spec | Start | End | PTM |
| --- | --- | --- | --- | --- | --- | --- | --- | --- | --- | --- | --- | --- |
| K.LEKTIDDLEDELY.A | Y | 42.92 | 1594.7614 | -1.6 | 798.3867 | 2 | 13.33 | 2012 | 1 | 304 | 316 |  |
| K.LEKTIDDLEDELYAQ.K | Y | 35.49 | 1793.8571 | -6.3 | 897.9302 | 2 | 13.28 | 2004 | 1 | 304 | 318 |  |
| K.LEKTIDDLEDE.L | Y | 19.62 | 1318.6140 | -0.6 | 660.3139 | 2 | 11.52 | 1693 | 1 | 304 | 314 |  |
| K.LKGTEDE.L | Y | 17.70 | 790.3708 | -0.3 | 396.1926 | 2 | 8.61 | 1214 | 1 | 92 | 98 |  |
| total 4 peptides |  |  |  |  |  |  |  |  |  |  |  |  |
| --- | --- | --- | --- | --- | --- | --- | --- | --- | --- | --- | --- | --- |

tr|A0A6P3VQ87|A0A6P3VQ87\_CLUHA

back to list

  

| Protein Coverage
| Supporting Peptides
|

Protein Coverage:

Supporting Peptides:

| Peptide | Uniq | -10lgP | Mass | ppm | m/z | z | RT | Scan | #Spec | Start | End | PTM |
| --- | --- | --- | --- | --- | --- | --- | --- | --- | --- | --- | --- | --- |
| K.LEKTIDDLEDELY.A | Y | 42.92 | 1594.7614 | -1.6 | 798.3867 | 2 | 13.33 | 2012 | 1 | 249 | 261 |  |
| K.LEKTIDDLEDELYAQ.K | Y | 35.49 | 1793.8571 | -6.3 | 897.9302 | 2 | 13.28 | 2004 | 1 | 249 | 263 |  |
| K.LEKTIDDLEDE.L | Y | 19.62 | 1318.6140 | -0.6 | 660.3139 | 2 | 11.52 | 1693 | 1 | 249 | 259 |  |
| K.LKGTEDE.L | Y | 17.70 | 790.3708 | -0.3 | 396.1926 | 2 | 8.61 | 1214 | 1 | 50 | 56 |  |
| total 4 peptides |  |  |  |  |  |  |  |  |  |  |  |  |
| --- | --- | --- | --- | --- | --- | --- | --- | --- | --- | --- | --- | --- |

tr|A0A4W4GLX2|A0A4W4GLX2\_ELEEL

back to list

  

| Protein Coverage
| Supporting Peptides
|

Protein Coverage:

Supporting Peptides:

| Peptide | Uniq | -10lgP | Mass | ppm | m/z | z | RT | Scan | #Spec | Start | End | PTM |
| --- | --- | --- | --- | --- | --- | --- | --- | --- | --- | --- | --- | --- |
| K.LEKTIDDLEDELY.A | Y | 42.92 | 1594.7614 | -1.6 | 798.3867 | 2 | 13.33 | 2012 | 1 | 244 | 256 |  |
| K.LEKTIDDLEDELYAQ.K | Y | 35.49 | 1793.8571 | -6.3 | 897.9302 | 2 | 13.28 | 2004 | 1 | 244 | 258 |  |
| K.LEKTIDDLEDE.L | Y | 19.62 | 1318.6140 | -0.6 | 660.3139 | 2 | 11.52 | 1693 | 1 | 244 | 254 |  |
| K.LKGTEDE.L | Y | 17.70 | 790.3708 | -0.3 | 396.1926 | 2 | 8.61 | 1214 | 1 | 50 | 56 |  |
| total 4 peptides |  |  |  |  |  |  |  |  |  |  |  |  |
| --- | --- | --- | --- | --- | --- | --- | --- | --- | --- | --- | --- | --- |

tr|A0A2I4D9D9|A0A2I4D9D9\_9TELE

back to list

  

| Protein Coverage
| Supporting Peptides
|

Protein Coverage:

Supporting Peptides:

| Peptide | Uniq | -10lgP | Mass | ppm | m/z | z | RT | Scan | #Spec | Start | End | PTM |
| --- | --- | --- | --- | --- | --- | --- | --- | --- | --- | --- | --- | --- |
| M.GPRGPPGPPGSSGPQG.P | N | 37.56 | 1400.6796 | -0.2 | 701.3469 | 2 | 9.77 | 1397 | 1 | 169 | 184 |  |
| R.GERGFPGLPGPS.G | N | 28.43 | 1169.5829 | -0.8 | 585.7983 | 2 | 12.09 | 1795 | 1 | 919 | 930 |  |
| G.FAGPPGADGQPGAK.G | N | 22.00 | 1268.6149 | -0.4 | 635.3145 | 2 | 10.23 | 1471 | 1 | 764 | 777 |  |
| S.GDRGETGSAGPA.G | Y | 17.49 | 1073.4738 | -0.3 | 537.7440 | 2 | 8.85 | 1256 | 1 | 1015 | 1026 |  |
| total 4 peptides |  |  |  |  |  |  |  |  |  |  |  |  |
| --- | --- | --- | --- | --- | --- | --- | --- | --- | --- | --- | --- | --- |

tr|A0A2I4D9D6|A0A2I4D9D6\_9TELE

back to list

  

| Protein Coverage
| Supporting Peptides
|

Protein Coverage:

Supporting Peptides:

| Peptide | Uniq | -10lgP | Mass | ppm | m/z | z | RT | Scan | #Spec | Start | End | PTM |
| --- | --- | --- | --- | --- | --- | --- | --- | --- | --- | --- | --- | --- |
| M.GPRGPPGPPGSSGPQG.P | N | 37.56 | 1400.6796 | -0.2 | 701.3469 | 2 | 9.77 | 1397 | 1 | 169 | 184 |  |
| R.GERGFPGLPGPS.G | N | 28.43 | 1169.5829 | -0.8 | 585.7983 | 2 | 12.09 | 1795 | 1 | 952 | 963 |  |
| G.FAGPPGADGQPGAK.G | N | 22.00 | 1268.6149 | -0.4 | 635.3145 | 2 | 10.23 | 1471 | 1 | 797 | 810 |  |
| S.GDRGETGSAGPA.G | Y | 17.49 | 1073.4738 | -0.3 | 537.7440 | 2 | 8.85 | 1256 | 1 | 1048 | 1059 |  |
| total 4 peptides |  |  |  |  |  |  |  |  |  |  |  |  |
| --- | --- | --- | --- | --- | --- | --- | --- | --- | --- | --- | --- | --- |

tr|A0A3P8WWA2|A0A3P8WWA2\_CYNSE

back to list

  

| Protein Coverage
| Supporting Peptides
|

Protein Coverage:

Supporting Peptides:

| Peptide | Uniq | -10lgP | Mass | ppm | m/z | z | RT | Scan | #Spec | Start | End | PTM |
| --- | --- | --- | --- | --- | --- | --- | --- | --- | --- | --- | --- | --- |
| A.GFAGDDAPRA.L | N | 39.52 | 975.4410 | -0.2 | 488.7277 | 2 | 10.22 | 1469 | 4 | 22 | 31 |  |
| Y.VGDEAQSK.R | N | 26.03 | 832.3926 | 0.0 | 417.2036 | 2 | 8.09 | 1115 | 1 | 56 | 63 |  |
| G.FAGDDAPRA.L | N | 18.34 | 918.4195 | 0.1 | 460.2171 | 2 | 17.21 | 2654 | 7 | 23 | 31 |  |
| G.FAGDDAPRAL.F | Y | 17.96 | 1031.5035 | -0.9 | 516.7586 | 2 | 11.22 | 1639 | 1 | 23 | 32 |  |
| total 4 peptides |  |  |  |  |  |  |  |  |  |  |  |  |
| --- | --- | --- | --- | --- | --- | --- | --- | --- | --- | --- | --- | --- |

tr|A0A3P8X278|A0A3P8X278\_CYNSE

back to list

  

| Protein Coverage
| Supporting Peptides
|

Protein Coverage:

Supporting Peptides:

| Peptide | Uniq | -10lgP | Mass | ppm | m/z | z | RT | Scan | #Spec | Start | End | PTM |
| --- | --- | --- | --- | --- | --- | --- | --- | --- | --- | --- | --- | --- |
| A.GFAGDDAPRA.L | N | 39.52 | 975.4410 | -0.2 | 488.7277 | 2 | 10.22 | 1469 | 4 | 19 | 28 |  |
| Y.VGDEAQSK.R | N | 26.03 | 832.3926 | 0.0 | 417.2036 | 2 | 8.09 | 1115 | 1 | 53 | 60 |  |
| G.FAGDDAPRA.L | N | 18.34 | 918.4195 | 0.1 | 460.2171 | 2 | 17.21 | 2654 | 7 | 20 | 28 |  |
| G.FAGDDAPRAL.F | Y | 17.96 | 1031.5035 | -0.9 | 516.7586 | 2 | 11.22 | 1639 | 1 | 20 | 29 |  |
| total 4 peptides |  |  |  |  |  |  |  |  |  |  |  |  |
| --- | --- | --- | --- | --- | --- | --- | --- | --- | --- | --- | --- | --- |

tr|A0A3P8X031|A0A3P8X031\_CYNSE

back to list

  

| Protein Coverage
| Supporting Peptides
|

Protein Coverage:

Supporting Peptides:

| Peptide | Uniq | -10lgP | Mass | ppm | m/z | z | RT | Scan | #Spec | Start | End | PTM |
| --- | --- | --- | --- | --- | --- | --- | --- | --- | --- | --- | --- | --- |
| A.GFAGDDAPRA.L | N | 39.52 | 975.4410 | -0.2 | 488.7277 | 2 | 10.22 | 1469 | 4 | 22 | 31 |  |
| Y.VGDEAQSK.R | N | 26.03 | 832.3926 | 0.0 | 417.2036 | 2 | 8.09 | 1115 | 1 | 56 | 63 |  |
| G.FAGDDAPRA.L | N | 18.34 | 918.4195 | 0.1 | 460.2171 | 2 | 17.21 | 2654 | 7 | 23 | 31 |  |
| G.FAGDDAPRAL.F | Y | 17.96 | 1031.5035 | -0.9 | 516.7586 | 2 | 11.22 | 1639 | 1 | 23 | 32 |  |
| total 4 peptides |  |  |  |  |  |  |  |  |  |  |  |  |
| --- | --- | --- | --- | --- | --- | --- | --- | --- | --- | --- | --- | --- |

tr|A0A3P8WTX4|A0A3P8WTX4\_CYNSE

back to list

  

| Protein Coverage
| Supporting Peptides
|

Protein Coverage:

Supporting Peptides:

| Peptide | Uniq | -10lgP | Mass | ppm | m/z | z | RT | Scan | #Spec | Start | End | PTM |
| --- | --- | --- | --- | --- | --- | --- | --- | --- | --- | --- | --- | --- |
| A.GFAGDDAPRA.L | N | 39.52 | 975.4410 | -0.2 | 488.7277 | 2 | 10.22 | 1469 | 4 | 22 | 31 |  |
| Y.VGDEAQSK.R | N | 26.03 | 832.3926 | 0.0 | 417.2036 | 2 | 8.09 | 1115 | 1 | 56 | 63 |  |
| G.FAGDDAPRA.L | N | 18.34 | 918.4195 | 0.1 | 460.2171 | 2 | 17.21 | 2654 | 7 | 23 | 31 |  |
| G.FAGDDAPRAL.F | Y | 17.96 | 1031.5035 | -0.9 | 516.7586 | 2 | 11.22 | 1639 | 1 | 23 | 32 |  |
| total 4 peptides |  |  |  |  |  |  |  |  |  |  |  |  |
| --- | --- | --- | --- | --- | --- | --- | --- | --- | --- | --- | --- | --- |

tr|A0A3P8WU36|A0A3P8WU36\_CYNSE

back to list

  

| Protein Coverage
| Supporting Peptides
|

Protein Coverage:

Supporting Peptides:

| Peptide | Uniq | -10lgP | Mass | ppm | m/z | z | RT | Scan | #Spec | Start | End | PTM |
| --- | --- | --- | --- | --- | --- | --- | --- | --- | --- | --- | --- | --- |
| A.GFAGDDAPRA.L | N | 39.52 | 975.4410 | -0.2 | 488.7277 | 2 | 10.22 | 1469 | 4 | 19 | 28 |  |
| Y.VGDEAQSK.R | N | 26.03 | 832.3926 | 0.0 | 417.2036 | 2 | 8.09 | 1115 | 1 | 53 | 60 |  |
| G.FAGDDAPRA.L | N | 18.34 | 918.4195 | 0.1 | 460.2171 | 2 | 17.21 | 2654 | 7 | 20 | 28 |  |
| G.FAGDDAPRAL.F | Y | 17.96 | 1031.5035 | -0.9 | 516.7586 | 2 | 11.22 | 1639 | 1 | 20 | 29 |  |
| total 4 peptides |  |  |  |  |  |  |  |  |  |  |  |  |
| --- | --- | --- | --- | --- | --- | --- | --- | --- | --- | --- | --- | --- |

tr|A0A3P8WUR0|A0A3P8WUR0\_CYNSE

back to list

  

| Protein Coverage
| Supporting Peptides
|

Protein Coverage:

Supporting Peptides:

| Peptide | Uniq | -10lgP | Mass | ppm | m/z | z | RT | Scan | #Spec | Start | End | PTM |
| --- | --- | --- | --- | --- | --- | --- | --- | --- | --- | --- | --- | --- |
| A.GFAGDDAPRA.L | N | 39.52 | 975.4410 | -0.2 | 488.7277 | 2 | 10.22 | 1469 | 4 | 22 | 31 |  |
| Y.VGDEAQSK.R | N | 26.03 | 832.3926 | 0.0 | 417.2036 | 2 | 8.09 | 1115 | 1 | 56 | 63 |  |
| G.FAGDDAPRA.L | N | 18.34 | 918.4195 | 0.1 | 460.2171 | 2 | 17.21 | 2654 | 7 | 23 | 31 |  |
| G.FAGDDAPRAL.F | Y | 17.96 | 1031.5035 | -0.9 | 516.7586 | 2 | 11.22 | 1639 | 1 | 23 | 32 |  |
| total 4 peptides |  |  |  |  |  |  |  |  |  |  |  |  |
| --- | --- | --- | --- | --- | --- | --- | --- | --- | --- | --- | --- | --- |

tr|A0A7N8WP58|A0A7N8WP58\_9TELE

back to list

  

| Protein Coverage
| Supporting Peptides
|

Protein Coverage:

Supporting Peptides:

| Peptide | Uniq | -10lgP | Mass | ppm | m/z | z | RT | Scan | #Spec | Start | End | PTM |
| --- | --- | --- | --- | --- | --- | --- | --- | --- | --- | --- | --- | --- |
| E.FDDLPIHEQ.S | Y | 35.62 | 1112.5138 | -1.1 | 557.2635 | 2 | 11.85 | 1752 | 1 | 621 | 629 |  |
| Y.TGREFDDLPIHEQ.S | Y | 24.24 | 1555.7267 | 1.3 | 778.8716 | 2 | 11.69 | 1722 | 2 | 617 | 629 |  |
| E.VSVGDKVPAD.I | Y | 23.82 | 985.5080 | -0.5 | 493.7610 | 2 | 10.19 | 1463 | 1 | 119 | 128 |  |
| L.GLPEALIPVQLL.W | Y | 16.99 | 1261.7645 | 0.0 | 631.8895 | 2 | 15.85 | 2438 | 1 | 745 | 756 |  |
| E.IVPGDIVE.V | Y | 15.52 | 840.4592 | 0.4 | 421.2371 | 2 | 12.27 | 1826 | 1 | 111 | 118 |  |
| total 5 peptides |  |  |  |  |  |  |  |  |  |  |  |  |
| --- | --- | --- | --- | --- | --- | --- | --- | --- | --- | --- | --- | --- |

tr|A0A668SKK1|A0A668SKK1\_OREAU

back to list

  

| Protein Coverage
| Supporting Peptides
|

Protein Coverage:

Supporting Peptides:

| Peptide | Uniq | -10lgP | Mass | ppm | m/z | z | RT | Scan | #Spec | Start | End | PTM |
| --- | --- | --- | --- | --- | --- | --- | --- | --- | --- | --- | --- | --- |
| E.FDDLPLHEQ.S | Y | 35.62 | 1112.5138 | -1.1 | 557.2635 | 2 | 11.85 | 1752 | 1 | 620 | 628 |  |
| Y.TGREFDDLPLHEQ.S | Y | 24.24 | 1555.7267 | 1.3 | 778.8716 | 2 | 11.69 | 1722 | 2 | 616 | 628 |  |
| E.VSVGDKVPAD.I | Y | 23.82 | 985.5080 | -0.5 | 493.7610 | 2 | 10.19 | 1463 | 1 | 118 | 127 |  |
| L.GLPEALIPVQLL.W | Y | 16.99 | 1261.7645 | 0.0 | 631.8895 | 2 | 15.85 | 2438 | 1 | 744 | 755 |  |
| E.IVPGDIVE.V | Y | 15.52 | 840.4592 | 0.4 | 421.2371 | 2 | 12.27 | 1826 | 1 | 110 | 117 |  |
| total 5 peptides |  |  |  |  |  |  |  |  |  |  |  |  |
| --- | --- | --- | --- | --- | --- | --- | --- | --- | --- | --- | --- | --- |

tr|A0A3Q3BHF7|A0A3Q3BHF7\_KRYMA

back to list

  

| Protein Coverage
| Supporting Peptides
|

Protein Coverage:

Supporting Peptides:

| Peptide | Uniq | -10lgP | Mass | ppm | m/z | z | RT | Scan | #Spec | Start | End | PTM |
| --- | --- | --- | --- | --- | --- | --- | --- | --- | --- | --- | --- | --- |
| E.FDDLPLHEQ.A | Y | 35.62 | 1112.5138 | -1.1 | 557.2635 | 2 | 11.85 | 1752 | 1 | 655 | 663 |  |
| Y.TGREFDDLPLHEQ.A | Y | 24.24 | 1555.7267 | 1.3 | 778.8716 | 2 | 11.69 | 1722 | 2 | 651 | 663 |  |
| E.VSVGDKVPAD.I | Y | 23.82 | 985.5080 | -0.5 | 493.7610 | 2 | 10.19 | 1463 | 1 | 153 | 162 |  |
| L.GLPEALIPVQLL.W | Y | 16.99 | 1261.7645 | 0.0 | 631.8895 | 2 | 15.85 | 2438 | 1 | 779 | 790 |  |
| E.IVPGDIVE.V | Y | 15.52 | 840.4592 | 0.4 | 421.2371 | 2 | 12.27 | 1826 | 1 | 145 | 152 |  |
| total 5 peptides |  |  |  |  |  |  |  |  |  |  |  |  |
| --- | --- | --- | --- | --- | --- | --- | --- | --- | --- | --- | --- | --- |

tr|A0A3Q2WQG2|A0A3Q2WQG2\_HAPBU

back to list

  

| Protein Coverage
| Supporting Peptides
|

Protein Coverage:

Supporting Peptides:

| Peptide | Uniq | -10lgP | Mass | ppm | m/z | z | RT | Scan | #Spec | Start | End | PTM |
| --- | --- | --- | --- | --- | --- | --- | --- | --- | --- | --- | --- | --- |
| E.FDDLPLHEQ.S | Y | 35.62 | 1112.5138 | -1.1 | 557.2635 | 2 | 11.85 | 1752 | 1 | 655 | 663 |  |
| Y.TGREFDDLPLHEQ.S | Y | 24.24 | 1555.7267 | 1.3 | 778.8716 | 2 | 11.69 | 1722 | 2 | 651 | 663 |  |
| E.VSVGDKVPAD.I | Y | 23.82 | 985.5080 | -0.5 | 493.7610 | 2 | 10.19 | 1463 | 1 | 153 | 162 |  |
| L.GLPEALIPVQLL.W | Y | 16.99 | 1261.7645 | 0.0 | 631.8895 | 2 | 15.85 | 2438 | 1 | 779 | 790 |  |
| E.IVPGDIVE.V | Y | 15.52 | 840.4592 | 0.4 | 421.2371 | 2 | 12.27 | 1826 | 1 | 145 | 152 |  |
| total 5 peptides |  |  |  |  |  |  |  |  |  |  |  |  |
| --- | --- | --- | --- | --- | --- | --- | --- | --- | --- | --- | --- | --- |

tr|A0A3B3DXK1|A0A3B3DXK1\_ORYME

back to list

  

| Protein Coverage
| Supporting Peptides
|

Protein Coverage:

Supporting Peptides:

| Peptide | Uniq | -10lgP | Mass | ppm | m/z | z | RT | Scan | #Spec | Start | End | PTM |
| --- | --- | --- | --- | --- | --- | --- | --- | --- | --- | --- | --- | --- |
| E.FDDLPLHEQ.A | Y | 35.62 | 1112.5138 | -1.1 | 557.2635 | 2 | 11.85 | 1752 | 1 | 655 | 663 |  |
| Y.TGREFDDLPLHEQ.A | Y | 24.24 | 1555.7267 | 1.3 | 778.8716 | 2 | 11.69 | 1722 | 2 | 651 | 663 |  |
| E.VSVGDKVPAD.I | Y | 23.82 | 985.5080 | -0.5 | 493.7610 | 2 | 10.19 | 1463 | 1 | 153 | 162 |  |
| L.GLPEALIPVQLL.W | Y | 16.99 | 1261.7645 | 0.0 | 631.8895 | 2 | 15.85 | 2438 | 1 | 779 | 790 |  |
| E.IVPGDIVE.V | Y | 15.52 | 840.4592 | 0.4 | 421.2371 | 2 | 12.27 | 1826 | 1 | 145 | 152 |  |
| total 5 peptides |  |  |  |  |  |  |  |  |  |  |  |  |
| --- | --- | --- | --- | --- | --- | --- | --- | --- | --- | --- | --- | --- |

tr|A0A3Q3BR63|A0A3Q3BR63\_HAPBU

back to list

  

| Protein Coverage
| Supporting Peptides
|

Protein Coverage:

Supporting Peptides:

| Peptide | Uniq | -10lgP | Mass | ppm | m/z | z | RT | Scan | #Spec | Start | End | PTM |
| --- | --- | --- | --- | --- | --- | --- | --- | --- | --- | --- | --- | --- |
| E.FDDLPLHEQ.S | Y | 35.62 | 1112.5138 | -1.1 | 557.2635 | 2 | 11.85 | 1752 | 1 | 655 | 663 |  |
| Y.TGREFDDLPLHEQ.S | Y | 24.24 | 1555.7267 | 1.3 | 778.8716 | 2 | 11.69 | 1722 | 2 | 651 | 663 |  |
| E.VSVGDKVPAD.I | Y | 23.82 | 985.5080 | -0.5 | 493.7610 | 2 | 10.19 | 1463 | 1 | 153 | 162 |  |
| L.GLPEALIPVQLL.W | Y | 16.99 | 1261.7645 | 0.0 | 631.8895 | 2 | 15.85 | 2438 | 1 | 779 | 790 |  |
| E.IVPGDIVE.V | Y | 15.52 | 840.4592 | 0.4 | 421.2371 | 2 | 12.27 | 1826 | 1 | 145 | 152 |  |
| total 5 peptides |  |  |  |  |  |  |  |  |  |  |  |  |
| --- | --- | --- | --- | --- | --- | --- | --- | --- | --- | --- | --- | --- |

tr|A0A3Q3H0F6|A0A3Q3H0F6\_KRYMA

back to list

  

| Protein Coverage
| Supporting Peptides
|

Protein Coverage:

Supporting Peptides:

| Peptide | Uniq | -10lgP | Mass | ppm | m/z | z | RT | Scan | #Spec | Start | End | PTM |
| --- | --- | --- | --- | --- | --- | --- | --- | --- | --- | --- | --- | --- |
| E.FDDLPLHEQ.A | Y | 35.62 | 1112.5138 | -1.1 | 557.2635 | 2 | 11.85 | 1752 | 1 | 655 | 663 |  |
| Y.TGREFDDLPLHEQ.A | Y | 24.24 | 1555.7267 | 1.3 | 778.8716 | 2 | 11.69 | 1722 | 2 | 651 | 663 |  |
| E.VSVGDKVPAD.I | Y | 23.82 | 985.5080 | -0.5 | 493.7610 | 2 | 10.19 | 1463 | 1 | 153 | 162 |  |
| L.GLPEALIPVQLL.W | Y | 16.99 | 1261.7645 | 0.0 | 631.8895 | 2 | 15.85 | 2438 | 1 | 779 | 790 |  |
| E.IVPGDIVE.V | Y | 15.52 | 840.4592 | 0.4 | 421.2371 | 2 | 12.27 | 1826 | 1 | 145 | 152 |  |
| total 5 peptides |  |  |  |  |  |  |  |  |  |  |  |  |
| --- | --- | --- | --- | --- | --- | --- | --- | --- | --- | --- | --- | --- |

tr|A0A3P9C9M1|A0A3P9C9M1\_9CICH

back to list

  

| Protein Coverage
| Supporting Peptides
|

Protein Coverage:

Supporting Peptides:

| Peptide | Uniq | -10lgP | Mass | ppm | m/z | z | RT | Scan | #Spec | Start | End | PTM |
| --- | --- | --- | --- | --- | --- | --- | --- | --- | --- | --- | --- | --- |
| E.FDDLPLHEQ.S | Y | 35.62 | 1112.5138 | -1.1 | 557.2635 | 2 | 11.85 | 1752 | 1 | 655 | 663 |  |
| Y.TGREFDDLPLHEQ.S | Y | 24.24 | 1555.7267 | 1.3 | 778.8716 | 2 | 11.69 | 1722 | 2 | 651 | 663 |  |
| E.VSVGDKVPAD.I | Y | 23.82 | 985.5080 | -0.5 | 493.7610 | 2 | 10.19 | 1463 | 1 | 153 | 162 |  |
| L.GLPEALIPVQLL.W | Y | 16.99 | 1261.7645 | 0.0 | 631.8895 | 2 | 15.85 | 2438 | 1 | 779 | 790 |  |
| E.IVPGDIVE.V | Y | 15.52 | 840.4592 | 0.4 | 421.2371 | 2 | 12.27 | 1826 | 1 | 145 | 152 |  |
| total 5 peptides |  |  |  |  |  |  |  |  |  |  |  |  |
| --- | --- | --- | --- | --- | --- | --- | --- | --- | --- | --- | --- | --- |

tr|A0A668SKH5|A0A668SKH5\_OREAU

back to list

  

| Protein Coverage
| Supporting Peptides
|

Protein Coverage:

Supporting Peptides:

| Peptide | Uniq | -10lgP | Mass | ppm | m/z | z | RT | Scan | #Spec | Start | End | PTM |
| --- | --- | --- | --- | --- | --- | --- | --- | --- | --- | --- | --- | --- |
| E.FDDLPLHEQ.S | Y | 35.62 | 1112.5138 | -1.1 | 557.2635 | 2 | 11.85 | 1752 | 1 | 655 | 663 |  |
| Y.TGREFDDLPLHEQ.S | Y | 24.24 | 1555.7267 | 1.3 | 778.8716 | 2 | 11.69 | 1722 | 2 | 651 | 663 |  |
| E.VSVGDKVPAD.I | Y | 23.82 | 985.5080 | -0.5 | 493.7610 | 2 | 10.19 | 1463 | 1 | 153 | 162 |  |
| L.GLPEALIPVQLL.W | Y | 16.99 | 1261.7645 | 0.0 | 631.8895 | 2 | 15.85 | 2438 | 1 | 779 | 790 |  |
| E.IVPGDIVE.V | Y | 15.52 | 840.4592 | 0.4 | 421.2371 | 2 | 12.27 | 1826 | 1 | 145 | 152 |  |
| total 5 peptides |  |  |  |  |  |  |  |  |  |  |  |  |
| --- | --- | --- | --- | --- | --- | --- | --- | --- | --- | --- | --- | --- |

tr|A0A669D951|A0A669D951\_ORENI

back to list

  

| Protein Coverage
| Supporting Peptides
|

Protein Coverage:

Supporting Peptides:

| Peptide | Uniq | -10lgP | Mass | ppm | m/z | z | RT | Scan | #Spec | Start | End | PTM |
| --- | --- | --- | --- | --- | --- | --- | --- | --- | --- | --- | --- | --- |
| E.FDDLPLHEQ.S | Y | 35.62 | 1112.5138 | -1.1 | 557.2635 | 2 | 11.85 | 1752 | 1 | 657 | 665 |  |
| Y.TGREFDDLPLHEQ.S | Y | 24.24 | 1555.7267 | 1.3 | 778.8716 | 2 | 11.69 | 1722 | 2 | 653 | 665 |  |
| E.VSVGDKVPAD.I | Y | 23.82 | 985.5080 | -0.5 | 493.7610 | 2 | 10.19 | 1463 | 1 | 153 | 162 |  |
| L.GLPEALIPVQLL.W | Y | 16.99 | 1261.7645 | 0.0 | 631.8895 | 2 | 15.85 | 2438 | 1 | 781 | 792 |  |
| E.IVPGDIVE.V | Y | 15.52 | 840.4592 | 0.4 | 421.2371 | 2 | 12.27 | 1826 | 1 | 145 | 152 |  |
| total 5 peptides |  |  |  |  |  |  |  |  |  |  |  |  |
| --- | --- | --- | --- | --- | --- | --- | --- | --- | --- | --- | --- | --- |

tr|A0A674PDV8|A0A674PDV8\_TAKRU

back to list

  

| Protein Coverage
| Supporting Peptides
|

Protein Coverage:

Supporting Peptides:

| Peptide | Uniq | -10lgP | Mass | ppm | m/z | z | RT | Scan | #Spec | Start | End | PTM |
| --- | --- | --- | --- | --- | --- | --- | --- | --- | --- | --- | --- | --- |
| E.FDDLPLHEQ.P | Y | 35.62 | 1112.5138 | -1.1 | 557.2635 | 2 | 11.85 | 1752 | 1 | 655 | 663 |  |
| Y.TGREFDDLPLHEQ.P | Y | 24.24 | 1555.7267 | 1.3 | 778.8716 | 2 | 11.69 | 1722 | 2 | 651 | 663 |  |
| E.VSVGDKVPAD.I | Y | 23.82 | 985.5080 | -0.5 | 493.7610 | 2 | 10.19 | 1463 | 1 | 153 | 162 |  |
| L.GLPEALIPVQLL.W | Y | 16.99 | 1261.7645 | 0.0 | 631.8895 | 2 | 15.85 | 2438 | 1 | 779 | 790 |  |
| E.IVPGDIVE.V | Y | 15.52 | 840.4592 | 0.4 | 421.2371 | 2 | 12.27 | 1826 | 1 | 145 | 152 |  |
| total 5 peptides |  |  |  |  |  |  |  |  |  |  |  |  |
| --- | --- | --- | --- | --- | --- | --- | --- | --- | --- | --- | --- | --- |

tr|A0A674P4B1|A0A674P4B1\_TAKRU

back to list

  

| Protein Coverage
| Supporting Peptides
|

Protein Coverage:

Supporting Peptides:

| Peptide | Uniq | -10lgP | Mass | ppm | m/z | z | RT | Scan | #Spec | Start | End | PTM |
| --- | --- | --- | --- | --- | --- | --- | --- | --- | --- | --- | --- | --- |
| E.FDDLPLHEQ.P | Y | 35.62 | 1112.5138 | -1.1 | 557.2635 | 2 | 11.85 | 1752 | 1 | 655 | 663 |  |
| Y.TGREFDDLPLHEQ.P | Y | 24.24 | 1555.7267 | 1.3 | 778.8716 | 2 | 11.69 | 1722 | 2 | 651 | 663 |  |
| E.VSVGDKVPAD.I | Y | 23.82 | 985.5080 | -0.5 | 493.7610 | 2 | 10.19 | 1463 | 1 | 153 | 162 |  |
| L.GLPEALIPVQLL.W | Y | 16.99 | 1261.7645 | 0.0 | 631.8895 | 2 | 15.85 | 2438 | 1 | 779 | 790 |  |
| E.IVPGDIVE.V | Y | 15.52 | 840.4592 | 0.4 | 421.2371 | 2 | 12.27 | 1826 | 1 | 145 | 152 |  |
| total 5 peptides |  |  |  |  |  |  |  |  |  |  |  |  |
| --- | --- | --- | --- | --- | --- | --- | --- | --- | --- | --- | --- | --- |

tr|A0A3Q1CVI2|A0A3Q1CVI2\_AMPOC

back to list

  

| Protein Coverage
| Supporting Peptides
|

Protein Coverage:

Supporting Peptides:

| Peptide | Uniq | -10lgP | Mass | ppm | m/z | z | RT | Scan | #Spec | Start | End | PTM |
| --- | --- | --- | --- | --- | --- | --- | --- | --- | --- | --- | --- | --- |
| F.GEKLKGADPEDVILS.A | Y | 39.52 | 1569.8250 | -1.0 | 785.9189 | 2 | 11.73 | 1729 | 1 | 66 | 80 |  |
| V.LDPEGTGSIK.K | Y | 24.55 | 1015.5186 | -0.5 | 508.7663 | 2 | 10.19 | 1464 | 1 | 85 | 94 |  |
| K.LKGADPEDVIL.S | Y | 19.66 | 1168.6339 | -0.5 | 585.3239 | 2 | 12.52 | 1872 | 1 | 69 | 79 |  |
| A.AFPPDVAGN.V | Y | 16.10 | 886.4185 | -1.1 | 444.2160 | 2 | 11.80 | 1741 | 1 | 120 | 128 |  |
| total 4 peptides |  |  |  |  |  |  |  |  |  |  |  |  |
| --- | --- | --- | --- | --- | --- | --- | --- | --- | --- | --- | --- | --- |

tr|A0A4U5VU95|A0A4U5VU95\_COLLU

back to list

  

| Protein Coverage
| Supporting Peptides
|

Protein Coverage:

Supporting Peptides:

| Peptide | Uniq | -10lgP | Mass | ppm | m/z | z | RT | Scan | #Spec | Start | End | PTM |
| --- | --- | --- | --- | --- | --- | --- | --- | --- | --- | --- | --- | --- |
| F.GEKLKGADPEDVILS.A | Y | 39.52 | 1569.8250 | -1.0 | 785.9189 | 2 | 11.73 | 1729 | 1 | 68 | 82 |  |
| V.LDPEGTGSIK.K | Y | 24.55 | 1015.5186 | -0.5 | 508.7663 | 2 | 10.19 | 1464 | 1 | 87 | 96 |  |
| K.LKGADPEDVIL.S | Y | 19.66 | 1168.6339 | -0.5 | 585.3239 | 2 | 12.52 | 1872 | 1 | 71 | 81 |  |
| A.AFPPDVAGN.V | Y | 16.10 | 886.4185 | -1.1 | 444.2160 | 2 | 11.80 | 1741 | 1 | 122 | 130 |  |
| total 4 peptides |  |  |  |  |  |  |  |  |  |  |  |  |
| --- | --- | --- | --- | --- | --- | --- | --- | --- | --- | --- | --- | --- |

tr|A0A6P7II88|A0A6P7II88\_9TELE

back to list

  

| Protein Coverage
| Supporting Peptides
|

Protein Coverage:

Supporting Peptides:

| Peptide | Uniq | -10lgP | Mass | ppm | m/z | z | RT | Scan | #Spec | Start | End | PTM |
| --- | --- | --- | --- | --- | --- | --- | --- | --- | --- | --- | --- | --- |
| F.GEKLKGADPEDVILS.A | Y | 39.52 | 1569.8250 | -1.0 | 785.9189 | 2 | 11.73 | 1729 | 1 | 90 | 104 |  |
| V.LDPEGTGSIK.K | Y | 24.55 | 1015.5186 | -0.5 | 508.7663 | 2 | 10.19 | 1464 | 1 | 109 | 118 |  |
| K.LKGADPEDVIL.S | Y | 19.66 | 1168.6339 | -0.5 | 585.3239 | 2 | 12.52 | 1872 | 1 | 93 | 103 |  |
| A.AFPPDVAGN.V | Y | 16.10 | 886.4185 | -1.1 | 444.2160 | 2 | 11.80 | 1741 | 1 | 144 | 152 |  |
| total 4 peptides |  |  |  |  |  |  |  |  |  |  |  |  |
| --- | --- | --- | --- | --- | --- | --- | --- | --- | --- | --- | --- | --- |

tr|A0A3Q4GCJ7|A0A3Q4GCJ7\_NEOBR

back to list

  

| Protein Coverage
| Supporting Peptides
|

Protein Coverage:

Supporting Peptides:

| Peptide | Uniq | -10lgP | Mass | ppm | m/z | z | RT | Scan | #Spec | Start | End | PTM |
| --- | --- | --- | --- | --- | --- | --- | --- | --- | --- | --- | --- | --- |
| F.GEKLKGADPEDVILS.A | Y | 39.52 | 1569.8250 | -1.0 | 785.9189 | 2 | 11.73 | 1729 | 1 | 90 | 104 |  |
| V.LDPEGTGSIK.K | Y | 24.55 | 1015.5186 | -0.5 | 508.7663 | 2 | 10.19 | 1464 | 1 | 109 | 118 |  |
| K.LKGADPEDVIL.S | Y | 19.66 | 1168.6339 | -0.5 | 585.3239 | 2 | 12.52 | 1872 | 1 | 93 | 103 |  |
| A.AFPPDVAGN.V | Y | 16.10 | 886.4185 | -1.1 | 444.2160 | 2 | 11.80 | 1741 | 1 | 144 | 152 |  |
| total 4 peptides |  |  |  |  |  |  |  |  |  |  |  |  |
| --- | --- | --- | --- | --- | --- | --- | --- | --- | --- | --- | --- | --- |

tr|A0A3P9IWY3|A0A3P9IWY3\_ORYLA

back to list

  

| Protein Coverage
| Supporting Peptides
|

Protein Coverage:

Supporting Peptides:

| Peptide | Uniq | -10lgP | Mass | ppm | m/z | z | RT | Scan | #Spec | Start | End | PTM |
| --- | --- | --- | --- | --- | --- | --- | --- | --- | --- | --- | --- | --- |
| F.GEKLKGADPEDVILS.A | Y | 39.52 | 1569.8250 | -1.0 | 785.9189 | 2 | 11.73 | 1729 | 1 | 90 | 104 |  |
| V.LDPEGTGSIK.K | Y | 24.55 | 1015.5186 | -0.5 | 508.7663 | 2 | 10.19 | 1464 | 1 | 109 | 118 |  |
| K.LKGADPEDVIL.S | Y | 19.66 | 1168.6339 | -0.5 | 585.3239 | 2 | 12.52 | 1872 | 1 | 93 | 103 |  |
| A.AFPPDVAGN.V | Y | 16.10 | 886.4185 | -1.1 | 444.2160 | 2 | 11.80 | 1741 | 1 | 144 | 152 |  |
| total 4 peptides |  |  |  |  |  |  |  |  |  |  |  |  |
| --- | --- | --- | --- | --- | --- | --- | --- | --- | --- | --- | --- | --- |

tr|H3C4G7|H3C4G7\_TETNG

back to list

  

| Protein Coverage
| Supporting Peptides
|

Protein Coverage:

Supporting Peptides:

| Peptide | Uniq | -10lgP | Mass | ppm | m/z | z | RT | Scan | #Spec | Start | End | PTM |
| --- | --- | --- | --- | --- | --- | --- | --- | --- | --- | --- | --- | --- |
| F.GEKLKGADPEDVILS.A | Y | 39.52 | 1569.8250 | -1.0 | 785.9189 | 2 | 11.73 | 1729 | 1 | 90 | 104 |  |
| V.LDPEGTGSIK.K | Y | 24.55 | 1015.5186 | -0.5 | 508.7663 | 2 | 10.19 | 1464 | 1 | 109 | 118 |  |
| K.LKGADPEDVIL.S | Y | 19.66 | 1168.6339 | -0.5 | 585.3239 | 2 | 12.52 | 1872 | 1 | 93 | 103 |  |
| A.AFPPDVAGN.V | Y | 16.10 | 886.4185 | -1.1 | 444.2160 | 2 | 11.80 | 1741 | 1 | 144 | 152 |  |
| total 4 peptides |  |  |  |  |  |  |  |  |  |  |  |  |
| --- | --- | --- | --- | --- | --- | --- | --- | --- | --- | --- | --- | --- |

tr|A0A3B4VIC8|A0A3B4VIC8\_SERDU

back to list

  

| Protein Coverage
| Supporting Peptides
|

Protein Coverage:

Supporting Peptides:

| Peptide | Uniq | -10lgP | Mass | ppm | m/z | z | RT | Scan | #Spec | Start | End | PTM |
| --- | --- | --- | --- | --- | --- | --- | --- | --- | --- | --- | --- | --- |
| F.GEKLKGADPEDVILS.S | Y | 39.52 | 1569.8250 | -1.0 | 785.9189 | 2 | 11.73 | 1729 | 1 | 90 | 104 |  |
| V.LDPEGTGSIK.K | Y | 24.55 | 1015.5186 | -0.5 | 508.7663 | 2 | 10.19 | 1464 | 1 | 109 | 118 |  |
| K.LKGADPEDVIL.S | Y | 19.66 | 1168.6339 | -0.5 | 585.3239 | 2 | 12.52 | 1872 | 1 | 93 | 103 |  |
| A.AFPPDVAGN.V | Y | 16.10 | 886.4185 | -1.1 | 444.2160 | 2 | 11.80 | 1741 | 1 | 144 | 152 |  |
| total 4 peptides |  |  |  |  |  |  |  |  |  |  |  |  |
| --- | --- | --- | --- | --- | --- | --- | --- | --- | --- | --- | --- | --- |

tr|I6U9W3|I6U9W3\_ORYJA

back to list

  

| Protein Coverage
| Supporting Peptides
|

Protein Coverage:

Supporting Peptides:

| Peptide | Uniq | -10lgP | Mass | ppm | m/z | z | RT | Scan | #Spec | Start | End | PTM |
| --- | --- | --- | --- | --- | --- | --- | --- | --- | --- | --- | --- | --- |
| F.GEKLKGADPEDVILS.A | Y | 39.52 | 1569.8250 | -1.0 | 785.9189 | 2 | 11.73 | 1729 | 1 | 90 | 104 |  |
| V.LDPEGTGSIK.K | Y | 24.55 | 1015.5186 | -0.5 | 508.7663 | 2 | 10.19 | 1464 | 1 | 109 | 118 |  |
| K.LKGADPEDVIL.S | Y | 19.66 | 1168.6339 | -0.5 | 585.3239 | 2 | 12.52 | 1872 | 1 | 93 | 103 |  |
| S.AFPPDVAGN.V | Y | 16.10 | 886.4185 | -1.1 | 444.2160 | 2 | 11.80 | 1741 | 1 | 144 | 152 |  |
| total 4 peptides |  |  |  |  |  |  |  |  |  |  |  |  |
| --- | --- | --- | --- | --- | --- | --- | --- | --- | --- | --- | --- | --- |

tr|A0A3P9KRI4|A0A3P9KRI4\_ORYLA

back to list

  

| Protein Coverage
| Supporting Peptides
|

Protein Coverage:

Supporting Peptides:

| Peptide | Uniq | -10lgP | Mass | ppm | m/z | z | RT | Scan | #Spec | Start | End | PTM |
| --- | --- | --- | --- | --- | --- | --- | --- | --- | --- | --- | --- | --- |
| F.GEKLKGADPEDVILS.A | Y | 39.52 | 1569.8250 | -1.0 | 785.9189 | 2 | 11.73 | 1729 | 1 | 112 | 126 |  |
| V.LDPEGTGSIK.K | Y | 24.55 | 1015.5186 | -0.5 | 508.7663 | 2 | 10.19 | 1464 | 1 | 131 | 140 |  |
| K.LKGADPEDVIL.S | Y | 19.66 | 1168.6339 | -0.5 | 585.3239 | 2 | 12.52 | 1872 | 1 | 115 | 125 |  |
| A.AFPPDVAGN.V | Y | 16.10 | 886.4185 | -1.1 | 444.2160 | 2 | 11.80 | 1741 | 1 | 166 | 174 |  |
| total 4 peptides |  |  |  |  |  |  |  |  |  |  |  |  |
| --- | --- | --- | --- | --- | --- | --- | --- | --- | --- | --- | --- | --- |

tr|A0A3B3HA26|A0A3B3HA26\_ORYLA

back to list

  

| Protein Coverage
| Supporting Peptides
|

Protein Coverage:

Supporting Peptides:

| Peptide | Uniq | -10lgP | Mass | ppm | m/z | z | RT | Scan | #Spec | Start | End | PTM |
| --- | --- | --- | --- | --- | --- | --- | --- | --- | --- | --- | --- | --- |
| F.GEKLKGADPEDVILS.A | Y | 39.52 | 1569.8250 | -1.0 | 785.9189 | 2 | 11.73 | 1729 | 1 | 177 | 191 |  |
| V.LDPEGTGSIK.K | Y | 24.55 | 1015.5186 | -0.5 | 508.7663 | 2 | 10.19 | 1464 | 1 | 196 | 205 |  |
| K.LKGADPEDVIL.S | Y | 19.66 | 1168.6339 | -0.5 | 585.3239 | 2 | 12.52 | 1872 | 1 | 180 | 190 |  |
| A.AFPPDVAGN.V | Y | 16.10 | 886.4185 | -1.1 | 444.2160 | 2 | 11.80 | 1741 | 1 | 231 | 239 |  |
| total 4 peptides |  |  |  |  |  |  |  |  |  |  |  |  |
| --- | --- | --- | --- | --- | --- | --- | --- | --- | --- | --- | --- | --- |

tr|A0A7N8YD85|A0A7N8YD85\_9TELE

back to list

  

| Protein Coverage
| Supporting Peptides
|

Protein Coverage:

Supporting Peptides:

| Peptide | Uniq | -10lgP | Mass | ppm | m/z | z | RT | Scan | #Spec | Start | End | PTM |
| --- | --- | --- | --- | --- | --- | --- | --- | --- | --- | --- | --- | --- |
| F.IFDVLEDPVEILDNSRE.L | N | 44.53 | 2001.9894 | -0.9 | 1002.0010 | 2 | 14.67 | 2236 | 1 | 142 | 158 |  |
| F.MDDPVVIPGKPY.T | N | 35.25 | 1329.6638 | -0.8 | 665.8386 | 2 | 12.28 | 1829 | 1 | 219 | 230 |  |
| total 2 peptides |  |  |  |  |  |  |  |  |  |  |  |  |
| --- | --- | --- | --- | --- | --- | --- | --- | --- | --- | --- | --- | --- |

tr|A0A7N8XIB6|A0A7N8XIB6\_9TELE

back to list

  

| Protein Coverage
| Supporting Peptides
|

Protein Coverage:

Supporting Peptides:

| Peptide | Uniq | -10lgP | Mass | ppm | m/z | z | RT | Scan | #Spec | Start | End | PTM |
| --- | --- | --- | --- | --- | --- | --- | --- | --- | --- | --- | --- | --- |
| F.IFDVLEDPVEILDNSRE.L | N | 44.53 | 2001.9894 | -0.9 | 1002.0010 | 2 | 14.67 | 2236 | 1 | 139 | 155 |  |
| F.MDDPVVIPGKPY.T | N | 35.25 | 1329.6638 | -0.8 | 665.8386 | 2 | 12.28 | 1829 | 1 | 222 | 233 |  |
| total 2 peptides |  |  |  |  |  |  |  |  |  |  |  |  |
| --- | --- | --- | --- | --- | --- | --- | --- | --- | --- | --- | --- | --- |

tr|A0A7N8Y4P6|A0A7N8Y4P6\_9TELE

back to list

  

| Protein Coverage
| Supporting Peptides
|

Protein Coverage:

Supporting Peptides:

| Peptide | Uniq | -10lgP | Mass | ppm | m/z | z | RT | Scan | #Spec | Start | End | PTM |
| --- | --- | --- | --- | --- | --- | --- | --- | --- | --- | --- | --- | --- |
| F.IFDVLEDPVEILDNSRE.L | N | 44.53 | 2001.9894 | -0.9 | 1002.0010 | 2 | 14.67 | 2236 | 1 | 139 | 155 |  |
| F.MDDPVVIPGKPY.T | N | 35.25 | 1329.6638 | -0.8 | 665.8386 | 2 | 12.28 | 1829 | 1 | 222 | 233 |  |
| total 2 peptides |  |  |  |  |  |  |  |  |  |  |  |  |
| --- | --- | --- | --- | --- | --- | --- | --- | --- | --- | --- | --- | --- |

tr|A0A7N8WMA4|A0A7N8WMA4\_9TELE

back to list

  

| Protein Coverage
| Supporting Peptides
|

Protein Coverage:

Supporting Peptides:

| Peptide | Uniq | -10lgP | Mass | ppm | m/z | z | RT | Scan | #Spec | Start | End | PTM |
| --- | --- | --- | --- | --- | --- | --- | --- | --- | --- | --- | --- | --- |
| F.IFDVLEDPVEILDNSRE.L | N | 44.53 | 2001.9894 | -0.9 | 1002.0010 | 2 | 14.67 | 2236 | 1 | 142 | 158 |  |
| F.MDDPVVIPGKPY.T | N | 35.25 | 1329.6638 | -0.8 | 665.8386 | 2 | 12.28 | 1829 | 1 | 225 | 236 |  |
| total 2 peptides |  |  |  |  |  |  |  |  |  |  |  |  |
| --- | --- | --- | --- | --- | --- | --- | --- | --- | --- | --- | --- | --- |

tr|A0A7N8XJX7|A0A7N8XJX7\_9TELE

back to list

  

| Protein Coverage
| Supporting Peptides
|

Protein Coverage:

Supporting Peptides:

| Peptide | Uniq | -10lgP | Mass | ppm | m/z | z | RT | Scan | #Spec | Start | End | PTM |
| --- | --- | --- | --- | --- | --- | --- | --- | --- | --- | --- | --- | --- |
| F.IFDVLEDPVEILDNSRE.L | N | 44.53 | 2001.9894 | -0.9 | 1002.0010 | 2 | 14.67 | 2236 | 1 | 142 | 158 |  |
| F.MDDPVVIPGKPY.T | N | 35.25 | 1329.6638 | -0.8 | 665.8386 | 2 | 12.28 | 1829 | 1 | 225 | 236 |  |
| total 2 peptides |  |  |  |  |  |  |  |  |  |  |  |  |
| --- | --- | --- | --- | --- | --- | --- | --- | --- | --- | --- | --- | --- |

tr|A0A3Q3SHA7|A0A3Q3SHA7\_9TELE

back to list

  

| Protein Coverage
| Supporting Peptides
|

Protein Coverage:

Supporting Peptides:

| Peptide | Uniq | -10lgP | Mass | ppm | m/z | z | RT | Scan | #Spec | Start | End | PTM |
| --- | --- | --- | --- | --- | --- | --- | --- | --- | --- | --- | --- | --- |
| F.IFDVLEDPVEILDNSRE.L | N | 44.53 | 2001.9894 | -0.9 | 1002.0010 | 2 | 14.67 | 2236 | 1 | 154 | 170 |  |
| F.MDDPVVIPGKPY.T | N | 35.25 | 1329.6638 | -0.8 | 665.8386 | 2 | 12.28 | 1829 | 1 | 237 | 248 |  |
| total 2 peptides |  |  |  |  |  |  |  |  |  |  |  |  |
| --- | --- | --- | --- | --- | --- | --- | --- | --- | --- | --- | --- | --- |

tr|A0A7N8XSZ9|A0A7N8XSZ9\_9TELE

back to list

  

| Protein Coverage
| Supporting Peptides
|

Protein Coverage:

Supporting Peptides:

| Peptide | Uniq | -10lgP | Mass | ppm | m/z | z | RT | Scan | #Spec | Start | End | PTM |
| --- | --- | --- | --- | --- | --- | --- | --- | --- | --- | --- | --- | --- |
| F.IFDVLEDPVEILDNSRE.L | N | 44.53 | 2001.9894 | -0.9 | 1002.0010 | 2 | 14.67 | 2236 | 1 | 154 | 170 |  |
| F.MDDPVVIPGKPY.T | N | 35.25 | 1329.6638 | -0.8 | 665.8386 | 2 | 12.28 | 1829 | 1 | 237 | 248 |  |
| total 2 peptides |  |  |  |  |  |  |  |  |  |  |  |  |
| --- | --- | --- | --- | --- | --- | --- | --- | --- | --- | --- | --- | --- |

tr|A0A7N8XRW1|A0A7N8XRW1\_9TELE

back to list

  

| Protein Coverage
| Supporting Peptides
|

Protein Coverage:

Supporting Peptides:

| Peptide | Uniq | -10lgP | Mass | ppm | m/z | z | RT | Scan | #Spec | Start | End | PTM |
| --- | --- | --- | --- | --- | --- | --- | --- | --- | --- | --- | --- | --- |
| F.IFDVLEDPVEILDNSRE.L | N | 44.53 | 2001.9894 | -0.9 | 1002.0010 | 2 | 14.67 | 2236 | 1 | 155 | 171 |  |
| F.MDDPVVIPGKPY.T | N | 35.25 | 1329.6638 | -0.8 | 665.8386 | 2 | 12.28 | 1829 | 1 | 238 | 249 |  |
| total 2 peptides |  |  |  |  |  |  |  |  |  |  |  |  |
| --- | --- | --- | --- | --- | --- | --- | --- | --- | --- | --- | --- | --- |

tr|A0A7N8Y2R6|A0A7N8Y2R6\_9TELE

back to list

  

| Protein Coverage
| Supporting Peptides
|

Protein Coverage:

Supporting Peptides:

| Peptide | Uniq | -10lgP | Mass | ppm | m/z | z | RT | Scan | #Spec | Start | End | PTM |
| --- | --- | --- | --- | --- | --- | --- | --- | --- | --- | --- | --- | --- |
| F.IFDVLEDPVEILDNSRE.L | N | 44.53 | 2001.9894 | -0.9 | 1002.0010 | 2 | 14.67 | 2236 | 1 | 155 | 171 |  |
| F.MDDPVVIPGKPY.T | N | 35.25 | 1329.6638 | -0.8 | 665.8386 | 2 | 12.28 | 1829 | 1 | 238 | 249 |  |
| total 2 peptides |  |  |  |  |  |  |  |  |  |  |  |  |
| --- | --- | --- | --- | --- | --- | --- | --- | --- | --- | --- | --- | --- |

tr|A0A7N8Y336|A0A7N8Y336\_9TELE

back to list

  

| Protein Coverage
| Supporting Peptides
|

Protein Coverage:

Supporting Peptides:

| Peptide | Uniq | -10lgP | Mass | ppm | m/z | z | RT | Scan | #Spec | Start | End | PTM |
| --- | --- | --- | --- | --- | --- | --- | --- | --- | --- | --- | --- | --- |
| F.IFDVLEDPVEILDNSRE.L | N | 44.53 | 2001.9894 | -0.9 | 1002.0010 | 2 | 14.67 | 2236 | 1 | 158 | 174 |  |
| F.MDDPVVIPGKPY.T | N | 35.25 | 1329.6638 | -0.8 | 665.8386 | 2 | 12.28 | 1829 | 1 | 241 | 252 |  |
| total 2 peptides |  |  |  |  |  |  |  |  |  |  |  |  |
| --- | --- | --- | --- | --- | --- | --- | --- | --- | --- | --- | --- | --- |

tr|I3JGN1|I3JGN1\_ORENI

back to list

  

| Protein Coverage
| Supporting Peptides
|

Protein Coverage:

Supporting Peptides:

| Peptide | Uniq | -10lgP | Mass | ppm | m/z | z | RT | Scan | #Spec | Start | End | PTM |
| --- | --- | --- | --- | --- | --- | --- | --- | --- | --- | --- | --- | --- |
| M.GPRGPPGPPGSSGPQG.F | N | 37.56 | 1400.6796 | -0.2 | 701.3469 | 2 | 9.77 | 1397 | 1 | 163 | 178 |  |
| G.LRGDKGETGEAGER.G | N | 23.05 | 1473.7172 | -0.3 | 492.2462 | 3 | 8.56 | 1206 | 1 | 900 | 913 |  |
| G.FAGPPGADGQPGAK.G | N | 22.00 | 1268.6149 | -0.4 | 635.3145 | 2 | 10.23 | 1471 | 1 | 677 | 690 |  |
| total 3 peptides |  |  |  |  |  |  |  |  |  |  |  |  |
| --- | --- | --- | --- | --- | --- | --- | --- | --- | --- | --- | --- | --- |

tr|G9M6I5|G9M6I5\_ORENI

back to list

  

| Protein Coverage
| Supporting Peptides
|

Protein Coverage:

Supporting Peptides:

| Peptide | Uniq | -10lgP | Mass | ppm | m/z | z | RT | Scan | #Spec | Start | End | PTM |
| --- | --- | --- | --- | --- | --- | --- | --- | --- | --- | --- | --- | --- |
| M.GPRGPPGPPGSSGPQG.F | N | 37.56 | 1400.6796 | -0.2 | 701.3469 | 2 | 9.77 | 1397 | 1 | 169 | 184 |  |
| G.LRGDKGETGEAGER.G | N | 23.05 | 1473.7172 | -0.3 | 492.2462 | 3 | 8.56 | 1206 | 1 | 1076 | 1089 |  |
| G.FAGPPGADGQPGAK.G | N | 22.00 | 1268.6149 | -0.4 | 635.3145 | 2 | 10.23 | 1471 | 1 | 797 | 810 |  |
| total 3 peptides |  |  |  |  |  |  |  |  |  |  |  |  |
| --- | --- | --- | --- | --- | --- | --- | --- | --- | --- | --- | --- | --- |

tr|Q4RE90|Q4RE90\_TETNG

back to list

  

| Protein Coverage
| Supporting Peptides
|

Protein Coverage:

Supporting Peptides:

| Peptide | Uniq | -10lgP | Mass | ppm | m/z | z | RT | Scan | #Spec | Start | End | PTM |
| --- | --- | --- | --- | --- | --- | --- | --- | --- | --- | --- | --- | --- |
| L.AVDPVYPPGPPAFPK.V | Y | 34.33 | 1550.8132 | -1.1 | 776.4130 | 2 | 12.36 | 1842 | 1 | 7117 | 7131 |  |
| T.VVDRPQPPEGPVVF.N | Y | 25.63 | 1534.8143 | -1.2 | 768.4135 | 2 | 12.54 | 1875 | 1 | 4491 | 4504 |  |
| E.VVDVPGPVTDLKP.V | Y | 25.28 | 1334.7445 | -1.1 | 668.3788 | 2 | 12.39 | 1848 | 1 | 2931 | 2943 |  |
| total 3 peptides |  |  |  |  |  |  |  |  |  |  |  |  |
| --- | --- | --- | --- | --- | --- | --- | --- | --- | --- | --- | --- | --- |

tr|A0A484CUS5|A0A484CUS5\_PERFV

back to list

  

| Protein Coverage
| Supporting Peptides
|

Protein Coverage:

Supporting Peptides:

| Peptide | Uniq | -10lgP | Mass | ppm | m/z | z | RT | Scan | #Spec | Start | End | PTM |
| --- | --- | --- | --- | --- | --- | --- | --- | --- | --- | --- | --- | --- |
| T.VVDRPTPPVGPVVF.E | Y | 45.69 | 1477.8292 | -1.2 | 739.9210 | 2 | 13.05 | 1964 | 1 | 3654 | 3667 |  |
| A.ILPPGPPTPW.I | N | 19.56 | 1073.5909 | 0.5 | 537.8030 | 2 | 13.74 | 2081 | 1 | 1746 | 1755 |  |
| total 2 peptides |  |  |  |  |  |  |  |  |  |  |  |  |
| --- | --- | --- | --- | --- | --- | --- | --- | --- | --- | --- | --- | --- |

tr|A0A6G0I8D2|A0A6G0I8D2\_LARCR

back to list

  

| Protein Coverage
| Supporting Peptides
|

Protein Coverage:

Supporting Peptides:

| Peptide | Uniq | -10lgP | Mass | ppm | m/z | z | RT | Scan | #Spec | Start | End | PTM |
| --- | --- | --- | --- | --- | --- | --- | --- | --- | --- | --- | --- | --- |
| R.IGEDFIRD.L | Y | 34.82 | 963.4661 | -1.1 | 482.7398 | 2 | 11.83 | 1747 | 1 | 508 | 515 |  |
| F.ADYEDYIK.C | Y | 26.24 | 1015.4498 | -0.9 | 508.7317 | 2 | 11.59 | 1705 | 1 | 776 | 783 |  |
| E.RIGEDFIRDL.D | Y | 17.59 | 1232.6512 | -1.5 | 617.3320 | 2 | 12.88 | 1935 | 1 | 507 | 516 |  |
| total 3 peptides |  |  |  |  |  |  |  |  |  |  |  |  |
| --- | --- | --- | --- | --- | --- | --- | --- | --- | --- | --- | --- | --- |

tr|A0A669CG83|A0A669CG83\_ORENI

back to list

  

| Protein Coverage
| Supporting Peptides
|

Protein Coverage:

Supporting Peptides:

| Peptide | Uniq | -10lgP | Mass | ppm | m/z | z | RT | Scan | #Spec | Start | End | PTM |
| --- | --- | --- | --- | --- | --- | --- | --- | --- | --- | --- | --- | --- |
| R.IGEDFIRD.L | Y | 34.82 | 963.4661 | -1.1 | 482.7398 | 2 | 11.83 | 1747 | 1 | 508 | 515 |  |
| F.ADYEDYIK.C | Y | 26.24 | 1015.4498 | -0.9 | 508.7317 | 2 | 11.59 | 1705 | 1 | 743 | 750 |  |
| E.RIGEDFIRDL.D | Y | 17.59 | 1232.6512 | -1.5 | 617.3320 | 2 | 12.88 | 1935 | 1 | 507 | 516 |  |
| total 3 peptides |  |  |  |  |  |  |  |  |  |  |  |  |
| --- | --- | --- | --- | --- | --- | --- | --- | --- | --- | --- | --- | --- |

tr|A0A3P8QJ74|A0A3P8QJ74\_ASTCA

back to list

  

| Protein Coverage
| Supporting Peptides
|

Protein Coverage:

Supporting Peptides:

| Peptide | Uniq | -10lgP | Mass | ppm | m/z | z | RT | Scan | #Spec | Start | End | PTM |
| --- | --- | --- | --- | --- | --- | --- | --- | --- | --- | --- | --- | --- |
| R.IGEDFIRD.L | Y | 34.82 | 963.4661 | -1.1 | 482.7398 | 2 | 11.83 | 1747 | 1 | 460 | 467 |  |
| F.ADYEDYIK.C | Y | 26.24 | 1015.4498 | -0.9 | 508.7317 | 2 | 11.59 | 1705 | 1 | 728 | 735 |  |
| E.RIGEDFIRDL.D | Y | 17.59 | 1232.6512 | -1.5 | 617.3320 | 2 | 12.88 | 1935 | 1 | 459 | 468 |  |
| total 3 peptides |  |  |  |  |  |  |  |  |  |  |  |  |
| --- | --- | --- | --- | --- | --- | --- | --- | --- | --- | --- | --- | --- |

tr|A0A669E543|A0A669E543\_ORENI

back to list

  

| Protein Coverage
| Supporting Peptides
|

Protein Coverage:

Supporting Peptides:

| Peptide | Uniq | -10lgP | Mass | ppm | m/z | z | RT | Scan | #Spec | Start | End | PTM |
| --- | --- | --- | --- | --- | --- | --- | --- | --- | --- | --- | --- | --- |
| R.IGEDFIRD.L | Y | 34.82 | 963.4661 | -1.1 | 482.7398 | 2 | 11.83 | 1747 | 1 | 508 | 515 |  |
| F.ADYEDYIK.C | Y | 26.24 | 1015.4498 | -0.9 | 508.7317 | 2 | 11.59 | 1705 | 1 | 748 | 755 |  |
| E.RIGEDFIRDL.D | Y | 17.59 | 1232.6512 | -1.5 | 617.3320 | 2 | 12.88 | 1935 | 1 | 507 | 516 |  |
| total 3 peptides |  |  |  |  |  |  |  |  |  |  |  |  |
| --- | --- | --- | --- | --- | --- | --- | --- | --- | --- | --- | --- | --- |

tr|A0A3B4C8L5|A0A3B4C8L5\_PYGNA

back to list

  

| Protein Coverage
| Supporting Peptides
|

Protein Coverage:

Supporting Peptides:

| Peptide | Uniq | -10lgP | Mass | ppm | m/z | z | RT | Scan | #Spec | Start | End | PTM |
| --- | --- | --- | --- | --- | --- | --- | --- | --- | --- | --- | --- | --- |
| R.IGEDFIRD.L | Y | 34.82 | 963.4661 | -1.1 | 482.7398 | 2 | 11.83 | 1747 | 1 | 486 | 493 |  |
| F.ADYEDYIK.C | Y | 26.24 | 1015.4498 | -0.9 | 508.7317 | 2 | 11.59 | 1705 | 1 | 753 | 760 |  |
| E.RIGEDFIRDL.D | Y | 17.59 | 1232.6512 | -1.5 | 617.3320 | 2 | 12.88 | 1935 | 1 | 485 | 494 |  |
| total 3 peptides |  |  |  |  |  |  |  |  |  |  |  |  |
| --- | --- | --- | --- | --- | --- | --- | --- | --- | --- | --- | --- | --- |

tr|A0A669F5E7|A0A669F5E7\_ORENI

back to list

  

| Protein Coverage
| Supporting Peptides
|

Protein Coverage:

Supporting Peptides:

| Peptide | Uniq | -10lgP | Mass | ppm | m/z | z | RT | Scan | #Spec | Start | End | PTM |
| --- | --- | --- | --- | --- | --- | --- | --- | --- | --- | --- | --- | --- |
| R.IGEDFIRD.L | Y | 34.82 | 963.4661 | -1.1 | 482.7398 | 2 | 11.83 | 1747 | 1 | 508 | 515 |  |
| F.ADYEDYIK.C | Y | 26.24 | 1015.4498 | -0.9 | 508.7317 | 2 | 11.59 | 1705 | 1 | 743 | 750 |  |
| E.RIGEDFIRDL.D | Y | 17.59 | 1232.6512 | -1.5 | 617.3320 | 2 | 12.88 | 1935 | 1 | 507 | 516 |  |
| total 3 peptides |  |  |  |  |  |  |  |  |  |  |  |  |
| --- | --- | --- | --- | --- | --- | --- | --- | --- | --- | --- | --- | --- |

tr|A0A672GIA2|A0A672GIA2\_SALFA

back to list

  

| Protein Coverage
| Supporting Peptides
|

Protein Coverage:

Supporting Peptides:

| Peptide | Uniq | -10lgP | Mass | ppm | m/z | z | RT | Scan | #Spec | Start | End | PTM |
| --- | --- | --- | --- | --- | --- | --- | --- | --- | --- | --- | --- | --- |
| R.IGEDFIRD.L | Y | 34.82 | 963.4661 | -1.1 | 482.7398 | 2 | 11.83 | 1747 | 1 | 501 | 508 |  |
| F.ADYEDYIK.C | Y | 26.24 | 1015.4498 | -0.9 | 508.7317 | 2 | 11.59 | 1705 | 1 | 769 | 776 |  |
| E.RIGEDFIRDL.D | Y | 17.59 | 1232.6512 | -1.5 | 617.3320 | 2 | 12.88 | 1935 | 1 | 500 | 509 |  |
| total 3 peptides |  |  |  |  |  |  |  |  |  |  |  |  |
| --- | --- | --- | --- | --- | --- | --- | --- | --- | --- | --- | --- | --- |

tr|A0A3B4B6P0|A0A3B4B6P0\_9GOBI

back to list

  

| Protein Coverage
| Supporting Peptides
|

Protein Coverage:

Supporting Peptides:

| Peptide | Uniq | -10lgP | Mass | ppm | m/z | z | RT | Scan | #Spec | Start | End | PTM |
| --- | --- | --- | --- | --- | --- | --- | --- | --- | --- | --- | --- | --- |
| R.IGEDFIRD.L | Y | 34.82 | 963.4661 | -1.1 | 482.7398 | 2 | 11.83 | 1747 | 1 | 508 | 515 |  |
| F.ADYEDYIK.C | Y | 26.24 | 1015.4498 | -0.9 | 508.7317 | 2 | 11.59 | 1705 | 1 | 776 | 783 |  |
| E.RIGEDFIRDL.D | Y | 17.59 | 1232.6512 | -1.5 | 617.3320 | 2 | 12.88 | 1935 | 1 | 507 | 516 |  |
| total 3 peptides |  |  |  |  |  |  |  |  |  |  |  |  |
| --- | --- | --- | --- | --- | --- | --- | --- | --- | --- | --- | --- | --- |

tr|A0A6P7PB25|A0A6P7PB25\_BETSP

back to list

  

| Protein Coverage
| Supporting Peptides
|

Protein Coverage:

Supporting Peptides:

| Peptide | Uniq | -10lgP | Mass | ppm | m/z | z | RT | Scan | #Spec | Start | End | PTM |
| --- | --- | --- | --- | --- | --- | --- | --- | --- | --- | --- | --- | --- |
| R.IGEDFIRD.L | Y | 34.82 | 963.4661 | -1.1 | 482.7398 | 2 | 11.83 | 1747 | 1 | 508 | 515 |  |
| F.ADYEDYIK.C | Y | 26.24 | 1015.4498 | -0.9 | 508.7317 | 2 | 11.59 | 1705 | 1 | 776 | 783 |  |
| E.RIGEDFIRDL.D | Y | 17.59 | 1232.6512 | -1.5 | 617.3320 | 2 | 12.88 | 1935 | 1 | 507 | 516 |  |
| total 3 peptides |  |  |  |  |  |  |  |  |  |  |  |  |
| --- | --- | --- | --- | --- | --- | --- | --- | --- | --- | --- | --- | --- |

tr|M4A9F5|M4A9F5\_XIPMA

back to list

  

| Protein Coverage
| Supporting Peptides
|

Protein Coverage:

Supporting Peptides:

| Peptide | Uniq | -10lgP | Mass | ppm | m/z | z | RT | Scan | #Spec | Start | End | PTM |
| --- | --- | --- | --- | --- | --- | --- | --- | --- | --- | --- | --- | --- |
| R.IGEDFIRD.L | Y | 34.82 | 963.4661 | -1.1 | 482.7398 | 2 | 11.83 | 1747 | 1 | 508 | 515 |  |
| F.ADYEDYIK.C | Y | 26.24 | 1015.4498 | -0.9 | 508.7317 | 2 | 11.59 | 1705 | 1 | 776 | 783 |  |
| E.RIGEDFIRDL.D | Y | 17.59 | 1232.6512 | -1.5 | 617.3320 | 2 | 12.88 | 1935 | 1 | 507 | 516 |  |
| total 3 peptides |  |  |  |  |  |  |  |  |  |  |  |  |
| --- | --- | --- | --- | --- | --- | --- | --- | --- | --- | --- | --- | --- |

tr|A0A498LG69|A0A498LG69\_LABRO

back to list

  

| Protein Coverage
| Supporting Peptides
|

Protein Coverage:

Supporting Peptides:

| Peptide | Uniq | -10lgP | Mass | ppm | m/z | z | RT | Scan | #Spec | Start | End | PTM |
| --- | --- | --- | --- | --- | --- | --- | --- | --- | --- | --- | --- | --- |
| R.IGEDFIRD.L | Y | 34.82 | 963.4661 | -1.1 | 482.7398 | 2 | 11.83 | 1747 | 1 | 508 | 515 |  |
| F.ADYEDYIK.C | Y | 26.24 | 1015.4498 | -0.9 | 508.7317 | 2 | 11.59 | 1705 | 1 | 776 | 783 |  |
| E.RIGEDFIRDL.D | Y | 17.59 | 1232.6512 | -1.5 | 617.3320 | 2 | 12.88 | 1935 | 1 | 507 | 516 |  |
| total 3 peptides |  |  |  |  |  |  |  |  |  |  |  |  |
| --- | --- | --- | --- | --- | --- | --- | --- | --- | --- | --- | --- | --- |

tr|A0A0S7H0F8|A0A0S7H0F8\_9TELE

back to list

  

| Protein Coverage
| Supporting Peptides
|

Protein Coverage:

Supporting Peptides:

| Peptide | Uniq | -10lgP | Mass | ppm | m/z | z | RT | Scan | #Spec | Start | End | PTM |
| --- | --- | --- | --- | --- | --- | --- | --- | --- | --- | --- | --- | --- |
| R.IGEDFIRD.L | Y | 34.82 | 963.4661 | -1.1 | 482.7398 | 2 | 11.83 | 1747 | 1 | 508 | 515 |  |
| F.ADYEDYIK.C | Y | 26.24 | 1015.4498 | -0.9 | 508.7317 | 2 | 11.59 | 1705 | 1 | 776 | 783 |  |
| E.RIGEDFIRDL.D | Y | 17.59 | 1232.6512 | -1.5 | 617.3320 | 2 | 12.88 | 1935 | 1 | 507 | 516 |  |
| total 3 peptides |  |  |  |  |  |  |  |  |  |  |  |  |
| --- | --- | --- | --- | --- | --- | --- | --- | --- | --- | --- | --- | --- |

tr|A0A5J5D262|A0A5J5D262\_9PERO

back to list

  

| Protein Coverage
| Supporting Peptides
|

Protein Coverage:

Supporting Peptides:

| Peptide | Uniq | -10lgP | Mass | ppm | m/z | z | RT | Scan | #Spec | Start | End | PTM |
| --- | --- | --- | --- | --- | --- | --- | --- | --- | --- | --- | --- | --- |
| R.IGEDFIRD.L | Y | 34.82 | 963.4661 | -1.1 | 482.7398 | 2 | 11.83 | 1747 | 1 | 508 | 515 |  |
| F.ADYEDYIK.C | Y | 26.24 | 1015.4498 | -0.9 | 508.7317 | 2 | 11.59 | 1705 | 1 | 767 | 774 |  |
| E.RIGEDFIRDL.D | Y | 17.59 | 1232.6512 | -1.5 | 617.3320 | 2 | 12.88 | 1935 | 1 | 507 | 516 |  |
| total 3 peptides |  |  |  |  |  |  |  |  |  |  |  |  |
| --- | --- | --- | --- | --- | --- | --- | --- | --- | --- | --- | --- | --- |

tr|A0A6I9P2J6|A0A6I9P2J6\_9TELE

back to list

  

| Protein Coverage
| Supporting Peptides
|

Protein Coverage:

Supporting Peptides:

| Peptide | Uniq | -10lgP | Mass | ppm | m/z | z | RT | Scan | #Spec | Start | End | PTM |
| --- | --- | --- | --- | --- | --- | --- | --- | --- | --- | --- | --- | --- |
| F.IFDVLEDPVEIIDNSRE.L | N | 44.53 | 2001.9894 | -0.9 | 1002.0010 | 2 | 14.67 | 2236 | 1 | 142 | 158 |  |
| S.IVWIDPDDFPL.L | N | 15.72 | 1328.6652 | -0.2 | 665.3397 | 2 | 16.16 | 2489 | 1 | 307 | 317 |  |
| total 2 peptides |  |  |  |  |  |  |  |  |  |  |  |  |
| --- | --- | --- | --- | --- | --- | --- | --- | --- | --- | --- | --- | --- |

tr|A0A2U9BKK8|A0A2U9BKK8\_SCOMX

back to list

  

| Protein Coverage
| Supporting Peptides
|

Protein Coverage:

Supporting Peptides:

| Peptide | Uniq | -10lgP | Mass | ppm | m/z | z | RT | Scan | #Spec | Start | End | PTM |
| --- | --- | --- | --- | --- | --- | --- | --- | --- | --- | --- | --- | --- |
| F.IFDVLEDPVEIIDNSRE.L | N | 44.53 | 2001.9894 | -0.9 | 1002.0010 | 2 | 14.67 | 2236 | 1 | 142 | 158 |  |
| S.IVWIDPDDFPL.L | N | 15.72 | 1328.6652 | -0.2 | 665.3397 | 2 | 16.16 | 2489 | 1 | 307 | 317 |  |
| total 2 peptides |  |  |  |  |  |  |  |  |  |  |  |  |
| --- | --- | --- | --- | --- | --- | --- | --- | --- | --- | --- | --- | --- |

tr|A0A6J2RKV3|A0A6J2RKV3\_COTGO

back to list

  

| Protein Coverage
| Supporting Peptides
|

Protein Coverage:

Supporting Peptides:

| Peptide | Uniq | -10lgP | Mass | ppm | m/z | z | RT | Scan | #Spec | Start | End | PTM |
| --- | --- | --- | --- | --- | --- | --- | --- | --- | --- | --- | --- | --- |
| F.IFDVLEDPVEIIDNSRE.L | N | 44.53 | 2001.9894 | -0.9 | 1002.0010 | 2 | 14.67 | 2236 | 1 | 142 | 158 |  |
| S.IVWIDPDDFPL.L | N | 15.72 | 1328.6652 | -0.2 | 665.3397 | 2 | 16.16 | 2489 | 1 | 307 | 317 |  |
| total 2 peptides |  |  |  |  |  |  |  |  |  |  |  |  |
| --- | --- | --- | --- | --- | --- | --- | --- | --- | --- | --- | --- | --- |

tr|A0A6P8WKA1|A0A6P8WKA1\_GYMAC

back to list

  

| Protein Coverage
| Supporting Peptides
|

Protein Coverage:

Supporting Peptides:

| Peptide | Uniq | -10lgP | Mass | ppm | m/z | z | RT | Scan | #Spec | Start | End | PTM |
| --- | --- | --- | --- | --- | --- | --- | --- | --- | --- | --- | --- | --- |
| F.IFDVLEDPVEIIDNSRE.L | N | 44.53 | 2001.9894 | -0.9 | 1002.0010 | 2 | 14.67 | 2236 | 1 | 142 | 158 |  |
| S.IVWIDPDDFPL.L | N | 15.72 | 1328.6652 | -0.2 | 665.3397 | 2 | 16.16 | 2489 | 1 | 307 | 317 |  |
| total 2 peptides |  |  |  |  |  |  |  |  |  |  |  |  |
| --- | --- | --- | --- | --- | --- | --- | --- | --- | --- | --- | --- | --- |

tr|A0A6A4TIJ9|A0A6A4TIJ9\_SCOMX

back to list

  

| Protein Coverage
| Supporting Peptides
|

Protein Coverage:

Supporting Peptides:

| Peptide | Uniq | -10lgP | Mass | ppm | m/z | z | RT | Scan | #Spec | Start | End | PTM |
| --- | --- | --- | --- | --- | --- | --- | --- | --- | --- | --- | --- | --- |
| F.IFDVLEDPVEIIDNSRE.L | N | 44.53 | 2001.9894 | -0.9 | 1002.0010 | 2 | 14.67 | 2236 | 1 | 142 | 158 |  |
| S.IVWIDPDDFPL.L | N | 15.72 | 1328.6652 | -0.2 | 665.3397 | 2 | 16.16 | 2489 | 1 | 307 | 317 |  |
| total 2 peptides |  |  |  |  |  |  |  |  |  |  |  |  |
| --- | --- | --- | --- | --- | --- | --- | --- | --- | --- | --- | --- | --- |

tr|A0A3P8VNQ7|A0A3P8VNQ7\_CYNSE

back to list

  

| Protein Coverage
| Supporting Peptides
|

Protein Coverage:

Supporting Peptides:

| Peptide | Uniq | -10lgP | Mass | ppm | m/z | z | RT | Scan | #Spec | Start | End | PTM |
| --- | --- | --- | --- | --- | --- | --- | --- | --- | --- | --- | --- | --- |
| F.IFDVLEDPVEIIDNSRE.L | N | 44.53 | 2001.9894 | -0.9 | 1002.0010 | 2 | 14.67 | 2236 | 1 | 142 | 158 |  |
| S.IVWIDPDDFPL.L | N | 15.72 | 1328.6652 | -0.2 | 665.3397 | 2 | 16.16 | 2489 | 1 | 307 | 317 |  |
| total 2 peptides |  |  |  |  |  |  |  |  |  |  |  |  |
| --- | --- | --- | --- | --- | --- | --- | --- | --- | --- | --- | --- | --- |

tr|A0A3P8VQY9|A0A3P8VQY9\_CYNSE

back to list

  

| Protein Coverage
| Supporting Peptides
|

Protein Coverage:

Supporting Peptides:

| Peptide | Uniq | -10lgP | Mass | ppm | m/z | z | RT | Scan | #Spec | Start | End | PTM |
| --- | --- | --- | --- | --- | --- | --- | --- | --- | --- | --- | --- | --- |
| F.IFDVLEDPVEIIDNSRE.L | N | 44.53 | 2001.9894 | -0.9 | 1002.0010 | 2 | 14.67 | 2236 | 1 | 164 | 180 |  |
| S.IVWIDPDDFPL.L | N | 15.72 | 1328.6652 | -0.2 | 665.3397 | 2 | 16.16 | 2489 | 1 | 329 | 339 |  |
| total 2 peptides |  |  |  |  |  |  |  |  |  |  |  |  |
| --- | --- | --- | --- | --- | --- | --- | --- | --- | --- | --- | --- | --- |

tr|A0A2I4B9K0|A0A2I4B9K0\_9TELE

back to list

  

| Protein Coverage
| Supporting Peptides
|

Protein Coverage:

Supporting Peptides:

| Peptide | Uniq | -10lgP | Mass | ppm | m/z | z | RT | Scan | #Spec | Start | End | PTM |
| --- | --- | --- | --- | --- | --- | --- | --- | --- | --- | --- | --- | --- |
| F.IFDVLEDPVEIIDNSRE.L | N | 44.53 | 2001.9894 | -0.9 | 1002.0010 | 2 | 14.67 | 2236 | 1 | 142 | 158 |  |
| S.IVWIDPDDFPL.L | N | 15.72 | 1328.6652 | -0.2 | 665.3397 | 2 | 16.16 | 2489 | 1 | 307 | 317 |  |
| total 2 peptides |  |  |  |  |  |  |  |  |  |  |  |  |
| --- | --- | --- | --- | --- | --- | --- | --- | --- | --- | --- | --- | --- |

tr|A0A673A8U8|A0A673A8U8\_9TELE

back to list

  

| Protein Coverage
| Supporting Peptides
|

Protein Coverage:

Supporting Peptides:

| Peptide | Uniq | -10lgP | Mass | ppm | m/z | z | RT | Scan | #Spec | Start | End | PTM |
| --- | --- | --- | --- | --- | --- | --- | --- | --- | --- | --- | --- | --- |
| M.GPRGPPGPPGSSGPQG.F | N | 37.56 | 1400.6796 | -0.2 | 701.3469 | 2 | 9.77 | 1397 | 1 | 78 | 93 |  |
| G.FSGLPGPSGEPGKQGPSG.V | Y | 27.23 | 1654.7950 | 0.1 | 828.4049 | 2 | 11.29 | 1652 | 1 | 663 | 680 |  |
| total 2 peptides |  |  |  |  |  |  |  |  |  |  |  |  |
| --- | --- | --- | --- | --- | --- | --- | --- | --- | --- | --- | --- | --- |

tr|A0A671U7M6|A0A671U7M6\_SPAAU

back to list

  

| Protein Coverage
| Supporting Peptides
|

Protein Coverage:

Supporting Peptides:

| Peptide | Uniq | -10lgP | Mass | ppm | m/z | z | RT | Scan | #Spec | Start | End | PTM |
| --- | --- | --- | --- | --- | --- | --- | --- | --- | --- | --- | --- | --- |
| H.LNEDKLKDKINE.L | Y | 41.87 | 1457.7725 | -0.2 | 729.8934 | 2 | 10.04 | 1439 | 1 | 217 | 228 |  |
| N.IDHLNEDKLKDKINE.L | Y | 15.27 | 1822.9424 | 0.3 | 608.6549 | 3 | 10.49 | 1513 | 1 | 214 | 228 |  |
| total 2 peptides |  |  |  |  |  |  |  |  |  |  |  |  |
| --- | --- | --- | --- | --- | --- | --- | --- | --- | --- | --- | --- | --- |

tr|A0A3B3ZS88|A0A3B3ZS88\_9GOBI

back to list

  

| Protein Coverage
| Supporting Peptides
|

Protein Coverage:

Supporting Peptides:

| Peptide | Uniq | -10lgP | Mass | ppm | m/z | z | RT | Scan | #Spec | Start | End | PTM |
| --- | --- | --- | --- | --- | --- | --- | --- | --- | --- | --- | --- | --- |
| H.LNEDKLKDKINE.L | Y | 41.87 | 1457.7725 | -0.2 | 729.8934 | 2 | 10.04 | 1439 | 1 | 157 | 168 |  |
| N.IDHLNEDKLKDKINE.L | Y | 15.27 | 1822.9424 | 0.3 | 608.6549 | 3 | 10.49 | 1513 | 1 | 154 | 168 |  |
| total 2 peptides |  |  |  |  |  |  |  |  |  |  |  |  |
| --- | --- | --- | --- | --- | --- | --- | --- | --- | --- | --- | --- | --- |

tr|A0A3Q1CJW7|A0A3Q1CJW7\_AMPOC

back to list

  

| Protein Coverage
| Supporting Peptides
|

Protein Coverage:

Supporting Peptides:

| Peptide | Uniq | -10lgP | Mass | ppm | m/z | z | RT | Scan | #Spec | Start | End | PTM |
| --- | --- | --- | --- | --- | --- | --- | --- | --- | --- | --- | --- | --- |
| H.LNEDKLKDKINE.L | Y | 41.87 | 1457.7725 | -0.2 | 729.8934 | 2 | 10.04 | 1439 | 1 | 157 | 168 |  |
| N.IDHLNEDKLKDKINE.L | Y | 15.27 | 1822.9424 | 0.3 | 608.6549 | 3 | 10.49 | 1513 | 1 | 154 | 168 |  |
| total 2 peptides |  |  |  |  |  |  |  |  |  |  |  |  |
| --- | --- | --- | --- | --- | --- | --- | --- | --- | --- | --- | --- | --- |

tr|A0A3Q3EFK1|A0A3Q3EFK1\_9LABR

back to list

  

| Protein Coverage
| Supporting Peptides
|

Protein Coverage:

Supporting Peptides:

| Peptide | Uniq | -10lgP | Mass | ppm | m/z | z | RT | Scan | #Spec | Start | End | PTM |
| --- | --- | --- | --- | --- | --- | --- | --- | --- | --- | --- | --- | --- |
| H.LNEDKLKDKINE.L | Y | 41.87 | 1457.7725 | -0.2 | 729.8934 | 2 | 10.04 | 1439 | 1 | 162 | 173 |  |
| N.IDHLNEDKLKDKINE.L | Y | 15.27 | 1822.9424 | 0.3 | 608.6549 | 3 | 10.49 | 1513 | 1 | 159 | 173 |  |
| total 2 peptides |  |  |  |  |  |  |  |  |  |  |  |  |
| --- | --- | --- | --- | --- | --- | --- | --- | --- | --- | --- | --- | --- |

tr|A0A3Q3AF19|A0A3Q3AF19\_KRYMA

back to list

  

| Protein Coverage
| Supporting Peptides
|

Protein Coverage:

Supporting Peptides:

| Peptide | Uniq | -10lgP | Mass | ppm | m/z | z | RT | Scan | #Spec | Start | End | PTM |
| --- | --- | --- | --- | --- | --- | --- | --- | --- | --- | --- | --- | --- |
| H.LNEDKLKDKINE.L | Y | 41.87 | 1457.7725 | -0.2 | 729.8934 | 2 | 10.04 | 1439 | 1 | 162 | 173 |  |
| N.IDHLNEDKLKDKINE.L | Y | 15.27 | 1822.9424 | 0.3 | 608.6549 | 3 | 10.49 | 1513 | 1 | 159 | 173 |  |
| total 2 peptides |  |  |  |  |  |  |  |  |  |  |  |  |
| --- | --- | --- | --- | --- | --- | --- | --- | --- | --- | --- | --- | --- |

tr|A0A3Q3AF32|A0A3Q3AF32\_KRYMA

back to list

  

| Protein Coverage
| Supporting Peptides
|

Protein Coverage:

Supporting Peptides:

| Peptide | Uniq | -10lgP | Mass | ppm | m/z | z | RT | Scan | #Spec | Start | End | PTM |
| --- | --- | --- | --- | --- | --- | --- | --- | --- | --- | --- | --- | --- |
| H.LNEDKLKDKINE.L | Y | 41.87 | 1457.7725 | -0.2 | 729.8934 | 2 | 10.04 | 1439 | 1 | 171 | 182 |  |
| N.IDHLNEDKLKDKINE.L | Y | 15.27 | 1822.9424 | 0.3 | 608.6549 | 3 | 10.49 | 1513 | 1 | 168 | 182 |  |
| total 2 peptides |  |  |  |  |  |  |  |  |  |  |  |  |
| --- | --- | --- | --- | --- | --- | --- | --- | --- | --- | --- | --- | --- |

tr|A0A3P8UNE3|A0A3P8UNE3\_CYNSE

back to list

  

| Protein Coverage
| Supporting Peptides
|

Protein Coverage:

Supporting Peptides:

| Peptide | Uniq | -10lgP | Mass | ppm | m/z | z | RT | Scan | #Spec | Start | End | PTM |
| --- | --- | --- | --- | --- | --- | --- | --- | --- | --- | --- | --- | --- |
| H.LNEDKLKDKINE.L | Y | 41.87 | 1457.7725 | -0.2 | 729.8934 | 2 | 10.04 | 1439 | 1 | 168 | 179 |  |
| N.IDHLNEDKLKDKINE.L | Y | 15.27 | 1822.9424 | 0.3 | 608.6549 | 3 | 10.49 | 1513 | 1 | 165 | 179 |  |
| total 2 peptides |  |  |  |  |  |  |  |  |  |  |  |  |
| --- | --- | --- | --- | --- | --- | --- | --- | --- | --- | --- | --- | --- |

tr|A0SJB3|A0SJB3\_SPAAU

back to list

  

| Protein Coverage
| Supporting Peptides
|

Protein Coverage:

Supporting Peptides:

| Peptide | Uniq | -10lgP | Mass | ppm | m/z | z | RT | Scan | #Spec | Start | End | PTM |
| --- | --- | --- | --- | --- | --- | --- | --- | --- | --- | --- | --- | --- |
| H.LNEDKLKDKINE.L | Y | 41.87 | 1457.7725 | -0.2 | 729.8934 | 2 | 10.04 | 1439 | 1 | 168 | 179 |  |
| N.IDHLNEDKLKDKINE.L | Y | 15.27 | 1822.9424 | 0.3 | 608.6549 | 3 | 10.49 | 1513 | 1 | 165 | 179 |  |
| total 2 peptides |  |  |  |  |  |  |  |  |  |  |  |  |
| --- | --- | --- | --- | --- | --- | --- | --- | --- | --- | --- | --- | --- |

tr|A0A672Y3T4|A0A672Y3T4\_9TELE

back to list

  

| Protein Coverage
| Supporting Peptides
|

Protein Coverage:

Supporting Peptides:

| Peptide | Uniq | -10lgP | Mass | ppm | m/z | z | RT | Scan | #Spec | Start | End | PTM |
| --- | --- | --- | --- | --- | --- | --- | --- | --- | --- | --- | --- | --- |
| H.LNEDKLKDKINE.L | Y | 41.87 | 1457.7725 | -0.2 | 729.8934 | 2 | 10.04 | 1439 | 1 | 168 | 179 |  |
| N.IDHLNEDKLKDKINE.L | Y | 15.27 | 1822.9424 | 0.3 | 608.6549 | 3 | 10.49 | 1513 | 1 | 165 | 179 |  |
| total 2 peptides |  |  |  |  |  |  |  |  |  |  |  |  |
| --- | --- | --- | --- | --- | --- | --- | --- | --- | --- | --- | --- | --- |

tr|A0SJB2|A0SJB2\_SPAAU

back to list

  

| Protein Coverage
| Supporting Peptides
|

Protein Coverage:

Supporting Peptides:

| Peptide | Uniq | -10lgP | Mass | ppm | m/z | z | RT | Scan | #Spec | Start | End | PTM |
| --- | --- | --- | --- | --- | --- | --- | --- | --- | --- | --- | --- | --- |
| H.LNEDKLKDKINE.L | Y | 41.87 | 1457.7725 | -0.2 | 729.8934 | 2 | 10.04 | 1439 | 1 | 171 | 182 |  |
| N.IDHLNEDKLKDKINE.L | Y | 15.27 | 1822.9424 | 0.3 | 608.6549 | 3 | 10.49 | 1513 | 1 | 168 | 182 |  |
| total 2 peptides |  |  |  |  |  |  |  |  |  |  |  |  |
| --- | --- | --- | --- | --- | --- | --- | --- | --- | --- | --- | --- | --- |

tr|A0A3P8UNF6|A0A3P8UNF6\_CYNSE

back to list

  

| Protein Coverage
| Supporting Peptides
|

Protein Coverage:

Supporting Peptides:

| Peptide | Uniq | -10lgP | Mass | ppm | m/z | z | RT | Scan | #Spec | Start | End | PTM |
| --- | --- | --- | --- | --- | --- | --- | --- | --- | --- | --- | --- | --- |
| H.LNEDKLKDKINE.L | Y | 41.87 | 1457.7725 | -0.2 | 729.8934 | 2 | 10.04 | 1439 | 1 | 190 | 201 |  |
| N.IDHLNEDKLKDKINE.L | Y | 15.27 | 1822.9424 | 0.3 | 608.6549 | 3 | 10.49 | 1513 | 1 | 187 | 201 |  |
| total 2 peptides |  |  |  |  |  |  |  |  |  |  |  |  |
| --- | --- | --- | --- | --- | --- | --- | --- | --- | --- | --- | --- | --- |

tr|A0A3B4UYC0|A0A3B4UYC0\_SERDU

back to list

  

| Protein Coverage
| Supporting Peptides
|

Protein Coverage:

Supporting Peptides:

| Peptide | Uniq | -10lgP | Mass | ppm | m/z | z | RT | Scan | #Spec | Start | End | PTM |
| --- | --- | --- | --- | --- | --- | --- | --- | --- | --- | --- | --- | --- |
| H.LNEDKLKDKINE.L | Y | 41.87 | 1457.7725 | -0.2 | 729.8934 | 2 | 10.04 | 1439 | 1 | 159 | 170 |  |
| N.IDHLNEDKLKDKINE.L | Y | 15.27 | 1822.9424 | 0.3 | 608.6549 | 3 | 10.49 | 1513 | 1 | 156 | 170 |  |
| total 2 peptides |  |  |  |  |  |  |  |  |  |  |  |  |
| --- | --- | --- | --- | --- | --- | --- | --- | --- | --- | --- | --- | --- |

tr|A0A3P8URR7|A0A3P8URR7\_CYNSE

back to list

  

| Protein Coverage
| Supporting Peptides
|

Protein Coverage:

Supporting Peptides:

| Peptide | Uniq | -10lgP | Mass | ppm | m/z | z | RT | Scan | #Spec | Start | End | PTM |
| --- | --- | --- | --- | --- | --- | --- | --- | --- | --- | --- | --- | --- |
| H.LNEDKLKDKINE.L | Y | 41.87 | 1457.7725 | -0.2 | 729.8934 | 2 | 10.04 | 1439 | 1 | 169 | 180 |  |
| N.IDHLNEDKLKDKINE.L | Y | 15.27 | 1822.9424 | 0.3 | 608.6549 | 3 | 10.49 | 1513 | 1 | 166 | 180 |  |
| total 2 peptides |  |  |  |  |  |  |  |  |  |  |  |  |
| --- | --- | --- | --- | --- | --- | --- | --- | --- | --- | --- | --- | --- |

tr|A0A3P8URM3|A0A3P8URM3\_CYNSE

back to list

  

| Protein Coverage
| Supporting Peptides
|

Protein Coverage:

Supporting Peptides:

| Peptide | Uniq | -10lgP | Mass | ppm | m/z | z | RT | Scan | #Spec | Start | End | PTM |
| --- | --- | --- | --- | --- | --- | --- | --- | --- | --- | --- | --- | --- |
| H.LNEDKLKDKINE.L | Y | 41.87 | 1457.7725 | -0.2 | 729.8934 | 2 | 10.04 | 1439 | 1 | 193 | 204 |  |
| N.IDHLNEDKLKDKINE.L | Y | 15.27 | 1822.9424 | 0.3 | 608.6549 | 3 | 10.49 | 1513 | 1 | 190 | 204 |  |
| total 2 peptides |  |  |  |  |  |  |  |  |  |  |  |  |
| --- | --- | --- | --- | --- | --- | --- | --- | --- | --- | --- | --- | --- |

tr|A0A3Q3XL97|A0A3Q3XL97\_MOLML

back to list

  

| Protein Coverage
| Supporting Peptides
|

Protein Coverage:

Supporting Peptides:

| Peptide | Uniq | -10lgP | Mass | ppm | m/z | z | RT | Scan | #Spec | Start | End | PTM |
| --- | --- | --- | --- | --- | --- | --- | --- | --- | --- | --- | --- | --- |
| H.LNEDKLKDKINE.L | Y | 41.87 | 1457.7725 | -0.2 | 729.8934 | 2 | 10.04 | 1439 | 1 | 204 | 215 |  |
| N.IDHLNEDKLKDKINE.L | Y | 15.27 | 1822.9424 | 0.3 | 608.6549 | 3 | 10.49 | 1513 | 1 | 201 | 215 |  |
| total 2 peptides |  |  |  |  |  |  |  |  |  |  |  |  |
| --- | --- | --- | --- | --- | --- | --- | --- | --- | --- | --- | --- | --- |

tr|A0A3P8ULW6|A0A3P8ULW6\_CYNSE

back to list

  

| Protein Coverage
| Supporting Peptides
|

Protein Coverage:

Supporting Peptides:

| Peptide | Uniq | -10lgP | Mass | ppm | m/z | z | RT | Scan | #Spec | Start | End | PTM |
| --- | --- | --- | --- | --- | --- | --- | --- | --- | --- | --- | --- | --- |
| H.LNEDKLKDKINE.L | Y | 41.87 | 1457.7725 | -0.2 | 729.8934 | 2 | 10.04 | 1439 | 1 | 206 | 217 |  |
| N.IDHLNEDKLKDKINE.L | Y | 15.27 | 1822.9424 | 0.3 | 608.6549 | 3 | 10.49 | 1513 | 1 | 203 | 217 |  |
| total 2 peptides |  |  |  |  |  |  |  |  |  |  |  |  |
| --- | --- | --- | --- | --- | --- | --- | --- | --- | --- | --- | --- | --- |

tr|A0A5C6P7G2|A0A5C6P7G2\_9TELE

back to list

  

| Protein Coverage
| Supporting Peptides
|

Protein Coverage:

Supporting Peptides:

| Peptide | Uniq | -10lgP | Mass | ppm | m/z | z | RT | Scan | #Spec | Start | End | PTM |
| --- | --- | --- | --- | --- | --- | --- | --- | --- | --- | --- | --- | --- |
| H.LNEDKLKDKINE.L | Y | 41.87 | 1457.7725 | -0.2 | 729.8934 | 2 | 10.04 | 1439 | 1 | 211 | 222 |  |
| N.IDHLNEDKLKDKINE.L | Y | 15.27 | 1822.9424 | 0.3 | 608.6549 | 3 | 10.49 | 1513 | 1 | 208 | 222 |  |
| total 2 peptides |  |  |  |  |  |  |  |  |  |  |  |  |
| --- | --- | --- | --- | --- | --- | --- | --- | --- | --- | --- | --- | --- |

tr|A0A3Q1CRB3|A0A3Q1CRB3\_AMPOC

back to list

  

| Protein Coverage
| Supporting Peptides
|

Protein Coverage:

Supporting Peptides:

| Peptide | Uniq | -10lgP | Mass | ppm | m/z | z | RT | Scan | #Spec | Start | End | PTM |
| --- | --- | --- | --- | --- | --- | --- | --- | --- | --- | --- | --- | --- |
| H.LNEDKLKDKINE.L | Y | 41.87 | 1457.7725 | -0.2 | 729.8934 | 2 | 10.04 | 1439 | 1 | 214 | 225 |  |
| N.IDHLNEDKLKDKINE.L | Y | 15.27 | 1822.9424 | 0.3 | 608.6549 | 3 | 10.49 | 1513 | 1 | 211 | 225 |  |
| total 2 peptides |  |  |  |  |  |  |  |  |  |  |  |  |
| --- | --- | --- | --- | --- | --- | --- | --- | --- | --- | --- | --- | --- |

tr|A0A665T7Y2|A0A665T7Y2\_ECHNA

back to list

  

| Protein Coverage
| Supporting Peptides
|

Protein Coverage:

Supporting Peptides:

| Peptide | Uniq | -10lgP | Mass | ppm | m/z | z | RT | Scan | #Spec | Start | End | PTM |
| --- | --- | --- | --- | --- | --- | --- | --- | --- | --- | --- | --- | --- |
| H.LNEDKLKDKINE.L | Y | 41.87 | 1457.7725 | -0.2 | 729.8934 | 2 | 10.04 | 1439 | 1 | 220 | 231 |  |
| N.IDHLNEDKLKDKINE.L | Y | 15.27 | 1822.9424 | 0.3 | 608.6549 | 3 | 10.49 | 1513 | 1 | 217 | 231 |  |
| total 2 peptides |  |  |  |  |  |  |  |  |  |  |  |  |
| --- | --- | --- | --- | --- | --- | --- | --- | --- | --- | --- | --- | --- |

tr|A0A3Q1BEW8|A0A3Q1BEW8\_AMPOC

back to list

  

| Protein Coverage
| Supporting Peptides
|

Protein Coverage:

Supporting Peptides:

| Peptide | Uniq | -10lgP | Mass | ppm | m/z | z | RT | Scan | #Spec | Start | End | PTM |
| --- | --- | --- | --- | --- | --- | --- | --- | --- | --- | --- | --- | --- |
| H.LNEDKLKDKINE.L | Y | 41.87 | 1457.7725 | -0.2 | 729.8934 | 2 | 10.04 | 1439 | 1 | 220 | 231 |  |
| N.IDHLNEDKLKDKINE.L | Y | 15.27 | 1822.9424 | 0.3 | 608.6549 | 3 | 10.49 | 1513 | 1 | 217 | 231 |  |
| total 2 peptides |  |  |  |  |  |  |  |  |  |  |  |  |
| --- | --- | --- | --- | --- | --- | --- | --- | --- | --- | --- | --- | --- |

tr|A0A3P8THB6|A0A3P8THB6\_AMPPE

back to list

  

| Protein Coverage
| Supporting Peptides
|

Protein Coverage:

Supporting Peptides:

| Peptide | Uniq | -10lgP | Mass | ppm | m/z | z | RT | Scan | #Spec | Start | End | PTM |
| --- | --- | --- | --- | --- | --- | --- | --- | --- | --- | --- | --- | --- |
| H.LNEDKLKDKINE.L | Y | 41.87 | 1457.7725 | -0.2 | 729.8934 | 2 | 10.04 | 1439 | 1 | 229 | 240 |  |
| N.IDHLNEDKLKDKINE.L | Y | 15.27 | 1822.9424 | 0.3 | 608.6549 | 3 | 10.49 | 1513 | 1 | 226 | 240 |  |
| total 2 peptides |  |  |  |  |  |  |  |  |  |  |  |  |
| --- | --- | --- | --- | --- | --- | --- | --- | --- | --- | --- | --- | --- |

tr|A0A671U7Q2|A0A671U7Q2\_SPAAU

back to list

  

| Protein Coverage
| Supporting Peptides
|

Protein Coverage:

Supporting Peptides:

| Peptide | Uniq | -10lgP | Mass | ppm | m/z | z | RT | Scan | #Spec | Start | End | PTM |
| --- | --- | --- | --- | --- | --- | --- | --- | --- | --- | --- | --- | --- |
| H.LNEDKLKDKINE.L | Y | 41.87 | 1457.7725 | -0.2 | 729.8934 | 2 | 10.04 | 1439 | 1 | 223 | 234 |  |
| N.IDHLNEDKLKDKINE.L | Y | 15.27 | 1822.9424 | 0.3 | 608.6549 | 3 | 10.49 | 1513 | 1 | 220 | 234 |  |
| total 2 peptides |  |  |  |  |  |  |  |  |  |  |  |  |
| --- | --- | --- | --- | --- | --- | --- | --- | --- | --- | --- | --- | --- |

tr|A0A672YCL7|A0A672YCL7\_9TELE

back to list

  

| Protein Coverage
| Supporting Peptides
|

Protein Coverage:

Supporting Peptides:

| Peptide | Uniq | -10lgP | Mass | ppm | m/z | z | RT | Scan | #Spec | Start | End | PTM |
| --- | --- | --- | --- | --- | --- | --- | --- | --- | --- | --- | --- | --- |
| H.LNEDKLKDKINE.L | Y | 41.87 | 1457.7725 | -0.2 | 729.8934 | 2 | 10.04 | 1439 | 1 | 223 | 234 |  |
| N.IDHLNEDKLKDKINE.L | Y | 15.27 | 1822.9424 | 0.3 | 608.6549 | 3 | 10.49 | 1513 | 1 | 220 | 234 |  |
| total 2 peptides |  |  |  |  |  |  |  |  |  |  |  |  |
| --- | --- | --- | --- | --- | --- | --- | --- | --- | --- | --- | --- | --- |

tr|A0A672YCL2|A0A672YCL2\_9TELE

back to list

  

| Protein Coverage
| Supporting Peptides
|

Protein Coverage:

Supporting Peptides:

| Peptide | Uniq | -10lgP | Mass | ppm | m/z | z | RT | Scan | #Spec | Start | End | PTM |
| --- | --- | --- | --- | --- | --- | --- | --- | --- | --- | --- | --- | --- |
| H.LNEDKLKDKINE.L | Y | 41.87 | 1457.7725 | -0.2 | 729.8934 | 2 | 10.04 | 1439 | 1 | 229 | 240 |  |
| N.IDHLNEDKLKDKINE.L | Y | 15.27 | 1822.9424 | 0.3 | 608.6549 | 3 | 10.49 | 1513 | 1 | 226 | 240 |  |
| total 2 peptides |  |  |  |  |  |  |  |  |  |  |  |  |
| --- | --- | --- | --- | --- | --- | --- | --- | --- | --- | --- | --- | --- |

tr|A0A6P7KCL6|A0A6P7KCL6\_9TELE

back to list

  

| Protein Coverage
| Supporting Peptides
|

Protein Coverage:

Supporting Peptides:

| Peptide | Uniq | -10lgP | Mass | ppm | m/z | z | RT | Scan | #Spec | Start | End | PTM |
| --- | --- | --- | --- | --- | --- | --- | --- | --- | --- | --- | --- | --- |
| H.LNEDKLKDKINE.L | Y | 41.87 | 1457.7725 | -0.2 | 729.8934 | 2 | 10.04 | 1439 | 1 | 233 | 244 |  |
| N.IDHLNEDKLKDKINE.L | Y | 15.27 | 1822.9424 | 0.3 | 608.6549 | 3 | 10.49 | 1513 | 1 | 230 | 244 |  |
| total 2 peptides |  |  |  |  |  |  |  |  |  |  |  |  |
| --- | --- | --- | --- | --- | --- | --- | --- | --- | --- | --- | --- | --- |

tr|A0A6P7KCQ1|A0A6P7KCQ1\_9TELE

back to list

  

| Protein Coverage
| Supporting Peptides
|

Protein Coverage:

Supporting Peptides:

| Peptide | Uniq | -10lgP | Mass | ppm | m/z | z | RT | Scan | #Spec | Start | End | PTM |
| --- | --- | --- | --- | --- | --- | --- | --- | --- | --- | --- | --- | --- |
| H.LNEDKLKDKINE.L | Y | 41.87 | 1457.7725 | -0.2 | 729.8934 | 2 | 10.04 | 1439 | 1 | 236 | 247 |  |
| N.IDHLNEDKLKDKINE.L | Y | 15.27 | 1822.9424 | 0.3 | 608.6549 | 3 | 10.49 | 1513 | 1 | 233 | 247 |  |
| total 2 peptides |  |  |  |  |  |  |  |  |  |  |  |  |
| --- | --- | --- | --- | --- | --- | --- | --- | --- | --- | --- | --- | --- |

tr|A0A671UB90|A0A671UB90\_SPAAU

back to list

  

| Protein Coverage
| Supporting Peptides
|

Protein Coverage:

Supporting Peptides:

| Peptide | Uniq | -10lgP | Mass | ppm | m/z | z | RT | Scan | #Spec | Start | End | PTM |
| --- | --- | --- | --- | --- | --- | --- | --- | --- | --- | --- | --- | --- |
| H.LNEDKLKDKINE.L | Y | 41.87 | 1457.7725 | -0.2 | 729.8934 | 2 | 10.04 | 1439 | 1 | 202 | 213 |  |
| N.IDHLNEDKLKDKINE.L | Y | 15.27 | 1822.9424 | 0.3 | 608.6549 | 3 | 10.49 | 1513 | 1 | 199 | 213 |  |
| total 2 peptides |  |  |  |  |  |  |  |  |  |  |  |  |
| --- | --- | --- | --- | --- | --- | --- | --- | --- | --- | --- | --- | --- |

tr|A0A671U8G8|A0A671U8G8\_SPAAU

back to list

  

| Protein Coverage
| Supporting Peptides
|

Protein Coverage:

Supporting Peptides:

| Peptide | Uniq | -10lgP | Mass | ppm | m/z | z | RT | Scan | #Spec | Start | End | PTM |
| --- | --- | --- | --- | --- | --- | --- | --- | --- | --- | --- | --- | --- |
| H.LNEDKLKDKINE.L | Y | 41.87 | 1457.7725 | -0.2 | 729.8934 | 2 | 10.04 | 1439 | 1 | 205 | 216 |  |
| N.IDHLNEDKLKDKINE.L | Y | 15.27 | 1822.9424 | 0.3 | 608.6549 | 3 | 10.49 | 1513 | 1 | 202 | 216 |  |
| total 2 peptides |  |  |  |  |  |  |  |  |  |  |  |  |
| --- | --- | --- | --- | --- | --- | --- | --- | --- | --- | --- | --- | --- |

tr|A0A671U7M0|A0A671U7M0\_SPAAU

back to list

  

| Protein Coverage
| Supporting Peptides
|

Protein Coverage:

Supporting Peptides:

| Peptide | Uniq | -10lgP | Mass | ppm | m/z | z | RT | Scan | #Spec | Start | End | PTM |
| --- | --- | --- | --- | --- | --- | --- | --- | --- | --- | --- | --- | --- |
| H.LNEDKLKDKINE.L | Y | 41.87 | 1457.7725 | -0.2 | 729.8934 | 2 | 10.04 | 1439 | 1 | 184 | 195 |  |
| N.IDHLNEDKLKDKINE.L | Y | 15.27 | 1822.9424 | 0.3 | 608.6549 | 3 | 10.49 | 1513 | 1 | 181 | 195 |  |
| total 2 peptides |  |  |  |  |  |  |  |  |  |  |  |  |
| --- | --- | --- | --- | --- | --- | --- | --- | --- | --- | --- | --- | --- |

tr|A0A671UB76|A0A671UB76\_SPAAU

back to list

  

| Protein Coverage
| Supporting Peptides
|

Protein Coverage:

Supporting Peptides:

| Peptide | Uniq | -10lgP | Mass | ppm | m/z | z | RT | Scan | #Spec | Start | End | PTM |
| --- | --- | --- | --- | --- | --- | --- | --- | --- | --- | --- | --- | --- |
| H.LNEDKLKDKINE.L | Y | 41.87 | 1457.7725 | -0.2 | 729.8934 | 2 | 10.04 | 1439 | 1 | 204 | 215 |  |
| N.IDHLNEDKLKDKINE.L | Y | 15.27 | 1822.9424 | 0.3 | 608.6549 | 3 | 10.49 | 1513 | 1 | 201 | 215 |  |
| total 2 peptides |  |  |  |  |  |  |  |  |  |  |  |  |
| --- | --- | --- | --- | --- | --- | --- | --- | --- | --- | --- | --- | --- |

tr|A0A6A5F2V4|A0A6A5F2V4\_PERFL

back to list

  

| Protein Coverage
| Supporting Peptides
|

Protein Coverage:

Supporting Peptides:

| Peptide | Uniq | -10lgP | Mass | ppm | m/z | z | RT | Scan | #Spec | Start | End | PTM |
| --- | --- | --- | --- | --- | --- | --- | --- | --- | --- | --- | --- | --- |
| K.AIGNPLPDIVW.L | N | 33.90 | 1193.6444 | -0.2 | 597.8293 | 2 | 15.28 | 2342 | 1 | 622 | 632 |  |
| A.ILPPGPPTPW.I | N | 19.56 | 1073.5909 | 0.5 | 537.8030 | 2 | 13.74 | 2081 | 1 | 7849 | 7858 |  |
| K.VVGRPMPDTYW.F | Y | 15.12 | 1319.6332 | -1.4 | 660.8229 | 2 | 12.61 | 1887 | 1 | 523 | 533 |  |
| total 3 peptides |  |  |  |  |  |  |  |  |  |  |  |  |
| --- | --- | --- | --- | --- | --- | --- | --- | --- | --- | --- | --- | --- |

tr|A0A0S7EPU5|A0A0S7EPU5\_9TELE

back to list

  

| Protein Coverage
| Supporting Peptides
|

Protein Coverage:

Supporting Peptides:

| Peptide | Uniq | -10lgP | Mass | ppm | m/z | z | RT | Scan | #Spec | Start | End | PTM |
| --- | --- | --- | --- | --- | --- | --- | --- | --- | --- | --- | --- | --- |
| K.AIGNPLPDIVW.L | N | 33.90 | 1193.6444 | -0.2 | 597.8293 | 2 | 15.28 | 2342 | 1 | 550 | 560 |  |
| L.IVEELPVKW.L | N | 29.07 | 1111.6277 | -0.4 | 556.8209 | 2 | 13.24 | 1997 | 1 | 3754 | 3762 |  |
| total 2 peptides |  |  |  |  |  |  |  |  |  |  |  |  |
| --- | --- | --- | --- | --- | --- | --- | --- | --- | --- | --- | --- | --- |

tr|A0A0S7ETZ8|A0A0S7ETZ8\_9TELE

back to list

  

| Protein Coverage
| Supporting Peptides
|

Protein Coverage:

Supporting Peptides:

| Peptide | Uniq | -10lgP | Mass | ppm | m/z | z | RT | Scan | #Spec | Start | End | PTM |
| --- | --- | --- | --- | --- | --- | --- | --- | --- | --- | --- | --- | --- |
| K.AIGNPLPDIVW.L | N | 33.90 | 1193.6444 | -0.2 | 597.8293 | 2 | 15.28 | 2342 | 1 | 550 | 560 |  |
| L.IVEELPVKW.L | N | 29.07 | 1111.6277 | -0.4 | 556.8209 | 2 | 13.24 | 1997 | 1 | 3754 | 3762 |  |
| total 2 peptides |  |  |  |  |  |  |  |  |  |  |  |  |
| --- | --- | --- | --- | --- | --- | --- | --- | --- | --- | --- | --- | --- |

tr|A0A0S7EQ56|A0A0S7EQ56\_9TELE

back to list

  

| Protein Coverage
| Supporting Peptides
|

Protein Coverage:

Supporting Peptides:

| Peptide | Uniq | -10lgP | Mass | ppm | m/z | z | RT | Scan | #Spec | Start | End | PTM |
| --- | --- | --- | --- | --- | --- | --- | --- | --- | --- | --- | --- | --- |
| K.AIGNPLPDIVW.L | N | 33.90 | 1193.6444 | -0.2 | 597.8293 | 2 | 15.28 | 2342 | 1 | 550 | 560 |  |
| L.IVEELPVKW.L | N | 29.07 | 1111.6277 | -0.4 | 556.8209 | 2 | 13.24 | 1997 | 1 | 3946 | 3954 |  |
| total 2 peptides |  |  |  |  |  |  |  |  |  |  |  |  |
| --- | --- | --- | --- | --- | --- | --- | --- | --- | --- | --- | --- | --- |

tr|A0A0S7F0D9|A0A0S7F0D9\_9TELE

back to list

  

| Protein Coverage
| Supporting Peptides
|

Protein Coverage:

Supporting Peptides:

| Peptide | Uniq | -10lgP | Mass | ppm | m/z | z | RT | Scan | #Spec | Start | End | PTM |
| --- | --- | --- | --- | --- | --- | --- | --- | --- | --- | --- | --- | --- |
| K.AIGNPLPDIVW.L | N | 33.90 | 1193.6444 | -0.2 | 597.8293 | 2 | 15.28 | 2342 | 1 | 550 | 560 |  |
| L.IVEELPVKW.L | N | 29.07 | 1111.6277 | -0.4 | 556.8209 | 2 | 13.24 | 1997 | 1 | 3946 | 3954 |  |
| total 2 peptides |  |  |  |  |  |  |  |  |  |  |  |  |
| --- | --- | --- | --- | --- | --- | --- | --- | --- | --- | --- | --- | --- |

tr|A0A146UF80|A0A146UF80\_FUNHE

back to list

  

| Protein Coverage
| Supporting Peptides
|

Protein Coverage:

Supporting Peptides:

| Peptide | Uniq | -10lgP | Mass | ppm | m/z | z | RT | Scan | #Spec | Start | End | PTM |
| --- | --- | --- | --- | --- | --- | --- | --- | --- | --- | --- | --- | --- |
| V.ELNIPTGIPLVY.E | Y | 33.55 | 1327.7388 | 0.0 | 664.8766 | 2 | 15.00 | 2292 | 1 | 219 | 230 |  |
| V.ELNIPTGIPLVYE.L | Y | 27.50 | 1456.7812 | -0.4 | 729.3976 | 2 | 14.71 | 2242 | 1 | 219 | 231 |  |
| total 2 peptides |  |  |  |  |  |  |  |  |  |  |  |  |
| --- | --- | --- | --- | --- | --- | --- | --- | --- | --- | --- | --- | --- |

tr|A0A671YMU3|A0A671YMU3\_SPAAU

back to list

  

| Protein Coverage
| Supporting Peptides
|

Protein Coverage:

Supporting Peptides:

| Peptide | Uniq | -10lgP | Mass | ppm | m/z | z | RT | Scan | #Spec | Start | End | PTM |
| --- | --- | --- | --- | --- | --- | --- | --- | --- | --- | --- | --- | --- |
| R.GERGFPGLPGPS.G | N | 28.43 | 1169.5829 | -0.8 | 585.7983 | 2 | 12.09 | 1795 | 1 | 790 | 801 |  |
| G.LRGDKGETGEAGER.G | N | 23.05 | 1473.7172 | -0.3 | 492.2462 | 3 | 8.56 | 1206 | 1 | 860 | 873 |  |
| G.FAGPPGADGQPGAK.G | N | 22.00 | 1268.6149 | -0.4 | 635.3145 | 2 | 10.23 | 1471 | 1 | 647 | 660 |  |
| total 3 peptides |  |  |  |  |  |  |  |  |  |  |  |  |
| --- | --- | --- | --- | --- | --- | --- | --- | --- | --- | --- | --- | --- |

tr|A0A671YMZ1|A0A671YMZ1\_SPAAU

back to list

  

| Protein Coverage
| Supporting Peptides
|

Protein Coverage:

Supporting Peptides:

| Peptide | Uniq | -10lgP | Mass | ppm | m/z | z | RT | Scan | #Spec | Start | End | PTM |
| --- | --- | --- | --- | --- | --- | --- | --- | --- | --- | --- | --- | --- |
| R.GERGFPGLPGPS.G | N | 28.43 | 1169.5829 | -0.8 | 585.7983 | 2 | 12.09 | 1795 | 1 | 816 | 827 |  |
| G.LRGDKGETGEAGER.G | N | 23.05 | 1473.7172 | -0.3 | 492.2462 | 3 | 8.56 | 1206 | 1 | 886 | 899 |  |
| G.FAGPPGADGQPGAK.G | N | 22.00 | 1268.6149 | -0.4 | 635.3145 | 2 | 10.23 | 1471 | 1 | 661 | 674 |  |
| total 3 peptides |  |  |  |  |  |  |  |  |  |  |  |  |
| --- | --- | --- | --- | --- | --- | --- | --- | --- | --- | --- | --- | --- |

tr|A0A671YQ85|A0A671YQ85\_SPAAU

back to list

  

| Protein Coverage
| Supporting Peptides
|

Protein Coverage:

Supporting Peptides:

| Peptide | Uniq | -10lgP | Mass | ppm | m/z | z | RT | Scan | #Spec | Start | End | PTM |
| --- | --- | --- | --- | --- | --- | --- | --- | --- | --- | --- | --- | --- |
| R.GERGFPGLPGPS.G | N | 28.43 | 1169.5829 | -0.8 | 585.7983 | 2 | 12.09 | 1795 | 1 | 816 | 827 |  |
| G.LRGDKGETGEAGER.G | N | 23.05 | 1473.7172 | -0.3 | 492.2462 | 3 | 8.56 | 1206 | 1 | 886 | 899 |  |
| G.FAGPPGADGQPGAK.G | N | 22.00 | 1268.6149 | -0.4 | 635.3145 | 2 | 10.23 | 1471 | 1 | 671 | 684 |  |
| total 3 peptides |  |  |  |  |  |  |  |  |  |  |  |  |
| --- | --- | --- | --- | --- | --- | --- | --- | --- | --- | --- | --- | --- |

tr|A0A671YMZ6|A0A671YMZ6\_SPAAU

back to list

  

| Protein Coverage
| Supporting Peptides
|

Protein Coverage:

Supporting Peptides:

| Peptide | Uniq | -10lgP | Mass | ppm | m/z | z | RT | Scan | #Spec | Start | End | PTM |
| --- | --- | --- | --- | --- | --- | --- | --- | --- | --- | --- | --- | --- |
| R.GERGFPGLPGPS.G | N | 28.43 | 1169.5829 | -0.8 | 585.7983 | 2 | 12.09 | 1795 | 1 | 833 | 844 |  |
| G.LRGDKGETGEAGER.G | N | 23.05 | 1473.7172 | -0.3 | 492.2462 | 3 | 8.56 | 1206 | 1 | 903 | 916 |  |
| G.FAGPPGADGQPGAK.G | N | 22.00 | 1268.6149 | -0.4 | 635.3145 | 2 | 10.23 | 1471 | 1 | 690 | 703 |  |
| total 3 peptides |  |  |  |  |  |  |  |  |  |  |  |  |
| --- | --- | --- | --- | --- | --- | --- | --- | --- | --- | --- | --- | --- |

tr|A0A671YNQ4|A0A671YNQ4\_SPAAU

back to list

  

| Protein Coverage
| Supporting Peptides
|

Protein Coverage:

Supporting Peptides:

| Peptide | Uniq | -10lgP | Mass | ppm | m/z | z | RT | Scan | #Spec | Start | End | PTM |
| --- | --- | --- | --- | --- | --- | --- | --- | --- | --- | --- | --- | --- |
| R.GERGFPGLPGPS.G | N | 28.43 | 1169.5829 | -0.8 | 585.7983 | 2 | 12.09 | 1795 | 1 | 833 | 844 |  |
| G.LRGDKGETGEAGER.G | N | 23.05 | 1473.7172 | -0.3 | 492.2462 | 3 | 8.56 | 1206 | 1 | 903 | 916 |  |
| G.FAGPPGADGQPGAK.G | N | 22.00 | 1268.6149 | -0.4 | 635.3145 | 2 | 10.23 | 1471 | 1 | 690 | 703 |  |
| total 3 peptides |  |  |  |  |  |  |  |  |  |  |  |  |
| --- | --- | --- | --- | --- | --- | --- | --- | --- | --- | --- | --- | --- |

tr|A0A671YN33|A0A671YN33\_SPAAU

back to list

  

| Protein Coverage
| Supporting Peptides
|

Protein Coverage:

Supporting Peptides:

| Peptide | Uniq | -10lgP | Mass | ppm | m/z | z | RT | Scan | #Spec | Start | End | PTM |
| --- | --- | --- | --- | --- | --- | --- | --- | --- | --- | --- | --- | --- |
| R.GERGFPGLPGPS.G | N | 28.43 | 1169.5829 | -0.8 | 585.7983 | 2 | 12.09 | 1795 | 1 | 892 | 903 |  |
| G.LRGDKGETGEAGER.G | N | 23.05 | 1473.7172 | -0.3 | 492.2462 | 3 | 8.56 | 1206 | 1 | 962 | 975 |  |
| G.FAGPPGADGQPGAK.G | N | 22.00 | 1268.6149 | -0.4 | 635.3145 | 2 | 10.23 | 1471 | 1 | 747 | 760 |  |
| total 3 peptides |  |  |  |  |  |  |  |  |  |  |  |  |
| --- | --- | --- | --- | --- | --- | --- | --- | --- | --- | --- | --- | --- |

tr|A0A671YQA1|A0A671YQA1\_SPAAU

back to list

  

| Protein Coverage
| Supporting Peptides
|

Protein Coverage:

Supporting Peptides:

| Peptide | Uniq | -10lgP | Mass | ppm | m/z | z | RT | Scan | #Spec | Start | End | PTM |
| --- | --- | --- | --- | --- | --- | --- | --- | --- | --- | --- | --- | --- |
| R.GERGFPGLPGPS.G | N | 28.43 | 1169.5829 | -0.8 | 585.7983 | 2 | 12.09 | 1795 | 1 | 900 | 911 |  |
| G.LRGDKGETGEAGER.G | N | 23.05 | 1473.7172 | -0.3 | 492.2462 | 3 | 8.56 | 1206 | 1 | 970 | 983 |  |
| G.FAGPPGADGQPGAK.G | N | 22.00 | 1268.6149 | -0.4 | 635.3145 | 2 | 10.23 | 1471 | 1 | 757 | 770 |  |
| total 3 peptides |  |  |  |  |  |  |  |  |  |  |  |  |
| --- | --- | --- | --- | --- | --- | --- | --- | --- | --- | --- | --- | --- |

tr|A0A671YNR4|A0A671YNR4\_SPAAU

back to list

  

| Protein Coverage
| Supporting Peptides
|

Protein Coverage:

Supporting Peptides:

| Peptide | Uniq | -10lgP | Mass | ppm | m/z | z | RT | Scan | #Spec | Start | End | PTM |
| --- | --- | --- | --- | --- | --- | --- | --- | --- | --- | --- | --- | --- |
| R.GERGFPGLPGPS.G | N | 28.43 | 1169.5829 | -0.8 | 585.7983 | 2 | 12.09 | 1795 | 1 | 921 | 932 |  |
| G.LRGDKGETGEAGER.G | N | 23.05 | 1473.7172 | -0.3 | 492.2462 | 3 | 8.56 | 1206 | 1 | 991 | 1004 |  |
| G.FAGPPGADGQPGAK.G | N | 22.00 | 1268.6149 | -0.4 | 635.3145 | 2 | 10.23 | 1471 | 1 | 778 | 791 |  |
| total 3 peptides |  |  |  |  |  |  |  |  |  |  |  |  |
| --- | --- | --- | --- | --- | --- | --- | --- | --- | --- | --- | --- | --- |

tr|A0A671YPG6|A0A671YPG6\_SPAAU

back to list

  

| Protein Coverage
| Supporting Peptides
|

Protein Coverage:

Supporting Peptides:

| Peptide | Uniq | -10lgP | Mass | ppm | m/z | z | RT | Scan | #Spec | Start | End | PTM |
| --- | --- | --- | --- | --- | --- | --- | --- | --- | --- | --- | --- | --- |
| R.GERGFPGLPGPS.G | N | 28.43 | 1169.5829 | -0.8 | 585.7983 | 2 | 12.09 | 1795 | 1 | 963 | 974 |  |
| G.LRGDKGETGEAGER.G | N | 23.05 | 1473.7172 | -0.3 | 492.2462 | 3 | 8.56 | 1206 | 1 | 1033 | 1046 |  |
| G.FAGPPGADGQPGAK.G | N | 22.00 | 1268.6149 | -0.4 | 635.3145 | 2 | 10.23 | 1471 | 1 | 817 | 830 |  |
| total 3 peptides |  |  |  |  |  |  |  |  |  |  |  |  |
| --- | --- | --- | --- | --- | --- | --- | --- | --- | --- | --- | --- | --- |

tr|A0A671YMN4|A0A671YMN4\_SPAAU

back to list

  

| Protein Coverage
| Supporting Peptides
|

Protein Coverage:

Supporting Peptides:

| Peptide | Uniq | -10lgP | Mass | ppm | m/z | z | RT | Scan | #Spec | Start | End | PTM |
| --- | --- | --- | --- | --- | --- | --- | --- | --- | --- | --- | --- | --- |
| R.GERGFPGLPGPS.G | N | 28.43 | 1169.5829 | -0.8 | 585.7983 | 2 | 12.09 | 1795 | 1 | 950 | 961 |  |
| G.LRGDKGETGEAGER.G | N | 23.05 | 1473.7172 | -0.3 | 492.2462 | 3 | 8.56 | 1206 | 1 | 1074 | 1087 |  |
| G.FAGPPGADGQPGAK.G | N | 22.00 | 1268.6149 | -0.4 | 635.3145 | 2 | 10.23 | 1471 | 1 | 795 | 808 |  |
| total 3 peptides |  |  |  |  |  |  |  |  |  |  |  |  |
| --- | --- | --- | --- | --- | --- | --- | --- | --- | --- | --- | --- | --- |

tr|A0A3Q3RR05|A0A3Q3RR05\_9TELE

back to list

  

| Protein Coverage
| Supporting Peptides
|

Protein Coverage:

Supporting Peptides:

| Peptide | Uniq | -10lgP | Mass | ppm | m/z | z | RT | Scan | #Spec | Start | End | PTM |
| --- | --- | --- | --- | --- | --- | --- | --- | --- | --- | --- | --- | --- |
| R.GERGFPGLPGPS.G | N | 28.43 | 1169.5829 | -0.8 | 585.7983 | 2 | 12.09 | 1795 | 1 | 752 | 763 |  |
| G.LRGDKGETGEAGER.G | N | 23.05 | 1473.7172 | -0.3 | 492.2462 | 3 | 8.56 | 1206 | 1 | 822 | 835 |  |
| G.FAGPPGADGQPGAK.G | N | 22.00 | 1268.6149 | -0.4 | 635.3145 | 2 | 10.23 | 1471 | 1 | 597 | 610 |  |
| total 3 peptides |  |  |  |  |  |  |  |  |  |  |  |  |
| --- | --- | --- | --- | --- | --- | --- | --- | --- | --- | --- | --- | --- |

tr|A0A0F8B7E5|A0A0F8B7E5\_LARCR

back to list

  

| Protein Coverage
| Supporting Peptides
|

Protein Coverage:

Supporting Peptides:

| Peptide | Uniq | -10lgP | Mass | ppm | m/z | z | RT | Scan | #Spec | Start | End | PTM |
| --- | --- | --- | --- | --- | --- | --- | --- | --- | --- | --- | --- | --- |
| R.GERGFPGLPGPS.G | N | 28.43 | 1169.5829 | -0.8 | 585.7983 | 2 | 12.09 | 1795 | 1 | 916 | 927 |  |
| G.LRGDKGETGEAGER.G | N | 23.05 | 1473.7172 | -0.3 | 492.2462 | 3 | 8.56 | 1206 | 1 | 1040 | 1053 |  |
| G.FAGPPGADGQPGAK.G | N | 22.00 | 1268.6149 | -0.4 | 635.3145 | 2 | 10.23 | 1471 | 1 | 761 | 774 |  |
| total 3 peptides |  |  |  |  |  |  |  |  |  |  |  |  |
| --- | --- | --- | --- | --- | --- | --- | --- | --- | --- | --- | --- | --- |

tr|A0A6G0HX74|A0A6G0HX74\_LARCR

back to list

  

| Protein Coverage
| Supporting Peptides
|

Protein Coverage:

Supporting Peptides:

| Peptide | Uniq | -10lgP | Mass | ppm | m/z | z | RT | Scan | #Spec | Start | End | PTM |
| --- | --- | --- | --- | --- | --- | --- | --- | --- | --- | --- | --- | --- |
| R.GERGFPGLPGPS.G | N | 28.43 | 1169.5829 | -0.8 | 585.7983 | 2 | 12.09 | 1795 | 1 | 952 | 963 |  |
| G.LRGDKGETGEAGER.G | N | 23.05 | 1473.7172 | -0.3 | 492.2462 | 3 | 8.56 | 1206 | 1 | 1076 | 1089 |  |
| G.FAGPPGADGQPGAK.G | N | 22.00 | 1268.6149 | -0.4 | 635.3145 | 2 | 10.23 | 1471 | 1 | 797 | 810 |  |
| total 3 peptides |  |  |  |  |  |  |  |  |  |  |  |  |
| --- | --- | --- | --- | --- | --- | --- | --- | --- | --- | --- | --- | --- |

tr|A0A5J6XWD3|A0A5J6XWD3\_NIBCO

back to list

  

| Protein Coverage
| Supporting Peptides
|

Protein Coverage:

Supporting Peptides:

| Peptide | Uniq | -10lgP | Mass | ppm | m/z | z | RT | Scan | #Spec | Start | End | PTM |
| --- | --- | --- | --- | --- | --- | --- | --- | --- | --- | --- | --- | --- |
| R.GERGFPGLPGPS.G | N | 28.43 | 1169.5829 | -0.8 | 585.7983 | 2 | 12.09 | 1795 | 1 | 952 | 963 |  |
| G.LRGDKGETGEAGER.G | N | 23.05 | 1473.7172 | -0.3 | 492.2462 | 3 | 8.56 | 1206 | 1 | 1076 | 1089 |  |
| G.FAGPPGADGQPGAK.G | N | 22.00 | 1268.6149 | -0.4 | 635.3145 | 2 | 10.23 | 1471 | 1 | 797 | 810 |  |
| total 3 peptides |  |  |  |  |  |  |  |  |  |  |  |  |
| --- | --- | --- | --- | --- | --- | --- | --- | --- | --- | --- | --- | --- |

tr|A0A7N8XDE6|A0A7N8XDE6\_9TELE

back to list

  

| Protein Coverage
| Supporting Peptides
|

Protein Coverage:

Supporting Peptides:

| Peptide | Uniq | -10lgP | Mass | ppm | m/z | z | RT | Scan | #Spec | Start | End | PTM |
| --- | --- | --- | --- | --- | --- | --- | --- | --- | --- | --- | --- | --- |
| R.GERGFPGLPGPS.G | N | 28.43 | 1169.5829 | -0.8 | 585.7983 | 2 | 12.09 | 1795 | 1 | 754 | 765 |  |
| G.LRGDKGETGEAGER.G | N | 23.05 | 1473.7172 | -0.3 | 492.2462 | 3 | 8.56 | 1206 | 1 | 824 | 837 |  |
| G.FAGPPGADGQPGAK.G | N | 22.00 | 1268.6149 | -0.4 | 635.3145 | 2 | 10.23 | 1471 | 1 | 599 | 612 |  |
| total 3 peptides |  |  |  |  |  |  |  |  |  |  |  |  |
| --- | --- | --- | --- | --- | --- | --- | --- | --- | --- | --- | --- | --- |

tr|A0A7N8WW01|A0A7N8WW01\_9TELE

back to list

  

| Protein Coverage
| Supporting Peptides
|

Protein Coverage:

Supporting Peptides:

| Peptide | Uniq | -10lgP | Mass | ppm | m/z | z | RT | Scan | #Spec | Start | End | PTM |
| --- | --- | --- | --- | --- | --- | --- | --- | --- | --- | --- | --- | --- |
| R.GERGFPGLPGPS.G | N | 28.43 | 1169.5829 | -0.8 | 585.7983 | 2 | 12.09 | 1795 | 1 | 821 | 832 |  |
| G.LRGDKGETGEAGER.G | N | 23.05 | 1473.7172 | -0.3 | 492.2462 | 3 | 8.56 | 1206 | 1 | 891 | 904 |  |
| G.FAGPPGADGQPGAK.G | N | 22.00 | 1268.6149 | -0.4 | 635.3145 | 2 | 10.23 | 1471 | 1 | 666 | 679 |  |
| total 3 peptides |  |  |  |  |  |  |  |  |  |  |  |  |
| --- | --- | --- | --- | --- | --- | --- | --- | --- | --- | --- | --- | --- |

tr|A0A7N8XAN7|A0A7N8XAN7\_9TELE

back to list

  

| Protein Coverage
| Supporting Peptides
|

Protein Coverage:

Supporting Peptides:

| Peptide | Uniq | -10lgP | Mass | ppm | m/z | z | RT | Scan | #Spec | Start | End | PTM |
| --- | --- | --- | --- | --- | --- | --- | --- | --- | --- | --- | --- | --- |
| R.GERGFPGLPGPS.G | N | 28.43 | 1169.5829 | -0.8 | 585.7983 | 2 | 12.09 | 1795 | 1 | 862 | 873 |  |
| G.LRGDKGETGEAGER.G | N | 23.05 | 1473.7172 | -0.3 | 492.2462 | 3 | 8.56 | 1206 | 1 | 932 | 945 |  |
| G.FAGPPGADGQPGAK.G | N | 22.00 | 1268.6149 | -0.4 | 635.3145 | 2 | 10.23 | 1471 | 1 | 707 | 720 |  |
| total 3 peptides |  |  |  |  |  |  |  |  |  |  |  |  |
| --- | --- | --- | --- | --- | --- | --- | --- | --- | --- | --- | --- | --- |

tr|A0A4W4EA72|A0A4W4EA72\_ELEEL

back to list

  

| Protein Coverage
| Supporting Peptides
|

Protein Coverage:

Supporting Peptides:

| Peptide | Uniq | -10lgP | Mass | ppm | m/z | z | RT | Scan | #Spec | Start | End | PTM |
| --- | --- | --- | --- | --- | --- | --- | --- | --- | --- | --- | --- | --- |
| F.IEEEELKLF.L | N | 30.75 | 1148.5964 | -0.3 | 575.3053 | 2 | 13.44 | 2031 | 1 | 59 | 67 |  |
| T.VLDRDKSGFIEEEELK.L | Y | 28.30 | 1905.9683 | -20.3 | 953.9720 | 2 | 11.67 | 1719 | 2 | 50 | 65 |  |
| total 2 peptides |  |  |  |  |  |  |  |  |  |  |  |  |
| --- | --- | --- | --- | --- | --- | --- | --- | --- | --- | --- | --- | --- |

tr|A0A6F9CEX9|A0A6F9CEX9\_9TELE

back to list

  

| Protein Coverage
| Supporting Peptides
|

Protein Coverage:

Supporting Peptides:

| Peptide | Uniq | -10lgP | Mass | ppm | m/z | z | RT | Scan | #Spec | Start | End | PTM |
| --- | --- | --- | --- | --- | --- | --- | --- | --- | --- | --- | --- | --- |
| A.LDPIDAPGKPDVIS.I | Y | 29.78 | 1435.7559 | -0.9 | 718.8846 | 2 | 11.96 | 1772 | 1 | 8748 | 8761 |  |
| L.IVEELPVKW.V | N | 29.07 | 1111.6277 | -0.4 | 556.8209 | 2 | 13.24 | 1997 | 1 | 577 | 585 |  |
| total 2 peptides |  |  |  |  |  |  |  |  |  |  |  |  |
| --- | --- | --- | --- | --- | --- | --- | --- | --- | --- | --- | --- | --- |

tr|A0A668V0Q0|A0A668V0Q0\_OREAU

back to list

  

| Protein Coverage
| Supporting Peptides
|

Protein Coverage:

Supporting Peptides:

| Peptide | Uniq | -10lgP | Mass | ppm | m/z | z | RT | Scan | #Spec | Start | End | PTM |
| --- | --- | --- | --- | --- | --- | --- | --- | --- | --- | --- | --- | --- |
| R.VIISAPSADAPMF.V | Y | 36.31 | 1317.6638 | 0.4 | 659.8394 | 2 | 13.94 | 2114 | 1 | 126 | 138 |  |
| F.HERDPAN.I | N | 15.89 | 837.3729 | -0.2 | 419.6937 | 2 | 7.17 | 946 | 3 | 85 | 91 |  |
| total 2 peptides |  |  |  |  |  |  |  |  |  |  |  |  |
| --- | --- | --- | --- | --- | --- | --- | --- | --- | --- | --- | --- | --- |

tr|Q8AWX8|Q8AWX8\_GADMO

back to list

  

| Protein Coverage
| Supporting Peptides
|

Protein Coverage:

Supporting Peptides:

| Peptide | Uniq | -10lgP | Mass | ppm | m/z | z | RT | Scan | #Spec | Start | End | PTM |
| --- | --- | --- | --- | --- | --- | --- | --- | --- | --- | --- | --- | --- |
| R.VIISAPSADAPMF.V | Y | 36.31 | 1317.6638 | 0.4 | 659.8394 | 2 | 13.94 | 2114 | 1 | 117 | 129 |  |
| F.HERDPAN.I | N | 15.89 | 837.3729 | -0.2 | 419.6937 | 2 | 7.17 | 946 | 3 | 76 | 82 |  |
| total 2 peptides |  |  |  |  |  |  |  |  |  |  |  |  |
| --- | --- | --- | --- | --- | --- | --- | --- | --- | --- | --- | --- | --- |

tr|A0A6P7KWB3|A0A6P7KWB3\_BETSP

back to list

  

| Protein Coverage
| Supporting Peptides
|

Protein Coverage:

Supporting Peptides:

| Peptide | Uniq | -10lgP | Mass | ppm | m/z | z | RT | Scan | #Spec | Start | End | PTM |
| --- | --- | --- | --- | --- | --- | --- | --- | --- | --- | --- | --- | --- |
| R.VIISAPSADAPMF.V | Y | 36.31 | 1317.6638 | 0.4 | 659.8394 | 2 | 13.94 | 2114 | 1 | 117 | 129 |  |
| F.HERDPAN.I | N | 15.89 | 837.3729 | -0.2 | 419.6937 | 2 | 7.17 | 946 | 3 | 76 | 82 |  |
| total 2 peptides |  |  |  |  |  |  |  |  |  |  |  |  |
| --- | --- | --- | --- | --- | --- | --- | --- | --- | --- | --- | --- | --- |

tr|Q4VKA7|Q4VKA7\_PARLE

back to list

  

| Protein Coverage
| Supporting Peptides
|

Protein Coverage:

Supporting Peptides:

| Peptide | Uniq | -10lgP | Mass | ppm | m/z | z | RT | Scan | #Spec | Start | End | PTM |
| --- | --- | --- | --- | --- | --- | --- | --- | --- | --- | --- | --- | --- |
| K.VIISAPSADAPMF.V | Y | 36.31 | 1317.6638 | 0.4 | 659.8394 | 2 | 13.94 | 2114 | 1 | 72 | 84 |  |
| F.HERDPAN.I | N | 15.89 | 837.3729 | -0.2 | 419.6937 | 2 | 7.17 | 946 | 3 | 31 | 37 |  |
| total 2 peptides |  |  |  |  |  |  |  |  |  |  |  |  |
| --- | --- | --- | --- | --- | --- | --- | --- | --- | --- | --- | --- | --- |

tr|A0A4W6CL45|A0A4W6CL45\_LATCA

back to list

  

| Protein Coverage
| Supporting Peptides
|

Protein Coverage:

Supporting Peptides:

| Peptide | Uniq | -10lgP | Mass | ppm | m/z | z | RT | Scan | #Spec | Start | End | PTM |
| --- | --- | --- | --- | --- | --- | --- | --- | --- | --- | --- | --- | --- |
| F.MDDPVVIPGKPY.S | N | 35.25 | 1329.6638 | -0.8 | 665.8386 | 2 | 12.28 | 1829 | 1 | 225 | 236 |  |
| S.IVWIDPDDFPL.L | N | 15.72 | 1328.6652 | -0.2 | 665.3397 | 2 | 16.16 | 2489 | 1 | 307 | 317 |  |
| total 2 peptides |  |  |  |  |  |  |  |  |  |  |  |  |
| --- | --- | --- | --- | --- | --- | --- | --- | --- | --- | --- | --- | --- |

tr|A0A671XZM8|A0A671XZM8\_SPAAU

back to list

  

| Protein Coverage
| Supporting Peptides
|

Protein Coverage:

Supporting Peptides:

| Peptide | Uniq | -10lgP | Mass | ppm | m/z | z | RT | Scan | #Spec | Start | End | PTM |
| --- | --- | --- | --- | --- | --- | --- | --- | --- | --- | --- | --- | --- |
| F.MDDPVVIPGKPY.T | N | 35.25 | 1329.6638 | -0.8 | 665.8386 | 2 | 12.28 | 1829 | 1 | 225 | 236 |  |
| S.IVWIDPDDFPL.L | N | 15.72 | 1328.6652 | -0.2 | 665.3397 | 2 | 16.16 | 2489 | 1 | 307 | 317 |  |
| total 2 peptides |  |  |  |  |  |  |  |  |  |  |  |  |
| --- | --- | --- | --- | --- | --- | --- | --- | --- | --- | --- | --- | --- |

tr|A0A671Y6A5|A0A671Y6A5\_SPAAU

back to list

  

| Protein Coverage
| Supporting Peptides
|

Protein Coverage:

Supporting Peptides:

| Peptide | Uniq | -10lgP | Mass | ppm | m/z | z | RT | Scan | #Spec | Start | End | PTM |
| --- | --- | --- | --- | --- | --- | --- | --- | --- | --- | --- | --- | --- |
| F.MDDPVVIPGKPY.T | N | 35.25 | 1329.6638 | -0.8 | 665.8386 | 2 | 12.28 | 1829 | 1 | 211 | 222 |  |
| S.IVWIDPDDFPL.L | N | 15.72 | 1328.6652 | -0.2 | 665.3397 | 2 | 16.16 | 2489 | 1 | 284 | 294 |  |
| total 2 peptides |  |  |  |  |  |  |  |  |  |  |  |  |
| --- | --- | --- | --- | --- | --- | --- | --- | --- | --- | --- | --- | --- |

tr|A0A672HWD2|A0A672HWD2\_SALFA

back to list

  

| Protein Coverage
| Supporting Peptides
|

Protein Coverage:

Supporting Peptides:

| Peptide | Uniq | -10lgP | Mass | ppm | m/z | z | RT | Scan | #Spec | Start | End | PTM |
| --- | --- | --- | --- | --- | --- | --- | --- | --- | --- | --- | --- | --- |
| F.MDDPVVIPGKPY.T | N | 35.25 | 1329.6638 | -0.8 | 665.8386 | 2 | 12.28 | 1829 | 1 | 211 | 222 |  |
| S.IVWIDPDDFPL.L | N | 15.72 | 1328.6652 | -0.2 | 665.3397 | 2 | 16.16 | 2489 | 1 | 293 | 303 |  |
| total 2 peptides |  |  |  |  |  |  |  |  |  |  |  |  |
| --- | --- | --- | --- | --- | --- | --- | --- | --- | --- | --- | --- | --- |

tr|A0A672HWL2|A0A672HWL2\_SALFA

back to list

  

| Protein Coverage
| Supporting Peptides
|

Protein Coverage:

Supporting Peptides:

| Peptide | Uniq | -10lgP | Mass | ppm | m/z | z | RT | Scan | #Spec | Start | End | PTM |
| --- | --- | --- | --- | --- | --- | --- | --- | --- | --- | --- | --- | --- |
| F.MDDPVVIPGKPY.T | N | 35.25 | 1329.6638 | -0.8 | 665.8386 | 2 | 12.28 | 1829 | 1 | 218 | 229 |  |
| S.IVWIDPDDFPL.L | N | 15.72 | 1328.6652 | -0.2 | 665.3397 | 2 | 16.16 | 2489 | 1 | 300 | 310 |  |
| total 2 peptides |  |  |  |  |  |  |  |  |  |  |  |  |
| --- | --- | --- | --- | --- | --- | --- | --- | --- | --- | --- | --- | --- |

tr|A0A671XZM4|A0A671XZM4\_SPAAU

back to list

  

| Protein Coverage
| Supporting Peptides
|

Protein Coverage:

Supporting Peptides:

| Peptide | Uniq | -10lgP | Mass | ppm | m/z | z | RT | Scan | #Spec | Start | End | PTM |
| --- | --- | --- | --- | --- | --- | --- | --- | --- | --- | --- | --- | --- |
| F.MDDPVVIPGKPY.T | N | 35.25 | 1329.6638 | -0.8 | 665.8386 | 2 | 12.28 | 1829 | 1 | 224 | 235 |  |
| S.IVWIDPDDFPL.L | N | 15.72 | 1328.6652 | -0.2 | 665.3397 | 2 | 16.16 | 2489 | 1 | 297 | 307 |  |
| total 2 peptides |  |  |  |  |  |  |  |  |  |  |  |  |
| --- | --- | --- | --- | --- | --- | --- | --- | --- | --- | --- | --- | --- |

tr|A0A672HWC2|A0A672HWC2\_SALFA

back to list

  

| Protein Coverage
| Supporting Peptides
|

Protein Coverage:

Supporting Peptides:

| Peptide | Uniq | -10lgP | Mass | ppm | m/z | z | RT | Scan | #Spec | Start | End | PTM |
| --- | --- | --- | --- | --- | --- | --- | --- | --- | --- | --- | --- | --- |
| F.MDDPVVIPGKPY.T | N | 35.25 | 1329.6638 | -0.8 | 665.8386 | 2 | 12.28 | 1829 | 1 | 227 | 238 |  |
| S.IVWIDPDDFPL.L | N | 15.72 | 1328.6652 | -0.2 | 665.3397 | 2 | 16.16 | 2489 | 1 | 309 | 319 |  |
| total 2 peptides |  |  |  |  |  |  |  |  |  |  |  |  |
| --- | --- | --- | --- | --- | --- | --- | --- | --- | --- | --- | --- | --- |

tr|A0A672HXH8|A0A672HXH8\_SALFA

back to list

  

| Protein Coverage
| Supporting Peptides
|

Protein Coverage:

Supporting Peptides:

| Peptide | Uniq | -10lgP | Mass | ppm | m/z | z | RT | Scan | #Spec | Start | End | PTM |
| --- | --- | --- | --- | --- | --- | --- | --- | --- | --- | --- | --- | --- |
| F.MDDPVVIPGKPY.T | N | 35.25 | 1329.6638 | -0.8 | 665.8386 | 2 | 12.28 | 1829 | 1 | 225 | 236 |  |
| S.IVWIDPDDFPL.L | N | 15.72 | 1328.6652 | -0.2 | 665.3397 | 2 | 16.16 | 2489 | 1 | 310 | 320 |  |
| total 2 peptides |  |  |  |  |  |  |  |  |  |  |  |  |
| --- | --- | --- | --- | --- | --- | --- | --- | --- | --- | --- | --- | --- |

tr|A0A672HWL0|A0A672HWL0\_SALFA

back to list

  

| Protein Coverage
| Supporting Peptides
|

Protein Coverage:

Supporting Peptides:

| Peptide | Uniq | -10lgP | Mass | ppm | m/z | z | RT | Scan | #Spec | Start | End | PTM |
| --- | --- | --- | --- | --- | --- | --- | --- | --- | --- | --- | --- | --- |
| F.MDDPVVIPGKPY.T | N | 35.25 | 1329.6638 | -0.8 | 665.8386 | 2 | 12.28 | 1829 | 1 | 228 | 239 |  |
| S.IVWIDPDDFPL.L | N | 15.72 | 1328.6652 | -0.2 | 665.3397 | 2 | 16.16 | 2489 | 1 | 310 | 320 |  |
| total 2 peptides |  |  |  |  |  |  |  |  |  |  |  |  |
| --- | --- | --- | --- | --- | --- | --- | --- | --- | --- | --- | --- | --- |

tr|A0A672HXF4|A0A672HXF4\_SALFA

back to list

  

| Protein Coverage
| Supporting Peptides
|

Protein Coverage:

Supporting Peptides:

| Peptide | Uniq | -10lgP | Mass | ppm | m/z | z | RT | Scan | #Spec | Start | End | PTM |
| --- | --- | --- | --- | --- | --- | --- | --- | --- | --- | --- | --- | --- |
| F.MDDPVVIPGKPY.T | N | 35.25 | 1329.6638 | -0.8 | 665.8386 | 2 | 12.28 | 1829 | 1 | 229 | 240 |  |
| S.IVWIDPDDFPL.L | N | 15.72 | 1328.6652 | -0.2 | 665.3397 | 2 | 16.16 | 2489 | 1 | 311 | 321 |  |
| total 2 peptides |  |  |  |  |  |  |  |  |  |  |  |  |
| --- | --- | --- | --- | --- | --- | --- | --- | --- | --- | --- | --- | --- |

tr|A0A671XZK3|A0A671XZK3\_SPAAU

back to list

  

| Protein Coverage
| Supporting Peptides
|

Protein Coverage:

Supporting Peptides:

| Peptide | Uniq | -10lgP | Mass | ppm | m/z | z | RT | Scan | #Spec | Start | End | PTM |
| --- | --- | --- | --- | --- | --- | --- | --- | --- | --- | --- | --- | --- |
| F.MDDPVVIPGKPY.T | N | 35.25 | 1329.6638 | -0.8 | 665.8386 | 2 | 12.28 | 1829 | 1 | 225 | 236 |  |
| S.IVWIDPDDFPL.L | N | 15.72 | 1328.6652 | -0.2 | 665.3397 | 2 | 16.16 | 2489 | 1 | 307 | 317 |  |
| total 2 peptides |  |  |  |  |  |  |  |  |  |  |  |  |
| --- | --- | --- | --- | --- | --- | --- | --- | --- | --- | --- | --- | --- |

tr|A0A672HWP0|A0A672HWP0\_SALFA

back to list

  

| Protein Coverage
| Supporting Peptides
|

Protein Coverage:

Supporting Peptides:

| Peptide | Uniq | -10lgP | Mass | ppm | m/z | z | RT | Scan | #Spec | Start | End | PTM |
| --- | --- | --- | --- | --- | --- | --- | --- | --- | --- | --- | --- | --- |
| F.MDDPVVIPGKPY.T | N | 35.25 | 1329.6638 | -0.8 | 665.8386 | 2 | 12.28 | 1829 | 1 | 233 | 244 |  |
| S.IVWIDPDDFPL.L | N | 15.72 | 1328.6652 | -0.2 | 665.3397 | 2 | 16.16 | 2489 | 1 | 315 | 325 |  |
| total 2 peptides |  |  |  |  |  |  |  |  |  |  |  |  |
| --- | --- | --- | --- | --- | --- | --- | --- | --- | --- | --- | --- | --- |

tr|A0A4W6CL16|A0A4W6CL16\_LATCA

back to list

  

| Protein Coverage
| Supporting Peptides
|

Protein Coverage:

Supporting Peptides:

| Peptide | Uniq | -10lgP | Mass | ppm | m/z | z | RT | Scan | #Spec | Start | End | PTM |
| --- | --- | --- | --- | --- | --- | --- | --- | --- | --- | --- | --- | --- |
| F.MDDPVVIPGKPY.S | N | 35.25 | 1329.6638 | -0.8 | 665.8386 | 2 | 12.28 | 1829 | 1 | 223 | 234 |  |
| S.IVWIDPDDFPL.L | N | 15.72 | 1328.6652 | -0.2 | 665.3397 | 2 | 16.16 | 2489 | 1 | 305 | 315 |  |
| total 2 peptides |  |  |  |  |  |  |  |  |  |  |  |  |
| --- | --- | --- | --- | --- | --- | --- | --- | --- | --- | --- | --- | --- |

tr|A0A4W6CL41|A0A4W6CL41\_LATCA

back to list

  

| Protein Coverage
| Supporting Peptides
|

Protein Coverage:

Supporting Peptides:

| Peptide | Uniq | -10lgP | Mass | ppm | m/z | z | RT | Scan | #Spec | Start | End | PTM |
| --- | --- | --- | --- | --- | --- | --- | --- | --- | --- | --- | --- | --- |
| F.MDDPVVIPGKPY.S | N | 35.25 | 1329.6638 | -0.8 | 665.8386 | 2 | 12.28 | 1829 | 1 | 225 | 236 |  |
| S.IVWIDPDDFPL.L | N | 15.72 | 1328.6652 | -0.2 | 665.3397 | 2 | 16.16 | 2489 | 1 | 307 | 317 |  |
| total 2 peptides |  |  |  |  |  |  |  |  |  |  |  |  |
| --- | --- | --- | --- | --- | --- | --- | --- | --- | --- | --- | --- | --- |

tr|A0A672HXA9|A0A672HXA9\_SALFA

back to list

  

| Protein Coverage
| Supporting Peptides
|

Protein Coverage:

Supporting Peptides:

| Peptide | Uniq | -10lgP | Mass | ppm | m/z | z | RT | Scan | #Spec | Start | End | PTM |
| --- | --- | --- | --- | --- | --- | --- | --- | --- | --- | --- | --- | --- |
| F.MDDPVVIPGKPY.T | N | 35.25 | 1329.6638 | -0.8 | 665.8386 | 2 | 12.28 | 1829 | 1 | 238 | 249 |  |
| S.IVWIDPDDFPL.L | N | 15.72 | 1328.6652 | -0.2 | 665.3397 | 2 | 16.16 | 2489 | 1 | 320 | 330 |  |
| total 2 peptides |  |  |  |  |  |  |  |  |  |  |  |  |
| --- | --- | --- | --- | --- | --- | --- | --- | --- | --- | --- | --- | --- |

tr|A0A672HW84|A0A672HW84\_SALFA

back to list

  

| Protein Coverage
| Supporting Peptides
|

Protein Coverage:

Supporting Peptides:

| Peptide | Uniq | -10lgP | Mass | ppm | m/z | z | RT | Scan | #Spec | Start | End | PTM |
| --- | --- | --- | --- | --- | --- | --- | --- | --- | --- | --- | --- | --- |
| F.MDDPVVIPGKPY.T | N | 35.25 | 1329.6638 | -0.8 | 665.8386 | 2 | 12.28 | 1829 | 1 | 240 | 251 |  |
| S.IVWIDPDDFPL.L | N | 15.72 | 1328.6652 | -0.2 | 665.3397 | 2 | 16.16 | 2489 | 1 | 322 | 332 |  |
| total 2 peptides |  |  |  |  |  |  |  |  |  |  |  |  |
| --- | --- | --- | --- | --- | --- | --- | --- | --- | --- | --- | --- | --- |

tr|A0A671XZP8|A0A671XZP8\_SPAAU

back to list

  

| Protein Coverage
| Supporting Peptides
|

Protein Coverage:

Supporting Peptides:

| Peptide | Uniq | -10lgP | Mass | ppm | m/z | z | RT | Scan | #Spec | Start | End | PTM |
| --- | --- | --- | --- | --- | --- | --- | --- | --- | --- | --- | --- | --- |
| F.MDDPVVIPGKPY.T | N | 35.25 | 1329.6638 | -0.8 | 665.8386 | 2 | 12.28 | 1829 | 1 | 253 | 264 |  |
| S.IVWIDPDDFPL.L | N | 15.72 | 1328.6652 | -0.2 | 665.3397 | 2 | 16.16 | 2489 | 1 | 326 | 336 |  |
| total 2 peptides |  |  |  |  |  |  |  |  |  |  |  |  |
| --- | --- | --- | --- | --- | --- | --- | --- | --- | --- | --- | --- | --- |

tr|A0A4U5UZN8|A0A4U5UZN8\_COLLU

back to list

  

| Protein Coverage
| Supporting Peptides
|

Protein Coverage:

Supporting Peptides:

| Peptide | Uniq | -10lgP | Mass | ppm | m/z | z | RT | Scan | #Spec | Start | End | PTM |
| --- | --- | --- | --- | --- | --- | --- | --- | --- | --- | --- | --- | --- |
| F.MDDPVVIPGKPY.S | N | 35.25 | 1329.6638 | -0.8 | 665.8386 | 2 | 12.28 | 1829 | 1 | 225 | 236 |  |
| S.IVWIDPDDFPL.L | N | 15.72 | 1328.6652 | -0.2 | 665.3397 | 2 | 16.16 | 2489 | 1 | 307 | 317 |  |
| total 2 peptides |  |  |  |  |  |  |  |  |  |  |  |  |
| --- | --- | --- | --- | --- | --- | --- | --- | --- | --- | --- | --- | --- |

tr|A0A3Q3JCJ6|A0A3Q3JCJ6\_MONAL

back to list

  

| Protein Coverage
| Supporting Peptides
|

Protein Coverage:

Supporting Peptides:

| Peptide | Uniq | -10lgP | Mass | ppm | m/z | z | RT | Scan | #Spec | Start | End | PTM |
| --- | --- | --- | --- | --- | --- | --- | --- | --- | --- | --- | --- | --- |
| F.MDDPVVIPGKPY.T | N | 35.25 | 1329.6638 | -0.8 | 665.8386 | 2 | 12.28 | 1829 | 1 | 218 | 229 |  |
| S.IVWIDPDDFPL.L | N | 15.72 | 1328.6652 | -0.2 | 665.3397 | 2 | 16.16 | 2489 | 1 | 300 | 310 |  |
| total 2 peptides |  |  |  |  |  |  |  |  |  |  |  |  |
| --- | --- | --- | --- | --- | --- | --- | --- | --- | --- | --- | --- | --- |

tr|A0A3B4WCW4|A0A3B4WCW4\_SERLL

back to list

  

| Protein Coverage
| Supporting Peptides
|

Protein Coverage:

Supporting Peptides:

| Peptide | Uniq | -10lgP | Mass | ppm | m/z | z | RT | Scan | #Spec | Start | End | PTM |
| --- | --- | --- | --- | --- | --- | --- | --- | --- | --- | --- | --- | --- |
| F.MDDPVVIPGKPY.T | N | 35.25 | 1329.6638 | -0.8 | 665.8386 | 2 | 12.28 | 1829 | 1 | 225 | 236 |  |
| S.IVWIDPDDFPL.L | N | 15.72 | 1328.6652 | -0.2 | 665.3397 | 2 | 16.16 | 2489 | 1 | 297 | 307 |  |
| total 2 peptides |  |  |  |  |  |  |  |  |  |  |  |  |
| --- | --- | --- | --- | --- | --- | --- | --- | --- | --- | --- | --- | --- |

tr|A0A3B4UFZ1|A0A3B4UFZ1\_SERDU

back to list

  

| Protein Coverage
| Supporting Peptides
|

Protein Coverage:

Supporting Peptides:

| Peptide | Uniq | -10lgP | Mass | ppm | m/z | z | RT | Scan | #Spec | Start | End | PTM |
| --- | --- | --- | --- | --- | --- | --- | --- | --- | --- | --- | --- | --- |
| F.MDDPVVIPGKPY.T | N | 35.25 | 1329.6638 | -0.8 | 665.8386 | 2 | 12.28 | 1829 | 1 | 225 | 236 |  |
| S.IVWIDPDDFPL.L | N | 15.72 | 1328.6652 | -0.2 | 665.3397 | 2 | 16.16 | 2489 | 1 | 296 | 306 |  |
| total 2 peptides |  |  |  |  |  |  |  |  |  |  |  |  |
| --- | --- | --- | --- | --- | --- | --- | --- | --- | --- | --- | --- | --- |

tr|A0A3B4WCV3|A0A3B4WCV3\_SERLL

back to list

  

| Protein Coverage
| Supporting Peptides
|

Protein Coverage:

Supporting Peptides:

| Peptide | Uniq | -10lgP | Mass | ppm | m/z | z | RT | Scan | #Spec | Start | End | PTM |
| --- | --- | --- | --- | --- | --- | --- | --- | --- | --- | --- | --- | --- |
| F.MDDPVVIPGKPY.T | N | 35.25 | 1329.6638 | -0.8 | 665.8386 | 2 | 12.28 | 1829 | 1 | 225 | 236 |  |
| S.IVWIDPDDFPL.L | N | 15.72 | 1328.6652 | -0.2 | 665.3397 | 2 | 16.16 | 2489 | 1 | 307 | 317 |  |
| total 2 peptides |  |  |  |  |  |  |  |  |  |  |  |  |
| --- | --- | --- | --- | --- | --- | --- | --- | --- | --- | --- | --- | --- |

tr|A0A484CIB2|A0A484CIB2\_PERFV

back to list

  

| Protein Coverage
| Supporting Peptides
|

Protein Coverage:

Supporting Peptides:

| Peptide | Uniq | -10lgP | Mass | ppm | m/z | z | RT | Scan | #Spec | Start | End | PTM |
| --- | --- | --- | --- | --- | --- | --- | --- | --- | --- | --- | --- | --- |
| G.LRGDKGETGEAGER.G | N | 23.05 | 1473.7172 | -0.3 | 492.2462 | 3 | 8.56 | 1206 | 1 | 1076 | 1089 |  |
| M.GPMGPRGPPGAP.G | N | 22.19 | 1089.5389 | -0.6 | 545.7764 | 2 | 10.30 | 1483 | 1 | 166 | 177 |  |
| G.FAGPPGADGQPGAK.G | N | 22.00 | 1268.6149 | -0.4 | 635.3145 | 2 | 10.23 | 1471 | 1 | 797 | 810 |  |
| total 3 peptides |  |  |  |  |  |  |  |  |  |  |  |  |
| --- | --- | --- | --- | --- | --- | --- | --- | --- | --- | --- | --- | --- |

tr|A0A6A4SNF9|A0A6A4SNF9\_SCOMX

back to list

  

| Protein Coverage
| Supporting Peptides
|

Protein Coverage:

Supporting Peptides:

| Peptide | Uniq | -10lgP | Mass | ppm | m/z | z | RT | Scan | #Spec | Start | End | PTM |
| --- | --- | --- | --- | --- | --- | --- | --- | --- | --- | --- | --- | --- |
| F.IEEEELKLF.L | N | 30.75 | 1148.5964 | -0.3 | 575.3053 | 2 | 13.44 | 2031 | 1 | 156 | 164 |  |
| A.FDIIDQDKSGFIE.E | Y | 21.42 | 1525.7300 | -2.3 | 763.8705 | 2 | 20.04 | 3125 | 1 | 226 | 238 |  |
| total 2 peptides |  |  |  |  |  |  |  |  |  |  |  |  |
| --- | --- | --- | --- | --- | --- | --- | --- | --- | --- | --- | --- | --- |

tr|A0A3B3CIU5|A0A3B3CIU5\_ORYME

back to list

  

| Protein Coverage
| Supporting Peptides
|

Protein Coverage:

Supporting Peptides:

| Peptide | Uniq | -10lgP | Mass | ppm | m/z | z | RT | Scan | #Spec | Start | End | PTM |
| --- | --- | --- | --- | --- | --- | --- | --- | --- | --- | --- | --- | --- |
| R.GERGFPGLPGPS.G | N | 28.43 | 1169.5829 | -0.8 | 585.7983 | 2 | 12.09 | 1795 | 1 | 704 | 715 |  |
| M.GPMGPRGPPGAP.G | N | 22.19 | 1089.5389 | -0.6 | 545.7764 | 2 | 10.30 | 1483 | 1 | 44 | 55 |  |
| total 2 peptides |  |  |  |  |  |  |  |  |  |  |  |  |
| --- | --- | --- | --- | --- | --- | --- | --- | --- | --- | --- | --- | --- |

tr|A0A834FQR3|A0A834FQR3\_ORYME

back to list

  

| Protein Coverage
| Supporting Peptides
|

Protein Coverage:

Supporting Peptides:

| Peptide | Uniq | -10lgP | Mass | ppm | m/z | z | RT | Scan | #Spec | Start | End | PTM |
| --- | --- | --- | --- | --- | --- | --- | --- | --- | --- | --- | --- | --- |
| R.GERGFPGLPGPS.G | N | 28.43 | 1169.5829 | -0.8 | 585.7983 | 2 | 12.09 | 1795 | 1 | 964 | 975 |  |
| S.GPMGPRGPPGAP.G | N | 22.19 | 1089.5389 | -0.6 | 545.7764 | 2 | 10.30 | 1483 | 1 | 253 | 264 |  |
| total 2 peptides |  |  |  |  |  |  |  |  |  |  |  |  |
| --- | --- | --- | --- | --- | --- | --- | --- | --- | --- | --- | --- | --- |

tr|A0A437DEJ9|A0A437DEJ9\_ORYJA

back to list

  

| Protein Coverage
| Supporting Peptides
|

Protein Coverage:

Supporting Peptides:

| Peptide | Uniq | -10lgP | Mass | ppm | m/z | z | RT | Scan | #Spec | Start | End | PTM |
| --- | --- | --- | --- | --- | --- | --- | --- | --- | --- | --- | --- | --- |
| R.GERGFPGLPGPS.G | N | 28.43 | 1169.5829 | -0.8 | 585.7983 | 2 | 12.09 | 1795 | 1 | 994 | 1005 |  |
| S.GPMGPRGPPGAP.G | N | 22.19 | 1089.5389 | -0.6 | 545.7764 | 2 | 10.30 | 1483 | 1 | 241 | 252 |  |
| total 2 peptides |  |  |  |  |  |  |  |  |  |  |  |  |
| --- | --- | --- | --- | --- | --- | --- | --- | --- | --- | --- | --- | --- |

tr|G3N515|G3N515\_GASAC

back to list

  

| Protein Coverage
| Supporting Peptides
|

Protein Coverage:

Supporting Peptides:

| Peptide | Uniq | -10lgP | Mass | ppm | m/z | z | RT | Scan | #Spec | Start | End | PTM |
| --- | --- | --- | --- | --- | --- | --- | --- | --- | --- | --- | --- | --- |
| L.IVEELPVKW.L | N | 29.07 | 1111.6277 | -0.4 | 556.8209 | 2 | 13.24 | 1997 | 1 | 130 | 138 |  |
| A.ILPPGPPTPW.I | N | 19.56 | 1073.5909 | 0.5 | 537.8030 | 2 | 13.74 | 2081 | 1 | 3917 | 3926 |  |
| total 2 peptides |  |  |  |  |  |  |  |  |  |  |  |  |
| --- | --- | --- | --- | --- | --- | --- | --- | --- | --- | --- | --- | --- |

tr|Q4T444|Q4T444\_TETNG

back to list

  

| Protein Coverage
| Supporting Peptides
|

Protein Coverage:

Supporting Peptides:

| Peptide | Uniq | -10lgP | Mass | ppm | m/z | z | RT | Scan | #Spec | Start | End | PTM |
| --- | --- | --- | --- | --- | --- | --- | --- | --- | --- | --- | --- | --- |
| L.IVEELPVKW.V | N | 29.07 | 1111.6277 | -0.4 | 556.8209 | 2 | 13.24 | 1997 | 1 | 625 | 633 |  |
| A.ILPPGPPTPW.I | N | 19.56 | 1073.5909 | 0.5 | 537.8030 | 2 | 13.74 | 2081 | 1 | 4399 | 4408 |  |
| total 2 peptides |  |  |  |  |  |  |  |  |  |  |  |  |
| --- | --- | --- | --- | --- | --- | --- | --- | --- | --- | --- | --- | --- |

tr|A0A6P7IXE2|A0A6P7IXE2\_9TELE

back to list

  

| Protein Coverage
| Supporting Peptides
|

Protein Coverage:

Supporting Peptides:

| Peptide | Uniq | -10lgP | Mass | ppm | m/z | z | RT | Scan | #Spec | Start | End | PTM |
| --- | --- | --- | --- | --- | --- | --- | --- | --- | --- | --- | --- | --- |
| I.SLAPAPAPAPAPAPAPTTT.S | Y | 37.39 | 1697.8988 | -22.4 | 849.9377 | 2 | 11.95 | 1770 | 1 | 395 | 413 |  |
| total 1 peptides |  |  |  |  |  |  |  |  |  |  |  |  |
| --- | --- | --- | --- | --- | --- | --- | --- | --- | --- | --- | --- | --- |

tr|A0A3Q1HW42|A0A3Q1HW42\_9TELE

back to list

  

| Protein Coverage
| Supporting Peptides
|

Protein Coverage:

Supporting Peptides:

| Peptide | Uniq | -10lgP | Mass | ppm | m/z | z | RT | Scan | #Spec | Start | End | PTM |
| --- | --- | --- | --- | --- | --- | --- | --- | --- | --- | --- | --- | --- |
| D.SELPVDDVFGQ.V | Y | 24.56 | 1204.5612 | 0.1 | 603.2879 | 2 | 13.67 | 2069 | 1 | 175 | 185 |  |
| K.VDSELPVDDVFGQ.V | Y | 23.48 | 1418.6565 | -0.3 | 710.3353 | 2 | 13.85 | 2099 | 1 | 173 | 185 |  |
| total 2 peptides |  |  |  |  |  |  |  |  |  |  |  |  |
| --- | --- | --- | --- | --- | --- | --- | --- | --- | --- | --- | --- | --- |

tr|A0A3B5BIY5|A0A3B5BIY5\_9TELE

back to list

  

| Protein Coverage
| Supporting Peptides
|

Protein Coverage:

Supporting Peptides:

| Peptide | Uniq | -10lgP | Mass | ppm | m/z | z | RT | Scan | #Spec | Start | End | PTM |
| --- | --- | --- | --- | --- | --- | --- | --- | --- | --- | --- | --- | --- |
| D.SELPVDDVFGQ.V | Y | 24.56 | 1204.5612 | 0.1 | 603.2879 | 2 | 13.67 | 2069 | 1 | 175 | 185 |  |
| K.VDSELPVDDVFGQ.V | Y | 23.48 | 1418.6565 | -0.3 | 710.3353 | 2 | 13.85 | 2099 | 1 | 173 | 185 |  |
| total 2 peptides |  |  |  |  |  |  |  |  |  |  |  |  |
| --- | --- | --- | --- | --- | --- | --- | --- | --- | --- | --- | --- | --- |

tr|A0A3Q4GK85|A0A3Q4GK85\_NEOBR

back to list

  

| Protein Coverage
| Supporting Peptides
|

Protein Coverage:

Supporting Peptides:

| Peptide | Uniq | -10lgP | Mass | ppm | m/z | z | RT | Scan | #Spec | Start | End | PTM |
| --- | --- | --- | --- | --- | --- | --- | --- | --- | --- | --- | --- | --- |
| D.SELPVDDVFGQ.V | Y | 24.56 | 1204.5612 | 0.1 | 603.2879 | 2 | 13.67 | 2069 | 1 | 174 | 184 |  |
| K.VDSELPVDDVFGQ.V | Y | 23.48 | 1418.6565 | -0.3 | 710.3353 | 2 | 13.85 | 2099 | 1 | 172 | 184 |  |
| total 2 peptides |  |  |  |  |  |  |  |  |  |  |  |  |
| --- | --- | --- | --- | --- | --- | --- | --- | --- | --- | --- | --- | --- |

tr|A0A3Q4GS43|A0A3Q4GS43\_NEOBR

back to list

  

| Protein Coverage
| Supporting Peptides
|

Protein Coverage:

Supporting Peptides:

| Peptide | Uniq | -10lgP | Mass | ppm | m/z | z | RT | Scan | #Spec | Start | End | PTM |
| --- | --- | --- | --- | --- | --- | --- | --- | --- | --- | --- | --- | --- |
| D.SELPVDDVFGQ.V | Y | 24.56 | 1204.5612 | 0.1 | 603.2879 | 2 | 13.67 | 2069 | 1 | 175 | 185 |  |
| K.VDSELPVDDVFGQ.V | Y | 23.48 | 1418.6565 | -0.3 | 710.3353 | 2 | 13.85 | 2099 | 1 | 173 | 185 |  |
| total 2 peptides |  |  |  |  |  |  |  |  |  |  |  |  |
| --- | --- | --- | --- | --- | --- | --- | --- | --- | --- | --- | --- | --- |

tr|A0A3P8P8S3|A0A3P8P8S3\_ASTCA

back to list

  

| Protein Coverage
| Supporting Peptides
|

Protein Coverage:

Supporting Peptides:

| Peptide | Uniq | -10lgP | Mass | ppm | m/z | z | RT | Scan | #Spec | Start | End | PTM |
| --- | --- | --- | --- | --- | --- | --- | --- | --- | --- | --- | --- | --- |
| D.SELPVDDVFGQ.V | Y | 24.56 | 1204.5612 | 0.1 | 603.2879 | 2 | 13.67 | 2069 | 1 | 175 | 185 |  |
| K.VDSELPVDDVFGQ.V | Y | 23.48 | 1418.6565 | -0.3 | 710.3353 | 2 | 13.85 | 2099 | 1 | 173 | 185 |  |
| total 2 peptides |  |  |  |  |  |  |  |  |  |  |  |  |
| --- | --- | --- | --- | --- | --- | --- | --- | --- | --- | --- | --- | --- |

tr|A0A3P9CWL7|A0A3P9CWL7\_9CICH

back to list

  

| Protein Coverage
| Supporting Peptides
|

Protein Coverage:

Supporting Peptides:

| Peptide | Uniq | -10lgP | Mass | ppm | m/z | z | RT | Scan | #Spec | Start | End | PTM |
| --- | --- | --- | --- | --- | --- | --- | --- | --- | --- | --- | --- | --- |
| D.SELPVDDVFGQ.V | Y | 24.56 | 1204.5612 | 0.1 | 603.2879 | 2 | 13.67 | 2069 | 1 | 175 | 185 |  |
| K.VDSELPVDDVFGQ.V | Y | 23.48 | 1418.6565 | -0.3 | 710.3353 | 2 | 13.85 | 2099 | 1 | 173 | 185 |  |
| total 2 peptides |  |  |  |  |  |  |  |  |  |  |  |  |
| --- | --- | --- | --- | --- | --- | --- | --- | --- | --- | --- | --- | --- |

tr|A0A3Q2VY92|A0A3Q2VY92\_HAPBU

back to list

  

| Protein Coverage
| Supporting Peptides
|

Protein Coverage:

Supporting Peptides:

| Peptide | Uniq | -10lgP | Mass | ppm | m/z | z | RT | Scan | #Spec | Start | End | PTM |
| --- | --- | --- | --- | --- | --- | --- | --- | --- | --- | --- | --- | --- |
| D.SELPVDDVFGQ.V | Y | 24.56 | 1204.5612 | 0.1 | 603.2879 | 2 | 13.67 | 2069 | 1 | 175 | 185 |  |
| K.VDSELPVDDVFGQ.V | Y | 23.48 | 1418.6565 | -0.3 | 710.3353 | 2 | 13.85 | 2099 | 1 | 173 | 185 |  |
| total 2 peptides |  |  |  |  |  |  |  |  |  |  |  |  |
| --- | --- | --- | --- | --- | --- | --- | --- | --- | --- | --- | --- | --- |

tr|A0A3B4FMK3|A0A3B4FMK3\_9CICH

back to list

  

| Protein Coverage
| Supporting Peptides
|

Protein Coverage:

Supporting Peptides:

| Peptide | Uniq | -10lgP | Mass | ppm | m/z | z | RT | Scan | #Spec | Start | End | PTM |
| --- | --- | --- | --- | --- | --- | --- | --- | --- | --- | --- | --- | --- |
| D.SELPVDDVFGQ.V | Y | 24.56 | 1204.5612 | 0.1 | 603.2879 | 2 | 13.67 | 2069 | 1 | 175 | 185 |  |
| K.VDSELPVDDVFGQ.V | Y | 23.48 | 1418.6565 | -0.3 | 710.3353 | 2 | 13.85 | 2099 | 1 | 173 | 185 |  |
| total 2 peptides |  |  |  |  |  |  |  |  |  |  |  |  |
| --- | --- | --- | --- | --- | --- | --- | --- | --- | --- | --- | --- | --- |

tr|A0A3Q3CWR9|A0A3Q3CWR9\_HAPBU

back to list

  

| Protein Coverage
| Supporting Peptides
|

Protein Coverage:

Supporting Peptides:

| Peptide | Uniq | -10lgP | Mass | ppm | m/z | z | RT | Scan | #Spec | Start | End | PTM |
| --- | --- | --- | --- | --- | --- | --- | --- | --- | --- | --- | --- | --- |
| D.SELPVDDVFGQ.V | Y | 24.56 | 1204.5612 | 0.1 | 603.2879 | 2 | 13.67 | 2069 | 1 | 193 | 203 |  |
| K.VDSELPVDDVFGQ.V | Y | 23.48 | 1418.6565 | -0.3 | 710.3353 | 2 | 13.85 | 2099 | 1 | 191 | 203 |  |
| total 2 peptides |  |  |  |  |  |  |  |  |  |  |  |  |
| --- | --- | --- | --- | --- | --- | --- | --- | --- | --- | --- | --- | --- |

tr|A0A3P8P8V3|A0A3P8P8V3\_ASTCA

back to list

  

| Protein Coverage
| Supporting Peptides
|

Protein Coverage:

Supporting Peptides:

| Peptide | Uniq | -10lgP | Mass | ppm | m/z | z | RT | Scan | #Spec | Start | End | PTM |
| --- | --- | --- | --- | --- | --- | --- | --- | --- | --- | --- | --- | --- |
| D.SELPVDDVFGQ.V | Y | 24.56 | 1204.5612 | 0.1 | 603.2879 | 2 | 13.67 | 2069 | 1 | 193 | 203 |  |
| K.VDSELPVDDVFGQ.V | Y | 23.48 | 1418.6565 | -0.3 | 710.3353 | 2 | 13.85 | 2099 | 1 | 191 | 203 |  |
| total 2 peptides |  |  |  |  |  |  |  |  |  |  |  |  |
| --- | --- | --- | --- | --- | --- | --- | --- | --- | --- | --- | --- | --- |

tr|A0A3Q3WSG6|A0A3Q3WSG6\_MOLML

back to list

  

| Protein Coverage
| Supporting Peptides
|

Protein Coverage:

Supporting Peptides:

| Peptide | Uniq | -10lgP | Mass | ppm | m/z | z | RT | Scan | #Spec | Start | End | PTM |
| --- | --- | --- | --- | --- | --- | --- | --- | --- | --- | --- | --- | --- |
| H.IKWGEAGAQ.Y | Y | 25.43 | 958.4872 | -0.5 | 480.2506 | 2 | 10.91 | 1584 | 1 | 83 | 91 |  |
| R.VVISAPSADAPMF.V | N | 15.30 | 1303.6482 | 0.0 | 652.8314 | 2 | 13.76 | 2084 | 1 | 117 | 129 |  |
| total 2 peptides |  |  |  |  |  |  |  |  |  |  |  |  |
| --- | --- | --- | --- | --- | --- | --- | --- | --- | --- | --- | --- | --- |

tr|A0A6P7K206|A0A6P7K206\_9TELE

back to list

  

| Protein Coverage
| Supporting Peptides
|

Protein Coverage:

Supporting Peptides:

| Peptide | Uniq | -10lgP | Mass | ppm | m/z | z | RT | Scan | #Spec | Start | End | PTM |
| --- | --- | --- | --- | --- | --- | --- | --- | --- | --- | --- | --- | --- |
| F.HERDPAN.I | N | 15.89 | 837.3729 | -0.2 | 419.6937 | 2 | 7.17 | 946 | 3 | 77 | 83 |  |
| R.VVISAPSADAPMF.V | N | 15.30 | 1303.6482 | 0.0 | 652.8314 | 2 | 13.76 | 2084 | 1 | 114 | 126 |  |
| total 2 peptides |  |  |  |  |  |  |  |  |  |  |  |  |
| --- | --- | --- | --- | --- | --- | --- | --- | --- | --- | --- | --- | --- |

tr|F5BZT7|F5BZT7\_EPIBR

back to list

  

| Protein Coverage
| Supporting Peptides
|

Protein Coverage:

Supporting Peptides:

| Peptide | Uniq | -10lgP | Mass | ppm | m/z | z | RT | Scan | #Spec | Start | End | PTM |
| --- | --- | --- | --- | --- | --- | --- | --- | --- | --- | --- | --- | --- |
| F.HERDPAN.I | N | 15.89 | 837.3729 | -0.2 | 419.6937 | 2 | 7.17 | 946 | 3 | 77 | 83 |  |
| R.VVISAPSADAPMF.V | N | 15.30 | 1303.6482 | 0.0 | 652.8314 | 2 | 13.76 | 2084 | 1 | 118 | 130 |  |
| total 2 peptides |  |  |  |  |  |  |  |  |  |  |  |  |
| --- | --- | --- | --- | --- | --- | --- | --- | --- | --- | --- | --- | --- |

tr|A0A076U5J2|A0A076U5J2\_SALFO

back to list

  

| Protein Coverage
| Supporting Peptides
|

Protein Coverage:

Supporting Peptides:

| Peptide | Uniq | -10lgP | Mass | ppm | m/z | z | RT | Scan | #Spec | Start | End | PTM |
| --- | --- | --- | --- | --- | --- | --- | --- | --- | --- | --- | --- | --- |
| F.HERDPAN.I | N | 15.89 | 837.3729 | -0.2 | 419.6937 | 2 | 7.17 | 946 | 3 | 42 | 48 |  |
| R.VVISAPSADAPMF.V | N | 15.30 | 1303.6482 | 0.0 | 652.8314 | 2 | 13.76 | 2084 | 1 | 83 | 95 |  |
| total 2 peptides |  |  |  |  |  |  |  |  |  |  |  |  |
| --- | --- | --- | --- | --- | --- | --- | --- | --- | --- | --- | --- | --- |

tr|A0A1A8SH28|A0A1A8SH28\_9TELE

back to list

  

| Protein Coverage
| Supporting Peptides
|

Protein Coverage:

Supporting Peptides:

| Peptide | Uniq | -10lgP | Mass | ppm | m/z | z | RT | Scan | #Spec | Start | End | PTM |
| --- | --- | --- | --- | --- | --- | --- | --- | --- | --- | --- | --- | --- |
| G.VSGASGPLGPP.G | Y | 23.48 | 937.4869 | 0.0 | 469.7507 | 2 | 10.51 | 1517 | 1 | 939 | 949 |  |
| total 1 peptides |  |  |  |  |  |  |  |  |  |  |  |  |
| --- | --- | --- | --- | --- | --- | --- | --- | --- | --- | --- | --- | --- |

tr|A0A1A8AFF3|A0A1A8AFF3\_NOTFU

back to list

  

| Protein Coverage
| Supporting Peptides
|

Protein Coverage:

Supporting Peptides:

| Peptide | Uniq | -10lgP | Mass | ppm | m/z | z | RT | Scan | #Spec | Start | End | PTM |
| --- | --- | --- | --- | --- | --- | --- | --- | --- | --- | --- | --- | --- |
| G.VSGASGPLGPP.G | Y | 23.48 | 937.4869 | 0.0 | 469.7507 | 2 | 10.51 | 1517 | 1 | 1328 | 1338 |  |
| total 1 peptides |  |  |  |  |  |  |  |  |  |  |  |  |
| --- | --- | --- | --- | --- | --- | --- | --- | --- | --- | --- | --- | --- |

tr|A0A6P6JF49|A0A6P6JF49\_CARAU

back to list

  

| Protein Coverage
| Supporting Peptides
|

Protein Coverage:

Supporting Peptides:

| Peptide | Uniq | -10lgP | Mass | ppm | m/z | z | RT | Scan | #Spec | Start | End | PTM |
| --- | --- | --- | --- | --- | --- | --- | --- | --- | --- | --- | --- | --- |
| G.VSGASGPLGPP.G | Y | 23.48 | 937.4869 | 0.0 | 469.7507 | 2 | 10.51 | 1517 | 1 | 1373 | 1383 |  |
| total 1 peptides |  |  |  |  |  |  |  |  |  |  |  |  |
| --- | --- | --- | --- | --- | --- | --- | --- | --- | --- | --- | --- | --- |

tr|A0A1A7ZX47|A0A1A7ZX47\_NOTFU

back to list

  

| Protein Coverage
| Supporting Peptides
|

Protein Coverage:

Supporting Peptides:

| Peptide | Uniq | -10lgP | Mass | ppm | m/z | z | RT | Scan | #Spec | Start | End | PTM |
| --- | --- | --- | --- | --- | --- | --- | --- | --- | --- | --- | --- | --- |
| G.VSGASGPLGPP.G | Y | 23.48 | 937.4869 | 0.0 | 469.7507 | 2 | 10.51 | 1517 | 1 | 1397 | 1407 |  |
| total 1 peptides |  |  |  |  |  |  |  |  |  |  |  |  |
| --- | --- | --- | --- | --- | --- | --- | --- | --- | --- | --- | --- | --- |

tr|A0A6P6JAI8|A0A6P6JAI8\_CARAU

back to list

  

| Protein Coverage
| Supporting Peptides
|

Protein Coverage:

Supporting Peptides:

| Peptide | Uniq | -10lgP | Mass | ppm | m/z | z | RT | Scan | #Spec | Start | End | PTM |
| --- | --- | --- | --- | --- | --- | --- | --- | --- | --- | --- | --- | --- |
| G.VSGASGPLGPP.G | Y | 23.48 | 937.4869 | 0.0 | 469.7507 | 2 | 10.51 | 1517 | 1 | 1402 | 1412 |  |
| total 1 peptides |  |  |  |  |  |  |  |  |  |  |  |  |
| --- | --- | --- | --- | --- | --- | --- | --- | --- | --- | --- | --- | --- |

tr|A0A1A8VA48|A0A1A8VA48\_NOTFU

back to list

  

| Protein Coverage
| Supporting Peptides
|

Protein Coverage:

Supporting Peptides:

| Peptide | Uniq | -10lgP | Mass | ppm | m/z | z | RT | Scan | #Spec | Start | End | PTM |
| --- | --- | --- | --- | --- | --- | --- | --- | --- | --- | --- | --- | --- |
| G.VSGASGPLGPP.G | Y | 23.48 | 937.4869 | 0.0 | 469.7507 | 2 | 10.51 | 1517 | 1 | 1488 | 1498 |  |
| total 1 peptides |  |  |  |  |  |  |  |  |  |  |  |  |
| --- | --- | --- | --- | --- | --- | --- | --- | --- | --- | --- | --- | --- |

tr|A0A6P6JA06|A0A6P6JA06\_CARAU

back to list

  

| Protein Coverage
| Supporting Peptides
|

Protein Coverage:

Supporting Peptides:

| Peptide | Uniq | -10lgP | Mass | ppm | m/z | z | RT | Scan | #Spec | Start | End | PTM |
| --- | --- | --- | --- | --- | --- | --- | --- | --- | --- | --- | --- | --- |
| G.VSGASGPLGPP.G | Y | 23.48 | 937.4869 | 0.0 | 469.7507 | 2 | 10.51 | 1517 | 1 | 1422 | 1432 |  |
| total 1 peptides |  |  |  |  |  |  |  |  |  |  |  |  |
| --- | --- | --- | --- | --- | --- | --- | --- | --- | --- | --- | --- | --- |

tr|A0A6P6J9V5|A0A6P6J9V5\_CARAU

back to list

  

| Protein Coverage
| Supporting Peptides
|

Protein Coverage:

Supporting Peptides:

| Peptide | Uniq | -10lgP | Mass | ppm | m/z | z | RT | Scan | #Spec | Start | End | PTM |
| --- | --- | --- | --- | --- | --- | --- | --- | --- | --- | --- | --- | --- |
| G.VSGASGPLGPP.G | Y | 23.48 | 937.4869 | 0.0 | 469.7507 | 2 | 10.51 | 1517 | 1 | 1474 | 1484 |  |
| total 1 peptides |  |  |  |  |  |  |  |  |  |  |  |  |
| --- | --- | --- | --- | --- | --- | --- | --- | --- | --- | --- | --- | --- |

tr|A0A1A8S1T5|A0A1A8S1T5\_9TELE

back to list

  

| Protein Coverage
| Supporting Peptides
|

Protein Coverage:

Supporting Peptides:

| Peptide | Uniq | -10lgP | Mass | ppm | m/z | z | RT | Scan | #Spec | Start | End | PTM |
| --- | --- | --- | --- | --- | --- | --- | --- | --- | --- | --- | --- | --- |
| G.VSGASGPLGPP.G | Y | 23.48 | 937.4869 | 0.0 | 469.7507 | 2 | 10.51 | 1517 | 1 | 1492 | 1502 |  |
| total 1 peptides |  |  |  |  |  |  |  |  |  |  |  |  |
| --- | --- | --- | --- | --- | --- | --- | --- | --- | --- | --- | --- | --- |

tr|A0A6P6JCL5|A0A6P6JCL5\_CARAU

back to list

  

| Protein Coverage
| Supporting Peptides
|

Protein Coverage:

Supporting Peptides:

| Peptide | Uniq | -10lgP | Mass | ppm | m/z | z | RT | Scan | #Spec | Start | End | PTM |
| --- | --- | --- | --- | --- | --- | --- | --- | --- | --- | --- | --- | --- |
| G.VSGASGPLGPP.G | Y | 23.48 | 937.4869 | 0.0 | 469.7507 | 2 | 10.51 | 1517 | 1 | 1503 | 1513 |  |
| total 1 peptides |  |  |  |  |  |  |  |  |  |  |  |  |
| --- | --- | --- | --- | --- | --- | --- | --- | --- | --- | --- | --- | --- |

tr|A0A3P8TNL0|A0A3P8TNL0\_AMPPE

back to list

  

| Protein Coverage
| Supporting Peptides
|

Protein Coverage:

Supporting Peptides:

| Peptide | Uniq | -10lgP | Mass | ppm | m/z | z | RT | Scan | #Spec | Start | End | PTM |
| --- | --- | --- | --- | --- | --- | --- | --- | --- | --- | --- | --- | --- |
| P.EVDAVRAL.P | Y | 22.74 | 871.4763 | -0.4 | 436.7452 | 2 | 11.50 | 1689 | 1 | 449 | 456 |  |
| total 1 peptides |  |  |  |  |  |  |  |  |  |  |  |  |
| --- | --- | --- | --- | --- | --- | --- | --- | --- | --- | --- | --- | --- |

tr|A0A6P6M1S2|A0A6P6M1S2\_CARAU

back to list

  

| Protein Coverage
| Supporting Peptides
|

Protein Coverage:

Supporting Peptides:

| Peptide | Uniq | -10lgP | Mass | ppm | m/z | z | RT | Scan | #Spec | Start | End | PTM |
| --- | --- | --- | --- | --- | --- | --- | --- | --- | --- | --- | --- | --- |
| S.LENQQLAQIL.K | Y | 22.02 | 1168.6451 | -8.7 | 585.3248 | 2 | 13.10 | 1974 | 2 | 351 | 360 |  |
| total 1 peptides |  |  |  |  |  |  |  |  |  |  |  |  |
| --- | --- | --- | --- | --- | --- | --- | --- | --- | --- | --- | --- | --- |

tr|A0A315V5V2|A0A315V5V2\_GAMAF

back to list

  

| Protein Coverage
| Supporting Peptides
|

Protein Coverage:

Supporting Peptides:

| Peptide | Uniq | -10lgP | Mass | ppm | m/z | z | RT | Scan | #Spec | Start | End | PTM |
| --- | --- | --- | --- | --- | --- | --- | --- | --- | --- | --- | --- | --- |
| F.LNLDNVVSGLSI.H | Y | 21.66 | 1242.6819 | -10.1 | 622.3419 | 2 | 18.21 | 2815 | 4 | 844 | 855 |  |
| total 1 peptides |  |  |  |  |  |  |  |  |  |  |  |  |
| --- | --- | --- | --- | --- | --- | --- | --- | --- | --- | --- | --- | --- |

tr|A0A3P8PXL3|A0A3P8PXL3\_ASTCA

back to list

  

| Protein Coverage
| Supporting Peptides
|

Protein Coverage:

Supporting Peptides:

| Peptide | Uniq | -10lgP | Mass | ppm | m/z | z | RT | Scan | #Spec | Start | End | PTM |
| --- | --- | --- | --- | --- | --- | --- | --- | --- | --- | --- | --- | --- |
| Q.SASPSPGPRMPM.P | Y | 21.04 | 1213.5583 | -22.2 | 607.7730 | 2 | 12.78 | 1917 | 1 | 109 | 120 |  |
| total 1 peptides |  |  |  |  |  |  |  |  |  |  |  |  |
| --- | --- | --- | --- | --- | --- | --- | --- | --- | --- | --- | --- | --- |

tr|A0A674A9W5|A0A674A9W5\_SALTR

back to list

  

| Protein Coverage
| Supporting Peptides
|

Protein Coverage:

Supporting Peptides:

| Peptide | Uniq | -10lgP | Mass | ppm | m/z | z | RT | Scan | #Spec | Start | End | PTM |
| --- | --- | --- | --- | --- | --- | --- | --- | --- | --- | --- | --- | --- |
| G.IVPIVEPEILPD.G | Y | 20.23 | 1332.7540 | 0.1 | 667.3843 | 2 | 14.32 | 2177 | 1 | 182 | 193 |  |
| total 1 peptides |  |  |  |  |  |  |  |  |  |  |  |  |
| --- | --- | --- | --- | --- | --- | --- | --- | --- | --- | --- | --- | --- |

---

Prepared with PEAKS ™ (bioinfor.com)
