# Supplementary material for: Proteomic and Functional Characterization of Antimicrobial Peptides Derived from Fisheries Bycatch via Enzymatic Hydrolysis
Source: Mar Drugs. 2026 Jan 10;24(1):36. doi: 10.3390/md24010036 (PMC12843163; doi:10.3390/md24010036)
Supplement: Supplementary file 1 [file marinedrugs-24-00036-s001.zip › SM42/PA/protein.html]

proteins


Summary

  

# 1. Notes

# 2. Result Statistics

**Figure 1.**
False discovery rate (FDR) curve. X axis is the number of peptide-spectrum matches (PSM) being kept. Y axis is the corresponding FDR.

  


  

**Figure 2.**
PSM score distribution.  **(a)**
Distribution of PEAKS peptide score;  **(b)**
Scatterplot of PEAKS peptide score versus precursor mass error.

**(a)**

**(b)**

**Figure 3.**
De novo result validation. Distribution of residue local confidence:  **(a)**
Residues in de novo sequences validated by confident database peptide assignment;  **(b)**
Residues in "de novo only" sequences.

**(a)**

**(b)**

**Table 1.**
Statistics of data.

|  |  |
| --- | --- |
| # of MS Scans | 1086 |
| # of MS/MS Scans | 2771 |


**Table 2.**
Result filtration parameters.

|  |  |
| --- | --- |
| Peptide -10lgP | ≥15 |
| Protein -10lgP | ≥20 |
| Proteins unique peptides | ≥0 |
| De novo ALC Score | ≥50% |


**Table 3.**
Statistics of filtered result.

|  |  |
| --- | --- |
| Peptide-Spectrum Matches | 86 |
| Peptide Sequences | 80 |
| Protein Groups | 26 |
| Proteins | 215 |
| Proteins (#Unique Peptides) | 57 (>2); 72 (=2); 86 (=1); |
| FDR (Peptide-Spectrum Matches) | 14.0% |
| FDR (Peptide Sequences) | 13.8% |
| FDR (Protein) | 0.9% |
| De Novo Only Spectra | 1288 |

**Table 4.**
PTM profile.

| Name | ∆Mass | #PSM | Position |
| --- | --- | --- | --- |

# 3. Experiment Control

**Figure 4.**
Precursor mass error of peptide-spectrum matches (PSM) in filtered result.  **(a)**
Distribution of precursor mass error in ppm;  **(b)**
Scatterplot of precursor m/z versus precursor mass error in ppm.

**(a)**

**(b)**

**Table 5.**
Number of identified peptides in each sample by the number of missed cleavages

|  |  |  |  |  |  |
| --- | --- | --- | --- | --- | --- |
| Missed Cleavages | 0 | 1 | 2 | 3 | 4+ |
| Orbi2222 | 0 | 0 | 0 | 0 | 80 |

# 4. Other Information

**Table 6.**
Search parameters.

Search Engine Name: PEAKS 7.0  
Parent Mass Error Tolerance: 0.03 Da  
Fragment Mass Error Tolerance: 0.1 Da  
Precursor Mass Search Type: monoisotopic  
Enzyme: None  
Max Missed Cleavages: 100  
Non-specific Cleavage: both  
Max variable PTM per peptide: 3  
Database: Teleosteo  
Taxon: All  
Searched Entry: 4529691  
FDR Estimation: Enabled  
Merge Options: 0.1 min. 0.03 Da  
Precursor Options: corrected  
Charge Options: no correction  
Filter Options: no filter  
Process: true

**Table 7.**
Instrument parameters.

Fractions: OB\_PIMENTA\_PA\_20220211\_03.raw  
Ion Source: ESI(nano-spray)  
Fragmentation Mode: CID/ECD  
MS Scan Mode: FT-ICR/Orbitrap  
MS/MS Scan Mode: Linear Ion Trap

  

Protein List

  

| Protein Group | Protein ID | Accession | -10lgP | Coverage (%) | #Peptides | #Unique | PTM | Avg. Mass | Description |
| --- | --- | --- | --- | --- | --- | --- | --- | --- | --- |
| 1 | 12040 | tr\|A0A3S2MUF9\|A0A3S2MUF9\_ORYJA | 81.95 | 16 | 9 | 9 | N | 41945 | Uncharacterized protein OS=Oryzias javanicus OX=123683 GN=OJAV\_G00024300 PE=3 SV=1 |
| 1 | 12038 | tr\|F5BZL8\|F5BZL8\_EPIBR | 81.95 | 16 | 9 | 9 | N | 41945 | Alpha actin (Fragment) OS=Epinephelus bruneus OX=323802 PE=2 SV=1 |
| 1 | 66846 | tr\|A0A7J6C441\|A0A7J6C441\_9TELE | 81.95 | 16 | 9 | 9 | N | 41959 | Uncharacterized protein OS=Onychostoma macrolepis OX=369639 GN=G5714\_016621 PE=3 SV=1 |
| 1 | 66847 | tr\|A0A6P7M219\|A0A6P7M219\_BETSP | 81.95 | 16 | 9 | 9 | N | 41959 | actin, alpha skeletal muscle OS=Betta splendens OX=158456 GN=acta1 PE=3 SV=1 |
| 2 | 66929 | tr\|A0A3P8SGW9\|A0A3P8SGW9\_AMPPE | 78.71 | 3 | 9 | 9 | N | 220651 | Uncharacterized protein OS=Amphiprion percula OX=161767 PE=3 SV=1 |
| 2 | 66812 | tr\|A0A6P7IKT0\|A0A6P7IKT0\_9TELE | 78.71 | 3 | 9 | 9 | N | 221389 | myosin heavy chain, fast skeletal muscle OS=Parambassis ranga OX=210632 GN=LOC114440528 PE=3 SV=1 |
| 2 | 66803 | tr\|A0A6P7IXS4\|A0A6P7IXS4\_9TELE | 78.71 | 3 | 9 | 9 | N | 221345 | myosin heavy chain, fast skeletal muscle-like OS=Parambassis ranga OX=210632 GN=LOC114440527 PE=3 SV=1 |
| 2 | 66811 | tr\|A0A3P9CRE7\|A0A3P9CRE7\_9CICH | 78.71 | 3 | 9 | 9 | N | 219343 | Myosin heavy chain, fast skeletal muscle OS=Maylandia zebra OX=106582 PE=3 SV=1 |
| 2 | 66915 | tr\|A0A3P8SE64\|A0A3P8SE64\_AMPPE | 78.71 | 3 | 9 | 9 | N | 224259 | Uncharacterized protein OS=Amphiprion percula OX=161767 PE=3 SV=1 |
| 2 | 71725 | tr\|A0A668A9K0\|A0A668A9K0\_9TELE | 78.71 | 3 | 9 | 9 | N | 222745 | Myosin heavy chain, fast skeletal muscle OS=Myripristis murdjan OX=586833 GN=LOC115363301 PE=3 SV=1 |
| 2 | 66813 | tr\|A0A3P8Q1P8\|A0A3P8Q1P8\_ASTCA | 78.71 | 3 | 9 | 9 | N | 221800 | Myosin heavy chain, fast skeletal muscle-like OS=Astatotilapia calliptera OX=8154 PE=3 SV=1 |
| 2 | 66814 | tr\|A0A3P8QMC2\|A0A3P8QMC2\_ASTCA | 78.71 | 3 | 9 | 9 | N | 222125 | Myosin heavy chain, fast skeletal muscle-like OS=Astatotilapia calliptera OX=8154 PE=3 SV=1 |
| 2 | 71726 | tr\|A0A671XUP5\|A0A671XUP5\_SPAAU | 78.71 | 3 | 9 | 9 | N | 221269 | Myosin heavy chain, fast skeletal muscle-like OS=Sparus aurata OX=8175 GN=LOC115575699 PE=3 SV=1 |
| 2 | 66815 | tr\|A0A3P8Q1G5\|A0A3P8Q1G5\_ASTCA | 78.71 | 3 | 9 | 9 | N | 222421 | Myosin heavy chain, fast skeletal muscle-like OS=Astatotilapia calliptera OX=8154 PE=3 SV=1 |
| 2 | 66801 | tr\|Q90YF6\|Q90YF6\_PARFO | 78.71 | 3 | 9 | 9 | N | 221374 | Myosin heavy chain OS=Paracirrhites forsteri OX=100206 GN=MyoHC-T54 PE=2 SV=1 |
| 14 | 71706 | tr\|A0A3Q3R2B2\|A0A3Q3R2B2\_MONAL | 67.79 | 21 | 2 | 2 | N | 10968 | Parvalbumin OS=Monopterus albus OX=43700 PE=3 SV=1 |
| 14 | 71707 | tr\|A0A3Q3JW30\|A0A3Q3JW30\_MONAL | 67.79 | 21 | 2 | 2 | N | 11216 | Parvalbumin OS=Monopterus albus OX=43700 PE=3 SV=1 |
| 14 | 71708 | tr\|A0A3Q3K7G4\|A0A3Q3K7G4\_MONAL | 67.79 | 21 | 2 | 2 | N | 11274 | Parvalbumin OS=Monopterus albus OX=43700 PE=3 SV=1 |
| 14 | 71709 | tr\|A0A3Q3K945\|A0A3Q3K945\_MONAL | 67.79 | 21 | 2 | 2 | N | 11220 | Parvalbumin OS=Monopterus albus OX=43700 PE=3 SV=1 |
| 14 | 71710 | tr\|H2V1I8\|H2V1I8\_TAKRU | 67.79 | 20 | 2 | 2 | N | 11356 | Parvalbumin OS=Takifugu rubripes OX=31033 GN=LOC101064450 PE=3 SV=1 |
| 14 | 67952 | tr\|A0A455R7U6\|A0A455R7U6\_CONMY | 67.79 | 20 | 2 | 2 | N | 11823 | Parvalbumin OS=Conger myriaster OX=7943 GN=PVAL\_B PE=2 SV=1 |
| 14 | 71711 | tr\|A0A4W4ED54\|A0A4W4ED54\_ELEEL | 67.79 | 20 | 2 | 2 | N | 11603 | Parvalbumin OS=Electrophorus electricus OX=8005 GN=LOC113569874 PE=3 SV=1 |
| 14 | 71712 | tr\|A0A6P7N9P3\|A0A6P7N9P3\_BETSP | 67.79 | 20 | 2 | 2 | N | 11661 | Parvalbumin OS=Betta splendens OX=158456 GN=LOC114860448 PE=3 SV=1 |
| 14 | 71713 | tr\|A0A3B4CKV2\|A0A3B4CKV2\_PYGNA | 67.79 | 20 | 2 | 2 | N | 11597 | Parvalbumin OS=Pygocentrus nattereri OX=42514 PE=3 SV=1 |
| 14 | 71714 | tr\|A0A3B4TWQ8\|A0A3B4TWQ8\_SERDU | 67.79 | 20 | 2 | 2 | N | 11752 | Parvalbumin OS=Seriola dumerili OX=41447 PE=3 SV=1 |
| 14 | 71715 | tr\|A0A7N8YBR8\|A0A7N8YBR8\_9TELE | 67.79 | 20 | 2 | 2 | N | 11613 | Parvalbumin OS=Mastacembelus armatus OX=205130 PE=3 SV=1 |
| 14 | 67953 | tr\|A0A3Q3W464\|A0A3Q3W464\_MOLML | 67.79 | 19 | 2 | 2 | N | 12300 | Parvalbumin OS=Mola mola OX=94237 PE=3 SV=1 |
| 14 | 71716 | tr\|A0A3Q2WZN1\|A0A3Q2WZN1\_HAPBU | 67.79 | 19 | 2 | 2 | N | 12192 | Parvalbumin OS=Haplochromis burtoni OX=8153 PE=3 SV=1 |
| 14 | 71717 | tr\|A0A674NSY1\|A0A674NSY1\_TAKRU | 67.79 | 19 | 2 | 2 | N | 12203 | Parvalbumin OS=Takifugu rubripes OX=31033 GN=LOC101064450 PE=3 SV=1 |
| 14 | 71718 | tr\|A0A3Q3K779\|A0A3Q3K779\_MONAL | 67.79 | 19 | 2 | 2 | N | 12568 | Parvalbumin OS=Monopterus albus OX=43700 PE=3 SV=1 |
| 14 | 71719 | tr\|I3J1U3\|I3J1U3\_ORENI | 67.79 | 18 | 2 | 2 | N | 12911 | Parvalbumin OS=Oreochromis niloticus OX=8128 GN=LOC100692640 PE=3 SV=2 |
| 14 | 67954 | tr\|A0A3Q3WAG3\|A0A3Q3WAG3\_MOLML | 67.79 | 18 | 2 | 2 | N | 13233 | Parvalbumin OS=Mola mola OX=94237 PE=3 SV=1 |
| 14 | 71720 | tr\|Q804Z0\|Q804Z0\_ICTPU | 67.79 | 18 | 2 | 2 | N | 13489 | Parvalbumin (Fragment) OS=Ictalurus punctatus OX=7998 PE=2 SV=1 |
| 14 | 67955 | tr\|A0A3Q4AW30\|A0A3Q4AW30\_MOLML | 67.79 | 17 | 2 | 2 | N | 14276 | Parvalbumin OS=Mola mola OX=94237 PE=3 SV=1 |
| 14 | 67956 | tr\|A0A3Q3W3Y6\|A0A3Q3W3Y6\_MOLML | 67.79 | 17 | 2 | 2 | N | 14526 | Parvalbumin OS=Mola mola OX=94237 PE=3 SV=1 |
| 14 | 71721 | tr\|A0A3Q3K924\|A0A3Q3K924\_MONAL | 67.79 | 16 | 2 | 2 | N | 15324 | Parvalbumin OS=Monopterus albus OX=43700 PE=3 SV=1 |
| 14 | 67625 | tr\|A0A674P8J8\|A0A674P8J8\_TAKRU | 67.79 | 15 | 2 | 2 | N | 16019 | Parvalbumin OS=Takifugu rubripes OX=31033 PE=3 SV=1 |
| 14 | 71722 | tr\|A0A7N9APR6\|A0A7N9APR6\_9TELE | 67.79 | 14 | 2 | 2 | N | 16398 | Parvalbumin OS=Mastacembelus armatus OX=205130 PE=3 SV=1 |
| 14 | 71723 | tr\|A0A3Q3K937\|A0A3Q3K937\_MONAL | 67.79 | 14 | 2 | 2 | N | 17292 | Parvalbumin OS=Monopterus albus OX=43700 PE=3 SV=1 |
| 14 | 71724 | tr\|A0A3Q3JXS6\|A0A3Q3JXS6\_MONAL | 67.79 | 12 | 2 | 2 | N | 20228 | Parvalbumin OS=Monopterus albus OX=43700 PE=3 SV=1 |
| 9 | 71731 | tr\|H3CFQ5\|H3CFQ5\_TETNG | 59.63 | 1 | 3 | 3 | N | 544663 | Uncharacterized protein OS=Tetraodon nigroviridis OX=99883 PE=4 SV=1 |
| 4 | 71733 | tr\|M4AVS2\|M4AVS2\_XIPMA | 57.11 | 3 | 4 | 4 | N | 108300 | Calcium-transporting ATPase OS=Xiphophorus maculatus OX=8083 PE=3 SV=2 |
| 4 | 71734 | tr\|A0A3B3VSS0\|A0A3B3VSS0\_9TELE | 57.11 | 3 | 4 | 4 | N | 108408 | Calcium-transporting ATPase OS=Poecilia latipinna OX=48699 PE=3 SV=1 |
| 4 | 71735 | tr\|A0A0S7JRJ0\|A0A0S7JRJ0\_9TELE | 57.11 | 3 | 4 | 4 | N | 108563 | Calcium-transporting ATPase OS=Poeciliopsis prolifica OX=188132 GN=AT2A1 PE=3 SV=1 |
| 4 | 71736 | tr\|A0A0S7JR62\|A0A0S7JR62\_9TELE | 57.11 | 3 | 4 | 4 | N | 109108 | Calcium-transporting ATPase OS=Poeciliopsis prolifica OX=188132 GN=AT2A1 PE=3 SV=1 |
| 4 | 71737 | tr\|A0A3B5QZF8\|A0A3B5QZF8\_XIPMA | 57.11 | 3 | 4 | 4 | N | 109027 | Calcium-transporting ATPase OS=Xiphophorus maculatus OX=8083 PE=3 SV=1 |
| 4 | 71738 | tr\|G3NWV4\|G3NWV4\_GASAC | 57.11 | 3 | 4 | 4 | N | 108017 | Calcium-transporting ATPase OS=Gasterosteus aculeatus OX=69293 PE=3 SV=1 |
| 4 | 67754 | tr\|A0A6J2RNI9\|A0A6J2RNI9\_COTGO | 57.11 | 3 | 4 | 4 | N | 108341 | Calcium-transporting ATPase OS=Cottoperca gobio OX=56716 GN=LOC115024282 PE=3 SV=1 |
| 4 | 67755 | tr\|A0A6J2RKY9\|A0A6J2RKY9\_COTGO | 57.11 | 3 | 4 | 4 | N | 108469 | Calcium-transporting ATPase OS=Cottoperca gobio OX=56716 GN=LOC115024282 PE=3 SV=1 |
| 4 | 71739 | tr\|A0A3B1JPG2\|A0A3B1JPG2\_ASTMX | 57.11 | 3 | 4 | 4 | N | 108879 | Calcium-transporting ATPase OS=Astyanax mexicanus OX=7994 PE=3 SV=1 |
| 4 | 71740 | tr\|A0A667WYB6\|A0A667WYB6\_9TELE | 57.11 | 3 | 4 | 4 | N | 108672 | Calcium-transporting ATPase OS=Myripristis murdjan OX=586833 PE=3 SV=1 |
| 4 | 71741 | tr\|A0A3B4VM00\|A0A3B4VM00\_SERDU | 57.11 | 3 | 4 | 4 | N | 109419 | Calcium-transporting ATPase OS=Seriola dumerili OX=41447 PE=3 SV=1 |
| 4 | 71742 | tr\|A0A667X953\|A0A667X953\_9TELE | 57.11 | 3 | 4 | 4 | N | 109525 | Calcium-transporting ATPase OS=Myripristis murdjan OX=586833 PE=3 SV=1 |
| 4 | 67212 | tr\|A0A7N8WP58\|A0A7N8WP58\_9TELE | 57.11 | 4 | 4 | 4 | N | 104947 | Calcium-transporting ATPase OS=Mastacembelus armatus OX=205130 PE=3 SV=1 |
| 4 | 71743 | tr\|A0A7N8Y7I9\|A0A7N8Y7I9\_9TELE | 57.11 | 4 | 4 | 4 | N | 105242 | Calcium-transporting ATPase OS=Mastacembelus armatus OX=205130 PE=3 SV=1 |
| 4 | 71744 | tr\|A0A671YJD0\|A0A671YJD0\_SPAAU | 57.11 | 4 | 4 | 4 | N | 105314 | Calcium-transporting ATPase OS=Sparus aurata OX=8175 GN=LOC115570920 PE=3 SV=1 |
| 4 | 71745 | tr\|A0A7N8XW64\|A0A7N8XW64\_9TELE | 57.11 | 3 | 4 | 4 | N | 108576 | Calcium-transporting ATPase OS=Mastacembelus armatus OX=205130 PE=3 SV=1 |
| 4 | 71746 | tr\|A0A7N9AUZ7\|A0A7N9AUZ7\_9TELE | 57.11 | 3 | 4 | 4 | N | 109022 | Calcium-transporting ATPase OS=Mastacembelus armatus OX=205130 PE=3 SV=1 |
| 4 | 71747 | tr\|A0A3Q3MF18\|A0A3Q3MF18\_9TELE | 57.11 | 3 | 4 | 4 | N | 109268 | Calcium-transporting ATPase OS=Mastacembelus armatus OX=205130 PE=3 SV=2 |
| 4 | 71748 | tr\|A0A672IVR0\|A0A672IVR0\_SALFA | 57.11 | 3 | 4 | 4 | N | 106289 | Calcium-transporting ATPase OS=Salarias fasciatus OX=181472 GN=LOC115392776 PE=3 SV=1 |
| 4 | 71749 | tr\|A0A3Q1BZF5\|A0A3Q1BZF5\_AMPOC | 57.11 | 3 | 4 | 4 | N | 108248 | Calcium-transporting ATPase OS=Amphiprion ocellaris OX=80972 PE=3 SV=1 |
| 4 | 71750 | tr\|A0A3Q0SE88\|A0A3Q0SE88\_AMPCI | 57.11 | 3 | 4 | 4 | N | 108313 | Calcium-transporting ATPase OS=Amphilophus citrinellus OX=61819 PE=3 SV=1 |
| 4 | 67759 | tr\|A0A3P8SU98\|A0A3P8SU98\_AMPPE | 57.11 | 3 | 4 | 4 | N | 108831 | Calcium-transporting ATPase OS=Amphiprion percula OX=161767 PE=3 SV=1 |
| 4 | 71751 | tr\|A0A672IVP1\|A0A672IVP1\_SALFA | 57.11 | 3 | 4 | 4 | N | 108522 | Calcium-transporting ATPase OS=Salarias fasciatus OX=181472 GN=LOC115392776 PE=3 SV=1 |
| 4 | 71752 | tr\|A0A3Q1BVL9\|A0A3Q1BVL9\_AMPOC | 57.11 | 3 | 4 | 4 | N | 108858 | Calcium-transporting ATPase OS=Amphiprion ocellaris OX=80972 PE=3 SV=1 |
| 4 | 71753 | tr\|A0A672ITB5\|A0A672ITB5\_SALFA | 57.11 | 3 | 4 | 4 | N | 109974 | Calcium-transporting ATPase OS=Salarias fasciatus OX=181472 GN=LOC115392776 PE=3 SV=1 |
| 4 | 71754 | tr\|A0A672IVQ1\|A0A672IVQ1\_SALFA | 57.11 | 3 | 4 | 4 | N | 112146 | Calcium-transporting ATPase OS=Salarias fasciatus OX=181472 GN=LOC115392776 PE=3 SV=1 |
| 6 | 69471 | tr\|A0A834CTG4\|A0A834CTG4\_ORYME | 52.57 | 1 | 3 | 2 | N | 850198 | Titin OS=Oryzias melastigma OX=30732 GN=FQA47\_016182 PE=4 SV=1 |
| 6 | 67005 | tr\|Q4RE90\|Q4RE90\_TETNG | 52.57 | 1 | 3 | 2 | N | 863297 | Chromosome undetermined SCAF15134, whole genome shotgun sequence (Fragment) OS=Tetraodon nigroviridis OX=99883 GN=GSTENG00035871001 PE=4 SV=1 |
| 3 | 67126 | tr\|A0A3P8Q9R4\|A0A3P8Q9R4\_ASTCA | 50.61 | 12 | 5 | 5 | N | 42650 | Creatine kinase OS=Astatotilapia calliptera OX=8154 GN=CKM PE=3 SV=1 |
| 3 | 67128 | tr\|A0A3Q4HL72\|A0A3Q4HL72\_NEOBR | 50.61 | 12 | 5 | 5 | N | 42778 | Creatine kinase OS=Neolamprologus brichardi OX=32507 PE=3 SV=1 |
| 3 | 67136 | tr\|A0A3B4TA77\|A0A3B4TA77\_SERDU | 50.61 | 12 | 5 | 5 | N | 42546 | Creatine kinase OS=Seriola dumerili OX=41447 GN=CKM PE=3 SV=1 |
| 3 | 67124 | tr\|A0A0F8AN20\|A0A0F8AN20\_LARCR | 50.61 | 12 | 5 | 5 | N | 42543 | Creatine kinase OS=Larimichthys crocea OX=215358 GN=EH28\_08927 PE=3 SV=1 |
| 3 | 67115 | tr\|C7ASM1\|C7ASM1\_SINCH | 50.61 | 12 | 5 | 5 | N | 42922 | Creatine kinase OS=Siniperca chuatsi OX=119488 PE=2 SV=1 |
| 3 | 67690 | tr\|G3PFX6\|G3PFX6\_GASAC | 50.61 | 11 | 5 | 5 | N | 46217 | Creatine kinase OS=Gasterosteus aculeatus OX=69293 GN=CKM PE=3 SV=1 |
| 3 | 67691 | tr\|A0A6P7JDS0\|A0A6P7JDS0\_9TELE | 50.61 | 10 | 5 | 5 | N | 51567 | Creatine kinase OS=Parambassis ranga OX=210632 GN=LOC114444487 PE=3 SV=1 |
| 5 | 67285 | tr\|A0A672MA11\|A0A672MA11\_SINGR | 47.78 | 17 | 3 | 3 | N | 16048 | Myosin regulatory light chain 2, skeletal muscle isoform type 2 OS=Sinocyclocheilus grahami OX=75366 GN=LOC107571443 PE=4 SV=1 |
| 5 | 67157 | tr\|A0A4U5VU95\|A0A4U5VU95\_COLLU | 47.78 | 16 | 3 | 3 | N | 16791 | Myosin regulatory light chain 2, skeletal muscle isoform DTNB OS=Collichthys lucidus OX=240159 GN=D9C73\_025504 PE=4 SV=1 |
| 5 | 67288 | tr\|A0A3B4BW75\|A0A3B4BW75\_PYGNA | 47.78 | 15 | 3 | 3 | N | 18272 | Myosin regulatory light chain 2, skeletal muscle isoform type 2 OS=Pygocentrus nattereri OX=42514 PE=4 SV=1 |
| 5 | 67295 | tr\|A0A7J6CHK6\|A0A7J6CHK6\_9TELE | 47.78 | 14 | 3 | 3 | N | 19163 | EF-hand domain-containing protein OS=Onychostoma macrolepis OX=369639 GN=G5714\_012740 PE=4 SV=1 |
| 5 | 67158 | tr\|A0A6P7II88\|A0A6P7II88\_9TELE | 47.78 | 14 | 3 | 3 | N | 18948 | myosin regulatory light chain 2, skeletal muscle isoform OS=Parambassis ranga OX=210632 GN=mylpf PE=4 SV=1 |
| 5 | 67303 | tr\|A0A668VT67\|A0A668VT67\_OREAU | 47.78 | 14 | 3 | 3 | N | 19739 | Myosin light chain, phosphorylatable, fast skeletal muscle b OS=Oreochromis aureus OX=47969 PE=4 SV=1 |
| 5 | 67300 | tr\|F8U029\|F8U029\_EPIBR | 47.78 | 14 | 3 | 3 | N | 18965 | Myosin light chain 2 (Fragment) OS=Epinephelus bruneus OX=323802 PE=2 SV=1 |
| 5 | 67308 | tr\|A0A3B5RBY4\|A0A3B5RBY4\_XIPMA | 47.78 | 10 | 3 | 3 | N | 27929 | Uncharacterized protein OS=Xiphophorus maculatus OX=8083 PE=4 SV=1 |
| 16 | 71774 | tr\|A0A7J5Y2W4\|A0A7J5Y2W4\_DISMA | 45.71 | 1 | 2 | 1 | N | 179397 | Uncharacterized protein OS=Dissostichus mawsoni OX=36200 GN=F7725\_002633 PE=4 SV=1 |
| 16 | 71780 | tr\|A0A4Z2HXD4\|A0A4Z2HXD4\_9TELE | 45.71 | 1 | 2 | 1 | N | 208336 | Titin OS=Liparis tanakae OX=230148 GN=TTN\_5 PE=4 SV=1 |
| 11 | 67382 | tr\|A0A672HWD2\|A0A672HWD2\_SALFA | 44.38 | 6 | 2 | 2 | N | 50618 | Calsequestrin OS=Salarias fasciatus OX=181472 PE=3 SV=1 |
| 11 | 67383 | tr\|A0A672HWL2\|A0A672HWL2\_SALFA | 44.38 | 6 | 2 | 2 | N | 51610 | Calsequestrin OS=Salarias fasciatus OX=181472 PE=3 SV=1 |
| 11 | 67388 | tr\|A0A672HXF4\|A0A672HXF4\_SALFA | 44.38 | 5 | 2 | 2 | N | 52636 | Calsequestrin OS=Salarias fasciatus OX=181472 PE=3 SV=1 |
| 11 | 67393 | tr\|A0A672HXA9\|A0A672HXA9\_SALFA | 44.38 | 5 | 2 | 2 | N | 53688 | Calsequestrin OS=Salarias fasciatus OX=181472 PE=3 SV=1 |
| 15 | 67800 | tr\|A0A3Q4GK85\|A0A3Q4GK85\_NEOBR | 41.12 | 7 | 2 | 2 | N | 21230 | Adenylate kinase isoenzyme 1 OS=Neolamprologus brichardi OX=32507 GN=AK1 PE=3 SV=1 |
| 15 | 67801 | tr\|A0A3Q4GS43\|A0A3Q4GS43\_NEOBR | 41.12 | 7 | 2 | 2 | N | 21240 | Adenylate kinase isoenzyme 1 OS=Neolamprologus brichardi OX=32507 GN=AK1 PE=3 SV=1 |
| 15 | 67802 | tr\|A0A3P8P8S3\|A0A3P8P8S3\_ASTCA | 41.12 | 7 | 2 | 2 | N | 21228 | Adenylate kinase isoenzyme 1 OS=Astatotilapia calliptera OX=8154 GN=AK1 PE=3 SV=1 |
| 15 | 67795 | tr\|A0A3Q1HW42\|A0A3Q1HW42\_9TELE | 41.12 | 7 | 2 | 2 | N | 21205 | Adenylate kinase isoenzyme 1 OS=Acanthochromis polyacanthus OX=80966 GN=AK1 PE=3 SV=1 |
| 15 | 67803 | tr\|A0A3P9CWL7\|A0A3P9CWL7\_9CICH | 41.12 | 7 | 2 | 2 | N | 21258 | Adenylate kinase isoenzyme 1 OS=Maylandia zebra OX=106582 GN=AK1 PE=3 SV=1 |
| 15 | 67796 | tr\|A0A3B5BIY5\|A0A3B5BIY5\_9TELE | 41.12 | 7 | 2 | 2 | N | 21206 | Adenylate kinase isoenzyme 1 OS=Stegastes partitus OX=144197 GN=AK1 PE=3 SV=1 |
| 15 | 67804 | tr\|A0A3Q2VY92\|A0A3Q2VY92\_HAPBU | 41.12 | 7 | 2 | 2 | N | 21228 | Adenylate kinase isoenzyme 1 OS=Haplochromis burtoni OX=8153 GN=AK1 PE=3 SV=1 |
| 15 | 67805 | tr\|A0A3B4FMK3\|A0A3B4FMK3\_9CICH | 41.12 | 7 | 2 | 2 | N | 21228 | Adenylate kinase isoenzyme 1 OS=Pundamilia nyererei OX=303518 GN=AK1 PE=3 SV=1 |
| 15 | 67806 | tr\|A0A3Q3CWR9\|A0A3Q3CWR9\_HAPBU | 41.12 | 7 | 2 | 2 | N | 23163 | Adenylate kinase isoenzyme 1 OS=Haplochromis burtoni OX=8153 GN=AK1 PE=3 SV=1 |
| 15 | 67807 | tr\|A0A3P8P8V3\|A0A3P8P8V3\_ASTCA | 41.12 | 7 | 2 | 2 | N | 23216 | Adenylate kinase isoenzyme 1 OS=Astatotilapia calliptera OX=8154 GN=AK1 PE=3 SV=1 |
| 17 | 69651 | tr\|A0A665WT54\|A0A665WT54\_ECHNA | 38.52 | 8 | 2 | 2 | N | 12976 | LIM domain-binding protein 3-like OS=Echeneis naucrates OX=173247 GN=LOC115060026 PE=4 SV=1 |
| 17 | 69652 | tr\|A0A671N228\|A0A671N228\_9TELE | 38.52 | 4 | 2 | 2 | N | 28684 | LIM domain-binding protein 3-like OS=Sinocyclocheilus anshuiensis OX=1608454 GN=LOC107698793 PE=4 SV=1 |
| 17 | 69653 | tr\|A0A3Q3Q7K2\|A0A3Q3Q7K2\_MONAL | 38.52 | 4 | 2 | 2 | N | 29692 | Synuclein-like OS=Monopterus albus OX=43700 PE=4 SV=1 |
| 17 | 69654 | tr\|A0A671MU99\|A0A671MU99\_9TELE | 38.52 | 4 | 2 | 2 | N | 29538 | LIM domain-binding protein 3-like OS=Sinocyclocheilus anshuiensis OX=1608454 GN=LOC107698793 PE=4 SV=1 |
| 17 | 69655 | tr\|A0A672KK19\|A0A672KK19\_SINGR | 38.52 | 4 | 2 | 2 | N | 30329 | LIM domain-binding protein 3-like OS=Sinocyclocheilus grahami OX=75366 GN=LOC107552745 PE=4 SV=1 |
| 17 | 69656 | tr\|A0A673LYE6\|A0A673LYE6\_9TELE | 38.52 | 4 | 2 | 2 | N | 30316 | LIM domain-binding protein 3-like OS=Sinocyclocheilus rhinocerous OX=307959 GN=LOC107749342 PE=4 SV=1 |
| 17 | 69657 | tr\|A0A3P8VEA1\|A0A3P8VEA1\_CYNSE | 38.52 | 4 | 2 | 2 | N | 30334 | LIM domain-binding protein 3-like OS=Cynoglossus semilaevis OX=244447 PE=4 SV=1 |
| 17 | 69658 | tr\|A0A3Q1II99\|A0A3Q1II99\_ANATE | 38.52 | 4 | 2 | 2 | N | 30310 | LIM domain binding 3b OS=Anabas testudineus OX=64144 PE=4 SV=1 |
| 17 | 69659 | tr\|A0A6P7L9I7\|A0A6P7L9I7\_BETSP | 38.52 | 4 | 2 | 2 | N | 30536 | LIM domain-binding protein 3-like isoform X2 OS=Betta splendens OX=158456 GN=LOC114846412 PE=4 SV=1 |
| 17 | 69660 | tr\|A0A3P8X8T4\|A0A3P8X8T4\_ESOLU | 38.52 | 4 | 2 | 2 | N | 30572 | LIM domain-binding protein 3 OS=Esox lucius OX=8010 PE=4 SV=1 |
| 17 | 69661 | tr\|A0A3Q0R4X6\|A0A3Q0R4X6\_AMPCI | 38.52 | 4 | 2 | 2 | N | 30403 | LIM domain binding 3b OS=Amphilophus citrinellus OX=61819 PE=4 SV=1 |
| 17 | 69662 | tr\|A0A3P8VK01\|A0A3P8VK01\_CYNSE | 38.52 | 4 | 2 | 2 | N | 30591 | LIM domain-binding protein 3-like OS=Cynoglossus semilaevis OX=244447 PE=4 SV=1 |
| 17 | 69663 | tr\|A0A3P8XBI5\|A0A3P8XBI5\_ESOLU | 38.52 | 4 | 2 | 2 | N | 30957 | LIM domain-binding protein 3 OS=Esox lucius OX=8010 PE=4 SV=2 |
| 17 | 69664 | tr\|A0A3Q1H919\|A0A3Q1H919\_ANATE | 38.52 | 3 | 2 | 2 | N | 31065 | LIM domain binding 3b OS=Anabas testudineus OX=64144 PE=4 SV=1 |
| 17 | 69665 | tr\|A0A3Q3KN21\|A0A3Q3KN21\_9TELE | 38.52 | 3 | 2 | 2 | N | 31352 | LIM domain binding 3b OS=Mastacembelus armatus OX=205130 PE=4 SV=2 |
| 17 | 69666 | tr\|A0A6P7L9I9\|A0A6P7L9I9\_BETSP | 38.52 | 3 | 2 | 2 | N | 31290 | LIM domain-binding protein 3-like isoform X1 OS=Betta splendens OX=158456 GN=LOC114846412 PE=4 SV=1 |
| 17 | 69667 | tr\|A0A3P8VE68\|A0A3P8VE68\_CYNSE | 38.52 | 3 | 2 | 2 | N | 31336 | LIM domain-binding protein 3-like OS=Cynoglossus semilaevis OX=244447 PE=4 SV=1 |
| 17 | 69668 | tr\|A0A6G0HYN5\|A0A6G0HYN5\_LARCR | 38.52 | 3 | 2 | 2 | N | 31123 | LIM domain-binding protein 3 Protein cypher Z-band alternatively spliced PDZ-motif protein OS=Larimichthys crocea OX=215358 GN=D5F01\_LYC17526 PE=4 SV=1 |
| 17 | 69669 | tr\|A0A3P8ZCR8\|A0A3P8ZCR8\_ESOLU | 38.52 | 3 | 2 | 2 | N | 31398 | LIM domain-binding protein 3 OS=Esox lucius OX=8010 PE=4 SV=1 |
| 17 | 69670 | tr\|A0A3Q3IQV7\|A0A3Q3IQV7\_MONAL | 38.52 | 3 | 2 | 2 | N | 31456 | Synuclein-like OS=Monopterus albus OX=43700 PE=4 SV=1 |
| 17 | 69671 | tr\|A0A665WTS5\|A0A665WTS5\_ECHNA | 38.52 | 3 | 2 | 2 | N | 31290 | LIM domain-binding protein 3-like OS=Echeneis naucrates OX=173247 GN=LOC115060026 PE=4 SV=1 |
| 17 | 69672 | tr\|A0A7N8Y585\|A0A7N8Y585\_9TELE | 38.52 | 3 | 2 | 2 | N | 31804 | LIM domain binding 3b OS=Mastacembelus armatus OX=205130 PE=4 SV=1 |
| 17 | 69673 | tr\|A0A6P6QFT2\|A0A6P6QFT2\_CARAU | 38.52 | 3 | 2 | 2 | N | 31663 | LIM domain-binding protein 3-like isoform X2 OS=Carassius auratus OX=7957 GN=LOC113111546 PE=4 SV=1 |
| 17 | 69674 | tr\|A0A672KNU9\|A0A672KNU9\_SINGR | 38.52 | 3 | 2 | 2 | N | 31638 | LIM domain-binding protein 3-like OS=Sinocyclocheilus grahami OX=75366 GN=LOC107552745 PE=4 SV=1 |
| 17 | 69675 | tr\|A0A673M1Q3\|A0A673M1Q3\_9TELE | 38.52 | 3 | 2 | 2 | N | 31625 | LIM domain-binding protein 3-like OS=Sinocyclocheilus rhinocerous OX=307959 GN=LOC107749342 PE=4 SV=1 |
| 17 | 69676 | tr\|A0A7J6CGV6\|A0A7J6CGV6\_9TELE | 38.52 | 3 | 2 | 2 | N | 31765 | PDZ domain-containing protein OS=Onychostoma macrolepis OX=369639 GN=G5714\_012537 PE=4 SV=1 |
| 17 | 69677 | tr\|A0A6P6QIW1\|A0A6P6QIW1\_CARAU | 38.52 | 3 | 2 | 2 | N | 34204 | LIM domain-binding protein 3-like isoform X1 OS=Carassius auratus OX=7957 GN=LOC113111546 PE=4 SV=1 |
| 17 | 69678 | tr\|A0A6Q2XWF7\|A0A6Q2XWF7\_ESOLU | 38.52 | 3 | 2 | 2 | N | 35095 | LIM domain-binding protein 3 OS=Esox lucius OX=8010 PE=4 SV=1 |
| 17 | 69679 | tr\|A0A0F8AMH6\|A0A0F8AMH6\_LARCR | 38.52 | 2 | 2 | 2 | N | 43721 | LIM domain-binding protein 3 OS=Larimichthys crocea OX=215358 GN=EH28\_03737 PE=4 SV=1 |
| 17 | 69680 | tr\|A0A6G0HYU6\|A0A6G0HYU6\_LARCR | 38.52 | 1 | 2 | 2 | N | 80212 | Melanopsin-A Mammalian-like melanopsin Melanopsin-M Opsin-4-A Opsin-4M OS=Larimichthys crocea OX=215358 GN=D5F01\_LYC17520 PE=4 SV=1 |
| 10 | 67490 | tr\|G3N515\|G3N515\_GASAC | 35.73 | 1 | 2 | 1 | N | 451257 | Uncharacterized protein OS=Gasterosteus aculeatus OX=69293 PE=4 SV=1 |
| 20 | 71775 | tr\|A0A3P8T7F6\|A0A3P8T7F6\_AMPPE | 34.93 | 8 | 1 | 1 | N | 20468 | Myosin, light chain 1, alkali; skeletal, fast OS=Amphiprion percula OX=161767 PE=4 SV=1 |
| 20 | 71776 | tr\|A0A3Q1AWA1\|A0A3Q1AWA1\_AMPOC | 34.93 | 8 | 1 | 1 | N | 20668 | Myosin light chain 1, skeletal muscle isoform OS=Amphiprion ocellaris OX=80972 PE=4 SV=1 |
| 20 | 71777 | tr\|A0A3P8T5J7\|A0A3P8T5J7\_AMPPE | 34.93 | 8 | 1 | 1 | N | 20668 | Myosin, light chain 1, alkali; skeletal, fast OS=Amphiprion percula OX=161767 PE=4 SV=1 |
| 20 | 71778 | tr\|A0A3P8T7H6\|A0A3P8T7H6\_AMPPE | 34.93 | 8 | 1 | 1 | N | 20912 | Myosin, light chain 1, alkali; skeletal, fast OS=Amphiprion percula OX=161767 PE=4 SV=1 |
| 20 | 71779 | tr\|A0A3Q1B6V7\|A0A3Q1B6V7\_AMPOC | 34.93 | 8 | 1 | 1 | N | 20912 | Myosin light chain 1, skeletal muscle isoform OS=Amphiprion ocellaris OX=80972 PE=4 SV=1 |
| 18 | 67404 | tr\|A0A6F9CEX9\|A0A6F9CEX9\_9TELE | 34.39 | 0 | 1 | 1 | N | 1022296 | Uncharacterized protein OS=Coregonus sp. 'balchen' OX=861768 GN=CSTEINMANNI\_LOCUS3957823 PE=4 SV=1 |
| 7 | 67341 | tr\|A0A484CUS5\|A0A484CUS5\_PERFV | 34.12 | 0 | 2 | 1 | N | 867139 | Uncharacterized protein OS=Perca flavescens OX=8167 GN=EPR50\_G00113850 PE=4 SV=1 |
| 19 | 69536 | tr\|A0A834CMG6\|A0A834CMG6\_ORYME | 32.89 | 1 | 1 | 1 | N | 157078 | Myomesin-2 OS=Oryzias melastigma OX=30732 GN=FQA47\_006815 PE=4 SV=1 |
| 19 | 69537 | tr\|A0A3B3BCL7\|A0A3B3BCL7\_ORYME | 32.89 | 1 | 1 | 1 | N | 157034 | Myomesin-2-like OS=Oryzias melastigma OX=30732 PE=4 SV=1 |
| 19 | 69541 | tr\|A0A3B3D3C5\|A0A3B3D3C5\_ORYME | 32.89 | 1 | 1 | 1 | N | 157566 | Myomesin-2-like OS=Oryzias melastigma OX=30732 PE=4 SV=1 |
| 19 | 69520 | tr\|A0A672ZI78\|A0A672ZI78\_9TELE | 32.89 | 1 | 1 | 1 | N | 126360 | Uncharacterized protein OS=Sphaeramia orbicularis OX=375764 GN=LOC115437523 PE=4 SV=1 |
| 19 | 69521 | tr\|A0A669ELF6\|A0A669ELF6\_ORENI | 32.89 | 1 | 1 | 1 | N | 132152 | Uncharacterized protein OS=Oreochromis niloticus OX=8128 GN=LOC100698303 PE=4 SV=1 |
| 19 | 71781 | tr\|A0A671Y3Y8\|A0A671Y3Y8\_SPAAU | 32.89 | 1 | 1 | 1 | N | 132216 | Myomesin-2-like OS=Sparus aurata OX=8175 GN=MYOM2 PE=4 SV=1 |
| 19 | 69522 | tr\|A0A4U5U6X9\|A0A4U5U6X9\_COLLU | 32.89 | 1 | 1 | 1 | N | 136304 | Myomesin-2 165 kDa connectin-associated protein 165 kDa titin-associated protein M-protein OS=Collichthys lucidus OX=240159 GN=D9C73\_004091 PE=4 SV=1 |
| 19 | 69524 | tr\|A0A669CKL2\|A0A669CKL2\_ORENI | 32.89 | 1 | 1 | 1 | N | 141158 | Uncharacterized protein OS=Oreochromis niloticus OX=8128 GN=LOC100698303 PE=4 SV=1 |
| 19 | 69525 | tr\|A0A671Y401\|A0A671Y401\_SPAAU | 32.89 | 1 | 1 | 1 | N | 145648 | Myomesin-2-like OS=Sparus aurata OX=8175 GN=MYOM2 PE=4 SV=1 |
| 19 | 69526 | tr\|A0A672FNF5\|A0A672FNF5\_SALFA | 32.89 | 1 | 1 | 1 | N | 148203 | Uncharacterized protein OS=Salarias fasciatus OX=181472 GN=LOC115388674 PE=4 SV=1 |
| 19 | 71782 | tr\|A0A671Y3W3\|A0A671Y3W3\_SPAAU | 32.89 | 1 | 1 | 1 | N | 152686 | Myomesin-2-like OS=Sparus aurata OX=8175 GN=MYOM2 PE=4 SV=1 |
| 19 | 69527 | tr\|A0A6G0J0W8\|A0A6G0J0W8\_LARCR | 32.89 | 1 | 1 | 1 | N | 152797 | Myomesin-2 165 kDa connectin-associated protein OS=Larimichthys crocea OX=215358 GN=D5F01\_LYC03862 PE=4 SV=1 |
| 19 | 69528 | tr\|A0A671Y575\|A0A671Y575\_SPAAU | 32.89 | 1 | 1 | 1 | N | 154320 | Myomesin-2-like OS=Sparus aurata OX=8175 GN=MYOM2 PE=4 SV=1 |
| 19 | 69530 | tr\|A0A6G1P7U8\|A0A6G1P7U8\_9TELE | 32.89 | 1 | 1 | 1 | N | 156806 | Myomesin-2 165 kDa connectin-associated protein OS=Channa argus OX=215402 GN=EXN66\_Car002007 PE=4 SV=1 |
| 19 | 69531 | tr\|A0A087YFH0\|A0A087YFH0\_POEFO | 32.89 | 1 | 1 | 1 | N | 156966 | Myomesin-2-like OS=Poecilia formosa OX=48698 GN=MYOM2 PE=4 SV=2 |
| 19 | 69532 | tr\|A0A3P9PGW1\|A0A3P9PGW1\_POERE | 32.89 | 1 | 1 | 1 | N | 156917 | Myomesin-2-like OS=Poecilia reticulata OX=8081 PE=4 SV=1 |
| 19 | 69493 | tr\|A0A3P8P4C2\|A0A3P8P4C2\_ASTCA | 32.89 | 1 | 1 | 1 | N | 157122 | Myomesin-2-like OS=Astatotilapia calliptera OX=8154 GN=MYOM2 PE=4 SV=1 |
| 19 | 69494 | tr\|A0A3Q1D109\|A0A3Q1D109\_AMPOC | 32.89 | 1 | 1 | 1 | N | 156426 | Myomesin-2-like OS=Amphiprion ocellaris OX=80972 GN=MYOM2 PE=4 SV=1 |
| 19 | 69495 | tr\|A0A3Q2WWP1\|A0A3Q2WWP1\_HAPBU | 32.89 | 1 | 1 | 1 | N | 156996 | Myomesin-2-like OS=Haplochromis burtoni OX=8153 PE=4 SV=1 |
| 19 | 69533 | tr\|A0A672ZHV0\|A0A672ZHV0\_9TELE | 32.89 | 1 | 1 | 1 | N | 156079 | Uncharacterized protein OS=Sphaeramia orbicularis OX=375764 GN=LOC115437523 PE=4 SV=1 |
| 19 | 69534 | tr\|I3JDJ1\|I3JDJ1\_ORENI | 32.89 | 1 | 1 | 1 | N | 157148 | Uncharacterized protein OS=Oreochromis niloticus OX=8128 GN=LOC100698303 PE=4 SV=2 |
| 19 | 69535 | tr\|A0A2I4AP94\|A0A2I4AP94\_9TELE | 32.89 | 1 | 1 | 1 | N | 156788 | M-protein, striated muscle-like OS=Austrofundulus limnaeus OX=52670 GN=LOC106513170 PE=4 SV=1 |
| 19 | 69540 | tr\|A0A3P9LTE0\|A0A3P9LTE0\_ORYLA | 32.89 | 1 | 1 | 1 | N | 157011 | Uncharacterized protein OS=Oryzias latipes OX=8090 PE=4 SV=1 |
| 19 | 69542 | tr\|A0A6P7LYN0\|A0A6P7LYN0\_BETSP | 32.89 | 1 | 1 | 1 | N | 158082 | myomesin-2-like isoform X1 OS=Betta splendens OX=158456 GN=LOC114852036 PE=4 SV=1 |
| 19 | 69519 | tr\|A0A3Q4GHK3\|A0A3Q4GHK3\_NEOBR | 32.89 | 2 | 1 | 1 | N | 99617 | Myomesin-2-like OS=Neolamprologus brichardi OX=32507 GN=MYOM2 PE=4 SV=1 |
| 19 | 69523 | tr\|A0A3Q4GXU7\|A0A3Q4GXU7\_NEOBR | 32.89 | 1 | 1 | 1 | N | 139979 | Myomesin-2-like OS=Neolamprologus brichardi OX=32507 GN=MYOM2 PE=4 SV=1 |
| 19 | 69529 | tr\|A0A3B3IJA0\|A0A3B3IJA0\_ORYLA | 32.89 | 1 | 1 | 1 | N | 155226 | Uncharacterized protein OS=Oryzias latipes OX=8090 GN=LOC101163890 PE=4 SV=1 |
| 19 | 69538 | tr\|A0A3P9IT90\|A0A3P9IT90\_ORYLA | 32.89 | 1 | 1 | 1 | N | 156872 | Uncharacterized protein OS=Oryzias latipes OX=8090 PE=4 SV=1 |
| 19 | 69539 | tr\|H2M2B3\|H2M2B3\_ORYLA | 32.89 | 1 | 1 | 1 | N | 156953 | Uncharacterized protein OS=Oryzias latipes OX=8090 GN=LOC101163890 PE=4 SV=2 |
| 19 | 69543 | tr\|A0A3B3HWS2\|A0A3B3HWS2\_ORYLA | 32.89 | 1 | 1 | 1 | N | 158285 | Uncharacterized protein OS=Oryzias latipes OX=8090 GN=LOC101163890 PE=4 SV=1 |
| 12 | 71846 | tr\|A0A6G0J5U6\|A0A6G0J5U6\_LARCR | 31.94 | 1 | 2 | 2 | N | 364928 | Titin OS=Larimichthys crocea OX=215358 GN=D5F01\_LYC01469 PE=4 SV=1 |
| 8 | 67357 | tr\|A0A6A5F2V4\|A0A6A5F2V4\_PERFL | 31.73 | 0 | 2 | 1 | N | 1083091 | Uncharacterized protein OS=Perca fluviatilis OX=8168 GN=PFLUV\_G00150840 PE=4 SV=1 |
| 25 | 71784 | tr\|A0A7N6BJ85\|A0A7N6BJ85\_ANATE | 29.58 | 2 | 1 | 1 | N | 83676 | Uncharacterized protein OS=Anabas testudineus OX=64144 PE=4 SV=1 |
| 33 | 71792 | tr\|A0A3B3QB10\|A0A3B3QB10\_9TELE | 28.56 | 1 | 1 | 1 | N | 135373 | Uncharacterized protein OS=Paramormyrops kingsleyae OX=1676925 PE=4 SV=1 |
| 34 | 71847 | tr\|A0A668VQH5\|A0A668VQH5\_OREAU | 26.40 | 4 | 1 | 1 | N | 33864 | 2-phospho-D-glycerate hydro-lyase OS=Oreochromis aureus OX=47969 PE=3 SV=1 |
| 34 | 71848 | tr\|A0A3P8Q0W3\|A0A3P8Q0W3\_ASTCA | 26.40 | 3 | 1 | 1 | N | 42382 | 2-phospho-D-glycerate hydro-lyase OS=Astatotilapia calliptera OX=8154 PE=3 SV=1 |
| 34 | 67043 | tr\|F5BZS7\|F5BZS7\_EPIBR | 26.40 | 3 | 1 | 1 | N | 43226 | 2-phospho-D-glycerate hydro-lyase (Fragment) OS=Epinephelus bruneus OX=323802 PE=2 SV=1 |
| 34 | 67478 | tr\|A0A4W4G2T4\|A0A4W4G2T4\_ELEEL | 26.40 | 3 | 1 | 1 | N | 44171 | 2-phospho-D-glycerate hydro-lyase OS=Electrophorus electricus OX=8005 GN=eno1 PE=3 SV=1 |
| 34 | 67479 | tr\|A0A4W4G3J1\|A0A4W4G3J1\_ELEEL | 26.40 | 3 | 1 | 1 | N | 44401 | 2-phospho-D-glycerate hydro-lyase OS=Electrophorus electricus OX=8005 GN=eno1 PE=3 SV=1 |
| 34 | 71849 | tr\|A0A668VQX9\|A0A668VQX9\_OREAU | 26.40 | 3 | 1 | 1 | N | 45670 | 2-phospho-D-glycerate hydro-lyase OS=Oreochromis aureus OX=47969 PE=3 SV=1 |
| 34 | 67422 | tr\|A0A4W4G4V3\|A0A4W4G4V3\_ELEEL | 26.40 | 3 | 1 | 1 | N | 45863 | 2-phospho-D-glycerate hydro-lyase OS=Electrophorus electricus OX=8005 GN=eno3 PE=3 SV=1 |
| 34 | 71850 | tr\|A0A668VU75\|A0A668VU75\_OREAU | 26.40 | 3 | 1 | 1 | N | 46006 | 2-phospho-D-glycerate hydro-lyase OS=Oreochromis aureus OX=47969 PE=3 SV=1 |
| 34 | 67480 | tr\|A0A4W4G338\|A0A4W4G338\_ELEEL | 26.40 | 3 | 1 | 1 | N | 46229 | 2-phospho-D-glycerate hydro-lyase OS=Electrophorus electricus OX=8005 GN=eno1 PE=3 SV=1 |
| 34 | 67481 | tr\|A0A3P9C6I0\|A0A3P9C6I0\_9CICH | 26.40 | 3 | 1 | 1 | N | 46321 | 2-phospho-D-glycerate hydro-lyase OS=Maylandia zebra OX=106582 PE=3 SV=1 |
| 34 | 71851 | tr\|A0A668V8T6\|A0A668V8T6\_OREAU | 26.40 | 3 | 1 | 1 | N | 47001 | 2-phospho-D-glycerate hydro-lyase OS=Oreochromis aureus OX=47969 PE=3 SV=1 |
| 34 | 71852 | tr\|A0A668VTY0\|A0A668VTY0\_OREAU | 26.40 | 3 | 1 | 1 | N | 47197 | 2-phospho-D-glycerate hydro-lyase OS=Oreochromis aureus OX=47969 PE=3 SV=1 |
| 34 | 67891 | tr\|A0A6P3VUG4\|A0A6P3VUG4\_CLUHA | 26.40 | 3 | 1 | 1 | N | 46885 | 2-phospho-D-glycerate hydro-lyase OS=Clupea harengus OX=7950 GN=eno1 PE=3 SV=1 |
| 34 | 67430 | tr\|A0A4W4FGS3\|A0A4W4FGS3\_ELEEL | 26.40 | 3 | 1 | 1 | N | 47188 | 2-phospho-D-glycerate hydro-lyase OS=Electrophorus electricus OX=8005 GN=LOC113568057 PE=3 SV=1 |
| 34 | 67482 | tr\|A0A3P8TV75\|A0A3P8TV75\_AMPPE | 26.40 | 3 | 1 | 1 | N | 47036 | 2-phospho-D-glycerate hydro-lyase OS=Amphiprion percula OX=161767 PE=3 SV=1 |
| 34 | 67483 | tr\|A0A6P7IPP6\|A0A6P7IPP6\_9TELE | 26.40 | 3 | 1 | 1 | N | 46964 | 2-phospho-D-glycerate hydro-lyase OS=Parambassis ranga OX=210632 GN=eno1 PE=3 SV=1 |
| 34 | 67431 | tr\|A0A6P3W3B1\|A0A6P3W3B1\_CLUHA | 26.40 | 3 | 1 | 1 | N | 47370 | 2-phospho-D-glycerate hydro-lyase OS=Clupea harengus OX=7950 GN=LOC105904565 PE=3 SV=1 |
| 34 | 67034 | tr\|A0A3B4CPC9\|A0A3B4CPC9\_PYGNA | 26.40 | 3 | 1 | 1 | N | 47482 | 2-phospho-D-glycerate hydro-lyase OS=Pygocentrus nattereri OX=42514 PE=3 SV=1 |
| 34 | 67484 | tr\|A0A6P3W5E7\|A0A6P3W5E7\_CLUHA | 26.40 | 3 | 1 | 1 | N | 47562 | 2-phospho-D-glycerate hydro-lyase OS=Clupea harengus OX=7950 GN=eno3 PE=3 SV=1 |
| 34 | 67421 | tr\|A0A3P8TVY9\|A0A3P8TVY9\_AMPPE | 26.40 | 3 | 1 | 1 | N | 47574 | 2-phospho-D-glycerate hydro-lyase OS=Amphiprion percula OX=161767 PE=3 SV=1 |
| 34 | 67964 | tr\|A0A6P7P1U1\|A0A6P7P1U1\_BETSP | 26.40 | 3 | 1 | 1 | N | 46869 | 2-phospho-D-glycerate hydro-lyase OS=Betta splendens OX=158456 GN=LOC114866120 PE=3 SV=1 |
| 34 | 67485 | tr\|A0A4W4G3C7\|A0A4W4G3C7\_ELEEL | 26.40 | 3 | 1 | 1 | N | 47371 | 2-phospho-D-glycerate hydro-lyase OS=Electrophorus electricus OX=8005 GN=eno1 PE=3 SV=1 |
| 34 | 67486 | tr\|A0A4W4G3N6\|A0A4W4G3N6\_ELEEL | 26.40 | 3 | 1 | 1 | N | 47770 | 2-phospho-D-glycerate hydro-lyase OS=Electrophorus electricus OX=8005 GN=eno1 PE=3 SV=1 |
| 34 | 67419 | tr\|A0A3P8NTT0\|A0A3P8NTT0\_ASTCA | 26.40 | 2 | 1 | 1 | N | 48687 | 2-phospho-D-glycerate hydro-lyase OS=Astatotilapia calliptera OX=8154 PE=3 SV=1 |
| 34 | 67892 | tr\|A0A6P7MN74\|A0A6P7MN74\_BETSP | 26.40 | 2 | 1 | 1 | N | 49625 | 2-phospho-D-glycerate hydro-lyase OS=Betta splendens OX=158456 GN=LOC114856168 PE=3 SV=1 |
| 34 | 67424 | tr\|A0A3B4ERE5\|A0A3B4ERE5\_PYGNA | 26.40 | 2 | 1 | 1 | N | 49548 | 2-phospho-D-glycerate hydro-lyase OS=Pygocentrus nattereri OX=42514 GN=ENO3 PE=3 SV=1 |
| 34 | 67425 | tr\|A0A3B4DDR1\|A0A3B4DDR1\_PYGNA | 26.40 | 2 | 1 | 1 | N | 51660 | 2-phospho-D-glycerate hydro-lyase OS=Pygocentrus nattereri OX=42514 GN=ENO3 PE=3 SV=1 |
| 34 | 67487 | tr\|A0A7J6BNX1\|A0A7J6BNX1\_9TELE | 26.40 | 2 | 1 | 1 | N | 52093 | 2-phospho-D-glycerate hydro-lyase OS=Onychostoma macrolepis OX=369639 GN=G5714\_022679 PE=3 SV=1 |
| 34 | 71853 | tr\|A0A6P7P064\|A0A6P7P064\_BETSP | 26.40 | 2 | 1 | 1 | N | 53271 | 2-phospho-D-glycerate hydro-lyase OS=Betta splendens OX=158456 GN=LOC114866120 PE=3 SV=1 |
| 26 | 71889 | tr\|A0A5A9PD51\|A0A5A9PD51\_9TELE | 23.02 | 1 | 1 | 1 | N | 89232 | BRCA1-A complex subunit RAP80 OS=Triplophysa tibetana OX=1572043 GN=E1301\_Tti012398 PE=3 SV=1 |
| 23 | 71815 | tr\|A0A6P8GQF7\|A0A6P8GQF7\_CLUHA | 22.74 | 1 | 1 | 1 | N | 186990 | kinase D-interacting substrate of 220 kDa B-like isoform X4 OS=Clupea harengus OX=7950 GN=LOC105907323 PE=4 SV=1 |
| 23 | 71816 | tr\|A0A6P8GCK4\|A0A6P8GCK4\_CLUHA | 22.74 | 1 | 1 | 1 | N | 188190 | kinase D-interacting substrate of 220 kDa B-like isoform X3 OS=Clupea harengus OX=7950 GN=LOC105907323 PE=4 SV=1 |
| 23 | 71817 | tr\|A0A6P8GCW3\|A0A6P8GCW3\_CLUHA | 22.74 | 1 | 1 | 1 | N | 191965 | kinase D-interacting substrate of 220 kDa B-like isoform X2 OS=Clupea harengus OX=7950 GN=LOC105907323 PE=4 SV=1 |
| 23 | 71818 | tr\|A0A6P8GNT0\|A0A6P8GNT0\_CLUHA | 22.74 | 1 | 1 | 1 | N | 193166 | kinase D-interacting substrate of 220 kDa B-like isoform X1 OS=Clupea harengus OX=7950 GN=LOC105907323 PE=4 SV=1 |
| 21 | 71820 | tr\|A0A674PAA1\|A0A674PAA1\_TAKRU | 22.10 | 5 | 1 | 1 | N | 20437 | Uncharacterized protein OS=Takifugu rubripes OX=31033 GN=tpm2 PE=3 SV=1 |
| 21 | 71821 | tr\|A0A4W5K3X5\|A0A4W5K3X5\_9TELE | 22.10 | 3 | 1 | 1 | N | 29693 | Tropomyosin 2 (beta) OS=Hucho hucho OX=62062 PE=3 SV=1 |
| 21 | 71822 | tr\|A0A4W5KNV1\|A0A4W5KNV1\_9TELE | 22.10 | 3 | 1 | 1 | N | 32307 | Tropomyosin 2 (beta) OS=Hucho hucho OX=62062 PE=3 SV=1 |
| 21 | 71823 | tr\|A0A3Q1CSK5\|A0A3Q1CSK5\_AMPOC | 22.10 | 3 | 1 | 1 | N | 32534 | Tropomyosin beta chain OS=Amphiprion ocellaris OX=80972 PE=3 SV=1 |
| 21 | 71824 | tr\|A0A4W5KNI2\|A0A4W5KNI2\_9TELE | 22.10 | 3 | 1 | 1 | N | 32801 | Tropomyosin 2 (beta) OS=Hucho hucho OX=62062 PE=3 SV=1 |
| 21 | 71825 | tr\|A0A6G0HZW7\|A0A6G0HZW7\_LARCR | 22.10 | 3 | 1 | 1 | N | 32504 | Uncharacterized protein OS=Larimichthys crocea OX=215358 GN=D5F01\_LYC16153 PE=3 SV=1 |
| 21 | 71875 | tr\|A0A6J2PEZ1\|A0A6J2PEZ1\_COTGO | 22.10 | 3 | 1 | 1 | N | 32878 | tropomyosin alpha-1 chain-like isoform X3 OS=Cottoperca gobio OX=56716 GN=LOC115005840 PE=3 SV=1 |
| 35 | 67518 | tr\|D2KQG3\|D2KQG3\_SINCH | 20.02 | 11 | 1 | 1 | N | 11617 | Parvalbumin OS=Siniperca chuatsi OX=119488 PE=2 SV=1 |
| 35 | 67519 | tr\|B9VJM3\|B9VJM3\_SINCH | 20.02 | 11 | 1 | 1 | N | 11589 | Parvalbumin OS=Siniperca chuatsi OX=119488 PE=2 SV=1 |
| total 215 proteins |  |  |  |  |  |  |  |  |  |
| --- | --- | --- | --- | --- | --- | --- | --- | --- | --- |

  

tr|A0A3S2MUF9|A0A3S2MUF9\_ORYJA

back to list

  

| Protein Coverage
| Supporting Peptides
|

Protein Coverage:

Supporting Peptides:

| Peptide | Uniq | -10lgP | Mass | ppm | m/z | z | RT | Scan | #Spec | Start | End | PTM |
| --- | --- | --- | --- | --- | --- | --- | --- | --- | --- | --- | --- | --- |
| K.AGFAGDDAPRAVFPS.I | Y | 32.99 | 1476.6997 | -0.9 | 739.3564 | 2 | 12.29 | 1805 | 1 | 21 | 35 |  |
| R.VAPEEHPTLL.T | Y | 29.42 | 1104.5815 | -0.1 | 553.2980 | 2 | 11.68 | 1691 | 1 | 98 | 107 |  |
| G.FAGDDAPRAVFPS.I | Y | 29.26 | 1348.6411 | -0.7 | 675.3274 | 2 | 12.03 | 1756 | 1 | 23 | 35 |  |
| A.GDDAPRAVFPS.I | Y | 28.88 | 1130.5356 | -1.2 | 566.2744 | 2 | 11.46 | 1652 | 1 | 25 | 35 |  |
| K.AGFAGDDAPRA.V | Y | 26.29 | 1046.4780 | -0.6 | 524.2460 | 2 | 10.36 | 1456 | 1 | 21 | 31 |  |
| L.TEAPLNPK.A | Y | 22.76 | 868.4654 | 0.1 | 435.2400 | 2 | 22.88 | 3566 | 17 | 108 | 115 |  |
| L.DAGDGVTH.N | Y | 22.53 | 770.3195 | -0.2 | 386.1669 | 2 | 8.67 | 1162 | 3 | 156 | 163 |  |
| F.AGDDAPRAVFPS.I | Y | 22.15 | 1201.5728 | 0.4 | 601.7939 | 2 | 17.84 | 2753 | 1 | 24 | 35 |  |
| K.IIAPPERKYSVWIGGSIL.A | Y | 20.07 | 1998.1301 | -2.6 | 1000.0698 | 2 | 13.65 | 2050 | 1 | 331 | 348 |  |
| total 9 peptides |  |  |  |  |  |  |  |  |  |  |  |  |
| --- | --- | --- | --- | --- | --- | --- | --- | --- | --- | --- | --- | --- |

tr|F5BZL8|F5BZL8\_EPIBR

back to list

  

| Protein Coverage
| Supporting Peptides
|

Protein Coverage:

Supporting Peptides:

| Peptide | Uniq | -10lgP | Mass | ppm | m/z | z | RT | Scan | #Spec | Start | End | PTM |
| --- | --- | --- | --- | --- | --- | --- | --- | --- | --- | --- | --- | --- |
| K.AGFAGDDAPRAVFPS.I | Y | 32.99 | 1476.6997 | -0.9 | 739.3564 | 2 | 12.29 | 1805 | 1 | 21 | 35 |  |
| R.VAPEEHPTLL.T | Y | 29.42 | 1104.5815 | -0.1 | 553.2980 | 2 | 11.68 | 1691 | 1 | 98 | 107 |  |
| G.FAGDDAPRAVFPS.I | Y | 29.26 | 1348.6411 | -0.7 | 675.3274 | 2 | 12.03 | 1756 | 1 | 23 | 35 |  |
| A.GDDAPRAVFPS.I | Y | 28.88 | 1130.5356 | -1.2 | 566.2744 | 2 | 11.46 | 1652 | 1 | 25 | 35 |  |
| K.AGFAGDDAPRA.V | Y | 26.29 | 1046.4780 | -0.6 | 524.2460 | 2 | 10.36 | 1456 | 1 | 21 | 31 |  |
| L.TEAPLNPK.A | Y | 22.76 | 868.4654 | 0.1 | 435.2400 | 2 | 22.88 | 3566 | 17 | 108 | 115 |  |
| L.DAGDGVTH.N | Y | 22.53 | 770.3195 | -0.2 | 386.1669 | 2 | 8.67 | 1162 | 3 | 156 | 163 |  |
| F.AGDDAPRAVFPS.I | Y | 22.15 | 1201.5728 | 0.4 | 601.7939 | 2 | 17.84 | 2753 | 1 | 24 | 35 |  |
| K.IIAPPERKYSVWIGGSIL.A | Y | 20.07 | 1998.1301 | -2.6 | 1000.0698 | 2 | 13.65 | 2050 | 1 | 331 | 348 |  |
| total 9 peptides |  |  |  |  |  |  |  |  |  |  |  |  |
| --- | --- | --- | --- | --- | --- | --- | --- | --- | --- | --- | --- | --- |

tr|A0A7J6C441|A0A7J6C441\_9TELE

back to list

  

| Protein Coverage
| Supporting Peptides
|

Protein Coverage:

Supporting Peptides:

| Peptide | Uniq | -10lgP | Mass | ppm | m/z | z | RT | Scan | #Spec | Start | End | PTM |
| --- | --- | --- | --- | --- | --- | --- | --- | --- | --- | --- | --- | --- |
| K.AGFAGDDAPRAVFPS.I | Y | 32.99 | 1476.6997 | -0.9 | 739.3564 | 2 | 12.29 | 1805 | 1 | 21 | 35 |  |
| R.VAPEEHPTLL.T | Y | 29.42 | 1104.5815 | -0.1 | 553.2980 | 2 | 11.68 | 1691 | 1 | 98 | 107 |  |
| G.FAGDDAPRAVFPS.I | Y | 29.26 | 1348.6411 | -0.7 | 675.3274 | 2 | 12.03 | 1756 | 1 | 23 | 35 |  |
| A.GDDAPRAVFPS.I | Y | 28.88 | 1130.5356 | -1.2 | 566.2744 | 2 | 11.46 | 1652 | 1 | 25 | 35 |  |
| K.AGFAGDDAPRA.V | Y | 26.29 | 1046.4780 | -0.6 | 524.2460 | 2 | 10.36 | 1456 | 1 | 21 | 31 |  |
| L.TEAPLNPK.A | Y | 22.76 | 868.4654 | 0.1 | 435.2400 | 2 | 22.88 | 3566 | 17 | 108 | 115 |  |
| L.DAGDGVTH.N | Y | 22.53 | 770.3195 | -0.2 | 386.1669 | 2 | 8.67 | 1162 | 3 | 156 | 163 |  |
| F.AGDDAPRAVFPS.I | Y | 22.15 | 1201.5728 | 0.4 | 601.7939 | 2 | 17.84 | 2753 | 1 | 24 | 35 |  |
| K.IIAPPERKYSVWIGGSIL.A | Y | 20.07 | 1998.1301 | -2.6 | 1000.0698 | 2 | 13.65 | 2050 | 1 | 331 | 348 |  |
| total 9 peptides |  |  |  |  |  |  |  |  |  |  |  |  |
| --- | --- | --- | --- | --- | --- | --- | --- | --- | --- | --- | --- | --- |

tr|A0A6P7M219|A0A6P7M219\_BETSP

back to list

  

| Protein Coverage
| Supporting Peptides
|

Protein Coverage:

Supporting Peptides:

| Peptide | Uniq | -10lgP | Mass | ppm | m/z | z | RT | Scan | #Spec | Start | End | PTM |
| --- | --- | --- | --- | --- | --- | --- | --- | --- | --- | --- | --- | --- |
| K.AGFAGDDAPRAVFPS.I | Y | 32.99 | 1476.6997 | -0.9 | 739.3564 | 2 | 12.29 | 1805 | 1 | 21 | 35 |  |
| R.VAPEEHPTLL.T | Y | 29.42 | 1104.5815 | -0.1 | 553.2980 | 2 | 11.68 | 1691 | 1 | 98 | 107 |  |
| G.FAGDDAPRAVFPS.I | Y | 29.26 | 1348.6411 | -0.7 | 675.3274 | 2 | 12.03 | 1756 | 1 | 23 | 35 |  |
| A.GDDAPRAVFPS.I | Y | 28.88 | 1130.5356 | -1.2 | 566.2744 | 2 | 11.46 | 1652 | 1 | 25 | 35 |  |
| K.AGFAGDDAPRA.V | Y | 26.29 | 1046.4780 | -0.6 | 524.2460 | 2 | 10.36 | 1456 | 1 | 21 | 31 |  |
| L.TEAPLNPK.A | Y | 22.76 | 868.4654 | 0.1 | 435.2400 | 2 | 22.88 | 3566 | 17 | 108 | 115 |  |
| L.DAGDGVTH.N | Y | 22.53 | 770.3195 | -0.2 | 386.1669 | 2 | 8.67 | 1162 | 3 | 156 | 163 |  |
| F.AGDDAPRAVFPS.I | Y | 22.15 | 1201.5728 | 0.4 | 601.7939 | 2 | 17.84 | 2753 | 1 | 24 | 35 |  |
| K.IIAPPERKYSVWIGGSIL.A | Y | 20.07 | 1998.1301 | -2.6 | 1000.0698 | 2 | 13.65 | 2050 | 1 | 331 | 348 |  |
| total 9 peptides |  |  |  |  |  |  |  |  |  |  |  |  |
| --- | --- | --- | --- | --- | --- | --- | --- | --- | --- | --- | --- | --- |

tr|A0A3P8SGW9|A0A3P8SGW9\_AMPPE

back to list

  

| Protein Coverage
| Supporting Peptides
|

Protein Coverage:

Supporting Peptides:

| Peptide | Uniq | -10lgP | Mass | ppm | m/z | z | RT | Scan | #Spec | Start | End | PTM |
| --- | --- | --- | --- | --- | --- | --- | --- | --- | --- | --- | --- | --- |
| W.LDKNKDPLNDSVVQ.L | Y | 41.85 | 1583.8154 | -0.2 | 792.9148 | 2 | 10.85 | 1542 | 1 | 596 | 609 |  |
| K.KRIEAPPHIF.S | Y | 24.65 | 1206.6873 | -0.7 | 604.3505 | 2 | 11.08 | 1582 | 2 | 147 | 156 |  |
| A.SERVGLL.H | Y | 20.45 | 772.4443 | -0.4 | 387.2292 | 2 | 11.54 | 1667 | 1 | 1709 | 1715 |  |
| W.LDKNKDPLNDS.V | Y | 19.64 | 1257.6201 | -1.1 | 629.8167 | 2 | 9.75 | 1352 | 1 | 596 | 606 |  |
| L.HLDDAVR.G | Y | 19.12 | 824.4141 | -1.0 | 413.2139 | 2 | 9.20 | 1258 | 1 | 1655 | 1661 |  |
| V.GWLDKNKDPLNDS.V | Y | 17.14 | 1500.7208 | -0.5 | 751.3673 | 2 | 11.84 | 1721 | 1 | 594 | 606 |  |
| K.RIEAPPHIF.S | Y | 17.08 | 1078.5923 | -0.7 | 540.3030 | 2 | 11.77 | 1708 | 1 | 148 | 156 |  |
| L.SKIEDEQSL.G | Y | 16.08 | 1047.5084 | -0.7 | 524.7611 | 2 | 10.21 | 1432 | 1 | 1091 | 1099 |  |
| F.EKPKPGKGKAE.A | Y | 15.04 | 1167.6611 | 0.4 | 584.8381 | 2 | 5.03 | 627 | 1 | 566 | 576 |  |
| total 9 peptides |  |  |  |  |  |  |  |  |  |  |  |  |
| --- | --- | --- | --- | --- | --- | --- | --- | --- | --- | --- | --- | --- |

tr|A0A6P7IKT0|A0A6P7IKT0\_9TELE

back to list

  

| Protein Coverage
| Supporting Peptides
|

Protein Coverage:

Supporting Peptides:

| Peptide | Uniq | -10lgP | Mass | ppm | m/z | z | RT | Scan | #Spec | Start | End | PTM |
| --- | --- | --- | --- | --- | --- | --- | --- | --- | --- | --- | --- | --- |
| W.LDKNKDPLNDSVVQ.L | Y | 41.85 | 1583.8154 | -0.2 | 792.9148 | 2 | 10.85 | 1542 | 1 | 596 | 609 |  |
| K.KRIEAPPHIF.S | Y | 24.65 | 1206.6873 | -0.7 | 604.3505 | 2 | 11.08 | 1582 | 2 | 147 | 156 |  |
| A.SERVGLL.H | Y | 20.45 | 772.4443 | -0.4 | 387.2292 | 2 | 11.54 | 1667 | 1 | 1714 | 1720 |  |
| W.LDKNKDPLNDS.V | Y | 19.64 | 1257.6201 | -1.1 | 629.8167 | 2 | 9.75 | 1352 | 1 | 596 | 606 |  |
| L.HLDDAVR.G | Y | 19.12 | 824.4141 | -1.0 | 413.2139 | 2 | 9.20 | 1258 | 1 | 1660 | 1666 |  |
| S.GWLDKNKDPLNDS.V | Y | 17.14 | 1500.7208 | -0.5 | 751.3673 | 2 | 11.84 | 1721 | 1 | 594 | 606 |  |
| K.RIEAPPHIF.S | Y | 17.08 | 1078.5923 | -0.7 | 540.3030 | 2 | 11.77 | 1708 | 1 | 148 | 156 |  |
| L.SKIEDEQSL.G | Y | 16.08 | 1047.5084 | -0.7 | 524.7611 | 2 | 10.21 | 1432 | 1 | 1096 | 1104 |  |
| F.EKPKPGKGKAE.A | Y | 15.04 | 1167.6611 | 0.4 | 584.8381 | 2 | 5.03 | 627 | 1 | 566 | 576 |  |
| total 9 peptides |  |  |  |  |  |  |  |  |  |  |  |  |
| --- | --- | --- | --- | --- | --- | --- | --- | --- | --- | --- | --- | --- |

tr|A0A6P7IXS4|A0A6P7IXS4\_9TELE

back to list

  

| Protein Coverage
| Supporting Peptides
|

Protein Coverage:

Supporting Peptides:

| Peptide | Uniq | -10lgP | Mass | ppm | m/z | z | RT | Scan | #Spec | Start | End | PTM |
| --- | --- | --- | --- | --- | --- | --- | --- | --- | --- | --- | --- | --- |
| W.LDKNKDPLNDSVVQ.L | Y | 41.85 | 1583.8154 | -0.2 | 792.9148 | 2 | 10.85 | 1542 | 1 | 596 | 609 |  |
| K.KRIEAPPHIF.S | Y | 24.65 | 1206.6873 | -0.7 | 604.3505 | 2 | 11.08 | 1582 | 2 | 147 | 156 |  |
| A.SERVGLL.H | Y | 20.45 | 772.4443 | -0.4 | 387.2292 | 2 | 11.54 | 1667 | 1 | 1715 | 1721 |  |
| W.LDKNKDPLNDS.V | Y | 19.64 | 1257.6201 | -1.1 | 629.8167 | 2 | 9.75 | 1352 | 1 | 596 | 606 |  |
| L.HLDDAVR.G | Y | 19.12 | 824.4141 | -1.0 | 413.2139 | 2 | 9.20 | 1258 | 1 | 1661 | 1667 |  |
| S.GWLDKNKDPLNDS.V | Y | 17.14 | 1500.7208 | -0.5 | 751.3673 | 2 | 11.84 | 1721 | 1 | 594 | 606 |  |
| K.RIEAPPHIF.S | Y | 17.08 | 1078.5923 | -0.7 | 540.3030 | 2 | 11.77 | 1708 | 1 | 148 | 156 |  |
| L.SKIEDEQSL.G | Y | 16.08 | 1047.5084 | -0.7 | 524.7611 | 2 | 10.21 | 1432 | 1 | 1097 | 1105 |  |
| F.EKPKPGKGKAE.A | Y | 15.04 | 1167.6611 | 0.4 | 584.8381 | 2 | 5.03 | 627 | 1 | 566 | 576 |  |
| total 9 peptides |  |  |  |  |  |  |  |  |  |  |  |  |
| --- | --- | --- | --- | --- | --- | --- | --- | --- | --- | --- | --- | --- |

tr|A0A3P9CRE7|A0A3P9CRE7\_9CICH

back to list

  

| Protein Coverage
| Supporting Peptides
|

Protein Coverage:

Supporting Peptides:

| Peptide | Uniq | -10lgP | Mass | ppm | m/z | z | RT | Scan | #Spec | Start | End | PTM |
| --- | --- | --- | --- | --- | --- | --- | --- | --- | --- | --- | --- | --- |
| W.LDKNKDPLNDSVVQ.L | Y | 41.85 | 1583.8154 | -0.2 | 792.9148 | 2 | 10.85 | 1542 | 1 | 571 | 584 |  |
| K.KRIEAPPHIF.S | Y | 24.65 | 1206.6873 | -0.7 | 604.3505 | 2 | 11.08 | 1582 | 2 | 140 | 149 |  |
| A.SERVGLL.H | Y | 20.45 | 772.4443 | -0.4 | 387.2292 | 2 | 11.54 | 1667 | 1 | 1682 | 1688 |  |
| W.LDKNKDPLNDS.V | Y | 19.64 | 1257.6201 | -1.1 | 629.8167 | 2 | 9.75 | 1352 | 1 | 571 | 581 |  |
| L.HLDDAVR.G | Y | 19.12 | 824.4141 | -1.0 | 413.2139 | 2 | 9.20 | 1258 | 1 | 1628 | 1634 |  |
| T.GWLDKNKDPLNDS.V | Y | 17.14 | 1500.7208 | -0.5 | 751.3673 | 2 | 11.84 | 1721 | 1 | 569 | 581 |  |
| K.RIEAPPHIF.S | Y | 17.08 | 1078.5923 | -0.7 | 540.3030 | 2 | 11.77 | 1708 | 1 | 141 | 149 |  |
| L.SKIEDEQSL.G | Y | 16.08 | 1047.5084 | -0.7 | 524.7611 | 2 | 10.21 | 1432 | 1 | 1064 | 1072 |  |
| F.EKPKPGKGKAE.A | Y | 15.04 | 1167.6611 | 0.4 | 584.8381 | 2 | 5.03 | 627 | 1 | 541 | 551 |  |
| total 9 peptides |  |  |  |  |  |  |  |  |  |  |  |  |
| --- | --- | --- | --- | --- | --- | --- | --- | --- | --- | --- | --- | --- |

tr|A0A3P8SE64|A0A3P8SE64\_AMPPE

back to list

  

| Protein Coverage
| Supporting Peptides
|

Protein Coverage:

Supporting Peptides:

| Peptide | Uniq | -10lgP | Mass | ppm | m/z | z | RT | Scan | #Spec | Start | End | PTM |
| --- | --- | --- | --- | --- | --- | --- | --- | --- | --- | --- | --- | --- |
| W.LDKNKDPLNDSVVQ.L | Y | 41.85 | 1583.8154 | -0.2 | 792.9148 | 2 | 10.85 | 1542 | 1 | 597 | 610 |  |
| K.KRIEAPPHIF.S | Y | 24.65 | 1206.6873 | -0.7 | 604.3505 | 2 | 11.08 | 1582 | 2 | 147 | 156 |  |
| A.SERVGLL.H | Y | 20.45 | 772.4443 | -0.4 | 387.2292 | 2 | 11.54 | 1667 | 1 | 1709 | 1715 |  |
| W.LDKNKDPLNDS.V | Y | 19.64 | 1257.6201 | -1.1 | 629.8167 | 2 | 9.75 | 1352 | 1 | 597 | 607 |  |
| L.HLDDAVR.G | Y | 19.12 | 824.4141 | -1.0 | 413.2139 | 2 | 9.20 | 1258 | 1 | 1655 | 1661 |  |
| T.GWLDKNKDPLNDS.V | Y | 17.14 | 1500.7208 | -0.5 | 751.3673 | 2 | 11.84 | 1721 | 1 | 595 | 607 |  |
| K.RIEAPPHIF.S | Y | 17.08 | 1078.5923 | -0.7 | 540.3030 | 2 | 11.77 | 1708 | 1 | 148 | 156 |  |
| L.SKIEDEQSL.G | Y | 16.08 | 1047.5084 | -0.7 | 524.7611 | 2 | 10.21 | 1432 | 1 | 1092 | 1100 |  |
| F.EKPKPGKGKAE.A | Y | 15.04 | 1167.6611 | 0.4 | 584.8381 | 2 | 5.03 | 627 | 1 | 567 | 577 |  |
| total 9 peptides |  |  |  |  |  |  |  |  |  |  |  |  |
| --- | --- | --- | --- | --- | --- | --- | --- | --- | --- | --- | --- | --- |

tr|A0A668A9K0|A0A668A9K0\_9TELE

back to list

  

| Protein Coverage
| Supporting Peptides
|

Protein Coverage:

Supporting Peptides:

| Peptide | Uniq | -10lgP | Mass | ppm | m/z | z | RT | Scan | #Spec | Start | End | PTM |
| --- | --- | --- | --- | --- | --- | --- | --- | --- | --- | --- | --- | --- |
| W.LDKNKDPLNDSVVQ.L | Y | 41.85 | 1583.8154 | -0.2 | 792.9148 | 2 | 10.85 | 1542 | 1 | 597 | 610 |  |
| K.KRIEAPPHIF.S | Y | 24.65 | 1206.6873 | -0.7 | 604.3505 | 2 | 11.08 | 1582 | 2 | 147 | 156 |  |
| A.SERVGLL.H | Y | 20.45 | 772.4443 | -0.4 | 387.2292 | 2 | 11.54 | 1667 | 1 | 1712 | 1718 |  |
| W.LDKNKDPLNDS.V | Y | 19.64 | 1257.6201 | -1.1 | 629.8167 | 2 | 9.75 | 1352 | 1 | 597 | 607 |  |
| L.HLDDAVR.G | Y | 19.12 | 824.4141 | -1.0 | 413.2139 | 2 | 9.20 | 1258 | 1 | 1658 | 1664 |  |
| A.GWLDKNKDPLNDS.V | Y | 17.14 | 1500.7208 | -0.5 | 751.3673 | 2 | 11.84 | 1721 | 1 | 595 | 607 |  |
| K.RIEAPPHIF.S | Y | 17.08 | 1078.5923 | -0.7 | 540.3030 | 2 | 11.77 | 1708 | 1 | 148 | 156 |  |
| L.SKIEDEQSL.G | Y | 16.08 | 1047.5084 | -0.7 | 524.7611 | 2 | 10.21 | 1432 | 1 | 1094 | 1102 |  |
| F.EKPKPGKGKAE.A | Y | 15.04 | 1167.6611 | 0.4 | 584.8381 | 2 | 5.03 | 627 | 1 | 567 | 577 |  |
| total 9 peptides |  |  |  |  |  |  |  |  |  |  |  |  |
| --- | --- | --- | --- | --- | --- | --- | --- | --- | --- | --- | --- | --- |

tr|A0A3P8Q1P8|A0A3P8Q1P8\_ASTCA

back to list

  

| Protein Coverage
| Supporting Peptides
|

Protein Coverage:

Supporting Peptides:

| Peptide | Uniq | -10lgP | Mass | ppm | m/z | z | RT | Scan | #Spec | Start | End | PTM |
| --- | --- | --- | --- | --- | --- | --- | --- | --- | --- | --- | --- | --- |
| W.LDKNKDPLNDSVVQ.L | Y | 41.85 | 1583.8154 | -0.2 | 792.9148 | 2 | 10.85 | 1542 | 1 | 596 | 609 |  |
| K.KRIEAPPHIF.S | Y | 24.65 | 1206.6873 | -0.7 | 604.3505 | 2 | 11.08 | 1582 | 2 | 147 | 156 |  |
| A.SERVGLL.H | Y | 20.45 | 772.4443 | -0.4 | 387.2292 | 2 | 11.54 | 1667 | 1 | 1711 | 1717 |  |
| W.LDKNKDPLNDS.V | Y | 19.64 | 1257.6201 | -1.1 | 629.8167 | 2 | 9.75 | 1352 | 1 | 596 | 606 |  |
| L.HLDDAVR.G | Y | 19.12 | 824.4141 | -1.0 | 413.2139 | 2 | 9.20 | 1258 | 1 | 1657 | 1663 |  |
| T.GWLDKNKDPLNDS.V | Y | 17.14 | 1500.7208 | -0.5 | 751.3673 | 2 | 11.84 | 1721 | 1 | 594 | 606 |  |
| K.RIEAPPHIF.S | Y | 17.08 | 1078.5923 | -0.7 | 540.3030 | 2 | 11.77 | 1708 | 1 | 148 | 156 |  |
| L.SKIEDEQSL.G | Y | 16.08 | 1047.5084 | -0.7 | 524.7611 | 2 | 10.21 | 1432 | 1 | 1093 | 1101 |  |
| F.EKPKPGKGKAE.A | Y | 15.04 | 1167.6611 | 0.4 | 584.8381 | 2 | 5.03 | 627 | 1 | 566 | 576 |  |
| total 9 peptides |  |  |  |  |  |  |  |  |  |  |  |  |
| --- | --- | --- | --- | --- | --- | --- | --- | --- | --- | --- | --- | --- |

tr|A0A3P8QMC2|A0A3P8QMC2\_ASTCA

back to list

  

| Protein Coverage
| Supporting Peptides
|

Protein Coverage:

Supporting Peptides:

| Peptide | Uniq | -10lgP | Mass | ppm | m/z | z | RT | Scan | #Spec | Start | End | PTM |
| --- | --- | --- | --- | --- | --- | --- | --- | --- | --- | --- | --- | --- |
| W.LDKNKDPLNDSVVQ.L | Y | 41.85 | 1583.8154 | -0.2 | 792.9148 | 2 | 10.85 | 1542 | 1 | 596 | 609 |  |
| K.KRIEAPPHIF.S | Y | 24.65 | 1206.6873 | -0.7 | 604.3505 | 2 | 11.08 | 1582 | 2 | 147 | 156 |  |
| A.SERVGLL.H | Y | 20.45 | 772.4443 | -0.4 | 387.2292 | 2 | 11.54 | 1667 | 1 | 1708 | 1714 |  |
| W.LDKNKDPLNDS.V | Y | 19.64 | 1257.6201 | -1.1 | 629.8167 | 2 | 9.75 | 1352 | 1 | 596 | 606 |  |
| L.HLDDAVR.G | Y | 19.12 | 824.4141 | -1.0 | 413.2139 | 2 | 9.20 | 1258 | 1 | 1654 | 1660 |  |
| T.GWLDKNKDPLNDS.V | Y | 17.14 | 1500.7208 | -0.5 | 751.3673 | 2 | 11.84 | 1721 | 1 | 594 | 606 |  |
| K.RIEAPPHIF.S | Y | 17.08 | 1078.5923 | -0.7 | 540.3030 | 2 | 11.77 | 1708 | 1 | 148 | 156 |  |
| L.SKIEDEQSL.G | Y | 16.08 | 1047.5084 | -0.7 | 524.7611 | 2 | 10.21 | 1432 | 1 | 1090 | 1098 |  |
| F.EKPKPGKGKAE.A | Y | 15.04 | 1167.6611 | 0.4 | 584.8381 | 2 | 5.03 | 627 | 1 | 566 | 576 |  |
| total 9 peptides |  |  |  |  |  |  |  |  |  |  |  |  |
| --- | --- | --- | --- | --- | --- | --- | --- | --- | --- | --- | --- | --- |

tr|A0A671XUP5|A0A671XUP5\_SPAAU

back to list

  

| Protein Coverage
| Supporting Peptides
|

Protein Coverage:

Supporting Peptides:

| Peptide | Uniq | -10lgP | Mass | ppm | m/z | z | RT | Scan | #Spec | Start | End | PTM |
| --- | --- | --- | --- | --- | --- | --- | --- | --- | --- | --- | --- | --- |
| W.LDKNKDPLNDSVVQ.L | Y | 41.85 | 1583.8154 | -0.2 | 792.9148 | 2 | 10.85 | 1542 | 1 | 589 | 602 |  |
| K.KRIEAPPHIF.S | Y | 24.65 | 1206.6873 | -0.7 | 604.3505 | 2 | 11.08 | 1582 | 2 | 147 | 156 |  |
| A.SERVGLL.H | Y | 20.45 | 772.4443 | -0.4 | 387.2292 | 2 | 11.54 | 1667 | 1 | 1705 | 1711 |  |
| W.LDKNKDPLNDS.V | Y | 19.64 | 1257.6201 | -1.1 | 629.8167 | 2 | 9.75 | 1352 | 1 | 589 | 599 |  |
| L.HLDDAVR.G | Y | 19.12 | 824.4141 | -1.0 | 413.2139 | 2 | 9.20 | 1258 | 1 | 1651 | 1657 |  |
| N.GWLDKNKDPLNDS.V | Y | 17.14 | 1500.7208 | -0.5 | 751.3673 | 2 | 11.84 | 1721 | 1 | 587 | 599 |  |
| K.RIEAPPHIF.S | Y | 17.08 | 1078.5923 | -0.7 | 540.3030 | 2 | 11.77 | 1708 | 1 | 148 | 156 |  |
| L.SKIEDEQSL.G | Y | 16.08 | 1047.5084 | -0.7 | 524.7611 | 2 | 10.21 | 1432 | 1 | 1087 | 1095 |  |
| F.EKPKPGKGKAE.A | Y | 15.04 | 1167.6611 | 0.4 | 584.8381 | 2 | 5.03 | 627 | 1 | 559 | 569 |  |
| total 9 peptides |  |  |  |  |  |  |  |  |  |  |  |  |
| --- | --- | --- | --- | --- | --- | --- | --- | --- | --- | --- | --- | --- |

tr|A0A3P8Q1G5|A0A3P8Q1G5\_ASTCA

back to list

  

| Protein Coverage
| Supporting Peptides
|

Protein Coverage:

Supporting Peptides:

| Peptide | Uniq | -10lgP | Mass | ppm | m/z | z | RT | Scan | #Spec | Start | End | PTM |
| --- | --- | --- | --- | --- | --- | --- | --- | --- | --- | --- | --- | --- |
| W.LDKNKDPLNDSVVQ.L | Y | 41.85 | 1583.8154 | -0.2 | 792.9148 | 2 | 10.85 | 1542 | 1 | 596 | 609 |  |
| K.KRIEAPPHIF.S | Y | 24.65 | 1206.6873 | -0.7 | 604.3505 | 2 | 11.08 | 1582 | 2 | 147 | 156 |  |
| A.SERVGLL.H | Y | 20.45 | 772.4443 | -0.4 | 387.2292 | 2 | 11.54 | 1667 | 1 | 1709 | 1715 |  |
| W.LDKNKDPLNDS.V | Y | 19.64 | 1257.6201 | -1.1 | 629.8167 | 2 | 9.75 | 1352 | 1 | 596 | 606 |  |
| L.HLDDAVR.G | Y | 19.12 | 824.4141 | -1.0 | 413.2139 | 2 | 9.20 | 1258 | 1 | 1655 | 1661 |  |
| T.GWLDKNKDPLNDS.V | Y | 17.14 | 1500.7208 | -0.5 | 751.3673 | 2 | 11.84 | 1721 | 1 | 594 | 606 |  |
| K.RIEAPPHIF.S | Y | 17.08 | 1078.5923 | -0.7 | 540.3030 | 2 | 11.77 | 1708 | 1 | 148 | 156 |  |
| L.SKIEDEQSL.G | Y | 16.08 | 1047.5084 | -0.7 | 524.7611 | 2 | 10.21 | 1432 | 1 | 1091 | 1099 |  |
| F.EKPKPGKGKAE.A | Y | 15.04 | 1167.6611 | 0.4 | 584.8381 | 2 | 5.03 | 627 | 1 | 566 | 576 |  |
| total 9 peptides |  |  |  |  |  |  |  |  |  |  |  |  |
| --- | --- | --- | --- | --- | --- | --- | --- | --- | --- | --- | --- | --- |

tr|Q90YF6|Q90YF6\_PARFO

back to list

  

| Protein Coverage
| Supporting Peptides
|

Protein Coverage:

Supporting Peptides:

| Peptide | Uniq | -10lgP | Mass | ppm | m/z | z | RT | Scan | #Spec | Start | End | PTM |
| --- | --- | --- | --- | --- | --- | --- | --- | --- | --- | --- | --- | --- |
| W.LDKNKDPLNDSVVQ.L | Y | 41.85 | 1583.8154 | -0.2 | 792.9148 | 2 | 10.85 | 1542 | 1 | 596 | 609 |  |
| K.KRIEAPPHIF.S | Y | 24.65 | 1206.6873 | -0.7 | 604.3505 | 2 | 11.08 | 1582 | 2 | 147 | 156 |  |
| A.SERVGLL.H | Y | 20.45 | 772.4443 | -0.4 | 387.2292 | 2 | 11.54 | 1667 | 1 | 1712 | 1718 |  |
| W.LDKNKDPLNDS.V | Y | 19.64 | 1257.6201 | -1.1 | 629.8167 | 2 | 9.75 | 1352 | 1 | 596 | 606 |  |
| L.HLDDAVR.G | Y | 19.12 | 824.4141 | -1.0 | 413.2139 | 2 | 9.20 | 1258 | 1 | 1658 | 1664 |  |
| S.GWLDKNKDPLNDS.V | Y | 17.14 | 1500.7208 | -0.5 | 751.3673 | 2 | 11.84 | 1721 | 1 | 594 | 606 |  |
| K.RIEAPPHIF.S | Y | 17.08 | 1078.5923 | -0.7 | 540.3030 | 2 | 11.77 | 1708 | 1 | 148 | 156 |  |
| L.SKIEDEQSL.G | Y | 16.08 | 1047.5084 | -0.7 | 524.7611 | 2 | 10.21 | 1432 | 1 | 1094 | 1102 |  |
| F.EKPKPGKGKAE.A | Y | 15.04 | 1167.6611 | 0.4 | 584.8381 | 2 | 5.03 | 627 | 1 | 566 | 576 |  |
| total 9 peptides |  |  |  |  |  |  |  |  |  |  |  |  |
| --- | --- | --- | --- | --- | --- | --- | --- | --- | --- | --- | --- | --- |

tr|A0A3Q3R2B2|A0A3Q3R2B2\_MONAL

back to list

  

| Protein Coverage
| Supporting Peptides
|

Protein Coverage:

Supporting Peptides:

| Peptide | Uniq | -10lgP | Mass | ppm | m/z | z | RT | Scan | #Spec | Start | End | PTM |
| --- | --- | --- | --- | --- | --- | --- | --- | --- | --- | --- | --- | --- |
| A.IIDQDKSGFIE.E | Y | 52.71 | 1263.6346 | 5.1 | 632.8278 | 2 | 11.60 | 1678 | 1 | 50 | 60 |  |
| K.AGDTDGDGKIG.F | Y | 30.16 | 1004.4410 | -0.1 | 503.2278 | 2 | 9.19 | 1256 | 1 | 89 | 99 |  |
| total 2 peptides |  |  |  |  |  |  |  |  |  |  |  |  |
| --- | --- | --- | --- | --- | --- | --- | --- | --- | --- | --- | --- | --- |

tr|A0A3Q3JW30|A0A3Q3JW30\_MONAL

back to list

  

| Protein Coverage
| Supporting Peptides
|

Protein Coverage:

Supporting Peptides:

| Peptide | Uniq | -10lgP | Mass | ppm | m/z | z | RT | Scan | #Spec | Start | End | PTM |
| --- | --- | --- | --- | --- | --- | --- | --- | --- | --- | --- | --- | --- |
| A.IIDQDKSGFIE.E | Y | 52.71 | 1263.6346 | 5.1 | 632.8278 | 2 | 11.60 | 1678 | 1 | 50 | 60 |  |
| K.AGDTDGDGKIG.V | Y | 30.16 | 1004.4410 | -0.1 | 503.2278 | 2 | 9.19 | 1256 | 1 | 89 | 99 |  |
| total 2 peptides |  |  |  |  |  |  |  |  |  |  |  |  |
| --- | --- | --- | --- | --- | --- | --- | --- | --- | --- | --- | --- | --- |

tr|A0A3Q3K7G4|A0A3Q3K7G4\_MONAL

back to list

  

| Protein Coverage
| Supporting Peptides
|

Protein Coverage:

Supporting Peptides:

| Peptide | Uniq | -10lgP | Mass | ppm | m/z | z | RT | Scan | #Spec | Start | End | PTM |
| --- | --- | --- | --- | --- | --- | --- | --- | --- | --- | --- | --- | --- |
| A.IIDQDKSGFIE.E | Y | 52.71 | 1263.6346 | 5.1 | 632.8278 | 2 | 11.60 | 1678 | 1 | 49 | 59 |  |
| K.AGDTDGDGKIG.V | Y | 30.16 | 1004.4410 | -0.1 | 503.2278 | 2 | 9.19 | 1256 | 1 | 87 | 97 |  |
| total 2 peptides |  |  |  |  |  |  |  |  |  |  |  |  |
| --- | --- | --- | --- | --- | --- | --- | --- | --- | --- | --- | --- | --- |

tr|A0A3Q3K945|A0A3Q3K945\_MONAL

back to list

  

| Protein Coverage
| Supporting Peptides
|

Protein Coverage:

Supporting Peptides:

| Peptide | Uniq | -10lgP | Mass | ppm | m/z | z | RT | Scan | #Spec | Start | End | PTM |
| --- | --- | --- | --- | --- | --- | --- | --- | --- | --- | --- | --- | --- |
| A.IIDQDKSGFIE.E | Y | 52.71 | 1263.6346 | 5.1 | 632.8278 | 2 | 11.60 | 1678 | 1 | 50 | 60 |  |
| K.AGDTDGDGKIG.G | Y | 30.16 | 1004.4410 | -0.1 | 503.2278 | 2 | 9.19 | 1256 | 1 | 89 | 99 |  |
| total 2 peptides |  |  |  |  |  |  |  |  |  |  |  |  |
| --- | --- | --- | --- | --- | --- | --- | --- | --- | --- | --- | --- | --- |

tr|H2V1I8|H2V1I8\_TAKRU

back to list

  

| Protein Coverage
| Supporting Peptides
|

Protein Coverage:

Supporting Peptides:

| Peptide | Uniq | -10lgP | Mass | ppm | m/z | z | RT | Scan | #Spec | Start | End | PTM |
| --- | --- | --- | --- | --- | --- | --- | --- | --- | --- | --- | --- | --- |
| G.IIDQDKSGFIE.E | Y | 52.71 | 1263.6346 | 5.1 | 632.8278 | 2 | 11.60 | 1678 | 1 | 50 | 60 |  |
| K.AGDTDGDGKIG.V | Y | 30.16 | 1004.4410 | -0.1 | 503.2278 | 2 | 9.19 | 1256 | 1 | 89 | 99 |  |
| total 2 peptides |  |  |  |  |  |  |  |  |  |  |  |  |
| --- | --- | --- | --- | --- | --- | --- | --- | --- | --- | --- | --- | --- |

tr|A0A455R7U6|A0A455R7U6\_CONMY

back to list

  

| Protein Coverage
| Supporting Peptides
|

Protein Coverage:

Supporting Peptides:

| Peptide | Uniq | -10lgP | Mass | ppm | m/z | z | RT | Scan | #Spec | Start | End | PTM |
| --- | --- | --- | --- | --- | --- | --- | --- | --- | --- | --- | --- | --- |
| D.IIDQDKSGFIE.E | Y | 52.71 | 1263.6346 | 5.1 | 632.8278 | 2 | 11.60 | 1678 | 1 | 48 | 58 |  |
| K.AGDTDGDGKIG.V | Y | 30.16 | 1004.4410 | -0.1 | 503.2278 | 2 | 9.19 | 1256 | 1 | 87 | 97 |  |
| total 2 peptides |  |  |  |  |  |  |  |  |  |  |  |  |
| --- | --- | --- | --- | --- | --- | --- | --- | --- | --- | --- | --- | --- |

tr|A0A4W4ED54|A0A4W4ED54\_ELEEL

back to list

  

| Protein Coverage
| Supporting Peptides
|

Protein Coverage:

Supporting Peptides:

| Peptide | Uniq | -10lgP | Mass | ppm | m/z | z | RT | Scan | #Spec | Start | End | PTM |
| --- | --- | --- | --- | --- | --- | --- | --- | --- | --- | --- | --- | --- |
| A.IIDQDKSGFIE.E | Y | 52.71 | 1263.6346 | 5.1 | 632.8278 | 2 | 11.60 | 1678 | 1 | 50 | 60 |  |
| K.AGDTDGDGKIG.A | Y | 30.16 | 1004.4410 | -0.1 | 503.2278 | 2 | 9.19 | 1256 | 1 | 89 | 99 |  |
| total 2 peptides |  |  |  |  |  |  |  |  |  |  |  |  |
| --- | --- | --- | --- | --- | --- | --- | --- | --- | --- | --- | --- | --- |

tr|A0A6P7N9P3|A0A6P7N9P3\_BETSP

back to list

  

| Protein Coverage
| Supporting Peptides
|

Protein Coverage:

Supporting Peptides:

| Peptide | Uniq | -10lgP | Mass | ppm | m/z | z | RT | Scan | #Spec | Start | End | PTM |
| --- | --- | --- | --- | --- | --- | --- | --- | --- | --- | --- | --- | --- |
| A.IIDQDKSGFIE.E | Y | 52.71 | 1263.6346 | 5.1 | 632.8278 | 2 | 11.60 | 1678 | 1 | 50 | 60 |  |
| Q.AGDTDGDGKIG.A | Y | 30.16 | 1004.4410 | -0.1 | 503.2278 | 2 | 9.19 | 1256 | 1 | 89 | 99 |  |
| total 2 peptides |  |  |  |  |  |  |  |  |  |  |  |  |
| --- | --- | --- | --- | --- | --- | --- | --- | --- | --- | --- | --- | --- |

tr|A0A3B4CKV2|A0A3B4CKV2\_PYGNA

back to list

  

| Protein Coverage
| Supporting Peptides
|

Protein Coverage:

Supporting Peptides:

| Peptide | Uniq | -10lgP | Mass | ppm | m/z | z | RT | Scan | #Spec | Start | End | PTM |
| --- | --- | --- | --- | --- | --- | --- | --- | --- | --- | --- | --- | --- |
| A.IIDQDKSGFIE.E | Y | 52.71 | 1263.6346 | 5.1 | 632.8278 | 2 | 11.60 | 1678 | 1 | 50 | 60 |  |
| K.AGDTDGDGKIG.A | Y | 30.16 | 1004.4410 | -0.1 | 503.2278 | 2 | 9.19 | 1256 | 1 | 89 | 99 |  |
| total 2 peptides |  |  |  |  |  |  |  |  |  |  |  |  |
| --- | --- | --- | --- | --- | --- | --- | --- | --- | --- | --- | --- | --- |

tr|A0A3B4TWQ8|A0A3B4TWQ8\_SERDU

back to list

  

| Protein Coverage
| Supporting Peptides
|

Protein Coverage:

Supporting Peptides:

| Peptide | Uniq | -10lgP | Mass | ppm | m/z | z | RT | Scan | #Spec | Start | End | PTM |
| --- | --- | --- | --- | --- | --- | --- | --- | --- | --- | --- | --- | --- |
| A.IIDQDKSGFIE.E | Y | 52.71 | 1263.6346 | 5.1 | 632.8278 | 2 | 11.60 | 1678 | 1 | 50 | 60 |  |
| K.AGDTDGDGKIG.V | Y | 30.16 | 1004.4410 | -0.1 | 503.2278 | 2 | 9.19 | 1256 | 1 | 89 | 99 |  |
| total 2 peptides |  |  |  |  |  |  |  |  |  |  |  |  |
| --- | --- | --- | --- | --- | --- | --- | --- | --- | --- | --- | --- | --- |

tr|A0A7N8YBR8|A0A7N8YBR8\_9TELE

back to list

  

| Protein Coverage
| Supporting Peptides
|

Protein Coverage:

Supporting Peptides:

| Peptide | Uniq | -10lgP | Mass | ppm | m/z | z | RT | Scan | #Spec | Start | End | PTM |
| --- | --- | --- | --- | --- | --- | --- | --- | --- | --- | --- | --- | --- |
| A.IIDQDKSGFIE.E | Y | 52.71 | 1263.6346 | 5.1 | 632.8278 | 2 | 11.60 | 1678 | 1 | 50 | 60 |  |
| K.AGDTDGDGKIG.V | Y | 30.16 | 1004.4410 | -0.1 | 503.2278 | 2 | 9.19 | 1256 | 1 | 89 | 99 |  |
| total 2 peptides |  |  |  |  |  |  |  |  |  |  |  |  |
| --- | --- | --- | --- | --- | --- | --- | --- | --- | --- | --- | --- | --- |

tr|A0A3Q3W464|A0A3Q3W464\_MOLML

back to list

  

| Protein Coverage
| Supporting Peptides
|

Protein Coverage:

Supporting Peptides:

| Peptide | Uniq | -10lgP | Mass | ppm | m/z | z | RT | Scan | #Spec | Start | End | PTM |
| --- | --- | --- | --- | --- | --- | --- | --- | --- | --- | --- | --- | --- |
| D.IIDQDKSGFIE.K | Y | 52.71 | 1263.6346 | 5.1 | 632.8278 | 2 | 11.60 | 1678 | 1 | 50 | 60 |  |
| R.AGDTDGDGKIG.A | Y | 30.16 | 1004.4410 | -0.1 | 503.2278 | 2 | 9.19 | 1256 | 1 | 89 | 99 |  |
| total 2 peptides |  |  |  |  |  |  |  |  |  |  |  |  |
| --- | --- | --- | --- | --- | --- | --- | --- | --- | --- | --- | --- | --- |

tr|A0A3Q2WZN1|A0A3Q2WZN1\_HAPBU

back to list

  

| Protein Coverage
| Supporting Peptides
|

Protein Coverage:

Supporting Peptides:

| Peptide | Uniq | -10lgP | Mass | ppm | m/z | z | RT | Scan | #Spec | Start | End | PTM |
| --- | --- | --- | --- | --- | --- | --- | --- | --- | --- | --- | --- | --- |
| A.IIDQDKSGFIE.E | Y | 52.71 | 1263.6346 | 5.1 | 632.8278 | 2 | 11.60 | 1678 | 1 | 50 | 60 |  |
| G.AGDTDGDGKIG.A | Y | 30.16 | 1004.4410 | -0.1 | 503.2278 | 2 | 9.19 | 1256 | 1 | 89 | 99 |  |
| total 2 peptides |  |  |  |  |  |  |  |  |  |  |  |  |
| --- | --- | --- | --- | --- | --- | --- | --- | --- | --- | --- | --- | --- |

tr|A0A674NSY1|A0A674NSY1\_TAKRU

back to list

  

| Protein Coverage
| Supporting Peptides
|

Protein Coverage:

Supporting Peptides:

| Peptide | Uniq | -10lgP | Mass | ppm | m/z | z | RT | Scan | #Spec | Start | End | PTM |
| --- | --- | --- | --- | --- | --- | --- | --- | --- | --- | --- | --- | --- |
| G.IIDQDKSGFIE.E | Y | 52.71 | 1263.6346 | 5.1 | 632.8278 | 2 | 11.60 | 1678 | 1 | 58 | 68 |  |
| K.AGDTDGDGKIG.V | Y | 30.16 | 1004.4410 | -0.1 | 503.2278 | 2 | 9.19 | 1256 | 1 | 97 | 107 |  |
| total 2 peptides |  |  |  |  |  |  |  |  |  |  |  |  |
| --- | --- | --- | --- | --- | --- | --- | --- | --- | --- | --- | --- | --- |

tr|A0A3Q3K779|A0A3Q3K779\_MONAL

back to list

  

| Protein Coverage
| Supporting Peptides
|

Protein Coverage:

Supporting Peptides:

| Peptide | Uniq | -10lgP | Mass | ppm | m/z | z | RT | Scan | #Spec | Start | End | PTM |
| --- | --- | --- | --- | --- | --- | --- | --- | --- | --- | --- | --- | --- |
| A.IIDQDKSGFIE.E | Y | 52.71 | 1263.6346 | 5.1 | 632.8278 | 2 | 11.60 | 1678 | 1 | 50 | 60 |  |
| K.AGDTDGDGKIG.V | Y | 30.16 | 1004.4410 | -0.1 | 503.2278 | 2 | 9.19 | 1256 | 1 | 89 | 99 |  |
| total 2 peptides |  |  |  |  |  |  |  |  |  |  |  |  |
| --- | --- | --- | --- | --- | --- | --- | --- | --- | --- | --- | --- | --- |

tr|I3J1U3|I3J1U3\_ORENI

back to list

  

| Protein Coverage
| Supporting Peptides
|

Protein Coverage:

Supporting Peptides:

| Peptide | Uniq | -10lgP | Mass | ppm | m/z | z | RT | Scan | #Spec | Start | End | PTM |
| --- | --- | --- | --- | --- | --- | --- | --- | --- | --- | --- | --- | --- |
| A.IIDQDKSGFIE.E | Y | 52.71 | 1263.6346 | 5.1 | 632.8278 | 2 | 11.60 | 1678 | 1 | 61 | 71 |  |
| G.AGDTDGDGKIG.A | Y | 30.16 | 1004.4410 | -0.1 | 503.2278 | 2 | 9.19 | 1256 | 1 | 100 | 110 |  |
| total 2 peptides |  |  |  |  |  |  |  |  |  |  |  |  |
| --- | --- | --- | --- | --- | --- | --- | --- | --- | --- | --- | --- | --- |

tr|A0A3Q3WAG3|A0A3Q3WAG3\_MOLML

back to list

  

| Protein Coverage
| Supporting Peptides
|

Protein Coverage:

Supporting Peptides:

| Peptide | Uniq | -10lgP | Mass | ppm | m/z | z | RT | Scan | #Spec | Start | End | PTM |
| --- | --- | --- | --- | --- | --- | --- | --- | --- | --- | --- | --- | --- |
| D.IIDQDKSGFIE.K | Y | 52.71 | 1263.6346 | 5.1 | 632.8278 | 2 | 11.60 | 1678 | 1 | 50 | 60 |  |
| R.AGDTDGDGKIG.A | Y | 30.16 | 1004.4410 | -0.1 | 503.2278 | 2 | 9.19 | 1256 | 1 | 89 | 99 |  |
| total 2 peptides |  |  |  |  |  |  |  |  |  |  |  |  |
| --- | --- | --- | --- | --- | --- | --- | --- | --- | --- | --- | --- | --- |

tr|Q804Z0|Q804Z0\_ICTPU

back to list

  

| Protein Coverage
| Supporting Peptides
|

Protein Coverage:

Supporting Peptides:

| Peptide | Uniq | -10lgP | Mass | ppm | m/z | z | RT | Scan | #Spec | Start | End | PTM |
| --- | --- | --- | --- | --- | --- | --- | --- | --- | --- | --- | --- | --- |
| A.IIDQDKSGFIE.E | Y | 52.71 | 1263.6346 | 5.1 | 632.8278 | 2 | 11.60 | 1678 | 1 | 66 | 76 |  |
| K.AGDTDGDGKIG.V | Y | 30.16 | 1004.4410 | -0.1 | 503.2278 | 2 | 9.19 | 1256 | 1 | 105 | 115 |  |
| total 2 peptides |  |  |  |  |  |  |  |  |  |  |  |  |
| --- | --- | --- | --- | --- | --- | --- | --- | --- | --- | --- | --- | --- |

tr|A0A3Q4AW30|A0A3Q4AW30\_MOLML

back to list

  

| Protein Coverage
| Supporting Peptides
|

Protein Coverage:

Supporting Peptides:

| Peptide | Uniq | -10lgP | Mass | ppm | m/z | z | RT | Scan | #Spec | Start | End | PTM |
| --- | --- | --- | --- | --- | --- | --- | --- | --- | --- | --- | --- | --- |
| D.IIDQDKSGFIE.K | Y | 52.71 | 1263.6346 | 5.1 | 632.8278 | 2 | 11.60 | 1678 | 1 | 50 | 60 |  |
| R.AGDTDGDGKIG.A | Y | 30.16 | 1004.4410 | -0.1 | 503.2278 | 2 | 9.19 | 1256 | 1 | 89 | 99 |  |
| total 2 peptides |  |  |  |  |  |  |  |  |  |  |  |  |
| --- | --- | --- | --- | --- | --- | --- | --- | --- | --- | --- | --- | --- |

tr|A0A3Q3W3Y6|A0A3Q3W3Y6\_MOLML

back to list

  

| Protein Coverage
| Supporting Peptides
|

Protein Coverage:

Supporting Peptides:

| Peptide | Uniq | -10lgP | Mass | ppm | m/z | z | RT | Scan | #Spec | Start | End | PTM |
| --- | --- | --- | --- | --- | --- | --- | --- | --- | --- | --- | --- | --- |
| D.IIDQDKSGFIE.K | Y | 52.71 | 1263.6346 | 5.1 | 632.8278 | 2 | 11.60 | 1678 | 1 | 50 | 60 |  |
| R.AGDTDGDGKIG.A | Y | 30.16 | 1004.4410 | -0.1 | 503.2278 | 2 | 9.19 | 1256 | 1 | 89 | 99 |  |
| total 2 peptides |  |  |  |  |  |  |  |  |  |  |  |  |
| --- | --- | --- | --- | --- | --- | --- | --- | --- | --- | --- | --- | --- |

tr|A0A3Q3K924|A0A3Q3K924\_MONAL

back to list

  

| Protein Coverage
| Supporting Peptides
|

Protein Coverage:

Supporting Peptides:

| Peptide | Uniq | -10lgP | Mass | ppm | m/z | z | RT | Scan | #Spec | Start | End | PTM |
| --- | --- | --- | --- | --- | --- | --- | --- | --- | --- | --- | --- | --- |
| A.IIDQDKSGFIE.E | Y | 52.71 | 1263.6346 | 5.1 | 632.8278 | 2 | 11.60 | 1678 | 1 | 50 | 60 |  |
| K.AGDTDGDGKIG.V | Y | 30.16 | 1004.4410 | -0.1 | 503.2278 | 2 | 9.19 | 1256 | 1 | 89 | 99 |  |
| total 2 peptides |  |  |  |  |  |  |  |  |  |  |  |  |
| --- | --- | --- | --- | --- | --- | --- | --- | --- | --- | --- | --- | --- |

tr|A0A674P8J8|A0A674P8J8\_TAKRU

back to list

  

| Protein Coverage
| Supporting Peptides
|

Protein Coverage:

Supporting Peptides:

| Peptide | Uniq | -10lgP | Mass | ppm | m/z | z | RT | Scan | #Spec | Start | End | PTM |
| --- | --- | --- | --- | --- | --- | --- | --- | --- | --- | --- | --- | --- |
| E.IIDQDKSGFIE.E | Y | 52.71 | 1263.6346 | 5.1 | 632.8278 | 2 | 11.60 | 1678 | 1 | 90 | 100 |  |
| K.AGDTDGDGKIG.A | Y | 30.16 | 1004.4410 | -0.1 | 503.2278 | 2 | 9.19 | 1256 | 1 | 129 | 139 |  |
| total 2 peptides |  |  |  |  |  |  |  |  |  |  |  |  |
| --- | --- | --- | --- | --- | --- | --- | --- | --- | --- | --- | --- | --- |

tr|A0A7N9APR6|A0A7N9APR6\_9TELE

back to list

  

| Protein Coverage
| Supporting Peptides
|

Protein Coverage:

Supporting Peptides:

| Peptide | Uniq | -10lgP | Mass | ppm | m/z | z | RT | Scan | #Spec | Start | End | PTM |
| --- | --- | --- | --- | --- | --- | --- | --- | --- | --- | --- | --- | --- |
| A.IIDQDKSGFIE.E | Y | 52.71 | 1263.6346 | 5.1 | 632.8278 | 2 | 11.60 | 1678 | 1 | 50 | 60 |  |
| K.AGDTDGDGKIG.V | Y | 30.16 | 1004.4410 | -0.1 | 503.2278 | 2 | 9.19 | 1256 | 1 | 89 | 99 |  |
| total 2 peptides |  |  |  |  |  |  |  |  |  |  |  |  |
| --- | --- | --- | --- | --- | --- | --- | --- | --- | --- | --- | --- | --- |

tr|A0A3Q3K937|A0A3Q3K937\_MONAL

back to list

  

| Protein Coverage
| Supporting Peptides
|

Protein Coverage:

Supporting Peptides:

| Peptide | Uniq | -10lgP | Mass | ppm | m/z | z | RT | Scan | #Spec | Start | End | PTM |
| --- | --- | --- | --- | --- | --- | --- | --- | --- | --- | --- | --- | --- |
| A.IIDQDKSGFIE.E | Y | 52.71 | 1263.6346 | 5.1 | 632.8278 | 2 | 11.60 | 1678 | 1 | 50 | 60 |  |
| K.AGDTDGDGKIG.T | Y | 30.16 | 1004.4410 | -0.1 | 503.2278 | 2 | 9.19 | 1256 | 1 | 89 | 99 |  |
| total 2 peptides |  |  |  |  |  |  |  |  |  |  |  |  |
| --- | --- | --- | --- | --- | --- | --- | --- | --- | --- | --- | --- | --- |

tr|A0A3Q3JXS6|A0A3Q3JXS6\_MONAL

back to list

  

| Protein Coverage
| Supporting Peptides
|

Protein Coverage:

Supporting Peptides:

| Peptide | Uniq | -10lgP | Mass | ppm | m/z | z | RT | Scan | #Spec | Start | End | PTM |
| --- | --- | --- | --- | --- | --- | --- | --- | --- | --- | --- | --- | --- |
| A.IIDQDKSGFIE.E | Y | 52.71 | 1263.6346 | 5.1 | 632.8278 | 2 | 11.60 | 1678 | 1 | 50 | 60 |  |
| K.AGDTDGDGKIG.H | Y | 30.16 | 1004.4410 | -0.1 | 503.2278 | 2 | 9.19 | 1256 | 1 | 89 | 99 |  |
| total 2 peptides |  |  |  |  |  |  |  |  |  |  |  |  |
| --- | --- | --- | --- | --- | --- | --- | --- | --- | --- | --- | --- | --- |

tr|H3CFQ5|H3CFQ5\_TETNG

back to list

  

| Protein Coverage
| Supporting Peptides
|

Protein Coverage:

Supporting Peptides:

| Peptide | Uniq | -10lgP | Mass | ppm | m/z | z | RT | Scan | #Spec | Start | End | PTM |
| --- | --- | --- | --- | --- | --- | --- | --- | --- | --- | --- | --- | --- |
| Y.TIPGPPSTPWLGF.V | Y | 45.63 | 1368.7078 | -0.6 | 685.3608 | 2 | 15.15 | 2310 | 1 | 619 | 631 |  |
| Q.GEFVAIGPI.K | Y | 17.46 | 901.4908 | -1.9 | 451.7519 | 2 | 19.18 | 2965 | 2 | 1496 | 1504 |  |
| A.IGPIKVI.D | Y | 15.80 | 738.5003 | -0.1 | 370.2574 | 2 | 12.15 | 1780 | 1 | 1501 | 1507 |  |
| total 3 peptides |  |  |  |  |  |  |  |  |  |  |  |  |
| --- | --- | --- | --- | --- | --- | --- | --- | --- | --- | --- | --- | --- |

tr|M4AVS2|M4AVS2\_XIPMA

back to list

  

| Protein Coverage
| Supporting Peptides
|

Protein Coverage:

Supporting Peptides:

| Peptide | Uniq | -10lgP | Mass | ppm | m/z | z | RT | Scan | #Spec | Start | End | PTM |
| --- | --- | --- | --- | --- | --- | --- | --- | --- | --- | --- | --- | --- |
| L.SENQSLLRMPPWSNF.W | Y | 34.74 | 1804.8567 | 3.7 | 903.4390 | 2 | 15.03 | 2289 | 1 | 912 | 926 |  |
| Q.SLLRMPPWSNF.W | Y | 25.24 | 1346.6805 | -0.7 | 674.3471 | 2 | 15.17 | 2314 | 1 | 916 | 926 |  |
| E.VSVGDKVPA.D | Y | 16.99 | 870.4811 | -1.5 | 436.2472 | 2 | 10.13 | 1418 | 1 | 151 | 159 |  |
| L.GFNPPDLDIM.G | Y | 16.33 | 1117.5114 | 0.4 | 559.7632 | 2 | 14.27 | 2156 | 1 | 803 | 812 |  |
| total 4 peptides |  |  |  |  |  |  |  |  |  |  |  |  |
| --- | --- | --- | --- | --- | --- | --- | --- | --- | --- | --- | --- | --- |

tr|A0A3B3VSS0|A0A3B3VSS0\_9TELE

back to list

  

| Protein Coverage
| Supporting Peptides
|

Protein Coverage:

Supporting Peptides:

| Peptide | Uniq | -10lgP | Mass | ppm | m/z | z | RT | Scan | #Spec | Start | End | PTM |
| --- | --- | --- | --- | --- | --- | --- | --- | --- | --- | --- | --- | --- |
| L.SENQSLLRMPPWSNF.W | Y | 34.74 | 1804.8567 | 3.7 | 903.4390 | 2 | 15.03 | 2289 | 1 | 912 | 926 |  |
| Q.SLLRMPPWSNF.W | Y | 25.24 | 1346.6805 | -0.7 | 674.3471 | 2 | 15.17 | 2314 | 1 | 916 | 926 |  |
| E.VSVGDKVPA.D | Y | 16.99 | 870.4811 | -1.5 | 436.2472 | 2 | 10.13 | 1418 | 1 | 151 | 159 |  |
| L.GFNPPDLDIM.G | Y | 16.33 | 1117.5114 | 0.4 | 559.7632 | 2 | 14.27 | 2156 | 1 | 803 | 812 |  |
| total 4 peptides |  |  |  |  |  |  |  |  |  |  |  |  |
| --- | --- | --- | --- | --- | --- | --- | --- | --- | --- | --- | --- | --- |

tr|A0A0S7JRJ0|A0A0S7JRJ0\_9TELE

back to list

  

| Protein Coverage
| Supporting Peptides
|

Protein Coverage:

Supporting Peptides:

| Peptide | Uniq | -10lgP | Mass | ppm | m/z | z | RT | Scan | #Spec | Start | End | PTM |
| --- | --- | --- | --- | --- | --- | --- | --- | --- | --- | --- | --- | --- |
| L.SENQSLLRMPPWSNF.W | Y | 34.74 | 1804.8567 | 3.7 | 903.4390 | 2 | 15.03 | 2289 | 1 | 914 | 928 |  |
| Q.SLLRMPPWSNF.W | Y | 25.24 | 1346.6805 | -0.7 | 674.3471 | 2 | 15.17 | 2314 | 1 | 918 | 928 |  |
| E.VSVGDKVPA.D | Y | 16.99 | 870.4811 | -1.5 | 436.2472 | 2 | 10.13 | 1418 | 1 | 153 | 161 |  |
| L.GFNPPDLDIM.G | Y | 16.33 | 1117.5114 | 0.4 | 559.7632 | 2 | 14.27 | 2156 | 1 | 805 | 814 |  |
| total 4 peptides |  |  |  |  |  |  |  |  |  |  |  |  |
| --- | --- | --- | --- | --- | --- | --- | --- | --- | --- | --- | --- | --- |

tr|A0A0S7JR62|A0A0S7JR62\_9TELE

back to list

  

| Protein Coverage
| Supporting Peptides
|

Protein Coverage:

Supporting Peptides:

| Peptide | Uniq | -10lgP | Mass | ppm | m/z | z | RT | Scan | #Spec | Start | End | PTM |
| --- | --- | --- | --- | --- | --- | --- | --- | --- | --- | --- | --- | --- |
| L.SENQSLLRMPPWSNF.W | Y | 34.74 | 1804.8567 | 3.7 | 903.4390 | 2 | 15.03 | 2289 | 1 | 914 | 928 |  |
| Q.SLLRMPPWSNF.W | Y | 25.24 | 1346.6805 | -0.7 | 674.3471 | 2 | 15.17 | 2314 | 1 | 918 | 928 |  |
| E.VSVGDKVPA.D | Y | 16.99 | 870.4811 | -1.5 | 436.2472 | 2 | 10.13 | 1418 | 1 | 153 | 161 |  |
| L.GFNPPDLDIM.G | Y | 16.33 | 1117.5114 | 0.4 | 559.7632 | 2 | 14.27 | 2156 | 1 | 805 | 814 |  |
| total 4 peptides |  |  |  |  |  |  |  |  |  |  |  |  |
| --- | --- | --- | --- | --- | --- | --- | --- | --- | --- | --- | --- | --- |

tr|A0A3B5QZF8|A0A3B5QZF8\_XIPMA

back to list

  

| Protein Coverage
| Supporting Peptides
|

Protein Coverage:

Supporting Peptides:

| Peptide | Uniq | -10lgP | Mass | ppm | m/z | z | RT | Scan | #Spec | Start | End | PTM |
| --- | --- | --- | --- | --- | --- | --- | --- | --- | --- | --- | --- | --- |
| L.SENQSLLRMPPWSNF.W | Y | 34.74 | 1804.8567 | 3.7 | 903.4390 | 2 | 15.03 | 2289 | 1 | 914 | 928 |  |
| Q.SLLRMPPWSNF.W | Y | 25.24 | 1346.6805 | -0.7 | 674.3471 | 2 | 15.17 | 2314 | 1 | 918 | 928 |  |
| E.VSVGDKVPA.D | Y | 16.99 | 870.4811 | -1.5 | 436.2472 | 2 | 10.13 | 1418 | 1 | 153 | 161 |  |
| L.GFNPPDLDIM.G | Y | 16.33 | 1117.5114 | 0.4 | 559.7632 | 2 | 14.27 | 2156 | 1 | 805 | 814 |  |
| total 4 peptides |  |  |  |  |  |  |  |  |  |  |  |  |
| --- | --- | --- | --- | --- | --- | --- | --- | --- | --- | --- | --- | --- |

tr|G3NWV4|G3NWV4\_GASAC

back to list

  

| Protein Coverage
| Supporting Peptides
|

Protein Coverage:

Supporting Peptides:

| Peptide | Uniq | -10lgP | Mass | ppm | m/z | z | RT | Scan | #Spec | Start | End | PTM |
| --- | --- | --- | --- | --- | --- | --- | --- | --- | --- | --- | --- | --- |
| L.SENQSLIRMPPWSNF.W | Y | 34.74 | 1804.8567 | 3.7 | 903.4390 | 2 | 15.03 | 2289 | 1 | 906 | 920 |  |
| Q.SLIRMPPWSNF.W | Y | 25.24 | 1346.6805 | -0.7 | 674.3471 | 2 | 15.17 | 2314 | 1 | 910 | 920 |  |
| E.VSVGDKVPA.D | Y | 16.99 | 870.4811 | -1.5 | 436.2472 | 2 | 10.13 | 1418 | 1 | 153 | 161 |  |
| L.GFNPPDLDIM.G | Y | 16.33 | 1117.5114 | 0.4 | 559.7632 | 2 | 14.27 | 2156 | 1 | 804 | 813 |  |
| total 4 peptides |  |  |  |  |  |  |  |  |  |  |  |  |
| --- | --- | --- | --- | --- | --- | --- | --- | --- | --- | --- | --- | --- |

tr|A0A6J2RNI9|A0A6J2RNI9\_COTGO

back to list

  

| Protein Coverage
| Supporting Peptides
|

Protein Coverage:

Supporting Peptides:

| Peptide | Uniq | -10lgP | Mass | ppm | m/z | z | RT | Scan | #Spec | Start | End | PTM |
| --- | --- | --- | --- | --- | --- | --- | --- | --- | --- | --- | --- | --- |
| L.SENQSLIRMPPWSNF.W | Y | 34.74 | 1804.8567 | 3.7 | 903.4390 | 2 | 15.03 | 2289 | 1 | 914 | 928 |  |
| Q.SLIRMPPWSNF.W | Y | 25.24 | 1346.6805 | -0.7 | 674.3471 | 2 | 15.17 | 2314 | 1 | 918 | 928 |  |
| E.VSVGDKVPA.D | Y | 16.99 | 870.4811 | -1.5 | 436.2472 | 2 | 10.13 | 1418 | 1 | 153 | 161 |  |
| L.GFNPPDLDIM.G | Y | 16.33 | 1117.5114 | 0.4 | 559.7632 | 2 | 14.27 | 2156 | 1 | 805 | 814 |  |
| total 4 peptides |  |  |  |  |  |  |  |  |  |  |  |  |
| --- | --- | --- | --- | --- | --- | --- | --- | --- | --- | --- | --- | --- |

tr|A0A6J2RKY9|A0A6J2RKY9\_COTGO

back to list

  

| Protein Coverage
| Supporting Peptides
|

Protein Coverage:

Supporting Peptides:

| Peptide | Uniq | -10lgP | Mass | ppm | m/z | z | RT | Scan | #Spec | Start | End | PTM |
| --- | --- | --- | --- | --- | --- | --- | --- | --- | --- | --- | --- | --- |
| L.SENQSLIRMPPWSNF.W | Y | 34.74 | 1804.8567 | 3.7 | 903.4390 | 2 | 15.03 | 2289 | 1 | 914 | 928 |  |
| Q.SLIRMPPWSNF.W | Y | 25.24 | 1346.6805 | -0.7 | 674.3471 | 2 | 15.17 | 2314 | 1 | 918 | 928 |  |
| E.VSVGDKVPA.D | Y | 16.99 | 870.4811 | -1.5 | 436.2472 | 2 | 10.13 | 1418 | 1 | 153 | 161 |  |
| L.GFNPPDLDIM.G | Y | 16.33 | 1117.5114 | 0.4 | 559.7632 | 2 | 14.27 | 2156 | 1 | 805 | 814 |  |
| total 4 peptides |  |  |  |  |  |  |  |  |  |  |  |  |
| --- | --- | --- | --- | --- | --- | --- | --- | --- | --- | --- | --- | --- |

tr|A0A3B1JPG2|A0A3B1JPG2\_ASTMX

back to list

  

| Protein Coverage
| Supporting Peptides
|

Protein Coverage:

Supporting Peptides:

| Peptide | Uniq | -10lgP | Mass | ppm | m/z | z | RT | Scan | #Spec | Start | End | PTM |
| --- | --- | --- | --- | --- | --- | --- | --- | --- | --- | --- | --- | --- |
| L.SENQSLIRMPPWSNF.W | Y | 34.74 | 1804.8567 | 3.7 | 903.4390 | 2 | 15.03 | 2289 | 1 | 914 | 928 |  |
| Q.SLIRMPPWSNF.W | Y | 25.24 | 1346.6805 | -0.7 | 674.3471 | 2 | 15.17 | 2314 | 1 | 918 | 928 |  |
| E.VSVGDKVPA.D | Y | 16.99 | 870.4811 | -1.5 | 436.2472 | 2 | 10.13 | 1418 | 1 | 153 | 161 |  |
| L.GFNPPDLDIM.G | Y | 16.33 | 1117.5114 | 0.4 | 559.7632 | 2 | 14.27 | 2156 | 1 | 805 | 814 |  |
| total 4 peptides |  |  |  |  |  |  |  |  |  |  |  |  |
| --- | --- | --- | --- | --- | --- | --- | --- | --- | --- | --- | --- | --- |

tr|A0A667WYB6|A0A667WYB6\_9TELE

back to list

  

| Protein Coverage
| Supporting Peptides
|

Protein Coverage:

Supporting Peptides:

| Peptide | Uniq | -10lgP | Mass | ppm | m/z | z | RT | Scan | #Spec | Start | End | PTM |
| --- | --- | --- | --- | --- | --- | --- | --- | --- | --- | --- | --- | --- |
| L.SENQSLLRMPPWSNF.W | Y | 34.74 | 1804.8567 | 3.7 | 903.4390 | 2 | 15.03 | 2289 | 1 | 906 | 920 |  |
| Q.SLLRMPPWSNF.W | Y | 25.24 | 1346.6805 | -0.7 | 674.3471 | 2 | 15.17 | 2314 | 1 | 910 | 920 |  |
| E.VSVGDKVPA.D | Y | 16.99 | 870.4811 | -1.5 | 436.2472 | 2 | 10.13 | 1418 | 1 | 151 | 159 |  |
| L.GFNPPDLDIM.G | Y | 16.33 | 1117.5114 | 0.4 | 559.7632 | 2 | 14.27 | 2156 | 1 | 797 | 806 |  |
| total 4 peptides |  |  |  |  |  |  |  |  |  |  |  |  |
| --- | --- | --- | --- | --- | --- | --- | --- | --- | --- | --- | --- | --- |

tr|A0A3B4VM00|A0A3B4VM00\_SERDU

back to list

  

| Protein Coverage
| Supporting Peptides
|

Protein Coverage:

Supporting Peptides:

| Peptide | Uniq | -10lgP | Mass | ppm | m/z | z | RT | Scan | #Spec | Start | End | PTM |
| --- | --- | --- | --- | --- | --- | --- | --- | --- | --- | --- | --- | --- |
| L.SENQSLIRMPPWSNF.W | Y | 34.74 | 1804.8567 | 3.7 | 903.4390 | 2 | 15.03 | 2289 | 1 | 912 | 926 |  |
| Q.SLIRMPPWSNF.W | Y | 25.24 | 1346.6805 | -0.7 | 674.3471 | 2 | 15.17 | 2314 | 1 | 916 | 926 |  |
| E.VSVGDKVPA.D | Y | 16.99 | 870.4811 | -1.5 | 436.2472 | 2 | 10.13 | 1418 | 1 | 151 | 159 |  |
| L.GFNPPDLDIM.G | Y | 16.33 | 1117.5114 | 0.4 | 559.7632 | 2 | 14.27 | 2156 | 1 | 803 | 812 |  |
| total 4 peptides |  |  |  |  |  |  |  |  |  |  |  |  |
| --- | --- | --- | --- | --- | --- | --- | --- | --- | --- | --- | --- | --- |

tr|A0A667X953|A0A667X953\_9TELE

back to list

  

| Protein Coverage
| Supporting Peptides
|

Protein Coverage:

Supporting Peptides:

| Peptide | Uniq | -10lgP | Mass | ppm | m/z | z | RT | Scan | #Spec | Start | End | PTM |
| --- | --- | --- | --- | --- | --- | --- | --- | --- | --- | --- | --- | --- |
| L.SENQSLLRMPPWSNF.W | Y | 34.74 | 1804.8567 | 3.7 | 903.4390 | 2 | 15.03 | 2289 | 1 | 914 | 928 |  |
| Q.SLLRMPPWSNF.W | Y | 25.24 | 1346.6805 | -0.7 | 674.3471 | 2 | 15.17 | 2314 | 1 | 918 | 928 |  |
| E.VSVGDKVPA.D | Y | 16.99 | 870.4811 | -1.5 | 436.2472 | 2 | 10.13 | 1418 | 1 | 153 | 161 |  |
| L.GFNPPDLDIM.G | Y | 16.33 | 1117.5114 | 0.4 | 559.7632 | 2 | 14.27 | 2156 | 1 | 805 | 814 |  |
| total 4 peptides |  |  |  |  |  |  |  |  |  |  |  |  |
| --- | --- | --- | --- | --- | --- | --- | --- | --- | --- | --- | --- | --- |

tr|A0A7N8WP58|A0A7N8WP58\_9TELE

back to list

  

| Protein Coverage
| Supporting Peptides
|

Protein Coverage:

Supporting Peptides:

| Peptide | Uniq | -10lgP | Mass | ppm | m/z | z | RT | Scan | #Spec | Start | End | PTM |
| --- | --- | --- | --- | --- | --- | --- | --- | --- | --- | --- | --- | --- |
| L.SENQSLLRMPPWSNF.W | Y | 34.74 | 1804.8567 | 3.7 | 903.4390 | 2 | 15.03 | 2289 | 1 | 880 | 894 |  |
| Q.SLLRMPPWSNF.W | Y | 25.24 | 1346.6805 | -0.7 | 674.3471 | 2 | 15.17 | 2314 | 1 | 884 | 894 |  |
| E.VSVGDKVPA.D | Y | 16.99 | 870.4811 | -1.5 | 436.2472 | 2 | 10.13 | 1418 | 1 | 119 | 127 |  |
| L.GFNPPDLDIM.G | Y | 16.33 | 1117.5114 | 0.4 | 559.7632 | 2 | 14.27 | 2156 | 1 | 771 | 780 |  |
| total 4 peptides |  |  |  |  |  |  |  |  |  |  |  |  |
| --- | --- | --- | --- | --- | --- | --- | --- | --- | --- | --- | --- | --- |

tr|A0A7N8Y7I9|A0A7N8Y7I9\_9TELE

back to list

  

| Protein Coverage
| Supporting Peptides
|

Protein Coverage:

Supporting Peptides:

| Peptide | Uniq | -10lgP | Mass | ppm | m/z | z | RT | Scan | #Spec | Start | End | PTM |
| --- | --- | --- | --- | --- | --- | --- | --- | --- | --- | --- | --- | --- |
| L.SENQSLLRMPPWSNF.W | Y | 34.74 | 1804.8567 | 3.7 | 903.4390 | 2 | 15.03 | 2289 | 1 | 884 | 898 |  |
| Q.SLLRMPPWSNF.W | Y | 25.24 | 1346.6805 | -0.7 | 674.3471 | 2 | 15.17 | 2314 | 1 | 888 | 898 |  |
| E.VSVGDKVPA.D | Y | 16.99 | 870.4811 | -1.5 | 436.2472 | 2 | 10.13 | 1418 | 1 | 153 | 161 |  |
| L.GFNPPDLDIM.G | Y | 16.33 | 1117.5114 | 0.4 | 559.7632 | 2 | 14.27 | 2156 | 1 | 775 | 784 |  |
| total 4 peptides |  |  |  |  |  |  |  |  |  |  |  |  |
| --- | --- | --- | --- | --- | --- | --- | --- | --- | --- | --- | --- | --- |

tr|A0A671YJD0|A0A671YJD0\_SPAAU

back to list

  

| Protein Coverage
| Supporting Peptides
|

Protein Coverage:

Supporting Peptides:

| Peptide | Uniq | -10lgP | Mass | ppm | m/z | z | RT | Scan | #Spec | Start | End | PTM |
| --- | --- | --- | --- | --- | --- | --- | --- | --- | --- | --- | --- | --- |
| L.SENQSLIRMPPWSNF.W | Y | 34.74 | 1804.8567 | 3.7 | 903.4390 | 2 | 15.03 | 2289 | 1 | 914 | 928 |  |
| Q.SLIRMPPWSNF.W | Y | 25.24 | 1346.6805 | -0.7 | 674.3471 | 2 | 15.17 | 2314 | 1 | 918 | 928 |  |
| E.VSVGDKVPA.D | Y | 16.99 | 870.4811 | -1.5 | 436.2472 | 2 | 10.13 | 1418 | 1 | 153 | 161 |  |
| L.GFNPPDLDIM.G | Y | 16.33 | 1117.5114 | 0.4 | 559.7632 | 2 | 14.27 | 2156 | 1 | 805 | 814 |  |
| total 4 peptides |  |  |  |  |  |  |  |  |  |  |  |  |
| --- | --- | --- | --- | --- | --- | --- | --- | --- | --- | --- | --- | --- |

tr|A0A7N8XW64|A0A7N8XW64\_9TELE

back to list

  

| Protein Coverage
| Supporting Peptides
|

Protein Coverage:

Supporting Peptides:

| Peptide | Uniq | -10lgP | Mass | ppm | m/z | z | RT | Scan | #Spec | Start | End | PTM |
| --- | --- | --- | --- | --- | --- | --- | --- | --- | --- | --- | --- | --- |
| L.SENQSLLRMPPWSNF.W | Y | 34.74 | 1804.8567 | 3.7 | 903.4390 | 2 | 15.03 | 2289 | 1 | 914 | 928 |  |
| Q.SLLRMPPWSNF.W | Y | 25.24 | 1346.6805 | -0.7 | 674.3471 | 2 | 15.17 | 2314 | 1 | 918 | 928 |  |
| E.VSVGDKVPA.D | Y | 16.99 | 870.4811 | -1.5 | 436.2472 | 2 | 10.13 | 1418 | 1 | 153 | 161 |  |
| L.GFNPPDLDIM.G | Y | 16.33 | 1117.5114 | 0.4 | 559.7632 | 2 | 14.27 | 2156 | 1 | 805 | 814 |  |
| total 4 peptides |  |  |  |  |  |  |  |  |  |  |  |  |
| --- | --- | --- | --- | --- | --- | --- | --- | --- | --- | --- | --- | --- |

tr|A0A7N9AUZ7|A0A7N9AUZ7\_9TELE

back to list

  

| Protein Coverage
| Supporting Peptides
|

Protein Coverage:

Supporting Peptides:

| Peptide | Uniq | -10lgP | Mass | ppm | m/z | z | RT | Scan | #Spec | Start | End | PTM |
| --- | --- | --- | --- | --- | --- | --- | --- | --- | --- | --- | --- | --- |
| L.SENQSLLRMPPWSNF.W | Y | 34.74 | 1804.8567 | 3.7 | 903.4390 | 2 | 15.03 | 2289 | 1 | 901 | 915 |  |
| Q.SLLRMPPWSNF.W | Y | 25.24 | 1346.6805 | -0.7 | 674.3471 | 2 | 15.17 | 2314 | 1 | 905 | 915 |  |
| E.VSVGDKVPA.D | Y | 16.99 | 870.4811 | -1.5 | 436.2472 | 2 | 10.13 | 1418 | 1 | 147 | 155 |  |
| L.GFNPPDLDIM.G | Y | 16.33 | 1117.5114 | 0.4 | 559.7632 | 2 | 14.27 | 2156 | 1 | 792 | 801 |  |
| total 4 peptides |  |  |  |  |  |  |  |  |  |  |  |  |
| --- | --- | --- | --- | --- | --- | --- | --- | --- | --- | --- | --- | --- |

tr|A0A3Q3MF18|A0A3Q3MF18\_9TELE

back to list

  

| Protein Coverage
| Supporting Peptides
|

Protein Coverage:

Supporting Peptides:

| Peptide | Uniq | -10lgP | Mass | ppm | m/z | z | RT | Scan | #Spec | Start | End | PTM |
| --- | --- | --- | --- | --- | --- | --- | --- | --- | --- | --- | --- | --- |
| L.SENQSLLRMPPWSNF.W | Y | 34.74 | 1804.8567 | 3.7 | 903.4390 | 2 | 15.03 | 2289 | 1 | 919 | 933 |  |
| Q.SLLRMPPWSNF.W | Y | 25.24 | 1346.6805 | -0.7 | 674.3471 | 2 | 15.17 | 2314 | 1 | 923 | 933 |  |
| E.VSVGDKVPA.D | Y | 16.99 | 870.4811 | -1.5 | 436.2472 | 2 | 10.13 | 1418 | 1 | 153 | 161 |  |
| L.GFNPPDLDIM.G | Y | 16.33 | 1117.5114 | 0.4 | 559.7632 | 2 | 14.27 | 2156 | 1 | 810 | 819 |  |
| total 4 peptides |  |  |  |  |  |  |  |  |  |  |  |  |
| --- | --- | --- | --- | --- | --- | --- | --- | --- | --- | --- | --- | --- |

tr|A0A672IVR0|A0A672IVR0\_SALFA

back to list

  

| Protein Coverage
| Supporting Peptides
|

Protein Coverage:

Supporting Peptides:

| Peptide | Uniq | -10lgP | Mass | ppm | m/z | z | RT | Scan | #Spec | Start | End | PTM |
| --- | --- | --- | --- | --- | --- | --- | --- | --- | --- | --- | --- | --- |
| L.SENQSLLRMPPWSNF.W | Y | 34.74 | 1804.8567 | 3.7 | 903.4390 | 2 | 15.03 | 2289 | 1 | 879 | 893 |  |
| Q.SLLRMPPWSNF.W | Y | 25.24 | 1346.6805 | -0.7 | 674.3471 | 2 | 15.17 | 2314 | 1 | 883 | 893 |  |
| E.VSVGDKVPA.D | Y | 16.99 | 870.4811 | -1.5 | 436.2472 | 2 | 10.13 | 1418 | 1 | 118 | 126 |  |
| L.GFNPPDLDIM.G | Y | 16.33 | 1117.5114 | 0.4 | 559.7632 | 2 | 14.27 | 2156 | 1 | 770 | 779 |  |
| total 4 peptides |  |  |  |  |  |  |  |  |  |  |  |  |
| --- | --- | --- | --- | --- | --- | --- | --- | --- | --- | --- | --- | --- |

tr|A0A3Q1BZF5|A0A3Q1BZF5\_AMPOC

back to list

  

| Protein Coverage
| Supporting Peptides
|

Protein Coverage:

Supporting Peptides:

| Peptide | Uniq | -10lgP | Mass | ppm | m/z | z | RT | Scan | #Spec | Start | End | PTM |
| --- | --- | --- | --- | --- | --- | --- | --- | --- | --- | --- | --- | --- |
| L.SENQSLLRMPPWSNF.W | Y | 34.74 | 1804.8567 | 3.7 | 903.4390 | 2 | 15.03 | 2289 | 1 | 912 | 926 |  |
| Q.SLLRMPPWSNF.W | Y | 25.24 | 1346.6805 | -0.7 | 674.3471 | 2 | 15.17 | 2314 | 1 | 916 | 926 |  |
| E.VSVGDKVPA.D | Y | 16.99 | 870.4811 | -1.5 | 436.2472 | 2 | 10.13 | 1418 | 1 | 151 | 159 |  |
| L.GFNPPDLDIM.G | Y | 16.33 | 1117.5114 | 0.4 | 559.7632 | 2 | 14.27 | 2156 | 1 | 803 | 812 |  |
| total 4 peptides |  |  |  |  |  |  |  |  |  |  |  |  |
| --- | --- | --- | --- | --- | --- | --- | --- | --- | --- | --- | --- | --- |

tr|A0A3Q0SE88|A0A3Q0SE88\_AMPCI

back to list

  

| Protein Coverage
| Supporting Peptides
|

Protein Coverage:

Supporting Peptides:

| Peptide | Uniq | -10lgP | Mass | ppm | m/z | z | RT | Scan | #Spec | Start | End | PTM |
| --- | --- | --- | --- | --- | --- | --- | --- | --- | --- | --- | --- | --- |
| L.SENQSLLRMPPWSNF.W | Y | 34.74 | 1804.8567 | 3.7 | 903.4390 | 2 | 15.03 | 2289 | 1 | 912 | 926 |  |
| Q.SLLRMPPWSNF.W | Y | 25.24 | 1346.6805 | -0.7 | 674.3471 | 2 | 15.17 | 2314 | 1 | 916 | 926 |  |
| E.VSVGDKVPA.D | Y | 16.99 | 870.4811 | -1.5 | 436.2472 | 2 | 10.13 | 1418 | 1 | 151 | 159 |  |
| L.GFNPPDLDIM.G | Y | 16.33 | 1117.5114 | 0.4 | 559.7632 | 2 | 14.27 | 2156 | 1 | 803 | 812 |  |
| total 4 peptides |  |  |  |  |  |  |  |  |  |  |  |  |
| --- | --- | --- | --- | --- | --- | --- | --- | --- | --- | --- | --- | --- |

tr|A0A3P8SU98|A0A3P8SU98\_AMPPE

back to list

  

| Protein Coverage
| Supporting Peptides
|

Protein Coverage:

Supporting Peptides:

| Peptide | Uniq | -10lgP | Mass | ppm | m/z | z | RT | Scan | #Spec | Start | End | PTM |
| --- | --- | --- | --- | --- | --- | --- | --- | --- | --- | --- | --- | --- |
| L.SENQSLLRMPPWSNF.W | Y | 34.74 | 1804.8567 | 3.7 | 903.4390 | 2 | 15.03 | 2289 | 1 | 914 | 928 |  |
| Q.SLLRMPPWSNF.W | Y | 25.24 | 1346.6805 | -0.7 | 674.3471 | 2 | 15.17 | 2314 | 1 | 918 | 928 |  |
| E.VSVGDKVPA.D | Y | 16.99 | 870.4811 | -1.5 | 436.2472 | 2 | 10.13 | 1418 | 1 | 153 | 161 |  |
| L.GFNPPDLDIM.G | Y | 16.33 | 1117.5114 | 0.4 | 559.7632 | 2 | 14.27 | 2156 | 1 | 805 | 814 |  |
| total 4 peptides |  |  |  |  |  |  |  |  |  |  |  |  |
| --- | --- | --- | --- | --- | --- | --- | --- | --- | --- | --- | --- | --- |

tr|A0A672IVP1|A0A672IVP1\_SALFA

back to list

  

| Protein Coverage
| Supporting Peptides
|

Protein Coverage:

Supporting Peptides:

| Peptide | Uniq | -10lgP | Mass | ppm | m/z | z | RT | Scan | #Spec | Start | End | PTM |
| --- | --- | --- | --- | --- | --- | --- | --- | --- | --- | --- | --- | --- |
| L.SENQSLLRMPPWSNF.W | Y | 34.74 | 1804.8567 | 3.7 | 903.4390 | 2 | 15.03 | 2289 | 1 | 914 | 928 |  |
| Q.SLLRMPPWSNF.W | Y | 25.24 | 1346.6805 | -0.7 | 674.3471 | 2 | 15.17 | 2314 | 1 | 918 | 928 |  |
| E.VSVGDKVPA.D | Y | 16.99 | 870.4811 | -1.5 | 436.2472 | 2 | 10.13 | 1418 | 1 | 153 | 161 |  |
| L.GFNPPDLDIM.G | Y | 16.33 | 1117.5114 | 0.4 | 559.7632 | 2 | 14.27 | 2156 | 1 | 805 | 814 |  |
| total 4 peptides |  |  |  |  |  |  |  |  |  |  |  |  |
| --- | --- | --- | --- | --- | --- | --- | --- | --- | --- | --- | --- | --- |

tr|A0A3Q1BVL9|A0A3Q1BVL9\_AMPOC

back to list

  

| Protein Coverage
| Supporting Peptides
|

Protein Coverage:

Supporting Peptides:

| Peptide | Uniq | -10lgP | Mass | ppm | m/z | z | RT | Scan | #Spec | Start | End | PTM |
| --- | --- | --- | --- | --- | --- | --- | --- | --- | --- | --- | --- | --- |
| L.SENQSLLRMPPWSNF.W | Y | 34.74 | 1804.8567 | 3.7 | 903.4390 | 2 | 15.03 | 2289 | 1 | 914 | 928 |  |
| Q.SLLRMPPWSNF.W | Y | 25.24 | 1346.6805 | -0.7 | 674.3471 | 2 | 15.17 | 2314 | 1 | 918 | 928 |  |
| E.VSVGDKVPA.D | Y | 16.99 | 870.4811 | -1.5 | 436.2472 | 2 | 10.13 | 1418 | 1 | 153 | 161 |  |
| L.GFNPPDLDIM.G | Y | 16.33 | 1117.5114 | 0.4 | 559.7632 | 2 | 14.27 | 2156 | 1 | 805 | 814 |  |
| total 4 peptides |  |  |  |  |  |  |  |  |  |  |  |  |
| --- | --- | --- | --- | --- | --- | --- | --- | --- | --- | --- | --- | --- |

tr|A0A672ITB5|A0A672ITB5\_SALFA

back to list

  

| Protein Coverage
| Supporting Peptides
|

Protein Coverage:

Supporting Peptides:

| Peptide | Uniq | -10lgP | Mass | ppm | m/z | z | RT | Scan | #Spec | Start | End | PTM |
| --- | --- | --- | --- | --- | --- | --- | --- | --- | --- | --- | --- | --- |
| L.SENQSLLRMPPWSNF.W | Y | 34.74 | 1804.8567 | 3.7 | 903.4390 | 2 | 15.03 | 2289 | 1 | 914 | 928 |  |
| Q.SLLRMPPWSNF.W | Y | 25.24 | 1346.6805 | -0.7 | 674.3471 | 2 | 15.17 | 2314 | 1 | 918 | 928 |  |
| E.VSVGDKVPA.D | Y | 16.99 | 870.4811 | -1.5 | 436.2472 | 2 | 10.13 | 1418 | 1 | 153 | 161 |  |
| L.GFNPPDLDIM.G | Y | 16.33 | 1117.5114 | 0.4 | 559.7632 | 2 | 14.27 | 2156 | 1 | 805 | 814 |  |
| total 4 peptides |  |  |  |  |  |  |  |  |  |  |  |  |
| --- | --- | --- | --- | --- | --- | --- | --- | --- | --- | --- | --- | --- |

tr|A0A672IVQ1|A0A672IVQ1\_SALFA

back to list

  

| Protein Coverage
| Supporting Peptides
|

Protein Coverage:

Supporting Peptides:

| Peptide | Uniq | -10lgP | Mass | ppm | m/z | z | RT | Scan | #Spec | Start | End | PTM |
| --- | --- | --- | --- | --- | --- | --- | --- | --- | --- | --- | --- | --- |
| L.SENQSLLRMPPWSNF.W | Y | 34.74 | 1804.8567 | 3.7 | 903.4390 | 2 | 15.03 | 2289 | 1 | 914 | 928 |  |
| Q.SLLRMPPWSNF.W | Y | 25.24 | 1346.6805 | -0.7 | 674.3471 | 2 | 15.17 | 2314 | 1 | 918 | 928 |  |
| E.VSVGDKVPA.D | Y | 16.99 | 870.4811 | -1.5 | 436.2472 | 2 | 10.13 | 1418 | 1 | 153 | 161 |  |
| L.GFNPPDLDIM.G | Y | 16.33 | 1117.5114 | 0.4 | 559.7632 | 2 | 14.27 | 2156 | 1 | 805 | 814 |  |
| total 4 peptides |  |  |  |  |  |  |  |  |  |  |  |  |
| --- | --- | --- | --- | --- | --- | --- | --- | --- | --- | --- | --- | --- |

tr|A0A834CTG4|A0A834CTG4\_ORYME

back to list

  

| Protein Coverage
| Supporting Peptides
|

Protein Coverage:

Supporting Peptides:

| Peptide | Uniq | -10lgP | Mass | ppm | m/z | z | RT | Scan | #Spec | Start | End | PTM |
| --- | --- | --- | --- | --- | --- | --- | --- | --- | --- | --- | --- | --- |
| L.AVDPVYPPGPPAFPK.V | N | 36.21 | 1550.8132 | -1.1 | 776.4130 | 2 | 12.35 | 1817 | 1 | 3990 | 4004 |  |
| T.VVDRPQPPEGPVVF.N | Y | 21.55 | 1534.8143 | -0.9 | 768.4138 | 2 | 12.52 | 1847 | 1 | 1315 | 1328 |  |
| A.MNPLFPPGPPKDL.H | Y | 16.76 | 1421.7377 | -1.0 | 711.8754 | 2 | 13.15 | 1962 | 1 | 1811 | 1823 |  |
| total 3 peptides |  |  |  |  |  |  |  |  |  |  |  |  |
| --- | --- | --- | --- | --- | --- | --- | --- | --- | --- | --- | --- | --- |

tr|Q4RE90|Q4RE90\_TETNG

back to list

  

| Protein Coverage
| Supporting Peptides
|

Protein Coverage:

Supporting Peptides:

| Peptide | Uniq | -10lgP | Mass | ppm | m/z | z | RT | Scan | #Spec | Start | End | PTM |
| --- | --- | --- | --- | --- | --- | --- | --- | --- | --- | --- | --- | --- |
| L.AVDPVYPPGPPAFPK.V | N | 36.21 | 1550.8132 | -1.1 | 776.4130 | 2 | 12.35 | 1817 | 1 | 7117 | 7131 |  |
| T.VVDRPQPPEGPVVF.N | Y | 21.55 | 1534.8143 | -0.9 | 768.4138 | 2 | 12.52 | 1847 | 1 | 4491 | 4504 |  |
| A.MNPLFPPGPPKDL.H | Y | 16.76 | 1421.7377 | -1.0 | 711.8754 | 2 | 13.15 | 1962 | 1 | 4987 | 4999 |  |
| total 3 peptides |  |  |  |  |  |  |  |  |  |  |  |  |
| --- | --- | --- | --- | --- | --- | --- | --- | --- | --- | --- | --- | --- |

tr|A0A3P8Q9R4|A0A3P8Q9R4\_ASTCA

back to list

  

| Protein Coverage
| Supporting Peptides
|

Protein Coverage:

Supporting Peptides:

| Peptide | Uniq | -10lgP | Mass | ppm | m/z | z | RT | Scan | #Spec | Start | End | PTM |
| --- | --- | --- | --- | --- | --- | --- | --- | --- | --- | --- | --- | --- |
| F.KDLLDPIISDR.H | Y | 24.21 | 1283.7085 | -1.3 | 642.8607 | 2 | 12.39 | 1822 | 1 | 85 | 95 |  |
| L.FDKPVSPL.L | Y | 23.03 | 901.4908 | -0.8 | 451.7523 | 2 | 23.12 | 3597 | 6 | 193 | 200 |  |
| T.GVDNPGHPF.I | Y | 22.06 | 938.4246 | -0.3 | 470.2195 | 2 | 10.75 | 1523 | 2 | 59 | 67 |  |
| R.HGGYKPT.D | Y | 17.16 | 758.3711 | 0.2 | 380.1929 | 2 | 8.46 | 1123 | 1 | 96 | 102 |  |
| W.VNEEDHLRVI.S | Y | 16.15 | 1222.6306 | -0.9 | 612.3220 | 2 | 11.23 | 1610 | 1 | 228 | 237 |  |
| total 5 peptides |  |  |  |  |  |  |  |  |  |  |  |  |
| --- | --- | --- | --- | --- | --- | --- | --- | --- | --- | --- | --- | --- |

tr|A0A3Q4HL72|A0A3Q4HL72\_NEOBR

back to list

  

| Protein Coverage
| Supporting Peptides
|

Protein Coverage:

Supporting Peptides:

| Peptide | Uniq | -10lgP | Mass | ppm | m/z | z | RT | Scan | #Spec | Start | End | PTM |
| --- | --- | --- | --- | --- | --- | --- | --- | --- | --- | --- | --- | --- |
| F.KDLLDPIISDR.H | Y | 24.21 | 1283.7085 | -1.3 | 642.8607 | 2 | 12.39 | 1822 | 1 | 85 | 95 |  |
| L.FDKPVSPL.L | Y | 23.03 | 901.4908 | -0.8 | 451.7523 | 2 | 23.12 | 3597 | 6 | 193 | 200 |  |
| T.GVDNPGHPF.I | Y | 22.06 | 938.4246 | -0.3 | 470.2195 | 2 | 10.75 | 1523 | 2 | 59 | 67 |  |
| R.HGGYKPT.D | Y | 17.16 | 758.3711 | 0.2 | 380.1929 | 2 | 8.46 | 1123 | 1 | 96 | 102 |  |
| W.VNEEDHLRVI.S | Y | 16.15 | 1222.6306 | -0.9 | 612.3220 | 2 | 11.23 | 1610 | 1 | 228 | 237 |  |
| total 5 peptides |  |  |  |  |  |  |  |  |  |  |  |  |
| --- | --- | --- | --- | --- | --- | --- | --- | --- | --- | --- | --- | --- |

tr|A0A3B4TA77|A0A3B4TA77\_SERDU

back to list

  

| Protein Coverage
| Supporting Peptides
|

Protein Coverage:

Supporting Peptides:

| Peptide | Uniq | -10lgP | Mass | ppm | m/z | z | RT | Scan | #Spec | Start | End | PTM |
| --- | --- | --- | --- | --- | --- | --- | --- | --- | --- | --- | --- | --- |
| F.KDLLDPIISDR.H | Y | 24.21 | 1283.7085 | -1.3 | 642.8607 | 2 | 12.39 | 1822 | 1 | 85 | 95 |  |
| L.FDKPVSPL.L | Y | 23.03 | 901.4908 | -0.8 | 451.7523 | 2 | 23.12 | 3597 | 6 | 193 | 200 |  |
| T.GVDNPGHPF.I | Y | 22.06 | 938.4246 | -0.3 | 470.2195 | 2 | 10.75 | 1523 | 2 | 59 | 67 |  |
| R.HGGYKPT.D | Y | 17.16 | 758.3711 | 0.2 | 380.1929 | 2 | 8.46 | 1123 | 1 | 96 | 102 |  |
| W.VNEEDHLRVI.S | Y | 16.15 | 1222.6306 | -0.9 | 612.3220 | 2 | 11.23 | 1610 | 1 | 228 | 237 |  |
| total 5 peptides |  |  |  |  |  |  |  |  |  |  |  |  |
| --- | --- | --- | --- | --- | --- | --- | --- | --- | --- | --- | --- | --- |

tr|A0A0F8AN20|A0A0F8AN20\_LARCR

back to list

  

| Protein Coverage
| Supporting Peptides
|

Protein Coverage:

Supporting Peptides:

| Peptide | Uniq | -10lgP | Mass | ppm | m/z | z | RT | Scan | #Spec | Start | End | PTM |
| --- | --- | --- | --- | --- | --- | --- | --- | --- | --- | --- | --- | --- |
| F.KDLLDPIISDR.H | Y | 24.21 | 1283.7085 | -1.3 | 642.8607 | 2 | 12.39 | 1822 | 1 | 85 | 95 |  |
| L.FDKPVSPL.L | Y | 23.03 | 901.4908 | -0.8 | 451.7523 | 2 | 23.12 | 3597 | 6 | 193 | 200 |  |
| T.GVDNPGHPF.I | Y | 22.06 | 938.4246 | -0.3 | 470.2195 | 2 | 10.75 | 1523 | 2 | 59 | 67 |  |
| R.HGGYKPT.D | Y | 17.16 | 758.3711 | 0.2 | 380.1929 | 2 | 8.46 | 1123 | 1 | 96 | 102 |  |
| W.VNEEDHLRVI.S | Y | 16.15 | 1222.6306 | -0.9 | 612.3220 | 2 | 11.23 | 1610 | 1 | 228 | 237 |  |
| total 5 peptides |  |  |  |  |  |  |  |  |  |  |  |  |
| --- | --- | --- | --- | --- | --- | --- | --- | --- | --- | --- | --- | --- |

tr|C7ASM1|C7ASM1\_SINCH

back to list

  

| Protein Coverage
| Supporting Peptides
|

Protein Coverage:

Supporting Peptides:

| Peptide | Uniq | -10lgP | Mass | ppm | m/z | z | RT | Scan | #Spec | Start | End | PTM |
| --- | --- | --- | --- | --- | --- | --- | --- | --- | --- | --- | --- | --- |
| F.KDLLDPIISDR.H | Y | 24.21 | 1283.7085 | -1.3 | 642.8607 | 2 | 12.39 | 1822 | 1 | 86 | 96 |  |
| L.FDKPVSPL.L | Y | 23.03 | 901.4908 | -0.8 | 451.7523 | 2 | 23.12 | 3597 | 6 | 194 | 201 |  |
| T.GVDNPGHPF.I | Y | 22.06 | 938.4246 | -0.3 | 470.2195 | 2 | 10.75 | 1523 | 2 | 60 | 68 |  |
| R.HGGYKPT.D | Y | 17.16 | 758.3711 | 0.2 | 380.1929 | 2 | 8.46 | 1123 | 1 | 97 | 103 |  |
| W.VNEEDHLRVI.S | Y | 16.15 | 1222.6306 | -0.9 | 612.3220 | 2 | 11.23 | 1610 | 1 | 229 | 238 |  |
| total 5 peptides |  |  |  |  |  |  |  |  |  |  |  |  |
| --- | --- | --- | --- | --- | --- | --- | --- | --- | --- | --- | --- | --- |

tr|G3PFX6|G3PFX6\_GASAC

back to list

  

| Protein Coverage
| Supporting Peptides
|

Protein Coverage:

Supporting Peptides:

| Peptide | Uniq | -10lgP | Mass | ppm | m/z | z | RT | Scan | #Spec | Start | End | PTM |
| --- | --- | --- | --- | --- | --- | --- | --- | --- | --- | --- | --- | --- |
| F.KDLLDPIISDR.H | Y | 24.21 | 1283.7085 | -1.3 | 642.8607 | 2 | 12.39 | 1822 | 1 | 119 | 129 |  |
| L.FDKPVSPL.L | Y | 23.03 | 901.4908 | -0.8 | 451.7523 | 2 | 23.12 | 3597 | 6 | 227 | 234 |  |
| T.GVDNPGHPF.I | Y | 22.06 | 938.4246 | -0.3 | 470.2195 | 2 | 10.75 | 1523 | 2 | 93 | 101 |  |
| R.HGGYKPT.D | Y | 17.16 | 758.3711 | 0.2 | 380.1929 | 2 | 8.46 | 1123 | 1 | 130 | 136 |  |
| W.VNEEDHLRVI.S | Y | 16.15 | 1222.6306 | -0.9 | 612.3220 | 2 | 11.23 | 1610 | 1 | 262 | 271 |  |
| total 5 peptides |  |  |  |  |  |  |  |  |  |  |  |  |
| --- | --- | --- | --- | --- | --- | --- | --- | --- | --- | --- | --- | --- |

tr|A0A6P7JDS0|A0A6P7JDS0\_9TELE

back to list

  

| Protein Coverage
| Supporting Peptides
|

Protein Coverage:

Supporting Peptides:

| Peptide | Uniq | -10lgP | Mass | ppm | m/z | z | RT | Scan | #Spec | Start | End | PTM |
| --- | --- | --- | --- | --- | --- | --- | --- | --- | --- | --- | --- | --- |
| F.KDLLDPIISDR.H | Y | 24.21 | 1283.7085 | -1.3 | 642.8607 | 2 | 12.39 | 1822 | 1 | 167 | 177 |  |
| L.FDKPVSPL.L | Y | 23.03 | 901.4908 | -0.8 | 451.7523 | 2 | 23.12 | 3597 | 6 | 275 | 282 |  |
| T.GVDNPGHPF.I | Y | 22.06 | 938.4246 | -0.3 | 470.2195 | 2 | 10.75 | 1523 | 2 | 141 | 149 |  |
| R.HGGYKPT.D | Y | 17.16 | 758.3711 | 0.2 | 380.1929 | 2 | 8.46 | 1123 | 1 | 178 | 184 |  |
| W.VNEEDHLRVI.S | Y | 16.15 | 1222.6306 | -0.9 | 612.3220 | 2 | 11.23 | 1610 | 1 | 310 | 319 |  |
| total 5 peptides |  |  |  |  |  |  |  |  |  |  |  |  |
| --- | --- | --- | --- | --- | --- | --- | --- | --- | --- | --- | --- | --- |

tr|A0A672MA11|A0A672MA11\_SINGR

back to list

  

| Protein Coverage
| Supporting Peptides
|

Protein Coverage:

Supporting Peptides:

| Peptide | Uniq | -10lgP | Mass | ppm | m/z | z | RT | Scan | #Spec | Start | End | PTM |
| --- | --- | --- | --- | --- | --- | --- | --- | --- | --- | --- | --- | --- |
| F.GEKLKGADPEDVILS.A | Y | 28.44 | 1569.8250 | -0.2 | 785.9196 | 2 | 11.67 | 1690 | 1 | 62 | 76 |  |
| F.GEKLKGADPEDVIL.S | Y | 24.67 | 1482.7930 | -0.8 | 742.4032 | 2 | 11.90 | 1732 | 1 | 62 | 75 |  |
| L.WAAFPPDVA.G | Y | 20.98 | 972.4705 | -0.5 | 487.2423 | 2 | 13.51 | 2026 | 4 | 114 | 122 |  |
| total 3 peptides |  |  |  |  |  |  |  |  |  |  |  |  |
| --- | --- | --- | --- | --- | --- | --- | --- | --- | --- | --- | --- | --- |

tr|A0A4U5VU95|A0A4U5VU95\_COLLU

back to list

  

| Protein Coverage
| Supporting Peptides
|

Protein Coverage:

Supporting Peptides:

| Peptide | Uniq | -10lgP | Mass | ppm | m/z | z | RT | Scan | #Spec | Start | End | PTM |
| --- | --- | --- | --- | --- | --- | --- | --- | --- | --- | --- | --- | --- |
| F.GEKLKGADPEDVILS.A | Y | 28.44 | 1569.8250 | -0.2 | 785.9196 | 2 | 11.67 | 1690 | 1 | 68 | 82 |  |
| F.GEKLKGADPEDVIL.S | Y | 24.67 | 1482.7930 | -0.8 | 742.4032 | 2 | 11.90 | 1732 | 1 | 68 | 81 |  |
| M.WAAFPPDVA.G | Y | 20.98 | 972.4705 | -0.5 | 487.2423 | 2 | 13.51 | 2026 | 4 | 120 | 128 |  |
| total 3 peptides |  |  |  |  |  |  |  |  |  |  |  |  |
| --- | --- | --- | --- | --- | --- | --- | --- | --- | --- | --- | --- | --- |

tr|A0A3B4BW75|A0A3B4BW75\_PYGNA

back to list

  

| Protein Coverage
| Supporting Peptides
|

Protein Coverage:

Supporting Peptides:

| Peptide | Uniq | -10lgP | Mass | ppm | m/z | z | RT | Scan | #Spec | Start | End | PTM |
| --- | --- | --- | --- | --- | --- | --- | --- | --- | --- | --- | --- | --- |
| F.GEKLKGADPEDVILS.G | Y | 28.44 | 1569.8250 | -0.2 | 785.9196 | 2 | 11.67 | 1690 | 1 | 82 | 96 |  |
| F.GEKLKGADPEDVIL.S | Y | 24.67 | 1482.7930 | -0.8 | 742.4032 | 2 | 11.90 | 1732 | 1 | 82 | 95 |  |
| L.WAAFPPDVA.G | Y | 20.98 | 972.4705 | -0.5 | 487.2423 | 2 | 13.51 | 2026 | 4 | 134 | 142 |  |
| total 3 peptides |  |  |  |  |  |  |  |  |  |  |  |  |
| --- | --- | --- | --- | --- | --- | --- | --- | --- | --- | --- | --- | --- |

tr|A0A7J6CHK6|A0A7J6CHK6\_9TELE

back to list

  

| Protein Coverage
| Supporting Peptides
|

Protein Coverage:

Supporting Peptides:

| Peptide | Uniq | -10lgP | Mass | ppm | m/z | z | RT | Scan | #Spec | Start | End | PTM |
| --- | --- | --- | --- | --- | --- | --- | --- | --- | --- | --- | --- | --- |
| F.GEKLKGADPEDVILS.A | Y | 28.44 | 1569.8250 | -0.2 | 785.9196 | 2 | 11.67 | 1690 | 1 | 89 | 103 |  |
| F.GEKLKGADPEDVIL.S | Y | 24.67 | 1482.7930 | -0.8 | 742.4032 | 2 | 11.90 | 1732 | 1 | 89 | 102 |  |
| L.WAAFPPDVA.G | Y | 20.98 | 972.4705 | -0.5 | 487.2423 | 2 | 13.51 | 2026 | 4 | 141 | 149 |  |
| total 3 peptides |  |  |  |  |  |  |  |  |  |  |  |  |
| --- | --- | --- | --- | --- | --- | --- | --- | --- | --- | --- | --- | --- |

tr|A0A6P7II88|A0A6P7II88\_9TELE

back to list

  

| Protein Coverage
| Supporting Peptides
|

Protein Coverage:

Supporting Peptides:

| Peptide | Uniq | -10lgP | Mass | ppm | m/z | z | RT | Scan | #Spec | Start | End | PTM |
| --- | --- | --- | --- | --- | --- | --- | --- | --- | --- | --- | --- | --- |
| F.GEKLKGADPEDVILS.A | Y | 28.44 | 1569.8250 | -0.2 | 785.9196 | 2 | 11.67 | 1690 | 1 | 90 | 104 |  |
| F.GEKLKGADPEDVIL.S | Y | 24.67 | 1482.7930 | -0.8 | 742.4032 | 2 | 11.90 | 1732 | 1 | 90 | 103 |  |
| M.WAAFPPDVA.G | Y | 20.98 | 972.4705 | -0.5 | 487.2423 | 2 | 13.51 | 2026 | 4 | 142 | 150 |  |
| total 3 peptides |  |  |  |  |  |  |  |  |  |  |  |  |
| --- | --- | --- | --- | --- | --- | --- | --- | --- | --- | --- | --- | --- |

tr|A0A668VT67|A0A668VT67\_OREAU

back to list

  

| Protein Coverage
| Supporting Peptides
|

Protein Coverage:

Supporting Peptides:

| Peptide | Uniq | -10lgP | Mass | ppm | m/z | z | RT | Scan | #Spec | Start | End | PTM |
| --- | --- | --- | --- | --- | --- | --- | --- | --- | --- | --- | --- | --- |
| F.GEKLKGADPEDVILS.A | Y | 28.44 | 1569.8250 | -0.2 | 785.9196 | 2 | 11.67 | 1690 | 1 | 97 | 111 |  |
| F.GEKLKGADPEDVIL.S | Y | 24.67 | 1482.7930 | -0.8 | 742.4032 | 2 | 11.90 | 1732 | 1 | 97 | 110 |  |
| L.WAAFPPDVA.G | Y | 20.98 | 972.4705 | -0.5 | 487.2423 | 2 | 13.51 | 2026 | 4 | 149 | 157 |  |
| total 3 peptides |  |  |  |  |  |  |  |  |  |  |  |  |
| --- | --- | --- | --- | --- | --- | --- | --- | --- | --- | --- | --- | --- |

tr|F8U029|F8U029\_EPIBR

back to list

  

| Protein Coverage
| Supporting Peptides
|

Protein Coverage:

Supporting Peptides:

| Peptide | Uniq | -10lgP | Mass | ppm | m/z | z | RT | Scan | #Spec | Start | End | PTM |
| --- | --- | --- | --- | --- | --- | --- | --- | --- | --- | --- | --- | --- |
| F.GEKLKGADPEDVILS.S | Y | 28.44 | 1569.8250 | -0.2 | 785.9196 | 2 | 11.67 | 1690 | 1 | 90 | 104 |  |
| F.GEKLKGADPEDVIL.S | Y | 24.67 | 1482.7930 | -0.8 | 742.4032 | 2 | 11.90 | 1732 | 1 | 90 | 103 |  |
| M.WAAFPPDVA.G | Y | 20.98 | 972.4705 | -0.5 | 487.2423 | 2 | 13.51 | 2026 | 4 | 142 | 150 |  |
| total 3 peptides |  |  |  |  |  |  |  |  |  |  |  |  |
| --- | --- | --- | --- | --- | --- | --- | --- | --- | --- | --- | --- | --- |

tr|A0A3B5RBY4|A0A3B5RBY4\_XIPMA

back to list

  

| Protein Coverage
| Supporting Peptides
|

Protein Coverage:

Supporting Peptides:

| Peptide | Uniq | -10lgP | Mass | ppm | m/z | z | RT | Scan | #Spec | Start | End | PTM |
| --- | --- | --- | --- | --- | --- | --- | --- | --- | --- | --- | --- | --- |
| F.GEKLKGADPEDVILS.A | Y | 28.44 | 1569.8250 | -0.2 | 785.9196 | 2 | 11.67 | 1690 | 1 | 169 | 183 |  |
| F.GEKLKGADPEDVIL.S | Y | 24.67 | 1482.7930 | -0.8 | 742.4032 | 2 | 11.90 | 1732 | 1 | 169 | 182 |  |
| M.WAAFPPDVA.G | Y | 20.98 | 972.4705 | -0.5 | 487.2423 | 2 | 13.51 | 2026 | 4 | 221 | 229 |  |
| total 3 peptides |  |  |  |  |  |  |  |  |  |  |  |  |
| --- | --- | --- | --- | --- | --- | --- | --- | --- | --- | --- | --- | --- |

tr|A0A7J5Y2W4|A0A7J5Y2W4\_DISMA

back to list

  

| Protein Coverage
| Supporting Peptides
|

Protein Coverage:

Supporting Peptides:

| Peptide | Uniq | -10lgP | Mass | ppm | m/z | z | RT | Scan | #Spec | Start | End | PTM |
| --- | --- | --- | --- | --- | --- | --- | --- | --- | --- | --- | --- | --- |
| L.AVDPVYPPGPPAFPK.V | N | 36.21 | 1550.8132 | -1.1 | 776.4130 | 2 | 12.35 | 1817 | 1 | 79 | 93 |  |
| C.TGPIKF.D | Y | 19.01 | 661.3799 | -0.8 | 331.6970 | 2 | 11.39 | 1639 | 1 | 564 | 569 |  |
| total 2 peptides |  |  |  |  |  |  |  |  |  |  |  |  |
| --- | --- | --- | --- | --- | --- | --- | --- | --- | --- | --- | --- | --- |

tr|A0A4Z2HXD4|A0A4Z2HXD4\_9TELE

back to list

  

| Protein Coverage
| Supporting Peptides
|

Protein Coverage:

Supporting Peptides:

| Peptide | Uniq | -10lgP | Mass | ppm | m/z | z | RT | Scan | #Spec | Start | End | PTM |
| --- | --- | --- | --- | --- | --- | --- | --- | --- | --- | --- | --- | --- |
| L.AVDPVYPPGPPAFPK.V | N | 36.21 | 1550.8132 | -1.1 | 776.4130 | 2 | 12.35 | 1817 | 1 | 60 | 74 |  |
| C.TGPIKF.D | Y | 19.01 | 661.3799 | -0.8 | 331.6970 | 2 | 11.39 | 1639 | 1 | 558 | 563 |  |
| total 2 peptides |  |  |  |  |  |  |  |  |  |  |  |  |
| --- | --- | --- | --- | --- | --- | --- | --- | --- | --- | --- | --- | --- |

tr|A0A672HWD2|A0A672HWD2\_SALFA

back to list

  

| Protein Coverage
| Supporting Peptides
|

Protein Coverage:

Supporting Peptides:

| Peptide | Uniq | -10lgP | Mass | ppm | m/z | z | RT | Scan | #Spec | Start | End | PTM |
| --- | --- | --- | --- | --- | --- | --- | --- | --- | --- | --- | --- | --- |
| V.WIDPDDFPLLLPH.W | Y | 32.22 | 1576.7925 | 0.6 | 789.4040 | 2 | 15.34 | 2344 | 1 | 295 | 307 |  |
| F.MDDPVVIPGKPY.T | Y | 24.31 | 1329.6638 | -1.0 | 665.8385 | 2 | 12.25 | 1798 | 1 | 211 | 222 |  |
| total 2 peptides |  |  |  |  |  |  |  |  |  |  |  |  |
| --- | --- | --- | --- | --- | --- | --- | --- | --- | --- | --- | --- | --- |

tr|A0A672HWL2|A0A672HWL2\_SALFA

back to list

  

| Protein Coverage
| Supporting Peptides
|

Protein Coverage:

Supporting Peptides:

| Peptide | Uniq | -10lgP | Mass | ppm | m/z | z | RT | Scan | #Spec | Start | End | PTM |
| --- | --- | --- | --- | --- | --- | --- | --- | --- | --- | --- | --- | --- |
| V.WIDPDDFPLLLPH.W | Y | 32.22 | 1576.7925 | 0.6 | 789.4040 | 2 | 15.34 | 2344 | 1 | 302 | 314 |  |
| F.MDDPVVIPGKPY.T | Y | 24.31 | 1329.6638 | -1.0 | 665.8385 | 2 | 12.25 | 1798 | 1 | 218 | 229 |  |
| total 2 peptides |  |  |  |  |  |  |  |  |  |  |  |  |
| --- | --- | --- | --- | --- | --- | --- | --- | --- | --- | --- | --- | --- |

tr|A0A672HXF4|A0A672HXF4\_SALFA

back to list

  

| Protein Coverage
| Supporting Peptides
|

Protein Coverage:

Supporting Peptides:

| Peptide | Uniq | -10lgP | Mass | ppm | m/z | z | RT | Scan | #Spec | Start | End | PTM |
| --- | --- | --- | --- | --- | --- | --- | --- | --- | --- | --- | --- | --- |
| V.WIDPDDFPLLLPH.W | Y | 32.22 | 1576.7925 | 0.6 | 789.4040 | 2 | 15.34 | 2344 | 1 | 313 | 325 |  |
| F.MDDPVVIPGKPY.T | Y | 24.31 | 1329.6638 | -1.0 | 665.8385 | 2 | 12.25 | 1798 | 1 | 229 | 240 |  |
| total 2 peptides |  |  |  |  |  |  |  |  |  |  |  |  |
| --- | --- | --- | --- | --- | --- | --- | --- | --- | --- | --- | --- | --- |

tr|A0A672HXA9|A0A672HXA9\_SALFA

back to list

  

| Protein Coverage
| Supporting Peptides
|

Protein Coverage:

Supporting Peptides:

| Peptide | Uniq | -10lgP | Mass | ppm | m/z | z | RT | Scan | #Spec | Start | End | PTM |
| --- | --- | --- | --- | --- | --- | --- | --- | --- | --- | --- | --- | --- |
| V.WIDPDDFPLLLPH.W | Y | 32.22 | 1576.7925 | 0.6 | 789.4040 | 2 | 15.34 | 2344 | 1 | 322 | 334 |  |
| F.MDDPVVIPGKPY.T | Y | 24.31 | 1329.6638 | -1.0 | 665.8385 | 2 | 12.25 | 1798 | 1 | 238 | 249 |  |
| total 2 peptides |  |  |  |  |  |  |  |  |  |  |  |  |
| --- | --- | --- | --- | --- | --- | --- | --- | --- | --- | --- | --- | --- |

tr|A0A3Q4GK85|A0A3Q4GK85\_NEOBR

back to list

  

| Protein Coverage
| Supporting Peptides
|

Protein Coverage:

Supporting Peptides:

| Peptide | Uniq | -10lgP | Mass | ppm | m/z | z | RT | Scan | #Spec | Start | End | PTM |
| --- | --- | --- | --- | --- | --- | --- | --- | --- | --- | --- | --- | --- |
| R.KVDSELPVDDVFGQ.V | Y | 31.17 | 1546.7515 | -1.0 | 774.3822 | 2 | 13.13 | 1957 | 1 | 171 | 184 |  |
| K.VDSELPVDDVFGQ.V | Y | 19.90 | 1418.6565 | -0.8 | 710.3350 | 2 | 13.87 | 2087 | 1 | 172 | 184 |  |
| total 2 peptides |  |  |  |  |  |  |  |  |  |  |  |  |
| --- | --- | --- | --- | --- | --- | --- | --- | --- | --- | --- | --- | --- |

tr|A0A3Q4GS43|A0A3Q4GS43\_NEOBR

back to list

  

| Protein Coverage
| Supporting Peptides
|

Protein Coverage:

Supporting Peptides:

| Peptide | Uniq | -10lgP | Mass | ppm | m/z | z | RT | Scan | #Spec | Start | End | PTM |
| --- | --- | --- | --- | --- | --- | --- | --- | --- | --- | --- | --- | --- |
| R.KVDSELPVDDVFGQ.V | Y | 31.17 | 1546.7515 | -1.0 | 774.3822 | 2 | 13.13 | 1957 | 1 | 172 | 185 |  |
| K.VDSELPVDDVFGQ.V | Y | 19.90 | 1418.6565 | -0.8 | 710.3350 | 2 | 13.87 | 2087 | 1 | 173 | 185 |  |
| total 2 peptides |  |  |  |  |  |  |  |  |  |  |  |  |
| --- | --- | --- | --- | --- | --- | --- | --- | --- | --- | --- | --- | --- |

tr|A0A3P8P8S3|A0A3P8P8S3\_ASTCA

back to list

  

| Protein Coverage
| Supporting Peptides
|

Protein Coverage:

Supporting Peptides:

| Peptide | Uniq | -10lgP | Mass | ppm | m/z | z | RT | Scan | #Spec | Start | End | PTM |
| --- | --- | --- | --- | --- | --- | --- | --- | --- | --- | --- | --- | --- |
| R.KVDSELPVDDVFGQ.V | Y | 31.17 | 1546.7515 | -1.0 | 774.3822 | 2 | 13.13 | 1957 | 1 | 172 | 185 |  |
| K.VDSELPVDDVFGQ.V | Y | 19.90 | 1418.6565 | -0.8 | 710.3350 | 2 | 13.87 | 2087 | 1 | 173 | 185 |  |
| total 2 peptides |  |  |  |  |  |  |  |  |  |  |  |  |
| --- | --- | --- | --- | --- | --- | --- | --- | --- | --- | --- | --- | --- |

tr|A0A3Q1HW42|A0A3Q1HW42\_9TELE

back to list

  

| Protein Coverage
| Supporting Peptides
|

Protein Coverage:

Supporting Peptides:

| Peptide | Uniq | -10lgP | Mass | ppm | m/z | z | RT | Scan | #Spec | Start | End | PTM |
| --- | --- | --- | --- | --- | --- | --- | --- | --- | --- | --- | --- | --- |
| R.KVDSELPVDDVFGQ.V | Y | 31.17 | 1546.7515 | -1.0 | 774.3822 | 2 | 13.13 | 1957 | 1 | 172 | 185 |  |
| K.VDSELPVDDVFGQ.V | Y | 19.90 | 1418.6565 | -0.8 | 710.3350 | 2 | 13.87 | 2087 | 1 | 173 | 185 |  |
| total 2 peptides |  |  |  |  |  |  |  |  |  |  |  |  |
| --- | --- | --- | --- | --- | --- | --- | --- | --- | --- | --- | --- | --- |

tr|A0A3P9CWL7|A0A3P9CWL7\_9CICH

back to list

  

| Protein Coverage
| Supporting Peptides
|

Protein Coverage:

Supporting Peptides:

| Peptide | Uniq | -10lgP | Mass | ppm | m/z | z | RT | Scan | #Spec | Start | End | PTM |
| --- | --- | --- | --- | --- | --- | --- | --- | --- | --- | --- | --- | --- |
| R.KVDSELPVDDVFGQ.V | Y | 31.17 | 1546.7515 | -1.0 | 774.3822 | 2 | 13.13 | 1957 | 1 | 172 | 185 |  |
| K.VDSELPVDDVFGQ.V | Y | 19.90 | 1418.6565 | -0.8 | 710.3350 | 2 | 13.87 | 2087 | 1 | 173 | 185 |  |
| total 2 peptides |  |  |  |  |  |  |  |  |  |  |  |  |
| --- | --- | --- | --- | --- | --- | --- | --- | --- | --- | --- | --- | --- |

tr|A0A3B5BIY5|A0A3B5BIY5\_9TELE

back to list

  

| Protein Coverage
| Supporting Peptides
|

Protein Coverage:

Supporting Peptides:

| Peptide | Uniq | -10lgP | Mass | ppm | m/z | z | RT | Scan | #Spec | Start | End | PTM |
| --- | --- | --- | --- | --- | --- | --- | --- | --- | --- | --- | --- | --- |
| R.KVDSELPVDDVFGQ.V | Y | 31.17 | 1546.7515 | -1.0 | 774.3822 | 2 | 13.13 | 1957 | 1 | 172 | 185 |  |
| K.VDSELPVDDVFGQ.V | Y | 19.90 | 1418.6565 | -0.8 | 710.3350 | 2 | 13.87 | 2087 | 1 | 173 | 185 |  |
| total 2 peptides |  |  |  |  |  |  |  |  |  |  |  |  |
| --- | --- | --- | --- | --- | --- | --- | --- | --- | --- | --- | --- | --- |

tr|A0A3Q2VY92|A0A3Q2VY92\_HAPBU

back to list

  

| Protein Coverage
| Supporting Peptides
|

Protein Coverage:

Supporting Peptides:

| Peptide | Uniq | -10lgP | Mass | ppm | m/z | z | RT | Scan | #Spec | Start | End | PTM |
| --- | --- | --- | --- | --- | --- | --- | --- | --- | --- | --- | --- | --- |
| R.KVDSELPVDDVFGQ.V | Y | 31.17 | 1546.7515 | -1.0 | 774.3822 | 2 | 13.13 | 1957 | 1 | 172 | 185 |  |
| K.VDSELPVDDVFGQ.V | Y | 19.90 | 1418.6565 | -0.8 | 710.3350 | 2 | 13.87 | 2087 | 1 | 173 | 185 |  |
| total 2 peptides |  |  |  |  |  |  |  |  |  |  |  |  |
| --- | --- | --- | --- | --- | --- | --- | --- | --- | --- | --- | --- | --- |

tr|A0A3B4FMK3|A0A3B4FMK3\_9CICH

back to list

  

| Protein Coverage
| Supporting Peptides
|

Protein Coverage:

Supporting Peptides:

| Peptide | Uniq | -10lgP | Mass | ppm | m/z | z | RT | Scan | #Spec | Start | End | PTM |
| --- | --- | --- | --- | --- | --- | --- | --- | --- | --- | --- | --- | --- |
| R.KVDSELPVDDVFGQ.V | Y | 31.17 | 1546.7515 | -1.0 | 774.3822 | 2 | 13.13 | 1957 | 1 | 172 | 185 |  |
| K.VDSELPVDDVFGQ.V | Y | 19.90 | 1418.6565 | -0.8 | 710.3350 | 2 | 13.87 | 2087 | 1 | 173 | 185 |  |
| total 2 peptides |  |  |  |  |  |  |  |  |  |  |  |  |
| --- | --- | --- | --- | --- | --- | --- | --- | --- | --- | --- | --- | --- |

tr|A0A3Q3CWR9|A0A3Q3CWR9\_HAPBU

back to list

  

| Protein Coverage
| Supporting Peptides
|

Protein Coverage:

Supporting Peptides:

| Peptide | Uniq | -10lgP | Mass | ppm | m/z | z | RT | Scan | #Spec | Start | End | PTM |
| --- | --- | --- | --- | --- | --- | --- | --- | --- | --- | --- | --- | --- |
| R.KVDSELPVDDVFGQ.V | Y | 31.17 | 1546.7515 | -1.0 | 774.3822 | 2 | 13.13 | 1957 | 1 | 190 | 203 |  |
| K.VDSELPVDDVFGQ.V | Y | 19.90 | 1418.6565 | -0.8 | 710.3350 | 2 | 13.87 | 2087 | 1 | 191 | 203 |  |
| total 2 peptides |  |  |  |  |  |  |  |  |  |  |  |  |
| --- | --- | --- | --- | --- | --- | --- | --- | --- | --- | --- | --- | --- |

tr|A0A3P8P8V3|A0A3P8P8V3\_ASTCA

back to list

  

| Protein Coverage
| Supporting Peptides
|

Protein Coverage:

Supporting Peptides:

| Peptide | Uniq | -10lgP | Mass | ppm | m/z | z | RT | Scan | #Spec | Start | End | PTM |
| --- | --- | --- | --- | --- | --- | --- | --- | --- | --- | --- | --- | --- |
| R.KVDSELPVDDVFGQ.V | Y | 31.17 | 1546.7515 | -1.0 | 774.3822 | 2 | 13.13 | 1957 | 1 | 190 | 203 |  |
| K.VDSELPVDDVFGQ.V | Y | 19.90 | 1418.6565 | -0.8 | 710.3350 | 2 | 13.87 | 2087 | 1 | 191 | 203 |  |
| total 2 peptides |  |  |  |  |  |  |  |  |  |  |  |  |
| --- | --- | --- | --- | --- | --- | --- | --- | --- | --- | --- | --- | --- |

tr|A0A665WT54|A0A665WT54\_ECHNA

back to list

  

| Protein Coverage
| Supporting Peptides
|

Protein Coverage:

Supporting Peptides:

| Peptide | Uniq | -10lgP | Mass | ppm | m/z | z | RT | Scan | #Spec | Start | End | PTM |
| --- | --- | --- | --- | --- | --- | --- | --- | --- | --- | --- | --- | --- |
| R.MDSPMPIIPH.Q | Y | 28.61 | 1136.5359 | -2.5 | 569.2738 | 2 | 12.55 | 1852 | 1 | 97 | 106 |  |
| M.DSPMPIIPH.Q | Y | 19.82 | 1005.4954 | -1.1 | 503.7544 | 2 | 12.29 | 1806 | 1 | 98 | 106 |  |
| total 2 peptides |  |  |  |  |  |  |  |  |  |  |  |  |
| --- | --- | --- | --- | --- | --- | --- | --- | --- | --- | --- | --- | --- |

tr|A0A671N228|A0A671N228\_9TELE

back to list

  

| Protein Coverage
| Supporting Peptides
|

Protein Coverage:

Supporting Peptides:

| Peptide | Uniq | -10lgP | Mass | ppm | m/z | z | RT | Scan | #Spec | Start | End | PTM |
| --- | --- | --- | --- | --- | --- | --- | --- | --- | --- | --- | --- | --- |
| R.MDSPMPIIPH.Q | Y | 28.61 | 1136.5359 | -2.5 | 569.2738 | 2 | 12.55 | 1852 | 1 | 97 | 106 |  |
| M.DSPMPIIPH.Q | Y | 19.82 | 1005.4954 | -1.1 | 503.7544 | 2 | 12.29 | 1806 | 1 | 98 | 106 |  |
| total 2 peptides |  |  |  |  |  |  |  |  |  |  |  |  |
| --- | --- | --- | --- | --- | --- | --- | --- | --- | --- | --- | --- | --- |

tr|A0A3Q3Q7K2|A0A3Q3Q7K2\_MONAL

back to list

  

| Protein Coverage
| Supporting Peptides
|

Protein Coverage:

Supporting Peptides:

| Peptide | Uniq | -10lgP | Mass | ppm | m/z | z | RT | Scan | #Spec | Start | End | PTM |
| --- | --- | --- | --- | --- | --- | --- | --- | --- | --- | --- | --- | --- |
| R.MDSPMPIIPH.Q | Y | 28.61 | 1136.5359 | -2.5 | 569.2738 | 2 | 12.55 | 1852 | 1 | 97 | 106 |  |
| M.DSPMPIIPH.Q | Y | 19.82 | 1005.4954 | -1.1 | 503.7544 | 2 | 12.29 | 1806 | 1 | 98 | 106 |  |
| total 2 peptides |  |  |  |  |  |  |  |  |  |  |  |  |
| --- | --- | --- | --- | --- | --- | --- | --- | --- | --- | --- | --- | --- |

tr|A0A671MU99|A0A671MU99\_9TELE

back to list

  

| Protein Coverage
| Supporting Peptides
|

Protein Coverage:

Supporting Peptides:

| Peptide | Uniq | -10lgP | Mass | ppm | m/z | z | RT | Scan | #Spec | Start | End | PTM |
| --- | --- | --- | --- | --- | --- | --- | --- | --- | --- | --- | --- | --- |
| R.MDSPMPIIPH.Q | Y | 28.61 | 1136.5359 | -2.5 | 569.2738 | 2 | 12.55 | 1852 | 1 | 97 | 106 |  |
| M.DSPMPIIPH.Q | Y | 19.82 | 1005.4954 | -1.1 | 503.7544 | 2 | 12.29 | 1806 | 1 | 98 | 106 |  |
| total 2 peptides |  |  |  |  |  |  |  |  |  |  |  |  |
| --- | --- | --- | --- | --- | --- | --- | --- | --- | --- | --- | --- | --- |

tr|A0A672KK19|A0A672KK19\_SINGR

back to list

  

| Protein Coverage
| Supporting Peptides
|

Protein Coverage:

Supporting Peptides:

| Peptide | Uniq | -10lgP | Mass | ppm | m/z | z | RT | Scan | #Spec | Start | End | PTM |
| --- | --- | --- | --- | --- | --- | --- | --- | --- | --- | --- | --- | --- |
| R.MDSPMPIIPH.Q | Y | 28.61 | 1136.5359 | -2.5 | 569.2738 | 2 | 12.55 | 1852 | 1 | 97 | 106 |  |
| M.DSPMPIIPH.Q | Y | 19.82 | 1005.4954 | -1.1 | 503.7544 | 2 | 12.29 | 1806 | 1 | 98 | 106 |  |
| total 2 peptides |  |  |  |  |  |  |  |  |  |  |  |  |
| --- | --- | --- | --- | --- | --- | --- | --- | --- | --- | --- | --- | --- |

tr|A0A673LYE6|A0A673LYE6\_9TELE

back to list

  

| Protein Coverage
| Supporting Peptides
|

Protein Coverage:

Supporting Peptides:

| Peptide | Uniq | -10lgP | Mass | ppm | m/z | z | RT | Scan | #Spec | Start | End | PTM |
| --- | --- | --- | --- | --- | --- | --- | --- | --- | --- | --- | --- | --- |
| R.MDSPMPIIPH.Q | Y | 28.61 | 1136.5359 | -2.5 | 569.2738 | 2 | 12.55 | 1852 | 1 | 97 | 106 |  |
| M.DSPMPIIPH.Q | Y | 19.82 | 1005.4954 | -1.1 | 503.7544 | 2 | 12.29 | 1806 | 1 | 98 | 106 |  |
| total 2 peptides |  |  |  |  |  |  |  |  |  |  |  |  |
| --- | --- | --- | --- | --- | --- | --- | --- | --- | --- | --- | --- | --- |

tr|A0A3P8VEA1|A0A3P8VEA1\_CYNSE

back to list

  

| Protein Coverage
| Supporting Peptides
|

Protein Coverage:

Supporting Peptides:

| Peptide | Uniq | -10lgP | Mass | ppm | m/z | z | RT | Scan | #Spec | Start | End | PTM |
| --- | --- | --- | --- | --- | --- | --- | --- | --- | --- | --- | --- | --- |
| R.MDSPMPIIPH.Q | Y | 28.61 | 1136.5359 | -2.5 | 569.2738 | 2 | 12.55 | 1852 | 1 | 97 | 106 |  |
| M.DSPMPIIPH.Q | Y | 19.82 | 1005.4954 | -1.1 | 503.7544 | 2 | 12.29 | 1806 | 1 | 98 | 106 |  |
| total 2 peptides |  |  |  |  |  |  |  |  |  |  |  |  |
| --- | --- | --- | --- | --- | --- | --- | --- | --- | --- | --- | --- | --- |

tr|A0A3Q1II99|A0A3Q1II99\_ANATE

back to list

  

| Protein Coverage
| Supporting Peptides
|

Protein Coverage:

Supporting Peptides:

| Peptide | Uniq | -10lgP | Mass | ppm | m/z | z | RT | Scan | #Spec | Start | End | PTM |
| --- | --- | --- | --- | --- | --- | --- | --- | --- | --- | --- | --- | --- |
| R.MDSPMPIIPH.Q | Y | 28.61 | 1136.5359 | -2.5 | 569.2738 | 2 | 12.55 | 1852 | 1 | 97 | 106 |  |
| M.DSPMPIIPH.Q | Y | 19.82 | 1005.4954 | -1.1 | 503.7544 | 2 | 12.29 | 1806 | 1 | 98 | 106 |  |
| total 2 peptides |  |  |  |  |  |  |  |  |  |  |  |  |
| --- | --- | --- | --- | --- | --- | --- | --- | --- | --- | --- | --- | --- |

tr|A0A6P7L9I7|A0A6P7L9I7\_BETSP

back to list

  

| Protein Coverage
| Supporting Peptides
|

Protein Coverage:

Supporting Peptides:

| Peptide | Uniq | -10lgP | Mass | ppm | m/z | z | RT | Scan | #Spec | Start | End | PTM |
| --- | --- | --- | --- | --- | --- | --- | --- | --- | --- | --- | --- | --- |
| R.MDSPMPIIPH.Q | Y | 28.61 | 1136.5359 | -2.5 | 569.2738 | 2 | 12.55 | 1852 | 1 | 97 | 106 |  |
| M.DSPMPIIPH.Q | Y | 19.82 | 1005.4954 | -1.1 | 503.7544 | 2 | 12.29 | 1806 | 1 | 98 | 106 |  |
| total 2 peptides |  |  |  |  |  |  |  |  |  |  |  |  |
| --- | --- | --- | --- | --- | --- | --- | --- | --- | --- | --- | --- | --- |

tr|A0A3P8X8T4|A0A3P8X8T4\_ESOLU

back to list

  

| Protein Coverage
| Supporting Peptides
|

Protein Coverage:

Supporting Peptides:

| Peptide | Uniq | -10lgP | Mass | ppm | m/z | z | RT | Scan | #Spec | Start | End | PTM |
| --- | --- | --- | --- | --- | --- | --- | --- | --- | --- | --- | --- | --- |
| R.MDSPMPIIPH.Q | Y | 28.61 | 1136.5359 | -2.5 | 569.2738 | 2 | 12.55 | 1852 | 1 | 96 | 105 |  |
| M.DSPMPIIPH.Q | Y | 19.82 | 1005.4954 | -1.1 | 503.7544 | 2 | 12.29 | 1806 | 1 | 97 | 105 |  |
| total 2 peptides |  |  |  |  |  |  |  |  |  |  |  |  |
| --- | --- | --- | --- | --- | --- | --- | --- | --- | --- | --- | --- | --- |

tr|A0A3Q0R4X6|A0A3Q0R4X6\_AMPCI

back to list

  

| Protein Coverage
| Supporting Peptides
|

Protein Coverage:

Supporting Peptides:

| Peptide | Uniq | -10lgP | Mass | ppm | m/z | z | RT | Scan | #Spec | Start | End | PTM |
| --- | --- | --- | --- | --- | --- | --- | --- | --- | --- | --- | --- | --- |
| R.MDSPMPIIPH.Q | Y | 28.61 | 1136.5359 | -2.5 | 569.2738 | 2 | 12.55 | 1852 | 1 | 97 | 106 |  |
| M.DSPMPIIPH.Q | Y | 19.82 | 1005.4954 | -1.1 | 503.7544 | 2 | 12.29 | 1806 | 1 | 98 | 106 |  |
| total 2 peptides |  |  |  |  |  |  |  |  |  |  |  |  |
| --- | --- | --- | --- | --- | --- | --- | --- | --- | --- | --- | --- | --- |

tr|A0A3P8VK01|A0A3P8VK01\_CYNSE

back to list

  

| Protein Coverage
| Supporting Peptides
|

Protein Coverage:

Supporting Peptides:

| Peptide | Uniq | -10lgP | Mass | ppm | m/z | z | RT | Scan | #Spec | Start | End | PTM |
| --- | --- | --- | --- | --- | --- | --- | --- | --- | --- | --- | --- | --- |
| R.MDSPMPIIPH.Q | Y | 28.61 | 1136.5359 | -2.5 | 569.2738 | 2 | 12.55 | 1852 | 1 | 97 | 106 |  |
| M.DSPMPIIPH.Q | Y | 19.82 | 1005.4954 | -1.1 | 503.7544 | 2 | 12.29 | 1806 | 1 | 98 | 106 |  |
| total 2 peptides |  |  |  |  |  |  |  |  |  |  |  |  |
| --- | --- | --- | --- | --- | --- | --- | --- | --- | --- | --- | --- | --- |

tr|A0A3P8XBI5|A0A3P8XBI5\_ESOLU

back to list

  

| Protein Coverage
| Supporting Peptides
|

Protein Coverage:

Supporting Peptides:

| Peptide | Uniq | -10lgP | Mass | ppm | m/z | z | RT | Scan | #Spec | Start | End | PTM |
| --- | --- | --- | --- | --- | --- | --- | --- | --- | --- | --- | --- | --- |
| R.MDSPMPIIPH.Q | Y | 28.61 | 1136.5359 | -2.5 | 569.2738 | 2 | 12.55 | 1852 | 1 | 96 | 105 |  |
| M.DSPMPIIPH.Q | Y | 19.82 | 1005.4954 | -1.1 | 503.7544 | 2 | 12.29 | 1806 | 1 | 97 | 105 |  |
| total 2 peptides |  |  |  |  |  |  |  |  |  |  |  |  |
| --- | --- | --- | --- | --- | --- | --- | --- | --- | --- | --- | --- | --- |

tr|A0A3Q1H919|A0A3Q1H919\_ANATE

back to list

  

| Protein Coverage
| Supporting Peptides
|

Protein Coverage:

Supporting Peptides:

| Peptide | Uniq | -10lgP | Mass | ppm | m/z | z | RT | Scan | #Spec | Start | End | PTM |
| --- | --- | --- | --- | --- | --- | --- | --- | --- | --- | --- | --- | --- |
| R.MDSPMPIIPH.Q | Y | 28.61 | 1136.5359 | -2.5 | 569.2738 | 2 | 12.55 | 1852 | 1 | 97 | 106 |  |
| M.DSPMPIIPH.Q | Y | 19.82 | 1005.4954 | -1.1 | 503.7544 | 2 | 12.29 | 1806 | 1 | 98 | 106 |  |
| total 2 peptides |  |  |  |  |  |  |  |  |  |  |  |  |
| --- | --- | --- | --- | --- | --- | --- | --- | --- | --- | --- | --- | --- |

tr|A0A3Q3KN21|A0A3Q3KN21\_9TELE

back to list

  

| Protein Coverage
| Supporting Peptides
|

Protein Coverage:

Supporting Peptides:

| Peptide | Uniq | -10lgP | Mass | ppm | m/z | z | RT | Scan | #Spec | Start | End | PTM |
| --- | --- | --- | --- | --- | --- | --- | --- | --- | --- | --- | --- | --- |
| R.MDSPMPIIPH.Q | Y | 28.61 | 1136.5359 | -2.5 | 569.2738 | 2 | 12.55 | 1852 | 1 | 97 | 106 |  |
| M.DSPMPIIPH.Q | Y | 19.82 | 1005.4954 | -1.1 | 503.7544 | 2 | 12.29 | 1806 | 1 | 98 | 106 |  |
| total 2 peptides |  |  |  |  |  |  |  |  |  |  |  |  |
| --- | --- | --- | --- | --- | --- | --- | --- | --- | --- | --- | --- | --- |

tr|A0A6P7L9I9|A0A6P7L9I9\_BETSP

back to list

  

| Protein Coverage
| Supporting Peptides
|

Protein Coverage:

Supporting Peptides:

| Peptide | Uniq | -10lgP | Mass | ppm | m/z | z | RT | Scan | #Spec | Start | End | PTM |
| --- | --- | --- | --- | --- | --- | --- | --- | --- | --- | --- | --- | --- |
| R.MDSPMPIIPH.Q | Y | 28.61 | 1136.5359 | -2.5 | 569.2738 | 2 | 12.55 | 1852 | 1 | 97 | 106 |  |
| M.DSPMPIIPH.Q | Y | 19.82 | 1005.4954 | -1.1 | 503.7544 | 2 | 12.29 | 1806 | 1 | 98 | 106 |  |
| total 2 peptides |  |  |  |  |  |  |  |  |  |  |  |  |
| --- | --- | --- | --- | --- | --- | --- | --- | --- | --- | --- | --- | --- |

tr|A0A3P8VE68|A0A3P8VE68\_CYNSE

back to list

  

| Protein Coverage
| Supporting Peptides
|

Protein Coverage:

Supporting Peptides:

| Peptide | Uniq | -10lgP | Mass | ppm | m/z | z | RT | Scan | #Spec | Start | End | PTM |
| --- | --- | --- | --- | --- | --- | --- | --- | --- | --- | --- | --- | --- |
| R.MDSPMPIIPH.Q | Y | 28.61 | 1136.5359 | -2.5 | 569.2738 | 2 | 12.55 | 1852 | 1 | 97 | 106 |  |
| M.DSPMPIIPH.Q | Y | 19.82 | 1005.4954 | -1.1 | 503.7544 | 2 | 12.29 | 1806 | 1 | 98 | 106 |  |
| total 2 peptides |  |  |  |  |  |  |  |  |  |  |  |  |
| --- | --- | --- | --- | --- | --- | --- | --- | --- | --- | --- | --- | --- |

tr|A0A6G0HYN5|A0A6G0HYN5\_LARCR

back to list

  

| Protein Coverage
| Supporting Peptides
|

Protein Coverage:

Supporting Peptides:

| Peptide | Uniq | -10lgP | Mass | ppm | m/z | z | RT | Scan | #Spec | Start | End | PTM |
| --- | --- | --- | --- | --- | --- | --- | --- | --- | --- | --- | --- | --- |
| R.MDSPMPIIPH.Q | Y | 28.61 | 1136.5359 | -2.5 | 569.2738 | 2 | 12.55 | 1852 | 1 | 97 | 106 |  |
| M.DSPMPIIPH.Q | Y | 19.82 | 1005.4954 | -1.1 | 503.7544 | 2 | 12.29 | 1806 | 1 | 98 | 106 |  |
| total 2 peptides |  |  |  |  |  |  |  |  |  |  |  |  |
| --- | --- | --- | --- | --- | --- | --- | --- | --- | --- | --- | --- | --- |

tr|A0A3P8ZCR8|A0A3P8ZCR8\_ESOLU

back to list

  

| Protein Coverage
| Supporting Peptides
|

Protein Coverage:

Supporting Peptides:

| Peptide | Uniq | -10lgP | Mass | ppm | m/z | z | RT | Scan | #Spec | Start | End | PTM |
| --- | --- | --- | --- | --- | --- | --- | --- | --- | --- | --- | --- | --- |
| R.MDSPMPIIPH.Q | Y | 28.61 | 1136.5359 | -2.5 | 569.2738 | 2 | 12.55 | 1852 | 1 | 96 | 105 |  |
| M.DSPMPIIPH.Q | Y | 19.82 | 1005.4954 | -1.1 | 503.7544 | 2 | 12.29 | 1806 | 1 | 97 | 105 |  |
| total 2 peptides |  |  |  |  |  |  |  |  |  |  |  |  |
| --- | --- | --- | --- | --- | --- | --- | --- | --- | --- | --- | --- | --- |

tr|A0A3Q3IQV7|A0A3Q3IQV7\_MONAL

back to list

  

| Protein Coverage
| Supporting Peptides
|

Protein Coverage:

Supporting Peptides:

| Peptide | Uniq | -10lgP | Mass | ppm | m/z | z | RT | Scan | #Spec | Start | End | PTM |
| --- | --- | --- | --- | --- | --- | --- | --- | --- | --- | --- | --- | --- |
| R.MDSPMPIIPH.Q | Y | 28.61 | 1136.5359 | -2.5 | 569.2738 | 2 | 12.55 | 1852 | 1 | 97 | 106 |  |
| M.DSPMPIIPH.Q | Y | 19.82 | 1005.4954 | -1.1 | 503.7544 | 2 | 12.29 | 1806 | 1 | 98 | 106 |  |
| total 2 peptides |  |  |  |  |  |  |  |  |  |  |  |  |
| --- | --- | --- | --- | --- | --- | --- | --- | --- | --- | --- | --- | --- |

tr|A0A665WTS5|A0A665WTS5\_ECHNA

back to list

  

| Protein Coverage
| Supporting Peptides
|

Protein Coverage:

Supporting Peptides:

| Peptide | Uniq | -10lgP | Mass | ppm | m/z | z | RT | Scan | #Spec | Start | End | PTM |
| --- | --- | --- | --- | --- | --- | --- | --- | --- | --- | --- | --- | --- |
| R.MDSPMPIIPH.Q | Y | 28.61 | 1136.5359 | -2.5 | 569.2738 | 2 | 12.55 | 1852 | 1 | 97 | 106 |  |
| M.DSPMPIIPH.Q | Y | 19.82 | 1005.4954 | -1.1 | 503.7544 | 2 | 12.29 | 1806 | 1 | 98 | 106 |  |
| total 2 peptides |  |  |  |  |  |  |  |  |  |  |  |  |
| --- | --- | --- | --- | --- | --- | --- | --- | --- | --- | --- | --- | --- |

tr|A0A7N8Y585|A0A7N8Y585\_9TELE

back to list

  

| Protein Coverage
| Supporting Peptides
|

Protein Coverage:

Supporting Peptides:

| Peptide | Uniq | -10lgP | Mass | ppm | m/z | z | RT | Scan | #Spec | Start | End | PTM |
| --- | --- | --- | --- | --- | --- | --- | --- | --- | --- | --- | --- | --- |
| R.MDSPMPIIPH.Q | Y | 28.61 | 1136.5359 | -2.5 | 569.2738 | 2 | 12.55 | 1852 | 1 | 100 | 109 |  |
| M.DSPMPIIPH.Q | Y | 19.82 | 1005.4954 | -1.1 | 503.7544 | 2 | 12.29 | 1806 | 1 | 101 | 109 |  |
| total 2 peptides |  |  |  |  |  |  |  |  |  |  |  |  |
| --- | --- | --- | --- | --- | --- | --- | --- | --- | --- | --- | --- | --- |

tr|A0A6P6QFT2|A0A6P6QFT2\_CARAU

back to list

  

| Protein Coverage
| Supporting Peptides
|

Protein Coverage:

Supporting Peptides:

| Peptide | Uniq | -10lgP | Mass | ppm | m/z | z | RT | Scan | #Spec | Start | End | PTM |
| --- | --- | --- | --- | --- | --- | --- | --- | --- | --- | --- | --- | --- |
| R.MDSPMPIIPH.Q | Y | 28.61 | 1136.5359 | -2.5 | 569.2738 | 2 | 12.55 | 1852 | 1 | 97 | 106 |  |
| M.DSPMPIIPH.Q | Y | 19.82 | 1005.4954 | -1.1 | 503.7544 | 2 | 12.29 | 1806 | 1 | 98 | 106 |  |
| total 2 peptides |  |  |  |  |  |  |  |  |  |  |  |  |
| --- | --- | --- | --- | --- | --- | --- | --- | --- | --- | --- | --- | --- |

tr|A0A672KNU9|A0A672KNU9\_SINGR

back to list

  

| Protein Coverage
| Supporting Peptides
|

Protein Coverage:

Supporting Peptides:

| Peptide | Uniq | -10lgP | Mass | ppm | m/z | z | RT | Scan | #Spec | Start | End | PTM |
| --- | --- | --- | --- | --- | --- | --- | --- | --- | --- | --- | --- | --- |
| R.MDSPMPIIPH.Q | Y | 28.61 | 1136.5359 | -2.5 | 569.2738 | 2 | 12.55 | 1852 | 1 | 97 | 106 |  |
| M.DSPMPIIPH.Q | Y | 19.82 | 1005.4954 | -1.1 | 503.7544 | 2 | 12.29 | 1806 | 1 | 98 | 106 |  |
| total 2 peptides |  |  |  |  |  |  |  |  |  |  |  |  |
| --- | --- | --- | --- | --- | --- | --- | --- | --- | --- | --- | --- | --- |

tr|A0A673M1Q3|A0A673M1Q3\_9TELE

back to list

  

| Protein Coverage
| Supporting Peptides
|

Protein Coverage:

Supporting Peptides:

| Peptide | Uniq | -10lgP | Mass | ppm | m/z | z | RT | Scan | #Spec | Start | End | PTM |
| --- | --- | --- | --- | --- | --- | --- | --- | --- | --- | --- | --- | --- |
| R.MDSPMPIIPH.Q | Y | 28.61 | 1136.5359 | -2.5 | 569.2738 | 2 | 12.55 | 1852 | 1 | 97 | 106 |  |
| M.DSPMPIIPH.Q | Y | 19.82 | 1005.4954 | -1.1 | 503.7544 | 2 | 12.29 | 1806 | 1 | 98 | 106 |  |
| total 2 peptides |  |  |  |  |  |  |  |  |  |  |  |  |
| --- | --- | --- | --- | --- | --- | --- | --- | --- | --- | --- | --- | --- |

tr|A0A7J6CGV6|A0A7J6CGV6\_9TELE

back to list

  

| Protein Coverage
| Supporting Peptides
|

Protein Coverage:

Supporting Peptides:

| Peptide | Uniq | -10lgP | Mass | ppm | m/z | z | RT | Scan | #Spec | Start | End | PTM |
| --- | --- | --- | --- | --- | --- | --- | --- | --- | --- | --- | --- | --- |
| R.MDSPMPIIPH.Q | Y | 28.61 | 1136.5359 | -2.5 | 569.2738 | 2 | 12.55 | 1852 | 1 | 97 | 106 |  |
| M.DSPMPIIPH.Q | Y | 19.82 | 1005.4954 | -1.1 | 503.7544 | 2 | 12.29 | 1806 | 1 | 98 | 106 |  |
| total 2 peptides |  |  |  |  |  |  |  |  |  |  |  |  |
| --- | --- | --- | --- | --- | --- | --- | --- | --- | --- | --- | --- | --- |

tr|A0A6P6QIW1|A0A6P6QIW1\_CARAU

back to list

  

| Protein Coverage
| Supporting Peptides
|

Protein Coverage:

Supporting Peptides:

| Peptide | Uniq | -10lgP | Mass | ppm | m/z | z | RT | Scan | #Spec | Start | End | PTM |
| --- | --- | --- | --- | --- | --- | --- | --- | --- | --- | --- | --- | --- |
| R.MDSPMPIIPH.Q | Y | 28.61 | 1136.5359 | -2.5 | 569.2738 | 2 | 12.55 | 1852 | 1 | 97 | 106 |  |
| M.DSPMPIIPH.Q | Y | 19.82 | 1005.4954 | -1.1 | 503.7544 | 2 | 12.29 | 1806 | 1 | 98 | 106 |  |
| total 2 peptides |  |  |  |  |  |  |  |  |  |  |  |  |
| --- | --- | --- | --- | --- | --- | --- | --- | --- | --- | --- | --- | --- |

tr|A0A6Q2XWF7|A0A6Q2XWF7\_ESOLU

back to list

  

| Protein Coverage
| Supporting Peptides
|

Protein Coverage:

Supporting Peptides:

| Peptide | Uniq | -10lgP | Mass | ppm | m/z | z | RT | Scan | #Spec | Start | End | PTM |
| --- | --- | --- | --- | --- | --- | --- | --- | --- | --- | --- | --- | --- |
| R.MDSPMPIIPH.Q | Y | 28.61 | 1136.5359 | -2.5 | 569.2738 | 2 | 12.55 | 1852 | 1 | 96 | 105 |  |
| M.DSPMPIIPH.Q | Y | 19.82 | 1005.4954 | -1.1 | 503.7544 | 2 | 12.29 | 1806 | 1 | 97 | 105 |  |
| total 2 peptides |  |  |  |  |  |  |  |  |  |  |  |  |
| --- | --- | --- | --- | --- | --- | --- | --- | --- | --- | --- | --- | --- |

tr|A0A0F8AMH6|A0A0F8AMH6\_LARCR

back to list

  

| Protein Coverage
| Supporting Peptides
|

Protein Coverage:

Supporting Peptides:

| Peptide | Uniq | -10lgP | Mass | ppm | m/z | z | RT | Scan | #Spec | Start | End | PTM |
| --- | --- | --- | --- | --- | --- | --- | --- | --- | --- | --- | --- | --- |
| R.MDSPMPIIPH.Q | Y | 28.61 | 1136.5359 | -2.5 | 569.2738 | 2 | 12.55 | 1852 | 1 | 97 | 106 |  |
| M.DSPMPIIPH.Q | Y | 19.82 | 1005.4954 | -1.1 | 503.7544 | 2 | 12.29 | 1806 | 1 | 98 | 106 |  |
| total 2 peptides |  |  |  |  |  |  |  |  |  |  |  |  |
| --- | --- | --- | --- | --- | --- | --- | --- | --- | --- | --- | --- | --- |

tr|A0A6G0HYU6|A0A6G0HYU6\_LARCR

back to list

  

| Protein Coverage
| Supporting Peptides
|

Protein Coverage:

Supporting Peptides:

| Peptide | Uniq | -10lgP | Mass | ppm | m/z | z | RT | Scan | #Spec | Start | End | PTM |
| --- | --- | --- | --- | --- | --- | --- | --- | --- | --- | --- | --- | --- |
| R.MDSPMPIIPH.Q | Y | 28.61 | 1136.5359 | -2.5 | 569.2738 | 2 | 12.55 | 1852 | 1 | 544 | 553 |  |
| M.DSPMPIIPH.Q | Y | 19.82 | 1005.4954 | -1.1 | 503.7544 | 2 | 12.29 | 1806 | 1 | 545 | 553 |  |
| total 2 peptides |  |  |  |  |  |  |  |  |  |  |  |  |
| --- | --- | --- | --- | --- | --- | --- | --- | --- | --- | --- | --- | --- |

tr|G3N515|G3N515\_GASAC

back to list

  

| Protein Coverage
| Supporting Peptides
|

Protein Coverage:

Supporting Peptides:

| Peptide | Uniq | -10lgP | Mass | ppm | m/z | z | RT | Scan | #Spec | Start | End | PTM |
| --- | --- | --- | --- | --- | --- | --- | --- | --- | --- | --- | --- | --- |
| N.LDVIDVPAPVKNL.R | Y | 24.20 | 1391.8024 | -0.5 | 696.9081 | 2 | 13.83 | 2080 | 1 | 1994 | 2006 |  |
| E.TDPVLIKDPIDPPWAPGKPVIR.D | N | 23.05 | 2423.3577 | -1.0 | 808.7924 | 3 | 12.93 | 1921 | 2 | 2882 | 2903 |  |
| total 2 peptides |  |  |  |  |  |  |  |  |  |  |  |  |
| --- | --- | --- | --- | --- | --- | --- | --- | --- | --- | --- | --- | --- |

tr|A0A3P8T7F6|A0A3P8T7F6\_AMPPE

back to list

  

| Protein Coverage
| Supporting Peptides
|

Protein Coverage:

Supporting Peptides:

| Peptide | Uniq | -10lgP | Mass | ppm | m/z | z | RT | Scan | #Spec | Start | End | PTM |
| --- | --- | --- | --- | --- | --- | --- | --- | --- | --- | --- | --- | --- |
| A.KKAEPAPAPAPAAPEP.A | Y | 34.93 | 1540.8248 | -1.0 | 771.4189 | 2 | 9.56 | 1321 | 1 | 12 | 27 |  |
| total 1 peptides |  |  |  |  |  |  |  |  |  |  |  |  |
| --- | --- | --- | --- | --- | --- | --- | --- | --- | --- | --- | --- | --- |

tr|A0A3Q1AWA1|A0A3Q1AWA1\_AMPOC

back to list

  

| Protein Coverage
| Supporting Peptides
|

Protein Coverage:

Supporting Peptides:

| Peptide | Uniq | -10lgP | Mass | ppm | m/z | z | RT | Scan | #Spec | Start | End | PTM |
| --- | --- | --- | --- | --- | --- | --- | --- | --- | --- | --- | --- | --- |
| A.KKAEPAPAPAPAAPEP.A | Y | 34.93 | 1540.8248 | -1.0 | 771.4189 | 2 | 9.56 | 1321 | 1 | 12 | 27 |  |
| total 1 peptides |  |  |  |  |  |  |  |  |  |  |  |  |
| --- | --- | --- | --- | --- | --- | --- | --- | --- | --- | --- | --- | --- |

tr|A0A3P8T5J7|A0A3P8T5J7\_AMPPE

back to list

  

| Protein Coverage
| Supporting Peptides
|

Protein Coverage:

Supporting Peptides:

| Peptide | Uniq | -10lgP | Mass | ppm | m/z | z | RT | Scan | #Spec | Start | End | PTM |
| --- | --- | --- | --- | --- | --- | --- | --- | --- | --- | --- | --- | --- |
| A.KKAEPAPAPAPAAPEP.A | Y | 34.93 | 1540.8248 | -1.0 | 771.4189 | 2 | 9.56 | 1321 | 1 | 12 | 27 |  |
| total 1 peptides |  |  |  |  |  |  |  |  |  |  |  |  |
| --- | --- | --- | --- | --- | --- | --- | --- | --- | --- | --- | --- | --- |

tr|A0A3P8T7H6|A0A3P8T7H6\_AMPPE

back to list

  

| Protein Coverage
| Supporting Peptides
|

Protein Coverage:

Supporting Peptides:

| Peptide | Uniq | -10lgP | Mass | ppm | m/z | z | RT | Scan | #Spec | Start | End | PTM |
| --- | --- | --- | --- | --- | --- | --- | --- | --- | --- | --- | --- | --- |
| A.KKAEPAPAPAPAAPEP.A | Y | 34.93 | 1540.8248 | -1.0 | 771.4189 | 2 | 9.56 | 1321 | 1 | 12 | 27 |  |
| total 1 peptides |  |  |  |  |  |  |  |  |  |  |  |  |
| --- | --- | --- | --- | --- | --- | --- | --- | --- | --- | --- | --- | --- |

tr|A0A3Q1B6V7|A0A3Q1B6V7\_AMPOC

back to list

  

| Protein Coverage
| Supporting Peptides
|

Protein Coverage:

Supporting Peptides:

| Peptide | Uniq | -10lgP | Mass | ppm | m/z | z | RT | Scan | #Spec | Start | End | PTM |
| --- | --- | --- | --- | --- | --- | --- | --- | --- | --- | --- | --- | --- |
| A.KKAEPAPAPAPAAPEP.A | Y | 34.93 | 1540.8248 | -1.0 | 771.4189 | 2 | 9.56 | 1321 | 1 | 12 | 27 |  |
| total 1 peptides |  |  |  |  |  |  |  |  |  |  |  |  |
| --- | --- | --- | --- | --- | --- | --- | --- | --- | --- | --- | --- | --- |

tr|A0A6F9CEX9|A0A6F9CEX9\_9TELE

back to list

  

| Protein Coverage
| Supporting Peptides
|

Protein Coverage:

Supporting Peptides:

| Peptide | Uniq | -10lgP | Mass | ppm | m/z | z | RT | Scan | #Spec | Start | End | PTM |
| --- | --- | --- | --- | --- | --- | --- | --- | --- | --- | --- | --- | --- |
| A.LDPIDAPGKPDVISIT.R | Y | 34.39 | 1649.8876 | -1.2 | 825.9501 | 2 | 12.86 | 1909 | 1 | 8748 | 8763 |  |
| total 1 peptides |  |  |  |  |  |  |  |  |  |  |  |  |
| --- | --- | --- | --- | --- | --- | --- | --- | --- | --- | --- | --- | --- |

tr|A0A484CUS5|A0A484CUS5\_PERFV

back to list

  

| Protein Coverage
| Supporting Peptides
|

Protein Coverage:

Supporting Peptides:

| Peptide | Uniq | -10lgP | Mass | ppm | m/z | z | RT | Scan | #Spec | Start | End | PTM |
| --- | --- | --- | --- | --- | --- | --- | --- | --- | --- | --- | --- | --- |
| E.TDPVLIKDPIDPPWAPGKPVIR.D | N | 23.05 | 2423.3577 | -1.0 | 808.7924 | 3 | 12.93 | 1921 | 2 | 710 | 731 |  |
| T.VVDRPTPPVGPV.V | Y | 22.13 | 1231.6924 | -0.6 | 616.8531 | 2 | 11.28 | 1619 | 1 | 3654 | 3665 |  |
| total 2 peptides |  |  |  |  |  |  |  |  |  |  |  |  |
| --- | --- | --- | --- | --- | --- | --- | --- | --- | --- | --- | --- | --- |

tr|A0A834CMG6|A0A834CMG6\_ORYME

back to list

  

| Protein Coverage
| Supporting Peptides
|

Protein Coverage:

Supporting Peptides:

| Peptide | Uniq | -10lgP | Mass | ppm | m/z | z | RT | Scan | #Spec | Start | End | PTM |
| --- | --- | --- | --- | --- | --- | --- | --- | --- | --- | --- | --- | --- |
| K.TIIPPKLPIEIPQPK.T | Y | 32.89 | 1683.0334 | -1.7 | 842.5226 | 2 | 13.09 | 1951 | 2 | 1366 | 1380 |  |
| total 1 peptides |  |  |  |  |  |  |  |  |  |  |  |  |
| --- | --- | --- | --- | --- | --- | --- | --- | --- | --- | --- | --- | --- |

tr|A0A3B3BCL7|A0A3B3BCL7\_ORYME

back to list

  

| Protein Coverage
| Supporting Peptides
|

Protein Coverage:

Supporting Peptides:

| Peptide | Uniq | -10lgP | Mass | ppm | m/z | z | RT | Scan | #Spec | Start | End | PTM |
| --- | --- | --- | --- | --- | --- | --- | --- | --- | --- | --- | --- | --- |
| K.TIIPPKLPIEIPQPK.T | Y | 32.89 | 1683.0334 | -1.7 | 842.5226 | 2 | 13.09 | 1951 | 2 | 1366 | 1380 |  |
| total 1 peptides |  |  |  |  |  |  |  |  |  |  |  |  |
| --- | --- | --- | --- | --- | --- | --- | --- | --- | --- | --- | --- | --- |

tr|A0A3B3D3C5|A0A3B3D3C5\_ORYME

back to list

  

| Protein Coverage
| Supporting Peptides
|

Protein Coverage:

Supporting Peptides:

| Peptide | Uniq | -10lgP | Mass | ppm | m/z | z | RT | Scan | #Spec | Start | End | PTM |
| --- | --- | --- | --- | --- | --- | --- | --- | --- | --- | --- | --- | --- |
| K.TIIPPKLPIEIPQPK.T | Y | 32.89 | 1683.0334 | -1.7 | 842.5226 | 2 | 13.09 | 1951 | 2 | 1371 | 1385 |  |
| total 1 peptides |  |  |  |  |  |  |  |  |  |  |  |  |
| --- | --- | --- | --- | --- | --- | --- | --- | --- | --- | --- | --- | --- |

tr|A0A672ZI78|A0A672ZI78\_9TELE

back to list

  

| Protein Coverage
| Supporting Peptides
|

Protein Coverage:

Supporting Peptides:

| Peptide | Uniq | -10lgP | Mass | ppm | m/z | z | RT | Scan | #Spec | Start | End | PTM |
| --- | --- | --- | --- | --- | --- | --- | --- | --- | --- | --- | --- | --- |
| K.TIIPPKLPIEIPQPK.T | Y | 32.89 | 1683.0334 | -1.7 | 842.5226 | 2 | 13.09 | 1951 | 2 | 1101 | 1115 |  |
| total 1 peptides |  |  |  |  |  |  |  |  |  |  |  |  |
| --- | --- | --- | --- | --- | --- | --- | --- | --- | --- | --- | --- | --- |

tr|A0A669ELF6|A0A669ELF6\_ORENI

back to list

  

| Protein Coverage
| Supporting Peptides
|

Protein Coverage:

Supporting Peptides:

| Peptide | Uniq | -10lgP | Mass | ppm | m/z | z | RT | Scan | #Spec | Start | End | PTM |
| --- | --- | --- | --- | --- | --- | --- | --- | --- | --- | --- | --- | --- |
| K.TIIPPKLPIEIPQPK.T | Y | 32.89 | 1683.0334 | -1.7 | 842.5226 | 2 | 13.09 | 1951 | 2 | 1146 | 1160 |  |
| total 1 peptides |  |  |  |  |  |  |  |  |  |  |  |  |
| --- | --- | --- | --- | --- | --- | --- | --- | --- | --- | --- | --- | --- |

tr|A0A671Y3Y8|A0A671Y3Y8\_SPAAU

back to list

  

| Protein Coverage
| Supporting Peptides
|

Protein Coverage:

Supporting Peptides:

| Peptide | Uniq | -10lgP | Mass | ppm | m/z | z | RT | Scan | #Spec | Start | End | PTM |
| --- | --- | --- | --- | --- | --- | --- | --- | --- | --- | --- | --- | --- |
| K.TIIPPKLPIEIPQPK.T | Y | 32.89 | 1683.0334 | -1.7 | 842.5226 | 2 | 13.09 | 1951 | 2 | 1151 | 1165 |  |
| total 1 peptides |  |  |  |  |  |  |  |  |  |  |  |  |
| --- | --- | --- | --- | --- | --- | --- | --- | --- | --- | --- | --- | --- |

tr|A0A4U5U6X9|A0A4U5U6X9\_COLLU

back to list

  

| Protein Coverage
| Supporting Peptides
|

Protein Coverage:

Supporting Peptides:

| Peptide | Uniq | -10lgP | Mass | ppm | m/z | z | RT | Scan | #Spec | Start | End | PTM |
| --- | --- | --- | --- | --- | --- | --- | --- | --- | --- | --- | --- | --- |
| K.TIIPPKLPIEIPQPK.T | Y | 32.89 | 1683.0334 | -1.7 | 842.5226 | 2 | 13.09 | 1951 | 2 | 1190 | 1204 |  |
| total 1 peptides |  |  |  |  |  |  |  |  |  |  |  |  |
| --- | --- | --- | --- | --- | --- | --- | --- | --- | --- | --- | --- | --- |

tr|A0A669CKL2|A0A669CKL2\_ORENI

back to list

  

| Protein Coverage
| Supporting Peptides
|

Protein Coverage:

Supporting Peptides:

| Peptide | Uniq | -10lgP | Mass | ppm | m/z | z | RT | Scan | #Spec | Start | End | PTM |
| --- | --- | --- | --- | --- | --- | --- | --- | --- | --- | --- | --- | --- |
| K.TIIPPKLPIEIPQPK.T | Y | 32.89 | 1683.0334 | -1.7 | 842.5226 | 2 | 13.09 | 1951 | 2 | 1224 | 1238 |  |
| total 1 peptides |  |  |  |  |  |  |  |  |  |  |  |  |
| --- | --- | --- | --- | --- | --- | --- | --- | --- | --- | --- | --- | --- |

tr|A0A671Y401|A0A671Y401\_SPAAU

back to list

  

| Protein Coverage
| Supporting Peptides
|

Protein Coverage:

Supporting Peptides:

| Peptide | Uniq | -10lgP | Mass | ppm | m/z | z | RT | Scan | #Spec | Start | End | PTM |
| --- | --- | --- | --- | --- | --- | --- | --- | --- | --- | --- | --- | --- |
| K.TIIPPKLPIEIPQPK.T | Y | 32.89 | 1683.0334 | -1.7 | 842.5226 | 2 | 13.09 | 1951 | 2 | 1268 | 1282 |  |
| total 1 peptides |  |  |  |  |  |  |  |  |  |  |  |  |
| --- | --- | --- | --- | --- | --- | --- | --- | --- | --- | --- | --- | --- |

tr|A0A672FNF5|A0A672FNF5\_SALFA

back to list

  

| Protein Coverage
| Supporting Peptides
|

Protein Coverage:

Supporting Peptides:

| Peptide | Uniq | -10lgP | Mass | ppm | m/z | z | RT | Scan | #Spec | Start | End | PTM |
| --- | --- | --- | --- | --- | --- | --- | --- | --- | --- | --- | --- | --- |
| K.TIIPPKLPIEIPQPK.T | Y | 32.89 | 1683.0334 | -1.7 | 842.5226 | 2 | 13.09 | 1951 | 2 | 1294 | 1308 |  |
| total 1 peptides |  |  |  |  |  |  |  |  |  |  |  |  |
| --- | --- | --- | --- | --- | --- | --- | --- | --- | --- | --- | --- | --- |

tr|A0A671Y3W3|A0A671Y3W3\_SPAAU

back to list

  

| Protein Coverage
| Supporting Peptides
|

Protein Coverage:

Supporting Peptides:

| Peptide | Uniq | -10lgP | Mass | ppm | m/z | z | RT | Scan | #Spec | Start | End | PTM |
| --- | --- | --- | --- | --- | --- | --- | --- | --- | --- | --- | --- | --- |
| K.TIIPPKLPIEIPQPK.T | Y | 32.89 | 1683.0334 | -1.7 | 842.5226 | 2 | 13.09 | 1951 | 2 | 1334 | 1348 |  |
| total 1 peptides |  |  |  |  |  |  |  |  |  |  |  |  |
| --- | --- | --- | --- | --- | --- | --- | --- | --- | --- | --- | --- | --- |

tr|A0A6G0J0W8|A0A6G0J0W8\_LARCR

back to list

  

| Protein Coverage
| Supporting Peptides
|

Protein Coverage:

Supporting Peptides:

| Peptide | Uniq | -10lgP | Mass | ppm | m/z | z | RT | Scan | #Spec | Start | End | PTM |
| --- | --- | --- | --- | --- | --- | --- | --- | --- | --- | --- | --- | --- |
| K.TIIPPKLPIEIPQPK.T | Y | 32.89 | 1683.0334 | -1.7 | 842.5226 | 2 | 13.09 | 1951 | 2 | 1335 | 1349 |  |
| total 1 peptides |  |  |  |  |  |  |  |  |  |  |  |  |
| --- | --- | --- | --- | --- | --- | --- | --- | --- | --- | --- | --- | --- |

tr|A0A671Y575|A0A671Y575\_SPAAU

back to list

  

| Protein Coverage
| Supporting Peptides
|

Protein Coverage:

Supporting Peptides:

| Peptide | Uniq | -10lgP | Mass | ppm | m/z | z | RT | Scan | #Spec | Start | End | PTM |
| --- | --- | --- | --- | --- | --- | --- | --- | --- | --- | --- | --- | --- |
| K.TIIPPKLPIEIPQPK.T | Y | 32.89 | 1683.0334 | -1.7 | 842.5226 | 2 | 13.09 | 1951 | 2 | 1349 | 1363 |  |
| total 1 peptides |  |  |  |  |  |  |  |  |  |  |  |  |
| --- | --- | --- | --- | --- | --- | --- | --- | --- | --- | --- | --- | --- |

tr|A0A6G1P7U8|A0A6G1P7U8\_9TELE

back to list

  

| Protein Coverage
| Supporting Peptides
|

Protein Coverage:

Supporting Peptides:

| Peptide | Uniq | -10lgP | Mass | ppm | m/z | z | RT | Scan | #Spec | Start | End | PTM |
| --- | --- | --- | --- | --- | --- | --- | --- | --- | --- | --- | --- | --- |
| K.TIIPPKLPIEIPQPK.T | Y | 32.89 | 1683.0334 | -1.7 | 842.5226 | 2 | 13.09 | 1951 | 2 | 1366 | 1380 |  |
| total 1 peptides |  |  |  |  |  |  |  |  |  |  |  |  |
| --- | --- | --- | --- | --- | --- | --- | --- | --- | --- | --- | --- | --- |

tr|A0A087YFH0|A0A087YFH0\_POEFO

back to list

  

| Protein Coverage
| Supporting Peptides
|

Protein Coverage:

Supporting Peptides:

| Peptide | Uniq | -10lgP | Mass | ppm | m/z | z | RT | Scan | #Spec | Start | End | PTM |
| --- | --- | --- | --- | --- | --- | --- | --- | --- | --- | --- | --- | --- |
| K.TIIPPKLPIEIPQPK.T | Y | 32.89 | 1683.0334 | -1.7 | 842.5226 | 2 | 13.09 | 1951 | 2 | 1365 | 1379 |  |
| total 1 peptides |  |  |  |  |  |  |  |  |  |  |  |  |
| --- | --- | --- | --- | --- | --- | --- | --- | --- | --- | --- | --- | --- |

tr|A0A3P9PGW1|A0A3P9PGW1\_POERE

back to list

  

| Protein Coverage
| Supporting Peptides
|

Protein Coverage:

Supporting Peptides:

| Peptide | Uniq | -10lgP | Mass | ppm | m/z | z | RT | Scan | #Spec | Start | End | PTM |
| --- | --- | --- | --- | --- | --- | --- | --- | --- | --- | --- | --- | --- |
| K.TIIPPKLPIEIPQPK.T | Y | 32.89 | 1683.0334 | -1.7 | 842.5226 | 2 | 13.09 | 1951 | 2 | 1365 | 1379 |  |
| total 1 peptides |  |  |  |  |  |  |  |  |  |  |  |  |
| --- | --- | --- | --- | --- | --- | --- | --- | --- | --- | --- | --- | --- |

tr|A0A3P8P4C2|A0A3P8P4C2\_ASTCA

back to list

  

| Protein Coverage
| Supporting Peptides
|

Protein Coverage:

Supporting Peptides:

| Peptide | Uniq | -10lgP | Mass | ppm | m/z | z | RT | Scan | #Spec | Start | End | PTM |
| --- | --- | --- | --- | --- | --- | --- | --- | --- | --- | --- | --- | --- |
| K.TIIPPKLPIEIPQPK.T | Y | 32.89 | 1683.0334 | -1.7 | 842.5226 | 2 | 13.09 | 1951 | 2 | 1366 | 1380 |  |
| total 1 peptides |  |  |  |  |  |  |  |  |  |  |  |  |
| --- | --- | --- | --- | --- | --- | --- | --- | --- | --- | --- | --- | --- |

tr|A0A3Q1D109|A0A3Q1D109\_AMPOC

back to list

  

| Protein Coverage
| Supporting Peptides
|

Protein Coverage:

Supporting Peptides:

| Peptide | Uniq | -10lgP | Mass | ppm | m/z | z | RT | Scan | #Spec | Start | End | PTM |
| --- | --- | --- | --- | --- | --- | --- | --- | --- | --- | --- | --- | --- |
| K.TIIPPKLPIEIPQPK.T | Y | 32.89 | 1683.0334 | -1.7 | 842.5226 | 2 | 13.09 | 1951 | 2 | 1366 | 1380 |  |
| total 1 peptides |  |  |  |  |  |  |  |  |  |  |  |  |
| --- | --- | --- | --- | --- | --- | --- | --- | --- | --- | --- | --- | --- |

tr|A0A3Q2WWP1|A0A3Q2WWP1\_HAPBU

back to list

  

| Protein Coverage
| Supporting Peptides
|

Protein Coverage:

Supporting Peptides:

| Peptide | Uniq | -10lgP | Mass | ppm | m/z | z | RT | Scan | #Spec | Start | End | PTM |
| --- | --- | --- | --- | --- | --- | --- | --- | --- | --- | --- | --- | --- |
| K.TIIPPKLPIEIPQPK.T | Y | 32.89 | 1683.0334 | -1.7 | 842.5226 | 2 | 13.09 | 1951 | 2 | 1366 | 1380 |  |
| total 1 peptides |  |  |  |  |  |  |  |  |  |  |  |  |
| --- | --- | --- | --- | --- | --- | --- | --- | --- | --- | --- | --- | --- |

tr|A0A672ZHV0|A0A672ZHV0\_9TELE

back to list

  

| Protein Coverage
| Supporting Peptides
|

Protein Coverage:

Supporting Peptides:

| Peptide | Uniq | -10lgP | Mass | ppm | m/z | z | RT | Scan | #Spec | Start | End | PTM |
| --- | --- | --- | --- | --- | --- | --- | --- | --- | --- | --- | --- | --- |
| K.TIIPPKLPIEIPQPK.T | Y | 32.89 | 1683.0334 | -1.7 | 842.5226 | 2 | 13.09 | 1951 | 2 | 1366 | 1380 |  |
| total 1 peptides |  |  |  |  |  |  |  |  |  |  |  |  |
| --- | --- | --- | --- | --- | --- | --- | --- | --- | --- | --- | --- | --- |

tr|I3JDJ1|I3JDJ1\_ORENI

back to list

  

| Protein Coverage
| Supporting Peptides
|

Protein Coverage:

Supporting Peptides:

| Peptide | Uniq | -10lgP | Mass | ppm | m/z | z | RT | Scan | #Spec | Start | End | PTM |
| --- | --- | --- | --- | --- | --- | --- | --- | --- | --- | --- | --- | --- |
| K.TIIPPKLPIEIPQPK.T | Y | 32.89 | 1683.0334 | -1.7 | 842.5226 | 2 | 13.09 | 1951 | 2 | 1366 | 1380 |  |
| total 1 peptides |  |  |  |  |  |  |  |  |  |  |  |  |
| --- | --- | --- | --- | --- | --- | --- | --- | --- | --- | --- | --- | --- |

tr|A0A2I4AP94|A0A2I4AP94\_9TELE

back to list

  

| Protein Coverage
| Supporting Peptides
|

Protein Coverage:

Supporting Peptides:

| Peptide | Uniq | -10lgP | Mass | ppm | m/z | z | RT | Scan | #Spec | Start | End | PTM |
| --- | --- | --- | --- | --- | --- | --- | --- | --- | --- | --- | --- | --- |
| K.TIIPPKLPIEIPQPK.T | Y | 32.89 | 1683.0334 | -1.7 | 842.5226 | 2 | 13.09 | 1951 | 2 | 1366 | 1380 |  |
| total 1 peptides |  |  |  |  |  |  |  |  |  |  |  |  |
| --- | --- | --- | --- | --- | --- | --- | --- | --- | --- | --- | --- | --- |

tr|A0A3P9LTE0|A0A3P9LTE0\_ORYLA

back to list

  

| Protein Coverage
| Supporting Peptides
|

Protein Coverage:

Supporting Peptides:

| Peptide | Uniq | -10lgP | Mass | ppm | m/z | z | RT | Scan | #Spec | Start | End | PTM |
| --- | --- | --- | --- | --- | --- | --- | --- | --- | --- | --- | --- | --- |
| K.TIIPPKLPIEIPQPK.T | Y | 32.89 | 1683.0334 | -1.7 | 842.5226 | 2 | 13.09 | 1951 | 2 | 1366 | 1380 |  |
| total 1 peptides |  |  |  |  |  |  |  |  |  |  |  |  |
| --- | --- | --- | --- | --- | --- | --- | --- | --- | --- | --- | --- | --- |

tr|A0A6P7LYN0|A0A6P7LYN0\_BETSP

back to list

  

| Protein Coverage
| Supporting Peptides
|

Protein Coverage:

Supporting Peptides:

| Peptide | Uniq | -10lgP | Mass | ppm | m/z | z | RT | Scan | #Spec | Start | End | PTM |
| --- | --- | --- | --- | --- | --- | --- | --- | --- | --- | --- | --- | --- |
| K.TIIPPKLPIELPQPK.T | Y | 32.89 | 1683.0334 | -1.7 | 842.5226 | 2 | 13.09 | 1951 | 2 | 1382 | 1396 |  |
| total 1 peptides |  |  |  |  |  |  |  |  |  |  |  |  |
| --- | --- | --- | --- | --- | --- | --- | --- | --- | --- | --- | --- | --- |

tr|A0A3Q4GHK3|A0A3Q4GHK3\_NEOBR

back to list

  

| Protein Coverage
| Supporting Peptides
|

Protein Coverage:

Supporting Peptides:

| Peptide | Uniq | -10lgP | Mass | ppm | m/z | z | RT | Scan | #Spec | Start | End | PTM |
| --- | --- | --- | --- | --- | --- | --- | --- | --- | --- | --- | --- | --- |
| K.TIIPPKLPIEIPQPK.P | Y | 32.89 | 1683.0334 | -1.7 | 842.5226 | 2 | 13.09 | 1951 | 2 | 858 | 872 |  |
| total 1 peptides |  |  |  |  |  |  |  |  |  |  |  |  |
| --- | --- | --- | --- | --- | --- | --- | --- | --- | --- | --- | --- | --- |

tr|A0A3Q4GXU7|A0A3Q4GXU7\_NEOBR

back to list

  

| Protein Coverage
| Supporting Peptides
|

Protein Coverage:

Supporting Peptides:

| Peptide | Uniq | -10lgP | Mass | ppm | m/z | z | RT | Scan | #Spec | Start | End | PTM |
| --- | --- | --- | --- | --- | --- | --- | --- | --- | --- | --- | --- | --- |
| K.TIIPPKLPIEIPQPK.P | Y | 32.89 | 1683.0334 | -1.7 | 842.5226 | 2 | 13.09 | 1951 | 2 | 1184 | 1198 |  |
| total 1 peptides |  |  |  |  |  |  |  |  |  |  |  |  |
| --- | --- | --- | --- | --- | --- | --- | --- | --- | --- | --- | --- | --- |

tr|A0A3B3IJA0|A0A3B3IJA0\_ORYLA

back to list

  

| Protein Coverage
| Supporting Peptides
|

Protein Coverage:

Supporting Peptides:

| Peptide | Uniq | -10lgP | Mass | ppm | m/z | z | RT | Scan | #Spec | Start | End | PTM |
| --- | --- | --- | --- | --- | --- | --- | --- | --- | --- | --- | --- | --- |
| K.TIIPPKLPIEIPQPK.T | Y | 32.89 | 1683.0334 | -1.7 | 842.5226 | 2 | 13.09 | 1951 | 2 | 1351 | 1365 |  |
| total 1 peptides |  |  |  |  |  |  |  |  |  |  |  |  |
| --- | --- | --- | --- | --- | --- | --- | --- | --- | --- | --- | --- | --- |

tr|A0A3P9IT90|A0A3P9IT90\_ORYLA

back to list

  

| Protein Coverage
| Supporting Peptides
|

Protein Coverage:

Supporting Peptides:

| Peptide | Uniq | -10lgP | Mass | ppm | m/z | z | RT | Scan | #Spec | Start | End | PTM |
| --- | --- | --- | --- | --- | --- | --- | --- | --- | --- | --- | --- | --- |
| K.TIIPPKLPIEIPQPK.T | Y | 32.89 | 1683.0334 | -1.7 | 842.5226 | 2 | 13.09 | 1951 | 2 | 1366 | 1380 |  |
| total 1 peptides |  |  |  |  |  |  |  |  |  |  |  |  |
| --- | --- | --- | --- | --- | --- | --- | --- | --- | --- | --- | --- | --- |

tr|H2M2B3|H2M2B3\_ORYLA

back to list

  

| Protein Coverage
| Supporting Peptides
|

Protein Coverage:

Supporting Peptides:

| Peptide | Uniq | -10lgP | Mass | ppm | m/z | z | RT | Scan | #Spec | Start | End | PTM |
| --- | --- | --- | --- | --- | --- | --- | --- | --- | --- | --- | --- | --- |
| K.TIIPPKLPIEIPQPK.T | Y | 32.89 | 1683.0334 | -1.7 | 842.5226 | 2 | 13.09 | 1951 | 2 | 1366 | 1380 |  |
| total 1 peptides |  |  |  |  |  |  |  |  |  |  |  |  |
| --- | --- | --- | --- | --- | --- | --- | --- | --- | --- | --- | --- | --- |

tr|A0A3B3HWS2|A0A3B3HWS2\_ORYLA

back to list

  

| Protein Coverage
| Supporting Peptides
|

Protein Coverage:

Supporting Peptides:

| Peptide | Uniq | -10lgP | Mass | ppm | m/z | z | RT | Scan | #Spec | Start | End | PTM |
| --- | --- | --- | --- | --- | --- | --- | --- | --- | --- | --- | --- | --- |
| K.TIIPPKLPIEIPQPK.T | Y | 32.89 | 1683.0334 | -1.7 | 842.5226 | 2 | 13.09 | 1951 | 2 | 1378 | 1392 |  |
| total 1 peptides |  |  |  |  |  |  |  |  |  |  |  |  |
| --- | --- | --- | --- | --- | --- | --- | --- | --- | --- | --- | --- | --- |

tr|A0A6G0J5U6|A0A6G0J5U6\_LARCR

back to list

  

| Protein Coverage
| Supporting Peptides
|

Protein Coverage:

Supporting Peptides:

| Peptide | Uniq | -10lgP | Mass | ppm | m/z | z | RT | Scan | #Spec | Start | End | PTM |
| --- | --- | --- | --- | --- | --- | --- | --- | --- | --- | --- | --- | --- |
| E.IVRPPQDMFEPPGSDIVF.E | Y | 21.70 | 2043.0134 | 2.4 | 1022.5165 | 2 | 14.33 | 2166 | 1 | 119 | 136 |  |
| E.IVRPPQDMFEPPGSDIVFE.V | Y | 20.48 | 2172.0562 | -0.7 | 1087.0345 | 2 | 13.99 | 2108 | 1 | 119 | 137 |  |
| total 2 peptides |  |  |  |  |  |  |  |  |  |  |  |  |
| --- | --- | --- | --- | --- | --- | --- | --- | --- | --- | --- | --- | --- |

tr|A0A6A5F2V4|A0A6A5F2V4\_PERFL

back to list

  

| Protein Coverage
| Supporting Peptides
|

Protein Coverage:

Supporting Peptides:

| Peptide | Uniq | -10lgP | Mass | ppm | m/z | z | RT | Scan | #Spec | Start | End | PTM |
| --- | --- | --- | --- | --- | --- | --- | --- | --- | --- | --- | --- | --- |
| E.TDPVLIKDPIDPPWAPGKPVIR.D | N | 23.05 | 2423.3577 | -1.0 | 808.7924 | 3 | 12.93 | 1921 | 2 | 6913 | 6934 |  |
| K.AIGNPLPDIVWL.K | Y | 17.35 | 1306.7285 | 0.2 | 654.3716 | 2 | 16.02 | 2459 | 1 | 622 | 633 |  |
| total 2 peptides |  |  |  |  |  |  |  |  |  |  |  |  |
| --- | --- | --- | --- | --- | --- | --- | --- | --- | --- | --- | --- | --- |

tr|A0A7N6BJ85|A0A7N6BJ85\_ANATE

back to list

  

| Protein Coverage
| Supporting Peptides
|

Protein Coverage:

Supporting Peptides:

| Peptide | Uniq | -10lgP | Mass | ppm | m/z | z | RT | Scan | #Spec | Start | End | PTM |
| --- | --- | --- | --- | --- | --- | --- | --- | --- | --- | --- | --- | --- |
| A.LDPVDPPGQPVPIF.V | Y | 29.58 | 1489.7816 | -0.1 | 745.8980 | 2 | 14.35 | 2169 | 1 | 17 | 30 |  |
| total 1 peptides |  |  |  |  |  |  |  |  |  |  |  |  |
| --- | --- | --- | --- | --- | --- | --- | --- | --- | --- | --- | --- | --- |

tr|A0A3B3QB10|A0A3B3QB10\_9TELE

back to list

  

| Protein Coverage
| Supporting Peptides
|

Protein Coverage:

Supporting Peptides:

| Peptide | Uniq | -10lgP | Mass | ppm | m/z | z | RT | Scan | #Spec | Start | End | PTM |
| --- | --- | --- | --- | --- | --- | --- | --- | --- | --- | --- | --- | --- |
| L.DRPGPPEGPISIY.G | Y | 28.56 | 1396.6986 | -1.1 | 699.3558 | 2 | 12.64 | 1868 | 1 | 760 | 772 |  |
| total 1 peptides |  |  |  |  |  |  |  |  |  |  |  |  |
| --- | --- | --- | --- | --- | --- | --- | --- | --- | --- | --- | --- | --- |

tr|A0A668VQH5|A0A668VQH5\_OREAU

back to list

  

| Protein Coverage
| Supporting Peptides
|

Protein Coverage:

Supporting Peptides:

| Peptide | Uniq | -10lgP | Mass | ppm | m/z | z | RT | Scan | #Spec | Start | End | PTM |
| --- | --- | --- | --- | --- | --- | --- | --- | --- | --- | --- | --- | --- |
| S.IEDPFDQDDWE.A | Y | 26.40 | 1407.5466 | -0.3 | 704.7804 | 2 | 14.04 | 2116 | 1 | 165 | 175 |  |
| total 1 peptides |  |  |  |  |  |  |  |  |  |  |  |  |
| --- | --- | --- | --- | --- | --- | --- | --- | --- | --- | --- | --- | --- |

tr|A0A3P8Q0W3|A0A3P8Q0W3\_ASTCA

back to list

  

| Protein Coverage
| Supporting Peptides
|

Protein Coverage:

Supporting Peptides:

| Peptide | Uniq | -10lgP | Mass | ppm | m/z | z | RT | Scan | #Spec | Start | End | PTM |
| --- | --- | --- | --- | --- | --- | --- | --- | --- | --- | --- | --- | --- |
| S.IEDPFDQDDWE.A | Y | 26.40 | 1407.5466 | -0.3 | 704.7804 | 2 | 14.04 | 2116 | 1 | 249 | 259 |  |
| total 1 peptides |  |  |  |  |  |  |  |  |  |  |  |  |
| --- | --- | --- | --- | --- | --- | --- | --- | --- | --- | --- | --- | --- |

tr|F5BZS7|F5BZS7\_EPIBR

back to list

  

| Protein Coverage
| Supporting Peptides
|

Protein Coverage:

Supporting Peptides:

| Peptide | Uniq | -10lgP | Mass | ppm | m/z | z | RT | Scan | #Spec | Start | End | PTM |
| --- | --- | --- | --- | --- | --- | --- | --- | --- | --- | --- | --- | --- |
| S.IEDPFDQDDWE.N | Y | 26.40 | 1407.5466 | -0.3 | 704.7804 | 2 | 14.04 | 2116 | 1 | 292 | 302 |  |
| total 1 peptides |  |  |  |  |  |  |  |  |  |  |  |  |
| --- | --- | --- | --- | --- | --- | --- | --- | --- | --- | --- | --- | --- |

tr|A0A4W4G2T4|A0A4W4G2T4\_ELEEL

back to list

  

| Protein Coverage
| Supporting Peptides
|

Protein Coverage:

Supporting Peptides:

| Peptide | Uniq | -10lgP | Mass | ppm | m/z | z | RT | Scan | #Spec | Start | End | PTM |
| --- | --- | --- | --- | --- | --- | --- | --- | --- | --- | --- | --- | --- |
| S.IEDPFDQDDWE.A | Y | 26.40 | 1407.5466 | -0.3 | 704.7804 | 2 | 14.04 | 2116 | 1 | 265 | 275 |  |
| total 1 peptides |  |  |  |  |  |  |  |  |  |  |  |  |
| --- | --- | --- | --- | --- | --- | --- | --- | --- | --- | --- | --- | --- |

tr|A0A4W4G3J1|A0A4W4G3J1\_ELEEL

back to list

  

| Protein Coverage
| Supporting Peptides
|

Protein Coverage:

Supporting Peptides:

| Peptide | Uniq | -10lgP | Mass | ppm | m/z | z | RT | Scan | #Spec | Start | End | PTM |
| --- | --- | --- | --- | --- | --- | --- | --- | --- | --- | --- | --- | --- |
| S.IEDPFDQDDWE.A | Y | 26.40 | 1407.5466 | -0.3 | 704.7804 | 2 | 14.04 | 2116 | 1 | 267 | 277 |  |
| total 1 peptides |  |  |  |  |  |  |  |  |  |  |  |  |
| --- | --- | --- | --- | --- | --- | --- | --- | --- | --- | --- | --- | --- |

tr|A0A668VQX9|A0A668VQX9\_OREAU

back to list

  

| Protein Coverage
| Supporting Peptides
|

Protein Coverage:

Supporting Peptides:

| Peptide | Uniq | -10lgP | Mass | ppm | m/z | z | RT | Scan | #Spec | Start | End | PTM |
| --- | --- | --- | --- | --- | --- | --- | --- | --- | --- | --- | --- | --- |
| S.IEDPFDQDDWE.A | Y | 26.40 | 1407.5466 | -0.3 | 704.7804 | 2 | 14.04 | 2116 | 1 | 292 | 302 |  |
| total 1 peptides |  |  |  |  |  |  |  |  |  |  |  |  |
| --- | --- | --- | --- | --- | --- | --- | --- | --- | --- | --- | --- | --- |

tr|A0A4W4G4V3|A0A4W4G4V3\_ELEEL

back to list

  

| Protein Coverage
| Supporting Peptides
|

Protein Coverage:

Supporting Peptides:

| Peptide | Uniq | -10lgP | Mass | ppm | m/z | z | RT | Scan | #Spec | Start | End | PTM |
| --- | --- | --- | --- | --- | --- | --- | --- | --- | --- | --- | --- | --- |
| S.IEDPFDQDDWE.N | Y | 26.40 | 1407.5466 | -0.3 | 704.7804 | 2 | 14.04 | 2116 | 1 | 292 | 302 |  |
| total 1 peptides |  |  |  |  |  |  |  |  |  |  |  |  |
| --- | --- | --- | --- | --- | --- | --- | --- | --- | --- | --- | --- | --- |

tr|A0A668VU75|A0A668VU75\_OREAU

back to list

  

| Protein Coverage
| Supporting Peptides
|

Protein Coverage:

Supporting Peptides:

| Peptide | Uniq | -10lgP | Mass | ppm | m/z | z | RT | Scan | #Spec | Start | End | PTM |
| --- | --- | --- | --- | --- | --- | --- | --- | --- | --- | --- | --- | --- |
| S.IEDPFDQDDWE.A | Y | 26.40 | 1407.5466 | -0.3 | 704.7804 | 2 | 14.04 | 2116 | 1 | 281 | 291 |  |
| total 1 peptides |  |  |  |  |  |  |  |  |  |  |  |  |
| --- | --- | --- | --- | --- | --- | --- | --- | --- | --- | --- | --- | --- |

tr|A0A4W4G338|A0A4W4G338\_ELEEL

back to list

  

| Protein Coverage
| Supporting Peptides
|

Protein Coverage:

Supporting Peptides:

| Peptide | Uniq | -10lgP | Mass | ppm | m/z | z | RT | Scan | #Spec | Start | End | PTM |
| --- | --- | --- | --- | --- | --- | --- | --- | --- | --- | --- | --- | --- |
| S.IEDPFDQDDWE.A | Y | 26.40 | 1407.5466 | -0.3 | 704.7804 | 2 | 14.04 | 2116 | 1 | 282 | 292 |  |
| total 1 peptides |  |  |  |  |  |  |  |  |  |  |  |  |
| --- | --- | --- | --- | --- | --- | --- | --- | --- | --- | --- | --- | --- |

tr|A0A3P9C6I0|A0A3P9C6I0\_9CICH

back to list

  

| Protein Coverage
| Supporting Peptides
|

Protein Coverage:

Supporting Peptides:

| Peptide | Uniq | -10lgP | Mass | ppm | m/z | z | RT | Scan | #Spec | Start | End | PTM |
| --- | --- | --- | --- | --- | --- | --- | --- | --- | --- | --- | --- | --- |
| S.IEDPFDQDDWE.A | Y | 26.40 | 1407.5466 | -0.3 | 704.7804 | 2 | 14.04 | 2116 | 1 | 283 | 293 |  |
| total 1 peptides |  |  |  |  |  |  |  |  |  |  |  |  |
| --- | --- | --- | --- | --- | --- | --- | --- | --- | --- | --- | --- | --- |

tr|A0A668V8T6|A0A668V8T6\_OREAU

back to list

  

| Protein Coverage
| Supporting Peptides
|

Protein Coverage:

Supporting Peptides:

| Peptide | Uniq | -10lgP | Mass | ppm | m/z | z | RT | Scan | #Spec | Start | End | PTM |
| --- | --- | --- | --- | --- | --- | --- | --- | --- | --- | --- | --- | --- |
| S.IEDPFDQDDWE.H | Y | 26.40 | 1407.5466 | -0.3 | 704.7804 | 2 | 14.04 | 2116 | 1 | 292 | 302 |  |
| total 1 peptides |  |  |  |  |  |  |  |  |  |  |  |  |
| --- | --- | --- | --- | --- | --- | --- | --- | --- | --- | --- | --- | --- |

tr|A0A668VTY0|A0A668VTY0\_OREAU

back to list

  

| Protein Coverage
| Supporting Peptides
|

Protein Coverage:

Supporting Peptides:

| Peptide | Uniq | -10lgP | Mass | ppm | m/z | z | RT | Scan | #Spec | Start | End | PTM |
| --- | --- | --- | --- | --- | --- | --- | --- | --- | --- | --- | --- | --- |
| S.IEDPFDQDDWE.A | Y | 26.40 | 1407.5466 | -0.3 | 704.7804 | 2 | 14.04 | 2116 | 1 | 292 | 302 |  |
| total 1 peptides |  |  |  |  |  |  |  |  |  |  |  |  |
| --- | --- | --- | --- | --- | --- | --- | --- | --- | --- | --- | --- | --- |

tr|A0A6P3VUG4|A0A6P3VUG4\_CLUHA

back to list

  

| Protein Coverage
| Supporting Peptides
|

Protein Coverage:

Supporting Peptides:

| Peptide | Uniq | -10lgP | Mass | ppm | m/z | z | RT | Scan | #Spec | Start | End | PTM |
| --- | --- | --- | --- | --- | --- | --- | --- | --- | --- | --- | --- | --- |
| S.IEDPFDQDDWE.A | Y | 26.40 | 1407.5466 | -0.3 | 704.7804 | 2 | 14.04 | 2116 | 1 | 292 | 302 |  |
| total 1 peptides |  |  |  |  |  |  |  |  |  |  |  |  |
| --- | --- | --- | --- | --- | --- | --- | --- | --- | --- | --- | --- | --- |

tr|A0A4W4FGS3|A0A4W4FGS3\_ELEEL

back to list

  

| Protein Coverage
| Supporting Peptides
|

Protein Coverage:

Supporting Peptides:

| Peptide | Uniq | -10lgP | Mass | ppm | m/z | z | RT | Scan | #Spec | Start | End | PTM |
| --- | --- | --- | --- | --- | --- | --- | --- | --- | --- | --- | --- | --- |
| S.IEDPFDQDDWE.N | Y | 26.40 | 1407.5466 | -0.3 | 704.7804 | 2 | 14.04 | 2116 | 1 | 290 | 300 |  |
| total 1 peptides |  |  |  |  |  |  |  |  |  |  |  |  |
| --- | --- | --- | --- | --- | --- | --- | --- | --- | --- | --- | --- | --- |

tr|A0A3P8TV75|A0A3P8TV75\_AMPPE

back to list

  

| Protein Coverage
| Supporting Peptides
|

Protein Coverage:

Supporting Peptides:

| Peptide | Uniq | -10lgP | Mass | ppm | m/z | z | RT | Scan | #Spec | Start | End | PTM |
| --- | --- | --- | --- | --- | --- | --- | --- | --- | --- | --- | --- | --- |
| S.IEDPFDQDDWE.A | Y | 26.40 | 1407.5466 | -0.3 | 704.7804 | 2 | 14.04 | 2116 | 1 | 292 | 302 |  |
| total 1 peptides |  |  |  |  |  |  |  |  |  |  |  |  |
| --- | --- | --- | --- | --- | --- | --- | --- | --- | --- | --- | --- | --- |

tr|A0A6P7IPP6|A0A6P7IPP6\_9TELE

back to list

  

| Protein Coverage
| Supporting Peptides
|

Protein Coverage:

Supporting Peptides:

| Peptide | Uniq | -10lgP | Mass | ppm | m/z | z | RT | Scan | #Spec | Start | End | PTM |
| --- | --- | --- | --- | --- | --- | --- | --- | --- | --- | --- | --- | --- |
| S.IEDPFDQDDWE.A | Y | 26.40 | 1407.5466 | -0.3 | 704.7804 | 2 | 14.04 | 2116 | 1 | 292 | 302 |  |
| total 1 peptides |  |  |  |  |  |  |  |  |  |  |  |  |
| --- | --- | --- | --- | --- | --- | --- | --- | --- | --- | --- | --- | --- |

tr|A0A6P3W3B1|A0A6P3W3B1\_CLUHA

back to list

  

| Protein Coverage
| Supporting Peptides
|

Protein Coverage:

Supporting Peptides:

| Peptide | Uniq | -10lgP | Mass | ppm | m/z | z | RT | Scan | #Spec | Start | End | PTM |
| --- | --- | --- | --- | --- | --- | --- | --- | --- | --- | --- | --- | --- |
| S.IEDPFDQDDWE.N | Y | 26.40 | 1407.5466 | -0.3 | 704.7804 | 2 | 14.04 | 2116 | 1 | 292 | 302 |  |
| total 1 peptides |  |  |  |  |  |  |  |  |  |  |  |  |
| --- | --- | --- | --- | --- | --- | --- | --- | --- | --- | --- | --- | --- |

tr|A0A3B4CPC9|A0A3B4CPC9\_PYGNA

back to list

  

| Protein Coverage
| Supporting Peptides
|

Protein Coverage:

Supporting Peptides:

| Peptide | Uniq | -10lgP | Mass | ppm | m/z | z | RT | Scan | #Spec | Start | End | PTM |
| --- | --- | --- | --- | --- | --- | --- | --- | --- | --- | --- | --- | --- |
| S.IEDPFDQDDWE.N | Y | 26.40 | 1407.5466 | -0.3 | 704.7804 | 2 | 14.04 | 2116 | 1 | 292 | 302 |  |
| total 1 peptides |  |  |  |  |  |  |  |  |  |  |  |  |
| --- | --- | --- | --- | --- | --- | --- | --- | --- | --- | --- | --- | --- |

tr|A0A6P3W5E7|A0A6P3W5E7\_CLUHA

back to list

  

| Protein Coverage
| Supporting Peptides
|

Protein Coverage:

Supporting Peptides:

| Peptide | Uniq | -10lgP | Mass | ppm | m/z | z | RT | Scan | #Spec | Start | End | PTM |
| --- | --- | --- | --- | --- | --- | --- | --- | --- | --- | --- | --- | --- |
| S.IEDPFDQDDWE.N | Y | 26.40 | 1407.5466 | -0.3 | 704.7804 | 2 | 14.04 | 2116 | 1 | 292 | 302 |  |
| total 1 peptides |  |  |  |  |  |  |  |  |  |  |  |  |
| --- | --- | --- | --- | --- | --- | --- | --- | --- | --- | --- | --- | --- |

tr|A0A3P8TVY9|A0A3P8TVY9\_AMPPE

back to list

  

| Protein Coverage
| Supporting Peptides
|

Protein Coverage:

Supporting Peptides:

| Peptide | Uniq | -10lgP | Mass | ppm | m/z | z | RT | Scan | #Spec | Start | End | PTM |
| --- | --- | --- | --- | --- | --- | --- | --- | --- | --- | --- | --- | --- |
| S.IEDPFDQDDWE.N | Y | 26.40 | 1407.5466 | -0.3 | 704.7804 | 2 | 14.04 | 2116 | 1 | 292 | 302 |  |
| total 1 peptides |  |  |  |  |  |  |  |  |  |  |  |  |
| --- | --- | --- | --- | --- | --- | --- | --- | --- | --- | --- | --- | --- |

tr|A0A6P7P1U1|A0A6P7P1U1\_BETSP

back to list

  

| Protein Coverage
| Supporting Peptides
|

Protein Coverage:

Supporting Peptides:

| Peptide | Uniq | -10lgP | Mass | ppm | m/z | z | RT | Scan | #Spec | Start | End | PTM |
| --- | --- | --- | --- | --- | --- | --- | --- | --- | --- | --- | --- | --- |
| S.IEDPFDQDDWE.A | Y | 26.40 | 1407.5466 | -0.3 | 704.7804 | 2 | 14.04 | 2116 | 1 | 292 | 302 |  |
| total 1 peptides |  |  |  |  |  |  |  |  |  |  |  |  |
| --- | --- | --- | --- | --- | --- | --- | --- | --- | --- | --- | --- | --- |

tr|A0A4W4G3C7|A0A4W4G3C7\_ELEEL

back to list

  

| Protein Coverage
| Supporting Peptides
|

Protein Coverage:

Supporting Peptides:

| Peptide | Uniq | -10lgP | Mass | ppm | m/z | z | RT | Scan | #Spec | Start | End | PTM |
| --- | --- | --- | --- | --- | --- | --- | --- | --- | --- | --- | --- | --- |
| S.IEDPFDQDDWE.A | Y | 26.40 | 1407.5466 | -0.3 | 704.7804 | 2 | 14.04 | 2116 | 1 | 322 | 332 |  |
| total 1 peptides |  |  |  |  |  |  |  |  |  |  |  |  |
| --- | --- | --- | --- | --- | --- | --- | --- | --- | --- | --- | --- | --- |

tr|A0A4W4G3N6|A0A4W4G3N6\_ELEEL

back to list

  

| Protein Coverage
| Supporting Peptides
|

Protein Coverage:

Supporting Peptides:

| Peptide | Uniq | -10lgP | Mass | ppm | m/z | z | RT | Scan | #Spec | Start | End | PTM |
| --- | --- | --- | --- | --- | --- | --- | --- | --- | --- | --- | --- | --- |
| S.IEDPFDQDDWE.A | Y | 26.40 | 1407.5466 | -0.3 | 704.7804 | 2 | 14.04 | 2116 | 1 | 298 | 308 |  |
| total 1 peptides |  |  |  |  |  |  |  |  |  |  |  |  |
| --- | --- | --- | --- | --- | --- | --- | --- | --- | --- | --- | --- | --- |

tr|A0A3P8NTT0|A0A3P8NTT0\_ASTCA

back to list

  

| Protein Coverage
| Supporting Peptides
|

Protein Coverage:

Supporting Peptides:

| Peptide | Uniq | -10lgP | Mass | ppm | m/z | z | RT | Scan | #Spec | Start | End | PTM |
| --- | --- | --- | --- | --- | --- | --- | --- | --- | --- | --- | --- | --- |
| S.IEDPFDQDDWE.Q | Y | 26.40 | 1407.5466 | -0.3 | 704.7804 | 2 | 14.04 | 2116 | 1 | 303 | 313 |  |
| total 1 peptides |  |  |  |  |  |  |  |  |  |  |  |  |
| --- | --- | --- | --- | --- | --- | --- | --- | --- | --- | --- | --- | --- |

tr|A0A6P7MN74|A0A6P7MN74\_BETSP

back to list

  

| Protein Coverage
| Supporting Peptides
|

Protein Coverage:

Supporting Peptides:

| Peptide | Uniq | -10lgP | Mass | ppm | m/z | z | RT | Scan | #Spec | Start | End | PTM |
| --- | --- | --- | --- | --- | --- | --- | --- | --- | --- | --- | --- | --- |
| S.IEDPFDQDDWE.A | Y | 26.40 | 1407.5466 | -0.3 | 704.7804 | 2 | 14.04 | 2116 | 1 | 311 | 321 |  |
| total 1 peptides |  |  |  |  |  |  |  |  |  |  |  |  |
| --- | --- | --- | --- | --- | --- | --- | --- | --- | --- | --- | --- | --- |

tr|A0A3B4ERE5|A0A3B4ERE5\_PYGNA

back to list

  

| Protein Coverage
| Supporting Peptides
|

Protein Coverage:

Supporting Peptides:

| Peptide | Uniq | -10lgP | Mass | ppm | m/z | z | RT | Scan | #Spec | Start | End | PTM |
| --- | --- | --- | --- | --- | --- | --- | --- | --- | --- | --- | --- | --- |
| S.IEDPFDQDDWE.N | Y | 26.40 | 1407.5466 | -0.3 | 704.7804 | 2 | 14.04 | 2116 | 1 | 312 | 322 |  |
| total 1 peptides |  |  |  |  |  |  |  |  |  |  |  |  |
| --- | --- | --- | --- | --- | --- | --- | --- | --- | --- | --- | --- | --- |

tr|A0A3B4DDR1|A0A3B4DDR1\_PYGNA

back to list

  

| Protein Coverage
| Supporting Peptides
|

Protein Coverage:

Supporting Peptides:

| Peptide | Uniq | -10lgP | Mass | ppm | m/z | z | RT | Scan | #Spec | Start | End | PTM |
| --- | --- | --- | --- | --- | --- | --- | --- | --- | --- | --- | --- | --- |
| S.IEDPFDQDDWE.N | Y | 26.40 | 1407.5466 | -0.3 | 704.7804 | 2 | 14.04 | 2116 | 1 | 328 | 338 |  |
| total 1 peptides |  |  |  |  |  |  |  |  |  |  |  |  |
| --- | --- | --- | --- | --- | --- | --- | --- | --- | --- | --- | --- | --- |

tr|A0A7J6BNX1|A0A7J6BNX1\_9TELE

back to list

  

| Protein Coverage
| Supporting Peptides
|

Protein Coverage:

Supporting Peptides:

| Peptide | Uniq | -10lgP | Mass | ppm | m/z | z | RT | Scan | #Spec | Start | End | PTM |
| --- | --- | --- | --- | --- | --- | --- | --- | --- | --- | --- | --- | --- |
| S.IEDPFDQDDWE.A | Y | 26.40 | 1407.5466 | -0.3 | 704.7804 | 2 | 14.04 | 2116 | 1 | 337 | 347 |  |
| total 1 peptides |  |  |  |  |  |  |  |  |  |  |  |  |
| --- | --- | --- | --- | --- | --- | --- | --- | --- | --- | --- | --- | --- |

tr|A0A6P7P064|A0A6P7P064\_BETSP

back to list

  

| Protein Coverage
| Supporting Peptides
|

Protein Coverage:

Supporting Peptides:

| Peptide | Uniq | -10lgP | Mass | ppm | m/z | z | RT | Scan | #Spec | Start | End | PTM |
| --- | --- | --- | --- | --- | --- | --- | --- | --- | --- | --- | --- | --- |
| S.IEDPFDQDDWE.A | Y | 26.40 | 1407.5466 | -0.3 | 704.7804 | 2 | 14.04 | 2116 | 1 | 349 | 359 |  |
| total 1 peptides |  |  |  |  |  |  |  |  |  |  |  |  |
| --- | --- | --- | --- | --- | --- | --- | --- | --- | --- | --- | --- | --- |

tr|A0A5A9PD51|A0A5A9PD51\_9TELE

back to list

  

| Protein Coverage
| Supporting Peptides
|

Protein Coverage:

Supporting Peptides:

| Peptide | Uniq | -10lgP | Mass | ppm | m/z | z | RT | Scan | #Spec | Start | End | PTM |
| --- | --- | --- | --- | --- | --- | --- | --- | --- | --- | --- | --- | --- |
| R.KEDKAGSQ.G | Y | 23.02 | 861.4192 | -0.4 | 431.7167 | 2 | 2.00 | 253 | 2 | 587 | 594 |  |
| total 1 peptides |  |  |  |  |  |  |  |  |  |  |  |  |
| --- | --- | --- | --- | --- | --- | --- | --- | --- | --- | --- | --- | --- |

tr|A0A6P8GQF7|A0A6P8GQF7\_CLUHA

back to list

  

| Protein Coverage
| Supporting Peptides
|

Protein Coverage:

Supporting Peptides:

| Peptide | Uniq | -10lgP | Mass | ppm | m/z | z | RT | Scan | #Spec | Start | End | PTM |
| --- | --- | --- | --- | --- | --- | --- | --- | --- | --- | --- | --- | --- |
| S.GADDKLKDK.I | Y | 22.74 | 988.5189 | -0.5 | 495.2665 | 2 | 8.10 | 1053 | 1 | 1371 | 1379 |  |
| total 1 peptides |  |  |  |  |  |  |  |  |  |  |  |  |
| --- | --- | --- | --- | --- | --- | --- | --- | --- | --- | --- | --- | --- |

tr|A0A6P8GCK4|A0A6P8GCK4\_CLUHA

back to list

  

| Protein Coverage
| Supporting Peptides
|

Protein Coverage:

Supporting Peptides:

| Peptide | Uniq | -10lgP | Mass | ppm | m/z | z | RT | Scan | #Spec | Start | End | PTM |
| --- | --- | --- | --- | --- | --- | --- | --- | --- | --- | --- | --- | --- |
| S.GADDKLKDK.I | Y | 22.74 | 988.5189 | -0.5 | 495.2665 | 2 | 8.10 | 1053 | 1 | 1381 | 1389 |  |
| total 1 peptides |  |  |  |  |  |  |  |  |  |  |  |  |
| --- | --- | --- | --- | --- | --- | --- | --- | --- | --- | --- | --- | --- |

tr|A0A6P8GCW3|A0A6P8GCW3\_CLUHA

back to list

  

| Protein Coverage
| Supporting Peptides
|

Protein Coverage:

Supporting Peptides:

| Peptide | Uniq | -10lgP | Mass | ppm | m/z | z | RT | Scan | #Spec | Start | End | PTM |
| --- | --- | --- | --- | --- | --- | --- | --- | --- | --- | --- | --- | --- |
| S.GADDKLKDK.I | Y | 22.74 | 988.5189 | -0.5 | 495.2665 | 2 | 8.10 | 1053 | 1 | 1414 | 1422 |  |
| total 1 peptides |  |  |  |  |  |  |  |  |  |  |  |  |
| --- | --- | --- | --- | --- | --- | --- | --- | --- | --- | --- | --- | --- |

tr|A0A6P8GNT0|A0A6P8GNT0\_CLUHA

back to list

  

| Protein Coverage
| Supporting Peptides
|

Protein Coverage:

Supporting Peptides:

| Peptide | Uniq | -10lgP | Mass | ppm | m/z | z | RT | Scan | #Spec | Start | End | PTM |
| --- | --- | --- | --- | --- | --- | --- | --- | --- | --- | --- | --- | --- |
| S.GADDKLKDK.I | Y | 22.74 | 988.5189 | -0.5 | 495.2665 | 2 | 8.10 | 1053 | 1 | 1424 | 1432 |  |
| total 1 peptides |  |  |  |  |  |  |  |  |  |  |  |  |
| --- | --- | --- | --- | --- | --- | --- | --- | --- | --- | --- | --- | --- |

tr|A0A674PAA1|A0A674PAA1\_TAKRU

back to list

  

| Protein Coverage
| Supporting Peptides
|

Protein Coverage:

Supporting Peptides:

| Peptide | Uniq | -10lgP | Mass | ppm | m/z | z | RT | Scan | #Spec | Start | End | PTM |
| --- | --- | --- | --- | --- | --- | --- | --- | --- | --- | --- | --- | --- |
| E.KTIDDLEDE.V | Y | 22.10 | 1076.4873 | -0.3 | 539.2508 | 2 | 10.91 | 1552 | 1 | 145 | 153 |  |
| total 1 peptides |  |  |  |  |  |  |  |  |  |  |  |  |
| --- | --- | --- | --- | --- | --- | --- | --- | --- | --- | --- | --- | --- |

tr|A0A4W5K3X5|A0A4W5K3X5\_9TELE

back to list

  

| Protein Coverage
| Supporting Peptides
|

Protein Coverage:

Supporting Peptides:

| Peptide | Uniq | -10lgP | Mass | ppm | m/z | z | RT | Scan | #Spec | Start | End | PTM |
| --- | --- | --- | --- | --- | --- | --- | --- | --- | --- | --- | --- | --- |
| E.KTIDDLEDE.V | Y | 22.10 | 1076.4873 | -0.3 | 539.2508 | 2 | 10.91 | 1552 | 1 | 225 | 233 |  |
| total 1 peptides |  |  |  |  |  |  |  |  |  |  |  |  |
| --- | --- | --- | --- | --- | --- | --- | --- | --- | --- | --- | --- | --- |

tr|A0A4W5KNV1|A0A4W5KNV1\_9TELE

back to list

  

| Protein Coverage
| Supporting Peptides
|

Protein Coverage:

Supporting Peptides:

| Peptide | Uniq | -10lgP | Mass | ppm | m/z | z | RT | Scan | #Spec | Start | End | PTM |
| --- | --- | --- | --- | --- | --- | --- | --- | --- | --- | --- | --- | --- |
| E.KTIDDLEDE.V | Y | 22.10 | 1076.4873 | -0.3 | 539.2508 | 2 | 10.91 | 1552 | 1 | 246 | 254 |  |
| total 1 peptides |  |  |  |  |  |  |  |  |  |  |  |  |
| --- | --- | --- | --- | --- | --- | --- | --- | --- | --- | --- | --- | --- |

tr|A0A3Q1CSK5|A0A3Q1CSK5\_AMPOC

back to list

  

| Protein Coverage
| Supporting Peptides
|

Protein Coverage:

Supporting Peptides:

| Peptide | Uniq | -10lgP | Mass | ppm | m/z | z | RT | Scan | #Spec | Start | End | PTM |
| --- | --- | --- | --- | --- | --- | --- | --- | --- | --- | --- | --- | --- |
| E.KTIDDLEDE.V | Y | 22.10 | 1076.4873 | -0.3 | 539.2508 | 2 | 10.91 | 1552 | 1 | 251 | 259 |  |
| total 1 peptides |  |  |  |  |  |  |  |  |  |  |  |  |
| --- | --- | --- | --- | --- | --- | --- | --- | --- | --- | --- | --- | --- |

tr|A0A4W5KNI2|A0A4W5KNI2\_9TELE

back to list

  

| Protein Coverage
| Supporting Peptides
|

Protein Coverage:

Supporting Peptides:

| Peptide | Uniq | -10lgP | Mass | ppm | m/z | z | RT | Scan | #Spec | Start | End | PTM |
| --- | --- | --- | --- | --- | --- | --- | --- | --- | --- | --- | --- | --- |
| E.KTIDDLEDE.V | Y | 22.10 | 1076.4873 | -0.3 | 539.2508 | 2 | 10.91 | 1552 | 1 | 251 | 259 |  |
| total 1 peptides |  |  |  |  |  |  |  |  |  |  |  |  |
| --- | --- | --- | --- | --- | --- | --- | --- | --- | --- | --- | --- | --- |

tr|A0A6G0HZW7|A0A6G0HZW7\_LARCR

back to list

  

| Protein Coverage
| Supporting Peptides
|

Protein Coverage:

Supporting Peptides:

| Peptide | Uniq | -10lgP | Mass | ppm | m/z | z | RT | Scan | #Spec | Start | End | PTM |
| --- | --- | --- | --- | --- | --- | --- | --- | --- | --- | --- | --- | --- |
| E.KTIDDLEDE.V | Y | 22.10 | 1076.4873 | -0.3 | 539.2508 | 2 | 10.91 | 1552 | 1 | 251 | 259 |  |
| total 1 peptides |  |  |  |  |  |  |  |  |  |  |  |  |
| --- | --- | --- | --- | --- | --- | --- | --- | --- | --- | --- | --- | --- |

tr|A0A6J2PEZ1|A0A6J2PEZ1\_COTGO

back to list

  

| Protein Coverage
| Supporting Peptides
|

Protein Coverage:

Supporting Peptides:

| Peptide | Uniq | -10lgP | Mass | ppm | m/z | z | RT | Scan | #Spec | Start | End | PTM |
| --- | --- | --- | --- | --- | --- | --- | --- | --- | --- | --- | --- | --- |
| E.KTIDDLEDE.L | Y | 22.10 | 1076.4873 | -0.3 | 539.2508 | 2 | 10.91 | 1552 | 1 | 251 | 259 |  |
| total 1 peptides |  |  |  |  |  |  |  |  |  |  |  |  |
| --- | --- | --- | --- | --- | --- | --- | --- | --- | --- | --- | --- | --- |

tr|D2KQG3|D2KQG3\_SINCH

back to list

  

| Protein Coverage
| Supporting Peptides
|

Protein Coverage:

Supporting Peptides:

| Peptide | Uniq | -10lgP | Mass | ppm | m/z | z | RT | Scan | #Spec | Start | End | PTM |
| --- | --- | --- | --- | --- | --- | --- | --- | --- | --- | --- | --- | --- |
| F.EVIDQDKSGFIE.E | Y | 20.02 | 1378.6616 | -1.6 | 690.3370 | 2 | 12.51 | 1846 | 1 | 49 | 60 |  |
| total 1 peptides |  |  |  |  |  |  |  |  |  |  |  |  |
| --- | --- | --- | --- | --- | --- | --- | --- | --- | --- | --- | --- | --- |

tr|B9VJM3|B9VJM3\_SINCH

back to list

  

| Protein Coverage
| Supporting Peptides
|

Protein Coverage:

Supporting Peptides:

| Peptide | Uniq | -10lgP | Mass | ppm | m/z | z | RT | Scan | #Spec | Start | End | PTM |
| --- | --- | --- | --- | --- | --- | --- | --- | --- | --- | --- | --- | --- |
| F.EVIDQDKSGFIE.E | Y | 20.02 | 1378.6616 | -1.6 | 690.3370 | 2 | 12.51 | 1846 | 1 | 49 | 60 |  |
| total 1 peptides |  |  |  |  |  |  |  |  |  |  |  |  |
| --- | --- | --- | --- | --- | --- | --- | --- | --- | --- | --- | --- | --- |

---

Prepared with PEAKS ™ (bioinfor.com)
