# Supplementary material for: Proteomic and Functional Characterization of Antimicrobial Peptides Derived from Fisheries Bycatch via Enzymatic Hydrolysis
Source: Mar Drugs. 2026 Jan 10;24(1):36. doi: 10.3390/md24010036 (PMC12843163; doi:10.3390/md24010036)
Supplement: Supplementary file 1 [file marinedrugs-24-00036-s001.zip › SM43/PP/protein.html]

proteins


Summary

  

# 1. Notes

# 2. Result Statistics

**Figure 1.**
False discovery rate (FDR) curve. X axis is the number of peptide-spectrum matches (PSM) being kept. Y axis is the corresponding FDR.

  


  

**Figure 2.**
PSM score distribution.  **(a)**
Distribution of PEAKS peptide score;  **(b)**
Scatterplot of PEAKS peptide score versus precursor mass error.

**(a)**

**(b)**

**Figure 3.**
De novo result validation. Distribution of residue local confidence:  **(a)**
Residues in de novo sequences validated by confident database peptide assignment;  **(b)**
Residues in "de novo only" sequences.

**(a)**

**(b)**

**Table 1.**
Statistics of data.

|  |  |
| --- | --- |
| # of MS Scans | 1037 |
| # of MS/MS Scans | 2829 |


**Table 2.**
Result filtration parameters.

|  |  |
| --- | --- |
| Peptide -10lgP | ≥15 |
| Protein -10lgP | ≥20 |
| Proteins unique peptides | ≥0 |
| De novo ALC Score | ≥50% |


**Table 3.**
Statistics of filtered result.

|  |  |
| --- | --- |
| Peptide-Spectrum Matches | 131 |
| Peptide Sequences | 123 |
| Protein Groups | 43 |
| Proteins | 256 |
| Proteins (#Unique Peptides) | 43 (>2); 47 (=2); 137 (=1); |
| FDR (Peptide-Spectrum Matches) | 10.7% |
| FDR (Peptide Sequences) | 11.4% |
| FDR (Protein) | 0.4% |
| De Novo Only Spectra | 1244 |

**Table 4.**
PTM profile.

| Name | ∆Mass | #PSM | Position |
| --- | --- | --- | --- |

# 3. Experiment Control

**Figure 4.**
Precursor mass error of peptide-spectrum matches (PSM) in filtered result.  **(a)**
Distribution of precursor mass error in ppm;  **(b)**
Scatterplot of precursor m/z versus precursor mass error in ppm.

**(a)**

**(b)**

**Table 5.**
Number of identified peptides in each sample by the number of missed cleavages

|  |  |  |  |  |  |
| --- | --- | --- | --- | --- | --- |
| Missed Cleavages | 0 | 1 | 2 | 3 | 4+ |
| Orbi2222 | 0 | 0 | 0 | 0 | 123 |

# 4. Other Information

**Table 6.**
Search parameters.

Search Engine Name: PEAKS 7.0  
Parent Mass Error Tolerance: 0.03 Da  
Fragment Mass Error Tolerance: 0.1 Da  
Precursor Mass Search Type: monoisotopic  
Enzyme: None  
Max Missed Cleavages: 100  
Non-specific Cleavage: both  
Max variable PTM per peptide: 3  
Database: Teleosteo  
Taxon: All  
Searched Entry: 4529691  
FDR Estimation: Enabled  
Merge Options: 0.1 min. 0.03 Da  
Precursor Options: corrected  
Charge Options: no correction  
Filter Options: no filter  
Process: true

**Table 7.**
Instrument parameters.

Fractions: OB\_PIMENTA\_PP\_20220211\_06.raw  
Ion Source: ESI(nano-spray)  
Fragmentation Mode: CID/ECD  
MS Scan Mode: FT-ICR/Orbitrap  
MS/MS Scan Mode: Linear Ion Trap

  

Protein List

  

| Protein Group | Protein ID | Accession | -10lgP | Coverage (%) | #Peptides | #Unique | PTM | Avg. Mass | Description |
| --- | --- | --- | --- | --- | --- | --- | --- | --- | --- |
| 1 | 12040 | tr\|A0A3S2MUF9\|A0A3S2MUF9\_ORYJA | 138.72 | 20 | 23 | 23 | N | 41945 | Uncharacterized protein OS=Oryzias javanicus OX=123683 GN=OJAV\_G00024300 PE=3 SV=1 |
| 1 | 66846 | tr\|A0A7J6C441\|A0A7J6C441\_9TELE | 138.72 | 20 | 23 | 23 | N | 41959 | Uncharacterized protein OS=Onychostoma macrolepis OX=369639 GN=G5714\_016621 PE=3 SV=1 |
| 10 | 73802 | tr\|A0A5C6NQR5\|A0A5C6NQR5\_9TELE | 131.22 | 7 | 8 | 0 | N | 52820 | Parvalbumin OS=Takifugu flavidus OX=433684 GN=D4764\_18G0006780 PE=3 SV=1 |
| 2 | 73777 | tr\|A0A6P6N9Z4\|A0A6P6N9Z4\_CARAU | 126.95 | 6 | 14 | 0 | N | 221582 | myosin heavy chain, fast skeletal muscle-like OS=Carassius auratus OX=7957 GN=LOC113076612 PE=3 SV=1 |
| 3 | 66917 | tr\|A0A3B4CZR3\|A0A3B4CZR3\_PYGNA | 126.54 | 6 | 14 | 0 | N | 213482 | Uncharacterized protein OS=Pygocentrus nattereri OX=42514 PE=3 SV=1 |
| 4 | 66800 | tr\|A0A674NSP7\|A0A674NSP7\_TAKRU | 125.65 | 6 | 13 | 0 | N | 223905 | Uncharacterized protein OS=Takifugu rubripes OX=31033 GN=myhm86-1 PE=3 SV=1 |
| 14 | 67489 | tr\|A0A6A4SNF9\|A0A6A4SNF9\_SCOMX | 124.92 | 4 | 6 | 0 | N | 77468 | Parvalbumin OS=Scophthalmus maximus OX=52904 GN=F2P81\_014924 PE=3 SV=1 |
| 5 | 66801 | tr\|Q90YF6\|Q90YF6\_PARFO | 124.71 | 5 | 12 | 0 | N | 221374 | Myosin heavy chain OS=Paracirrhites forsteri OX=100206 GN=MyoHC-T54 PE=2 SV=1 |
| 12 | 73788 | tr\|W5LNC0\|W5LNC0\_ASTMX | 115.33 | 33 | 7 | 0 | N | 11525 | Parvalbumin OS=Astyanax mexicanus OX=7994 PE=3 SV=2 |
| 12 | 73789 | tr\|A0A2I4BRJ5\|A0A2I4BRJ5\_9TELE | 115.33 | 33 | 7 | 0 | N | 11457 | Parvalbumin OS=Austrofundulus limnaeus OX=52670 GN=LOC106522062 PE=3 SV=1 |
| 12 | 73790 | tr\|A0A3B4TWT0\|A0A3B4TWT0\_SERDU | 115.33 | 33 | 7 | 0 | N | 11497 | Parvalbumin OS=Seriola dumerili OX=41447 PE=3 SV=1 |
| 12 | 73791 | tr\|A0A1A8L493\|A0A1A8L493\_9TELE | 115.33 | 33 | 7 | 0 | N | 11514 | Parvalbumin OS=Nothobranchius pienaari OX=704102 GN=PVALB1 PE=3 SV=1 |
| 12 | 73792 | tr\|A0A672FI61\|A0A672FI61\_SALFA | 115.33 | 33 | 7 | 0 | N | 11436 | Parvalbumin OS=Salarias fasciatus OX=181472 GN=LOC115387769 PE=3 SV=1 |
| 12 | 73793 | tr\|A0A1A8G5F7\|A0A1A8G5F7\_9TELE | 115.33 | 33 | 7 | 0 | N | 11544 | Parvalbumin OS=Nothobranchius korthausae OX=1143690 GN=PVALB1 PE=3 SV=1 |
| 12 | 73794 | tr\|A0A4W6DV48\|A0A4W6DV48\_LATCA | 115.33 | 33 | 7 | 0 | N | 11469 | Parvalbumin OS=Lates calcarifer OX=8187 GN=LOC108880037 PE=3 SV=1 |
| 12 | 67611 | tr\|F8U037\|F8U037\_EPIBR | 115.33 | 33 | 7 | 0 | N | 11432 | Parvalbumin (Fragment) OS=Epinephelus bruneus OX=323802 PE=2 SV=1 |
| 12 | 67613 | tr\|A0A1A7WW39\|A0A1A7WW39\_9TELE | 115.33 | 33 | 7 | 0 | N | 11532 | Parvalbumin OS=Iconisemion striatum OX=60296 GN=PVALB1 PE=3 SV=1 |
| 12 | 73795 | tr\|A0A1A8C322\|A0A1A8C322\_9TELE | 115.33 | 33 | 7 | 0 | N | 11498 | Parvalbumin OS=Nothobranchius kadleci OX=1051664 GN=PVALB1 PE=3 SV=1 |
| 12 | 73796 | tr\|A0A3Q3GY11\|A0A3Q3GY11\_9LABR | 115.33 | 33 | 7 | 0 | N | 11308 | Parvalbumin OS=Labrus bergylta OX=56723 PE=3 SV=1 |
| 12 | 73797 | tr\|A0A1A8J4D2\|A0A1A8J4D2\_NOTKU | 115.33 | 33 | 7 | 0 | N | 11498 | Parvalbumin OS=Nothobranchius kuhntae OX=321403 GN=PVALB1 PE=3 SV=1 |
| 13 | 67241 | tr\|A0A672Y4P6\|A0A672Y4P6\_9TELE | 102.98 | 23 | 6 | 6 | N | 17050 | Nucleoside diphosphate kinase B OS=Sphaeramia orbicularis OX=375764 GN=LOC115423821 PE=3 SV=1 |
| 13 | 67242 | tr\|A0A2I4CF45\|A0A2I4CF45\_9TELE | 102.98 | 23 | 6 | 6 | N | 16890 | Nucleoside diphosphate kinase B OS=Austrofundulus limnaeus OX=52670 GN=LOC106528082 PE=3 SV=1 |
| 13 | 67245 | tr\|A0A6P7ISB4\|A0A6P7ISB4\_9TELE | 102.98 | 23 | 6 | 6 | N | 16935 | Nucleoside diphosphate kinase B OS=Parambassis ranga OX=210632 GN=LOC114440125 PE=3 SV=1 |
| 13 | 67246 | tr\|A0A3Q4I698\|A0A3Q4I698\_NEOBR | 102.98 | 23 | 6 | 6 | N | 16950 | Nucleoside diphosphate kinase B OS=Neolamprologus brichardi OX=32507 PE=3 SV=1 |
| 13 | 73835 | tr\|A0A3B3Z6H5\|A0A3B3Z6H5\_9TELE | 102.98 | 23 | 6 | 6 | N | 16834 | Nucleoside diphosphate kinase B OS=Poecilia mexicana OX=48701 PE=3 SV=1 |
| 13 | 67249 | tr\|A0A3Q3A341\|A0A3Q3A341\_KRYMA | 102.98 | 23 | 6 | 6 | N | 16887 | Nucleoside diphosphate kinase B OS=Kryptolebias marmoratus OX=37003 PE=3 SV=1 |
| 13 | 67251 | tr\|A0A3P8Q104\|A0A3P8Q104\_ASTCA | 102.98 | 23 | 6 | 6 | N | 17195 | Nucleoside diphosphate kinase B OS=Astatotilapia calliptera OX=8154 PE=3 SV=1 |
| 13 | 67252 | tr\|A0A3Q3CUD8\|A0A3Q3CUD8\_HAPBU | 102.98 | 23 | 6 | 6 | N | 17195 | Nucleoside diphosphate kinase B OS=Haplochromis burtoni OX=8153 PE=3 SV=1 |
| 13 | 67253 | tr\|A0A3Q4I6B8\|A0A3Q4I6B8\_NEOBR | 102.98 | 23 | 6 | 6 | N | 17195 | Nucleoside diphosphate kinase B OS=Neolamprologus brichardi OX=32507 PE=3 SV=1 |
| 13 | 67258 | tr\|M4ADR4\|M4ADR4\_XIPMA | 102.98 | 22 | 6 | 6 | N | 18277 | Nucleoside diphosphate kinase B OS=Xiphophorus maculatus OX=8083 PE=3 SV=2 |
| 13 | 73836 | tr\|A0A3B3Z5H2\|A0A3B3Z5H2\_9TELE | 102.98 | 21 | 6 | 6 | N | 18371 | Nucleoside diphosphate kinase B OS=Poecilia mexicana OX=48701 PE=3 SV=1 |
| 13 | 73837 | tr\|A0A087YID2\|A0A087YID2\_POEFO | 102.98 | 21 | 6 | 6 | N | 18371 | Nucleoside diphosphate kinase B OS=Poecilia formosa OX=48698 PE=3 SV=2 |
| 13 | 73838 | tr\|A0A3Q2PNK7\|A0A3Q2PNK7\_FUNHE | 102.98 | 14 | 6 | 6 | N | 26728 | Nucleoside diphosphate kinase B OS=Fundulus heteroclitus OX=8078 PE=3 SV=1 |
| 9 | 67400 | tr\|A0A4W4EA72\|A0A4W4EA72\_ELEEL | 102.77 | 16 | 7 | 5 | N | 11756 | Parvalbumin OS=Electrophorus electricus OX=8005 GN=LOC113569881 PE=3 SV=1 |
| 15 | 73844 | tr\|A0A3B4TLJ3\|A0A3B4TLJ3\_SERDU | 96.43 | 29 | 5 | 0 | N | 11348 | Parvalbumin OS=Seriola dumerili OX=41447 PE=3 SV=1 |
| 15 | 73845 | tr\|A9ZTF1\|A9ZTF1\_KATPE | 96.43 | 28 | 5 | 0 | N | 11442 | Parvalbumin OS=Katsuwonus pelamis OX=8226 GN=Kat p 1 PE=2 SV=1 |
| 15 | 73846 | tr\|G8GWA1\|G8GWA1\_CARAU | 96.43 | 28 | 5 | 0 | N | 11655 | Parvalbumin OS=Carassius auratus OX=7957 PE=2 SV=2 |
| 15 | 73847 | tr\|A0A3B4TM81\|A0A3B4TM81\_SERDU | 96.43 | 28 | 5 | 0 | N | 11521 | Parvalbumin OS=Seriola dumerili OX=41447 PE=3 SV=1 |
| 15 | 73848 | tr\|A0A6P6KMF1\|A0A6P6KMF1\_CARAU | 96.43 | 28 | 5 | 0 | N | 11567 | Parvalbumin OS=Carassius auratus OX=7957 GN=LOC113052464 PE=3 SV=1 |
| 6 | 73779 | tr\|A0A6G0IXD3\|A0A6G0IXD3\_LARCR | 90.98 | 5 | 12 | 1 | N | 217948 | Myosin heavy chain, fast skeletal muscle OS=Larimichthys crocea OX=215358 GN=D5F01\_LYC04733 PE=3 SV=1 |
| 6 | 73780 | tr\|A0A6G0IXM2\|A0A6G0IXM2\_LARCR | 90.98 | 5 | 12 | 1 | N | 219009 | Myosin heavy chain, fast skeletal muscle OS=Larimichthys crocea OX=215358 GN=D5F01\_LYC04735 PE=3 SV=1 |
| 6 | 73781 | tr\|A0A6G0IXG4\|A0A6G0IXG4\_LARCR | 90.98 | 5 | 12 | 1 | N | 220119 | Myosin heavy chain, fast skeletal muscle OS=Larimichthys crocea OX=215358 GN=D5F01\_LYC04732 PE=3 SV=1 |
| 7 | 73782 | tr\|G3NEV3\|G3NEV3\_GASAC | 87.61 | 5 | 10 | 0 | N | 220538 | Uncharacterized protein OS=Gasterosteus aculeatus OX=69293 PE=3 SV=1 |
| 7 | 73783 | tr\|A0A6P8W5C3\|A0A6P8W5C3\_GYMAC | 87.61 | 5 | 10 | 0 | N | 221042 | myosin heavy chain, fast skeletal muscle-like OS=Gymnodraco acuticeps OX=8218 GN=LOC117560824 PE=3 SV=1 |
| 7 | 73784 | tr\|A0A6P8VS48\|A0A6P8VS48\_GYMAC | 87.61 | 5 | 10 | 0 | N | 221057 | myosin heavy chain, fast skeletal muscle-like OS=Gymnodraco acuticeps OX=8218 GN=LOC117560783 PE=3 SV=1 |
| 24 | 67953 | tr\|A0A3Q3W464\|A0A3Q3W464\_MOLML | 85.00 | 17 | 3 | 1 | N | 12300 | Parvalbumin OS=Mola mola OX=94237 PE=3 SV=1 |
| 24 | 67954 | tr\|A0A3Q3WAG3\|A0A3Q3WAG3\_MOLML | 85.00 | 15 | 3 | 1 | N | 13233 | Parvalbumin OS=Mola mola OX=94237 PE=3 SV=1 |
| 24 | 67955 | tr\|A0A3Q4AW30\|A0A3Q4AW30\_MOLML | 85.00 | 15 | 3 | 1 | N | 14276 | Parvalbumin OS=Mola mola OX=94237 PE=3 SV=1 |
| 24 | 67956 | tr\|A0A3Q3W3Y6\|A0A3Q3W3Y6\_MOLML | 85.00 | 14 | 3 | 1 | N | 14526 | Parvalbumin OS=Mola mola OX=94237 PE=3 SV=1 |
| 8 | 66953 | tr\|A0A6P8GNA2\|A0A6P8GNA2\_CLUHA | 83.39 | 4 | 9 | 1 | N | 221189 | myosin heavy chain, fast skeletal muscle-like isoform X1 OS=Clupea harengus OX=7950 GN=LOC105899558 PE=3 SV=1 |
| 17 | 67795 | tr\|A0A3Q1HW42\|A0A3Q1HW42\_9TELE | 73.03 | 12 | 5 | 1 | N | 21205 | Adenylate kinase isoenzyme 1 OS=Acanthochromis polyacanthus OX=80966 GN=AK1 PE=3 SV=1 |
| 17 | 67796 | tr\|A0A3B5BIY5\|A0A3B5BIY5\_9TELE | 73.03 | 12 | 5 | 1 | N | 21206 | Adenylate kinase isoenzyme 1 OS=Stegastes partitus OX=144197 GN=AK1 PE=3 SV=1 |
| 25 | 67396 | tr\|A0A4U5UZN8\|A0A4U5UZN8\_COLLU | 67.80 | 3 | 3 | 3 | N | 98899 | Calsequestrin OS=Collichthys lucidus OX=240159 GN=D9C73\_013496 PE=3 SV=1 |
| 25 | 67200 | tr\|A0A7N8YD85\|A0A7N8YD85\_9TELE | 67.80 | 6 | 3 | 3 | N | 50163 | Calsequestrin OS=Mastacembelus armatus OX=205130 PE=3 SV=1 |
| 25 | 67202 | tr\|A0A7N8Y4P6\|A0A7N8Y4P6\_9TELE | 67.80 | 6 | 3 | 3 | N | 50544 | Calsequestrin OS=Mastacembelus armatus OX=205130 PE=3 SV=1 |
| 25 | 67204 | tr\|A0A7N8XJX7\|A0A7N8XJX7\_9TELE | 67.80 | 6 | 3 | 3 | N | 52431 | Calsequestrin OS=Mastacembelus armatus OX=205130 PE=3 SV=1 |
| 25 | 67205 | tr\|A0A3Q3SHA7\|A0A3Q3SHA7\_9TELE | 67.80 | 6 | 3 | 3 | N | 52425 | Calsequestrin OS=Mastacembelus armatus OX=205130 PE=3 SV=2 |
| 25 | 67206 | tr\|A0A7N8XSZ9\|A0A7N8XSZ9\_9TELE | 67.80 | 6 | 3 | 3 | N | 52256 | Calsequestrin OS=Mastacembelus armatus OX=205130 PE=3 SV=1 |
| 25 | 67207 | tr\|A0A7N8XRW1\|A0A7N8XRW1\_9TELE | 67.80 | 6 | 3 | 3 | N | 52191 | Calsequestrin OS=Mastacembelus armatus OX=205130 PE=3 SV=1 |
| 25 | 67208 | tr\|A0A7N8Y2R6\|A0A7N8Y2R6\_9TELE | 67.80 | 6 | 3 | 3 | N | 52373 | Calsequestrin OS=Mastacembelus armatus OX=205130 PE=3 SV=1 |
| 25 | 67209 | tr\|A0A7N8Y336\|A0A7N8Y336\_9TELE | 67.80 | 6 | 3 | 3 | N | 52793 | Calsequestrin OS=Mastacembelus armatus OX=205130 PE=3 SV=1 |
| 22 | 67099 | tr\|A0A1A8GBU0\|A0A1A8GBU0\_9TELE | 67.52 | 28 | 4 | 1 | N | 11460 | Parvalbumin OS=Nothobranchius korthausae OX=1143690 GN=PVALB4 PE=3 SV=1 |
| 31 | 67449 | tr\|A0A3B3ZS88\|A0A3B3ZS88\_9GOBI | 66.66 | 9 | 2 | 2 | N | 25528 | Uncharacterized protein OS=Periophthalmus magnuspinnatus OX=409849 PE=3 SV=1 |
| 31 | 67450 | tr\|A0A3Q1CJW7\|A0A3Q1CJW7\_AMPOC | 66.66 | 9 | 2 | 2 | N | 26282 | Troponin T, fast skeletal muscle isoforms-like OS=Amphiprion ocellaris OX=80972 PE=3 SV=1 |
| 31 | 67451 | tr\|A0A3Q3EFK1\|A0A3Q3EFK1\_9LABR | 66.66 | 9 | 2 | 2 | N | 26666 | Troponin T, fast skeletal muscle isoforms-like OS=Labrus bergylta OX=56723 PE=3 SV=1 |
| 31 | 67452 | tr\|A0A3Q3AF19\|A0A3Q3AF19\_KRYMA | 66.66 | 9 | 2 | 2 | N | 26771 | Troponin T, fast skeletal muscle isoforms-like OS=Kryptolebias marmoratus OX=37003 PE=3 SV=1 |
| 31 | 67453 | tr\|A0A3Q3AF32\|A0A3Q3AF32\_KRYMA | 66.66 | 9 | 2 | 2 | N | 26884 | Troponin T, fast skeletal muscle isoforms-like OS=Kryptolebias marmoratus OX=37003 PE=3 SV=1 |
| 31 | 67454 | tr\|A0A3P8UNE3\|A0A3P8UNE3\_CYNSE | 66.66 | 9 | 2 | 2 | N | 27351 | Troponin T, fast skeletal muscle isoforms-like OS=Cynoglossus semilaevis OX=244447 PE=3 SV=1 |
| 31 | 67456 | tr\|A0A672Y3T4\|A0A672Y3T4\_9TELE | 66.66 | 9 | 2 | 2 | N | 27423 | Uncharacterized protein OS=Sphaeramia orbicularis OX=375764 GN=LOC115421585 PE=3 SV=1 |
| 31 | 67458 | tr\|A0A3P8UNF6\|A0A3P8UNF6\_CYNSE | 66.66 | 8 | 2 | 2 | N | 28353 | Troponin T, fast skeletal muscle isoforms-like OS=Cynoglossus semilaevis OX=244447 PE=3 SV=1 |
| 31 | 67459 | tr\|A0A3B4UYC0\|A0A3B4UYC0\_SERDU | 66.66 | 8 | 2 | 2 | N | 29388 | Troponin T type 3a (skeletal, fast) OS=Seriola dumerili OX=41447 PE=3 SV=1 |
| 31 | 67460 | tr\|A0A3P8URR7\|A0A3P8URR7\_CYNSE | 66.66 | 8 | 2 | 2 | N | 29425 | Troponin T, fast skeletal muscle isoforms-like OS=Cynoglossus semilaevis OX=244447 PE=3 SV=1 |
| 31 | 67461 | tr\|A0A3P8URM3\|A0A3P8URM3\_CYNSE | 66.66 | 8 | 2 | 2 | N | 30206 | Troponin T, fast skeletal muscle isoforms-like OS=Cynoglossus semilaevis OX=244447 PE=3 SV=1 |
| 31 | 67462 | tr\|A0A3Q3XL97\|A0A3Q3XL97\_MOLML | 66.66 | 8 | 2 | 2 | N | 30493 | Troponin T type 3a (skeletal, fast) OS=Mola mola OX=94237 PE=3 SV=1 |
| 31 | 67463 | tr\|A0A3P8ULW6\|A0A3P8ULW6\_CYNSE | 66.66 | 7 | 2 | 2 | N | 31797 | Troponin T, fast skeletal muscle isoforms-like OS=Cynoglossus semilaevis OX=244447 PE=3 SV=1 |
| 31 | 67464 | tr\|A0A5C6P7G2\|A0A5C6P7G2\_9TELE | 66.66 | 7 | 2 | 2 | N | 32128 | Troponin T, fast skeletal muscle isoforms OS=Takifugu flavidus OX=433684 GN=D4764\_13G0000270 PE=3 SV=1 |
| 31 | 67465 | tr\|A0A3Q1CRB3\|A0A3Q1CRB3\_AMPOC | 66.66 | 7 | 2 | 2 | N | 32379 | Troponin T, fast skeletal muscle isoforms-like OS=Amphiprion ocellaris OX=80972 PE=3 SV=1 |
| 31 | 67466 | tr\|A0A665T7Y2\|A0A665T7Y2\_ECHNA | 66.66 | 7 | 2 | 2 | N | 33067 | Troponin T type 3a (skeletal, fast) OS=Echeneis naucrates OX=173247 PE=3 SV=1 |
| 31 | 67467 | tr\|A0A3Q1BEW8\|A0A3Q1BEW8\_AMPOC | 66.66 | 7 | 2 | 2 | N | 33080 | Troponin T, fast skeletal muscle isoforms-like OS=Amphiprion ocellaris OX=80972 PE=3 SV=1 |
| 31 | 67468 | tr\|A0A3P8THB6\|A0A3P8THB6\_AMPPE | 66.66 | 7 | 2 | 2 | N | 33365 | Troponin T type 3a (skeletal, fast) OS=Amphiprion percula OX=161767 PE=3 SV=1 |
| 31 | 67470 | tr\|A0A672YCL7\|A0A672YCL7\_9TELE | 66.66 | 7 | 2 | 2 | N | 33478 | Uncharacterized protein OS=Sphaeramia orbicularis OX=375764 GN=LOC115421585 PE=3 SV=1 |
| 31 | 67471 | tr\|A0A672YCL2\|A0A672YCL2\_9TELE | 66.66 | 7 | 2 | 2 | N | 34165 | Uncharacterized protein OS=Sphaeramia orbicularis OX=375764 GN=LOC115421585 PE=3 SV=1 |
| 31 | 67472 | tr\|A0A6P7KCL6\|A0A6P7KCL6\_9TELE | 66.66 | 7 | 2 | 2 | N | 34619 | troponin T, fast skeletal muscle isoforms-like isoform X3 OS=Parambassis ranga OX=210632 GN=LOC114452235 PE=3 SV=1 |
| 31 | 67473 | tr\|A0A6P7KCQ1\|A0A6P7KCQ1\_9TELE | 66.66 | 7 | 2 | 2 | N | 35105 | troponin T, fast skeletal muscle isoforms-like isoform X2 OS=Parambassis ranga OX=210632 GN=LOC114452235 PE=3 SV=1 |
| 16 | 67269 | tr\|A0A6P7KDL5\|A0A6P7KDL5\_9TELE | 65.64 | 15 | 5 | 5 | N | 32714 | tropomyosin alpha-1 chain isoform X1 OS=Parambassis ranga OX=210632 GN=LOC114452528 PE=3 SV=1 |
| 16 | 67270 | tr\|A0A6P7MR93\|A0A6P7MR93\_BETSP | 65.64 | 15 | 5 | 5 | N | 32714 | tropomyosin alpha-1 chain isoform X1 OS=Betta splendens OX=158456 GN=tpm1 PE=3 SV=1 |
| 16 | 67282 | tr\|X2J4M2\|X2J4M2\_PAROL | 65.64 | 15 | 5 | 5 | N | 32670 | Tropomyosin alpha-1 OS=Paralichthys olivaceus OX=8255 PE=2 SV=1 |
| 16 | 67271 | tr\|A0A3P9D7I0\|A0A3P9D7I0\_9CICH | 65.64 | 15 | 5 | 5 | N | 32822 | Tropomyosin 1 OS=Maylandia zebra OX=106582 GN=TPM1 PE=3 SV=1 |
| 16 | 67272 | tr\|A0A665VR95\|A0A665VR95\_ECHNA | 65.64 | 15 | 5 | 5 | N | 32714 | Tropomyosin 1 OS=Echeneis naucrates OX=173247 GN=tpm1 PE=3 SV=1 |
| 20 | 73841 | tr\|A0A3P9LRU7\|A0A3P9LRU7\_ORYLA | 65.15 | 8 | 4 | 2 | N | 42871 | Creatine kinase OS=Oryzias latipes OX=8090 PE=3 SV=1 |
| 20 | 67264 | tr\|A0A2I4BW49\|A0A2I4BW49\_9TELE | 65.15 | 8 | 4 | 2 | N | 43000 | Creatine kinase OS=Austrofundulus limnaeus OX=52670 GN=LOC106523169 PE=3 SV=1 |
| 20 | 73842 | tr\|A0A3B3I369\|A0A3B3I369\_ORYLA | 65.15 | 8 | 4 | 2 | N | 42871 | Creatine kinase OS=Oryzias latipes OX=8090 GN=LOC101166239 PE=3 SV=1 |
| 20 | 73843 | tr\|A0A3P9HLZ1\|A0A3P9HLZ1\_ORYLA | 65.15 | 8 | 4 | 2 | N | 42898 | Creatine kinase OS=Oryzias latipes OX=8090 PE=3 SV=1 |
| 20 | 67263 | tr\|A0A3P8WTN2\|A0A3P8WTN2\_CYNSE | 65.15 | 8 | 4 | 2 | N | 42864 | Creatine kinase OS=Cynoglossus semilaevis OX=244447 PE=3 SV=1 |
| 19 | 67126 | tr\|A0A3P8Q9R4\|A0A3P8Q9R4\_ASTCA | 61.93 | 8 | 4 | 2 | N | 42650 | Creatine kinase OS=Astatotilapia calliptera OX=8154 GN=CKM PE=3 SV=1 |
| 19 | 67691 | tr\|A0A6P7JDS0\|A0A6P7JDS0\_9TELE | 61.93 | 6 | 4 | 2 | N | 51567 | Creatine kinase OS=Parambassis ranga OX=210632 GN=LOC114444487 PE=3 SV=1 |
| 19 | 67717 | tr\|A0A3Q2Q7Q3\|A0A3Q2Q7Q3\_FUNHE | 61.93 | 9 | 4 | 2 | N | 38158 | Creatine kinase OS=Fundulus heteroclitus OX=8078 GN=CKM PE=3 SV=1 |
| 19 | 67705 | tr\|A0A3B4BZV1\|A0A3B4BZV1\_PYGNA | 61.93 | 8 | 4 | 2 | N | 42761 | Creatine kinase OS=Pygocentrus nattereri OX=42514 PE=3 SV=1 |
| 19 | 67706 | tr\|A0A6P7PB38\|A0A6P7PB38\_BETSP | 61.93 | 8 | 4 | 2 | N | 42709 | Creatine kinase OS=Betta splendens OX=158456 GN=LOC114867752 PE=3 SV=1 |
| 19 | 67693 | tr\|A0A3P8NKE2\|A0A3P8NKE2\_ASTCA | 61.93 | 8 | 4 | 2 | N | 43092 | Creatine kinase OS=Astatotilapia calliptera OX=8154 PE=3 SV=1 |
| 19 | 67134 | tr\|A0A6P7JIX2\|A0A6P7JIX2\_9TELE | 61.93 | 8 | 4 | 2 | N | 43056 | Creatine kinase OS=Parambassis ranga OX=210632 GN=LOC114445771 PE=3 SV=1 |
| 19 | 67716 | tr\|A0A6P7PLI8\|A0A6P7PLI8\_BETSP | 61.93 | 8 | 4 | 2 | N | 42883 | Creatine kinase OS=Betta splendens OX=158456 GN=LOC114869114 PE=3 SV=1 |
| 19 | 67711 | tr\|A0A674PS54\|A0A674PS54\_TAKRU | 61.93 | 8 | 4 | 2 | N | 40422 | Creatine kinase OS=Takifugu rubripes OX=31033 GN=LOC101077669 PE=3 SV=1 |
| 19 | 67401 | tr\|A0A437CN94\|A0A437CN94\_ORYJA | 61.93 | 8 | 4 | 2 | N | 42979 | Creatine kinase OS=Oryzias javanicus OX=123683 GN=OJAV\_G00145090 PE=3 SV=1 |
| 19 | 67715 | tr\|A0A6J2R235\|A0A6J2R235\_COTGO | 61.93 | 8 | 4 | 2 | N | 42867 | Creatine kinase OS=Cottoperca gobio OX=56716 GN=LOC115019392 PE=3 SV=1 |
| 11 | 67157 | tr\|A0A4U5VU95\|A0A4U5VU95\_COLLU | 60.50 | 30 | 6 | 6 | N | 16791 | Myosin regulatory light chain 2, skeletal muscle isoform DTNB OS=Collichthys lucidus OX=240159 GN=D9C73\_025504 PE=4 SV=1 |
| 11 | 67158 | tr\|A0A6P7II88\|A0A6P7II88\_9TELE | 60.50 | 26 | 6 | 6 | N | 18948 | myosin regulatory light chain 2, skeletal muscle isoform OS=Parambassis ranga OX=210632 GN=mylpf PE=4 SV=1 |
| 11 | 67285 | tr\|A0A672MA11\|A0A672MA11\_SINGR | 60.50 | 31 | 6 | 6 | N | 16048 | Myosin regulatory light chain 2, skeletal muscle isoform type 2 OS=Sinocyclocheilus grahami OX=75366 GN=LOC107571443 PE=4 SV=1 |
| 11 | 67287 | tr\|A0A669DZW9\|A0A669DZW9\_ORENI | 60.50 | 28 | 6 | 6 | N | 18203 | Myosin light chain, phosphorylatable, fast skeletal muscle a OS=Oreochromis niloticus OX=8128 PE=4 SV=1 |
| 11 | 67288 | tr\|A0A3B4BW75\|A0A3B4BW75\_PYGNA | 60.50 | 28 | 6 | 6 | N | 18272 | Myosin regulatory light chain 2, skeletal muscle isoform type 2 OS=Pygocentrus nattereri OX=42514 PE=4 SV=1 |
| 11 | 67289 | tr\|I3JU24\|I3JU24\_ORENI | 60.50 | 28 | 6 | 6 | N | 18257 | Myosin light chain, phosphorylatable, fast skeletal muscle a OS=Oreochromis niloticus OX=8128 PE=4 SV=2 |
| 11 | 67295 | tr\|A0A7J6CHK6\|A0A7J6CHK6\_9TELE | 60.50 | 26 | 6 | 6 | N | 19163 | EF-hand domain-containing protein OS=Onychostoma macrolepis OX=369639 GN=G5714\_012740 PE=4 SV=1 |
| 11 | 67298 | tr\|A0A0F8CBC9\|A0A0F8CBC9\_LARCR | 60.50 | 26 | 6 | 6 | N | 19051 | Myosin regulatory light chain 2, skeletal muscle isoform type 2 OS=Larimichthys crocea OX=215358 GN=D5F01\_LYC18898 PE=4 SV=1 |
| 11 | 67305 | tr\|A0A7N8XBQ9\|A0A7N8XBQ9\_9TELE | 60.50 | 23 | 6 | 6 | N | 21687 | Myosin light chain, phosphorylatable, fast skeletal muscle a OS=Mastacembelus armatus OX=205130 PE=4 SV=1 |
| 11 | 67300 | tr\|F8U029\|F8U029\_EPIBR | 60.50 | 26 | 6 | 6 | N | 18965 | Myosin light chain 2 (Fragment) OS=Epinephelus bruneus OX=323802 PE=2 SV=1 |
| 11 | 67308 | tr\|A0A3B5RBY4\|A0A3B5RBY4\_XIPMA | 60.50 | 18 | 6 | 6 | N | 27929 | Uncharacterized protein OS=Xiphophorus maculatus OX=8083 PE=4 SV=1 |
| 28 | 67490 | tr\|G3N515\|G3N515\_GASAC | 57.97 | 1 | 3 | 1 | N | 451257 | Uncharacterized protein OS=Gasterosteus aculeatus OX=69293 PE=4 SV=1 |
| 27 | 67497 | tr\|Q4T444\|Q4T444\_TETNG | 57.72 | 0 | 3 | 1 | N | 1041680 | Chromosome undetermined SCAF9829, whole genome shotgun sequence (Fragment) OS=Tetraodon nigroviridis OX=99883 GN=GSTENG00007498001 PE=4 SV=1 |
| 21 | 67518 | tr\|D2KQG3\|D2KQG3\_SINCH | 56.80 | 12 | 2 | 2 | N | 11617 | Parvalbumin OS=Siniperca chuatsi OX=119488 PE=2 SV=1 |
| 21 | 67519 | tr\|B9VJM3\|B9VJM3\_SINCH | 56.80 | 12 | 2 | 2 | N | 11589 | Parvalbumin OS=Siniperca chuatsi OX=119488 PE=2 SV=1 |
| 18 | 67896 | tr\|A0A3P8TDG8\|A0A3P8TDG8\_AMPPE | 54.14 | 13 | 5 | 1 | N | 21234 | Adenylate kinase isoenzyme 1 OS=Amphiprion percula OX=161767 GN=AK1 PE=3 SV=1 |
| 26 | 67111 | tr\|A0A556TIT9\|A0A556TIT9\_BAGYA | 52.70 | 13 | 3 | 0 | N | 21698 | Parvalbumin OS=Bagarius yarrelli OX=175774 GN=Baya\_0626 PE=3 SV=1 |
| 35 | 67341 | tr\|A0A484CUS5\|A0A484CUS5\_PERFV | 51.89 | 0 | 2 | 1 | N | 867139 | Uncharacterized protein OS=Perca flavescens OX=8167 GN=EPR50\_G00113850 PE=4 SV=1 |
| 23 | 69598 | tr\|A0A2I4CWS8\|A0A2I4CWS8\_9TELE | 46.95 | 2 | 3 | 3 | N | 108703 | Calcium-transporting ATPase OS=Austrofundulus limnaeus OX=52670 GN=LOC106532815 PE=3 SV=1 |
| 23 | 67219 | tr\|A0A3P9C9M1\|A0A3P9C9M1\_9CICH | 46.95 | 2 | 3 | 3 | N | 109087 | Calcium-transporting ATPase OS=Maylandia zebra OX=106582 PE=3 SV=1 |
| 42 | 68221 | tr\|A0A7J6CZM2\|A0A7J6CZM2\_9TELE | 43.01 | 2 | 1 | 1 | N | 53517 | 2-phospho-D-glycerate hydro-lyase OS=Onychostoma macrolepis OX=369639 GN=G5714\_007188 PE=3 SV=1 |
| 32 | 69517 | tr\|A0A0F8AZU0\|A0A0F8AZU0\_LARCR | 39.53 | 1 | 2 | 0 | N | 246883 | Vacuolar fusion protein CCZ1 OS=Larimichthys crocea OX=215358 GN=EH28\_05173 PE=3 SV=1 |
| 36 | 73896 | tr\|A0A4W4GVK8\|A0A4W4GVK8\_ELEEL | 37.84 | 7 | 1 | 1 | N | 16665 | EF-hand domain-containing protein OS=Electrophorus electricus OX=8005 GN=LOC113579190 PE=4 SV=1 |
| 36 | 73897 | tr\|A0A4W4GPW6\|A0A4W4GPW6\_ELEEL | 37.84 | 7 | 1 | 1 | N | 16847 | Uncharacterized protein OS=Electrophorus electricus OX=8005 GN=LOC113579190 PE=4 SV=1 |
| 36 | 73898 | tr\|A0A4W4GW96\|A0A4W4GW96\_ELEEL | 37.84 | 7 | 1 | 1 | N | 16889 | EF-hand domain-containing protein OS=Electrophorus electricus OX=8005 GN=LOC113579190 PE=4 SV=1 |
| 36 | 73926 | tr\|A0A665WHZ7\|A0A665WHZ7\_ECHNA | 37.84 | 8 | 1 | 1 | N | 16102 | Myosin light chain 3, skeletal muscle isoform OS=Echeneis naucrates OX=173247 GN=LOC115051647 PE=4 SV=1 |
| 36 | 73927 | tr\|A0A3B4FQL1\|A0A3B4FQL1\_9CICH | 37.84 | 7 | 1 | 1 | N | 16666 | Myosin light chain 1 OS=Pundamilia nyererei OX=303518 PE=4 SV=1 |
| 36 | 73928 | tr\|A0A3P8PB88\|A0A3P8PB88\_ASTCA | 37.84 | 7 | 1 | 1 | N | 16666 | Myosin light chain 1 OS=Astatotilapia calliptera OX=8154 PE=4 SV=1 |
| 36 | 73929 | tr\|A0A665WHJ1\|A0A665WHJ1\_ECHNA | 37.84 | 7 | 1 | 1 | N | 16702 | Myosin light chain 3, skeletal muscle isoform OS=Echeneis naucrates OX=173247 GN=LOC115051647 PE=4 SV=1 |
| 36 | 73930 | tr\|F2WR27\|F2WR27\_EPICO | 37.84 | 7 | 1 | 1 | N | 16657 | Myosin light chain 3 OS=Epinephelus coioides OX=94232 PE=2 SV=1 |
| 36 | 73931 | tr\|A0A4W6FFD1\|A0A4W6FFD1\_LATCA | 37.84 | 7 | 1 | 1 | N | 16686 | Myosin light chain 3, skeletal muscle isoform OS=Lates calcarifer OX=8187 GN=LOC108873036 PE=4 SV=1 |
| 36 | 73932 | tr\|A0A3B3C1H6\|A0A3B3C1H6\_ORYME | 37.84 | 7 | 1 | 1 | N | 16638 | Myosin light chain 3, skeletal muscle isoform OS=Oryzias melastigma OX=30732 PE=4 SV=1 |
| 36 | 73933 | tr\|A0A3Q1B309\|A0A3Q1B309\_AMPOC | 37.84 | 7 | 1 | 1 | N | 16588 | Myosin light chain 3, skeletal muscle isoform OS=Amphiprion ocellaris OX=80972 PE=4 SV=1 |
| 36 | 73934 | tr\|A0A3B4VIP0\|A0A3B4VIP0\_SERDU | 37.84 | 7 | 1 | 1 | N | 17424 | Myosin light chain 3, skeletal muscle isoform OS=Seriola dumerili OX=41447 PE=4 SV=1 |
| 36 | 73935 | tr\|A0A3B5RBB9\|A0A3B5RBB9\_XIPMA | 37.84 | 7 | 1 | 1 | N | 17895 | Uncharacterized protein OS=Xiphophorus maculatus OX=8083 PE=4 SV=1 |
| 36 | 73936 | tr\|A0A3B5R1L0\|A0A3B5R1L0\_XIPMA | 37.84 | 7 | 1 | 1 | N | 17816 | EF-hand domain-containing protein OS=Xiphophorus maculatus OX=8083 PE=4 SV=1 |
| 36 | 73937 | tr\|A0A3P9CCX0\|A0A3P9CCX0\_9CICH | 37.84 | 7 | 1 | 1 | N | 17897 | Myosin light chain 1 OS=Maylandia zebra OX=106582 PE=4 SV=1 |
| 36 | 73938 | tr\|A0A665WHZ4\|A0A665WHZ4\_ECHNA | 37.84 | 7 | 1 | 1 | N | 18047 | Myosin light chain 3, skeletal muscle isoform OS=Echeneis naucrates OX=173247 GN=LOC115051647 PE=4 SV=1 |
| 36 | 73939 | tr\|A0A4W6FCG2\|A0A4W6FCG2\_LATCA | 37.84 | 7 | 1 | 1 | N | 18787 | Myosin light chain 3, skeletal muscle isoform OS=Lates calcarifer OX=8187 GN=LOC108873036 PE=4 SV=1 |
| 36 | 73940 | tr\|A0A4W6FFD6\|A0A4W6FFD6\_LATCA | 37.84 | 6 | 1 | 1 | N | 18836 | Myosin light chain 3, skeletal muscle isoform OS=Lates calcarifer OX=8187 GN=LOC108873036 PE=4 SV=1 |
| 36 | 73941 | tr\|A0A665WI19\|A0A665WI19\_ECHNA | 37.84 | 6 | 1 | 1 | N | 18811 | Myosin light chain 3, skeletal muscle isoform OS=Echeneis naucrates OX=173247 GN=LOC115051647 PE=4 SV=1 |
| 36 | 73942 | tr\|B9V2Y1\|B9V2Y1\_EPICO | 37.84 | 6 | 1 | 1 | N | 19048 | Myosin light chain 3 (Fragment) OS=Epinephelus coioides OX=94232 PE=2 SV=1 |
| 36 | 73943 | tr\|A0A4W6FDH6\|A0A4W6FDH6\_LATCA | 37.84 | 6 | 1 | 1 | N | 20136 | Myosin light chain 3, skeletal muscle isoform OS=Lates calcarifer OX=8187 GN=LOC108873036 PE=4 SV=1 |
| 36 | 73944 | tr\|A0A669EYH8\|A0A669EYH8\_ORENI | 37.84 | 5 | 1 | 1 | N | 23743 | Uncharacterized protein OS=Oreochromis niloticus OX=8128 GN=LOC100698429 PE=4 SV=1 |
| 36 | 73945 | tr\|A0A3B4VK07\|A0A3B4VK07\_SERDU | 37.84 | 5 | 1 | 1 | N | 24449 | Myosin light chain 3, skeletal muscle isoform OS=Seriola dumerili OX=41447 PE=4 SV=1 |
| 36 | 73946 | tr\|A0A3B4VK38\|A0A3B4VK38\_SERDU | 37.84 | 5 | 1 | 1 | N | 25360 | Myosin light chain 3, skeletal muscle isoform OS=Seriola dumerili OX=41447 PE=4 SV=1 |
| 36 | 73947 | tr\|A0A834C4M6\|A0A834C4M6\_ORYME | 37.84 | 5 | 1 | 1 | N | 26998 | Myosin light chain 3, skeletal muscle isoform OS=Oryzias melastigma OX=30732 GN=FQA47\_014212 PE=4 SV=1 |
| 36 | 73948 | tr\|A0A315W7Q8\|A0A315W7Q8\_GAMAF | 37.84 | 5 | 1 | 1 | N | 26671 | Uncharacterized protein OS=Gambusia affinis OX=33528 GN=CCH79\_00013367 PE=4 SV=1 |
| 36 | 73958 | tr\|A0A672K127\|A0A672K127\_SINGR | 37.84 | 9 | 1 | 1 | N | 12988 | Myosin light chain 3, skeletal muscle isoform-like OS=Sinocyclocheilus grahami OX=75366 GN=LOC107579269 PE=4 SV=1 |
| 36 | 73959 | tr\|A0A7J6DIX6\|A0A7J6DIX6\_9TELE | 37.84 | 7 | 1 | 1 | N | 16756 | Uncharacterized protein OS=Onychostoma macrolepis OX=369639 GN=G5714\_001122 PE=4 SV=1 |
| 36 | 73960 | tr\|A0A4W5MNE5\|A0A4W5MNE5\_9TELE | 37.84 | 7 | 1 | 1 | N | 17699 | Myosin, light chain 1, alkali; skeletal, fast OS=Hucho hucho OX=62062 PE=4 SV=1 |
| 36 | 73961 | tr\|A0A4W5MRK6\|A0A4W5MRK6\_9TELE | 37.84 | 7 | 1 | 1 | N | 18798 | Myosin, light chain 1, alkali; skeletal, fast OS=Hucho hucho OX=62062 PE=4 SV=1 |
| 36 | 73962 | tr\|A0A4W5MMD2\|A0A4W5MMD2\_9TELE | 37.84 | 6 | 1 | 1 | N | 20159 | Myosin, light chain 1, alkali; skeletal, fast OS=Hucho hucho OX=62062 PE=4 SV=1 |
| 36 | 73963 | tr\|A0A3Q3JIG8\|A0A3Q3JIG8\_MONAL | 37.84 | 6 | 1 | 1 | N | 20937 | Myosin light chain 1, skeletal muscle isoform-like OS=Monopterus albus OX=43700 PE=4 SV=1 |
| 36 | 73964 | tr\|Q90332\|Q90332\_CYPCA | 37.84 | 6 | 1 | 1 | N | 21134 | Fast skeletal myosin light chain 1b OS=Cyprinus carpio OX=7962 PE=2 SV=1 |
| 36 | 73965 | tr\|Q90WC7\|Q90WC7\_ONCMY | 37.84 | 6 | 1 | 1 | N | 21057 | Myosin light chain 1 OS=Oncorhynchus mykiss OX=8022 PE=2 SV=1 |
| 36 | 72069 | tr\|A0A7J6CQG1\|A0A7J6CQG1\_9TELE | 37.84 | 6 | 1 | 1 | N | 21007 | Uncharacterized protein OS=Onychostoma macrolepis OX=369639 GN=G5714\_009908 PE=4 SV=1 |
| 36 | 72070 | tr\|Q90W52\|Q90W52\_PSEDN | 37.84 | 6 | 1 | 1 | N | 20741 | Myosin light chain 1 OS=Pseudocaranx dentex OX=349646 GN=mlc1 PE=2 SV=1 |
| 36 | 73966 | tr\|Q90W46\|Q90W46\_DECMR | 37.84 | 6 | 1 | 1 | N | 21098 | Myosin light chain 1 OS=Decapterus macrosoma OX=166264 GN=mlc1 PE=2 SV=1 |
| 36 | 73967 | tr\|A0A4W5MQC0\|A0A4W5MQC0\_9TELE | 37.84 | 5 | 1 | 1 | N | 22406 | Myosin, light chain 1, alkali; skeletal, fast OS=Hucho hucho OX=62062 PE=4 SV=1 |
| 36 | 73970 | tr\|A0A0P7TY12\|A0A0P7TY12\_SCLFO | 37.84 | 7 | 1 | 1 | N | 19172 | Myosin light chain 3 skeletal muscle-like (Fragment) OS=Scleropages formosus OX=113540 GN=Z043\_115197 PE=4 SV=1 |
| 36 | 73979 | tr\|A0A672GTD6\|A0A672GTD6\_SALFA | 37.84 | 7 | 1 | 1 | N | 16528 | Myosin, light polypeptide 3, skeletal muscle OS=Salarias fasciatus OX=181472 PE=4 SV=1 |
| 36 | 73980 | tr\|A0A3P8V7S6\|A0A3P8V7S6\_CYNSE | 37.84 | 7 | 1 | 1 | N | 16686 | Myosin light chain 3, skeletal muscle isoform OS=Cynoglossus semilaevis OX=244447 PE=4 SV=1 |
| 36 | 73981 | tr\|A0A671WWZ3\|A0A671WWZ3\_SPAAU | 37.84 | 7 | 1 | 1 | N | 16973 | Myosin light chain 3, skeletal muscle isoform OS=Sparus aurata OX=8175 GN=LOC115577181 PE=4 SV=1 |
| 36 | 73982 | tr\|A0A672GWW2\|A0A672GWW2\_SALFA | 37.84 | 7 | 1 | 1 | N | 17237 | Myosin, light polypeptide 3, skeletal muscle OS=Salarias fasciatus OX=181472 PE=4 SV=1 |
| 36 | 73983 | tr\|A0A671X3G3\|A0A671X3G3\_SPAAU | 37.84 | 7 | 1 | 1 | N | 17479 | Myosin light chain 3, skeletal muscle isoform OS=Sparus aurata OX=8175 GN=LOC115577181 PE=4 SV=1 |
| 36 | 73984 | tr\|A0A672H327\|A0A672H327\_SALFA | 37.84 | 7 | 1 | 1 | N | 17490 | Myosin, light polypeptide 3, skeletal muscle OS=Salarias fasciatus OX=181472 PE=4 SV=1 |
| 36 | 73985 | tr\|A0A7N8Y8C7\|A0A7N8Y8C7\_9TELE | 37.84 | 7 | 1 | 1 | N | 17841 | Myosin, light polypeptide 3, skeletal muscle OS=Mastacembelus armatus OX=205130 PE=4 SV=1 |
| 36 | 73986 | tr\|A0A672GRY1\|A0A672GRY1\_SALFA | 37.84 | 7 | 1 | 1 | N | 17938 | Myosin, light polypeptide 3, skeletal muscle OS=Salarias fasciatus OX=181472 PE=4 SV=1 |
| 36 | 73987 | tr\|A0A3Q2XPR8\|A0A3Q2XPR8\_HIPCM | 37.84 | 6 | 1 | 1 | N | 19433 | Myosin light chain 1, skeletal muscle isoform OS=Hippocampus comes OX=109280 PE=4 SV=1 |
| 36 | 73988 | tr\|A0A6J2QQE0\|A0A6J2QQE0\_COTGO | 37.84 | 6 | 1 | 1 | N | 20126 | LOW QUALITY PROTEIN: myosin light chain 3, skeletal muscle isoform-like OS=Cottoperca gobio OX=56716 GN=LOC115016756 PE=4 SV=1 |
| 36 | 73989 | tr\|A0A3Q3D9S9\|A0A3Q3D9S9\_HIPCM | 37.84 | 5 | 1 | 1 | N | 22021 | Myosin light chain 1, skeletal muscle isoform OS=Hippocampus comes OX=109280 PE=4 SV=1 |
| 29 | 67640 | tr\|A0A4Z2CI05\|A0A4Z2CI05\_9TELE | 35.86 | 0 | 2 | 0 | N | 507153 | Uncharacterized protein OS=Takifugu bimaculatus OX=433685 GN=fugu\_000743 PE=4 SV=1 |
| 30 | 67005 | tr\|Q4RE90\|Q4RE90\_TETNG | 35.57 | 0 | 2 | 1 | N | 863297 | Chromosome undetermined SCAF15134, whole genome shotgun sequence (Fragment) OS=Tetraodon nigroviridis OX=99883 GN=GSTENG00035871001 PE=4 SV=1 |
| 33 | 67404 | tr\|A0A6F9CEX9\|A0A6F9CEX9\_9TELE | 35.33 | 0 | 2 | 1 | N | 1022296 | Uncharacterized protein OS=Coregonus sp. 'balchen' OX=861768 GN=CSTEINMANNI\_LOCUS3957823 PE=4 SV=1 |
| 33 | 67667 | tr\|A0A060W3X5\|A0A060W3X5\_ONCMY | 35.33 | 1 | 2 | 1 | N | 453353 | Uncharacterized protein OS=Oncorhynchus mykiss OX=8022 GN=GSONMT00066266001 PE=4 SV=1 |
| 34 | 67431 | tr\|A0A6P3W3B1\|A0A6P3W3B1\_CLUHA | 31.61 | 3 | 2 | 2 | N | 47370 | 2-phospho-D-glycerate hydro-lyase OS=Clupea harengus OX=7950 GN=LOC105904565 PE=3 SV=1 |
| 34 | 67043 | tr\|F5BZS7\|F5BZS7\_EPIBR | 31.61 | 3 | 2 | 2 | N | 43226 | 2-phospho-D-glycerate hydro-lyase (Fragment) OS=Epinephelus bruneus OX=323802 PE=2 SV=1 |
| 34 | 71851 | tr\|A0A668V8T6\|A0A668V8T6\_OREAU | 31.61 | 3 | 2 | 2 | N | 47001 | 2-phospho-D-glycerate hydro-lyase OS=Oreochromis aureus OX=47969 PE=3 SV=1 |
| 34 | 67430 | tr\|A0A4W4FGS3\|A0A4W4FGS3\_ELEEL | 31.61 | 3 | 2 | 2 | N | 47188 | 2-phospho-D-glycerate hydro-lyase OS=Electrophorus electricus OX=8005 GN=LOC113568057 PE=3 SV=1 |
| 34 | 67034 | tr\|A0A3B4CPC9\|A0A3B4CPC9\_PYGNA | 31.61 | 3 | 2 | 2 | N | 47482 | 2-phospho-D-glycerate hydro-lyase OS=Pygocentrus nattereri OX=42514 PE=3 SV=1 |
| 34 | 67421 | tr\|A0A3P8TVY9\|A0A3P8TVY9\_AMPPE | 31.61 | 3 | 2 | 2 | N | 47574 | 2-phospho-D-glycerate hydro-lyase OS=Amphiprion percula OX=161767 PE=3 SV=1 |
| 34 | 67419 | tr\|A0A3P8NTT0\|A0A3P8NTT0\_ASTCA | 31.61 | 2 | 2 | 2 | N | 48687 | 2-phospho-D-glycerate hydro-lyase OS=Astatotilapia calliptera OX=8154 PE=3 SV=1 |
| 43 | 67539 | tr\|A0A7J6BP23\|A0A7J6BP23\_9TELE | 29.88 | 12 | 1 | 1 | N | 13461 | Uncharacterized protein OS=Onychostoma macrolepis OX=369639 GN=G5714\_022729 PE=4 SV=1 |
| 43 | 67166 | tr\|A0A4Z2FMJ8\|A0A4Z2FMJ8\_9TELE | 29.88 | 11 | 1 | 1 | N | 14750 | Troponin C, skeletal muscle OS=Liparis tanakae OX=230148 GN=TNNC2 PE=4 SV=1 |
| 43 | 67020 | tr\|A0A1A8D9W7\|A0A1A8D9W7\_9TELE | 29.88 | 10 | 1 | 1 | N | 15353 | Troponin C type 2 (Fast) OS=Nothobranchius kadleci OX=1051664 GN=TNNC2 PE=4 SV=1 |
| 43 | 67540 | tr\|A0A6P3VH61\|A0A6P3VH61\_CLUHA | 29.88 | 10 | 1 | 1 | N | 16370 | troponin C, skeletal muscle OS=Clupea harengus OX=7950 GN=LOC105890625 PE=4 SV=1 |
| 43 | 67167 | tr\|A0A3B4CI86\|A0A3B4CI86\_PYGNA | 29.88 | 10 | 1 | 1 | N | 16455 | Troponin C type 2 (fast) OS=Pygocentrus nattereri OX=42514 PE=4 SV=1 |
| 43 | 67168 | tr\|A0A3B4E1V9\|A0A3B4E1V9\_PYGNA | 29.88 | 9 | 1 | 1 | N | 17769 | Troponin C type 2 (fast) OS=Pygocentrus nattereri OX=42514 PE=4 SV=1 |
| 43 | 67170 | tr\|A0A6J2Q142\|A0A6J2Q142\_COTGO | 29.88 | 9 | 1 | 1 | N | 18257 | troponin C, skeletal muscle OS=Cottoperca gobio OX=56716 GN=LOC115011252 PE=4 SV=1 |
| 43 | 67171 | tr\|A0A3B4E4D7\|A0A3B4E4D7\_PYGNA | 29.88 | 9 | 1 | 1 | N | 18081 | Troponin C, skeletal muscle OS=Pygocentrus nattereri OX=42514 PE=4 SV=1 |
| 43 | 67172 | tr\|H2SBN2\|H2SBN2\_TAKRU | 29.88 | 9 | 1 | 1 | N | 18200 | Uncharacterized protein OS=Takifugu rubripes OX=31033 GN=LOC101074449 PE=4 SV=2 |
| 43 | 67174 | tr\|A0A2R8QMW7\|A0A2R8QMW7\_DANRE | 29.88 | 9 | 1 | 1 | N | 18043 | Si:rp71-17i16.4 OS=Danio rerio OX=7955 GN=si:rp71-17i16.4 PE=4 SV=1 |
| 43 | 67175 | tr\|A0A4W4EUJ0\|A0A4W4EUJ0\_ELEEL | 29.88 | 9 | 1 | 1 | N | 18156 | Uncharacterized protein OS=Electrophorus electricus OX=8005 GN=LOC113569977 PE=4 SV=1 |
| 43 | 67176 | tr\|A0A3Q3G540\|A0A3Q3G540\_9LABR | 29.88 | 9 | 1 | 1 | N | 18223 | Troponin C, skeletal muscle-like OS=Labrus bergylta OX=56723 PE=4 SV=1 |
| 43 | 67030 | tr\|G3NJM6\|G3NJM6\_GASAC | 29.88 | 9 | 1 | 1 | N | 18300 | Troponin C type 2 (fast) OS=Gasterosteus aculeatus OX=69293 PE=4 SV=1 |
| 43 | 66968 | tr\|W5UMX1\|W5UMX1\_ICTPU | 29.88 | 9 | 1 | 1 | N | 18231 | Troponin C, skeletal muscle OS=Ictalurus punctatus OX=7998 GN=TNNC2 PE=2 SV=1 |
| 43 | 67178 | tr\|A0A3P8P1R5\|A0A3P8P1R5\_ASTCA | 29.88 | 9 | 1 | 1 | N | 18230 | Troponin C, skeletal muscle OS=Astatotilapia calliptera OX=8154 PE=4 SV=1 |
| 43 | 67179 | tr\|A0A4W4EW29\|A0A4W4EW29\_ELEEL | 29.88 | 9 | 1 | 1 | N | 18171 | Uncharacterized protein OS=Electrophorus electricus OX=8005 GN=LOC113569977 PE=4 SV=1 |
| 43 | 67180 | tr\|A0A6P7IM54\|A0A6P7IM54\_9TELE | 29.88 | 9 | 1 | 1 | N | 18225 | troponin C, skeletal muscle OS=Parambassis ranga OX=210632 GN=LOC114439031 PE=4 SV=1 |
| 43 | 67181 | tr\|A0A4W4EVB1\|A0A4W4EVB1\_ELEEL | 29.88 | 9 | 1 | 1 | N | 18179 | Uncharacterized protein OS=Electrophorus electricus OX=8005 GN=LOC113569977 PE=4 SV=1 |
| 43 | 67182 | tr\|I3IZI3\|I3IZI3\_ORENI | 29.88 | 9 | 1 | 1 | N | 18271 | Uncharacterized protein OS=Oreochromis niloticus OX=8128 GN=LOC100703845 PE=4 SV=2 |
| 43 | 67183 | tr\|A0A3Q1CAA4\|A0A3Q1CAA4\_AMPOC | 29.88 | 9 | 1 | 1 | N | 18190 | Troponin C, skeletal muscle OS=Amphiprion ocellaris OX=80972 PE=4 SV=1 |
| 43 | 67184 | tr\|A0A3Q3G8Q9\|A0A3Q3G8Q9\_9LABR | 29.88 | 9 | 1 | 1 | N | 18220 | Troponin C, skeletal muscle-like OS=Labrus bergylta OX=56723 PE=4 SV=1 |
| 43 | 67541 | tr\|A0A4W5NYP8\|A0A4W5NYP8\_9TELE | 29.88 | 9 | 1 | 1 | N | 18187 | Troponin C type 2 (fast) OS=Hucho hucho OX=62062 PE=4 SV=1 |
| 43 | 67185 | tr\|A0A672J300\|A0A672J300\_SALFA | 29.88 | 9 | 1 | 1 | N | 18135 | Troponin C type 2 (fast) OS=Salarias fasciatus OX=181472 PE=4 SV=1 |
| 43 | 67187 | tr\|A0A3Q2W3W0\|A0A3Q2W3W0\_HAPBU | 29.88 | 9 | 1 | 1 | N | 18305 | Troponin C, skeletal muscle OS=Haplochromis burtoni OX=8153 PE=4 SV=1 |
| 43 | 67542 | tr\|A0A3Q2WC94\|A0A3Q2WC94\_HAPBU | 29.88 | 8 | 1 | 1 | N | 19498 | Troponin C, skeletal muscle-like OS=Haplochromis burtoni OX=8153 PE=4 SV=1 |
| 43 | 67188 | tr\|A0A669E7L6\|A0A669E7L6\_ORENI | 29.88 | 8 | 1 | 1 | N | 19478 | Uncharacterized protein OS=Oreochromis niloticus OX=8128 GN=LOC100703845 PE=4 SV=1 |
| 43 | 67189 | tr\|A0A3B3TEJ6\|A0A3B3TEJ6\_9TELE | 29.88 | 8 | 1 | 1 | N | 19607 | Troponin C, skeletal muscle OS=Paramormyrops kingsleyae OX=1676925 PE=4 SV=1 |
| 43 | 67192 | tr\|A0A3P8P1N2\|A0A3P8P1N2\_ASTCA | 29.88 | 8 | 1 | 1 | N | 20987 | Troponin C, skeletal muscle OS=Astatotilapia calliptera OX=8154 PE=4 SV=1 |
| 43 | 67194 | tr\|A0A3P9N7Z1\|A0A3P9N7Z1\_POERE | 29.88 | 6 | 1 | 1 | N | 24749 | Troponin C, skeletal muscle OS=Poecilia reticulata OX=8081 PE=4 SV=1 |
| 43 | 67195 | tr\|A0A6F9C7M8\|A0A6F9C7M8\_9TELE | 29.88 | 4 | 1 | 1 | N | 42179 | Uncharacterized protein OS=Coregonus sp. 'balchen' OX=861768 GN=CSTEINMANNI\_LOCUS3751028 PE=4 SV=1 |
| 44 | 74035 | tr\|A0A315VUM5\|A0A315VUM5\_GAMAF | 24.10 | 1 | 1 | 1 | N | 199740 | Uncharacterized protein OS=Gambusia affinis OX=33528 GN=CCH79\_00015210 PE=4 SV=1 |
| 45 | 74042 | tr\|A0A3Q0S4A7\|A0A3Q0S4A7\_AMPCI | 23.39 | 1 | 1 | 1 | N | 104043 | Ankyrin repeat and FYVE domain containing 1 OS=Amphilophus citrinellus OX=61819 PE=4 SV=1 |
| 45 | 74043 | tr\|A0A3B4V2S1\|A0A3B4V2S1\_SERDU | 23.39 | 1 | 1 | 1 | N | 104725 | Ankyrin repeat and FYVE domain containing 1 OS=Seriola dumerili OX=41447 PE=4 SV=1 |
| 45 | 74044 | tr\|A0A4W4FZE0\|A0A4W4FZE0\_ELEEL | 23.39 | 1 | 1 | 1 | N | 114557 | FYVE-type domain-containing protein OS=Electrophorus electricus OX=8005 GN=ankfy1 PE=4 SV=1 |
| 45 | 74045 | tr\|A0A1A8RP46\|A0A1A8RP46\_9TELE | 23.39 | 1 | 1 | 1 | N | 115767 | Ankyrin repeat and FYVE domain containing 1 OS=Nothobranchius rachovii OX=451742 GN=ANKFY1 PE=4 SV=1 |
| 45 | 74046 | tr\|A0A6Q2ZPR8\|A0A6Q2ZPR8\_ESOLU | 23.39 | 1 | 1 | 1 | N | 118078 | Ankyrin repeat and FYVE domain containing 1 OS=Esox lucius OX=8010 PE=4 SV=1 |
| 45 | 74047 | tr\|A0A674AQG9\|A0A674AQG9\_SALTR | 23.39 | 1 | 1 | 1 | N | 121601 | Rabankyrin-5-like OS=Salmo trutta OX=8032 GN=LOC115153911 PE=4 SV=1 |
| 45 | 74048 | tr\|A0A3P8T5S7\|A0A3P8T5S7\_AMPPE | 23.39 | 1 | 1 | 1 | N | 125156 | Ankyrin repeat and FYVE domain containing 1 OS=Amphiprion percula OX=161767 PE=4 SV=1 |
| 45 | 74049 | tr\|A0A4W4FZ81\|A0A4W4FZ81\_ELEEL | 23.39 | 1 | 1 | 1 | N | 126038 | Uncharacterized protein OS=Electrophorus electricus OX=8005 GN=ankfy1 PE=4 SV=1 |
| 45 | 74050 | tr\|A0A4W5QGX5\|A0A4W5QGX5\_9TELE | 23.39 | 1 | 1 | 1 | N | 125662 | Ankyrin repeat and FYVE domain containing 1 OS=Hucho hucho OX=62062 PE=4 SV=1 |
| 45 | 74051 | tr\|A0A3B3DDJ3\|A0A3B3DDJ3\_ORYME | 23.39 | 1 | 1 | 1 | N | 126170 | Ankyrin repeat and FYVE domain containing 1 OS=Oryzias melastigma OX=30732 PE=4 SV=1 |
| 45 | 74052 | tr\|A0A4W4FW94\|A0A4W4FW94\_ELEEL | 23.39 | 1 | 1 | 1 | N | 126337 | Uncharacterized protein OS=Electrophorus electricus OX=8005 GN=ankfy1 PE=4 SV=1 |
| 45 | 74053 | tr\|A0A674AP52\|A0A674AP52\_SALTR | 23.39 | 1 | 1 | 1 | N | 126358 | Rabankyrin-5-like OS=Salmo trutta OX=8032 GN=LOC115153911 PE=4 SV=1 |
| 45 | 74054 | tr\|A0A834KWF7\|A0A834KWF7\_ORYME | 23.39 | 1 | 1 | 1 | N | 127178 | Rabankyrin-5 OS=Oryzias melastigma OX=30732 GN=FQA47\_010782 PE=4 SV=1 |
| 45 | 74055 | tr\|A0A667ZHJ6\|A0A667ZHJ6\_9TELE | 23.39 | 1 | 1 | 1 | N | 127368 | Ankyrin repeat and FYVE domain containing 1 OS=Myripristis murdjan OX=586833 GN=ankfy1 PE=4 SV=1 |
| 45 | 74056 | tr\|A0A3B4DWJ2\|A0A3B4DWJ2\_PYGNA | 23.39 | 1 | 1 | 1 | N | 127828 | Ankyrin repeat and FYVE domain containing 1 OS=Pygocentrus nattereri OX=42514 PE=4 SV=1 |
| 45 | 74057 | tr\|A0A3Q2XWS4\|A0A3Q2XWS4\_HIPCM | 23.39 | 1 | 1 | 1 | N | 127881 | Ankyrin repeat and FYVE domain containing 1 OS=Hippocampus comes OX=109280 PE=4 SV=1 |
| 45 | 74058 | tr\|A0A667ZHH4\|A0A667ZHH4\_9TELE | 23.39 | 1 | 1 | 1 | N | 127517 | Ankyrin repeat and FYVE domain containing 1 OS=Myripristis murdjan OX=586833 GN=ankfy1 PE=4 SV=1 |
| 45 | 74059 | tr\|A0A3Q3G3V8\|A0A3Q3G3V8\_KRYMA | 23.39 | 1 | 1 | 1 | N | 127886 | Ankyrin repeat and FYVE domain containing 1 OS=Kryptolebias marmoratus OX=37003 PE=4 SV=1 |
| 45 | 74060 | tr\|A0A3P8R774\|A0A3P8R774\_ASTCA | 23.39 | 1 | 1 | 1 | N | 128068 | Ankyrin repeat and FYVE domain containing 1 OS=Astatotilapia calliptera OX=8154 PE=4 SV=1 |
| 45 | 74061 | tr\|A0A484CLN2\|A0A484CLN2\_PERFV | 23.39 | 1 | 1 | 1 | N | 127679 | Uncharacterized protein OS=Perca flavescens OX=8167 GN=EPR50\_G00135050 PE=4 SV=1 |
| 45 | 74062 | tr\|A0A4W4FZM1\|A0A4W4FZM1\_ELEEL | 23.39 | 1 | 1 | 1 | N | 128150 | Uncharacterized protein OS=Electrophorus electricus OX=8005 GN=ankfy1 PE=4 SV=1 |
| 45 | 74063 | tr\|A0A4W5M1U7\|A0A4W5M1U7\_9TELE | 23.39 | 1 | 1 | 1 | N | 127954 | Ankyrin repeat and FYVE domain containing 1 OS=Hucho hucho OX=62062 PE=4 SV=1 |
| 45 | 74064 | tr\|A0A674ARJ6\|A0A674ARJ6\_SALTR | 23.39 | 1 | 1 | 1 | N | 128040 | Rabankyrin-5-like OS=Salmo trutta OX=8032 GN=LOC115153911 PE=4 SV=1 |
| 45 | 74065 | tr\|A0A4W4FZR2\|A0A4W4FZR2\_ELEEL | 23.39 | 1 | 1 | 1 | N | 128360 | Uncharacterized protein OS=Electrophorus electricus OX=8005 GN=ankfy1 PE=4 SV=1 |
| 45 | 74066 | tr\|A0A4W5M1X7\|A0A4W5M1X7\_9TELE | 23.39 | 1 | 1 | 1 | N | 128147 | Ankyrin repeat and FYVE domain containing 1 OS=Hucho hucho OX=62062 PE=4 SV=1 |
| 45 | 74067 | tr\|A0A3Q3Q182\|A0A3Q3Q182\_MONAL | 23.39 | 1 | 1 | 1 | N | 128884 | Ankyrin repeat and FYVE domain containing 1 OS=Monopterus albus OX=43700 PE=4 SV=1 |
| 45 | 74068 | tr\|A0A4W5QMX9\|A0A4W5QMX9\_9TELE | 23.39 | 1 | 1 | 1 | N | 128910 | Ankyrin repeat and FYVE domain containing 1 OS=Hucho hucho OX=62062 PE=4 SV=1 |
| 45 | 74069 | tr\|A0A673Z592\|A0A673Z592\_SALTR | 23.39 | 1 | 1 | 1 | N | 129266 | Rabankyrin-5-like OS=Salmo trutta OX=8032 GN=LOC115148449 PE=4 SV=1 |
| 45 | 74070 | tr\|A0A5N5JWC6\|A0A5N5JWC6\_PANHP | 23.39 | 1 | 1 | 1 | N | 131933 | Uncharacterized protein OS=Pangasianodon hypophthalmus OX=310915 GN=PHYPO\_G00159810 PE=4 SV=1 |
| 45 | 74071 | tr\|A0A4W4FWH2\|A0A4W4FWH2\_ELEEL | 23.39 | 1 | 1 | 1 | N | 132613 | Uncharacterized protein OS=Electrophorus electricus OX=8005 GN=ankfy1 PE=4 SV=1 |
| 46 | 74072 | tr\|A0A2D0QGH0\|A0A2D0QGH0\_ICTPU | 22.34 | 0 | 1 | 1 | N | 391648 | lysosomal-trafficking regulator isoform X5 OS=Ictalurus punctatus OX=7998 GN=lyst PE=4 SV=1 |
| 46 | 74073 | tr\|A0A2D0QIP4\|A0A2D0QIP4\_ICTPU | 22.34 | 0 | 1 | 1 | N | 425932 | lysosomal-trafficking regulator isoform X3 OS=Ictalurus punctatus OX=7998 GN=lyst PE=4 SV=1 |
| 46 | 74074 | tr\|A0A2D0QFG9\|A0A2D0QFG9\_ICTPU | 22.34 | 0 | 1 | 1 | N | 428327 | lysosomal-trafficking regulator isoform X1 OS=Ictalurus punctatus OX=7998 GN=lyst PE=4 SV=1 |
| 47 | 74075 | tr\|A0EXC6\|A0EXC6\_DANRE | 21.77 | 3 | 1 | 1 | N | 33478 | Sulfotransferase OS=Danio rerio OX=7955 GN=sult2st3 PE=2 SV=1 |
| 47 | 74076 | tr\|A4FUP0\|A4FUP0\_DANRE | 21.77 | 3 | 1 | 1 | N | 33460 | Sulfotransferase OS=Danio rerio OX=7955 GN=sult2st3 PE=1 SV=1 |
| 37 | 68305 | tr\|A0A4V6ASI9\|A0A4V6ASI9\_COLLU | 20.00 | 1 | 1 | 1 | N | 126874 | Collagen alpha-2(I) chain OS=Collichthys lucidus OX=240159 GN=D9C73\_023018 PE=4 SV=1 |
| 37 | 71897 | tr\|A0A5J6XX13\|A0A5J6XX13\_NIBCO | 20.00 | 1 | 1 | 1 | N | 126736 | Collagen type I alpha 2 chain OS=Nibea coibor OX=396775 GN=COL1A2 PE=2 SV=1 |
| total 256 proteins |  |  |  |  |  |  |  |  |  |
| --- | --- | --- | --- | --- | --- | --- | --- | --- | --- |

  

tr|A0A3S2MUF9|A0A3S2MUF9\_ORYJA

back to list

  

| Protein Coverage
| Supporting Peptides
|

Protein Coverage:

Supporting Peptides:

| Peptide | Uniq | -10lgP | Mass | ppm | m/z | z | RT | Scan | #Spec | Start | End | PTM |
| --- | --- | --- | --- | --- | --- | --- | --- | --- | --- | --- | --- | --- |
| E.KSYELPDGQVITIGNE.R | Y | 52.22 | 1761.8784 | -0.6 | 881.9460 | 2 | 13.41 | 1921 | 1 | 240 | 255 |  |
| S.LEKSYELPDGQVITI.G | Y | 50.90 | 1703.8981 | 0.2 | 852.9565 | 2 | 13.97 | 2015 | 1 | 238 | 252 |  |
| E.KSYELPDGQVITIG.N | Y | 37.16 | 1518.7930 | -0.3 | 760.4035 | 2 | 13.49 | 1935 | 2 | 240 | 253 |  |
| L.EKSYELPDGQVIT.I | Y | 35.16 | 1477.7300 | -4.0 | 739.8693 | 2 | 12.16 | 1702 | 1 | 239 | 251 |  |
| A.GFAGDDAPRA.V | Y | 33.15 | 975.4410 | 2.2 | 488.7289 | 2 | 10.28 | 1386 | 1 | 22 | 31 |  |
| K.SYELPDGQVITIG.N | Y | 31.23 | 1390.6980 | 0.0 | 696.3563 | 2 | 14.16 | 2047 | 1 | 241 | 253 |  |
| K.ANREKMTQ.I | Y | 28.79 | 976.4760 | 0.2 | 489.2454 | 2 | 8.23 | 1140 | 1 | 116 | 123 |  |
| S.LEKSYELPDGQVITIG.N | Y | 27.54 | 1760.9196 | -0.4 | 881.4667 | 2 | 13.69 | 1969 | 1 | 238 | 253 |  |
| L.DAGDGVTH.N | Y | 25.64 | 770.3195 | -0.1 | 386.1670 | 2 | 8.68 | 1226 | 7 | 156 | 163 |  |
| G.IITNWDDMEK.I | Y | 23.28 | 1263.5806 | 5.9 | 632.8013 | 2 | 12.12 | 1695 | 1 | 77 | 86 |  |
| E.KSYELPDGQ.V | Y | 22.22 | 1035.4873 | 2.3 | 518.7521 | 2 | 10.74 | 1457 | 1 | 240 | 248 |  |
| S.LEKSYELPDGQ.V | Y | 21.06 | 1277.6139 | -0.6 | 639.8138 | 2 | 11.19 | 1533 | 1 | 238 | 248 |  |
| S.LEKSYELPDGQVI.T | Y | 20.25 | 1489.7664 | -0.7 | 745.8900 | 2 | 12.83 | 1820 | 1 | 238 | 250 |  |
| K.SYELPDGQVIT.I | Y | 19.84 | 1220.5924 | -1.2 | 611.3027 | 2 | 13.08 | 1863 | 1 | 241 | 251 |  |
| K.SYELPDGQVITI.G | Y | 19.30 | 1333.6765 | -1.6 | 667.8445 | 2 | 14.45 | 2096 | 1 | 241 | 252 |  |
| G.FAGDDAPRA.V | Y | 18.98 | 918.4195 | 1.4 | 460.2177 | 2 | 15.22 | 2229 | 11 | 23 | 31 |  |
| E.KSYELPDGQV.I | Y | 18.67 | 1134.5557 | 0.0 | 568.2851 | 2 | 11.34 | 1560 | 1 | 240 | 249 |  |
| S.LEKSYELPDGQVIT.I | Y | 18.03 | 1590.8141 | -0.8 | 796.4137 | 2 | 12.31 | 1729 | 1 | 238 | 251 |  |
| D.LAGRDLTDY.L | Y | 17.35 | 1022.5032 | -0.4 | 512.2587 | 2 | 11.31 | 1553 | 1 | 182 | 190 |  |
| T.MYPGIADRM.Q | Y | 17.18 | 1052.4783 | -0.5 | 527.2462 | 2 | 11.87 | 1654 | 1 | 307 | 315 |  |
| D.LAGRDLTDYLMK.I | Y | 17.06 | 1394.7228 | -1.0 | 698.3680 | 2 | 12.55 | 1772 | 1 | 182 | 193 |  |
| E.KSYELPDGQVITI.G | Y | 15.81 | 1461.7715 | -1.4 | 731.8920 | 2 | 13.59 | 1952 | 1 | 240 | 252 |  |
| E.KSYELPDGQVIT.I | Y | 15.38 | 1348.6874 | -0.7 | 675.3505 | 2 | 12.00 | 1676 | 1 | 240 | 251 |  |
| total 23 peptides |  |  |  |  |  |  |  |  |  |  |  |  |
| --- | --- | --- | --- | --- | --- | --- | --- | --- | --- | --- | --- | --- |

tr|A0A7J6C441|A0A7J6C441\_9TELE

back to list

  

| Protein Coverage
| Supporting Peptides
|

Protein Coverage:

Supporting Peptides:

| Peptide | Uniq | -10lgP | Mass | ppm | m/z | z | RT | Scan | #Spec | Start | End | PTM |
| --- | --- | --- | --- | --- | --- | --- | --- | --- | --- | --- | --- | --- |
| E.KSYELPDGQVITIGNE.R | Y | 52.22 | 1761.8784 | -0.6 | 881.9460 | 2 | 13.41 | 1921 | 1 | 240 | 255 |  |
| S.LEKSYELPDGQVITI.G | Y | 50.90 | 1703.8981 | 0.2 | 852.9565 | 2 | 13.97 | 2015 | 1 | 238 | 252 |  |
| E.KSYELPDGQVITIG.N | Y | 37.16 | 1518.7930 | -0.3 | 760.4035 | 2 | 13.49 | 1935 | 2 | 240 | 253 |  |
| L.EKSYELPDGQVIT.I | Y | 35.16 | 1477.7300 | -4.0 | 739.8693 | 2 | 12.16 | 1702 | 1 | 239 | 251 |  |
| A.GFAGDDAPRA.V | Y | 33.15 | 975.4410 | 2.2 | 488.7289 | 2 | 10.28 | 1386 | 1 | 22 | 31 |  |
| K.SYELPDGQVITIG.N | Y | 31.23 | 1390.6980 | 0.0 | 696.3563 | 2 | 14.16 | 2047 | 1 | 241 | 253 |  |
| K.ANREKMTQ.I | Y | 28.79 | 976.4760 | 0.2 | 489.2454 | 2 | 8.23 | 1140 | 1 | 116 | 123 |  |
| S.LEKSYELPDGQVITIG.N | Y | 27.54 | 1760.9196 | -0.4 | 881.4667 | 2 | 13.69 | 1969 | 1 | 238 | 253 |  |
| L.DAGDGVTH.N | Y | 25.64 | 770.3195 | -0.1 | 386.1670 | 2 | 8.68 | 1226 | 7 | 156 | 163 |  |
| G.IITNWDDMEK.I | Y | 23.28 | 1263.5806 | 5.9 | 632.8013 | 2 | 12.12 | 1695 | 1 | 77 | 86 |  |
| E.KSYELPDGQ.V | Y | 22.22 | 1035.4873 | 2.3 | 518.7521 | 2 | 10.74 | 1457 | 1 | 240 | 248 |  |
| S.LEKSYELPDGQ.V | Y | 21.06 | 1277.6139 | -0.6 | 639.8138 | 2 | 11.19 | 1533 | 1 | 238 | 248 |  |
| S.LEKSYELPDGQVI.T | Y | 20.25 | 1489.7664 | -0.7 | 745.8900 | 2 | 12.83 | 1820 | 1 | 238 | 250 |  |
| K.SYELPDGQVIT.I | Y | 19.84 | 1220.5924 | -1.2 | 611.3027 | 2 | 13.08 | 1863 | 1 | 241 | 251 |  |
| K.SYELPDGQVITI.G | Y | 19.30 | 1333.6765 | -1.6 | 667.8445 | 2 | 14.45 | 2096 | 1 | 241 | 252 |  |
| G.FAGDDAPRA.V | Y | 18.98 | 918.4195 | 1.4 | 460.2177 | 2 | 15.22 | 2229 | 11 | 23 | 31 |  |
| E.KSYELPDGQV.I | Y | 18.67 | 1134.5557 | 0.0 | 568.2851 | 2 | 11.34 | 1560 | 1 | 240 | 249 |  |
| S.LEKSYELPDGQVIT.I | Y | 18.03 | 1590.8141 | -0.8 | 796.4137 | 2 | 12.31 | 1729 | 1 | 238 | 251 |  |
| D.LAGRDLTDY.L | Y | 17.35 | 1022.5032 | -0.4 | 512.2587 | 2 | 11.31 | 1553 | 1 | 182 | 190 |  |
| T.MYPGIADRM.Q | Y | 17.18 | 1052.4783 | -0.5 | 527.2462 | 2 | 11.87 | 1654 | 1 | 307 | 315 |  |
| D.LAGRDLTDYLMK.I | Y | 17.06 | 1394.7228 | -1.0 | 698.3680 | 2 | 12.55 | 1772 | 1 | 182 | 193 |  |
| E.KSYELPDGQVITI.G | Y | 15.81 | 1461.7715 | -1.4 | 731.8920 | 2 | 13.59 | 1952 | 1 | 240 | 252 |  |
| E.KSYELPDGQVIT.I | Y | 15.38 | 1348.6874 | -0.7 | 675.3505 | 2 | 12.00 | 1676 | 1 | 240 | 251 |  |
| total 23 peptides |  |  |  |  |  |  |  |  |  |  |  |  |
| --- | --- | --- | --- | --- | --- | --- | --- | --- | --- | --- | --- | --- |

tr|A0A5C6NQR5|A0A5C6NQR5\_9TELE

back to list

  

| Protein Coverage
| Supporting Peptides
|

Protein Coverage:

Supporting Peptides:

| Peptide | Uniq | -10lgP | Mass | ppm | m/z | z | RT | Scan | #Spec | Start | End | PTM |
| --- | --- | --- | --- | --- | --- | --- | --- | --- | --- | --- | --- | --- |
| A.IIDQDNSGFIEEEELK.L | N | 67.46 | 1877.8894 | -1.0 | 939.9510 | 2 | 12.28 | 1724 | 1 | 207 | 222 |  |
| E.IIDQDKSGFIEEEELK.L | N | 49.57 | 1891.9414 | -0.6 | 946.9774 | 2 | 11.72 | 1627 | 1 | 304 | 319 |  |
| E.IIDQDKSGFIE.E | N | 44.01 | 1263.6346 | -0.5 | 632.8243 | 2 | 11.60 | 1605 | 1 | 304 | 314 |  |
| S.GFIEEEELK.L | N | 31.42 | 1092.5338 | -0.1 | 547.2741 | 2 | 11.80 | 1641 | 1 | 214 | 222 |  |
| A.IIDQDNSGFIEEEELKLF.L | N | 30.80 | 2138.0417 | 0.6 | 1070.0288 | 2 | 14.58 | 2119 | 1 | 207 | 224 |  |
| F.AIIDQDNSGFIE.E | N | 26.76 | 1320.6198 | -0.6 | 661.3168 | 2 | 12.86 | 1825 | 1 | 206 | 217 |  |
| Q.DKSGFIEEEELK.L | N | 25.85 | 1422.6877 | -0.4 | 712.3508 | 2 | 11.40 | 1570 | 1 | 308 | 319 |  |
| A.IIDQDNSGFIEEEE.L | N | 16.99 | 1636.7103 | 0.3 | 819.3627 | 2 | 12.36 | 1738 | 1 | 207 | 220 |  |
| total 8 peptides |  |  |  |  |  |  |  |  |  |  |  |  |
| --- | --- | --- | --- | --- | --- | --- | --- | --- | --- | --- | --- | --- |

tr|A0A6P6N9Z4|A0A6P6N9Z4\_CARAU

back to list

  

| Protein Coverage
| Supporting Peptides
|

Protein Coverage:

Supporting Peptides:

| Peptide | Uniq | -10lgP | Mass | ppm | m/z | z | RT | Scan | #Spec | Start | End | PTM |
| --- | --- | --- | --- | --- | --- | --- | --- | --- | --- | --- | --- | --- |
| W.LDKNKDPLNDSVVQL.Y | N | 55.11 | 1696.8995 | -0.3 | 849.4568 | 2 | 12.23 | 1715 | 1 | 596 | 610 |  |
| W.LDKNKDPLNDSVVQ.L | N | 49.71 | 1583.8154 | -0.7 | 792.9144 | 2 | 10.89 | 1481 | 5 | 596 | 609 |  |
| L.SKIEDEQSLGAQ.L | N | 35.68 | 1303.6255 | 4.7 | 652.8231 | 2 | 10.20 | 1374 | 1 | 1095 | 1106 |  |
| K.LEQQVDDLEGSLE.Q | N | 32.76 | 1473.6835 | -0.5 | 737.8486 | 2 | 12.65 | 1789 | 1 | 1030 | 1042 |  |
| K.NDENVRQ.L | N | 29.03 | 873.3940 | -0.4 | 437.7041 | 2 | 8.49 | 1190 | 1 | 1266 | 1272 |  |
| Q.GEVDDTVQEAR.N | N | 25.82 | 1217.5524 | 4.7 | 609.7863 | 2 | 10.04 | 1355 | 1 | 1742 | 1752 |  |
| K.KMEGDLNEM.E | N | 25.67 | 1065.4470 | -0.7 | 533.7304 | 2 | 11.02 | 1504 | 1 | 1620 | 1628 |  |
| K.FDKIEDM.A | N | 23.34 | 896.3950 | -0.6 | 449.2045 | 2 | 11.46 | 1581 | 2 | 84 | 90 |  |
| E.LKKEQDTSAH.L | N | 22.49 | 1155.5884 | -0.2 | 578.8013 | 2 | 7.77 | 1050 | 1 | 1772 | 1781 |  |
| Q.GEVDDTVQEA.R | N | 20.03 | 1061.4513 | -0.3 | 531.7328 | 2 | 10.64 | 1439 | 1 | 1742 | 1751 |  |
| Q.TALEEAEGTL.E | N | 19.11 | 1032.4974 | -0.7 | 517.2556 | 2 | 12.43 | 1750 | 1 | 1545 | 1554 |  |
| M.AEELKKE.Q | N | 18.25 | 845.4494 | -0.4 | 423.7318 | 2 | 8.54 | 1200 | 1 | 1769 | 1775 |  |
| M.NPPKFDKIEDM.A | N | 17.32 | 1332.6383 | -0.5 | 667.3261 | 2 | 11.50 | 1587 | 1 | 80 | 90 |  |
| K.SINDVEEF.I | N | 15.40 | 951.4185 | -0.3 | 476.7164 | 2 | 12.52 | 1766 | 1 | 324 | 331 |  |
| total 14 peptides |  |  |  |  |  |  |  |  |  |  |  |  |
| --- | --- | --- | --- | --- | --- | --- | --- | --- | --- | --- | --- | --- |

tr|A0A3B4CZR3|A0A3B4CZR3\_PYGNA

back to list

  

| Protein Coverage
| Supporting Peptides
|

Protein Coverage:

Supporting Peptides:

| Peptide | Uniq | -10lgP | Mass | ppm | m/z | z | RT | Scan | #Spec | Start | End | PTM |
| --- | --- | --- | --- | --- | --- | --- | --- | --- | --- | --- | --- | --- |
| W.LDKNKDPLNDSVVQL.Y | N | 55.11 | 1696.8995 | -0.3 | 849.4568 | 2 | 12.23 | 1715 | 1 | 558 | 572 |  |
| W.LDKNKDPLNDSVVQ.L | N | 49.71 | 1583.8154 | -0.7 | 792.9144 | 2 | 10.89 | 1481 | 5 | 558 | 571 |  |
| L.SKIEDEQSLGAQ.L | N | 35.68 | 1303.6255 | 4.7 | 652.8231 | 2 | 10.20 | 1374 | 1 | 1055 | 1066 |  |
| K.LEQQVDDLEGSLE.Q | N | 32.76 | 1473.6835 | -0.5 | 737.8486 | 2 | 12.65 | 1789 | 1 | 990 | 1002 |  |
| A.EWKQKYEEGQAELE.G | N | 26.99 | 1765.8158 | -0.6 | 883.9147 | 2 | 11.00 | 1500 | 1 | 1418 | 1431 |  |
| Q.GEVDDTVQEAR.N | N | 25.82 | 1217.5524 | 4.7 | 609.7863 | 2 | 10.04 | 1355 | 1 | 1702 | 1712 |  |
| K.KMEGDLNEM.E | N | 25.67 | 1065.4470 | -0.7 | 533.7304 | 2 | 11.02 | 1504 | 1 | 1580 | 1588 |  |
| K.FDKIEDM.A | N | 23.34 | 896.3950 | -0.6 | 449.2045 | 2 | 11.46 | 1581 | 2 | 84 | 90 |  |
| E.LKKEQDTSAH.L | N | 22.49 | 1155.5884 | -0.2 | 578.8013 | 2 | 7.77 | 1050 | 1 | 1732 | 1741 |  |
| Q.GEVDDTVQEA.R | N | 20.03 | 1061.4513 | -0.3 | 531.7328 | 2 | 10.64 | 1439 | 1 | 1702 | 1711 |  |
| Q.TALEEAEGTL.E | N | 19.11 | 1032.4974 | -0.7 | 517.2556 | 2 | 12.43 | 1750 | 1 | 1505 | 1514 |  |
| M.AEELKKE.Q | N | 18.25 | 845.4494 | -0.4 | 423.7318 | 2 | 8.54 | 1200 | 1 | 1729 | 1735 |  |
| M.NPPKFDKIEDM.A | N | 17.32 | 1332.6383 | -0.5 | 667.3261 | 2 | 11.50 | 1587 | 1 | 80 | 90 |  |
| K.SINDVEEF.I | N | 15.40 | 951.4185 | -0.3 | 476.7164 | 2 | 12.52 | 1766 | 1 | 316 | 323 |  |
| total 14 peptides |  |  |  |  |  |  |  |  |  |  |  |  |
| --- | --- | --- | --- | --- | --- | --- | --- | --- | --- | --- | --- | --- |

tr|A0A674NSP7|A0A674NSP7\_TAKRU

back to list

  

| Protein Coverage
| Supporting Peptides
|

Protein Coverage:

Supporting Peptides:

| Peptide | Uniq | -10lgP | Mass | ppm | m/z | z | RT | Scan | #Spec | Start | End | PTM |
| --- | --- | --- | --- | --- | --- | --- | --- | --- | --- | --- | --- | --- |
| W.LDKNKDPLNDSVVQL.Y | N | 55.11 | 1696.8995 | -0.3 | 849.4568 | 2 | 12.23 | 1715 | 1 | 597 | 611 |  |
| W.LDKNKDPLNDSVVQ.L | N | 49.71 | 1583.8154 | -0.7 | 792.9144 | 2 | 10.89 | 1481 | 5 | 597 | 610 |  |
| L.SKIEDEQSLGAQ.L | N | 35.68 | 1303.6255 | 4.7 | 652.8231 | 2 | 10.20 | 1374 | 1 | 1097 | 1108 |  |
| K.LEQQVDDLEGSLE.Q | N | 32.76 | 1473.6835 | -0.5 | 737.8486 | 2 | 12.65 | 1789 | 1 | 1032 | 1044 |  |
| K.NDENVRQ.L | N | 29.03 | 873.3940 | -0.4 | 437.7041 | 2 | 8.49 | 1190 | 1 | 1268 | 1274 |  |
| A.EWKQKYEEGQAELE.G | N | 26.99 | 1765.8158 | -0.6 | 883.9147 | 2 | 11.00 | 1500 | 1 | 1460 | 1473 |  |
| K.KMEGDLNEM.E | N | 25.67 | 1065.4470 | -0.7 | 533.7304 | 2 | 11.02 | 1504 | 1 | 1622 | 1630 |  |
| K.FDKIEDM.A | N | 23.34 | 896.3950 | -0.6 | 449.2045 | 2 | 11.46 | 1581 | 2 | 85 | 91 |  |
| E.LKKEQDTSAH.L | N | 22.49 | 1155.5884 | -0.2 | 578.8013 | 2 | 7.77 | 1050 | 1 | 1774 | 1783 |  |
| Q.TALEEAEGTL.E | N | 19.11 | 1032.4974 | -0.7 | 517.2556 | 2 | 12.43 | 1750 | 1 | 1547 | 1556 |  |
| M.AEELKKE.Q | N | 18.25 | 845.4494 | -0.4 | 423.7318 | 2 | 8.54 | 1200 | 1 | 1771 | 1777 |  |
| M.NPPKFDKIEDM.A | N | 17.32 | 1332.6383 | -0.5 | 667.3261 | 2 | 11.50 | 1587 | 1 | 81 | 91 |  |
| K.SINDVEEF.I | N | 15.40 | 951.4185 | -0.3 | 476.7164 | 2 | 12.52 | 1766 | 1 | 325 | 332 |  |
| total 13 peptides |  |  |  |  |  |  |  |  |  |  |  |  |
| --- | --- | --- | --- | --- | --- | --- | --- | --- | --- | --- | --- | --- |

tr|A0A6A4SNF9|A0A6A4SNF9\_SCOMX

back to list

  

| Protein Coverage
| Supporting Peptides
|

Protein Coverage:

Supporting Peptides:

| Peptide | Uniq | -10lgP | Mass | ppm | m/z | z | RT | Scan | #Spec | Start | End | PTM |
| --- | --- | --- | --- | --- | --- | --- | --- | --- | --- | --- | --- | --- |
| Q.IIDQDNSGFIEEEELK.L | N | 67.46 | 1877.8894 | -1.0 | 939.9510 | 2 | 12.28 | 1724 | 1 | 147 | 162 |  |
| A.FDIIDQDKSGFIE.E | N | 51.87 | 1525.7300 | -1.4 | 763.8712 | 2 | 13.28 | 1898 | 1 | 226 | 238 |  |
| D.IIDQDKSGFIE.E | N | 44.01 | 1263.6346 | -0.5 | 632.8243 | 2 | 11.60 | 1605 | 1 | 228 | 238 |  |
| S.GFIEEEELK.L | N | 31.42 | 1092.5338 | -0.1 | 547.2741 | 2 | 11.80 | 1641 | 1 | 154 | 162 |  |
| Q.IIDQDNSGFIEEEELKLF.L | N | 30.80 | 2138.0417 | 0.6 | 1070.0288 | 2 | 14.58 | 2119 | 1 | 147 | 164 |  |
| Q.IIDQDNSGFIEEEE.L | N | 16.99 | 1636.7103 | 0.3 | 819.3627 | 2 | 12.36 | 1738 | 1 | 147 | 160 |  |
| total 6 peptides |  |  |  |  |  |  |  |  |  |  |  |  |
| --- | --- | --- | --- | --- | --- | --- | --- | --- | --- | --- | --- | --- |

tr|Q90YF6|Q90YF6\_PARFO

back to list

  

| Protein Coverage
| Supporting Peptides
|

Protein Coverage:

Supporting Peptides:

| Peptide | Uniq | -10lgP | Mass | ppm | m/z | z | RT | Scan | #Spec | Start | End | PTM |
| --- | --- | --- | --- | --- | --- | --- | --- | --- | --- | --- | --- | --- |
| W.LDKNKDPLNDSVVQL.Y | N | 55.11 | 1696.8995 | -0.3 | 849.4568 | 2 | 12.23 | 1715 | 1 | 596 | 610 |  |
| W.LDKNKDPLNDSVVQ.L | N | 49.71 | 1583.8154 | -0.7 | 792.9144 | 2 | 10.89 | 1481 | 5 | 596 | 609 |  |
| L.SKIEDEQSLGAQ.L | N | 35.68 | 1303.6255 | 4.7 | 652.8231 | 2 | 10.20 | 1374 | 1 | 1094 | 1105 |  |
| K.LEQQVDDLEGSLE.Q | N | 32.76 | 1473.6835 | -0.5 | 737.8486 | 2 | 12.65 | 1789 | 1 | 1029 | 1041 |  |
| K.NDENVRQ.L | N | 29.03 | 873.3940 | -0.4 | 437.7041 | 2 | 8.49 | 1190 | 1 | 1265 | 1271 |  |
| A.EWKQKYEEGQAELE.G | N | 26.99 | 1765.8158 | -0.6 | 883.9147 | 2 | 11.00 | 1500 | 1 | 1457 | 1470 |  |
| Q.GEVDDTVQEAR.N | N | 25.82 | 1217.5524 | 4.7 | 609.7863 | 2 | 10.04 | 1355 | 1 | 1741 | 1751 |  |
| K.KMEGDLNEM.E | N | 25.67 | 1065.4470 | -0.7 | 533.7304 | 2 | 11.02 | 1504 | 1 | 1619 | 1627 |  |
| E.LKKEQDTSAH.L | N | 22.49 | 1155.5884 | -0.2 | 578.8013 | 2 | 7.77 | 1050 | 1 | 1771 | 1780 |  |
| Q.GEVDDTVQEA.R | N | 20.03 | 1061.4513 | -0.3 | 531.7328 | 2 | 10.64 | 1439 | 1 | 1741 | 1750 |  |
| M.AEELKKE.Q | N | 18.25 | 845.4494 | -0.4 | 423.7318 | 2 | 8.54 | 1200 | 1 | 1768 | 1774 |  |
| K.SINDVEEF.I | N | 15.40 | 951.4185 | -0.3 | 476.7164 | 2 | 12.52 | 1766 | 1 | 324 | 331 |  |
| total 12 peptides |  |  |  |  |  |  |  |  |  |  |  |  |
| --- | --- | --- | --- | --- | --- | --- | --- | --- | --- | --- | --- | --- |

tr|W5LNC0|W5LNC0\_ASTMX

back to list

  

| Protein Coverage
| Supporting Peptides
|

Protein Coverage:

Supporting Peptides:

| Peptide | Uniq | -10lgP | Mass | ppm | m/z | z | RT | Scan | #Spec | Start | End | PTM |
| --- | --- | --- | --- | --- | --- | --- | --- | --- | --- | --- | --- | --- |
| A.IIDQDNSGFIEEEELK.L | N | 67.46 | 1877.8894 | -1.0 | 939.9510 | 2 | 12.28 | 1724 | 1 | 50 | 65 |  |
| A.GDSDGDGKIGVDEF.A | N | 35.47 | 1409.5946 | -1.1 | 705.8038 | 2 | 11.99 | 1675 | 1 | 90 | 103 |  |
| S.GFIEEEELK.L | N | 31.42 | 1092.5338 | -0.1 | 547.2741 | 2 | 11.80 | 1641 | 1 | 57 | 65 |  |
| A.IIDQDNSGFIEEEELKLF.L | N | 30.80 | 2138.0417 | 0.6 | 1070.0288 | 2 | 14.58 | 2119 | 1 | 50 | 67 |  |
| F.AIIDQDNSGFIE.E | N | 26.76 | 1320.6198 | -0.6 | 661.3168 | 2 | 12.86 | 1825 | 1 | 49 | 60 |  |
| F.LAAGDSDGDGKIGVD.E | N | 25.04 | 1388.6418 | -0.5 | 695.3278 | 2 | 10.74 | 1456 | 1 | 87 | 101 |  |
| A.IIDQDNSGFIEEEE.L | N | 16.99 | 1636.7103 | 0.3 | 819.3627 | 2 | 12.36 | 1738 | 1 | 50 | 63 |  |
| total 7 peptides |  |  |  |  |  |  |  |  |  |  |  |  |
| --- | --- | --- | --- | --- | --- | --- | --- | --- | --- | --- | --- | --- |

tr|A0A2I4BRJ5|A0A2I4BRJ5\_9TELE

back to list

  

| Protein Coverage
| Supporting Peptides
|

Protein Coverage:

Supporting Peptides:

| Peptide | Uniq | -10lgP | Mass | ppm | m/z | z | RT | Scan | #Spec | Start | End | PTM |
| --- | --- | --- | --- | --- | --- | --- | --- | --- | --- | --- | --- | --- |
| A.IIDQDNSGFIEEEELK.L | N | 67.46 | 1877.8894 | -1.0 | 939.9510 | 2 | 12.28 | 1724 | 1 | 50 | 65 |  |
| A.GDSDGDGKIGVDEF.A | N | 35.47 | 1409.5946 | -1.1 | 705.8038 | 2 | 11.99 | 1675 | 1 | 90 | 103 |  |
| S.GFIEEEELK.L | N | 31.42 | 1092.5338 | -0.1 | 547.2741 | 2 | 11.80 | 1641 | 1 | 57 | 65 |  |
| A.IIDQDNSGFIEEEELKLF.L | N | 30.80 | 2138.0417 | 0.6 | 1070.0288 | 2 | 14.58 | 2119 | 1 | 50 | 67 |  |
| F.AIIDQDNSGFIE.E | N | 26.76 | 1320.6198 | -0.6 | 661.3168 | 2 | 12.86 | 1825 | 1 | 49 | 60 |  |
| F.LAAGDSDGDGKIGVD.E | N | 25.04 | 1388.6418 | -0.5 | 695.3278 | 2 | 10.74 | 1456 | 1 | 87 | 101 |  |
| A.IIDQDNSGFIEEEE.L | N | 16.99 | 1636.7103 | 0.3 | 819.3627 | 2 | 12.36 | 1738 | 1 | 50 | 63 |  |
| total 7 peptides |  |  |  |  |  |  |  |  |  |  |  |  |
| --- | --- | --- | --- | --- | --- | --- | --- | --- | --- | --- | --- | --- |

tr|A0A3B4TWT0|A0A3B4TWT0\_SERDU

back to list

  

| Protein Coverage
| Supporting Peptides
|

Protein Coverage:

Supporting Peptides:

| Peptide | Uniq | -10lgP | Mass | ppm | m/z | z | RT | Scan | #Spec | Start | End | PTM |
| --- | --- | --- | --- | --- | --- | --- | --- | --- | --- | --- | --- | --- |
| A.IIDQDNSGFIEEEELK.L | N | 67.46 | 1877.8894 | -1.0 | 939.9510 | 2 | 12.28 | 1724 | 1 | 50 | 65 |  |
| A.GDSDGDGKIGVDEF.A | N | 35.47 | 1409.5946 | -1.1 | 705.8038 | 2 | 11.99 | 1675 | 1 | 90 | 103 |  |
| S.GFIEEEELK.L | N | 31.42 | 1092.5338 | -0.1 | 547.2741 | 2 | 11.80 | 1641 | 1 | 57 | 65 |  |
| A.IIDQDNSGFIEEEELKLF.L | N | 30.80 | 2138.0417 | 0.6 | 1070.0288 | 2 | 14.58 | 2119 | 1 | 50 | 67 |  |
| F.AIIDQDNSGFIE.E | N | 26.76 | 1320.6198 | -0.6 | 661.3168 | 2 | 12.86 | 1825 | 1 | 49 | 60 |  |
| F.LAAGDSDGDGKIGVD.E | N | 25.04 | 1388.6418 | -0.5 | 695.3278 | 2 | 10.74 | 1456 | 1 | 87 | 101 |  |
| A.IIDQDNSGFIEEEE.L | N | 16.99 | 1636.7103 | 0.3 | 819.3627 | 2 | 12.36 | 1738 | 1 | 50 | 63 |  |
| total 7 peptides |  |  |  |  |  |  |  |  |  |  |  |  |
| --- | --- | --- | --- | --- | --- | --- | --- | --- | --- | --- | --- | --- |

tr|A0A1A8L493|A0A1A8L493\_9TELE

back to list

  

| Protein Coverage
| Supporting Peptides
|

Protein Coverage:

Supporting Peptides:

| Peptide | Uniq | -10lgP | Mass | ppm | m/z | z | RT | Scan | #Spec | Start | End | PTM |
| --- | --- | --- | --- | --- | --- | --- | --- | --- | --- | --- | --- | --- |
| A.IIDQDNSGFIEEEELK.L | N | 67.46 | 1877.8894 | -1.0 | 939.9510 | 2 | 12.28 | 1724 | 1 | 50 | 65 |  |
| A.GDSDGDGKIGVDEF.A | N | 35.47 | 1409.5946 | -1.1 | 705.8038 | 2 | 11.99 | 1675 | 1 | 90 | 103 |  |
| S.GFIEEEELK.L | N | 31.42 | 1092.5338 | -0.1 | 547.2741 | 2 | 11.80 | 1641 | 1 | 57 | 65 |  |
| A.IIDQDNSGFIEEEELKLF.L | N | 30.80 | 2138.0417 | 0.6 | 1070.0288 | 2 | 14.58 | 2119 | 1 | 50 | 67 |  |
| F.AIIDQDNSGFIE.E | N | 26.76 | 1320.6198 | -0.6 | 661.3168 | 2 | 12.86 | 1825 | 1 | 49 | 60 |  |
| F.LAAGDSDGDGKIGVD.E | N | 25.04 | 1388.6418 | -0.5 | 695.3278 | 2 | 10.74 | 1456 | 1 | 87 | 101 |  |
| A.IIDQDNSGFIEEEE.L | N | 16.99 | 1636.7103 | 0.3 | 819.3627 | 2 | 12.36 | 1738 | 1 | 50 | 63 |  |
| total 7 peptides |  |  |  |  |  |  |  |  |  |  |  |  |
| --- | --- | --- | --- | --- | --- | --- | --- | --- | --- | --- | --- | --- |

tr|A0A672FI61|A0A672FI61\_SALFA

back to list

  

| Protein Coverage
| Supporting Peptides
|

Protein Coverage:

Supporting Peptides:

| Peptide | Uniq | -10lgP | Mass | ppm | m/z | z | RT | Scan | #Spec | Start | End | PTM |
| --- | --- | --- | --- | --- | --- | --- | --- | --- | --- | --- | --- | --- |
| A.IIDQDNSGFIEEEELK.L | N | 67.46 | 1877.8894 | -1.0 | 939.9510 | 2 | 12.28 | 1724 | 1 | 50 | 65 |  |
| A.GDSDGDGKIGVDEF.A | N | 35.47 | 1409.5946 | -1.1 | 705.8038 | 2 | 11.99 | 1675 | 1 | 90 | 103 |  |
| S.GFIEEEELK.L | N | 31.42 | 1092.5338 | -0.1 | 547.2741 | 2 | 11.80 | 1641 | 1 | 57 | 65 |  |
| A.IIDQDNSGFIEEEELKLF.L | N | 30.80 | 2138.0417 | 0.6 | 1070.0288 | 2 | 14.58 | 2119 | 1 | 50 | 67 |  |
| F.AIIDQDNSGFIE.E | N | 26.76 | 1320.6198 | -0.6 | 661.3168 | 2 | 12.86 | 1825 | 1 | 49 | 60 |  |
| F.LAAGDSDGDGKIGVD.E | N | 25.04 | 1388.6418 | -0.5 | 695.3278 | 2 | 10.74 | 1456 | 1 | 87 | 101 |  |
| A.IIDQDNSGFIEEEE.L | N | 16.99 | 1636.7103 | 0.3 | 819.3627 | 2 | 12.36 | 1738 | 1 | 50 | 63 |  |
| total 7 peptides |  |  |  |  |  |  |  |  |  |  |  |  |
| --- | --- | --- | --- | --- | --- | --- | --- | --- | --- | --- | --- | --- |

tr|A0A1A8G5F7|A0A1A8G5F7\_9TELE

back to list

  

| Protein Coverage
| Supporting Peptides
|

Protein Coverage:

Supporting Peptides:

| Peptide | Uniq | -10lgP | Mass | ppm | m/z | z | RT | Scan | #Spec | Start | End | PTM |
| --- | --- | --- | --- | --- | --- | --- | --- | --- | --- | --- | --- | --- |
| A.IIDQDNSGFIEEEELK.L | N | 67.46 | 1877.8894 | -1.0 | 939.9510 | 2 | 12.28 | 1724 | 1 | 50 | 65 |  |
| A.GDSDGDGKIGVDEF.A | N | 35.47 | 1409.5946 | -1.1 | 705.8038 | 2 | 11.99 | 1675 | 1 | 90 | 103 |  |
| S.GFIEEEELK.L | N | 31.42 | 1092.5338 | -0.1 | 547.2741 | 2 | 11.80 | 1641 | 1 | 57 | 65 |  |
| A.IIDQDNSGFIEEEELKLF.L | N | 30.80 | 2138.0417 | 0.6 | 1070.0288 | 2 | 14.58 | 2119 | 1 | 50 | 67 |  |
| F.AIIDQDNSGFIE.E | N | 26.76 | 1320.6198 | -0.6 | 661.3168 | 2 | 12.86 | 1825 | 1 | 49 | 60 |  |
| F.LAAGDSDGDGKIGVD.E | N | 25.04 | 1388.6418 | -0.5 | 695.3278 | 2 | 10.74 | 1456 | 1 | 87 | 101 |  |
| A.IIDQDNSGFIEEEE.L | N | 16.99 | 1636.7103 | 0.3 | 819.3627 | 2 | 12.36 | 1738 | 1 | 50 | 63 |  |
| total 7 peptides |  |  |  |  |  |  |  |  |  |  |  |  |
| --- | --- | --- | --- | --- | --- | --- | --- | --- | --- | --- | --- | --- |

tr|A0A4W6DV48|A0A4W6DV48\_LATCA

back to list

  

| Protein Coverage
| Supporting Peptides
|

Protein Coverage:

Supporting Peptides:

| Peptide | Uniq | -10lgP | Mass | ppm | m/z | z | RT | Scan | #Spec | Start | End | PTM |
| --- | --- | --- | --- | --- | --- | --- | --- | --- | --- | --- | --- | --- |
| A.IIDQDNSGFIEEEELK.L | N | 67.46 | 1877.8894 | -1.0 | 939.9510 | 2 | 12.28 | 1724 | 1 | 50 | 65 |  |
| A.GDSDGDGKIGVDEF.A | N | 35.47 | 1409.5946 | -1.1 | 705.8038 | 2 | 11.99 | 1675 | 1 | 90 | 103 |  |
| S.GFIEEEELK.L | N | 31.42 | 1092.5338 | -0.1 | 547.2741 | 2 | 11.80 | 1641 | 1 | 57 | 65 |  |
| A.IIDQDNSGFIEEEELKLF.L | N | 30.80 | 2138.0417 | 0.6 | 1070.0288 | 2 | 14.58 | 2119 | 1 | 50 | 67 |  |
| F.AIIDQDNSGFIE.E | N | 26.76 | 1320.6198 | -0.6 | 661.3168 | 2 | 12.86 | 1825 | 1 | 49 | 60 |  |
| F.LAAGDSDGDGKIGVD.E | N | 25.04 | 1388.6418 | -0.5 | 695.3278 | 2 | 10.74 | 1456 | 1 | 87 | 101 |  |
| A.IIDQDNSGFIEEEE.L | N | 16.99 | 1636.7103 | 0.3 | 819.3627 | 2 | 12.36 | 1738 | 1 | 50 | 63 |  |
| total 7 peptides |  |  |  |  |  |  |  |  |  |  |  |  |
| --- | --- | --- | --- | --- | --- | --- | --- | --- | --- | --- | --- | --- |

tr|F8U037|F8U037\_EPIBR

back to list

  

| Protein Coverage
| Supporting Peptides
|

Protein Coverage:

Supporting Peptides:

| Peptide | Uniq | -10lgP | Mass | ppm | m/z | z | RT | Scan | #Spec | Start | End | PTM |
| --- | --- | --- | --- | --- | --- | --- | --- | --- | --- | --- | --- | --- |
| A.IIDQDNSGFIEEEELK.L | N | 67.46 | 1877.8894 | -1.0 | 939.9510 | 2 | 12.28 | 1724 | 1 | 50 | 65 |  |
| A.GDSDGDGKIGVDEF.A | N | 35.47 | 1409.5946 | -1.1 | 705.8038 | 2 | 11.99 | 1675 | 1 | 90 | 103 |  |
| S.GFIEEEELK.L | N | 31.42 | 1092.5338 | -0.1 | 547.2741 | 2 | 11.80 | 1641 | 1 | 57 | 65 |  |
| A.IIDQDNSGFIEEEELKLF.L | N | 30.80 | 2138.0417 | 0.6 | 1070.0288 | 2 | 14.58 | 2119 | 1 | 50 | 67 |  |
| F.AIIDQDNSGFIE.E | N | 26.76 | 1320.6198 | -0.6 | 661.3168 | 2 | 12.86 | 1825 | 1 | 49 | 60 |  |
| F.LAAGDSDGDGKIGVD.E | N | 25.04 | 1388.6418 | -0.5 | 695.3278 | 2 | 10.74 | 1456 | 1 | 87 | 101 |  |
| A.IIDQDNSGFIEEEE.L | N | 16.99 | 1636.7103 | 0.3 | 819.3627 | 2 | 12.36 | 1738 | 1 | 50 | 63 |  |
| total 7 peptides |  |  |  |  |  |  |  |  |  |  |  |  |
| --- | --- | --- | --- | --- | --- | --- | --- | --- | --- | --- | --- | --- |

tr|A0A1A7WW39|A0A1A7WW39\_9TELE

back to list

  

| Protein Coverage
| Supporting Peptides
|

Protein Coverage:

Supporting Peptides:

| Peptide | Uniq | -10lgP | Mass | ppm | m/z | z | RT | Scan | #Spec | Start | End | PTM |
| --- | --- | --- | --- | --- | --- | --- | --- | --- | --- | --- | --- | --- |
| A.IIDQDNSGFIEEEELK.L | N | 67.46 | 1877.8894 | -1.0 | 939.9510 | 2 | 12.28 | 1724 | 1 | 50 | 65 |  |
| A.GDSDGDGKIGVDEF.A | N | 35.47 | 1409.5946 | -1.1 | 705.8038 | 2 | 11.99 | 1675 | 1 | 90 | 103 |  |
| S.GFIEEEELK.L | N | 31.42 | 1092.5338 | -0.1 | 547.2741 | 2 | 11.80 | 1641 | 1 | 57 | 65 |  |
| A.IIDQDNSGFIEEEELKLF.L | N | 30.80 | 2138.0417 | 0.6 | 1070.0288 | 2 | 14.58 | 2119 | 1 | 50 | 67 |  |
| F.AIIDQDNSGFIE.E | N | 26.76 | 1320.6198 | -0.6 | 661.3168 | 2 | 12.86 | 1825 | 1 | 49 | 60 |  |
| F.LAAGDSDGDGKIGVD.E | N | 25.04 | 1388.6418 | -0.5 | 695.3278 | 2 | 10.74 | 1456 | 1 | 87 | 101 |  |
| A.IIDQDNSGFIEEEE.L | N | 16.99 | 1636.7103 | 0.3 | 819.3627 | 2 | 12.36 | 1738 | 1 | 50 | 63 |  |
| total 7 peptides |  |  |  |  |  |  |  |  |  |  |  |  |
| --- | --- | --- | --- | --- | --- | --- | --- | --- | --- | --- | --- | --- |

tr|A0A1A8C322|A0A1A8C322\_9TELE

back to list

  

| Protein Coverage
| Supporting Peptides
|

Protein Coverage:

Supporting Peptides:

| Peptide | Uniq | -10lgP | Mass | ppm | m/z | z | RT | Scan | #Spec | Start | End | PTM |
| --- | --- | --- | --- | --- | --- | --- | --- | --- | --- | --- | --- | --- |
| A.IIDQDNSGFIEEEELK.L | N | 67.46 | 1877.8894 | -1.0 | 939.9510 | 2 | 12.28 | 1724 | 1 | 50 | 65 |  |
| A.GDSDGDGKIGVDEF.A | N | 35.47 | 1409.5946 | -1.1 | 705.8038 | 2 | 11.99 | 1675 | 1 | 90 | 103 |  |
| S.GFIEEEELK.L | N | 31.42 | 1092.5338 | -0.1 | 547.2741 | 2 | 11.80 | 1641 | 1 | 57 | 65 |  |
| A.IIDQDNSGFIEEEELKLF.L | N | 30.80 | 2138.0417 | 0.6 | 1070.0288 | 2 | 14.58 | 2119 | 1 | 50 | 67 |  |
| F.AIIDQDNSGFIE.E | N | 26.76 | 1320.6198 | -0.6 | 661.3168 | 2 | 12.86 | 1825 | 1 | 49 | 60 |  |
| F.LAAGDSDGDGKIGVD.E | N | 25.04 | 1388.6418 | -0.5 | 695.3278 | 2 | 10.74 | 1456 | 1 | 87 | 101 |  |
| A.IIDQDNSGFIEEEE.L | N | 16.99 | 1636.7103 | 0.3 | 819.3627 | 2 | 12.36 | 1738 | 1 | 50 | 63 |  |
| total 7 peptides |  |  |  |  |  |  |  |  |  |  |  |  |
| --- | --- | --- | --- | --- | --- | --- | --- | --- | --- | --- | --- | --- |

tr|A0A3Q3GY11|A0A3Q3GY11\_9LABR

back to list

  

| Protein Coverage
| Supporting Peptides
|

Protein Coverage:

Supporting Peptides:

| Peptide | Uniq | -10lgP | Mass | ppm | m/z | z | RT | Scan | #Spec | Start | End | PTM |
| --- | --- | --- | --- | --- | --- | --- | --- | --- | --- | --- | --- | --- |
| A.IIDQDNSGFIEEEELK.L | N | 67.46 | 1877.8894 | -1.0 | 939.9510 | 2 | 12.28 | 1724 | 1 | 50 | 65 |  |
| A.GDSDGDGKIGVDEF.A | N | 35.47 | 1409.5946 | -1.1 | 705.8038 | 2 | 11.99 | 1675 | 1 | 90 | 103 |  |
| S.GFIEEEELK.L | N | 31.42 | 1092.5338 | -0.1 | 547.2741 | 2 | 11.80 | 1641 | 1 | 57 | 65 |  |
| A.IIDQDNSGFIEEEELKLF.L | N | 30.80 | 2138.0417 | 0.6 | 1070.0288 | 2 | 14.58 | 2119 | 1 | 50 | 67 |  |
| F.AIIDQDNSGFIE.E | N | 26.76 | 1320.6198 | -0.6 | 661.3168 | 2 | 12.86 | 1825 | 1 | 49 | 60 |  |
| F.LAAGDSDGDGKIGVD.E | N | 25.04 | 1388.6418 | -0.5 | 695.3278 | 2 | 10.74 | 1456 | 1 | 87 | 101 |  |
| A.IIDQDNSGFIEEEE.L | N | 16.99 | 1636.7103 | 0.3 | 819.3627 | 2 | 12.36 | 1738 | 1 | 50 | 63 |  |
| total 7 peptides |  |  |  |  |  |  |  |  |  |  |  |  |
| --- | --- | --- | --- | --- | --- | --- | --- | --- | --- | --- | --- | --- |

tr|A0A1A8J4D2|A0A1A8J4D2\_NOTKU

back to list

  

| Protein Coverage
| Supporting Peptides
|

Protein Coverage:

Supporting Peptides:

| Peptide | Uniq | -10lgP | Mass | ppm | m/z | z | RT | Scan | #Spec | Start | End | PTM |
| --- | --- | --- | --- | --- | --- | --- | --- | --- | --- | --- | --- | --- |
| A.IIDQDNSGFIEEEELK.L | N | 67.46 | 1877.8894 | -1.0 | 939.9510 | 2 | 12.28 | 1724 | 1 | 50 | 65 |  |
| A.GDSDGDGKIGVDEF.A | N | 35.47 | 1409.5946 | -1.1 | 705.8038 | 2 | 11.99 | 1675 | 1 | 90 | 103 |  |
| S.GFIEEEELK.L | N | 31.42 | 1092.5338 | -0.1 | 547.2741 | 2 | 11.80 | 1641 | 1 | 57 | 65 |  |
| A.IIDQDNSGFIEEEELKLF.L | N | 30.80 | 2138.0417 | 0.6 | 1070.0288 | 2 | 14.58 | 2119 | 1 | 50 | 67 |  |
| F.AIIDQDNSGFIE.E | N | 26.76 | 1320.6198 | -0.6 | 661.3168 | 2 | 12.86 | 1825 | 1 | 49 | 60 |  |
| F.LAAGDSDGDGKIGVD.E | N | 25.04 | 1388.6418 | -0.5 | 695.3278 | 2 | 10.74 | 1456 | 1 | 87 | 101 |  |
| A.IIDQDNSGFIEEEE.L | N | 16.99 | 1636.7103 | 0.3 | 819.3627 | 2 | 12.36 | 1738 | 1 | 50 | 63 |  |
| total 7 peptides |  |  |  |  |  |  |  |  |  |  |  |  |
| --- | --- | --- | --- | --- | --- | --- | --- | --- | --- | --- | --- | --- |

tr|A0A672Y4P6|A0A672Y4P6\_9TELE

back to list

  

| Protein Coverage
| Supporting Peptides
|

Protein Coverage:

Supporting Peptides:

| Peptide | Uniq | -10lgP | Mass | ppm | m/z | z | RT | Scan | #Spec | Start | End | PTM |
| --- | --- | --- | --- | --- | --- | --- | --- | --- | --- | --- | --- | --- |
| M.LGETNPADSKPGSIR.G | Y | 61.86 | 1540.7844 | 4.9 | 771.4033 | 2 | 10.00 | 1349 | 1 | 88 | 102 |  |
| H.YLDLKDMPFYA.G | Y | 42.76 | 1374.6530 | -0.5 | 688.3334 | 2 | 14.24 | 2059 | 1 | 49 | 59 |  |
| H.YLDLKDMPFYAGL.C | Y | 24.94 | 1544.7584 | -0.8 | 773.3859 | 2 | 14.97 | 2184 | 1 | 49 | 61 |  |
| E.VDLWFK.A | Y | 19.10 | 806.4326 | -0.8 | 404.2233 | 2 | 13.17 | 1879 | 1 | 127 | 132 |  |
| Y.LDLKDMPFY.A | Y | 18.92 | 1140.5525 | 0.0 | 571.2835 | 2 | 14.02 | 2023 | 1 | 50 | 58 |  |
| E.VDLWFKA.E | Y | 17.14 | 877.4697 | -1.0 | 439.7417 | 2 | 13.37 | 1915 | 1 | 127 | 133 |  |
| total 6 peptides |  |  |  |  |  |  |  |  |  |  |  |  |
| --- | --- | --- | --- | --- | --- | --- | --- | --- | --- | --- | --- | --- |

tr|A0A2I4CF45|A0A2I4CF45\_9TELE

back to list

  

| Protein Coverage
| Supporting Peptides
|

Protein Coverage:

Supporting Peptides:

| Peptide | Uniq | -10lgP | Mass | ppm | m/z | z | RT | Scan | #Spec | Start | End | PTM |
| --- | --- | --- | --- | --- | --- | --- | --- | --- | --- | --- | --- | --- |
| M.LGETNPADSKPGSIR.G | Y | 61.86 | 1540.7844 | 4.9 | 771.4033 | 2 | 10.00 | 1349 | 1 | 88 | 102 |  |
| H.YLDLKDMPFYA.G | Y | 42.76 | 1374.6530 | -0.5 | 688.3334 | 2 | 14.24 | 2059 | 1 | 49 | 59 |  |
| H.YLDLKDMPFYAGL.C | Y | 24.94 | 1544.7584 | -0.8 | 773.3859 | 2 | 14.97 | 2184 | 1 | 49 | 61 |  |
| E.VDLWFK.A | Y | 19.10 | 806.4326 | -0.8 | 404.2233 | 2 | 13.17 | 1879 | 1 | 127 | 132 |  |
| Y.LDLKDMPFY.A | Y | 18.92 | 1140.5525 | 0.0 | 571.2835 | 2 | 14.02 | 2023 | 1 | 50 | 58 |  |
| E.VDLWFKA.E | Y | 17.14 | 877.4697 | -1.0 | 439.7417 | 2 | 13.37 | 1915 | 1 | 127 | 133 |  |
| total 6 peptides |  |  |  |  |  |  |  |  |  |  |  |  |
| --- | --- | --- | --- | --- | --- | --- | --- | --- | --- | --- | --- | --- |

tr|A0A6P7ISB4|A0A6P7ISB4\_9TELE

back to list

  

| Protein Coverage
| Supporting Peptides
|

Protein Coverage:

Supporting Peptides:

| Peptide | Uniq | -10lgP | Mass | ppm | m/z | z | RT | Scan | #Spec | Start | End | PTM |
| --- | --- | --- | --- | --- | --- | --- | --- | --- | --- | --- | --- | --- |
| M.LGETNPADSKPGSIR.G | Y | 61.86 | 1540.7844 | 4.9 | 771.4033 | 2 | 10.00 | 1349 | 1 | 88 | 102 |  |
| H.YLDLKDMPFYA.G | Y | 42.76 | 1374.6530 | -0.5 | 688.3334 | 2 | 14.24 | 2059 | 1 | 49 | 59 |  |
| H.YLDLKDMPFYAGL.C | Y | 24.94 | 1544.7584 | -0.8 | 773.3859 | 2 | 14.97 | 2184 | 1 | 49 | 61 |  |
| E.VDLWFK.A | Y | 19.10 | 806.4326 | -0.8 | 404.2233 | 2 | 13.17 | 1879 | 1 | 127 | 132 |  |
| Y.LDLKDMPFY.A | Y | 18.92 | 1140.5525 | 0.0 | 571.2835 | 2 | 14.02 | 2023 | 1 | 50 | 58 |  |
| E.VDLWFKA.E | Y | 17.14 | 877.4697 | -1.0 | 439.7417 | 2 | 13.37 | 1915 | 1 | 127 | 133 |  |
| total 6 peptides |  |  |  |  |  |  |  |  |  |  |  |  |
| --- | --- | --- | --- | --- | --- | --- | --- | --- | --- | --- | --- | --- |

tr|A0A3Q4I698|A0A3Q4I698\_NEOBR

back to list

  

| Protein Coverage
| Supporting Peptides
|

Protein Coverage:

Supporting Peptides:

| Peptide | Uniq | -10lgP | Mass | ppm | m/z | z | RT | Scan | #Spec | Start | End | PTM |
| --- | --- | --- | --- | --- | --- | --- | --- | --- | --- | --- | --- | --- |
| M.LGETNPADSKPGSIR.G | Y | 61.86 | 1540.7844 | 4.9 | 771.4033 | 2 | 10.00 | 1349 | 1 | 88 | 102 |  |
| H.YLDLKDMPFYA.G | Y | 42.76 | 1374.6530 | -0.5 | 688.3334 | 2 | 14.24 | 2059 | 1 | 49 | 59 |  |
| H.YLDLKDMPFYAGL.C | Y | 24.94 | 1544.7584 | -0.8 | 773.3859 | 2 | 14.97 | 2184 | 1 | 49 | 61 |  |
| E.VDLWFK.A | Y | 19.10 | 806.4326 | -0.8 | 404.2233 | 2 | 13.17 | 1879 | 1 | 127 | 132 |  |
| Y.LDLKDMPFY.A | Y | 18.92 | 1140.5525 | 0.0 | 571.2835 | 2 | 14.02 | 2023 | 1 | 50 | 58 |  |
| E.VDLWFKA.E | Y | 17.14 | 877.4697 | -1.0 | 439.7417 | 2 | 13.37 | 1915 | 1 | 127 | 133 |  |
| total 6 peptides |  |  |  |  |  |  |  |  |  |  |  |  |
| --- | --- | --- | --- | --- | --- | --- | --- | --- | --- | --- | --- | --- |

tr|A0A3B3Z6H5|A0A3B3Z6H5\_9TELE

back to list

  

| Protein Coverage
| Supporting Peptides
|

Protein Coverage:

Supporting Peptides:

| Peptide | Uniq | -10lgP | Mass | ppm | m/z | z | RT | Scan | #Spec | Start | End | PTM |
| --- | --- | --- | --- | --- | --- | --- | --- | --- | --- | --- | --- | --- |
| M.LGETNPADSKPGSIR.G | Y | 61.86 | 1540.7844 | 4.9 | 771.4033 | 2 | 10.00 | 1349 | 1 | 88 | 102 |  |
| H.YLDLKDMPFYA.G | Y | 42.76 | 1374.6530 | -0.5 | 688.3334 | 2 | 14.24 | 2059 | 1 | 49 | 59 |  |
| H.YLDLKDMPFYAGL.C | Y | 24.94 | 1544.7584 | -0.8 | 773.3859 | 2 | 14.97 | 2184 | 1 | 49 | 61 |  |
| E.VDLWFK.A | Y | 19.10 | 806.4326 | -0.8 | 404.2233 | 2 | 13.17 | 1879 | 1 | 127 | 132 |  |
| Y.LDLKDMPFY.A | Y | 18.92 | 1140.5525 | 0.0 | 571.2835 | 2 | 14.02 | 2023 | 1 | 50 | 58 |  |
| E.VDLWFKA.E | Y | 17.14 | 877.4697 | -1.0 | 439.7417 | 2 | 13.37 | 1915 | 1 | 127 | 133 |  |
| total 6 peptides |  |  |  |  |  |  |  |  |  |  |  |  |
| --- | --- | --- | --- | --- | --- | --- | --- | --- | --- | --- | --- | --- |

tr|A0A3Q3A341|A0A3Q3A341\_KRYMA

back to list

  

| Protein Coverage
| Supporting Peptides
|

Protein Coverage:

Supporting Peptides:

| Peptide | Uniq | -10lgP | Mass | ppm | m/z | z | RT | Scan | #Spec | Start | End | PTM |
| --- | --- | --- | --- | --- | --- | --- | --- | --- | --- | --- | --- | --- |
| M.LGETNPADSKPGSIR.G | Y | 61.86 | 1540.7844 | 4.9 | 771.4033 | 2 | 10.00 | 1349 | 1 | 88 | 102 |  |
| H.YLDLKDMPFYA.G | Y | 42.76 | 1374.6530 | -0.5 | 688.3334 | 2 | 14.24 | 2059 | 1 | 49 | 59 |  |
| H.YLDLKDMPFYAGL.C | Y | 24.94 | 1544.7584 | -0.8 | 773.3859 | 2 | 14.97 | 2184 | 1 | 49 | 61 |  |
| E.VDLWFK.A | Y | 19.10 | 806.4326 | -0.8 | 404.2233 | 2 | 13.17 | 1879 | 1 | 127 | 132 |  |
| Y.LDLKDMPFY.A | Y | 18.92 | 1140.5525 | 0.0 | 571.2835 | 2 | 14.02 | 2023 | 1 | 50 | 58 |  |
| E.VDLWFKA.E | Y | 17.14 | 877.4697 | -1.0 | 439.7417 | 2 | 13.37 | 1915 | 1 | 127 | 133 |  |
| total 6 peptides |  |  |  |  |  |  |  |  |  |  |  |  |
| --- | --- | --- | --- | --- | --- | --- | --- | --- | --- | --- | --- | --- |

tr|A0A3P8Q104|A0A3P8Q104\_ASTCA

back to list

  

| Protein Coverage
| Supporting Peptides
|

Protein Coverage:

Supporting Peptides:

| Peptide | Uniq | -10lgP | Mass | ppm | m/z | z | RT | Scan | #Spec | Start | End | PTM |
| --- | --- | --- | --- | --- | --- | --- | --- | --- | --- | --- | --- | --- |
| M.LGETNPADSKPGSIR.G | Y | 61.86 | 1540.7844 | 4.9 | 771.4033 | 2 | 10.00 | 1349 | 1 | 90 | 104 |  |
| H.YLDLKDMPFYA.G | Y | 42.76 | 1374.6530 | -0.5 | 688.3334 | 2 | 14.24 | 2059 | 1 | 51 | 61 |  |
| H.YLDLKDMPFYAGL.C | Y | 24.94 | 1544.7584 | -0.8 | 773.3859 | 2 | 14.97 | 2184 | 1 | 51 | 63 |  |
| E.VDLWFK.A | Y | 19.10 | 806.4326 | -0.8 | 404.2233 | 2 | 13.17 | 1879 | 1 | 129 | 134 |  |
| Y.LDLKDMPFY.A | Y | 18.92 | 1140.5525 | 0.0 | 571.2835 | 2 | 14.02 | 2023 | 1 | 52 | 60 |  |
| E.VDLWFKA.E | Y | 17.14 | 877.4697 | -1.0 | 439.7417 | 2 | 13.37 | 1915 | 1 | 129 | 135 |  |
| total 6 peptides |  |  |  |  |  |  |  |  |  |  |  |  |
| --- | --- | --- | --- | --- | --- | --- | --- | --- | --- | --- | --- | --- |

tr|A0A3Q3CUD8|A0A3Q3CUD8\_HAPBU

back to list

  

| Protein Coverage
| Supporting Peptides
|

Protein Coverage:

Supporting Peptides:

| Peptide | Uniq | -10lgP | Mass | ppm | m/z | z | RT | Scan | #Spec | Start | End | PTM |
| --- | --- | --- | --- | --- | --- | --- | --- | --- | --- | --- | --- | --- |
| M.LGETNPADSKPGSIR.G | Y | 61.86 | 1540.7844 | 4.9 | 771.4033 | 2 | 10.00 | 1349 | 1 | 90 | 104 |  |
| H.YLDLKDMPFYA.G | Y | 42.76 | 1374.6530 | -0.5 | 688.3334 | 2 | 14.24 | 2059 | 1 | 51 | 61 |  |
| H.YLDLKDMPFYAGL.C | Y | 24.94 | 1544.7584 | -0.8 | 773.3859 | 2 | 14.97 | 2184 | 1 | 51 | 63 |  |
| E.VDLWFK.A | Y | 19.10 | 806.4326 | -0.8 | 404.2233 | 2 | 13.17 | 1879 | 1 | 129 | 134 |  |
| Y.LDLKDMPFY.A | Y | 18.92 | 1140.5525 | 0.0 | 571.2835 | 2 | 14.02 | 2023 | 1 | 52 | 60 |  |
| E.VDLWFKA.E | Y | 17.14 | 877.4697 | -1.0 | 439.7417 | 2 | 13.37 | 1915 | 1 | 129 | 135 |  |
| total 6 peptides |  |  |  |  |  |  |  |  |  |  |  |  |
| --- | --- | --- | --- | --- | --- | --- | --- | --- | --- | --- | --- | --- |

tr|A0A3Q4I6B8|A0A3Q4I6B8\_NEOBR

back to list

  

| Protein Coverage
| Supporting Peptides
|

Protein Coverage:

Supporting Peptides:

| Peptide | Uniq | -10lgP | Mass | ppm | m/z | z | RT | Scan | #Spec | Start | End | PTM |
| --- | --- | --- | --- | --- | --- | --- | --- | --- | --- | --- | --- | --- |
| M.LGETNPADSKPGSIR.G | Y | 61.86 | 1540.7844 | 4.9 | 771.4033 | 2 | 10.00 | 1349 | 1 | 90 | 104 |  |
| H.YLDLKDMPFYA.G | Y | 42.76 | 1374.6530 | -0.5 | 688.3334 | 2 | 14.24 | 2059 | 1 | 51 | 61 |  |
| H.YLDLKDMPFYAGL.C | Y | 24.94 | 1544.7584 | -0.8 | 773.3859 | 2 | 14.97 | 2184 | 1 | 51 | 63 |  |
| E.VDLWFK.A | Y | 19.10 | 806.4326 | -0.8 | 404.2233 | 2 | 13.17 | 1879 | 1 | 129 | 134 |  |
| Y.LDLKDMPFY.A | Y | 18.92 | 1140.5525 | 0.0 | 571.2835 | 2 | 14.02 | 2023 | 1 | 52 | 60 |  |
| E.VDLWFKA.E | Y | 17.14 | 877.4697 | -1.0 | 439.7417 | 2 | 13.37 | 1915 | 1 | 129 | 135 |  |
| total 6 peptides |  |  |  |  |  |  |  |  |  |  |  |  |
| --- | --- | --- | --- | --- | --- | --- | --- | --- | --- | --- | --- | --- |

tr|M4ADR4|M4ADR4\_XIPMA

back to list

  

| Protein Coverage
| Supporting Peptides
|

Protein Coverage:

Supporting Peptides:

| Peptide | Uniq | -10lgP | Mass | ppm | m/z | z | RT | Scan | #Spec | Start | End | PTM |
| --- | --- | --- | --- | --- | --- | --- | --- | --- | --- | --- | --- | --- |
| M.LGETNPADSKPGSIR.G | Y | 61.86 | 1540.7844 | 4.9 | 771.4033 | 2 | 10.00 | 1349 | 1 | 101 | 115 |  |
| H.YLDLKDMPFYA.G | Y | 42.76 | 1374.6530 | -0.5 | 688.3334 | 2 | 14.24 | 2059 | 1 | 62 | 72 |  |
| H.YLDLKDMPFYAGL.C | Y | 24.94 | 1544.7584 | -0.8 | 773.3859 | 2 | 14.97 | 2184 | 1 | 62 | 74 |  |
| E.VDLWFK.A | Y | 19.10 | 806.4326 | -0.8 | 404.2233 | 2 | 13.17 | 1879 | 1 | 140 | 145 |  |
| Y.LDLKDMPFY.A | Y | 18.92 | 1140.5525 | 0.0 | 571.2835 | 2 | 14.02 | 2023 | 1 | 63 | 71 |  |
| E.VDLWFKA.E | Y | 17.14 | 877.4697 | -1.0 | 439.7417 | 2 | 13.37 | 1915 | 1 | 140 | 146 |  |
| total 6 peptides |  |  |  |  |  |  |  |  |  |  |  |  |
| --- | --- | --- | --- | --- | --- | --- | --- | --- | --- | --- | --- | --- |

tr|A0A3B3Z5H2|A0A3B3Z5H2\_9TELE

back to list

  

| Protein Coverage
| Supporting Peptides
|

Protein Coverage:

Supporting Peptides:

| Peptide | Uniq | -10lgP | Mass | ppm | m/z | z | RT | Scan | #Spec | Start | End | PTM |
| --- | --- | --- | --- | --- | --- | --- | --- | --- | --- | --- | --- | --- |
| M.LGETNPADSKPGSIR.G | Y | 61.86 | 1540.7844 | 4.9 | 771.4033 | 2 | 10.00 | 1349 | 1 | 102 | 116 |  |
| H.YLDLKDMPFYA.G | Y | 42.76 | 1374.6530 | -0.5 | 688.3334 | 2 | 14.24 | 2059 | 1 | 63 | 73 |  |
| H.YLDLKDMPFYAGL.C | Y | 24.94 | 1544.7584 | -0.8 | 773.3859 | 2 | 14.97 | 2184 | 1 | 63 | 75 |  |
| E.VDLWFK.A | Y | 19.10 | 806.4326 | -0.8 | 404.2233 | 2 | 13.17 | 1879 | 1 | 141 | 146 |  |
| Y.LDLKDMPFY.A | Y | 18.92 | 1140.5525 | 0.0 | 571.2835 | 2 | 14.02 | 2023 | 1 | 64 | 72 |  |
| E.VDLWFKA.E | Y | 17.14 | 877.4697 | -1.0 | 439.7417 | 2 | 13.37 | 1915 | 1 | 141 | 147 |  |
| total 6 peptides |  |  |  |  |  |  |  |  |  |  |  |  |
| --- | --- | --- | --- | --- | --- | --- | --- | --- | --- | --- | --- | --- |

tr|A0A087YID2|A0A087YID2\_POEFO

back to list

  

| Protein Coverage
| Supporting Peptides
|

Protein Coverage:

Supporting Peptides:

| Peptide | Uniq | -10lgP | Mass | ppm | m/z | z | RT | Scan | #Spec | Start | End | PTM |
| --- | --- | --- | --- | --- | --- | --- | --- | --- | --- | --- | --- | --- |
| M.LGETNPADSKPGSIR.G | Y | 61.86 | 1540.7844 | 4.9 | 771.4033 | 2 | 10.00 | 1349 | 1 | 102 | 116 |  |
| H.YLDLKDMPFYA.G | Y | 42.76 | 1374.6530 | -0.5 | 688.3334 | 2 | 14.24 | 2059 | 1 | 63 | 73 |  |
| H.YLDLKDMPFYAGL.C | Y | 24.94 | 1544.7584 | -0.8 | 773.3859 | 2 | 14.97 | 2184 | 1 | 63 | 75 |  |
| E.VDLWFK.A | Y | 19.10 | 806.4326 | -0.8 | 404.2233 | 2 | 13.17 | 1879 | 1 | 141 | 146 |  |
| Y.LDLKDMPFY.A | Y | 18.92 | 1140.5525 | 0.0 | 571.2835 | 2 | 14.02 | 2023 | 1 | 64 | 72 |  |
| E.VDLWFKA.E | Y | 17.14 | 877.4697 | -1.0 | 439.7417 | 2 | 13.37 | 1915 | 1 | 141 | 147 |  |
| total 6 peptides |  |  |  |  |  |  |  |  |  |  |  |  |
| --- | --- | --- | --- | --- | --- | --- | --- | --- | --- | --- | --- | --- |

tr|A0A3Q2PNK7|A0A3Q2PNK7\_FUNHE

back to list

  

| Protein Coverage
| Supporting Peptides
|

Protein Coverage:

Supporting Peptides:

| Peptide | Uniq | -10lgP | Mass | ppm | m/z | z | RT | Scan | #Spec | Start | End | PTM |
| --- | --- | --- | --- | --- | --- | --- | --- | --- | --- | --- | --- | --- |
| M.LGETNPADSKPGSIR.G | Y | 61.86 | 1540.7844 | 4.9 | 771.4033 | 2 | 10.00 | 1349 | 1 | 181 | 195 |  |
| H.YLDLKDMPFYA.G | Y | 42.76 | 1374.6530 | -0.5 | 688.3334 | 2 | 14.24 | 2059 | 1 | 142 | 152 |  |
| H.YLDLKDMPFYAGL.C | Y | 24.94 | 1544.7584 | -0.8 | 773.3859 | 2 | 14.97 | 2184 | 1 | 142 | 154 |  |
| E.VDLWFK.A | Y | 19.10 | 806.4326 | -0.8 | 404.2233 | 2 | 13.17 | 1879 | 1 | 220 | 225 |  |
| Y.LDLKDMPFY.A | Y | 18.92 | 1140.5525 | 0.0 | 571.2835 | 2 | 14.02 | 2023 | 1 | 143 | 151 |  |
| E.VDLWFKA.E | Y | 17.14 | 877.4697 | -1.0 | 439.7417 | 2 | 13.37 | 1915 | 1 | 220 | 226 |  |
| total 6 peptides |  |  |  |  |  |  |  |  |  |  |  |  |
| --- | --- | --- | --- | --- | --- | --- | --- | --- | --- | --- | --- | --- |

tr|A0A4W4EA72|A0A4W4EA72\_ELEEL

back to list

  

| Protein Coverage
| Supporting Peptides
|

Protein Coverage:

Supporting Peptides:

| Peptide | Uniq | -10lgP | Mass | ppm | m/z | z | RT | Scan | #Spec | Start | End | PTM |
| --- | --- | --- | --- | --- | --- | --- | --- | --- | --- | --- | --- | --- |
| T.VLDRDKSGFIEEEELK.L | Y | 60.50 | 1905.9683 | -23.1 | 953.9694 | 2 | 11.67 | 1618 | 2 | 50 | 65 |  |
| F.TVLDRDKSGFIEEEE.L | Y | 32.23 | 1765.8369 | -27.6 | 883.9014 | 2 | 12.46 | 1756 | 1 | 49 | 63 |  |
| S.GFIEEEELK.L | N | 31.42 | 1092.5338 | -0.1 | 547.2741 | 2 | 11.80 | 1641 | 1 | 57 | 65 |  |
| R.DKSGFIEEEELK.L | N | 25.85 | 1422.6877 | -0.4 | 712.3508 | 2 | 11.40 | 1570 | 1 | 54 | 65 |  |
| F.TVLDRDKSGFIE.E | Y | 20.41 | 1378.7092 | -34.7 | 690.3380 | 2 | 15.47 | 2282 | 4 | 49 | 60 |  |
| T.VLDRDKSGFIEEEE.L | Y | 17.77 | 1664.7893 | -22.2 | 833.3835 | 2 | 11.56 | 1599 | 1 | 50 | 63 |  |
| T.VLDRDKSGFIEEEEL.K | Y | 15.17 | 1777.8733 | -21.4 | 889.9249 | 2 | 12.64 | 1787 | 1 | 50 | 64 |  |
| total 7 peptides |  |  |  |  |  |  |  |  |  |  |  |  |
| --- | --- | --- | --- | --- | --- | --- | --- | --- | --- | --- | --- | --- |

tr|A0A3B4TLJ3|A0A3B4TLJ3\_SERDU

back to list

  

| Protein Coverage
| Supporting Peptides
|

Protein Coverage:

Supporting Peptides:

| Peptide | Uniq | -10lgP | Mass | ppm | m/z | z | RT | Scan | #Spec | Start | End | PTM |
| --- | --- | --- | --- | --- | --- | --- | --- | --- | --- | --- | --- | --- |
| A.IIDQDKSGFIEEEELK.L | N | 49.57 | 1891.9414 | -0.6 | 946.9774 | 2 | 11.72 | 1627 | 1 | 46 | 61 |  |
| A.IIDQDKSGFIE.E | N | 44.01 | 1263.6346 | -0.5 | 632.8243 | 2 | 11.60 | 1605 | 1 | 46 | 56 |  |
| A.GDSDGDGKIGVDEF.A | N | 35.47 | 1409.5946 | -1.1 | 705.8038 | 2 | 11.99 | 1675 | 1 | 86 | 99 |  |
| S.GFIEEEELK.L | N | 31.42 | 1092.5338 | -0.1 | 547.2741 | 2 | 11.80 | 1641 | 1 | 53 | 61 |  |
| Q.DKSGFIEEEELK.L | N | 25.85 | 1422.6877 | -0.4 | 712.3508 | 2 | 11.40 | 1570 | 1 | 50 | 61 |  |
| total 5 peptides |  |  |  |  |  |  |  |  |  |  |  |  |
| --- | --- | --- | --- | --- | --- | --- | --- | --- | --- | --- | --- | --- |

tr|A9ZTF1|A9ZTF1\_KATPE

back to list

  

| Protein Coverage
| Supporting Peptides
|

Protein Coverage:

Supporting Peptides:

| Peptide | Uniq | -10lgP | Mass | ppm | m/z | z | RT | Scan | #Spec | Start | End | PTM |
| --- | --- | --- | --- | --- | --- | --- | --- | --- | --- | --- | --- | --- |
| A.IIDQDKSGFIEEEELK.L | N | 49.57 | 1891.9414 | -0.6 | 946.9774 | 2 | 11.72 | 1627 | 1 | 49 | 64 |  |
| A.IIDQDKSGFIE.E | N | 44.01 | 1263.6346 | -0.5 | 632.8243 | 2 | 11.60 | 1605 | 1 | 49 | 59 |  |
| A.GDSDGDGKIGVDEF.A | N | 35.47 | 1409.5946 | -1.1 | 705.8038 | 2 | 11.99 | 1675 | 1 | 89 | 102 |  |
| S.GFIEEEELK.L | N | 31.42 | 1092.5338 | -0.1 | 547.2741 | 2 | 11.80 | 1641 | 1 | 56 | 64 |  |
| Q.DKSGFIEEEELK.L | N | 25.85 | 1422.6877 | -0.4 | 712.3508 | 2 | 11.40 | 1570 | 1 | 53 | 64 |  |
| total 5 peptides |  |  |  |  |  |  |  |  |  |  |  |  |
| --- | --- | --- | --- | --- | --- | --- | --- | --- | --- | --- | --- | --- |

tr|G8GWA1|G8GWA1\_CARAU

back to list

  

| Protein Coverage
| Supporting Peptides
|

Protein Coverage:

Supporting Peptides:

| Peptide | Uniq | -10lgP | Mass | ppm | m/z | z | RT | Scan | #Spec | Start | End | PTM |
| --- | --- | --- | --- | --- | --- | --- | --- | --- | --- | --- | --- | --- |
| A.IIDQDKSGFIEEEELK.L | N | 49.57 | 1891.9414 | -0.6 | 946.9774 | 2 | 11.72 | 1627 | 1 | 50 | 65 |  |
| A.IIDQDKSGFIE.E | N | 44.01 | 1263.6346 | -0.5 | 632.8243 | 2 | 11.60 | 1605 | 1 | 50 | 60 |  |
| A.GDSDGDGKIGVDEF.T | N | 35.47 | 1409.5946 | -1.1 | 705.8038 | 2 | 11.99 | 1675 | 1 | 90 | 103 |  |
| S.GFIEEEELK.L | N | 31.42 | 1092.5338 | -0.1 | 547.2741 | 2 | 11.80 | 1641 | 1 | 57 | 65 |  |
| Q.DKSGFIEEEELK.L | N | 25.85 | 1422.6877 | -0.4 | 712.3508 | 2 | 11.40 | 1570 | 1 | 54 | 65 |  |
| total 5 peptides |  |  |  |  |  |  |  |  |  |  |  |  |
| --- | --- | --- | --- | --- | --- | --- | --- | --- | --- | --- | --- | --- |

tr|A0A3B4TM81|A0A3B4TM81\_SERDU

back to list

  

| Protein Coverage
| Supporting Peptides
|

Protein Coverage:

Supporting Peptides:

| Peptide | Uniq | -10lgP | Mass | ppm | m/z | z | RT | Scan | #Spec | Start | End | PTM |
| --- | --- | --- | --- | --- | --- | --- | --- | --- | --- | --- | --- | --- |
| A.IIDQDKSGFIEEEELK.L | N | 49.57 | 1891.9414 | -0.6 | 946.9774 | 2 | 11.72 | 1627 | 1 | 50 | 65 |  |
| A.IIDQDKSGFIE.E | N | 44.01 | 1263.6346 | -0.5 | 632.8243 | 2 | 11.60 | 1605 | 1 | 50 | 60 |  |
| A.GDSDGDGKIGVDEF.A | N | 35.47 | 1409.5946 | -1.1 | 705.8038 | 2 | 11.99 | 1675 | 1 | 90 | 103 |  |
| S.GFIEEEELK.L | N | 31.42 | 1092.5338 | -0.1 | 547.2741 | 2 | 11.80 | 1641 | 1 | 57 | 65 |  |
| Q.DKSGFIEEEELK.L | N | 25.85 | 1422.6877 | -0.4 | 712.3508 | 2 | 11.40 | 1570 | 1 | 54 | 65 |  |
| total 5 peptides |  |  |  |  |  |  |  |  |  |  |  |  |
| --- | --- | --- | --- | --- | --- | --- | --- | --- | --- | --- | --- | --- |

tr|A0A6P6KMF1|A0A6P6KMF1\_CARAU

back to list

  

| Protein Coverage
| Supporting Peptides
|

Protein Coverage:

Supporting Peptides:

| Peptide | Uniq | -10lgP | Mass | ppm | m/z | z | RT | Scan | #Spec | Start | End | PTM |
| --- | --- | --- | --- | --- | --- | --- | --- | --- | --- | --- | --- | --- |
| A.IIDQDKSGFIEEEELK.L | N | 49.57 | 1891.9414 | -0.6 | 946.9774 | 2 | 11.72 | 1627 | 1 | 50 | 65 |  |
| A.IIDQDKSGFIE.E | N | 44.01 | 1263.6346 | -0.5 | 632.8243 | 2 | 11.60 | 1605 | 1 | 50 | 60 |  |
| A.GDSDGDGKIGVDEF.T | N | 35.47 | 1409.5946 | -1.1 | 705.8038 | 2 | 11.99 | 1675 | 1 | 90 | 103 |  |
| S.GFIEEEELK.L | N | 31.42 | 1092.5338 | -0.1 | 547.2741 | 2 | 11.80 | 1641 | 1 | 57 | 65 |  |
| Q.DKSGFIEEEELK.L | N | 25.85 | 1422.6877 | -0.4 | 712.3508 | 2 | 11.40 | 1570 | 1 | 54 | 65 |  |
| total 5 peptides |  |  |  |  |  |  |  |  |  |  |  |  |
| --- | --- | --- | --- | --- | --- | --- | --- | --- | --- | --- | --- | --- |

tr|A0A6G0IXD3|A0A6G0IXD3\_LARCR

back to list

  

| Protein Coverage
| Supporting Peptides
|

Protein Coverage:

Supporting Peptides:

| Peptide | Uniq | -10lgP | Mass | ppm | m/z | z | RT | Scan | #Spec | Start | End | PTM |
| --- | --- | --- | --- | --- | --- | --- | --- | --- | --- | --- | --- | --- |
| L.SKIEDEQSLGAQ.L | N | 35.68 | 1303.6255 | 4.7 | 652.8231 | 2 | 10.20 | 1374 | 1 | 1059 | 1070 |  |
| K.LEQQVDDLEGSLE.Q | N | 32.76 | 1473.6835 | -0.5 | 737.8486 | 2 | 12.65 | 1789 | 1 | 994 | 1006 |  |
| K.NDENVRQ.M | N | 29.03 | 873.3940 | -0.4 | 437.7041 | 2 | 8.49 | 1190 | 1 | 1230 | 1236 |  |
| A.EWKQKYEEGQAELE.G | N | 26.99 | 1765.8158 | -0.6 | 883.9147 | 2 | 11.00 | 1500 | 1 | 1422 | 1435 |  |
| Q.GEVDDTVQEAR.N | N | 25.82 | 1217.5524 | 4.7 | 609.7863 | 2 | 10.04 | 1355 | 1 | 1706 | 1716 |  |
| K.KMEGDLNEM.E | N | 25.67 | 1065.4470 | -0.7 | 533.7304 | 2 | 11.02 | 1504 | 1 | 1584 | 1592 |  |
| K.FDKIEDM.A | N | 23.34 | 896.3950 | -0.6 | 449.2045 | 2 | 11.46 | 1581 | 2 | 86 | 92 |  |
| Q.GEVDDTVQEA.R | N | 20.03 | 1061.4513 | -0.3 | 531.7328 | 2 | 10.64 | 1439 | 1 | 1706 | 1715 |  |
| K.GFPSRIPY.A | Y | 19.70 | 935.4865 | 6.1 | 468.7534 | 2 | 12.11 | 1693 | 1 | 683 | 690 |  |
| Q.TALEEAEGTL.E | N | 19.11 | 1032.4974 | -0.7 | 517.2556 | 2 | 12.43 | 1750 | 1 | 1509 | 1518 |  |
| M.AEELKKE.Q | N | 18.25 | 845.4494 | -0.4 | 423.7318 | 2 | 8.54 | 1200 | 1 | 1733 | 1739 |  |
| M.NPPKFDKIEDM.A | N | 17.32 | 1332.6383 | -0.5 | 667.3261 | 2 | 11.50 | 1587 | 1 | 82 | 92 |  |
| total 12 peptides |  |  |  |  |  |  |  |  |  |  |  |  |
| --- | --- | --- | --- | --- | --- | --- | --- | --- | --- | --- | --- | --- |

tr|A0A6G0IXM2|A0A6G0IXM2\_LARCR

back to list

  

| Protein Coverage
| Supporting Peptides
|

Protein Coverage:

Supporting Peptides:

| Peptide | Uniq | -10lgP | Mass | ppm | m/z | z | RT | Scan | #Spec | Start | End | PTM |
| --- | --- | --- | --- | --- | --- | --- | --- | --- | --- | --- | --- | --- |
| L.SKIEDEQSLGAQ.L | N | 35.68 | 1303.6255 | 4.7 | 652.8231 | 2 | 10.20 | 1374 | 1 | 1069 | 1080 |  |
| K.LEQQVDDLEGSLE.Q | N | 32.76 | 1473.6835 | -0.5 | 737.8486 | 2 | 12.65 | 1789 | 1 | 1004 | 1016 |  |
| K.NDENVRQ.I | N | 29.03 | 873.3940 | -0.4 | 437.7041 | 2 | 8.49 | 1190 | 1 | 1240 | 1246 |  |
| A.EWKQKYEEGQAELE.G | N | 26.99 | 1765.8158 | -0.6 | 883.9147 | 2 | 11.00 | 1500 | 1 | 1432 | 1445 |  |
| Q.GEVDDTVQEAR.N | N | 25.82 | 1217.5524 | 4.7 | 609.7863 | 2 | 10.04 | 1355 | 1 | 1716 | 1726 |  |
| K.KMEGDLNEM.E | N | 25.67 | 1065.4470 | -0.7 | 533.7304 | 2 | 11.02 | 1504 | 1 | 1594 | 1602 |  |
| K.FDKIEDM.A | N | 23.34 | 896.3950 | -0.6 | 449.2045 | 2 | 11.46 | 1581 | 2 | 86 | 92 |  |
| Q.GEVDDTVQEA.R | N | 20.03 | 1061.4513 | -0.3 | 531.7328 | 2 | 10.64 | 1439 | 1 | 1716 | 1725 |  |
| K.GFPSRIPY.A | Y | 19.70 | 935.4865 | 6.1 | 468.7534 | 2 | 12.11 | 1693 | 1 | 685 | 692 |  |
| Q.TALEEAEGTL.E | N | 19.11 | 1032.4974 | -0.7 | 517.2556 | 2 | 12.43 | 1750 | 1 | 1519 | 1528 |  |
| M.AEELKKE.Q | N | 18.25 | 845.4494 | -0.4 | 423.7318 | 2 | 8.54 | 1200 | 1 | 1743 | 1749 |  |
| M.NPPKFDKIEDM.A | N | 17.32 | 1332.6383 | -0.5 | 667.3261 | 2 | 11.50 | 1587 | 1 | 82 | 92 |  |
| total 12 peptides |  |  |  |  |  |  |  |  |  |  |  |  |
| --- | --- | --- | --- | --- | --- | --- | --- | --- | --- | --- | --- | --- |

tr|A0A6G0IXG4|A0A6G0IXG4\_LARCR

back to list

  

| Protein Coverage
| Supporting Peptides
|

Protein Coverage:

Supporting Peptides:

| Peptide | Uniq | -10lgP | Mass | ppm | m/z | z | RT | Scan | #Spec | Start | End | PTM |
| --- | --- | --- | --- | --- | --- | --- | --- | --- | --- | --- | --- | --- |
| L.SKIEDEQSLGAQ.L | N | 35.68 | 1303.6255 | 4.7 | 652.8231 | 2 | 10.20 | 1374 | 1 | 1079 | 1090 |  |
| K.LEQQVDDLEGSLE.Q | N | 32.76 | 1473.6835 | -0.5 | 737.8486 | 2 | 12.65 | 1789 | 1 | 1014 | 1026 |  |
| K.NDENVRQ.M | N | 29.03 | 873.3940 | -0.4 | 437.7041 | 2 | 8.49 | 1190 | 1 | 1250 | 1256 |  |
| A.EWKQKYEEGQAELE.G | N | 26.99 | 1765.8158 | -0.6 | 883.9147 | 2 | 11.00 | 1500 | 1 | 1442 | 1455 |  |
| Q.GEVDDTVQEAR.N | N | 25.82 | 1217.5524 | 4.7 | 609.7863 | 2 | 10.04 | 1355 | 1 | 1726 | 1736 |  |
| K.KMEGDLNEM.E | N | 25.67 | 1065.4470 | -0.7 | 533.7304 | 2 | 11.02 | 1504 | 1 | 1604 | 1612 |  |
| K.FDKIEDM.A | N | 23.34 | 896.3950 | -0.6 | 449.2045 | 2 | 11.46 | 1581 | 2 | 86 | 92 |  |
| Q.GEVDDTVQEA.R | N | 20.03 | 1061.4513 | -0.3 | 531.7328 | 2 | 10.64 | 1439 | 1 | 1726 | 1735 |  |
| K.GFPSRIPY.A | Y | 19.70 | 935.4865 | 6.1 | 468.7534 | 2 | 12.11 | 1693 | 1 | 703 | 710 |  |
| Q.TALEEAEGTL.E | N | 19.11 | 1032.4974 | -0.7 | 517.2556 | 2 | 12.43 | 1750 | 1 | 1529 | 1538 |  |
| M.AEELKKE.Q | N | 18.25 | 845.4494 | -0.4 | 423.7318 | 2 | 8.54 | 1200 | 1 | 1753 | 1759 |  |
| M.NPPKFDKIEDM.A | N | 17.32 | 1332.6383 | -0.5 | 667.3261 | 2 | 11.50 | 1587 | 1 | 82 | 92 |  |
| total 12 peptides |  |  |  |  |  |  |  |  |  |  |  |  |
| --- | --- | --- | --- | --- | --- | --- | --- | --- | --- | --- | --- | --- |

tr|G3NEV3|G3NEV3\_GASAC

back to list

  

| Protein Coverage
| Supporting Peptides
|

Protein Coverage:

Supporting Peptides:

| Peptide | Uniq | -10lgP | Mass | ppm | m/z | z | RT | Scan | #Spec | Start | End | PTM |
| --- | --- | --- | --- | --- | --- | --- | --- | --- | --- | --- | --- | --- |
| L.SKIEDEQSLGAQ.L | N | 35.68 | 1303.6255 | 4.7 | 652.8231 | 2 | 10.20 | 1374 | 1 | 1082 | 1093 |  |
| K.LEQQVDDLEGSLE.Q | N | 32.76 | 1473.6835 | -0.5 | 737.8486 | 2 | 12.65 | 1789 | 1 | 1017 | 1029 |  |
| K.NDENVRQ.I | N | 29.03 | 873.3940 | -0.4 | 437.7041 | 2 | 8.49 | 1190 | 1 | 1253 | 1259 |  |
| A.EWKQKYEEGQAELE.G | N | 26.99 | 1765.8158 | -0.6 | 883.9147 | 2 | 11.00 | 1500 | 1 | 1447 | 1460 |  |
| Q.GEVDDTVQEAR.N | N | 25.82 | 1217.5524 | 4.7 | 609.7863 | 2 | 10.04 | 1355 | 1 | 1731 | 1741 |  |
| K.KMEGDLNEM.E | N | 25.67 | 1065.4470 | -0.7 | 533.7304 | 2 | 11.02 | 1504 | 1 | 1609 | 1617 |  |
| E.LKKEQDTSAH.L | N | 22.49 | 1155.5884 | -0.2 | 578.8013 | 2 | 7.77 | 1050 | 1 | 1761 | 1770 |  |
| Q.GEVDDTVQEA.R | N | 20.03 | 1061.4513 | -0.3 | 531.7328 | 2 | 10.64 | 1439 | 1 | 1731 | 1740 |  |
| Q.TALEEAEGTL.E | N | 19.11 | 1032.4974 | -0.7 | 517.2556 | 2 | 12.43 | 1750 | 1 | 1534 | 1543 |  |
| M.AEELKKE.Q | N | 18.25 | 845.4494 | -0.4 | 423.7318 | 2 | 8.54 | 1200 | 1 | 1758 | 1764 |  |
| total 10 peptides |  |  |  |  |  |  |  |  |  |  |  |  |
| --- | --- | --- | --- | --- | --- | --- | --- | --- | --- | --- | --- | --- |

tr|A0A6P8W5C3|A0A6P8W5C3\_GYMAC

back to list

  

| Protein Coverage
| Supporting Peptides
|

Protein Coverage:

Supporting Peptides:

| Peptide | Uniq | -10lgP | Mass | ppm | m/z | z | RT | Scan | #Spec | Start | End | PTM |
| --- | --- | --- | --- | --- | --- | --- | --- | --- | --- | --- | --- | --- |
| L.SKIEDEQSLGAQ.L | N | 35.68 | 1303.6255 | 4.7 | 652.8231 | 2 | 10.20 | 1374 | 1 | 1086 | 1097 |  |
| K.LEQQVDDLEGSLE.Q | N | 32.76 | 1473.6835 | -0.5 | 737.8486 | 2 | 12.65 | 1789 | 1 | 1021 | 1033 |  |
| K.NDENVRQ.I | N | 29.03 | 873.3940 | -0.4 | 437.7041 | 2 | 8.49 | 1190 | 1 | 1257 | 1263 |  |
| A.EWKQKYEEGQAELE.G | N | 26.99 | 1765.8158 | -0.6 | 883.9147 | 2 | 11.00 | 1500 | 1 | 1449 | 1462 |  |
| Q.GEVDDTVQEAR.N | N | 25.82 | 1217.5524 | 4.7 | 609.7863 | 2 | 10.04 | 1355 | 1 | 1733 | 1743 |  |
| K.KMEGDLNEM.E | N | 25.67 | 1065.4470 | -0.7 | 533.7304 | 2 | 11.02 | 1504 | 1 | 1611 | 1619 |  |
| E.LKKEQDTSAH.L | N | 22.49 | 1155.5884 | -0.2 | 578.8013 | 2 | 7.77 | 1050 | 1 | 1763 | 1772 |  |
| Q.GEVDDTVQEA.R | N | 20.03 | 1061.4513 | -0.3 | 531.7328 | 2 | 10.64 | 1439 | 1 | 1733 | 1742 |  |
| Q.TALEEAEGTL.E | N | 19.11 | 1032.4974 | -0.7 | 517.2556 | 2 | 12.43 | 1750 | 1 | 1536 | 1545 |  |
| M.AEELKKE.Q | N | 18.25 | 845.4494 | -0.4 | 423.7318 | 2 | 8.54 | 1200 | 1 | 1760 | 1766 |  |
| total 10 peptides |  |  |  |  |  |  |  |  |  |  |  |  |
| --- | --- | --- | --- | --- | --- | --- | --- | --- | --- | --- | --- | --- |

tr|A0A6P8VS48|A0A6P8VS48\_GYMAC

back to list

  

| Protein Coverage
| Supporting Peptides
|

Protein Coverage:

Supporting Peptides:

| Peptide | Uniq | -10lgP | Mass | ppm | m/z | z | RT | Scan | #Spec | Start | End | PTM |
| --- | --- | --- | --- | --- | --- | --- | --- | --- | --- | --- | --- | --- |
| L.SKIEDEQSLGAQ.L | N | 35.68 | 1303.6255 | 4.7 | 652.8231 | 2 | 10.20 | 1374 | 1 | 1086 | 1097 |  |
| K.LEQQVDDLEGSLE.Q | N | 32.76 | 1473.6835 | -0.5 | 737.8486 | 2 | 12.65 | 1789 | 1 | 1021 | 1033 |  |
| K.NDENVRQ.I | N | 29.03 | 873.3940 | -0.4 | 437.7041 | 2 | 8.49 | 1190 | 1 | 1257 | 1263 |  |
| A.EWKQKYEEGQAELE.G | N | 26.99 | 1765.8158 | -0.6 | 883.9147 | 2 | 11.00 | 1500 | 1 | 1449 | 1462 |  |
| Q.GEVDDTVQEAR.N | N | 25.82 | 1217.5524 | 4.7 | 609.7863 | 2 | 10.04 | 1355 | 1 | 1733 | 1743 |  |
| K.KMEGDLNEM.E | N | 25.67 | 1065.4470 | -0.7 | 533.7304 | 2 | 11.02 | 1504 | 1 | 1611 | 1619 |  |
| E.LKKEQDTSAH.L | N | 22.49 | 1155.5884 | -0.2 | 578.8013 | 2 | 7.77 | 1050 | 1 | 1763 | 1772 |  |
| Q.GEVDDTVQEA.R | N | 20.03 | 1061.4513 | -0.3 | 531.7328 | 2 | 10.64 | 1439 | 1 | 1733 | 1742 |  |
| Q.TALEEAEGTL.E | N | 19.11 | 1032.4974 | -0.7 | 517.2556 | 2 | 12.43 | 1750 | 1 | 1536 | 1545 |  |
| M.AEELKKE.Q | N | 18.25 | 845.4494 | -0.4 | 423.7318 | 2 | 8.54 | 1200 | 1 | 1760 | 1766 |  |
| total 10 peptides |  |  |  |  |  |  |  |  |  |  |  |  |
| --- | --- | --- | --- | --- | --- | --- | --- | --- | --- | --- | --- | --- |

tr|A0A3Q3W464|A0A3Q3W464\_MOLML

back to list

  

| Protein Coverage
| Supporting Peptides
|

Protein Coverage:

Supporting Peptides:

| Peptide | Uniq | -10lgP | Mass | ppm | m/z | z | RT | Scan | #Spec | Start | End | PTM |
| --- | --- | --- | --- | --- | --- | --- | --- | --- | --- | --- | --- | --- |
| A.FDIIDQDKSGFIE.K | N | 51.87 | 1525.7300 | -1.4 | 763.8712 | 2 | 13.28 | 1898 | 1 | 48 | 60 |  |
| D.IIDQDKSGFIE.K | N | 44.01 | 1263.6346 | -0.5 | 632.8243 | 2 | 11.60 | 1605 | 1 | 50 | 60 |  |
| S.DELKKAFDIIDQDKSGFIE.K | Y | 33.36 | 2210.1106 | -17.3 | 1106.0435 | 2 | 13.70 | 1970 | 1 | 42 | 60 |  |
| total 3 peptides |  |  |  |  |  |  |  |  |  |  |  |  |
| --- | --- | --- | --- | --- | --- | --- | --- | --- | --- | --- | --- | --- |

tr|A0A3Q3WAG3|A0A3Q3WAG3\_MOLML

back to list

  

| Protein Coverage
| Supporting Peptides
|

Protein Coverage:

Supporting Peptides:

| Peptide | Uniq | -10lgP | Mass | ppm | m/z | z | RT | Scan | #Spec | Start | End | PTM |
| --- | --- | --- | --- | --- | --- | --- | --- | --- | --- | --- | --- | --- |
| A.FDIIDQDKSGFIE.K | N | 51.87 | 1525.7300 | -1.4 | 763.8712 | 2 | 13.28 | 1898 | 1 | 48 | 60 |  |
| D.IIDQDKSGFIE.K | N | 44.01 | 1263.6346 | -0.5 | 632.8243 | 2 | 11.60 | 1605 | 1 | 50 | 60 |  |
| S.DELKKAFDIIDQDKSGFIE.K | Y | 33.36 | 2210.1106 | -17.3 | 1106.0435 | 2 | 13.70 | 1970 | 1 | 42 | 60 |  |
| total 3 peptides |  |  |  |  |  |  |  |  |  |  |  |  |
| --- | --- | --- | --- | --- | --- | --- | --- | --- | --- | --- | --- | --- |

tr|A0A3Q4AW30|A0A3Q4AW30\_MOLML

back to list

  

| Protein Coverage
| Supporting Peptides
|

Protein Coverage:

Supporting Peptides:

| Peptide | Uniq | -10lgP | Mass | ppm | m/z | z | RT | Scan | #Spec | Start | End | PTM |
| --- | --- | --- | --- | --- | --- | --- | --- | --- | --- | --- | --- | --- |
| A.FDIIDQDKSGFIE.K | N | 51.87 | 1525.7300 | -1.4 | 763.8712 | 2 | 13.28 | 1898 | 1 | 48 | 60 |  |
| D.IIDQDKSGFIE.K | N | 44.01 | 1263.6346 | -0.5 | 632.8243 | 2 | 11.60 | 1605 | 1 | 50 | 60 |  |
| S.DELKKAFDIIDQDKSGFIE.K | Y | 33.36 | 2210.1106 | -17.3 | 1106.0435 | 2 | 13.70 | 1970 | 1 | 42 | 60 |  |
| total 3 peptides |  |  |  |  |  |  |  |  |  |  |  |  |
| --- | --- | --- | --- | --- | --- | --- | --- | --- | --- | --- | --- | --- |

tr|A0A3Q3W3Y6|A0A3Q3W3Y6\_MOLML

back to list

  

| Protein Coverage
| Supporting Peptides
|

Protein Coverage:

Supporting Peptides:

| Peptide | Uniq | -10lgP | Mass | ppm | m/z | z | RT | Scan | #Spec | Start | End | PTM |
| --- | --- | --- | --- | --- | --- | --- | --- | --- | --- | --- | --- | --- |
| A.FDIIDQDKSGFIE.K | N | 51.87 | 1525.7300 | -1.4 | 763.8712 | 2 | 13.28 | 1898 | 1 | 48 | 60 |  |
| D.IIDQDKSGFIE.K | N | 44.01 | 1263.6346 | -0.5 | 632.8243 | 2 | 11.60 | 1605 | 1 | 50 | 60 |  |
| S.DELKKAFDIIDQDKSGFIE.K | Y | 33.36 | 2210.1106 | -17.3 | 1106.0435 | 2 | 13.70 | 1970 | 1 | 42 | 60 |  |
| total 3 peptides |  |  |  |  |  |  |  |  |  |  |  |  |
| --- | --- | --- | --- | --- | --- | --- | --- | --- | --- | --- | --- | --- |

tr|A0A6P8GNA2|A0A6P8GNA2\_CLUHA

back to list

  

| Protein Coverage
| Supporting Peptides
|

Protein Coverage:

Supporting Peptides:

| Peptide | Uniq | -10lgP | Mass | ppm | m/z | z | RT | Scan | #Spec | Start | End | PTM |
| --- | --- | --- | --- | --- | --- | --- | --- | --- | --- | --- | --- | --- |
| L.SKIEDEQSLGAQ.L | N | 35.68 | 1303.6255 | 4.7 | 652.8231 | 2 | 10.20 | 1374 | 1 | 1092 | 1103 |  |
| K.LEQQVDDLEGSLE.Q | N | 32.76 | 1473.6835 | -0.5 | 737.8486 | 2 | 12.65 | 1789 | 1 | 1027 | 1039 |  |
| A.EWKQKYEEGQAELE.G | N | 26.99 | 1765.8158 | -0.6 | 883.9147 | 2 | 11.00 | 1500 | 1 | 1455 | 1468 |  |
| K.KMEGDLNEM.E | N | 25.67 | 1065.4470 | -0.7 | 533.7304 | 2 | 11.02 | 1504 | 1 | 1617 | 1625 |  |
| P.VLYPPVVEE.T | Y | 25.37 | 1043.5538 | -0.5 | 522.7839 | 2 | 12.77 | 1809 | 1 | 621 | 629 |  |
| K.FDKIEDM.A | N | 23.34 | 896.3950 | -0.6 | 449.2045 | 2 | 11.46 | 1581 | 2 | 85 | 91 |  |
| Q.TALEEAEGTL.E | N | 19.11 | 1032.4974 | -0.7 | 517.2556 | 2 | 12.43 | 1750 | 1 | 1542 | 1551 |  |
| M.AEELKKE.Q | N | 18.25 | 845.4494 | -0.4 | 423.7318 | 2 | 8.54 | 1200 | 1 | 1766 | 1772 |  |
| M.NPPKFDKIEDM.A | N | 17.32 | 1332.6383 | -0.5 | 667.3261 | 2 | 11.50 | 1587 | 1 | 81 | 91 |  |
| total 9 peptides |  |  |  |  |  |  |  |  |  |  |  |  |
| --- | --- | --- | --- | --- | --- | --- | --- | --- | --- | --- | --- | --- |

tr|A0A3Q1HW42|A0A3Q1HW42\_9TELE

back to list

  

| Protein Coverage
| Supporting Peptides
|

Protein Coverage:

Supporting Peptides:

| Peptide | Uniq | -10lgP | Mass | ppm | m/z | z | RT | Scan | #Spec | Start | End | PTM |
| --- | --- | --- | --- | --- | --- | --- | --- | --- | --- | --- | --- | --- |
| K.VDSELPVDDVFGQ.V | Y | 49.54 | 1418.6565 | -1.0 | 710.3348 | 2 | 13.82 | 1991 | 1 | 173 | 185 |  |
| P.LDTVLDMIKDA.M | N | 20.90 | 1232.6322 | -0.8 | 617.3229 | 2 | 14.44 | 2094 | 1 | 69 | 79 |  |
| L.DTVLDMIK.D | N | 17.70 | 933.4841 | -1.1 | 467.7488 | 2 | 13.23 | 1891 | 1 | 70 | 77 |  |
| D.TVLDMIKDA.M | N | 16.00 | 1004.5212 | -1.1 | 503.2673 | 2 | 13.42 | 1923 | 1 | 71 | 79 |  |
| K.VDSELPVDDVF.G | N | 15.68 | 1233.5764 | -0.5 | 617.7952 | 2 | 14.17 | 2049 | 1 | 173 | 183 |  |
| total 5 peptides |  |  |  |  |  |  |  |  |  |  |  |  |
| --- | --- | --- | --- | --- | --- | --- | --- | --- | --- | --- | --- | --- |

tr|A0A3B5BIY5|A0A3B5BIY5\_9TELE

back to list

  

| Protein Coverage
| Supporting Peptides
|

Protein Coverage:

Supporting Peptides:

| Peptide | Uniq | -10lgP | Mass | ppm | m/z | z | RT | Scan | #Spec | Start | End | PTM |
| --- | --- | --- | --- | --- | --- | --- | --- | --- | --- | --- | --- | --- |
| K.VDSELPVDDVFGQ.V | Y | 49.54 | 1418.6565 | -1.0 | 710.3348 | 2 | 13.82 | 1991 | 1 | 173 | 185 |  |
| P.LDTVLDMIKDA.M | N | 20.90 | 1232.6322 | -0.8 | 617.3229 | 2 | 14.44 | 2094 | 1 | 69 | 79 |  |
| L.DTVLDMIK.D | N | 17.70 | 933.4841 | -1.1 | 467.7488 | 2 | 13.23 | 1891 | 1 | 70 | 77 |  |
| D.TVLDMIKDA.M | N | 16.00 | 1004.5212 | -1.1 | 503.2673 | 2 | 13.42 | 1923 | 1 | 71 | 79 |  |
| K.VDSELPVDDVF.G | N | 15.68 | 1233.5764 | -0.5 | 617.7952 | 2 | 14.17 | 2049 | 1 | 173 | 183 |  |
| total 5 peptides |  |  |  |  |  |  |  |  |  |  |  |  |
| --- | --- | --- | --- | --- | --- | --- | --- | --- | --- | --- | --- | --- |

tr|A0A4U5UZN8|A0A4U5UZN8\_COLLU

back to list

  

| Protein Coverage
| Supporting Peptides
|

Protein Coverage:

Supporting Peptides:

| Peptide | Uniq | -10lgP | Mass | ppm | m/z | z | RT | Scan | #Spec | Start | End | PTM |
| --- | --- | --- | --- | --- | --- | --- | --- | --- | --- | --- | --- | --- |
| F.MDDPVVIPGKPY.S | Y | 42.46 | 1329.6638 | -0.6 | 665.8388 | 2 | 12.27 | 1723 | 1 | 225 | 236 |  |
| D.VIEDPVEIIDN.S | Y | 35.61 | 1254.6343 | -0.3 | 628.3242 | 2 | 13.52 | 1941 | 1 | 145 | 155 |  |
| D.VIEDPVEIIDNSRE.L | Y | 22.59 | 1626.8101 | -0.5 | 814.4119 | 2 | 13.15 | 1877 | 1 | 145 | 158 |  |
| total 3 peptides |  |  |  |  |  |  |  |  |  |  |  |  |
| --- | --- | --- | --- | --- | --- | --- | --- | --- | --- | --- | --- | --- |

tr|A0A7N8YD85|A0A7N8YD85\_9TELE

back to list

  

| Protein Coverage
| Supporting Peptides
|

Protein Coverage:

Supporting Peptides:

| Peptide | Uniq | -10lgP | Mass | ppm | m/z | z | RT | Scan | #Spec | Start | End | PTM |
| --- | --- | --- | --- | --- | --- | --- | --- | --- | --- | --- | --- | --- |
| F.MDDPVVIPGKPY.T | Y | 42.46 | 1329.6638 | -0.6 | 665.8388 | 2 | 12.27 | 1723 | 1 | 219 | 230 |  |
| D.VLEDPVEILDN.S | Y | 35.61 | 1254.6343 | -0.3 | 628.3242 | 2 | 13.52 | 1941 | 1 | 145 | 155 |  |
| D.VLEDPVEILDNSRE.L | Y | 22.59 | 1626.8101 | -0.5 | 814.4119 | 2 | 13.15 | 1877 | 1 | 145 | 158 |  |
| total 3 peptides |  |  |  |  |  |  |  |  |  |  |  |  |
| --- | --- | --- | --- | --- | --- | --- | --- | --- | --- | --- | --- | --- |

tr|A0A7N8Y4P6|A0A7N8Y4P6\_9TELE

back to list

  

| Protein Coverage
| Supporting Peptides
|

Protein Coverage:

Supporting Peptides:

| Peptide | Uniq | -10lgP | Mass | ppm | m/z | z | RT | Scan | #Spec | Start | End | PTM |
| --- | --- | --- | --- | --- | --- | --- | --- | --- | --- | --- | --- | --- |
| F.MDDPVVIPGKPY.T | Y | 42.46 | 1329.6638 | -0.6 | 665.8388 | 2 | 12.27 | 1723 | 1 | 222 | 233 |  |
| D.VLEDPVEILDN.S | Y | 35.61 | 1254.6343 | -0.3 | 628.3242 | 2 | 13.52 | 1941 | 1 | 142 | 152 |  |
| D.VLEDPVEILDNSRE.L | Y | 22.59 | 1626.8101 | -0.5 | 814.4119 | 2 | 13.15 | 1877 | 1 | 142 | 155 |  |
| total 3 peptides |  |  |  |  |  |  |  |  |  |  |  |  |
| --- | --- | --- | --- | --- | --- | --- | --- | --- | --- | --- | --- | --- |

tr|A0A7N8XJX7|A0A7N8XJX7\_9TELE

back to list

  

| Protein Coverage
| Supporting Peptides
|

Protein Coverage:

Supporting Peptides:

| Peptide | Uniq | -10lgP | Mass | ppm | m/z | z | RT | Scan | #Spec | Start | End | PTM |
| --- | --- | --- | --- | --- | --- | --- | --- | --- | --- | --- | --- | --- |
| F.MDDPVVIPGKPY.T | Y | 42.46 | 1329.6638 | -0.6 | 665.8388 | 2 | 12.27 | 1723 | 1 | 225 | 236 |  |
| D.VLEDPVEILDN.S | Y | 35.61 | 1254.6343 | -0.3 | 628.3242 | 2 | 13.52 | 1941 | 1 | 145 | 155 |  |
| D.VLEDPVEILDNSRE.L | Y | 22.59 | 1626.8101 | -0.5 | 814.4119 | 2 | 13.15 | 1877 | 1 | 145 | 158 |  |
| total 3 peptides |  |  |  |  |  |  |  |  |  |  |  |  |
| --- | --- | --- | --- | --- | --- | --- | --- | --- | --- | --- | --- | --- |

tr|A0A3Q3SHA7|A0A3Q3SHA7\_9TELE

back to list

  

| Protein Coverage
| Supporting Peptides
|

Protein Coverage:

Supporting Peptides:

| Peptide | Uniq | -10lgP | Mass | ppm | m/z | z | RT | Scan | #Spec | Start | End | PTM |
| --- | --- | --- | --- | --- | --- | --- | --- | --- | --- | --- | --- | --- |
| F.MDDPVVIPGKPY.T | Y | 42.46 | 1329.6638 | -0.6 | 665.8388 | 2 | 12.27 | 1723 | 1 | 237 | 248 |  |
| D.VLEDPVEILDN.S | Y | 35.61 | 1254.6343 | -0.3 | 628.3242 | 2 | 13.52 | 1941 | 1 | 157 | 167 |  |
| D.VLEDPVEILDNSRE.L | Y | 22.59 | 1626.8101 | -0.5 | 814.4119 | 2 | 13.15 | 1877 | 1 | 157 | 170 |  |
| total 3 peptides |  |  |  |  |  |  |  |  |  |  |  |  |
| --- | --- | --- | --- | --- | --- | --- | --- | --- | --- | --- | --- | --- |

tr|A0A7N8XSZ9|A0A7N8XSZ9\_9TELE

back to list

  

| Protein Coverage
| Supporting Peptides
|

Protein Coverage:

Supporting Peptides:

| Peptide | Uniq | -10lgP | Mass | ppm | m/z | z | RT | Scan | #Spec | Start | End | PTM |
| --- | --- | --- | --- | --- | --- | --- | --- | --- | --- | --- | --- | --- |
| F.MDDPVVIPGKPY.T | Y | 42.46 | 1329.6638 | -0.6 | 665.8388 | 2 | 12.27 | 1723 | 1 | 237 | 248 |  |
| D.VLEDPVEILDN.S | Y | 35.61 | 1254.6343 | -0.3 | 628.3242 | 2 | 13.52 | 1941 | 1 | 157 | 167 |  |
| D.VLEDPVEILDNSRE.L | Y | 22.59 | 1626.8101 | -0.5 | 814.4119 | 2 | 13.15 | 1877 | 1 | 157 | 170 |  |
| total 3 peptides |  |  |  |  |  |  |  |  |  |  |  |  |
| --- | --- | --- | --- | --- | --- | --- | --- | --- | --- | --- | --- | --- |

tr|A0A7N8XRW1|A0A7N8XRW1\_9TELE

back to list

  

| Protein Coverage
| Supporting Peptides
|

Protein Coverage:

Supporting Peptides:

| Peptide | Uniq | -10lgP | Mass | ppm | m/z | z | RT | Scan | #Spec | Start | End | PTM |
| --- | --- | --- | --- | --- | --- | --- | --- | --- | --- | --- | --- | --- |
| F.MDDPVVIPGKPY.T | Y | 42.46 | 1329.6638 | -0.6 | 665.8388 | 2 | 12.27 | 1723 | 1 | 238 | 249 |  |
| D.VLEDPVEILDN.S | Y | 35.61 | 1254.6343 | -0.3 | 628.3242 | 2 | 13.52 | 1941 | 1 | 158 | 168 |  |
| D.VLEDPVEILDNSRE.L | Y | 22.59 | 1626.8101 | -0.5 | 814.4119 | 2 | 13.15 | 1877 | 1 | 158 | 171 |  |
| total 3 peptides |  |  |  |  |  |  |  |  |  |  |  |  |
| --- | --- | --- | --- | --- | --- | --- | --- | --- | --- | --- | --- | --- |

tr|A0A7N8Y2R6|A0A7N8Y2R6\_9TELE

back to list

  

| Protein Coverage
| Supporting Peptides
|

Protein Coverage:

Supporting Peptides:

| Peptide | Uniq | -10lgP | Mass | ppm | m/z | z | RT | Scan | #Spec | Start | End | PTM |
| --- | --- | --- | --- | --- | --- | --- | --- | --- | --- | --- | --- | --- |
| F.MDDPVVIPGKPY.T | Y | 42.46 | 1329.6638 | -0.6 | 665.8388 | 2 | 12.27 | 1723 | 1 | 238 | 249 |  |
| D.VLEDPVEILDN.S | Y | 35.61 | 1254.6343 | -0.3 | 628.3242 | 2 | 13.52 | 1941 | 1 | 158 | 168 |  |
| D.VLEDPVEILDNSRE.L | Y | 22.59 | 1626.8101 | -0.5 | 814.4119 | 2 | 13.15 | 1877 | 1 | 158 | 171 |  |
| total 3 peptides |  |  |  |  |  |  |  |  |  |  |  |  |
| --- | --- | --- | --- | --- | --- | --- | --- | --- | --- | --- | --- | --- |

tr|A0A7N8Y336|A0A7N8Y336\_9TELE

back to list

  

| Protein Coverage
| Supporting Peptides
|

Protein Coverage:

Supporting Peptides:

| Peptide | Uniq | -10lgP | Mass | ppm | m/z | z | RT | Scan | #Spec | Start | End | PTM |
| --- | --- | --- | --- | --- | --- | --- | --- | --- | --- | --- | --- | --- |
| F.MDDPVVIPGKPY.T | Y | 42.46 | 1329.6638 | -0.6 | 665.8388 | 2 | 12.27 | 1723 | 1 | 241 | 252 |  |
| D.VLEDPVEILDN.S | Y | 35.61 | 1254.6343 | -0.3 | 628.3242 | 2 | 13.52 | 1941 | 1 | 161 | 171 |  |
| D.VLEDPVEILDNSRE.L | Y | 22.59 | 1626.8101 | -0.5 | 814.4119 | 2 | 13.15 | 1877 | 1 | 161 | 174 |  |
| total 3 peptides |  |  |  |  |  |  |  |  |  |  |  |  |
| --- | --- | --- | --- | --- | --- | --- | --- | --- | --- | --- | --- | --- |

tr|A0A1A8GBU0|A0A1A8GBU0\_9TELE

back to list

  

| Protein Coverage
| Supporting Peptides
|

Protein Coverage:

Supporting Peptides:

| Peptide | Uniq | -10lgP | Mass | ppm | m/z | z | RT | Scan | #Spec | Start | End | PTM |
| --- | --- | --- | --- | --- | --- | --- | --- | --- | --- | --- | --- | --- |
| A.GDSDGDGKIGVDEF.A | N | 35.47 | 1409.5946 | -1.1 | 705.8038 | 2 | 11.99 | 1675 | 1 | 90 | 103 |  |
| S.GFIEEEELK.L | N | 31.42 | 1092.5338 | -0.1 | 547.2741 | 2 | 11.80 | 1641 | 1 | 57 | 65 |  |
| F.AVIDQDKSGFIEEEELK.L | Y | 29.62 | 1948.9629 | -18.8 | 975.4704 | 2 | 12.63 | 1786 | 1 | 49 | 65 |  |
| Q.DKSGFIEEEELK.L | N | 25.85 | 1422.6877 | -0.4 | 712.3508 | 2 | 11.40 | 1570 | 1 | 54 | 65 |  |
| total 4 peptides |  |  |  |  |  |  |  |  |  |  |  |  |
| --- | --- | --- | --- | --- | --- | --- | --- | --- | --- | --- | --- | --- |

tr|A0A3B3ZS88|A0A3B3ZS88\_9GOBI

back to list

  

| Protein Coverage
| Supporting Peptides
|

Protein Coverage:

Supporting Peptides:

| Peptide | Uniq | -10lgP | Mass | ppm | m/z | z | RT | Scan | #Spec | Start | End | PTM |
| --- | --- | --- | --- | --- | --- | --- | --- | --- | --- | --- | --- | --- |
| H.LNEDKLKDKINE.L | Y | 56.63 | 1457.7725 | 5.3 | 729.8974 | 2 | 10.03 | 1354 | 2 | 157 | 168 |  |
| R.AERAEQQR.V | Y | 20.06 | 986.4893 | -0.1 | 494.2519 | 2 | 7.59 | 1018 | 1 | 66 | 73 |  |
| total 2 peptides |  |  |  |  |  |  |  |  |  |  |  |  |
| --- | --- | --- | --- | --- | --- | --- | --- | --- | --- | --- | --- | --- |

tr|A0A3Q1CJW7|A0A3Q1CJW7\_AMPOC

back to list

  

| Protein Coverage
| Supporting Peptides
|

Protein Coverage:

Supporting Peptides:

| Peptide | Uniq | -10lgP | Mass | ppm | m/z | z | RT | Scan | #Spec | Start | End | PTM |
| --- | --- | --- | --- | --- | --- | --- | --- | --- | --- | --- | --- | --- |
| H.LNEDKLKDKINE.L | Y | 56.63 | 1457.7725 | 5.3 | 729.8974 | 2 | 10.03 | 1354 | 2 | 157 | 168 |  |
| R.AERAEQQR.V | Y | 20.06 | 986.4893 | -0.1 | 494.2519 | 2 | 7.59 | 1018 | 1 | 66 | 73 |  |
| total 2 peptides |  |  |  |  |  |  |  |  |  |  |  |  |
| --- | --- | --- | --- | --- | --- | --- | --- | --- | --- | --- | --- | --- |

tr|A0A3Q3EFK1|A0A3Q3EFK1\_9LABR

back to list

  

| Protein Coverage
| Supporting Peptides
|

Protein Coverage:

Supporting Peptides:

| Peptide | Uniq | -10lgP | Mass | ppm | m/z | z | RT | Scan | #Spec | Start | End | PTM |
| --- | --- | --- | --- | --- | --- | --- | --- | --- | --- | --- | --- | --- |
| H.LNEDKLKDKINE.L | Y | 56.63 | 1457.7725 | 5.3 | 729.8974 | 2 | 10.03 | 1354 | 2 | 162 | 173 |  |
| R.AERAEQQR.I | Y | 20.06 | 986.4893 | -0.1 | 494.2519 | 2 | 7.59 | 1018 | 1 | 71 | 78 |  |
| total 2 peptides |  |  |  |  |  |  |  |  |  |  |  |  |
| --- | --- | --- | --- | --- | --- | --- | --- | --- | --- | --- | --- | --- |

tr|A0A3Q3AF19|A0A3Q3AF19\_KRYMA

back to list

  

| Protein Coverage
| Supporting Peptides
|

Protein Coverage:

Supporting Peptides:

| Peptide | Uniq | -10lgP | Mass | ppm | m/z | z | RT | Scan | #Spec | Start | End | PTM |
| --- | --- | --- | --- | --- | --- | --- | --- | --- | --- | --- | --- | --- |
| H.LNEDKLKDKINE.L | Y | 56.63 | 1457.7725 | 5.3 | 729.8974 | 2 | 10.03 | 1354 | 2 | 162 | 173 |  |
| R.AERAEQQR.V | Y | 20.06 | 986.4893 | -0.1 | 494.2519 | 2 | 7.59 | 1018 | 1 | 71 | 78 |  |
| total 2 peptides |  |  |  |  |  |  |  |  |  |  |  |  |
| --- | --- | --- | --- | --- | --- | --- | --- | --- | --- | --- | --- | --- |

tr|A0A3Q3AF32|A0A3Q3AF32\_KRYMA

back to list

  

| Protein Coverage
| Supporting Peptides
|

Protein Coverage:

Supporting Peptides:

| Peptide | Uniq | -10lgP | Mass | ppm | m/z | z | RT | Scan | #Spec | Start | End | PTM |
| --- | --- | --- | --- | --- | --- | --- | --- | --- | --- | --- | --- | --- |
| H.LNEDKLKDKINE.L | Y | 56.63 | 1457.7725 | 5.3 | 729.8974 | 2 | 10.03 | 1354 | 2 | 171 | 182 |  |
| R.AERAEQQR.V | Y | 20.06 | 986.4893 | -0.1 | 494.2519 | 2 | 7.59 | 1018 | 1 | 80 | 87 |  |
| total 2 peptides |  |  |  |  |  |  |  |  |  |  |  |  |
| --- | --- | --- | --- | --- | --- | --- | --- | --- | --- | --- | --- | --- |

tr|A0A3P8UNE3|A0A3P8UNE3\_CYNSE

back to list

  

| Protein Coverage
| Supporting Peptides
|

Protein Coverage:

Supporting Peptides:

| Peptide | Uniq | -10lgP | Mass | ppm | m/z | z | RT | Scan | #Spec | Start | End | PTM |
| --- | --- | --- | --- | --- | --- | --- | --- | --- | --- | --- | --- | --- |
| H.LNEDKLKDKINE.L | Y | 56.63 | 1457.7725 | 5.3 | 729.8974 | 2 | 10.03 | 1354 | 2 | 168 | 179 |  |
| R.AERAEQQR.V | Y | 20.06 | 986.4893 | -0.1 | 494.2519 | 2 | 7.59 | 1018 | 1 | 77 | 84 |  |
| total 2 peptides |  |  |  |  |  |  |  |  |  |  |  |  |
| --- | --- | --- | --- | --- | --- | --- | --- | --- | --- | --- | --- | --- |

tr|A0A672Y3T4|A0A672Y3T4\_9TELE

back to list

  

| Protein Coverage
| Supporting Peptides
|

Protein Coverage:

Supporting Peptides:

| Peptide | Uniq | -10lgP | Mass | ppm | m/z | z | RT | Scan | #Spec | Start | End | PTM |
| --- | --- | --- | --- | --- | --- | --- | --- | --- | --- | --- | --- | --- |
| H.LNEDKLKDKINE.L | Y | 56.63 | 1457.7725 | 5.3 | 729.8974 | 2 | 10.03 | 1354 | 2 | 168 | 179 |  |
| R.AERAEQQR.V | Y | 20.06 | 986.4893 | -0.1 | 494.2519 | 2 | 7.59 | 1018 | 1 | 77 | 84 |  |
| total 2 peptides |  |  |  |  |  |  |  |  |  |  |  |  |
| --- | --- | --- | --- | --- | --- | --- | --- | --- | --- | --- | --- | --- |

tr|A0A3P8UNF6|A0A3P8UNF6\_CYNSE

back to list

  

| Protein Coverage
| Supporting Peptides
|

Protein Coverage:

Supporting Peptides:

| Peptide | Uniq | -10lgP | Mass | ppm | m/z | z | RT | Scan | #Spec | Start | End | PTM |
| --- | --- | --- | --- | --- | --- | --- | --- | --- | --- | --- | --- | --- |
| H.LNEDKLKDKINE.L | Y | 56.63 | 1457.7725 | 5.3 | 729.8974 | 2 | 10.03 | 1354 | 2 | 190 | 201 |  |
| R.AERAEQQR.V | Y | 20.06 | 986.4893 | -0.1 | 494.2519 | 2 | 7.59 | 1018 | 1 | 99 | 106 |  |
| total 2 peptides |  |  |  |  |  |  |  |  |  |  |  |  |
| --- | --- | --- | --- | --- | --- | --- | --- | --- | --- | --- | --- | --- |

tr|A0A3B4UYC0|A0A3B4UYC0\_SERDU

back to list

  

| Protein Coverage
| Supporting Peptides
|

Protein Coverage:

Supporting Peptides:

| Peptide | Uniq | -10lgP | Mass | ppm | m/z | z | RT | Scan | #Spec | Start | End | PTM |
| --- | --- | --- | --- | --- | --- | --- | --- | --- | --- | --- | --- | --- |
| H.LNEDKLKDKINE.L | Y | 56.63 | 1457.7725 | 5.3 | 729.8974 | 2 | 10.03 | 1354 | 2 | 159 | 170 |  |
| R.AERAEQQR.V | Y | 20.06 | 986.4893 | -0.1 | 494.2519 | 2 | 7.59 | 1018 | 1 | 68 | 75 |  |
| total 2 peptides |  |  |  |  |  |  |  |  |  |  |  |  |
| --- | --- | --- | --- | --- | --- | --- | --- | --- | --- | --- | --- | --- |

tr|A0A3P8URR7|A0A3P8URR7\_CYNSE

back to list

  

| Protein Coverage
| Supporting Peptides
|

Protein Coverage:

Supporting Peptides:

| Peptide | Uniq | -10lgP | Mass | ppm | m/z | z | RT | Scan | #Spec | Start | End | PTM |
| --- | --- | --- | --- | --- | --- | --- | --- | --- | --- | --- | --- | --- |
| H.LNEDKLKDKINE.L | Y | 56.63 | 1457.7725 | 5.3 | 729.8974 | 2 | 10.03 | 1354 | 2 | 169 | 180 |  |
| R.AERAEQQR.V | Y | 20.06 | 986.4893 | -0.1 | 494.2519 | 2 | 7.59 | 1018 | 1 | 78 | 85 |  |
| total 2 peptides |  |  |  |  |  |  |  |  |  |  |  |  |
| --- | --- | --- | --- | --- | --- | --- | --- | --- | --- | --- | --- | --- |

tr|A0A3P8URM3|A0A3P8URM3\_CYNSE

back to list

  

| Protein Coverage
| Supporting Peptides
|

Protein Coverage:

Supporting Peptides:

| Peptide | Uniq | -10lgP | Mass | ppm | m/z | z | RT | Scan | #Spec | Start | End | PTM |
| --- | --- | --- | --- | --- | --- | --- | --- | --- | --- | --- | --- | --- |
| H.LNEDKLKDKINE.L | Y | 56.63 | 1457.7725 | 5.3 | 729.8974 | 2 | 10.03 | 1354 | 2 | 193 | 204 |  |
| R.AERAEQQR.V | Y | 20.06 | 986.4893 | -0.1 | 494.2519 | 2 | 7.59 | 1018 | 1 | 102 | 109 |  |
| total 2 peptides |  |  |  |  |  |  |  |  |  |  |  |  |
| --- | --- | --- | --- | --- | --- | --- | --- | --- | --- | --- | --- | --- |

tr|A0A3Q3XL97|A0A3Q3XL97\_MOLML

back to list

  

| Protein Coverage
| Supporting Peptides
|

Protein Coverage:

Supporting Peptides:

| Peptide | Uniq | -10lgP | Mass | ppm | m/z | z | RT | Scan | #Spec | Start | End | PTM |
| --- | --- | --- | --- | --- | --- | --- | --- | --- | --- | --- | --- | --- |
| H.LNEDKLKDKINE.L | Y | 56.63 | 1457.7725 | 5.3 | 729.8974 | 2 | 10.03 | 1354 | 2 | 204 | 215 |  |
| R.AERAEQQR.V | Y | 20.06 | 986.4893 | -0.1 | 494.2519 | 2 | 7.59 | 1018 | 1 | 113 | 120 |  |
| total 2 peptides |  |  |  |  |  |  |  |  |  |  |  |  |
| --- | --- | --- | --- | --- | --- | --- | --- | --- | --- | --- | --- | --- |

tr|A0A3P8ULW6|A0A3P8ULW6\_CYNSE

back to list

  

| Protein Coverage
| Supporting Peptides
|

Protein Coverage:

Supporting Peptides:

| Peptide | Uniq | -10lgP | Mass | ppm | m/z | z | RT | Scan | #Spec | Start | End | PTM |
| --- | --- | --- | --- | --- | --- | --- | --- | --- | --- | --- | --- | --- |
| H.LNEDKLKDKINE.L | Y | 56.63 | 1457.7725 | 5.3 | 729.8974 | 2 | 10.03 | 1354 | 2 | 206 | 217 |  |
| R.AERAEQQR.V | Y | 20.06 | 986.4893 | -0.1 | 494.2519 | 2 | 7.59 | 1018 | 1 | 115 | 122 |  |
| total 2 peptides |  |  |  |  |  |  |  |  |  |  |  |  |
| --- | --- | --- | --- | --- | --- | --- | --- | --- | --- | --- | --- | --- |

tr|A0A5C6P7G2|A0A5C6P7G2\_9TELE

back to list

  

| Protein Coverage
| Supporting Peptides
|

Protein Coverage:

Supporting Peptides:

| Peptide | Uniq | -10lgP | Mass | ppm | m/z | z | RT | Scan | #Spec | Start | End | PTM |
| --- | --- | --- | --- | --- | --- | --- | --- | --- | --- | --- | --- | --- |
| H.LNEDKLKDKINE.L | Y | 56.63 | 1457.7725 | 5.3 | 729.8974 | 2 | 10.03 | 1354 | 2 | 211 | 222 |  |
| R.AERAEQQR.I | Y | 20.06 | 986.4893 | -0.1 | 494.2519 | 2 | 7.59 | 1018 | 1 | 120 | 127 |  |
| total 2 peptides |  |  |  |  |  |  |  |  |  |  |  |  |
| --- | --- | --- | --- | --- | --- | --- | --- | --- | --- | --- | --- | --- |

tr|A0A3Q1CRB3|A0A3Q1CRB3\_AMPOC

back to list

  

| Protein Coverage
| Supporting Peptides
|

Protein Coverage:

Supporting Peptides:

| Peptide | Uniq | -10lgP | Mass | ppm | m/z | z | RT | Scan | #Spec | Start | End | PTM |
| --- | --- | --- | --- | --- | --- | --- | --- | --- | --- | --- | --- | --- |
| H.LNEDKLKDKINE.L | Y | 56.63 | 1457.7725 | 5.3 | 729.8974 | 2 | 10.03 | 1354 | 2 | 214 | 225 |  |
| R.AERAEQQR.V | Y | 20.06 | 986.4893 | -0.1 | 494.2519 | 2 | 7.59 | 1018 | 1 | 123 | 130 |  |
| total 2 peptides |  |  |  |  |  |  |  |  |  |  |  |  |
| --- | --- | --- | --- | --- | --- | --- | --- | --- | --- | --- | --- | --- |

tr|A0A665T7Y2|A0A665T7Y2\_ECHNA

back to list

  

| Protein Coverage
| Supporting Peptides
|

Protein Coverage:

Supporting Peptides:

| Peptide | Uniq | -10lgP | Mass | ppm | m/z | z | RT | Scan | #Spec | Start | End | PTM |
| --- | --- | --- | --- | --- | --- | --- | --- | --- | --- | --- | --- | --- |
| H.LNEDKLKDKINE.L | Y | 56.63 | 1457.7725 | 5.3 | 729.8974 | 2 | 10.03 | 1354 | 2 | 220 | 231 |  |
| R.AERAEQQR.I | Y | 20.06 | 986.4893 | -0.1 | 494.2519 | 2 | 7.59 | 1018 | 1 | 129 | 136 |  |
| total 2 peptides |  |  |  |  |  |  |  |  |  |  |  |  |
| --- | --- | --- | --- | --- | --- | --- | --- | --- | --- | --- | --- | --- |

tr|A0A3Q1BEW8|A0A3Q1BEW8\_AMPOC

back to list

  

| Protein Coverage
| Supporting Peptides
|

Protein Coverage:

Supporting Peptides:

| Peptide | Uniq | -10lgP | Mass | ppm | m/z | z | RT | Scan | #Spec | Start | End | PTM |
| --- | --- | --- | --- | --- | --- | --- | --- | --- | --- | --- | --- | --- |
| H.LNEDKLKDKINE.L | Y | 56.63 | 1457.7725 | 5.3 | 729.8974 | 2 | 10.03 | 1354 | 2 | 220 | 231 |  |
| R.AERAEQQR.V | Y | 20.06 | 986.4893 | -0.1 | 494.2519 | 2 | 7.59 | 1018 | 1 | 129 | 136 |  |
| total 2 peptides |  |  |  |  |  |  |  |  |  |  |  |  |
| --- | --- | --- | --- | --- | --- | --- | --- | --- | --- | --- | --- | --- |

tr|A0A3P8THB6|A0A3P8THB6\_AMPPE

back to list

  

| Protein Coverage
| Supporting Peptides
|

Protein Coverage:

Supporting Peptides:

| Peptide | Uniq | -10lgP | Mass | ppm | m/z | z | RT | Scan | #Spec | Start | End | PTM |
| --- | --- | --- | --- | --- | --- | --- | --- | --- | --- | --- | --- | --- |
| H.LNEDKLKDKINE.L | Y | 56.63 | 1457.7725 | 5.3 | 729.8974 | 2 | 10.03 | 1354 | 2 | 229 | 240 |  |
| R.AERAEQQR.V | Y | 20.06 | 986.4893 | -0.1 | 494.2519 | 2 | 7.59 | 1018 | 1 | 138 | 145 |  |
| total 2 peptides |  |  |  |  |  |  |  |  |  |  |  |  |
| --- | --- | --- | --- | --- | --- | --- | --- | --- | --- | --- | --- | --- |

tr|A0A672YCL7|A0A672YCL7\_9TELE

back to list

  

| Protein Coverage
| Supporting Peptides
|

Protein Coverage:

Supporting Peptides:

| Peptide | Uniq | -10lgP | Mass | ppm | m/z | z | RT | Scan | #Spec | Start | End | PTM |
| --- | --- | --- | --- | --- | --- | --- | --- | --- | --- | --- | --- | --- |
| H.LNEDKLKDKINE.L | Y | 56.63 | 1457.7725 | 5.3 | 729.8974 | 2 | 10.03 | 1354 | 2 | 223 | 234 |  |
| R.AERAEQQR.V | Y | 20.06 | 986.4893 | -0.1 | 494.2519 | 2 | 7.59 | 1018 | 1 | 132 | 139 |  |
| total 2 peptides |  |  |  |  |  |  |  |  |  |  |  |  |
| --- | --- | --- | --- | --- | --- | --- | --- | --- | --- | --- | --- | --- |

tr|A0A672YCL2|A0A672YCL2\_9TELE

back to list

  

| Protein Coverage
| Supporting Peptides
|

Protein Coverage:

Supporting Peptides:

| Peptide | Uniq | -10lgP | Mass | ppm | m/z | z | RT | Scan | #Spec | Start | End | PTM |
| --- | --- | --- | --- | --- | --- | --- | --- | --- | --- | --- | --- | --- |
| H.LNEDKLKDKINE.L | Y | 56.63 | 1457.7725 | 5.3 | 729.8974 | 2 | 10.03 | 1354 | 2 | 229 | 240 |  |
| R.AERAEQQR.V | Y | 20.06 | 986.4893 | -0.1 | 494.2519 | 2 | 7.59 | 1018 | 1 | 138 | 145 |  |
| total 2 peptides |  |  |  |  |  |  |  |  |  |  |  |  |
| --- | --- | --- | --- | --- | --- | --- | --- | --- | --- | --- | --- | --- |

tr|A0A6P7KCL6|A0A6P7KCL6\_9TELE

back to list

  

| Protein Coverage
| Supporting Peptides
|

Protein Coverage:

Supporting Peptides:

| Peptide | Uniq | -10lgP | Mass | ppm | m/z | z | RT | Scan | #Spec | Start | End | PTM |
| --- | --- | --- | --- | --- | --- | --- | --- | --- | --- | --- | --- | --- |
| H.LNEDKLKDKINE.L | Y | 56.63 | 1457.7725 | 5.3 | 729.8974 | 2 | 10.03 | 1354 | 2 | 233 | 244 |  |
| R.AERAEQQR.V | Y | 20.06 | 986.4893 | -0.1 | 494.2519 | 2 | 7.59 | 1018 | 1 | 142 | 149 |  |
| total 2 peptides |  |  |  |  |  |  |  |  |  |  |  |  |
| --- | --- | --- | --- | --- | --- | --- | --- | --- | --- | --- | --- | --- |

tr|A0A6P7KCQ1|A0A6P7KCQ1\_9TELE

back to list

  

| Protein Coverage
| Supporting Peptides
|

Protein Coverage:

Supporting Peptides:

| Peptide | Uniq | -10lgP | Mass | ppm | m/z | z | RT | Scan | #Spec | Start | End | PTM |
| --- | --- | --- | --- | --- | --- | --- | --- | --- | --- | --- | --- | --- |
| H.LNEDKLKDKINE.L | Y | 56.63 | 1457.7725 | 5.3 | 729.8974 | 2 | 10.03 | 1354 | 2 | 236 | 247 |  |
| R.AERAEQQR.V | Y | 20.06 | 986.4893 | -0.1 | 494.2519 | 2 | 7.59 | 1018 | 1 | 145 | 152 |  |
| total 2 peptides |  |  |  |  |  |  |  |  |  |  |  |  |
| --- | --- | --- | --- | --- | --- | --- | --- | --- | --- | --- | --- | --- |

tr|A0A6P7KDL5|A0A6P7KDL5\_9TELE

back to list

  

| Protein Coverage
| Supporting Peptides
|

Protein Coverage:

Supporting Peptides:

| Peptide | Uniq | -10lgP | Mass | ppm | m/z | z | RT | Scan | #Spec | Start | End | PTM |
| --- | --- | --- | --- | --- | --- | --- | --- | --- | --- | --- | --- | --- |
| K.LEKTIDDLEDELY.A | Y | 35.82 | 1594.7614 | -1.1 | 798.3871 | 2 | 13.31 | 1904 | 1 | 249 | 261 |  |
| K.AAEDRSKQLEDDLVAL.Q | Y | 28.57 | 1771.8951 | -1.0 | 886.9540 | 2 | 12.87 | 1828 | 1 | 31 | 46 |  |
| K.LEKTIDDLEDE.L | Y | 23.72 | 1318.6140 | -0.4 | 660.3140 | 2 | 11.50 | 1588 | 1 | 249 | 259 |  |
| K.LKGTEDELDKY.S | Y | 18.29 | 1309.6401 | -0.1 | 437.5539 | 3 | 10.75 | 1459 | 1 | 50 | 60 |  |
| K.LEKTIDDLEDELYAQ.K | Y | 15.24 | 1793.8571 | -1.4 | 897.9346 | 2 | 13.29 | 1900 | 1 | 249 | 263 |  |
| total 5 peptides |  |  |  |  |  |  |  |  |  |  |  |  |
| --- | --- | --- | --- | --- | --- | --- | --- | --- | --- | --- | --- | --- |

tr|A0A6P7MR93|A0A6P7MR93\_BETSP

back to list

  

| Protein Coverage
| Supporting Peptides
|

Protein Coverage:

Supporting Peptides:

| Peptide | Uniq | -10lgP | Mass | ppm | m/z | z | RT | Scan | #Spec | Start | End | PTM |
| --- | --- | --- | --- | --- | --- | --- | --- | --- | --- | --- | --- | --- |
| K.LEKTIDDLEDELY.A | Y | 35.82 | 1594.7614 | -1.1 | 798.3871 | 2 | 13.31 | 1904 | 1 | 249 | 261 |  |
| K.AAEDRSKQLEDDLVAL.Q | Y | 28.57 | 1771.8951 | -1.0 | 886.9540 | 2 | 12.87 | 1828 | 1 | 31 | 46 |  |
| K.LEKTIDDLEDE.L | Y | 23.72 | 1318.6140 | -0.4 | 660.3140 | 2 | 11.50 | 1588 | 1 | 249 | 259 |  |
| K.LKGTEDELDKY.S | Y | 18.29 | 1309.6401 | -0.1 | 437.5539 | 3 | 10.75 | 1459 | 1 | 50 | 60 |  |
| K.LEKTIDDLEDELYAQ.K | Y | 15.24 | 1793.8571 | -1.4 | 897.9346 | 2 | 13.29 | 1900 | 1 | 249 | 263 |  |
| total 5 peptides |  |  |  |  |  |  |  |  |  |  |  |  |
| --- | --- | --- | --- | --- | --- | --- | --- | --- | --- | --- | --- | --- |

tr|X2J4M2|X2J4M2\_PAROL

back to list

  

| Protein Coverage
| Supporting Peptides
|

Protein Coverage:

Supporting Peptides:

| Peptide | Uniq | -10lgP | Mass | ppm | m/z | z | RT | Scan | #Spec | Start | End | PTM |
| --- | --- | --- | --- | --- | --- | --- | --- | --- | --- | --- | --- | --- |
| K.LEKTIDDLEDELY.A | Y | 35.82 | 1594.7614 | -1.1 | 798.3871 | 2 | 13.31 | 1904 | 1 | 249 | 261 |  |
| K.AAEDRSKQLEDDLVAL.Q | Y | 28.57 | 1771.8951 | -1.0 | 886.9540 | 2 | 12.87 | 1828 | 1 | 31 | 46 |  |
| K.LEKTIDDLEDE.L | Y | 23.72 | 1318.6140 | -0.4 | 660.3140 | 2 | 11.50 | 1588 | 1 | 249 | 259 |  |
| K.LKGTEDELDKY.S | Y | 18.29 | 1309.6401 | -0.1 | 437.5539 | 3 | 10.75 | 1459 | 1 | 50 | 60 |  |
| K.LEKTIDDLEDELYAQ.K | Y | 15.24 | 1793.8571 | -1.4 | 897.9346 | 2 | 13.29 | 1900 | 1 | 249 | 263 |  |
| total 5 peptides |  |  |  |  |  |  |  |  |  |  |  |  |
| --- | --- | --- | --- | --- | --- | --- | --- | --- | --- | --- | --- | --- |

tr|A0A3P9D7I0|A0A3P9D7I0\_9CICH

back to list

  

| Protein Coverage
| Supporting Peptides
|

Protein Coverage:

Supporting Peptides:

| Peptide | Uniq | -10lgP | Mass | ppm | m/z | z | RT | Scan | #Spec | Start | End | PTM |
| --- | --- | --- | --- | --- | --- | --- | --- | --- | --- | --- | --- | --- |
| K.LEKTIDDLEDELY.A | Y | 35.82 | 1594.7614 | -1.1 | 798.3871 | 2 | 13.31 | 1904 | 1 | 249 | 261 |  |
| K.AAEDRSKQLEDDLVAL.Q | Y | 28.57 | 1771.8951 | -1.0 | 886.9540 | 2 | 12.87 | 1828 | 1 | 31 | 46 |  |
| K.LEKTIDDLEDE.L | Y | 23.72 | 1318.6140 | -0.4 | 660.3140 | 2 | 11.50 | 1588 | 1 | 249 | 259 |  |
| K.LKGTEDELDKY.S | Y | 18.29 | 1309.6401 | -0.1 | 437.5539 | 3 | 10.75 | 1459 | 1 | 50 | 60 |  |
| K.LEKTIDDLEDELYAQ.K | Y | 15.24 | 1793.8571 | -1.4 | 897.9346 | 2 | 13.29 | 1900 | 1 | 249 | 263 |  |
| total 5 peptides |  |  |  |  |  |  |  |  |  |  |  |  |
| --- | --- | --- | --- | --- | --- | --- | --- | --- | --- | --- | --- | --- |

tr|A0A665VR95|A0A665VR95\_ECHNA

back to list

  

| Protein Coverage
| Supporting Peptides
|

Protein Coverage:

Supporting Peptides:

| Peptide | Uniq | -10lgP | Mass | ppm | m/z | z | RT | Scan | #Spec | Start | End | PTM |
| --- | --- | --- | --- | --- | --- | --- | --- | --- | --- | --- | --- | --- |
| K.LEKTIDDLEDELY.A | Y | 35.82 | 1594.7614 | -1.1 | 798.3871 | 2 | 13.31 | 1904 | 1 | 249 | 261 |  |
| K.AAEDRSKQLEDDLVAL.Q | Y | 28.57 | 1771.8951 | -1.0 | 886.9540 | 2 | 12.87 | 1828 | 1 | 31 | 46 |  |
| K.LEKTIDDLEDE.L | Y | 23.72 | 1318.6140 | -0.4 | 660.3140 | 2 | 11.50 | 1588 | 1 | 249 | 259 |  |
| K.LKGTEDELDKY.S | Y | 18.29 | 1309.6401 | -0.1 | 437.5539 | 3 | 10.75 | 1459 | 1 | 50 | 60 |  |
| K.LEKTIDDLEDELYAQ.K | Y | 15.24 | 1793.8571 | -1.4 | 897.9346 | 2 | 13.29 | 1900 | 1 | 249 | 263 |  |
| total 5 peptides |  |  |  |  |  |  |  |  |  |  |  |  |
| --- | --- | --- | --- | --- | --- | --- | --- | --- | --- | --- | --- | --- |

tr|A0A3P9LRU7|A0A3P9LRU7\_ORYLA

back to list

  

| Protein Coverage
| Supporting Peptides
|

Protein Coverage:

Supporting Peptides:

| Peptide | Uniq | -10lgP | Mass | ppm | m/z | z | RT | Scan | #Spec | Start | End | PTM |
| --- | --- | --- | --- | --- | --- | --- | --- | --- | --- | --- | --- | --- |
| T.GIDNPGHPFIM.T | Y | 41.01 | 1196.5648 | -0.7 | 599.2892 | 2 | 12.79 | 1813 | 1 | 60 | 70 |  |
| N.LKGGDDLDPNY.V | N | 26.11 | 1205.5564 | -0.8 | 603.7850 | 2 | 11.03 | 1505 | 1 | 115 | 125 |  |
| T.GIDNPGHPF.I | Y | 19.24 | 952.4402 | -0.2 | 477.2273 | 2 | 11.27 | 1547 | 2 | 60 | 68 |  |
| Q.LVVDGVKL.M | N | 18.62 | 841.5273 | -1.0 | 421.7705 | 2 | 12.90 | 1832 | 1 | 352 | 359 |  |
| total 4 peptides |  |  |  |  |  |  |  |  |  |  |  |  |
| --- | --- | --- | --- | --- | --- | --- | --- | --- | --- | --- | --- | --- |

tr|A0A2I4BW49|A0A2I4BW49\_9TELE

back to list

  

| Protein Coverage
| Supporting Peptides
|

Protein Coverage:

Supporting Peptides:

| Peptide | Uniq | -10lgP | Mass | ppm | m/z | z | RT | Scan | #Spec | Start | End | PTM |
| --- | --- | --- | --- | --- | --- | --- | --- | --- | --- | --- | --- | --- |
| T.GIDNPGHPFIM.T | Y | 41.01 | 1196.5648 | -0.7 | 599.2892 | 2 | 12.79 | 1813 | 1 | 60 | 70 |  |
| N.LKGGDDLDPNY.V | N | 26.11 | 1205.5564 | -0.8 | 603.7850 | 2 | 11.03 | 1505 | 1 | 115 | 125 |  |
| T.GIDNPGHPF.I | Y | 19.24 | 952.4402 | -0.2 | 477.2273 | 2 | 11.27 | 1547 | 2 | 60 | 68 |  |
| Q.LVVDGVKL.M | N | 18.62 | 841.5273 | -1.0 | 421.7705 | 2 | 12.90 | 1832 | 1 | 352 | 359 |  |
| total 4 peptides |  |  |  |  |  |  |  |  |  |  |  |  |
| --- | --- | --- | --- | --- | --- | --- | --- | --- | --- | --- | --- | --- |

tr|A0A3B3I369|A0A3B3I369\_ORYLA

back to list

  

| Protein Coverage
| Supporting Peptides
|

Protein Coverage:

Supporting Peptides:

| Peptide | Uniq | -10lgP | Mass | ppm | m/z | z | RT | Scan | #Spec | Start | End | PTM |
| --- | --- | --- | --- | --- | --- | --- | --- | --- | --- | --- | --- | --- |
| T.GIDNPGHPFIM.T | Y | 41.01 | 1196.5648 | -0.7 | 599.2892 | 2 | 12.79 | 1813 | 1 | 60 | 70 |  |
| N.LKGGDDLDPNY.V | N | 26.11 | 1205.5564 | -0.8 | 603.7850 | 2 | 11.03 | 1505 | 1 | 115 | 125 |  |
| T.GIDNPGHPF.I | Y | 19.24 | 952.4402 | -0.2 | 477.2273 | 2 | 11.27 | 1547 | 2 | 60 | 68 |  |
| Q.LVVDGVKL.M | N | 18.62 | 841.5273 | -1.0 | 421.7705 | 2 | 12.90 | 1832 | 1 | 352 | 359 |  |
| total 4 peptides |  |  |  |  |  |  |  |  |  |  |  |  |
| --- | --- | --- | --- | --- | --- | --- | --- | --- | --- | --- | --- | --- |

tr|A0A3P9HLZ1|A0A3P9HLZ1\_ORYLA

back to list

  

| Protein Coverage
| Supporting Peptides
|

Protein Coverage:

Supporting Peptides:

| Peptide | Uniq | -10lgP | Mass | ppm | m/z | z | RT | Scan | #Spec | Start | End | PTM |
| --- | --- | --- | --- | --- | --- | --- | --- | --- | --- | --- | --- | --- |
| T.GIDNPGHPFIM.T | Y | 41.01 | 1196.5648 | -0.7 | 599.2892 | 2 | 12.79 | 1813 | 1 | 60 | 70 |  |
| N.LKGGDDLDPNY.V | N | 26.11 | 1205.5564 | -0.8 | 603.7850 | 2 | 11.03 | 1505 | 1 | 115 | 125 |  |
| T.GIDNPGHPF.I | Y | 19.24 | 952.4402 | -0.2 | 477.2273 | 2 | 11.27 | 1547 | 2 | 60 | 68 |  |
| Q.LVVDGVKL.M | N | 18.62 | 841.5273 | -1.0 | 421.7705 | 2 | 12.90 | 1832 | 1 | 352 | 359 |  |
| total 4 peptides |  |  |  |  |  |  |  |  |  |  |  |  |
| --- | --- | --- | --- | --- | --- | --- | --- | --- | --- | --- | --- | --- |

tr|A0A3P8WTN2|A0A3P8WTN2\_CYNSE

back to list

  

| Protein Coverage
| Supporting Peptides
|

Protein Coverage:

Supporting Peptides:

| Peptide | Uniq | -10lgP | Mass | ppm | m/z | z | RT | Scan | #Spec | Start | End | PTM |
| --- | --- | --- | --- | --- | --- | --- | --- | --- | --- | --- | --- | --- |
| T.GIDNPGHPFIM.T | Y | 41.01 | 1196.5648 | -0.7 | 599.2892 | 2 | 12.79 | 1813 | 1 | 60 | 70 |  |
| N.LKGGDDLDPNY.V | N | 26.11 | 1205.5564 | -0.8 | 603.7850 | 2 | 11.03 | 1505 | 1 | 115 | 125 |  |
| T.GIDNPGHPF.I | Y | 19.24 | 952.4402 | -0.2 | 477.2273 | 2 | 11.27 | 1547 | 2 | 60 | 68 |  |
| Q.LVVDGVKL.M | N | 18.62 | 841.5273 | -1.0 | 421.7705 | 2 | 12.90 | 1832 | 1 | 352 | 359 |  |
| total 4 peptides |  |  |  |  |  |  |  |  |  |  |  |  |
| --- | --- | --- | --- | --- | --- | --- | --- | --- | --- | --- | --- | --- |

tr|A0A3P8Q9R4|A0A3P8Q9R4\_ASTCA

back to list

  

| Protein Coverage
| Supporting Peptides
|

Protein Coverage:

Supporting Peptides:

| Peptide | Uniq | -10lgP | Mass | ppm | m/z | z | RT | Scan | #Spec | Start | End | PTM |
| --- | --- | --- | --- | --- | --- | --- | --- | --- | --- | --- | --- | --- |
| T.GVDNPGHPF.I | Y | 34.94 | 938.4246 | -0.6 | 470.2193 | 2 | 10.88 | 1479 | 2 | 59 | 67 |  |
| T.GVDNPGHPFIM.T | Y | 27.25 | 1182.5492 | -0.4 | 592.2816 | 2 | 12.47 | 1757 | 1 | 59 | 69 |  |
| N.LKGGDDLDPNY.V | N | 26.11 | 1205.5564 | -0.8 | 603.7850 | 2 | 11.03 | 1505 | 1 | 114 | 124 |  |
| Q.LVVDGVKL.M | N | 18.62 | 841.5273 | -1.0 | 421.7705 | 2 | 12.90 | 1832 | 1 | 351 | 358 |  |
| total 4 peptides |  |  |  |  |  |  |  |  |  |  |  |  |
| --- | --- | --- | --- | --- | --- | --- | --- | --- | --- | --- | --- | --- |

tr|A0A6P7JDS0|A0A6P7JDS0\_9TELE

back to list

  

| Protein Coverage
| Supporting Peptides
|

Protein Coverage:

Supporting Peptides:

| Peptide | Uniq | -10lgP | Mass | ppm | m/z | z | RT | Scan | #Spec | Start | End | PTM |
| --- | --- | --- | --- | --- | --- | --- | --- | --- | --- | --- | --- | --- |
| T.GVDNPGHPF.I | Y | 34.94 | 938.4246 | -0.6 | 470.2193 | 2 | 10.88 | 1479 | 2 | 141 | 149 |  |
| T.GVDNPGHPFIM.T | Y | 27.25 | 1182.5492 | -0.4 | 592.2816 | 2 | 12.47 | 1757 | 1 | 141 | 151 |  |
| N.LKGGDDLDPNY.V | N | 26.11 | 1205.5564 | -0.8 | 603.7850 | 2 | 11.03 | 1505 | 1 | 196 | 206 |  |
| Q.LVVDGVKL.M | N | 18.62 | 841.5273 | -1.0 | 421.7705 | 2 | 12.90 | 1832 | 1 | 433 | 440 |  |
| total 4 peptides |  |  |  |  |  |  |  |  |  |  |  |  |
| --- | --- | --- | --- | --- | --- | --- | --- | --- | --- | --- | --- | --- |

tr|A0A3Q2Q7Q3|A0A3Q2Q7Q3\_FUNHE

back to list

  

| Protein Coverage
| Supporting Peptides
|

Protein Coverage:

Supporting Peptides:

| Peptide | Uniq | -10lgP | Mass | ppm | m/z | z | RT | Scan | #Spec | Start | End | PTM |
| --- | --- | --- | --- | --- | --- | --- | --- | --- | --- | --- | --- | --- |
| T.GVDNPGHPF.I | Y | 34.94 | 938.4246 | -0.6 | 470.2193 | 2 | 10.88 | 1479 | 2 | 61 | 69 |  |
| T.GVDNPGHPFIM.T | Y | 27.25 | 1182.5492 | -0.4 | 592.2816 | 2 | 12.47 | 1757 | 1 | 61 | 71 |  |
| N.LKGGDDLDPNY.V | N | 26.11 | 1205.5564 | -0.8 | 603.7850 | 2 | 11.03 | 1505 | 1 | 116 | 126 |  |
| Q.LVVDGVKL.M | N | 18.62 | 841.5273 | -1.0 | 421.7705 | 2 | 12.90 | 1832 | 1 | 315 | 322 |  |
| total 4 peptides |  |  |  |  |  |  |  |  |  |  |  |  |
| --- | --- | --- | --- | --- | --- | --- | --- | --- | --- | --- | --- | --- |

tr|A0A3B4BZV1|A0A3B4BZV1\_PYGNA

back to list

  

| Protein Coverage
| Supporting Peptides
|

Protein Coverage:

Supporting Peptides:

| Peptide | Uniq | -10lgP | Mass | ppm | m/z | z | RT | Scan | #Spec | Start | End | PTM |
| --- | --- | --- | --- | --- | --- | --- | --- | --- | --- | --- | --- | --- |
| T.GVDNPGHPF.I | Y | 34.94 | 938.4246 | -0.6 | 470.2193 | 2 | 10.88 | 1479 | 2 | 59 | 67 |  |
| T.GVDNPGHPFIM.T | Y | 27.25 | 1182.5492 | -0.4 | 592.2816 | 2 | 12.47 | 1757 | 1 | 59 | 69 |  |
| N.LKGGDDLDPNY.V | N | 26.11 | 1205.5564 | -0.8 | 603.7850 | 2 | 11.03 | 1505 | 1 | 114 | 124 |  |
| Q.LVVDGVKL.M | N | 18.62 | 841.5273 | -1.0 | 421.7705 | 2 | 12.90 | 1832 | 1 | 351 | 358 |  |
| total 4 peptides |  |  |  |  |  |  |  |  |  |  |  |  |
| --- | --- | --- | --- | --- | --- | --- | --- | --- | --- | --- | --- | --- |

tr|A0A6P7PB38|A0A6P7PB38\_BETSP

back to list

  

| Protein Coverage
| Supporting Peptides
|

Protein Coverage:

Supporting Peptides:

| Peptide | Uniq | -10lgP | Mass | ppm | m/z | z | RT | Scan | #Spec | Start | End | PTM |
| --- | --- | --- | --- | --- | --- | --- | --- | --- | --- | --- | --- | --- |
| T.GVDNPGHPF.I | Y | 34.94 | 938.4246 | -0.6 | 470.2193 | 2 | 10.88 | 1479 | 2 | 59 | 67 |  |
| T.GVDNPGHPFIM.T | Y | 27.25 | 1182.5492 | -0.4 | 592.2816 | 2 | 12.47 | 1757 | 1 | 59 | 69 |  |
| N.LKGGDDLDPNY.V | N | 26.11 | 1205.5564 | -0.8 | 603.7850 | 2 | 11.03 | 1505 | 1 | 114 | 124 |  |
| Q.LVVDGVKL.M | N | 18.62 | 841.5273 | -1.0 | 421.7705 | 2 | 12.90 | 1832 | 1 | 351 | 358 |  |
| total 4 peptides |  |  |  |  |  |  |  |  |  |  |  |  |
| --- | --- | --- | --- | --- | --- | --- | --- | --- | --- | --- | --- | --- |

tr|A0A3P8NKE2|A0A3P8NKE2\_ASTCA

back to list

  

| Protein Coverage
| Supporting Peptides
|

Protein Coverage:

Supporting Peptides:

| Peptide | Uniq | -10lgP | Mass | ppm | m/z | z | RT | Scan | #Spec | Start | End | PTM |
| --- | --- | --- | --- | --- | --- | --- | --- | --- | --- | --- | --- | --- |
| T.GVDNPGHPF.I | Y | 34.94 | 938.4246 | -0.6 | 470.2193 | 2 | 10.88 | 1479 | 2 | 60 | 68 |  |
| T.GVDNPGHPFIM.T | Y | 27.25 | 1182.5492 | -0.4 | 592.2816 | 2 | 12.47 | 1757 | 1 | 60 | 70 |  |
| N.LKGGDDLDPNY.V | N | 26.11 | 1205.5564 | -0.8 | 603.7850 | 2 | 11.03 | 1505 | 1 | 115 | 125 |  |
| Q.LVVDGVKL.M | N | 18.62 | 841.5273 | -1.0 | 421.7705 | 2 | 12.90 | 1832 | 1 | 352 | 359 |  |
| total 4 peptides |  |  |  |  |  |  |  |  |  |  |  |  |
| --- | --- | --- | --- | --- | --- | --- | --- | --- | --- | --- | --- | --- |

tr|A0A6P7JIX2|A0A6P7JIX2\_9TELE

back to list

  

| Protein Coverage
| Supporting Peptides
|

Protein Coverage:

Supporting Peptides:

| Peptide | Uniq | -10lgP | Mass | ppm | m/z | z | RT | Scan | #Spec | Start | End | PTM |
| --- | --- | --- | --- | --- | --- | --- | --- | --- | --- | --- | --- | --- |
| T.GVDNPGHPF.I | Y | 34.94 | 938.4246 | -0.6 | 470.2193 | 2 | 10.88 | 1479 | 2 | 60 | 68 |  |
| T.GVDNPGHPFIM.T | Y | 27.25 | 1182.5492 | -0.4 | 592.2816 | 2 | 12.47 | 1757 | 1 | 60 | 70 |  |
| N.LKGGDDLDPNY.V | N | 26.11 | 1205.5564 | -0.8 | 603.7850 | 2 | 11.03 | 1505 | 1 | 115 | 125 |  |
| Q.LVVDGVKL.M | N | 18.62 | 841.5273 | -1.0 | 421.7705 | 2 | 12.90 | 1832 | 1 | 352 | 359 |  |
| total 4 peptides |  |  |  |  |  |  |  |  |  |  |  |  |
| --- | --- | --- | --- | --- | --- | --- | --- | --- | --- | --- | --- | --- |

tr|A0A6P7PLI8|A0A6P7PLI8\_BETSP

back to list

  

| Protein Coverage
| Supporting Peptides
|

Protein Coverage:

Supporting Peptides:

| Peptide | Uniq | -10lgP | Mass | ppm | m/z | z | RT | Scan | #Spec | Start | End | PTM |
| --- | --- | --- | --- | --- | --- | --- | --- | --- | --- | --- | --- | --- |
| T.GVDNPGHPF.I | Y | 34.94 | 938.4246 | -0.6 | 470.2193 | 2 | 10.88 | 1479 | 2 | 60 | 68 |  |
| T.GVDNPGHPFIM.T | Y | 27.25 | 1182.5492 | -0.4 | 592.2816 | 2 | 12.47 | 1757 | 1 | 60 | 70 |  |
| N.LKGGDDLDPNY.V | N | 26.11 | 1205.5564 | -0.8 | 603.7850 | 2 | 11.03 | 1505 | 1 | 115 | 125 |  |
| Q.LVVDGVKL.M | N | 18.62 | 841.5273 | -1.0 | 421.7705 | 2 | 12.90 | 1832 | 1 | 352 | 359 |  |
| total 4 peptides |  |  |  |  |  |  |  |  |  |  |  |  |
| --- | --- | --- | --- | --- | --- | --- | --- | --- | --- | --- | --- | --- |

tr|A0A674PS54|A0A674PS54\_TAKRU

back to list

  

| Protein Coverage
| Supporting Peptides
|

Protein Coverage:

Supporting Peptides:

| Peptide | Uniq | -10lgP | Mass | ppm | m/z | z | RT | Scan | #Spec | Start | End | PTM |
| --- | --- | --- | --- | --- | --- | --- | --- | --- | --- | --- | --- | --- |
| T.GVDNPGHPF.I | Y | 34.94 | 938.4246 | -0.6 | 470.2193 | 2 | 10.88 | 1479 | 2 | 54 | 62 |  |
| T.GVDNPGHPFIM.T | Y | 27.25 | 1182.5492 | -0.4 | 592.2816 | 2 | 12.47 | 1757 | 1 | 54 | 64 |  |
| N.LKGGDDLDPNY.V | N | 26.11 | 1205.5564 | -0.8 | 603.7850 | 2 | 11.03 | 1505 | 1 | 109 | 119 |  |
| Q.LVVDGVKL.M | N | 18.62 | 841.5273 | -1.0 | 421.7705 | 2 | 12.90 | 1832 | 1 | 334 | 341 |  |
| total 4 peptides |  |  |  |  |  |  |  |  |  |  |  |  |
| --- | --- | --- | --- | --- | --- | --- | --- | --- | --- | --- | --- | --- |

tr|A0A437CN94|A0A437CN94\_ORYJA

back to list

  

| Protein Coverage
| Supporting Peptides
|

Protein Coverage:

Supporting Peptides:

| Peptide | Uniq | -10lgP | Mass | ppm | m/z | z | RT | Scan | #Spec | Start | End | PTM |
| --- | --- | --- | --- | --- | --- | --- | --- | --- | --- | --- | --- | --- |
| T.GVDNPGHPF.I | Y | 34.94 | 938.4246 | -0.6 | 470.2193 | 2 | 10.88 | 1479 | 2 | 60 | 68 |  |
| T.GVDNPGHPFIM.T | Y | 27.25 | 1182.5492 | -0.4 | 592.2816 | 2 | 12.47 | 1757 | 1 | 60 | 70 |  |
| N.LKGGDDLDPNY.V | N | 26.11 | 1205.5564 | -0.8 | 603.7850 | 2 | 11.03 | 1505 | 1 | 115 | 125 |  |
| Q.LVVDGVKL.M | N | 18.62 | 841.5273 | -1.0 | 421.7705 | 2 | 12.90 | 1832 | 1 | 352 | 359 |  |
| total 4 peptides |  |  |  |  |  |  |  |  |  |  |  |  |
| --- | --- | --- | --- | --- | --- | --- | --- | --- | --- | --- | --- | --- |

tr|A0A6J2R235|A0A6J2R235\_COTGO

back to list

  

| Protein Coverage
| Supporting Peptides
|

Protein Coverage:

Supporting Peptides:

| Peptide | Uniq | -10lgP | Mass | ppm | m/z | z | RT | Scan | #Spec | Start | End | PTM |
| --- | --- | --- | --- | --- | --- | --- | --- | --- | --- | --- | --- | --- |
| T.GVDNPGHPF.I | Y | 34.94 | 938.4246 | -0.6 | 470.2193 | 2 | 10.88 | 1479 | 2 | 60 | 68 |  |
| T.GVDNPGHPFIM.T | Y | 27.25 | 1182.5492 | -0.4 | 592.2816 | 2 | 12.47 | 1757 | 1 | 60 | 70 |  |
| N.LKGGDDLDPNY.V | N | 26.11 | 1205.5564 | -0.8 | 603.7850 | 2 | 11.03 | 1505 | 1 | 115 | 125 |  |
| Q.LVVDGVKL.M | N | 18.62 | 841.5273 | -1.0 | 421.7705 | 2 | 12.90 | 1832 | 1 | 352 | 359 |  |
| total 4 peptides |  |  |  |  |  |  |  |  |  |  |  |  |
| --- | --- | --- | --- | --- | --- | --- | --- | --- | --- | --- | --- | --- |

tr|A0A4U5VU95|A0A4U5VU95\_COLLU

back to list

  

| Protein Coverage
| Supporting Peptides
|

Protein Coverage:

Supporting Peptides:

| Peptide | Uniq | -10lgP | Mass | ppm | m/z | z | RT | Scan | #Spec | Start | End | PTM |
| --- | --- | --- | --- | --- | --- | --- | --- | --- | --- | --- | --- | --- |
| A.AFPPDVAGN.V | Y | 29.50 | 886.4185 | -0.5 | 444.2163 | 2 | 11.38 | 1567 | 3 | 122 | 130 |  |
| T.IIDQNRDGIISKD.D | Y | 24.69 | 1485.7787 | 0.3 | 743.8969 | 2 | 10.47 | 1413 | 1 | 16 | 28 |  |
| S.KDDLRDVLAS.M | Y | 24.23 | 1130.5931 | -0.5 | 566.3036 | 2 | 11.55 | 1597 | 1 | 27 | 36 |  |
| K.LKGADPEDVILS.A | Y | 19.92 | 1255.6659 | -0.1 | 628.8401 | 2 | 12.66 | 1790 | 2 | 71 | 82 |  |
| K.GADPEDVILS.A | Y | 15.25 | 1014.4869 | -0.1 | 508.2507 | 2 | 12.72 | 1801 | 1 | 73 | 82 |  |
| F.GEKLKGADPEDVILS.A | Y | 15.24 | 1569.8250 | -0.6 | 785.9193 | 2 | 11.75 | 1631 | 1 | 68 | 82 |  |
| total 6 peptides |  |  |  |  |  |  |  |  |  |  |  |  |
| --- | --- | --- | --- | --- | --- | --- | --- | --- | --- | --- | --- | --- |

tr|A0A6P7II88|A0A6P7II88\_9TELE

back to list

  

| Protein Coverage
| Supporting Peptides
|

Protein Coverage:

Supporting Peptides:

| Peptide | Uniq | -10lgP | Mass | ppm | m/z | z | RT | Scan | #Spec | Start | End | PTM |
| --- | --- | --- | --- | --- | --- | --- | --- | --- | --- | --- | --- | --- |
| A.AFPPDVAGN.V | Y | 29.50 | 886.4185 | -0.5 | 444.2163 | 2 | 11.38 | 1567 | 3 | 144 | 152 |  |
| T.IIDQNRDGIISKD.D | Y | 24.69 | 1485.7787 | 0.3 | 743.8969 | 2 | 10.47 | 1413 | 1 | 38 | 50 |  |
| S.KDDLRDVLAS.M | Y | 24.23 | 1130.5931 | -0.5 | 566.3036 | 2 | 11.55 | 1597 | 1 | 49 | 58 |  |
| K.LKGADPEDVILS.A | Y | 19.92 | 1255.6659 | -0.1 | 628.8401 | 2 | 12.66 | 1790 | 2 | 93 | 104 |  |
| K.GADPEDVILS.A | Y | 15.25 | 1014.4869 | -0.1 | 508.2507 | 2 | 12.72 | 1801 | 1 | 95 | 104 |  |
| F.GEKLKGADPEDVILS.A | Y | 15.24 | 1569.8250 | -0.6 | 785.9193 | 2 | 11.75 | 1631 | 1 | 90 | 104 |  |
| total 6 peptides |  |  |  |  |  |  |  |  |  |  |  |  |
| --- | --- | --- | --- | --- | --- | --- | --- | --- | --- | --- | --- | --- |

tr|A0A672MA11|A0A672MA11\_SINGR

back to list

  

| Protein Coverage
| Supporting Peptides
|

Protein Coverage:

Supporting Peptides:

| Peptide | Uniq | -10lgP | Mass | ppm | m/z | z | RT | Scan | #Spec | Start | End | PTM |
| --- | --- | --- | --- | --- | --- | --- | --- | --- | --- | --- | --- | --- |
| A.AFPPDVAGN.V | Y | 29.50 | 886.4185 | -0.5 | 444.2163 | 2 | 11.38 | 1567 | 3 | 116 | 124 |  |
| T.IIDQNRDGIISKD.D | Y | 24.69 | 1485.7787 | 0.3 | 743.8969 | 2 | 10.47 | 1413 | 1 | 10 | 22 |  |
| S.KDDLRDVLAS.M | Y | 24.23 | 1130.5931 | -0.5 | 566.3036 | 2 | 11.55 | 1597 | 1 | 21 | 30 |  |
| K.LKGADPEDVILS.A | Y | 19.92 | 1255.6659 | -0.1 | 628.8401 | 2 | 12.66 | 1790 | 2 | 65 | 76 |  |
| K.GADPEDVILS.A | Y | 15.25 | 1014.4869 | -0.1 | 508.2507 | 2 | 12.72 | 1801 | 1 | 67 | 76 |  |
| F.GEKLKGADPEDVILS.A | Y | 15.24 | 1569.8250 | -0.6 | 785.9193 | 2 | 11.75 | 1631 | 1 | 62 | 76 |  |
| total 6 peptides |  |  |  |  |  |  |  |  |  |  |  |  |
| --- | --- | --- | --- | --- | --- | --- | --- | --- | --- | --- | --- | --- |

tr|A0A669DZW9|A0A669DZW9\_ORENI

back to list

  

| Protein Coverage
| Supporting Peptides
|

Protein Coverage:

Supporting Peptides:

| Peptide | Uniq | -10lgP | Mass | ppm | m/z | z | RT | Scan | #Spec | Start | End | PTM |
| --- | --- | --- | --- | --- | --- | --- | --- | --- | --- | --- | --- | --- |
| A.AFPPDVAGN.V | Y | 29.50 | 886.4185 | -0.5 | 444.2163 | 2 | 11.38 | 1567 | 3 | 135 | 143 |  |
| T.IIDQNRDGIISKD.D | Y | 24.69 | 1485.7787 | 0.3 | 743.8969 | 2 | 10.47 | 1413 | 1 | 29 | 41 |  |
| S.KDDLRDVLAS.M | Y | 24.23 | 1130.5931 | -0.5 | 566.3036 | 2 | 11.55 | 1597 | 1 | 40 | 49 |  |
| K.LKGADPEDVILS.A | Y | 19.92 | 1255.6659 | -0.1 | 628.8401 | 2 | 12.66 | 1790 | 2 | 84 | 95 |  |
| K.GADPEDVILS.A | Y | 15.25 | 1014.4869 | -0.1 | 508.2507 | 2 | 12.72 | 1801 | 1 | 86 | 95 |  |
| F.GEKLKGADPEDVILS.A | Y | 15.24 | 1569.8250 | -0.6 | 785.9193 | 2 | 11.75 | 1631 | 1 | 81 | 95 |  |
| total 6 peptides |  |  |  |  |  |  |  |  |  |  |  |  |
| --- | --- | --- | --- | --- | --- | --- | --- | --- | --- | --- | --- | --- |

tr|A0A3B4BW75|A0A3B4BW75\_PYGNA

back to list

  

| Protein Coverage
| Supporting Peptides
|

Protein Coverage:

Supporting Peptides:

| Peptide | Uniq | -10lgP | Mass | ppm | m/z | z | RT | Scan | #Spec | Start | End | PTM |
| --- | --- | --- | --- | --- | --- | --- | --- | --- | --- | --- | --- | --- |
| A.AFPPDVAGN.V | Y | 29.50 | 886.4185 | -0.5 | 444.2163 | 2 | 11.38 | 1567 | 3 | 136 | 144 |  |
| T.IIDQNRDGIISKD.D | Y | 24.69 | 1485.7787 | 0.3 | 743.8969 | 2 | 10.47 | 1413 | 1 | 30 | 42 |  |
| S.KDDLRDVLAS.M | Y | 24.23 | 1130.5931 | -0.5 | 566.3036 | 2 | 11.55 | 1597 | 1 | 41 | 50 |  |
| K.LKGADPEDVILS.G | Y | 19.92 | 1255.6659 | -0.1 | 628.8401 | 2 | 12.66 | 1790 | 2 | 85 | 96 |  |
| K.GADPEDVILS.G | Y | 15.25 | 1014.4869 | -0.1 | 508.2507 | 2 | 12.72 | 1801 | 1 | 87 | 96 |  |
| F.GEKLKGADPEDVILS.G | Y | 15.24 | 1569.8250 | -0.6 | 785.9193 | 2 | 11.75 | 1631 | 1 | 82 | 96 |  |
| total 6 peptides |  |  |  |  |  |  |  |  |  |  |  |  |
| --- | --- | --- | --- | --- | --- | --- | --- | --- | --- | --- | --- | --- |

tr|I3JU24|I3JU24\_ORENI

back to list

  

| Protein Coverage
| Supporting Peptides
|

Protein Coverage:

Supporting Peptides:

| Peptide | Uniq | -10lgP | Mass | ppm | m/z | z | RT | Scan | #Spec | Start | End | PTM |
| --- | --- | --- | --- | --- | --- | --- | --- | --- | --- | --- | --- | --- |
| A.AFPPDVAGN.V | Y | 29.50 | 886.4185 | -0.5 | 444.2163 | 2 | 11.38 | 1567 | 3 | 137 | 145 |  |
| T.IIDQNRDGIISKD.D | Y | 24.69 | 1485.7787 | 0.3 | 743.8969 | 2 | 10.47 | 1413 | 1 | 31 | 43 |  |
| S.KDDLRDVLAS.M | Y | 24.23 | 1130.5931 | -0.5 | 566.3036 | 2 | 11.55 | 1597 | 1 | 42 | 51 |  |
| K.LKGADPEDVILS.A | Y | 19.92 | 1255.6659 | -0.1 | 628.8401 | 2 | 12.66 | 1790 | 2 | 86 | 97 |  |
| K.GADPEDVILS.A | Y | 15.25 | 1014.4869 | -0.1 | 508.2507 | 2 | 12.72 | 1801 | 1 | 88 | 97 |  |
| F.GEKLKGADPEDVILS.A | Y | 15.24 | 1569.8250 | -0.6 | 785.9193 | 2 | 11.75 | 1631 | 1 | 83 | 97 |  |
| total 6 peptides |  |  |  |  |  |  |  |  |  |  |  |  |
| --- | --- | --- | --- | --- | --- | --- | --- | --- | --- | --- | --- | --- |

tr|A0A7J6CHK6|A0A7J6CHK6\_9TELE

back to list

  

| Protein Coverage
| Supporting Peptides
|

Protein Coverage:

Supporting Peptides:

| Peptide | Uniq | -10lgP | Mass | ppm | m/z | z | RT | Scan | #Spec | Start | End | PTM |
| --- | --- | --- | --- | --- | --- | --- | --- | --- | --- | --- | --- | --- |
| A.AFPPDVAGN.V | Y | 29.50 | 886.4185 | -0.5 | 444.2163 | 2 | 11.38 | 1567 | 3 | 143 | 151 |  |
| T.IIDQNRDGIISKD.D | Y | 24.69 | 1485.7787 | 0.3 | 743.8969 | 2 | 10.47 | 1413 | 1 | 37 | 49 |  |
| S.KDDLRDVLAS.M | Y | 24.23 | 1130.5931 | -0.5 | 566.3036 | 2 | 11.55 | 1597 | 1 | 48 | 57 |  |
| K.LKGADPEDVILS.A | Y | 19.92 | 1255.6659 | -0.1 | 628.8401 | 2 | 12.66 | 1790 | 2 | 92 | 103 |  |
| K.GADPEDVILS.A | Y | 15.25 | 1014.4869 | -0.1 | 508.2507 | 2 | 12.72 | 1801 | 1 | 94 | 103 |  |
| F.GEKLKGADPEDVILS.A | Y | 15.24 | 1569.8250 | -0.6 | 785.9193 | 2 | 11.75 | 1631 | 1 | 89 | 103 |  |
| total 6 peptides |  |  |  |  |  |  |  |  |  |  |  |  |
| --- | --- | --- | --- | --- | --- | --- | --- | --- | --- | --- | --- | --- |

tr|A0A0F8CBC9|A0A0F8CBC9\_LARCR

back to list

  

| Protein Coverage
| Supporting Peptides
|

Protein Coverage:

Supporting Peptides:

| Peptide | Uniq | -10lgP | Mass | ppm | m/z | z | RT | Scan | #Spec | Start | End | PTM |
| --- | --- | --- | --- | --- | --- | --- | --- | --- | --- | --- | --- | --- |
| A.AFPPDVAGN.V | Y | 29.50 | 886.4185 | -0.5 | 444.2163 | 2 | 11.38 | 1567 | 3 | 144 | 152 |  |
| T.IIDQNRDGIISKD.D | Y | 24.69 | 1485.7787 | 0.3 | 743.8969 | 2 | 10.47 | 1413 | 1 | 38 | 50 |  |
| S.KDDLRDVLAS.M | Y | 24.23 | 1130.5931 | -0.5 | 566.3036 | 2 | 11.55 | 1597 | 1 | 49 | 58 |  |
| K.LKGADPEDVILS.A | Y | 19.92 | 1255.6659 | -0.1 | 628.8401 | 2 | 12.66 | 1790 | 2 | 93 | 104 |  |
| K.GADPEDVILS.A | Y | 15.25 | 1014.4869 | -0.1 | 508.2507 | 2 | 12.72 | 1801 | 1 | 95 | 104 |  |
| F.GEKLKGADPEDVILS.A | Y | 15.24 | 1569.8250 | -0.6 | 785.9193 | 2 | 11.75 | 1631 | 1 | 90 | 104 |  |
| total 6 peptides |  |  |  |  |  |  |  |  |  |  |  |  |
| --- | --- | --- | --- | --- | --- | --- | --- | --- | --- | --- | --- | --- |

tr|A0A7N8XBQ9|A0A7N8XBQ9\_9TELE

back to list

  

| Protein Coverage
| Supporting Peptides
|

Protein Coverage:

Supporting Peptides:

| Peptide | Uniq | -10lgP | Mass | ppm | m/z | z | RT | Scan | #Spec | Start | End | PTM |
| --- | --- | --- | --- | --- | --- | --- | --- | --- | --- | --- | --- | --- |
| A.AFPPDVAGN.V | Y | 29.50 | 886.4185 | -0.5 | 444.2163 | 2 | 11.38 | 1567 | 3 | 166 | 174 |  |
| T.IIDQNRDGIISKD.D | Y | 24.69 | 1485.7787 | 0.3 | 743.8969 | 2 | 10.47 | 1413 | 1 | 60 | 72 |  |
| S.KDDLRDVLAS.M | Y | 24.23 | 1130.5931 | -0.5 | 566.3036 | 2 | 11.55 | 1597 | 1 | 71 | 80 |  |
| K.LKGADPEDVILS.A | Y | 19.92 | 1255.6659 | -0.1 | 628.8401 | 2 | 12.66 | 1790 | 2 | 115 | 126 |  |
| K.GADPEDVILS.A | Y | 15.25 | 1014.4869 | -0.1 | 508.2507 | 2 | 12.72 | 1801 | 1 | 117 | 126 |  |
| F.GEKLKGADPEDVILS.A | Y | 15.24 | 1569.8250 | -0.6 | 785.9193 | 2 | 11.75 | 1631 | 1 | 112 | 126 |  |
| total 6 peptides |  |  |  |  |  |  |  |  |  |  |  |  |
| --- | --- | --- | --- | --- | --- | --- | --- | --- | --- | --- | --- | --- |

tr|F8U029|F8U029\_EPIBR

back to list

  

| Protein Coverage
| Supporting Peptides
|

Protein Coverage:

Supporting Peptides:

| Peptide | Uniq | -10lgP | Mass | ppm | m/z | z | RT | Scan | #Spec | Start | End | PTM |
| --- | --- | --- | --- | --- | --- | --- | --- | --- | --- | --- | --- | --- |
| A.AFPPDVAGN.V | Y | 29.50 | 886.4185 | -0.5 | 444.2163 | 2 | 11.38 | 1567 | 3 | 144 | 152 |  |
| T.IIDQNRDGIISKD.D | Y | 24.69 | 1485.7787 | 0.3 | 743.8969 | 2 | 10.47 | 1413 | 1 | 38 | 50 |  |
| S.KDDLRDVLAS.L | Y | 24.23 | 1130.5931 | -0.5 | 566.3036 | 2 | 11.55 | 1597 | 1 | 49 | 58 |  |
| K.LKGADPEDVILS.S | Y | 19.92 | 1255.6659 | -0.1 | 628.8401 | 2 | 12.66 | 1790 | 2 | 93 | 104 |  |
| K.GADPEDVILS.S | Y | 15.25 | 1014.4869 | -0.1 | 508.2507 | 2 | 12.72 | 1801 | 1 | 95 | 104 |  |
| F.GEKLKGADPEDVILS.S | Y | 15.24 | 1569.8250 | -0.6 | 785.9193 | 2 | 11.75 | 1631 | 1 | 90 | 104 |  |
| total 6 peptides |  |  |  |  |  |  |  |  |  |  |  |  |
| --- | --- | --- | --- | --- | --- | --- | --- | --- | --- | --- | --- | --- |

tr|A0A3B5RBY4|A0A3B5RBY4\_XIPMA

back to list

  

| Protein Coverage
| Supporting Peptides
|

Protein Coverage:

Supporting Peptides:

| Peptide | Uniq | -10lgP | Mass | ppm | m/z | z | RT | Scan | #Spec | Start | End | PTM |
| --- | --- | --- | --- | --- | --- | --- | --- | --- | --- | --- | --- | --- |
| A.AFPPDVAGN.V | Y | 29.50 | 886.4185 | -0.5 | 444.2163 | 2 | 11.38 | 1567 | 3 | 223 | 231 |  |
| T.IIDQNRDGIISKD.D | Y | 24.69 | 1485.7787 | 0.3 | 743.8969 | 2 | 10.47 | 1413 | 1 | 117 | 129 |  |
| S.KDDLRDVLAS.M | Y | 24.23 | 1130.5931 | -0.5 | 566.3036 | 2 | 11.55 | 1597 | 1 | 128 | 137 |  |
| K.LKGADPEDVILS.A | Y | 19.92 | 1255.6659 | -0.1 | 628.8401 | 2 | 12.66 | 1790 | 2 | 172 | 183 |  |
| K.GADPEDVILS.A | Y | 15.25 | 1014.4869 | -0.1 | 508.2507 | 2 | 12.72 | 1801 | 1 | 174 | 183 |  |
| F.GEKLKGADPEDVILS.A | Y | 15.24 | 1569.8250 | -0.6 | 785.9193 | 2 | 11.75 | 1631 | 1 | 169 | 183 |  |
| total 6 peptides |  |  |  |  |  |  |  |  |  |  |  |  |
| --- | --- | --- | --- | --- | --- | --- | --- | --- | --- | --- | --- | --- |

tr|G3N515|G3N515\_GASAC

back to list

  

| Protein Coverage
| Supporting Peptides
|

Protein Coverage:

Supporting Peptides:

| Peptide | Uniq | -10lgP | Mass | ppm | m/z | z | RT | Scan | #Spec | Start | End | PTM |
| --- | --- | --- | --- | --- | --- | --- | --- | --- | --- | --- | --- | --- |
| A.ILPPGPPTPW.I | N | 38.27 | 1073.5909 | 0.1 | 537.8028 | 2 | 13.74 | 1976 | 1 | 3917 | 3926 |  |
| L.IVEELPVKW.L | N | 27.75 | 1111.6277 | -1.0 | 556.8206 | 2 | 13.27 | 1897 | 1 | 130 | 138 |  |
| N.LDVIDVPAPVKN.L | Y | 17.46 | 1278.7183 | -0.5 | 640.3661 | 2 | 12.62 | 1784 | 1 | 1994 | 2005 |  |
| total 3 peptides |  |  |  |  |  |  |  |  |  |  |  |  |
| --- | --- | --- | --- | --- | --- | --- | --- | --- | --- | --- | --- | --- |

tr|Q4T444|Q4T444\_TETNG

back to list

  

| Protein Coverage
| Supporting Peptides
|

Protein Coverage:

Supporting Peptides:

| Peptide | Uniq | -10lgP | Mass | ppm | m/z | z | RT | Scan | #Spec | Start | End | PTM |
| --- | --- | --- | --- | --- | --- | --- | --- | --- | --- | --- | --- | --- |
| A.ILPPGPPTPW.I | N | 38.27 | 1073.5909 | 0.1 | 537.8028 | 2 | 13.74 | 1976 | 1 | 4399 | 4408 |  |
| L.IVEELPVKW.V | N | 27.75 | 1111.6277 | -1.0 | 556.8206 | 2 | 13.27 | 1897 | 1 | 625 | 633 |  |
| F.LLWEPPK.Y | Y | 16.69 | 881.5010 | -0.4 | 441.7576 | 2 | 11.92 | 1663 | 1 | 3071 | 3077 |  |
| total 3 peptides |  |  |  |  |  |  |  |  |  |  |  |  |
| --- | --- | --- | --- | --- | --- | --- | --- | --- | --- | --- | --- | --- |

tr|D2KQG3|D2KQG3\_SINCH

back to list

  

| Protein Coverage
| Supporting Peptides
|

Protein Coverage:

Supporting Peptides:

| Peptide | Uniq | -10lgP | Mass | ppm | m/z | z | RT | Scan | #Spec | Start | End | PTM |
| --- | --- | --- | --- | --- | --- | --- | --- | --- | --- | --- | --- | --- |
| F.EVIDQDKSGFIE.E | Y | 45.95 | 1378.6616 | -0.9 | 690.3375 | 2 | 12.49 | 1760 | 7 | 49 | 60 |  |
| A.FEVIDQDKSGFIE.E | Y | 21.69 | 1525.7300 | -1.1 | 763.8714 | 2 | 12.69 | 1796 | 1 | 48 | 60 |  |
| total 2 peptides |  |  |  |  |  |  |  |  |  |  |  |  |
| --- | --- | --- | --- | --- | --- | --- | --- | --- | --- | --- | --- | --- |

tr|B9VJM3|B9VJM3\_SINCH

back to list

  

| Protein Coverage
| Supporting Peptides
|

Protein Coverage:

Supporting Peptides:

| Peptide | Uniq | -10lgP | Mass | ppm | m/z | z | RT | Scan | #Spec | Start | End | PTM |
| --- | --- | --- | --- | --- | --- | --- | --- | --- | --- | --- | --- | --- |
| F.EVIDQDKSGFIE.E | Y | 45.95 | 1378.6616 | -0.9 | 690.3375 | 2 | 12.49 | 1760 | 7 | 49 | 60 |  |
| A.FEVIDQDKSGFIE.E | Y | 21.69 | 1525.7300 | -1.1 | 763.8714 | 2 | 12.69 | 1796 | 1 | 48 | 60 |  |
| total 2 peptides |  |  |  |  |  |  |  |  |  |  |  |  |
| --- | --- | --- | --- | --- | --- | --- | --- | --- | --- | --- | --- | --- |

tr|A0A3P8TDG8|A0A3P8TDG8\_AMPPE

back to list

  

| Protein Coverage
| Supporting Peptides
|

Protein Coverage:

Supporting Peptides:

| Peptide | Uniq | -10lgP | Mass | ppm | m/z | z | RT | Scan | #Spec | Start | End | PTM |
| --- | --- | --- | --- | --- | --- | --- | --- | --- | --- | --- | --- | --- |
| K.VDSELPVDDVFAQV.S | Y | 30.65 | 1531.7406 | 0.1 | 766.8776 | 2 | 14.97 | 2185 | 1 | 173 | 186 |  |
| P.LDTVLDMIKDA.M | N | 20.90 | 1232.6322 | -0.8 | 617.3229 | 2 | 14.44 | 2094 | 1 | 69 | 79 |  |
| L.DTVLDMIK.D | N | 17.70 | 933.4841 | -1.1 | 467.7488 | 2 | 13.23 | 1891 | 1 | 70 | 77 |  |
| D.TVLDMIKDA.M | N | 16.00 | 1004.5212 | -1.1 | 503.2673 | 2 | 13.42 | 1923 | 1 | 71 | 79 |  |
| K.VDSELPVDDVF.A | N | 15.68 | 1233.5764 | -0.5 | 617.7952 | 2 | 14.17 | 2049 | 1 | 173 | 183 |  |
| total 5 peptides |  |  |  |  |  |  |  |  |  |  |  |  |
| --- | --- | --- | --- | --- | --- | --- | --- | --- | --- | --- | --- | --- |

tr|A0A556TIT9|A0A556TIT9\_BAGYA

back to list

  

| Protein Coverage
| Supporting Peptides
|

Protein Coverage:

Supporting Peptides:

| Peptide | Uniq | -10lgP | Mass | ppm | m/z | z | RT | Scan | #Spec | Start | End | PTM |
| --- | --- | --- | --- | --- | --- | --- | --- | --- | --- | --- | --- | --- |
| S.GFIEEEELK.L | N | 31.42 | 1092.5338 | -0.1 | 547.2741 | 2 | 11.80 | 1641 | 1 | 57 | 65 |  |
| Q.DKSGFIEEEELK.L | N | 25.85 | 1422.6877 | -0.4 | 712.3508 | 2 | 11.40 | 1570 | 1 | 54 | 65 |  |
| F.LAAGDSDGDGKIGVD.A | N | 25.04 | 1388.6418 | -0.5 | 695.3278 | 2 | 10.74 | 1456 | 1 | 87 | 101 |  |
| total 3 peptides |  |  |  |  |  |  |  |  |  |  |  |  |
| --- | --- | --- | --- | --- | --- | --- | --- | --- | --- | --- | --- | --- |

tr|A0A484CUS5|A0A484CUS5\_PERFV

back to list

  

| Protein Coverage
| Supporting Peptides
|

Protein Coverage:

Supporting Peptides:

| Peptide | Uniq | -10lgP | Mass | ppm | m/z | z | RT | Scan | #Spec | Start | End | PTM |
| --- | --- | --- | --- | --- | --- | --- | --- | --- | --- | --- | --- | --- |
| A.ILPPGPPTPW.I | N | 38.27 | 1073.5909 | 0.1 | 537.8028 | 2 | 13.74 | 1976 | 1 | 1746 | 1755 |  |
| T.VVDRPTPPVGPVVF.E | Y | 27.22 | 1477.8292 | -1.8 | 739.9206 | 2 | 13.07 | 1861 | 1 | 3654 | 3667 |  |
| total 2 peptides |  |  |  |  |  |  |  |  |  |  |  |  |
| --- | --- | --- | --- | --- | --- | --- | --- | --- | --- | --- | --- | --- |

tr|A0A2I4CWS8|A0A2I4CWS8\_9TELE

back to list

  

| Protein Coverage
| Supporting Peptides
|

Protein Coverage:

Supporting Peptides:

| Peptide | Uniq | -10lgP | Mass | ppm | m/z | z | RT | Scan | #Spec | Start | End | PTM |
| --- | --- | --- | --- | --- | --- | --- | --- | --- | --- | --- | --- | --- |
| Y.TGREFDDLPLHEQ.S | Y | 28.57 | 1555.7267 | -0.4 | 778.8703 | 2 | 11.70 | 1623 | 1 | 651 | 663 |  |
| E.IVPGDIVEV.S | Y | 24.00 | 939.5276 | -0.7 | 470.7708 | 2 | 13.35 | 1910 | 1 | 145 | 153 |  |
| E.FDDLPLHEQ.S | Y | 19.12 | 1112.5138 | -0.1 | 557.2641 | 2 | 11.86 | 1652 | 1 | 655 | 663 |  |
| total 3 peptides |  |  |  |  |  |  |  |  |  |  |  |  |
| --- | --- | --- | --- | --- | --- | --- | --- | --- | --- | --- | --- | --- |

tr|A0A3P9C9M1|A0A3P9C9M1\_9CICH

back to list

  

| Protein Coverage
| Supporting Peptides
|

Protein Coverage:

Supporting Peptides:

| Peptide | Uniq | -10lgP | Mass | ppm | m/z | z | RT | Scan | #Spec | Start | End | PTM |
| --- | --- | --- | --- | --- | --- | --- | --- | --- | --- | --- | --- | --- |
| Y.TGREFDDLPLHEQ.S | Y | 28.57 | 1555.7267 | -0.4 | 778.8703 | 2 | 11.70 | 1623 | 1 | 651 | 663 |  |
| E.IVPGDIVEV.S | Y | 24.00 | 939.5276 | -0.7 | 470.7708 | 2 | 13.35 | 1910 | 1 | 145 | 153 |  |
| E.FDDLPLHEQ.S | Y | 19.12 | 1112.5138 | -0.1 | 557.2641 | 2 | 11.86 | 1652 | 1 | 655 | 663 |  |
| total 3 peptides |  |  |  |  |  |  |  |  |  |  |  |  |
| --- | --- | --- | --- | --- | --- | --- | --- | --- | --- | --- | --- | --- |

tr|A0A7J6CZM2|A0A7J6CZM2\_9TELE

back to list

  

| Protein Coverage
| Supporting Peptides
|

Protein Coverage:

Supporting Peptides:

| Peptide | Uniq | -10lgP | Mass | ppm | m/z | z | RT | Scan | #Spec | Start | End | PTM |
| --- | --- | --- | --- | --- | --- | --- | --- | --- | --- | --- | --- | --- |
| V.TVEDPFDQDDWE.A | Y | 43.01 | 1494.5786 | -0.2 | 748.2964 | 2 | 14.25 | 2062 | 1 | 344 | 355 |  |
| total 1 peptides |  |  |  |  |  |  |  |  |  |  |  |  |
| --- | --- | --- | --- | --- | --- | --- | --- | --- | --- | --- | --- | --- |

tr|A0A0F8AZU0|A0A0F8AZU0\_LARCR

back to list

  

| Protein Coverage
| Supporting Peptides
|

Protein Coverage:

Supporting Peptides:

| Peptide | Uniq | -10lgP | Mass | ppm | m/z | z | RT | Scan | #Spec | Start | End | PTM |
| --- | --- | --- | --- | --- | --- | --- | --- | --- | --- | --- | --- | --- |
| S.GFIEEEELK.L | N | 31.42 | 1092.5338 | -0.1 | 547.2741 | 2 | 11.80 | 1641 | 1 | 57 | 65 |  |
| R.EGEEEK.K | N | 16.22 | 719.2973 | 0.9 | 360.6562 | 2 | 7.44 | 989 | 1 | 269 | 274 |  |
| total 2 peptides |  |  |  |  |  |  |  |  |  |  |  |  |
| --- | --- | --- | --- | --- | --- | --- | --- | --- | --- | --- | --- | --- |

tr|A0A4W4GVK8|A0A4W4GVK8\_ELEEL

back to list

  

| Protein Coverage
| Supporting Peptides
|

Protein Coverage:

Supporting Peptides:

| Peptide | Uniq | -10lgP | Mass | ppm | m/z | z | RT | Scan | #Spec | Start | End | PTM |
| --- | --- | --- | --- | --- | --- | --- | --- | --- | --- | --- | --- | --- |
| R.VFDKEGNGTVM.G | Y | 37.84 | 1195.5543 | -0.9 | 598.7839 | 2 | 11.12 | 1522 | 1 | 92 | 102 |  |
| total 1 peptides |  |  |  |  |  |  |  |  |  |  |  |  |
| --- | --- | --- | --- | --- | --- | --- | --- | --- | --- | --- | --- | --- |

tr|A0A4W4GPW6|A0A4W4GPW6\_ELEEL

back to list

  

| Protein Coverage
| Supporting Peptides
|

Protein Coverage:

Supporting Peptides:

| Peptide | Uniq | -10lgP | Mass | ppm | m/z | z | RT | Scan | #Spec | Start | End | PTM |
| --- | --- | --- | --- | --- | --- | --- | --- | --- | --- | --- | --- | --- |
| R.VFDKEGNGTVM.G | Y | 37.84 | 1195.5543 | -0.9 | 598.7839 | 2 | 11.12 | 1522 | 1 | 94 | 104 |  |
| total 1 peptides |  |  |  |  |  |  |  |  |  |  |  |  |
| --- | --- | --- | --- | --- | --- | --- | --- | --- | --- | --- | --- | --- |

tr|A0A4W4GW96|A0A4W4GW96\_ELEEL

back to list

  

| Protein Coverage
| Supporting Peptides
|

Protein Coverage:

Supporting Peptides:

| Peptide | Uniq | -10lgP | Mass | ppm | m/z | z | RT | Scan | #Spec | Start | End | PTM |
| --- | --- | --- | --- | --- | --- | --- | --- | --- | --- | --- | --- | --- |
| R.VFDKEGNGTVM.G | Y | 37.84 | 1195.5543 | -0.9 | 598.7839 | 2 | 11.12 | 1522 | 1 | 95 | 105 |  |
| total 1 peptides |  |  |  |  |  |  |  |  |  |  |  |  |
| --- | --- | --- | --- | --- | --- | --- | --- | --- | --- | --- | --- | --- |

tr|A0A665WHZ7|A0A665WHZ7\_ECHNA

back to list

  

| Protein Coverage
| Supporting Peptides
|

Protein Coverage:

Supporting Peptides:

| Peptide | Uniq | -10lgP | Mass | ppm | m/z | z | RT | Scan | #Spec | Start | End | PTM |
| --- | --- | --- | --- | --- | --- | --- | --- | --- | --- | --- | --- | --- |
| R.VFDKEGNGTVM.G | Y | 37.84 | 1195.5543 | -0.9 | 598.7839 | 2 | 11.12 | 1522 | 1 | 89 | 99 |  |
| total 1 peptides |  |  |  |  |  |  |  |  |  |  |  |  |
| --- | --- | --- | --- | --- | --- | --- | --- | --- | --- | --- | --- | --- |

tr|A0A3B4FQL1|A0A3B4FQL1\_9CICH

back to list

  

| Protein Coverage
| Supporting Peptides
|

Protein Coverage:

Supporting Peptides:

| Peptide | Uniq | -10lgP | Mass | ppm | m/z | z | RT | Scan | #Spec | Start | End | PTM |
| --- | --- | --- | --- | --- | --- | --- | --- | --- | --- | --- | --- | --- |
| R.VFDKEGNGTVM.G | Y | 37.84 | 1195.5543 | -0.9 | 598.7839 | 2 | 11.12 | 1522 | 1 | 94 | 104 |  |
| total 1 peptides |  |  |  |  |  |  |  |  |  |  |  |  |
| --- | --- | --- | --- | --- | --- | --- | --- | --- | --- | --- | --- | --- |

tr|A0A3P8PB88|A0A3P8PB88\_ASTCA

back to list

  

| Protein Coverage
| Supporting Peptides
|

Protein Coverage:

Supporting Peptides:

| Peptide | Uniq | -10lgP | Mass | ppm | m/z | z | RT | Scan | #Spec | Start | End | PTM |
| --- | --- | --- | --- | --- | --- | --- | --- | --- | --- | --- | --- | --- |
| R.VFDKEGNGTVM.G | Y | 37.84 | 1195.5543 | -0.9 | 598.7839 | 2 | 11.12 | 1522 | 1 | 94 | 104 |  |
| total 1 peptides |  |  |  |  |  |  |  |  |  |  |  |  |
| --- | --- | --- | --- | --- | --- | --- | --- | --- | --- | --- | --- | --- |

tr|A0A665WHJ1|A0A665WHJ1\_ECHNA

back to list

  

| Protein Coverage
| Supporting Peptides
|

Protein Coverage:

Supporting Peptides:

| Peptide | Uniq | -10lgP | Mass | ppm | m/z | z | RT | Scan | #Spec | Start | End | PTM |
| --- | --- | --- | --- | --- | --- | --- | --- | --- | --- | --- | --- | --- |
| R.VFDKEGNGTVM.G | Y | 37.84 | 1195.5543 | -0.9 | 598.7839 | 2 | 11.12 | 1522 | 1 | 94 | 104 |  |
| total 1 peptides |  |  |  |  |  |  |  |  |  |  |  |  |
| --- | --- | --- | --- | --- | --- | --- | --- | --- | --- | --- | --- | --- |

tr|F2WR27|F2WR27\_EPICO

back to list

  

| Protein Coverage
| Supporting Peptides
|

Protein Coverage:

Supporting Peptides:

| Peptide | Uniq | -10lgP | Mass | ppm | m/z | z | RT | Scan | #Spec | Start | End | PTM |
| --- | --- | --- | --- | --- | --- | --- | --- | --- | --- | --- | --- | --- |
| R.VFDKEGNGTVM.G | Y | 37.84 | 1195.5543 | -0.9 | 598.7839 | 2 | 11.12 | 1522 | 1 | 94 | 104 |  |
| total 1 peptides |  |  |  |  |  |  |  |  |  |  |  |  |
| --- | --- | --- | --- | --- | --- | --- | --- | --- | --- | --- | --- | --- |

tr|A0A4W6FFD1|A0A4W6FFD1\_LATCA

back to list

  

| Protein Coverage
| Supporting Peptides
|

Protein Coverage:

Supporting Peptides:

| Peptide | Uniq | -10lgP | Mass | ppm | m/z | z | RT | Scan | #Spec | Start | End | PTM |
| --- | --- | --- | --- | --- | --- | --- | --- | --- | --- | --- | --- | --- |
| R.VFDKEGNGTVM.G | Y | 37.84 | 1195.5543 | -0.9 | 598.7839 | 2 | 11.12 | 1522 | 1 | 94 | 104 |  |
| total 1 peptides |  |  |  |  |  |  |  |  |  |  |  |  |
| --- | --- | --- | --- | --- | --- | --- | --- | --- | --- | --- | --- | --- |

tr|A0A3B3C1H6|A0A3B3C1H6\_ORYME

back to list

  

| Protein Coverage
| Supporting Peptides
|

Protein Coverage:

Supporting Peptides:

| Peptide | Uniq | -10lgP | Mass | ppm | m/z | z | RT | Scan | #Spec | Start | End | PTM |
| --- | --- | --- | --- | --- | --- | --- | --- | --- | --- | --- | --- | --- |
| R.VFDKEGNGTVM.G | Y | 37.84 | 1195.5543 | -0.9 | 598.7839 | 2 | 11.12 | 1522 | 1 | 94 | 104 |  |
| total 1 peptides |  |  |  |  |  |  |  |  |  |  |  |  |
| --- | --- | --- | --- | --- | --- | --- | --- | --- | --- | --- | --- | --- |

tr|A0A3Q1B309|A0A3Q1B309\_AMPOC

back to list

  

| Protein Coverage
| Supporting Peptides
|

Protein Coverage:

Supporting Peptides:

| Peptide | Uniq | -10lgP | Mass | ppm | m/z | z | RT | Scan | #Spec | Start | End | PTM |
| --- | --- | --- | --- | --- | --- | --- | --- | --- | --- | --- | --- | --- |
| R.VFDKEGNGTVM.G | Y | 37.84 | 1195.5543 | -0.9 | 598.7839 | 2 | 11.12 | 1522 | 1 | 94 | 104 |  |
| total 1 peptides |  |  |  |  |  |  |  |  |  |  |  |  |
| --- | --- | --- | --- | --- | --- | --- | --- | --- | --- | --- | --- | --- |

tr|A0A3B4VIP0|A0A3B4VIP0\_SERDU

back to list

  

| Protein Coverage
| Supporting Peptides
|

Protein Coverage:

Supporting Peptides:

| Peptide | Uniq | -10lgP | Mass | ppm | m/z | z | RT | Scan | #Spec | Start | End | PTM |
| --- | --- | --- | --- | --- | --- | --- | --- | --- | --- | --- | --- | --- |
| R.VFDKEGNGTVM.G | Y | 37.84 | 1195.5543 | -0.9 | 598.7839 | 2 | 11.12 | 1522 | 1 | 103 | 113 |  |
| total 1 peptides |  |  |  |  |  |  |  |  |  |  |  |  |
| --- | --- | --- | --- | --- | --- | --- | --- | --- | --- | --- | --- | --- |

tr|A0A3B5RBB9|A0A3B5RBB9\_XIPMA

back to list

  

| Protein Coverage
| Supporting Peptides
|

Protein Coverage:

Supporting Peptides:

| Peptide | Uniq | -10lgP | Mass | ppm | m/z | z | RT | Scan | #Spec | Start | End | PTM |
| --- | --- | --- | --- | --- | --- | --- | --- | --- | --- | --- | --- | --- |
| R.VFDKEGNGTVM.G | Y | 37.84 | 1195.5543 | -0.9 | 598.7839 | 2 | 11.12 | 1522 | 1 | 104 | 114 |  |
| total 1 peptides |  |  |  |  |  |  |  |  |  |  |  |  |
| --- | --- | --- | --- | --- | --- | --- | --- | --- | --- | --- | --- | --- |

tr|A0A3B5R1L0|A0A3B5R1L0\_XIPMA

back to list

  

| Protein Coverage
| Supporting Peptides
|

Protein Coverage:

Supporting Peptides:

| Peptide | Uniq | -10lgP | Mass | ppm | m/z | z | RT | Scan | #Spec | Start | End | PTM |
| --- | --- | --- | --- | --- | --- | --- | --- | --- | --- | --- | --- | --- |
| R.VFDKEGNGTVM.G | Y | 37.84 | 1195.5543 | -0.9 | 598.7839 | 2 | 11.12 | 1522 | 1 | 105 | 115 |  |
| total 1 peptides |  |  |  |  |  |  |  |  |  |  |  |  |
| --- | --- | --- | --- | --- | --- | --- | --- | --- | --- | --- | --- | --- |

tr|A0A3P9CCX0|A0A3P9CCX0\_9CICH

back to list

  

| Protein Coverage
| Supporting Peptides
|

Protein Coverage:

Supporting Peptides:

| Peptide | Uniq | -10lgP | Mass | ppm | m/z | z | RT | Scan | #Spec | Start | End | PTM |
| --- | --- | --- | --- | --- | --- | --- | --- | --- | --- | --- | --- | --- |
| R.VFDKEGNGTVM.G | Y | 37.84 | 1195.5543 | -0.9 | 598.7839 | 2 | 11.12 | 1522 | 1 | 94 | 104 |  |
| total 1 peptides |  |  |  |  |  |  |  |  |  |  |  |  |
| --- | --- | --- | --- | --- | --- | --- | --- | --- | --- | --- | --- | --- |

tr|A0A665WHZ4|A0A665WHZ4\_ECHNA

back to list

  

| Protein Coverage
| Supporting Peptides
|

Protein Coverage:

Supporting Peptides:

| Peptide | Uniq | -10lgP | Mass | ppm | m/z | z | RT | Scan | #Spec | Start | End | PTM |
| --- | --- | --- | --- | --- | --- | --- | --- | --- | --- | --- | --- | --- |
| R.VFDKEGNGTVM.G | Y | 37.84 | 1195.5543 | -0.9 | 598.7839 | 2 | 11.12 | 1522 | 1 | 108 | 118 |  |
| total 1 peptides |  |  |  |  |  |  |  |  |  |  |  |  |
| --- | --- | --- | --- | --- | --- | --- | --- | --- | --- | --- | --- | --- |

tr|A0A4W6FCG2|A0A4W6FCG2\_LATCA

back to list

  

| Protein Coverage
| Supporting Peptides
|

Protein Coverage:

Supporting Peptides:

| Peptide | Uniq | -10lgP | Mass | ppm | m/z | z | RT | Scan | #Spec | Start | End | PTM |
| --- | --- | --- | --- | --- | --- | --- | --- | --- | --- | --- | --- | --- |
| R.VFDKEGNGTVM.G | Y | 37.84 | 1195.5543 | -0.9 | 598.7839 | 2 | 11.12 | 1522 | 1 | 89 | 99 |  |
| total 1 peptides |  |  |  |  |  |  |  |  |  |  |  |  |
| --- | --- | --- | --- | --- | --- | --- | --- | --- | --- | --- | --- | --- |

tr|A0A4W6FFD6|A0A4W6FFD6\_LATCA

back to list

  

| Protein Coverage
| Supporting Peptides
|

Protein Coverage:

Supporting Peptides:

| Peptide | Uniq | -10lgP | Mass | ppm | m/z | z | RT | Scan | #Spec | Start | End | PTM |
| --- | --- | --- | --- | --- | --- | --- | --- | --- | --- | --- | --- | --- |
| R.VFDKEGNGTVM.G | Y | 37.84 | 1195.5543 | -0.9 | 598.7839 | 2 | 11.12 | 1522 | 1 | 91 | 101 |  |
| total 1 peptides |  |  |  |  |  |  |  |  |  |  |  |  |
| --- | --- | --- | --- | --- | --- | --- | --- | --- | --- | --- | --- | --- |

tr|A0A665WI19|A0A665WI19\_ECHNA

back to list

  

| Protein Coverage
| Supporting Peptides
|

Protein Coverage:

Supporting Peptides:

| Peptide | Uniq | -10lgP | Mass | ppm | m/z | z | RT | Scan | #Spec | Start | End | PTM |
| --- | --- | --- | --- | --- | --- | --- | --- | --- | --- | --- | --- | --- |
| R.VFDKEGNGTVM.G | Y | 37.84 | 1195.5543 | -0.9 | 598.7839 | 2 | 11.12 | 1522 | 1 | 115 | 125 |  |
| total 1 peptides |  |  |  |  |  |  |  |  |  |  |  |  |
| --- | --- | --- | --- | --- | --- | --- | --- | --- | --- | --- | --- | --- |

tr|B9V2Y1|B9V2Y1\_EPICO

back to list

  

| Protein Coverage
| Supporting Peptides
|

Protein Coverage:

Supporting Peptides:

| Peptide | Uniq | -10lgP | Mass | ppm | m/z | z | RT | Scan | #Spec | Start | End | PTM |
| --- | --- | --- | --- | --- | --- | --- | --- | --- | --- | --- | --- | --- |
| R.VFDKEGNGTVM.G | Y | 37.84 | 1195.5543 | -0.9 | 598.7839 | 2 | 11.12 | 1522 | 1 | 115 | 125 |  |
| total 1 peptides |  |  |  |  |  |  |  |  |  |  |  |  |
| --- | --- | --- | --- | --- | --- | --- | --- | --- | --- | --- | --- | --- |

tr|A0A4W6FDH6|A0A4W6FDH6\_LATCA

back to list

  

| Protein Coverage
| Supporting Peptides
|

Protein Coverage:

Supporting Peptides:

| Peptide | Uniq | -10lgP | Mass | ppm | m/z | z | RT | Scan | #Spec | Start | End | PTM |
| --- | --- | --- | --- | --- | --- | --- | --- | --- | --- | --- | --- | --- |
| R.VFDKEGNGTVM.G | Y | 37.84 | 1195.5543 | -0.9 | 598.7839 | 2 | 11.12 | 1522 | 1 | 101 | 111 |  |
| total 1 peptides |  |  |  |  |  |  |  |  |  |  |  |  |
| --- | --- | --- | --- | --- | --- | --- | --- | --- | --- | --- | --- | --- |

tr|A0A669EYH8|A0A669EYH8\_ORENI

back to list

  

| Protein Coverage
| Supporting Peptides
|

Protein Coverage:

Supporting Peptides:

| Peptide | Uniq | -10lgP | Mass | ppm | m/z | z | RT | Scan | #Spec | Start | End | PTM |
| --- | --- | --- | --- | --- | --- | --- | --- | --- | --- | --- | --- | --- |
| R.VFDKEGNGTVM.G | Y | 37.84 | 1195.5543 | -0.9 | 598.7839 | 2 | 11.12 | 1522 | 1 | 94 | 104 |  |
| total 1 peptides |  |  |  |  |  |  |  |  |  |  |  |  |
| --- | --- | --- | --- | --- | --- | --- | --- | --- | --- | --- | --- | --- |

tr|A0A3B4VK07|A0A3B4VK07\_SERDU

back to list

  

| Protein Coverage
| Supporting Peptides
|

Protein Coverage:

Supporting Peptides:

| Peptide | Uniq | -10lgP | Mass | ppm | m/z | z | RT | Scan | #Spec | Start | End | PTM |
| --- | --- | --- | --- | --- | --- | --- | --- | --- | --- | --- | --- | --- |
| R.VFDKEGNGTVM.G | Y | 37.84 | 1195.5543 | -0.9 | 598.7839 | 2 | 11.12 | 1522 | 1 | 163 | 173 |  |
| total 1 peptides |  |  |  |  |  |  |  |  |  |  |  |  |
| --- | --- | --- | --- | --- | --- | --- | --- | --- | --- | --- | --- | --- |

tr|A0A3B4VK38|A0A3B4VK38\_SERDU

back to list

  

| Protein Coverage
| Supporting Peptides
|

Protein Coverage:

Supporting Peptides:

| Peptide | Uniq | -10lgP | Mass | ppm | m/z | z | RT | Scan | #Spec | Start | End | PTM |
| --- | --- | --- | --- | --- | --- | --- | --- | --- | --- | --- | --- | --- |
| R.VFDKEGNGTVM.G | Y | 37.84 | 1195.5543 | -0.9 | 598.7839 | 2 | 11.12 | 1522 | 1 | 163 | 173 |  |
| total 1 peptides |  |  |  |  |  |  |  |  |  |  |  |  |
| --- | --- | --- | --- | --- | --- | --- | --- | --- | --- | --- | --- | --- |

tr|A0A834C4M6|A0A834C4M6\_ORYME

back to list

  

| Protein Coverage
| Supporting Peptides
|

Protein Coverage:

Supporting Peptides:

| Peptide | Uniq | -10lgP | Mass | ppm | m/z | z | RT | Scan | #Spec | Start | End | PTM |
| --- | --- | --- | --- | --- | --- | --- | --- | --- | --- | --- | --- | --- |
| R.VFDKEGNGTVM.G | Y | 37.84 | 1195.5543 | -0.9 | 598.7839 | 2 | 11.12 | 1522 | 1 | 106 | 116 |  |
| total 1 peptides |  |  |  |  |  |  |  |  |  |  |  |  |
| --- | --- | --- | --- | --- | --- | --- | --- | --- | --- | --- | --- | --- |

tr|A0A315W7Q8|A0A315W7Q8\_GAMAF

back to list

  

| Protein Coverage
| Supporting Peptides
|

Protein Coverage:

Supporting Peptides:

| Peptide | Uniq | -10lgP | Mass | ppm | m/z | z | RT | Scan | #Spec | Start | End | PTM |
| --- | --- | --- | --- | --- | --- | --- | --- | --- | --- | --- | --- | --- |
| R.VFDKEGNGTVM.G | Y | 37.84 | 1195.5543 | -0.9 | 598.7839 | 2 | 11.12 | 1522 | 1 | 128 | 138 |  |
| total 1 peptides |  |  |  |  |  |  |  |  |  |  |  |  |
| --- | --- | --- | --- | --- | --- | --- | --- | --- | --- | --- | --- | --- |

tr|A0A672K127|A0A672K127\_SINGR

back to list

  

| Protein Coverage
| Supporting Peptides
|

Protein Coverage:

Supporting Peptides:

| Peptide | Uniq | -10lgP | Mass | ppm | m/z | z | RT | Scan | #Spec | Start | End | PTM |
| --- | --- | --- | --- | --- | --- | --- | --- | --- | --- | --- | --- | --- |
| R.VFDKEGNGTVM.G | Y | 37.84 | 1195.5543 | -0.9 | 598.7839 | 2 | 11.12 | 1522 | 1 | 95 | 105 |  |
| total 1 peptides |  |  |  |  |  |  |  |  |  |  |  |  |
| --- | --- | --- | --- | --- | --- | --- | --- | --- | --- | --- | --- | --- |

tr|A0A7J6DIX6|A0A7J6DIX6\_9TELE

back to list

  

| Protein Coverage
| Supporting Peptides
|

Protein Coverage:

Supporting Peptides:

| Peptide | Uniq | -10lgP | Mass | ppm | m/z | z | RT | Scan | #Spec | Start | End | PTM |
| --- | --- | --- | --- | --- | --- | --- | --- | --- | --- | --- | --- | --- |
| R.VFDKEGNGTVM.G | Y | 37.84 | 1195.5543 | -0.9 | 598.7839 | 2 | 11.12 | 1522 | 1 | 95 | 105 |  |
| total 1 peptides |  |  |  |  |  |  |  |  |  |  |  |  |
| --- | --- | --- | --- | --- | --- | --- | --- | --- | --- | --- | --- | --- |

tr|A0A4W5MNE5|A0A4W5MNE5\_9TELE

back to list

  

| Protein Coverage
| Supporting Peptides
|

Protein Coverage:

Supporting Peptides:

| Peptide | Uniq | -10lgP | Mass | ppm | m/z | z | RT | Scan | #Spec | Start | End | PTM |
| --- | --- | --- | --- | --- | --- | --- | --- | --- | --- | --- | --- | --- |
| R.VFDKEGNGTVM.G | Y | 37.84 | 1195.5543 | -0.9 | 598.7839 | 2 | 11.12 | 1522 | 1 | 87 | 97 |  |
| total 1 peptides |  |  |  |  |  |  |  |  |  |  |  |  |
| --- | --- | --- | --- | --- | --- | --- | --- | --- | --- | --- | --- | --- |

tr|A0A4W5MRK6|A0A4W5MRK6\_9TELE

back to list

  

| Protein Coverage
| Supporting Peptides
|

Protein Coverage:

Supporting Peptides:

| Peptide | Uniq | -10lgP | Mass | ppm | m/z | z | RT | Scan | #Spec | Start | End | PTM |
| --- | --- | --- | --- | --- | --- | --- | --- | --- | --- | --- | --- | --- |
| R.VFDKEGNGTVM.G | Y | 37.84 | 1195.5543 | -0.9 | 598.7839 | 2 | 11.12 | 1522 | 1 | 96 | 106 |  |
| total 1 peptides |  |  |  |  |  |  |  |  |  |  |  |  |
| --- | --- | --- | --- | --- | --- | --- | --- | --- | --- | --- | --- | --- |

tr|A0A4W5MMD2|A0A4W5MMD2\_9TELE

back to list

  

| Protein Coverage
| Supporting Peptides
|

Protein Coverage:

Supporting Peptides:

| Peptide | Uniq | -10lgP | Mass | ppm | m/z | z | RT | Scan | #Spec | Start | End | PTM |
| --- | --- | --- | --- | --- | --- | --- | --- | --- | --- | --- | --- | --- |
| R.VFDKEGNGTVM.G | Y | 37.84 | 1195.5543 | -0.9 | 598.7839 | 2 | 11.12 | 1522 | 1 | 122 | 132 |  |
| total 1 peptides |  |  |  |  |  |  |  |  |  |  |  |  |
| --- | --- | --- | --- | --- | --- | --- | --- | --- | --- | --- | --- | --- |

tr|A0A3Q3JIG8|A0A3Q3JIG8\_MONAL

back to list

  

| Protein Coverage
| Supporting Peptides
|

Protein Coverage:

Supporting Peptides:

| Peptide | Uniq | -10lgP | Mass | ppm | m/z | z | RT | Scan | #Spec | Start | End | PTM |
| --- | --- | --- | --- | --- | --- | --- | --- | --- | --- | --- | --- | --- |
| R.VFDKEGNGTVM.G | Y | 37.84 | 1195.5543 | -0.9 | 598.7839 | 2 | 11.12 | 1522 | 1 | 137 | 147 |  |
| total 1 peptides |  |  |  |  |  |  |  |  |  |  |  |  |
| --- | --- | --- | --- | --- | --- | --- | --- | --- | --- | --- | --- | --- |

tr|Q90332|Q90332\_CYPCA

back to list

  

| Protein Coverage
| Supporting Peptides
|

Protein Coverage:

Supporting Peptides:

| Peptide | Uniq | -10lgP | Mass | ppm | m/z | z | RT | Scan | #Spec | Start | End | PTM |
| --- | --- | --- | --- | --- | --- | --- | --- | --- | --- | --- | --- | --- |
| R.VFDKEGNGTVM.G | Y | 37.84 | 1195.5543 | -0.9 | 598.7839 | 2 | 11.12 | 1522 | 1 | 137 | 147 |  |
| total 1 peptides |  |  |  |  |  |  |  |  |  |  |  |  |
| --- | --- | --- | --- | --- | --- | --- | --- | --- | --- | --- | --- | --- |

tr|Q90WC7|Q90WC7\_ONCMY

back to list

  

| Protein Coverage
| Supporting Peptides
|

Protein Coverage:

Supporting Peptides:

| Peptide | Uniq | -10lgP | Mass | ppm | m/z | z | RT | Scan | #Spec | Start | End | PTM |
| --- | --- | --- | --- | --- | --- | --- | --- | --- | --- | --- | --- | --- |
| R.VFDKEGNGTVM.G | Y | 37.84 | 1195.5543 | -0.9 | 598.7839 | 2 | 11.12 | 1522 | 1 | 137 | 147 |  |
| total 1 peptides |  |  |  |  |  |  |  |  |  |  |  |  |
| --- | --- | --- | --- | --- | --- | --- | --- | --- | --- | --- | --- | --- |

tr|A0A7J6CQG1|A0A7J6CQG1\_9TELE

back to list

  

| Protein Coverage
| Supporting Peptides
|

Protein Coverage:

Supporting Peptides:

| Peptide | Uniq | -10lgP | Mass | ppm | m/z | z | RT | Scan | #Spec | Start | End | PTM |
| --- | --- | --- | --- | --- | --- | --- | --- | --- | --- | --- | --- | --- |
| R.VFDKEGNGTVM.G | Y | 37.84 | 1195.5543 | -0.9 | 598.7839 | 2 | 11.12 | 1522 | 1 | 137 | 147 |  |
| total 1 peptides |  |  |  |  |  |  |  |  |  |  |  |  |
| --- | --- | --- | --- | --- | --- | --- | --- | --- | --- | --- | --- | --- |

tr|Q90W52|Q90W52\_PSEDN

back to list

  

| Protein Coverage
| Supporting Peptides
|

Protein Coverage:

Supporting Peptides:

| Peptide | Uniq | -10lgP | Mass | ppm | m/z | z | RT | Scan | #Spec | Start | End | PTM |
| --- | --- | --- | --- | --- | --- | --- | --- | --- | --- | --- | --- | --- |
| R.VFDKEGNGTVM.G | Y | 37.84 | 1195.5543 | -0.9 | 598.7839 | 2 | 11.12 | 1522 | 1 | 138 | 148 |  |
| total 1 peptides |  |  |  |  |  |  |  |  |  |  |  |  |
| --- | --- | --- | --- | --- | --- | --- | --- | --- | --- | --- | --- | --- |

tr|Q90W46|Q90W46\_DECMR

back to list

  

| Protein Coverage
| Supporting Peptides
|

Protein Coverage:

Supporting Peptides:

| Peptide | Uniq | -10lgP | Mass | ppm | m/z | z | RT | Scan | #Spec | Start | End | PTM |
| --- | --- | --- | --- | --- | --- | --- | --- | --- | --- | --- | --- | --- |
| R.VFDKEGNGTVM.G | Y | 37.84 | 1195.5543 | -0.9 | 598.7839 | 2 | 11.12 | 1522 | 1 | 141 | 151 |  |
| total 1 peptides |  |  |  |  |  |  |  |  |  |  |  |  |
| --- | --- | --- | --- | --- | --- | --- | --- | --- | --- | --- | --- | --- |

tr|A0A4W5MQC0|A0A4W5MQC0\_9TELE

back to list

  

| Protein Coverage
| Supporting Peptides
|

Protein Coverage:

Supporting Peptides:

| Peptide | Uniq | -10lgP | Mass | ppm | m/z | z | RT | Scan | #Spec | Start | End | PTM |
| --- | --- | --- | --- | --- | --- | --- | --- | --- | --- | --- | --- | --- |
| R.VFDKEGNGTVM.G | Y | 37.84 | 1195.5543 | -0.9 | 598.7839 | 2 | 11.12 | 1522 | 1 | 131 | 141 |  |
| total 1 peptides |  |  |  |  |  |  |  |  |  |  |  |  |
| --- | --- | --- | --- | --- | --- | --- | --- | --- | --- | --- | --- | --- |

tr|A0A0P7TY12|A0A0P7TY12\_SCLFO

back to list

  

| Protein Coverage
| Supporting Peptides
|

Protein Coverage:

Supporting Peptides:

| Peptide | Uniq | -10lgP | Mass | ppm | m/z | z | RT | Scan | #Spec | Start | End | PTM |
| --- | --- | --- | --- | --- | --- | --- | --- | --- | --- | --- | --- | --- |
| R.VFDKEGNGTVM.G | Y | 37.84 | 1195.5543 | -0.9 | 598.7839 | 2 | 11.12 | 1522 | 1 | 93 | 103 |  |
| total 1 peptides |  |  |  |  |  |  |  |  |  |  |  |  |
| --- | --- | --- | --- | --- | --- | --- | --- | --- | --- | --- | --- | --- |

tr|A0A672GTD6|A0A672GTD6\_SALFA

back to list

  

| Protein Coverage
| Supporting Peptides
|

Protein Coverage:

Supporting Peptides:

| Peptide | Uniq | -10lgP | Mass | ppm | m/z | z | RT | Scan | #Spec | Start | End | PTM |
| --- | --- | --- | --- | --- | --- | --- | --- | --- | --- | --- | --- | --- |
| R.VFDKEGNGTVM.G | Y | 37.84 | 1195.5543 | -0.9 | 598.7839 | 2 | 11.12 | 1522 | 1 | 93 | 103 |  |
| total 1 peptides |  |  |  |  |  |  |  |  |  |  |  |  |
| --- | --- | --- | --- | --- | --- | --- | --- | --- | --- | --- | --- | --- |

tr|A0A3P8V7S6|A0A3P8V7S6\_CYNSE

back to list

  

| Protein Coverage
| Supporting Peptides
|

Protein Coverage:

Supporting Peptides:

| Peptide | Uniq | -10lgP | Mass | ppm | m/z | z | RT | Scan | #Spec | Start | End | PTM |
| --- | --- | --- | --- | --- | --- | --- | --- | --- | --- | --- | --- | --- |
| R.VFDKEGNGTVM.G | Y | 37.84 | 1195.5543 | -0.9 | 598.7839 | 2 | 11.12 | 1522 | 1 | 94 | 104 |  |
| total 1 peptides |  |  |  |  |  |  |  |  |  |  |  |  |
| --- | --- | --- | --- | --- | --- | --- | --- | --- | --- | --- | --- | --- |

tr|A0A671WWZ3|A0A671WWZ3\_SPAAU

back to list

  

| Protein Coverage
| Supporting Peptides
|

Protein Coverage:

Supporting Peptides:

| Peptide | Uniq | -10lgP | Mass | ppm | m/z | z | RT | Scan | #Spec | Start | End | PTM |
| --- | --- | --- | --- | --- | --- | --- | --- | --- | --- | --- | --- | --- |
| R.VFDKEGNGTVM.G | Y | 37.84 | 1195.5543 | -0.9 | 598.7839 | 2 | 11.12 | 1522 | 1 | 97 | 107 |  |
| total 1 peptides |  |  |  |  |  |  |  |  |  |  |  |  |
| --- | --- | --- | --- | --- | --- | --- | --- | --- | --- | --- | --- | --- |

tr|A0A672GWW2|A0A672GWW2\_SALFA

back to list

  

| Protein Coverage
| Supporting Peptides
|

Protein Coverage:

Supporting Peptides:

| Peptide | Uniq | -10lgP | Mass | ppm | m/z | z | RT | Scan | #Spec | Start | End | PTM |
| --- | --- | --- | --- | --- | --- | --- | --- | --- | --- | --- | --- | --- |
| R.VFDKEGNGTVM.G | Y | 37.84 | 1195.5543 | -0.9 | 598.7839 | 2 | 11.12 | 1522 | 1 | 99 | 109 |  |
| total 1 peptides |  |  |  |  |  |  |  |  |  |  |  |  |
| --- | --- | --- | --- | --- | --- | --- | --- | --- | --- | --- | --- | --- |

tr|A0A671X3G3|A0A671X3G3\_SPAAU

back to list

  

| Protein Coverage
| Supporting Peptides
|

Protein Coverage:

Supporting Peptides:

| Peptide | Uniq | -10lgP | Mass | ppm | m/z | z | RT | Scan | #Spec | Start | End | PTM |
| --- | --- | --- | --- | --- | --- | --- | --- | --- | --- | --- | --- | --- |
| R.VFDKEGNGTVM.G | Y | 37.84 | 1195.5543 | -0.9 | 598.7839 | 2 | 11.12 | 1522 | 1 | 102 | 112 |  |
| total 1 peptides |  |  |  |  |  |  |  |  |  |  |  |  |
| --- | --- | --- | --- | --- | --- | --- | --- | --- | --- | --- | --- | --- |

tr|A0A672H327|A0A672H327\_SALFA

back to list

  

| Protein Coverage
| Supporting Peptides
|

Protein Coverage:

Supporting Peptides:

| Peptide | Uniq | -10lgP | Mass | ppm | m/z | z | RT | Scan | #Spec | Start | End | PTM |
| --- | --- | --- | --- | --- | --- | --- | --- | --- | --- | --- | --- | --- |
| R.VFDKEGNGTVM.G | Y | 37.84 | 1195.5543 | -0.9 | 598.7839 | 2 | 11.12 | 1522 | 1 | 102 | 112 |  |
| total 1 peptides |  |  |  |  |  |  |  |  |  |  |  |  |
| --- | --- | --- | --- | --- | --- | --- | --- | --- | --- | --- | --- | --- |

tr|A0A7N8Y8C7|A0A7N8Y8C7\_9TELE

back to list

  

| Protein Coverage
| Supporting Peptides
|

Protein Coverage:

Supporting Peptides:

| Peptide | Uniq | -10lgP | Mass | ppm | m/z | z | RT | Scan | #Spec | Start | End | PTM |
| --- | --- | --- | --- | --- | --- | --- | --- | --- | --- | --- | --- | --- |
| R.VFDKEGNGTVM.G | Y | 37.84 | 1195.5543 | -0.9 | 598.7839 | 2 | 11.12 | 1522 | 1 | 95 | 105 |  |
| total 1 peptides |  |  |  |  |  |  |  |  |  |  |  |  |
| --- | --- | --- | --- | --- | --- | --- | --- | --- | --- | --- | --- | --- |

tr|A0A672GRY1|A0A672GRY1\_SALFA

back to list

  

| Protein Coverage
| Supporting Peptides
|

Protein Coverage:

Supporting Peptides:

| Peptide | Uniq | -10lgP | Mass | ppm | m/z | z | RT | Scan | #Spec | Start | End | PTM |
| --- | --- | --- | --- | --- | --- | --- | --- | --- | --- | --- | --- | --- |
| R.VFDKEGNGTVM.G | Y | 37.84 | 1195.5543 | -0.9 | 598.7839 | 2 | 11.12 | 1522 | 1 | 109 | 119 |  |
| total 1 peptides |  |  |  |  |  |  |  |  |  |  |  |  |
| --- | --- | --- | --- | --- | --- | --- | --- | --- | --- | --- | --- | --- |

tr|A0A3Q2XPR8|A0A3Q2XPR8\_HIPCM

back to list

  

| Protein Coverage
| Supporting Peptides
|

Protein Coverage:

Supporting Peptides:

| Peptide | Uniq | -10lgP | Mass | ppm | m/z | z | RT | Scan | #Spec | Start | End | PTM |
| --- | --- | --- | --- | --- | --- | --- | --- | --- | --- | --- | --- | --- |
| R.VFDKEGNGTVM.G | Y | 37.84 | 1195.5543 | -0.9 | 598.7839 | 2 | 11.12 | 1522 | 1 | 126 | 136 |  |
| total 1 peptides |  |  |  |  |  |  |  |  |  |  |  |  |
| --- | --- | --- | --- | --- | --- | --- | --- | --- | --- | --- | --- | --- |

tr|A0A6J2QQE0|A0A6J2QQE0\_COTGO

back to list

  

| Protein Coverage
| Supporting Peptides
|

Protein Coverage:

Supporting Peptides:

| Peptide | Uniq | -10lgP | Mass | ppm | m/z | z | RT | Scan | #Spec | Start | End | PTM |
| --- | --- | --- | --- | --- | --- | --- | --- | --- | --- | --- | --- | --- |
| R.VFDKEGNGTVM.G | Y | 37.84 | 1195.5543 | -0.9 | 598.7839 | 2 | 11.12 | 1522 | 1 | 126 | 136 |  |
| total 1 peptides |  |  |  |  |  |  |  |  |  |  |  |  |
| --- | --- | --- | --- | --- | --- | --- | --- | --- | --- | --- | --- | --- |

tr|A0A3Q3D9S9|A0A3Q3D9S9\_HIPCM

back to list

  

| Protein Coverage
| Supporting Peptides
|

Protein Coverage:

Supporting Peptides:

| Peptide | Uniq | -10lgP | Mass | ppm | m/z | z | RT | Scan | #Spec | Start | End | PTM |
| --- | --- | --- | --- | --- | --- | --- | --- | --- | --- | --- | --- | --- |
| R.VFDKEGNGTVM.G | Y | 37.84 | 1195.5543 | -0.9 | 598.7839 | 2 | 11.12 | 1522 | 1 | 139 | 149 |  |
| total 1 peptides |  |  |  |  |  |  |  |  |  |  |  |  |
| --- | --- | --- | --- | --- | --- | --- | --- | --- | --- | --- | --- | --- |

tr|A0A4Z2CI05|A0A4Z2CI05\_9TELE

back to list

  

| Protein Coverage
| Supporting Peptides
|

Protein Coverage:

Supporting Peptides:

| Peptide | Uniq | -10lgP | Mass | ppm | m/z | z | RT | Scan | #Spec | Start | End | PTM |
| --- | --- | --- | --- | --- | --- | --- | --- | --- | --- | --- | --- | --- |
| L.IVEELPVKW.I | N | 27.75 | 1111.6277 | -1.0 | 556.8206 | 2 | 13.27 | 1897 | 1 | 770 | 778 |  |
| K.EGEEEK.K | N | 16.22 | 719.2973 | 0.9 | 360.6562 | 2 | 7.44 | 989 | 1 | 4321 | 4326 |  |
| total 2 peptides |  |  |  |  |  |  |  |  |  |  |  |  |
| --- | --- | --- | --- | --- | --- | --- | --- | --- | --- | --- | --- | --- |

tr|Q4RE90|Q4RE90\_TETNG

back to list

  

| Protein Coverage
| Supporting Peptides
|

Protein Coverage:

Supporting Peptides:

| Peptide | Uniq | -10lgP | Mass | ppm | m/z | z | RT | Scan | #Spec | Start | End | PTM |
| --- | --- | --- | --- | --- | --- | --- | --- | --- | --- | --- | --- | --- |
| T.VVDRPQPPEGPVVF.N | Y | 27.46 | 1534.8143 | -1.0 | 768.4137 | 2 | 12.56 | 1774 | 1 | 4491 | 4504 |  |
| K.EGEEEK.K | N | 16.22 | 719.2973 | 0.9 | 360.6562 | 2 | 7.44 | 989 | 1 | 2532 | 2537 |  |
| total 2 peptides |  |  |  |  |  |  |  |  |  |  |  |  |
| --- | --- | --- | --- | --- | --- | --- | --- | --- | --- | --- | --- | --- |

tr|A0A6F9CEX9|A0A6F9CEX9\_9TELE

back to list

  

| Protein Coverage
| Supporting Peptides
|

Protein Coverage:

Supporting Peptides:

| Peptide | Uniq | -10lgP | Mass | ppm | m/z | z | RT | Scan | #Spec | Start | End | PTM |
| --- | --- | --- | --- | --- | --- | --- | --- | --- | --- | --- | --- | --- |
| L.IVEELPVKW.V | N | 27.75 | 1111.6277 | -1.0 | 556.8206 | 2 | 13.27 | 1897 | 1 | 577 | 585 |  |
| D.VFEPPGSDIVFE.V | Y | 15.16 | 1334.6394 | -0.2 | 668.3268 | 2 | 13.99 | 2019 | 1 | 2259 | 2270 |  |
| total 2 peptides |  |  |  |  |  |  |  |  |  |  |  |  |
| --- | --- | --- | --- | --- | --- | --- | --- | --- | --- | --- | --- | --- |

tr|A0A060W3X5|A0A060W3X5\_ONCMY

back to list

  

| Protein Coverage
| Supporting Peptides
|

Protein Coverage:

Supporting Peptides:

| Peptide | Uniq | -10lgP | Mass | ppm | m/z | z | RT | Scan | #Spec | Start | End | PTM |
| --- | --- | --- | --- | --- | --- | --- | --- | --- | --- | --- | --- | --- |
| L.IVEELPVKW.V | N | 27.75 | 1111.6277 | -1.0 | 556.8206 | 2 | 13.27 | 1897 | 1 | 1746 | 1754 |  |
| D.VFEPPGSDIVFE.V | Y | 15.16 | 1334.6394 | -0.2 | 668.3268 | 2 | 13.99 | 2019 | 1 | 3457 | 3468 |  |
| total 2 peptides |  |  |  |  |  |  |  |  |  |  |  |  |
| --- | --- | --- | --- | --- | --- | --- | --- | --- | --- | --- | --- | --- |

tr|A0A6P3W3B1|A0A6P3W3B1\_CLUHA

back to list

  

| Protein Coverage
| Supporting Peptides
|

Protein Coverage:

Supporting Peptides:

| Peptide | Uniq | -10lgP | Mass | ppm | m/z | z | RT | Scan | #Spec | Start | End | PTM |
| --- | --- | --- | --- | --- | --- | --- | --- | --- | --- | --- | --- | --- |
| A.GYPDKIIIGM.D | Y | 23.66 | 1105.5841 | -1.5 | 553.7985 | 2 | 12.99 | 1847 | 1 | 235 | 244 |  |
| A.GYPDKIIIGMD.V | Y | 15.88 | 1220.6111 | -0.6 | 611.3124 | 2 | 12.77 | 1810 | 1 | 235 | 245 |  |
| total 2 peptides |  |  |  |  |  |  |  |  |  |  |  |  |
| --- | --- | --- | --- | --- | --- | --- | --- | --- | --- | --- | --- | --- |

tr|F5BZS7|F5BZS7\_EPIBR

back to list

  

| Protein Coverage
| Supporting Peptides
|

Protein Coverage:

Supporting Peptides:

| Peptide | Uniq | -10lgP | Mass | ppm | m/z | z | RT | Scan | #Spec | Start | End | PTM |
| --- | --- | --- | --- | --- | --- | --- | --- | --- | --- | --- | --- | --- |
| A.GYPDKIIIGM.D | Y | 23.66 | 1105.5841 | -1.5 | 553.7985 | 2 | 12.99 | 1847 | 1 | 235 | 244 |  |
| A.GYPDKIIIGMD.V | Y | 15.88 | 1220.6111 | -0.6 | 611.3124 | 2 | 12.77 | 1810 | 1 | 235 | 245 |  |
| total 2 peptides |  |  |  |  |  |  |  |  |  |  |  |  |
| --- | --- | --- | --- | --- | --- | --- | --- | --- | --- | --- | --- | --- |

tr|A0A668V8T6|A0A668V8T6\_OREAU

back to list

  

| Protein Coverage
| Supporting Peptides
|

Protein Coverage:

Supporting Peptides:

| Peptide | Uniq | -10lgP | Mass | ppm | m/z | z | RT | Scan | #Spec | Start | End | PTM |
| --- | --- | --- | --- | --- | --- | --- | --- | --- | --- | --- | --- | --- |
| A.GYPDKIIIGM.D | Y | 23.66 | 1105.5841 | -1.5 | 553.7985 | 2 | 12.99 | 1847 | 1 | 235 | 244 |  |
| A.GYPDKIIIGMD.V | Y | 15.88 | 1220.6111 | -0.6 | 611.3124 | 2 | 12.77 | 1810 | 1 | 235 | 245 |  |
| total 2 peptides |  |  |  |  |  |  |  |  |  |  |  |  |
| --- | --- | --- | --- | --- | --- | --- | --- | --- | --- | --- | --- | --- |

tr|A0A4W4FGS3|A0A4W4FGS3\_ELEEL

back to list

  

| Protein Coverage
| Supporting Peptides
|

Protein Coverage:

Supporting Peptides:

| Peptide | Uniq | -10lgP | Mass | ppm | m/z | z | RT | Scan | #Spec | Start | End | PTM |
| --- | --- | --- | --- | --- | --- | --- | --- | --- | --- | --- | --- | --- |
| A.GYPDKIIIGM.D | Y | 23.66 | 1105.5841 | -1.5 | 553.7985 | 2 | 12.99 | 1847 | 1 | 233 | 242 |  |
| A.GYPDKIIIGMD.V | Y | 15.88 | 1220.6111 | -0.6 | 611.3124 | 2 | 12.77 | 1810 | 1 | 233 | 243 |  |
| total 2 peptides |  |  |  |  |  |  |  |  |  |  |  |  |
| --- | --- | --- | --- | --- | --- | --- | --- | --- | --- | --- | --- | --- |

tr|A0A3B4CPC9|A0A3B4CPC9\_PYGNA

back to list

  

| Protein Coverage
| Supporting Peptides
|

Protein Coverage:

Supporting Peptides:

| Peptide | Uniq | -10lgP | Mass | ppm | m/z | z | RT | Scan | #Spec | Start | End | PTM |
| --- | --- | --- | --- | --- | --- | --- | --- | --- | --- | --- | --- | --- |
| A.GYPDKIIIGM.D | Y | 23.66 | 1105.5841 | -1.5 | 553.7985 | 2 | 12.99 | 1847 | 1 | 235 | 244 |  |
| A.GYPDKIIIGMD.V | Y | 15.88 | 1220.6111 | -0.6 | 611.3124 | 2 | 12.77 | 1810 | 1 | 235 | 245 |  |
| total 2 peptides |  |  |  |  |  |  |  |  |  |  |  |  |
| --- | --- | --- | --- | --- | --- | --- | --- | --- | --- | --- | --- | --- |

tr|A0A3P8TVY9|A0A3P8TVY9\_AMPPE

back to list

  

| Protein Coverage
| Supporting Peptides
|

Protein Coverage:

Supporting Peptides:

| Peptide | Uniq | -10lgP | Mass | ppm | m/z | z | RT | Scan | #Spec | Start | End | PTM |
| --- | --- | --- | --- | --- | --- | --- | --- | --- | --- | --- | --- | --- |
| A.GYPDKIIIGM.D | Y | 23.66 | 1105.5841 | -1.5 | 553.7985 | 2 | 12.99 | 1847 | 1 | 235 | 244 |  |
| A.GYPDKIIIGMD.V | Y | 15.88 | 1220.6111 | -0.6 | 611.3124 | 2 | 12.77 | 1810 | 1 | 235 | 245 |  |
| total 2 peptides |  |  |  |  |  |  |  |  |  |  |  |  |
| --- | --- | --- | --- | --- | --- | --- | --- | --- | --- | --- | --- | --- |

tr|A0A3P8NTT0|A0A3P8NTT0\_ASTCA

back to list

  

| Protein Coverage
| Supporting Peptides
|

Protein Coverage:

Supporting Peptides:

| Peptide | Uniq | -10lgP | Mass | ppm | m/z | z | RT | Scan | #Spec | Start | End | PTM |
| --- | --- | --- | --- | --- | --- | --- | --- | --- | --- | --- | --- | --- |
| A.GYPDKIIIGM.D | Y | 23.66 | 1105.5841 | -1.5 | 553.7985 | 2 | 12.99 | 1847 | 1 | 246 | 255 |  |
| A.GYPDKIIIGMD.V | Y | 15.88 | 1220.6111 | -0.6 | 611.3124 | 2 | 12.77 | 1810 | 1 | 246 | 256 |  |
| total 2 peptides |  |  |  |  |  |  |  |  |  |  |  |  |
| --- | --- | --- | --- | --- | --- | --- | --- | --- | --- | --- | --- | --- |

tr|A0A7J6BP23|A0A7J6BP23\_9TELE

back to list

  

| Protein Coverage
| Supporting Peptides
|

Protein Coverage:

Supporting Peptides:

| Peptide | Uniq | -10lgP | Mass | ppm | m/z | z | RT | Scan | #Spec | Start | End | PTM |
| --- | --- | --- | --- | --- | --- | --- | --- | --- | --- | --- | --- | --- |
| E.VDEDGSGTIDFEEF.L | Y | 29.88 | 1558.6311 | 0.0 | 780.3228 | 2 | 13.98 | 2017 | 1 | 20 | 33 |  |
| total 1 peptides |  |  |  |  |  |  |  |  |  |  |  |  |
| --- | --- | --- | --- | --- | --- | --- | --- | --- | --- | --- | --- | --- |

tr|A0A4Z2FMJ8|A0A4Z2FMJ8\_9TELE

back to list

  

| Protein Coverage
| Supporting Peptides
|

Protein Coverage:

Supporting Peptides:

| Peptide | Uniq | -10lgP | Mass | ppm | m/z | z | RT | Scan | #Spec | Start | End | PTM |
| --- | --- | --- | --- | --- | --- | --- | --- | --- | --- | --- | --- | --- |
| E.VDEDGSGTIDFEEF.L | Y | 29.88 | 1558.6311 | 0.0 | 780.3228 | 2 | 13.98 | 2017 | 1 | 63 | 76 |  |
| total 1 peptides |  |  |  |  |  |  |  |  |  |  |  |  |
| --- | --- | --- | --- | --- | --- | --- | --- | --- | --- | --- | --- | --- |

tr|A0A1A8D9W7|A0A1A8D9W7\_9TELE

back to list

  

| Protein Coverage
| Supporting Peptides
|

Protein Coverage:

Supporting Peptides:

| Peptide | Uniq | -10lgP | Mass | ppm | m/z | z | RT | Scan | #Spec | Start | End | PTM |
| --- | --- | --- | --- | --- | --- | --- | --- | --- | --- | --- | --- | --- |
| E.VDEDGSGTIDFEEF.L | Y | 29.88 | 1558.6311 | 0.0 | 780.3228 | 2 | 13.98 | 2017 | 1 | 38 | 51 |  |
| total 1 peptides |  |  |  |  |  |  |  |  |  |  |  |  |
| --- | --- | --- | --- | --- | --- | --- | --- | --- | --- | --- | --- | --- |

tr|A0A6P3VH61|A0A6P3VH61\_CLUHA

back to list

  

| Protein Coverage
| Supporting Peptides
|

Protein Coverage:

Supporting Peptides:

| Peptide | Uniq | -10lgP | Mass | ppm | m/z | z | RT | Scan | #Spec | Start | End | PTM |
| --- | --- | --- | --- | --- | --- | --- | --- | --- | --- | --- | --- | --- |
| E.VDEDGSGTIDFEEF.L | Y | 29.88 | 1558.6311 | 0.0 | 780.3228 | 2 | 13.98 | 2017 | 1 | 48 | 61 |  |
| total 1 peptides |  |  |  |  |  |  |  |  |  |  |  |  |
| --- | --- | --- | --- | --- | --- | --- | --- | --- | --- | --- | --- | --- |

tr|A0A3B4CI86|A0A3B4CI86\_PYGNA

back to list

  

| Protein Coverage
| Supporting Peptides
|

Protein Coverage:

Supporting Peptides:

| Peptide | Uniq | -10lgP | Mass | ppm | m/z | z | RT | Scan | #Spec | Start | End | PTM |
| --- | --- | --- | --- | --- | --- | --- | --- | --- | --- | --- | --- | --- |
| E.VDEDGSGTIDFEEF.L | Y | 29.88 | 1558.6311 | 0.0 | 780.3228 | 2 | 13.98 | 2017 | 1 | 48 | 61 |  |
| total 1 peptides |  |  |  |  |  |  |  |  |  |  |  |  |
| --- | --- | --- | --- | --- | --- | --- | --- | --- | --- | --- | --- | --- |

tr|A0A3B4E1V9|A0A3B4E1V9\_PYGNA

back to list

  

| Protein Coverage
| Supporting Peptides
|

Protein Coverage:

Supporting Peptides:

| Peptide | Uniq | -10lgP | Mass | ppm | m/z | z | RT | Scan | #Spec | Start | End | PTM |
| --- | --- | --- | --- | --- | --- | --- | --- | --- | --- | --- | --- | --- |
| E.VDEDGSGTIDFEEF.L | Y | 29.88 | 1558.6311 | 0.0 | 780.3228 | 2 | 13.98 | 2017 | 1 | 59 | 72 |  |
| total 1 peptides |  |  |  |  |  |  |  |  |  |  |  |  |
| --- | --- | --- | --- | --- | --- | --- | --- | --- | --- | --- | --- | --- |

tr|A0A6J2Q142|A0A6J2Q142\_COTGO

back to list

  

| Protein Coverage
| Supporting Peptides
|

Protein Coverage:

Supporting Peptides:

| Peptide | Uniq | -10lgP | Mass | ppm | m/z | z | RT | Scan | #Spec | Start | End | PTM |
| --- | --- | --- | --- | --- | --- | --- | --- | --- | --- | --- | --- | --- |
| E.VDEDGSGTIDFEEF.L | Y | 29.88 | 1558.6311 | 0.0 | 780.3228 | 2 | 13.98 | 2017 | 1 | 63 | 76 |  |
| total 1 peptides |  |  |  |  |  |  |  |  |  |  |  |  |
| --- | --- | --- | --- | --- | --- | --- | --- | --- | --- | --- | --- | --- |

tr|A0A3B4E4D7|A0A3B4E4D7\_PYGNA

back to list

  

| Protein Coverage
| Supporting Peptides
|

Protein Coverage:

Supporting Peptides:

| Peptide | Uniq | -10lgP | Mass | ppm | m/z | z | RT | Scan | #Spec | Start | End | PTM |
| --- | --- | --- | --- | --- | --- | --- | --- | --- | --- | --- | --- | --- |
| E.VDEDGSGTIDFEEF.L | Y | 29.88 | 1558.6311 | 0.0 | 780.3228 | 2 | 13.98 | 2017 | 1 | 63 | 76 |  |
| total 1 peptides |  |  |  |  |  |  |  |  |  |  |  |  |
| --- | --- | --- | --- | --- | --- | --- | --- | --- | --- | --- | --- | --- |

tr|H2SBN2|H2SBN2\_TAKRU

back to list

  

| Protein Coverage
| Supporting Peptides
|

Protein Coverage:

Supporting Peptides:

| Peptide | Uniq | -10lgP | Mass | ppm | m/z | z | RT | Scan | #Spec | Start | End | PTM |
| --- | --- | --- | --- | --- | --- | --- | --- | --- | --- | --- | --- | --- |
| E.VDEDGSGTIDFEEF.L | Y | 29.88 | 1558.6311 | 0.0 | 780.3228 | 2 | 13.98 | 2017 | 1 | 63 | 76 |  |
| total 1 peptides |  |  |  |  |  |  |  |  |  |  |  |  |
| --- | --- | --- | --- | --- | --- | --- | --- | --- | --- | --- | --- | --- |

tr|A0A2R8QMW7|A0A2R8QMW7\_DANRE

back to list

  

| Protein Coverage
| Supporting Peptides
|

Protein Coverage:

Supporting Peptides:

| Peptide | Uniq | -10lgP | Mass | ppm | m/z | z | RT | Scan | #Spec | Start | End | PTM |
| --- | --- | --- | --- | --- | --- | --- | --- | --- | --- | --- | --- | --- |
| E.VDEDGSGTIDFEEF.L | Y | 29.88 | 1558.6311 | 0.0 | 780.3228 | 2 | 13.98 | 2017 | 1 | 63 | 76 |  |
| total 1 peptides |  |  |  |  |  |  |  |  |  |  |  |  |
| --- | --- | --- | --- | --- | --- | --- | --- | --- | --- | --- | --- | --- |

tr|A0A4W4EUJ0|A0A4W4EUJ0\_ELEEL

back to list

  

| Protein Coverage
| Supporting Peptides
|

Protein Coverage:

Supporting Peptides:

| Peptide | Uniq | -10lgP | Mass | ppm | m/z | z | RT | Scan | #Spec | Start | End | PTM |
| --- | --- | --- | --- | --- | --- | --- | --- | --- | --- | --- | --- | --- |
| E.VDEDGSGTIDFEEF.L | Y | 29.88 | 1558.6311 | 0.0 | 780.3228 | 2 | 13.98 | 2017 | 1 | 63 | 76 |  |
| total 1 peptides |  |  |  |  |  |  |  |  |  |  |  |  |
| --- | --- | --- | --- | --- | --- | --- | --- | --- | --- | --- | --- | --- |

tr|A0A3Q3G540|A0A3Q3G540\_9LABR

back to list

  

| Protein Coverage
| Supporting Peptides
|

Protein Coverage:

Supporting Peptides:

| Peptide | Uniq | -10lgP | Mass | ppm | m/z | z | RT | Scan | #Spec | Start | End | PTM |
| --- | --- | --- | --- | --- | --- | --- | --- | --- | --- | --- | --- | --- |
| E.VDEDGSGTIDFEEF.L | Y | 29.88 | 1558.6311 | 0.0 | 780.3228 | 2 | 13.98 | 2017 | 1 | 63 | 76 |  |
| total 1 peptides |  |  |  |  |  |  |  |  |  |  |  |  |
| --- | --- | --- | --- | --- | --- | --- | --- | --- | --- | --- | --- | --- |

tr|G3NJM6|G3NJM6\_GASAC

back to list

  

| Protein Coverage
| Supporting Peptides
|

Protein Coverage:

Supporting Peptides:

| Peptide | Uniq | -10lgP | Mass | ppm | m/z | z | RT | Scan | #Spec | Start | End | PTM |
| --- | --- | --- | --- | --- | --- | --- | --- | --- | --- | --- | --- | --- |
| E.VDEDGSGTIDFEEF.L | Y | 29.88 | 1558.6311 | 0.0 | 780.3228 | 2 | 13.98 | 2017 | 1 | 63 | 76 |  |
| total 1 peptides |  |  |  |  |  |  |  |  |  |  |  |  |
| --- | --- | --- | --- | --- | --- | --- | --- | --- | --- | --- | --- | --- |

tr|W5UMX1|W5UMX1\_ICTPU

back to list

  

| Protein Coverage
| Supporting Peptides
|

Protein Coverage:

Supporting Peptides:

| Peptide | Uniq | -10lgP | Mass | ppm | m/z | z | RT | Scan | #Spec | Start | End | PTM |
| --- | --- | --- | --- | --- | --- | --- | --- | --- | --- | --- | --- | --- |
| E.VDEDGSGTIDFEEF.L | Y | 29.88 | 1558.6311 | 0.0 | 780.3228 | 2 | 13.98 | 2017 | 1 | 63 | 76 |  |
| total 1 peptides |  |  |  |  |  |  |  |  |  |  |  |  |
| --- | --- | --- | --- | --- | --- | --- | --- | --- | --- | --- | --- | --- |

tr|A0A3P8P1R5|A0A3P8P1R5\_ASTCA

back to list

  

| Protein Coverage
| Supporting Peptides
|

Protein Coverage:

Supporting Peptides:

| Peptide | Uniq | -10lgP | Mass | ppm | m/z | z | RT | Scan | #Spec | Start | End | PTM |
| --- | --- | --- | --- | --- | --- | --- | --- | --- | --- | --- | --- | --- |
| E.VDEDGSGTIDFEEF.L | Y | 29.88 | 1558.6311 | 0.0 | 780.3228 | 2 | 13.98 | 2017 | 1 | 64 | 77 |  |
| total 1 peptides |  |  |  |  |  |  |  |  |  |  |  |  |
| --- | --- | --- | --- | --- | --- | --- | --- | --- | --- | --- | --- | --- |

tr|A0A4W4EW29|A0A4W4EW29\_ELEEL

back to list

  

| Protein Coverage
| Supporting Peptides
|

Protein Coverage:

Supporting Peptides:

| Peptide | Uniq | -10lgP | Mass | ppm | m/z | z | RT | Scan | #Spec | Start | End | PTM |
| --- | --- | --- | --- | --- | --- | --- | --- | --- | --- | --- | --- | --- |
| E.VDEDGSGTIDFEEF.L | Y | 29.88 | 1558.6311 | 0.0 | 780.3228 | 2 | 13.98 | 2017 | 1 | 64 | 77 |  |
| total 1 peptides |  |  |  |  |  |  |  |  |  |  |  |  |
| --- | --- | --- | --- | --- | --- | --- | --- | --- | --- | --- | --- | --- |

tr|A0A6P7IM54|A0A6P7IM54\_9TELE

back to list

  

| Protein Coverage
| Supporting Peptides
|

Protein Coverage:

Supporting Peptides:

| Peptide | Uniq | -10lgP | Mass | ppm | m/z | z | RT | Scan | #Spec | Start | End | PTM |
| --- | --- | --- | --- | --- | --- | --- | --- | --- | --- | --- | --- | --- |
| E.VDEDGSGTIDFEEF.L | Y | 29.88 | 1558.6311 | 0.0 | 780.3228 | 2 | 13.98 | 2017 | 1 | 64 | 77 |  |
| total 1 peptides |  |  |  |  |  |  |  |  |  |  |  |  |
| --- | --- | --- | --- | --- | --- | --- | --- | --- | --- | --- | --- | --- |

tr|A0A4W4EVB1|A0A4W4EVB1\_ELEEL

back to list

  

| Protein Coverage
| Supporting Peptides
|

Protein Coverage:

Supporting Peptides:

| Peptide | Uniq | -10lgP | Mass | ppm | m/z | z | RT | Scan | #Spec | Start | End | PTM |
| --- | --- | --- | --- | --- | --- | --- | --- | --- | --- | --- | --- | --- |
| E.VDEDGSGTIDFEEF.L | Y | 29.88 | 1558.6311 | 0.0 | 780.3228 | 2 | 13.98 | 2017 | 1 | 64 | 77 |  |
| total 1 peptides |  |  |  |  |  |  |  |  |  |  |  |  |
| --- | --- | --- | --- | --- | --- | --- | --- | --- | --- | --- | --- | --- |

tr|I3IZI3|I3IZI3\_ORENI

back to list

  

| Protein Coverage
| Supporting Peptides
|

Protein Coverage:

Supporting Peptides:

| Peptide | Uniq | -10lgP | Mass | ppm | m/z | z | RT | Scan | #Spec | Start | End | PTM |
| --- | --- | --- | --- | --- | --- | --- | --- | --- | --- | --- | --- | --- |
| E.VDEDGSGTIDFEEF.L | Y | 29.88 | 1558.6311 | 0.0 | 780.3228 | 2 | 13.98 | 2017 | 1 | 64 | 77 |  |
| total 1 peptides |  |  |  |  |  |  |  |  |  |  |  |  |
| --- | --- | --- | --- | --- | --- | --- | --- | --- | --- | --- | --- | --- |

tr|A0A3Q1CAA4|A0A3Q1CAA4\_AMPOC

back to list

  

| Protein Coverage
| Supporting Peptides
|

Protein Coverage:

Supporting Peptides:

| Peptide | Uniq | -10lgP | Mass | ppm | m/z | z | RT | Scan | #Spec | Start | End | PTM |
| --- | --- | --- | --- | --- | --- | --- | --- | --- | --- | --- | --- | --- |
| E.VDEDGSGTIDFEEF.L | Y | 29.88 | 1558.6311 | 0.0 | 780.3228 | 2 | 13.98 | 2017 | 1 | 64 | 77 |  |
| total 1 peptides |  |  |  |  |  |  |  |  |  |  |  |  |
| --- | --- | --- | --- | --- | --- | --- | --- | --- | --- | --- | --- | --- |

tr|A0A3Q3G8Q9|A0A3Q3G8Q9\_9LABR

back to list

  

| Protein Coverage
| Supporting Peptides
|

Protein Coverage:

Supporting Peptides:

| Peptide | Uniq | -10lgP | Mass | ppm | m/z | z | RT | Scan | #Spec | Start | End | PTM |
| --- | --- | --- | --- | --- | --- | --- | --- | --- | --- | --- | --- | --- |
| E.VDEDGSGTIDFEEF.L | Y | 29.88 | 1558.6311 | 0.0 | 780.3228 | 2 | 13.98 | 2017 | 1 | 64 | 77 |  |
| total 1 peptides |  |  |  |  |  |  |  |  |  |  |  |  |
| --- | --- | --- | --- | --- | --- | --- | --- | --- | --- | --- | --- | --- |

tr|A0A4W5NYP8|A0A4W5NYP8\_9TELE

back to list

  

| Protein Coverage
| Supporting Peptides
|

Protein Coverage:

Supporting Peptides:

| Peptide | Uniq | -10lgP | Mass | ppm | m/z | z | RT | Scan | #Spec | Start | End | PTM |
| --- | --- | --- | --- | --- | --- | --- | --- | --- | --- | --- | --- | --- |
| E.VDEDGSGTIDFEEF.L | Y | 29.88 | 1558.6311 | 0.0 | 780.3228 | 2 | 13.98 | 2017 | 1 | 64 | 77 |  |
| total 1 peptides |  |  |  |  |  |  |  |  |  |  |  |  |
| --- | --- | --- | --- | --- | --- | --- | --- | --- | --- | --- | --- | --- |

tr|A0A672J300|A0A672J300\_SALFA

back to list

  

| Protein Coverage
| Supporting Peptides
|

Protein Coverage:

Supporting Peptides:

| Peptide | Uniq | -10lgP | Mass | ppm | m/z | z | RT | Scan | #Spec | Start | End | PTM |
| --- | --- | --- | --- | --- | --- | --- | --- | --- | --- | --- | --- | --- |
| E.VDEDGSGTIDFEEF.L | Y | 29.88 | 1558.6311 | 0.0 | 780.3228 | 2 | 13.98 | 2017 | 1 | 64 | 77 |  |
| total 1 peptides |  |  |  |  |  |  |  |  |  |  |  |  |
| --- | --- | --- | --- | --- | --- | --- | --- | --- | --- | --- | --- | --- |

tr|A0A3Q2W3W0|A0A3Q2W3W0\_HAPBU

back to list

  

| Protein Coverage
| Supporting Peptides
|

Protein Coverage:

Supporting Peptides:

| Peptide | Uniq | -10lgP | Mass | ppm | m/z | z | RT | Scan | #Spec | Start | End | PTM |
| --- | --- | --- | --- | --- | --- | --- | --- | --- | --- | --- | --- | --- |
| E.VDEDGSGTIDFEEF.L | Y | 29.88 | 1558.6311 | 0.0 | 780.3228 | 2 | 13.98 | 2017 | 1 | 64 | 77 |  |
| total 1 peptides |  |  |  |  |  |  |  |  |  |  |  |  |
| --- | --- | --- | --- | --- | --- | --- | --- | --- | --- | --- | --- | --- |

tr|A0A3Q2WC94|A0A3Q2WC94\_HAPBU

back to list

  

| Protein Coverage
| Supporting Peptides
|

Protein Coverage:

Supporting Peptides:

| Peptide | Uniq | -10lgP | Mass | ppm | m/z | z | RT | Scan | #Spec | Start | End | PTM |
| --- | --- | --- | --- | --- | --- | --- | --- | --- | --- | --- | --- | --- |
| E.VDEDGSGTIDFEEF.L | Y | 29.88 | 1558.6311 | 0.0 | 780.3228 | 2 | 13.98 | 2017 | 1 | 63 | 76 |  |
| total 1 peptides |  |  |  |  |  |  |  |  |  |  |  |  |
| --- | --- | --- | --- | --- | --- | --- | --- | --- | --- | --- | --- | --- |

tr|A0A669E7L6|A0A669E7L6\_ORENI

back to list

  

| Protein Coverage
| Supporting Peptides
|

Protein Coverage:

Supporting Peptides:

| Peptide | Uniq | -10lgP | Mass | ppm | m/z | z | RT | Scan | #Spec | Start | End | PTM |
| --- | --- | --- | --- | --- | --- | --- | --- | --- | --- | --- | --- | --- |
| E.VDEDGSGTIDFEEF.L | Y | 29.88 | 1558.6311 | 0.0 | 780.3228 | 2 | 13.98 | 2017 | 1 | 64 | 77 |  |
| total 1 peptides |  |  |  |  |  |  |  |  |  |  |  |  |
| --- | --- | --- | --- | --- | --- | --- | --- | --- | --- | --- | --- | --- |

tr|A0A3B3TEJ6|A0A3B3TEJ6\_9TELE

back to list

  

| Protein Coverage
| Supporting Peptides
|

Protein Coverage:

Supporting Peptides:

| Peptide | Uniq | -10lgP | Mass | ppm | m/z | z | RT | Scan | #Spec | Start | End | PTM |
| --- | --- | --- | --- | --- | --- | --- | --- | --- | --- | --- | --- | --- |
| E.VDEDGSGTIDFEEF.L | Y | 29.88 | 1558.6311 | 0.0 | 780.3228 | 2 | 13.98 | 2017 | 1 | 64 | 77 |  |
| total 1 peptides |  |  |  |  |  |  |  |  |  |  |  |  |
| --- | --- | --- | --- | --- | --- | --- | --- | --- | --- | --- | --- | --- |

tr|A0A3P8P1N2|A0A3P8P1N2\_ASTCA

back to list

  

| Protein Coverage
| Supporting Peptides
|

Protein Coverage:

Supporting Peptides:

| Peptide | Uniq | -10lgP | Mass | ppm | m/z | z | RT | Scan | #Spec | Start | End | PTM |
| --- | --- | --- | --- | --- | --- | --- | --- | --- | --- | --- | --- | --- |
| E.VDEDGSGTIDFEEF.L | Y | 29.88 | 1558.6311 | 0.0 | 780.3228 | 2 | 13.98 | 2017 | 1 | 87 | 100 |  |
| total 1 peptides |  |  |  |  |  |  |  |  |  |  |  |  |
| --- | --- | --- | --- | --- | --- | --- | --- | --- | --- | --- | --- | --- |

tr|A0A3P9N7Z1|A0A3P9N7Z1\_POERE

back to list

  

| Protein Coverage
| Supporting Peptides
|

Protein Coverage:

Supporting Peptides:

| Peptide | Uniq | -10lgP | Mass | ppm | m/z | z | RT | Scan | #Spec | Start | End | PTM |
| --- | --- | --- | --- | --- | --- | --- | --- | --- | --- | --- | --- | --- |
| E.VDEDGSGTIDFEEF.L | Y | 29.88 | 1558.6311 | 0.0 | 780.3228 | 2 | 13.98 | 2017 | 1 | 64 | 77 |  |
| total 1 peptides |  |  |  |  |  |  |  |  |  |  |  |  |
| --- | --- | --- | --- | --- | --- | --- | --- | --- | --- | --- | --- | --- |

tr|A0A6F9C7M8|A0A6F9C7M8\_9TELE

back to list

  

| Protein Coverage
| Supporting Peptides
|

Protein Coverage:

Supporting Peptides:

| Peptide | Uniq | -10lgP | Mass | ppm | m/z | z | RT | Scan | #Spec | Start | End | PTM |
| --- | --- | --- | --- | --- | --- | --- | --- | --- | --- | --- | --- | --- |
| E.VDEDGSGTIDFEEF.L | Y | 29.88 | 1558.6311 | 0.0 | 780.3228 | 2 | 13.98 | 2017 | 1 | 274 | 287 |  |
| total 1 peptides |  |  |  |  |  |  |  |  |  |  |  |  |
| --- | --- | --- | --- | --- | --- | --- | --- | --- | --- | --- | --- | --- |

tr|A0A315VUM5|A0A315VUM5\_GAMAF

back to list

  

| Protein Coverage
| Supporting Peptides
|

Protein Coverage:

Supporting Peptides:

| Peptide | Uniq | -10lgP | Mass | ppm | m/z | z | RT | Scan | #Spec | Start | End | PTM |
| --- | --- | --- | --- | --- | --- | --- | --- | --- | --- | --- | --- | --- |
| A.LGNPIPHIVW.R | Y | 24.10 | 1144.6393 | -43.6 | 573.3019 | 2 | 15.20 | 2226 | 1 | 919 | 928 |  |
| total 1 peptides |  |  |  |  |  |  |  |  |  |  |  |  |
| --- | --- | --- | --- | --- | --- | --- | --- | --- | --- | --- | --- | --- |

tr|A0A3Q0S4A7|A0A3Q0S4A7\_AMPCI

back to list

  

| Protein Coverage
| Supporting Peptides
|

Protein Coverage:

Supporting Peptides:

| Peptide | Uniq | -10lgP | Mass | ppm | m/z | z | RT | Scan | #Spec | Start | End | PTM |
| --- | --- | --- | --- | --- | --- | --- | --- | --- | --- | --- | --- | --- |
| Q.DLATICSVLLEN.G | Y | 23.39 | 1289.6537 | -2.6 | 645.8325 | 2 | 15.41 | 2264 | 1 | 738 | 749 |  |
| total 1 peptides |  |  |  |  |  |  |  |  |  |  |  |  |
| --- | --- | --- | --- | --- | --- | --- | --- | --- | --- | --- | --- | --- |

tr|A0A3B4V2S1|A0A3B4V2S1\_SERDU

back to list

  

| Protein Coverage
| Supporting Peptides
|

Protein Coverage:

Supporting Peptides:

| Peptide | Uniq | -10lgP | Mass | ppm | m/z | z | RT | Scan | #Spec | Start | End | PTM |
| --- | --- | --- | --- | --- | --- | --- | --- | --- | --- | --- | --- | --- |
| Q.DLATICSVLLEN.G | Y | 23.39 | 1289.6537 | -2.6 | 645.8325 | 2 | 15.41 | 2264 | 1 | 741 | 752 |  |
| total 1 peptides |  |  |  |  |  |  |  |  |  |  |  |  |
| --- | --- | --- | --- | --- | --- | --- | --- | --- | --- | --- | --- | --- |

tr|A0A4W4FZE0|A0A4W4FZE0\_ELEEL

back to list

  

| Protein Coverage
| Supporting Peptides
|

Protein Coverage:

Supporting Peptides:

| Peptide | Uniq | -10lgP | Mass | ppm | m/z | z | RT | Scan | #Spec | Start | End | PTM |
| --- | --- | --- | --- | --- | --- | --- | --- | --- | --- | --- | --- | --- |
| Q.DLATICSVLLEN.G | Y | 23.39 | 1289.6537 | -2.6 | 645.8325 | 2 | 15.41 | 2264 | 1 | 830 | 841 |  |
| total 1 peptides |  |  |  |  |  |  |  |  |  |  |  |  |
| --- | --- | --- | --- | --- | --- | --- | --- | --- | --- | --- | --- | --- |

tr|A0A1A8RP46|A0A1A8RP46\_9TELE

back to list

  

| Protein Coverage
| Supporting Peptides
|

Protein Coverage:

Supporting Peptides:

| Peptide | Uniq | -10lgP | Mass | ppm | m/z | z | RT | Scan | #Spec | Start | End | PTM |
| --- | --- | --- | --- | --- | --- | --- | --- | --- | --- | --- | --- | --- |
| Q.DLATICSVLLEN.G | Y | 23.39 | 1289.6537 | -2.6 | 645.8325 | 2 | 15.41 | 2264 | 1 | 947 | 958 |  |
| total 1 peptides |  |  |  |  |  |  |  |  |  |  |  |  |
| --- | --- | --- | --- | --- | --- | --- | --- | --- | --- | --- | --- | --- |

tr|A0A6Q2ZPR8|A0A6Q2ZPR8\_ESOLU

back to list

  

| Protein Coverage
| Supporting Peptides
|

Protein Coverage:

Supporting Peptides:

| Peptide | Uniq | -10lgP | Mass | ppm | m/z | z | RT | Scan | #Spec | Start | End | PTM |
| --- | --- | --- | --- | --- | --- | --- | --- | --- | --- | --- | --- | --- |
| Q.DLATICSVLLEN.G | Y | 23.39 | 1289.6537 | -2.6 | 645.8325 | 2 | 15.41 | 2264 | 1 | 897 | 908 |  |
| total 1 peptides |  |  |  |  |  |  |  |  |  |  |  |  |
| --- | --- | --- | --- | --- | --- | --- | --- | --- | --- | --- | --- | --- |

tr|A0A674AQG9|A0A674AQG9\_SALTR

back to list

  

| Protein Coverage
| Supporting Peptides
|

Protein Coverage:

Supporting Peptides:

| Peptide | Uniq | -10lgP | Mass | ppm | m/z | z | RT | Scan | #Spec | Start | End | PTM |
| --- | --- | --- | --- | --- | --- | --- | --- | --- | --- | --- | --- | --- |
| Q.DLATICSVLLEN.G | Y | 23.39 | 1289.6537 | -2.6 | 645.8325 | 2 | 15.41 | 2264 | 1 | 894 | 905 |  |
| total 1 peptides |  |  |  |  |  |  |  |  |  |  |  |  |
| --- | --- | --- | --- | --- | --- | --- | --- | --- | --- | --- | --- | --- |

tr|A0A3P8T5S7|A0A3P8T5S7\_AMPPE

back to list

  

| Protein Coverage
| Supporting Peptides
|

Protein Coverage:

Supporting Peptides:

| Peptide | Uniq | -10lgP | Mass | ppm | m/z | z | RT | Scan | #Spec | Start | End | PTM |
| --- | --- | --- | --- | --- | --- | --- | --- | --- | --- | --- | --- | --- |
| Q.DLATICSVLLEN.G | Y | 23.39 | 1289.6537 | -2.6 | 645.8325 | 2 | 15.41 | 2264 | 1 | 925 | 936 |  |
| total 1 peptides |  |  |  |  |  |  |  |  |  |  |  |  |
| --- | --- | --- | --- | --- | --- | --- | --- | --- | --- | --- | --- | --- |

tr|A0A4W4FZ81|A0A4W4FZ81\_ELEEL

back to list

  

| Protein Coverage
| Supporting Peptides
|

Protein Coverage:

Supporting Peptides:

| Peptide | Uniq | -10lgP | Mass | ppm | m/z | z | RT | Scan | #Spec | Start | End | PTM |
| --- | --- | --- | --- | --- | --- | --- | --- | --- | --- | --- | --- | --- |
| Q.DLATICSVLLEN.G | Y | 23.39 | 1289.6537 | -2.6 | 645.8325 | 2 | 15.41 | 2264 | 1 | 929 | 940 |  |
| total 1 peptides |  |  |  |  |  |  |  |  |  |  |  |  |
| --- | --- | --- | --- | --- | --- | --- | --- | --- | --- | --- | --- | --- |

tr|A0A4W5QGX5|A0A4W5QGX5\_9TELE

back to list

  

| Protein Coverage
| Supporting Peptides
|

Protein Coverage:

Supporting Peptides:

| Peptide | Uniq | -10lgP | Mass | ppm | m/z | z | RT | Scan | #Spec | Start | End | PTM |
| --- | --- | --- | --- | --- | --- | --- | --- | --- | --- | --- | --- | --- |
| Q.DLATICSVLLEN.G | Y | 23.39 | 1289.6537 | -2.6 | 645.8325 | 2 | 15.41 | 2264 | 1 | 931 | 942 |  |
| total 1 peptides |  |  |  |  |  |  |  |  |  |  |  |  |
| --- | --- | --- | --- | --- | --- | --- | --- | --- | --- | --- | --- | --- |

tr|A0A3B3DDJ3|A0A3B3DDJ3\_ORYME

back to list

  

| Protein Coverage
| Supporting Peptides
|

Protein Coverage:

Supporting Peptides:

| Peptide | Uniq | -10lgP | Mass | ppm | m/z | z | RT | Scan | #Spec | Start | End | PTM |
| --- | --- | --- | --- | --- | --- | --- | --- | --- | --- | --- | --- | --- |
| Q.DLATICSVLLEN.G | Y | 23.39 | 1289.6537 | -2.6 | 645.8325 | 2 | 15.41 | 2264 | 1 | 937 | 948 |  |
| total 1 peptides |  |  |  |  |  |  |  |  |  |  |  |  |
| --- | --- | --- | --- | --- | --- | --- | --- | --- | --- | --- | --- | --- |

tr|A0A4W4FW94|A0A4W4FW94\_ELEEL

back to list

  

| Protein Coverage
| Supporting Peptides
|

Protein Coverage:

Supporting Peptides:

| Peptide | Uniq | -10lgP | Mass | ppm | m/z | z | RT | Scan | #Spec | Start | End | PTM |
| --- | --- | --- | --- | --- | --- | --- | --- | --- | --- | --- | --- | --- |
| Q.DLATICSVLLEN.G | Y | 23.39 | 1289.6537 | -2.6 | 645.8325 | 2 | 15.41 | 2264 | 1 | 935 | 946 |  |
| total 1 peptides |  |  |  |  |  |  |  |  |  |  |  |  |
| --- | --- | --- | --- | --- | --- | --- | --- | --- | --- | --- | --- | --- |

tr|A0A674AP52|A0A674AP52\_SALTR

back to list

  

| Protein Coverage
| Supporting Peptides
|

Protein Coverage:

Supporting Peptides:

| Peptide | Uniq | -10lgP | Mass | ppm | m/z | z | RT | Scan | #Spec | Start | End | PTM |
| --- | --- | --- | --- | --- | --- | --- | --- | --- | --- | --- | --- | --- |
| Q.DLATICSVLLEN.G | Y | 23.39 | 1289.6537 | -2.6 | 645.8325 | 2 | 15.41 | 2264 | 1 | 935 | 946 |  |
| total 1 peptides |  |  |  |  |  |  |  |  |  |  |  |  |
| --- | --- | --- | --- | --- | --- | --- | --- | --- | --- | --- | --- | --- |

tr|A0A834KWF7|A0A834KWF7\_ORYME

back to list

  

| Protein Coverage
| Supporting Peptides
|

Protein Coverage:

Supporting Peptides:

| Peptide | Uniq | -10lgP | Mass | ppm | m/z | z | RT | Scan | #Spec | Start | End | PTM |
| --- | --- | --- | --- | --- | --- | --- | --- | --- | --- | --- | --- | --- |
| Q.DLATICSVLLEN.G | Y | 23.39 | 1289.6537 | -2.6 | 645.8325 | 2 | 15.41 | 2264 | 1 | 925 | 936 |  |
| total 1 peptides |  |  |  |  |  |  |  |  |  |  |  |  |
| --- | --- | --- | --- | --- | --- | --- | --- | --- | --- | --- | --- | --- |

tr|A0A667ZHJ6|A0A667ZHJ6\_9TELE

back to list

  

| Protein Coverage
| Supporting Peptides
|

Protein Coverage:

Supporting Peptides:

| Peptide | Uniq | -10lgP | Mass | ppm | m/z | z | RT | Scan | #Spec | Start | End | PTM |
| --- | --- | --- | --- | --- | --- | --- | --- | --- | --- | --- | --- | --- |
| Q.DLATICSVLLEN.G | Y | 23.39 | 1289.6537 | -2.6 | 645.8325 | 2 | 15.41 | 2264 | 1 | 944 | 955 |  |
| total 1 peptides |  |  |  |  |  |  |  |  |  |  |  |  |
| --- | --- | --- | --- | --- | --- | --- | --- | --- | --- | --- | --- | --- |

tr|A0A3B4DWJ2|A0A3B4DWJ2\_PYGNA

back to list

  

| Protein Coverage
| Supporting Peptides
|

Protein Coverage:

Supporting Peptides:

| Peptide | Uniq | -10lgP | Mass | ppm | m/z | z | RT | Scan | #Spec | Start | End | PTM |
| --- | --- | --- | --- | --- | --- | --- | --- | --- | --- | --- | --- | --- |
| Q.DLATICSVLLEN.G | Y | 23.39 | 1289.6537 | -2.6 | 645.8325 | 2 | 15.41 | 2264 | 1 | 947 | 958 |  |
| total 1 peptides |  |  |  |  |  |  |  |  |  |  |  |  |
| --- | --- | --- | --- | --- | --- | --- | --- | --- | --- | --- | --- | --- |

tr|A0A3Q2XWS4|A0A3Q2XWS4\_HIPCM

back to list

  

| Protein Coverage
| Supporting Peptides
|

Protein Coverage:

Supporting Peptides:

| Peptide | Uniq | -10lgP | Mass | ppm | m/z | z | RT | Scan | #Spec | Start | End | PTM |
| --- | --- | --- | --- | --- | --- | --- | --- | --- | --- | --- | --- | --- |
| Q.DLATICSVLLEN.G | Y | 23.39 | 1289.6537 | -2.6 | 645.8325 | 2 | 15.41 | 2264 | 1 | 947 | 958 |  |
| total 1 peptides |  |  |  |  |  |  |  |  |  |  |  |  |
| --- | --- | --- | --- | --- | --- | --- | --- | --- | --- | --- | --- | --- |

tr|A0A667ZHH4|A0A667ZHH4\_9TELE

back to list

  

| Protein Coverage
| Supporting Peptides
|

Protein Coverage:

Supporting Peptides:

| Peptide | Uniq | -10lgP | Mass | ppm | m/z | z | RT | Scan | #Spec | Start | End | PTM |
| --- | --- | --- | --- | --- | --- | --- | --- | --- | --- | --- | --- | --- |
| Q.DLATICSVLLEN.G | Y | 23.39 | 1289.6537 | -2.6 | 645.8325 | 2 | 15.41 | 2264 | 1 | 947 | 958 |  |
| total 1 peptides |  |  |  |  |  |  |  |  |  |  |  |  |
| --- | --- | --- | --- | --- | --- | --- | --- | --- | --- | --- | --- | --- |

tr|A0A3Q3G3V8|A0A3Q3G3V8\_KRYMA

back to list

  

| Protein Coverage
| Supporting Peptides
|

Protein Coverage:

Supporting Peptides:

| Peptide | Uniq | -10lgP | Mass | ppm | m/z | z | RT | Scan | #Spec | Start | End | PTM |
| --- | --- | --- | --- | --- | --- | --- | --- | --- | --- | --- | --- | --- |
| Q.DLATICSVLLEN.G | Y | 23.39 | 1289.6537 | -2.6 | 645.8325 | 2 | 15.41 | 2264 | 1 | 947 | 958 |  |
| total 1 peptides |  |  |  |  |  |  |  |  |  |  |  |  |
| --- | --- | --- | --- | --- | --- | --- | --- | --- | --- | --- | --- | --- |

tr|A0A3P8R774|A0A3P8R774\_ASTCA

back to list

  

| Protein Coverage
| Supporting Peptides
|

Protein Coverage:

Supporting Peptides:

| Peptide | Uniq | -10lgP | Mass | ppm | m/z | z | RT | Scan | #Spec | Start | End | PTM |
| --- | --- | --- | --- | --- | --- | --- | --- | --- | --- | --- | --- | --- |
| Q.DLATICSVLLEN.G | Y | 23.39 | 1289.6537 | -2.6 | 645.8325 | 2 | 15.41 | 2264 | 1 | 947 | 958 |  |
| total 1 peptides |  |  |  |  |  |  |  |  |  |  |  |  |
| --- | --- | --- | --- | --- | --- | --- | --- | --- | --- | --- | --- | --- |

tr|A0A484CLN2|A0A484CLN2\_PERFV

back to list

  

| Protein Coverage
| Supporting Peptides
|

Protein Coverage:

Supporting Peptides:

| Peptide | Uniq | -10lgP | Mass | ppm | m/z | z | RT | Scan | #Spec | Start | End | PTM |
| --- | --- | --- | --- | --- | --- | --- | --- | --- | --- | --- | --- | --- |
| Q.DLATICSVLLEN.G | Y | 23.39 | 1289.6537 | -2.6 | 645.8325 | 2 | 15.41 | 2264 | 1 | 947 | 958 |  |
| total 1 peptides |  |  |  |  |  |  |  |  |  |  |  |  |
| --- | --- | --- | --- | --- | --- | --- | --- | --- | --- | --- | --- | --- |

tr|A0A4W4FZM1|A0A4W4FZM1\_ELEEL

back to list

  

| Protein Coverage
| Supporting Peptides
|

Protein Coverage:

Supporting Peptides:

| Peptide | Uniq | -10lgP | Mass | ppm | m/z | z | RT | Scan | #Spec | Start | End | PTM |
| --- | --- | --- | --- | --- | --- | --- | --- | --- | --- | --- | --- | --- |
| Q.DLATICSVLLEN.G | Y | 23.39 | 1289.6537 | -2.6 | 645.8325 | 2 | 15.41 | 2264 | 1 | 950 | 961 |  |
| total 1 peptides |  |  |  |  |  |  |  |  |  |  |  |  |
| --- | --- | --- | --- | --- | --- | --- | --- | --- | --- | --- | --- | --- |

tr|A0A4W5M1U7|A0A4W5M1U7\_9TELE

back to list

  

| Protein Coverage
| Supporting Peptides
|

Protein Coverage:

Supporting Peptides:

| Peptide | Uniq | -10lgP | Mass | ppm | m/z | z | RT | Scan | #Spec | Start | End | PTM |
| --- | --- | --- | --- | --- | --- | --- | --- | --- | --- | --- | --- | --- |
| Q.DLATICSVLLEN.G | Y | 23.39 | 1289.6537 | -2.6 | 645.8325 | 2 | 15.41 | 2264 | 1 | 952 | 963 |  |
| total 1 peptides |  |  |  |  |  |  |  |  |  |  |  |  |
| --- | --- | --- | --- | --- | --- | --- | --- | --- | --- | --- | --- | --- |

tr|A0A674ARJ6|A0A674ARJ6\_SALTR

back to list

  

| Protein Coverage
| Supporting Peptides
|

Protein Coverage:

Supporting Peptides:

| Peptide | Uniq | -10lgP | Mass | ppm | m/z | z | RT | Scan | #Spec | Start | End | PTM |
| --- | --- | --- | --- | --- | --- | --- | --- | --- | --- | --- | --- | --- |
| Q.DLATICSVLLEN.G | Y | 23.39 | 1289.6537 | -2.6 | 645.8325 | 2 | 15.41 | 2264 | 1 | 952 | 963 |  |
| total 1 peptides |  |  |  |  |  |  |  |  |  |  |  |  |
| --- | --- | --- | --- | --- | --- | --- | --- | --- | --- | --- | --- | --- |

tr|A0A4W4FZR2|A0A4W4FZR2\_ELEEL

back to list

  

| Protein Coverage
| Supporting Peptides
|

Protein Coverage:

Supporting Peptides:

| Peptide | Uniq | -10lgP | Mass | ppm | m/z | z | RT | Scan | #Spec | Start | End | PTM |
| --- | --- | --- | --- | --- | --- | --- | --- | --- | --- | --- | --- | --- |
| Q.DLATICSVLLEN.G | Y | 23.39 | 1289.6537 | -2.6 | 645.8325 | 2 | 15.41 | 2264 | 1 | 952 | 963 |  |
| total 1 peptides |  |  |  |  |  |  |  |  |  |  |  |  |
| --- | --- | --- | --- | --- | --- | --- | --- | --- | --- | --- | --- | --- |

tr|A0A4W5M1X7|A0A4W5M1X7\_9TELE

back to list

  

| Protein Coverage
| Supporting Peptides
|

Protein Coverage:

Supporting Peptides:

| Peptide | Uniq | -10lgP | Mass | ppm | m/z | z | RT | Scan | #Spec | Start | End | PTM |
| --- | --- | --- | --- | --- | --- | --- | --- | --- | --- | --- | --- | --- |
| Q.DLATICSVLLEN.G | Y | 23.39 | 1289.6537 | -2.6 | 645.8325 | 2 | 15.41 | 2264 | 1 | 953 | 964 |  |
| total 1 peptides |  |  |  |  |  |  |  |  |  |  |  |  |
| --- | --- | --- | --- | --- | --- | --- | --- | --- | --- | --- | --- | --- |

tr|A0A3Q3Q182|A0A3Q3Q182\_MONAL

back to list

  

| Protein Coverage
| Supporting Peptides
|

Protein Coverage:

Supporting Peptides:

| Peptide | Uniq | -10lgP | Mass | ppm | m/z | z | RT | Scan | #Spec | Start | End | PTM |
| --- | --- | --- | --- | --- | --- | --- | --- | --- | --- | --- | --- | --- |
| Q.DLATICSVLLEN.G | Y | 23.39 | 1289.6537 | -2.6 | 645.8325 | 2 | 15.41 | 2264 | 1 | 957 | 968 |  |
| total 1 peptides |  |  |  |  |  |  |  |  |  |  |  |  |
| --- | --- | --- | --- | --- | --- | --- | --- | --- | --- | --- | --- | --- |

tr|A0A4W5QMX9|A0A4W5QMX9\_9TELE

back to list

  

| Protein Coverage
| Supporting Peptides
|

Protein Coverage:

Supporting Peptides:

| Peptide | Uniq | -10lgP | Mass | ppm | m/z | z | RT | Scan | #Spec | Start | End | PTM |
| --- | --- | --- | --- | --- | --- | --- | --- | --- | --- | --- | --- | --- |
| Q.DLATICSVLLEN.G | Y | 23.39 | 1289.6537 | -2.6 | 645.8325 | 2 | 15.41 | 2264 | 1 | 954 | 965 |  |
| total 1 peptides |  |  |  |  |  |  |  |  |  |  |  |  |
| --- | --- | --- | --- | --- | --- | --- | --- | --- | --- | --- | --- | --- |

tr|A0A673Z592|A0A673Z592\_SALTR

back to list

  

| Protein Coverage
| Supporting Peptides
|

Protein Coverage:

Supporting Peptides:

| Peptide | Uniq | -10lgP | Mass | ppm | m/z | z | RT | Scan | #Spec | Start | End | PTM |
| --- | --- | --- | --- | --- | --- | --- | --- | --- | --- | --- | --- | --- |
| Q.DLATICSVLLEN.G | Y | 23.39 | 1289.6537 | -2.6 | 645.8325 | 2 | 15.41 | 2264 | 1 | 964 | 975 |  |
| total 1 peptides |  |  |  |  |  |  |  |  |  |  |  |  |
| --- | --- | --- | --- | --- | --- | --- | --- | --- | --- | --- | --- | --- |

tr|A0A5N5JWC6|A0A5N5JWC6\_PANHP

back to list

  

| Protein Coverage
| Supporting Peptides
|

Protein Coverage:

Supporting Peptides:

| Peptide | Uniq | -10lgP | Mass | ppm | m/z | z | RT | Scan | #Spec | Start | End | PTM |
| --- | --- | --- | --- | --- | --- | --- | --- | --- | --- | --- | --- | --- |
| Q.DLATICSVLLEN.G | Y | 23.39 | 1289.6537 | -2.6 | 645.8325 | 2 | 15.41 | 2264 | 1 | 984 | 995 |  |
| total 1 peptides |  |  |  |  |  |  |  |  |  |  |  |  |
| --- | --- | --- | --- | --- | --- | --- | --- | --- | --- | --- | --- | --- |

tr|A0A4W4FWH2|A0A4W4FWH2\_ELEEL

back to list

  

| Protein Coverage
| Supporting Peptides
|

Protein Coverage:

Supporting Peptides:

| Peptide | Uniq | -10lgP | Mass | ppm | m/z | z | RT | Scan | #Spec | Start | End | PTM |
| --- | --- | --- | --- | --- | --- | --- | --- | --- | --- | --- | --- | --- |
| Q.DLATICSVLLEN.G | Y | 23.39 | 1289.6537 | -2.6 | 645.8325 | 2 | 15.41 | 2264 | 1 | 988 | 999 |  |
| total 1 peptides |  |  |  |  |  |  |  |  |  |  |  |  |
| --- | --- | --- | --- | --- | --- | --- | --- | --- | --- | --- | --- | --- |

tr|A0A2D0QGH0|A0A2D0QGH0\_ICTPU

back to list

  

| Protein Coverage
| Supporting Peptides
|

Protein Coverage:

Supporting Peptides:

| Peptide | Uniq | -10lgP | Mass | ppm | m/z | z | RT | Scan | #Spec | Start | End | PTM |
| --- | --- | --- | --- | --- | --- | --- | --- | --- | --- | --- | --- | --- |
| I.LTGFANILGQ.S | Y | 22.34 | 1032.5604 | -11.7 | 517.2814 | 2 | 14.98 | 2187 | 1 | 1362 | 1371 |  |
| total 1 peptides |  |  |  |  |  |  |  |  |  |  |  |  |
| --- | --- | --- | --- | --- | --- | --- | --- | --- | --- | --- | --- | --- |

tr|A0A2D0QIP4|A0A2D0QIP4\_ICTPU

back to list

  

| Protein Coverage
| Supporting Peptides
|

Protein Coverage:

Supporting Peptides:

| Peptide | Uniq | -10lgP | Mass | ppm | m/z | z | RT | Scan | #Spec | Start | End | PTM |
| --- | --- | --- | --- | --- | --- | --- | --- | --- | --- | --- | --- | --- |
| I.LTGFANILGQ.S | Y | 22.34 | 1032.5604 | -11.7 | 517.2814 | 2 | 14.98 | 2187 | 1 | 1362 | 1371 |  |
| total 1 peptides |  |  |  |  |  |  |  |  |  |  |  |  |
| --- | --- | --- | --- | --- | --- | --- | --- | --- | --- | --- | --- | --- |

tr|A0A2D0QFG9|A0A2D0QFG9\_ICTPU

back to list

  

| Protein Coverage
| Supporting Peptides
|

Protein Coverage:

Supporting Peptides:

| Peptide | Uniq | -10lgP | Mass | ppm | m/z | z | RT | Scan | #Spec | Start | End | PTM |
| --- | --- | --- | --- | --- | --- | --- | --- | --- | --- | --- | --- | --- |
| I.LTGFANILGQ.S | Y | 22.34 | 1032.5604 | -11.7 | 517.2814 | 2 | 14.98 | 2187 | 1 | 1362 | 1371 |  |
| total 1 peptides |  |  |  |  |  |  |  |  |  |  |  |  |
| --- | --- | --- | --- | --- | --- | --- | --- | --- | --- | --- | --- | --- |

tr|A0EXC6|A0EXC6\_DANRE

back to list

  

| Protein Coverage
| Supporting Peptides
|

Protein Coverage:

Supporting Peptides:

| Peptide | Uniq | -10lgP | Mass | ppm | m/z | z | RT | Scan | #Spec | Start | End | PTM |
| --- | --- | --- | --- | --- | --- | --- | --- | --- | --- | --- | --- | --- |
| V.NKFLSGEI.V | Y | 21.77 | 906.4811 | -13.0 | 454.2419 | 2 | 14.84 | 2162 | 1 | 152 | 159 |  |
| total 1 peptides |  |  |  |  |  |  |  |  |  |  |  |  |
| --- | --- | --- | --- | --- | --- | --- | --- | --- | --- | --- | --- | --- |

tr|A4FUP0|A4FUP0\_DANRE

back to list

  

| Protein Coverage
| Supporting Peptides
|

Protein Coverage:

Supporting Peptides:

| Peptide | Uniq | -10lgP | Mass | ppm | m/z | z | RT | Scan | #Spec | Start | End | PTM |
| --- | --- | --- | --- | --- | --- | --- | --- | --- | --- | --- | --- | --- |
| V.NKFLSGEI.V | Y | 21.77 | 906.4811 | -13.0 | 454.2419 | 2 | 14.84 | 2162 | 1 | 152 | 159 |  |
| total 1 peptides |  |  |  |  |  |  |  |  |  |  |  |  |
| --- | --- | --- | --- | --- | --- | --- | --- | --- | --- | --- | --- | --- |

tr|A0A4V6ASI9|A0A4V6ASI9\_COLLU

back to list

  

| Protein Coverage
| Supporting Peptides
|

Protein Coverage:

Supporting Peptides:

| Peptide | Uniq | -10lgP | Mass | ppm | m/z | z | RT | Scan | #Spec | Start | End | PTM |
| --- | --- | --- | --- | --- | --- | --- | --- | --- | --- | --- | --- | --- |
| E.AGPGGPAGPVGAA.G | Y | 20.00 | 977.4930 | 0.2 | 489.7538 | 2 | 10.68 | 1446 | 1 | 974 | 986 |  |
| total 1 peptides |  |  |  |  |  |  |  |  |  |  |  |  |
| --- | --- | --- | --- | --- | --- | --- | --- | --- | --- | --- | --- | --- |

tr|A0A5J6XX13|A0A5J6XX13\_NIBCO

back to list

  

| Protein Coverage
| Supporting Peptides
|

Protein Coverage:

Supporting Peptides:

| Peptide | Uniq | -10lgP | Mass | ppm | m/z | z | RT | Scan | #Spec | Start | End | PTM |
| --- | --- | --- | --- | --- | --- | --- | --- | --- | --- | --- | --- | --- |
| E.AGPGGPAGPVGAA.G | Y | 20.00 | 977.4930 | 0.2 | 489.7538 | 2 | 10.68 | 1446 | 1 | 971 | 983 |  |
| total 1 peptides |  |  |  |  |  |  |  |  |  |  |  |  |
| --- | --- | --- | --- | --- | --- | --- | --- | --- | --- | --- | --- | --- |

---

Prepared with PEAKS ™ (bioinfor.com)
