# Supplementary material for: Proteomic and Functional Characterization of Antimicrobial Peptides Derived from Fisheries Bycatch via Enzymatic Hydrolysis
Source: Mar Drugs. 2026 Jan 10;24(1):36. doi: 10.3390/md24010036 (PMC12843163; doi:10.3390/md24010036)
Supplement: Supplementary file 1 [file marinedrugs-24-00036-s001.zip › SM44.pdf]

Appendix E – De novo peptide sequencing by LC–MS/MS

De novo peptide sequencing was performed by liquid chromatography coupled to tandem mass spectrometry (LC–MS/MS) to identify peptide sequences generated after enzymatic hydrolysis of fisheries bycatch samples. Peptide sequences were inferred from MS/MS fragmentation patterns using de novo sequencing algorithms, and confidence scores were assigned by the data processing software.

Only confidently identified peptide sequences were included in this appendix. The complete list of peptides identified for each hydrolysate is provided to ensure transparency and reproducibility and to support the bioinformatic and functional analyses presented in the main manuscript.

Table E1. De novo peptide sequences identified by LC–MS/MS in the Alcalase hydrolysate of *Hepatus pudibundus* (HPA). Peptide sequences were identified by LC–MS/MS de novo sequencing based on MS/MS fragmentation patterns. ALC (%) values represent the confidence of sequence assignment provided by the software.

Scan	Peptide	Tag length	ALC (%)	m/z	z	RT	Mass	ppm	local confidence (%)	tag (>=0%)	mode
1308	LTKLL	5	97	2.942.098	2	11.72	5.864.054	-0.5	96 95 97 99 99	LTKLL	CID
1128	LPTKF	5	95	3.031.871	2	10.77	6.043.584	2.1	97 94 94 95 98	LPTKF	CID
986	VVAKKPQE	8	95	4.497.803	2	10.02	8.975.283	19.8	98 99 99 99 94 88 89 96	VVAKKPQE	CID
762	KLHWH	5	94	3.607.007	2	8.76	7.193.867	0.1	98 100 88 88 98	KLHWH	CID
993	VHLKLPK	7	94	4.177.813	2	10.05	8.335.486	-0.7	92 96 98 90 96 94 94	VHLKLPK	CID
1523	MLSLKEM	7	93	4.262.292	2	12.91	8.504.293	17.1	91 97 97 97 96 92 83	MLSLKEM	CID
1518	WPGPLNF	7	92	4.157.058	2	12.89	8.294.122	-18.2	86 87 82 97 99 96 98	WPGPLNF	CID
1109	FGDKFT	6	92	3.576.764	2	10.67	7.133.384	-0.4	93 88 86 88 97 99	FGDKFT	CID
899	LDAPSQPEDF	10	91	5.597.551	2	9.53	11.174.927	2.6	89 94 97 98 90 95 90 92 89 80	LDAPSQPEDF	CID
1646	VVLPKNME	8	91	4.652.707	2	13.61	9.285.052	23.4	83 91 98 95 82 84 98 99	VVLPKNME	CID
1697	DFDLSLMFM	9	91	5.597.630	2	13.92	11.174.824	26.0	68 74 96 99 97 96 92 97 97	DFDLSLMFM	CID
1026	ELLPNTDAGDY	11	90	6.042.846	2	10.23	12.065.405	11.7	71 65 88 95 95 99 99 99 98 96 90	ELLPNTDAGDY	CID

599	VGDEAKTN	8	90	4.172.036	2	7.67	8.323.926	0.0	91 91 98 99 99 88 81 73	VGDEAKTN	CID
1221	YPGLADTW	8	90	4.617.260	2	11.25	9.214.232	15.5	94 95 97 99 96 92 79 69	YPGLADTW	CID
1328	VSVGDKLPAMP	11	90	5.573.112	2	11.82	11.125.898	16.1	89 92 92 94 98 98 99 92 91 83 65	VSVGDKLPAMP	CID
560	VGDEAQSK	8	90	4.172.036	2	7.39	8.323.926	0.0	92 92 97 99 94 84 84 77	VGDEAQSK	CID
953	GFAGDDAPR	9	89	4.532.093	2	9.83	9.044.039	0.1	70 89 96 98 98 95 98 85 79	GFAGDDAPR	CID
1247	DDRLSL	6	89	3.596.901	2	11.39	7.173.657	0.1	91 86 73 92 97 99	DDRLSL	CID
1275	LDRADF	6	89	3.686.973	2	11.54	7.353.551	33.8	89 91 81 94 93 89	LDRADF	CID
1087	MVAYFAH	7	89	4.197.014	2	10.56	8.373.843	4.6	93 96 98 98 95 72 74	MVAYFAH	CID
1002	DPVDNYPR	8	89	4.882.300	2	10.10	9.744.457	-0.3	84 91 97 99 92 96 83 74	DPVDNYPR	CID
1553	APEFPHPLPQF	11	89	6.403.275	2	13.08	12.786.396	0.6	67 76 97 99 95 95 92 96 96 86 83	APEFPHPLPQF	CID
614	KKPKF	5	89	3.242.155	2	7.78	6.464.166	-0.3	96 94 79 88 90	KKPKF	CID
1071	NYTDVPPVPK	10	89	5.652.995	2	10.47	11.285.815	2.6	69 77 98 99 100 99 94 89 84 85	NYTDVPPVPK	CID
1157	VTVPSLDPQF	10	89	5.517.860	2	10.92	11.015.706	-11.9	82 87 96 98 97 97 96 82 83 75	VTVPSLDPQF	CID
786	AGACPL	6	89	2.661.479	2	8.89	5.302.523	54.9	85 85 86 86 96 97	AGACPL	CID
1003	LDGANEDNLRE	11	89	6.232.885	2	10.11	12.445.632	-0.6	77 86 92 95 97 99 95 97 98 52 91	LDGANEDNLRE	CID
1805	DVFLGTFLYEYSR	13	89	8.053.983	2	14.62	16.087.825	-0.2	52 55 88 100 100 100 100 100 100 100 94 92 78	DVFLGTFLYEYSR	CID

1626	LAVLLVVEEGVAK	13	89	6.704.135	2	13.49	13.388.123	0.2	54 51 91 100 100 100 100 100 100 98 96 89 79	LAVLLVVEEGVAK	CID
1165	VNVDDPGKF	9	88	4.957.486	2	10.96	9.894.818	0.9	92 92 94 97 88 73 69 97 94	VNVDDPGKF	CID
1009	LTEEVAAALH	10	88	5.272.699	2	10.14	10.525.503	-23.8	86 82 99 99 96 87 62 79 98 94	LTEEVAAALH	CID
1119	SKLPKW	6	88	3.797.321	2	10.72	7.574.486	1.3	82 86 97 93 88 83	SKLPKW	CID
1557	EALEHLLL	8	88	4.692.711	2	13.10	9.365.280	-0.4	84 68 92 98 86 85 97 96	EALEHLLL	CID
1163	SLEDELR	7	88	4.312.197	2	10.95	8.604.240	1.0	79 85 99 94 99 86 73	SLEDELR	CID
1286	VLDLLK	6	88	3.507.338	2	11.60	6.994.531	-0.1	96 97 79 86 82 88	VLDLLK	CID
1027	TGDGVNDAPALK	12	87	5.792.952	2	10.24	11.565.723	3.1	61 69 91 94 99 99 99 92 86 89 89 83	TGDGVNDAPALK	CID
1175	LKYPLE	6	87	3.817.238	2	11.01	7.614.323	1.0	95 92 79 73 89 99	LKYPLE	CID
1152	NPLDKL	6	87	3.502.057	2	10.89	6.983.962	0.9	83 83 91 94 84 90	NPLDKL	CID
1301	MYPGLADRM	9	87	5.272.464	2	11.68	10.524.783	0.0	92 92 93 96 98 98 80 60 81	MYPGLADRM	CID
1065	LDAPSKPEDM	10	87	5.517.589	2	10.44	11.015.012	1.9	69 77 93 97 95 89 73 96 95 92	LDAPSKPEDM	CID
1261	KLLEDVHQN	9	87	5.483.057	2	11.46	10.945.720	22.7	86 96 95 99 95 97 88 72 61	KLLEDVHQN	CID
1335	FGPST	5	87	2.546.370	2	11.86	5.072.329	52.2	96 93 71 86 92	FGPST	CID
1333	VSKEVLDEM	9	87	5.252.688	2	11.85	10.485.110	11.5	92 95 95 90 48 79 98 98 89	VSKEVLDEM	CID
3160	LVRGPR	6	87	3.492.269	2	23.43	6.964.395	-0.4	96 96 85 82 85 79	LVRGPR	CID
1548	FSEWPGPW	8	87	5.032.325	2	13.05	10.044.392	11.2	71 78 98 94 98 95 80 79	FSEWPGPW	CID
1127	FGDKF	5	86	3.071.532	2	10.77	6.122.907	1.8	97 91 73 83 88	FGDKF	CID
1362	VLRLL	5	86	3.072.234	2	12.01	6.124.323	0.1	91 93 65 85 98	VLRLL	CID

1256	MDPLLVGPK	9	86	4.852.756	2	11.44	9.685.364	0.2	94 95 95 99 98 96 51 63 84	MDPLLVGPK	CID
1161	NYTDVPRHK	9	86	5.652.990	2	10.94	11.285.676	14.0	59 78 93 99 99 94 79 89 83	NYTDVPRHK	CID
1131	SDELKDL	7	86	4.102.223	2	10.78	8.184.022	34.1	85 87 97 99 80 72 82	SDELKDL	CID
1321	DSQLPELKDL	10	86	5.792.969	2	11.78	11.565.974	-15.6	68 71 79 98 97 99 95 94 77 81	DSQLPELKDL	CID
1590	ALEMDLFVK	9	86	5.332.861	2	13.28	10.645.576	0.1	71 77 97 93 78 79 90 95 92	ALEMDLFVK	CID
1205	FCELPDGQ	8	86	4.547.034	2	11.17	9.073.746	19.5	77 82 97 99 96 78 77 80	FCELPDGQ	CID
1615	NFDPTFVEDVKDF	13	86	7.868.641	2	13.43	15.717.144	-0.4	45 50 98 97 97 98 99 100 98 99 96 78 62	NFDPTFVEDVKDF	CID
1655	VENPFWWKDTY	11	85	7.428.453	2	13.66	14.836.772	-0.8	50 90 79 91 95 97 96 84 90 88 82	VENPFWWKDTY	CID
1371	LWKVY	5	85	3.547.076	2	12.06	7.074.006	-0.1	88 77 78 89 96	LWKVY	CID
1459	VTVPSLDDKEDMQF	14	85	8.123.815	2	12.56	16.227.498	-0.8	61 67 82 91 99 99 98 93 82 96 89 89 74 74	VTVPSLDDKEDMQF	CID
1376	LDLPRM	6	85	3.727.072	2	12.09	7.434.000	0.0	89 89 95 84 70 84	LDLPRM	CID
1579	APEEPHALL	9	85	4.887.516	2	13.22	9.755.025	-14.1	69 78 99 95 74 79 89 93 91	APEEPHALL	CID
1005	RQPDKRLM	8	85	5.222.852	2	10.12	10.425.706	-14.1	86 85 89 97 91 65 89 79	RQPDKRLM	CID
1206	VKLPKL	6	85	3.492.520	2	11.18	6.964.897	-0.4	66 84 90 85 86 98	VKLPKL	CID
926	WGSKGPHEW	9	85	5.422.462	2	9.68	10.824.934	-14.3	79 91 97 89 72 60 96 97 82	WGSKGPHEW	CID
1023	LPDWHDLDQV	10	85	6.192.948	2	10.22	12.365.774	-1.9	84 86 94 89 96 97 81 73 76 72	LPDWHDLDQV	CID
1596	TVEDELKVL	9	85	5.232.798	2	13.32	10.445.703	-24.2	41 63 98 99 100 99 81 90 91	TVEDELKVL	CID

Table E2. De novo peptide sequences identified by LC–MS/MS in the Protamex hydrolysate of *Hepatus pudibundus* (HPP). Peptide sequences were identified by LC–MS/MS de novo sequencing based on MS/MS fragmentation patterns. ALC (%) values represent the confidence of sequence assignment provided by the software.

Scan	Peptide	Tag length	ALC (%)	m/z	z	RT	Mass	ppm	local confidence (%)	tag (>=0%)	mode
1571	LKYPLE	6	95	3.817.234	2	11.29	7.614.323	0.0	98 96 92 94 94 99	LKYPLE	CID
1565	VNVDPDGKF	9	94	4.957.480	2	11.26	9.894.818	-0.2	92 94 98 99 91 96 91 96 94	VNVDPDGKF	CID
1369	LPDWHPMDR	9	93	5.837.753	2	10.20	11.655.339	1.9	97 97 98 98 99 91 82 90 91	LPDWHPMDR	CID
1354	VHLKLPK	7	93	4.177.815	2	10.11	8.335.486	-0.2	86 94 98 99 95 87 92	VHLKLPK	CID
2016	LDKFF	5	91	3.351.840	2	13.73	6.683.533	0.2	85 83 95 96 96	LDKFF	CID
1923	MLSLQFL	7	91	4.262.291	2	13.21	8.504.622	-21.8	93 99 91 91 90 88 86	MLSLQFL	CID
2247	VDFLGTFLYEYSR	13	90	8.053.986	2	15.06	16.087.825	0.1	60 63 81 97 99 100 100 100 100 100 99 95 80	VDFLGTFLYEYSR	CID
1570	LKMPL	5	90	3.011.995	2	11.28	6.003.669	29.2	96 83 80 94 98	LKMPL	CID
1464	LDAPSKPEDM	10	90	5.517.578	2	10.72	11.015.012	-0.1	90 92 95 97 92 97 63 94 92 86	LDAPSKPEDM	CID
1881	LHLML	5	89	3.136.866	2	12.98	6.253.621	-5.6	83 82 89 95 98	LHLML	CID
1691	TLKLL	5	89	2.942.096	2	11.93	5.864.054	-1.2	82 88 79 97 98	TLKLL	CID
1367	LLAPPERQHL	10	89	5.873.345	2	10.19	11.726.665	-10.2	98 99 99 95 76 95 74 90 81 82	LLAPPERQHL	CID
1482	APNELLAMK	9	88	4.937.794	2	10.82	9.855.266	18.0	86 89 92 99 95 85 82 86 85	APNELLAMK	CID
2004	VFAALVT	7	88	3.607.132	2	13.67	7.194.218	-13.8	96 97 94 94 84 74 81	VFAALVT	CID
1608	YPGLADWT	8	88	4.617.259	2	11.49	9.214.232	15.1	95 95 91 99 96 79 81 72	YPGLADWT	CID
1806	SPGVLDAGLVMH	12	88	5.983.101	2	12.56	11.946.067	-0.9	68 77 85 96 100 98 96 90 97 90 86 79	SPGVLDAGLVMH	CID
1402	LLAPLLVGQY	10	88	5.438.184	2	10.38	10.856.484	-24.2	97 98 98 98 97 92 74 67 82 80	LLAPLLVGQY	CID
1518	AKLPKW	6	88	3.717.338	2	11.01	7.414.537	-0.9	82 92 96 97 75 87	AKLPKW	CID
1668	LDNLFR	6	88	3.892.162	2	11.80	7.764.180	-0.3	92 94 92 97 77 75	LDNLFR	CID
1554	KLLP	4	87	2.356.703	2	11.20	4.693.264	-0.5	90 88 90 81	KLLP	CID
1909	VKLPVPVVGEQHTK	14	87	7.659.469	2	13.13	15.298.928	-8.9	75 82 94 86 90 90 94 98 85 98 86 87 80 77	VKLPVPVVGEQHTK	CID
1721	FGKFL	5	87	3.061.811	2	12.08	6.103.478	-0.3	94 87 79 82 95	FGKFL	CID
2023	FNDPTFVEDVQYV	13	87	7.868.642	2	13.77	15.717.144	-0.3	57 60 93 98 99 99 100 100 98 97 85 77 69	FNDPTFVEDVQYV	CID
1918	APEESFALL	9	86	4.887.516	2	13.18	9.754.913	-2.8	92 93 99 98 85 67 65 90 90	APEESFALL	CID
1307	KLPKL	5	86	2.997.178	2	9.84	5.974.213	-0.3	98 97 65 86 86	KLPKL	CID
1718	EKPLW	5	86	3.366.948	2	12.07	6.713.643	16.1	85 82 85 89 89	EKPLW	CID
908	EARE	4	86	2.526.244	2	7.59	5.032.339	0.6	95 95 56 98	EARE	CID
1667	APEESSML	8	86	4.322.091	2	11.80	8.623.742	34.1	89 90 97 79 62 85 92 93	APEESSML	CID
1648	MDPLLVKGP	9	86	4.852.751	2	11.70	9.685.364	-0.9	90 91 91 99 100 99 92 56 55	MDPLLVKGP	CID
1024	TEMEQ	5	85	3.191.284	2	8.26	6.362.425	-0.3	84 98 82 95 67	TEMEQ	CID
1588	APEESPVLLK	10	85	5.418.079	2	11.38	10.816.018	-0.6	76 81 98 99 98 60 65 95 95 88	APEESPVLLK	CID
1422	LPDGEHDLDRA	11	85	6.192.936	2	10.49	12.365.735	-0.7	92 92 96 73 98 97 98 78 73 73 68	LPDGEHDLDRA	CID

309	FHTDSQ	6	85	3.676.695	2	2.60	7.333.031	29.2	91 96 89 78 79 79	FHTDSQ	CID
1468	NYTDVPPPVK	10	85	5.652.980	2	10.74	11.285.815	-0.1	70 79 96 99 99 96 89 57 78 86	NYTDVPPPVK	CID
1152	KLHEGH	6	85	3.607.008	2	8.93	7.193.715	21.6	90 96 86 93 55 91	KLHEGH	CID
1905	FCLQLPF	7	85	4.342.393	2	13.11	8.664.360	32.2	81 83 93 88 90 76 84	FCLQLPF	CID
2097	LQWFDLLDKDKSGFNQ	16	85	9.774.758	2	14.20	19.529.631	-13.3	57 52 88 99 100 100 100 100 99 98 88 86 85 82 68 56	LQWFDLLDKDKSGFNQ	CID
1686	LKPLKL	6	85	3.562.597	2	11.90	7.105.054	-0.8	93 92 85 79 69 91	LKPLKL	CID

Table E3. De novo peptide sequences identified by LC–MS/MS in the Alcalase hydrolysate of *Micropogonias furnieri* (MFA). Peptide sequences were identified by LC–MS/MS de novo sequencing based on MS/MS fragmentation patterns. ALC (%) values represent the confidence of sequence assignment provided by the software.

Scan	Peptide	Tag length	ALC (%)	m/z	z	RT	Mass	ppm	local confidence (%)	tag (>=0%)	mode
2094	LDEVLKFF	8	95	5.057.813	2	14.37	10.095.484	-0.4	90 95 99 99 100 95 97 91	LDEVLKFF	CID
1054	LEHEE	5	95	3.286.490	2	8.51	6.552.813	3.2	89 96 97 98 97	LEHEE	CID
1619	LNNLL	5	93	2.936.705	2	11.73	5.853.486	-37.8	92 90 90 98 99	LNNLL	CID
1395	TKTPGLME	8	93	4.387.279	2	10.50	8.754.423	-1.2	95 96 97 86 85 94 97 98	TKTPGLME	CID
114	KLKKK	5	93	3.227.447	2	0.94	6.434.744	0.5	90 92 90 96 98	KLKKK	CID
2002	MKFLW	5	92	3.626.955	2	13.84	7.233.778	-1.8	86 91 97 97 91	MKFLW	CID
1528	LKYPLE	6	92	3.817.229	2	11.25	7.614.323	-1.5	97 96 97 74 90 98	LKYPLE	CID
1610	APPHLF	6	91	3.411.891	2	11.68	6.803.646	-1.5	95 95 86 87 91 96	APPHLF	CID
1311	LLAPPEVGKY	10	91	5.438.178	2	10.01	10.856.121	8.3	98 99 99 99 92 99 81 70 90 89	LLAPPEVGKY	CID
1526	VVDGVKL	7	91	3.652.283	2	11.24	7.284.432	-1.5	99 99 99 88 86 81 89	VVDGVKL	CID
2265	WLDPDDFPLLLFS	13	91	7.894.025	2	15.34	15.767.812	5.8	74 84 95 96 98 98 98 90 96 100 99 82 75	WLDPDDFPLLLFS	CID
1550	FLFGSKDKDPP	11	90	6.258.155	2	11.36	12.496.343	-14.3	96 98 96 91 97 98 98 96 98 70 59	FLFGSKDKDPP	CID
1137	AGDDAPVK	8	90	3.866.826	2	8.96	7.713.762	-33.0	88 87 99 99 99 79 86 87	AGDDAPVK	CID
2185	LLVYPW	6	90	3.957.285	2	14.89	7.894.425	0.0	92 94 94 95 83 85	LLVYPW	CID
147	KKTSPAE	7	90	3.807.189	2	1.29	7.594.127	14.0	98 96 91 88 86 82 93	KKTSPAE	CID
1493	FDDLQVR	7	90	4.467.291	2	11.05	8.914.450	-1.5	90 97 99 98 94 81 75	FDDLQVR	CID
1737	KDLLMVLTHSK	11	90	6.428.607	2	12.36	12.837.271	-15.7	90 94 99 99 95 95 99 95 76 77 72	KDLLMVLTHSK	CID
1907	KFLVLADLW	9	90	5.528.275	2	13.30	11.036.379	2.2	92 96 99 99 99 94 67 88 75	KFLVLADLW	CID
1300	LLAPLLHFYS	10	89	5.873.343	2	9.95	11.726.594	-4.6	92 97 95 96 82 89 89 93 87 77	LLAPLLHFYS	CID
1128	TERGY	5	89	3.131.508	2	8.90	6.242.867	0.4	87 96 72 93 98	TERGY	CID
1701	LDLLEKDL	8	89	4.797.668	2	12.17	9.575.382	-19.9	82 88 96 100 99 84 80 82	LDLLEKDL	CID
330	KCVHR	5	89	3.216.878	2	2.82	6.413.431	28.0	92 90 91 93 78	KCVHR	CID
1224	KDGCPGPPGLPGEH	14	89	6.808.327	2	9.50	13.596.240	19.7	48 80 93 99 100 99 99 97 94 98 94 95 87 61	KDGCPGPPGLPGEH	CID
1546	NVLRL	5	88	3.077.021	2	11.34	6.133.911	-2.2	85 91 92 82 93	NVLRL	CID

1587	ADDWLR	6	88	3.881.899	2	11.56	7.743.660	-1.1	79 90 95 96 93 79	ADDWLR	CID
1148	MENVAK	6	88	3.461.756	2	9.03	6.903.370	-0.7	85 95 81 85 91 95	MENVAK	CID
561	EARE	4	88	2.526.244	2	5.06	5.032.339	0.4	98 95 70 91	EARE	CID
2215	MDLFAHNE	8	88	4.887.139	2	15.06	9.754.120	1.2	91 93 93 90 76 77 90 95	MDLFAHNE	CID
23	EKKK	4	88	2.666.763	2	0.15	5.313.380	0.0	92 82 87 92	EKKK	CID
1509	VGDKLNL	7	88	3.797.235	2	11.14	7.574.334	-1.2	93 88 92 85 91 80 86	VGDKLNL	CID
1426	LDYVPR	6	87	3.817.104	2	10.67	7.614.072	-1.1	95 97 99 98 66 70	LDYVPR	CID
1926	FKLPF	5	87	3.261.964	2	13.40	6.503.792	-1.4	91 95 97 77 78	FKLPF	CID
14	FHET	4	87	2.671.338	2	0.10	5.322.281	46.6	93 82 93 81	FHET	CID
711	LVHR	4	87	2.626.689	2	6.34	5.233.231	0.2	95 91 91 72	LVHR	CID
1640	MYPGLADRM	9	87	5.272.455	2	11.84	10.524.783	-1.8	93 94 97 82 90 97 79 79 73	MYPGLADRM	CID
720	EARE	4	87	2.526.244	2	6.40	5.032.339	0.4	99 95 62 92	EARE	CID
974	QDGVTH	6	87	3.286.536	2	8.10	6.552.925	0.2	72 90 79 89 96 94	QDGVTH	CID
1352	NTLEDEKSL	9	87	5.247.610	2	10.24	10.475.083	-0.7	41 67 97 100 100 100 96 93 90	NTLEDEKSL	CID
243	AERE	4	87	2.526.244	2	2.14	5.032.339	0.6	86 95 77 89	AERE	CID
869	RKMHE	5	86	3.506.961	2	7.54	6.993.486	41.6	70 94 95 83 91	RKMHE	CID
1532	NGRHLL	6	86	3.551.971	2	11.27	7.084.031	-33.0	81 77 73 90 99 100	NGRHLL	CID
1510	FDARL	5	86	3.111.836	2	11.15	6.203.282	39.3	94 95 83 63 97	FDARL	CID
1617	KDLVDQL	7	86	4.157.338	2	11.72	8.294.545	-1.7	81 79 88 94 92 78 90	KDLVDQL	CID
1503	LLAGHE	6	86	3.201.888	2	11.11	6.383.387	38.2	97 97 94 54 75 99	LLAGHE	CID
1538	FKLKPL	6	86	3.732.514	2	11.30	7.444.897	-1.9	84 90 95 86 70 90	FKLKPL	CID
1322	APKLDPGE	8	86	4.137.180	2	10.07	8.254.232	-2.0	97 95 88 86 82 71 76 92	APKLDPGE	CID
2125	MAGMLDFDEFLQ	12	86	7.088.210	2	14.54	14.156.101	12.3	57 57 75 80 98 100 99 95 99 97 91 81	MAGMLDFDEFLQ	CID
1394	VAPMKAY	7	86	3.902.181	2	10.49	7.784.047	21.7	97 96 62 70 78 99 99	VAPMKAY	CID
1484	SVGASGLPGNH	11	85	4.982.604	2	11.00	9.944.832	23.2	61 69 83 86 95 92 99 98 98 83 78	SVGASGLPGNH	CID
2394	FDVALQPL	8	85	4.517.526	2	16.09	9.014.909	-0.4	85 90 94 95 89 74 76 79	FDVALQPL	CID
2051	LVLLPEVPKVL	11	85	6.104.048	2	14.12	12.187.952	-0.1	63 82 98 99 77 91 79 72 93 91 91	LVLLPEVPKVL	CID
1496	VNPYVE	6	85	3.606.814	2	11.07	7.193.490	-1.1	54 69 94 97 98 100	VNPYVE	CID
1479	YLKVE	5	85	3.261.888	2	10.97	6.503.639	-1.4	86 90 82 72 95	YLKVE	CID
454	KKTPA	5	85	2.726.821	2	3.99	5.433.380	21.5	98 92 73 81 82	KKTPA	CID
1346	LLPYMMNLK	9	85	5.617.925	2	10.21	11.215.977	-24.3	98 99 95 85 79 55 78 93 86	LLPYMMNLK	CID
1466	TSLDWFQ	7	85	4.487.210	2	10.90	8.954.076	22.3	94 96 98 90 77 70 69	TSLDWFQ	CID
1622	LFDKF	5	85	3.351.834	2	11.75	6.683.533	-1.5	78 72 96 87 92	LFDKF	CID
1702	MKKNEL	6	85	3.817.231	2	12.17	7.614.105	27.6	71 75 90 90 96 89	MKKNEL	CID
2067	MDLLRW	6	85	4.172.057	2	14.21	8.324.265	-35.7	98 99 100 91 43 79	MDLLRW	CID

Table E4. De novo peptide sequences identified by LC–MS/MS in the Protamex hydrolysate of *Micropogonias furnieri* (MFP). Peptide sequences were identified by LC–MS/MS de novo sequencing based on MS/MS fragmentation patterns. ALC (%) values represent the confidence of sequence assignment provided by the software.

Scan	Peptide	Tag length	ALC (%)	m/z	z	RT	Mass	ppm	local confidence (%)	tag (>=0%)	mode
2031	LEEEELKLF	9	97	5.753.053	2	13.44	11.485.964	-0.3	92 98 99 100 100 99 98 98 93	LEEEELKLF	CID
1897	FLDLDQDKKFEE	12	94	7.638.717	2	12.66	15.257.300	-0.7	79 92 99 99 99 98 99 97 89 90 96 91	FLDLDQDKKFEE	CID
1892	LVLHL	5	93	2.977.020	2	12.64	5.933.901	-1.2	85 95 99 93 96	LVLHL	CID
1669	VLDQDKSGFLE	11	93	6.258.165	2	11.38	12.496.189	-0.4	86 90 97 96 99 97 92 85 94 95 97	VLDQDKSGFLE	CID
1676	LEKERL	6	93	3.942.227	2	11.42	7.864.599	-37.0	94 98 91 97 85 94	LEKERL	CID
1650	LVLDAGDRTH	10	92	5.487.853	2	11.28	10.955.671	-10.2	79 86 95 98 99 96 97 94 93 90	LVLDAGDRTH	CID
1997	LVEELPARW	9	92	5.568.209	2	13.24	11.116.025	22.3	97 97 100 99 96 92 79 84 89	LVEELPARW	CID
484	LHDQH	5	92	3.251.564	2	3.70	6.482.980	0.3	89 86 93 97 99	LHDQH	CID
1115	VGDEAQSK	8	92	4.172.036	2	8.09	8.323.926	0.0	94 94 98 99 96 88 90 78	VGDEAQSK	CID
710	VMLKQ	5	91	3.096.761	2	5.47	6.173.571	-31.3	90 91 98 90 90	VMLKQ	CID
1976	FSVDEEFPDLSQH	13	91	7.753.446	2	13.11	15.486.731	1.0	65 68 91 97 100 100 100 99 98 98 93 88 86	FSVDEEFPDLSQH	CID
1572	LEDQYSELK	9	91	5.627.770	2	10.84	11.235.398	-0.3	95 99 92 95 93 75 94 91 84	LEDQYSELK	CID
1784	DVEEFPDLSHQ	11	90	6.582.932	2	12.03	13.145.728	-0.7	78 79 97 100 100 99 98 97 90 85 77	DVEEFPDLSHQ	CID
1639	FAGDDAPRAL	10	90	5.167.586	2	11.22	10.315.035	-0.9	94 96 93 100 99 99 70 79 89 88	FAGDDAPRAL	CID
1171	LKEAETR	7	90	4.237.376	2	8.38	8.454.606	0.1	87 89 98 95 99 82 79	LKEAETR	CID
1649	VVDRQL	6	89	3.652.289	2	11.27	7.284.181	34.5	97 97 93 72 88 90	VVDRQL	CID
2060	LDTVLDLKM	9	89	5.242.910	2	13.62	10.465.681	-0.7	90 88 94 99 99 83 85 83 82	LDTVLDLKM	CID
1841	LLDPLLSRD	9	89	5.213.002	2	12.35	10.405.865	-0.6	97 96 97 98 98 97 76 72 71	LLDPLLSRD	CID
2832	FAGDDAVKP	9	89	4.602.170	2	18.30	9.184.446	-27.4	90 97 92 99 95 97 81 83 65	FAGDDAVKP	CID
1682	FTNEKLQQF	9	88	5.777.957	2	11.46	11.535.767	0.1	78 86 92 99 90 93 94 88 80	FTNEKLQQF	CID
1565	LLWDPQY	7	88	4.677.477	2	10.80	9.334.596	22.7	99 99 92 97 70 77 88	LLWDPQY	CID
1506	WVDDAVQELN	10	88	5.947.783	2	10.44	11.875.459	-3.2	70 98 97 98 91 90 93 98 82 68	WVDDAVQELN	CID
199	WDEAKKK	7	88	4.527.402	2	1.52	9.034.814	-17.1	62 85 96 86 93 98 98	WDEAKKK	CID

1745	FGLEEEELK	9	88	5.472.736	2	11.82	10.925.339	-1.3	69 60 95 99 94 93 93 97 94	FGLEEEELK	CID
311	AGGERK	6	88	3.091.719	2	2.39	6.163.292	0.0	85 82 91 97 80 95	AGGERK	CID
3236	KTKLLL	6	88	3.582.678	2	20.70	7.145.003	28.9	75 76 85 97 97 98	KTKLLL	CID
1747	LGEDFLRD	8	88	4.827.398	2	11.83	9.634.661	-1.1	94 86 98 100 99 93 54 79	LGEDFLRD	CID
2301	FAGDSFYL	8	88	4.602.206	2	15.05	9.184.123	15.6	88 90 78 93 95 89 88 81	FAGDSFYL	CID
1517	WGASGLPGPP	10	87	4.697.507	2	10.51	9.374.658	22.5	66 90 97 100 99 100 99 95 70 62	WGASGLPGPP	CID
1181	NDENVRQ	7	87	4.377.042	2	8.43	8.733.940	-0.2	81 95 98 81 92 85 81	NDENVRQ	CID
650	LHDQH	5	87	3.251.563	2	5.03	6.482.980	0.1	79 81 92 90 95	LHDQH	CID
124	TKEKKA	6	87	3.527.191	2	0.96	7.034.228	1.1	81 88 96 95 82 81	TKEKKA	CID
1675	LFKYP	5	87	3.341.942	2	11.42	6.663.741	-0.2	85 78 90 94 88	LFKYP	CID
3760	WGGHEW	6	87	3.861.671	2	23.76	7.703.136	7.9	95 94 87 78 90 79	WGGHEW	CID
1691	FEEELFGSKDKDGH	14	87	8.193.803	2	11.51	16.367.368	5.7	74 96 99 100 100 100 98 99 99 97 96 90 32 40	FEEELFGSKDKDGH	CID
2163	YLDLKDMPFYA	11	87	6.883.329	2	14.23	13.746.528	-1.2	79 86 95 96 95 93 95 79 88 80 70	YLDLKDMPFYA	CID
441	AAEDTQAAK	9	87	4.527.276	2	3.36	9.034.297	12.0	85 91 97 92 91 79 74 86 86	AAEDTQAAK	CID
1689	LDDAVRAL	8	86	4.367.452	2	11.50	8.714.763	-0.4	86 91 99 99 96 61 77 85	LDDAVRAL	CID
770	KTLQQ	5	86	3.091.719	2	5.93	6.163.544	-40.7	97 88 83 77 88	KTLQQ	CID
443	VTRE	4	86	2.526.308	2	3.32	5.032.704	-46.2	90 95 66 96	VTRE	CID
1706	LTDVLHVT	8	86	4.492.552	2	11.60	8.964.967	-1.0	89 90 91 86 96 82 80 78	LTDVLHVT	CID
1705	ADYEDLYK	8	86	5.087.317	2	11.59	10.154.498	-0.9	84 92 99 99 76 82 79 80	ADYEDLYK	CID
1596	FDELDDNLP	9	86	5.392.504	2	10.98	10.764.661	18.8	92 96 99 98 96 97 62 80 55	FDELDDNLP	CID
3023	FAGDDAPVK	9	86	4.602.169	2	19.43	9.184.446	-27.6	94 95 91 98 99 94 68 74 62	FAGDDAPVK	CID
1791	MEPKSHHM	8	86	4.987.296	2	12.07	9.954.317	13.0	93 98 91 89 88 73 81 75	MEPKSHHM	CID
1867	GFTADEKLGLY	11	86	6.073.082	2	12.49	12.126.025	-0.5	46 70 85 96 97 99 94 90 83 94 93	GFTADEKLGLY	CID
3805	DAGAAETH	8	86	3.861.668	2	24.04	7.703.195	-0.4	74 86 85 90 96 95 81 82	DAGAAETH	CID
1748	LLFK	4	86	2.606.780	2	11.83	5.193.420	-1.2	89 86 90 79	LLFK	CID
1439	LNEDKLQDKLDK	12	86	7.298.934	2	10.04	14.577.725	-0.2	55 78 92 98 93 95 95 99 93 91 78 66	LNEDKLQDKLDK	CID
183	VDKR	4	86	2.591.583	2	1.40	5.163.020	0.2	89 91 85 79	VDKR	CID
1800	WEQVKLW	7	86	4.947.582	2	12.12	9.875.178	-16.2	75 98 97 97 82 76 76	WEQVKLW	CID
2129	VELKDMPFY	9	86	5.712.835	2	14.03	11.405.525	0.0	62 85 90 85 84 96 82 93 95	VELKDMPFY	CID

2166	VTEDPFDQTEDAENQEK	17	85	9.979.260	2	14.25	19.938.389	-0.7	54 78 97 99 98 99 95 87 76 92 88 98 99 85 66 86 56	VTEDPFDQTEDAENQEK	CID
3821	LVNR	4	85	2.511.590	2	24.14	5.003.071	-7.3	92 85 84 80	LVNR	CID
2699	LETAQL	6	85	3.376.803	2	17.49	6.733.646	-27.5	88 96 86 91 66 86	LETAQL	CID
1124	VLEVGR	6	85	3.367.112	2	8.14	6.713.966	16.8	72 94 94 78 84 89	VLEVGR	CID
1693	NQKTLDDLEDE	11	85	6.603.139	2	11.52	13.185.889	18.5	29 32 89 99 99 99 99 99 98 95 96	NQKTLDDLEDE	CID
1542	LDKVDEDRYDME	12	85	7.643.349	2	10.66	15.266.558	-0.3	55 75 72 95 99 98 93 75 90 84 87 95	LDKVDEDRYDME	CID

Table E5. De novo peptide sequences identified by LC–MS/MS in the Alcalase hydrolysate of *Paralonchurus brasiliensis* (PBA). Peptide sequences were identified by LC–MS/MS de novo sequencing based on MS/MS fragmentation patterns. ALC (%) values represent the confidence of sequence assignment provided by the software.

Scan	Peptide	Tag length	ALC (%)	m/z	z	RT	Mass	ppm	local confidence (%)	tag (>=0%)	mode
1584	LLAPPE	6	97	3.201.890	2	11.09	6.383.639	-0.8	95 95 96 99 99 100	LLAPPE	CID
90	YEKK	4	96	2.841.605	2	0.69	5.663.064	0.1	90 97 99 99	YEKK	CID
2104	LVYPSV	6	94	3.391.865	2	13.97	6.763.796	-31.2	95 95 98 93 93 94	LVYPSV	CID
1612	VKLPKL	6	94	3.492.519	2	11.25	6.964.897	-0.6	94 96 97 86 95 96	VKLPKL	CID
1606	LKYPLE	6	94	3.817.231	2	11.22	7.614.323	-0.8	97 96 97 84 93 99	LKYPLE	CID
2257	LDFDEFLMK	9	94	5.792.814	2	14.85	11.565.474	0.7	89 93 98 98 100 98 96 90 85	LDFDEFLMK	CID
1678	LLDQDKSGFLE	11	93	6.328.278	2	11.60	12.636.345	5.2	99 99 98 97 99 96 89 76 90 93 95	LLDQDKSGFLE	CID
1667	SERRLL	6	93	3.872.292	2	11.54	7.724.555	-15.0	79 93 93 94 99 99	SERRLL	CID
874	EYKK	4	92	2.841.605	2	7.02	5.663.064	0.0	96 90 87 96	EYKK	CID
2266	LLVYPW	6	91	3.957.284	2	14.90	7.894.425	-0.4	83 90 92 97 95 93	LLVYPW	CID
1639	TGPLKF	6	91	3.316.970	2	11.39	6.613.799	-0.8	87 85 90 94 96 96	TGPLKF	CID
1961	VDLVSFK	7	91	4.042.233	2	13.15	8.064.538	-26.9	93 95 97 89 85 90 92	VDLVSFK	CID
1053	QDDKLKDK	8	91	4.952.665	2	8.10	9.885.189	-0.5	82 93 92 95 94 89 97 89	QDDKLKDK	CID
257	VGADKK	6	90	3.091.841	2	2.03	6.163.544	-1.3	91 88 87 88 93 93	VGADKK	CID
1600	VVDRLK	6	90	3.652.286	2	11.18	7.284.545	-16.1	97 96 94 74 90 88	VVDRLK	CID
1984	LDLPKLM	7	89	4.152.462	2	13.28	8.284.779	-0.2	95 94 96 93 79 82 86	LDLPKLM	CID
1204	TDALQR	6	89	3.521.902	2	8.91	7.023.660	-0.3	88 93 97 99 76 82	TDALQR	CID
1547	TSLDWFQ	7	89	4.487.218	2	10.88	8.954.076	23.9	93 96 97 88 91 78 80	TSLDWFQ	CID
2568	WGDRTH	6	89	3.861.669	2	16.69	7.703.460	-34.8	86 95 94 87 82 90	WGDRTH	CID
1572	VNPYVE	6	88	3.606.816	2	11.02	7.193.490	-0.5	65 79 93 96 98 99	VNPYVE	CID
1400	APKLPDGDKV	10	88	5.202.917	2	10.03	10.385.709	-1.9	71 82 92 100 99 99 76 92 89 83	APKLPDGDKV	CID
1552	FDELDDPNL	9	88	5.392.508	2	10.91	10.764.661	19.5	92 96 99 99 99 95 57 78 77	FDELDDPNL	CID
467	DPWLR	5	88	3.436.884	2	3.69	6.853.547	10.9	84 94 91 96 75	DPWLR	CID
1495	EVDLR	5	88	3.161.739	2	10.59	6.303.337	-0.6	90 76 91 95 88	EVDLR	CID
2262	FVLLAHPL	8	88	4.552.682	2	14.88	9.085.483	-29.3	77 89 95 99 86 75 92 91	FVLLAHPL	CID
274	AERE	4	87	2.526.244	2	2.16	5.032.339	0.6	83 92 82 92	AERE	CID
1705	YFKLKPL	7	87	4.547.835	2	11.75	9.075.531	-0.8	90 94 97 99 98 59 76	YFKLKPL	CID

1271	KDGSSGLPGLPGEY	14	87	6.888.306	2	9.27	13.756.619	-11.0	39 75 84 99 98 92 98 99 99 100 99 97 85 55	KDGSSGLPGLPGEY	CID
8	KLLT	4	87	2.376.737	2	0.07	4.733.213	24.3	94 86 90 78	KLLT	CID
2051	VAKVLQVL	8	86	4.352.942	2	13.66	8.685.746	-0.9	62 70 89 95 89 92 98 98	VAKVLQVL	CID
1908	FGLRT	5	86	2.971.756	2	12.85	5.923.333	5.8	97 94 96 75 70	FGLRT	CID
1577	FDDLQVR	7	86	4.467.296	2	11.05	8.914.450	-0.4	80 97 89 93 88 85 71	FDDLQVR	CID
1601	FDEMDPPLKD	10	86	6.037.719	2	11.19	12.055.273	1.6	85 94 99 92 89 83 93 95 70 61	FDEMDPPLKD	CID
1963	MMVGLL	6	86	3.321.945	2	13.16	6.623.495	37.6	77 86 93 70 96 94	MMVGLL	CID
1658	WQLDNL	6	86	3.946.929	2	11.49	7.873.864	-19.2	84 85 93 79 82 92	WQLDNL	CID
759	KDDLR	5	86	3.236.853	2	6.12	6.453.445	17.8	90 76 79 91 93	KDDLR	CID
1780	LGPLKVL	7	85	3.702.574	2	12.15	7.385.003	-0.1	95 87 82 86 68 87 95	LGPLKVL	CID
1644	YPGLADSAE	9	85	4.617.257	2	11.42	9.214.080	31.4	84 88 90 99 97 77 65 81 89	YPGLADSAE	CID
2068	AFLPM	5	85	2.896.538	2	13.76	5.772.934	-0.7	74 79 89 89 95	AFLPM	CID
2130	ADLFPM	6	85	3.471.674	2	14.12	6.923.203	0.1	79 85 85 79 92 90	ADLFPM	CID
1679	QDDLLEKL	8	85	4.872.719	2	11.61	9.725.128	17.0	74 93 94 94 86 82 75 81	QDDLLEKL	CID
1804	LKWL	4	85	2.801.835	2	12.28	5.583.530	-1.0	93 88 78 80	LKWL	CID

Table E6. De novo peptide sequences identified by LC–MS/MS in the Protamex hydrolysate of *Paralonchurus brasiliensis* (PBP). Peptide sequences were identified by LC–MS/MS de novo sequencing based on MS/MS fragmentation patterns. ALC (%) values represent the confidence of sequence assignment provided by the software.

Scan	Peptide	Tag length	ALC (%)	m/z	z	RT	Mass	ppm	local confidence (%)	tag (>=0%)	mode
1927	LEEEELKLF	9	97	5.753.052	2	13.44	11.485.964	-0.4	89 98 99 99 99 98 97 99 95	LEEEELKLF	CID
324	EYKK	4	95	2.841.604	2	2.46	5.663.064	-0.3	99 95 91 98	EYKK	CID
1879	VDLWFK	6	95	4.042.233	2	13.17	8.064.327	-0.9	96 97 98 95 93 92	VDLWFK	CID
1961	LKLFL	5	94	3.172.202	2	13.64	6.324.261	-0.4	90 94 93 95 98	LKLFL	CID
1696	LEDDLVALK	9	92	5.082.898	2	12.13	10.145.597	5.3	88 96 92 96 94 95 93 94 87	LEDDLVALK	CID
1124	VGDEAKSQ	8	91	4.172.036	2	8.15	8.323.926	0.1	90 90 97 99 96 91 87 77	VGDEAKSQ	CID
1915	VDLWFKA	7	90	4.397.417	2	13.37	8.774.698	-1.1	92 95 99 86 90 84 89	VDLWFKA	CID
1551	TVDDKVELE	9	90	5.242.637	2	11.30	10.465.132	-0.4	79 82 94 96 92 82 93 97 97	TVDDKVELE	CID
1832	LVVDRKL	7	90	4.217.705	2	12.90	8.415.385	-14.4	95 94 95 83 71 97 96	LVVDRKL	CID
1392	LLDKNRDGLLSQ	12	90	6.863.862	2	10.32	13.707.517	4.4	86 93 88 93 85 85 98 89 98 98 92 79	LLDKNRDGLLSQ	CID
542	EARE	4	90	2.526.242	2	4.23	5.032.339	-0.1	95 93 76 96	EARE	CID
635	QGREK	5	90	3.091.719	2	4.94	6.163.292	0.1	90 80 84 98 96	QGREK	CID
1897	LVEELPVKW	9	89	5.568.206	2	13.27	11.116.277	-1.0	89 88 100 100 99 80 75 93 85	LVEELPVKW	CID
1374	KSLEDEQSLGAQ	12	89	6.528.231	2	10.20	13.036.255	4.7	59 79 91 100 100 100 98 99 99 84 92 78	KSLEDEQSLGAQ	CID
28	AKTK	4	89	2.241.501	2	0.18	4.462.853	0.8	86 94 90 89	AKTK	CID
1786	ALLDQDNSGFLEEEELK	17	89	9.754.704	2	12.63	19.489.265	-0.1	41 86 89 92 95 98 94 97 91 96 100 100 99 99 90 83 75	ALLDQDNSGFLEEEELK	CID
3687	WGDHEQ	6	89	3.861.672	2	23.87	7.702.983	27.9	93 90 93 82 92 86	WGDHEQ	CID
143	KGREQ	5	89	3.091.719	2	1.06	6.163.292	0.1	92 84 82 96 94	KGREQ	CID
1815	KEFLEEL	7	89	4.542.419	2	12.80	9.064.698	-0.5	57 89 89 97 99 99 98	KEFLEEL	CID

1959	LGEDFLTGLL	10	89	5.392.854	2	13.63	10.765.754	-17.8	89 78 96 99 99 99 82 78 89 86	LGEDFLTGLL	CID
1993	FDMDKLPL	8	89	4.897.516	2	13.83	9.774.892	-0.7	85 89 91 91 94 98 80 86	FDMDKLPL	CID
1190	NDENVRQ	7	89	4.377.041	2	8.49	8.733.940	-0.4	72 90 99 95 97 86 84	NDENVRQ	CID
791	FCDKH	5	89	3.251.561	2	6.10	6.482.690	44.2	90 86 91 92 86	FCDKH	CID
1596	FAGDDAPRAF	10	89	5.337.512	2	11.54	10.654.878	0.0	93 96 90 99 99 99 92 81 68 71	FAGDDAPRAF	CID
1522	VFCGNGEKLET	11	88	5.987.839	2	11.12	11.955.542	-0.8	85 93 91 78 92 95 98 96 99 88 62	VFCGNGEKLET	CID
1756	FEEELFGSKDKDHW	14	88	8.839.014	2	12.46	17.657.947	-3.7	92 99 100 100 98 92 79 95 98 99 95 94 56 44	FEEELFGSKDKDHW	CID
1588	FDELDDLTKSH	11	88	6.603.140	2	11.50	13.186.040	7.2	82 94 100 99 99 100 99 98 97 59 49	FDELDDLTKSH	CID
1080	LVKER	5	88	3.227.081	2	7.92	6.434.017	0.1	84 83 88 95 91	LVKER	CID
315	NDQQKSDEK	9	88	5.462.517	2	2.39	10.904.890	-0.1	62 80 85 96 94 95 95 97 86	NDQQKSDEK	CID
1664	LELLEKRE	8	88	5.152.857	2	11.93	10.285.865	-28.9	87 97 98 99 98 93 57 75	LELLEKRE	CID
1641	GFLEEEEKL	9	88	5.472.741	2	11.80	10.925.339	-0.2	46 82 97 99 99 96 97 88 88	GFLEEEEKL	CID
7	VGDEAQSK	8	87	4.172.018	2	0.04	8.323.926	-4.4	82 82 95 99 96 82 89 78	VGDEAQSK	CID
145	KLKKK	5	87	3.227.447	2	1.08	6.434.744	0.7	68 85 91 95 98	KLKKK	CID
1995	NFLLDFADQLEPQ	13	87	7.753.726	2	13.84	15.487.458	-9.8	54 60 99 99 99 99 98 98 90 95 95 79 73	NFLLDFADQLEPQ	CID
2094	VETVLDMLKW	10	87	6.173.229	2	14.44	12.326.475	-13.2	55 82 85 98 99 98 91 97 87 81	VETVLDMLKW	CID
95	AERE	4	87	2.526.244	2	0.73	5.032.339	0.7	85 95 76 92	AERE	CID
1005	LHDQH	5	87	3.251.563	2	7.53	6.482.980	0.1	82 85 93 79 95	LHDQH	CID
598	QGKER	5	87	3.091.720	2	4.68	6.163.292	0.5	93 93 72 92 85	QGKER	CID
1850	QLDAFDLLDKDTN	13	86	7.543.659	2	13.00	15.067.202	-2.0	57 88 91 98 100 100 100 100 99 99 86 65 44	QLDAFDLLDKDTN	CID
281	QGERK	5	86	3.091.720	2	2.12	6.163.292	0.5	71 89 95 85 93	QGERK	CID
1809	FEVPKSGSPP	10	86	5.227.839	2	12.77	10.435.288	23.5	88 97 98 90 84 88 91 92 72 66	FEVPKSGSPP	CID
399	LVHKR	5	86	3.267.162	2	3.07	6.514.180	-0.3	94 95 98 88 56	LVHKR	CID
1760	NNLDQDKSGFLE	12	86	6.903.375	2	12.49	13.786.365	17.3	57 79 98 97 98 99 98 81 63 75 94 93	NNLDQDKSGFLE	CID
1354	MNEPKLKDKLNE	12	86	7.298.974	2	10.03	14.577.549	17.4	48 49 95 97 100 99 97 99 90 88 85 90	MNEPKLKDKLNE	CID
1469	LLADRM	6	86	3.596.982	2	10.82	7.173.843	-3.5	99 99 88 89 61 80	LLADRM	CID
1548	LALASF	6	86	3.111.858	2	11.28	6.203.533	6.1	96 96 96 73 80 74	LALASF	CID
1732	FAPPDVQN	8	86	4.442.165	2	12.33	8.864.185	0.0	69 74 85 96 97 94 92 80	FAPPDVQN	CID
1605	FLFGSKDKDHA	11	86	6.328.243	2	11.60	12.636.248	7.3	91 93 86 89 96 88 90 94 88 68 60	FLFGSKDKDHA	CID
1529	LFDRVGDSQ	9	85	5.187.564	2	11.16	10.354.985	-0.3	91 93 95 92 95 61 78 92 72	LFDRVGDSQ	CID
2582	LQDFADLLDKDKSGFNQ	17	85	9.774.777	2	17.33	19.529.480	-3.7	52 75 79 82 90 99 100 100 100 99 99 99 95 82 77 76 51	LQDFADLLDKDKSGFNQ	CID
1614	LLDKVDEDRFML	12	85	7.473.760	2	11.65	14.927.595	-14.8	94 95 92 97 100 99 92 79 74 68 62 75	LLDKVDEDRFML	CID
1724	LLDQDNSGFLEEENQK	16	85	9.399.510	2	12.28	18.778.643	12.4	70 69 90 96 99 98 99 98 99 99 100 100 96 43 45 67	LLDQDNSGFLEEENQK	CID
1847	CCLLKLLVAM	10	85	5.537.985	2	12.99	11.056.062	-21.4	85 85 98 99 81 66 89 85 85 79	CCLLKLLVAM	CID
1928	VDLWFK	6	85	4.042.235	2	13.45	8.064.327	-0.3	90 93 95 69 85 81	VDLWFK	CID

1479	RDNPGHPF	8	85	4.702.193	2	10.88	9.384.359	-12.6	66 92 96 88 92 95 80 73	RDNPGHPF	CID
1898	MMLLDQDKSGFNK	13	85	7.638.712	2	13.28	15.257.268	0.7	55 62 87 99 100 98 98 95 91 89 95 72 68	MMLLDQDKSGFNK	CID
1599	FEEELFGSRDSRPP	14	85	8.333.835	2	11.56	16.647.793	-16.1	68 95 99 99 100 99 96 98 76 86 78 62 72 66	FEEELFGSRDSRPP	CID
2191	FLTLGTFLYECFR	13	85	8.053.986	2	15.01	16.088.010	-11.5	52 67 84 98 97 99 100 100 100 100 80 71 61	FLTLGTFLYECFR	CID
1814	VKNWPW	6	85	4.152.213	2	12.80	8.284.282	-0.3	91 88 80 79 84 88	VKNWPW	CID
698	KEQK	4	85	2.666.582	2	5.44	5.313.016	0.4	71 95 85 89	KEQK	CID
697	AERE	4	85	2.526.244	2	5.43	5.032.339	0.4	74 92 79 95	AERE	CID
96	KAKT	4	85	2.241.500	2	0.73	4.462.853	0.6	85 86 84 85	KAKT	CID
2668	FLFGTTQRSHW	11	85	6.903.382	2	17.85	13.786.782	-11.9	86 95 95 82 92 85 88 87 94 70 61	FLFGTTQRSHW	CID
1533	LEKSYELPDGQ	11	85	6.398.138	2	11.19	12.776.140	-0.7	66 84 87 91 95 99 99 85 86 69 73	LEKSYELPDGQ	CID
2038	VLLLPEPVKVL	11	85	6.104.048	2	14.11	12.187.952	0.0	60 76 98 99 93 97 67 77 91 89 88	VLLLPEPVKVL	CID
1186	LVRD	4	85	2.516.529	2	8.47	5.012.911	0.4	89 82 81 88	LVRD	CID
1730	LLDPLLSRD	9	85	5.213.004	2	12.32	10.405.865	-0.3	98 97 97 92 99 98 72 55 56	LLDPLLSRD	CID
2145	LNHQGTWL	8	85	4.847.383	2	14.74	9.674.875	-26.4	90 91 94 81 65 90 83 84	LNHQGTWL	CID
