# Supplementary material for: Proteomic and Functional Characterization of Antimicrobial Peptides Derived from Fisheries Bycatch via Enzymatic Hydrolysis
Source: Mar Drugs. 2026 Jan 10;24(1):36. doi: 10.3390/md24010036 (PMC12843163; doi:10.3390/md24010036)
Supplement: Supplementary file 1 [file marinedrugs-24-00036-s001.zip › SM45.pdf]

**Appendix F / Figure 4.** Raw antimicrobial inhibition data (%) of peptide hydrolysates and fractions obtained from *Paralonchurus brasiliensis*, *Micropogonias furnieri*, and *Hepatus pudibundus* following enzymatic hydrolysis and RP-HPLC fractionation. Assays were performed against *Staphylococcus aureus* ATCC 25923, *Escherichia coli* ATCC 25922, and *Candida albicans* ATCC 10231. Hydrolysate and fraction assays were conducted in duplicate (n = 2), while negative and positive controls were performed in triplicate (n = 3). Results are expressed as mean ± standard deviation. Negative inhibition values indicate microbial growth exceeding the negative control. Given the exploratory nature of this screening assay and the limited number of replicates, no inferential statistical analyses were applied.

*S. aureus ATCC 25923:*

| Fraction | Replicate 1 (%) | Replicate 2 (%) | Mean | SD |
|---|---|---|---|---|
| MPA 1 mg/mL | -30.08 | -37.40 | -33.74 | 5.18 |
| MPA 250 µg/mL | -34.15 | -19.11 | -26.63 | 10.63 |
| MPP 1 mg/mL | -19.51 | -19.11 | -19.31 | 0.28 |
| MPP 250 µg/mL | -9.76 | -1.22 | -5.49 | 6.04 |
| PBA 1 mg/Ml | 2.28 | -1.22 | 0.53 | 2.48 |
| PBA 250 µg/mL | 33.33 | 25.20 | 29.27 | 5.75 |
| PBP 1 mg/mL | 6.91 | 42.68 | 24.79 | 25.29 |
| PBP 250 µg/ml | 0.81 | 17.07 | 8.94 | 11.49 |
| HPA 1 mg/mL | -14.22 | 38.62 | 12.20 | 37.36 |
| HPA 250 µg/mL | 20.73 | 18.29 | 19.51 | 1.73 |

*C. albicans ATCC 10231:*

| Fraction | Replicate 1 (%) | Replicate 2 (%) | Mean | SD |
|---|---|---|---|---|
| MPA 1 mg/mL | 23.53 | 21.8 | 22.67 | 1.22 |
| MPA 250 µg/mL | 83.39 | 23.01 | 53.2 | 42.69 |
| MPP 1 mg/mL | 9.52 | 24.91 | 17.22 | 10.88 |
| MPP 250 µg/mL | 40.14 | 52.77 | 46.46 | 8.93 |
| PBA 1 mg/Ml | 28.55 | 38.41 | 33.48 | 6.97 |
| PBA 250 µg/mL | 26.82 | 84.95 | 55.89 | 41.10 |
| PBP 1 mg/mL | 37.54 | 53.11 | 45.33 | 11.00 |
| PBP 250 µg/ml | 81.31 | 81.14 | 81.23 | 0.12 |
| HPA 1 mg/mL | 13.15 | 64.88 | 39.01 | 36.58 |
| HPA 250 µg/mL | 6.92 | 1.56 | 4.24 | 3.79 |

*E. coli ATCC 25922*:

| Fraction | Replicate 1 (%) | Replicate 2 (%) | Mean | SD |
|---|---|---|---|---|
| MPA 1 mg/mL | -1.73 | -1.15 | -1.44 | 0.41 |
| MPA 250 µg/mL | 7.29 | 7.49 | 7.39 | 0.14 |
| MPP 1 mg/mL | -4.22 | -5.37 | -4.79 | 0.81 |
| MPP 250 µg/mL | 7.1 | 7.87 | 7.48 | 0.54 |
| PBA 1 mg/Ml | -1.73 | 0.96 | -0.38 | 1.90 |
| PBA 250 µg/mL | 0.96 | 7.68 | 4.32 | 4.75 |
| PBP 1 mg/mL | -2.5 | -1.92 | -2.21 | 0.41 |
| PBP 250 µg/ml | 9.99 | 8.45 | 9.22 | 1.09 |
| HPA 1 mg/mL | -1.15 | -4.41 | -2.78 | 2.30 |
| HPA 250 µg/mL | -1.73 | -1.92 | -1.82 | 0.13 |

No treatment:

| | **Replicate 1 (%)** | **Replicate 2 (%)** | **Replicate 3 (%)** | **Média** | **DP** |
|---|---|---|---|---|---|
| ***S. aureus* ATCC 25923** | 0.24 | 0.26 | 0.25 | 0.25 | 0.01 |
| ***E. coli* ATCC 25922** | 0.50 | 0.52 | 0.53 | 0.52 | 0.01 |
| ***C. albicans* ATCC 10231** | 0.57 | 0.57 | 0.61 | 0.58 | 0.02 |

Positive control - Ampicillin, 2 μg/mL (for bacteria):

| | **Replicate 1 (%)** | **Replicate 2 (%)** | **Replicate 3 (%)** | **Média** | **DP** |
|---|---|---|---|---|---|
| ***S. aureus* ATCC 25923** | 99.19 | 100.00 | 99.60 | 99.60 | 0.40 |
| ***E. coli* ATCC 25922** | 100.00 | 100.00 | 99.61 | 99.87 | 0.22 |

Positive control - fluconazole, 1 μg/mL (for *C. albicans):*

| | **Replicate 1 (%)** | **Replicate 2 (%)** | **Replicate 3 (%)** | **Média** | **DP** |
|---|---|---|---|---|---|
| ***C. albicans* ATCC 10231** | 100.00 | 100.00 | 99.65 | 99.88 | 0.20 |
