# Supplementary material for: Proteomic and Functional Characterization of Antimicrobial Peptides Derived from Fisheries Bycatch via Enzymatic Hydrolysis
Source: Mar Drugs. 2026 Jan 10;24(1):36. doi: 10.3390/md24010036 (PMC12843163; doi:10.3390/md24010036)
Supplement: Supplementary file 1 [file marinedrugs-24-00036-s001.zip › SM46.pdf]

Appendix F. Raw antimicrobial inhibition data (%) of chromatographic fractions obtained from *Paralonchurus brasiliensis* Protamex hydrolysate (PBP) following RP-HPLC separation. Assays were performed against *Staphylococcus aureus* ATCC 25923, *Escherichia coli* ATCC 25922, and *Candida albicans* ATCC 10231 in duplicate (n = 2). Results are expressed as mean ± standard deviation.

Given the exploratory nature of this screening assay and the limited number of replicates, no inferential statistical analyses were applied. Inhibition values below 1% were considered negligible and recorded as zero.

S. aureus ATCC 25923:

Fraction	Replicate 1 (%)	Replicate 2 (%)	Mean	SD
PBP 10% ACN	0.00	0.00	0.00	0.00
PBP 50% ACN	85.67	77.62	81.65	5.69
PBP 100% ACN	12.59	21.68	17.13	6.43
PBP non-retained	3.50	21.68	12.59	12.85

C. albicans ATCC 10231:

Fraction	Replicate 1 (%)	Replicate 2 (%)	Mean	SD
PBP 10% ACN	17.08	19.93	18.50	2.01
PBP 50% ACN	91.46	93.06	92.26	1.13
PBP 100% ACN	12.28	19.93	16.11	5.41
PBP non-retained	0.00	0.00	0.00	0.00

E. coli ATCC 25922:

Fraction	Replicate 1 (%)	Replicate 2 (%)	Mean	SD
PBP 10% ACN	0.00	0.00	0.00	0.00
PBP 50% ACN	1.11	0.00	0.55	0.78
PBP 100% ACN	0.00	0.00	0.00	0.00
PBP non-retained	0.00	0.00	0.00	0.00
